# Altered neuroepithelial morphogenesis and migration defects in iPSC-derived cerebral organoids and 2D neural stem cells in familial bipolar disorder

**DOI:** 10.1093/oons/kvae007

**Published:** 2024-04-03

**Authors:** Kruttika Phalnikar, M Srividya, S V Mythri, N S Vasavi, Archisha Ganguly, Aparajita Kumar, Padmaja S, Kishan Kalia, Srishti S Mishra, Sreeja Kumari Dhanya, Pradip Paul, Bharath Holla, Suhas Ganesh, Puli Chandramouli Reddy, Reeteka Sud, Biju Viswanath, Bhavana Muralidharan

**Affiliations:** Institute for Stem Cell Science and Regenerative Medicine (inStem), GKVK - Post, Bellary Road, Bengaluru, Karnataka, India-560065; National Institute of Mental Health and Neurosciences (NIMHANS), Hosur Road Bengaluru, Karnataka, India-560029; National Institute of Mental Health and Neurosciences (NIMHANS), Hosur Road Bengaluru, Karnataka, India-560029; National Institute of Mental Health and Neurosciences (NIMHANS), Hosur Road Bengaluru, Karnataka, India-560029; Institute for Stem Cell Science and Regenerative Medicine (inStem), GKVK - Post, Bellary Road, Bengaluru, Karnataka, India-560065; Institute for Stem Cell Science and Regenerative Medicine (inStem), GKVK - Post, Bellary Road, Bengaluru, Karnataka, India-560065; Institute for Stem Cell Science and Regenerative Medicine (inStem), GKVK - Post, Bellary Road, Bengaluru, Karnataka, India-560065; Institute for Stem Cell Science and Regenerative Medicine (inStem), GKVK - Post, Bellary Road, Bengaluru, Karnataka, India-560065; Institute for Stem Cell Science and Regenerative Medicine (inStem), GKVK - Post, Bellary Road, Bengaluru, Karnataka, India-560065; Institute for Stem Cell Science and Regenerative Medicine (inStem), GKVK - Post, Bellary Road, Bengaluru, Karnataka, India-560065; National Institute of Mental Health and Neurosciences (NIMHANS), Hosur Road Bengaluru, Karnataka, India-560029; National Institute of Mental Health and Neurosciences (NIMHANS), Hosur Road Bengaluru, Karnataka, India-560029; National Institute of Mental Health and Neurosciences (NIMHANS), Hosur Road Bengaluru, Karnataka, India-560029; Centre of Excellence in Epigenetics, Department of Life Sciences, Shiv Nadar Institution of Eminence, Delhi-NCR, India-201314; National Institute of Mental Health and Neurosciences (NIMHANS), Hosur Road Bengaluru, Karnataka, India-560029; National Institute of Mental Health and Neurosciences (NIMHANS), Hosur Road Bengaluru, Karnataka, India-560029; Institute for Stem Cell Science and Regenerative Medicine (inStem), GKVK - Post, Bellary Road, Bengaluru, Karnataka, India-560065

**Keywords:** cortical organoids, bipolar disorder patients, neuroepithelial bud organization, cellular migration

## Abstract

Bipolar disorder (BD) is a severe mental illness that can result from neurodevelopmental aberrations, particularly in familial BD, which may include causative genetic variants. In the present study, we derived cortical organoids from BD patients and healthy (control) individuals from a clinically dense family in the Indian population. Our data reveal that the patient organoids show neurodevelopmental anomalies, including organisational, proliferation and migration defects. The BD organoids show a reduction in both the number of neuroepithelial buds/cortical rosettes and the ventricular zone size. Additionally, patient organoids show a lower number of SOX2-positive and EdU-positive cycling progenitors, suggesting a progenitor proliferation defect. Further, the patient neurons show abnormal positioning in the ventricular/intermediate zone of the neuroepithelial bud. Transcriptomic analysis of control and patient organoids supports our cellular topology data and reveals dysregulation of genes crucial for progenitor proliferation and neuronal migration. Lastly, time-lapse imaging of neural stem cells in 2D *in vitro* cultures reveals abnormal cellular migration in BD samples. Overall, our study pinpoints a cellular and molecular deficit in BD patient-derived organoids and neural stem cell cultures.

## Teaser

Psychiatric disorders such as bipolar disorder pose a major burden on the population worldwide. However, it is very challenging to understand the neuropathological mechanisms of these disorders as we have very limited or no access to the patient brain samples. The next best model to study mental illnesses is brain organoids that are generated using patient-derived stem cells, which retain the genetic properties of the patient. Brain organoids are self-organising three dimensional ‘in a dish’ cellular models that recapitulate specific brain tissue regions during development. We can generate brain organoids or neuronal cultures using these stem cells and compare them across patients and healthy controls to unravel some of the biological features of the disorder.

In this study, we compare the topology of 3D brain organoids and neural stem cell migratory properties across two bipolar patients and two healthy controls. We find that the proliferative, migratory and self-organising capacity of neural progenitor cells is compromised in patients as compared to controls. In summary, in this study we model early neurodevelopmental deficits in bipolar patients from a clinically dense family using 2D and 3D neuronal cultures.

## Graphical Abstract

**Graphical Abstract:**
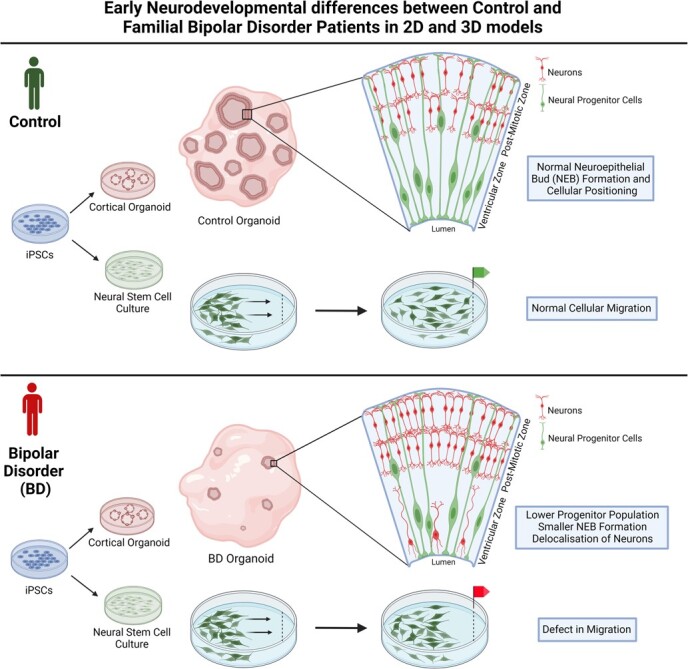

## INTRODUCTION

Bipolar disorder (BD) is a severe, chronic psychiatric illness defined by recurring episodes of manic and depressed moods, impacting emotion, perception, and social behaviour [1, 2]. BD is associated with decreased quality of life and accounts for 6.8% of global mental disorder disability adjusted life years (DALYs) [3] . A 2016 study indicated a ~ 50% global rise in BD cases from 1990 to 2013 [4]. In India, specifically, 7.6 million BD cases were reported in 2017, with a 0.6% prevalence [5].

The underlying mechanisms of BD remain poorly understood, with a weak prognosis and uncertain treatment outcomes [6]. Therefore, studying the molecular and cellular mechanisms behind BD is crucial. This is particularly challenging due to BD’s polygenic complex inheritance, environmental risk factors and lack of access to human brain tissue [7]. Cerebral organoids and 2D neural cultures generated from patient iPSCs allow for human *in vitro* disease modelling and can overcome limitations associated with animal models or post-mortem brain samples [8].

Bipolar disorder (BD) is highly heritable, with the majority of cases being familial. Genetic factors account for 60% to 85% of the risk [10–11] and for first-degree relatives, the risk of developing BD is 5–10%. However, there is a small fraction (0.5–1.5%) of patients with non-familial or sporadic BD [9]. A study comparing clinical characteristics of familial and non-familial probands revealed that both groups experienced a similar number of symptomatic episodes. Familial BD was more likely to exhibit mixed states (co-occurrence of mania and depression), whereas non-familial BD displayed a higher frequency of depressive episodes [12]. Besides genetics, non-genetic factors including environmental factors such as prenatal and perinatal stress, life events, and trauma also contribute to BD [13].

The pathogenesis of BD is not fully understood at the molecular and cellular levels. However, limited known mechanisms point towards genetic and epigenetic components associated with BD [14–16].Number of GWAS studies have unravelled the genetic variants associated with BD, of which many are involved in early neurodevelopment [17, 18]. Some of the genetic risk variants associated with BD are *CACNA1C, SYNE1, TRANK1, NFIX, MAD1L1, ODZ4 and AKAP11* [19–22]. In addition to GWAS, transcriptome profiling and gene ontology analysis in a subset of BD patient derived-organoids show differential expression of genes involved in early neurodevelopment, cell migration, synaptic biology, cell adhesion, and epigenetic changes such as H3K4 methylation [23, 24].

Earlier studies have utilized patient-derived iPSCs to create both 2D neuronal cultures and 3D brain organoids in order to elucidate the mechanistic basis of BD pathogenesis. Some of the most significant pathophysiological mechanisms identified thus far include ion channel dysfunction [25, 26], impaired calcium signalling [27, 28], mitochondrial dysfunction [29, 30], inflammation [31], and neuronal hyperexcitability [32, 33]. A recent study demonstrated that iPSC-derived cortical spheroids from BD patients exhibit smaller sizes, a lower proportion of neurons, and reduced neural network activity in comparison to control spheroids [34]. In another study, patient-derived iPSC neuronal cultures were utilized to demonstrate the presence of mitochondrial abnormalities and hyperactive action potential firing in young neurons from BD patients. Interestingly, this hyperactivity was selectively reversed by lithium treatment, but only in BD Lithium-responder neurons [35]. In a separate study, it was observed that BD-iPSC-derived neural progenitor cells (NPCs) and neurons displayed reduced store-operated Ca^2+^ entry and exhibited a distinctive transcriptome profile, indicating accelerated neuronal differentiation [36]. Furthermore, another research group generated astrocytes from BD-patient iPSCs and demonstrated that BD astrocytes release IL-6, which impacts neuronal activity. Notably, BD patients also exhibited elevated levels of circulating IL-6 in their blood [37].

In the current study, we specifically assess the cell-types and gene expression profiles that are affected during early stages of neurodevelopment in 2D and 3D *in vitro* models derived using BD patient-iPSCs. We also focus on cellular organization in BD organoids to understand how tissue topology might be affected in BD.

Neurodevelopment in the cortex starts with proliferating neural progenitors or stem cells, which divide to generate various neurons and glia. These neurons then migrate to their appropriate locations in the cortical plate, where they mature and form connections to both cortical and sub-cortical areas [38]. Disruptions in these processes are implicated in schizophrenia, a neuropsychiatric disorder which shares several molecular mechanisms with BD [39, 40].

To investigate if and how early neurodevelopment might be impacted in BD, we specifically chose to study a clinically dense multiplex family characterized by a high incidence of BD within the first generation [41]. Such clinically dense families, with a high penetrance of the disorder, provide unique opportunities as an experimental design to comprehend any potential neurodevelopmental origins of the disorder. We have utilised iPSC lines from BD patients and healthy controls to study altered neurodevelopmental phenotypes in an *in vitro* cortical organoid and 2D neural stem cell disease modelling system. These iPSCs were sourced from The Accelerator Program for Discovery in Brain Disorders using Stem Cells (ADBS) [42]. It is a longitudinal study which has recruited individuals from clinically dense Indian families with a strong history of mental illnesses including patients and unaffected family-controls, along with unrelated healthy individuals (non-familial control). The primary aim is to comprehend the neuropathological mechanisms underlying neuropsychiatric syndromes within the Indian population.

Cortical organoids from BD patients exhibit altered neuroepithelial morphogenesis compared to healthy controls. The number of neuroepithelial buds formed is reduced, and the ventricular zone (VZ) area is also diminished, along with a marked decrease in the count of SOX2-positive progenitors. TUJ1-positive neurons in the VZ of patients show disarray, suggesting abnormal or dysregulated neuron placement/migration.

For molecular insights, we conducted transcriptomic analysis on organoids derived from BD patients. We observed significant downregulation of genes crucial for progenitor proliferation, as well as dysregulation in neuronal migration when compared to control. Further, deconvolution of the bulk-RNA sequencing data revealed clusters of different neural cell types, specifically highlighting the reduction in radial glia population in patients.

Using an EdU-based proliferation assay, we further validated that patient organoids show proliferation deficits compared to control organoids. Interestingly, these proliferation deficits were not observed in iPSCs suggesting that the deficit is specific to neuronal cell types, thereby shedding valuable insight into the disease itself. Furthermore, we performed time-lapse imaging of neural stem cells (NSCs) and observed abnormal cellular migration, altered speed and changed directionality of NSCs in patients.

Additionally, we also derived insights from the whole exome sequence analysis of patients and controls to understand patient-specific genetic variations that might be causal to BD phenotype. We found 7 variants in genes that are known to be associated with neurodevelopment and disorders. Furthermore, the Magnetic Resonance Imaging (MRI) data from patients also revealed white matter anomalies in patient A1.

Altogether, our study presents a comprehensive assessment of early neurodevelopmental phenotypes associated with BD pathogenesis using 3D and 2D *in vitro* models, revealing altered tissue topology and molecular changes associated with BD, with additional insights on potential genetic causes and structural variations in brain scans of BD patients.

## MATERIALS AND METHODS:

### Human induced pluripotent stem cell (hiPSCs):

**Figure 1:**
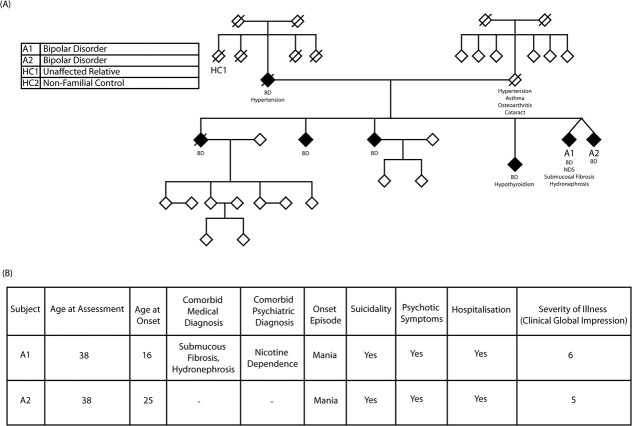
(A) Pedigree of clinically dense family and (B) table showing the clinical information for BD patients

The hiPSC lines for BD patients and healthy controls were obtained from Accelerator Program for Discovery in Brain disorders using Stem cells (ADBS). A multiplex, clinically dense family with several BD patients was chosen due to the high penetration. The clinical details of the patients are included in Fig. 1. From this family, the available iPSCs were acquired from ADBS consortium, including the non-identical twins (A1 and A2), one healthy familial control HC1 and one healthy non-familial control (HC2) [43]. The study was approved by the Institutional Human Ethics and Stem Cell Committee of inStem, Bengaluru, India and the National Institute of Mental Health and Neurosciences, Bengaluru, India.

The derivation of iPSCs was performed as previously described [42]. The iPSCs had normal karyotype and showed tri-germ layer differentiation potential as assessed by markers by PCR- Nestin (ectoderm), Nodal (mesoderm), GATA4(endoderm). All iPSCs lines were positive for standard pluripotency markers (Supplementary Fig. 4), and were negative for mycoplasma.

### Culturing hiPSCs and generating cortical organoids

The hiPSCs were cultured using StemFlex™ Medium (Thermo Fisher, A3349401) in 6-well, Matrigel®-coated plates (Corning® Matrigel®, 354 277). The colonies of iPSCs were passaged manually at 70%–80% confluency and were maintained in the incubator at 37°C, with 5% CO_2_, in humidified air. The iPSCs were passaged no more than 10 passages.

The cortical organoids were generated using the previously established protocol [44], specifically to obtain dorsal forebrain fate. Briefly, 70% confluent hiPSCs were treated with StemPro™ Accutase**®** (Thermo Fisher, A1110501) and were incubated at 37°C for ~5 minutes. The cells were detached using StemFlex™ medium and single cell suspension was formed by gentle pipetting. The cells were centrifuged at 1000 rpm for 5 minutes, and the pellet was resuspended in StemFlex™ medium with 10 μM Rock Inhibitor (Cell guidance systems, Y-27632). From the cell suspension, 10 000 cells were seeded in each well of the 96-well Clear Round Bottom Ultra-Low Attachment Microplate (Corning® Costar®CLS7007- 24EA, Sigma) with 200 μl of StemFlex™ medium in each well. After 1 day, the spheroids were transferred to Ultra-Low Attachment 6 well plate (Corning® Costar® CLS3471-24EA, Sigma), in an Induction medium with 10 μM SB (SB 431542, Tocris, TB1614-GMP) and 5 μM Dorsomorphin (Tocris, 3093) from day 0 to day 5, with daily media change. The spheroids were then transferred to Neural Differentiation medium with 20 ng/ml FGF (Peprotech 100-18B), and 20 ng/ml EGF (AF-100-15) from day 6 to day 25, with daily media change for first 5 days, followed by alternate day media change. Further, EGF and FGF were replaced with 20 ng/ml BDNF (Peprotech, 450–02) and 20 ng/ml NT3 (Peprotech, 450–03) from day 25 to day 43, with media change every 1–2 days. After this point, organoids were maintained in the Maintenance medium without any growth factors with media change every 2–3 days. Organoids were maintained on an orbital shaker at 80 rpm. See Supplementary file for media compositions.

Using immunohistochemistry (IHC), we characterized the organoids for cortical and cell-specific markers for presence of PAX6 (marker for dorsal pallium and neural stem cells), TBR1 and CTIP2 (deep-layer neurons), NESTIN and SOX2 (neural stem cell markers), TUJ1 and DCX (neuronal markers) (Supplementary Fig. 1 and 2) [44–46].

To account for technical variation, for each line, we performed two neural inductions (separate organoid batches). The number of organoids (biological replicates) used for each analysis is specified in the respective methods section and figure legends.

### Generation and maintenance of hiPSC-derived neural stem cells

Neural Stem cells (NSCs) were generated using the monolayer protocol using the STEMdiff™ SMADi Neural Induction Kit (STEMCELL™ Technologies, 05835). In brief, hiPSCs cultured in Stemflex media were dissociated into single cells using Accutase**®** (Sigma, A6964) and plated in 6 well plates in Neural Induction media. The cells were cultured in Neural Induction Media for three passages and were subsequently maintained in Neural Progenitor media (STEMCELL™ Technologies, 05833) as described in the protocol. The resulting NSCs were characterised by immunocytochemistry for NSC markers NESTIN and PAX6. The medium was replaced with Neural Expansion Medium (NEM) 2 days before passaging cells for migration experiments [47], refer to Supplementary file for media composition.

### Immunohistochemistry of organoids

Organoids were harvested at desired time-point and were fixed with 4% paraformaldehyde (PFA) overnight. Organoids were then transferred to 30% Sucrose in phosphate buffered saline (PBS) for overnight equilibration. Organoids were then embedded in Optimal cutting temperature compound (O.C.T)/cryogenic glue, were allowed to snap-freeze on dry-ice and were stored at −80°C till sectioning. Organoids were sectioned at 15 μm thickness on SLEE MEV+ cryostat and the sections were mounted on positively charged slides that were stored in −80°C for long term storage.

Before IHC, the slides were dried for 2 hours at 37°C. The sections were fixed with 4% PFA for 15 minutes at room temperature (RT), followed by two 5-minute washes with PBS. The sections were treated with the quenching solution (0.1 M Glycine) for 20–30 minutes at RT, followed by two 5 minutes’ washes with PBS. Sections were permeabilized with 0.3% Triton™ X-100 (Sigma, T8787) in PBS, followed by a blocking solution containing 5% Fetal Bovine Serum (Thermo Fisher, 10 270 106) in 0.3% PBST at RT for 1 hr. The sections were then incubated with primary antibodies overnight at 4°C. We used antibodies against Sox2 (Santa Cruz, sc-365 823), Tuj1 (Cell Signalling, 5666), Pax6 (BioLegend,901 301), Ctip2 (abcam, ab18465), Nestin (Merck, mab5326), Tbr1 (Abclonal, A19550), Ki67 (abcam, ab16667) and DCX (Abcam, Anti-Doublecortin antibody ab18723), each at dilution of 1:200 in blocking solution with 3% FBS. After the incubation with primary antibodies, sections were washed with PBS (3 washes, 5 minutes each). Sections were then incubated with secondary antibodies at a dilution of 1:1000 in blocking solution with 3% FBS, at RT, in the dark for 2 hrs. We used Donkey Anti-Rabbit IgG, Alexa Fluor™ Plus 555, and Donkey Anti-Mouse IgG, Alexa Fluor™ Plus 488 (Thermo Fisher, A32794 and A32766). Sections were then washed with 0.3% PBST, 4 washes, 5 minutes each. Finally, sections were DAPI-stained (Thermo Fisher, D1306) for 5–10 minutes, and the slides were washed with PBS for 5 minutes. At the end, mounting medium Fluoroshield™ (Merck, F6182) was added onto each section, and covered with coverslips.

### Immunocytochemistry of iPSCs for pluripotency markers

To test the iPSCs for pluripotency markers, iPSCs were seeded in 8-well chamber slides (Thermo Fisher, 154 534) until the formation of separate well-grown colonies. The cells were stained with pluripotency markers using Pluripotent Stem Cell 4-Marker Immunocytochemistry Kit (Thermo Fisher, A24881). The kit protocol was followed for the immunostaining procedure. The slides were mounted and stored as described in the Immunohistochemistry protocol.

### Cell proliferation assay for organoids and iPSCs

The cell proliferation assay with EdU (5-ethynyl 2′-deoxyuridine) was performed using Click-iT™ EdU imaging kit (Thermo Fisher, C10638) as per the manufacturer’s protocol. Cortical organoids were pulsed with 10 μM EdU for 12-hours at DIV40, followed by tissue fixation with 4% PFA for 5–6 hours, followed by IHC procedure as described earlier. For each patient and control line, three organoids harvested as biological replicates (N = 3), and organoid sections were processed for detecting EdU, SOX2 and Ki67.

For the iPSCs lines, each line was grown to ~70% confluency in a 6-well plate. The cells were treated with StemPro™ Accutase® (Thermo Fisher, A1110501) for 5–7 minutes to make a single cell suspension. The cells were centrifuged at 1000 rpm 5 minutes, and were re-suspended in 2 mL of StemFlex™ Medium (Thermo Fisher, A3349401) with 10 μM Rock Inhibitor (Y-27632 Cell guidance systems). Total 20 000 cells were seeded into each well of the 8-well chamber slide (Thermo Fisher, 154 534) pre-coated with Matrigel (Corning® Matrigel®, 354 277). Cells were allowed to grow for 2 days, and were pulsed with Edu (10 μm) for 1 hour. Post EdU pulse, 4% PFA was used to fix the iPSCs for 30 min.. The experiment was performed with three biological replicates. The cells were processed for detecting EdU, SOX2, Ki67.

### Imaging and analysis of cortical organoids

The immunostained organoid sections were imaged on a FV3000 Confocal Laser Scanning Microscope at the Central Imaging & Flow Cytometry Facility (CIFF) at inStem. The imaging was carried out using 10X air and 40X oil objectives. The image analysis was carried out using ImageJ.

In each NEB, we specified two regions for quantification of cell counts and intensity, VZ/IZ and PMZ that correspond to the Ventricular Zone (VZ proliferative NSCs), Intermediate zone (IZ) and post-mitotic zone PMZ (neurons), respectively. We manually distinguished the VZ/IZ and PMZ zones using cellular organisation that separated a tightly organised band of cells (VZ/IZ) from the region surrounding it.

The proportion of SOX2 positive, EdU positive and Ki67 positive cells were manually counted bythe overlaying with DAPI-stained nuclei across Z-stacks. The intensity of SOX2 and TUJ1 staining was measured for VZ/IZ and PMZ regions as mean grey value of the region of interest (ROI).

For quantifying the neuroepithelial bud (NEB) size, the boundaries were drawn manually around VZ/IZ. The outer boundary encircled the entire NEB whereas the inner boundary encircled the lumen (Fig. 3C). The area captured between outer and inner boundaries was quantified as area in μm^2^.

**Figure 2:**
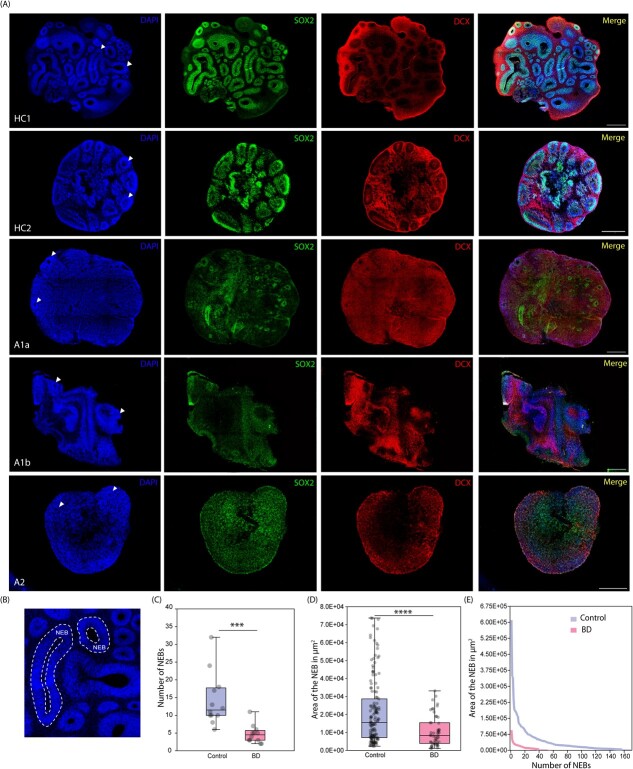
Qualitative and quantitative representation of cortical organoid structure across healthy controls (HC1 and HC2) and patients (A1 and A2). (A) IHC of organoids with DAPI, SOX2 and DCX to show overall topology of organoids. The arrowheads in DAPI panel show NEBs. (B) Representation of boundaries drawn to quantify the area of the VZ/IZ region in NEBs, where area of VZ/IZ = area of the outer boundary of NEB - area of the lumen (C) box plot showing total number of NEBs found in each organoid across patients and healthy controls, where N = 12 organoids, 6 organoids/subject, p < 0.001. (D) the box plot shows the area of VZ/IZ in μm2 across organoids of healthy control (N = 171 NEBs) and patients (N = 56 NEBs), p < 0.0001.(E) the line graph shows the size distribution and number of NEBs. Scale bar represents 500 μm. Statistically significant differences are shown as asterisks

The number of organoids used for each measurement, number of cells/fields counted are specified in the figure legends, and provided in Supplementary Table 1.

### RT-qPCR for organoid and neural stem cell culture

RT-qPCR was performed to validate the DEGs observed in the RNA sequencing analysis. RNA was extracted using the NucleoSpin™ RNA Plus Kit (15 370 195, Macherey-Nagel™). RNA concentration was measured using the Qubit™ (Thermo Fisher, Q33238). cDNA was synthesised using the SuperScript™ IV First-Strand Synthesis System (Invitrogen™, 18 091 050). qPCR was performed using the Power-Up SYBR Green Mixture (Thermo Fisher, A25742) on the QuantStudio 5 machine (Thermo Fisher, A34322). Reactions were run in technical duplicates of each of three biological replicates per line. The PCR reaction protocol was as follows: denaturation at 95°C, annealing at 60°C, followed by Melt Curve Stage. Analysis was performed using the ΔΔCt method, with β Actin (ACTB) as the reference gene. The primers used are listed in Supplementary File.

### Migration analysis in 2D NSC cultures

NSC migration experiments were done as described previously [48]. In brief, cells were seeded in ibidi Culture-Insert 2-well in μ-dish (ibidi, Germany,81 176,) at a density of 20,000–30,000 cells per well for HC1, A1 and A2 and 70,000 cells per well for HC2 in NEM. Passage numbers for NSCs ranged between P2 - P6. The movement of cells across the 500 μm gap in the ibidi dish was recorded in time-lapse images every 15 min for 20 h with a Zeiss Axio Observer microscope using a 10X dry objective. The experiments for each cell line were done in three biological replicates. Migration of NSCs was tracked for 20 h in a 37°C humidified chamber with 5% CO2.

Using ImageJ, a manual tracking plugin, the displacement trajectories of migrating NSCs of the 2 healthy controls (HC1 and HC2) and 2 patient lines (A1 and A2) were tracked with the help of *x*- and *y-*coordinates from the images taken at every 15 minutes for a time period of 20 h (Supplementary Video). From the trajectories of each cell, we estimated the average effective displacement (displacement between the initial and final locations) and average total displacement (overall displacement). From the trajectories of each cell, we estimated the total displacement (0.59*SQRT((X3-X2)^2^ + (Y3-Y2)^2^)) and average speed (0.59*SQRT((X3-X2)^2^ + (Y3-Y2)^2^)/15) across timepoints, where X and Y are the pixel values for each timepoint and 0.59 is the calibration factor for the Zeiss imaging system used. For the average speed, it is displacement/time, which is 15 minutes between each point.

### RNA sequencing and analysis

We harvested 6–7, 43-day old organoids per biological replicate of HC1, A1 and A2 and directly preserved them in TRIzol™ Reagent (Thermo Fisher, 15 596 026), and followed the company protocol for RNA isolation. The experiment was performed with three biological replicates. The sequencing was performed in the Next Generation Genomics facility on campus. The RNA quantification was carried out using Qubit™ RNA Assay Kit (Thermo Fisher, Q10210). The mRNA was isolated using the kit NEBNext Poly(A) mRNA Magnetic Isolation Module (E7490L). The Kit used for Poly (A) selected RNA library prep was NEBNext® Ultra™ II Directional RNA Library Prep with Sample Purification Beads (E7765L). After library preparation, sequencing was performed on NovaSeq 6000 platform using SP flow-cell with 2x100bp sequencing read length to generate approximately 60 Million paired-end reads per sample.

The quality of total RNA-seq reads was verified using FastQC v0.11.7 [49]. First few bases having low quality was removed using Cutadapt v1.18 (parameters -u 5) and reads > = 38 [50]. Phred scores were mapped to the human genome (hg19/GRCh37) using STAR v2.7.3a [51], and the aligned reads files were then interconverted and manipulated using Samtools (v1.6) [52] and BedTools (v2.25.0) [53]. Aligned read counts against gene features were extracted using multiBamCov [53], a part of the bedtools v2.25.0 using Ensembl release 104 (GRCh37) gene annotations. Downstream analyses including mRNA expression levels normalisation were performed using R with edgeR [54]. Differentially expressed genes (DEGs) were then further analysed by G: profiler [55] and AmiGO [56] online, with default parameters, to provide the significant canonical pathway and functions.

To draw comparison between our cortical organoids and human foetal brain transcriptome, we used dataset by Trevino et al, 2021. This dataset uses both scRNA-seq and scATAC-seq data from the developing human brain to call and annotate cell clusters with marker genes (see Supplementary Table 2 for abbreviated cell types). The DEGs identified from our bulk RNA-seq (1601 in number) were compared to these marker genes of each cell-type specific cluster, by a simple list comparison. This generated a cluster-wise breakdown of the DEGs, and these lists were used for downstream analysis.

### Deconvolution analysis of bulk RNA sequencing data

CIBERSORTx was developed by Newman et al, 2019 [57, 58], and offers two modules for analysis. We have used the Cell Fractions module, which breaks down the bulk tissue expression data into cell subpopulations/clusters. The single-cell RNA-sequencing data we have used for the deconvolution analysis was taken from Trevino et al, 2021. The scRNA library contains 4 samples at post-conception week (PCW) or Gestational week (GW) 16, 20, 21, and 24 – we have used scRNA-seq data of PCW 16. CIBERSORTx uses the scRNA data to generate a matrix of signature genes for each cluster, which it then uses to deconvolute the bulk-RNA-seq data. The signature matrix was generated using the default parameters of the tool. To impute the cell fractions, the default parameters were used – the analysis was done using batch correction, with the number of permutations being set to 100.

### Magnetic resonance imaging (MRI) of patients

For our study, T1-weighted (T1w) structural MRI data were utilized, obtained from the ADBS program, that employs a deep-phenotype approach to investigate five severe mental illnesses [59]. At the time of our data analysis, the cohort included individuals diagnosed with Alzheimer’s disease (ALZ, n = 49), Bipolar Disorder (BD, n = 127, including data from patients A1 and A2 included), Obsessive-Compulsive Disorder (OCD, n = 122), Schizophrenia (SCZ, n = 96), Substance Use Disorder (SUD, n = 131), and a healthy control group (CN, n = 622).

The analysis employed Generalized Additive Models for Location, Scale, and Shape (GAMLSS) model fits from a normative database for out-of-sample centile calculations of total cortical, subcortical, and white matter volumes, using maximum likelihood estimation. This method aligned our dataset with the global brain volume reference curves, accounting for both inter-individual variations and potential batch effects intrinsic to the dataset. By benchmarking against established norms, this approach provides a robust context to interpret the variations observed in the MRI data of subjects A1 and A2.

### Statistical analysis

For graphical presentation and statistical analysis, we pooled data for both patients and controls, as practiced in most of the earlier reports that used brain organoid models of neurodevelopmental or psychiatric disorders using more than one iPSC lines [23, 34, 60–62].

Biological replicates used for statistical tests are reported in each figure legend and Supplementary Table 1. Data are presented as boxplots or bar plots, and are regarded statistically significant if p < 0.05. We analysed data using Welch’s T-test, an adaptation of Student’s T-test that is suitable for unequal sample sizes. Additionally, we tested whether there is any batch-effect from two independent inductions for organoid generation using two-way ANOVA mixed model analysis. We found that the batch effect was not significant (P < 0.05) except for one parameter - total number of NEBs, where the batch effect accounted for 11.3% of the total variation (Supplementary Table 1). All statistical analyses were performed using v10.0.2 (GraphPad Software) and R statistical software package (https://www.R-project.org/).

## RESULTS

We have selected a clinically dense multiplex family with a strong inheritance of BD, in the next generation (Fig. 1A). The family pedigree reveals multiple first-degree relatives affected with the disorder and one unaffected family control. For our study, we focused on two individuals, A1 and A2, with severe illness based on clinical parameters (Fig. 1B), one unaffected family control (HC1), and one non-familial healthy control (HC2).

### Patient-derived organoids display fewer and less organised neuroepithelial buds

**Figure 3:**
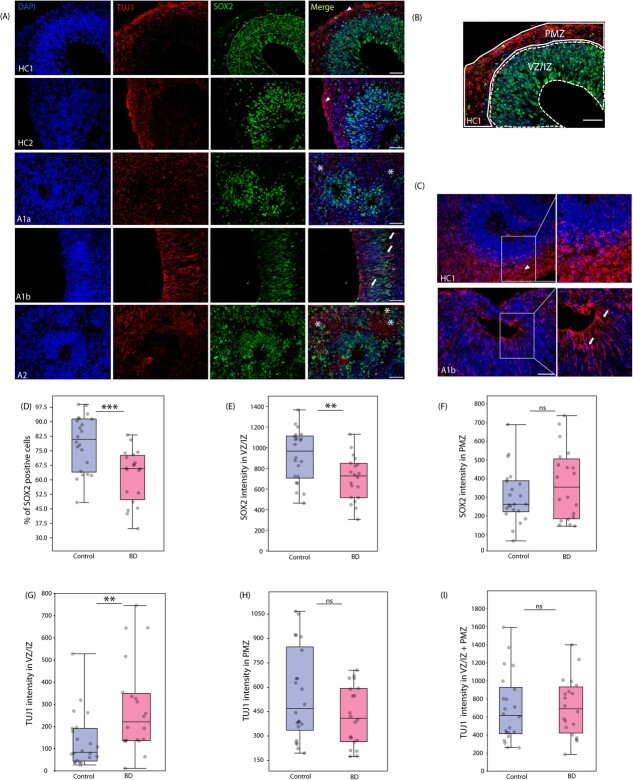
Fewer progenitors and abnormally positioned neurons in BD organoids (A) immunostaining of NEBs with SOX2, apical progenitor marker and TUJ1(βIII tubulin), neuronal marker (B) representative image showing the area defined as VZ/IZ and PMZ in NEBs for quantification of SOX2 and TUJ1 intensity (C) presence of TUJ1 positive neurons in VZ of patient organoid showing neuronal migration defect when compared to control (D) box plot showing percentage of SOX2 positive cells out of ~200 DAPI stained cells counted across patients and control (p-value <0.001). (E and F) box plot showing quantification of SOX2 intensity in VZ (p < 0.01) and PMZ regions (non-significant). (G, H, I) box plots showing quantification of TUJ1 intensity in VZ/IZ (p < 0.01), PMZ regions (non-significant) and the combined intensities of VZ/IZ and PMZ regions (non-significant). For panels D-I, N = 20–22, for each line of patient and control, 5–6 organoids per subject, and 2 fields per organoid. The scale bar represents 50 μm

To capture disease etiology arising during early neurodevelopment in BD patients vs in healthy controls, we examined 40–50-day old iPSC-derived cortical organoids. At this stage, organoids display the formation of neural rosettes or neuroepithelial buds (NEBs) (Fig. 2A, B) which are organised structures reminiscent of the developing neural tube that eventually develops into different regions of the central nervous system, including cerebral cortex. Thus, in this study, we consider a neuroepithelial bud as a radial arrangement of neural stem cells, with lumen at the centre, as described by previous studies [45, 63–66].The cortical organoids reveal distinct regions that mimic the organization and cellular composition of the cerebral cortex during early stages of development which primarily consists of two main areas. The first consisting of region mimicking ventricular zone and intermediate zone (henceforth, called VZ/IZ) (Fig. 3B), which is mainly composed of apical radial glia progenitors organised radially around a central lumen. These apical progenitors (APs) proliferate and differentiate to give rise to neurons. The newly born neurons migrate and ultimately reside outside the VZ/IZ region mimicking the cortical plate, henceforth called as post-mitotic zone (PMZ) (Fig. 3B) consisting of different neuronal subtypes present in cortical layers.

We conducted a characterization of the cortical organoids to confirm the expression of apical radial glia markers, including PAX6, SOX2, NESTIN, as well as pan-neuronal markers like TUJ1 and DCX. We also assessed the presence of deep layer cortical neuronal subtype markers such as TBR1 and CTIP2 (Supplementary Fig. 1).

We observed that the overall tissue topology was suboptimal in BD patient-organoids compared to healthy controls (Fig. 2A). We quantified the total number and size of NEBs using DAPI-stained cross-sections of organoids (Fig. 2B). We found that the total number of NEBs in patients were significantly lower (Fig. 2C). On average, patient derived organoids exhibited approximately 3 times lesser number of NEBs compared to those found in healthy controls.

Similarly, the area of the VZ/IZ region within each NEB displayed a significant reduction. Also, the control organoids on an average showed higher number of large-sized NEBs (Fig. 2D and 2E). Specifically, in patient A1, we found two types of organoid morphologies. One tissue topology showed smaller NEBs, both in size and number, with poorly formed VZ/IZ as compared to the control (Fig. 2A, A1a). The other type showed fewer and smaller NEBs along with bands of tightly packed progenitors at the outer boundary of the organoid (Fig. 2A, A1b). These bands of progenitor cells often did not form well-organised NEBs. Generation of NEBs involves cis-fusion of the ends of the epithelium to generate a lumen in the centre [67]. From the peculiar morphology observed in A1b, it seems that cis-fusion was altered, leading to the inability of the developing neuroepithelial bud to bend and create a NEB with lumen. In patient A2, the NEBs were significantly small in size, with thinner VZ/IZ regions (Fig. 2A). Overall, the control NEBs were, on average, three times larger than patient NEBs (Fig. 2D).

Together, these data showed that healthy controls tend to form well-organised NEBs that are larger in size and higher in numbers as compared to patients.

### BD organoids have fewer progenitors and abnormally positioned neurons

To further understand how APs and differentiated neurons are organised within NEBs, we examined the VZ/IZ and PMZ regions immunostained with AP marker SOX2 and neuronal marker TUJ1 (Fig. 3Aand C). Compared to control, which had at least 2–3 layers of SOX2-positive progenitors, we observed fewer palisades of SOX2-positive APs surrounding the cortical rosettes in patient organoids (Figure 3A, denoted by lines in SOX2 panel). To quantitatively assess a region of interest in the NEB, we analysed and counted the percentage of SOX2 positive cells in the VZ/IZ region of NEB of each organoid, and found that in patients, on an average, there was ~20% reduction in SOX2 positive cells. (Fig. 3D). Additionally, we measured SOX2 intensity of the VZ/IZ region, and found that the intensity was significantly reduced by ~25% in patients compared to controls (Fig. 3E). However, there was no significant variation in SOX2 intensity in the PMZ region (Fig. 3F). These data pinpoint a clear reduction in the number of progenitors leading to sub-optimally formed NEBs affecting early neurodevelopmental trajectories in BD patient-derived organoids.

In control organoids, TUJ1 positive neurons were seen concentrated in the PMZ (Fig. 3A, 3B) They had long distinct processes both parallel and perpendicular to the basal cortical surface (Fig. 3A HC1/HC2, 3C, HC1, denoted by arrowheads). In contrast, the neurons in the BD organoids were seen in VZ/IZ regions (Fig. 3A and 3C, A1b, denoted by arrows) and were randomly oriented with respect to the cortical surface (Fig. 3A, A1a, A2, denoted by asterisks).

We measured TUJ1 intensity in the VZ/IZ region and found that in BD organoids, the TUJ1 intensity was significantly higher compared to healthy controls, suggesting an aberrant placement or migration defect in neurons (Fig. 3G). Further, we measured the TUJ1 intensity in the PMZ region to quantify the population of differentiated neurons outside VZ and found a decrease between control and patients, though not significant (Fig. 3H). Overall TUJ1 intensity measured across a radial column including VZ/IZ and PMZ did not show any change at this stage (Fig. 3I).

Altogether, these data imply that there are two deficits driving the differential neurodevelopment in patient-derived organoids. First, the lesser number and lower intensity of SOX2 positive progenitors in the VZ/IZ region and second, the misplaced or non-migrated neurons that are stuck in the VZ/IZ region and are not oriented properly leading to aberrant neuronal organization in BD organoids.

### Molecular changes associated with BD cortical organoids

**Figure 4:**
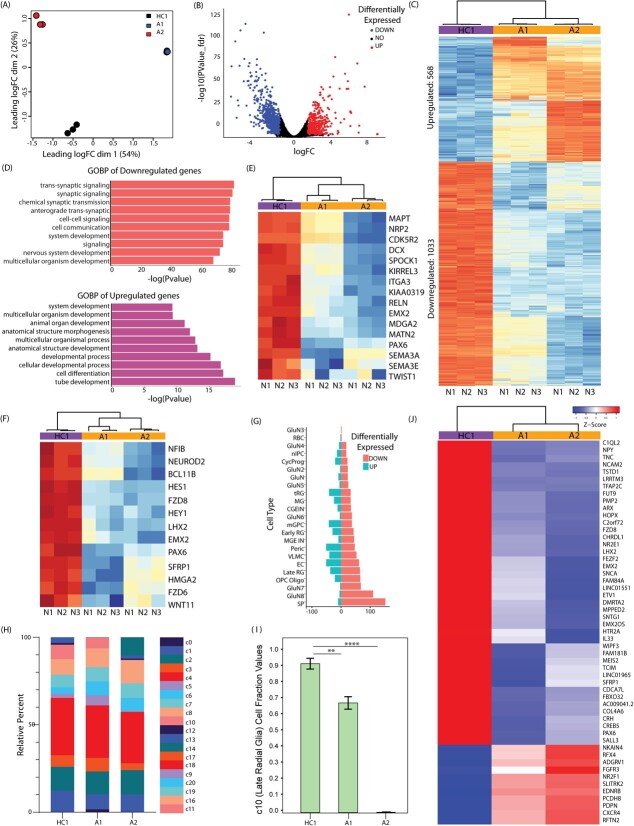
Transcriptome of cortical organoids derived from BD patients and healthy controls. (A) Principal component analysis showing clustering of patients and control based on their transcriptome profile. (B) Volcano plot showing the logFC and -log of p values of differentially expressed genes (DEGs). (C) the heatmap shows the normalised expression values for significant DEGs in BD organoids compared to healthy control. (D) GO ontology enrichment analysis for all DEGs in patients (upregulated and downregulated). (E, F) Heatmaps show DEGs for genes involved in progenitor proliferation and neuronal migration. (G) Bar graph showing the number of dysregulated genes present in a cell type-specific manner when compared to Trevino et al., 2021. Refer to Supplementary Table 2 for the full description of abbreviated cell types.(H) relative cell fractions of different cell type-specific clusters in the bulk transcriptome of HC1, A1 and A2 organoids, after deconvolution using CIBERSORTx. Refer to Supplementary Table 2 for the full description of abbreviated cell types. (I) Abundance of late radial glia (cluster 10) across HC1, A1 (p < 0.01), and A2 (p < 0.001) organoids, estimated through deconvolution.(J) DEGs in late radial glia (cluster 10), across control and patient organoids

To explore the molecular mechanisms behind the dysregulated cortical morphogenesis in organoids, we conducted bulk RNA sequencing on DIV43 organoids (Fig. 4). We used Principal Component Analysis (PCA) to evaluate variability both within and between groups. The PCA revealed group-specific segregation in gene expression between BD and control organoids (Fig. 4A). A total of 1601 differentially expressed genes (DEGs) were identified, with 1033 being downregulated and 568 upregulated (Fig. 4B and Supplementary Table 2). Heat map analysis highlighted distinct gene expression differences between control and BD patient organoids (Fig. 4C). The most significantly downregulated Biological Processes in BD organoids included nervous system development, synaptic signalling and transmission, as well as cell–cell signalling (Fig. 4D), which aligns with earlier study that has reported transcriptomic changes in BD organoids [23].

Trevino et al., 2021 generated single-cell RNA-Seq data from various germinal zones and the cortical plate of the developing human cerebral cortex (Gestational week GW16–24), identifying differentially expressed transcripts in progenitors and neurons [68]. We compared our RNA-Seq data to this dataset to pinpoint cellular-subtype specific genes that are dysregulated in BD patient organoids (Fig. 4G and Supplementary Table 2).

We discovered dysregulation in several genes associated with cycling progenitors, radial glia, and intermediate progenitors in BD (Fig. 4F). Our most significantly downregulated gene was *LHX2*, which is crucial for cerebral cortex specification, progenitor proliferation, and neuron subtype specification [68–70] (Supplementary Table 2). Other vital genes like *EMX2, PAX6*, and *HES1* [71–73], which also play critical roles in progenitor proliferation and maintenance, were downregulated as well (Fig. 4F, Supplementary Table 2). These results were validated using quantitative RT-PCR. (Supplementary Fig. 3A).

In order to gain insights into the proportions of different cell types in patient versus control organoids, we performed a deconvolution analysis on our bulk RNA sequencing data. Utilizing the Trevino et al. scRNA-seq data as a reference, we generated a signature matrix (Supplementary Fig. 2). This matrix was subsequently employed to deconvolute our bulk transcriptome data into different cell fractions (Fig. 4H).

We identified a total of 20 clusters of different neural cell types in the developing brain (Fig. 4H and Supplementary Table 2). Notably, the cluster representing late radial glia (LRG) (Radial glia expressing CD9 and GPX3 and expressed during GW20–24) displayed the highest degree of variation across control and patient samples (Fig. 4I). Of particular significance was the reduction in the number of LRGs observed in patient organoids. This observation aligns with our experimental findings suggesting deficits in proliferation among patients. Given the lower number of LRGs in patient organoids, we sought to determine whether genes specific to LRGs were downregulated in patients, as indicated by the cluster 10/LRG cluster heat map (Fig. 4J). Among the 869 genes that exhibited high expression within the c10/LRG cluster and were extracted from the signature matrix, a total of 53 genes displayed differential regulation between patients and controls. Out of these, 42 genes exhibited downregulation, while 11 were upregulated. Notably, the cluster-specific downregulated genes included critical proliferation genes such as *PAX6, EMX2, LHX2, HOPX, SFRP1,* and *FZD8*.

The dysregulation of these key genes could lead to reduced progenitor proliferation, resulting in fewer progenitors and a reduced number as well as the size of NEBs in patient organoids.

Genes associated with excitatory glutamatergic and subplate neurons are also impacted in our study (Fig. 4G and Supplementary Table 2). These findings are consistent with other research showing that excitatory cell types are often most affected in psychiatric diseases [74].

Given the defective neuronal migration observed in our topological analysis of cortical organoids, we examined our dataset for genes critical to this process using the Gene Ontology term ‘neuron migration’ (GO:0001764) via UniProt. Notably, we found downregulation of essential genes for neuronal migration, such as REELIN and Semaphorins (Sema3A and Sema3E) (Fig. 4E) [75, 76], which was also validated using quantitative RT-PCR (Supplementary Fig. 3A). REELIN plays a significant role in regulating radial migration and the inside-out patterning of the cerebral cortex [77, 78]. A deficit in REELIN is implicated in neuropsychiatric disorders that result in cognitive impairments [79]. The downregulation of *REELIN* and Semaphorins in our study supports the observed migration defects, aligning with broader understandings of neuropsychiatric disease mechanisms.

The generation of cortical organoids involves a series of critical tissue-state transitions, including the induction of cortical fate, lumen formation, NEB development, progenitor proliferation, and, ultimately, the formation of different neuronal subtypes. Changes in the gene expression programs that regulate these steps could disrupt the entire process. Our transcriptomic analysis reveals that critical genes involved in these stages are dysregulated, providing an explanation for the cellular defects observed in the cortical organoids.

### BD organoids show proliferation deficits in apical progenitors

**Figure 5:**
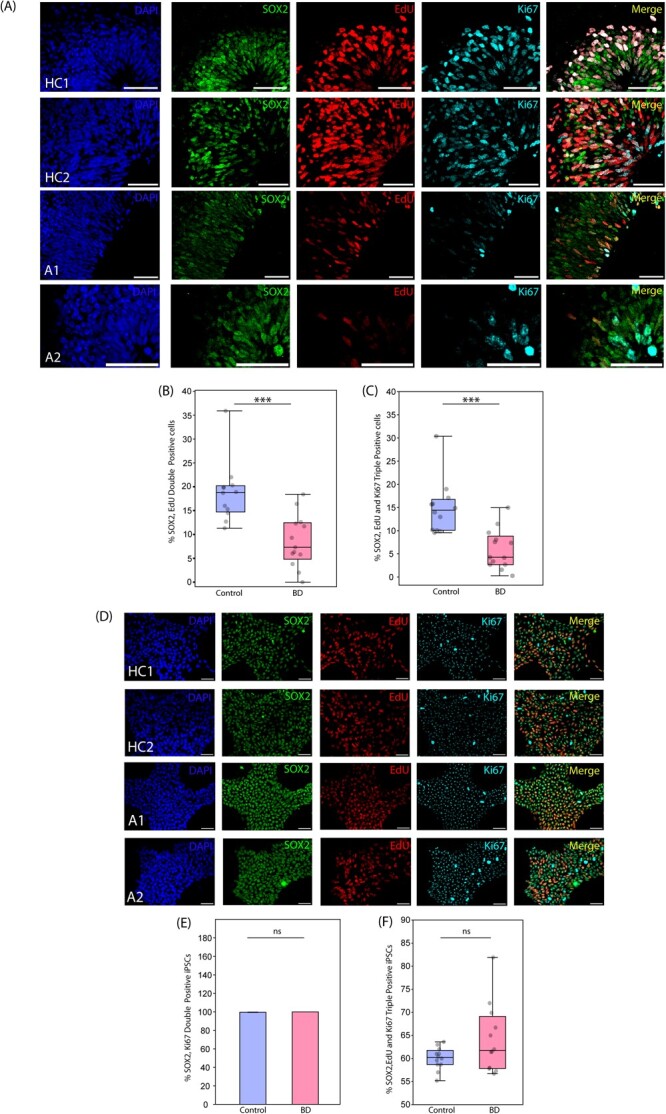
Cell proliferation assay for organoids and iPSCs lines. (A) Representative IHC images of controls and patient organoids showing SOX2, EdU and Ki67.(B, C) percentage of cells showing the presence of proliferation markers in organoids (N = 12–13 fields) (B) p < 0.001, (C) p < 0.001(D) representative IHC images of controls and patient iPSCs showing SOX2, EdU and Ki67 (N = 12 fields).(E, F) percentage of cells showing the presence of proliferation markers in iPSCs, p is non-significant. Scale bar is 50 μm

The lower number of SOX2-positive progenitors from immunohistochemistry experiments and downregulation of progenitor proliferation-specific genes from bulk-RNA sequencing data suggest proliferation deficits in patient organoids. To further validate this, we conducted a cell proliferation assay using EdU (5-ethynyl 2′-deoxyuridine). Organoids were immunostained for SOX2, a progenitor marker along with EdU and Ki67, the cell proliferation markers (Fig. 5A). We quantified the number of SOX2+ EdU+ (double positive), and SOX2+, EdU+, and Ki67+ (triple positive) cells across NEBs of patients and control organoids (Fig. 5B, C). We found that the percentage of cycling neuronal progenitors was significantly lower in the patient group compared to controls. These findings solidify our earlier observations and mechanistically demonstrate the proliferation deficits in patient organoids.

To understand whether these differences between controls and patients stem from variation in iPSC lines, we performed the same assay on iPSC cultures of patients and controls. We found no significant variation in iPSC proliferation (Fig. 5D-F).

### Neural stem cells (NSCs) derived from BD patients exhibit aberrant migration patterns in a 2D-assay

To quantify the cellular migration defects observed in cortical organoids, we conducted a 2D assay to evaluate the migratory capacity of neural stem cells (NSCs) *in vitro* from both control and patient samples, as recently described [48]. NSCs were derived from iPSCs of control (HC1, HC2) and patient (A1, A2) groups. These NSCs were confirmed to express progenitor markers NESTIN and PAX6 through immunocytochemistry (Supplementary Fig. 5). Live cell imaging was used to assess the migratory capacity across all four lines, focusing on parameters such as speed and directionality of NSCs.

**Figure 6:**
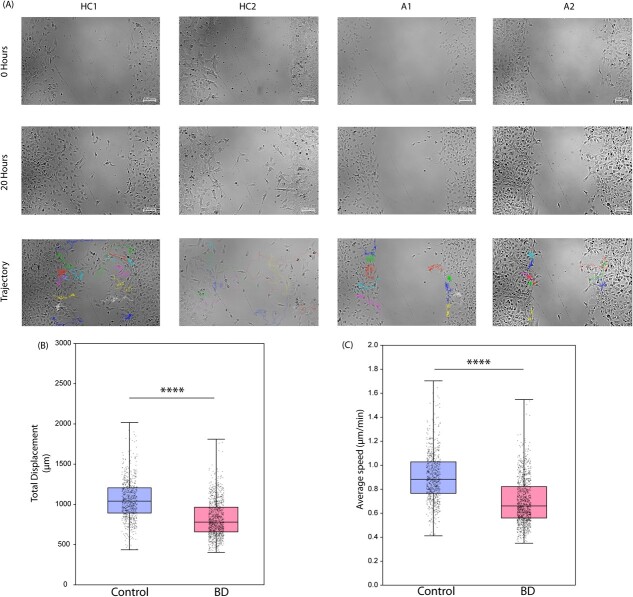
2D NSC migration assay (A) phase contrast images of the cell migration assay at 0 hours and 20 hours. The trajectories of NSC migration are superimposed to show the displacement of single cells from their original position. Box plot of (B) total displacement and (C) average speed of the migrating cells after 20 h for n of healthy controls =735 and patients =1080 (p < 0.0001)

We analysed NSCs from controls and patients for migration over a 20-hour period, capturing images at 15-minute intervals (Supplementary Videos). Only control NSCs had somewhat covered the gap by the 20-hour mark, in contrast to the patient NSCs (Fig. 6A). The migratory trajectories showed minimal movement from the patient NSCs’ initial positions.

Quantitative analysis indicated significantly lower total displacement and average speed for patient-derived NSCs compared to controls (Fig. 6B and C). On average, patient NSCs showed ~20% less displacement and ~ 20% reduced speed, highlighting the aberrant migratory behaviour in these cells.

NSCs are foundational for the entire nervous system, and their migratory characteristics play a crucial role in brain development [80–83]. The observed migratory deficits in patient-derived NSCs may disrupt collective cell behaviour, which aligns with the suboptimal organisation of ventricular and intermediate zones (VZ/IZ) seen in patient organoids.

Subsequently, we investigated whether the molecular changes identified through organoid bulk RNA sequencing were also reflected in NSC cultures. To accomplish this, we specifically selected key proliferative and migratory genes that had been identified as downregulated in the patient organoids and quantified their expression in NSC cultures using qPCR. Our analysis revealed significant downregulation of *PAX6*, *HES1*, *LHX2*, and *REELIN* in patient-derived NSCs though *SEMA3A* remained unchanged (Supplementary Fig. 3B). Prior research has provided evidence of REELIN’s critical role in NSC migration [84–86] and the observed downregulation of REELIN may explain the migration defects observed in 2D NSCs derived from patients.

### Patient-specific genetic variations from whole exome sequencing and observations from MRI of patients

In cases of familial BD, pathological genetic variations often play a significant role. To investigate the potential genetic basis of deficits observed in patient-derived organoids, we curated a set of patient-specific genetic variants. A previous study by *Ganesh* et al.*2022* and the ADBS consortium conducted whole exome sequence analysis in clinically dense families and identified potentially deleterious genetic variants [87]. This study included the control and patient lines used in our current study. We accessed the list of variants identified in this study and analysed the variants specifically present in patients A1 and/or A2, which were absent in both control lines (Table 1). We found 7 variants that were associated with neurodevelopment and brain disorders, of which, 4 variants were found in both patients, namely in genes *CTBP2, CDC27, UNC13D* and *IRF8*, and 3 variants found in either of the patients, *NDUFAF3, MRPS25, MORC3*. Of these, especially *CTBP2* is noteworthy, as it is a transcriptional corepressor and its role in neurodevelopment is well established (Table1). It is expressed in neural stem cells as well as neurons, and its function is critical for proliferation, neuronal differentiation, migration and survival. In both patients, we find *CTBP2* stop-gain variant which should produce a truncated *CTBP2* protein. Overall, *CTBP2*, along with other variants, might cumulatively produce phenotypes that we observe in patient-derived organoids as well as 2D NSCs.

**Table 1:** List of Genetic Variations observed in BD patients

**Chromosome position**	**Reference > alternate**	**Genetic variation type**	**Gene**	**Associated biological function or process**	**Role in neurodevelopment or disorders**
			**Variants found in both patients A1 and A2, but not in controls**		
chr10_126727602	T > A	stopgain	CTBP2	Transcriptional repressor	• Involved in regulation of neural stem cell proliferation and neuronal migration during neocortex development [88–90]
chr17_45249365	C > T	nonsynonymous SNV	CDC27	Component of anaphase-promoting complex (APC)	• Core part of APC, and crucial for cell proliferation during neurogenesis [91]. • Functionally important partner of Microcephalin (MCPH1), a causative gene for microcephaly [92] • Identified as one of the hub genes co-expressed across Alzheimer’s disease and Major Depressive Disorder [93]
chr17_73835960	C > T	nonsynonymous SNV	UNC13D	Exocytosis	• Genetic variants are associated with Cerebral small vessel disease, a leading contributor of cognitive decline and dementia [94]. • Genetic variants are associated with autoimmune and neuro-inflammatory central nervous system disorders [95]
chr16_85936725	T > C	nonsynonymous SNV	IRF8	Transcription factor/Interferon regulatory factor	• Downregulated in Schizophrenia [96]. • Required for microglia maturation [97, 98], associated with inflammation of CNS and loss of IRF8 affects neuronal function [99]
			**Variants found in either A1 or A2, but not in controls**		
chr3_49060600	C > T	stopgain	NDUFAF3 (A1)	Mitochondrial complex I assembly protein	• Important for neuronal survival [100] • Mutations in the gene cause Leigh syndrome, a neurometabolic disorder [101]
chr3_15100896	G > T	nonsynonymous SNV	MRPS25 (A2)	Mitochondrial Ribosomal Protein S25	• Gene variant causes cerebral palsy [102]
chr21_37710067	G > A	nonsynonymous SNV	MORC3 (A2)	Protein localizes to the nuclear matrix and forms nuclear bodies	• Associated with cell proliferation during neurogenesis [103] • Upregulated in Down’s syndrome [104]

**Figure 7:**
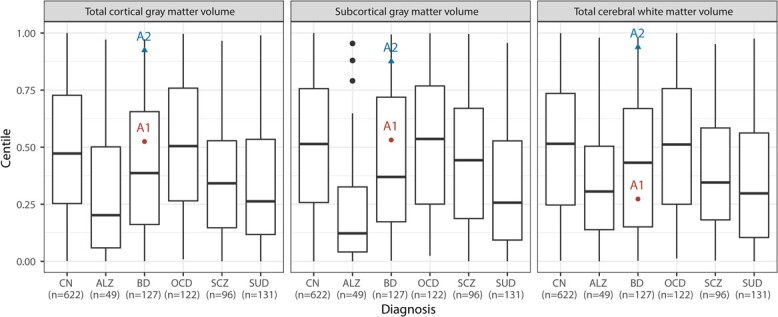
Position of data points representing patients A1 and A2 on the boxplots of T1-weighted structural MRI data for five severe mental illnesses, representing centile calculations of total cortical GMV, subcortical GMV, and total cerebral WMV, using maximum likelihood estimation

Furthermore, to gain insight into the variations in the patients’ brains, we obtained MRI data for patients A1 and A2 from a previously published study [59]. In this study, the analysis focused on understanding the global brain volume variations in the patient cohorts, including A1 and A2 (Fig. 7). For patient A1, we observed that the grey matter volume (GMV) and total subcortical grey matter volume (sGMV) were at the median-centile, which is higher than typically observed in other BD patients. Conversely, A1’s total white matter volume (WMV) was lower. This observation aligns with multiple previous studies that have reported white matter abnormalities in bipolar patients [105–109]. In contrast, patient A2’s volumes were around the 0.9 centile, indicating a deviation from both the bipolar and control groups.

Although it is difficult to derive direct correlations or causation between patient organoids and brain MRI scans, the suboptimal morphology and cellular dynamics in patient organoids can help design insightful hypothesis related to brain anomalies in BD patients. For instance, previous studies have suggested a correlation between neuronal migration defects and white matter abnormalities [110–112]. This correlation could potentially explain the neuronal migration defects observed in the organoids of patient A1, which may be related to the abnormality in white matter volume (WMV) observed in the MRI scans.

## DISCUSSION

Understanding the mechanisms behind BD has long been a challenge due to the disorder’s complexity and the difficulties in modelling it [113, 114]. Evidence suggests that the origins of BD in some cases may be rooted in the early neurodevelopmental stages [115–118] . Clinical symptoms may manifest at later stages of life, possibly due to the interplay between genetic and environmental factors [119]. Many studies on BD patient derived samples has uncovered on late neurodevelopmental phenotypes, such as altered neuronal electrophysiology [120, 121], cellular responses to Lithium (Li) [122, 123], mitochondrial dysfunction [124, 125], and genetic risk factors [11, 19]. However, early neurodevelopmental alterations in tissue morphology or organisation are highly underexplored, and in this study, we analyse early tissue topological deficits using iPSC-derived 3D organoid and 2D NSC models from BD patients and healthy controls.

In this present study, we have used cortical organoids and NSCs derived from iPSCs of BD patients and healthy controls to model the neurodevelopmental changes in BD. Using migration assays, we show that BD NSCs show slower and aberrant migration compared to healthy controls. Similarly, using 3D brain organoids, we show that BD patient organoids have suboptimal organoid topology compared to control. BD patient-organoids show smaller and thinner VZ/IZ areas, with significantly diminished SOX2 positive and EdU/Ki67 positive proliferating population of neural stem cells and fewer NEBs. Importantly, our findings indicate no significant variation in the percentage of cells undergoing cell division between iPSC lines of patients and controls. This suggests that the observed variations in organoid cultures are attributable to differences in the neural stem cells generated post-differentiation from iPSCs into the neuroectodermal lineage. This implies that altered proliferation deficits manifest upon differentiation into the neural lineage, possibly illuminating why the disease may present as a neurological disorder despite the potential presence of strong genetic risk variants. Notably, one of the two patients showed aberrant neuronal positioning in the VZ/IZ region of the neuroepithelial buds, which could be arising from a neuronal migration defect. Interestingly, we observed no change in overall TUJ1 intensity across a radial column, including VZ/IZ and PMZ, at this stage. This could be because defective progenitor proliferation which leads to progenitors exiting the cell cycle prematurely and becoming post-mitotic neurons. This phenomenon, previously observed in LHX2 gene knockout studies, results in a temporary increase in neuronal numbers but ultimately leads to a shrunken cortex due to a reduced number of progenitors [69]. Additionally, the neurons generated are unable to reach the cortical plate due to migration defects, becoming trapped in the VZ/IZ.

Supporting our cerebral organoid findings, our transcriptomic data reveals downregulation of key progenitor proliferation genes like *LHX2, PAX6, and EMX2*. The observed neuronal migration defect is also consistent with the reduced expression of REELIN, a critical gene for neuronal migration that is widely implicated in neuropsychiatric diseases, in patient-derived organoids. Along with genes related to proliferation and migration, we also observed downregulation of 9 genes that are involved in neural tube development as per Gene Ontology analysis (GO 0021915). These genes included *LHX2, SFRP1, FZD6, HES1, TWIST1* (Fig. 5E, 5F) as well as *PRKACB, NOG, SEMA3C,* and *SPINT1.* Earlier studies have shown that TWIST1, FZD6 and SFRP1 are associated with neural tube defects [126–128]. Thus, downregulation in these genes can explain the organizational differences in NEBs that we observe across patient and control groups. Finally, the cellular and molecular variations that we observe across patients and controls can be attributed to some of the genetic variants that were exclusively found in patients.

From the deconvolution analysis of our RNA sequencing data, we have identified the distinct cluster most affected in the BD patient organoids. We observed reduced radial glia population in patients, along with downregulation of 42 genes. Besides proliferation and migration genes, some of the other noticeable genes from this list are *COL4A6* and *NCAM2*, which are involved in cell adhesion. Studies have shown that *NCAM2* is associated with autism and Down’s syndrome, whereas *COL4A6* methylation is linked to high genetic risk of development of mood disorders [129–132].

The analysis also underscores that the organoids generated in our study appear to encompass distinct cell types, suggestive of these organoids mimicking relevant physiological development of human fetal neural tissue.

Our findings on altered early neurodevelopmental trajectories in BD organoids align with recent studies on neurodevelopmental and psychiatric illnesses that have utilized organoid models [36, 60, 61, 133–135]. A recent study reported similar reductions in NEB size and number in BD patient-derived organoids [36], and similar findings were also reported in patient-iPSC derived NSCs from schizophrenia patients [133]. Another study found comparable topological deficits in organoids from Schizophrenia patients [60], which is pertinent since BD and Schizophrenia share overlapping mechanisms [18, 136, 137]. These fundamental disruptions in early neurodevelopment may disrupt the cortical cellular organisation and predispose the cortex to develop such disorders in later stages of life, especially during adolescent stages when the brain undergoes major changes [138–140].

The formation of healthy cortex and neuronal circuits relies on critical early neurodevelopmental hallmarks, including NSC proliferation, migration, and the precise positioning of neuronal subtypes into specific cortical layers [141–143]. These processes are governed by a coordinated expression of multiple genes, fine-tuned cell-to-cell communication, and controlled cellular migration. Any deficits in these areas during early development could potentially disrupt normal circuitry.

In this regard, our data gives a valuable insight into potential cellular deficits at early stages of neurodevelopment in BD patients.

Lastly, it is important to consider that this study focuses on a single clinically dense family, and thus, the phenotypes shown by patient-derived 2D NSCs or organoids might arise from the family-specific profile of genetic variants, which at this point may not be generalised to a broader cohort of BD patients. However, the cellular phenotypes, biological functions, and genes affected in the patients can feed into the broader mechanistic pathways involved in BD pathogenesis.

## Supplementary Material

Web_Material_kvae007

## Data Availability

The RNA sequencing data associated with this manuscript have been submitted to Sequence Read Archive (SRA) with Bioproject Accession number: PRJNA1012902/SAMN37279658.
