# Supplementary material for: Altered neuroepithelial morphogenesis and migration defects in iPSC-derived cerebral organoids and 2D neural stem cells in familial bipolar disorder
Source: Oxf Open Neurosci. 2024 Apr 3;3:kvae007. doi: 10.1093/oons/kvae007 (PMC11024480; doi:10.1093/oons/kvae007)
Supplement: Web_Material_kvae007 [file web_material_kvae007.zip › Phalnikar_et_al_OON_2024_Supplementary Information.pdf]

SUPPLEMENTARY INFORMATION

Phalnikar et al., 2024

Supplementary Figures 1 to 5

Supplementary File

Supplementary Table 1

Supplementary Table 2

Supplementary Figures

Supplementary Figure 1

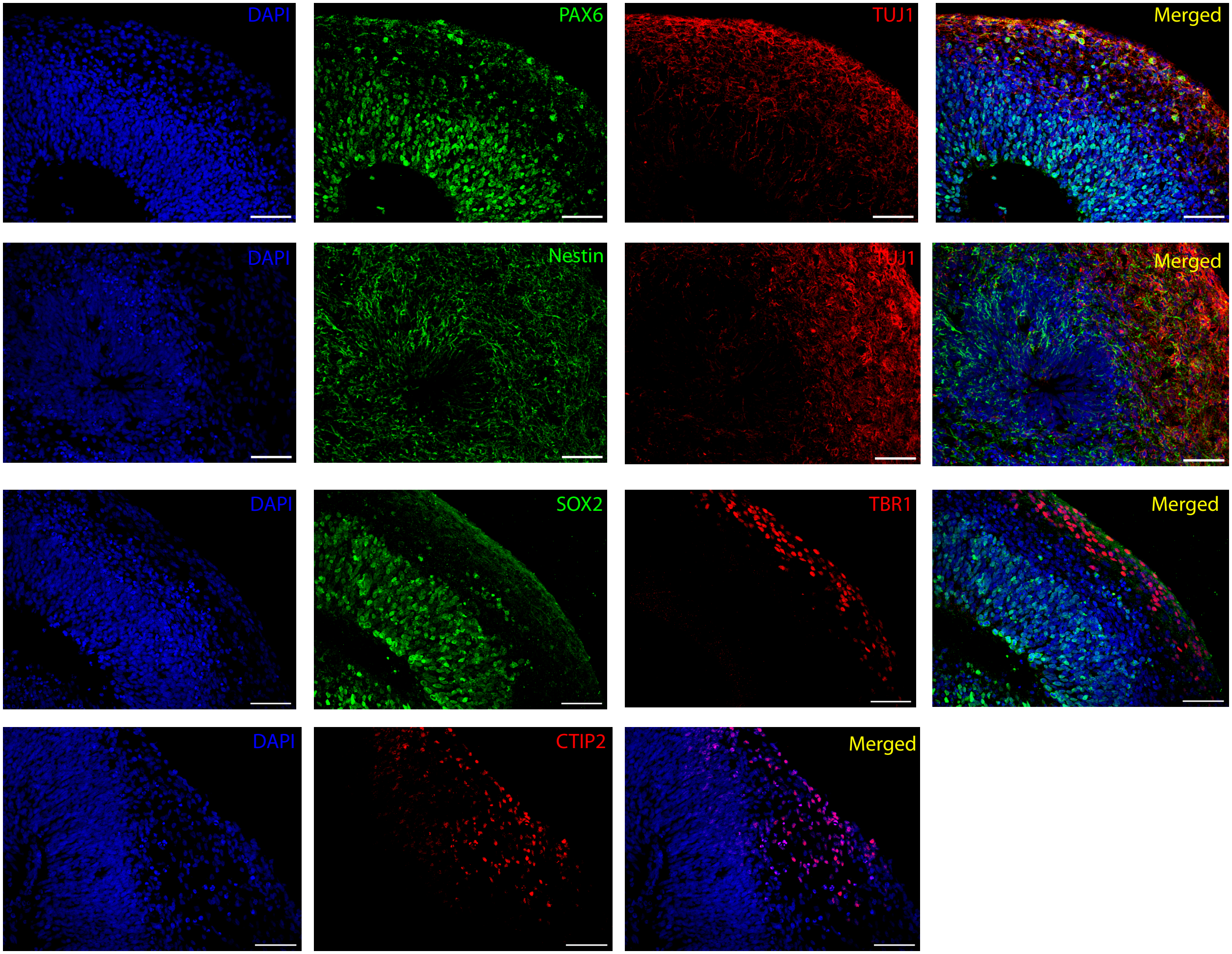

Characterisation of Cortical Organoids using specific markers.

Supplementary Figure 2

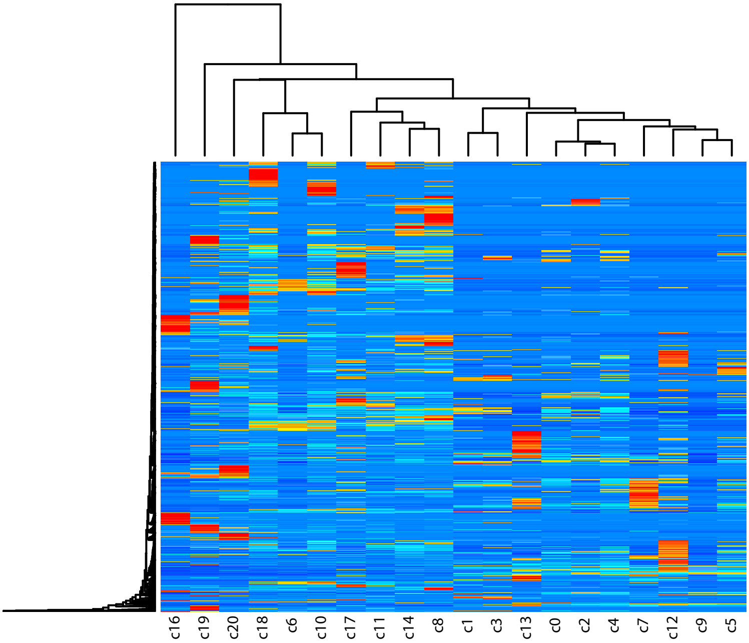

Signature Matrix File generated from Trevino et al (2021) using CIBERSORTx. Refer to Supplementary Table 2 for abbreviated cell types.

Supplementary Figure 3

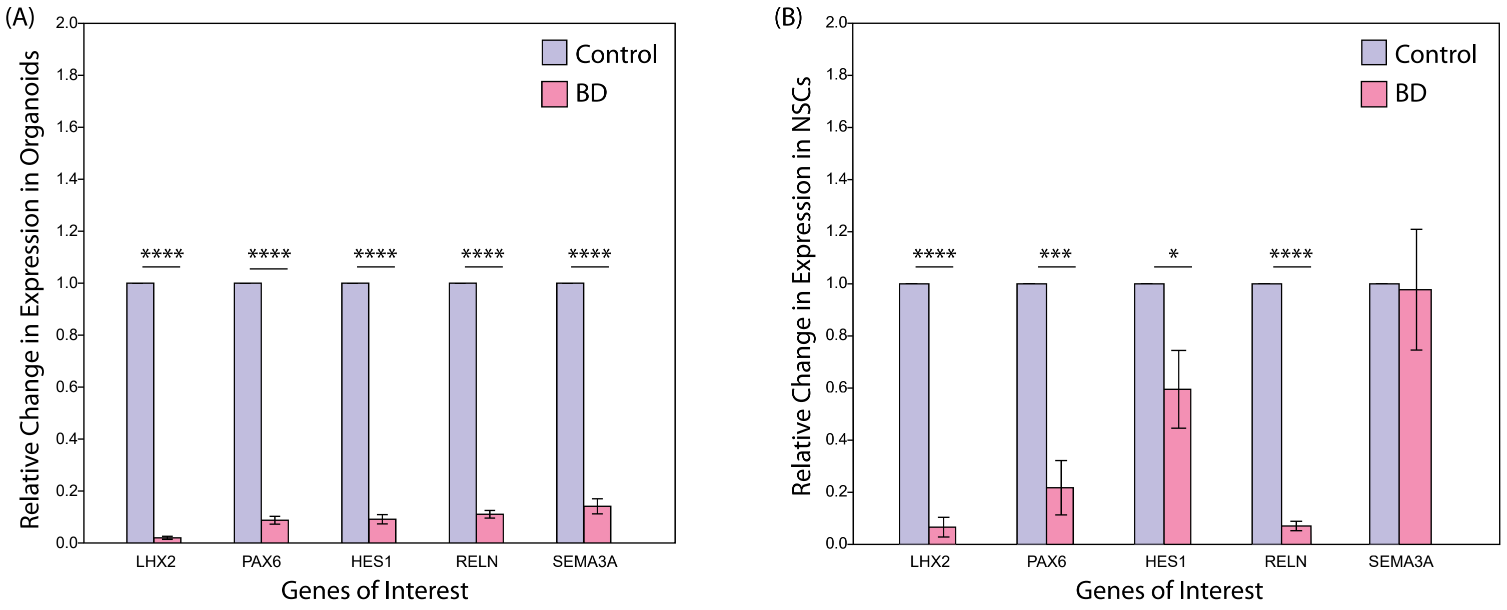

(A) Bar Plot showing relative change in expression of genes of interest which include LHX2, PAX6, HES1, RELN, SEMA3A and SEMA3E in Healthy Control vs Patients (Organoids). Fold Change was normalised to control (Actin). (B) Bar Plot showing relative change in expression of genes of interest which include LHX2, PAX6, HES1, RELN, SEMA3A and SEMA3E in Healthy Control vs Patients (Organoids). p Value <0.0001 (****), pValue=0.0007 (***), pvalue=0.0421(*). n=3.

Supplementary Figure 4

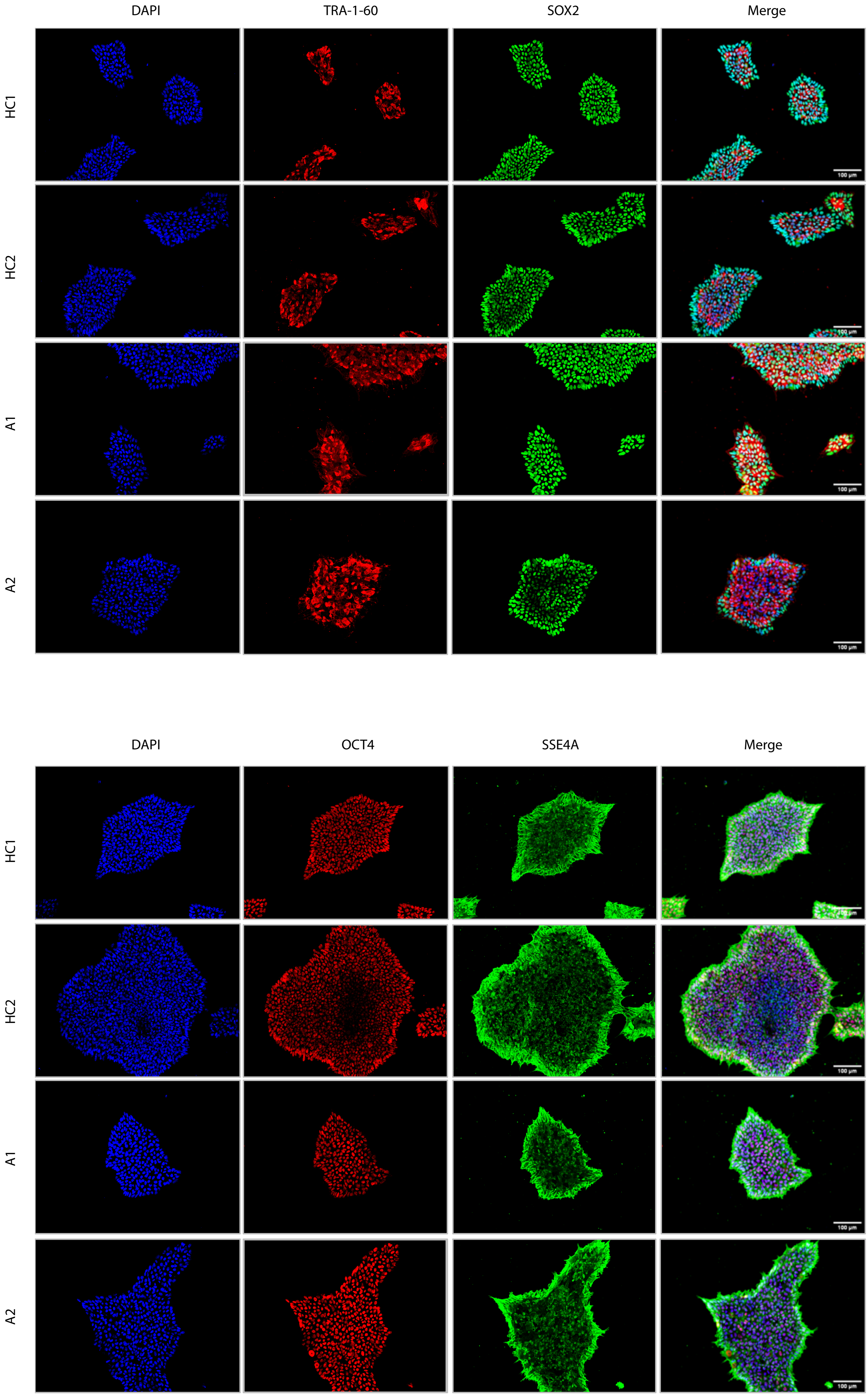

Immunostaining of iPSC lines for pluripotency markers (Scale bar: 100 μm)

Supplementary Figure 5

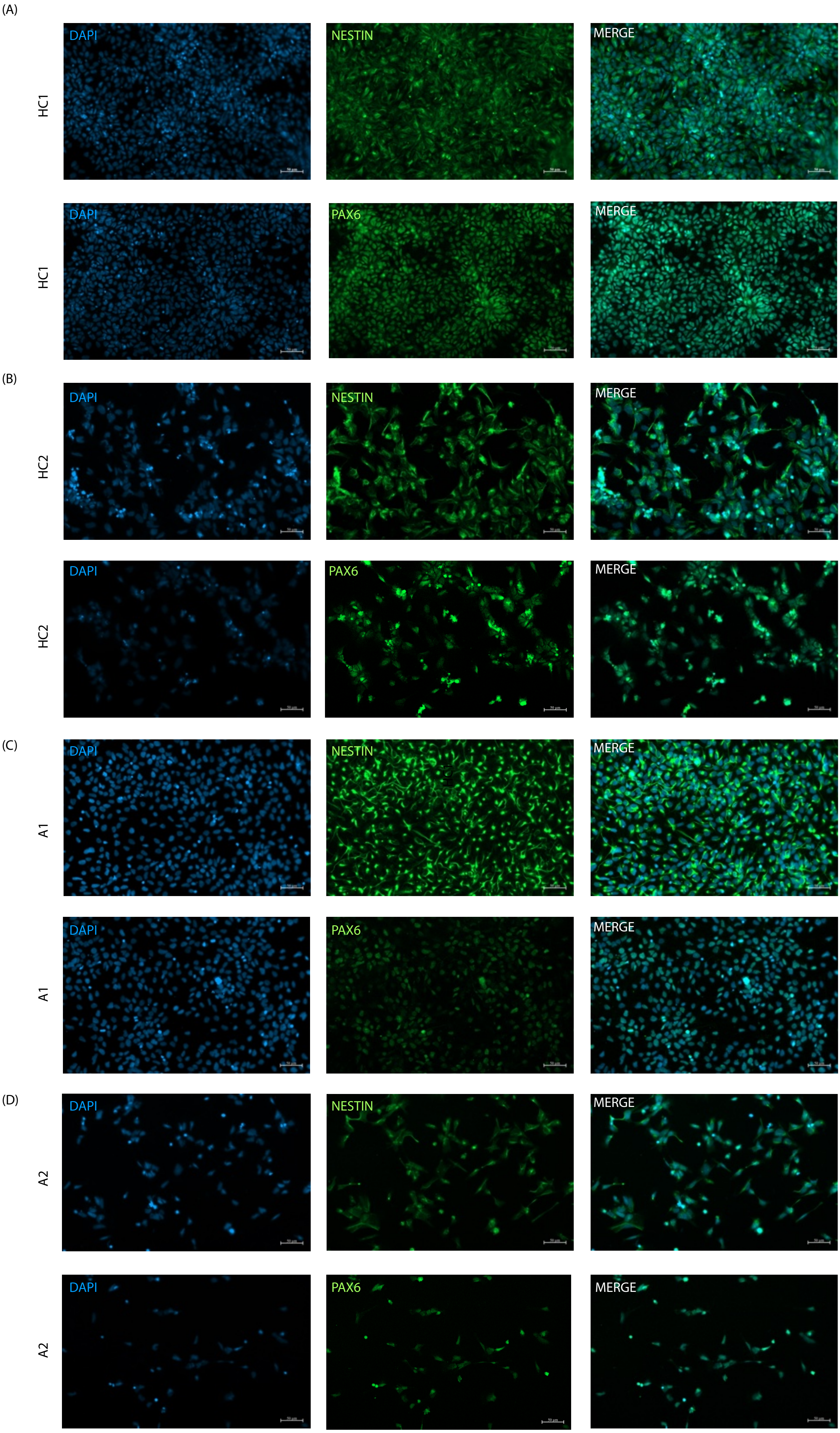

iPSC-derivedNSCs (A) HC1, (B) HC2, (C) A1 and, (D) A2 were characterised for the expression of NSC markers NESTIN and PAX6 (green) and nuclear marker DAPI (blue). The representative images for the same are shown (Scale bar: 50 μm).

Supplementary File

Supplementary Information

Media composition for organoid cultures:

Neural induction media:

Reagents	Concentration	Catalogue Number
DMEM-F12		21331020
KnockOut™ Serum Replacement	20% (vol/vol)	10828028
Penicillin-Streptomycin	1% (vol/vol)	15140122
GlutaMAX™	0.5% (vol/vol)	35050-061
MEM Non-Essential Amino Acids Solution	1% (vol/vol	11140050
2-mercaptoethanol (50mM)	0.1 mM	31350010
SB431542	10 µM	1614
Dorsomorphin	5 µM	3093
XAV-939	5 µM	2848932

Neural differentiation media:

Reagents	Concentration	Catalogue Number
Neurobasal™		21103049
Penicillin-Streptomycin	1% (vol/vol)	15140122
GlutaMAX™	1% (vol/vol)	35050-061
B27 without vit. A supplement	2% (vol/vol)	12587010
FGF	20 ng/ml	PHG0264
EGF(1mg/ml)	20 ng/ml	AF-100-15-1mg
BDNF (20ng/µL)	20 ng/ml	450-02-10ug
NT3(20ng/µL)	20 ng/ml	45003

Neural maintenance media:

Reagents	Concentration	Catalogue Number
Neurobasal™		21103049
Penicillin-Streptomycin	1% (vol/vol)	15140122
GlutaMAX™	1% (vol/vol)	35050-061
B27 without vit. A supplement	2% (vol/vol)	12587010

Neural Expansion Media composition for Neural Stem Cell culture:

Reagents	Concentration	Catalogue Number
DMEM-F12		10656018
B27 without vit. A supplement	2% (vol/vol)	12587010
Penicillin-Streptomycin	1% (vol/vol)	15140122
GlutaMAX™	1% (vol/vol)	35050-061
MEM Non-Essential Amino Acids Solution	1% (vol/vol)	11140050
bFGF	8 ng/ml	PHG0264
Heparin	2 µg/ml	H3149
N2 Supplement	1% (vol/vol)	17502048

qPCR Primer Sequences

Gene	5' – 3' Sequence	Amplicon Size	Source
ACTB F	ACAGAGCCTCGCCTTTG	110bp	[1]
ACTB R	CCTTGCACATGCCGGAG		
RELN F	CCTCGTCCAAGAAGAATGCCTTCC	140bp	Primer-BLAST
RELN R	CGGGTAGCACTGGACCAAG		
SEMA3A F	CACTGCAAAGAGACGCACAA	170bp	Primer-BLAST
SEMA3A R	CTCTCTGCGACTTCGGACTG		
LHX2 F	TCCCTACTACAATGGCGTGG	244bp	[2]
LHX2 R	GTCGGGGTTGTGGTTAATGG		
PAX6 F	CCAGGGCAATCGGTGGTAG	143bp	[3]
PAX6 R	ATCGTTGGTACAGACCCCCT		
HES1 F	TCAACACGACACCGGATAAA	152bp	[4]
HES1 R	CCGCGAGCTATCTTTCTTCA		

References:

1. Roy, J.G., J.E. McElhaney, and C.P. Verschoor, *Reliable reference genes for the quantification of mRNA in human T-cells and PBMCs stimulated with live influenza virus.* BMC Immunology, 2020. **21**(1): p. 4.
2. Krasnytska, D.A., et al., *The impact of glutamine deprivation on the expression of MEIS3, SPAG4, LHX1, LHX2, and LHX6 genes in ERN1 knockdown U87 glioma cells.* Endocr Regul, 2022. **56**(1): p. 38-47.
3. Lee, S.E., et al., *Human iNSC-derived brain organoid model of lysosomal storage disorder in Niemann-Pick disease type C.* Cell Death Dis, 2020. **11**(12): p. 1059.
4. Xu, L., et al., *HES1 promotes extracellular matrix protein expression and inhibits proliferation and migration in human trabecular meshwork cells under oxidative stress.* Oncotarget, 2017. **8**(13): p. 21818-21833.

Supplementary Table 1

Counting Metadata Fig 2,3,5,6

Quantification NEB number and NEB area (Figure 2)			
**Control and Patient line**	**Number of organoid batches (B1 and B2)**	**Number of organoids assessed**	**Number of sections per organoid**
HC1	2	6 (3 B1, 3 B2)	1 section
HC2	2	6 (3 B1, 3 B2)	1 section
A1	2	6 (3 B1, 3 B2)	1 section
A2	2	6 (3 B1, 3 B2)	1 section

Quantification of percent SOX2+ cells, SOX2 and TUJ1 intensity (Figure 3)				
**Control and Patient line**	**Number of organoid batches (B1 and B2)**	**Number of organoids assessed**	**Number of sections per organoid**	**Number of cells counted per field**
HC1	2	5 (2 B1, 3 B2)	1 section, 2 fields (NEB)	180-210 cells per NEB
HC2	2	6 (3 B1, 3 B2)	1 section, 2 fields (NEB)	180-210 cells per NEB
A1	2	5 (2 B1, 3 B2)	1 section, 2 fields (NEB)	180-210 cells per NEB
A2	2	6 (3 B1, 3 B2)	1 section, 2 fields (NEB)	180-210 cells per NEB

EdU Click-it assay for organoids (Figure 5)				
**Control and Patient line**	**Number of organoids**	**Number of sections per organoid**	**Number of cells counted per field**	**Total number of cells counted**
HC1	3	1 section, 2 fields (NEB)	130-200 cells per field	260-400 cells
HC2	3	1 section, 2 fields (NEB)	130-200 cells per field	260-400 cells
A1	3	1 section, 3 fields (NEB)	130-200 cells per field	260-400 cells
A2	3	1 section, 2 fields (NEB)	130-200 cells per field	260-400 cells

EdU Click-it assay for iPSCs (Figure 5)				
**Control and Patient line**	**Number of replicates**	**Number of sections**	**Number of cells counted per field**	**Total number of cells counted**
HC1	3	2 fields per replicate	~100 cells per field	~200 cells
HC2	3	2 fields per replicate	~100 cells per field	~200 cells
A1	3	2 fields per replicate	~100 cells per field	~200 cells
A2	3	2 fields per replicate	~100 cells per field	~200 cells

NSC migration assay (Figure 6)		
**Control and Patient line**	**Number of replicates**	**Total number of cells analyzed**
HC1	3	600
HC2	3	135
A1	3	480
A2	3	600

# P values for all graphs

P values for all Graphs	Controls	Patients
**Figure 2**		
**Area of NEBs - outliers removed**		
p-value <0.0001		****
Mean	21229	10640
**Area of NEBs - including outliers**		
p-value <0.0001		****
Mean	41872	13357
**Number of NEBs**		
p value = 0.0009		***
Mean	14	5
**Figure 3**		
**%SOX2 + cells**		
p-value = 0.0003		***
Mean	79.08	62.27
**SOX2 Intensity in VZ**		
p-value = 0.0034		**
Mean	924.2	701.6
**SOX2 Intensity in PMZ**		
p-value = 0.2019		ns
Mean	299.1	367.2
**TUJ1 Intensity in VZ**		
p-value = 0.0091		**
Mean	136.1	283.7
**TUJ1 Intensity in PMZ**		
p-value = 0.0786		ns
Mean	556.8	425
**TUJ1 Intensity in VZ + PMZ**		
p-value = 0.8849		ns
Mean	692.9	708.6
**Figure 5**		
**%SOX2 EdU organoids**		
p-value = 0.0003		***
Mean	18.78	8.608
**%SOX2 EdU Ki67 organoids**		
p-value = 0.0003		***
Mean	14.68	5.731
**%SOX2 EdU iPSCs**		
p-value=0.0890		ns
Mean	60.02	64.18
**%SOX2 Ki67 iPSCs**		
p-value = 0.0548		ns
Mean	99.61	100
**Figure 6**		
**NSCs - Displacement**		
p-value <0.0001		****
Mean	1058	823.3
**NSCs - Speed**		
p-value <0.0001		****
Mean	0.9018	0.6999

Symbol	Meaning
ns	P > 0.05
*	P ≤ 0.05
**	P ≤ 0.01
***	P ≤ 0.001
****	P ≤ 0.0001

Mixed Model analysis

Figure 2		Significance of Batch effect
**Area of NEBs**		
p-value = 0.18		p-value summary
%Total variation contributed by batch	0.66	ns
**Number of NEBs**		
p-value = 0.018		p-value summary
%Total variation contributed by batch	11.36	*

Figure 3		
**%SOX2 + cells**		
p-value = 0.65		p-value summary
%Total variation contributed by batch	0.23	ns
**SOX2 Intensity in VZ**		
p-value = 0.1443		p-value summary
%Total variation contributed by batch	3.78	ns
**SOX2 Intensity in PMZ**		
p-value = 0.1182		p-value summary
%Total variation contributed by batch	5.19	ns
**TUJ1 Intensity in VZ**		
p-value=0.4795		p-value summary
%Total variation contributed by batch	0.63	ns
**TUJ1 Intensity in PMZ**		
p-value= 0.0287		p-value summary
%Total variation contributed by batch	9.17	*

Supplementary Table 2

Fig4 ABCEF Downreg_gene

EMSEMBL_ID	Gene_name	logFC	logCPM	Pvalue	FDR	description
ENSG00000106689	LHX2	-5.98707498	4.764842229	1.40E-43	5.16E-41	LIM homeobox 2 [Source:HGNC Symbol;Acc:HGNC:6594]
ENSG00000153820	SPHKAP	-5.934902532	3.702166074	7.84E-75	1.49E-71	SPHK1 interactor
ENSG00000104888	SLC17A7	-5.579443055	3.095104926	2.05E-100	1.09E-96	solute carrier family 17 member 7 [Source:HGNC Symbol;Acc:HGNC:16704]
ENSG00000170370	EMX2	-5.448680257	4.096424391	1.38E-21	1.09E-19	empty spiracles homeobox 2 [Source:HGNC Symbol;Acc:HGNC:3341]
ENSG00000258114	None	-5.340941943	3.740407744	1.16E-39	3.53E-37	novel transcript
ENSG00000229847	EMX2OS	-5.272291702	4.366606181	2.41E-34	5.35E-32	EMX2 opposite strand/antisense RNA [Source:HGNC Symbol;Acc:HGNC:18511]
ENSG00000174453	VWC2L	-5.170950135	2.894480181	1.87E-75	4.14E-72	von Willebrand factor C domain containing 2 like [Source:HGNC Symbol;Acc:HGNC:37203]
ENSG00000186732	MPPED1	-5.082771126	3.261331723	9.57E-112	8.48E-108	metallophosphoesterase domain containing 1 [Source:HGNC Symbol;Acc:HGNC:1306]
ENSG00000180730	SHISA2	-4.772098556	3.254344899	2.16E-59	1.74E-56	shisa family member 2 [Source:HGNC Symbol;Acc:HGNC:20366]
ENSG00000156804	FBXO32	-4.722063898	3.480109949	1.30E-82	4.94E-79	F-box protein 32 [Source:HGNC Symbol;Acc:HGNC:16731]
ENSG00000177283	FZD8	-4.609198232	4.871562122	1.56E-118	2.08E-114	frizzled class receptor 8 [Source:HGNC Symbol;Acc:HGNC:4046]
ENSG00000166006	KCNC2	-4.510522025	3.574688265	3.12E-34	6.74E-32	potassium voltage-gated channel subfamily C member 2 [Source:HGNC Symbol;Acc:HGNC:6234]
ENSG00000163630	SYNPR	-4.411611873	3.38322769	1.45E-59	1.29E-56	synaptoporin [Source:HGNC Symbol;Acc:HGNC:16507]
ENSG00000162989	KCNJ3	-4.32446458	2.953988864	7.51E-60	6.88E-57	potassium inwardly rectifying channel subfamily J member 3 [Source:HGNC Symbol;Acc:HGNC:6264]
ENSG00000117154	IGSF21	-4.31638001	4.222602016	5.51E-75	1.13E-71	immunoglobin superfamily member 21 [Source:HGNC Symbol;Acc:HGNC:28246]
ENSG00000106113	CRHR2	-4.311867557	1.531957731	4.00E-55	2.95E-52	corticotropin releasing hormone receptor 2 [Source:HGNC Symbol;Acc:HGNC:2358]
ENSG00000108231	LGI1	-4.227423187	2.766910528	2.71E-78	8.00E-75	leucine rich glioma inactivated 1 [Source:HGNC Symbol;Acc:HGNC:6572]
ENSG00000171532	NEUROD2	-4.184661721	5.03436219	2.39E-24	2.46E-22	neuronal differentiation 2 [Source:HGNC Symbol;Acc:HGNC:7763]
ENSG00000186297	GABRA5	-4.169676494	2.020475626	1.59E-52	9.21E-50	gamma-aminobutyric acid type A receptor subunit alpha5 [Source:HGNC Symbol;Acc:HGNC:4079]
ENSG00000130037	KCNA5	-4.141723661	1.574850937	2.18E-68	3.40E-65	potassium voltage-gated channel subfamily A member 5 [Source:HGNC Symbol;Acc:HGNC:6224]
ENSG00000159248	GJD2	-4.10963809	2.10322668	4.50E-56	3.41E-53	gap junction protein delta 2 [Source:HGNC Symbol;Acc:HGNC:19154]
ENSG00000147256	ARHGAP36	-4.105381376	2.291827986	2.59E-64	2.87E-61	Rho GTPase activating protein 36 [Source:HGNC Symbol;Acc:HGNC:26388]
ENSG00000104332	SFRP1	-4.072521121	6.91465922	3.56E-17	1.89E-15	secreted frizzled related protein 1 [Source:HGNC Symbol;Acc:HGNC:10776]
ENSG00000065717	TLE2	-4.024570366	2.399576448	4.27E-65	5.40E-62	TLE family member 2
ENSG00000197747	S100A10	-3.86146393	1.737022444	5.82E-50	2.86E-47	S100 calcium binding protein A10 [Source:HGNC Symbol;Acc:HGNC:10487]
ENSG00000155926	SLA	-3.821997471	3.235756619	1.60E-12	5.03E-11	Src like adaptor [Source:HGNC Symbol;Acc:HGNC:10902]
ENSG00000255082	GRM5-AS1	-3.796213333	3.279361658	1.77E-16	8.67E-15	GRM5 antisense RNA 1 [Source:HGNC Symbol;Acc:HGNC:40265]
ENSG00000260918	None	-3.76090739	1.591118203	2.01E-40	6.50E-38	novel transcript
ENSG00000234207	LINC01830	-3.750123067	0.732139446	7.08E-43	2.51E-40	long intergenic non-protein coding RNA 1830 [Source:HGNC Symbol;Acc:HGNC:52636]
ENSG00000183715	OPCML	-3.748772195	5.294960278	8.04E-53	4.74E-50	opioid binding protein/cell adhesion molecule like [Source:HGNC Symbol;Acc:HGNC:8143]
ENSG00000215845	TSTD1	-3.734114625	0.562659111	2.85E-53	1.80E-50	thiosulfate sulfurtransferase like domain containing 1 [Source:HGNC Symbol;Acc:HGNC:35410]
ENSG00000259460	None	-3.729102772	3.608546341	7.54E-23	6.72E-21	novel transcript
ENSG00000172995	ARPP21	-3.725071166	3.910777015	2.03E-59	1.68E-56	cAMP regulated phosphoprotein 21 [Source:HGNC Symbol;Acc:HGNC:16968]
ENSG00000131771	PPP1R1B	-3.649163519	1.646843234	1.26E-44	5.01E-42	protein phosphatase 1 regulatory inhibitor subunit 1B [Source:HGNC Symbol;Acc:HGNC:9287]
ENSG00000254489	MPPED2-AS1	-3.646564806	2.621267018	1.34E-21	1.06E-19	MPPED2 antisense RNA 1 [Source:HGNC Symbol;Acc:HGNC:53909]
ENSG00000110975	SYT10	-3.641522033	2.790280122	2.67E-60	2.54E-57	synaptotagmin 10 [Source:HGNC Symbol;Acc:HGNC:19266]
ENSG00000042832	TG	-3.603286113	3.612948549	1.90E-14	7.44E-13	thyroglobulin [Source:HGNC Symbol;Acc:HGNC:11764]
ENSG00000104369	JPH1	-3.587797127	3.125806817	4.71E-69	7.82E-66	junctophilin 1 [Source:HGNC Symbol;Acc:HGNC:14201]
ENSG00000196090	PTPRT	-3.585301805	4.957461429	3.69E-21	2.79E-19	protein tyrosine phosphatase receptor type T [Source:HGNC Symbol;Acc:HGNC:9682]
ENSG00000066735	KIF26A	-3.572333102	4.915223811	2.20E-64	2.65E-61	kinesin family member 26A [Source:HGNC Symbol;Acc:HGNC:20226]
ENSG00000154678	PDE1C	-3.557327512	2.605547374	7.84E-40	2.42E-37	phosphodiesterase 1C [Source:HGNC Symbol;Acc:HGNC:8776]
ENSG00000181291	TMEM132E	-3.544916973	3.482459272	3.01E-59	2.35E-56	transmembrane protein 132E [Source:HGNC Symbol;Acc:HGNC:26991]
ENSG00000145721	LIX1	-3.537596858	3.377068574	5.37E-55	3.86E-52	limb and CNS expressed 1 [Source:HGNC Symbol;Acc:HGNC:18581]
ENSG00000197565	COL4A6	-3.535789745	6.294448042	2.32E-76	5.60E-73	collagen type IV alpha 6 chain [Source:HGNC Symbol;Acc:HGNC:2208]
ENSG00000144596	GRIP2	-3.521179497	3.937011289	3.12E-82	1.04E-78	glutamate receptor interacting protein 2 [Source:HGNC Symbol;Acc:HGNC:23841]
ENSG00000066382	MPPED2	-3.493348177	6.017948851	9.68E-42	3.30E-39	metallophosphoesterase domain containing 2 [Source:HGNC Symbol;Acc:HGNC:1180]
ENSG00000041982	TNC	-3.460010195	5.534541389	1.65E-38	4.86E-36	tenascin C [Source:HGNC Symbol;Acc:HGNC:5318]
ENSG00000134138	MEIS2	-3.449255048	6.548171922	6.88E-24	6.79E-22	Meis homeobox 2 [Source:HGNC Symbol;Acc:HGNC:7001]
ENSG00000168959	GRM5	-3.428514795	4.157908485	3.79E-20	2.65E-18	glutamate metabotropic receptor 5 [Source:HGNC Symbol;Acc:HGNC:4597]
ENSG00000256463	SALL3	-3.422377519	4.754659893	3.93E-41	1.32E-38	spalt like transcription factor 3 [Source:HGNC Symbol;Acc:HGNC:10527]
ENSG00000184160	ADRA2C	-3.417162094	0.660924727	1.28E-29	1.97E-27	adrenoceptor alpha 2C [Source:HGNC Symbol;Acc:HGNC:283]
ENSG00000197585	None	-3.413501965	3.203046524	7.80E-61	7.97E-58	novel transcript
ENSG00000158747	NBL1	-3.393087554	3.200544094	1.59E-40	5.20E-38	NBL1
ENSG00000122584	NXPH1	-3.385524423	4.584325264	3.49E-20	2.44E-18	neurexophilin 1 [Source:HGNC Symbol;Acc:HGNC:20693]
ENSG00000260990	None	-3.377205165	3.099001049	3.06E-15	1.31E-13	novel transcript
ENSG00000058335	RASGRF1	-3.35361106	4.3925585	2.52E-49	1.22E-46	Ras protein specific guanine nucleotide releasing factor 1 [Source:HGNC Symbol;Acc:HGNC:9875]
ENSG00000163032	VSNL1	-3.337442594	3.163419676	6.38E-63	6.78E-60	visinin like 1 [Source:HGNC Symbol;Acc:HGNC:12722]
ENSG00000070182	SPTB	-3.312451367	2.991414807	4.55E-29	6.60E-27	spectrin beta
ENSG00000198797	BRINP2	-3.294450632	3.734604464	5.38E-13	1.81E-11	BMP/retinoic acid inducible neural specific 2 [Source:HGNC Symbol;Acc:HGNC:13746]
ENSG00000110148	CCKBR	-3.284008674	1.322005867	6.70E-32	1.25E-29	cholecystokinin B receptor [Source:HGNC Symbol;Acc:HGNC:1571]
ENSG00000089250	NOS1	-3.242746207	1.14739716	2.90E-29	4.28E-27	nitric oxide synthase 1 [Source:HGNC Symbol;Acc:HGNC:7872]
ENSG00000116147	TNR	-3.232090209	3.784364359	5.39E-17	2.80E-15	tenascin R [Source:HGNC Symbol;Acc:HGNC:11953]
ENSG00000145864	GABRB2	-3.220296389	4.0020232	2.90E-19	1.84E-17	gamma-aminobutyric acid type A receptor subunit beta2 [Source:HGNC Symbol;Acc:HGNC:4082]
ENSG00000116183	PAPPA2	-3.217549351	2.879253251	3.58E-36	9.23E-34	pappalysin 2 [Source:HGNC Symbol;Acc:HGNC:14615]
ENSG00000171246	NPTX1	-3.212400411	4.600839343	4.93E-50	2.47E-47	neuronal pentraxin 1 [Source:HGNC Symbol;Acc:HGNC:7952]
ENSG00000167178	ISLR2	-3.204371374	6.723815276	1.48E-26	1.79E-24	immunoglobulin superfamily containing leucine rich repeat 2 [Source:HGNC Symbol;Acc:HGNC:29286]
ENSG00000188517	COL25A1	-3.194365347	3.866291243	2.45E-72	4.33E-69	collagen type XXV alpha 1 chain [Source:HGNC Symbol;Acc:HGNC:18603]
ENSG00000155886	SLC24A2	-3.171579718	2.285462993	1.22E-42	4.27E-40	solute carrier family 24 member 2 [Source:HGNC Symbol;Acc:HGNC:10976]
ENSG00000101187	SLCO4A1	-3.122596355	2.121308935	1.85E-45	7.78E-43	solute carrier organic anion transporter family member 4A1 [Source:HGNC Symbol;Acc:HGNC:10953]
ENSG00000101188	NTSR1	-3.117091839	3.274476184	1.10E-48	5.02E-46	neurotensin receptor 1 [Source:HGNC Symbol;Acc:HGNC:8039]
ENSG00000235849	HS6ST2-AS1	-3.095358013	0.999290247	3.43E-40	1.10E-37	HS6ST2 antisense RNA 1 [Source:HGNC Symbol;Acc:HGNC:40870]
ENSG00000158560	DYNC1I1	-3.077956656	3.99108417	1.23E-46	5.36E-44	dynein cytoplasmic 1 intermediate chain 1 [Source:HGNC Symbol;Acc:HGNC:2963]
ENSG00000118785	SPP1	-3.07747484	1.174357551	7.42E-18	4.21E-16	secreted phosphoprotein 1 [Source:HGNC Symbol;Acc:HGNC:11255]
ENSG00000133687	TMTC1	-3.045424277	3.972121416	6.01E-66	7.98E-63	transmembrane O-mannosyltransferase targeting cadherins 1 [Source:HGNC Symbol;Acc:HGNC:24099]
ENSG00000127152	BCL11B	-3.037820034	4.731072401	2.68E-10	6.34E-09	BAF chromatin remodeling complex subunit BCL11B [Source:HGNC Symbol;Acc:HGNC:13222]
ENSG00000249896	JAKMIP1-DT	-3.024653385	0.762706441	6.22E-29	8.97E-27	JAKMIP1 divergent transcript [Source:HGNC Symbol;Acc:HGNC:27728]
ENSG00000198915	RASGEF1A	-3.022461451	1.478457136	1.49E-26	1.80E-24	RasGEF domain family member 1A [Source:HGNC Symbol;Acc:HGNC:24246]
ENSG00000066248	NGEF	-3.019571836	2.56843285	5.08E-49	2.41E-46	neuronal guanine nucleotide exchange factor [Source:HGNC Symbol;Acc:HGNC:7807]
ENSG00000136267	DGKB	-3.01524862	3.916922323	6.40E-51	3.27E-48	diacylglycerol kinase beta [Source:HGNC Symbol;Acc:HGNC:2850]
ENSG00000120457	KCNJ5	-3.004444608	0.917819999	4.43E-19	2.74E-17	potassium inwardly rectifying channel subfamily J member 5 [Source:HGNC Symbol;Acc:HGNC:6266]
ENSG00000177699	None	-2.997757043	0.857311681	7.50E-35	1.73E-32	novel transcript
ENSG00000253859	None	-2.99540536	1.173433178	8.43E-27	1.05E-24	novel transcript
ENSG00000272636	DOC2B	-2.956242296	1.765983364	1.14E-23	1.11E-21	double C2 domain beta [Source:HGNC Symbol;Acc:HGNC:2986]
ENSG00000127399	LRRC61	-2.945698643	2.023230175	7.51E-11	1.91E-09	leucine rich repeat containing 61 [Source:HGNC Symbol;Acc:HGNC:21704]
ENSG00000158816	VWA5B1	-2.916259781	0.323285963	1.61E-32	3.16E-30	von Willebrand factor A domain containing 5B1 [Source:HGNC Symbol;Acc:HGNC:26538]
ENSG00000115616	SLC9A2	-2.911667685	2.450507426	1.10E-30	1.80E-28	solute carrier family 9 member A2 [Source:HGNC Symbol;Acc:HGNC:11072]
ENSG00000075035	WSCD2	-2.90897042	2.851848696	1.28E-28	1.81E-26	WSC domain containing 2 [Source:HGNC Symbol;Acc:HGNC:29117]
ENSG00000232675	None	-2.900210306	0.788106797	6.40E-22	5.25E-20	novel transcript

ENSG00000259240	MIR4713HG	-2.89753233	1.215941733	1.62E-18	9.63E-17	MIR4713 host gene [Source:HGNC Symbol;Acc:HGNC:53124]
ENSG00000198010	DLGAP2	-2.889141031	1.832374375	1.56E-24	1.64E-22	DLG associated protein 2 [Source:HGNC Symbol;Acc:HGNC:2906]
ENSG00000042493	CAPG	-2.888689874	0.226416954	1.14E-23	1.11E-21	capping actin protein
ENSG00000073331	ALPK1	-2.884141343	1.845047151	5.89E-32	1.10E-29	alpha kinase 1 [Source:HGNC Symbol;Acc:HGNC:20917]
ENSG00000006128	TAC1	-2.878469372	2.991970731	6.36E-43	2.28E-40	tachykinin precursor 1 [Source:HGNC Symbol;Acc:HGNC:11517]
ENSG00000182103	FAM181B	-2.876259974	3.917157395	2.70E-24	2.75E-22	family with sequence similarity 181 member B [Source:HGNC Symbol;Acc:HGNC:28512]
ENSG00000171004	HS6ST2	-2.870582528	5.846526607	2.55E-109	1.70E-105	heparan sulfate 6-O-sulfotransferase 2 [Source:HGNC Symbol;Acc:HGNC:19133]
ENSG00000186377	CYP4X1	-2.860923437	2.2730172	1.48E-22	1.28E-20	cytochrome P450 family 4 subfamily X member 1 [Source:HGNC Symbol;Acc:HGNC:20244]
ENSG00000259168	None	-2.833286477	1.308302946	2.52E-24	2.57E-22	novel transcript
ENSG00000113763	UNC5A	-2.833194398	2.786650544	2.92E-34	6.37E-32	unc-5 netrin receptor A [Source:HGNC Symbol;Acc:HGNC:12567]
ENSG00000185742	C11orf87	-2.832798197	3.546119026	1.51E-16	7.45E-15	chromosome 11 open reading frame 87 [Source:HGNC Symbol;Acc:HGNC:33788]
ENSG00000240065	PSMB9	-2.827309732	1.185898696	4.90E-32	9.37E-30	proteasome 20S subunit beta 9 [Source:HGNC Symbol;Acc:HGNC:9546]
ENSG00000197971	MBP	-2.826925586	1.326259837	2.43E-33	4.86E-31	myelin basic protein [Source:HGNC Symbol;Acc:HGNC:6925]
ENSG00000266248	None	-2.826000662	1.318109628	5.08E-16	2.37E-14	novel transcript
ENSG00000253041	None	-2.818775033	0.382056716	6.00E-22	4.95E-20	None
ENSG00000267633	None	-2.763341853	1.390568627	1.36E-22	1.18E-20	novel transcript
ENSG00000136297	MMD2	-2.750993609	1.26528195	5.75E-26	6.68E-24	monocyte to macrophage differentiation associated 2 [Source:HGNC Symbol;Acc:HGNC:30133]
ENSG00000165731	RET	-2.747411605	3.084627236	1.22E-33	2.48E-31	ret proto-oncogene [Source:HGNC Symbol;Acc:HGNC:9967]
ENSG00000101938	CHRDL1	-2.737221401	5.374314198	1.57E-60	1.55E-57	chordin like 1 [Source:HGNC Symbol;Acc:HGNC:29861]
ENSG00000196353	CPNE4	-2.734095077	3.089383496	8.96E-24	8.81E-22	copine 4 [Source:HGNC Symbol;Acc:HGNC:2317]
ENSG00000168394	TAP1	-2.731121738	1.086699669	5.08E-30	7.94E-28	transporter 1
ENSG00000107742	SPOCK2	-2.729104854	5.420520271	5.30E-53	3.20E-50	SPARC (osteonectin)
ENSG00000127252	PLAAT1	-2.7219901	1.583515302	6.35E-31	1.08E-28	phospholipase A and acyltransferase 1 [Source:HGNC Symbol;Acc:HGNC:14922]
ENSG00000136999	CCN3	-2.72144734	0.935188082	8.07E-21	5.86E-19	cellular communication network factor 3 [Source:HGNC Symbol;Acc:HGNC:7885]
ENSG00000185518	SV2B	-2.720709843	1.631068832	1.14E-20	8.21E-19	synaptic vesicle glycoprotein 2B [Source:HGNC Symbol;Acc:HGNC:16874]
ENSG00000122574	WIPF3	-2.720598599	3.244226749	1.18E-22	1.03E-20	WAS/WASL interacting protein family member 3 [Source:HGNC Symbol;Acc:HGNC:22004]
ENSG00000167964	RAB26	-2.716859446	2.314127973	6.05E-31	1.03E-28	RAB26
ENSG00000223563	None	-2.703699552	0.819906617	3.52E-24	3.56E-22	novel transcript
ENSG00000264515	VSTM2B-DT	-2.69706509	1.619724726	4.36E-18	2.52E-16	VSTM2B divergent transcript [Source:HGNC Symbol;Acc:HGNC:27615]
ENSG00000007237	GAS7	-2.692053662	5.010034346	1.73E-67	2.55E-64	growth arrest specific 7 [Source:HGNC Symbol;Acc:HGNC:4169]
ENSG00000037757	MRI1	-2.690874585	2.693701545	3.92E-20	2.72E-18	methylthioribose-1-phosphate isomerase 1 [Source:HGNC Symbol;Acc:HGNC:28469]
ENSG00000004846	ABCB5	-2.674466262	0.564795036	1.44E-19	9.41E-18	ATP binding cassette subfamily B member 5 [Source:HGNC Symbol;Acc:HGNC:46]
ENSG00000171951	SCG2	-2.667172719	5.498085618	7.26E-31	1.21E-28	secretogranin II [Source:HGNC Symbol;Acc:HGNC:10575]
ENSG00000137252	HCRTR2	-2.667055226	0.537517747	3.97E-22	3.33E-20	hypocretin receptor 2 [Source:HGNC Symbol;Acc:HGNC:4849]
ENSG00000139318	DUSP6	-2.666400741	5.007457218	3.83E-25	4.23E-23	dual specificity phosphatase 6 [Source:HGNC Symbol;Acc:HGNC:3072]
ENSG00000151062	CACNA2D4	-2.664406774	0.488377064	3.21E-18	1.87E-16	calcium voltage-gated channel auxiliary subunit alpha2delta 4 [Source:HGNC Symbol;Acc:HGNC:20202]
ENSG00000163673	DCLK3	-2.663778717	0.72322133	8.93E-17	4.55E-15	doublecortin like kinase 3 [Source:HGNC Symbol;Acc:HGNC:19005]
ENSG00000204103	MAFB	-2.658373265	2.561514633	5.40E-31	9.38E-29	MAF bZIP transcription factor B [Source:HGNC Symbol;Acc:HGNC:6408]
ENSG00000146592	CREB5	-2.658279915	5.588056826	8.35E-36	2.05E-33	cAMP responsive element binding protein 5 [Source:HGNC Symbol;Acc:HGNC:16844]
ENSG00000154736	ADAMTS5	-2.653165924	1.137798438	2.42E-23	2.27E-21	ADAM metallopeptidase with thrombospondin type 1 motif 5 [Source:HGNC Symbol;Acc:HGNC:221]
ENSG00000228659	LINC01201	-2.649456544	0.640999335	3.40E-29	4.99E-27	long intergenic non-protein coding RNA 1201 [Source:HGNC Symbol;Acc:HGNC:49632]
ENSG00000100302	RASD2	-2.648813424	1.973748367	4.33E-29	6.32E-27	RASD family member 2 [Source:HGNC Symbol;Acc:HGNC:18229]
ENSG00000074211	PPP2R2C	-2.646100913	4.66719185	1.27E-18	7.68E-17	protein phosphatase 2 regulatory subunit Bgamma [Source:HGNC Symbol;Acc:HGNC:9306]
ENSG00000168490	PHYHIP	-2.640839693	3.219379292	1.07E-37	3.02E-35	phytanoyl-CoA 2-hydroxylase interacting protein [Source:HGNC Symbol;Acc:HGNC:16865]
ENSG00000165983	PTER	-2.640124685	0.101728526	9.93E-22	7.97E-20	phosphotriesterase related [Source:HGNC Symbol;Acc:HGNC:9590]
ENSG00000268266	None	-2.632815827	1.603154676	1.72E-33	3.49E-31	novel transcript
ENSG00000117600	PLPPR4	-2.626659981	3.475106823	7.61E-28	1.01E-25	phospholipid phosphatase related 4 [Source:HGNC Symbol;Acc:HGNC:23496]
ENSG00000117266	CDK18	-2.623327835	2.810767212	8.31E-17	4.26E-15	cyclin dependent kinase 18 [Source:HGNC Symbol;Acc:HGNC:8751]
ENSG00000231437	LINC01750	-2.621841482	1.303042701	1.08E-23	1.05E-21	long intergenic non-protein coding RNA 1750 [Source:HGNC Symbol;Acc:HGNC:52538]
ENSG00000169116	PARM1	-2.617930306	3.004208193	2.20E-27	2.83E-25	prostate androgen-regulated mucin-like protein 1 [Source:HGNC Symbol;Acc:HGNC:24536]
ENSG00000148123	PLPPR1	-2.616979804	5.041542183	3.53E-40	1.12E-37	phospholipid phosphatase related 1 [Source:HGNC Symbol;Acc:HGNC:25993]
ENSG00000198739	LRRTM3	-2.615606007	4.078951093	3.26E-54	2.28E-51	leucine rich repeat transmembrane neuronal 3 [Source:HGNC Symbol;Acc:HGNC:19410]
ENSG00000248245	None	-2.606303707	2.75464656	3.68E-36	9.40E-34	novel transcript
ENSG00000171385	KCND3	-2.598555091	4.766968804	1.32E-29	2.01E-27	potassium voltage-gated channel subfamily D member 3 [Source:HGNC Symbol;Acc:HGNC:6239]
ENSG00000163288	GABRB1	-2.598393907	1.990568902	4.48E-18	2.58E-16	gamma-aminobutyric acid type A receptor subunit beta1 [Source:HGNC Symbol;Acc:HGNC:4081]
ENSG00000144681	STAC	-2.596782331	0.91793225	1.06E-15	4.80E-14	SH3 and cysteine rich domain [Source:HGNC Symbol;Acc:HGNC:11353]
ENSG00000183114	FAM43B	-2.594419555	1.91949205	7.95E-26	9.14E-24	family with sequence similarity 43 member B [Source:HGNC Symbol;Acc:HGNC:31791]
ENSG00000224514	LINC00620	-2.590624081	0.304902656	2.45E-24	2.51E-22	long intergenic non-protein coding RNA 620 [Source:HGNC Symbol;Acc:HGNC:44223]
ENSG00000261649	GOLGA6L7	-2.590086087	-0.126736911	5.29E-27	6.72E-25	golgin A6 family like 7 [Source:HGNC Symbol;Acc:HGNC:37442]
ENSG00000172137	CALB2	-2.586326369	4.735458741	1.03E-15	4.66E-14	calbindin 2 [Source:HGNC Symbol;Acc:HGNC:1435]
ENSG00000122254	HS3ST2	-2.58017692	1.036767149	1.86E-26	2.24E-24	heparan sulfate-glucosamine 3-sulfotransferase 2 [Source:HGNC Symbol;Acc:HGNC:5195]
ENSG00000092758	COL9A3	-2.576354054	2.12556088	7.28E-27	9.12E-25	collagen type IX alpha 3 chain [Source:HGNC Symbol;Acc:HGNC:2219]
ENSG00000136997	MYC	-2.571765936	3.278470188	1.99E-21	1.53E-19	MYC proto-oncogene
ENSG00000125851	PCSK2	-2.566754152	4.319024823	4.21E-48	1.89E-45	proprotein convertase subtilisin/kexin type 2 [Source:HGNC Symbol;Acc:HGNC:8744]
ENSG00000185519	FAM131C	-2.564868169	0.777082131	4.11E-14	1.54E-12	family with sequence similarity 131 member C [Source:HGNC Symbol;Acc:HGNC:26717]
ENSG00000233005	None	-2.557854045	0.888595696	1.09E-12	3.53E-11	novel transcript
ENSG00000174951	FUT1	-2.555843119	-0.529794119	7.70E-25	8.25E-23	fucosyltransferase 1 (H blood group) [Source:HGNC Symbol;Acc:HGNC:4012]
ENSG00000257456	None	-2.549659952	-0.026633194	1.11E-19	7.35E-18	novel transcript
ENSG00000214688	C10orf105	-2.549654554	2.702008963	2.65E-29	3.95E-27	chromosome 10 open reading frame 105 [Source:HGNC Symbol;Acc:HGNC:20304]
ENSG00000131979	GCH1	-2.536138363	1.015105192	2.81E-28	3.89E-26	GTP cyclohydrolase 1 [Source:HGNC Symbol;Acc:HGNC:4193]
ENSG00000259031	None	-2.525792196	1.256970521	2.38E-30	3.80E-28	novel transcript
ENSG00000244953	None	-2.524512018	-0.315562074	1.11E-23	1.08E-21	novel transcript
ENSG00000233251	None	-2.52363156	1.034688632	2.26E-19	1.45E-17	novel transcript
ENSG00000125845	BMP2	-2.520130933	1.415042871	1.15E-27	1.51E-25	bone morphogenetic protein 2 [Source:HGNC Symbol;Acc:HGNC:1069]
ENSG00000107338	SHB	-2.516513369	4.194645507	3.76E-51	2.00E-48	SH2 domain containing adaptor protein B [Source:HGNC Symbol;Acc:HGNC:10838]
ENSG00000259234	ANKRD34C-AS1	-2.516152596	-0.143273726	3.50E-25	3.89E-23	ANKRD34C antisense RNA 1 [Source:HGNC Symbol;Acc:HGNC:48618]
ENSG00000188848	BEND4	-2.512782905	3.377563329	2.24E-26	2.68E-24	BEN domain containing 4 [Source:HGNC Symbol;Acc:HGNC:23815]
ENSG00000116194	ANGPTL1	-2.512286447	2.406412268	1.69E-27	2.19E-25	angiopoietin like 1 [Source:HGNC Symbol;Acc:HGNC:489]
ENSG00000164483	SAMD3	-2.510026317	2.022956394	6.55E-25	7.07E-23	sterile alpha motif domain containing 3 [Source:HGNC Symbol;Acc:HGNC:21574]
ENSG00000111907	TPD52L1	-2.504573436	1.489771548	7.49E-23	6.70E-21	TPD52 like 1 [Source:HGNC Symbol;Acc:HGNC:12006]
ENSG00000122733	PHF24	-2.502237168	3.024033332	3.51E-15	1.50E-13	PHD finger protein 24 [Source:HGNC Symbol;Acc:HGNC:29180]
ENSG00000229953	BCAN-AS2	-2.49938168	4.307464346	3.76E-05	0.000335898	BCAN antisense RNA 2 [Source:HGNC Symbol;Acc:HGNC:56267]
ENSG00000230006	ANKRD36BP2	-2.492425312	1.173701195	4.35E-21	3.26E-19	ankyrin repeat domain 36B pseudogene 2 [Source:HGNC Symbol;Acc:HGNC:33607]
ENSG00000187135	VSTM2B	-2.489995637	2.663678429	1.91E-13	6.73E-12	V-set and transmembrane domain containing 2B [Source:HGNC Symbol;Acc:HGNC:33595]
ENSG00000147676	MAL2	-2.480375542	2.311022624	1.74E-32	3.39E-30	mal
ENSG00000185052	SLC24A3	-2.478172572	3.789968022	2.40E-20	1.70E-18	solute carrier family 24 member 3 [Source:HGNC Symbol;Acc:HGNC:10977]
ENSG00000258871	None	-2.477247182	1.366280037	7.05E-17	3.62E-15	novel transcript
ENSG00000179292	TMEM151A	-2.474725422	2.460878404	2.87E-23	2.66E-21	transmembrane protein 151A [Source:HGNC Symbol;Acc:HGNC:28497]
ENSG00000107736	CDH23	-2.470337834	5.361050653	1.05E-35	2.57E-33	cadherin related 23 [Source:HGNC Symbol;Acc:HGNC:13733]
ENSG00000253972	MAL2-AS1	-2.465165965	2.197118179	8.94E-31	1.48E-28	MAL2 antisense RNA 1 [Source:HGNC Symbol;Acc:HGNC:53626]

ENSG00000137959	IFI44L	-2.465156305	3.228539646	6.91E-07	9.13E-06	interferon induced protein 44 like [Source:HGNC Symbol;Acc:HGNC:17817]
ENSG00000006468	ETV1	-2.458065867	5.570958209	2.19E-22	1.86E-20	ETS variant transcription factor 1 [Source:HGNC Symbol;Acc:HGNC:3490]
ENSG00000189056	RELN	-2.457490121	6.670019045	5.29E-25	5.80E-23	reelin [Source:HGNC Symbol;Acc:HGNC:9957]
ENSG00000132692	BCAN	-2.454868705	4.403757007	1.79E-05	0.000174654	brevican [Source:HGNC Symbol;Acc:HGNC:23059]
ENSG00000235244	DANT2	-2.447177237	4.09338571	2.07E-11	5.71E-10	DXZ4 associated non-coding transcript 2
ENSG00000197635	DPP4	-2.443431934	0.382337995	2.57E-18	1.51E-16	dipeptidyl peptidase 4 [Source:HGNC Symbol;Acc:HGNC:3009]
ENSG00000253398	None	-2.439941542	0.13876775	2.46E-19	1.57E-17	novel transcript
ENSG00000150594	ADRA2A	-2.434675278	3.285542398	9.55E-32	1.74E-29	adrenoceptor alpha 2A [Source:HGNC Symbol;Acc:HGNC:281]
ENSG00000255028	None	-2.434432051	3.862462348	7.68E-19	4.68E-17	novel transcript
ENSG00000179542	SLITRK4	-2.432103481	3.339267804	3.96E-23	3.61E-21	SLIT and NTRK like family member 4 [Source:HGNC Symbol;Acc:HGNC:23502]
ENSG00000187479	C11orf96	-2.425425192	3.438481695	1.75E-39	5.27E-37	chromosome 11 open reading frame 96 [Source:HGNC Symbol;Acc:HGNC:38675]
ENSG00000160932	LY6E	-2.423951511	3.080446094	2.75E-30	4.37E-28	lymphocyte antigen 6 family member E [Source:HGNC Symbol;Acc:HGNC:6727]
ENSG00000144290	SLC4A10	-2.423539543	6.118601214	2.00E-22	1.71E-20	solute carrier family 4 member 10 [Source:HGNC Symbol;Acc:HGNC:13811]
ENSG00000100346	CACNA1I	-2.40892708	3.305778508	7.01E-34	1.44E-31	calcium voltage-gated channel subunit alpha1 I [Source:HGNC Symbol;Acc:HGNC:1396]
ENSG00000175906	ARL4D	-2.406418105	4.162275066	1.44E-44	5.53E-42	ADP ribosylation factor like GTPase 4D [Source:HGNC Symbol;Acc:HGNC:656]
ENSG00000198133	TMEM229B	-2.401482852	3.287532073	2.12E-16	1.03E-14	transmembrane protein 229B [Source:HGNC Symbol;Acc:HGNC:20130]
ENSG00000113327	GABRG2	-2.400575859	3.540931273	5.55E-25	6.07E-23	gamma-aminobutyric acid type A receptor subunit gamma2 [Source:HGNC Symbol;Acc:HGNC:4087]
ENSG00000004399	PLXND1	-2.392088141	4.084834647	5.01E-21	3.71E-19	plexin D1 [Source:HGNC Symbol;Acc:HGNC:9107]
ENSG00000132561	MATN2	-2.391453782	3.712453058	2.87E-34	6.30E-32	matrilin 2 [Source:HGNC Symbol;Acc:HGNC:6908]
ENSG00000172889	EGFL7	-2.38627377	0.963180777	6.39E-24	6.36E-22	EGF like domain multiple 7 [Source:HGNC Symbol;Acc:HGNC:20594]
ENSG00000116254	CHD5	-2.380923169	5.684989002	2.90E-06	3.37E-05	chromodomain helicase DNA binding protein 5 [Source:HGNC Symbol;Acc:HGNC:16816]
ENSG00000198108	CHSY3	-2.37942634	1.477197238	1.91E-21	1.48E-19	chondroitin sulfate synthase 3 [Source:HGNC Symbol;Acc:HGNC:24293]
ENSG00000234715	SLC26A5-AS1	-2.377814111	5.235446936	1.39E-28	1.97E-26	SLC26A5 antisense RNA 1 [Source:HGNC Symbol;Acc:HGNC:55680]
ENSG00000236333	TRHDE-AS1	-2.369756207	2.842381175	4.42E-23	4.01E-21	TRHDE antisense RNA 1 [Source:HGNC Symbol;Acc:HGNC:27471]
ENSG00000121310	ECHDC2	-2.36717628	1.533027088	1.35E-19	8.88E-18	enoyl-CoA hydratase domain containing 2 [Source:HGNC Symbol;Acc:HGNC:23408]
ENSG00000182870	GALNT9	-2.366901306	3.258966748	8.44E-19	5.13E-17	polypeptide N-acetylgalactosaminyltransferase 9 [Source:HGNC Symbol;Acc:HGNC:4131]
ENSG00000100985	MMP9	-2.352727527	0.700898385	1.09E-21	8.67E-20	matrix metallopeptidase 9 [Source:HGNC Symbol;Acc:HGNC:7176]
ENSG00000162975	KCNF1	-2.350345358	2.773966939	2.24E-30	3.60E-28	potassium voltage-gated channel modifier subfamily F member 1 [Source:HGNC Symbol;Acc:HGNC:6246]
ENSG00000221866	PLXNA4	-2.347951164	6.320438196	2.23E-12	6.87E-11	plexin A4 [Source:HGNC Symbol;Acc:HGNC:9102]
ENSG00000151952	TMEM132D	-2.346764348	2.340718655	8.17E-06	8.63E-05	transmembrane protein 132D [Source:HGNC Symbol;Acc:HGNC:29411]
ENSG00000139874	SSTR1	-2.342583481	1.703427133	1.05E-10	2.62E-09	somatostatin receptor 1 [Source:HGNC Symbol;Acc:HGNC:11330]
ENSG00000136104	RNASEH2B	-2.341903867	3.255994499	8.78E-27	1.08E-24	ribonuclease H2 subunit B [Source:HGNC Symbol;Acc:HGNC:25671]
ENSG00000175206	NPPA	-2.340375376	0.183252708	1.52E-16	7.47E-15	natriuretic peptide A [Source:HGNC Symbol;Acc:HGNC:7939]
ENSG00000144847	IGSF11	-2.338419205	2.921783153	1.56E-34	3.52E-32	immunoglobulin superfamily member 11 [Source:HGNC Symbol;Acc:HGNC:16669]
ENSG00000154027	AK5	-2.337608478	2.28513582	7.50E-22	6.13E-20	adenylate kinase 5 [Source:HGNC Symbol;Acc:HGNC:365]
ENSG00000248995	LINC02231	-2.327094122	0.830453039	2.13E-10	5.11E-09	long intergenic non-protein coding RNA 2231 [Source:HGNC Symbol;Acc:HGNC:53100]
ENSG00000072657	TRHDE	-2.325192447	3.755158013	4.91E-22	4.09E-20	thyrotropin releasing hormone degrading enzyme [Source:HGNC Symbol;Acc:HGNC:30748]
ENSG00000053108	FSTL4	-2.324433996	3.001063489	5.81E-31	9.95E-29	follistatin like 4 [Source:HGNC Symbol;Acc:HGNC:21389]
ENSG00000267280	TBX2-AS1	-2.321221468	-0.067104624	1.37E-23	1.31E-21	TBX2 antisense RNA 1 [Source:HGNC Symbol;Acc:HGNC:50355]
ENSG00000175426	PCSK1	-2.31658815	3.433280446	1.16E-22	1.01E-20	proprotein convertase subtilisin/kexin type 1 [Source:HGNC Symbol;Acc:HGNC:8743]
ENSG00000137460	FHDC1	-2.310640211	2.658107495	5.70E-28	7.65E-26	FH2 domain containing 1 [Source:HGNC Symbol;Acc:HGNC:29363]
ENSG00000165169	DYNLT3	-2.308007841	2.144179262	2.84E-23	2.63E-21	dynein light chain Tctex-type 3 [Source:HGNC Symbol;Acc:HGNC:11694]
ENSG00000131386	GALNT15	-2.30681088	0.777731364	1.19E-17	6.67E-16	polypeptide N-acetylgalactosaminyltransferase 15 [Source:HGNC Symbol;Acc:HGNC:21531]
ENSG00000148488	ST8SIA6	-2.305658626	-0.168172321	1.10E-17	6.21E-16	ST8 alpha-N-acetyl-neuraminide alpha-2
ENSG00000078725	BRINP1	-2.304967788	4.549059634	1.44E-44	5.53E-42	BMP/retinoic acid inducible neural specific 1 [Source:HGNC Symbol;Acc:HGNC:2687]
ENSG00000213047	DENND1B	-2.303819555	3.410720325	1.33E-35	3.19E-33	DENN domain containing 1B [Source:HGNC Symbol;Acc:HGNC:28404]
ENSG00000152377	SPOCK1	-2.302235553	4.710352117	2.02E-14	7.85E-13	SPARC (osteonectin)
ENSG00000074317	SNCB	-2.299823134	3.400610503	4.34E-24	4.37E-22	synuclein beta [Source:HGNC Symbol;Acc:HGNC:11140]
ENSG00000171587	DSCAM	-2.286242916	4.47766905	7.78E-25	8.30E-23	DS cell adhesion molecule [Source:HGNC Symbol;Acc:HGNC:3039]
ENSG00000215612	HMX1	-2.284729253	0.811081868	2.33E-13	8.16E-12	H6 family homeobox 1 [Source:HGNC Symbol;Acc:HGNC:5017]
ENSG00000183092	BEGAIN	-2.284548788	5.139780788	1.60E-38	4.77E-36	brain enriched guanylate kinase associated [Source:HGNC Symbol;Acc:HGNC:24163]
ENSG00000091490	SEL1L3	-2.282455542	3.285956983	3.28E-24	3.32E-22	SEL1L family member 3 [Source:HGNC Symbol;Acc:HGNC:29108]
ENSG00000254733	None	-2.282139186	2.714186066	3.03E-31	5.37E-29	novel transcript
ENSG00000126822	PLEKHG3	-2.279121581	2.26606785	1.47E-21	1.16E-19	pleckstrin homology and RhoGEF domain containing G3 [Source:HGNC Symbol;Acc:HGNC:20364]
ENSG00000147481	SNTG1	-2.276955397	4.65174622	3.25E-28	4.42E-26	syntrophin gamma 1 [Source:HGNC Symbol;Acc:HGNC:13740]
ENSG00000147246	HTR2C	-2.274895094	0.614539352	7.59E-19	4.64E-17	5-hydroxytryptamine receptor 2C [Source:HGNC Symbol;Acc:HGNC:5295]
ENSG00000214772	GUCY2C-AS1	-2.274338016	-0.197979744	1.56E-21	1.22E-19	GUCY2C antisense RNA 1 [Source:HGNC Symbol;Acc:HGNC:56054]
ENSG00000101542	CDH20	-2.271976438	4.480610296	3.06E-45	1.27E-42	cadherin 20 [Source:HGNC Symbol;Acc:HGNC:1760]
ENSG00000100311	PDGFB	-2.271277878	1.042046052	1.34E-21	1.07E-19	platelet derived growth factor subunit B [Source:HGNC Symbol;Acc:HGNC:8800]
ENSG00000134207	SYT6	-2.270369596	3.46552947	6.51E-37	1.75E-34	synaptotagmin 6 [Source:HGNC Symbol;Acc:HGNC:18638]
ENSG00000107829	FBXW4	-2.264847159	3.009149106	9.10E-31	1.50E-28	F-box and WD repeat domain containing 4 [Source:HGNC Symbol;Acc:HGNC:10847]
ENSG00000072315	TRPC5	-2.260123912	1.513465498	5.28E-23	4.77E-21	transient receptor potential cation channel subfamily C member 5 [Source:HGNC Symbol;Acc:HGNC:12337]
ENSG00000133519	ZDHHC8BP	-2.258867006	2.234480112	6.37E-21	4.67E-19	ZDHHC8B
ENSG00000106341	PPP1R17	-2.257728632	1.944860055	3.78E-07	5.25E-06	protein phosphatase 1 regulatory subunit 17 [Source:HGNC Symbol;Acc:HGNC:16973]
ENSG00000254409	None	-2.256353624	3.559115436	4.35E-34	9.23E-32	novel transcript
ENSG00000145920	CPLX2	-2.254124109	7.625399081	3.21E-25	3.58E-23	complexin 2 [Source:HGNC Symbol;Acc:HGNC:2310]
ENSG00000148600	CDHR1	-2.25095393	3.269282634	4.99E-26	5.81E-24	cadherin related family member 1 [Source:HGNC Symbol;Acc:HGNC:14550]
ENSG00000081138	CDH7	-2.248868236	2.857924037	4.14E-15	1.74E-13	cadherin 7 [Source:HGNC Symbol;Acc:HGNC:1766]
ENSG00000138944	SHISAL1	-2.245102345	5.370383108	7.65E-49	3.57E-46	shisa like 1 [Source:HGNC Symbol;Acc:HGNC:29335]
ENSG00000183654	MARCHF11	-2.245026476	1.130420905	1.68E-16	8.24E-15	membrane associated ring-CH-type finger 11 [Source:HGNC Symbol;Acc:HGNC:33609]
ENSG00000085741	WNT11	-2.243670235	0.680011151	1.58E-10	3.86E-09	Wnt family member 11 [Source:HGNC Symbol;Acc:HGNC:12776]
ENSG00000099260	PALMD	-2.24334898	2.871384017	1.03E-24	1.10E-22	palmdelphin [Source:HGNC Symbol;Acc:HGNC:15846]
ENSG00000145247	OCIAD2	-2.242672091	3.242266759	1.79E-18	1.06E-16	OCIA domain containing 2 [Source:HGNC Symbol;Acc:HGNC:28685]
ENSG00000241158	ADAMTS9-AS1	-2.242526649	-0.372827031	1.67E-17	9.30E-16	ADAMTS9 antisense RNA 1 [Source:HGNC Symbol;Acc:HGNC:40625]
ENSG00000181656	GPR88	-2.240224523	0.305100418	8.92E-19	5.41E-17	G protein-coupled receptor 88 [Source:HGNC Symbol;Acc:HGNC:4539]
ENSG00000214546	None	-2.238559525	-0.593798813	5.89E-19	3.61E-17	novel transcript
ENSG00000140280	LYSMD2	-2.237487718	1.029354762	3.21E-16	1.53E-14	LysM domain containing 2 [Source:HGNC Symbol;Acc:HGNC:28571]
ENSG00000165186	PTCHD1	-2.236730225	2.248844554	4.59E-11	1.20E-09	patched domain containing 1 [Source:HGNC Symbol;Acc:HGNC:26392]
ENSG00000137965	IFI44	-2.236216867	3.134941023	3.28E-06	3.76E-05	interferon induced protein 44 [Source:HGNC Symbol;Acc:HGNC:16938]
ENSG00000168993	CPLX1	-2.234600395	3.562479759	2.05E-17	1.12E-15	complexin 1 [Source:HGNC Symbol;Acc:HGNC:2309]
ENSG00000181234	TMEM132C	-2.231419478	2.053450138	2.92E-15	1.26E-13	transmembrane protein 132C [Source:HGNC Symbol;Acc:HGNC:25436]
ENSG00000189108	IL1RAPL2	-2.231403982	2.901336325	1.46E-23	1.39E-21	interleukin 1 receptor accessory protein like 2 [Source:HGNC Symbol;Acc:HGNC:5997]
ENSG00000130813	SHFL	-2.2273704	2.025084013	9.35E-22	7.58E-20	shiftless antiviral inhibitor of ribosomal frameshifting [Source:HGNC Symbol;Acc:HGNC:25649]
ENSG00000164434	FABP7	-2.227270716	6.610595829	2.80E-07	3.96E-06	fatty acid binding protein 7 [Source:HGNC Symbol;Acc:HGNC:3562]
ENSG00000169891	REPS2	-2.225920281	2.590810731	2.22E-30	3.59E-28	RALBP1 associated Eps domain containing 2 [Source:HGNC Symbol;Acc:HGNC:9963]
ENSG00000164649	CDCA7L	-2.225816387	4.625999894	4.39E-43	1.60E-40	cell division cycle associated 7 like [Source:HGNC Symbol;Acc:HGNC:30777]
ENSG00000109794	FAM149A	-2.221642513	3.566178619	2.10E-38	6.12E-36	family with sequence similarity 149 member A [Source:HGNC Symbol;Acc:HGNC:24527]
ENSG00000100593	ISM2	-2.216231567	0.064298296	8.24E-15	3.36E-13	isthmin 2 [Source:HGNC Symbol;Acc:HGNC:23176]
ENSG00000159640	ACE	-2.215043777	-0.060465462	1.26E-16	6.29E-15	angiotensin I converting enzyme [Source:HGNC Symbol;Acc:HGNC:2707]
ENSG00000151376	ME3	-2.208090215	2.618527075	7.86E-28	1.04E-25	malic enzyme 3 [Source:HGNC Symbol;Acc:HGNC:6985]

ENSG00000141485	SLC13A5	-2.202346552	-0.174250828	4.74E-22	3.96E-20	solute carrier family 13 member 5 [Source:HGNC Symbol;Acc:HGNC:23089]
ENSG00000136928	GABBR2	-2.191197605	4.655077684	4.69E-19	2.89E-17	gamma-aminobutyric acid type B receptor subunit 2 [Source:HGNC Symbol;Acc:HGNC:4507]
ENSG00000152969	JAKMIP1	-2.190771719	3.869282732	2.34E-14	9.01E-13	janus kinase and microtubule interacting protein 1 [Source:HGNC Symbol;Acc:HGNC:26460]
ENSG00000260807	CEROX1	-2.18974688	4.232774216	3.82E-34	8.19E-32	cytoplasmic endogenous regulator of oxidative phosphorylation 1 [Source:HGNC Symbol;Acc:HGNC:53928]
ENSG00000151834	GABRA2	-2.186554199	4.121961043	1.49E-10	3.67E-09	gamma-aminobutyric acid type A receptor subunit alpha2 [Source:HGNC Symbol;Acc:HGNC:4076]
ENSG00000145217	SLC26A1	-2.185510976	2.454821495	2.61E-28	3.63E-26	solute carrier family 26 member 1 [Source:HGNC Symbol;Acc:HGNC:10993]
ENSG00000222386	RN7SKP86	-2.183248358	-0.054643372	1.23E-16	6.16E-15	RN7SK pseudogene 86 [Source:HGNC Symbol;Acc:HGNC:45810]
ENSG00000115155	OTOF	-2.181838258	1.383478748	2.54E-10	6.04E-09	otoferlin [Source:HGNC Symbol;Acc:HGNC:8515]
ENSG00000266329	MIR4281	-2.175971309	0.241044862	2.48E-16	1.19E-14	microRNA 4281 [Source:HGNC Symbol;Acc:HGNC:38357]
ENSG00000080166	DCT	-2.174163915	1.5331742	4.87E-15	2.03E-13	dopachrome tautomerase [Source:HGNC Symbol;Acc:HGNC:2709]
ENSG00000181790	ADGRB1	-2.172210528	5.348868123	4.10E-44	1.56E-41	adhesion G protein-coupled receptor B1 [Source:HGNC Symbol;Acc:HGNC:943]
ENSG00000267112	None	-2.171759382	2.11399366	2.87E-13	9.98E-12	novel transcript
ENSG00000124507	PACSIN1	-2.165221365	2.876311663	2.00E-23	1.88E-21	protein kinase C and casein kinase substrate in neurons 1 [Source:HGNC Symbol;Acc:HGNC:8570]
ENSG00000188916	INSYN2A	-2.164169633	3.521879388	4.21E-30	6.61E-28	inhibitory synaptic factor 2A [Source:HGNC Symbol;Acc:HGNC:33859]
ENSG00000111341	MGP	-2.163852788	0.402442078	1.43E-06	1.76E-05	matrix Gla protein [Source:HGNC Symbol;Acc:HGNC:7060]
ENSG00000267387	None	-2.158361114	-0.193825406	5.15E-17	2.69E-15	novel transcript
ENSG00000132975	GPR12	-2.156383123	3.810396606	4.70E-05	0.000408764	G protein-coupled receptor 12 [Source:HGNC Symbol;Acc:HGNC:4466]
ENSG00000169933	FRMPD4	-2.15448681	2.337611642	1.53E-09	3.27E-08	FERM and PDZ domain containing 4 [Source:HGNC Symbol;Acc:HGNC:29007]
ENSG00000084636	COL16A1	-2.15376776	1.629804147	1.91E-16	9.35E-15	collagen type XVI alpha 1 chain [Source:HGNC Symbol;Acc:HGNC:2193]
ENSG00000155265	GOLGA7B	-2.152390506	4.279416518	1.48E-07	2.20E-06	golgin A7 family member B [Source:HGNC Symbol;Acc:HGNC:31668]
ENSG00000204128	C2orf72	-2.148746979	3.952939879	2.65E-32	5.14E-30	chromosome 2 open reading frame 72 [Source:HGNC Symbol;Acc:HGNC:27418]
ENSG00000248540	None	-2.144799149	1.243441568	2.92E-16	1.39E-14	novel transcript
ENSG00000178773	CPNE7	-2.144334015	2.219834639	2.50E-16	1.20E-14	copine 7 [Source:HGNC Symbol;Acc:HGNC:2320]
ENSG00000183230	CTNNA3	-2.143986722	4.567800824	1.23E-36	3.25E-34	catenin alpha 3 [Source:HGNC Symbol;Acc:HGNC:2511]
ENSG00000233483	EFCAB15P	-2.142511181	0.36164843	4.56E-15	1.90E-13	EF-hand calcium binding domain 15
ENSG00000105877	DNAH11	-2.140550862	4.164200616	3.63E-37	9.95E-35	dynein axonemal heavy chain 11 [Source:HGNC Symbol;Acc:HGNC:2942]
ENSG00000101846	STS	-2.140400978	2.863707611	7.98E-13	2.64E-11	steroid sulfatase [Source:HGNC Symbol;Acc:HGNC:11425]
ENSG00000101445	PPP1R16B	-2.138836849	2.495892811	1.60E-14	6.37E-13	protein phosphatase 1 regulatory subunit 16B [Source:HGNC Symbol;Acc:HGNC:15850]
ENSG00000174145	NWD2	-2.137616981	1.487662851	1.51E-15	6.78E-14	NACHT and WD repeat domain containing 2 [Source:HGNC Symbol;Acc:HGNC:29229]
ENSG00000144407	PTH2R	-2.136056827	1.414211192	2.02E-15	8.86E-14	parathyroid hormone 2 receptor [Source:HGNC Symbol;Acc:HGNC:9609]
ENSG00000145863	GABRA6	-2.135922891	0.762797306	4.52E-10	1.05E-08	gamma-aminobutyric acid type A receptor subunit alpha6 [Source:HGNC Symbol;Acc:HGNC:4080]
ENSG00000223817	CDH23-AS1	-2.134765124	0.817406611	4.32E-12	1.29E-10	CDH23 antisense RNA 1 [Source:HGNC Symbol;Acc:HGNC:31433]
ENSG00000008441	NFIX	-2.133669437	2.36205386	1.15E-06	1.44E-05	nuclear factor I X [Source:HGNC Symbol;Acc:HGNC:7788]
ENSG00000150722	PPP1R1C	-2.130941437	1.409997417	3.51E-19	2.20E-17	protein phosphatase 1 regulatory inhibitor subunit 1C [Source:HGNC Symbol;Acc:HGNC:14940]
ENSG00000138798	EGF	-2.129325297	1.624327577	1.27E-07	1.92E-06	epidermal growth factor [Source:HGNC Symbol;Acc:HGNC:3229]
ENSG00000145365	TIFA	-2.124635857	2.346631798	1.09E-12	3.52E-11	TRAF interacting protein with forkhead associated domain [Source:HGNC Symbol;Acc:HGNC:19075]
ENSG00000204071	TCEAL6	-2.122662764	0.224577674	4.34E-11	1.15E-09	transcription elongation factor A like 6 [Source:HGNC Symbol;Acc:HGNC:24553]
ENSG00000041353	RAB27B	-2.120944056	2.447923014	5.87E-14	2.16E-12	RAB27B
ENSG00000263644	None	-2.120921034	5.594370518	2.90E-29	4.28E-27	novel transcript
ENSG00000168743	NPNT	-2.120465413	1.45001399	2.64E-17	1.42E-15	nephronectin [Source:HGNC Symbol;Acc:HGNC:27405]
ENSG00000184564	SLITRK6	-2.11877982	1.336379227	3.58E-19	2.23E-17	SLIT and NTRK like family member 6 [Source:HGNC Symbol;Acc:HGNC:23503]
ENSG00000140015	KCNH5	-2.118134453	0.728008458	8.01E-13	2.64E-11	potassium voltage-gated channel subfamily H member 5 [Source:HGNC Symbol;Acc:HGNC:6254]
ENSG00000177133	PRDM16-DT	-2.117604798	3.058464912	4.49E-22	3.76E-20	PRDM16 divergent transcript [Source:HGNC Symbol;Acc:HGNC:48664]
ENSG00000256083	None	-2.117345781	0.403478397	1.43E-06	1.77E-05	novel transcript
ENSG00000213468	FIRRE	-2.115629282	2.593906593	2.91E-06	3.38E-05	firre intergenic repeating RNA element [Source:HGNC Symbol;Acc:HGNC:49627]
ENSG00000135549	PKIB	-2.111519782	3.166514451	2.96E-15	1.27E-13	cAMP-dependent protein kinase inhibitor beta [Source:HGNC Symbol;Acc:HGNC:9018]
ENSG00000164171	ITGA2	-2.109685442	2.429984081	2.31E-24	2.41E-22	integrin subunit alpha 2 [Source:HGNC Symbol;Acc:HGNC:6137]
ENSG00000145335	SNCA	-2.105192507	4.890103709	2.20E-23	2.07E-21	synuclein alpha [Source:HGNC Symbol;Acc:HGNC:11138]
ENSG00000214595	EML6	-2.104474401	4.180183598	1.28E-27	1.66E-25	EMAP like 6 [Source:HGNC Symbol;Acc:HGNC:35412]
ENSG00000256306	None	-2.104134217	5.199337938	1.47E-08	2.67E-07	novel transcript
ENSG00000102290	PCDH11X	-2.103930166	4.656946475	5.74E-34	1.19E-31	protocadherin 11 X-linked [Source:HGNC Symbol;Acc:HGNC:8656]
ENSG00000137261	KIAA0319	-2.102711516	2.093681963	1.00E-13	3.63E-12	KIAA0319 [Source:HGNC Symbol;Acc:HGNC:21580]
ENSG00000101638	ST8SIA5	-2.102119887	3.36149412	2.69E-21	2.06E-19	ST8 alpha-N-acetyl-neuraminide alpha-2
ENSG00000213694	S1PR3	-2.100296037	1.728964251	6.85E-12	1.98E-10	sphingosine-1-phosphate receptor 3 [Source:HGNC Symbol;Acc:HGNC:3167]
ENSG00000263724	DLGAP1-AS3	-2.099098042	1.323031941	1.07E-13	3.85E-12	DLGAP1 antisense RNA 3 [Source:HGNC Symbol;Acc:HGNC:27317]
ENSG00000259905	PWRN1	-2.098872656	2.315444927	6.28E-12	1.83E-10	Prader-Willi region non-protein coding RNA 1 [Source:HGNC Symbol;Acc:HGNC:33235]
ENSG00000108551	RASD1	-2.097969725	1.680719396	7.58E-08	1.20E-06	ras related dexamethasone induced 1 [Source:HGNC Symbol;Acc:HGNC:15828]
ENSG00000196277	GRM7	-2.097265093	3.963585521	5.05E-34	1.06E-31	glutamate metabotropic receptor 7 [Source:HGNC Symbol;Acc:HGNC:4599]
ENSG00000157152	SYN2	-2.096150774	4.434501976	3.12E-23	2.88E-21	synapsin II [Source:HGNC Symbol;Acc:HGNC:11495]
ENSG00000207647	MIR153-1	-2.09578056	-0.389082557	5.22E-17	2.73E-15	microRNA 153-1 [Source:HGNC Symbol;Acc:HGNC:31539]
ENSG00000116132	PRRX1	-2.095702185	1.635880804	2.53E-08	4.38E-07	paired related homeobox 1 [Source:HGNC Symbol;Acc:HGNC:9142]
ENSG00000237515	SHISA9	-2.089299943	2.849045446	2.30E-17	1.25E-15	shisa family member 9 [Source:HGNC Symbol;Acc:HGNC:37231]
ENSG00000185133	INPP5J	-2.086237747	2.434883343	1.45E-16	7.17E-15	inositol polyphosphate-5-phosphatase J [Source:HGNC Symbol;Acc:HGNC:8956]
ENSG00000256633	PDE2A-AS2	-2.084386983	0.01560742	4.57E-16	2.15E-14	PDE2A antisense RNA 2 [Source:HGNC Symbol;Acc:HGNC:40434]
ENSG00000166250	CLMP	-2.081705774	2.418047286	2.76E-19	1.76E-17	CXADR like membrane protein [Source:HGNC Symbol;Acc:HGNC:24039]
ENSG00000075223	SEMA3C	-2.080444102	3.230194074	9.03E-17	4.59E-15	semaphorin 3C [Source:HGNC Symbol;Acc:HGNC:10725]
ENSG00000264529	DNAJB6P8	-2.07585044	-0.781550744	6.43E-16	2.98E-14	DNAJB6 pseudogene 8 [Source:HGNC Symbol;Acc:HGNC:54761]
ENSG00000107954	NEURL1	-2.075129684	4.229394966	6.72E-25	7.23E-23	neuralized E3 ubiquitin protein ligase 1 [Source:HGNC Symbol;Acc:HGNC:7761]
ENSG00000247775	SNCA-AS1	-2.07352065	2.030367846	1.54E-17	8.60E-16	SNCA antisense RNA 1 [Source:HGNC Symbol;Acc:HGNC:50600]
ENSG00000188582	PAQR9	-2.072674216	1.44646755	1.87E-20	1.33E-18	progestin and adipoQ receptor family member 9 [Source:HGNC Symbol;Acc:HGNC:30131]
ENSG00000164120	HPGD	-2.070625058	1.357705946	7.02E-07	9.25E-06	15-hydroxyprostaglandin dehydrogenase [Source:HGNC Symbol;Acc:HGNC:5154]
ENSG00000172350	ABCG4	-2.066806389	1.282019338	1.40E-18	8.38E-17	ATP binding cassette subfamily G member 4 [Source:HGNC Symbol;Acc:HGNC:13884]
ENSG00000172461	FUT9	-2.066051961	6.178316098	3.48E-53	2.15E-50	fucosyltransferase 9 [Source:HGNC Symbol;Acc:HGNC:4020]
ENSG00000273108	None	-2.057492792	1.173712799	1.11E-12	3.57E-11	novel transcript
ENSG00000167895	TMC8	-2.056973679	0.132286782	2.66E-13	9.26E-12	transmembrane channel like 8 [Source:HGNC Symbol;Acc:HGNC:20474]
ENSG00000120875	DUSP4	-2.056812874	6.962195274	2.23E-10	5.31E-09	dual specificity phosphatase 4 [Source:HGNC Symbol;Acc:HGNC:3070]
ENSG00000068831	RASGRP2	-2.054821164	1.029060532	5.33E-20	3.66E-18	RAS guanyl releasing protein 2 [Source:HGNC Symbol;Acc:HGNC:9879]
ENSG00000260664	None	-2.054248986	2.506197454	1.20E-20	8.64E-19	novel transcript
ENSG00000074416	MGLL	-2.053581017	1.221015209	8.97E-08	1.40E-06	monoglyceride lipase [Source:HGNC Symbol;Acc:HGNC:17038]
ENSG00000267868	None	-2.053479838	0.124120869	2.54E-13	8.88E-12	novel transcript
ENSG00000244128	LINC01322	-2.052088669	2.065427047	3.53E-19	2.21E-17	long intergenic non-protein coding RNA 1322 [Source:HGNC Symbol;Acc:HGNC:50528]
ENSG00000187210	GCNT1	-2.051962732	0.603088848	7.31E-10	1.64E-08	glucosaminyl (N-acetyl) transferase 1 [Source:HGNC Symbol;Acc:HGNC:4203]
ENSG00000092054	MYH7	-2.051538926	2.46262411	1.69E-05	0.000166752	myosin heavy chain 7 [Source:HGNC Symbol;Acc:HGNC:7577]
ENSG00000111452	ADGRD1	-2.051344336	0.692254251	2.58E-20	1.83E-18	adhesion G protein-coupled receptor D1 [Source:HGNC Symbol;Acc:HGNC:19893]
ENSG00000226797	None	-2.050696136	3.326328307	8.82E-22	7.17E-20	novel transcript
ENSG00000170775	GPR37	-2.047357115	1.291497602	2.04E-17	1.12E-15	G protein-coupled receptor 37 [Source:HGNC Symbol;Acc:HGNC:4494]
ENSG00000188803	SHISA6	-2.042162118	3.638444764	2.82E-18	1.65E-16	shisa family member 6 [Source:HGNC Symbol;Acc:HGNC:34491]
ENSG00000225449	RAB6C-AS1	-2.041424276	0.705577881	1.05E-13	3.80E-12	RAB6C antisense RNA 1 [Source:HGNC Symbol;Acc:HGNC:49278]
ENSG00000255693	LINC02389	-2.041260296	1.584397985	1.31E-09	2.81E-08	long intergenic non-protein coding RNA 2389 [Source:HGNC Symbol;Acc:HGNC:53316]
ENSG00000271894	None	-2.038203474	3.316038411	6.46E-20	4.41E-18	novel transcript

ENSG00000196878	LAMB3	-2.035709106	-0.494095794	2.36E-16	1.14E-14	laminin subunit beta 3 [Source:HGNC Symbol;Acc:HGNC:6490]
ENSG00000146674	IGFBP3	-2.034805624	0.891730298	5.29E-17	2.76E-15	insulin like growth factor binding protein 3 [Source:HGNC Symbol;Acc:HGNC:5472]
ENSG00000231252	None	-2.034037818	1.8511199	1.41E-12	4.49E-11	novel transcript
ENSG00000250327	RPSAP70	-2.029147034	-0.373510402	8.82E-17	4.51E-15	ribosomal protein SA pseudogene 70 [Source:HGNC Symbol;Acc:HGNC:51923]
ENSG00000158089	GALNT14	-2.028597561	2.822949423	1.74E-17	9.66E-16	polypeptide N-acetylgalactosaminyltransferase 14 [Source:HGNC Symbol;Acc:HGNC:22946]
ENSG00000248165	None	-2.028576483	0.702196496	5.67E-13	1.90E-11	novel transcript
ENSG00000011083	SLC6A7	-2.024491644	3.318124842	7.40E-27	9.23E-25	solute carrier family 6 member 7 [Source:HGNC Symbol;Acc:HGNC:11054]
ENSG00000121905	HPCA	-2.023632313	2.4786983	1.98E-14	7.73E-13	hippocalcin [Source:HGNC Symbol;Acc:HGNC:5144]
ENSG00000207146	Y_RNA	-2.023258742	0.524639201	1.49E-08	2.71E-07	Y RNA [Source:RFAM;Acc:RF00019]
ENSG00000133401	PDZD2	-2.02270655	3.613092546	3.96E-31	6.97E-29	PDZ domain containing 2 [Source:HGNC Symbol;Acc:HGNC:18486]
ENSG00000177181	RIMKLA	-2.018046337	4.416886769	3.13E-09	6.39E-08	ribosomal modification protein rimK like family member A [Source:HGNC Symbol;Acc:HGNC:28725]
ENSG00000204065	TCEAL5	-2.016130124	4.108931019	6.03E-14	2.21E-12	transcription elongation factor A like 5 [Source:HGNC Symbol;Acc:HGNC:22282]
ENSG00000258702	None	-2.013829947	-0.422752002	1.44E-14	5.73E-13	novel transcript
ENSG00000188549	CCDC9B	-2.013193091	-0.083076037	6.31E-12	1.83E-10	coiled-coil domain containing 9B [Source:HGNC Symbol;Acc:HGNC:33488]
ENSG00000006116	CACNG3	-2.011425445	0.703208478	4.41E-18	2.55E-16	calcium voltage-gated channel auxiliary subunit gamma 3 [Source:HGNC Symbol;Acc:HGNC:1407]
ENSG00000224394	GPC6-AS2	-2.009996956	0.235594978	7.35E-13	2.45E-11	GPC6 antisense RNA 2 [Source:HGNC Symbol;Acc:HGNC:39910]
ENSG00000260230	FRRS1L	-2.00690797	5.004274798	9.89E-20	6.63E-18	ferric chelate reductase 1 like [Source:HGNC Symbol;Acc:HGNC:1362]
ENSG00000155846	PPARGC1B	-2.005674793	2.617350508	6.53E-25	7.07E-23	PPARG coactivator 1 beta [Source:HGNC Symbol;Acc:HGNC:30022]
ENSG00000180613	GSX2	-2.004193954	1.861738093	7.46E-13	2.48E-11	GS homeobox 2 [Source:HGNC Symbol;Acc:HGNC:24959]
ENSG00000251314	None	-2.003562458	3.589127731	4.08E-21	3.08E-19	novel transcript
ENSG00000007372	PAX6	-2.003123201	7.090040876	8.80E-32	1.61E-29	paired box 6 [Source:HGNC Symbol;Acc:HGNC:8620]
ENSG00000267466	None	-2.002205236	0.146967895	6.15E-12	1.79E-10	novel transcript
ENSG00000060709	RIMBP2	-2.001311206	4.777017858	1.05E-05	0.000108425	RIMS binding protein 2 [Source:HGNC Symbol;Acc:HGNC:30339]
ENSG00000196990	FAM163B	-2.000279839	0.030718828	3.65E-17	1.93E-15	family with sequence similarity 163 member B [Source:HGNC Symbol;Acc:HGNC:33277]
ENSG00000055813	CCDC85A	-1.999410782	3.171243228	4.36E-19	2.71E-17	coiled-coil domain containing 85A [Source:HGNC Symbol;Acc:HGNC:29400]
ENSG00000170921	TANC2	-1.997364609	6.144638186	1.30E-31	2.35E-29	tetratricopeptide repeat
ENSG00000236279	CLEC2L	-1.995322241	1.311183252	5.24E-10	1.20E-08	C-type lectin domain family 2 member L [Source:HGNC Symbol;Acc:HGNC:21969]
ENSG00000273079	GRIN2B	-1.993186868	6.374884838	2.29E-08	4.01E-07	glutamate ionotropic receptor NMDA type subunit 2B [Source:HGNC Symbol;Acc:HGNC:4586]
ENSG00000186642	PDE2A	-1.992813549	3.016305699	2.48E-16	1.19E-14	phosphodiesterase 2A [Source:HGNC Symbol;Acc:HGNC:8777]
ENSG00000101825	MXRA5	-1.992403771	1.669537875	8.53E-14	3.11E-12	matrix remodeling associated 5 [Source:HGNC Symbol;Acc:HGNC:7539]
ENSG00000226112	FCF1P4	-1.990780245	0.508281991	1.38E-16	6.87E-15	FCF1 pseudogene 4 [Source:HGNC Symbol;Acc:HGNC:44616]
ENSG00000161267	BDH1	-1.990273393	3.699081226	4.00E-30	6.32E-28	3-hydroxybutyrate dehydrogenase 1 [Source:HGNC Symbol;Acc:HGNC:1027]
ENSG00000113083	LOX	-1.990091106	3.822720448	1.81E-14	7.10E-13	lysyl oxidase [Source:HGNC Symbol;Acc:HGNC:6664]
ENSG00000175874	CREG2	-1.989832421	3.208309069	1.89E-10	4.58E-09	cellular repressor of E1A stimulated genes 2 [Source:HGNC Symbol;Acc:HGNC:14272]
ENSG00000159173	TNNI1	-1.98942784	0.213593584	8.64E-09	1.64E-07	troponin I1
ENSG00000253851	None	-1.989330384	2.5606679	8.94E-09	1.69E-07	novel transcript
ENSG00000180616	SSTR2	-1.985950599	2.331244203	1.74E-12	5.45E-11	somatostatin receptor 2 [Source:HGNC Symbol;Acc:HGNC:11331]
ENSG00000267767	LINC01801	-1.98005301	3.400896072	3.63E-27	4.65E-25	long intergenic non-protein coding RNA 1801 [Source:HGNC Symbol;Acc:HGNC:52592]
ENSG00000230432	None	-1.979640506	3.693254924	4.36E-20	3.03E-18	novel transcript
ENSG00000261754	None	-1.977738107	1.948981947	1.51E-22	1.29E-20	novel transcript
ENSG00000139263	LRIG3	-1.977276096	2.174762705	4.32E-09	8.64E-08	leucine rich repeats and immunoglobulin like domains 3 [Source:HGNC Symbol;Acc:HGNC:30991]
ENSG00000162981	LRATD1	-1.976505389	5.062533156	4.48E-20	3.09E-18	LRAT domain containing 1 [Source:HGNC Symbol;Acc:HGNC:20743]
ENSG00000167414	GNG8	-1.975946904	-0.449512367	1.19E-13	4.26E-12	G protein subunit gamma 8 [Source:HGNC Symbol;Acc:HGNC:19664]
ENSG00000095713	CRTAC1	-1.974662126	4.321850481	4.59E-15	1.91E-13	cartilage acidic protein 1 [Source:HGNC Symbol;Acc:HGNC:14882]
ENSG00000178718	RPP25	-1.973064177	3.501077965	3.33E-05	0.000301005	ribonuclease P and MRP subunit p25 [Source:HGNC Symbol;Acc:HGNC:30361]
ENSG00000259863	SH3RF3-AS1	-1.971737925	0.541779751	3.79E-08	6.35E-07	SH3RF3 antisense RNA 1 [Source:HGNC Symbol;Acc:HGNC:44168]
ENSG00000133816	MICAL2	-1.968856632	1.429119288	1.56E-07	2.31E-06	microtubule associated monooxygenase
ENSG00000104140	RHOV	-1.968058597	0.118961221	8.95E-09	1.69E-07	ras homolog family member V [Source:HGNC Symbol;Acc:HGNC:18313]
ENSG00000126785	RHOJ	-1.967766083	2.130894543	9.40E-06	9.77E-05	ras homolog family member J [Source:HGNC Symbol;Acc:HGNC:688]
ENSG00000180921	FAM83H	-1.966769028	2.38050674	2.65E-20	1.87E-18	family with sequence similarity 83 member H [Source:HGNC Symbol;Acc:HGNC:24797]
ENSG00000135973	GPR45	-1.96321869	-0.358956904	2.14E-11	5.87E-10	G protein-coupled receptor 45 [Source:HGNC Symbol;Acc:HGNC:4503]
ENSG00000249362	LINC02488	-1.961949311	1.593746799	5.11E-07	6.94E-06	long intergenic non-protein coding RNA 2488 [Source:HGNC Symbol;Acc:HGNC:53467]
ENSG00000166145	SPINT1	-1.959702844	-0.776056682	1.93E-14	7.54E-13	serine peptidase inhibitor
ENSG00000114315	HES1	-1.958721901	4.081090013	4.59E-31	8.03E-29	hes family bHLH transcription factor 1 [Source:HGNC Symbol;Acc:HGNC:5192]
ENSG00000238243	OR2W3	-1.958051537	0.500219742	6.04E-12	1.76E-10	olfactory receptor family 2 subfamily W member 3 [Source:HGNC Symbol;Acc:HGNC:15021]
ENSG00000170959	DCDC1	-1.957168122	4.206872861	2.06E-31	3.69E-29	doublecortin domain containing 1 [Source:HGNC Symbol;Acc:HGNC:20625]
ENSG00000158286	RNF207	-1.956860151	1.69219986	9.36E-13	3.04E-11	ring finger protein 207 [Source:HGNC Symbol;Acc:HGNC:32947]
ENSG00000134627	PIWIL4	-1.956659298	1.358705278	3.61E-14	1.37E-12	piwi like RNA-mediated gene silencing 4 [Source:HGNC Symbol;Acc:HGNC:18444]
ENSG00000121335	PRB2	-1.955471214	0.454964368	7.57E-12	2.17E-10	proline rich protein BstNI subfamily 2 [Source:HGNC Symbol;Acc:HGNC:9338]
ENSG00000253645	None	-1.952968021	0.852192659	4.74E-17	2.49E-15	novel transcript
ENSG00000203685	STUM	-1.951808428	3.752725199	6.26E-11	1.61E-09	stum
ENSG00000177272	KCNA3	-1.950672011	2.346634544	2.10E-16	1.02E-14	potassium voltage-gated channel subfamily A member 3 [Source:HGNC Symbol;Acc:HGNC:6221]
ENSG00000263146	LINC01896	-1.95055777	3.104886287	1.70E-13	5.99E-12	long intergenic non-protein coding RNA 1896 [Source:HGNC Symbol;Acc:HGNC:52715]
ENSG00000183098	GPC6	-1.949329675	4.5450034	1.16E-19	7.66E-18	glypican 6 [Source:HGNC Symbol;Acc:HGNC:4454]
ENSG00000147724	FAM135B	-1.946718137	1.791682113	1.57E-10	3.85E-09	family with sequence similarity 135 member B [Source:HGNC Symbol;Acc:HGNC:28029]
ENSG00000151490	PTPRO	-1.94671215	5.941611488	1.15E-19	7.65E-18	protein tyrosine phosphatase receptor type O [Source:HGNC Symbol;Acc:HGNC:9678]
ENSG00000054356	PTPRN	-1.946679901	4.982906793	2.40E-26	2.86E-24	protein tyrosine phosphatase receptor type N [Source:HGNC Symbol;Acc:HGNC:9676]
ENSG00000169071	ROR2	-1.942596322	2.064322519	4.99E-10	1.15E-08	receptor tyrosine kinase like orphan receptor 2 [Source:HGNC Symbol;Acc:HGNC:10257]
ENSG00000135363	LMO2	-1.942218894	2.91204264	1.73E-07	2.53E-06	LIM domain only 2 [Source:HGNC Symbol;Acc:HGNC:6642]
ENSG00000166501	PRKCB	-1.940701544	5.446584746	4.10E-11	1.09E-09	protein kinase C beta [Source:HGNC Symbol;Acc:HGNC:9395]
ENSG00000272180	None	-1.938997565	1.333780138	4.78E-08	7.83E-07	novel transcript
ENSG00000149654	CDH22	-1.938951079	3.150486277	2.96E-18	1.73E-16	cadherin 22 [Source:HGNC Symbol;Acc:HGNC:13251]
ENSG00000169894	MUC3A	-1.938781622	0.667469991	2.68E-08	4.63E-07	mucin 3A
ENSG00000163995	ABLIM2	-1.938170486	3.344584078	3.70E-23	3.39E-21	actin binding LIM protein family member 2 [Source:HGNC Symbol;Acc:HGNC:19195]
ENSG00000179981	TSHZ1	-1.938160712	5.184103034	5.79E-32	1.09E-29	teashirt zinc finger homeobox 1 [Source:HGNC Symbol;Acc:HGNC:10669]
ENSG00000175772	LINC01106	-1.936824006	3.589584826	8.48E-10	1.89E-08	long intergenic non-protein coding RNA 1106 [Source:HGNC Symbol;Acc:HGNC:26769]
ENSG00000196371	FUT4	-1.932397041	0.60857963	1.89E-14	7.43E-13	fucosyltransferase 4 [Source:HGNC Symbol;Acc:HGNC:4015]
ENSG00000154654	NCAM2	-1.931906793	4.505091873	2.00E-37	5.54E-35	neural cell adhesion molecule 2 [Source:HGNC Symbol;Acc:HGNC:7657]
ENSG00000175040	CHST2	-1.931230157	3.925385934	1.72E-28	2.42E-26	carbohydrate sulfotransferase 2 [Source:HGNC Symbol;Acc:HGNC:1970]
ENSG00000235470	NEURL1-AS1	-1.928952336	1.0092368	5.32E-13	1.80E-11	NEURL1 antisense RNA 1 [Source:HGNC Symbol;Acc:HGNC:51220]
ENSG00000164512	ANKRD55	-1.928771935	1.164309506	3.62E-14	1.37E-12	ankyrin repeat domain 55 [Source:HGNC Symbol;Acc:HGNC:25681]
ENSG00000163638	ADAMTS9	-1.922987539	3.876764393	9.69E-14	3.52E-12	ADAM metallopeptidase with thrombospondin type 1 motif 9 [Source:HGNC Symbol;Acc:HGNC:13202]
ENSG00000165810	BTNL9	-1.921932829	1.719943839	3.18E-13	1.10E-11	butyrophilin like 9 [Source:HGNC Symbol;Acc:HGNC:24176]
ENSG00000251095	None	-1.921386986	3.431056505	7.16E-16	3.31E-14	novel transcript
ENSG00000168481	LGI3	-1.919293574	0.319469163	6.97E-06	7.48E-05	leucine rich repeat LGI family member 3 [Source:HGNC Symbol;Acc:HGNC:18711]
ENSG00000188580	NKAIN2	-1.915778973	4.269541569	4.33E-28	5.84E-26	sodium/potassium transporting ATPase interacting 2 [Source:HGNC Symbol;Acc:HGNC:16443]
ENSG00000188158	NHS	-1.908848387	5.34032667	6.53E-20	4.45E-18	NHS actin remodeling regulator [Source:HGNC Symbol;Acc:HGNC:7820]
ENSG00000018236	CNTN1	-1.906740626	6.173600033	1.97E-10	4.76E-09	contactin 1 [Source:HGNC Symbol;Acc:HGNC:2171]
ENSG00000138028	CGREF1	-1.906689543	3.540511749	1.59E-12	5.02E-11	cell growth regulator with EF-hand domain 1 [Source:HGNC Symbol;Acc:HGNC:16962]
ENSG00000234626	None	-1.904565923	1.076199318	1.98E-06	2.38E-05	novel transcript

ENSG00000112319	EYA4	-1.903148161	0.211591048	1.53E-08	2.76E-07	EYA transcriptional coactivator and phosphatase 4 [Source:HGNC Symbol;Acc:HGNC:3522]
ENSG00000174469	CNTNAP2	-1.901244144	7.496112955	6.15E-36	1.53E-33	contactin associated protein 2 [Source:HGNC Symbol;Acc:HGNC:13830]
ENSG00000179630	LACC1	-1.899265798	1.93539987	3.92E-16	1.85E-14	laccase domain containing 1 [Source:HGNC Symbol;Acc:HGNC:26789]
ENSG00000241679	None	-1.896494956	-0.170181701	2.51E-15	1.09E-13	novel transcript
ENSG00000183166	CALN1	-1.895335334	3.42227149	1.18E-13	4.23E-12	calneuron 1 [Source:HGNC Symbol;Acc:HGNC:13248]
ENSG00000258903	None	-1.895069795	4.160909112	6.87E-13	2.29E-11	novel transcript
ENSG00000006047	YBX2	-1.89490078	1.622096647	1.42E-08	2.59E-07	Y-box binding protein 2 [Source:HGNC Symbol;Acc:HGNC:17948]
ENSG00000181072	CHRM2	-1.89457797	2.145070004	6.54E-09	1.27E-07	cholinergic receptor muscarinic 2 [Source:HGNC Symbol;Acc:HGNC:1951]
ENSG00000007264	MATK	-1.892317974	1.107040569	2.06E-08	3.64E-07	megakaryocyte-associated tyrosine kinase [Source:HGNC Symbol;Acc:HGNC:6906]
ENSG00000203877	RIPPLY2	-1.891204841	0.676786606	3.87E-08	6.46E-07	ripply transcriptional repressor 2 [Source:HGNC Symbol;Acc:HGNC:21390]
ENSG00000169427	KCNK9	-1.891084992	3.465923231	2.04E-10	4.91E-09	potassium two pore domain channel subfamily K member 9 [Source:HGNC Symbol;Acc:HGNC:6283]
ENSG00000132170	PPARG	-1.891062979	-0.153589157	1.63E-12	5.13E-11	peroxisome proliferator activated receptor gamma [Source:HGNC Symbol;Acc:HGNC:9236]
ENSG00000149633	KIAA1755	-1.89058551	-0.140847643	1.66E-11	4.62E-10	KIAA1755 [Source:HGNC Symbol;Acc:HGNC:29372]
ENSG00000165028	NIPSNAP3B	-1.888440311	1.017150081	8.73E-16	4.00E-14	nipsnap homolog 3B [Source:HGNC Symbol;Acc:HGNC:23641]
ENSG00000184922	FMNL1	-1.886737759	2.61262831	4.75E-10	1.10E-08	formin like 1 [Source:HGNC Symbol;Acc:HGNC:1212]
ENSG00000172575	RASGRP1	-1.886247328	3.629548199	2.03E-28	2.83E-26	RAS guanyl releasing protein 1 [Source:HGNC Symbol;Acc:HGNC:9878]
ENSG00000204588	LINC01123	-1.882921003	3.333046311	2.37E-09	4.94E-08	long intergenic non-protein coding RNA 1123 [Source:HGNC Symbol;Acc:HGNC:49269]
ENSG00000138653	NDST4	-1.882342495	3.357167012	3.58E-22	3.03E-20	N-deacetylase and N-sulfotransferase 4 [Source:HGNC Symbol;Acc:HGNC:20779]
ENSG00000162951	LRRTM1	-1.880291474	2.754264682	1.40E-18	8.38E-17	leucine rich repeat transmembrane neuronal 1 [Source:HGNC Symbol;Acc:HGNC:19408]
ENSG00000226521	None	-1.87893718	-0.902413287	2.29E-12	7.03E-11	ribosomal protein S6 kinase
ENSG00000233635	None	-1.878013277	3.230797423	4.84E-21	3.60E-19	novel transcript
ENSG00000158714	SLAMF8	-1.876828044	0.102670608	3.53E-15	1.50E-13	SLAM family member 8 [Source:HGNC Symbol;Acc:HGNC:21391]
ENSG00000184304	PRKD1	-1.87606558	4.112594649	5.59E-25	6.09E-23	protein kinase D1 [Source:HGNC Symbol;Acc:HGNC:9407]
ENSG00000133110	POSTN	-1.875811808	2.015331007	1.10E-05	0.000112605	periostin [Source:HGNC Symbol;Acc:HGNC:16953]
ENSG00000182732	RGS6	-1.872191784	2.558873479	1.26E-14	5.06E-13	regulator of G protein signaling 6 [Source:HGNC Symbol;Acc:HGNC:10002]
ENSG00000233672	RNASEH2B-AS1	-1.869203132	1.101717743	4.33E-09	8.65E-08	RNASEH2B antisense RNA 1 [Source:HGNC Symbol;Acc:HGNC:39967]
ENSG00000184227	ACOT1	-1.86890016	0.436527592	2.28E-12	6.99E-11	acyl-CoA thioesterase 1 [Source:HGNC Symbol;Acc:HGNC:33128]
ENSG00000163536	SERPINI1	-1.866159659	3.236974093	5.47E-23	4.92E-21	serpin family I member 1 [Source:HGNC Symbol;Acc:HGNC:8943]
ENSG00000127415	IDUA	-1.865726185	3.449316382	5.32E-21	3.91E-19	alpha-L-iduronidase [Source:HGNC Symbol;Acc:HGNC:5391]
ENSG00000197291	RAMP2-AS1	-1.865650795	1.379168575	1.63E-12	5.11E-11	RAMP2 antisense RNA 1 [Source:HGNC Symbol;Acc:HGNC:44358]
ENSG00000214922	HLA-F-AS1	-1.865472386	-0.171418542	5.58E-15	2.30E-13	HLA-F antisense RNA 1 [Source:HGNC Symbol;Acc:HGNC:26645]
ENSG00000197106	SLC6A17	-1.865217521	4.340120368	2.17E-06	2.59E-05	solute carrier family 6 member 17 [Source:HGNC Symbol;Acc:HGNC:31399]
ENSG00000075213	SEMA3A	-1.863160293	4.890742493	5.22E-09	1.03E-07	semaphorin 3A [Source:HGNC Symbol;Acc:HGNC:10723]
ENSG00000011347	SYT7	-1.862748937	5.731204698	5.76E-27	7.28E-25	synaptotagmin 7 [Source:HGNC Symbol;Acc:HGNC:11514]
ENSG00000132932	ATP8A2	-1.861246294	5.505397903	5.17E-11	1.35E-09	ATPase phospholipid transporting 8A2 [Source:HGNC Symbol;Acc:HGNC:13533]
ENSG00000183690	EFHC2	-1.86121346	0.0029979	1.97E-07	2.85E-06	EF-hand domain containing 2 [Source:HGNC Symbol;Acc:HGNC:26233]
ENSG00000272405	None	-1.860905159	0.166813137	7.25E-07	9.53E-06	novel transcript
ENSG00000235280	MCF2L-AS1	-1.860432918	1.013270115	1.49E-12	4.71E-11	MCF2L antisense RNA 1 [Source:HGNC Symbol;Acc:HGNC:39825]
ENSG00000198865	CCDC152	-1.86035219	2.045817806	1.02E-08	1.92E-07	coiled-coil domain containing 152 [Source:HGNC Symbol;Acc:HGNC:34438]
ENSG00000156564	LRFN2	-1.856239288	2.281232371	1.56E-21	1.22E-19	leucine rich repeat and fibronectin type III domain containing 2 [Source:HGNC Symbol;Acc:HGNC:21226]
ENSG00000021300	PLEKHB1	-1.856116638	0.852660912	2.95E-15	1.27E-13	pleckstrin homology domain containing B1 [Source:HGNC Symbol;Acc:HGNC:19079]
ENSG00000135333	EPHA7	-1.854788539	5.564002183	6.52E-09	1.26E-07	EPH receptor A7 [Source:HGNC Symbol;Acc:HGNC:3390]
ENSG00000197991	None	-1.854258357	1.050632607	4.27E-05	0.00037576	novel protein
ENSG00000198626	RYR2	-1.852082653	2.578377899	3.31E-13	1.14E-11	ryanodine receptor 2 [Source:HGNC Symbol;Acc:HGNC:10484]
ENSG00000188153	COL4A5	-1.850380525	6.808605727	8.35E-10	1.86E-08	collagen type IV alpha 5 chain [Source:HGNC Symbol;Acc:HGNC:2207]
ENSG00000186310	NAP1L3	-1.846181514	6.472764663	1.64E-15	7.28E-14	nucleosome assembly protein 1 like 3 [Source:HGNC Symbol;Acc:HGNC:7639]
ENSG00000138166	DUSP5	-1.843571197	1.982416049	1.29E-10	3.21E-09	dual specificity phosphatase 5 [Source:HGNC Symbol;Acc:HGNC:3071]
ENSG00000269699	ZIM2	-1.841027469	0.070050922	3.11E-15	1.33E-13	zinc finger imprinted 2 [Source:HGNC Symbol;Acc:HGNC:12875]
ENSG00000238272	None	-1.840132582	3.105959057	3.06E-09	6.27E-08	novel transcript
ENSG00000258548	LINC00645	-1.839340053	1.648762883	1.30E-12	4.13E-11	long intergenic non-protein coding RNA 645 [Source:HGNC Symbol;Acc:HGNC:44299]
ENSG00000083454	P2RX5	-1.838941952	1.157248075	5.53E-16	2.57E-14	purinergic receptor P2X 5 [Source:HGNC Symbol;Acc:HGNC:8536]
ENSG00000246627	CACNA1C-AS1	-1.837462659	3.325411867	2.57E-11	7.02E-10	CACNA1C antisense RNA 1 [Source:HGNC Symbol;Acc:HGNC:40119]
ENSG00000269793	ZIM2-AS1	-1.837249093	1.556234026	9.15E-17	4.65E-15	ZIM2 antisense RNA 1 [Source:HGNC Symbol;Acc:HGNC:51304]
ENSG00000173727	FAUP4	-1.835797155	1.976477215	2.58E-17	1.39E-15	FAU pseudogene 4 [Source:HGNC Symbol;Acc:HGNC:56253]
ENSG00000127084	FGD3	-1.835398722	2.477068724	1.68E-05	0.000165582	FYVE
ENSG00000130643	CALY	-1.833630271	3.595733373	2.16E-15	9.44E-14	calcyon neuron specific vesicular protein [Source:HGNC Symbol;Acc:HGNC:17938]
ENSG00000133019	CHRM3	-1.833059579	2.988585076	2.07E-10	4.98E-09	cholinergic receptor muscarinic 3 [Source:HGNC Symbol;Acc:HGNC:1952]
ENSG00000125637	PSD4	-1.830509811	0.064944943	9.05E-13	2.95E-11	pleckstrin and Sec7 domain containing 4 [Source:HGNC Symbol;Acc:HGNC:19096]
ENSG00000162728	KCNJ9	-1.8285958	3.903544064	8.54E-13	2.80E-11	potassium inwardly rectifying channel subfamily J member 9 [Source:HGNC Symbol;Acc:HGNC:6270]
ENSG00000272341	None	-1.825195736	1.945951244	1.37E-19	8.94E-18	novel transcript
ENSG00000101282	RSPO4	-1.82495583	1.169403383	4.40E-11	1.16E-09	R-spondin 4 [Source:HGNC Symbol;Acc:HGNC:16175]
ENSG00000215105	TTC3P1	-1.824607336	4.175943552	3.25E-05	0.000294961	tetratricopeptide repeat domain 3 pseudogene 1 [Source:HGNC Symbol;Acc:HGNC:23318]
ENSG00000168546	GFRA2	-1.82013845	3.169586925	4.20E-17	2.21E-15	GDNF family receptor alpha 2 [Source:HGNC Symbol;Acc:HGNC:4244]
ENSG00000259326	None	-1.819980537	-0.319852995	1.19E-14	4.77E-13	novel transcript
ENSG00000061337	LZTS1	-1.817249037	5.29695378	2.78E-21	2.13E-19	leucine zipper tumor suppressor 1 [Source:HGNC Symbol;Acc:HGNC:13861]
ENSG00000081320	STK17B	-1.815535253	3.826394294	3.68E-15	1.56E-13	serine/threonine kinase 17b [Source:HGNC Symbol;Acc:HGNC:11396]
ENSG00000188004	SNHG28	-1.814936187	0.941693682	1.58E-16	7.78E-15	small nucleolar RNA host gene 28 [Source:HGNC Symbol;Acc:HGNC:27647]
ENSG00000204789	ZNF204P	-1.813401195	2.052620109	2.92E-15	1.26E-13	zinc finger protein 204
ENSG00000231173	None	-1.811323895	1.330470658	5.26E-15	2.18E-13	novel transcript
ENSG00000184697	CLDN6	-1.809475151	-0.229853812	6.24E-08	9.98E-07	claudin 6 [Source:HGNC Symbol;Acc:HGNC:2048]
ENSG00000136546	SCN7A	-1.80916714	0.478173005	4.93E-12	1.45E-10	sodium voltage-gated channel alpha subunit 7 [Source:HGNC Symbol;Acc:HGNC:10594]
ENSG00000171992	SYNPO	-1.80806551	0.287333276	5.72E-10	1.31E-08	synaptopodin [Source:HGNC Symbol;Acc:HGNC:30672]
ENSG00000144583	MARCHF4	-1.807401162	5.065201118	0.000107973	0.000851851	membrane associated ring-CH-type finger 4 [Source:HGNC Symbol;Acc:HGNC:29269]
ENSG00000102452	NALCN	-1.805242584	3.946093932	7.68E-12	2.20E-10	sodium leak channel
ENSG00000099953	MMP11	-1.803531795	0.753287053	7.82E-10	1.75E-08	matrix metallopeptidase 11 [Source:HGNC Symbol;Acc:HGNC:7157]
ENSG00000143674	MAP3K21	-1.802119454	2.16387764	3.23E-13	1.12E-11	mitogen-activated protein kinase kinase kinase 21 [Source:HGNC Symbol;Acc:HGNC:29798]
ENSG00000236341	None	-1.799192734	3.0844083	7.38E-16	3.40E-14	novel transcript
ENSG00000171867	PRNP	-1.797987708	5.028308361	7.63E-30	1.18E-27	prion protein [Source:HGNC Symbol;Acc:HGNC:9449]
ENSG00000135828	RNASEL	-1.797026045	1.903493026	1.05E-11	2.99E-10	ribonuclease L [Source:HGNC Symbol;Acc:HGNC:10050]
ENSG00000145808	ADAMTS19	-1.796699534	1.48842751	3.34E-06	3.82E-05	ADAM metallopeptidase with thrombospondin type 1 motif 19 [Source:HGNC Symbol;Acc:HGNC:17111]
ENSG00000227091	SLC6A17-AS1	-1.796285751	1.118629945	1.81E-05	0.00017588	SLC6A17 antisense RNA 1 [Source:HGNC Symbol;Acc:HGNC:41279]
ENSG00000156968	MPV17L	-1.794663081	0.797329483	1.91E-14	7.46E-13	MPV17 mitochondrial inner membrane protein like [Source:HGNC Symbol;Acc:HGNC:26827]
ENSG00000226725	PABPC1L2B-AS1	-1.793911024	0.389020466	1.03E-13	3.73E-12	PABPC1L2B antisense RNA 1 (head to head) [Source:HGNC Symbol;Acc:HGNC:50345]
ENSG00000231092	None	-1.792900063	0.643140966	0.000295594	0.002067449	novel transcript
ENSG00000166897	ELFN2	-1.791317585	5.62003308	4.68E-19	2.89E-17	extracellular leucine rich repeat and fibronectin type III domain containing 2 [Source:HGNC Symbol;Acc:HGNC:29396]
ENSG00000173511	VEGFB	-1.790899111	3.621582667	3.09E-20	2.16E-18	vascular endothelial growth factor B [Source:HGNC Symbol;Acc:HGNC:12681]
ENSG00000240710	None	-1.788897368	0.398394173	1.22E-09	2.64E-08	novel transcript
ENSG00000115461	IGFBP5	-1.787790038	5.541565615	1.73E-07	2.53E-06	insulin like growth factor binding protein 5 [Source:HGNC Symbol;Acc:HGNC:5474]
ENSG00000207957	MIR105-1	-1.786338306	0.264015424	1.45E-10	3.57E-09	microRNA 105-1 [Source:HGNC Symbol;Acc:HGNC:31492]
ENSG00000211583	MIR767	-1.786338241	0.264015424	1.45E-10	3.57E-09	microRNA 767 [Source:HGNC Symbol;Acc:HGNC:33136]

ENSG00000207818	MIR105-2	-1.786338234	0.264015424	1.45E-10	3.57E-09	microRNA 105-2 [Source:HGNC Symbol;Acc:HGNC:31493]
ENSG00000177103	DSCAML1	-1.785569299	4.825869153	2.60E-36	6.77E-34	DS cell adhesion molecule like 1 [Source:HGNC Symbol;Acc:HGNC:14656]
ENSG00000231963	None	-1.784719172	0.382206003	2.75E-13	9.56E-12	novel transcript
ENSG00000164076	CAMKV	-1.784153825	5.587469315	3.15E-19	1.99E-17	CaM kinase like vesicle associated [Source:HGNC Symbol;Acc:HGNC:28788]
ENSG00000164929	BAALC	-1.784049263	4.220297416	8.69E-10	1.93E-08	BAALC binder of MAP3K1 and KLF4 [Source:HGNC Symbol;Acc:HGNC:14333]
ENSG00000247081	BAALC-AS1	-1.783852801	4.113131456	2.18E-09	4.55E-08	BAALC antisense RNA 1 [Source:HGNC Symbol;Acc:HGNC:50461]
ENSG00000176956	LY6H	-1.783217413	4.346170802	4.54E-08	7.48E-07	lymphocyte antigen 6 family member H [Source:HGNC Symbol;Acc:HGNC:6728]
ENSG00000258185	None	-1.782272267	0.173160563	1.92E-11	5.31E-10	novel transcript
ENSG00000169499	PLEKHA2	-1.78217808	2.396853103	9.46E-16	4.30E-14	pleckstrin homology domain containing A2 [Source:HGNC Symbol;Acc:HGNC:14336]
ENSG00000226065	ZBTB45P2	-1.781297798	1.149353492	3.61E-07	5.02E-06	zinc finger and BTB domain containing 45 pseudogene 2 [Source:HGNC Symbol;Acc:HGNC:49228]
ENSG00000163013	FBXO41	-1.775006611	5.042488278	6.04E-09	1.18E-07	F-box protein 41 [Source:HGNC Symbol;Acc:HGNC:29409]
ENSG00000142686	C1orf216	-1.773649365	3.748634439	3.39E-23	3.11E-21	chromosome 1 open reading frame 216 [Source:HGNC Symbol;Acc:HGNC:26800]
ENSG00000130812	ANGPTL6	-1.773256429	0.967426803	9.30E-16	4.24E-14	angiopoietin like 6 [Source:HGNC Symbol;Acc:HGNC:23140]
ENSG00000262884	None	-1.770869624	0.529969512	1.77E-09	3.75E-08	novel transcript
ENSG00000160460	SPTBN4	-1.770475887	4.724329411	1.78E-17	9.83E-16	spectrin beta
ENSG00000069018	TRPC7	-1.770060022	-0.40095493	8.58E-09	1.63E-07	transient receptor potential cation channel subfamily C member 7 [Source:HGNC Symbol;Acc:HGNC:20754]
ENSG00000149948	HMGA2	-1.76673993	5.42162382	1.52E-10	3.73E-09	high mobility group AT-hook 2 [Source:HGNC Symbol;Acc:HGNC:5009]
ENSG00000264706	RN7SL217P	-1.765247678	0.403358228	1.76E-11	4.89E-10	RNA
ENSG00000272694	None	-1.764870568	2.634591553	4.55E-12	1.34E-10	novel transcript
ENSG00000137727	ARHGAP20	-1.76268735	2.515721705	1.22E-09	2.63E-08	Rho GTPase activating protein 20 [Source:HGNC Symbol;Acc:HGNC:18357]
ENSG00000179841	AKAP5	-1.761174613	2.41066118	1.41E-13	5.02E-12	A-kinase anchoring protein 5 [Source:HGNC Symbol;Acc:HGNC:375]
ENSG00000229939	None	-1.75981206	1.123050446	7.05E-14	2.58E-12	novel pseudogene (PRO1843)
ENSG00000171772	SYCE1	-1.759223382	0.088295535	1.88E-15	8.26E-14	synaptonemal complex central element protein 1 [Source:HGNC Symbol;Acc:HGNC:28852]
ENSG00000180287	PLD5	-1.758085651	2.045278195	1.24E-14	4.99E-13	phospholipase D family member 5 [Source:HGNC Symbol;Acc:HGNC:26879]
ENSG00000131477	RAMP2	-1.751017938	1.185597369	1.08E-13	3.88E-12	receptor activity modifying protein 2 [Source:HGNC Symbol;Acc:HGNC:9844]
ENSG00000225108	ZBTB45P1	-1.748686098	1.1718245	9.30E-07	1.19E-05	zinc finger and BTB domain containing 45 pseudogene 1 [Source:HGNC Symbol;Acc:HGNC:49227]
ENSG00000271781	None	-1.743700721	-0.176421002	2.73E-06	3.19E-05	novel transcript
ENSG00000226476	LINC01748	-1.743301755	2.358235926	3.79E-09	7.64E-08	long intergenic non-protein coding RNA 1748 [Source:HGNC Symbol;Acc:HGNC:52535]
ENSG00000243902	ELFN2	-1.742997001	5.720684485	1.57E-20	1.12E-18	extracellular leucine rich repeat and fibronectin type III domain containing 2 [Source:NCBI gene (formerly Entrezgene);Acc:114794]
ENSG00000267580	None	-1.742989546	0.021215936	2.45E-11	6.70E-10	novel transcript
ENSG00000177511	ST8SIA3	-1.742739071	5.14076384	3.07E-26	3.61E-24	ST8 alpha-N-acetyl-neuraminide alpha-2
ENSG00000137766	UNC13C	-1.737416254	2.265228827	5.28E-05	0.000452734	unc-13 homolog C [Source:HGNC Symbol;Acc:HGNC:23149]
ENSG00000241525	None	-1.736311289	1.903459126	3.07E-15	1.31E-13	novel transcript
ENSG00000185760	KCNQ5	-1.735190514	0.836088889	1.31E-09	2.82E-08	potassium voltage-gated channel subfamily Q member 5 [Source:HGNC Symbol;Acc:HGNC:6299]
ENSG00000145526	CDH18	-1.734704065	2.839824753	7.74E-08	1.22E-06	cadherin 18 [Source:HGNC Symbol;Acc:HGNC:1757]
ENSG00000197461	PDGFA	-1.734321167	1.408575424	1.43E-11	4.00E-10	platelet derived growth factor subunit A [Source:HGNC Symbol;Acc:HGNC:8799]
ENSG00000237919	LRRC7-AS1	-1.733332917	2.228256421	2.73E-11	7.43E-10	LRRC7 antisense RNA 1 [Source:HGNC Symbol;Acc:HGNC:40843]
ENSG00000160284	SPATC1L	-1.732556987	2.664764622	1.97E-19	1.27E-17	spermatogenesis and centriole associated 1 like [Source:HGNC Symbol;Acc:HGNC:1298]
ENSG00000106633	GCK	-1.730686772	0.742393863	4.70E-12	1.38E-10	glucokinase [Source:HGNC Symbol;Acc:HGNC:4195]
ENSG00000270002	None	-1.730686732	-0.428270825	2.39E-11	6.55E-10	novel transcript
ENSG00000158716	DUSP23	-1.729856803	0.460682181	6.22E-15	2.55E-13	dual specificity phosphatase 23 [Source:HGNC Symbol;Acc:HGNC:21480]
ENSG00000171243	SOSTDC1	-1.728863465	-0.348385018	5.13E-10	1.18E-08	sclerostin domain containing 1 [Source:HGNC Symbol;Acc:HGNC:21748]
ENSG00000104368	PLAT	-1.726706901	2.636707061	3.31E-17	1.76E-15	plasminogen activator
ENSG00000253915	MAPRE1P1	-1.719945638	0.828587699	2.15E-10	5.13E-09	MAPRE1 pseudogene 1 [Source:HGNC Symbol;Acc:HGNC:29624]
ENSG00000164604	GPR85	-1.719613238	3.761773879	3.80E-18	2.21E-16	G protein-coupled receptor 85 [Source:HGNC Symbol;Acc:HGNC:4536]
ENSG00000056998	GYG2	-1.718752111	3.78709968	4.61E-10	1.07E-08	glycogenin 2 [Source:HGNC Symbol;Acc:HGNC:4700]
ENSG00000261292	None	-1.716718505	-0.03445598	1.54E-12	4.87E-11	novel transcript
ENSG00000089169	RPH3A	-1.715447839	2.040628406	1.01E-07	1.55E-06	rabphilin 3A [Source:HGNC Symbol;Acc:HGNC:17056]
ENSG00000212373	RNA5SP171	-1.713257675	-0.610404775	9.33E-10	2.05E-08	RNA
ENSG00000267506	None	-1.712711932	1.180444494	7.76E-08	1.22E-06	novel transcript
ENSG00000135063	ENTREP1	-1.707596295	0.499681428	1.33E-10	3.30E-09	endosomal transmembrane epsin interactor 1 [Source:HGNC Symbol;Acc:HGNC:24820]
ENSG00000260412	None	-1.706909642	1.711792419	1.73E-15	7.67E-14	novel transcript
ENSG00000251003	ZFPM2-AS1	-1.706055399	2.78526214	4.56E-13	1.55E-11	ZFPM2 antisense RNA 1 [Source:HGNC Symbol;Acc:HGNC:50698]
ENSG00000197410	DCHS2	-1.705189302	3.260013701	6.32E-12	1.84E-10	dachsous cadherin-related 2 [Source:HGNC Symbol;Acc:HGNC:23111]
ENSG00000070808	CAMK2A	-1.700938083	4.716187639	8.29E-30	1.28E-27	calcium/calmodulin dependent protein kinase II alpha [Source:HGNC Symbol;Acc:HGNC:1460]
ENSG00000223947	None	-1.699038795	0.371534534	4.91E-13	1.67E-11	novel transcript
ENSG00000239945	None	-1.699004243	0.540262222	8.75E-11	2.21E-09	novel transcript
ENSG00000162545	CAMK2N1	-1.696692101	5.944270882	1.82E-35	4.32E-33	calcium/calmodulin dependent protein kinase II inhibitor 1 [Source:HGNC Symbol;Acc:HGNC:24190]
ENSG00000125266	EFNB2	-1.696637879	7.429914742	4.43E-36	1.12E-33	ephrin B2 [Source:HGNC Symbol;Acc:HGNC:3227]
ENSG00000171388	APLN	-1.696063817	1.467691522	1.78E-05	0.00017413	apelin [Source:HGNC Symbol;Acc:HGNC:16665]
ENSG00000215861	None	-1.695772333	2.319404894	4.60E-08	7.56E-07	phosphodiesterase 4D interacting protein (myomegalin) (PDE4DIP) pseudogene
ENSG00000152953	STK32B	-1.694801931	2.812800782	4.88E-08	7.97E-07	serine/threonine kinase 32B [Source:HGNC Symbol;Acc:HGNC:14217]
ENSG00000222014	RAB6C	-1.691373602	0.903858949	4.02E-08	6.69E-07	RAB6C
ENSG00000008118	CAMK1G	-1.6882578	2.172639556	3.31E-12	1.00E-10	calcium/calmodulin dependent protein kinase IG [Source:HGNC Symbol;Acc:HGNC:14585]
ENSG00000225811	None	-1.687605983	0.432934964	2.43E-06	2.87E-05	novel transcript
ENSG00000119946	CNNM1	-1.686915389	2.668785489	5.08E-16	2.37E-14	cyclin and CBS domain divalent metal cation transport mediator 1 [Source:HGNC Symbol;Acc:HGNC:102]
ENSG00000169213	RAB3B	-1.686721227	5.309961529	1.28E-35	3.10E-33	RAB3B
ENSG00000264513	None	-1.686120714	0.152584007	9.96E-14	3.61E-12	novel transcript
ENSG00000131187	F12	-1.685384198	0.690431674	2.00E-12	6.16E-11	coagulation factor XII [Source:HGNC Symbol;Acc:HGNC:3530]
ENSG00000158234	FAIM	-1.683698762	0.897257588	4.28E-13	1.46E-11	Fas apoptotic inhibitory molecule [Source:HGNC Symbol;Acc:HGNC:18703]
ENSG00000120708	TGFBI	-1.683563883	1.306381734	7.81E-08	1.23E-06	transforming growth factor beta induced [Source:HGNC Symbol;Acc:HGNC:11771]
ENSG00000229951	FOSL2-AS1	-1.683126961	0.937467476	3.19E-09	6.50E-08	FOSL2 antisense RNA 1 [Source:HGNC Symbol;Acc:HGNC:55784]
ENSG00000236939	BAALC-AS2	-1.681718705	1.747704079	2.10E-09	4.40E-08	BAALC antisense RNA 2 [Source:HGNC Symbol;Acc:HGNC:28595]
ENSG00000267333	ASNSP6	-1.680581912	-0.344238315	2.00E-11	5.54E-10	ASNS pseudogene 6 [Source:HGNC Symbol;Acc:HGNC:49876]
ENSG00000262691	None	-1.679914823	-0.328685548	6.08E-12	1.77E-10	novel transcript
ENSG00000162552	WNT4	-1.679439998	2.610945219	1.48E-16	7.32E-15	Wnt family member 4 [Source:HGNC Symbol;Acc:HGNC:12783]
ENSG00000239911	PRKAG2-AS1	-1.679240507	-0.109591741	1.91E-12	5.91E-11	PRKAG2 antisense RNA 1 [Source:HGNC Symbol;Acc:HGNC:40468]
ENSG00000199520	RNU6-386P	-1.678922707	-0.146085055	1.75E-14	6.90E-13	RNA
ENSG00000124313	IQSEC2	-1.677889512	3.921327429	5.44E-17	2.82E-15	IQ motif and Sec7 domain ArfGEF 2 [Source:HGNC Symbol;Acc:HGNC:29059]
ENSG00000205856	C22orf42	-1.676414462	1.292129009	5.83E-13	1.96E-11	chromosome 22 open reading frame 42 [Source:HGNC Symbol;Acc:HGNC:27160]
ENSG00000007129	CEACAM21	-1.675932001	1.780630735	2.28E-07	3.28E-06	CEA cell adhesion molecule 21 [Source:HGNC Symbol;Acc:HGNC:28834]
ENSG00000182450	KCNK4	-1.675569207	-0.38340648	9.24E-13	3.00E-11	potassium two pore domain channel subfamily K member 4 [Source:HGNC Symbol;Acc:HGNC:6279]
ENSG00000162595	DIRAS3	-1.675310751	1.86290634	3.22E-14	1.22E-12	DIRAS family GTPase 3 [Source:HGNC Symbol;Acc:HGNC:687]
ENSG00000236064	None	-1.67481	3.02915026	1.13E-07	1.72E-06	novel transcript
ENSG00000238279	None	-1.673511139	-0.307176158	3.13E-09	6.39E-08	novel transcript
ENSG00000197444	OGDHL	-1.673055142	3.767437741	1.18E-22	1.03E-20	oxoglutarate dehydrogenase L [Source:HGNC Symbol;Acc:HGNC:25590]
ENSG00000157514	TSC22D3	-1.672424142	5.060033409	3.17E-21	2.42E-19	TSC22 domain family member 3 [Source:HGNC Symbol;Acc:HGNC:3051]
ENSG00000223751	None	-1.67052375	0.383582292	4.43E-11	1.17E-09	novel transcript
ENSG00000154975	CA10	-1.669705117	4.008466529	7.31E-16	3.38E-14	carbonic anhydrase 10 [Source:HGNC Symbol;Acc:HGNC:1369]
ENSG00000159307	SCUBE1	-1.669163334	5.473444971	3.46E-12	1.04E-10	signal peptide

ENSG00000141524	TMC6	-1.668604655	2.050599492	1.23E-13	4.40E-12	transmembrane channel like 6 [Source:HGNC Symbol;Acc:HGNC:18021]
ENSG00000108018	SORCS1	-1.668135938	5.325591432	5.95E-10	1.36E-08	sortilin related VPS10 domain containing receptor 1 [Source:HGNC Symbol;Acc:HGNC:16697]
ENSG00000157782	CABP1	-1.668102336	1.379261147	2.12E-11	5.84E-10	calcium binding protein 1 [Source:HGNC Symbol;Acc:HGNC:1384]
ENSG00000256271	CACNA1C-AS2	-1.667228192	0.260089217	3.25E-08	5.50E-07	CACNA1C antisense RNA 2 [Source:HGNC Symbol;Acc:HGNC:40118]
ENSG00000144331	ZNF385B	-1.666937352	0.583865614	4.50E-08	7.42E-07	zinc finger protein 385B [Source:HGNC Symbol;Acc:HGNC:26332]
ENSG00000227067	DPPA3P1	-1.666770294	-0.050650384	1.09E-10	2.72E-09	developmental pluripotency associated 3 pseudogene 1 [Source:HGNC Symbol;Acc:HGNC:32345]
ENSG00000134256	CD101	-1.665877583	1.260708841	1.62E-14	6.45E-13	CD101 molecule [Source:HGNC Symbol;Acc:HGNC:5949]
ENSG00000273492	APP-DT	-1.665398623	0.005366567	5.88E-12	1.72E-10	APP divergent transcript [Source:HGNC Symbol;Acc:HGNC:55075]
ENSG00000101460	MAP1LC3A	-1.664455535	4.412476733	5.85E-19	3.60E-17	microtubule associated protein 1 light chain 3 alpha [Source:HGNC Symbol;Acc:HGNC:6838]
ENSG00000189212	DPY19L2P1	-1.662673796	3.231012781	1.54E-18	9.17E-17	DPY19L2 pseudogene 1 [Source:HGNC Symbol;Acc:HGNC:22305]
ENSG00000181195	PENK	-1.656744148	0.130872021	2.92E-11	7.93E-10	proenkephalin [Source:HGNC Symbol;Acc:HGNC:8831]
ENSG00000169306	IL1RAPL1	-1.656577818	3.448963917	1.84E-17	1.01E-15	interleukin 1 receptor accessory protein like 1 [Source:HGNC Symbol;Acc:HGNC:5996]
ENSG00000251680	None	-1.655616164	1.70576734	2.19E-08	3.85E-07	novel transcript
ENSG00000070729	CNGB1	-1.654725445	2.159972642	2.05E-06	2.45E-05	cyclic nucleotide gated channel subunit beta 1 [Source:HGNC Symbol;Acc:HGNC:2151]
ENSG00000128045	RASL11B	-1.654103313	1.286967259	5.62E-12	1.65E-10	RAS like family 11 member B [Source:HGNC Symbol;Acc:HGNC:23804]
ENSG00000139973	SYT16	-1.653842766	2.639838386	3.80E-08	6.36E-07	synaptotagmin 16 [Source:HGNC Symbol;Acc:HGNC:23142]
ENSG00000178695	KCTD12	-1.653348089	4.635103254	1.46E-17	8.18E-16	potassium channel tetramerization domain containing 12 [Source:HGNC Symbol;Acc:HGNC:14678]
ENSG00000187944	C2orf66	-1.652718692	-0.345393884	1.17E-09	2.55E-08	chromosome 2 open reading frame 66 [Source:HGNC Symbol;Acc:HGNC:33809]
ENSG00000157601	MX1	-1.65213197	-0.262769669	1.32E-09	2.84E-08	MX dynamin like GTPase 1 [Source:HGNC Symbol;Acc:HGNC:7532]
ENSG00000252291	None	-1.651833617	-0.458720373	6.87E-10	1.55E-08	None
ENSG00000152056	AP1S3	-1.650158265	2.621994015	6.21E-11	1.60E-09	adaptor related protein complex 1 subunit sigma 3 [Source:HGNC Symbol;Acc:HGNC:18971]
ENSG00000259459	LINC02568	-1.650127033	0.293025925	2.91E-11	7.89E-10	long intergenic non-protein coding RNA 2568 [Source:HGNC Symbol;Acc:HGNC:51430]
ENSG00000249695	None	-1.649454474	1.777087271	1.70E-08	3.04E-07	novel transcript
ENSG00000182050	MGAT4C	-1.649261411	2.666416293	2.85E-14	1.09E-12	MGAT4 family member C [Source:HGNC Symbol;Acc:HGNC:30871]
ENSG00000271303	SRXN1	-1.648964385	2.334108424	9.63E-17	4.88E-15	sulfiredoxin 1 [Source:HGNC Symbol;Acc:HGNC:16132]
ENSG00000250764	None	-1.648511379	1.551587787	2.12E-14	8.24E-13	novel transcript
ENSG00000137841	PLCB2	-1.648287179	0.996243675	5.26E-11	1.37E-09	phospholipase C beta 2 [Source:HGNC Symbol;Acc:HGNC:9055]
ENSG00000263970	None	-1.648286402	-0.51057654	1.01E-08	1.90E-07	novel transcript
ENSG00000226067	LINC00623	-1.647727641	0.486752157	3.32E-11	8.94E-10	long intergenic non-protein coding RNA 623 [Source:HGNC Symbol;Acc:HGNC:44252]
ENSG00000245848	CEBPA	-1.646355242	0.929763236	2.86E-10	6.76E-09	CCAAT enhancer binding protein alpha [Source:HGNC Symbol;Acc:HGNC:1833]
ENSG00000104899	AMH	-1.64630845	4.189621036	3.28E-08	5.55E-07	anti-Mullerian hormone [Source:HGNC Symbol;Acc:HGNC:464]
ENSG00000223591	CENPVL1	-1.644697464	0.54772211	7.93E-09	1.51E-07	centromere protein V like 1 [Source:HGNC Symbol;Acc:HGNC:31851]
ENSG00000116544	DLGAP3	-1.644309485	4.825137169	5.99E-09	1.17E-07	DLG associated protein 3 [Source:HGNC Symbol;Acc:HGNC:30368]
ENSG00000149571	KIRREL3	-1.641939664	4.994477757	8.22E-10	1.83E-08	kirre like nephrin family adhesion molecule 3 [Source:HGNC Symbol;Acc:HGNC:23204]
ENSG00000187624	C17orf97	-1.641322811	2.546003732	2.39E-14	9.19E-13	chromosome 17 open reading frame 97 [Source:HGNC Symbol;Acc:HGNC:33800]
ENSG00000134533	RERG	-1.640391675	2.499245016	1.20E-12	3.83E-11	RAS like estrogen regulated growth inhibitor [Source:HGNC Symbol;Acc:HGNC:15980]
ENSG00000077279	DCX	-1.636841696	9.672377642	4.79E-14	1.79E-12	doublecortin [Source:HGNC Symbol;Acc:HGNC:2714]
ENSG00000164116	GUCY1A1	-1.636250811	2.480895843	5.50E-11	1.43E-09	guanylate cyclase 1 soluble subunit alpha 1 [Source:HGNC Symbol;Acc:HGNC:4685]
ENSG00000127325	BEST3	-1.636097705	-0.366697668	2.00E-10	4.82E-09	bestrophin 3 [Source:HGNC Symbol;Acc:HGNC:17105]
ENSG00000149575	SCN2B	-1.635505621	1.116335735	2.45E-06	2.89E-05	sodium voltage-gated channel beta subunit 2 [Source:HGNC Symbol;Acc:HGNC:10589]
ENSG00000119771	KLHL29	-1.635260691	5.368307193	1.17E-19	7.73E-18	kelch like family member 29 [Source:HGNC Symbol;Acc:HGNC:29404]
ENSG00000075426	FOSL2	-1.632353273	2.907138986	1.27E-08	2.33E-07	FOS like 2
ENSG00000065054	SLC9A3R2	-1.631194021	2.316732482	1.57E-14	6.25E-13	SLC9A3 regulator 2 [Source:HGNC Symbol;Acc:HGNC:11076]
ENSG00000233327	USP32P2	-1.631184866	2.71872265	7.87E-16	3.62E-14	ubiquitin specific peptidase 32 pseudogene 2 [Source:HGNC Symbol;Acc:HGNC:30751]
ENSG00000104833	TUBB4A	-1.630977974	6.210616403	2.81E-12	8.56E-11	tubulin beta 4A class IVa [Source:HGNC Symbol;Acc:HGNC:20774]
ENSG00000162746	FCRLB	-1.629165949	-0.447648705	6.38E-13	2.13E-11	Fc receptor like B [Source:HGNC Symbol;Acc:HGNC:26431]
ENSG00000152214	RIT2	-1.626601543	0.151762029	1.18E-05	0.000120351	Ras like without CAAX 2 [Source:HGNC Symbol;Acc:HGNC:10017]
ENSG00000166106	ADAMTS15	-1.626263808	1.475085265	3.12E-12	9.45E-11	ADAM metallopeptidase with thrombospondin type 1 motif 15 [Source:HGNC Symbol;Acc:HGNC:16305]
ENSG00000187527	ATP13A5	-1.625843866	-0.777347998	3.95E-10	9.24E-09	ATPase 13A5 [Source:HGNC Symbol;Acc:HGNC:31789]
ENSG00000147437	GNRH1	-1.624856196	1.883669291	8.20E-09	1.56E-07	gonadotropin releasing hormone 1 [Source:HGNC Symbol;Acc:HGNC:4419]
ENSG00000101134	DOK5	-1.62264126	4.41439836	1.28E-17	7.15E-16	docking protein 5 [Source:HGNC Symbol;Acc:HGNC:16173]
ENSG00000181381	DDX60L	-1.621691597	0.408892609	2.10E-09	4.41E-08	DExD/H-box 60 like [Source:HGNC Symbol;Acc:HGNC:26429]
ENSG00000156395	SORCS3	-1.620250697	4.044415349	6.11E-24	6.10E-22	sortilin related VPS10 domain containing receptor 3 [Source:HGNC Symbol;Acc:HGNC:16699]
ENSG00000233175	FMNL1-AS1	-1.619553085	1.632270232	3.74E-07	5.19E-06	FMNL1 antisense RNA 1 [Source:HGNC Symbol;Acc:HGNC:55717]
ENSG00000120162	MOB3B	-1.619323743	3.866089718	3.23E-13	1.12E-11	MOB kinase activator 3B [Source:HGNC Symbol;Acc:HGNC:23825]
ENSG00000202609	MIR488	-1.618473543	-0.411000487	2.53E-09	5.25E-08	microRNA 488 [Source:HGNC Symbol;Acc:HGNC:32073]
ENSG00000134326	CMPK2	-1.617757223	0.715562663	4.15E-10	9.67E-09	cytidine/uridine monophosphate kinase 2 [Source:HGNC Symbol;Acc:HGNC:27015]
ENSG00000136883	KIF12	-1.615846901	0.135149312	4.02E-11	1.07E-09	kinesin family member 12 [Source:HGNC Symbol;Acc:HGNC:21495]
ENSG00000231728	TMSB15B-AS1	-1.615592278	1.923233924	2.99E-12	9.09E-11	TMSB15B antisense RNA 1 [Source:HGNC Symbol;Acc:HGNC:53858]
ENSG00000112561	TFEB	-1.614638027	2.162061447	1.24E-08	2.27E-07	transcription factor EB [Source:HGNC Symbol;Acc:HGNC:11753]
ENSG00000250692	None	-1.613554756	1.215018632	6.89E-08	1.10E-06	novel transcript
ENSG00000256443	None	-1.613192467	1.35902117	1.11E-09	2.42E-08	novel transcript
ENSG00000268191	None	-1.613087574	4.189445662	1.05E-09	2.29E-08	novel transcript
ENSG00000234345	ELF2P1	-1.611746185	0.44958382	7.92E-11	2.01E-09	ELF2 pseudogene 1 [Source:HGNC Symbol;Acc:HGNC:33539]
ENSG00000268203	None	-1.610534884	3.646354996	4.20E-09	8.43E-08	novel transcript
ENSG00000252977	RNA5SP70	-1.609727103	-0.801064441	5.36E-08	8.68E-07	RNA
ENSG00000263873	THY1-AS1	-1.608910716	3.519278549	5.94E-08	9.56E-07	THY1 antisense RNA 1 [Source:HGNC Symbol;Acc:HGNC:54852]
ENSG00000131773	KHDRBS3	-1.606381559	5.180159824	7.34E-20	4.97E-18	KH RNA binding domain containing
ENSG00000172878	METAP1D	-1.605966299	4.11047341	5.42E-10	1.24E-08	methionyl aminopeptidase type 1D
ENSG00000234892	None	-1.605289047	0.291021773	1.97E-08	3.48E-07	novel transcript
ENSG00000264860	None	-1.604632855	4.289880477	1.08E-16	5.46E-15	novel transcript
ENSG00000125895	TMEM74B	-1.603167272	3.929122969	3.52E-12	1.06E-10	transmembrane protein 74B [Source:HGNC Symbol;Acc:HGNC:15893]
ENSG00000160190	SLC37A1	-1.599855709	4.249171893	2.89E-16	1.38E-14	solute carrier family 37 member 1 [Source:HGNC Symbol;Acc:HGNC:11024]
ENSG00000102349	KLF8	-1.598723975	2.273401313	7.05E-15	2.89E-13	KLF transcription factor 8 [Source:HGNC Symbol;Acc:HGNC:6351]
ENSG00000124772	CPNE5	-1.59650228	3.098762166	1.12E-12	3.59E-11	copine 5 [Source:HGNC Symbol;Acc:HGNC:2318]
ENSG00000254418	SPON1-AS1	-1.595924718	2.351775516	6.29E-11	1.62E-09	SPON1 antisense RNA 1 [Source:HGNC Symbol;Acc:HGNC:53117]
ENSG00000157219	HTR5A	-1.594589892	0.677915946	3.42E-06	3.91E-05	5-hydroxytryptamine receptor 5A [Source:HGNC Symbol;Acc:HGNC:5300]
ENSG00000058404	CAMK2B	-1.593629971	5.746677537	6.43E-11	1.65E-09	calcium/calmodulin dependent protein kinase II beta [Source:HGNC Symbol;Acc:HGNC:1461]
ENSG00000257225	None	-1.592820178	1.943112201	2.59E-14	9.90E-13	novel transcript
ENSG00000040731	CDH10	-1.592254236	4.345752954	7.59E-10	1.70E-08	cadherin 10 [Source:HGNC Symbol;Acc:HGNC:1749]
ENSG00000180155	LYNX1	-1.592212011	2.949602358	2.52E-17	1.36E-15	Ly6/neurotoxin 1 [Source:HGNC Symbol;Acc:HGNC:29604]
ENSG00000122025	FLT3	-1.591380335	-0.214508773	2.50E-09	5.21E-08	fms related receptor tyrosine kinase 3 [Source:HGNC Symbol;Acc:HGNC:3765]
ENSG00000232599	None	-1.590567326	0.593188138	5.86E-13	1.97E-11	odz
ENSG00000256540	IQSEC3-AS1	-1.590319983	3.589578848	4.85E-12	1.43E-10	IQSEC3 antisense RNA 1 [Source:HGNC Symbol;Acc:HGNC:56002]
ENSG00000171798	KNDC1	-1.590110321	4.401290791	4.80E-11	1.26E-09	kinase non-catalytic C-lobe domain containing 1 [Source:HGNC Symbol;Acc:HGNC:29374]
ENSG00000132164	SLC6A11	-1.5883113	3.136504054	1.80E-05	0.000175234	solute carrier family 6 member 11 [Source:HGNC Symbol;Acc:HGNC:11044]
ENSG00000267707	None	-1.586776935	5.351661206	2.54E-12	7.75E-11	novel transcript
ENSG00000233806	LINC01237	-1.585171027	4.160946588	5.48E-14	2.02E-12	long intergenic non-protein coding RNA 1237 [Source:HGNC Symbol;Acc:HGNC:49793]
ENSG00000267121	FMNL1-DT	-1.583651722	0.186962005	4.10E-12	1.22E-10	FMNL1 divergent transcript [Source:HGNC Symbol;Acc:HGNC:55311]
ENSG00000058866	DGKG	-1.583601898	1.350301431	3.53E-06	4.02E-05	diacylglycerol kinase gamma [Source:HGNC Symbol;Acc:HGNC:2853]

ENSG00000258842	None	-1.583522628	0.978920902	1.48E-08	2.68E-07	novel transcript
ENSG00000169255	B3GALNT1	-1.582199296	3.945568578	2.33E-09	4.87E-08	beta-1
ENSG00000145113	MUC4	-1.582056453	-0.139078463	2.82E-06	3.29E-05	mucin 4
ENSG00000237181	PRKAR1B-AS1	-1.574620777	1.972726136	5.24E-08	8.52E-07	PRKAR1B antisense RNA 1 [Source:HGNC Symbol;Acc:HGNC:40470]
ENSG00000241684	ADAMTS9-AS2	-1.574164302	2.540838205	1.08E-13	3.87E-12	ADAMTS9 antisense RNA 2 [Source:HGNC Symbol;Acc:HGNC:42435]
ENSG00000197872	CYRIA	-1.571854985	5.253855033	3.02E-11	8.17E-10	CYFIP related Rac1 interactor A [Source:HGNC Symbol;Acc:HGNC:25373]
ENSG00000253570	RNF5P1	-1.569968756	1.809634071	4.53E-11	1.19E-09	ring finger protein 5 pseudogene 1 [Source:HGNC Symbol;Acc:HGNC:31053]
ENSG00000177807	KCNJ10	-1.569534578	0.720989185	3.73E-12	1.12E-10	potassium inwardly rectifying channel subfamily J member 10 [Source:HGNC Symbol;Acc:HGNC:6256]
ENSG00000241749	RPSAP52	-1.569235609	3.321970998	1.60E-06	1.95E-05	ribosomal protein SA pseudogene 52 [Source:HGNC Symbol;Acc:HGNC:35752]
ENSG00000188176	SMTNL2	-1.567972663	-0.335245356	2.69E-08	4.64E-07	smoothelin like 2 [Source:HGNC Symbol;Acc:HGNC:24764]
ENSG00000188859	FAM78B	-1.56715369	3.91388894	1.00E-06	1.28E-05	family with sequence similarity 78 member B [Source:HGNC Symbol;Acc:HGNC:13495]
ENSG00000244490	RWDD4P1	-1.56707213	0.060128581	0.00031279	0.002170572	RWD domain containing 4 pseudogene 1 [Source:HGNC Symbol;Acc:HGNC:23753]
ENSG00000267491	None	-1.565694842	0.770712741	4.35E-12	1.29E-10	novel transcript
ENSG00000147036	LANCL3	-1.564120567	0.426987259	1.83E-10	4.44E-09	LanC like family member 3 [Source:HGNC Symbol;Acc:HGNC:24767]
ENSG00000240204	SMKR1	-1.562382056	0.475996908	8.49E-13	2.79E-11	small lysine rich protein 1 [Source:HGNC Symbol;Acc:HGNC:43561]
ENSG00000146090	RASGEF1C	-1.560651016	1.99343697	2.01E-13	7.06E-12	RasGEF domain family member 1C [Source:HGNC Symbol;Acc:HGNC:27400]
ENSG00000128604	IRF5	-1.559769343	-0.491012996	1.03E-08	1.92E-07	interferon regulatory factor 5 [Source:HGNC Symbol;Acc:HGNC:6120]
ENSG00000196104	SPOCK3	-1.559381973	4.067968208	1.05E-07	1.62E-06	SPARC (osteonectin)
ENSG00000065989	PDE4A	-1.558906549	3.748478141	3.02E-19	1.91E-17	phosphodiesterase 4A [Source:HGNC Symbol;Acc:HGNC:8780]
ENSG00000233009	NALCN-AS1	-1.557090105	1.736961744	1.32E-08	2.42E-07	NALCN antisense RNA 1 [Source:HGNC Symbol;Acc:HGNC:42743]
ENSG00000157005	SST	-1.556545794	2.788397843	1.73E-11	4.81E-10	somatostatin [Source:HGNC Symbol;Acc:HGNC:11329]
ENSG00000164683	HEY1	-1.554297445	3.035951656	8.27E-15	3.37E-13	hes related family bHLH transcription factor with YRPW motif 1 [Source:HGNC Symbol;Acc:HGNC:4880]
ENSG00000118898	PPL	-1.554016929	1.467014903	3.35E-12	1.01E-10	periplakin [Source:HGNC Symbol;Acc:HGNC:9273]
ENSG00000174473	GALNTL6	-1.55313477	3.097690384	3.05E-11	8.26E-10	polypeptide N-acetylgalactosaminyltransferase like 6 [Source:HGNC Symbol;Acc:HGNC:33844]
ENSG00000179909	ZNF154	-1.551866295	4.429604602	4.39E-20	3.04E-18	zinc finger protein 154 [Source:HGNC Symbol;Acc:HGNC:12939]
ENSG00000272733	None	-1.550903932	-0.599922252	6.87E-09	1.33E-07	novel transcript
ENSG00000124493	GRM4	-1.550177404	3.712079909	6.33E-08	1.01E-06	glutamate metabotropic receptor 4 [Source:HGNC Symbol;Acc:HGNC:4596]
ENSG00000090975	PITPNM2	-1.549772694	3.607501562	9.13E-13	2.97E-11	phosphatidylinositol transfer protein membrane associated 2 [Source:HGNC Symbol;Acc:HGNC:21044]
ENSG00000197956	S100A6	-1.549581217	0.903718844	7.13E-09	1.37E-07	S100 calcium binding protein A6 [Source:HGNC Symbol;Acc:HGNC:10496]
ENSG00000107819	SFXN3	-1.549041844	4.392326327	8.37E-08	1.31E-06	sideroflexin 3 [Source:HGNC Symbol;Acc:HGNC:16087]
ENSG00000006757	PNPLA4	-1.548680376	1.56450825	1.18E-12	3.79E-11	patatin like phospholipase domain containing 4 [Source:HGNC Symbol;Acc:HGNC:24887]
ENSG00000117598	PLPPR5	-1.548224024	3.223343959	1.18E-12	3.79E-11	phospholipid phosphatase related 5 [Source:HGNC Symbol;Acc:HGNC:31703]
ENSG00000185008	ROBO2	-1.547953468	6.52646705	1.69E-25	1.91E-23	roundabout guidance receptor 2 [Source:HGNC Symbol;Acc:HGNC:10250]
ENSG00000233642	GPR158-AS1	-1.547113362	0.133829102	2.81E-09	5.77E-08	GPR158 antisense RNA 1 [Source:HGNC Symbol;Acc:HGNC:44163]
ENSG00000264268	MIR4767	-1.546840532	-0.648234255	9.16E-10	2.02E-08	microRNA 4767 [Source:HGNC Symbol;Acc:HGNC:41548]
ENSG00000185761	ADAMTSL5	-1.54514816	-0.21388054	6.24E-11	1.61E-09	ADAMTS like 5 [Source:HGNC Symbol;Acc:HGNC:27912]
ENSG00000008283	CYB561	-1.544829967	3.180254023	1.60E-13	5.68E-12	cytochrome b561 [Source:HGNC Symbol;Acc:HGNC:2571]
ENSG00000267202	None	-1.544645544	3.721717468	5.68E-07	7.65E-06	novel transcript
ENSG00000082293	COL19A1	-1.544091411	0.825385859	2.73E-05	0.000252953	collagen type XIX alpha 1 chain [Source:HGNC Symbol;Acc:HGNC:2196]
ENSG00000172936	MYD88	-1.542777484	0.849289272	2.37E-08	4.13E-07	MYD88 innate immune signal transduction adaptor [Source:HGNC Symbol;Acc:HGNC:7562]
ENSG00000255929	PIWIL4-AS1	-1.542767656	1.259605467	1.75E-10	4.26E-09	PIWIL4 antisense RNA 1 [Source:HGNC Symbol;Acc:HGNC:55493]
ENSG00000229931	ATXN1-AS1	-1.541505613	1.111457187	4.34E-12	1.29E-10	ATXN1 antisense RNA 1 [Source:HGNC Symbol;Acc:HGNC:40515]
ENSG00000142408	CACNG8	-1.539214832	5.563355387	1.89E-06	2.28E-05	calcium voltage-gated channel auxiliary subunit gamma 8 [Source:HGNC Symbol;Acc:HGNC:13628]
ENSG00000228594	FNDC10	-1.538139396	3.051018865	6.83E-10	1.54E-08	fibronectin type III domain containing 10 [Source:HGNC Symbol;Acc:HGNC:42951]
ENSG00000180938	ZNF572	-1.538057213	-0.093976105	1.04E-10	2.61E-09	zinc finger protein 572 [Source:HGNC Symbol;Acc:HGNC:26758]
ENSG00000174460	ZCCHC12	-1.535667959	5.303236041	1.58E-09	3.36E-08	zinc finger CCHC-type containing 12 [Source:HGNC Symbol;Acc:HGNC:27273]
ENSG00000215237	None	-1.535160247	0.96229275	0.000117264	0.000917793	chromosome 4 open reading frame 27 (C4orf27) pseudogene
ENSG00000128564	VGF	-1.534516191	6.663977314	1.57E-06	1.93E-05	VGF nerve growth factor inducible [Source:HGNC Symbol;Acc:HGNC:12684]
ENSG00000184388	PABPC1L2B	-1.53402222	1.219051823	9.40E-08	1.46E-06	poly(A) binding protein cytoplasmic 1 like 2B [Source:HGNC Symbol;Acc:HGNC:31852]
ENSG00000184185	KCNJ12	-1.533504121	2.064274217	4.59E-13	1.56E-11	potassium inwardly rectifying channel subfamily J member 12 [Source:HGNC Symbol;Acc:HGNC:6258]
ENSG00000118257	NRP2	-1.533017659	6.825301549	4.35E-07	5.98E-06	neuropilin 2 [Source:HGNC Symbol;Acc:HGNC:8005]
ENSG00000167281	RBFOX3	-1.532347184	4.275167802	7.67E-23	6.81E-21	RNA binding fox-1 homolog 3 [Source:HGNC Symbol;Acc:HGNC:27097]
ENSG00000154096	THY1	-1.532264275	4.432596526	4.85E-08	7.94E-07	Thy-1 cell surface antigen [Source:HGNC Symbol;Acc:HGNC:11801]
ENSG00000100604	CHGA	-1.530192826	5.319394241	4.33E-18	2.51E-16	chromogranin A [Source:HGNC Symbol;Acc:HGNC:1929]
ENSG00000249955	None	-1.529143959	1.552738501	9.08E-12	2.59E-10	novel transcript
ENSG00000170214	ADRA1B	-1.528165261	0.732675904	8.68E-10	1.93E-08	adrenoceptor alpha 1B [Source:HGNC Symbol;Acc:HGNC:278]
ENSG00000080561	MID2	-1.526088725	2.442779898	1.58E-07	2.33E-06	midline 2 [Source:HGNC Symbol;Acc:HGNC:7096]
ENSG00000261324	None	-1.525502665	0.632833259	4.06E-09	8.17E-08	novel transcript
ENSG00000163359	COL6A3	-1.525209039	4.031565143	0.000230234	0.001661941	collagen type VI alpha 3 chain [Source:HGNC Symbol;Acc:HGNC:2213]
ENSG00000217236	SP9	-1.525107815	3.573346729	1.88E-10	4.54E-09	Sp9 transcription factor [Source:HGNC Symbol;Acc:HGNC:30690]
ENSG00000117602	RCAN3	-1.524879619	3.560288021	9.87E-07	1.26E-05	RCAN family member 3 [Source:HGNC Symbol;Acc:HGNC:3042]
ENSG00000264443	NCMAP-DT	-1.523943358	1.889209666	8.24E-05	0.000670886	NCMAP divergent transcript [Source:HGNC Symbol;Acc:HGNC:54327]
ENSG00000115380	EFEMP1	-1.523129772	0.944407201	3.53E-08	5.95E-07	EGF containing fibulin extracellular matrix protein 1 [Source:HGNC Symbol;Acc:HGNC:3218]
ENSG00000251294	None	-1.521178961	1.709775036	3.92E-08	6.54E-07	novel transcript
ENSG00000130707	ASS1	-1.520664261	3.605834916	1.40E-11	3.93E-10	argininosuccinate synthase 1 [Source:HGNC Symbol;Acc:HGNC:758]
ENSG00000087085	ACHE	-1.520179734	3.658176444	3.30E-16	1.56E-14	acetylcholinesterase (Cartwright blood group) [Source:HGNC Symbol;Acc:HGNC:108]
ENSG00000225206	MIR137HG	-1.519306974	1.774701518	7.48E-06	7.96E-05	MIR137 host gene [Source:HGNC Symbol;Acc:HGNC:42871]
ENSG00000124302	CHST8	-1.518474034	2.819526735	1.56E-07	2.31E-06	carbohydrate sulfotransferase 8 [Source:HGNC Symbol;Acc:HGNC:15993]
ENSG00000162753	SLC9C2	-1.518341935	1.24178465	6.96E-06	7.47E-05	solute carrier family 9 member C2 (putative) [Source:HGNC Symbol;Acc:HGNC:28664]
ENSG00000265126	None	-1.518328721	0.130914458	1.10E-09	2.40E-08	novel transcript
ENSG00000128253	RFPL2	-1.5178178	2.218714272	0.001221047	0.006902724	ret finger protein like 2 [Source:HGNC Symbol;Acc:HGNC:9979]
ENSG00000263680	None	-1.517443761	-0.491499803	5.01E-10	1.15E-08	novel transcript
ENSG00000179083	FAM133A	-1.516723999	1.618693176	3.61E-13	1.24E-11	family with sequence similarity 133 member A [Source:HGNC Symbol;Acc:HGNC:26748]
ENSG00000183691	NOG	-1.516428701	2.853081527	0.000254918	0.001814482	noggin [Source:HGNC Symbol;Acc:HGNC:7866]
ENSG00000229981	LINC01435	-1.516398386	-0.216933168	1.02E-06	1.30E-05	long intergenic non-protein coding RNA 1435 [Source:HGNC Symbol;Acc:HGNC:50753]
ENSG00000186288	PABPC1L2A	-1.515917062	1.300616689	7.90E-08	1.24E-06	poly(A) binding protein cytoplasmic 1 like 2A [Source:HGNC Symbol;Acc:HGNC:27989]
ENSG00000238186	None	-1.515399961	0.601219339	1.20E-10	2.99E-09	novel transcript
ENSG00000116983	HPCAL4	-1.515147075	5.297536089	1.72E-10	4.20E-09	hippocalcin like 4 [Source:HGNC Symbol;Acc:HGNC:18212]
ENSG00000256043	CTSO	-1.51451481	0.76279533	4.14E-08	6.88E-07	cathepsin O [Source:HGNC Symbol;Acc:HGNC:2542]
ENSG00000172985	SH3RF3	-1.51341899	3.668619203	1.15E-08	2.12E-07	SH3 domain containing ring finger 3 [Source:HGNC Symbol;Acc:HGNC:24699]
ENSG00000079337	RAPGEF3	-1.512249064	1.605734627	2.62E-12	7.98E-11	Rap guanine nucleotide exchange factor 3 [Source:HGNC Symbol;Acc:HGNC:16629]
ENSG00000254222	None	-1.51186504	0.004114966	2.02E-06	2.42E-05	novel transcript
ENSG00000171094	ALK	-1.510598857	3.304144025	1.64E-07	2.41E-06	ALK receptor tyrosine kinase [Source:HGNC Symbol;Acc:HGNC:427]
ENSG00000119408	NEK6	-1.506158631	4.390115869	1.98E-12	6.14E-11	NIMA related kinase 6 [Source:HGNC Symbol;Acc:HGNC:7749]
ENSG00000155511	GRIA1	-1.50572709	7.820081178	1.10E-19	7.33E-18	glutamate ionotropic receptor AMPA type subunit 1 [Source:HGNC Symbol;Acc:HGNC:4571]
ENSG00000101489	CELF4	-1.504011602	7.266910392	2.60E-11	7.10E-10	CUGBP Elav-like family member 4 [Source:HGNC Symbol;Acc:HGNC:14015]
ENSG00000123570	RAB9B	-1.503086651	4.699002598	4.13E-13	1.41E-11	RAB9B
ENSG00000114279	FGF12	-1.502999074	3.868887849	3.75E-16	1.77E-14	fibroblast growth factor 12 [Source:HGNC Symbol;Acc:HGNC:3668]
ENSG00000181773	GPR3	-1.502936798	0.486776236	7.59E-11	1.93E-09	G protein-coupled receptor 3 [Source:HGNC Symbol;Acc:HGNC:4484]
ENSG00000137628	DDX60	-1.50234422	0.924317303	5.41E-09	1.06E-07	DExD/H-box helicase 60 [Source:HGNC Symbol;Acc:HGNC:25942]

ENSG00000110324	IL10RA	-1.502133153	-0.364769801	6.67E-09	1.29E-07	interleukin 10 receptor subunit alpha [Source:HGNC Symbol;Acc:HGNC:5964]
ENSG00000267751	BSG-AS1	-1.50181365	-0.213612062	2.16E-08	3.80E-07	BSG antisense RNA 1 [Source:HGNC Symbol;Acc:HGNC:55286]
ENSG00000233493	TMEM238	-1.501687461	-0.827949019	1.48E-08	2.68E-07	transmembrane protein 238 [Source:HGNC Symbol;Acc:HGNC:40042]
ENSG00000231245	C1DP1	-1.500886682	-0.750580163	1.67E-08	2.99E-07	C1D nuclear receptor corepressor pseudogene 1 [Source:HGNC Symbol;Acc:HGNC:31465]
ENSG00000057593	F7	-1.500563449	-0.397759995	2.21E-05	0.000210931	coagulation factor VII [Source:HGNC Symbol;Acc:HGNC:3544]
ENSG00000127561	SYNGR3	-1.500266361	4.10469966	7.17E-21	5.23E-19	synaptogyrin 3 [Source:HGNC Symbol;Acc:HGNC:11501]
ENSG00000078114	NEBL	-1.499040118	3.765557874	4.34E-19	2.70E-17	nebulette [Source:HGNC Symbol;Acc:HGNC:16932]
ENSG00000262412	None	-1.499014045	-0.213266468	2.77E-09	5.71E-08	novel transcript
ENSG00000161243	FBXO27	-1.498426215	2.065938462	7.15E-11	1.83E-09	F-box protein 27 [Source:HGNC Symbol;Acc:HGNC:18753]
ENSG00000166676	TVP23A	-1.498383295	3.49048159	7.53E-15	3.08E-13	trans-golgi network vesicle protein 23 homolog A [Source:HGNC Symbol;Acc:HGNC:20398]
ENSG00000258986	TMEM179	-1.498248947	4.440086931	4.94E-21	3.67E-19	transmembrane protein 179 [Source:HGNC Symbol;Acc:HGNC:20137]
ENSG00000232057	None	-1.497782398	0.034494444	0.000246306	0.001762155	novel transcript
ENSG00000159176	CSRP1	-1.497674285	2.124319172	2.51E-12	7.67E-11	cysteine and glycine rich protein 1 [Source:HGNC Symbol;Acc:HGNC:2469]
ENSG00000179431	FJX1	-1.496793893	5.640820548	7.01E-31	1.18E-28	four-jointed box kinase 1 [Source:HGNC Symbol;Acc:HGNC:17166]
ENSG00000230327	MTCO1P42	-1.496370466	-0.826022096	2.88E-07	4.06E-06	MT-CO1 pseudogene 42 [Source:HGNC Symbol;Acc:HGNC:52107]
ENSG00000133134	BEX2	-1.495836848	5.72412601	3.41E-19	2.14E-17	brain expressed X-linked 2 [Source:HGNC Symbol;Acc:HGNC:30933]
ENSG00000130021	PUDP	-1.494969593	3.782606834	1.06E-17	6.01E-16	pseudouridine 5'-phosphatase [Source:HGNC Symbol;Acc:HGNC:16818]
ENSG00000233087	RAB6D	-1.493894478	1.25024014	7.40E-10	1.66E-08	RAB6D
ENSG00000267296	CEBPA-DT	-1.491679707	0.745808858	3.90E-11	1.04E-09	CEBPA divergent transcript [Source:HGNC Symbol;Acc:HGNC:25710]
ENSG00000225216	None	-1.491497059	2.572805259	4.03E-06	4.53E-05	novel transcript
ENSG00000266127	ZNF415P1	-1.489886051	-0.564776751	1.55E-09	3.31E-08	zinc finger protein 415 pseudogene 1 [Source:HGNC Symbol;Acc:HGNC:49869]
ENSG00000253733	LZTS1-AS1	-1.487980427	-0.137201779	3.40E-08	5.73E-07	LZTS1 antisense RNA 1 [Source:HGNC Symbol;Acc:HGNC:43630]
ENSG00000205683	DPF3	-1.487698888	2.576134081	7.42E-12	2.13E-10	double PHD fingers 3 [Source:HGNC Symbol;Acc:HGNC:17427]
ENSG00000178171	AMER3	-1.487038962	3.906408414	1.15E-12	3.69E-11	APC membrane recruitment protein 3 [Source:HGNC Symbol;Acc:HGNC:26771]
ENSG00000181031	RPH3AL	-1.486893123	1.513038537	5.59E-10	1.28E-08	rabphilin 3A like (without C2 domains) [Source:HGNC Symbol;Acc:HGNC:10296]
ENSG00000197837	H4C16	-1.486888663	-0.026769375	1.04E-10	2.60E-09	H4 histone 16 [Source:HGNC Symbol;Acc:HGNC:20510]
ENSG00000153930	ANKFN1	-1.486459134	1.326106006	1.77E-10	4.31E-09	ankyrin repeat and fibronectin type III domain containing 1 [Source:HGNC Symbol;Acc:HGNC:26766]
ENSG00000256576	LINC02361	-1.48621569	-0.205043119	1.16E-08	2.14E-07	long intergenic non-protein coding RNA 2361 [Source:HGNC Symbol;Acc:HGNC:53283]
ENSG00000107295	SH3GL2	-1.485982945	3.670581176	1.18E-09	2.56E-08	SH3 domain containing GRB2 like 2
ENSG00000128250	RFPL1	-1.483780511	3.254562103	2.60E-05	0.000243293	ret finger protein like 1 [Source:HGNC Symbol;Acc:HGNC:9977]
ENSG00000009694	TENM1	-1.48286253	5.998312931	2.90E-28	3.99E-26	teneurin transmembrane protein 1 [Source:HGNC Symbol;Acc:HGNC:8117]
ENSG00000250619	None	-1.481309577	4.197957399	2.27E-14	8.78E-13	novel transcript
ENSG00000204291	COL15A1	-1.481119709	0.696616548	9.84E-05	0.000783836	collagen type XV alpha 1 chain [Source:HGNC Symbol;Acc:HGNC:2192]
ENSG00000204060	FOXO6	-1.479614923	3.698143276	2.33E-05	0.000220309	forkhead box O6 [Source:HGNC Symbol;Acc:HGNC:24814]
ENSG00000227278	None	-1.479338749	0.51654492	2.75E-11	7.49E-10	novel transcript
ENSG00000267036	None	-1.479097972	0.4688466	6.36E-06	6.89E-05	Rho GTPase activating protein 33 (ARHGAP33) pseudogene
ENSG00000075429	CACNG5	-1.478178989	1.52002259	4.03E-11	1.07E-09	calcium voltage-gated channel auxiliary subunit gamma 5 [Source:HGNC Symbol;Acc:HGNC:1409]
ENSG00000142875	PRKACB	-1.477658872	6.760885037	1.76E-29	2.66E-27	protein kinase cAMP-activated catalytic subunit beta [Source:HGNC Symbol;Acc:HGNC:9381]
ENSG00000231937	None	-1.476765268	4.287108445	1.15E-07	1.75E-06	novel transcript
ENSG00000177932	ZNF354C	-1.476070463	4.227688216	9.16E-06	9.55E-05	zinc finger protein 354C [Source:HGNC Symbol;Acc:HGNC:16736]
ENSG00000155254	MARVELD1	-1.475983169	2.466462122	9.24E-10	2.04E-08	MARVEL domain containing 1 [Source:HGNC Symbol;Acc:HGNC:28674]
ENSG00000234352	None	-1.475439855	2.484179504	1.25E-07	1.89E-06	novel transcript
ENSG00000162374	ELAVL4	-1.473857261	6.217051954	6.28E-29	9.02E-27	ELAV like RNA binding protein 4 [Source:HGNC Symbol;Acc:HGNC:3315]
ENSG00000232434	AJM1	-1.473531361	3.346796045	2.98E-15	1.28E-13	apical junction component 1 homolog [Source:HGNC Symbol;Acc:HGNC:37284]
ENSG00000138161	CUZD1	-1.471841021	1.45433962	1.78E-05	0.00017413	CUB and zona pellucida like domains 1 [Source:HGNC Symbol;Acc:HGNC:17937]
ENSG00000205744	DENND1C	-1.471702778	0.334496916	4.28E-11	1.13E-09	DENN domain containing 1C [Source:HGNC Symbol;Acc:HGNC:26225]
ENSG00000168309	FAM107A	-1.471042326	4.818418981	6.05E-08	9.72E-07	family with sequence similarity 107 member A [Source:HGNC Symbol;Acc:HGNC:30827]
ENSG00000164002	EXO5	-1.469474586	2.401357802	2.40E-14	9.21E-13	exonuclease 5 [Source:HGNC Symbol;Acc:HGNC:26115]
ENSG00000047597	XK	-1.468106836	0.764423743	1.49E-10	3.67E-09	X-linked Kx blood group antigen
ENSG00000158258	CLSTN2	-1.46565737	4.976101075	5.95E-08	9.57E-07	calsyntenin 2 [Source:HGNC Symbol;Acc:HGNC:17448]
ENSG00000151079	KCNA6	-1.465483308	4.286850854	4.84E-09	9.57E-08	potassium voltage-gated channel subfamily A member 6 [Source:HGNC Symbol;Acc:HGNC:6225]
ENSG00000180543	TSPYL5	-1.464622649	1.982758601	2.86E-08	4.89E-07	TSPY like 5 [Source:HGNC Symbol;Acc:HGNC:29367]
ENSG00000166707	ZCCHC18	-1.464442328	3.991015318	2.16E-17	1.18E-15	zinc finger CCHC-type containing 18 [Source:HGNC Symbol;Acc:HGNC:32459]
ENSG00000074706	IPCEF1	-1.463820246	0.436108401	2.44E-06	2.88E-05	interaction protein for cytohesin exchange factors 1 [Source:HGNC Symbol;Acc:HGNC:21204]
ENSG00000213185	FAM24B	-1.463218544	1.350930661	1.90E-05	0.000183294	family with sequence similarity 24 member B [Source:HGNC Symbol;Acc:HGNC:23475]
ENSG00000250761	None	-1.461858043	2.173547186	0.001013167	0.005904732	novel transcript
ENSG00000258560	None	-1.460534573	2.235555118	6.69E-05	0.000558065	novel transcript
ENSG00000043143	JADE2	-1.460267654	2.547555408	7.56E-10	1.70E-08	jade family PHD finger 2 [Source:HGNC Symbol;Acc:HGNC:22984]
ENSG00000168461	RAB31	-1.460089671	4.371171818	1.53E-20	1.10E-18	RAB31
ENSG00000197177	ADGRA1	-1.458825005	4.237343216	2.17E-19	1.39E-17	adhesion G protein-coupled receptor A1 [Source:HGNC Symbol;Acc:HGNC:13838]
ENSG00000068366	ACSL4	-1.456874548	6.374355069	1.99E-17	1.09E-15	acyl-CoA synthetase long chain family member 4 [Source:HGNC Symbol;Acc:HGNC:3571]
ENSG00000120156	TEK	-1.456866195	0.761462407	4.29E-06	4.80E-05	TEK receptor tyrosine kinase [Source:HGNC Symbol;Acc:HGNC:11724]
ENSG00000139915	MDGA2	-1.456846332	4.665828124	1.93E-17	1.06E-15	MAM domain containing glycosylphosphatidylinositol anchor 2 [Source:HGNC Symbol;Acc:HGNC:19835]
ENSG00000270055	None	-1.454044311	1.213149943	9.59E-10	2.11E-08	novel transcript
ENSG00000184961	RPL7AP49	-1.452543763	0.092938362	8.61E-08	1.34E-06	ribosomal protein L7a pseudogene 49 [Source:HGNC Symbol;Acc:HGNC:35507]
ENSG00000245248	USP2-AS1	-1.450472127	4.533881707	4.29E-08	7.10E-07	USP2 antisense RNA 1 [Source:HGNC Symbol;Acc:HGNC:48673]
ENSG00000234257	SOD2P1	-1.448069574	-0.618825546	7.66E-08	1.21E-06	superoxide dismutase 2 pseudogene 1 [Source:HGNC Symbol;Acc:HGNC:45268]
ENSG00000119514	GALNT12	-1.446185051	1.639654813	1.05E-07	1.61E-06	polypeptide N-acetylgalactosaminyltransferase 12 [Source:HGNC Symbol;Acc:HGNC:19877]
ENSG00000246214	RETREG1-AS1	-1.443710256	0.96582286	4.94E-09	9.75E-08	RETREG1 antisense RNA 1 [Source:HGNC Symbol;Acc:HGNC:55551]
ENSG00000253368	TRNP1	-1.441747532	2.59142427	1.01E-10	2.55E-09	TMF1 regulated nuclear protein 1 [Source:HGNC Symbol;Acc:HGNC:34348]
ENSG00000250348	None	-1.441591221	0.099028512	6.95E-09	1.34E-07	novel transcript
ENSG00000144460	NYAP2	-1.438806516	2.297329088	4.24E-09	8.49E-08	neuronal tyrosine-phosphorylated phosphoinositide-3-kinase adaptor 2 [Source:HGNC Symbol;Acc:HGNC:29291]
ENSG00000103528	SYT17	-1.438767066	2.769794615	6.74E-07	8.94E-06	synaptotagmin 17 [Source:HGNC Symbol;Acc:HGNC:24119]
ENSG00000185567	AHNAK2	-1.438612303	4.243145432	3.91E-08	6.53E-07	AHNAK nucleoprotein 2 [Source:HGNC Symbol;Acc:HGNC:20125]
ENSG00000162670	BRINP3	-1.437297717	3.263938826	3.18E-05	0.000289467	BMP/retinoic acid inducible neural specific 3 [Source:HGNC Symbol;Acc:HGNC:22393]
ENSG00000232063	None	-1.435419852	4.027839389	9.99E-19	6.05E-17	novel transcript
ENSG00000126218	F10	-1.430151778	-0.218391734	2.70E-09	5.57E-08	coagulation factor X [Source:HGNC Symbol;Acc:HGNC:3528]
ENSG00000160963	COL26A1	-1.428949481	4.40262568	3.31E-17	1.76E-15	collagen type XXVI alpha 1 chain [Source:HGNC Symbol;Acc:HGNC:18038]
ENSG00000223843	EFCAB6-AS1	-1.428699056	-0.121093729	7.17E-06	7.66E-05	EFCAB6 antisense RNA 1 [Source:HGNC Symbol;Acc:HGNC:39999]
ENSG00000113361	CDH6	-1.428176767	3.532239827	2.30E-08	4.02E-07	cadherin 6 [Source:HGNC Symbol;Acc:HGNC:1765]
ENSG00000167994	RAB3IL1	-1.427293301	0.551244574	9.00E-10	1.99E-08	RAB3A interacting protein like 1 [Source:HGNC Symbol;Acc:HGNC:9780]
ENSG00000184368	MAP7D2	-1.427136633	3.522753868	1.91E-07	2.77E-06	MAP7 domain containing 2 [Source:HGNC Symbol;Acc:HGNC:25899]
ENSG00000143479	DYRK3	-1.426311757	3.353034098	6.11E-08	9.79E-07	dual specificity tyrosine phosphorylation regulated kinase 3 [Source:HGNC Symbol;Acc:HGNC:3094]
ENSG00000149527	PLCH2	-1.42566397	3.653402617	1.57E-10	3.84E-09	phospholipase C eta 2 [Source:HGNC Symbol;Acc:HGNC:29037]
ENSG00000178038	ALS2CL	-1.425325216	-0.473476879	1.52E-08	2.74E-07	ALS2 C-terminal like [Source:HGNC Symbol;Acc:HGNC:20605]
ENSG00000078295	ADCY2	-1.423224206	4.877548695	0.000193726	0.001435063	adenylate cyclase 2 [Source:HGNC Symbol;Acc:HGNC:233]
ENSG00000011677	GABRA3	-1.423061498	5.008596038	1.11E-08	2.06E-07	gamma-aminobutyric acid type A receptor subunit alpha3 [Source:HGNC Symbol;Acc:HGNC:4077]
ENSG00000214076	CPSF1P1	-1.421670561	2.838601975	3.69E-07	5.13E-06	CPSF1 pseudogene 1 [Source:HGNC Symbol;Acc:HGNC:19251]
ENSG00000134198	TSPAN2	-1.421374467	2.641524667	4.08E-10	9.51E-09	tetraspanin 2 [Source:HGNC Symbol;Acc:HGNC:20659]
ENSG00000174521	TTC9B	-1.42034525	3.888322612	8.19E-07	1.06E-05	tetratricopeptide repeat domain 9B [Source:HGNC Symbol;Acc:HGNC:26395]

ENSG00000228550	None	-1.420225766	-1.096094597	1.53E-07	2.27E-06	ribosomal protein
ENSG00000259425	None	-1.418840064	1.064814702	6.72E-11	1.72E-09	Golgin subfamily A member 6 pseudogene
ENSG00000226576	None	-1.417720107	0.917855958	0.001053429	0.006100564	novel transcript
ENSG00000155897	ADCY8	-1.41720839	2.605149167	2.26E-08	3.97E-07	adenylate cyclase 8 [Source:HGNC Symbol;Acc:HGNC:239]
ENSG00000162458	FBLIM1	-1.416618126	2.850815024	4.91E-10	1.13E-08	filamin binding LIM protein 1 [Source:HGNC Symbol;Acc:HGNC:24686]
ENSG00000113070	HBEGF	-1.416198147	2.535283381	1.18E-08	2.18E-07	heparin binding EGF like growth factor [Source:HGNC Symbol;Acc:HGNC:3059]
ENSG00000233766	CAVIN2-AS1	-1.415204534	3.719395611	4.30E-15	1.80E-13	CAVIN2 and TMEFF2 antisense RNA 1 [Source:HGNC Symbol;Acc:HGNC:40517]
ENSG00000157388	CACNA1D	-1.415072744	4.697721455	1.93E-12	5.98E-11	calcium voltage-gated channel subunit alpha1 D [Source:HGNC Symbol;Acc:HGNC:1391]
ENSG00000140285	FGF7	-1.414988467	-0.710185015	1.04E-07	1.60E-06	fibroblast growth factor 7 [Source:HGNC Symbol;Acc:HGNC:3685]
ENSG00000254942	None	-1.411547398	0.784699153	3.25E-05	0.000294978	novel transcript
ENSG00000272744	None	-1.410688452	-0.815243281	2.69E-06	3.15E-05	novel transcript
ENSG00000112902	SEMA5A	-1.40985892	4.955491513	4.12E-16	1.94E-14	semaphorin 5A [Source:HGNC Symbol;Acc:HGNC:10736]
ENSG00000048540	LMO3	-1.409806065	6.331263497	1.56E-15	6.97E-14	LIM domain only 3 [Source:HGNC Symbol;Acc:HGNC:6643]
ENSG00000147862	NFIB	-1.408753899	5.883569355	3.63E-14	1.37E-12	nuclear factor I B [Source:HGNC Symbol;Acc:HGNC:7785]
ENSG00000158458	NRG2	-1.40871436	3.813138594	2.94E-07	4.14E-06	neuregulin 2 [Source:HGNC Symbol;Acc:HGNC:7998]
ENSG00000119673	ACOT2	-1.407859852	1.537443468	6.94E-10	1.56E-08	acyl-CoA thioesterase 2 [Source:HGNC Symbol;Acc:HGNC:18431]
ENSG00000114115	RBP1	-1.407024607	2.377880131	6.74E-05	0.000561882	retinol binding protein 1 [Source:HGNC Symbol;Acc:HGNC:9919]
ENSG00000186868	MAPT	-1.406412156	8.618465969	2.92E-06	3.39E-05	microtubule associated protein tau [Source:HGNC Symbol;Acc:HGNC:6893]
ENSG00000164930	FZD6	-1.403591841	2.644766632	1.90E-08	3.38E-07	frizzled class receptor 6 [Source:HGNC Symbol;Acc:HGNC:4044]
ENSG00000197859	ADAMTSL2	-1.403199415	0.003311969	2.05E-06	2.45E-05	ADAMTS like 2 [Source:HGNC Symbol;Acc:HGNC:14631]
ENSG00000134917	ADAMTS8	-1.402658759	-0.550214788	7.44E-07	9.76E-06	ADAM metallopeptidase with thrombospondin type 1 motif 8 [Source:HGNC Symbol;Acc:HGNC:224]
ENSG00000167800	TBX10	-1.401582546	1.700441309	2.12E-10	5.09E-09	T-box transcription factor 10 [Source:HGNC Symbol;Acc:HGNC:11593]
ENSG00000112379	ARFGEF3	-1.39882509	5.345029856	4.05E-26	4.74E-24	ARFGEF family member 3 [Source:HGNC Symbol;Acc:HGNC:21213]
ENSG00000110786	PTPN5	-1.397408491	5.532030673	1.11E-17	6.27E-16	protein tyrosine phosphatase non-receptor type 5 [Source:HGNC Symbol;Acc:HGNC:9657]
ENSG00000132359	RAP1GAP2	-1.396210354	4.736998182	1.10E-19	7.32E-18	RAP1 GTPase activating protein 2 [Source:HGNC Symbol;Acc:HGNC:29176]
ENSG00000198121	LPAR1	-1.393853152	1.712612786	2.12E-09	4.45E-08	lysophosphatidic acid receptor 1 [Source:HGNC Symbol;Acc:HGNC:3166]
ENSG00000126217	MCF2L	-1.393148565	6.342710341	1.99E-13	7.00E-12	MCF.2 cell line derived transforming sequence like [Source:HGNC Symbol;Acc:HGNC:14576]
ENSG00000101347	SAMHD1	-1.392766804	3.494245464	9.03E-16	4.12E-14	SAM and HD domain containing deoxynucleoside triphosphate triphosphohydrolase 1 [Source:HGNC Symbol;Acc:HGNC:15925]
ENSG00000251034	None	-1.391657762	0.952128565	3.77E-09	7.62E-08	novel transcript
ENSG00000184905	TCEAL2	-1.39127417	5.181299028	6.29E-07	8.38E-06	transcription elongation factor A like 2 [Source:HGNC Symbol;Acc:HGNC:29818]
ENSG00000213578	CPLX3	-1.391225351	0.183059928	4.26E-05	0.000375023	complexin 3 [Source:HGNC Symbol;Acc:HGNC:27652]
ENSG00000162068	NTN3	-1.390324645	0.117488283	3.86E-10	9.04E-09	netrin 3 [Source:HGNC Symbol;Acc:HGNC:8030]
ENSG00000146858	ZC3HAV1L	-1.389094992	0.682198985	2.65E-10	6.29E-09	zinc finger CCCH-type containing
ENSG00000188338	SLC38A3	-1.388894671	0.792682667	2.44E-07	3.48E-06	solute carrier family 38 member 3 [Source:HGNC Symbol;Acc:HGNC:18044]
ENSG00000138496	PARP9	-1.387595802	-0.003982511	7.06E-06	7.56E-05	poly(ADP-ribose) polymerase family member 9 [Source:HGNC Symbol;Acc:HGNC:24118]
ENSG00000154429	CCSAP	-1.387548747	5.724807294	2.81E-11	7.65E-10	centriole
ENSG00000100095	SEZ6L	-1.387281115	6.618966779	1.66E-07	2.44E-06	seizure related 6 homolog like [Source:HGNC Symbol;Acc:HGNC:10763]
ENSG00000255624	C10orf88B	-1.386101917	-0.558307706	2.82E-07	3.98E-06	C10orf88B (pseudogene) [Source:HGNC Symbol;Acc:HGNC:44080]
ENSG00000230312	SLC25A53P1	-1.384739285	-0.677054137	2.39E-08	4.16E-07	SLC25A53 pseudogene 1 [Source:HGNC Symbol;Acc:HGNC:55040]
ENSG00000185477	GPRIN3	-1.384712377	3.660077272	3.19E-07	4.47E-06	GPRIN family member 3 [Source:HGNC Symbol;Acc:HGNC:27733]
ENSG00000174640	SLCO2A1	-1.383791891	-0.712429085	8.50E-06	8.95E-05	solute carrier organic anion transporter family member 2A1 [Source:HGNC Symbol;Acc:HGNC:10955]
ENSG00000251148	None	-1.382742553	0.33642523	7.29E-09	1.40E-07	novel transcript
ENSG00000187164	SHTN1	-1.382634917	6.352340911	7.61E-20	5.13E-18	shootin 1 [Source:HGNC Symbol;Acc:HGNC:29319]
ENSG00000260305	NTRK3-AS1	-1.382578127	2.053401346	3.44E-11	9.23E-10	NTRK3 antisense RNA 1 [Source:HGNC Symbol;Acc:HGNC:27532]
ENSG00000227200	None	-1.382086164	-0.255690488	1.12E-06	1.41E-05	novel transcript
ENSG00000160588	MPZL3	-1.381576298	-0.012979128	5.86E-05	0.000496285	myelin protein zero like 3 [Source:HGNC Symbol;Acc:HGNC:27279]
ENSG00000101342	TLDC2	-1.380390519	2.554241807	1.19E-12	3.80E-11	TBC/LysM-associated domain containing 2 [Source:HGNC Symbol;Acc:HGNC:16112]
ENSG00000113389	NPR3	-1.379836694	1.429172728	1.87E-09	3.95E-08	natriuretic peptide receptor 3 [Source:HGNC Symbol;Acc:HGNC:7945]
ENSG00000272755	None	-1.379663337	1.724422641	2.63E-05	0.000245597	novel transcript
ENSG00000215218	UBE2QL1	-1.379229517	4.713688919	1.15E-07	1.75E-06	ubiquitin conjugating enzyme E2 Q family like 1 [Source:HGNC Symbol;Acc:HGNC:37269]
ENSG00000259658	UBE2Q2P13	-1.376370037	0.18377734	1.00E-08	1.88E-07	UBE2Q2 pseudogene 13 [Source:HGNC Symbol;Acc:HGNC:56078]
ENSG00000256694	None	-1.376338041	0.129678152	1.14E-07	1.74E-06	novel transcript
ENSG00000164440	TXLNB	-1.376288453	-0.332259116	6.12E-09	1.19E-07	taxilin beta [Source:HGNC Symbol;Acc:HGNC:21617]
ENSG00000041515	MYO16	-1.375907174	3.863030964	1.10E-16	5.53E-15	myosin XVI [Source:HGNC Symbol;Acc:HGNC:29822]
ENSG00000224109	CENPVL3	-1.375839937	-0.499775071	6.97E-07	9.20E-06	centromere protein V like 3 [Source:HGNC Symbol;Acc:HGNC:43880]
ENSG00000152402	GUCY1A2	-1.374127564	5.366186695	8.68E-10	1.93E-08	guanylate cyclase 1 soluble subunit alpha 2 [Source:HGNC Symbol;Acc:HGNC:4684]
ENSG00000085563	ABCB1	-1.373879569	2.98029413	8.98E-11	2.27E-09	ATP binding cassette subfamily B member 1 [Source:HGNC Symbol;Acc:HGNC:40]
ENSG00000243384	None	-1.373438111	0.788172427	8.21E-07	1.06E-05	novel transcript
ENSG00000183831	ANKRD45	-1.373249681	2.323860291	7.90E-08	1.24E-06	ankyrin repeat domain 45 [Source:HGNC Symbol;Acc:HGNC:24786]
ENSG00000246375	PPM1K-DT	-1.372985833	0.275855349	1.85E-05	0.000178893	PPM1K divergent transcript [Source:HGNC Symbol;Acc:HGNC:54093]
ENSG00000136531	SCN2A	-1.371820649	5.724613138	2.44E-14	9.35E-13	sodium voltage-gated channel alpha subunit 2 [Source:HGNC Symbol;Acc:HGNC:10588]
ENSG00000151729	SLC25A4	-1.370898841	5.769925938	1.52E-09	3.24E-08	solute carrier family 25 member 4 [Source:HGNC Symbol;Acc:HGNC:10990]
ENSG00000170381	SEMA3E	-1.370630239	1.452127533	3.04E-06	3.51E-05	semaphorin 3E [Source:HGNC Symbol;Acc:HGNC:10727]
ENSG00000183570	PCBP3	-1.369701782	4.515917601	2.03E-19	1.31E-17	poly(rC) binding protein 3 [Source:HGNC Symbol;Acc:HGNC:8651]
ENSG00000185274	GALNT17	-1.367398599	4.22204674	2.42E-15	1.05E-13	polypeptide N-acetylgalactosaminyltransferase 17 [Source:HGNC Symbol;Acc:HGNC:16347]
ENSG00000151876	FBXO4	-1.366595386	0.400153773	6.97E-07	9.20E-06	F-box protein 4 [Source:HGNC Symbol;Acc:HGNC:13583]
ENSG00000197536	IRF1-AS1	-1.366509542	1.132897681	4.33E-10	1.01E-08	IRF1 antisense RNA 1 [Source:HGNC Symbol;Acc:HGNC:33838]
ENSG00000237481	None	-1.36618343	4.219821416	3.15E-08	5.36E-07	novel transcript
ENSG00000267026	None	-1.365685844	-0.262626528	2.09E-05	0.000200144	novel transcript
ENSG00000085831	TTC39A	-1.365492194	-0.268003803	4.59E-06	5.12E-05	tetratricopeptide repeat domain 39A [Source:HGNC Symbol;Acc:HGNC:18657]
ENSG00000146938	NLGN4X	-1.36526415	5.812825067	5.23E-25	5.77E-23	neuroligin 4 X-linked [Source:HGNC Symbol;Acc:HGNC:14287]
ENSG00000188316	ENO4	-1.365069328	5.994366371	1.05E-22	9.27E-21	enolase 4 [Source:HGNC Symbol;Acc:HGNC:31670]
ENSG00000238178	None	-1.362078075	-0.546939238	5.95E-09	1.16E-07	novel transcript
ENSG00000005884	ITGA3	-1.361030534	3.922292995	7.27E-12	2.09E-10	integrin subunit alpha 3 [Source:HGNC Symbol;Acc:HGNC:6139]
ENSG00000124140	SLC12A5	-1.358875656	5.121485899	4.66E-12	1.38E-10	solute carrier family 12 member 5 [Source:HGNC Symbol;Acc:HGNC:13818]
ENSG00000162692	VCAM1	-1.358725002	2.452329828	3.15E-08	5.36E-07	vascular cell adhesion molecule 1 [Source:HGNC Symbol;Acc:HGNC:12663]
ENSG00000137877	SPTBN5	-1.358147224	0.103764239	8.01E-07	1.04E-05	spectrin beta
ENSG00000203280	KIAA1671-AS1	-1.357239077	-0.358033265	1.46E-05	0.000145948	KIAA1671 antisense RNA 1 [Source:HGNC Symbol;Acc:HGNC:41238]
ENSG00000253144	None	-1.356745351	1.211129042	0.000134385	0.001035324	pseudogene similat to part of cold shock domain containing E1
ENSG00000179141	MTUS2-AS1	-1.356397623	-0.142101246	3.75E-07	5.20E-06	MTUS2 antisense RNA 1 [Source:HGNC Symbol;Acc:HGNC:40924]
ENSG00000002587	HS3ST1	-1.355658162	2.000072554	1.23E-11	3.48E-10	heparan sulfate-glucosamine 3-sulfotransferase 1 [Source:HGNC Symbol;Acc:HGNC:5194]
ENSG00000166448	TMEM130	-1.354211714	4.746565211	5.60E-14	2.07E-12	transmembrane protein 130 [Source:HGNC Symbol;Acc:HGNC:25429]
ENSG00000266149	None	-1.353865731	0.376204851	6.49E-07	8.62E-06	novel transcript
ENSG00000013293	SLC7A14	-1.352868128	3.754748389	8.26E-07	1.07E-05	solute carrier family 7 member 14 [Source:HGNC Symbol;Acc:HGNC:29326]
ENSG00000099822	HCN2	-1.351877148	4.273020834	1.57E-08	2.82E-07	hyperpolarization activated cyclic nucleotide gated potassium and sodium channel 2 [Source:HGNC Symbol;Acc:HGNC:4846]
ENSG00000236426	None	-1.351661625	-0.533297192	0.000337846	0.002318406	novel transcript
ENSG00000237337	ELAVL4-AS1	-1.350141917	1.235439269	1.48E-10	3.65E-09	ELAVL4 antisense RNA 1 [Source:HGNC Symbol;Acc:HGNC:40210]
ENSG00000115468	EFHD1	-1.350010436	1.13752133	2.58E-08	4.46E-07	EF-hand domain family member D1 [Source:HGNC Symbol;Acc:HGNC:29556]
ENSG00000258405	ZNF578	-1.349857775	0.051854028	2.19E-05	0.000208588	zinc finger protein 578 [Source:HGNC Symbol;Acc:HGNC:26449]
ENSG00000212380	SNORD115-45	-1.349183729	-1.053769629	6.71E-07	8.90E-06	small nucleolar RNA

ENSG00000254305	MRPL9P1	-1.348442737	0.192970409	1.77E-06	2.15E-05	mitochondrial ribosomal protein L9 pseudogene 1 [Source:HGNC Symbol;Acc:HGNC:29726]
ENSG00000234232	PDE4DIPP7	-1.347831932	3.277558115	1.73E-11	4.81E-10	PDE4DIP pseudogene 7 [Source:HGNC Symbol;Acc:HGNC:55098]
ENSG00000106789	CORO2A	-1.347455007	0.867645012	1.86E-09	3.92E-08	coronin 2A [Source:HGNC Symbol;Acc:HGNC:2255]
ENSG00000099994	SUSD2	-1.347008649	-0.266096467	1.09E-08	2.03E-07	sushi domain containing 2 [Source:HGNC Symbol;Acc:HGNC:30667]
ENSG00000122691	TWIST1	-1.346424261	0.070519633	2.22E-06	2.64E-05	twist family bHLH transcription factor 1 [Source:HGNC Symbol;Acc:HGNC:12428]
ENSG00000103449	SALL1	-1.345976094	5.677869119	8.55E-07	1.10E-05	spalt like transcription factor 1 [Source:HGNC Symbol;Acc:HGNC:10524]
ENSG00000074590	NUAK1	-1.344413515	5.140841623	1.85E-12	5.75E-11	NUAK family kinase 1 [Source:HGNC Symbol;Acc:HGNC:14311]
ENSG00000175229	GAL3ST3	-1.344228792	2.845642477	4.11E-13	1.41E-11	galactose-3-O-sulfotransferase 3 [Source:HGNC Symbol;Acc:HGNC:24144]
ENSG00000060656	PTPRU	-1.343936123	4.328814124	5.44E-14	2.01E-12	protein tyrosine phosphatase receptor type U [Source:HGNC Symbol;Acc:HGNC:9683]
ENSG00000145198	VWA5B2	-1.342000868	2.095143988	6.10E-10	1.39E-08	von Willebrand factor A domain containing 5B2 [Source:HGNC Symbol;Acc:HGNC:25144]
ENSG00000169047	IRS1	-1.34130032	4.351964092	7.19E-18	4.09E-16	insulin receptor substrate 1 [Source:HGNC Symbol;Acc:HGNC:6125]
ENSG00000158220	ESYT3	-1.341018299	-0.43934086	1.83E-08	3.25E-07	extended synaptotagmin 3 [Source:HGNC Symbol;Acc:HGNC:24295]
ENSG00000067842	ATP2B3	-1.340824719	4.014930343	7.95E-05	0.000650749	ATPase plasma membrane Ca2+ transporting 3 [Source:HGNC Symbol;Acc:HGNC:816]
ENSG00000131018	SYNE1	-1.339860866	5.969336746	9.81E-22	7.92E-20	spectrin repeat containing nuclear envelope protein 1 [Source:HGNC Symbol;Acc:HGNC:17089]
ENSG00000262881	None	-1.339476885	2.813485724	3.50E-05	0.000314876	novel transcript
ENSG00000157087	ATP2B2	-1.339300561	4.807494362	2.94E-08	5.03E-07	ATPase plasma membrane Ca2+ transporting 2 [Source:HGNC Symbol;Acc:HGNC:815]
ENSG00000164418	GRIK2	-1.338827565	5.241735914	2.11E-05	0.000202139	glutamate ionotropic receptor kainate type subunit 2 [Source:HGNC Symbol;Acc:HGNC:4580]
ENSG00000196660	SLC30A10	-1.336441769	1.927767091	4.10E-11	1.09E-09	solute carrier family 30 member 10 [Source:HGNC Symbol;Acc:HGNC:25355]
ENSG00000254966	IGSF22-AS1	-1.335448467	5.179816967	1.79E-18	1.06E-16	IGSF22 antisense RNA 1 [Source:HGNC Symbol;Acc:HGNC:55511]
ENSG00000233073	GRM3-AS1	-1.334991617	1.564112104	1.89E-08	3.36E-07	GRM3 antisense RNA 1 [Source:HGNC Symbol;Acc:HGNC:40264]
ENSG00000188730	VWC2	-1.334988765	2.091320826	2.71E-08	4.66E-07	von Willebrand factor C domain containing 2 [Source:HGNC Symbol;Acc:HGNC:30200]
ENSG00000221603	MIR1184-3	-1.334948867	-0.022304127	6.90E-07	9.12E-06	microRNA 1184-3 [Source:HGNC Symbol;Acc:HGNC:38389]
ENSG00000151917	BEND6	-1.334625841	2.04813517	4.95E-11	1.29E-09	BEN domain containing 6 [Source:HGNC Symbol;Acc:HGNC:20871]
ENSG00000107738	VSIR	-1.334551467	-0.569639804	2.55E-08	4.41E-07	V-set immunoregulatory receptor [Source:HGNC Symbol;Acc:HGNC:30085]
ENSG00000120738	EGR1	-1.334126226	3.072916456	1.53E-07	2.27E-06	early growth response 1 [Source:HGNC Symbol;Acc:HGNC:3238]
ENSG00000257228	None	-1.33389093	-0.803967156	7.99E-07	1.04E-05	novel transcript
ENSG00000264589	MAPT-AS1	-1.333250183	3.799485122	1.56E-06	1.92E-05	MAPT antisense RNA 1 [Source:HGNC Symbol;Acc:HGNC:43738]
ENSG00000225689	None	-1.332960693	-0.836688673	2.41E-06	2.86E-05	novel transcript
ENSG00000171450	CDK5R2	-1.332819055	4.005047415	0.000282528	0.001985998	cyclin dependent kinase 5 regulatory subunit 2 [Source:HGNC Symbol;Acc:HGNC:1776]
ENSG00000144339	TMEFF2	-1.332030389	4.354921234	1.11E-16	5.59E-15	transmembrane protein with EGF like and two follistatin like domains 2 [Source:HGNC Symbol;Acc:HGNC:11867]
ENSG00000169851	PCDH7	-1.331828903	5.283833466	8.98E-14	3.27E-12	protocadherin 7 [Source:HGNC Symbol;Acc:HGNC:8659]
ENSG00000255737	AGAP2-AS1	-1.330618136	4.11598456	2.35E-05	0.000222194	AGAP2 antisense RNA 1 [Source:HGNC Symbol;Acc:HGNC:48633]
ENSG00000189221	MAOA	-1.330261656	1.647606137	5.80E-07	7.81E-06	monoamine oxidase A [Source:HGNC Symbol;Acc:HGNC:6833]
ENSG00000232825	PLPPR5-AS1	-1.329200121	1.112770319	1.66E-06	2.03E-05	PLPPR5 antisense RNA 1 [Source:HGNC Symbol;Acc:HGNC:55720]
ENSG00000257069	KCNK4-TEX40	-1.32827653	0.120633782	2.48E-08	4.31E-07	KCNK4-TEX40 readthrough [Source:NCBI gene (formerly Entrezgene);Acc:106780802]
ENSG00000135439	AGAP2	-1.327646442	6.074976866	1.49E-05	0.000148538	ArfGAP with GTPase domain
ENSG00000250167	None	-1.326618074	-0.185483571	2.82E-05	0.000260166	novel transcript
ENSG00000159200	RCAN1	-1.326183758	4.558977755	8.96E-09	1.69E-07	regulator of calcineurin 1 [Source:HGNC Symbol;Acc:HGNC:3040]
ENSG00000198216	CACNA1E	-1.324643978	6.339894387	3.45E-07	4.82E-06	calcium voltage-gated channel subunit alpha1 E [Source:HGNC Symbol;Acc:HGNC:1392]
ENSG00000261008	LINC01572	-1.32382231	4.089919233	7.34E-13	2.44E-11	long intergenic non-protein coding RNA 1572 [Source:HGNC Symbol;Acc:HGNC:51385]
ENSG00000179148	ALOXE3	-1.323569517	0.055313654	3.28E-08	5.55E-07	arachidonate lipoxygenase 3 [Source:HGNC Symbol;Acc:HGNC:13743]
ENSG00000250722	SELENOP	-1.322785507	2.288182879	5.83E-06	6.37E-05	selenoprotein P [Source:HGNC Symbol;Acc:HGNC:10751]
ENSG00000079691	CARMIL1	-1.321189442	4.31079248	2.80E-17	1.49E-15	capping protein regulator and myosin 1 linker 1 [Source:HGNC Symbol;Acc:HGNC:21581]
ENSG00000143318	CASQ1	-1.320925152	0.544717089	2.08E-08	3.67E-07	calsequestrin 1 [Source:HGNC Symbol;Acc:HGNC:1512]
ENSG00000124191	TOX2	-1.320438649	2.967330875	2.41E-10	5.73E-09	TOX high mobility group box family member 2 [Source:HGNC Symbol;Acc:HGNC:16095]
ENSG00000260641	TSPAN5-DT	-1.320132229	0.959431677	7.30E-09	1.40E-07	TSPAN5 divergent transcript [Source:HGNC Symbol;Acc:HGNC:55389]

Fig4 ABCEF Upreg_genes

EMSEMBL_ID	Gene_name	logFC	logCPM	Pvalue	FDR	description
ENSG00000270641	TSIX	7.942454217	8.886513682	0.000450564	0.002970657	TSIX transcript
ENSG00000229807	XIST	7.897087221	9.809317043	0.000725221	0.004444005	X inactive specific transcript [Source:HGNC Symbol;Acc:HGNC:12810]
ENSG00000112238	PRDM13	6.714502833	5.296614785	7.11E-54	4.85E-51	PR/SET domain 13 [Source:HGNC Symbol;Acc:HGNC:13998]
ENSG00000215474	SKOR2	6.200191721	5.074692985	2.81E-13	9.75E-12	SKI family transcriptional corepressor 2 [Source:HGNC Symbol;Acc:HGNC:32695]
ENSG00000231827	None	6.142711493	5.130683479	9.90E-06	0.000102411	immunoglobulin superfamily
ENSG00000206538	VGLL3	6.018464328	5.844462912	6.78E-78	1.80E-74	vestigial like family member 3 [Source:HGNC Symbol;Acc:HGNC:24327]
ENSG00000137203	TFAP2A	5.435187526	6.495191016	1.29E-26	1.58E-24	transcription factor AP-2 alpha [Source:HGNC Symbol;Acc:HGNC:11742]
ENSG00000177468	OLIG3	5.374125365	3.125874781	3.21E-28	4.39E-26	oligodendrocyte transcription factor 3 [Source:HGNC Symbol;Acc:HGNC:18003]
ENSG00000207955	MIR219A2HG	5.356139053	3.341817742	7.54E-13	2.50E-11	MIR219A2 host gene [Source:HGNC Symbol;Acc:HGNC:55938]
ENSG00000229950	TFAP2A-AS1	5.344300252	4.701217136	8.88E-28	1.17E-25	TFAP2A antisense RNA 1 [Source:HGNC Symbol;Acc:HGNC:40579]
ENSG00000126259	KIRREL2	5.219846302	6.757664224	3.76E-128	9.98E-124	kirre like nephrin family adhesion molecule 2 [Source:HGNC Symbol;Acc:HGNC:18816]
ENSG00000185985	SLITRK2	5.153307893	4.435526766	1.17E-51	6.47E-49	SLIT and NTRK like family member 2 [Source:HGNC Symbol;Acc:HGNC:13449]
ENSG00000163081	CCDC140	5.121630563	3.75181406	1.74E-59	1.49E-56	CCDC140 long non-coding RNA [Source:HGNC Symbol;Acc:HGNC:26514]
ENSG00000135903	PAX3	5.006529138	7.228250409	1.35E-51	7.30E-49	paired box 3 [Source:HGNC Symbol;Acc:HGNC:8617]
ENSG00000166863	TAC3	4.8508369	3.29666652	2.95E-67	4.12E-64	tachykinin precursor 3 [Source:HGNC Symbol;Acc:HGNC:11521]
ENSG00000175785	PRIMA1	4.769958922	4.432152182	2.98E-20	2.09E-18	proline rich membrane anchor 1 [Source:HGNC Symbol;Acc:HGNC:18319]
ENSG00000169840	GSX1	4.43713826	2.125693963	1.62E-16	7.93E-15	GS homeobox 1 [Source:HGNC Symbol;Acc:HGNC:20374]
ENSG00000136960	ENPP2	4.338381658	6.451676463	8.06E-52	4.55E-49	ectonucleotide pyrophosphatase/phosphodiesterase 2 [Source:HGNC Symbol;Acc:HGNC:3357]
ENSG00000266941	None	4.224744416	2.30289534	3.47E-06	3.96E-05	novel transcript
ENSG00000165970	SLC6A5	4.165712273	2.229438245	1.13E-07	1.73E-06	solute carrier family 6 member 5 [Source:HGNC Symbol;Acc:HGNC:11051]
ENSG00000236516	KLF2P4	4.144900257	2.641129534	0.000171702	0.001289905	Kruppel like factor 2 pseudogene 4 [Source:HGNC Symbol;Acc:HGNC:49283]
ENSG00000116711	PLA2G4A	3.976140479	1.640980637	4.44E-21	3.32E-19	phospholipase A2 group IVA [Source:HGNC Symbol;Acc:HGNC:9035]
ENSG00000247809	NR2F2-AS1	3.929179581	5.653658426	6.66E-27	8.39E-25	NR2F2 antisense RNA 1 [Source:HGNC Symbol;Acc:HGNC:44222]
ENSG00000114654	EFCC1	3.917222795	5.176274671	1.67E-84	7.39E-81	EF-hand and coiled-coil domain containing 1 [Source:HGNC Symbol;Acc:HGNC:25692]
ENSG00000008196	TFAP2B	3.748992505	8.184134915	2.56E-64	2.87E-61	transcription factor AP-2 beta [Source:HGNC Symbol;Acc:HGNC:11743]
ENSG00000086205	FOLH1	3.677262469	2.490970723	0.001100612	0.006320075	folate hydrolase 1 [Source:HGNC Symbol;Acc:HGNC:3788]
ENSG00000099725	PRKY	3.654067385	2.944875775	0.000383423	0.002586401	protein kinase Y-linked (pseudogene) [Source:HGNC Symbol;Acc:HGNC:9444]
ENSG00000154134	ROBO3	3.592354004	8.112982851	7.18E-41	2.38E-38	roundabout guidance receptor 3 [Source:HGNC Symbol;Acc:HGNC:13433]
ENSG00000188779	SKOR1	3.574648557	5.551207981	1.80E-15	7.97E-14	SKI family transcriptional corepressor 1 [Source:HGNC Symbol;Acc:HGNC:21326]
ENSG00000075290	WNT8B	3.463969023	1.497933294	1.26E-12	4.01E-11	Wnt family member 8B [Source:HGNC Symbol;Acc:HGNC:12789]
ENSG00000260141	None	3.448530598	2.773632323	0.000339471	0.002328354	novel transcript
ENSG00000168505	GBX2	3.418440238	3.973926908	2.23E-53	1.44E-50	gastrulation brain homeobox 2 [Source:HGNC Symbol;Acc:HGNC:4186]
ENSG00000233611	GBX2-AS1	3.398883648	3.441018461	8.73E-45	3.51E-42	GBX2 and ASB18 antisense RNA 1 [Source:HGNC Symbol;Acc:HGNC:55714]
ENSG00000081479	LRP2	3.352767915	6.622674871	5.99E-18	3.42E-16	LDL receptor related protein 2 [Source:HGNC Symbol;Acc:HGNC:6694]
ENSG00000237819	CDK6-AS1	3.329493424	1.170019353	1.60E-30	2.62E-28	CDK6 antisense RNA 1 [Source:HGNC Symbol;Acc:HGNC:40141]
ENSG00000162493	PDPN	3.260187001	5.908040238	9.61E-44	3.60E-41	podoplanin [Source:HGNC Symbol;Acc:HGNC:29602]
ENSG00000161638	ITGA5	3.227709148	3.471516276	4.54E-12	1.34E-10	integrin subunit alpha 5 [Source:HGNC Symbol;Acc:HGNC:6141]
ENSG00000169218	RSPO1	3.223466645	2.730682727	1.23E-20	8.84E-19	R-spondin 1 [Source:HGNC Symbol;Acc:HGNC:21679]
ENSG00000250131	AGA-DT	3.211404254	3.841724766	5.31E-32	1.01E-29	AGA divergent transcript [Source:HGNC Symbol;Acc:HGNC:27730]
ENSG00000185739	SRL	3.172772989	0.965819297	2.43E-17	1.32E-15	sarcalumenin [Source:HGNC Symbol;Acc:HGNC:11295]
ENSG00000259410	None	3.153148119	3.320338956	1.20E-12	3.83E-11	novel transcript
ENSG00000101115	SALL4	3.132210372	4.450869064	1.74E-05	0.000170569	spalt like transcription factor 4 [Source:HGNC Symbol;Acc:HGNC:15924]
ENSG00000205213	LGR4	3.11950159	6.881682998	1.75E-24	1.83E-22	leucine rich repeat containing G protein-coupled receptor 4 [Source:HGNC Symbol;Acc:HGNC:13299]
ENSG00000213025	COX20P1	3.117344371	1.096518793	7.64E-22	6.23E-20	COX20 pseudogene 1 [Source:HGNC Symbol;Acc:HGNC:31435]
ENSG00000108001	EBF3	3.058959153	7.314052792	1.50E-08	2.72E-07	EBF transcription factor 3 [Source:HGNC Symbol;Acc:HGNC:19087]
ENSG00000151090	THRB	3.039436205	4.539601037	8.04E-16	3.70E-14	thyroid hormone receptor beta [Source:HGNC Symbol;Acc:HGNC:11799]
ENSG00000143842	SOX13	3.027276252	5.553110967	5.36E-18	3.08E-16	SRY-box transcription factor 13 [Source:HGNC Symbol;Acc:HGNC:11192]
ENSG00000231431	FAR2P4	3.016627058	5.715583629	0.000246735	0.001764745	fatty acyl-CoA reductase 2 pseudogene 4 [Source:HGNC Symbol;Acc:HGNC:49286]
ENSG00000119715	ESRRB	2.991510385	3.98627584	4.49E-05	0.000392389	estrogen related receptor beta [Source:HGNC Symbol;Acc:HGNC:3473]
ENSG00000143195	ILDR2	2.983156087	6.852972882	8.48E-20	5.71E-18	immunoglobulin like domain containing receptor 2 [Source:HGNC Symbol;Acc:HGNC:18131]
ENSG00000118523	CCN2	2.955301187	3.344766922	9.97E-12	2.84E-10	cellular communication network factor 2 [Source:HGNC Symbol;Acc:HGNC:2500]
ENSG00000182177	ASB18	2.927526978	1.093289289	2.04E-16	9.92E-15	ankyrin repeat and SOCS box containing 18 [Source:HGNC Symbol;Acc:HGNC:19770]
ENSG00000182983	ZNF662	2.92743014	0.561263787	1.81E-12	5.66E-11	zinc finger protein 662 [Source:HGNC Symbol;Acc:HGNC:31930]
ENSG00000132321	IQCA1	2.922855934	3.30813484	7.81E-23	6.92E-21	IQ motif containing with AAA domain 1 [Source:HGNC Symbol;Acc:HGNC:26195]
ENSG00000189184	PCDH18	2.887505258	6.427937028	3.03E-08	5.18E-07	protocadherin 18 [Source:HGNC Symbol;Acc:HGNC:14268]
ENSG00000272449	None	2.87979262	3.112050845	2.30E-14	8.88E-13	novel transcript
ENSG00000123307	NEUROD4	2.87445097	4.008417489	6.60E-40	2.06E-37	neuronal differentiation 4 [Source:HGNC Symbol;Acc:HGNC:13802]
ENSG00000137834	SMAD6	2.872218036	1.479429549	2.18E-19	1.40E-17	SMAD family member 6 [Source:HGNC Symbol;Acc:HGNC:6772]
ENSG00000130528	HRC	2.86787475	1.352537735	0.000308459	0.002144441	histidine rich calcium binding protein [Source:HGNC Symbol;Acc:HGNC:5178]
ENSG00000261308	FIGNL2	2.790970064	2.303728292	5.17E-09	1.02E-07	fidgetin like 2 [Source:HGNC Symbol;Acc:HGNC:13287]
ENSG00000241219	None	2.779931866	0.787696872	5.11E-08	8.31E-07	novel transcript
ENSG00000185551	NR2F2	2.763501602	6.621822696	4.22E-51	2.20E-48	nuclear receptor subfamily 2 group F member 2 [Source:HGNC Symbol;Acc:HGNC:7976]
ENSG00000156869	FRRS1	2.758034868	0.740923887	1.07E-09	2.34E-08	ferric chelate reductase 1 [Source:HGNC Symbol;Acc:HGNC:27622]
ENSG00000128567	PODXL	2.749766515	5.678384966	2.55E-42	8.81E-40	podocalyxin like [Source:HGNC Symbol;Acc:HGNC:9171]
ENSG00000068078	FGFR3	2.7450128	6.998871699	1.18E-10	2.94E-09	fibroblast growth factor receptor 3 [Source:HGNC Symbol;Acc:HGNC:3690]
ENSG00000010278	CD9	2.741063151	2.795962001	3.18E-16	1.51E-14	CD9 molecule [Source:HGNC Symbol;Acc:HGNC:1709]
ENSG00000150636	CCDC102B	2.733659899	4.409848746	4.29E-28	5.82E-26	coiled-coil domain containing 102B [Source:HGNC Symbol;Acc:HGNC:26295]
ENSG00000179673	RPRML	2.726936085	2.51763102	1.27E-18	1.02E-16	reprimo like [Source:HGNC Symbol;Acc:HGNC:32422]
ENSG00000259124	None	2.71677149	3.428223134	0.000169631	0.001275872	novel transcript
ENSG00000100033	PRODH	2.688687903	4.897560147	3.70E-22	3.12E-20	proline dehydrogenase 1 [Source:HGNC Symbol;Acc:HGNC:9453]
ENSG00000178538	CA8	2.65931322	3.910081803	1.85E-05	0.000179634	carbonic anhydrase 8 [Source:HGNC Symbol;Acc:HGNC:1382]
ENSG00000189398	OR7E12P	2.632885556	1.469928788	0.001359591	0.007566769	olfactory receptor family 7 subfamily E member 12 pseudogene [Source:HGNC Symbol;Acc:HGNC:8383]
ENSG00000259416	LINC02883	2.616598711	1.8471147	4.75E-14	1.77E-12	long intergenic non-protein coding RNA 2883 [Source:HGNC Symbol;Acc:HGNC:54806]
ENSG00000177551	NHLH2	2.614086542	4.493832394	1.53E-29	2.32E-27	nescient helix-loop-helix 2 [Source:HGNC Symbol;Acc:HGNC:7818]
ENSG00000174080	CTSF	2.607246813	3.250610175	6.02E-16	2.80E-14	cathepsin F [Source:HGNC Symbol;Acc:HGNC:2531]
ENSG00000125726	CD70	2.605896821	0.08538996	6.20E-18	3.53E-16	CD70 molecule [Source:HGNC Symbol;Acc:HGNC:11937]
ENSG00000266676	MIR4297	2.605580283	1.495607373	4.84E-07	6.60E-06	microRNA 4297 [Source:HGNC Symbol;Acc:HGNC:38393]
ENSG00000136160	EDNRB	2.604380956	6.86784648	1.79E-53	1.19E-50	endothelin receptor type B [Source:HGNC Symbol;Acc:HGNC:3180]
ENSG00000261586	None	2.589309018	2.898617253	3.16E-08	5.38E-07	novel transcript
ENSG00000145888	GLRA1	2.576655024	2.16256595	4.44E-12	1.32E-10	glycine receptor alpha 1 [Source:HGNC Symbol;Acc:HGNC:4326]
ENSG00000259711	None	2.574460066	1.975313665	6.09E-08	9.76E-07	novel transcript
ENSG00000254862	LGR4-AS1	2.561448229	3.888192192	4.59E-21	3.42E-19	LGR4 antisense RNA 1 [Source:HGNC Symbol;Acc:HGNC:40629]
ENSG00000086991	NOX4	2.547590356	1.646083607	1.46E-13	5.17E-12	NADPH oxidase 4 [Source:HGNC Symbol;Acc:HGNC:7891]
ENSG00000128510	CPA4	2.547583954	0.574285925	3.55E-12	1.07E-10	carboxypeptidase A4 [Source:HGNC Symbol;Acc:HGNC:15740]
ENSG00000259762	None	2.536690508	2.390165501	2.70E-15	1.17E-13	novel transcript
ENSG00000265751	None	2.53164111	4.800594745	1.21E-23	1.17E-21	novel transcript
ENSG00000166426	CRABP1	2.520322611	6.487984344	6.82E-32	1.26E-29	cellular retinoic acid binding protein 1 [Source:HGNC Symbol;Acc:HGNC:2338]
ENSG00000185483	ROR1	2.517307768	5.083076023	1.21E-45	5.19E-43	receptor tyrosine kinase like orphan receptor 1 [Source:HGNC Symbol;Acc:HGNC:10256]
ENSG00000129204	USP6	2.514082813	2.794544434	0.000393534	0.002647209	ubiquitin specific peptidase 6 [Source:HGNC Symbol;Acc:HGNC:12629]
ENSG00000182168	UNC5C	2.493104618	6.163880127	7.74E-48	3.43E-45	unc-5 netrin receptor C [Source:HGNC Symbol;Acc:HGNC:12569]
ENSG00000154188	ANGPT1	2.476781151	2.452590521	2.83E-05	0.000261201	angiopoietin 1 [Source:HGNC Symbol;Acc:HGNC:484]
ENSG00000149243	KLHL35	2.472530099	5.367831963	3.72E-45	1.52E-42	kelch like family member 35 [Source:HGNC Symbol;Acc:HGNC:26597]
ENSG00000141449	GREB1L	2.464668394	7.407546383	5.66E-23	5.08E-21	GREB1 like retinoic acid receptor coactivator [Source:HGNC Symbol;Acc:HGNC:31042]
ENSG00000171956	FOXB1	2.463983273	2.421229438	3.25E-10	7.64E-09	forkhead box B1 [Source:HGNC Symbol;Acc:HGNC:3799]
ENSG00000173376	NDNF	2.456818535	5.321283242	1.59E-18	9.48E-17	neuron derived neurotrophic factor [Source:HGNC Symbol;Acc:HGNC:26256]
ENSG00000120907	ADRA1A	2.447171542	5.579511749	1.20E-13	4.27E-12	adrenoceptor alpha 1A [Source:HGNC Symbol;Acc:HGNC:277]
ENSG00000144857	BOC	2.444847168	8.102213886	5.02E-13	1.70E-11	BOC cell adhesion associated
ENSG00000082556	OPRK1	2.441584755	2.313456852	2.15E-10	5.13E-09	opioid receptor kappa 1 [Source:HGNC Symbol;Acc:HGNC:8154]
ENSG00000188856	RPSAP47	2.435236994	3.716654339	0.000486895	0.003167741	ribosomal protein SA pseudogene 47 [Source:HGNC Symbol;Acc:HGNC:36162]
ENSG00000236544	EN2-DT	2.42481596	2.71729438	2.48E-26	2.93E-24	EN2 divergent transcript [Source:HGNC Symbol;Acc:HGNC:55659]
ENSG00000270605	None	2.416343125	2.053248442	4.25E-06	4.77E-05	novel transcript
ENSG00000139835	GRTP1	2.413182192	1.850832726	1.72E-10	4.20E-09	growth hormone regulated TBC protein 1 [Source:HGNC Symbol;Acc:HGNC:20310]
ENSG00000050628	PTGER3	2.407106732	3.740991133	0.000247445	0.001768871	prostaglandin E receptor 3 [Source:HGNC Symbol;Acc:HGNC:9595]
ENSG00000245694	CRNDE	2.400167941	7.320133791	1.44E-06	1.77E-05	colorectal neoplasia differentially expressed [Source:HGNC Symbol;Acc:HGNC:37078]
ENSG00000170927	PKHD1	2.398980621	1.793595767	1.10E-14	4.43E-13	PKHD1 ciliary IPT domain containing fibrocystin/polyductin [Source:HGNC Symbol;Acc:HGNC:9016]

ENSG00000234546	LNCTAM34A	2.395598316	1.609212699	3.88E-05	0.000344865	long non coding transcriptional activator of miR34a [Source:HGNC Symbol;Acc:HGNC:52548]
ENSG00000121966	CXCR4	2.394308904	6.607055254	1.82E-29	2.73E-27	C-X-C motif chemokine receptor 4 [Source:HGNC Symbol;Acc:HGNC:2561]
ENSG00000223949	ROR1-AS1	2.39323641	3.85754931	7.35E-28	9.82E-26	ROR1 antisense RNA 1 [Source:HGNC Symbol;Acc:HGNC:40508]
ENSG00000254687	None	2.392335836	2.076596967	2.17E-09	4.53E-08	novel transcript
ENSG00000141338	ABCA8	2.38968583	1.571701079	1.43E-12	4.52E-11	ATP binding cassette subfamily A member 8 [Source:HGNC Symbol;Acc:HGNC:38]
ENSG00000245812	LINC02202	2.386903852	1.619572386	2.51E-17	1.35E-15	long intergenic non-protein coding RNA 2202 [Source:HGNC Symbol;Acc:HGNC:53068]
ENSG00000138829	FBN2	2.385354714	5.33133343	2.60E-09	5.39E-08	fibrillin 2 [Source:HGNC Symbol;Acc:HGNC:3604]
ENSG00000122644	ARL4A	2.374875533	4.544542817	6.88E-11	1.76E-09	ADP ribosylation factor like GTPase 4A [Source:HGNC Symbol;Acc:HGNC:695]
ENSG00000109991	P2RX3	2.367218573	4.750603029	1.33E-15	5.98E-14	purinergic receptor P2X 3 [Source:HGNC Symbol;Acc:HGNC:8534]
ENSG00000241956	LINC03000	2.363481099	4.257494283	1.50E-34	3.42E-32	long intergenic non-protein coding RNA 3000 [Source:HGNC Symbol;Acc:HGNC:56116]
ENSG00000207971	MIR125B1	2.358340292	0.770573114	1.81E-06	2.19E-05	microRNA 125b-1 [Source:HGNC Symbol;Acc:HGNC:31506]
ENSG00000003137	CYP26B1	2.357621343	6.896202071	6.14E-36	1.53E-33	cytochrome P450 family 26 subfamily B member 1 [Source:HGNC Symbol;Acc:HGNC:20581]
ENSG00000154864	PIEZO2	2.352749659	5.073683965	4.71E-08	7.73E-07	piezo type mechanosensitive ion channel component 2 [Source:HGNC Symbol;Acc:HGNC:26270]
ENSG00000130513	GDF15	2.342859811	2.297739499	7.60E-14	2.78E-12	growth differentiation factor 15 [Source:HGNC Symbol;Acc:HGNC:30142]
ENSG00000271898	MIR4791	2.340540513	-0.027192419	1.06E-08	1.98E-07	microRNA 4791 [Source:HGNC Symbol;Acc:HGNC:41704]
ENSG00000255580	None	2.337043033	0.002783531	0.000116419	0.000911452	novel transcript
ENSG00000228526	MIR34AHG	2.336906741	2.11468435	2.07E-05	0.000199074	MIR34A host gene [Source:HGNC Symbol;Acc:HGNC:51913]
ENSG00000183960	KCNH8	2.335207965	6.646550373	1.06E-21	8.51E-20	potassium voltage-gated channel subfamily H member 8 [Source:HGNC Symbol;Acc:HGNC:18864]
ENSG00000271776	None	2.332579212	2.967656327	6.26E-22	5.15E-20	novel transcript
ENSG00000226864	ATE1OSP	2.332094405	1.304282931	2.85E-11	7.73E-10	ATE1 opposite strand
ENSG00000136732	GYPC	2.331357998	2.763927764	1.28E-15	5.74E-14	glycophorin C (Gerbich blood group) [Source:HGNC Symbol;Acc:HGNC:4704]
ENSG00000136099	PCDH8	2.318982266	6.28031913	8.95E-33	1.77E-30	protocadherin 8 [Source:HGNC Symbol;Acc:HGNC:8660]
ENSG00000250337	PURPL	2.306352416	4.436436939	2.08E-12	6.41E-11	p53 upregulated regulator of p53 levels [Source:HGNC Symbol;Acc:HGNC:48995]
ENSG00000187773	DIPK1C	2.305798042	3.753449831	1.74E-06	2.11E-05	divergent protein kinase domain 1C [Source:HGNC Symbol;Acc:HGNC:31729]
ENSG00000099251	HSD17B7P2	2.298294744	4.078210069	2.65E-05	0.000246482	hydroxysteroid 17-beta dehydrogenase 7 pseudogene 2 [Source:HGNC Symbol;Acc:HGNC:28120]
ENSG00000148053	NTRK2	2.297906479	8.6121305	1.70E-34	3.79E-32	neurotrophic receptor tyrosine kinase 2 [Source:HGNC Symbol;Acc:HGNC:8032]
ENSG00000142627	EPHA2	2.296876472	4.439056765	1.18E-06	1.48E-05	EPH receptor A2 [Source:HGNC Symbol;Acc:HGNC:3386]
ENSG00000111783	RFX4	2.294149405	6.789510262	5.44E-18	3.12E-16	regulatory factor X4 [Source:HGNC Symbol;Acc:HGNC:9985]
ENSG00000240057	NEPRO-AS1	2.285563952	4.669064921	8.11E-08	1.27E-06	NEPRO antisense RNA 1 [Source:HGNC Symbol;Acc:HGNC:41049]
ENSG00000244694	PTCHD4	2.283762944	4.028763519	3.65E-09	7.40E-08	patched domain containing 4 [Source:HGNC Symbol;Acc:HGNC:21345]
ENSG00000162944	RFTN2	2.273677387	5.445298427	4.03E-27	5.14E-25	raftlin family member 2 [Source:HGNC Symbol;Acc:HGNC:26402]
ENSG00000202058	RN7SKP80	2.269305689	0.49492719	3.39E-05	0.000305914	RN7SK pseudogene 80 [Source:HGNC Symbol;Acc:HGNC:45804]
ENSG00000272512	None	2.265298727	4.63199421	1.22E-25	1.40E-23	novel transcript
ENSG00000152192	POU4F1	2.260126886	5.297253582	2.48E-35	5.84E-33	POU class 4 homeobox 1 [Source:HGNC Symbol;Acc:HGNC:9218]
ENSG00000112562	SMOC2	2.258379196	3.203072829	5.18E-16	2.41E-14	SPARC related modular calcium binding 2 [Source:HGNC Symbol;Acc:HGNC:20323]
ENSG00000250344	NDNF-AS1	2.255012556	2.607979803	7.74E-13	2.56E-11	NDNF antisense RNA 1 [Source:HGNC Symbol;Acc:HGNC:55553]
ENSG00000175745	NR2F1	2.250570401	7.474851611	3.70E-32	7.11E-30	nuclear receptor subfamily 2 group F member 1 [Source:HGNC Symbol;Acc:HGNC:7975]
ENSG00000224842	LMX1B-DT	2.247855362	3.00679716	2.64E-07	3.76E-06	LMX1B divergent transcript [Source:HGNC Symbol;Acc:HGNC:55849]
ENSG00000148204	CRB2	2.244309468	6.977473087	1.11E-07	1.70E-06	crumbs cell polarity complex component 2 [Source:HGNC Symbol;Acc:HGNC:18688]
ENSG00000206120	EGFEM1P	2.239874995	5.243337184	1.03E-08	1.92E-07	EGF like and EMI domain containing 1
ENSG00000164330	EBF1	2.234355885	6.072523007	2.12E-08	3.73E-07	EBF transcription factor 1 [Source:HGNC Symbol;Acc:HGNC:3126]
ENSG00000174721	FGFBP3	2.211378995	8.27858341	8.85E-06	9.28E-05	fibroblast growth factor binding protein 3 [Source:HGNC Symbol;Acc:HGNC:23428]
ENSG00000148942	SLC5A12	2.210124545	0.967001098	0.001075235	0.006205204	solute carrier family 5 member 12 [Source:HGNC Symbol;Acc:HGNC:28750]
ENSG00000143858	SYT2	2.208124036	4.872540924	4.62E-37	1.25E-34	synaptotagmin 2 [Source:HGNC Symbol;Acc:HGNC:11510]
ENSG00000069431	ABCC9	2.189659929	0.083150676	9.68E-07	1.23E-05	ATP binding cassette subfamily C member 9 [Source:HGNC Symbol;Acc:HGNC:60]
ENSG00000248441	LETR1	2.179146047	0.285205651	1.12E-07	1.72E-06	lymphatic endothelial transcriptional regulator lncRNA 1 [Source:HGNC Symbol;Acc:HGNC:49597]
ENSG00000152822	GRM1	2.176657068	5.938873085	1.63E-21	1.27E-19	glutamate metabotropic receptor 1 [Source:HGNC Symbol;Acc:HGNC:4593]
ENSG00000272817	None	2.170034698	6.959844326	1.66E-05	0.000163422	novel transcript
ENSG00000145794	MEGF10	2.166644747	7.345396767	6.85E-12	1.98E-10	multiple EGF like domains 10 [Source:HGNC Symbol;Acc:HGNC:29634]
ENSG00000162761	LMX1A	2.156719172	0.821526708	0.001198808	0.006798704	LIM homeobox transcription factor 1 alpha [Source:HGNC Symbol;Acc:HGNC:6653]
ENSG00000164778	EN2	2.154204532	5.48241801	3.53E-21	2.68E-19	engrailed homeobox 2 [Source:HGNC Symbol;Acc:HGNC:3343]
ENSG00000161270	NPHS1	2.15005279	7.326184716	5.78E-31	9.95E-29	NPHS1 adhesion molecule
ENSG00000139292	LGR5	2.145885451	3.049323548	0.00039404	0.002649275	leucine rich repeat containing G protein-coupled receptor 5 [Source:HGNC Symbol;Acc:HGNC:4504]
ENSG00000234993	CUBNP2	2.139376506	0.414468962	2.55E-06	3.00E-05	cubilin pseudogene 2 [Source:HGNC Symbol;Acc:HGNC:44984]
ENSG00000163064	EN1	2.134190298	2.408867425	4.12E-14	1.54E-12	engrailed homeobox 1 [Source:HGNC Symbol;Acc:HGNC:3342]
ENSG00000117318	ID3	2.129267991	1.085971876	3.06E-05	0.000279109	inhibitor of DNA binding 3 [Source:HGNC Symbol;Acc:HGNC:5362]
ENSG00000141384	TAF4B	2.127052178	2.279971412	1.46E-07	2.18E-06	TATA-box binding protein associated factor 4b [Source:HGNC Symbol;Acc:HGNC:11538]
ENSG00000126803	HSPA2	2.120549339	0.322191358	1.99E-07	2.89E-06	heat shock protein family A (Hsp70) member 2 [Source:HGNC Symbol;Acc:HGNC:5235]
ENSG00000254463	PPIAP41	2.117826507	0.420604061	5.67E-06	6.21E-05	peptidylprolyl isomerase A pseudogene 41 [Source:HGNC Symbol;Acc:HGNC:53665]
ENSG00000224765	LINC02931	2.106698332	0.939770967	6.90E-12	2.00E-10	long intergenic non-protein coding RNA 2931 [Source:HGNC Symbol;Acc:HGNC:55853]
ENSG00000007062	PROM1	2.10636587	4.515883525	1.53E-05	0.000152032	prominin 1 [Source:HGNC Symbol;Acc:HGNC:9454]
ENSG00000264843	None	2.102971802	1.138032438	8.93E-06	9.34E-05	novel transcript
ENSG00000206129	None	2.102200808	-0.18894326	0.000642956	0.004007386	novel transcript
ENSG00000257711	None	2.09992838	1.794150549	1.70E-14	6.71E-13	novel transcript
ENSG00000069702	TGFBR3	2.09616959	2.002918759	1.07E-07	1.64E-06	transforming growth factor beta receptor 3 [Source:HGNC Symbol;Acc:HGNC:11774]
ENSG00000174498	IGDCC3	2.093365169	7.277948269	3.03E-06	3.50E-05	immunoglobulin superfamily DCC subclass member 3 [Source:HGNC Symbol;Acc:HGNC:9700]
ENSG00000203867	RBM20	2.09176893	1.425425961	1.26E-15	5.70E-14	RNA binding motif protein 20 [Source:HGNC Symbol;Acc:HGNC:27424]
ENSG00000164199	ADGRV1	2.091396516	7.0404068	1.49E-21	1.17E-19	adhesion G protein-coupled receptor V1 [Source:HGNC Symbol;Acc:HGNC:17416]
ENSG00000164438	TLX3	2.084198547	2.91832964	1.19E-08	2.20E-07	T cell leukemia homeobox 3 [Source:HGNC Symbol;Acc:HGNC:13532]
ENSG00000255753	None	2.082242566	0.949746292	1.94E-07	2.81E-06	WAS protein family homolog pseudogene
ENSG00000198719	DLL1	2.08092721	7.438820248	1.39E-17	7.76E-16	delta like canonical Notch ligand 1 [Source:HGNC Symbol;Acc:HGNC:2908]
ENSG00000227365	None	2.080724685	1.417374437	3.43E-10	8.05E-09	novel transcript
ENSG00000164694	FNDC1	2.075763853	-0.201563007	7.15E-06	7.64E-05	fibronectin type III domain containing 1 [Source:HGNC Symbol;Acc:HGNC:21184]
ENSG00000108379	WNT3	2.073694707	5.137781865	4.38E-34	9.23E-32	Wnt family member 3 [Source:HGNC Symbol;Acc:HGNC:12782]
ENSG00000257918	None	2.064189197	4.022035647	1.28E-16	6.38E-15	novel transcript
ENSG00000253693	None	2.053555706	2.697241128	2.38E-17	1.29E-15	novel transcript
ENSG00000246763	RGMB-AS1	2.052121032	4.905843713	5.62E-38	1.62E-35	RGMB antisense RNA 1 [Source:HGNC Symbol;Acc:HGNC:48666]
ENSG00000026103	FAS	2.045255307	1.973481329	3.91E-07	5.40E-06	Fas cell surface death receptor [Source:HGNC Symbol;Acc:HGNC:11920]
ENSG00000153993	SEMA3D	2.044282361	3.243976959	1.82E-17	1.01E-15	semaphorin 3D [Source:HGNC Symbol;Acc:HGNC:10726]
ENSG00000256546	LINC02985	2.041090508	0.392411582	7.90E-07	1.03E-05	long intergenic non-protein coding RNA 2985 [Source:HGNC Symbol;Acc:HGNC:56073]
ENSG00000224761	CCNYL4	2.034258261	1.153069339	4.00E-07	5.51E-06	cyclin Y like 4 (pseudogene) [Source:HGNC Symbol;Acc:HGNC:54655]
ENSG00000198729	PPP1R14C	2.032042846	4.516914512	2.35E-31	4.19E-29	protein phosphatase 1 regulatory inhibitor subunit 14C [Source:HGNC Symbol;Acc:HGNC:14952]
ENSG00000091879	ANGPT2	2.02821941	2.068802778	7.09E-11	1.81E-09	angiopoietin 2 [Source:HGNC Symbol;Acc:HGNC:485]
ENSG00000165309	ARMC3	2.027701268	1.451758644	3.63E-14	1.37E-12	armadillo repeat containing 3 [Source:HGNC Symbol;Acc:HGNC:30964]
ENSG00000176040	TMPRSS7	2.020718562	0.07232136	2.37E-08	4.14E-07	transmembrane serine protease 7 [Source:HGNC Symbol;Acc:HGNC:30846]
ENSG00000101198	NKAIN4	2.01678323	6.028058333	2.50E-17	1.35E-15	sodium/potassium transporting ATPase interacting 4 [Source:HGNC Symbol;Acc:HGNC:16191]
ENSG00000257545	None	2.014226362	6.914473324	6.98E-17	3.59E-15	novel transcript
ENSG00000163331	DAPL1	2.014139562	2.850357085	6.82E-10	1.54E-08	death associated protein like 1 [Source:HGNC Symbol;Acc:HGNC:21490]
ENSG00000064666	CNN2	2.010486193	4.646064327	1.38E-25	1.58E-23	calponin 2 [Source:HGNC Symbol;Acc:HGNC:2156]
ENSG00000141540	TTYH2	2.001537654	5.149488936	7.39E-20	5.00E-18	tweety family member 2 [Source:HGNC Symbol;Acc:HGNC:13877]
ENSG00000140519	RHCG	1.997776023	5.991477497	1.14E-08	2.12E-07	Rh family C glycoprotein [Source:HGNC Symbol;Acc:HGNC:18140]
ENSG00000237187	NR2F1-AS1	1.996875152	6.915319185	3.21E-35	7.48E-33	NR2F1 antisense RNA 1 [Source:HGNC Symbol;Acc:HGNC:48622]
ENSG00000135048	CEMIP2	1.993411381	6.716294679	1.49E-05	0.000148394	cell migration inducing hyaluronidase 2 [Source:HGNC Symbol;Acc:HGNC:11869]
ENSG00000114251	WNT5A	1.989994101	4.199558761	5.88E-22	4.87E-20	Wnt family member 5A [Source:HGNC Symbol;Acc:HGNC:12784]
ENSG00000130940	CASZ1	1.988426845	5.137468695	6.68E-24	6.62E-22	castor zinc finger 1 [Source:HGNC Symbol;Acc:HGNC:26002]
ENSG00000130958	SLC35D2	1.987330178	1.879520349	4.90E-05	0.000424303	solute carrier family 35 member D2 [Source:HGNC Symbol;Acc:HGNC:20799]
ENSG00000254066	LINC01938	1.981363031	0.610385867	1.12E-11	3.17E-10	long intergenic non-protein coding RNA 1938 [Source:HGNC Symbol;Acc:HGNC:52761]
ENSG00000125246	CLYBL	1.975558325	4.287493137	1.54E-15	6.89E-14	citramalyl-CoA lyase [Source:HGNC Symbol;Acc:HGNC:18355]
ENSG00000272078	None	1.962493635	3.788703773	1.35E-18	8.12E-17	novel transcript
ENSG00000116729	WLS	1.961529932	6.676822831	1.37E-37	3.83E-35	Wnt ligand secretion mediator [Source:HGNC Symbol;Acc:HGNC:30238]
ENSG00000063127	SLC6A16	1.96081714	4.064639322	4.57E-11	1.20E-09	solute carrier family 6 member 16 [Source:HGNC Symbol;Acc:HGNC:13622]
ENSG00000257242	LINC01619	1.950375177	3.767407756	2.79E-23	2.60E-21	long intergenic non-protein coding RNA 1619 [Source:HGNC Symbol;Acc:HGNC:27409]
ENSG00000117707	PROX1	1.950137728	3.603726646	1.68E-14	6.64E-13	prospero homeobox 1 [Source:HGNC Symbol;Acc:HGNC:9459]
ENSG00000146910	CNPY1	1.944213762	5.57193757	1.18E-28	1.69E-26	canopy FGF signaling regulator 1 [Source:HGNC Symbol;Acc:HGNC:27786]
ENSG00000112893	MAN2A1	1.943001872	5.770255992	4.89E-14	1.82E-12	mannosidase alpha class 2A member 1 [Source:HGNC Symbol;Acc:HGNC:6824]
ENSG00000257458	None	1.941780292	2.720173665	1.86E-15	8.19E-14	novel transcript
ENSG00000070882	OSBPL3	1.941330075	3.612730258	5.22E-10	1.20E-08	oxysterol binding protein like 3 [Source:HGNC Symbol;Acc:HGNC:16370]

ENSG00000260473	None	1.940939053	1.168718712	1.66E-08	2.98E-07 novel transcript
ENSG00000124942	AHNAK	1.938182437	4.946031709	1.14E-22	9.98E-21 AHNAK nucleoprotein [Source:HGNC Symbol;Acc:HGNC:347]
ENSG00000230267	HERC2P4	1.931666526	3.714862	0.000380513	0.002572653 HERC2 pseudogene 4 [Source:HGNC Symbol;Acc:HGNC:4872]
ENSG00000263551	None	1.919627049	1.503139699	0.001802147	0.009607109 novel transcript
ENSG00000257986	LINC02306	1.915175642	0.987107356	5.44E-06	5.97E-05 long intergenic non-protein coding RNA 2306 [Source:HGNC Symbol;Acc:HGNC:53225]
ENSG00000240747	KRBOX1	1.914710055	1.381905513	8.20E-13	2.70E-11 KRAB box domain containing 1 [Source:HGNC Symbol;Acc:HGNC:38708]
ENSG00000249846	LINC02021	1.909347531	0.30959167	3.99E-08	6.65E-07 long intergenic non-protein coding RNA 2021 [Source:HGNC Symbol;Acc:HGNC:52856]
ENSG00000229715	EEF1DP3	1.909306508	1.120639025	2.54E-11	6.93E-10 eukaryotic translation elongation factor 1 delta pseudogene 3 [Source:HGNC Symbol;Acc:HGNC:30486]
ENSG00000108375	RNF43	1.905448461	2.855151852	2.88E-16	1.38E-14 ring finger protein 43 [Source:HGNC Symbol;Acc:HGNC:18505]
ENSG00000197921	HES5	1.904594892	6.34156856	1.15E-24	1.21E-22 hes family bHLH transcription factor 5 [Source:HGNC Symbol;Acc:HGNC:19764]
ENSG00000101384	JAG1	1.901182265	6.499263133	1.05E-08	1.96E-07 jagged canonical Notch ligand 1 [Source:HGNC Symbol;Acc:HGNC:6188]
ENSG00000166831	RBPMS2	1.898571585	2.310795446	1.60E-05	0.000158325 RNA binding protein
ENSG00000165462	PHOX2A	1.89776707	-0.008797882	3.27E-11	8.82E-10 paired like homeobox 2A [Source:HGNC Symbol;Acc:HGNC:691]
ENSG00000272573	MUSTN1	1.893044862	1.800105178	4.02E-06	4.53E-05 musculoskeletal
ENSG00000228956	SATB1-AS1	1.892014002	3.100991835	1.25E-11	3.52E-10 SATB1 antisense RNA 1 [Source:HGNC Symbol;Acc:HGNC:50687]
ENSG00000095596	CYP26A1	1.890862769	4.896096929	6.93E-21	5.07E-19 cytochrome P450 family 26 subfamily A member 1 [Source:HGNC Symbol;Acc:HGNC:2603]
ENSG00000230922	None	1.890488592	1.437155474	2.36E-05	0.000222333 novel transcript
ENSG00000197813	None	1.878095667	4.862916345	7.07E-10	1.59E-08 novel transcript
ENSG00000088992	TESC	1.871907544	0.304364096	2.19E-05	0.000208588 tescalcin [Source:HGNC Symbol;Acc:HGNC:26065]
ENSG00000231007	CDC20P1	1.863137072	0.098179791	1.24E-06	1.55E-05 cell division cycle 20 pseudogene 1 [Source:HGNC Symbol;Acc:HGNC:29487]
ENSG00000156925	ZIC3	1.861003582	7.142112781	1.43E-25	1.63E-23 Zic family member 3 [Source:HGNC Symbol;Acc:HGNC:12874]
ENSG00000188219	POTEE	1.857651881	4.309951722	5.62E-05	0.000478353 POTE ankyrin domain family member E [Source:HGNC Symbol;Acc:HGNC:33895]
ENSG00000271474	UNC5C-AS1	1.854931223	2.429421536	1.02E-08	1.91E-07 UNC5C antisense RNA 1 [Source:HGNC Symbol;Acc:HGNC:55246]
ENSG00000198435	NRARP	1.853423969	5.555533244	4.32E-13	1.47E-11 NOTCH regulated ankyrin repeat protein [Source:HGNC Symbol;Acc:HGNC:33843]
ENSG00000140511	HAPLN3	1.852639015	0.918555578	3.32E-06	3.81E-05 hyaluronan and proteoglycan link protein 3 [Source:HGNC Symbol;Acc:HGNC:21446]
ENSG00000121005	CRISPLD1	1.852289696	5.828182347	7.60E-38	2.17E-35 cysteine rich secretory protein LCCL domain containing 1 [Source:HGNC Symbol;Acc:HGNC:18206]
ENSG00000176771	NCKAP5	1.843544253	6.343597988	6.31E-20	4.32E-18 NCK associated protein 5 [Source:HGNC Symbol;Acc:HGNC:29847]
ENSG00000231738	TSPAN19	1.842135878	1.606585118	5.97E-08	9.61E-07 tetraspanin 19 [Source:HGNC Symbol;Acc:HGNC:31886]
ENSG00000248243	LINC02014	1.837809441	-0.416128606	6.55E-08	1.05E-06 long intergenic non-protein coding RNA 2014 [Source:HGNC Symbol;Acc:HGNC:52849]
ENSG00000188396	DYNLT4	1.836889289	-0.436821757	1.84E-06	2.23E-05 dynein light chain Tctex-type 4 [Source:HGNC Symbol;Acc:HGNC:32315]
ENSG00000185015	CA13	1.834432865	1.242756334	1.49E-09	3.19E-08 carbonic anhydrase 13 [Source:HGNC Symbol;Acc:HGNC:14914]
ENSG00000141314	RHBDL3	1.830937168	5.549772777	4.08E-08	6.78E-07 rhomboid like 3 [Source:HGNC Symbol;Acc:HGNC:16502]
ENSG00000163132	MSX1	1.825458799	2.002523417	2.02E-10	4.87E-09 msh homeobox 1 [Source:HGNC Symbol;Acc:HGNC:7391]
ENSG00000229618	None	1.823752868	0.873401454	5.31E-09	1.04E-07 novel transcript
ENSG00000262223	LINC03048	1.823531612	3.029622173	2.66E-09	5.49E-08 long intergenic non-protein coding RNA 3048 [Source:HGNC Symbol;Acc:HGNC:56272]
ENSG00000253372	None	1.819510661	-0.003683479	5.03E-10	1.16E-08 novel transcript
ENSG00000230461	PROX1-AS1	1.819279436	0.313439765	9.47E-10	2.08E-08 PROX1 antisense RNA 1 [Source:HGNC Symbol;Acc:HGNC:43656]
ENSG00000056558	TRAF1	1.812810668	2.367529456	2.67E-05	0.000248342 TNF receptor associated factor 1 [Source:HGNC Symbol;Acc:HGNC:12031]
ENSG00000255723	None	1.811417991	2.366853421	1.71E-07	2.50E-06 novel transcript
ENSG00000143194	MAEL	1.810504587	-0.244035917	2.97E-09	6.08E-08 maelstrom spermatogenic transposon silencer [Source:HGNC Symbol;Acc:HGNC:25929]
ENSG00000234383	CTBP2P8	1.81023053	1.546468041	6.32E-13	2.12E-11 CTBP2 pseudogene 8 [Source:HGNC Symbol;Acc:HGNC:45200]
ENSG00000236559	BCL2L1-AS1	1.809681104	1.351986532	2.64E-07	3.76E-06 BCL2L1 antisense RNA 1 [Source:HGNC Symbol;Acc:HGNC:40095]
ENSG00000225511	LINC00475	1.808788382	-0.353042375	0.000506733	0.003276741 long intergenic non-protein coding RNA 475 [Source:HGNC Symbol;Acc:HGNC:23569]
ENSG00000223561	LINC03007	1.808011473	0.786271771	1.82E-11	5.04E-10 long intergenic non-protein coding RNA 3007 [Source:HGNC Symbol;Acc:HGNC:56132]
ENSG00000111218	PRMT8	1.807449954	6.241802962	1.23E-34	2.81E-32 protein arginine methyltransferase 8 [Source:HGNC Symbol;Acc:HGNC:5188]
ENSG00000101463	SYNDIG1	1.802649605	4.548779183	1.98E-21	1.53E-19 synapse differentiation inducing 1 [Source:HGNC Symbol;Acc:HGNC:15885]
ENSG00000114204	SERPINI2	1.801137836	2.148662395	1.53E-11	4.29E-10 serpin family I member 2 [Source:HGNC Symbol;Acc:HGNC:8945]
ENSG00000127324	TSPAN8	1.80081324	0.10218559	0.000498364	0.003229701 tetraspanin 8 [Source:HGNC Symbol;Acc:HGNC:11855]
ENSG00000084453	SLCO1A2	1.799716097	2.015421334	1.20E-06	1.50E-05 solute carrier organic anion transporter family member 1A2 [Source:HGNC Symbol;Acc:HGNC:10956]
ENSG00000228252	COL6A4P2	1.798186155	1.639534929	4.56E-05	0.000397177 collagen type VI alpha 4 pseudogene 2 [Source:HGNC Symbol;Acc:HGNC:38501]
ENSG00000227959	EPHA2-AS1	1.796243816	0.833996328	0.000414244	0.002764816 EPHA2 antisense RNA 1 [Source:HGNC Symbol;Acc:HGNC:40216]
ENSG00000237732	CT75	1.795466985	4.435006633	4.26E-23	3.87E-21 cancer/testis associated transcript 75 [Source:HGNC Symbol;Acc:HGNC:37174]
ENSG00000138696	BMPR1B	1.794159015	4.54659976	3.85E-11	1.03E-09 bone morphogenetic protein receptor type 1B [Source:HGNC Symbol;Acc:HGNC:1077]
ENSG00000089116	LHX5	1.792922961	6.150185978	3.36E-08	5.68E-07 LIM homeobox 5 [Source:HGNC Symbol;Acc:HGNC:14216]
ENSG00000150361	KLHL1	1.791153997	5.4816771	6.57E-19	4.02E-17 kelch like family member 1 [Source:HGNC Symbol;Acc:HGNC:6352]
ENSG00000137801	THBS1	1.791023696	2.474124352	5.15E-12	1.51E-10 thrombospondin 1 [Source:HGNC Symbol;Acc:HGNC:11785]
ENSG00000176842	IRX5	1.783472817	5.089501564	2.13E-07	3.07E-06 iroquois homeobox 5 [Source:HGNC Symbol;Acc:HGNC:14361]
ENSG00000227533	SLC2A1-DT	1.773998776	2.929309328	2.89E-05	0.00026593 SLC2A1 divergent transcript [Source:HGNC Symbol;Acc:HGNC:44187]
ENSG00000158352	SHROOM4	1.765957948	3.082971637	1.50E-09	3.21E-08 shroom family member 4 [Source:HGNC Symbol;Acc:HGNC:29215]
ENSG00000182871	COL18A1	1.763597207	5.508807853	2.93E-05	0.000269363 collagen type XVIII alpha 1 chain [Source:HGNC Symbol;Acc:HGNC:2195]
ENSG00000205592	MUC19	1.760070934	1.01813163	8.80E-10	1.95E-08 mucin 19
ENSG00000233237	LINC00472	1.758758467	4.58843791	1.01E-16	5.12E-15 long intergenic non-protein coding RNA 472 [Source:HGNC Symbol;Acc:HGNC:21380]
ENSG00000254746	None	1.758752699	0.929746434	6.61E-07	8.78E-06 novel transcript
ENSG00000049323	LTBP1	1.757500368	6.108839611	5.63E-05	0.000478903 latent transforming growth factor beta binding protein 1 [Source:HGNC Symbol;Acc:HGNC:6714]
ENSG00000169860	P2RY1	1.754587268	2.100523857	1.04E-13	3.75E-12 purinergic receptor P2Y1 [Source:HGNC Symbol;Acc:HGNC:8539]
ENSG00000130147	SH3BP4	1.754097871	6.427237941	4.43E-15	1.85E-13 SH3 domain binding protein 4 [Source:HGNC Symbol;Acc:HGNC:10826]
ENSG00000143341	HMCN1	1.747849094	2.09842438	7.55E-12	2.17E-10 hemicentin 1 [Source:HGNC Symbol;Acc:HGNC:19194]
ENSG00000236595	MED15P5	1.744459129	0.817897588	3.72E-05	0.000332717 mediator complex subunit 15 pseudogene 5 [Source:HGNC Symbol;Acc:HGNC:48654]
ENSG00000264272	None	1.741953038	3.647652412	2.15E-14	8.33E-13 novel transcript
ENSG00000162496	DHRS3	1.733563262	3.893070351	4.17E-21	3.13E-19 dehydrogenase/reductase 3 [Source:HGNC Symbol;Acc:HGNC:17693]
ENSG00000267313	KC6	1.727877477	5.469232908	6.46E-07	8.59E-06 keratoconus gene 6 [Source:NCBI gene (formerly Entrezgene);Acc:641516]
ENSG00000254238	None	1.727644315	1.810567476	1.20E-12	3.83E-11 novel transcript
ENSG00000185499	MUC1	1.724633526	3.445778339	2.02E-14	7.85E-13 mucin 1
ENSG00000234398	None	1.722343788	0.818052901	4.90E-05	0.000423888 pseudogene similar to part of ATPase
ENSG00000258039	None	1.719953979	0.354851996	0.000278647	0.001964952 novel transcript
ENSG00000158715	SLC45A3	1.716782289	3.193127446	3.60E-06	4.09E-05 solute carrier family 45 member 3 [Source:HGNC Symbol;Acc:HGNC:8642]
ENSG00000272168	CASC15	1.716191134	6.499540459	1.25E-23	1.20E-21 cancer susceptibility 15 [Source:HGNC Symbol;Acc:HGNC:28245]
ENSG00000272843	None	1.714597035	0.016182373	0.000177697	0.001331172 novel transcript
ENSG00000114423	CBLB	1.711377691	6.976682505	5.06E-24	5.07E-22 Cbl proto-oncogene B [Source:HGNC Symbol;Acc:HGNC:1542]
ENSG00000198221	AFDN-DT	1.711371919	6.047664974	3.45E-10	8.09E-09 AFDN divergent transcript [Source:HGNC Symbol;Acc:HGNC:21236]
ENSG00000007866	TEAD3	1.708818742	4.828828388	1.48E-09	3.17E-08 TEA domain transcription factor 3 [Source:HGNC Symbol;Acc:HGNC:11716]
ENSG00000225783	MIAT	1.702554164	11.33004439	1.90E-23	1.80E-21 myocardial infarction associated transcript [Source:HGNC Symbol;Acc:HGNC:33425]
ENSG00000206028	None	1.702336617	9.986677121	1.02E-23	1.00E-21 novel transcript
ENSG00000007384	RHBDF1	1.701277617	2.875798721	1.59E-06	1.95E-05 rhomboid 5 homolog 1 [Source:HGNC Symbol;Acc:HGNC:20561]
ENSG00000158859	ADAMTS4	1.700636721	1.435544387	3.51E-11	9.41E-10 ADAM metallopeptidase with thrombospondin type 1 motif 4 [Source:HGNC Symbol;Acc:HGNC:220]
ENSG00000248136	MTND6P8	1.700538878	0.780854256	0.000320203	0.00221359 MT-ND6 pseudogene 8 [Source:HGNC Symbol;Acc:HGNC:39471]
ENSG00000234934	MTND5P29	1.700538871	0.780854256	0.000320254	0.00221359 MT-ND5 pseudogene 29 [Source:HGNC Symbol;Acc:HGNC:42291]
ENSG00000233550	MTCYBP8	1.70053885	0.780854256	0.000320396	0.00221359 MT-CYB pseudogene 8 [Source:HGNC Symbol;Acc:HGNC:51965]
ENSG00000224087	POTEF-AS1	1.700246859	1.185496328	0.000468243	0.003063646 POTEF antisense RNA 1 [Source:HGNC Symbol;Acc:HGNC:41294]
ENSG00000123360	PDE1B	1.699031394	5.253586243	1.89E-06	2.28E-05 phosphodiesterase 1B [Source:HGNC Symbol;Acc:HGNC:8775]
ENSG00000170549	IRX1	1.69707803	5.74115405	2.50E-25	2.81E-23 iroquois homeobox 1 [Source:HGNC Symbol;Acc:HGNC:14358]
ENSG00000154065	ANKRD29	1.694427396	3.218281491	2.23E-15	9.73E-14 ankyrin repeat domain 29 [Source:HGNC Symbol;Acc:HGNC:27110]
ENSG00000215156	None	1.694382993	2.967177436	1.40E-05	0.000140425 POM121-like protein 1 pseudogene
ENSG00000233729	NCKAP5-AS1	1.694002726	1.008349924	1.80E-09	3.82E-08 NCKAP5 antisense RNA 1 [Source:HGNC Symbol;Acc:HGNC:41246]
ENSG00000134853	PDGFRA	1.689646802	3.197944595	3.79E-09	7.64E-08 platelet derived growth factor receptor alpha [Source:HGNC Symbol;Acc:HGNC:8803]
ENSG00000224881	None	1.688103832	0.467460856	2.09E-10	5.02E-09 novel pseudogene
ENSG00000259603	None	1.685583092	5.175981962	2.37E-24	2.45E-22 novel transcript
ENSG00000137441	FGFBP2	1.682561941	1.81550999	0.000182128	0.001362066 fibroblast growth factor binding protein 2 [Source:HGNC Symbol;Acc:HGNC:29451]
ENSG00000263958	NETO1-DT	1.681865571	2.540683445	0.00145355	0.007984306 NETO1 divergent transcript [Source:HGNC Symbol;Acc:HGNC:55333]
ENSG00000234618	RPSAP9	1.681474862	2.922093809	0.00117474	0.006686193 ribosomal protein SA pseudogene 9 [Source:HGNC Symbol;Acc:HGNC:31463]
ENSG00000244625	MIATNB	1.679324529	10.20021104	9.69E-23	8.55E-21 MIAT neighbor [Source:HGNC Symbol;Acc:HGNC:50731]
ENSG00000259555	None	1.679158001	-0.237454778	1.81E-10	4.39E-09 novel transcript
ENSG00000196187	TMEM63A	1.675811868	3.477255871	2.26E-05	0.000214609 transmembrane protein 63A [Source:HGNC Symbol;Acc:HGNC:29118]
ENSG00000227172	YAE1-DT	1.674619519	-0.724531418	7.67E-05	0.000630789 YAE1 divergent transcript [Source:HGNC Symbol;Acc:HGNC:55820]
ENSG00000083937	CHMP2B	1.673862369	5.776436599	6.50E-06	7.03E-05 charged multivesicular body protein 2B [Source:HGNC Symbol;Acc:HGNC:24537]
ENSG00000249395	CASC9	1.671191474	2.140610522	2.93E-12	8.92E-11 cancer susceptibility 9 [Source:HGNC Symbol;Acc:HGNC:48906]
ENSG00000272009	None	1.670200947	-0.113940006	7.11E-06	7.60E-05 novel transcript

ENSG00000129474	AJUBA	1.669951309	4.405336068	5.65E-07	7.62E-06	ajuba LIM protein [Source:HGNC Symbol;Acc:HGNC:20250]
ENSG00000226915	RPL19P4	1.66836934	0.789532023	0.000402748	0.002699663	ribosomal protein L19 pseudogene 4 [Source:HGNC Symbol;Acc:HGNC:36539]
ENSG00000106003	LFNG	1.666392968	5.639608184	6.91E-08	1.10E-06	LFNG O-fucosylpeptide 3-beta-N-acetylglucosaminyltransferase [Source:HGNC Symbol;Acc:HGNC:6560]
ENSG00000018625	ATP1A2	1.665869538	6.052974265	4.90E-05	0.00042433	ATPase Na+/K+ transporting subunit alpha 2 [Source:HGNC Symbol;Acc:HGNC:800]
ENSG00000221340	RNU6ATAC18P	1.662359117	-0.610886796	2.64E-09	5.46E-08	RNA
ENSG00000196932	TMEM26	1.661489397	-0.053554424	6.64E-10	1.51E-08	transmembrane protein 26 [Source:HGNC Symbol;Acc:HGNC:28550]
ENSG00000178031	ADAMTSL1	1.660221804	3.192422175	1.19E-09	2.58E-08	ADAMTS like 1 [Source:HGNC Symbol;Acc:HGNC:14632]
ENSG00000106025	TSPAN12	1.655454696	3.030912883	4.67E-12	1.38E-10	tetraspanin 12 [Source:HGNC Symbol;Acc:HGNC:21641]
ENSG00000143028	SYPL2	1.654291726	-0.661323767	3.25E-07	4.54E-06	synaptophysin like 2 [Source:HGNC Symbol;Acc:HGNC:27638]
ENSG00000198400	NTRK1	1.649825637	1.613764601	0.001396781	0.007731627	neurotrophic receptor tyrosine kinase 1 [Source:HGNC Symbol;Acc:HGNC:8031]
ENSG00000254900	None	1.648831379	-0.717095205	6.90E-06	7.41E-05	novel transcript
ENSG00000257097	CLIP1-AS1	1.646052009	1.222271099	6.78E-10	1.54E-08	CLIP1 antisense RNA 1 [Source:HGNC Symbol;Acc:HGNC:48586]
ENSG00000188290	HES4	1.644365678	7.198773985	1.26E-14	5.04E-13	hes family bHLH transcription factor 4 [Source:HGNC Symbol;Acc:HGNC:24149]
ENSG00000265194	None	1.642884307	2.431537865	8.78E-05	0.000710574	novel transcript
ENSG00000133246	PRAM1	1.641195002	1.692642374	4.46E-07	6.12E-06	PML-RARA regulated adaptor molecule 1 [Source:HGNC Symbol;Acc:HGNC:30091]
ENSG00000259349	APPBP2-DT	1.635733449	0.907139583	2.31E-06	2.74E-05	APPBP2 divergent transcript [Source:HGNC Symbol;Acc:HGNC:55423]
ENSG00000229797	None	1.635311155	0.883145081	0.001461761	0.008021113	novel transcript
ENSG00000170430	MGMT	1.633221451	2.692245588	1.39E-12	4.41E-11	O-6-methylguanine-DNA methyltransferase [Source:HGNC Symbol;Acc:HGNC:7059]
ENSG00000249231	CASC16	1.631417279	0.150001236	0.000174544	0.001309403	cancer susceptibility 16 [Source:HGNC Symbol;Acc:HGNC:48608]
ENSG00000226113	None	1.628671717	0.268604034	2.48E-05	0.00023294	phospholipase D family member 5 (PLD5) pseudogene
ENSG00000258410	None	1.622467283	2.27029639	1.88E-10	4.54E-09	novel transcript
ENSG00000143333	RGS16	1.620002451	4.775016538	8.82E-13	2.88E-11	regulator of G protein signaling 16 [Source:HGNC Symbol;Acc:HGNC:9997]
ENSG00000139679	LPAR6	1.619993398	2.35273766	0.000199859	0.001472028	lysophosphatidic acid receptor 6 [Source:HGNC Symbol;Acc:HGNC:15520]
ENSG00000157978	LDLRAP1	1.61642929	2.677779556	2.80E-05	0.000259034	low density lipoprotein receptor adaptor protein 1 [Source:HGNC Symbol;Acc:HGNC:18640]
ENSG00000237722	MTND1P29	1.614545187	0.79387159	0.000609897	0.003843401	MT-ND1 pseudogene 29 [Source:HGNC Symbol;Acc:HGNC:42078]
ENSG00000264357	MIR4648	1.613260722	1.405307801	2.82E-05	0.000260166	microRNA 4648 [Source:HGNC Symbol;Acc:HGNC:41560]
ENSG00000253554	LINC01414	1.61317227	5.019143321	8.14E-07	1.05E-05	long intergenic non-protein coding RNA 1414 [Source:HGNC Symbol;Acc:HGNC:50707]
ENSG00000140545	MFGE8	1.612056864	5.512211403	1.54E-08	2.78E-07	milk fat globule EGF and factor V/VIII domain containing [Source:HGNC Symbol;Acc:HGNC:7036]
ENSG00000198205	ZXDA	1.61196469	2.351824977	2.01E-10	4.84E-09	zinc finger X-linked duplicated A [Source:HGNC Symbol;Acc:HGNC:13198]
ENSG00000231954	MTND2P22	1.611648722	0.791418498	0.00061084	0.003845741	MT-ND2 pseudogene 22 [Source:HGNC Symbol;Acc:HGNC:42123]
ENSG00000236211	MTCO1P7	1.611648719	0.791418498	0.000610745	0.003845741	MT-CO1 pseudogene 7 [Source:HGNC Symbol;Acc:HGNC:52009]
ENSG00000157322	CLEC18A	1.60874671	2.124237969	0.00113439	0.006483205	C-type lectin domain family 18 member A [Source:HGNC Symbol;Acc:HGNC:30388]
ENSG00000144476	ACKR3	1.608545234	4.673372228	1.00E-05	0.000103551	atypical chemokine receptor 3 [Source:HGNC Symbol;Acc:HGNC:23692]
ENSG00000241313	WWTR1-AS1	1.607718621	1.560135735	3.22E-11	8.71E-10	WWTR1 antisense RNA 1 [Source:HGNC Symbol;Acc:HGNC:41035]
ENSG00000230496	MTCO2P7	1.606952876	0.796240546	0.000615048	0.003866362	MT-CO2 pseudogene 7 [Source:HGNC Symbol;Acc:HGNC:52023]
ENSG00000118298	CA14	1.606088942	4.088121715	1.22E-07	1.84E-06	carbonic anhydrase 14 [Source:HGNC Symbol;Acc:HGNC:1372]
ENSG00000232990	MTATP6P7	1.60420053	0.793897861	0.000633507	0.003954754	MT-ATP6 pseudogene 7 [Source:HGNC Symbol;Acc:HGNC:44581]
ENSG00000249997	MTCO3P7	1.604200529	0.793897861	0.000633483	0.003954754	MT-CO3 pseudogene 7 [Source:HGNC Symbol;Acc:HGNC:52037]
ENSG00000230548	MTND4P27	1.604200526	0.793897861	0.000633418	0.003954754	MT-ND4 pseudogene 27 [Source:HGNC Symbol;Acc:HGNC:42214]
ENSG00000111788	None	1.603964608	4.94245716	3.25E-06	3.72E-05	DEAD/H (Asp-Glu-Ala-Asp/His) box polypeptide like pseudogene
ENSG00000178764	ZHX2	1.603700317	4.685630925	1.70E-22	1.45E-20	zinc fingers and homeoboxes 2 [Source:HGNC Symbol;Acc:HGNC:18513]
ENSG00000271435	TEKT4P3	1.602822459	0.792725074	0.000644956	0.004017969	tektin 4 pseudogene 3 [Source:HGNC Symbol;Acc:HGNC:42330]
ENSG00000090932	DLL3	1.599918205	5.968197375	9.51E-27	1.17E-24	delta like canonical Notch ligand 3 [Source:HGNC Symbol;Acc:HGNC:2909]
ENSG00000177508	IRX3	1.599604344	5.530754247	0.000107298	0.000847536	iroquois homeobox 3 [Source:HGNC Symbol;Acc:HGNC:14360]
ENSG00000100376	FAM118A	1.599278065	5.465919518	6.84E-08	1.09E-06	family with sequence similarity 118 member A [Source:HGNC Symbol;Acc:HGNC:1313]
ENSG00000243620	None	1.598646391	3.650660869	1.25E-05	0.000126686	novel transcript
ENSG00000263727	None	1.597638377	1.176078805	3.92E-06	4.43E-05	novel transcript
ENSG00000124762	CDKN1A	1.597337879	6.795234419	3.81E-15	1.61E-13	cyclin dependent kinase inhibitor 1A [Source:HGNC Symbol;Acc:HGNC:1784]
ENSG00000259887	FIGNL2-DT	1.59498685	2.136495116	0.00010265	0.000814214	FIGNL2 divergent transcript [Source:HGNC Symbol;Acc:HGNC:53299]
ENSG00000245870	LINC00682	1.593184901	-0.641474016	1.71E-05	0.000167788	long intergenic non-protein coding RNA 682 [Source:HGNC Symbol;Acc:HGNC:44466]
ENSG00000232284	GNG12-AS1	1.5931515	6.687439539	2.43E-26	2.88E-24	GNG12
ENSG00000151136	ABTB3	1.592296149	3.815092715	1.04E-15	4.71E-14	ankyrin repeat and BTB domain containing 3 [Source:HGNC Symbol;Acc:HGNC:23844]
ENSG00000172197	MBOAT1	1.582883767	2.165860589	7.83E-07	1.02E-05	membrane bound O-acyltransferase domain containing 1 [Source:HGNC Symbol;Acc:HGNC:21579]
ENSG00000263674	None	1.581412875	-0.293829054	0.000237226	0.001704536	novel transcript
ENSG00000006071	ABCC8	1.581266004	4.45304498	2.25E-16	1.08E-14	ATP binding cassette subfamily C member 8 [Source:HGNC Symbol;Acc:HGNC:59]
ENSG00000151468	CCDC3	1.579748622	4.312114031	2.69E-17	1.44E-15	coiled-coil domain containing 3 [Source:HGNC Symbol;Acc:HGNC:23813]
ENSG00000173535	TNFRSF10C	1.577956508	0.091232945	0.001382935	0.00766319	TNF receptor superfamily member 10c [Source:HGNC Symbol;Acc:HGNC:11906]
ENSG00000164853	UNCX	1.575085565	5.866493628	1.94E-06	2.34E-05	UNC homeobox [Source:HGNC Symbol;Acc:HGNC:33194]
ENSG00000203392	None	1.57436691	-0.054708431	1.83E-05	0.000177816	novel transcript
ENSG00000139352	ASCL1	1.572767675	6.186874177	2.92E-19	1.85E-17	achaete-scute family bHLH transcription factor 1 [Source:HGNC Symbol;Acc:HGNC:738]
ENSG00000248266	None	1.568308293	2.567222128	7.21E-12	2.08E-10	novel transcript
ENSG00000229273	None	1.565809534	1.958286995	4.72E-07	6.46E-06	novel pseudogene
ENSG00000255644	None	1.565392442	-0.256751849	2.86E-08	4.90E-07	novel transcript
ENSG00000120332	TNN	1.5635935	-0.274701504	3.04E-05	0.000278298	tenascin N [Source:HGNC Symbol;Acc:HGNC:22942]
ENSG00000163545	NUAK2	1.563420388	3.949695194	5.66E-09	1.11E-07	NUAK family kinase 2 [Source:HGNC Symbol;Acc:HGNC:29558]
ENSG00000214776	None	1.55842337	2.089616379	4.69E-08	7.71E-07	alpha-2-macroglobulin like 1 pseudogene
ENSG00000216811	None	1.557945028	-0.106847105	0.000103786	0.000821838	phosphoribosyl pyrophosphate synthetase (PRPS1
ENSG00000154553	PDLIM3	1.554255782	4.984636331	9.66E-08	1.49E-06	PDZ and LIM domain 3 [Source:HGNC Symbol;Acc:HGNC:20767]
ENSG00000260423	LINC02367	1.55213242	0.357851206	2.55E-06	3.00E-05	long intergenic non-protein coding RNA 2367 [Source:HGNC Symbol;Acc:HGNC:53290]
ENSG00000174021	GNG5	1.550044333	5.203840188	7.40E-26	8.55E-24	G protein subunit gamma 5 [Source:HGNC Symbol;Acc:HGNC:4408]
ENSG00000113396	SLC27A6	1.546134621	1.179947966	3.69E-09	7.47E-08	solute carrier family 27 member 6 [Source:HGNC Symbol;Acc:HGNC:11000]
ENSG00000233178	None	1.545723483	0.227841732	8.34E-07	1.08E-05	novel transcript
ENSG00000184384	MAML2	1.545048794	4.709768727	1.72E-19	1.12E-17	mastermind like transcriptional coactivator 2 [Source:HGNC Symbol;Acc:HGNC:16259]
ENSG00000185668	POU3F1	1.544490121	4.196608899	2.93E-05	0.000269644	POU class 3 homeobox 1 [Source:HGNC Symbol;Acc:HGNC:9214]
ENSG00000095587	TLL2	1.543129126	1.916027812	0.000331231	0.002279485	tolloid like 2 [Source:HGNC Symbol;Acc:HGNC:11844]
ENSG00000123700	KCNJ2	1.541745971	0.513046742	0.000365094	0.002477864	potassium inwardly rectifying channel subfamily J member 2 [Source:HGNC Symbol;Acc:HGNC:6263]
ENSG00000251018	HMMR-AS1	1.541301921	2.381916619	2.41E-07	3.45E-06	HMMR antisense RNA 1 [Source:HGNC Symbol;Acc:HGNC:49149]
ENSG00000151615	POU4F2	1.536601947	1.602729151	2.35E-09	4.91E-08	POU class 4 homeobox 2 [Source:HGNC Symbol;Acc:HGNC:9219]
ENSG00000273291	None	1.5344174	1.815989174	1.53E-10	3.75E-09	novel protein
ENSG00000177822	TENM3-AS1	1.534089531	4.875974577	3.20E-17	1.70E-15	TENM3 antisense RNA 1 [Source:HGNC Symbol;Acc:HGNC:28076]
ENSG00000091986	CCDC80	1.533625857	1.380230931	3.49E-07	4.87E-06	coiled-coil domain containing 80 [Source:HGNC Symbol;Acc:HGNC:30649]
ENSG00000214534	ZNF705EP	1.531590262	-0.069616695	0.000208525	0.001528495	zinc finger protein 705E
ENSG00000147509	RGS20	1.530268174	5.344914224	1.35E-08	2.46E-07	regulator of G protein signaling 20 [Source:HGNC Symbol;Acc:HGNC:14600]
ENSG00000231064	THBS3-AS1	1.53009015	6.073191579	1.07E-12	3.45E-11	THBS3 antisense RNA 1 [Source:HGNC Symbol;Acc:HGNC:40582]
ENSG00000145242	EPHA5	1.529859628	7.31888918	0.000846851	0.00507461	EPH receptor A5 [Source:HGNC Symbol;Acc:HGNC:3389]
ENSG00000196834	POTEI	1.526796044	3.043131962	6.02E-06	6.55E-05	POTE ankyrin domain family member I [Source:HGNC Symbol;Acc:HGNC:37093]
ENSG00000188064	WNT7B	1.52658322	6.342695704	7.28E-10	1.64E-08	Wnt family member 7B [Source:HGNC Symbol;Acc:HGNC:12787]
ENSG00000198189	HSD17B11	1.522387239	3.18315601	2.07E-06	2.48E-05	hydroxysteroid 17-beta dehydrogenase 11 [Source:HGNC Symbol;Acc:HGNC:22960]
ENSG00000023839	ABCC2	1.52190404	1.758861027	6.39E-07	8.50E-06	ATP binding cassette subfamily C member 2 [Source:HGNC Symbol;Acc:HGNC:53]
ENSG00000172340	SUCLG2	1.517388737	4.25427139	2.52E-11	6.91E-10	succinate-CoA ligase GDP-forming subunit beta [Source:HGNC Symbol;Acc:HGNC:11450]
ENSG00000130158	DOCK6	1.515265442	5.004650992	7.46E-09	1.43E-07	dedicator of cytokinesis 6 [Source:HGNC Symbol;Acc:HGNC:19189]
ENSG00000079134	THOC1	1.512748216	6.173200571	1.37E-11	3.85E-10	THO complex subunit 1 [Source:HGNC Symbol;Acc:HGNC:19070]
ENSG00000216901	ZNF603P	1.511990724	-0.463624689	2.99E-06	3.47E-05	zinc finger protein 603
ENSG00000214081	CYP4F30P	1.510851913	4.11723155	0.000560849	0.003582176	cytochrome P450 family 4 subfamily F member 30
ENSG00000257062	None	1.510307482	0.045057218	8.26E-08	1.29E-06	novel transcript
ENSG00000171759	PAH	1.508339472	4.727332198	8.86E-16	4.05E-14	phenylalanine hydroxylase [Source:HGNC Symbol;Acc:HGNC:8582]
ENSG00000184414	IRS3P	1.508324476	-0.20110406	4.49E-05	0.000392389	insulin receptor substrate 3
ENSG00000158955	WNT9B	1.507590585	1.540250914	1.04E-08	1.95E-07	Wnt family member 9B [Source:HGNC Symbol;Acc:HGNC:12779]
ENSG00000143995	MEIS1	1.507560446	6.429836472	1.92E-20	1.36E-18	Meis homeobox 1 [Source:HGNC Symbol;Acc:HGNC:7000]
ENSG00000076356	PLXNA2	1.506961066	8.57353172	3.63E-11	9.71E-10	plexin A2 [Source:HGNC Symbol;Acc:HGNC:9100]
ENSG00000182118	FAM89A	1.504222671	3.19790138	7.93E-10	1.77E-08	family with sequence similarity 89 member A [Source:HGNC Symbol;Acc:HGNC:25057]
ENSG00000257595	LINC02356	1.504213688	0.285193592	7.19E-06	7.67E-05	long intergenic non-protein coding RNA 2356 [Source:HGNC Symbol;Acc:HGNC:53278]
ENSG00000114656	CFAP92	1.502280724	5.237974252	4.16E-15	1.75E-13	cilia and flagella associated protein 92 (putative) [Source:HGNC Symbol;Acc:HGNC:29231]
ENSG00000103460	TOX3	1.502151953	6.329279698	2.73E-25	3.06E-23	TOX high mobility group box family member 3 [Source:HGNC Symbol;Acc:HGNC:11972]
ENSG00000204556	None	1.502130019	0.967357012	0.001104276	0.006333347	zinc-finger pseudogene
ENSG00000163362	INAVA	1.498615099	2.830893518	1.43E-07	2.14E-06	innate immunity activator [Source:HGNC Symbol;Acc:HGNC:25599]
ENSG00000124224	PPP4R1L	1.496897019	3.449013357	1.13E-06	1.42E-05	protein phosphatase 4 regulatory subunit 1 like (pseudogene) [Source:HGNC Symbol;Acc:HGNC:15755]

ENSG00000175592	FOSL1	1.496718276	-0.630407068	0.000135254	0.001041116	FOS like 1
ENSG00000162745	OLFML2B	1.493856019	0.690852907	5.64E-06	6.18E-05	olfactomedin like 2B [Source:HGNC Symbol;Acc:HGNC:24558]
ENSG00000132688	NES	1.492067264	9.709938582	5.12E-22	4.25E-20	nestin [Source:HGNC Symbol;Acc:HGNC:7756]
ENSG00000236908	LINC02827	1.49143918	0.157934231	3.87E-08	6.46E-07	long intergenic non-protein coding RNA 2827 [Source:HGNC Symbol;Acc:HGNC:54358]
ENSG00000239402	CYP4F62P	1.491433313	4.167192664	0.000338822	0.002324499	cytochrome P450 family 4 subfamily F member 62
ENSG00000251131	None	1.489982002	-0.028070551	6.05E-08	9.72E-07	novel transcript
ENSG00000121064	SCPEP1	1.487219635	3.700480613	4.07E-06	4.57E-05	serine carboxypeptidase 1 [Source:HGNC Symbol;Acc:HGNC:29507]
ENSG00000138738	PRDM5	1.486445241	4.095278546	3.90E-06	4.41E-05	PR/SET domain 5 [Source:HGNC Symbol;Acc:HGNC:9349]
ENSG00000187498	COL4A1	1.484383956	5.41598907	3.69E-07	5.13E-06	collagen type IV alpha 1 chain [Source:HGNC Symbol;Acc:HGNC:2202]
ENSG00000260361	None	1.484294111	0.04681495	0.000547928	0.003509803	novel transcript
ENSG00000196593	ANKRD20A19P	1.481795687	0.672811699	8.98E-06	9.39E-05	ankyrin repeat domain 20 family member A19
ENSG00000105810	CDK6	1.481485817	5.599165974	1.12E-24	1.18E-22	cyclin dependent kinase 6 [Source:HGNC Symbol;Acc:HGNC:1777]
ENSG00000174137	FAM53A	1.477680572	0.848185856	9.61E-05	0.000768127	family with sequence similarity 53 member A [Source:HGNC Symbol;Acc:HGNC:31860]
ENSG00000260518	BMS1P8	1.476656547	1.275156009	8.06E-05	0.00065786	BMS1 pseudogene 8 [Source:HGNC Symbol;Acc:HGNC:49152]
ENSG00000072571	HMMR	1.474730709	3.693046761	5.39E-08	8.73E-07	hyaluronan mediated motility receptor [Source:HGNC Symbol;Acc:HGNC:5012]
ENSG00000248479	None	1.473229007	-0.456782779	0.000618125	0.003880849	novel transcript
ENSG00000223685	LINC00571	1.471333524	2.22294474	2.93E-06	3.40E-05	long intergenic non-protein coding RNA 571 [Source:HGNC Symbol;Acc:HGNC:43721]
ENSG00000162843	WDR64	1.469124318	-0.204539279	1.36E-06	1.68E-05	WD repeat domain 64 [Source:HGNC Symbol;Acc:HGNC:26570]
ENSG00000258086	GPR84-AS1	1.469087547	4.880933689	1.20E-08	2.21E-07	GPR84
ENSG00000169231	THBS3	1.466946581	6.376024135	3.07E-13	1.07E-11	thrombospondin 3 [Source:HGNC Symbol;Acc:HGNC:11787]
ENSG00000234948	LINC01524	1.466122372	-0.300141893	6.62E-05	0.000554069	long intergenic non-protein coding RNA 1524 [Source:HGNC Symbol;Acc:HGNC:51228]
ENSG00000079335	CDC14A	1.466074243	4.085096335	8.93E-13	2.92E-11	cell division cycle 14A [Source:HGNC Symbol;Acc:HGNC:1718]
ENSG00000129654	FOXJ1	1.465020122	2.956213308	2.62E-07	3.74E-06	forkhead box J1 [Source:HGNC Symbol;Acc:HGNC:3816]
ENSG00000236591	UST-AS2	1.462416356	-0.474027831	4.93E-08	8.03E-07	UST antisense RNA 2 [Source:HGNC Symbol;Acc:HGNC:40803]
ENSG00000144485	HES6	1.462309664	7.484127068	7.60E-21	5.53E-19	hes family bHLH transcription factor 6 [Source:HGNC Symbol;Acc:HGNC:18254]
ENSG00000260467	None	1.460288862	-0.065495103	1.80E-07	2.62E-06	novel transcript
ENSG00000184486	POU3F2	1.459946801	7.916214759	3.13E-22	2.66E-20	POU class 3 homeobox 2 [Source:HGNC Symbol;Acc:HGNC:9215]
ENSG00000214826	DDX12P	1.458052422	4.929824299	1.54E-05	0.000152851	DEAD/H-box helicase 12
ENSG00000258137	GPR84-AS1	1.453980776	4.89349813	1.33E-08	2.43E-07	GPR84
ENSG00000227110	LMCD1-AS1	1.453128198	3.544494807	2.02E-12	6.22E-11	LMCD1 antisense RNA 1 [Source:HGNC Symbol;Acc:HGNC:44477]
ENSG00000180758	GPR157	1.45180534	0.969220497	0.000276689	0.001953742	G protein-coupled receptor 157 [Source:HGNC Symbol;Acc:HGNC:23687]
ENSG00000270792	None	1.451224225	2.780650532	1.93E-07	2.80E-06	novel transcript
ENSG00000173110	HSPA6	1.447954773	1.321267912	5.32E-06	5.86E-05	heat shock protein family A (Hsp70) member 6 [Source:HGNC Symbol;Acc:HGNC:5239]
ENSG00000167664	TMIGD2	1.447633115	-0.647732954	8.37E-06	8.82E-05	transmembrane and immunoglobulin domain containing 2 [Source:HGNC Symbol;Acc:HGNC:28324]
ENSG00000172345	STARD5	1.446914428	1.684548451	4.77E-07	6.52E-06	StAR related lipid transfer domain containing 5 [Source:HGNC Symbol;Acc:HGNC:18065]
ENSG00000093072	ADA2	1.446814294	2.520115892	2.40E-07	3.43E-06	adenosine deaminase 2 [Source:HGNC Symbol;Acc:HGNC:1839]
ENSG00000231908	IDH1-AS1	1.446370484	-0.629966162	5.91E-07	7.92E-06	IDH1 antisense RNA 1 [Source:HGNC Symbol;Acc:HGNC:40292]
ENSG00000013573	DDX11	1.443865716	5.260485933	7.20E-09	1.39E-07	DEAD/H-box helicase 11 [Source:HGNC Symbol;Acc:HGNC:2736]
ENSG00000231702	None	1.442992662	0.314935267	0.000359943	0.002445422	ribosomal protein L39 (RPL39) pseudogene
ENSG00000100292	HMOX1	1.440264509	1.201555176	6.18E-08	9.89E-07	heme oxygenase 1 [Source:HGNC Symbol;Acc:HGNC:5013]
ENSG00000273328	None	1.436951783	0.456037706	1.07E-08	1.99E-07	novel transcript
ENSG00000231473	RB1-DT	1.436147669	1.072689435	0.000236732	0.00170145	RB1 divergent transcript [Source:HGNC Symbol;Acc:HGNC:42778]
ENSG00000250846	EPHA5-AS1	1.433807528	4.590839306	1.03E-06	1.30E-05	EPHA5 antisense RNA 1 [Source:HGNC Symbol;Acc:HGNC:50602]
ENSG00000163219	ARHGAP25	1.433283158	-0.193203281	5.26E-06	5.79E-05	Rho GTPase activating protein 25 [Source:HGNC Symbol;Acc:HGNC:28951]
ENSG00000197594	ENPP1	1.428209109	1.577553276	9.70E-06	0.000100598	ectonucleotide pyrophosphatase/phosphodiesterase 1 [Source:HGNC Symbol;Acc:HGNC:3356]
ENSG00000187054	TMPRSS11A	1.425644621	-0.220615623	3.33E-08	5.63E-07	transmembrane serine protease 11A [Source:HGNC Symbol;Acc:HGNC:27954]
ENSG00000225549	SELENOKP3	1.424918728	-0.504851821	3.01E-06	3.49E-05	selenoprotein K pseudogene 3 [Source:HGNC Symbol;Acc:HGNC:53726]
ENSG00000224969	None	1.424015431	1.719041536	0.000409126	0.00273738	novel transcript
ENSG00000104894	CD37	1.423229954	5.43336568	1.22E-10	3.03E-09	CD37 molecule [Source:HGNC Symbol;Acc:HGNC:1666]
ENSG00000118985	ELL2	1.421695646	3.544942242	6.04E-06	6.57E-05	elongation factor for RNA polymerase II 2 [Source:HGNC Symbol;Acc:HGNC:17064]
ENSG00000204516	MICB	1.420419559	-0.308259782	8.74E-06	9.17E-05	MHC class I polypeptide-related sequence B [Source:HGNC Symbol;Acc:HGNC:7091]
ENSG00000244159	RPS27AP13	1.418498614	0.250927615	8.80E-07	1.13E-05	RPS27A pseudogene 13 [Source:HGNC Symbol;Acc:HGNC:36667]
ENSG00000079385	CEACAM1	1.418358681	2.365961009	5.95E-06	6.48E-05	CEA cell adhesion molecule 1 [Source:HGNC Symbol;Acc:HGNC:1814]
ENSG00000050555	LAMC3	1.417442058	0.734248918	0.001283785	0.007202209	laminin subunit gamma 3 [Source:HGNC Symbol;Acc:HGNC:6494]
ENSG00000154330	PGM5	1.414246117	3.26197357	2.91E-07	4.10E-06	phosphoglucomutase 5 [Source:HGNC Symbol;Acc:HGNC:8908]
ENSG00000257579	None	1.412179897	0.56399015	3.76E-08	6.30E-07	novel transcript
ENSG00000177663	IL17RA	1.412101586	3.170656102	4.83E-06	5.35E-05	interleukin 17 receptor A [Source:HGNC Symbol;Acc:HGNC:5985]
ENSG00000173548	SNX33	1.411271229	4.403081818	5.17E-08	8.40E-07	sorting nexin 33 [Source:HGNC Symbol;Acc:HGNC:28468]
ENSG00000260644	HERC2P5	1.410397755	3.25942769	0.000462767	0.003034547	HERC2 pseudogene 5 [Source:HGNC Symbol;Acc:HGNC:4873]
ENSG00000242242	NECTIN3-AS1	1.409707792	1.796483017	2.02E-09	4.24E-08	NECTIN3 antisense RNA 1 [Source:HGNC Symbol;Acc:HGNC:40813]
ENSG00000245017	LINC02453	1.408919499	-0.936317151	6.51E-05	0.000545119	long intergenic non-protein coding RNA 2453 [Source:HGNC Symbol;Acc:HGNC:53392]
ENSG00000271078	None	1.406845303	-0.886674966	7.70E-06	8.16E-05	NEDD4 binding protein 2 (N4BP2) pseudogene
ENSG00000272983	None	1.40446609	2.103859586	1.62E-05	0.000160208	novel transcript
ENSG00000230223	ATXN8OS	1.404391831	2.408755817	2.38E-05	0.000224699	ATXN8 opposite strand lncRNA [Source:HGNC Symbol;Acc:HGNC:10561]
ENSG00000260876	LINC01229	1.401151073	0.62029969	2.32E-05	0.000219467	long intergenic non-protein coding RNA 1229 [Source:HGNC Symbol;Acc:HGNC:49682]
ENSG00000273112	None	1.39858673	1.565685132	2.11E-06	2.52E-05	novel transcript
ENSG00000267082	None	1.397844361	3.190387795	2.40E-06	2.84E-05	novel transcript
ENSG00000167077	MEI1	1.396189231	-0.482542765	1.95E-05	0.000187627	meiotic double-stranded break formation protein 1 [Source:HGNC Symbol;Acc:HGNC:28613]
ENSG00000237027	None	1.395602562	1.144606624	0.001334392	0.007446344	novel transcript
ENSG00000270497	None	1.395286841	0.894767735	0.000798759	0.004833311	ribosomal protein S3A (RPS3A) pseudogene
ENSG00000229009	TMPRSS11GP	1.395240063	0.238185103	4.00E-07	5.52E-06	transmembrane serine protease 11G
ENSG00000230749	MEIS1-AS2	1.394487465	1.838356634	5.11E-07	6.94E-06	MEIS1 antisense RNA 2 [Source:HGNC Symbol;Acc:HGNC:40370]
ENSG00000105855	ITGB8	1.394253081	7.581027504	1.78E-08	3.18E-07	integrin subunit beta 8 [Source:HGNC Symbol;Acc:HGNC:6163]
ENSG00000257354	MIRLET7IHG	1.393389482	3.973304678	0.000146028	0.001115963	MIRLET7I host gene [Source:HGNC Symbol;Acc:HGNC:55478]
ENSG00000099139	PCSK5	1.392115529	4.099211498	1.76E-08	3.14E-07	proprotein convertase subtilisin/kexin type 5 [Source:HGNC Symbol;Acc:HGNC:8747]
ENSG00000162600	OMA1	1.391525072	3.39136203	3.03E-05	0.000277654	OMA1 zinc metallopeptidase [Source:HGNC Symbol;Acc:HGNC:29661]
ENSG00000102760	RGCC	1.390728623	0.285014962	0.001112436	0.006371441	regulator of cell cycle [Source:HGNC Symbol;Acc:HGNC:20369]
ENSG00000171208	NETO2	1.390111054	7.346257185	0.00030529	0.002127421	neuropilin and tolloid like 2 [Source:HGNC Symbol;Acc:HGNC:14644]
ENSG00000248779	None	1.389306647	0.748744749	1.25E-05	0.000126807	novel transcript
ENSG00000260689	HNRNPA3P11	1.389238095	2.395757725	1.47E-10	3.62E-09	heterogeneous nuclear ribonucleoprotein A3 pseudogene 11 [Source:HGNC Symbol;Acc:HGNC:48757]
ENSG00000253762	None	1.388616941	1.689977611	1.77E-05	0.000173365	novel transcript
ENSG00000250899	None	1.386881249	-0.588589186	4.69E-06	5.22E-05	novel transcript
ENSG00000232046	LINC01798	1.382765804	1.669180156	2.63E-08	4.54E-07	long intergenic non-protein coding RNA 1798 [Source:HGNC Symbol;Acc:HGNC:52588]
ENSG00000188383	GPAT2P2	1.380912983	0.271167402	0.000118075	0.000923329	glycerol-3-phosphate acyltransferase 2 pseudogene 2 [Source:HGNC Symbol;Acc:HGNC:52331]
ENSG00000143578	CREB3L4	1.379956765	3.620234812	1.28E-08	2.34E-07	cAMP responsive element binding protein 3 like 4 [Source:HGNC Symbol;Acc:HGNC:18854]
ENSG00000203747	FCGR3A	1.3794264	-0.426732213	1.61E-05	0.000159085	Fc gamma receptor IIIa [Source:HGNC Symbol;Acc:HGNC:3619]
ENSG00000144115	THNSL2	1.379262197	2.740002218	0.000304476	0.002122304	threonine synthase like 2 [Source:HGNC Symbol;Acc:HGNC:25602]
ENSG00000235091	None	1.378669163	3.222035301	1.33E-05	0.000133473	novel transcript
ENSG00000228115	None	1.375852138	-0.612147435	0.001805564	0.009617607	novel transcript
ENSG00000221227	MIR1305	1.375510752	-0.319548129	1.06E-06	1.34E-05	microRNA 1305 [Source:HGNC Symbol;Acc:HGNC:35303]
ENSG00000018408	WWTR1	1.372970691	4.414398288	3.13E-14	1.19E-12	WW domain containing transcription regulator 1 [Source:HGNC Symbol;Acc:HGNC:24042]
ENSG00000249036	None	1.372891742	-0.688340707	2.98E-05	0.000273435	novel transcript
ENSG00000213225	NOC2LP1	1.370747674	0.960746185	0.000428272	0.002842735	NOC2 like nucleolar associated transcriptional repressor pseudogene 1 [Source:HGNC Symbol;Acc:HGNC:52285]
ENSG00000250062	MAPK10-AS1	1.370593449	4.56956246	4.47E-14	1.67E-12	MAPK10 antisense RNA 1 [Source:HGNC Symbol;Acc:HGNC:40359]
ENSG00000175264	CHST1	1.370329427	6.158897354	1.02E-20	7.38E-19	carbohydrate sulfotransferase 1 [Source:HGNC Symbol;Acc:HGNC:1969]
ENSG00000161513	FDXR	1.369359922	4.724546965	1.45E-05	0.00014443	ferredoxin reductase [Source:HGNC Symbol;Acc:HGNC:3642]
ENSG00000009709	PAX7	1.367888746	6.946980391	2.26E-07	3.24E-06	paired box 7 [Source:HGNC Symbol;Acc:HGNC:8621]
ENSG00000186907	RTN4RL2	1.366499458	4.303847308	1.80E-12	5.63E-11	reticulon 4 receptor like 2 [Source:HGNC Symbol;Acc:HGNC:23053]
ENSG00000121361	KCNJ8	1.365684023	0.419650853	1.72E-07	2.52E-06	potassium inwardly rectifying channel subfamily J member 8 [Source:HGNC Symbol;Acc:HGNC:6269]
ENSG00000197061	H4C3	1.365237285	-0.776540342	0.000669528	0.004150603	H4 clustered histone 3 [Source:HGNC Symbol;Acc:HGNC:4787]
ENSG00000227757	None	1.362683683	1.924251947	2.93E-07	4.12E-06	novel transcript
ENSG00000008438	PGLYRP1	1.360596496	0.779427567	1.14E-05	0.00011643	peptidoglycan recognition protein 1 [Source:HGNC Symbol;Acc:HGNC:8904]
ENSG00000165338	HECTD2	1.35990914	6.575654807	2.29E-07	3.29E-06	HECT domain E3 ubiquitin protein ligase 2 [Source:HGNC Symbol;Acc:HGNC:26736]
ENSG00000184497	TMEM255B	1.356775271	0.023701328	1.35E-07	2.03E-06	transmembrane protein 255B [Source:HGNC Symbol;Acc:HGNC:28297]
ENSG00000270955	None	1.356431968	-0.202491323	0.000157915	0.001196821	NEDD4 binding protein 2 (N4BP2) pseudogene
ENSG00000234377	OBI1-AS1	1.355914785	6.100236088	3.53E-15	1.50E-13	OBI1 antisense RNA 1 [Source:HGNC Symbol;Acc:HGNC:42700]
ENSG00000197153	H3C12	1.355747397	0.766762647	2.55E-06	3.01E-05	H3 clustered histone 12 [Source:HGNC Symbol;Acc:HGNC:4774]

ENSG00000250938	MAD2L1-DT	1.35478359	2.667653762	6.32E-05	0.000530373	MAD2L1 divergent transcript [Source:HGNC Symbol;Acc:HGNC:55546]
ENSG00000185332	TMEM105	1.354577309	-0.648768469	0.000575677	0.003661068	TMEM105 long non-coding RNA [Source:HGNC Symbol;Acc:HGNC:26794]
ENSG00000263874	LINC00672	1.354124025	1.822608189	1.83E-05	0.000177709	long intergenic non-protein coding RNA 672 [Source:HGNC Symbol;Acc:HGNC:44353]
ENSG00000123552	USP45	1.353625145	5.238035075	3.37E-08	5.69E-07	ubiquitin specific peptidase 45 [Source:HGNC Symbol;Acc:HGNC:20080]
ENSG00000146242	TPBG	1.353525352	4.003814681	3.29E-08	5.57E-07	trophoblast glycoprotein [Source:HGNC Symbol;Acc:HGNC:12004]
ENSG00000172139	SLC9C1	1.353411554	1.177257564	9.96E-06	0.000103008	solute carrier family 9 member C1 [Source:HGNC Symbol;Acc:HGNC:31401]
ENSG00000240966	RN7SL681P	1.352651396	-0.708724507	5.73E-05	0.000487048	RNA
ENSG00000214946	TBC1D26	1.350303764	1.823988074	0.001382974	0.00766319	TBC1 domain family member 26 [Source:HGNC Symbol;Acc:HGNC:28745]
ENSG00000261599	HERC2P8	1.35003788	3.218569406	0.000290688	0.002038732	HERC2 pseudogene 8 [Source:HGNC Symbol;Acc:HGNC:38747]
ENSG00000225057	None	1.349163043	7.559885982	1.48E-18	8.85E-17	novel transcript
ENSG00000270964	MAP2K5-DT	1.34898517	0.148926958	1.00E-05	0.00010349	MAP2K5 divergent transcript [Source:HGNC Symbol;Acc:HGNC:55261]
ENSG00000134115	CNTN6	1.348969645	3.534160357	1.48E-06	1.82E-05	contactin 6 [Source:HGNC Symbol;Acc:HGNC:2176]
ENSG00000130173	ANGPTL8	1.347701983	1.06028097	9.19E-06	9.58E-05	angiopoietin like 8 [Source:HGNC Symbol;Acc:HGNC:24933]
ENSG00000187608	ISG15	1.346034458	2.0792005	0.000107721	0.000850119	ISG15 ubiquitin like modifier [Source:HGNC Symbol;Acc:HGNC:4053]
ENSG00000071282	LMCD1	1.345396679	1.072298602	1.56E-07	2.31E-06	LIM and cysteine rich domains 1 [Source:HGNC Symbol;Acc:HGNC:6633]
ENSG00000232855	None	1.342976224	1.333593825	1.12E-05	0.000114252	novel transcript
ENSG00000228043	SPOPL-DT	1.342339969	0.893299118	0.000232393	0.001675244	SPOPL divergent transcript [Source:HGNC Symbol;Acc:HGNC:55802]
ENSG00000116830	TTF2	1.338794846	4.112553721	1.29E-05	0.000129707	transcription termination factor 2 [Source:HGNC Symbol;Acc:HGNC:12398]
ENSG00000188372	ZP3	1.332794498	1.975442543	1.75E-08	3.12E-07	zona pellucida glycoprotein 3 [Source:HGNC Symbol;Acc:HGNC:13189]
ENSG00000095209	TMEM38B	1.331247561	4.40022797	2.74E-14	1.05E-12	transmembrane protein 38B [Source:HGNC Symbol;Acc:HGNC:25535]
ENSG00000130821	SLC6A8	1.330382134	7.456345818	1.99E-18	1.17E-16	solute carrier family 6 member 8 [Source:HGNC Symbol;Acc:HGNC:11055]
ENSG00000243244	STON1	1.330325782	4.714117139	2.18E-13	7.62E-12	stonin 1 [Source:HGNC Symbol;Acc:HGNC:17003]
ENSG00000100433	KCNK10	1.329008187	5.657988997	6.80E-20	4.62E-18	potassium two pore domain channel subfamily K member 10 [Source:HGNC Symbol;Acc:HGNC:6273]
ENSG00000259132	None	1.327930877	5.050570276	2.62E-06	3.08E-05	novel protein
ENSG00000269300	None	1.326579516	1.980360156	5.12E-06	5.65E-05	novel transcript
ENSG00000187010	RHD	1.32588278	2.693574331	7.68E-08	1.21E-06	Rh blood group D antigen [Source:HGNC Symbol;Acc:HGNC:10009]
ENSG00000179104	TMTC2	1.325694858	6.869315964	4.63E-13	1.57E-11	transmembrane O-mannosyltransferase targeting cadherins 2 [Source:HGNC Symbol;Acc:HGNC:25440]
ENSG00000270071	None	1.324565599	2.840600139	1.20E-07	1.82E-06	novel transcript
ENSG00000265179	None	1.322901581	2.70835845	3.21E-08	5.45E-07	novel transcript
ENSG00000226772	None	1.32199161	0.651374719	5.91E-07	7.92E-06	novel transcript
ENSG00000110090	CPT1A	1.321401937	1.98823363	3.23E-05	0.000293626	carnitine palmitoyltransferase 1A [Source:HGNC Symbol;Acc:HGNC:2328]
ENSG00000189067	LITAF	1.321143079	3.997825813	5.05E-08	8.21E-07	lipopolysaccharide induced TNF factor [Source:HGNC Symbol;Acc:HGNC:16841]
ENSG00000255159	DENND2B-AS1	1.321082986	1.373596578	8.60E-08	1.34E-06	DENND2B antisense RNA 1 [Source:HGNC Symbol;Acc:HGNC:56176]
ENSG00000265671	None	1.320136483	2.872989925	2.96E-08	5.06E-07	novel transcript

Fig4D GO_downregulated_genes

[illegible]

DPF3	LPAR1
MIR105-2	CACNA1E
MIR105-1	RYR2
MIR767	LRRTM3
DENND1B	BRINP2
CPLX3	RASGEF1A
S1PR3	MIR488
HMX1	RIPPLY2
TSTD1	FOXD6
SP9	MAFB
PLXNA4	COL15A1
RAB6C	DPF3
MIR137HG	MIR105-2
SHISA9	MIR105-1
OR2W3	MIR767
PSMB9	DENND1B
CEBPA	CPLX3
SELENOP	S1PR3
TRNP1	HMX1
SALL3	TSTD1
ZNF578	SP9
FRRS1L	PLXNA4
ZIM2	RAB6C
DOC2B	MIR137HG
GRIN2B	SHISA9
	OR2W3
	PSMB9
	CEBPA
	SELENOP
	TRNP1
	SALL3
	ZNF578
	FRRS1L
	ZIM2
	DOC2B
	GRIN2B

GO:BP GO:0034220 monoatomic_ion_transmembrane_transport 86	GO:BP GO:0044057 regulation_of_system_process 60	GO:BP GO:0043269 regulation_of_monoatomic_ion_transport 61	GO:BP GO:0048812 neuron_projection_morphogenesis 64	GO:BP GO:0030001 metal_ion_transport 77	GO:BP GO:0000902 cell_morphogenesis 81	GO:BP GO:0120039 plasma_membrane_bounded_cell_projection_morphogenesis 64
CACNG3	CAMK2B	CACNG3	PLXND1	CACNG3	PLXND1	PLXND1
SLC6A7	ATP2B3	CNTN1	ETV1	SLC6A7	ETV1	ETV1
GABRA3	MGLL	CAMK2B	GAS7	CNTN1	GAS7	GAS7
ATP2B3	CACNG5	CAMK2A	PAX6	CAMK2B	PAX6	PAX6
TRPC7	NOS1	P2RX5	CNTN1	ATP2B3	CNTN1	CNTN1
CNGB1	MYH7	ABCB1	MYO16	TRPC7	CDH10	MYO16
TRPC5	HCN2	NOS1	JADE2	CAMK2A	MYO16	JADE2
CACNG5	PDGFB	HCN2	XK	TRPC5	JADE2	XK
P2RX5	CHGA	PDGFB	FSTL4	CACNG5	XK	FSTL4
ABCB1	NTSR1	CACNA1I	CAMK2B	P2RX5	FSTL4	CAMK2B
NOS1	CELF4	MMP9	LZTS1	NOS1	CAMK2B	LZTS1
HCN2	HBEGF	NTSR1	NGEF	HCN2	LZTS1	NGEF
CACNA1I	FGF12	NALCN	TRPC5	PDGFB	NGEF	TRPC5
MMP9	IGFBP5	JPH1	SEMA3A	CACNA1I	TRPC5	SEMA3A
NTSR1	TNR	GCK	SEMA3C	NTSR1	SEMA3A	SEMA3C
NALCN	DLGAP3	FGF12	DCX	NALCN	SEMA3C	DCX
JPH1	SPP1	HES1	SYT17	JPH1	DCX	SYT17
SLC17A7	KCNJ5	KCNJ5	LHX2	SLC17A7	CDH7	LHX2
SFXN3	KCNA5	HPCA	SH3GL2	GCK	PALMD	SH3GL2
GABRG2	GCH1	TWIST1	LGI1	CDH23	CDH20	LGI1
FGF12	PPARG	BEST3	SEMA5A	FGF12	SYT17	SEMA5A
SLC9A2	CHRM3	KCNA5	UNC5A	HES1	LHX2	UNC5A
KCNJ5	CCN3	SCN2A	TNR	SLC9A2	SH3GL2	TNR
HPCA	CASQ1	SCN7A	PLPPR4	KCNJ5	CDH23	PLPPR4
TWIST1	IGSF11	EGF	NRP2	HPCA	LGI1	NRP2
SLC12A5	NLGN4X	KCNH5	SPP1	SLC12A5	SEMA5A	SPP1
BEST3	HTR2C	CACNG8	PACSIN1	KCNA5	CDH6	PACSIN1
KCNA5	SCN2B	CASQ1	CPNE5	RAMP2	UNC5A	CPNE5
SLC6A11	ADRA2A	STAC	EFNB2	SLC6A11	HES1	EFNB2
SCN2A	PTPRO	SNCA	BCL11B	SCN2A	TNR	BCL11B
[illegible]	[illegible]	[illegible]	[illegible]	[illegible]	[illegible]	[illegible]

GO:BP GO:0051049 regulation_of_transport 119	GO:BP GO:0048858 cell_projection_morphogenesis 64	GO:BP GO:0009966 regulation_of_signal_transduction 171	GO:BP GO:0030030 cell_projection_organization 109	GO:BP GO:0007610 behavior 62	GO:BP GO:0010975 regulation_of_neuron_projection_development 50	GO:BP GO:0032990 cell_part_morphogenesis 64
CACNG3	PLXND1	ITGA3	PLXND1	ITGA3	PLXND1	PLXND1
TENM1	ETV1	CACNG3	ITGA3	TAC1	ITGA3	ETV1
SYT7	GAS7	TENM1	ETV1	ETV1	CNTN1	GAS7
CNTN1	PAX6	LMO3	GAS7	CNTN1	XK	PAX6
RAB27B	CNTN1	FSTL4	PAX6	RASGRF1	FSTL4	CNTN1
PTPRN	MYO16	F7	TENM1	BRINP1	CAMK2B	MYO16
RASGRF1	JADE2	RASGRF1	CNTN1	RAPGEF3	DGKG	JADE2
CAMK2B	XK	DGKG	MYO16	RASD2	LZTS1	XK
ACSL4	FSTL4	PTPRU	TNC	NTSR1	NGEF	FSTL4
CAMK2A	CAMK2B	TLE2	CAPG	DNAH11	TRPC5	CAMK2B
CACNG5	LZTS1	PDE4A	JADE2	CDH23	SEMA3A	LZTS1
RAPGEF3	NGEF	NGEF	XK	GABRG2	SEMA3C	NGEF
P2RX5	TRPC5	KIF26A	FSTL4	FGF12	SFRP1	TRPC5
ABCB1	SEMA3A	CAMK2A	RASGRF1	TNR	SEMA5A	SEMA3A
ACHE	SEMA3C	ALPK1	CAMK2B	EGR1	HES1	SEMA3C
RPH3A	DCX	MGLL	DGKG	SLC12A5	PRRX1	DCX
NOS1	SYT17	NUAK1	LZTS1	VGF	TNR	SYT17
HCN2	LHX2	SEMA3A	NGEF	PPP1R1B	PLPPR5	LHX2
PDGFB	SH3GL2	CACNG5	CAMK2A	ATP8A2	SPP1	SH3GL2
CACNA1I	LGI1	MID2	TRPC5	MEIS2	PACSIN1	LGI1
CHGA	SEMA5A	P2RX5	ALPK1	SCN2A	EFNB2	SEMA5A
MMP9	UNC5A	WNT11	SEMA3A	HCRTR2	ATP8A2	UNC5A
NTSR1	TNR	SEZ6L	SEMA3C	SLC4A10	EPHA7	TNR
NALCN	PLPPR4	RASD2	DCX	SNCA	KIAA0319	PLPPR4
SYT17	NRP2	PDGFB	CARMIL1	NLGN4X	PTPRO	NRP2
SFRP1	SPP1	CHGA	CRTAC1	HTR2C	RIT2	SPP1
JPH1	PACSIN1	MMP9	PPP1R16B	KIRREL3	SPOCK1	PACSIN1
SLC17A7	CPNE5	DOK5	SYT17	ANKFN1	THY1	CPNE5
GCK	EFNB2	NTSR1	SFRP1	GRIA1	ELAVL4	EFNB2
SYT10	BCL11B	RSPO4	LHX2	SLC24A2	SERPINI1	BCL11B
[illegible]	[illegible]	[illegible]	[illegible]	[illegible]	[illegible]	[illegible]

GO:BP GO:0120036 plasma_membrane_bounded_cell_projection_organization 106	GO:BP GO:0034762 regulation_of_transmembrane_transport 58	GO:BP GO:0055085 transmembrane_transport 106	GO:BP GO:0098660 inorganic_ion_transmembrane_transport 75	GO:BP GO:0006812 monoatomic_cation_transport 82	GO:BP GO:0032989 cellular_component_morphogenesis 68	GO:BP GO:0030154 cell_differentiation 218
PLXND1	CACNG3	ABCB5	CACNG3	CACNG3	PLXND1	PLXND1
ITGA3	RASGRF1	CACNG3	SLC6A7	SLC6A7	ETV1	ABCB5
ETV1	CACNG5	SLC6A7	GABRA3	CNTN1	GAS7	ITGA3
GAS7	P2RX5	GABRA3	ATP2B3	CAMK2B	PAX6	YBX2
PAX6	ABCB1	SLC7A14	TRPC7	ATP2B3	CNTN1	ETV1
TENM1	NOS1	RASGRF1	TRPC5	TRPC7	MYO16	GAS7
CNTN1	HCN2	ATP2B3	CACNG5	CNGB1	JADE2	PAX6
MYO16	CACNA1I	TRPC7	P2RX5	CAMK2A	XK	TENM1
TNC	MMP9	CNGB1	NOS1	TRPC5	FSTL4	CNTN1
JADE2	NTSR1	TRPC5	HCN2	CACNG5	CAMK2B	PLEKHB1
XK	NALCN	CACNG5	CACNA1I	P2RX5	LZTS1	MYO16
FSTL4	JPH1	P2RX5	NTSR1	NOS1	NGEF	TNC
RASGRF1	FGF12	ABCB1	NALCN	HCN2	TRPC5	JADE2
CAMK2B	KCNJ5	NOS1	JPH1	PDGFB	SEMA3A	XK
DGKG	HPCA	HCN2	SLC17A7	CACNA1I	SEMA3C	LMO3
LZTS1	TWIST1	CACNA1I	GABRG2	MMP9	DCX	FSTL4
NGEF	KCNA5	MMP9	FGF12	NTSR1	NEBL	RASGRF1
CAMK2A	CHRM3	SLCO4A1	SLC9A2	NALCN	MYH7	CAMK2B
TRPC5	RNASEL	NTSR1	KCNJ5	JPH1	SYT17	DGKG
ALPK1	SCN2A	NALCN	HPCA	SLC17A7	LHX2	PTPRU
SEMA3A	SCN7A	JPH1	TWIST1	GCK	SH3GL2	LZTS1
SEMA3C	KCNH5	SLC17A7	SLC12A5	CDH23	LGI1	NGEF
DCX	CACNG8	SFXN3	BEST3	FGF12	SEMA5A	ACSL4
CARMIL1	CASQ1	GABRG2	KCNA5	HES1	UNC5A	CNGB1
CRTAC1	STAC	FGF12	SLC6A11	SLC9A2	TNR	CAMK2A
PPP1R16B	SNCA	SLC9A2	ADAMTS8	KCNJ5	PLPPR4	TRPC5
SYT17	SCN2B	CNNM1	SCN2A	HPCA	NRP2	SEMA3A
SFRP1	ADRA2A	KCNJ5	SCN7A	TWIST1	SPP1	SEMA3C
LHX2	CACNA2D4	HPCA	KCNH5	SLC12A5	PACSIN1	FOSL2
SH3GL2	KCNA6	TWIST1	CACNG8	KCNA5	CPNE5	DCX
[illegible]	[illegible]	[illegible]	[illegible]	[illegible]	[illegible]	[illegible]

GO:BP GO:0048468 cell_development 157	GO:BP GO:0007417 central_nervous_system_development 83	GO:BP GO:0042391 regulation_of_membrane_potential 47	GO:BP GO:0051239 regulation_of_multicellular_organismal_process 167	GO:BP GO:0034765 regulation_of_monoatomic_ion_transmembrane_transport 49	GO:BP GO:0048869 cellular_developmental_process 218	GO:BP GO:0032879 regulation_of_localization 129
PLXND1	PAX6	ABCB5	PLXND1	CACNG3	PLXND1	ITGA3
ITGA3	CNTN1	GABRA3	ITGA3	P2RX5	ABCB5	CACNG3
YBX2	MYO16	CNGB1	YBX2	ABCB1	ITGA3	TENM1
ETV1	CAPG	TRPC5	PAX6	NOS1	YBX2	SYT7
GAS7	SEMA3A	P2RX5	SYT7	HCN2	ETV1	CNTN1
PAX6	DCX	HCN2	TG	CACNA1I	GAS7	RAB27B
TENM1	DCT	CACNA1I	JADE2	MMP9	PAX6	PTPRN
CNTN1	SALL1	NTSR1	FSTL4	NTSR1	TENM1	RASGRF1
MYO16	SFRP1	CELF4	F7	NALCN	CNTN1	CAMK2B
TNC	SLC17A7	NALCN	CAMK2B	JPH1	PLEKHB1	ACSL4
JADE2	PPP1R17	GABRG2	ATP2B3	FGF12	MYO16	CAMK2A
XK	LHX2	FGF12	TRPC5	KCNJ5	TNC	CACNG5
FSTL4	SH3GL2	KCNJ5	MGLL	HPCA	JADE2	RAPGEF3
RASGRF1	NEURL1	KCNA5	SEMA3A	TWIST1	XK	P2RX5
CAMK2B	SEMA5A	SCN2A	SEMA3C	KCNA5	LMO3	ABCB1
DGKG	HES1	SCN7A	CACNG5	SCN2A	FSTL4	ACHE
LZTS1	TNR	KCNH5	BRINP1	SCN7A	RASGRF1	RPH3A
NGEF	CHD5	IGSF11	RAPGEF3	KCNH5	CAMK2B	NOS1
CNGB1	HPCAL4	SNCA	DCT	CACNG8	DGKG	HCN2
CAMK2A	NRP2	GABRA6	WNT11	CASQ1	PTPRU	PDGFB
TRPC5	HPCA	GABRB2	ACHE	STAC	LZTS1	CACNA1I
SEMA3A	CHST8	NLGN4X	NOS1	SNCA	NGEF	CHGA
SEMA3C	BMP2	SCN2B	MYH7	SCN2B	ACSL4	MMP9
FOSL2	BCL11B	GABRA2	HCN2	ADRA2A	CNGB1	NTSR1
DCX	SYNGR3	GRIA1	PDGFB	CACNA2D4	CAMK2A	NALCN
NEBL	SLC6A11	CACNA1D	CHGA	KCNA6	TRPC5	SYT17
BRINP1	PPARG	RNF207	MMP9	THY1	SEMA3A	SFRP1
RAPGEF3	BCAN	GJD2	NTSR1	CACNA1D	SEMA3C	JPH1
DCT	MEIS2	KCNJ9	PPP1R16B	CABP1	FOSL2	SLC17A7
ACHE	TSPAN2	KCNJ3	CELF4	RNF207	DCX	GCK
[illegible]	[illegible]	[illegible]	[illegible]	[illegible]	[illegible]	[illegible]

GO:BP GO:0048667 cell_morphogenesis_involved_in_neuron_differentiation 56
PLXND1, ETV1, PAX6, CNTN1, XK, FSTL4, CAMK2B, LZTS1, NGEF, TRPC5, SEMA3A, SEMA3C, DCX, LHX2, CDH23, LGI1, SEMA5A, UNC5A, HES1, TNR, PLPPR4, NRP2, SPP1, EFNB2, BCL11B, ATP8A2, EPHA7, KIAA0319, NFIB, CDHR1, PTPRO, THY1, SPTBN4, NTN3, ELAVL4, RET, ISLR2, IL1RAPL1, SEMA3E, TANC2, NPTX1, CDK5R2, DSCAM, KNDC1, DSCAML1, SLITRK4, ADGRB1, NOG, SLITRK6, ROBO2, MAPT, SHTN1, [illegible]

GO:BP GO:0007420 brain_development 67
PAX6, CNTN1, MYO16, SEMA3A, DCX, DCT, SALL1, SFRP1, SLC17A7, LHX2, NEURL1, SEMA5A, HES1, TNR, CHD5, NRP2, HPCA, BMP2, BCL11B, SYNGR3, SLC6A11, BCAN, MEIS2, TSPAN2, EPHA7, EGF, SSTR1, SLC4A10, NLGN4X, NFIB, KIRREL3, CDH22, CA10, ATP2B2, HTR5A, ACE, ELAVL4, WNT4, FZD6, PTCHD1, KCNC2, EMX2, SEMA3E, ALK, CDK5R2, NEUROD2, KNDC1, CNTNAP2, FUT1, PCSK1, DSCAML1, KCNA3, [illegible]

GO:BP GO:0061564 axon_development 51
PLXND1, ETV1, PAX6, CNTN1, TNC, XK, FSTL4, TRPC5, SEMA3A, SEMA3C, DCX, CRTAC1, LHX2, LGI1, SEMA5A, UNC5A, TNR, PLPPR4, NRP2, SPP1, EFNB2, BCL11B, ATP8A2, TSPAN2, EPHA7, KIAA0319, NFIB, PTPRO, THY1, NCAM2, SPTBN4, NTN3, RET, ISLR2, SEMA3E, NPTX1, CDK5R2, DSCAM, DSCAML1, SLITRK4, ADGRB1, NOG, SLITRK6, ROBO2, MAPT, SHTN1, RELN, GRM7, S100A6, MBP, PLXNA4

GO:BP GO:0048583 regulation_of_response_to_stimulus 204
ITGA3, CACNG3, TENM1, TNC, LMO3, FSTL4, F7, RASGRF1, CAMK2B, DGKG, PTPRU, TLE2, PDE4A, NGEF, KIF26A, CAMK2A, ALPK1, MGLL, NUAK1, SEMA3A, SEMA3C, CACNG5, MID2, P2RX5, ABCB1, WNT11, MYH7, SEZ6L, RASD2, PDGFB, CHGA, MMP9, DOK5, NTSR1, RSPO4, SAMHD1, PPP1R16B, CELF4, CHRDL1, SALL1, SFRP1, PLAT, SHB, VSIR, NEURL1, IL10RA, TPD52L1, EYA4, ARFGEF3, SEMA5A, HBEGF, LOX, [illegible]

GO:BP GO:0040011 locomotion 93
PLXND1, ITGA3, ETV1, PAX6, CNTN1, TNC, F7, CAMK2B, PTPRU, CAMK2A, SEMA3A, SEMA3C, CARMIL1, WNT11, PDGFB, CHGA, MMP9, SFRP1, LHX2, VSIR, SPOCK2, LGI1, SEMA5A, HBEGF, LOX, UNC5A, IGFBP5, TNR, NRP2, TEK, TWIST1, EFNB2, BMP2, F10, RHOJ, PLEKHG3, BCL11B, PPARG, EPHA7, CCN3, HCRTR2, EGF, FGF7, TMEFF2, SNCA, IGFBP3, GNRH1, NFIB, ADRA2A, PTPRO, THY1, CCSAP, [illegible]

GO:BP GO:0050877 nervous_system_process 100
ITGA3, CACNG3, TAC1, PAX6, GABRA3, RASGRF1, PDE4A, CNGB1, MGLL, CACNG5, BRINP1, RAPGEF3, P2RX5, MYH7, CACNA1I, NTSR1, CELF4, DNAH11, CDH23, SYT10, EYA4, GABRG2, FGF12, OTOF, EFEMP1, TNR, DLGAP3, TGFBI, PHF24, SLC12A5, PPP1R1B, GCH1, ATP8A2, MEIS2, SCN2A, SCN7A, CCN3, PLCB2, CACNG8, CASQ1, SLC4A10, STAC, IGSF11, SNCA, GABRA6, GABRB2, NLGN4X, HTR2C, ADRA2A, CACNA2D4, GABRA2, JAKMIP1, [illegible]

GO:BP GO:0007166 cell_surface_receptor_signaling_pathway 157
PLXND1, ITGA3, PAX6, GABRA3, CNTN1, JADE2, FSTL4, F7, PTPRU, TLE2, NGEF, CAMK2A, SEMA3A, SEMA3C, P2RX5, COL16A1, WNT11, PDGFB, MMP9, DOK5, NTSR1, RSPO4, SAMHD1, CELF4, CHRDL1, SALL1, SFRP1, PLAT, AMH, CRHR2, SHB, FBXW4, NEURL1, CCKBR, IL10RA, ADGRD1, EYA4, SEMA5A, HBEGF, LOX, GABRG2, CDH6, UNC5A, FGF12, HES1, EFEMP1, IGFBP5, PRRX1, PAPPA2, ANGPTL1, NRP2, TEK, [illegible]

GO:BP GO:0035556 intracellular_signal_transduction 148
ITGA3, TENM1, MYO16, JADE2, LMO3, F7, RASGRF1, DGKG, PDE4A, NGEF, RASGRP2, ALPK1, NUAK1, SEMA3A, DCX, ADCY2, RAPGEF3, MID2, STK17B, P2RX5, WNT11, NOS1, PITPNM2, SEZ6L, RASD2, PDGFB, MMP9, DOK5, PPP1R16B, [illegible]

GO:BP GO:0010647 positive_regulation_of_cell_communication 109
CACNG3, TENM1, LMO3, F7, RASGRF1, ACSL4, CAMK2A, ALPK1, SEMA3A, CACNG5, MID2, P2RX5, WNT11, RASD2, PDGFB, CHGA, MMP9, DOK5, RSPO4, NALCN, SALL1, SFRP1, CRHR2, GCK, NEURL1, LGI1, IL10RA, TPD52L1, SEMA5A, [illegible]

GO:BP GO:0031344 regulation_of_cell_projection_organization 57
PLXND1, ITGA3, TENM1, CNTN1, XK, FSTL4, CAMK2B, DGKG, LZTS1, NGEF, TRPC5, SEMA3A, SEMA3C, CARMIL1, PPP1R16B, SFRP1, NEURL1, SEMA5A, HES1, PRRX1, TNR, PLPPR5, SPP1, PACSIN1, EFNB2, ATP8A2, EPHA7, KIAA0319, PTPRO, [illegible]

GO:BP GO:0007409 axonogenesis 46
PLXND1, ETV1, PAX6, CNTN1, XK, FSTL4, TRPC5, SEMA3A, SEMA3C, DCX, LHX2, LGI1, SEMA5A, UNC5A, TNR, PLPPR4, NRP2, SPP1, EFNB2, BCL11B, ATP8A2, EPHA7, KIAA0319, NFIB, PTPRO, THY1, SPTBN4, NTN3, RET, [illegible]

GO:BP GO:0060322 head_development 67
PAX6, CNTN1, MYO16, SEMA3A, DCX, DCT, SALL1, SFRP1, SLC17A7, LHX2, NEURL1, SEMA5A, HES1, TNR, CHD5, NRP2, HPCA, BMP2, BCL11B, SYNGR3, SLC6A11, BCAN, MEIS2, TSPAN2, EPHA7, EGF, SSTR1, SLC4A10, NLGN4X, [illegible]

GO:BP GO:0034329 cell_junction_assembly 45
PLXND1, CDH10, LZTS1, CDH7, COL16A1, WNT11, ACHE, CDH20, SFRP1, SPOCK2, GABRG2, CDH6, TEK, EFNB2, RAMP2, EPHA7, SNCA, CDH18, GABRB2, NLGN4X, KIRREL3, CDH22, PTPRO, GABRA2, THY1, CLSTN2, ACE, WNT4, LRRTM1, [illegible]

GO:BP GO:0051960 regulation_of_nervous_system_development 46
PLXND1, PAX6, TG, JADE2, FSTL4, CAMK2B, TRPC5, SEMA3A, SEMA3C, BRINP1, DCT, LHX2, NEURL1, SEMA5A, HES1, TNR, SPP1, BMP2, PPARG, EPHA7, KIAA0319, THY1, FAIM, CLSTN2, ACE, LRRTM1, HEY1, SPINT1, ISLR2, [illegible]

GO:BP GO:0040012 regulation_of_locomotion 76
PLXND1, ITGA3, PAX6, TNC, F7, CAMK2B, PTPRU, CAMK2A, SEMA3A, SEMA3C, CARMIL1, WNT11, PDGFB, MMP9, SFRP1, VSIR, SPOCK2, SEMA5A, HBEGF, IGFBP5, TNR, NRP2, TEK, TWIST1, EFNB2, BMP2, F10, RHOJ, PLEKHG3, [illegible]

GO:BP GO:0120035 regulation_of_plasma_membrane_bounded_cell_projection_organization 56
PLXND1, ITGA3, TENM1, CNTN1, XK, FSTL4, CAMK2B, DGKG, LZTS1, NGEF, TRPC5, SEMA3A, SEMA3C, CARMIL1, PPP1R16B, SFRP1, NEURL1, SEMA5A, HES1, PRRX1, TNR, PLPPR5, SPP1, PACSIN1, EFNB2, ATP8A2, EPHA7, KIAA0319, PTPRO, [illegible]

GO:BP GO:0000904 cell_morphogenesis_involved_in_differentiation 57
PLXND1, ETV1, PAX6, CNTN1, XK, FSTL4, CAMK2B, LZTS1, NGEF, TRPC5, SEMA3A, SEMA3C, DCX, LHX2, CDH23, LGI1, SEMA5A, UNC5A, HES1, TNR, PLPPR4, NRP2, SPP1, EFNB2, BCL11B, ATP8A2, EPHA7, KIAA0319, NFIB, [illegible]

GO:BP GO:0098655 monoatomic_cation_transmembrane_transport 67
CACNG3, SLC6A7, ATP2B3, TRPC7, CNGB1, TRPC5, CACNG5, P2RX5, NOS1, HCN2, CACNA1I, MMP9, NTSR1, NALCN, JPH1, SLC17A7, FGF12, SLC9A2, KCNJ5, HPCA, TWIST1, SLC12A5, KCNA5, SLC6A11, SCN2A, SCN7A, KCNH5, CACNG8, CASQ1, [illegible]

GO:BP GO:0023056 positive_regulation_of_signaling 108
CACNG3, TENM1, LMO3, F7, RASGRF1, ACSL4, CAMK2A, ALPK1, SEMA3A, CACNG5, MID2, P2RX5, WNT11, RASD2, PDGFB, CHGA, MMP9, DOK5, RSPO4, NALCN, SALL1, SFRP1, CRHR2, GCK, NEURL1, LGI1, IL10RA, TPD52L1, SEMA5A, [illegible]

GO:BP GO:0007155 cell_adhesion 97
PLXND1, ITGA3, TENM1, CNTN1, CDH10, TNC, PTPRU, NUAK1, CARMIL1, CDH7, COL19A1, COL16A1, ACHE, PDGFB, CDH20, PCDH11X, SFRP1, SHB, CDH23, VSIR, SPOCK2, SEMA5A, CDH6, HES1, TNR, IGSF21, NRP2, SPP1, TEK, [illegible]

GO:BP GO:0098662 inorganic_cation_transmembrane_transport 64
CACNG3, SLC6A7, ATP2B3, TRPC7, TRPC5, CACNG5, P2RX5, NOS1, HCN2, CACNA1I, NTSR1, NALCN, JPH1, SLC17A7, FGF12, SLC9A2, KCNJ5, HPCA, TWIST1, SLC12A5, KCNA5, SLC6A11, SCN2A, SCN7A, KCNH5, CACNG8, CASQ1, SLC4A10, STAC, [illegible]

GO:BP GO:0007188 adenylate_cyclase-modulating_G_protein-coupled_receptor_signaling_pathway 31
PDE4A, ADCY2, CHGA, CRHR2, ADGRD1, NPR3, GRM4, RAMP2, GPR12, CHRM3, GABBR2, PRKACB, PTH2R, ADRA2A, RIT2, ADCY8, HTR5A, CACNA1D, GRM5, ADRA1B, GPR37, AKAP5, SSTR2, CHRM2, GPR3, ADGRB1, ADRA2C, PDE2A, GRM7, [illegible]

GO:BP GO:0050767 regulation_of_neurogenesis 39
PLXND1, PAX6, JADE2, FSTL4, CAMK2B, TRPC5, SEMA3A, SEMA3C, BRINP1, DCT, LHX2, NEURL1, SEMA5A, HES1, TNR, SPP1, BMP2, PPARG, EPHA7, KIAA0319, THY1, FAIM, ACE, HEY1, SPINT1, ISLR2, GRM5, IL1RAPL1, SEMA3E, [illegible]

GO:BP GO:0050793 regulation_of_developmental_process 136
PLXND1, PAX6, PLEKHB1, TG, JADE2, XK, LMO3, FSTL4, CAMK2B, DGKG, LZTS1, NGEF, TRPC5, SEMA3A, SEMA3C, BRINP1, RAPGEF3, DCT, WNT11, PALMD, MMP11, PDGFB, MMP9, PPP1R16B, CELF4, SYT17, SFRP1, AMH, LHX2, [illegible]

GO:BP GO:0007626 locomotory_behavior 28
CNTN1, RASD2, NTSR1, CDH23, FGF12, TNR, EGR1, PPP1R1B, SLC4A10, SNCA, HTR2C, ANKFN1, ADCY8, RCAN1, SPTBN4, ELAVL4, LRRTM1, GRM5, GPR37, ALK, DSCAM, KCNJ10, PENK, GPR88, SLITRK6, RELN, DPP4, SELENOP

GO:BP GO:0006813 potassium_ion_transport 30
NOS1, HCN2, NALCN, SLC17A7, GCK, SLC9A2, KCNJ5, SLC12A5, KCNA5, KCNH5, ADRA2A, KCNA6, SLC24A2, CACNA1D, RNF207, KCNJ9, SLC9C2, KCNF1, KCNJ3, KCNC2, KCNK9, KCND3, PRNP, NPPA, KCNA3, KCNJ10, KCNK4, KCNJ12, SLC24A3, [illegible]

GO:BP GO:0006816 calcium_ion_transport 42
CACNG3, CAMK2B, ATP2B3, TRPC7, CAMK2A, TRPC5, CACNG5, P2RX5, NOS1, PDGFB, CACNA1I, NTSR1, NALCN, JPH1, GCK, CDH23, HES1, HPCA, RAMP2, EGF, CACNG8, CASQ1, STAC, SNCA, HTR2C, ADRA2A, CACNA2D4, THY1, SLC24A2, [illegible]

GO:BP GO:0051128 regulation_of_cellular_component_organization 134
PLXND1, ITGA3, TENM1, SYT7, CNTN1, TNC, CAPG, JADE2, XK, FSTL4, CAMK2B, DGKG, LZTS1, NGEF, KIF26A, ACSL4, RASGRP2, SPTB, TRPC5, SEMA3A, SEMA3C, RAPGEF3, CARMIL1, COL16A1, WNT11, PDGFB, MMP9, PPP1R16B, SYT17, [illegible]

GO:BP
GO:0032409
regulation_of_transporter_activity
33

CACNG3, RASGRF1, CACNG5, ABCB1, NOS1, HCN2, MMP9, NTSR1, JPH1, FGF12, HPCA, TWIST1, SYNGR3, PPARG, CHRM3, CACNG8, CASQ1, STAC, SNCA, SCN2B, ADRA2A, CACNA1D, CABP1, RNF207, KCNC2, GRM5, PRNP, NPPA, AKAP5, SHISA6, RELN, RYR2, SHISA9

GO:BP
GO:0007611
learning_or_memory
32

ITGA3, RASGRF1, BRINP1, RAPGEF3, NTSR1, DNAH11, TNR, SLC12A5, PPP1R1B, MEIS2, SCN2A, NLGN4X, GRIA1, SLC24A2, ADCY8, SORCS3, RCAN1, ELAVL4, CAMK2N1, PTCHD1, GRM5, NEUROD2, PRNP, CNTNAP2, GPR88, KCNK4, NOG, GABRA5, MAPT, RELN, FOXO6, GRIN2B

GO:BP
GO:0050890
cognition
34

ITGA3, RASGRF1, BRINP1, RAPGEF3, NTSR1, DNAH11, TNR, SLC12A5, PPP1R1B, MEIS2, SCN2A, NLGN4X, JAKMIP1, GRIA1, SLC24A2, ADCY8, SORCS3, RCAN1, ELAVL4, CAMK2N1, PTCHD1, FAM107A, GRM5, NEUROD2, PRNP, CNTNAP2, GPR88, KCNK4, NOG, GABRA5, MAPT, RELN, FOXO6, GRIN2B

GO:BP
GO:2000145
regulation_of_cell_motility
69

PLXND1, ITGA3, PAX6, TNC, F7, CAMK2B, PTPRU, CAMK2A, SEMA3A, SEMA3C, CARMIL1, WNT11, PDGFB, MMP9, SFRP1, VSIR, SPOCK2, SEMA5A, HBEGF, IGFBP5, TNR, NRP2, TEK, TWIST1, BMP2, F10, RHOJ, PLEKHG3, PPARG, CCN3, EGF, FGF7, TMEFF2, IGFBP3, GNRH1, ADRA2A, THY1, CCSAP, SST, SLAMF8, NBL1, ACE, WNT4, ADAMTS9, ITGA2, RET, FAM107A, ROR2, SEMA3E, FUT9, MYD88, VEGFB, FUT1, CHST2, GSX2, FAM83H, ADGRB1, NOG, PRKD1, SHTN1, GCNT1, RELN, PTPRT, SPOCK3, FUT4, PDGFA, DPP4, LPAR1, PLXNA4

GO:BP
GO:0099505
regulation_of_presynaptic_membrane_potential
11

GABRA3, GABRB2, GABRA2, GRIA1, KCNJ9, KCNJ3, GABRB1, GRIK2, KCNC2, GABRA5, GRIN2B

GO:BP
GO:0030334
regulation_of_cell_migration
66

PLXND1, ITGA3, PAX6, TNC, F7, CAMK2B, PTPRU, CAMK2A, SEMA3A, SEMA3C, CARMIL1, WNT11, PDGFB, MMP9, SFRP1, VSIR, SEMA5A, HBEGF, IGFBP5, TNR, NRP2, TEK, TWIST1, BMP2, F10, RHOJ, PLEKHG3, PPARG, CCN3, EGF, FGF7, TMEFF2, IGFBP3, GNRH1, ADRA2A, THY1, SST, SLAMF8, NBL1, ACE, WNT4, ADAMTS9, ITGA2, RET, FAM107A, ROR2, SEMA3E, FUT9, MYD88, VEGFB, FUT1, CHST2, GSX2, FAM83H, ADGRB1, NOG, PRKD1, SHTN1, GCNT1, RELN, PTPRT, FUT4, PDGFA, DPP4, LPAR1, PLXNA4

GO:BP
GO:0022898
regulation_of_transmembrane_transporter_activity
31

CACNG3, RASGRF1, CACNG5, ABCB1, NOS1, HCN2, MMP9, NTSR1, JPH1, FGF12, HPCA, TWIST1, CHRM3, CACNG8, CASQ1, STAC, SNCA, SCN2B, ADRA2A, CACNA1D, CABP1, RNF207, KCNC2, GRM5, PRNP, NPPA, AKAP5, SHISA6, RELN, RYR2, SHISA9

GO:BP
GO:0071805
potassium_ion_transmembrane_transport
27

HCN2, NALCN, SLC17A7, SLC9A2, KCNJ5, SLC12A5, KCNA5, KCNH5, KCNA6, SLC24A2, CACNA1D, RNF207, KCNJ9, SLC9C2, KCNF1, KCNJ3, KCNC2, KCNK9, KCND3, PRNP, NPPA, KCNA3, KCNJ10, KCNK4, KCNJ12, SLC24A3, KCNQ5

GO:BP
GO:0050803
regulation_of_synapse_structure_or_activity
29

CAMK2B, LZTS1, NGEF, SLC17A7, NEURL1, IL10RA, NRP2, EPHA7, DGKB, SNCA, PTPRO, CLSTN2, LRRTM1, CAMKV, ROR2, IL1RAPL1, FRMPD4, TANC2, NEUROD2, PRNP, SLITRK4, ADGRB1, GPC6, SLITRK6, ROBO2, RELN, IL1RAPL2, LRRTM3, GRIN2B

GO:BP
GO:0006810
transport
220

ABCB5, CACNG3, TENM1, SLC6A7, SYT7, GABRA3, SLC7A14, CNTN1, RAB27B, TG, XK, PTPRN, RASGRF1, CAMK2B, ATP2B3, ACSL4, TRPC7, CNGB1, CAMK2A, TRPC5, SNCB, IPCEF1, CACNG5, RAPGEF3, CARMIL1, P2RX5, ABCB1, ACHE, RPH3A, [illegible]

GO:BP
GO:0048522
positive_regulation_of_cellular_process
249

PLXND1, ITGA3, CACNG3, ETV1, MATK, PAX6, NFIX, TENM1, SYT7, CNTN1, RAB27B, TNC, JADE2, LMO3, PTPRN, F7, RASGRF1, CAMK2B, PTPRU, ACSL4, CAMK2A, TRPC5, ALPK1, SEMA3A, SEMA3C, FOSL2, CACNG5, BRINP1, RAPGEF3, [illegible]

GO:BP
GO:0022603
regulation_of_anatomical_structure_morphogenesis
63

PLXND1, XK, FSTL4, CAMK2B, LZTS1, NGEF, TRPC5, SEMA3A, SEMA3C, RAPGEF3, WNT11, PALMD, PPP1R16B, SYT17, SFRP1, SEMA5A, HES1, TNR, SPP1, TEK, TWIST1, CPNE5, EFNB2, BMP2, RHOJ, FGD3, RAMP2, PPARG, EPHA7, [illegible]

GO:BP
GO:0048168
regulation_of_neuronal_synaptic_plasticity
14

RASGRF1, CAMK2B, CAMK2A, NEURL1, VGF, SLC4A10, SNCA, GRIK2, GRM5, KCNJ10, NOG, SHISA6, SHISA9, GRIN2B

GO:BP
GO:0009719
response_to_endogenous_stimulus
100

ITGA3, TAC1, LMO3, F7, PTPRU, CAMK2A, RAPGEF3, COL16A1, ACHE, NOS1, HCN2, PDGFB, DOK5, MKRAS, CHRDL1, SFRP1, PLAT, CRHR2, GCK, SH3GL2, NEURL1, LOX, GABRG2, FGF12, HES1, IGFBP5, NRP2, SPP1, EGR1, FLT3, TWIST1, BMP2, RASL11B, VGF, CALY, ASS1, RAMP2, PPP1R1B, PPARG, CHRM3, MEIS2, RERG, LMO2, AGAP2, MYC, DUSP6, SSTR1, FGF7, SNCA, GABRB2, HTR2C, GNRH1, ADRA2A, PPARGC1B, ADCY8, FBXO32, SST, HTR5A, NBL1, ACE, [illegible]

GO:BP
GO:0030900
forebrain_development
38

PAX6, SEMA3A, DCX, DCT, SALL1, LHX2, SEMA5A, HES1, TNR, CHD5, NRP2, BMP2, BCL11B, BCAN, SSTR1, SLC4A10, NFIB, KIRREL3, HTR5A, ELAVL4, WNT4, PTCHD1, KCNC2, EMX2, SEMA3E, ALK, CDK5R2, CNTNAP2, FUT1, PCSK1, KCNA3, GSX2, SSTR2, NOG, ROBO2, RELN, LPAR1, PLXNA4

GO:BP
GO:0050807
regulation_of_synapse_organization
28

CAMK2B, LZTS1, NGEF, NEURL1, IL10RA, NRP2, EPHA7, DGKB, SNCA, PTPRO, CLSTN2, LRRTM1, CAMKV, ROR2, IL1RAPL1, FRMPD4, TANC2, NEUROD2, PRNP, SLITRK4, ADGRB1, GPC6, SLITRK6, ROBO2, RELN, IL1RAPL2, LRRTM3, GRIN2B

GO:BP
GO:1901701
cellular_response_to_oxygen-containing_compound
81

SYT7, PTPRN, RASGRP2, CAMK2A, ADCY2, BRINP1, RAPGEF3, COL16A1, WNT11, ACHE, HCN2, PDGFB, MMP9, MAP1LC3A, SYT17, SFRP1, PLAT, CRHR2, GCK, NEURL1, SYT10, HES1, IGFBP5, SPP1, EGR1, HPCA, CPNE5, EFNB2, CALY, ASS1, PPP1R1B, GCH1, PPARG, CHRM3, POSTN, SYT6, CMPK2, AGAP2, SSTR1, SLC13A5, SNCA, HTR2C, ADRA2A, PPARGC1B, ADCY8, FBXO32, HTR5A, ACE, BRINP3, VCAM1, VSNL1, ITGA2, RET, KCNC2, PRKCB, RAB31, GRM5, IRS1, GPR37, ALK, [illegible]

GO:BP
GO:0065009
regulation_of_molecular_function
135

PLXND1, CACNG3, PAX6, TENM1, RASGRF1, NGEF, RASGRP2, CAMK2A, NUAK1, CACNG5, RAPGEF3, MID2, ABCB1, WNT11, NOS1, HCN2, PDGFB, MMP9, NTSR1, SFRP1, JPH1, AMH, PPP1R17, LHX2, SHB, VSIR, SPOCK2, NEURL1, TPD52L1, [illegible]

GO:BP
GO:0048518
positive_regulation_of_biological_process
273

PLXND1, ITGA3, YBX2, CACNG3, ETV1, MATK, PAX6, NFIX, TENM1, SYT7, CNTN1, RAB27B, TNC, JADE2, LMO3, PTPRN, F7, RASGRF1, CAMK2B, PTPRU, ACSL4, CNGB1, CAMK2A, TRPC5, ALPK1, SEMA3A, SEMA3C, FOSL2, CACNG5, [illegible]

GO:BP
GO:0140352
export_from_cell
62

SYT7, RAB27B, PTPRN, ATP2B3, ACSL4, CAMK2A, ABCB1, ACHE, RPH3A, CACNA1I, CHGA, NTSR1, SYT17, SFRP1, GCK, SYT10, OTOF, SPP1, GRM4, BMP2, SYNGR3, VGF, KCNA5, PPARG, CHRM3, SYT6, CCN3, UNC13C, SYT16, [illegible]

GO:BP
GO:0051179
localization
246

ABCB5, ITGA3, CACNG3, PAX6, TENM1, SLC6A7, SYT7, GABRA3, SLC7A14, CNTN1, RAB27B, TG, XK, PTPRN, RASGRF1, CAMK2B, PTPRU, SLC9A3R2, ATP2B3, ACSL4, TRPC7, CNGB1, CAMK2A, TRPC5, SNCB, IPCEF1, CACNG5, RAPGEF3, CARMIL1, [illegible]

GO:BP
GO:0007416
synapse_assembly
25

PLXND1, LZTS1, ACHE, SPOCK2, GABRG2, EFNB2, EPHA7, SNCA, GABRB2, NLGN4X, KIRREL3, GABRA2, CLSTN2, LRRTM1, IL1RAPL1, NPTX1, DSCAM, SLITRK4, ADGRB1, GPC6, SLITRK6, ROBO2, MAPT, IL1RAPL2, LRRTM3

GO:BP
GO:0051234
establishment_of_localization
223

ABCB5, CACNG3, PAX6, TENM1, SLC6A7, SYT7, GABRA3, SLC7A14, CNTN1, RAB27B, TG, XK, PTPRN, RASGRF1, CAMK2B, ATP2B3, ACSL4, TRPC7, CNGB1, CAMK2A, TRPC5, SNCB, IPCEF1, CACNG5, RAPGEF3, CARMIL1, P2RX5, ABCB1, ACHE, [illegible]

GO:BP
GO:0032412
regulation_of_monoatomic_ion_transmembrane_transporter_activity
29

CACNG3, RASGRF1, CACNG5, ABCB1, NOS1, HCN2, MMP9, NTSR1, JPH1, FGF12, HPCA, CHRM3, CACNG8, CASQ1, STAC, SCN2B, ADRA2A, CACNA1D, CABP1, RNF207, KCNC2, GRM5, PRNP, NPPA, AKAP5, SHISA6, RELN, RYR2, SHISA9

GO:BP
GO:0051094
positive_regulation_of_developmental_process
81

PLXND1, PAX6, JADE2, LMO3, CAMK2B, TRPC5, BRINP1, RAPGEF3, DCT, WNT11, PDGFB, SYT17, SFRP1, SHB, VSIR, NEURL1, SEMA5A, HES1, TEK, TWIST1, PACSIN1, CPNE5, EFNB2, BMP2, RAMP2, PPARG, ATP8A2, CD101, AGAP2, MMD2, MYC, EGF, IGFBP3, HTR2C, HMGA2, BEND6, PPARGC1B, FAIM, CLSTN2, NBL1, ACE, SPTBN4, ELAVL4, WNT4, BRINP3, LRRTM1, ADAMTS9, RET, SPINT1, PRKCB, ISLR2, NPNT, GRM5, ROR2, IL1RAPL1, ALK, NEUROD2, DSCAM, RASGRP1, VEGFB, [illegible]

GO:BP
GO:0008015
blood_circulation
43

ATP2B3, TRHDE, NOS1, MYH7, HCN2, PDGFB, CHGA, NTSR1, SH3GL2, HBEGF, NPR3, FGF12, TEK, KCNJ5, KCNA5, RAMP2, GCH1, PPARG, CHRM3, GRIP2, SCN2B, ADRA2A, PTPRO, ATP2B2, CACNA1D, RNF207, TNNI1, ACE, SPTBN4, KCNJ3, GUCY1A1, ADRA1B, KCND3, APLN, VEGFB, NPPA, CHRM2, CTNNA3, ADRA2C, KCNJ12, PDE2A, SMTNL2, RYR2

GO:BP
GO:0051962
positive_regulation_of_nervous_system_development
30

PLXND1, PAX6, JADE2, CAMK2B, TRPC5, DCT, NEURL1, SEMA5A, HES1, BMP2, PPARG, FAIM, CLSTN2, ACE, LRRTM1, SPINT1, ISLR2, GRM5, IL1RAPL1, DSCAM, SLITRK4, GSX2, ADGRB1, SLITRK6, ROBO2, MAPT, SHTN1, RELN, LRRTM3, PLXNA4

GO:BP
GO:1903522
regulation_of_blood_circulation
28

ATP2B3, NOS1, MYH7, HCN2, CHGA, HBEGF, KCNJ5, KCNA5, GCH1, CHRM3, SCN2B, ADRA2A, ATP2B2, CACNA1D, RNF207, ACE, SPTBN4, KCNJ3, ADRA1B, KCND3, APLN, NPPA, CHRM2, CTNNA3, ADRA2C, KCNJ12, SMTNL2, RYR2

GO:BP
GO:0010469
regulation_of_signaling_receptor_activity 22
CACNG3, RASGRF1, CACNG5, NEURL1, HBEGF, DLGAP3, PPARG, EGF, CACNG8, MUC4, ADRA2A, LY6E, NPTX1, LY6H, LYNX1, BEGAIN, NOG, ADRA2C, SHISA6, RELN, DLGAP2, SHISA9

GO:BP
GO:0032940
secretion_by_cell 58
SYT7, RAB27B, PTPRN, ACSL4, CAMK2A, ACHE, RPH3A, CACNA1I, CHGA, NTSR1, SYT17, SFRP1, GCK, SYT10, OTOF, SPP1, GRM4, BMP2, SYNGR3, VGF, KCNA5, PPARG, CHRM3, SYT6, CCN3, UNC13C, SYT16, FGF7, SNCA, CPLX2, HTR2C, ADRA2A, ADCY8, SYN2, VSNL1, PRKCB, TVP23A, RAB26, RAB3IL1, RAB31, LGI3, CPLX1, IRS1, RAB3B, IL1RAPL1, CDK5R2, SCG2, RASGRP1, PCSK1, ARL4D, RPH3AL, ADRA2C, SV2B, SLC38A3, GRM7, S100A10, CPLX3, DOC2B

GO:BP
GO:0003013
circulatory_system_process 47
ATP2B3, TRHDE, ABCB1, NOS1, MYH7, HCN2, PDGFB, CHGA, NTSR1, SH3GL2, HBEGF, NPR3, FGF12, TEK, KCNJ5, KCNA5, RAMP2, GCH1, PPARG, CHRM3, GRIP2, SCN2B, ADRA2A, PTPRO, ATP2B2, CACNA1D, RNF207, TNNI1, ACE, SPTBN4, KCNJ3, GUCY1A1, ADRA1B, KCND3, APLN, VEGFB, NPPA, CHRM2, CTNNA3, ADRA2C, KCNJ12, SLC24A3, PDE2A, SMTNL2, SLC38A3, SLC6A17, RYR2

GO:BP
GO:0035295
tube_development 70
PLXND1, ITGA3, PAX6, SEMA3C, RAPGEF3, WNT11, PPP1R16B, SALL1, SFRP1, LHX2, SHB, CCKBR, SEMA5A, LOX, NPR3, HES1, IGFBP5, PRRX1, NRP2, TEK, TGFBI, TWIST1, EFNB2, BMP2, RHOJ, ASS1, ANGPTL6, RAMP2, PPARG, MICAL2, EPHA7, MYC, CCN3, EGF, FGF7, PRKACB, NFIB, HMGA2, THY1, RNF207, ACE, WNT4, ADAMTS9, HPGD, HEY1, FZD6, RET, SPINT1, CLMP, PRKCB, NPNT, EMX2, SEMA3E, SOSTDC1, APLN, SCG2, EGFL7, VEGFB, FUT1, FZD8, ADGRB1, NOG, PRKD1, ROBO2, PDGFA, ADAMTSL2, RYR2, COL15A1, PLXNA4, CEBPA

GO:BP
GO:0021675
nerve_development 16
CNGB1, SEMA3A, SALL1, HES1, NRP2, GABRB2, VCAM1, RET, KCNC2, NPTX1, KCNA3, SLITRK6, GABRA5, LPAR1, MAFB, PLXNA4

GO:BP
GO:0048646
anatomical_structure_formation_involved_in_morphogenesis 74
PLXND1, ITGA3, PAX6, CNTN1, SEMA3C, DCX, NEBL, RAPGEF3, WNT11, NOS1, MYH7, MMP9, PPP1R16B, SALL1, SFRP1, LHX2, SHB, SEMA5A, NPR3, HES1, NRP2, TEK, TGFBI, DUSP4, TWIST1, EFNB2, BMP2, RHOJ, ANGPTL6, RAMP2, PPARG, ATP8A2, CCN3, DUSP5, EGF, PRKACB, CASQ1, NFIB, HMGA2, THY1, ADAMTS5, PPARGC1B, CSRP1, ACE, WNT4, ADAMTS9, ITGA2, HEY1, FZD6, RET, ADAMTS15, SPINT1, PRKCB, SEMA3E, APLN, CDK5R2, DSCAM, KNDC1, SCG2, EGFL7, VEGFB, FUT1, FZD8, FJX1, ADGRB1, NOG, PRKD1, ROBO2, RELN, LAMB3, PDGFA, RIPPLY2, MAFB, COL15A1

GO:BP
GO:0060047
heart_contraction 27
ATP2B3, NOS1, MYH7, HCN2, CHGA, HBEGF, FGF12, KCNJ5, KCNA5, GCH1, SCN2B, ATP2B2, CACNA1D, RNF207, TNNI1, ACE, SPTBN4, KCNJ3, ADRA1B, KCND3, APLN, VEGFB, NPPA, CHRM2, CTNNA3, KCNJ12, RYR2

GO:BP
GO:0046903
secretion 64
SYT7, RAB27B, PTPRN, ACSL4, CAMK2A, ACHE, RPH3A, CACNA1I, CHGA, NTSR1, SYT17, SFRP1, GCK, NEURL1, CCKBR, SYT10, NPR3, OTOF, SPP1, GRM4, BMP2, SYNGR3, VGF, KCNA5, PPARG, CHRM3, SYT6, CCN3, UNC13C, EGF, SYT16, FGF7, SNCA, CPLX2, HTR2C, ADRA2A, ADCY8, SYN2, ACE, VSNL1, PRKCB, TVP23A, RAB26, RAB3IL1, RAB31, LGI3, CPLX1, IRS1, RAB3B, IL1RAPL1, APLN, CDK5R2, SCG2, RASGRP1, PCSK1, ARL4D, RPH3AL, ADRA2C, SV2B, SLC38A3, GRM7, S100A10, CPLX3, DOC2B

GO:BP
GO:0044093
positive_regulation_of_molecular_function 89
PLXND1, CACNG3, PAX6, TENM1, RASGRF1, NGEF, RASGRP2, CAMK2A, RAPGEF3, MID2, ABCB1, WNT11, NOS1, PDGFB, MMP9, NTSR1, SFRP1, AMH, VSIR, NEURL1, TPD52L1, HBEGF, NPR3, HES1, TEK, EGR1, HPCA, FLT3, TWIST1, BMP2, SYNGR3, RFPL1, GCH1, PPARG, RAP1GAP2, ENTREP1, EPHA7, AGAP2, PKIB, MMD2, MYC, PLCB2, PARP9, EGF, CASQ1, STAC, SNCA, RASGEF1C, HMGA2, ADRA2A, THY1, PPARGC1B, ADCY8, CACNA1D, RNF207, ACE, SPATC1L, WNT4, ITGA2, RET, KCNC2, PRKCB, NPNT, GRM5, IRS1, ROR2, PARM1, ALK, CDK5R2, NEUROD2, PRNP, RASGRP1, MYD88, NPPA, FZD8, AKAP5, RGS6, ADRA2C, PRKD1, MAPT, RELN, PDGFA, S100A10, MBP, LPAR1, RYR2, RASGEF1A, DENND1B, GRIN2B

GO:BP
GO:0007193
adenylate_cyclase-inhibiting_G_protein-coupled_receptor_signaling_pathway 15
NPR3, GRM4, CHRM3, GABBR2, ADRA2A, HTR5A, GRM5, GPR37, AKAP5, SSTR2, CHRM2, PDE2A, GRM7, LPAR1, S1PR3

GO:BP
GO:0050806
positive_regulation_of_synaptic_transmission 22
CACNG3, CACNG5, NALCN, CRHR2, LGI1, TNR, CACNG8, IGSF11, SNCA, SLC24A2, ADCY8, CLSTN2, LRRTM1, GRIK2, FAM107A, ROR2, PRNP, CALB2, AKAP5, NOG, RELN, GRIN2B

GO:BP
GO:0006836
neurotransmitter_transport 24
SLC6A7, SYT7, CAMK2A, RPH3A, NOS1, SLC17A7, OTOF, GRM4, SYNGR3, SLC6A11, UNC13C, SNCA, CPLX2, GABRA2, SYN2, PRKCB, CPLX1, RAB3B, KCNJ10, RPH3AL, SV2B, SLC6A17, CPLX3, DOC2B

GO:BP
GO:0007411
axon_guidance 27
PLXND1, ETV1, PAX6, CNTN1, SEMA3A, SEMA3C, LHX2, LGI1, SEMA5A, UNC5A, TNR, NRP2, EFNB2, BCL11B, EPHA7, NFIB, PTPRO, NTN3, RET, SEMA3E, CDK5R2, DSCAM, DSCAML1, NOG, ROBO2, RELN, PLXNA4

GO:BP
GO:0048870
cell_motility 96
PLXND1, ITGA3, PAX6, TNC, F7, CAMK2B, PTPRU, CAMK2A, SEMA3A, SEMA3C, DCX, CARMIL1, WNT11, PDGFB, CACNA1I, CHGA, MMP9, SFRP1, PLAT, DNAH11, VSIR, SPOCK2, NEURL1, SEMA5A, HBEGF, LOX, HES1, IGFBP5, TNR, NRP2, TEK, TWIST1, EFNB2, BMP2, F10, RHOJ, PLEKHG3, BCL11B, PPARG, CCN3, KIAA0319, PARP9, EGF, FGF7, TMEFF2, IGFBP3, GNRH1, KIRREL3, ADRA2A, PTPRO, SPOCK1, THY1, CCSAP, SST, SLAMF8, NBL1, ACE, WNT4, VCAM1, LRATD1, ADAMTS9, ITGA2, RET, FAM107A, ROR2, EMX2, SEMA3E, CDK5R2, SCG2, FUT9, MYD88, VEGFB, FUT1, CHST2, GSX2, FAM83H, ADGRB1, GPC6, CTNNA3, EFHC2, NOG, PRKD1, FMNL1, SHTN1, GCNT1, ENO4, RELN, PTPRT, SPOCK3, FUT4, LAMB3, PDGFA, DPP4, LPAR1, RASGEF1A, PLXNA4

GO:BP
GO:0097485
neuron_projection_guidance 27
PLXND1, ETV1, PAX6, CNTN1, SEMA3A, SEMA3C, LHX2, LGI1, SEMA5A, UNC5A, TNR, NRP2, EFNB2, BCL11B, EPHA7, NFIB, PTPRO, NTN3, RET, SEMA3E, CDK5R2, DSCAM, DSCAML1, NOG, ROBO2, RELN, PLXNA4

GO:BP
GO:0099072
regulation_of_postsynaptic_membrane_neurotransmitter_receptor_levels 16
CACNG3, CACNG5, LGI1, HPCA, IQSEC2, EFNB2, CALY, CACNG8, GRIP2, SST, CPLX1, NPTX1, GPC6, SHISA6, LPAR1, FRRS1L

GO:BP
GO:0050770
regulation_of_axonogenesis 21
PLXND1, XK, FSTL4, TRPC5, SEMA3A, SEMA3C, SEMA5A, TNR, SPP1, EPHA7, KIAA0319, THY1, RET, ISLR2, SEMA3E, DSCAM, ROBO2, MAPT, SHTN1, MBP, PLXNA4

GO:BP
GO:0031345
negative_regulation_of_cell_projection_organization 23
ITGA3, FSTL4, NGEF, TRPC5, SEMA3A, SEMA3C, SEMA5A, HES1, TNR, SPP1, EFNB2, EPHA7, KIAA0319, PTPRO, RIT2, SPOCK1, THY1, SEMA3E, PRNP, INPP5J, MBP, LPAR1, GRIN2B

GO:BP
GO:0003015
heart_process 27
ATP2B3, NOS1, MYH7, HCN2, CHGA, HBEGF, FGF12, KCNJ5, KCNA5, GCH1, SCN2B, ATP2B2, CACNA1D, RNF207, TNNI1, ACE, SPTBN4, KCNJ3, ADRA1B, KCND3, APLN, VEGFB, NPPA, CHRM2, CTNNA3, KCNJ12, RYR2

GO:BP
GO:0016477
cell_migration 87
PLXND1, ITGA3, PAX6, TNC, F7, CAMK2B, PTPRU, CAMK2A, SEMA3A, SEMA3C, DCX, CARMIL1, WNT11, PDGFB, CHGA, MMP9, SFRP1, PLAT, VSIR, SEMA5A, HBEGF, LOX, HES1, IGFBP5, TNR, NRP2, TEK, TWIST1, EFNB2, BMP2, F10, RHOJ, PLEKHG3, BCL11B, PPARG, CCN3, KIAA0319, PARP9, EGF, FGF7, TMEFF2, IGFBP3, GNRH1, KIRREL3, ADRA2A, PTPRO, SPOCK1, THY1, SST, SLAMF8, NBL1, ACE, WNT4, VCAM1, ADAMTS9, ITGA2, RET, FAM107A, ROR2, EMX2, SEMA3E, CDK5R2, SCG2, FUT9, MYD88, VEGFB, FUT1, CHST2, GSX2, FAM83H, ADGRB1, GPC6, CTNNA3, NOG, PRKD1, FMNL1, SHTN1, GCNT1, RELN, PTPRT, FUT4, LAMB3, PDGFA, DPP4, LPAR1, RASGEF1A, PLXNA4

GO:BP
GO:1901700
response_to_oxygen-containing_compound 99
SYT7, PTPRN, F7, RASGRP2, CAMK2A, ADCY2, BRINP1, RAPGEF3, P2RX5, COL16A1, WNT11, ACHE, NOS1, HCN2, PDGFB, MMP9, MAP1LC3A, SYT17, SFRP1, PLAT, CRHR2, GCK, NEURL1, IL10RA, SYT10, RBP1, HES1, IGFBP5, SPP1, EGR1, HPCA, CPNE5, EFNB2, VGF, IRF5, KCNA5, CALY, ASS1, PPP1R1B, GCH1, PPARG, CHRM3, POSTN, SYT6, CMPK2, AGAP2, SSTR1, SLC13A5, SNCA, HTR2C, GNRH1, ADRA2A, PPARGC1B, ADCY8, FBXO32, SST, HTR5A, ACE, ELAVL4, BRINP3, VCAM1, VSNL1, GABRB1, HPGD, ITGA2, RET, KCNC2, PRKCB, RAB31, GRM5, IRS1, GPR37, ALK, NPTX1, NEUROD2, PRNP, MYD88, PCSK1, LY6H, KCNJ10, CPNE7, SSTR2, CHRM2, PENK, KCNK4, PRKD1, PDE2A, MAPT, GCNT1, CPNE4, SLC30A10, PDGFA, COL4A6, MBP, LPAR1, RYR2, BRINP2, CEBPA, GRIN2B

GO:BP
GO:0051480
regulation_of_cytosolic_calcium_ion_concentration 13
ATP2B3, TRPC7, TRPC5, CDH23, HCRTR2, GRIA1, ADCY8, ATP2B2, GRM5, CALB2, GPR3, SV2B, RYR2

GO:BP
GO:0007167
enzyme-linked_receptor_protein_signaling_pathway 64
ITGA3, JADE2, FSTL4, F7, PTPRU, NGEF, PDGFB, MMP9, DOK5, CHRDL1, SFRP1, PLAT, AMH, NEURL1, HBEGF, LOX, FGF12, HES1, EFEMP1, IGFBP5, ANGPTL1, NRP2, TEK, EGR1, FLT3, EFNB2, BMP2, RASL11B, PPARG, EPHA7, CCN3, KIAA0319, EGF, FGF7, SNCA, IGFBP3, ADRA2A, SLA, NBL1, WNT4, HPGD, RET, PRKCB, GFRA2, NPNT, IRS1, ROR2, REPS2, ALK, SOSTDC1, APLN, VEGFB, VWC2L, NPPA, SHISA2, NOG, PRKD1, COL4A5, VWC2, PTPRT, SLC30A10, PDGFA, COL4A6, ADAMTSL2

GO:BP
GO:0070588
calcium_ion_transmembrane_transport 32
CACNG3, ATP2B3, TRPC7, TRPC5, CACNG5, P2RX5, NOS1, CACNA1I, NTSR1, NALCN, JPH1, HPCA, CACNG8, CASQ1, STAC, SNCA, HTR2C, ADRA2A, CACNA2D4, THY1, SLC24A2, ATP2B2, CACNA1D, CABP1, PRNP, NPPA, AKAP5, PRKD1, SLC24A3, CACNA1E, RYR2, GRIN2B

GO:BP
GO:0055080
monoatomic_cation_homeostasis 45
CYB561, XK, ATP2B3, TRPC7, TRPC5, NOS1, NTSR1, JPH1, CDH23, CCKBR, SLC9A2, CNNM1, SLC12A5, KCNA5, GPR12, SCN7A, MYC, HCRTR2, CASQ1, SLC4A10, SNCA, HTR2C, THY1, GRIA1, SLC24A2, ADCY8, ATP2B2, SLAMF8, ACE, SLC9C2, GRIK2, PRKCB, TMC8, GRM5, PRNP, CALB2, KCNJ10, LACC1, GPR3, PRKD1, SLC24A3, SV2B, ATP13A5, SLC30A10, RYR2

GO:BP
GO:2000026
regulation_of_multicellular_organismal_development 82
PLXND1, PAX6, TG, JADE2, FSTL4, CAMK2B, TRPC5, SEMA3A, SEMA3C, BRINP1, RAPGEF3, DCT, WNT11, MMP9, PPP1R16B, SFRP1, LHX2, SHB, VSIR, NEURL1, MGP, SEMA5A, LOX, RBP1, HES1, EFEMP1, TNR, SPP1, TEK, TWIST1, BMP2, RHOJ, RAMP2, PPARG, MEIS2, CD101, EPHA7, MYC, KIAA0319, EGF, DUSP6, HMGA2, THY1, CCSAP, PPARGC1B, FAIM, CLSTN2, SLAMF8, ACE, WNT4, LRRTM1, ADAMTS9, HEY1, SPINT1, PRKCB, ISLR2, GRM5, ROR2, IL1RAPL1, SEMA3E, DSCAM, RASGRP1, VEGFB, FUT1, SLITRK4, GSX2, ADGRB1, NOG, PRKD1, SLITRK6, ROBO2, MAPT, SHTN1, RELN, S100A10, H4C16, MBP, LRRTM3, MAFB, S1PR3, PLXNA4, CEBPA

GO:BP
GO:0035239
tube_morphogenesis 58
PLXND1, RAPGEF3, WNT11, PPP1R16B, SALL1, SFRP1, LHX2, SHB, SEMA5A, LOX, NPR3, HES1, PRRX1, NRP2, TEK, TGFBI, TWIST1, EFNB2, BMP2, RHOJ, ANGPTL6, RAMP2, PPARG, MICAL2, EPHA7, MYC, CCN3, EGF, PRKACB, NFIB, HMGA2, THY1, RNF207, ACE, WNT4, ADAMTS9, HPGD, HEY1, FZD6, RET, SPINT1, PRKCB, NPNT, EMX2, SEMA3E, SOSTDC1, APLN, SCG2, EGFL7, VEGFB, FUT1, FZD8, ADGRB1, NOG, PRKD1, PDGFA, RYR2, COL15A1

GO:BP
GO:0010977
negative_regulation_of_neuron_projection_development 19
FSTL4, NGEF, SEMA3A, SEMA3C, SEMA5A, HES1, TNR, SPP1, EFNB2, EPHA7, KIAA0319, PTPRO, RIT2, SPOCK1, THY1, SEMA3E, INPP5J, MBP, LPAR1

GO:BP
GO:0007214
gamma-aminobutyric_acid_signaling_pathway
9
GABRA3
GABRG2
PHF24
GABBR2
GABRA6
GABRB2
GABRA2
GABRB1
GABRA5

GO:BP
GO:0050801
monoatomic_ion_homeostasis
45
CYB561
XK
ATP2B3
TRPC7
TRPC5
NOS1
NTSR1
JPH1
CDH23
CCKBR
SLC9A2
CNNM1
SLC12A5
KCNA5
GPR12
SCN7A
MYC
HCRTR2
CASQ1
SLC4A10
SNCA
HTR2C
THY1
GRIA1
SLC24A2
ADCY8
ATP2B2
SLAMF8
ACE
SLC9C2
GRIK2
PRKCB
TMC8
GRM5
PRNP
CALB2
KCNJ10
LACC1
GPR3
PRKD1
SLC24A3
SV2B
ATP13A5
SLC30A10
RYR2

GO:BP
GO:0071495
cellular_response_to_endogenous_stimulus
84
ITGA3
TAC1
LMO3
CAMK2A
RAPGEF3
COL16A1
ACHE
NOS1
HCN2
PDGFB
DOK5
CHRDL1
SFRP1
PLAT
CRHR2
GCK
SH3GL2
NEURL1
LOX
GABRG2
FGF12
HES1
IGFBP5
NRP2
EGR1
FLT3
TWIST1
BMP2
RASL11B
CALY
ASS1
RAMP2
PPP1R1B
PPARG
CHRM3
LMO2
AGAP2
SSTR1
FGF7
SNCA
GABRB2
HTR2C
ADRA2A
PPARGC1B
ADCY8
FBXO32
SST
HTR5A
NBL1
ACE
ELAVL4
WNT4
VCAM1
GABRB1
HPGD
SPINT1
PRKCB
FAM107A
RAB31
NPNT
GRM5
IRS1
ALK
SOSTDC1
APLN
PRNP
VEGFB
VWC2L
LYGH
SSTR2
SHISA2
CHRM2
PENK
NOG
PRKD1
ROBO2
PDE2A
MAPT
VWC2
SLC30A10
PDGFA
COL4A6
ADAMTSL2
RYR2

GO:BP
GO:1902075
cellular_response_to_salt
23
SYT7
RASGRP2
ACHE
PDGFB
SYT17
SYT10
EGR1
HPCA
CPNE5
PPP1R1B
CHRM3
SYT6
SLC13A5
ADCY8
PRKCB
NPTX1
NEUROD2
LYGH
KCNJ10
CPNE7
CHRM2
CPNE4
CEBPA

GO:BP
GO:0060284
regulation_of_cell_development
56
PLXND1
PAX6
JADE2
FSTL4
CAMK2B
TRPC5
SEMA3A
SEMA3C
BRINP1
DCT
SFRP1
LHX2
SHB
VSIR
NEURL1
SEMA5A
LOX
RBP1
HES1
TNR
SPP1
BMP2
PPARG
MEIS2
CD101
EPHA7
MYC
KIAA0319
THY1
PPARGC1B
FAIM
SLAMF8
ACE
HEY1
RET
SPINT1
ISLR2
GRM5
ROR2
IL1RAPL1
SEMA3E
DSCAM
RASGRP1
GSX2
NOG
ROBO2
MAPT
SHTN1
RELN
S100A10
H4C16
MBP
MAFB
S1PR3
PLXNA4
CEBPA

GO:BP
GO:0019226
transmission_of_nerve_impulse
14
CACNG3
CACNG5
CACNA1I
FGF12
TNR
SCN2A
SCN7A
CACNG8
GJD2
SPTBN4
ITGA2
GRIK2
CNTNAP2
GPR88

GO:BP
GO:0098742
cell-cell_adhesion_via_plasma-membrane_adhesion_molecules
28
ITGA3
TENM1
CDH10
CDH7
CDH20
PCDH11X
CDH23
CDH6
IGSF21
BMP2
IGSF11
CDH18
CDHR1
KIRREL3
CDH22
CLSTN2
VCAM1
RET
IL1RAPL1
PCDH7
DSCAM
DSCAML1
GPC6
CLDN6
ROBO2
PTPRT
DCHS2
MBP

GO:BP
GO:0019220
regulation_of_phosphate_metabolic_process
78
PAX6
TENM1
CNTN1
TRPC5
NUAK1
RAPGEF3
NOS1
PDGFB
MMP9
NTSR1
PPP1R16B
SFRP1
PPP1R17
SH3GL2
SHB
NEURL1
TPD52L1
HBEGF
LOX
HES1
TEK
MOB3B
EGR1
HPCA
FLT3
BMP2
PPP1R1B
PPARG
EPHA7
AGAP2
PKIB
MMD2
PARP9
EGF
DUSP6
FGF7
SNCA
IGFBP3
HTR2C
HMGA2
ADRA2A
PTPRO
RIT2
THY1
PPARGC1B
ADCY8
SLAMF8
RCAN1
ACE
SPATC1L
SPTBN4
CAMK2N1
DIRAS3
ITGA2
RET
NPNT
GRM5
IRS1
ROR2
ALK
CDK5R2
DSCAM
KNDC1
PRNP
RASGRP1
VEGFB
NPPA
FZD8
LACC1
NOG
ADRA2C
PRKD1
INPP5J
MAPT
RELN
PTPRT
PDGFA
CEBPA

GO:BP
GO:0006887
exocytosis
32
SYT7
RAB27B
RPH3A
CACNA1I
CHGA
SYT17
SYT10
OTOF
SYNGR3
SYT6
UNC13C
SYT16
SNCA
CPLX2
ADRA2A
SYN2
VSNL1
PRKCB
RAB26
RAB3IL1
RAB31
LGI3
CPLX1
RAB3B
IL1RAPL1
CDK5R2
RASGRP1
RPH3AL
SV2B
S100A10
CPLX3
DOC2B

GO:BP
GO:0051174
regulation_of_phosphorus_metabolic_process
78
PAX6
TENM1
CNTN1
TRPC5
NUAK1
RAPGEF3
NOS1
PDGFB
MMP9
NTSR1
PPP1R16B
SFRP1
PPP1R17
SH3GL2
SHB
NEURL1
TPD52L1
HBEGF
LOX
HES1
TEK
MOB3B
EGR1
HPCA
FLT3
BMP2
PPP1R1B
PPARG
EPHA7
AGAP2
PKIB
MMD2
PARP9
EGF
DUSP6
FGF7
SNCA
IGFBP3
HTR2C
HMGA2
ADRA2A
PTPRO
RIT2
THY1
PPARGC1B
ADCY8
SLAMF8
RCAN1
ACE
SPATC1L
SPTBN4
CAMK2N1
DIRAS3
ITGA2
RET
NPNT
GRM5
IRS1
ROR2
ALK
CDK5R2
DSCAM
KNDC1
PRNP
RASGRP1
VEGFB
NPPA
FZD8
LACC1
NOG
ADRA2C
PRKD1
INPP5J
MAPT
RELN
PTPRT
PDGFA
CEBPA

GO:BP
GO:0001508
action_potential
19
HCN2
CACNA1I
NTSR1
FGF12
KCNJ5
KCNA5
SCN2A
SCN7A
SCN2B
CACNA1D
RNF207
GJD2
KCNJ3
GRIK2
KCNC2
KCND3
GPR88
CTNNA3
RYR2

GO:BP
GO:0006936
muscle_contraction
31
NOS1
MYH7
CHGA
FGF12
KCNJ5
KCNA5
ATP8A2
CHRM3
SCN7A
CASQ1
GRIP2
STAC
SCN2B
ADRA2A
CACNA1D
RNF207
TNNI1
KCNJ3
GUCY1A1
ITGA2
NPNT
ADRA1B
KCND3
VEGFB
NPPA
SSTR2
CHRM2
CTNNA3
ADRA2C
KCNJ12
RYR2

GO:BP
GO:0090066
regulation_of_anatomical_structure_size
40
TENM1
CAPG
XK
FSTL4
SPTB
TRPC5
SEMA3A
SEMA3C
CARMIL1
NOS1
SEMA5A
NPR3
TNR
SPP1
SLC12A5
KCNA5
GCH1
RAP1GAP2
CHRM3
EPHA7
KIAA0319
SPTBN5
GRIP2
ADRA2A
ACE
SPTBN4
GUCY1A1
RET
ISLR2
RAB3B
ADRA1B
SEMA3E
DSCAM
NPPA
ADRA2C
MAPT
SHTN1
SMTNL2
CYRIA
PLXNA4

GO:BP
GO:0008016
regulation_of_heart_contraction
23
ATP2B3
NOS1
MYH7
HCN2
CHGA
HBEGF
KCNJ5
KCNA5
GCH1
SCN2B
ATP2B2
CACNA1D
RNF207
SPTBN4
KCNJ3
ADRA1B
KCND3
APLN
NPPA
CHRM2
CTNNA3
KCNJ12
RYR2

GO:BP
GO:0033555
multicellular_organismal_response_to_stress
15
TAC1
BRINP1
NOS1
GCH1
KIAA0319
HTR2C
ADRA2A
ANKFN1
GRIK2
RET
NEUROD2
PENK
GABRA5
RELN
DPP4

GO:BP
GO:0035249
synaptic_transmission__glutamatergic
16
CACNG3
CACNG5
PLAT
SLC17A7
TNR
PLPPR4
GRM4
UNC13C
CACNG8
GRIK2
GRM5
ROR2
RELN
GRM7
FRRS1L
GRIN2B

GO:BP
GO:1902531
regulation_of_intracellular_signal_transduction
94
ITGA3
TENM1
LMO3
F7
RASGRF1
DGKG
PDE4A
NGEF
ALPK1
NUAK1
SEMA3A
MID2
P2RX5
WNT11
SEZ6L
RASD2
PDGFB
MMP9
DOK5
PPP1R16B
SFRP1
TPD52L1
ARFGEF3
SEMA5A
HBEGF
IGFBP5
CHD5
NEK6
TEK
MOB3B
DUSP4
FLT3
TWIST1
IQSEC2
GRM4
PSD4
BMP2
MCF2L
F10
PLEKHG3
FGD3
PPARG
RAP1GAP2
EPHA7
AGAP2
RNASEH2B
MMD2
MYC
CCN3
KIAA0319
ARHGAP20
DUSP5
EGF
DUSP6
DYRK3
TIFA
IGFBP3
HTR2C
ADRA2A
FBXO4
RIT2
GUCY1A2
RCAN1
SPATC1L
GUCY1A1
RET
PRKCB
NPNT
GRM5
ROR2
ADRA1B
SEMA3E
GPR37
ALK
NEUROD2
PRNP
RASGRP1
MYD88
SH3RF3
VEGFB
NPPA
FZD8
AKAP5
TSPYL5
GPR3
ADRA2C
PRKD1
PDE2A
MAPT
RELN
SLC30A10
PDGFA
LPAR1
RASGEF1A

GO:BP
GO:0060078
regulation_of_postsynaptic_membrane_potential
19
GABRA3
P2RX5
NTSR1
CELF4
GABRG2
IGSF11
SNCA
GABRA6
GABRB2
NLGN4X
GABRA2
GRIA1
GABRB1
GRIK2
GRM5
GABRA5
INSYN2A
RELN
GRIN2B

GO:BP
GO:0048878
chemical_homeostasis
63
PNPLA4
PAX6
CYB561
XK
PTPRN
ATP2B3
TRPC7
TRPC5
NOS1
NTSR1
JPH1
SLC17A7
GCK
CDH23
CCKBR
RBP1
IGFBP5
SLC9A2
CNNM1
SLC12A5
VGF
KCNA5
PPARG
GPR12
SCN7A
MYC
HCRTR2
PRKACB
CASQ1
SLC4A10
SNCA
HTR2C
ADRA2A
THY1
GRIA1
SLC24A2
ADCY8
ATP2B2
SLAMF8
ACE
SLC9C2
VSNL1
GRIK2
PRKCB
TMC8
GRM5
IRS1
NPTX1
PRNP
CALB2
ABCG4
KCNJ10
LACC1
RPH3AL
GPR3
PRKD1
SLC24A3
SV2B
ATP13A5
SLC38A3
SLC30A10
RYR2
CEBPA

GO:BP
GO:0099003
vesicle-mediated_transport_in_synapse
24
SYT7
RAB27B
SNCB
RPH3A
SLC17A7
SH3GL2
OTOF
HPCA
PACSIN1
EFNB2
CALY
UNC13C
GRIP2
SNCA
CPLX2
NLGN4X
SYN2
PRKCB
CPLX1
RAB3B
SV2B
LPAR1
CPLX3
DOC2B

GO:BP
GO:0060560
developmental_growth_involved_in_morphogenesis
25
JADE2
FSTL4
TRPC5
SEMA3A
SEMA3C
DCX
WNT11
SALL1
SYT17
SFRP1
LHX2
SH3GL2
SEMA5A
TNR
NRP2
SPP1
CPNE5
EPHA7
KIAA0319
ISLR2
SEMA3E
DSCAM
MAPT
SHTN1
PLXNA4

GO:BP
GO:0045595
regulation_of_cell_differentiation
87
PLXND1
PAX6
PLEKHB1
JADE2
LMO3
FSTL4
CAMK2B
TRPC5
SEMA3A
SEMA3C
BRINP1
DCT
MMP11
PDGFB
MMP9
SFRP1
LHX2
SHB
VSIR
SPOCK2
NEURL1
SEMA5A
LOX
RBP1
HES1
EFEMP1
IGFBP5
TNR
SPP1
TWIST1
EFNB2
BMP2
BCL11B
RAMP2
PPARG
MEIS2
CD101
EPHA7
MMD2
MYC
CCN3
KIAA0319
IGFBP3
HTR2C
BEND6
THY1
PPARGC1B
FAIM
SLAMF8
NBL1
ACE
WNT4
BRINP3
ADAMTS9
HEY1
RET
SPINT1
ISLR2
NPNT
GRM5
ROR2
IL1RAPL1
SEMA3E
ALK
SOSTDC1
NEUROD2
DSCAM
RASGRP1
VWC2L
GSX2
NOG
ADRA2C
PRKD1
ROBO2
MAPT
SHTN1
VWC2
RELN
S100A10
H4C16
MBP
BRINP2
MAFB
DPF3
S1PR3
PLXNA4
CEBPA

GO:BP
GO:0001568
blood_vessel_development
49
PLXND1
PAX6
SEMA3C
RAPGEF3
WNT11
PDGFB
PPP1R16B
SFRP1
SHB
SEMA5A
LOX
NPR3
HES1
PRRX1
NRP2
TEK
TGFBI
EGR1
TWIST1
EFNB2
RHOJ
ANGPTL6
RAMP2
PPARG
CCN3
EGF
HMGA2
THY1
ACE
WNT4
ADAMTS9
HPGD
HEY1
SPINT1
PRKCB
SEMA3E
APLN
SCG2
EGFL7
VEGFB
FUT1
FZD8
ADGRB1
NOG
PRKD1
ROBO2
PDE2A
PDGFA
COL15A1

GO:BP
GO:0001505
regulation_of_neurotransmitter_levels
23
SYT7
CAMK2A
ACHE
RPH3A
NOS1
OTOF
GRM4
SYNGR3
SLC6A11
UNC13C
SNCA
CPLX2
GABRA2
SYN2
PRKCB
CPLX1
RAB3B
KCNJ10
RPH3AL
SV2B
MAOA
CPLX3
DOC2B

GO:BP
GO:0051046
regulation_of_secretion
45
SYT7
RAB27B
PTPRN
ACSL4
CAMK2A
ACHE
RPH3A
CACNA1I
CHGA
NTSR1
SYT17
SFRP1
GCK
SYT10
SPP1
BMP2
KCNA5
PPARG
CHRM3
SYT6
CCN3
EGF
SNCA
CPLX2
HTR2C
ADRA2A
ADCY8
ACE
VSNL1
PRKCB
RAB26
LGI3
CPLX1
IRS1
RAB3B
IL1RAPL1
CDK5R2
PCSK1
RPH3AL
ADRA2C
SV2B
GRM7
S100A10
CPLX3
DOC2B

GO:BP
GO:0031644
regulation_of_nervous_system_process
19
MGLL
CACNG5
NTSR1
CELF4
FGF12
TNR
DLGAP3
CCN3
IGSF11
NLGN4X
HTR2C
ITGA2
NPTX1
BEGAIN
SHISA6
RELN
DLGAP2
SHISA9
GRIN2B

GO:BP
GO:0003012
muscle_system_process
35
CAMK2B
NOS1
MYH7
CHGA
FGF12
IGFBP5
KCNJ5
KCNA5
PPARG
ATP8A2
CHRM3
SCN7A
CASQ1
GRIP2
STAC
SCN2B
ADRA2A
FBXO32
CACNA1D
RNF207
TNNI1
KCNJ3
GUCY1A1
ITGA2
NPNT
ADRA1B
KCND3
VEGFB
NPPA
SSTR2
CHRM2
CTNNA3
ADRA2C
KCNJ12
RYR2

GO:BP
GO:1903530
regulation_of_secretion_by_cell
42
SYT7
RAB27B
ACSL4
CAMK2A
ACHE
RPH3A
CACNA1I
CHGA
NTSR1
SYT17
SFRP1
GCK
SYT10
SPP1
BMP2
KCNA5
PPARG
CHRM3
SYT6
CCN3
SNCA
CPLX2
HTR2C
ADRA2A
ADCY8
VSNL1
PRKCB
RAB26
LGI3
CPLX1
IRS1
RAB3B
IL1RAPL1
CDK5R2
PCSK1
RPH3AL
ADRA2C
SV2B
GRM7
S100A10
CPLX3
DOC2B

GO:BP
GO:0030029
actin_filament-based_process
54
GAS7
TENM1
CAPG
SPTB
NEBL
RAPGEF3
CARMIL1
WNT11
MYH7
RHOV
SFRP1
ARFGEF3
SEMA5A
FGF12
TEK
KCNJ5
WIPF3
IQSEC2
PACSIN1
RHOJ
FGD3
KCNA5
MICAL2
AGAP2
FHDC1
SPTBN5
FGF7
CASQ1
TMEFF2
SCN2B
ADRA2A
PPARGC1B
CACNA1D
RNF207
CSRP1
SPATC1L
SPTBN4
WNT4
KCNJ3
ABLIM2
FAM107A
FRMPD4
SEMA3E
KCND3
SYNPO
CTNNA3
FMNL1
SHTN1
SMTNL2
PDGFA
S100A10
CYRIA
LPAR1
RYR2

GO:BP
GO:1902533
positive_regulation_of_intracellular_signal_transduction
62
TENM1
F7
RASGRF1
ALPK1
SEMA3A
MID2
P2RX5
WNT11
RASD2
PDGFB
DOK5
TPD52L1
SEMA5A
HBEGF
IGFBP5
CHD5
NEK6
TEK
FLT3
GRM4
BMP2
MCF2L
F10
PPARG
AGAP2
MMD2
MYC
KIAA0319
EGF
TIFA
IGFBP3
HTR2C
ADRA2A
RIT2
GUCY1A2
SPATC1L
GUCY1A1
RET
PRKCB
NPNT
GRM5
ROR2
ADRA1B
SEMA3E
GPR37
ALK
NEUROD2
RASGRP1
MYD88
SH3RF3
VEGFB
FZD8
AKAP5
TSPYL5
GPR3
ADRA2C
PRKD1
RELN
SLC30A10
PDGFA
LPAR1
RASGEF1A

GO:BP
GO:0017157
regulation_of_exocytosis
22
SYT7
RAB27B
RPH3A
CACNA1I
SYT17
SYT10
SYT6
SNCA
CPLX2
ADRA2A
VSNL1
PRKCB
RAB26
LGI3
CPLX1
RAB3B
IL1RAPL1
CDK5R2
RPH3AL
SV2B
S100A10
DOC2B

GO:BP
GO:0045055
regulated_exocytosis
24
SYT7
RPH3A
CACNA1I
CHGA
SYT17
SYT10
OTOF
SYNGR3
SYT6
UNC13C
SNCA
CPLX2
ADRA2A
SYN2
PRKCB
RAB26
RAB31
CPLX1
CDK5R2
RASGRP1
RPH3AL
SV2B
CPLX3
DOC2B

GO:BP
GO:0006874
intracellular_calcium_ion_homeostasis
28
XK
ATP2B3
TRPC7
TRPC5
NOS1
NTSR1
JPH1
CDH23
GPR12
HCRTR2
CASQ1
SNCA
HTR2C
THY1
GRIA1
SLC24A2
ADCY8
ATP2B2
GRIK2
PRKCB
GRM5
CALB2
GPR3
PRKD1
SLC24A3
SV2B
ATP13A5
RYR2

GO:BP
GO:0043085
positive_regulation_of_catalytic_activity
67
TENM1
RASGRF1
NGEF
RASGRP2
RAPGEF3
WNT11
NOS1
PDGFB
SFRP1
VSIR
NEURL1
TPD52L1
HBEGF
NPR3
TEK
EGR1
HPCA
FLT3
BMP2
RFPL1
GCH1
PPARG
RAP1GAP2
ENTREP1
EPHA7
AGAP2
PKIB
MMD2
MYC
PLCB2
EGF
SNCA
RASGEF1C
HMGA2
ADRA2A
THY1
PPARGC1B
ADCY8
CACNA1D
ACE
SPATC1L
WNT4
ITGA2
RET
NPNT
GRM5
IRS1
ROR2
PARM1
ALK
CDK5R2
PRNP
RASGRP1
FZD8
AKAP5
RGS6
ADRA2C
PRKD1
MAPT
RELN
PDGFA
S100A10
MBP
LPAR1
RASGEF1A
DENND1B
GRIN2B

GO:BP
GO:0050769
positive_regulation_of_neurogenesis
24
PLXND1
PAX6
JADE2
CAMK2B
TRPC5
DCT
NEURL1
SEMA5A
HES1
BMP2
PPARG
FAIM
ACE
SPINT1
ISLR2
GRM5
IL1RAPL1
DSCAM
GSX2
ROBO2
MAPT
SHTN1
RELN
PLXNA4

GO:BP
GO:0007156
homophilic_cell_adhesion_via_plasma_membrane_adhesion_molecules
20
CDH10
CDH7
CDH20
PCDH11X
CDH23
CDH6
IGSF21
IGSF11
CDH18
CDHR1
KIRREL3
CDH22
CLSTN2
RET
PCDH7
DSCAM
DSCAML1
ROBO2
PTPRT
DCHS2

GO:BP
GO:0016310
phosphorylation
95
MATK
PAX6
CAMK1G
TENM1
CNTN1
CAMK2B
DGKG
CAMK2A
TRPC5
ALPK1
NUAK1
RAPGEF3
STK17B
WNT11
NOS1
PDGFB
MMP9
SFRP1
AMH
GCK
SH3GL2
SHB
NEURL1
TPD52L1
HBEGF
LOX
HES1
EFEMP1
CDK18
NEK6
TEK
MOB3B
EGR1
FLT3
PACSIN1
BMP2
PPP1R1B
PPARG
CMPK2
EPHA7
AGAP2
PKIB
RNASEL
DGKB
MMD2
PARP9
EGF
DUSP6
FGF7
PRKACB
DYRK3
MAP3K21
SNCA
IGFBP3
HMGA2
ADRA2A
PTPRO
RIT2
STK32B
AK5
THY1
PPARGC1B
ADCY8
SLAMF8
ACE
SPATC1L
SPTBN4
CAMK2N1
DIRAS3
DCLK3
CAMKV
RET
PRKCB
GRM5
IRS1
ROR2
ALK
CDK5R2
DSCAM
KNDC1
PRNP
RASGRP1
VEGFB
NPPA
FZD8
BEGAIN
NOG
ADRA2C
PRKD1
INPP5J
MAPT
RELN
PTPRT
PDGFA
CEBPA

GO:BP
GO:0017158
regulation_of_calcium_ion-dependent_exocytosis
10
SYT7
RPH3A
CACNA1I
SYT17
SYT10
SYT6
ADRA2A
CDK5R2
RPH3AL
DOC2B

GO:BP
GO:0098771
inorganic_ion_homeostasis
40
CYB561
XK
ATP2B3
TRPC7
TRPC5
NOS1
NTSR1
JPH1
SLC17A7
CDH23
CNNM1
SLC12A5
KCNA5
GPR12
SCN7A
MYC
HCRTR2
CASQ1
SNCA
HTR2C
THY1
GRIA1
SLC24A2
ADCY8
ATP2B2
ACE
GRIK2
PRKCB
TMC8
GRM5
PRNP
CALB2
KCNJ10
GPR3
PRKD1
SLC24A3
SV2B
ATP13A5
SLC30A10
RYR2

GO:BP
GO:0030003
intracellular_monoatomic_cation_homeostasis
39
CYB561
XK
ATP2B3
TRPC7
TRPC5
NOS1
NTSR1
JPH1
CDH23
SLC9A2
SLC12A5
GPR12
MYC
HCRTR2
CASQ1
SLC4A10
SNCA
HTR2C
THY1
GRIA1
SLC24A2
ADCY8
ATP2B2
SLAMF8
SLC9C2
GRIK2
PRKCB
TMC8
GRM5
PRNP
CALB2
LACC1
GPR3
PRKD1
SLC24A3
SV2B
ATP13A5
SLC30A10
RYR2

GO:BP
GO:1902074
response_to_salt
32
SYT7
F7
RASGRP2
P2RX5
ACHE
PDGFB
MAP1LC3A
SYT17
SYT10
RBP1
EGR1
HPCA
CPNE5
PPP1R1B
CHRM3
SYT6
SLC13A5
ADCY8
ELAVL4
ITGA2
PRKCB
NPTX1
NEUROD2
PCSK1
LY6H
KCNJ10
CPNE7
CHRM2
PENK
CPNE4
RYR2
CEBPA

GO:BP
GO:0099601
regulation_of_neurotransmitter_receptor_activity
12
CACNG3
RASGRF1
CACNG5
DLGAP3
CACNG8
NPTX1
LYNX1
BEGAIN
SHISA6
RELN
DLGAP2
SHISA9

GO:BP
GO:0009967
positive_regulation_of_signal_transduction
84
TENM1
LMO3
F7
RASGRF1
CAMK2A
ALPK1
SEMA3A
MID2
P2RX5
WNT11
RASD2
PDGFB
CHGA
MMP9
DOK5
RSPO4
SALL1
SFRP1
NEURL1
IL10RA
TPD52L1
SEMA5A
HBEGF
HES1
IGFBP5
PRRX1
CHD5
NEK6
TEK
FLT3
GRM4
BMP2
MCF2L
F10
PPARG
AGAP2
MMD2
MYC
CCN3
KIAA0319
DDX60
PARP9
EGF
IGSF11
OCIAD2
TIFA
IGFBP3
HTR2C
ADRA2A
RIT2
GUCY1A2
SCUBE1
SPATC1L
GUCY1A1
RET
PRKCB
NPNT
GRM5
IRS1
ROR2
ADRA1B
SEMA3E
GPR37
ALK
NEUROD2
RASGRP1
MYD88
SH3RF3
VEGFB
FZD8
AKAP5
TSPYL5
GSX2
GPR3
ADRA2C
PRKD1
ROBO2
RELN
MAOA
SLC30A10
PDGFA
LPAR1
RASGEF1A
GRIN2B

GO:BP
GO:0099565
chemical_synaptic_transmission__postsynaptic
17
GABRA3
P2RX5
NTSR1
CELF4
GABRG2
IGSF11
SNCA
GABRA6
GABRB2
NLGN4X
GABRA2
GABRB1
GRIK2
GABRA5
INSYN2A
RELN
GRIN2B

GO:BP
GO:0006873
intracellular_monoatomic_ion_homeostasis
39
CYB561
XK
ATP2B3
TRPC7
TRPC5
NOS1
NTSR1
JPH1
CDH23
SLC9A2
SLC12A5
GPR12
MYC
HCRTR2
CASQ1
SLC4A10
SNCA
HTR2C
THY1
GRIA1
SLC24A2
ADCY8
ATP2B2
SLAMF8
SLC9C2
GRIK2
PRKCB
TMC8
GRM5
PRNP
CALB2
LACC1
GPR3
PRKD1
SLC24A3
SV2B
ATP13A5
SLC30A10
RYR2

GO:BP
GO:0016049
cell_growth
38
TNC
JADE2
FSTL4
KIF26A
ACSL4
RASGRP2
TRPC5
SEMA3A
SEMA3C
DCX
WNT11
SYT17
SFRP1
LHX2
SH3GL2
LGI1
SEMA5A
HBEGF
IGFBP5
TNR
PAPPA2
NRP2
SPP1
CPNE5
PPARG
RERG
EPHA7
CCN3
KIAA0319
IGFBP3
SPOCK1
ISLR2
FAM107A
SEMA3E
DSCAM
MAPT
SHTN1
PLXNA4

GO:BP
GO:0017156
calcium-ion_regulated_exocytosis
12
SYT7
RPH3A
CACNA1I
SYT17
SYT10
SYT6
UNC13C
ADRA2A
SYN2
CDK5R2
RPH3AL
DOC2B

GO:BP
GO:0021536
diencephalon_development
13
PAX6
SALL1
SEMA5A
HES1
NRP2
BMP2
WNT4
PTCHD1
KCNC2
SEMA3E
CNTNAP2
PCSK1
NOG

GO:BP
GO:0016358
dendrite_development
24
FSTL4
CAMK2B
DGKG
LZTS1
NGEF
CAMK2A
TRPC5
SEMA3A
DCX
NEURL1
SLC12A5
PACSIN1
KIAA0319
NTN3
ELAVL4
IL1RAPL1
GPR37
TANC2
ALK
DSCAM
KNDC1
RELN
LPAR1
FOXO6

GO:BP
GO:0010959
regulation_of_metal_ion_transport
33
CNTN1
CAMK2B
CAMK2A
P2RX5
NOS1
PDGFB
NTSR1
JPH1
GCK
FGF12
HES1
HPCA
KCNA5
EGF
CASQ1
STAC
SNCA
SCN2B
ADRA2A
THY1
CACNA1D
CABP1
RNF207
ACE
SPTBN4
KCNC2
PRNP
NPPA
AKAP5
PRKD1
NKAIN2
SLC30A10
RYR2

GO:BP
GO:0019932
second-messenger-mediated_signaling
28
ADCY2
RAPGEF3
P2RX5
NOS1
RASD2
RASD1
RCAN3
HPCA
CHRM3
CASQ1
HTR2C
RIT2
GUCY1A2
RCAN1
VCAM1
GUCY1A1
KCNC2
GRM5
NEUROD2
PRNP
NPPA
AKAP5
GPR3
PDE2A
MAPT
LPAR1
RYR2
GRIN2B

GO:BP
GO:0006941
striated_muscle_contraction
20
NOS1
MYH7
CHGA
FGF12
KCNJ5
KCNA5
ATP8A2
CASQ1
STAC
SCN2B
CACNA1D
RNF207
TNNI1
KCNJ3
ADRA1B
KCND3
VEGFB
NPPA
CTNNA3
RYR2

GO:BP
GO:0001944
vasculature_development
49
PLXND1
PAX6
SEMA3C
RAPGEF3
WNT11
PDGFB
PPP1R16B
SFRP1
SHB
SEMA5A
LOX
NPR3
HES1
PRRX1
NRP2
TEK
TGFBI
EGR1
TWIST1
EFNB2
RHOJ
ANGPTL6
RAMP2
PPARG
CCN3
EGF
HMGA2
THY1
ACE
WNT4
ADAMTS9
HPGD
HEY1
SPINT1
PRKCB
SEMA3E
APLN
SCG2
EGFL7
VEGFB
FUT1
FZD8
ADGRB1
NOG
PRKD1
ROBO2
PDE2A
PDGFA
COL15A1

GO:BP
GO:0051966
regulation_of_synaptic_transmission__glutamatergic
13
CACNG3
CACNG5
TNR
PLPPR4
GRM4
CACNG8
GRIK2
GRM5
ROR2
RELN
GRM7
FRRS1L
GRIN2B

GO:BP
GO:0006935
chemotaxis
44
PLXND1
ETV1
PAX6
CNTN1
F7
SEMA3A
SEMA3C
PDGFB
CHGA
LHX2
LGI1
SEMA5A
HBEGF
LOX
UNC5A
TNR
NRP2
EFNB2
BCL11B
EPHA7
CCN3
FGF7
NFIB
PTPRO
SLAMF8
NBL1
NTN3
VCAM1
ITGA2
RET
SEMA3E
CDK5R2
DSCAM
SCG2
VEGFB
DSCAML1
NOG
PRKD1
ROBO2
RELN
PDGFA
DPP4
LPAR1
PLXNA4

GO:BP
GO:0007169
transmembrane_receptor_protein_tyrosine_kinase_signaling_pathway
44

FSTL4
F7
NGEF
PDGFB
MMP9
DOK5
PLAT
NEURL1
HBEGF
LOX
FGF12
EFEMP1
IGFBP5
ANGPTL1
NRP2
TEK
FLT3
EFNB2
BMP2
EPHA7
EGF
FGF7
SNCA
IGFBP3
ADRA2A
SLA
WNT4
RET
PRKCB
GFRA2
IRS1
ROR2
REPS2
ALK
APLN
VEGFB
SHISA2
NOG
PRKD1
COL4A5
PTPRT
SLC30A10
PDGFA
COL4A6

GO:BP
GO:0045597
positive_regulation_of_cell_differentiation
54

PLXND1
PAX6
JADE2
LMO3
CAMK2B
TRPC5
BRINP1
DCT
SFRP1
SHB
VSIR
NEURL1
SEMA5A
HES1
TWIST1
EFNB2
BMP2
RAMP2
PPARG
CD101
MMD2
IGFBP3
HTR2C
BEND6
PPARGC1B
FAIM
NBL1
ACE
WNT4
BRINP3
ADAMTS9
RET
SPINT1
ISLR2
NPNT
GRM5
ROR2
IL1RAPL1
NEUROD2
DSCAM
RASGRP1
VWC2L
GSX2
ADRA2C
PRKD1
ROBO2
MAPT
SHTN1
VWC2
RELN
BRINP2
DPF3
PLXNA4
CEBPA

GO:BP
GO:0042330
taxis
44

PLXND1
ETV1
PAX6
CNTN1
F7
SEMA3A
SEMA3C
PDGFB
CHGA
LHX2
LGI1
SEMA5A
HBEGF
LOX
UNC5A
TNR
NRP2
EFNB2
BCL11B
EPHA7
CCN3
FGF7
NFIB
PTPRO
SLAMF8
NBL1
NTN3
VCAM1
ITGA2
RET
SEMA3E
CDK5R2
DSCAM
SCG2
VEGFB
DSCAML1
NOG
PRKD1
ROBO2
RELN
PDGFA
DPP4
LPAR1
PLXNA4

GO:BP
GO:0009628
response_to_abiotic_stimulus
67

TAC1
ETV1
PLEKHB1
F7
CNGB1
DCT
ABCB1
WNT11
NOS1
MMP9
NTSR1
SFRP1
PLAT
EFHD1
PAPPA2
PPL
EGR1
HPCA
TWIST1
PHF24
SLC12A5
BMP2
VGF
KCNA5
PPP1R1B
PPARG
ATP8A2
MEIS2
SCN2A
MYC
KIAA0319
CASQ1
SLC4A10
STAC
SCN2B
CACNA2D4
FBXO4
GUCY1A2
SLC24A2
SST
TSC22D3
ACE
VCAM1
KCNJ3
GUCY1A1
ITGA2
GRIK2
KCNC2
NEUROD2
MYD88
ARPP21
VEGFB
CNTNAP2
NPPA
TSPYL5
PENK
GPR88
KCNK4
NOG
SLITRK6
PDE2A
MAPT
SLC38A3
PDGFA
DPP4
LPAR1
RYR2

GO:BP
GO:0072359
circulatory_system_development
66

PLXND1
ITGA3
PAX6
SEMA3C
NEBL
RAPGEF3
WNT11
MYH7
PDGFB
PPP1R16B
SALL1
SFRP1
DNAH11
SHB
SEMA5A
LOX
NPR3
FGF12
HES1
PRRX1
NRP2
TEK
TGFBI
EGR1
TWIST1
EFNB2
BMP2
RHOJ
ANGPTL6
RAMP2
PPARG
MICAL2
CCN3
EGF
DUSP6
HMGA2
THY1
ADAMTS5
RNF207
TNNI1
SCUBE1
ACE
WNT4
VCAM1
ADAMTS9
HPGD
HEY1
SPINT1
PRKCB
SEMA3E
APLN
SCG2
EGFL7
VEGFB
FUT1
NPPA
FZD8
ADGRB1
NOG
PRKD1
ROBO2
PDE2A
PDGFA
RYR2
COL15A1
PLXNA4

GO:BP
GO:0070887
cellular_response_to_chemical_stimulus
130

PAX6
SYT7
LMO3
PTPRN
F7
PDE4A
RASGRP2
CAMK2A
IPCEF1
ADCY2
BRINP1
RAPGEF3
COL16A1
ABCB1
WNT11
ACHE
NOS1
HCN2
PDGFB
CHGA
MMP9
SAMHD1
MAP1LC3A
SYT17
SFRP1
PLAT
CRHR2
GCK
SPOCK2
NEURL1
IL10RA
SYT10
SEMA5A
HBEGF
LOX
GABRG2
HES1
IGFBP5
EFHD1
NRP2
SPP1
EGR1
HPCA
FLT3
TWIST1
CPNE5
EFNB2
BMP2
IRF5
CALY
ASS1
RAMP2
PPP1R1B
GCH1
PPARG
CHRM3
POSTN
SYT6
CMPK2
ENTREP1
LMO2
AGAP2
SCN2A
MYC
CCN3
PARP9
SSTR1
SLC13A5
SNCA
GABRB2
HTR2C
ADRA2A
PTPRO
GRIA1
PPARGC1B
ADCY8
FBXO32
SST
HTR5A
MX1
SLAMF8
NBL1
ACE
BRINP3
VCAM1
VSNL1
GABRB1
ITGA2
RET
KCNC2
PRKCB
GNG8
FAM107A
RAB31
NPNT
GRM5
IRS1
GPR37
ALK
NPTX1
NEUROD2
PRNP
SCG2
ABCG4
MYD88
VEGFB
LY6H
KCNJ10
CPNE7
SSTR2
CHRM2
PENK
KCNK4
EFHC2
PRKD1
ROBO2
PDE2A
MAPT
IL1RAPL2
PTPRT
CPNE4
SLC30A10
COL4A6
DPP4
LPAR1
RYR2
BRINP2
PLXNA4
CEBPA
SRXN1

GO:BP
GO:0001558
regulation_of_cell_growth
34

TNC
JADE2
FSTL4
KIF26A
ACSL4
RASGRP2
TRPC5
SEMA3A
SEMA3C
WNT11
SYT17
SFRP1
LGI1
SEMA5A
HBEGF
IGFBP5
TNR
PAPPA2
SPP1
CPNE5
PPARG
RERG
EPHA7
CCN3
KIAA0319
IGFBP3
SPOCK1
ISLR2
FAM107A
SEMA3E
DSCAM
MAPT
SHTN1
PLXNA4

GO:BP
GO:1901888
regulation_of_cell_junction_assembly
22

LZTS1
COL16A1
SFRP1
TEK
EPHA7
SNCA
THY1
CLSTN2
ACE
WNT4
LRRTM1
FAM107A
IL1RAPL1
CNTNAP2
SLITRK4
ADGRB1
GPC6
SLITRK6
ROBO2
IL1RAPL2
S100A10
LRRTM3

GO:BP
GO:0051050
positive_regulation_of_transport
57

CACNG3
TENM1
SYT7
CNTN1
RAB27B
ACSL4
CAMK2A
RAPGEF3
P2RX5
ABCB1
ACHE
NOS1
PDGFB
CACNA1I
NTSR1
GCK
SYT10
FGF12
SPP1
HPCA
BMP2
SYNGR3
CALY
PPARG
ATP8A2
CHRM3
RNASEL
EGF
CASQ1
STAC
SNCA
ADRA2A
THY1
ADCY8
CACNA1D
RNF207
VSNL1
ITGA2
KCNC2
PRKCB
RAB31
IRS1
RAB3B
APLN
CDK5R2
PRNP
ABCG4
NPPA
PCSK1
AKAP5
RPH3AL
PRKD1
SLC38A3
RELN
S100A10
RYR2
DOC2B

GO:BP
GO:0060048
cardiac_muscle_contraction
17

NOS1
MYH7
CHGA
FGF12
KCNJ5
KCNA5
SCN2B
CACNA1D
RNF207
TNNI1
KCNJ3
ADRA1B
KCND3
VEGFB
NPPA
CTNNA3
RYR2

GO:BP
GO:0098657
import_into_cell
24

NOS1
HCN2
NTSR1
IL10RA
SLC9A2
PLPPR4
KCNJ5
SLC12A5
SYNGR3
SLC6A11
SNCA
SLC24A2
CACNA1D
KCNJ9
SLC9C2
KCNJ3
RAB3B
KCNK9
PRNP
KCNJ10
AKAP5
KCNJ12
SLC38A3
CACNA1E

GO:BP
GO:0050919
negative_chemotaxis
10

SEMA3A
SEMA3C
SEMA5A
NRP2
EPHA7
SEMA3E
ROBO2
PDGFA
DPP4
PLXNA4

GO:BP
GO:0042325
regulation_of_phosphorylation
68

PAX6
TENM1
CNTN1
TRPC5
RAPGEF3
NOS1
PDGFB
MMP9
SFRP1
SH3GL2
SHB
NEURL1
TP53INP1
HBEGF
LOX
HES1
TEK
MOB3B
EGR1
FLT3
BMP2
PPP1R1B
PPARG
EPHA7
AGAP2
PKIB
MMD2
PARP9
EGF
DUSP6
FGF7
SNCA
IGFBP3
HMGA2
ADRA2A
PTPRO
RIT2
THY1
PPARGC1B
ADCY8
SLAMF8
ACE
SPATC1L
SPTBN4
CAMK2N1
DIRAS3
RET
GRM5
IRS1
ROR2
ALK
CDK5R2
DSCAM
KNDC1
PRNP
RASGRP1
VEGFB
NPPA
FZD8
NOG
ADRA2C
PRKD1
INPP5J
MAPT
RELN
PTPRT
PDGFA
CEBPA

GO:BP
GO:0072001
renal_system_development
28

PLXND1
ITGA3
WNT11
PDGFB
MMP9
SALL1
SFRP1
HES1
TEK
EGR1
EFNB2
BMP2
ASS1
EPHA7
MYC
KIRREL3
PTPRO
ACE
WNT4
HPGD
RET
NPNT
EMX2
NOG
ROBO2
GCNT1
DCHS2
PDGFA

GO:BP
GO:0030198
extracellular_matrix_organization
28

COL19A1
COL16A1
COL9A3
MMP11
MMP9
SPOCK2
LOX
TNR
TGFBI
BMP2
RAMP2
MATN2
POSTN
ADAMTS8
ADAMTS19
ADAMTS5
MPZL3
ADAMTS9
ADAMTS15
SPINT1
NPNT
ADAMTSL5
COL4A5
LAMB3
COL4A6
DPP4
ADAMTSL2
COL15A1

GO:BP
GO:0007613
memory
16

ITGA3
RASGRF1
BRINP1
SCN2A
GRIA1
SLC24A2
ADCY8
SORCS3
RCAN1
CAMK2N1
PTCHD1
PRNP
KCNK4
MAPT
RELN
FOXD6

GO:BP
GO:0051961
negative_regulation_of_nervous_system_development
18

PAX6
FSTL4
TRPC5
SEMA3A
SEMA3C
BRINP1
LHX2
SEMA5A
HES1
TNR
SPP1
EPHA7
KIAA0319
THY1
SEMA3E
NOG
ROBO2
MBP

GO:BP
GO:0043062
extracellular_structure_organization
28

COL19A1
COL16A1
COL9A3
MMP11
MMP9
SPOCK2
LOX
TNR
TGFBI
BMP2
RAMP2
MATN2
POSTN
ADAMTS8
ADAMTS19
ADAMTS5
MPZL3
ADAMTS9
ADAMTS15
SPINT1
NPNT
ADAMTSL5
COL4A5
LAMB3
COL4A6
DPP4
ADAMTSL2
COL15A1

GO:BP
GO:0006468
protein_phosphorylation
83

MATK
PAX6
CAMK1G
TENM1
CNTN1
CAMK2B
CAMK2A
TRPC5
ALPK1
NUAK1
RAPGEF3
STK17B
WNT11
NOS1
PDGFB
MMP9
SFRP1
AMH
SH3GL2
SHB
NEURL1
TP53INP1
HBEGF
LOX
HES1
EFEMP1
CDK18
NEK6
TEK
MOB3B
EGR1
FLT3
BMP2
PPP1R1B
PPARG
EPHA7
AGAP2
PKIB
RNASEL
MMD2
PARP9
EGF
DUSP6
FGF7
PRKACB
DYRK3
MAP3K21
SNCA
IGFBP3
HMGA2
ADRA2A
PTPRO
RIT2
STK32B
THY1
ADCY8
ACE
SPATC1L
SPTBN4
CAMK2N1
DIRAS3
DCLK3
CAMKV
RET
PRKCB
GRM5
ROR2
ALK
CDK5R2
KNDC1
PRNP
RASGRP1
VEGFB
NPPA
FZD8
NOG
ADRA2C
PRKD1
INPP5J
RELN
PTPRT
PDGFA
CEBPA

GO:BP
GO:0055074
calcium_ion_homeostasis
28

XK
ATP2B3
TRPC7
TRPC5
NOS1
NTSR1
JPH1
CDH23
GPR12
HCRTR2
CASQ1
SNCA
HTR2C
THY1
GRIA1
SLC24A2
ADCY8
ATP2B2
GRIK2
PRKCB
GRM5
CALB2
GPR3
PRKD1
SLC24A3
SV2B
ATP13A5
RYR2

GO:BP
GO:0045229
external_encapsulating_structure_organization
28

COL19A1
COL16A1
COL9A3
MMP11
MMP9
SPOCK2
LOX
TNR
TGFBI
BMP2
RAMP2
MATN2
POSTN
ADAMTS8
ADAMTS19
ADAMTS5
MPZL3
ADAMTS9
ADAMTS15
SPINT1
NPNT
ADAMTSL5
COL4A5
LAMB3
COL4A6
DPP4
ADAMTSL2
COL15A1

GO:BP
GO:0014070
response_to_organic_cyclic_compound
57

LMO3
F7
PTPRU
ADCY2
RAPGEF3
P2RX5
HCN2
PDGFB
MAP1LC3A
SFRP1
PLAT
AMH
LOX
GABRG2
RBP1
HES1
IGFBP5
SPP1
EGR1
HPCA
FLT3
BMP2
VGF
CALY
ASS1
PPP1R1B
PPARG
CHRM3
DUSP6
SSTR1
SNCA
GABRB2
HTR2C
GNRH1
PPARGC1B
ADCY8
FBXO32
SST
HTR5A
ACE
ELAVL4
GABRB1
HPGD
ITGA2
KCNC2
FAM107A
ALK
MYD88
PCSK1
SSTR2
CHRM2
PENK
PDE2A
PDGFA
MBP
RYR2
CEBPA

GO:BP
GO:0031623
receptor_internalization
16

CACNG3
CACNG5
ACHE
HPCA
EFNB2
CALY
RAMP2
ENTREP1
EGF
CACNG8
SNCA
GRIA1
LRRTM1
RAB31
APLN
LPAR1

GO:BP
GO:0009887
animal_organ_morphogenesis
63

PLXND1
PAX6
TNC
TLE2
SEMA3C
WNT11
MYH7
SALL1
SFRP1
DNAH11
CDH23
MGP
HES1
EFEMP1
IGFBP5
PRRX1
PAPPA2
NRP2
TEK
TWIST1
EFNB2
BMP2
BCL11B
ATP8A2
MICAL2
MEIS2
MYC
LRIG3
FGF7
SLC4A10
TMEFF2
NFIB
THY1
ADAMTS5
PPARGC1B
RNF207
NBL1
TNNI1
NTN3
WNT4
ITGA2
HEY1
FZD6
NPNT
ROR2
EMX2
SOSTDC1
DSCAM
LY6H
DSCAML1
FJX1
TSHZ1
GSX2
GPC6
NOG
SLITRK6
ROBO2
GCNT1
LAMB3
PDGFA
RYR2
RIPPLY2
MAFB

GO:BP
GO:0061387
regulation_of_extent_of_cell_growth
15

FSTL4
TRPC5
SEMA3A
SEMA3C
SEMA5A
TNR
SPP1
EPHA7
KIAA0319
ISLR2
SEMA3E
DSCAM
MAPT
SHTN1
PLXNA4

GO:BP
GO:0006796
phosphate-containing_compound_metabolic_process
130

MATK
PAX6
CAMK1G
TENM1
CNTN1
PTPRN
CAMK2B
DGKG
PTPRU
PDE4A
ACSL4
CAMK2A
TRPC5
ALPK1
NUAK1
ADCY2
RAPGEF3
STK17B
WNT11
NOS1
PITPNM2
MYH7
PDGFB
MMP9
NTSR1
SAMHD1
PPP1R16B
SFRP1
AMH
PPP1R17
GCK
SH3GL2
SHB
NEURL1
PTPN5
TP53INP1
HBEGF
LOX
HES1
EFEMP1
CDK18
PLPPR5
PLPPR4
NEK6
ACOT2
TEK
MOB3B
EGR1
DUSP4
HPCA
FLT3
PACSIN1
BMP2
PLAAT1
PUDP
PPP1R1B
GCH1
PPARG
CMPK2
EPHA7
AGAP2
PKIB
RNASEL
RNASEH2B
DGKB
MMD2
PLCB2
DUSP5
PARP9
EGF
DUSP6
FGF7
PRKACB
DYRK3
MAP3K21
SNCA
IGFBP3
HTR2C
PLPPR1
PLCH2
HMGA2
ADRA2A
PTPRO
RIT2
GUCY1A2
STK32B
AK5
THY1
PPARGC1B
ADCY8
SLAMF8
DUSP23
RCAN1
ACE
SPATC1L
SPTBN4
CAMK2N1
DIRAS3
DCLK3
CAMKV
GUCY1A1
ITGA2
RET
PRKCB
NPNT
GRM5
IRS1
ROR2
ALK
CDK5R2
DSCAM
KNDC1
PRNP
RASGRP1
VEGFB
NPPA
FZD8
LACC1
BEGAIN
NOG
ADRA2C
ACOT1
PRKD1
INPP5J
PDE2A
MAPT
RELN
PTPRT
PDGFA
CEBPA

GO:BP
GO:0042221
response_to_chemical
180

PLXND1
ITGA3
ETV1
PAX6
SYT7
CNTN1
LMO3
PTPRN
F7
PTPRU
PDE4A
RASGRP2
CNGB1
CAMK2A
IPCEF1
SEMA3A
SEMA3C
ADCY2
BRINP1
RAPGEF3
P2RX5
COL16A1
ABCB1
WNT11
ACHE
NOS1
HCN2
PDGFB
CHGA
MMP9
NTSR1
SAMHD1
MAP1LC3A
SYT17
SFRP1
PLAT
AMH
CRHR2
GCK
LHX2
SPOCK2
NEURL1
LGI1
IL10RA
SYT10
SEMA5A
HBEGF
LOX
GABRG2
UNC5A
RBP1
HES1
IGFBP5
EFHD1
TNR
NRP2
SPP1
EGR1
HPCA
FLT3
TWIST1
SLC12A5
CPNE5
EFNB2
BMP2
BCL11B
VGF
IRF5
KCNA5
CALY
ASS1
SHFL
F12
RAMP2
PPP1R1B
GCH1
SLC6A11
PPARG
CHRM3
POSTN
SYT6
CMPK2
RERG
ENTREP1
EPHA7
LMO2
AGAP2
SCN2A
MYC
CCN3
PLCB2
PARP9
DUSP6
SSTR1
FGF7
SLC13A5
CASQ1
SNCA
GABRB2
HTR2C
GNRH1
NFIB
SCN2B
ADRA2A
PTPRO
GRIA1
PPARGC1B
ADCY8
FBXO32
SST
HTR5A
MX1
SLAMF8
NBL1
ACE
FBXO27
NTN3
ELAVL4
BRINP3
VCAM1
VSNL1
GABRB1
HPGD
ITGA2
RET
KCNC2
PRKCB
GNG8
FAM107A
RAB31
NPNT
GRM5
IRS1
EMX2
SEMA3E
GPR37
ALK
NPTX1
CDK5R2
NEUROD2
DSCAM
PRNP
SCG2
ABCG4
MYD88
VEGFB
PCSK1
LY6H
DSCAML1
KCNJ10
CPNE7
SSTR2
CHRM2
PENK
KCNK4
EFHC2
NOG
PRKD1
ROBO2
PDE2A
MAPT
GCNT1
RELN
IL1RAPL2
PTPRT
CPNE4
SLC30A10
PDGFA
COL4A6
DPP4
MBP
LPAR1
RYR2
BRINP2
PLXNA4
RAB6C
OR2W3
CEBPA
SRXN1
GRIN2B

GO:BP
GO:0035640
exploration_behavior
8

ITGA3
BRINP1
TNR
SLC4A10
LRRTM1
PENK
NOG
DPP4

GO:BP
GO:0086002
cardiac_muscle_cell_action_potential_involved_in_contraction
10

FGF12
KCNJ5
KCNA5
SCN2B
CACNA1D
RNF207
KCNJ3
KCND3
CTNNA3
RYR2

GO:BP
GO:0001822
kidney_development
27

PLXND1
ITGA3
WNT11
PDGFB
MMP9
SALL1
SFRP1
HES1
TEK
EGR1
EFNB2
BMP2
ASS1
EPHA7
MYC
KIRREL3
PTPRO
ACE
WNT4
HPGD
RET
NPNT
NOG
ROBO2
GCNT1
DCHS2
PDGFA

GO:BP
GO:0048729
tissue_morphogenesis
43

PLXND1
ITGA3
TNC
SEMA3C
WNT11
MYH7
SALL1
SFRP1
LHX2
HBEGF
HES1
IGFBP5
NRP2
TWIST1
EFNB2
BMP2
MICAL2
EPHA7
MYC
EGF
FGF7
PRKACB
TMEFF2
HMGA2
ADAMTS5
RNF207
TNNI1
WNT4
ITGA2
HEY1
FZD6
RET
SPINT1
NPNT
ROR2
SEMA3E
SOSTDC1
GPC6
NOG
ROBO2
GCNT1
PDGFA
RYR2

GO:BP
GO:0055082
intracellular_chemical_homeostasis
46

CYB561
XK
PTPRN
ATP2B3
TRPC7
TRPC5
NOS1
NTSR1
JPH1
GCK
CDH23
SLC9A2
SLC12A5
GPR12
MYC
HCRTR2
CASQ1
SLC4A10
SNCA
HTR2C
ADRA2A
THY1
GRIA1
SLC24A2
ADCY8
ATP2B2
SLAMF8
ACE
SLC9C2
VSNL1
GRIK2
PRKCB
TMC8
GRM5
NPTX1
PRNP
CALB2
LACC1
GPR3
PRKD1
SLC24A3
SV2B
ATP13A5
SLC38A3
SLC30A10
RYR2

GO:BP
GO:0098962
regulation_of_postsynaptic_neurotransmitter_receptor_activity
7

CACNG5
DLGAP3
NPTX1
BEGAIN
SHISA6
DLGAP2
SHISA9

GO:BP
GO:0050790
regulation_of_catalytic_activity
94

[illegible]

GO:BP
GO:0060627
regulation_of_vesicle-mediated_transport
38

SYT7
RAB27B
RPH3A
CACNA1I
SYT17
SLC17A7
SYT10
HPCA
PACSIN1
EFNB2
CALY
PPARG
SYT6
EGF
SNCA
CPLX2
ADRA2A
RIT2
LRRTM1
VSNL1
ITGA2
PRKCB
RAB26
RAB31
LGI3
CPLX1
RAB3B
IL1RAPL1
APLN
CDK5R2
AKAP5
RPH3AL
PRKD1
SV2B
S100A10
LPAR1
CPLX3
DOC2B

GO:BP
GO:0031346
positive_regulation_of_cell_projection_organization
29

PLXND1
ITGA3
TENM1
CNTN1
CAMK2B
TRPC5
CARMIL1
NEURL1
SEMA5A
PLPPR5
PACSIN1
ATP8A2
RIT2
ELAVL4
SERPINI1
ITGA2
RET
ISLR2
ROR2
IL1RAPL1
ALK
DSCAM
FUT9
PRKD1
ROBO2
MAPT
SHTN1
RELN
PLXNA4

GO:BP
GO:0048015
phosphatidylinositol-mediated_signaling
19

MYO16
PITPNM2
PDGFB
PPP1R16B
NPR3
TEK
FLT3
TWIST1
AGAP2
PLCB2
EGF
NYAP2
PLCH2
IRS1
ROR2
SEMA3E
RASGRP1
RELN
PDGFA

GO:BP
GO:0048514
blood_vessel_morphogenesis
42

PLXND1
RAPGEF3
WNT11
PPP1R16B
SFRP1
SHB
SEMA5A
LOX
NPR3
HES1
PRRX1
NRP2
TEK
TGFBI
TWIST1
EFNB2
RHOJ
ANGPTL6
RAMP2
PPARG
CCN3
EGF
HMGA2
THY1
ACE
WNT4
ADAMTS9
HPGD
HEY1
PRKCB
SEMA3E
APLN
SCG2
EGFL7
VEGFB
FUT1
FZD8
ADGRB1
NOG
PRKD1
PDGFA
COL15A1

GO:CC
GO:0045202
synapse
172

[illegible]

GO:CC
GO:0071944
cell_periphery
376

[illegible]

GO:CC
GO:0030054
cell_junction
200

[illegible]

GO:CC
GO:0043005
neuron_projection
142

[illegible]

GO:CC
GO:0005886
plasma_membrane
339

[illegible]

GO:CC
GO:0098794
postsynapse
88

[illegible]

GO:CC
GO:0042995
cell_projection
180

[illegible]

GO:CC
GO:0036477
somatodendritic_compartment
98

[illegible]

GO:CC
GO:0120025
plasma_membrane_bounded_cell_projection
169

[illegible]

GO:CC
GO:0098978
glutamatergic_synapse
64

[illegible]

GO:CC
GO:0016020
membrane
447

[illegible]

GO:CC
GO:0097060
synaptic_membrane
59

[illegible]

GO:CC
GO:0097447
dendritic_tree
75

[illegible]

GO:CC
GO:0098793
presynapse
70

[illegible]

GO:CC
GO:0030425
dendrite
74

[illegible]

GO:CC
GO:0045211
postsynaptic_membrane
48

[illegible]

GO:CC
GO:0099572
postsynaptic_specialization
52

[illegible]

GO:CC
GO:0030424
axon
68

[illegible]

GO:CC
GO:0044297
cell_body
63

[illegible]

GO:CC
GO:0098984
neuron_to_neuron_synapse
50

[illegible]

GO:CC
GO:0043025
neuronal_cell_body
58

[illegible]

GO:CC
GO:0098590
plasma_membrane_region
101

[illegible]

GO:CC
GO:0099634
postsynaptic_specialization_membrane
28

CACNG3
GABRA3
CDH10
CACNG5
GABRG2
PLPPR4
EFNB2
EPHA7
CACNG8
GABRB2
NLGN4X
ADRA2A
PTPRO
GABRA2
GRIA1
SORCS3
ATP2B2
HTR5A
CLSTN2
LRRTM1
GABRB1
GRM5
KCND3
GABRA5
SHISA6
LRRTM3
SHISA9
GRIN2B

GO:CC
GO:0032279
asymmetric_synapse
46

CACNG3
LZTS1
CAMK2A
CACNG5
NOS1
NEURL1
DLGAP3
PLPPR4
EFNB2
EPHA7
CACNG8
GRIP2
IGSF11
NLGN4X
ADRA2A
PTPRO
SPOCK1
GRIA1
ADCY8
SORCS3
ATP2B2
SYN2
CABP1
CLSTN2
CAMK2N1
GRIK2
BAALC
GRM5
FRMPD4
TANC2
KCND3
PRNP
SYNPO
MYD88
AKAP5
CHRM2
ADGRB1
SHISA6
INSYN2A
GRM7
ADGRA1
DLGAP2
LRRTM3
PLXNA4
SHISA9
GRIN2B

GO:CC
GO:0034702
monoatomic_ion_channel_complex
42

CACNG3
GABRA3
TRPC7
CNGB1
TRPC5
CACNG5
HCN2
CACNA1I
SLC17A7
GABRG2
KCNJ5
BEST3
KCNA5
SCN2A
SCN7A
CACNG8
GABRA6
GABRB2
SCN2B
CACNA2D4
KCNA6
GABRA2
GRIA1
CACNA1D
KCNF1
KCNJ3
GABRB1
GRIK2
KCNC2
KCND3
VWC2L
CNTNAP2
KCNA3
KCNK4
KCNQ5
GABRA5
VWC2
SHISA6
CACNA1E
RYR2
SHISA9
GRIN2B

GO:CC
GO:0014069
postsynaptic_density
43

CACNG3
LZTS1
CAMK2A
CACNG5
NOS1
NEURL1
DLGAP3
PLPPR4
EFNB2
EPHA7
CACNG8
GRIP2
IGSF11
NLGN4X
ADRA2A
PTPRO
SPOCK1
GRIA1
ADCY8
SORCS3
ATP2B2
SYN2
CABP1
CLSTN2
CAMK2N1
GRIK2
BAALC
GRM5
FRMPD4
TANC2
KCND3
PRNP
SYNPO
MYD88
AKAP5
ADGRB1
SHISA6
INSYN2A
ADGRA1
DLGAP2
LRRTM3
SHISA9
GRIN2B

GO:CC
GO:0032589
neuron_projection_membrane
18

GABRA3
GABRG2
UNC5A
HPCA
SLC12A5
GABRA6
GABRA2
THY1
GRIA1
ATP2B2
KCNC2
CNTNAP2
AKAP5
ROBO2
GABRA5
MAPT
SHISA6
SHISA9

GO:CC
GO:0042734
presynaptic_membrane
28

ITGA3
SYT7
GABRA3
CNTN1
CDH10
ATP2B3
OTOF
IGSF21
EFNB2
SLC6A11
SCN2A
UNC13C
GABRB2
ADRA2A
ADCY8
ATP2B2
KCNJ9
KCNJ3
GABRB1
GRIK2
KCNC2
KCNA3
KCTD12
CHRM2
GABRA5
PDE2A
LPAR1
CPLX3

GO:CC
GO:0031012
extracellular_matrix
54

TNC
F7
COL19A1
COL16A1
WNT11
ACHE
COL9A3
MMP11
PDGFB
MMP9
MXRA5
SFRP1
SPOCK2
MGP
LOX
EFEMP1
TNR
ANGPTL1
TGFBI
ANGPTL6
F12
MATN2
BCAN
POSTN
ADAMTS8
CCN3
LRIG3
TMEFF2
MUC4
ADAMTS5
COL26A1
NTN3
WNT4
LRRTM1
COL6A3
ADAMTS9
ADAMTS15
NPNT
EGFL7
NPPA
GPC6
ADAMTSL5
COL4A5
COL25A1
VWC2
RELN
SPOCK3
LAMB3
COL4A6
S100A10
ADAMTSL2
S100A6
LRRTM3
COL15A1

GO:CC
GO:0030312
external_encapsulating_structure
54

TNC
F7
COL19A1
COL16A1
WNT11
ACHE
COL9A3
MMP11
PDGFB
MMP9
MXRA5
SFRP1
SPOCK2
MGP
LOX
EFEMP1
TNR
ANGPTL1
TGFBI
ANGPTL6
F12
MATN2
BCAN
POSTN
ADAMTS8
CCN3
LRIG3
TMEFF2
MUC4
ADAMTS5
COL26A1
NTN3
WNT4
LRRTM1
COL6A3
ADAMTS9
ADAMTS15
NPNT
EGFL7
NPPA
GPC6
ADAMTSL5
COL4A5
COL25A1
VWC2
RELN
SPOCK3
LAMB3
COL4A6
S100A10
ADAMTSL2
S100A6
LRRTM3
COL15A1

GO:CC
GO:0098982
GABA-ergic_synapse
20

GABRA3
CDH10
ATP2B3
GABRG2
SLC6A11
GABRB2
NLGN4X
ADRA2A
PTPRO
GABRA2
SST
ATP2B2
LRRTM1
GABRB1
GUCY1A1
KCNC2
KCND3
GABRA5
SLC6A17
LPAR1

GO:CC
GO:0150034
distal_axon
35

ITGA3
PTPRN
RASGRF1
NGEF
TRPC5
SNCB
NTSR1
CRHR2
PACSIN1
UNC13C
SLC4A10
SNCA
CPLX2
ADRA2A
KCNA6
PTPRO
THY1
ELAVL4
LRRTM1
ITGA2
KCNC2
PRKCB
CPLX1
CDK5R2
DSCAM
PRNP
CALB2
PCSK1
KCNA3
CHRM2
PENK
INPP5J
MAPT
SHTN1
CPLX3

GO:CC
GO:0043679
axon_terminus
22
PTPRN
SNCB
NTSR1
CRHR2
PACSIN1
UNC13C
SLC4A10
SNCA
CPLX2
ADRA2A
KCNA6
ITGA2
KCNC2
PRKCB
CPLX1
PRNP
CALB2
PCSK1
KCNA3
CHRM2
PENK
CPLX3

GO:CC
GO:0032590
dendrite_membrane
14
GABRA3
GABRG2
HPCA
SLC12A5
GABRA6
GABRA2
THY1
GRIA1
ATP2B2
KCNC2
AKAP5
GABRA5
SHISA6
SHISA9

GO:CC
GO:0044306
neuron_projection_terminus
23
PTPRN
SNCB
NTSR1
CRHR2
PACSIN1
GCH1
UNC13C
SLC4A10
SNCA
CPLX2
ADRA2A
KCNA6
ITGA2
KCNC2
PRKCB
CPLX1
PRNP
CALB2
PCSK1
KCNA3
CHRM2
PENK
CPLX3

GO:CC
GO:0031256
leading_edge_membrane
27
PLXND1
GABRA3
PDE4A
RASGRP2
GABRG2
UNC5A
HPCA
SLC12A5
PACSIN1
PSD4
GABRA6
SNTG1
GABRA2
THY1
GRIA1
ATP2B2
KCNC2
FAM107A
CNTNAP2
AKAP5
ROBO2
INPP5J
GABRA5
MAPT
SHISA6
DPP4
SHISA9

GO:CC
GO:1902495
transmembrane_transporter_complex
42
CACNG3
GABRA3
TRPC7
CNGB1
TRPC5
CACNG5
HCN2
CACNA1I
SLC17A7
GABRG2
KCNJ5
BEST3
KCNA5
SCN2A
SCN7A
CACNG8
GABRA6
GABRB2
SCN2B
CACNA2D4
KCNA6
GABRA2
GRIA1
CACNA1D
KCNF1
KCNJ3
GABRB1
GRIK2
KCNC2
KCND3
VWC2L
CNTNAP2
KCNA3
KCNK4
KCNQ5
GABRA5
VWC2
SHISA6
CACNA1E
RYR2
SHISA9
GRIN2B

GO:CC
GO:1990351
transporter_complex
43
CACNG3
GABRA3
TRPC7
CNGB1
TRPC5
CACNG5
HCN2
CACNA1I
SLC17A7
GABRG2
KCNJ5
BEST3
KCNA5
ATP8A2
SCN2A
SCN7A
CACNG8
GABRA6
GABRB2
SCN2B
CACNA2D4
KCNA6
GABRA2
GRIA1
CACNA1D
KCNF1
KCNJ3
GABRB1
GRIK2
KCNC2
KCND3
VWC2L
CNTNAP2
KCNA3
KCNK4
KCNQ5
GABRA5
VWC2
SHISA6
CACNA1E
RYR2
SHISA9
GRIN2B

GO:CC
GO:0062023
collagen-containing_extracellular_matrix
42
TNC
F7
COL19A1
COL16A1
ACHE
COL9A3
PDGFB
MMP9
MXRA5
SFRP1
MGP
LOX
EFEMP1
TNR
ANGPTL1
TGFBI
ANGPTL6
F12
MATN2
BCAN
POSTN
ADAMTS8
CCN3
TMEFF2
ADAMTS5
COL26A1
NTN3
COL6A3
ADAMTS9
ADAMTS15
NPNT
EGFL7
NPPA
GPC6
COL4A5
COL25A1
VWC2
LAMB3
COL4A6
S100A10
S100A6
COL15A1

GO:CC
GO:0060076
excitatory_synapse
16
ITGA3
CACNG3
ATP2B3
SLC17A7
UNC13C
IGSF11
NLGN4X
GRIA1
ADCY8
ATP2B2
KCNJ9
KCNJ3
CALB2
AKAP5
SHISA6
PLXNA4

GO:CC
GO:0098839
postsynaptic_density_membrane
18
CACNG3
CACNG5
PLPPR4
EFNB2
EPHA7
CACNG8
NLGN4X
ADRA2A
PTPRO
GRIA1
SORCS3
ATP2B2
CLSTN2
GRM5
SHISA6
LRRTM3
SHISA9
GRIN2B

GO:CC
GO:0043204
perikaryon
23
PTPRN
NTSR1
NEURL1
HPCA
SLC12A5
CPNE5
PCSK2
ASS1
SLC4A10
CPLX2
HTR5A
ELAVL4
SERPINI1
KCNC2
RBFOX3
CPLX1
KNDC1
SYNPO
CNTNAP2
NPPA
PCSK1
PENK
SHTN1

GO:CC
GO:0034703
cation_channel_complex
26
CACNG3
TRPC7
CNGB1
TRPC5
HCN2
CACNA1I
KCNJ5
KCNA5
SCN2A
SCN7A
CACNG8
SCN2B
CACNA2D4
KCNA6
CACNA1D
KCNF1
KCNJ3
GRIK2
KCNC2
KCND3
CNTNAP2
KCNA3
KCNK4
KCNQ5
CACNA1E
RYR2

GO:CC
GO:0044309
neuron_spine
24
LZTS1
CAMK2A
RPH3A
NOS1
PALMD
NTSR1
NEURL1
HPCA
GRIP2
PTPRO
GRIA1
SLA
ATP2B2
GRM5
FRMPD4
TANC2
KCND3
SYNPO
AKAP5
ADGRB1
MAPT
SHISA6
LPAR1
SHISA9

GO:CC
GO:0043197
dendritic_spine
23
LZTS1
CAMK2A
RPH3A
NOS1
PALMD
NTSR1
NEURL1
HPCA
PTPRO
GRIA1
SLA
ATP2B2
GRM5
FRMPD4
TANC2
KCND3
SYNPO
AKAP5
ADGRB1
MAPT
SHISA6
LPAR1
SHISA9

GO:CC
GO:0099501
exocytic_vesicle_membrane
19
SYT7
RAB27B
RPH3A
SLC17A7
OTOF
SYNGR3
SYT6
UNC13C
SNCA
MAL2
GABRA2
GRIA1
SYN2
SYNPR
RAB26
RAB3B
SV2B
SLC6A17
CPLX3

GO:CC
GO:0030672
synaptic_vesicle_membrane
19
SYT7
RAB27B
RPH3A
SLC17A7
OTOF
SYNGR3
SYT6
UNC13C
SNCA
MAL2
GABRA2
GRIA1
SYN2
SYNPR
RAB26
RAB3B
SV2B
SLC6A17
CPLX3

GO:CC
GO:0070382
exocytic_vesicle
26
SYT7
RAB27B
RPH3A
SYT17
SLC17A7
SYT10
OTOF
SYNGR3
SYT6
UNC13C
SNCA
MAL2
KIRREL3
GABRA2
GRIA1
SYN2
SYNPR
RAB26
RAB3IL1
LGI3
RAB3B
KCNK9
PENK
SV2B
SLC6A17
CPLX3

GO:CC
GO:0005794
Golgi_apparatus
93
HS3ST1
CAMK1G
TENM1
RAB27B
PTPRN
F7
TRPC7
ACHE
RPH3A
MMP11
PDGFB
NTSR1
ST8SIA5
SYT17
SH3GL2
SORCS1
LGI1
ARFGEF3
OTOF
SPP1
GALNT12
TGFBI
HS3ST2
CHST8
F10
FGD3
VGF
KCNA5
SYNE1
GALNT15
BCAN
ATP8A2
POSTN
MMD2
FHDC1
PLCB2
NDST4
FGF7
TMC6
CASQ1
MARCHF4
MUC4
OCIAD2
ST8SIA6
AP1S3
RIT2
GOLGA7B
HTR5A
CABP1
GALNT14
CLSTN2
COL26A1
NTN3
WNT4
VCAM1
TMEM130
TVP23A
TMC8
RAB26
RAB31
PARM1
RAB3B
B3GALNT1
MUC3A
HS6ST2
PRNP
FUT9
RASGRP1
CNTNAP2
GALNTL6
FUT1
CHST2
GAL3ST3
PCSK1
CREG2
FZD8
ST8SIA3
MGAT4C
GALNT9
GPC6
CALN1
PRKD1
GALNT17
PDE2A
GCNT1
NHS
FUT4
SLC30A10
PDGFA
CHSY3
RAB6C
RAB6D
GOLGA6L7

GO:CC
GO:0012505
endomembrane_system
213
HS3ST1
CAMK1G
CYB561
TENM1
SYT7
RAB27B
TNC
XK
FSTL4
PTPRN
F7
CAMK2B
SLC9A3R2
ACSL4
TRPC7
MGLL
BRINP1
RAPGEF3
CARMIL1
COL19A1
P2RX5
COL16A1
ACHE
RPH3A
NOS1
PITPNM2
COL9A3
MMP11
SEZ6L
PDGFB
CHGA
MMP9
NTSR1
MAP1LC3A
ST8SIA5
CHRDL1
SYT17
RHOV
PLAT
JPH1
SLC17A7
SH3GL2
SORCS1
LGI1
RASD1
PTPN5
SYT10
ARFGEF3
OTOF
IGFBP5
SPP1
GALNT12
TGFBI
FLT3
HS3ST2
WIPF3
RAB9B
CHST8
PACSIN1
PCSK2
MCF2L
F10
FGD3
PLAAT1
SYNGR3
VGF
KCNA5
CALY
ASS1
ANGPTL6
SYNE1
F12
GALNT15
RAMP2
GCH1
RAP1GAP2
BCAN
ATP8A2
CHRM3
POSTN
PDZD2
TMTC1
SYT6
ENTREP1
AGAP2
MMD2
MYC
KIAA0319
FHDC1
UNC13C
PLCB2
CUZD1
NDST4
EGF
FGF7
TMC6
CASQ1
MARCHF4
MUC4
OCIAD2
SNCA
IGFBP3
MAL2
ST8SIA6
KIRREL3
GABRA2
AP1S3
RIT2
THY1
ADAMTS5
GOLGA7B
GRIA1
ADCY8
SLA
SST
SYN2
HTR5A
MX1
CABP1
GALNT14
ESYT3
CLSTN2
DYNC1I1
ACE
SLC37A1
COL26A1
NTN3
ELAVL4
WNT4
BRINP3
VCAM1
FCRLB
LRRTM1
GABRB1
COL6A3
SERPINI1
[illegible]

GO:CC
GO:0031252
cell_leading_edge
36
PLXND1
GABRA3
CAPG
PDE4A
RASGRP2
RAPGEF3
CARMIL1
GABRG2
UNC5A
HPCA
SLC12A5
PACSIN1
PSD4
FGD3
GABRA6
SNTG1
PTPRO
GABRA2
THY1
GRIA1
ATP2B2
KCNC2
FAM107A
CNTNAP2
AKAP5
CTNNA3
ROBO2
INPP5J
GABRA5
MAPT
SHTN1
NHS
SHISA6
DPP4
S100A6
SHISA9

GO:CC
GO:1902710
GABA_receptor_complex
8
GABRA3
GABRG2
GABBR2
GABRA6
GABRB2
GABRA2
GABRB1
GABRA5

GO:CC
GO:0030133
transport_vesicle
36
SYT7
RAB27B
PTPRN
RPH3A
CHGA
SYT17
SLC17A7
SYT10
ARFGEF3
OTOF
PCSK2
SYNGR3
VGF
GALNT15
SYT6
UNC13C
SNCA
MAL2
KIRREL3
GABRA2
AP1S3
GRIA1
SYN2
SYNPR
RAB26
RAB3IL1
LGI3
RAB3B
KCNK9
NPTX1
PCSK1
RPH3AL
PENK
SV2B
SLC6A17
CPLX3

GO:CC
GO:0008328
ionotropic_glutamate_receptor_complex
10
CACNG3
CACNG5
CACNG8
GRIA1
GRIK2
VWC2L
VWC2
SHISA6
SHISA9
GRIN2B

GO:CC
GO:0008021
synaptic_vesicle
23
SYT7
RAB27B
RPH3A
SLC17A7
OTOF
SYNGR3
SYT6
UNC13C
SNCA
MAL2
KIRREL3
GABRA2
GRIA1
SYN2
SYNPR
RAB26
LGI3
RAB3B
KCNK9
PENK
SV2B
SLC6A17
CPLX3

GO:CC
GO:0031253
cell_projection_membrane
31
PLXND1
ITGA3
GABRA3
PDE4A
RASGRP2
CNGB1
GABRG2
UNC5A
HPCA
SLC12A5
PACSIN1
PSD4
GABRA6
SNTG1
CDHR1
GABRA2
THY1
GRIA1
ATP2B2
ACE
KCNC2
FAM107A
CNTNAP2
AKAP5
ROBO2
INPP5J
GABRA5
MAPT
SHISA6
DPP4
SHISA9

GO:CC
GO:0030658
transport_vesicle_membrane
24
SYT7
RAB27B
PTPRN
RPH3A
SLC17A7
SYT10
ARFGEF3
OTOF
SYNGR3
SYT6
UNC13C
SNCA
MAL2
GABRA2
AP1S3
GRIA1
SYN2
SYNPR
RAB26
RAB3B
RPH3AL
SV2B
SLC6A17
CPLX3

GO:CC
GO:0032281
AMPA_glutamate_receptor_complex
8
CACNG3
CACNG5
CACNG8
GRIA1
VWC2L
VWC2
SHISA6
SHISA9

GO:CC
GO:1902711
GABA-A_receptor_complex
7
GABRA3
GABRG2
GABRA6
GABRB2
GABRA2
GABRB1
GABRA5

GO:CC
GO:0098878
neurotransmitter_receptor_complex
10
CACNG3
CACNG5
CACNG8
GRIA1
GRIK2
VWC2L
VWC2
SHISA6
SHISA9
GRIN2B

GO:CC
GO:0098797
plasma_membrane_protein_complex
48
ITGA3
CACNG3
CDH10
CACNG5
CDH7
HCN2
CACNA1I
CDH20
CDH23
CDH6
KCNJ5
BMP2
KCNA5
RAMP2
SCN2A
SCN7A
GABBR2
CACNG8
CDH18
SNTG1
SCN2B
CDH22
CACNA2D4
KCNA6
GRIA1
CACNA1D
GJD2
VCAM1
KCNF1
KCNJ3
ITGA2
GRIK2
RET
KCNC2
GNG8
IRS1
KCND3
VWC2L
CNTNAP2
KCNA3
FZD8
KCNQ5
VWC2
SHISA6
S100A10
CACNA1E
SHISA9
GRIN2B

GO:CC
GO:0048787
presynaptic_active_zone_membrane
9
ITGA3
GABRA3
CDH10
OTOF
GABRB2
ADRA2A
ATP2B2
GABRB1
CPLX3

GO:CC
GO:0005576
extracellular_region
185
ITGA3
TAC1
TENM1
SYT7
CNTN1
RAB27B
TNC
CAPG
TG
JADE2
FSTL4
F7
SLC9A3R2
TLE2
ATP2B3
ACSL4
TRHDE
SEMA3A
SEMA3C
NEBL
RAPGEF3
CARMIL1
MID2
COL19A1
COL16A1
ABCB1
WNT11
ACHE
COL9A3
CRTAC1
MMP11
SUSD2
PDGFB
ISM2
CHGA
MMP9
RSPO4
MXRA5
CHRDL1
SFRP1
PLAT
TUBB4A
AMH
DNAH11
SPOCK2
LGI1
MGP
SEMA5A
HBEGF
LOX
NPR3
FGF12
EFEMP1
IGFBP5
TNR
PAPPA2
ANGPTL1
NRP2
SPP1
PPL
TEK
TGFBI
NXPH1
CPNE5
BMP2
PCSK2
MCF2L
F10
RHOJ
IDUA
VGF
ASS1
ANGPTL6
F12
MATN2
BCAN
POSTN
PDZD2
CD101
ADAMTS8
AGAP2
CCN3
CGREF1
EGF
LRIG3
MDGA2
FGF7
TMC6
PRKACB
TMEFF2
MUC4
SNCA
ADAMTS19
GABRB2
CREB5
IGFBP3
GNRH1
MAL2
KIRREL3
PTPRO
SPOCK1
THY1
ADAMTS5
GRIA1
SST
ATP2B2
CABP1
NRG2
NBL1
VWA5B1
CSRP1
SCUBE1
ACE
SPTBN4
LY6E
COL26A1
NTN3
WNT4
BRINP3
VCAM1
LRRTM1
COL6A3
SERPINI1
ADAMTS9
HPGD
PTER
ADAMTS15
SPINT1
PRKCB
TMC8
LGI3
NPNT
RAB3B
MUC3A
SEMA3E
ALK
[illegible]

GO:CC
GO:0048786
presynaptic_active_zone
13
ITGA3
GABRA3
CDH10
SLC17A7
OTOF
UNC13C
GABRB2
ADRA2A
ADCY8
ATP2B2
GABRB1
GRM7
CPLX3

GO:CC
GO:0044304
main_axon
11
TUBB4A
LGI1
SCN2A
SPOCK1
THY1
SPTBN4
KCNC2
CNTNAP2
ROBO2
MAPT
MBP

GO:CC
GO:0043235
receptor_complex
38
PLXND1
ITGA3
CACNG3
GABRA3
CACNG5
GABRG2
NRP2
TEK
FLT3
BMP2
RAMP2
PPARG
EPHA7
GABBR2
CACNG8
GABRA6
GABRB2
HTR2C
ADRA2A
GABRA2
GRIA1
GABRB1
ITGA2
GRIK2
RET
GFRA2
IRS1
ROR2
GPR37
ALK
VWC2L
GABRA5
VWC2
SHISA6
GRM7
PLXNA4
SHISA9
GRIN2B

GO:CC
GO:0034705
potassium_channel_complex
13
HCN2
KCNJ5
KCNA5
KCNA6
KCNF1
KCNJ3
GRIK2
KCNC2
KCND3
CNTNAP2
KCNA3
KCNK4
KCNQ5

GO:CC
GO:0005737
cytoplasm
452
HS3ST1
ITGA3
YBX2
CACNG3
PNPLA4
GAS7
MATK
PAX6
CAMK1G
CYB561
TENM1
SYT7
SLC7A14
PLEKHB1
MRI1
RAB27B
MYO16
TNC
CAPG
XK
LMO3
FSTL4
PTPRN
GYG2
F7
RASGRF1
CAMK2B
DGKG
LZTS1
PDE4A
NGEF
KIF26A
ACSL4
RASGRP2
TRPC7
SPTB
CNGB1
CAMK2A
TRPC5
TRHDE
ALPK1
PPP2R2C
SNCB
MGLL
NUAK1
IPCEF1
DCX
NEBL
ADCY2
BRINP1
RAPGEF3
CARMIL1
DCT
MID2
STK17B
COL19A1
P2RX5
COL16A1
ABCB1
WNT11
ACHE
RPH3A
NOS1
PITPNM2
MYH7
COL9A3
PALMD
MMP11
SEZ6L
PDGFB
CHGA
MMP9
DOK5
NTSR1
PPP1R16B
MAP1LC3A
CELF4
ST8SIA5
CHRDL1
KLF8
SALL1
SYT17
RHOV
SFRP1
PLAT
JPH1
TUBB4A
SLC17A7
DNAH11
GCK
SH3GL2
SHB
SFXN3
FBXW4
NEURL1
SORCS1
LGI1
RASD1
IL10RA
PTPN5
SYT10
TPD52L1
EYA4
ARFGEF3
TFEB
HBEGF
GABRG2
RBP1
FGF12
HES1
OTOF
IGFBP5
EFHD1
PRRX1
PAPPA2
CHD5
CDK18
RCAN3
SPP1
PPL
NEK6
GALNT12
ACOT2
TEK
MOB3B
TGFBI
EGR1
DUSP4
ECHDC2
HPCA
FLT3
HS3ST2
WIPF3
RAB9B
CHST8
IQSEC2
[illegible]

GO:CC
GO:0031982
vesicle
172
ITGA3
CACNG3
CYB561
SYT7
CNTN1
RAB27B
CAPG
JADE2
FSTL4
PTPRN
F7
CAMK2B
SLC9A3R2
ATP2B3
ACSL4
CNGB1
CAMK2A
TRHDE
SEMA3C
NEBL
RAPGEF3
CARMIL1
DCT
MID2
ABCB1
RPH3A
NOS1
CRTAC1
SUSD2
PDGFB
CHGA
MMP9
MAP1LC3A
MXRA5
SYT17
RHOV
SFRP1
PLAT
TUBB4A
SLC17A7
SH3GL2
SYT10
MGP
ARFGEF3
SEMA5A
HBEGF
GABRG2
NPR3
OTOF
EFEMP1
PAPPA2
ANGPTL1
SPP1
PPL
TGFBI
FLT3
WIPF3
RAB9B
GRM4
PACSIN1
CPNE5
PCSK2
RHOJ
IDUA
SYNGR3
VGF
CALY
ASS1
ANGPTL6
F12
GALNT15
GCH1
ATP8A2
SYT6
CD101
ENTREP1
AGAP2
KIAA0319
UNC13C
PLCB2
CUZD1
EGF
LRIG3
TMC6
CACNG8
PRKACB
MUC4
OCIAD2
SNCA
GABRB2
CREB5
MAL2
KIRREL3
PTPRO
GABRA2
AP1S3
THY1
GRIA1
ADCY8
SLA
SST
ATP2B2
SYN2
DYNC1I1
CSRP1
ACE
SPTBN4
WNT4
VCAM1
COL6A3
SERPINI1
SYNPR
CAMKV
HPGD
FZD6
RET
PTER
KCNC2
SPINT1
PRKCB
TMC8
RAB26
RAB3IL1
TAP1
RAB31
LGI3
NPNT
ROR2
PARM1
RAB3B
KCNK9
PCDH7
ALK
NPTX1
PRNP
SCG2
[illegible]

GO:CC
GO:0032809
neuronal_cell_body_membrane
7
UNC5A
HPCA
THY1
ADCY8
ATP2B2
KCNC2
GABRA5

GO:MF
GO:0022839
monoatomic_ion_gated_channel_activity
42
CACNG3
GABRA3
CNGB1
CACNG5
P2RX5
HCN2
CACNA1I
NALCN
GABRG2
KCNJ5
KCNA5
SCN2A
SCN7A
KCNH5
TMC6
CACNG8
GABRA6
GABRB2
SCN2B
CACNA2D4
KCNA6
GABRA2
GRIA1
CACNA1D
KCNJ9
KCNF1
KCNJ3
GABRB1
GRIK2
KCNC2
TMC8
KCNK9
KCND3
KCNA3
KCNJ10
KCNK4
KCNJ12
KCNQ5
GABRA5
CACNA1E
RYR2
GRIN2B

GO:MF
GO:0005509
calcium_ion_binding
67
SYT7
CDH10
CAPG
FSTL4
F7
DGKG
RASGRP2
SNCB
CDH7
RPH3A
PITPNM2
CRTAC1
CDH20
PCDH11X
SYT17
TUBB4A
CDH23
SPOCK2
SYT10
MGP
CDH6
OTOF
EFEMP1
EFHD1
HPCAL4
HPCA
F10
F12
GCH1
MATN2
SYT6
DGKB
UNC13C
PLCB2
CGREF1
EGF
CASQ1
SNCA
CDH18
CDHR1
PLCH2
CDH22
SPOCK1
ATP2B2
CABP1
ESYT3
CLSTN2
SCUBE1
VSNL1
RET
NPNT
PCDH7
REPS2
PRNP
CALB2
RASGRP1
EGFL7
CALN1
EFHC2
SPOCK3
GRM7
DCHS2
S100A10
S100A6
CACNA1E
RYR2
DOC2B

GO:MF
GO:0022836
gated_channel_activity 42

CACNG3
GABRA3
CNGB1
CACNG5
P2RX5
HCN2
CACNA1I
NALCN
GABRG2
KCNJ5
KCNA5
SCN2A
SCN7A
KCNH5
TMC6
CACNG8
GABRA6
GABRB2
SCN2B
CACNA2D4
KCNA6
GABRA2
GRIA1
CACNA1D
KCNJ9
KCNF1
KCNJ3
GABRB1
GRIK2
KCNC2
TMC8
KCNK9
KCND3
KCNA3
KCNJ10
KCNK4
KCNJ12
KCNQ5
GABRA5
CACNA1E
RYR2
GRIN2B

GO:MF
GO:0005216
monoatomic_ion_channel_activity 48

CACNG3
GABRA3
TRPC7
CNGB1
TRPC5
CACNG5
P2RX5
HCN2
CACNA1I
NALCN
SLC17A7
GABRG2
KCNJ5
BEST3
KCNA5
SCN2A
SCN7A
KCNH5
TMC6
CACNG8
GABRA6
GABRB2
SCN2B
CACNA2D4
KCNA6
GABRA2
GRIA1
SLC24A2
CACNA1D
KCNJ9
KCNF1
KCNJ3
GABRB1
GRIK2
KCNC2
TMC8
KCNK9
KCND3
KCNA3
KCNJ10
KCNK4
KCNJ12
SLC24A3
KCNQ5
GABRA5
CACNA1E
RYR2
GRIN2B

GO:MF
GO:1901702
salt_transmembrane_transporter_activity 56

CACNG3
SLC6A7
GABRA3
ATP2B3
TRPC7
TRPC5
CACNG5
HCN2
CACNA1I
NALCN
SLC17A7
GABRG2
SLC9A2
KCNJ5
SLC12A5
BEST3
KCNA5
SLC6A11
SCN2A
SCN7A
KCNH5
SLC13A5
CACNG8
SLC4A10
SLC26A1
GABRA6
GABRB2
SCN2B
CACNA2D4
KCNA6
SLC25A4
GABRA2
SLC24A2
ATP2B2
CACNA1D
KCNJ9
SLC9C2
KCNF1
KCNJ3
GABRB1
GRIK2
KCNC2
KCNK9
KCND3
KCNA3
KCNJ10
KCNK4
KCNJ12
SLC24A3
KCNQ5
GABRA5
SLC38A3
SLC30A10
CACNA1E
RYR2
GRIN2B

GO:MF
GO:0015075
monoatomic_ion_transmembrane_transporter_activity 64

CACNG3
SLC6A7
GABRA3
ATP2B3
TRPC7
CNGB1
TRPC5
CACNG5
P2RX5
HCN2
CACNA1I
NALCN
SLC17A7
SFXN3
GABRG2
SLC9A2
KCNJ5
SLC12A5
BEST3
KCNA5
SLC6A11
SCN2A
SCN7A
KCNH5
SLC13A5
TMC6
CACNG8
SLC4A10
SLC26A1
GABRA6
GABRB2
SCN2B
CACNA2D4
KCNA6
SLC25A4
GABRA2
GRIA1
SLC24A2
ATP2B2
CACNA1D
KCNJ9
SLC9C2
KCNF1
KCNJ3
GABRB1
GRIK2
KCNC2
TMC8
KCNK9
KCND3
KCNA3
KCNJ10
KCNK4
KCNJ12
SLC24A3
KCNQ5
GABRA5
ATP13A5
SLC38A3
SLC30A10
S100A6
CACNA1E
RYR2
GRIN2B

GO:MF
GO:0015267
channel_activity 50

CACNG3
GABRA3
TRPC7
CNGB1
TRPC5
CACNG5
P2RX5
HCN2
CACNA1I
NALCN
SLC17A7
GABRG2
KCNJ5
SLC12A5
BEST3
KCNA5
SCN2A
SCN7A
KCNH5
TMC6
CACNG8
GABRA6
GABRB2
SCN2B
CACNA2D4
KCNA6
GABRA2
GRIA1
SLC24A2
CACNA1D
GJD2
KCNJ9
KCNF1
KCNJ3
GABRB1
GRIK2
KCNC2
TMC8
KCNK9
KCND3
KCNA3
KCNJ10
KCNK4
KCNJ12
SLC24A3
KCNQ5
GABRA5
CACNA1E
RYR2
GRIN2B

GO:MF
GO:0022803
passive_transmembrane_transporter_activity 50

CACNG3
GABRA3
TRPC7
CNGB1
TRPC5
CACNG5
P2RX5
HCN2
CACNA1I
NALCN
SLC17A7
GABRG2
KCNJ5
SLC12A5
BEST3
KCNA5
SCN2A
SCN7A
KCNH5
TMC6
CACNG8
GABRA6
GABRB2
SCN2B
CACNA2D4
KCNA6
GABRA2
GRIA1
SLC24A2
CACNA1D
GJD2
KCNJ9
KCNF1
KCNJ3
GABRB1
GRIK2
KCNC2
TMC8
KCNK9
KCND3
KCNA3
KCNJ10
KCNK4
KCNJ12
SLC24A3
KCNQ5
GABRA5
CACNA1E
RYR2
GRIN2B

GO:MF
GO:0046873
metal_ion_transmembrane_transporter_activity 46

CACNG3
SLC6A7
ATP2B3
TRPC7
TRPC5
CACNG5
HCN2
CACNA1I
NALCN
SLC17A7
SLC9A2
KCNJ5
SLC12A5
KCNA5
SLC6A11
SCN2A
SCN7A
KCNH5
SLC13A5
CACNG8
SLC4A10
SCN2B
CACNA2D4
KCNA6
SLC24A2
ATP2B2
CACNA1D
KCNJ9
SLC9C2
KCNF1
KCNJ3
GRIK2
KCNC2
KCNK9
KCND3
KCNA3
KCNJ10
KCNK4
KCNJ12
SLC24A3
KCNQ5
SLC38A3
SLC30A10
CACNA1E
RYR2
GRIN2B

GO:MF
GO:0015318
inorganic_molecular_entity_transmembrane_transporter_activity 59

CACNG3
SLC6A7
GABRA3
ATP2B3
TRPC7
TRPC5
CACNG5
HCN2
CACNA1I
NALCN
SLC17A7
GABRG2
SLC9A2
KCNJ5
SLC12A5
BEST3
KCNA5
SLC6A11
ADAMTS8
SCN2A
SCN7A
KCNH5
SLC13A5
CACNG8
SLC4A10
SLC26A1
GABRA6
GABRB2
SCN2B
CACNA2D4
KCNA6
SLC25A4
GABRA2
GRIA1
SLC24A2
ATP2B2
CACNA1D
SLC37A1
KCNJ9
SLC9C2
KCNF1
KCNJ3
GABRB1
GRIK2
KCNC2
KCNK9
KCND3
KCNA3
KCNJ10
KCNK4
KCNJ12
SLC24A3
KCNQ5
GABRA5
SLC38A3
SLC30A10
CACNA1E
RYR2
GRIN2B

GO:MF
GO:0022843
voltage-gated_monoatomic_cation_channel_activity 24

CACNG3
CACNG5
HCN2
CACNA1I
KCNJ5
KCNA5
KCNH5
CACNG8
SCN2B
CACNA2D4
KCNA6
CACNA1D
KCNJ9
KCNF1
KCNJ3
KCNC2
KCNK9
KCND3
KCNA3
KCNJ10
KCNK4
KCNJ12
KCNQ5
CACNA1E

GO:MF
GO:0005244
voltage-gated_monoatomic_ion_channel_activity 28

CACNG3
CACNG5
HCN2
CACNA1I
NALCN
KCNJ5
KCNA5
SCN2A
SCN7A
KCNH5
CACNG8
SCN2B
CACNA2D4
KCNA6
CACNA1D
KCNJ9
KCNF1
KCNJ3
KCNC2
KCNK9
KCND3
KCNA3
KCNJ10
KCNK4
KCNJ12
KCNQ5
CACNA1E
GRIN2B

GO:MF
GO:0022832
voltage-gated_channel_activity 28

CACNG3
CACNG5
HCN2
CACNA1I
NALCN
KCNJ5
KCNA5
SCN2A
SCN7A
KCNH5
CACNG8
SCN2B
CACNA2D4
KCNA6
CACNA1D
KCNJ9
KCNF1
KCNJ3
KCNC2
KCNK9
KCND3
KCNA3
KCNJ10
KCNK4
KCNJ12
KCNQ5
CACNA1E
GRIN2B

GO:MF
GO:0022857
transmembrane_transporter_activity 79

ABCB5
CACNG3
SLC6A7
GABRA3
SLC7A14
ATP2B3
TRPC7
CNGB1
TRPC5
CACNG5
P2RX5
ABCB1
HCN2
CACNA1I
SLCO4A1
NALCN
SLC17A7
SFXN3
GABRG2
SLC9A2
CNNM1
KCNJ5
SLC12A5
BEST3
KCNA5
SLC6A11
ADAMTS8
SCN2A
SCN7A
KCNH5
SLC13A5
TMC6
CACNG8
SLC4A10
SLC26A1
GABRA6
GABRB2
SCN2B
CACNA2D4
KCNA6
SLC25A4
GABRA2
GRIA1
SLC24A2
ATP2B2
CACNA1D
GJD2
SLC37A1
KCNJ9
SLC9C2
KCNF1
KCNJ3
GABRB1
GRIK2
KCNC2
TMC8
TAP1
CPLX1
KCNK9
KCND3
ABCG4
SLCO2A1
KCNA3
KCNJ10
KCNK4
KCNJ12
SLC24A3
SV2B
KCNQ5
GABRA5
ATP13A5
SLC38A3
SLC30A10
SLC6A17
S100A6
CACNA1E
RYR2
CPLX3
GRIN2B

GO:MF
GO:0005261
monoatomic_cation_channel_activity 36

CACNG3
TRPC7
CNGB1
TRPC5
CACNG5
P2RX5
HCN2
CACNA1I
NALCN
KCNJ5
KCNA5
SCN2A
SCN7A
KCNH5
CACNG8
SCN2B
CACNA2D4
KCNA6
SLC24A2
CACNA1D
KCNJ9
KCNF1
KCNJ3
GRIK2
KCNC2
KCNK9
KCND3
KCNA3
KCNJ10
KCNK4
KCNJ12
SLC24A3
KCNQ5
CACNA1E
RYR2
GRIN2B

GO:MF
GO:0015079
potassium_ion_transmembrane_transporter_activity 24

HCN2
SLC17A7
SLC9A2
KCNJ5
SLC12A5
KCNA5
KCNH5
SCN2B
KCNA6
SLC24A2
KCNJ9
SLC9C2
KCNF1
KCNJ3
GRIK2
KCNC2
KCNK9
KCND3
KCNA3
KCNJ10
KCNK4
KCNJ12
SLC24A3
KCNQ5

GO:MF
GO:0022890
inorganic_cation_transmembrane_transporter_activity 48

CACNG3
SLC6A7
ATP2B3
TRPC7
TRPC5
CACNG5
HCN2
CACNA1I
NALCN
SLC17A7
SLC9A2
KCNJ5
SLC12A5
KCNA5
SLC6A11
SCN2A
SCN7A
KCNH5
SLC13A5
CACNG8
SLC4A10
SCN2B
CACNA2D4
KCNA6
SLC25A4
GRIA1
SLC24A2
ATP2B2
CACNA1D
KCNJ9
SLC9C2
KCNF1
KCNJ3
GRIK2
KCNC2
KCNK9
KCND3
KCNA3
KCNJ10
KCNK4
KCNJ12
SLC24A3
KCNQ5
SLC38A3
SLC30A10
CACNA1E
RYR2
GRIN2B

GO:MF
GO:0008324
monoatomic_cation_transmembrane_transporter_activity 50

CACNG3
SLC6A7
ATP2B3
TRPC7
CNGB1
TRPC5
CACNG5
P2RX5
HCN2
CACNA1I
NALCN
SLC17A7
SLC9A2
KCNJ5
SLC12A5
KCNA5
SLC6A11
SCN2A
SCN7A
KCNH5
SLC13A5
CACNG8
SLC4A10
SCN2B
CACNA2D4
KCNA6
SLC25A4
SLC24A2
ATP2B2
CACNA1D
KCNJ9
SLC9C2
KCNF1
KCNJ3
GRIK2
KCNC2
KCNK9
KCND3
KCNA3
KCNJ10
KCNK4
KCNJ12
SLC24A3
KCNQ5
ATP13A5
SLC38A3
SLC30A10
CACNA1E
RYR2
GRIN2B

GO:MF
GO:0005215
transporter_activity 81

ABCB5
CACNG3
SLC6A7
GABRA3
SLC7A14
ATP2B3
TRPC7
CNGB1
TRPC5
CACNG5
P2RX5
ABCB1
PITPNM2
HCN2
CACNA1I
SLCO4A1
NALCN
SLC17A7
SFXN3
GABRG2
SLC9A2
CNNM1
KCNJ5
SLC12A5
BEST3
KCNA5
SLC6A11
ATP8A2
ADAMTS8
SCN2A
SCN7A
KCNH5
SLC13A5
TMC6
CACNG8
SLC4A10
SLC26A1
GABRA6
GABRB2
SCN2B
CACNA2D4
KCNA6
SLC25A4
GABRA2
GRIA1
SLC24A2
ATP2B2
CACNA1D
GJD2
SLC37A1
KCNJ9
SLC9C2
KCNF1
KCNJ3
GABRB1
GRIK2
KCNC2
TMC8
TAP1
CPLX1
KCNK9
KCND3
ABCG4
SLCO2A1
KCNA3
KCNJ10
KCNK4
KCNJ12
SLC24A3
SV2B
KCNQ5
GABRA5
ATP13A5
SLC38A3
SLC30A10
SLC6A17
S100A6
CACNA1E
RYR2
CPLX3
GRIN2B

GO:MF
GO:0099507
ligand-gated_monoatomic_ion_channel_activity_involved_in_regulation_of_presynaptic_membrane_potential 8

GABRA3
GABRB2
GABRA2
GRIA1
GABRB1
GRIK2
GABRA5
GRIN2B

GO:MF
GO:0005249
voltage-gated_potassium_channel_activity 17

HCN2
KCNJ5
KCNA5
KCNH5
SCN2B
KCNA6
KCNJ9
KCNF1
KCNJ3
KCNC2
KCNK9
KCND3
KCNA3
KCNJ10
KCNK4
KCNJ12
KCNQ5

GO:MF
GO:0022851
GABA-gated_chloride_ion_channel_activity 7

GABRA3
GABRG2
GABRA6
GABRB2
GABRA2
GABRB1
GABRA5

GO:MF
GO:0005267
potassium_channel_activity 18

HCN2
KCNJ5
KCNA5
KCNH5
SCN2B
KCNA6
KCNJ9
KCNF1
KCNJ3
GRIK2
KCNC2
KCNK9
KCND3
KCNA3
KCNJ10
KCNK4
KCNJ12
KCNQ5

GO:MF
GO:0016917
GABA_receptor_activity 8

GABRA3
GABRG2
GABBR2
GABRA6
GABRB2
GABRA2
GABRB1
GABRA5

GO:MF
GO:0015276
ligand-gated_monoatomic_ion_channel_activity 19

GABRA3
CNGB1
P2RX5
HCN2
GABRG2
KCNJ5
GABRA6
GABRB2
GABRA2
GRIA1
KCNJ9
KCNJ3
GABRB1
GRIK2
KCNJ10
KCNJ12
GABRA5
RYR2
GRIN2B

GO:MF
GO:0022834
ligand-gated_channel_activity
19

GABRA3
CNGB1
P2RX5
HCN2
GABRG2
KCNJ5
GABRA6
GABRB2
GABRA2
GRIA1
KCNJ9
KCNJ3
GABRB1
GRIK2
KCNJ10
KCNJ12
GABRA5
RYR2
GRIN2B

GO:MF
GO:0004890
GABA-A_receptor_activity
7

GABRA3
GABRG2
GABRA6
GABRB2
GABRA2
GABRB1
GABRA5

GO:MF
GO:0099095
ligand-gated_monoatomic_anion_channel_activity
7

GABRA3
GABRG2
GABRA6
GABRB2
GABRA2
GABRB1
GABRA5

GO:MF
GO:0005102
signaling_receptor_binding
89

ITGA3
CACNG3
TAC1
TG
F7
RASGRF1
SLC9A3R2
CAMK2A
SEMA3A
SEMA3C
COL16A1
WNT11
PITPNM2
PDGFB
SFRP1
PLAT
AMH
LGI1
CCKBR
SEMA5A
HBEGF
FGF12
EFEMP1
TNR
ANGPTL1
SPP1
TGFBI
NXPH1
EFNB2
BMP2
IDUA
RASL11B
VGF
KCNA5
ANGPTL6
BEX2
ADAMTS8
EPHA7
CCN3
EGF
FGF7
GRIP2
IGSF11
MUC4
NLGN4X
GNRH1
ADRA2A
RIT2
JAKMIP1
THY1
ADAMTS5
SLA
SST
ATP2B2
NRG2
NBL1
ACE
NTN3
WNT4
VCAM1
GABRB1
ITGA2
BTNL9
TAP1
NPNT
GRM5
IRS1
IL1RAPL1
SEMA3E
APLN
DSCAM
PRNP
SCG2
EGFL7
MYD88
VEGFB
NPPA
LY6H
FZD8
AKAP5
LYNX1
PENK
ADRA2C
GABRA5
SHISA6
RELN
PDGFA
DPP4
S1PR3

GO:MF
GO:0035254
glutamate_receptor_binding
10

CACNG3
RASGRF1
SLC9A3R2
CAMK2A
GRIP2
IGSF11
ATP2B2
PRNP
AKAP5
SHISA6

GO:MF
GO:0030594
neurotransmitter_receptor_activity
15

GABRA3
GABRG2
CHRM3
GABRA6
GABRB2
HTR2C
GABRA2
GRIA1
HTR5A
GABRB1
GRIK2
GRM5
CHRM2
GABRA5
GRIN2B

GO:MF
GO:0005237
inhibitory_extracellular_ligand-gated_monoatomic_ion_channel_activity
6

GABRA3
GABRG2
GABRA6
GABRB2
GABRA2
GABRA5

GO:MF
GO:0030165
PDZ_domain_binding
13

CACNG3
ATP2B3
GRIA1
ATP2B2
PLEKHA2
GPR37
FZD8
SSTR2
ADGRB1
SHISA6
GRM7
LPAR1
SHISA9

GO:MF
GO:0098960
postsynaptic_neurotransmitter_receptor_activity
12

GABRA3
GABRG2
CHRM3
GABRA6
GABRB2
GABRA2
GRIA1
GABRB1
GRIK2
GRM5
CHRM2
GABRA5

GO:MF
GO:0005201
extracellular_matrix_structural_constituent
19

TNC
COL19A1
COL16A1
COL9A3
MXRA5
MGP
EFEMP1
TGFBI
MATN2
POSTN
MUC4
COL6A3
NPNT
MUC3A
COL4A5
COL25A1
LAMB3
COL4A6
COL15A1

GO:MF
GO:0008376
acetylgalactosaminyltransferase_activity
8

GALNT12
GALNT15
GALNT14
B3GALNT1
GALNTL6
GALNT9
GALNT17
CHSY3

HPA
HPA:0090991
cerebellum;_molecular_layer_-_neuropil[â‰¥Low]
39

GAS7
SYT7
CNTN1
CAMK2B
SPTB
SNCB
CDH7
RPH3A
NOS1
PDGFB
MAP1LC3A
SLC17A7
SH3GL2
SPOCK2
EFEMP1
TNR
DLGAP3
SLC12A5
PACSIN1
SYNGR3
SCN2A
SNCA
GABRA6
KIRREL3
RIT2
SPHKAP
THY1
CA10
DYNC1I1
SYNPR
FABP7
NPTX1
PRNP
CNTNAP2
TTC9B
SLITRK4
CTNNA3
SV2B
MAPT

HPA
HPA:0091062
cerebellum;_synaptic_glomeruli_-_core[â‰¥Medium]
31

SYT7
CAMK2B
ATP2B3
SPTB
SNCB
CDH7
RPH3A
PDGFB
MAP1LC3A
SLC17A7
SH3GL2
SPOCK2
TNR
DLGAP3
SLC12A5
BCAN
SCN2A
GABBR2
SLC4A10
GABRA6
GABRB2
KIRREL3
DYNC1I1
RCAN1
BDH1
KCNJ9
SYNPR
CALB2
TTC9B
SV2B
CLEC2L

HPA
HPA:0091061
cerebellum;_synaptic_glomeruli_-_core[â‰¥Low]
35

SYT7
GABRA3
CAMK2B
ATP2B3
ACSL4
SPTB
SNCB
CDH7
RPH3A
PDGFB
MAP1LC3A
SLC17A7
SH3GL2
SPOCK2
TNR
DLGAP3
SLC12A5
BCAN
SCN2A
GABBR2
SLC4A10
GABRA6
GABRB2
KIRREL3
DYNC1I1
RCAN1
BDH1
KCNJ9
SYNPR
FABP7
CALB2
TTC9B
SLITRK4
SV2B
CLEC2L

HPA
HPA:0100141
cerebral_cortex;_neuronal_projections[â‰¥Low]
12

CACNG3
TAC1
PTPRN
DCX
PCDH11X
CALY
KIAA0319
SPTBN4
NPTX1
SCG2
ABCG4
SLC6A17

HPA
HPA:0091031
cerebellum;_processes_in_molecular_layer[â‰¥Low]
28

ATP2B3
SPTB
NEURL1
SLC12A5
GRM4
SYNGR3
CALY
SLC6A11
BCAN
CACNG8
SLC4A10
GABRA6
SNTG1
PLPPR1
RIT2
GRIA1
SPTBN4
KCNF1
DCLK3
FABP7
CREG2
SHTN1
FUT4
PDGFA
S100A6
MBP
DPF3
FRRS1L

HPA
HPA:0100142
cerebral_cortex;_neuronal_projections[â‰¥Medium[
11

CACNG3
TAC1
PTPRN
PCDH11X
CALY
KIAA0319
SPTBN4
NPTX1
SCG2
ABCG4
SLC6A17

HPA
HPA:0090941
cerebellum;_Purkinje_cells_-_cytoplasm/membrane[â‰¥Low]
44

ITGA3
SYT7
CAMK2B
ATP2B3
ACSL4
SPTB
SNCB
P2RX5
RPH3A
PDGFB
MAP1LC3A
PPP1R17
SH3GL2
SPOCK2
EFEMP1
DLGAP3
GRM4
PACSIN1
IDUA
RFPL2
CALY
GABBR2
SLC4A10
GABRA6
SPHKAP
CCSAP
CA10
DYNC1I1
RCAN1
BDH1
ELAVL4
CAMKV
NPTX1
DSCAM
PRNP
CALB2
CNTNAP2
TTC9B
ST8SIA3
EFHC2
MAP7D2
MAPT
FAM163B
CLEC2L

HPA
HPA:0091032
cerebellum;_processes_in_molecular_layer[â‰¥Medium]
26

ATP2B3
SPTB
NEURL1
SLC12A5
GRM4
SYNGR3
CALY
SLC6A11
BCAN
CACNG8
SLC4A10
GABRA6
SNTG1
PLPPR1
GRIA1
SPTBN4
KCNF1
DCLK3
FABP7
CREG2
SHTN1
FUT4
PDGFA
MBP
DPF3
FRRS1L

HPA
HPA:0020062
adrenal_gland;_medullary_cells[â‰¥Medium[
11

PTPRN
PCSK2
VGF
CPLX2
ARHGAP36
SST
SPTBN4
ELAVL4
CPLX1
SCG2
PENK

HPA
HPA:0020061
adrenal_gland;_medullary_cells[â‰¥Low]
11

PTPRN
PCSK2
VGF
CPLX2
ARHGAP36
SST
SPTBN4
ELAVL4
CPLX1
SCG2
PENK

HPA
HPA:0090992
cerebellum;_molecular_layer_-_neuropil[â‰¥Medium[
22

GAS7
SYT7
CNTN1
CAMK2B
SNCB
CDH7
RPH3A
NOS1
SLC17A7
SH3GL2
SPOCK2
TNR
DLGAP3
SLC12A5
PACSIN1
SYNGR3
KIRREL3
SYNPR
FABP7
CNTNAP2
SV2B
MAPT

HPA
HPA:0091071
cerebellum;_white_matter_cells_-_cytoplasm/membrane[â‰¥Low]
32
CAMK2B
ACSL4
SPTB
PDGFB
MAP1LC3A
NEURL1
DLGAP3
IDUA
CALY
SLC6A11
SLC4A10
GABRA6
PLPPR1
KIRREL3
AK5
THY1
DYNC1I1
DCLK3
FABP7
RBFOX3
EMX2
NPTX1
PRNP
CALB2
CNTNAP2
CTNNA3
SV2B
SHTN1
FAM163B
PDGFA
S100A6
CLEC2L

HPA
HPA:0091041
cerebellum;_processes_in_white_matter[â‰¥Low]
26
SYT7
CNTN1
CAMK2B
CHGA
MAP1LC3A
PPP1R17
SPOCK2
TNR
PACSIN1
SYNGR3
RFPL2
BCAN
CACNG8
SPTBN4
KCNF1
DCLK3
CALB2
CREG2
EFHC2
SHTN1
FUT4
PDGFA
S100A6
MBP
CLEC2L
FRRS1L

HPA
HPA:0020063
adrenal_gland;_medullary_cells[High]
8
PTPRN
PCSK2
CPLX2
ARHGAP36
ELAVL4
CPLX1
SCG2
PENK

HPA
HPA:0091042
cerebellum;_processes_in_white_matter[â‰¥Medium]
23
CNTN1
CAMK2B
CHGA
PPP1R17
SPOCK2
TNR
PACSIN1
SYNGR3
RFPL2
BCAN
CACNG8
SPTBN4
KCNF1
DCLK3
CALB2
CREG2
EFHC2
SHTN1
FUT4
PDGFA
MBP
CLEC2L
FRRS1L

HPA
HPA:0090971
cerebellum;_granular_cells_-_cytoplasm/membrane[â‰¥Low]
31
CNTN1
CAMK2B
ATP2B3
SPTB
FOSL2
NOS1
NEURL1
DLGAP3
IDUA
SYNGR3
HCRTR2
CACNG8
PLPPR1
KIRREL3
SPHKAP
AK5
THY1
CCSAP
CA10
DYNC1I1
SPTBN4
CAMKV
FABP7
EMX2
DSCAM
PRNP
CALB2
MAP7D2
SHTN1
PDGFA
S100A6

HPA
HPA:0091033
cerebellum;_processes_in_molecular_layer[High]
17
ATP2B3
NEURL1
SYNGR3
CALY
SLC6A11
BCAN
CACNG8
SLC4A10
PLPPR1
SPTBN4
KCNF1
FABP7
CREG2
SHTN1
FUT4
PDGFA
MBP

HPA
HPA:0090942
cerebellum;_Purkinje_cells_-_cytoplasm/membrane[â‰¥Medium]
34
ITGA3
SYT7
CAMK2B
ATP2B3
SPTB
SNCB
RPH3A
PDGFB
MAP1LC3A
PPP1R17
SH3GL2
SPOCK2
EFEMP1
GRM4
PACSIN1
IDUA
RFPL2
CALY
GABBR2
SLC4A10
GABRA6
CCSAP
CA10
DYNC1I1
RCAN1
BDH1
NPTX1
DSCAM
PRNP
CALB2
CNTNAP2
MAP7D2
FAM163B
CLEC2L

HPA
HPA:0091072
cerebellum;_white_matter_cells_-_cytoplasm/membrane[â‰¥Medium]
25
CAMK2B
SPTB
PDGFB
MAP1LC3A
NEURL1
DLGAP3
IDUA
CALY
SLC6A11
GABRA6
PLPPR1
KIRREL3
AK5
DYNC1I1
DCLK3
FABP7
CALB2
CNTNAP2
CTNNA3
SV2B
SHTN1
FAM163B
PDGFA
S100A6
CLEC2L

HPA
HPA:0260000
hypothalamus
10
PTPRN
PPP1R17
PCSK2
VGF
CALY
ARHGAP36
GNRH1
SST
SCG2
PABPC1L2B

HPA
HPA:0091001
cerebellum;_molecular_layer_cells_-_cytoplasm/membrane[â‰¥Low]
32
GAS7
SYT7
ACSL4
SNCB
FOSL2
RPH3A
NOS1
MAP1LC3A
SPOCK2
NEURL1
SLC12A5
PACSIN1
BCAN
CCSAP
CA10
DYNC1I1
RCAN1
BDH1
ELAVL4
KCNJ9
CAMKV
FABP7
RBFOX3
DSCAM
CALB2
CNTNAP2
SLITRK4
MAP7D2
MAPT
FAM163B
PDGFA
CLEC2L

HPA
HPA:0090972
cerebellum;_granular_cells_-_cytoplasm/membrane[â‰¥Medium]
25
CNTN1
CAMK2B
ATP2B3
SPTB
FOSL2
NEURL1
DLGAP3
IDUA
SYNGR3
HCRTR2
CACNG8
KIRREL3
SPHKAP
AK5
CCSAP
DYNC1I1
SPTBN4
CAMKV
FABP7
DSCAM
CALB2
MAP7D2
SHTN1
PDGFA
S100A6

HPA
HPA:0091052
cerebellum;_synaptic_glomeruli_-_capsule[â‰¥Medium]
13
SYT7
CNTN1
SNCB
SH3GL2
TNR
PACSIN1
SLC6A11
BCAN
DYNC1I1
CALB2
CNTNAP2
MAPT
CLEC2L

HPA
HPA:0091063
cerebellum;_synaptic_glomeruli_-_core[High]
17
CAMK2B
ATP2B3
SPTB
SNCB
CDH7
PDGFB
SLC17A7
SH3GL2
BCAN
SCN2A
GABBR2
SLC4A10
GABRB2
RCAN1
BDH1
SYNPR
SV2B

KEGG
KEGG:04020
Calcium_signaling_pathway
33
CAMK1G
CAMK2B
ATP2B3
CAMK2A
ADCY2
P2RX5
NOS1
PDGFB
CACNA1I
NTSR1
CCKBR
CHRM3
PLCB2
EGF
FGF7
PRKACB
CASQ1
HTR2C
SLC25A4
PDE1C
ADCY8
ATP2B2
HTR5A
CACNA1D
RET
PRKCB
GRM5
ADRA1B
VEGFB
CHRM2
PDGFA
CACNA1E
RYR2

KEGG
KEGG:04713
Circadian_entrainment
20
CAMK2B
CAMK2A
ADCY2
NOS1
CACNA1I
RASD1
KCNJ5
PLCB2
PRKACB
GUCY1A2
GRIA1
ADCY8
CACNA1D
KCNJ9
KCNJ3
GUCY1A1
PRKCB
GNG8
RYR2
GRIN2B

KEGG
KEGG:05032
Morphine_addiction
19
GABRA3
PDE4A
ADCY2
GABRG2
KCNJ5
GABBR2
PRKACB
GABRA6
GABRB2
GABRA2
PDE1C
ADCY8
KCNJ9
KCNJ3
GABRB1
PRKCB
GNG8
GABRA5
PDE2A

KEGG
KEGG:04080
Neuroactive_ligand-receptor_interaction
39
TAC1
GABRA3
P2RX5
NTSR1
CRHR2
CCKBR
GABRG2
GRM4
VGF
CHRM3
GABBR2
HCRTR2
SSTR1
PTH2R
GABRA6
GABRB2
HTR2C
GNRH1
ADRA2A
GABRA2
GRIA1
SST
HTR5A
GABRB1
GRIK2
GRM5
ADRA1B
APLN
LYNX1
SSTR2
CHRM2
PENK
ADRA2C
GABRA5
PAQR9
GRM7
LPAR1
S1PR3
GRIN2B

KEGG
KEGG:04925
Aldosterone_synthesis_and_secretion
18
CAMK1G
CAMK2B
ATP2B3
CAMK2A
ADCY2
CACNA1I
KCNJ5
PLCB2
PRKACB
CREB5
ADCY8
ATP2B2
CACNA1D
PRKCB
KCNK9
NPPA
PRKD1
PDE2A

KEGG
KEGG:04727
GABAergic_synapse
17
GABRA3
ADCY2
GABRG2
SLC12A5
SLC6A11
GABBR2
PRKACB
GABRA6
GABRB2
GABRA2
ADCY8
CACNA1D
GABRB1
PRKCB
GNG8
GABRA5
SLC38A3

KEGG
KEGG:04921
Oxytocin_signaling_pathway
22
CACNG3
CAMK1G
CAMK2B
CAMK2A
CACNG5
ADCY2
KCNJ5
PLCB2
CACNG8
PRKACB
CACNA2D4
GUCY1A2
ADCY8
CACNA1D
RCAN1
KCNJ9
KCNJ3
GUCY1A1
PRKCB
NPPA
KCNJ12
RYR2

KEGG
KEGG:04723
Retrograde_endocannabinoid_signaling
21
GABRA3
MGLL
ADCY2
SLC17A7
GABRG2
KCNJ5
PLCB2
PRKACB
GABRA6
GABRB2
GABRA2
GRIA1
ADCY8
CACNA1D
KCNJ9
KCNJ3
GABRB1
PRKCB
GNG8
GRM5
GABRA5

KEGG
KEGG:04540
Gap_junction
15
ADCY2
PDGFB
TUBB4A
PLCB2
EGF
PRKACB
HTR2C
GUCY1A2
ADCY8
GJD2
GUCY1A1
PRKCB
GRM5
PDGFA
LPAR1

KEGG
KEGG:04024
cAMP_signaling_pathway
25
CAMK2B
PDE4A
ATP2B3
CNGB1
CAMK2A
ADCY2
RAPGEF3
HCN2
AMH
CRHR2
PPP1R1B
GABBR2
SSTR1
PRKACB
CREB5
GRIA1
ADCY8
SST
ATP2B2
CACNA1D
NPPA
SSTR2
CHRM2
RYR2
GRIN2B

KEGG	KEGG	KEGG	KEGG	KEGG	KEGG	KEGG
KEGG:04261	KEGG:05033	KEGG:04929	KEGG:04725	KEGG:04724	KEGG:04728	KEGG:04726
Adrenergic_signaling_in_cardiomyocytes	Nicotine_addiction	GnRH_secretion	Cholinergic_synapse	Glutamatergic_synapse	Dopaminergic_synapse	Serotonergic_synapse
20	10	12	16	16	17	15
CACNG3	GABRA3	TRPC5	CAMK2B	ADCY2	CAMK2B	RAPGEF3
CAMK2B	SLC17A7	HCN2	CAMK2A	SLC17A7	CAMK2A	KCNJ5
ATP2B3	GABRG2	CACNA1I	ADCY2	GRM4	PPP2R2C	PLCB2
CAMK2A	GABRA6	SPP1	ACHE	PLCB2	KCNJ5	PRKACB
PPP2R2C	GABRB2	KCNJ5	CHRM3	PRKACB	CALY	GABRB2
CACNG5	GABRA2	GABBR2	PLCB2	GRIA1	PPP1R1B	HTR2C
ADCY2	GRIA1	PLCB2	PRKACB	ADCY8	PLCB2	HTR5A
RAPGEF3	GABRB1	GNRH1	CREB5	CACNA1D	PRKACB	CACNA1D
MYH7	GABRA5	CACNA1D	ADCY8	KCNJ3	CREB5	KCNJ9
SCN7A	GRIN2B	KCNJ9	CACNA1D	GRIK2	GRIA1	KCNJ3
PLCB2		KCNJ3	KCNJ3	PRKCB	CACNA1D	GABRB1
CACNG8		PRKCB	PRKCB	GNG8	KCNJ9	PRKCB
PRKACB			GNG8	GRM5	KCNJ3	GNG8
CREB5			CHRM2	SLC38A3	PRKCB	CYP4X1
CACNA2D4			KCNJ12	GRM7	GNG8	MAOA
ADCY8			KCNQ5	GRIN2B	MAOA	
ATP2B2					GRIN2B	
CACNA1D						
ADRA1B						
RYR2						

KEGG	KEGG	KEGG	KEGG	KEGG	KEGG
KEGG:04512	KEGG:04971	KEGG:04010	KEGG:04022	KEGG:04742	KEGG:04911
ECM-receptor_interaction	Gastric_acid_secretion	MAPK_signaling_pathway	cGMP-PKG_signaling_pathway	Taste_transduction	Insulin_secretion
13	12	26	18	12	12
ITGA3	CAMK2B	CACNG3	ATP2B3	GABRA3	CAMK2B
TNC	CAMK2A	RASGRF1	CNGB1	GRM4	CAMK2A
COL9A3	ADCY2	RASGRP2	ADCY2	CHRM3	ADCY2
TNR	CCKBR	CACNG5	MYH7	SCN2A	GCK
SPP1	CHRM3	PDGFB	PLCB2	GABBR2	CHRM3
COL6A3	PLCB2	CACNA1I	CREB5	PLCB2	PLCB2
ITGA2	PRKACB	PTPN5	ADRA2A	PRKACB	PRKACB
NPNT	ADCY8	TEK	SLC25A4	GABRA6	CREB5
SV2B	SST	DUSP4	GUCY1A2	GABRA2	ADCY8
COL4A5	PRKCB	FLT3	ADCY8	PDE1C	CACNA1D
RELN	KCNJ10	MYC	ATP2B2	ADCY8	PRKCB
LAMB3	SSTR2	DUSP5	CACNA1D	GABRA5	RYR2
COL4A6		EGF	GUCY1A1		
		DUSP6	IRS1		
		FGF7	ADRA1B		
		CACNG8	NPPA		
		PRKACB	ADRA2C		
		CACNA2D4	PDE2A		
		CACNA1D			
		PRKCB			
		RASGRP1			
		MYD88			
		VEGFB			
		MAPT			
		PDGFA			
		CACNA1E			

MIRNA
MIRNA:hsa-miR-335-5p
hsa-miR-335-5p
149
PLXND1
YBX2
ETV1
CEACAM21
MATK
CYB561
PLEKHB1
TNC
LMO3
PTPRU
PDE4A
MPPED2
SPTB
CNGB1
SNCB
RAPGEF3
PITPNM2
SEL1L3
COL9A3
SLCO4A1
NTSR1
RSPO4
MAP1LC3A
CELF4
CDH20
KLF8
SYT17
SFRP1
PLAT
SLC17A7
CRHR2
PPP1R17
GCK
RASD1
FAM149A
MGP
TPD52L1
LOX
NPR3
RBP1
IGSF21
CDK18
PLPPR5
PLPPR4
NRP2
SPP1
PPL
GALNT12
FLT3
TOX2
CHST8
GRM4
CPNE5
BMP2
F10
PLEKHG3
FGD3
IDUA
SYNGR3
GCH1
CHRM3
BEX2
PDZD2
CD101
RERG
PIWIL4
RNASEL
MYC
SPTBN5
DUSP5
SHISAL1
DUSP6
MDGA2
TMC6
CACNG8
CASQ1
TMEFF2
GRIP2
MUC4
RASGEF1C
ARHGAP36
MAL2
CACNA2D4
GUCY1A2
JAKMIP1
CCSAP
NCAM2
GRIA1
SORCS3
TSC22D3
CABP1
DYNC1I1
TNNI1
RCAN1
ACE
SLC37A1
NTN3
FBLIM1
KCNJ9
SYNPR
ITGA2
HEY1
TMEM130
TVP23A
GNG8
TMC8
RAB26
PHYHIP
GRM5
ROR2
KCNK9
REPS2
MUC3A
EMX2
GPR37
TANC2
SOSTDC1
NEUROD2
CALB2
FUT9
RASGRP1
LY6H
FZD8
KCTD12
ZNF572
RPH3AL
CHRM2
TMEM132C
KCNK4
PCBP3
MARCHF11
FAM131C
PDE2A
COL4A5
SMTNL2
NKAIN2
INSYN2A
PTPRT
CPNE4
DCHS2
OGDHL
CYRIA
RYR2
RASGEF1A
RIPPLY2
MAFB
COL15A1
TSTD1
PLXNA4

REAC	REAC	REAC	REAC
REAC:R-HSA-112316	REAC:R-HSA-112315	REAC:R-HSA-112314	REAC:R-HSA-977443
Neuronal_System	Transmission_across_Chemical_Synapses	Neurotransmitter_receptors_and_postsynaptic_signal_transmission	GABA_receptor_activation
62	37	30	16
CACNG3	CACNG3	CACNG3	GABRA3
SYT7	GABRA3	GABRA3	ADCY2
GABRA3	RASGRF1	RASGRF1	GABRG2
RASGRF1	CAMK2B	CAMK2B	KCNJ5
CAMK2B	CAMK2A	CAMK2A	GABBR2
CAMK2A	ADCY2	ADCY2	GABRA6
ADCY2	ACHE	TUBB4A	GABRB2
ACHE	TUBB4A	GABRG2	GABRA2
HCN2	SLC17A7	KCNJ5	ADCY8
TUBB4A	GABRG2	GABBR2	KCNJ9
SLC17A7	KCNJ5	PLCB2	KCNJ3
SYT10	SLC6A11	CACNG8	GABRB1
GABRG2	GABBR2	PRKACB	GNG8
DLGAP3	PLCB2	GRIP2	KCNJ10
KCNJ5	CACNG8	GABRA6	KCNJ12
KCNA5	PRKACB	GABRB2	GABRA5
SLC6A11	GRIP2	GABRA2	
GABBR2	GABRA6	GRIA1	
PLCB2	GABRB2	ADCY8	
KCNH5	GABRA2	KCNJ9	
CACNG8	GRIA1	KCNJ3	
PRKACB	ADCY8	GABRB1	
GRIP2	SYN2	GRIK2	
GABRA6	KCNJ9	PRKCB	
GABRB2	KCNJ3	GNG8	
NLGN4X	GABRB1	KCNJ10	
KCNA6	GRIK2	AKAP5	
GABRA2	PRKCB	KCNJ12	
GRIA1	GNG8	GABRA5	
ADCY8	CPLX1	GRIN2B	
SYN2	KCNJ10		
GJD2	AKAP5		
KCNJ9	KCNJ12		
LRRTM1	GABRA5		
KCNF1	MAOA		
KCNJ3	CACNA1E		
GABRB1	GRIN2B		
GRIK2			
KCNC2			
PRKCB			
GNG8			
GRM5			
CPLX1			
IL1RAPL1			
KCNK9			
KCND3			
KCNA3			
KCNJ10			
SLITRK4			
AKAP5			
KCNK4			
BEGAIN			
KCNJ12			
SLITRK6			
KCNQ5			
GABRA5			
IL1RAPL2			
MAOA			
DLGAP2			
CACNA1E			
LRRTM3			
GRIN2B			

REAC	REAC	REAC	REAC
REAC:R-HSA-372790	REAC:R-HSA-1296071	REAC:R-HSA-388396	REAC:R-HSA-6794362
Signaling_by_GPCR	Potassium_Channels	GPCR_downstream_signalling	Protein-protein_interactions_at_synapses
59	18	51	15
TAC1	HCN2	TAC1	SYT7
XK	KCNJ5	CAMK2B	SYT10
CAMK2B	KCNA5	DGKG	DLGAP3
DGKG	GABBR2	PDE4A	NLGN4X
PDE4A	KCNH5	NGEF	GRIA1
NGEF	KCNA6	RASGRP2	LRRTM1
RASGRP2	KCNJ9	TRPC7	GRM5
TRPC7	KCNF1	CAMK2A	IL1RAPL1
CAMK2A	KCNJ3	MGLL	SLITRK4
MGLL	KCNC2	ADCY2	BEGAIN
ADCY2	GNG8	NTSR1	SLITRK6
WNT11	KCNK9	CRHR2	IL1RAPL2
NTSR1	KCND3	CCKBR	DLGAP2
CRHR2	KCNA3	HBEGF	LRRTM3
CCKBR	KCNJ10	GRM4	GRIN2B
HBEGF	KCNK4	MCF2L	
PLPPR5	KCNJ12	FGD3	
PLPPR4	KCNQ5	RAMP2	
GRM4		PPP1R1B	
MCF2L		CHRM3	
FGD3		GPR45	
RAMP2		DGKB	
PPP1R1B		GABBR2	
CHRM3		HCRTR2	
GPR45		PLCB2	
DGKB		SSTR1	
GABBR2		PRKACB	
HCRTR2		PTH2R	
PLCB2		HTR2C	
SSTR1		GNRH1	
PRKACB		ADRA2A	
PTH2R		PDE1C	
HTR2C		ADCY8	
GNRH1		SST	
PLPPR1		HTR5A	
ADRA2A		PRKCB	
PDE1C		GNG8	
ADCY8		GRM5	
SST		ADRA1B	
HTR5A		GPR37	
WNT4		APLN	
FZD6		RASGRP1	
PRKCB		SSTR2	
GNG8		CHRM2	
GRM5		PENK	
ADRA1B		RGS6	
GPR37		ADRA2C	
APLN		PDE2A	
RASGRP1		GRM7	
FZD8		LPAR1	
SSTR2		S1PR3	
CHRM2			
PENK			
RGS6			
ADRA2C			
PDE2A			
GRM7			
LPAR1			
S1PR3			

REAC	REAC	REAC	REAC
REAC:R-HSA-422475	REAC:R-HSA-9675108	REAC:R-HSA-1474228	REAC:R-HSA-5576891
Axon_guidance	Nervous_system_development	Degradation_of_the_extracellular_matrix	Cardiac_conduction
45	46	19	18
PLXND1	PLXND1	COL19A1	CAMK2B
CNTN1	CNTN1	COL16A1	ATP2B3
NGEF	NGEF	COL9A3	CAMK2A
TRPC7	TRPC7	MMP11	NOS1
SPTB	SPTB	MMP9	FGF12
TRPC5	TRPC5	SPP1	KCNA5
SEMA3A	SEMA3A	BCAN	SCN2A
DCX	DCX	ADAMTS8	SCN7A
COL9A3	COL9A3	ADAMTS5	CACNG8
CACNA1I	CACNA1I	SCUBE1	CASQ1
MMP9	MMP9	COL26A1	SCN2B
DOK5	DOK5	COL6A3	ATP2B2
TUBB4A	TUBB4A	ADAMTS9	KCNK9
LHX2	LHX2	COL4A5	KCND3
SH3GL2	SH3GL2	COL25A1	NPPA
SEMA5A	SEMA5A	SPOCK3	KCNK4
UNC5A	UNC5A	LAMB3	KCNJ12
NRP2	NRP2	COL4A6	RYR2
EFNB2	EFNB2	COL15A1	
EPHA7	EPHA7		
AGAP2	AGAP2		
SCN2A	SCN2A		
SCN7A	SCN7A		
SPTBN5	SPTBN5		
PRKACB	PRKACB		
SCN2B	SCN2B		
CACNA1D	CACNA1D		
SPTBN4	SPTBN4		
COL6A3	COL6A3		
ABLIM2	ABLIM2		
ITGA2	ITGA2		
RET	RET		
GFRA2	GFRA2		
SEMA3E	SEMA3E		
DSCAM	DSCAM		
PRNP	PRNP		
DSCAML1	DSCAML1		
AKAP5	AKAP5		
ROBO2	ROBO2		
SHTN1	SHTN1		
COL4A5	COL4A5		
RELN	RELN		
PLXNA4	MBP		
PSMB9	PLXNA4		
GRIN2B	PSMB9		
	GRIN2B		

REAC	REAC	REAC	REAC
REAC:R-HSA-1474244	REAC:R-HSA-399719	REAC:R-HSA-399721	REAC:R-HSA-422356
Extracellular_matrix_organization	Trafficking_of_AMPA_receptors	Glutamate_binding_activation_of_AMPA_receptors_and_synaptic_plasticity	Regulation_of_insulin_secretion
29	8	8	13
ITGA3	CACNG3	CACNG3	ACSL4
TNC	CAMK2B	CAMK2B	RAPGEF3
COL19A1	CAMK2A	CAMK2A	CHRM3
COL16A1	CACNG8	CACNG8	PLCB2
COL9A3	GRIP2	GRIP2	PRKACB
MMP11	GRIA1	GRIA1	ADRA2A
PDGFB	PRKCB	PRKCB	ADCY8
MMP9	AKAP5	AKAP5	CACNA1D
LOX			KCNC2
EFEMP1			GNG8
TNR			AKAP5
SPP1			ADRA2C
BMP2			CACNA1E
BCAN			
ADAMTS8			
ADAMTS5			
SCUBE1			
COL26A1			
VCAM1			
COL6A3			
ADAMTS9			
ITGA2			
COL4A5			
COL25A1			
SPOCK3			
LAMB3			
PDGFA			
COL4A6			
COL15A1			

REAC
REAC:R-HSA-162582
Signal_Transduction
135
PLXND1
ITGA3
TAC1
MATK
CNTN1
XK
RASGRF1
CAMK2B
DGKG
PTPRU
TLE2
PDE4A
NGEF
RASGRP2
TRPC7
SPTB
CAMK2A
MGLL
ADCY2
RAPGEF3
WNT11
COL9A3
PDGFB
MMP9
NTSR1
RSPO4
CHRDL1
RHOV
SFRP1
PLAT
TUBB4A
AMH
CRHR2
SH3GL2
SHB
NEURL1
CCKBR
HBEGF
GABRG2
HES1
PLPPR5
PLPPR4
NRP2
SPP1
TEK
EGR1
DUSP4
FLT3
WIPF3
RAB9B
GRM4
BMP2
MCF2L
RHOJ
PLEKHG3
FGD3
VGF
RAMP2
PPP1R1B
KHDRBS3
PPARG
CHRM3
GPR45
DGKB
GABBR2
MYC
HCRTR2
ARHGAP20
PLCB2
SPTBN5
DUSP5
EGF
DUSP6
SSTR1
FGF7
PRKACB
PTH2R
GABRB2
HTR2C
GNRH1
PLPPR1
ADRA2A
PTPRO
RIT2
PDE1C
ADCY8
SST
HTR5A
NRG2
DYNC1I1
SPTBN4
WNT4
GABRB1
COL6A3
ITGA2
FABP7
HEY1
FZD6
RET
SPINT1
PRKCB
GNG8
GFRA2
GRM5
IRS1
ROR2
PCDH7
ADRA1B
GPR37
ALK
APLN
CDK5R2
RASGRP1
MYD88
VEGFB
FZD8
SSTR2
CHRM2
PENK
RGS6
NOG
ADRA2C
FMNL1
PDE2A
COL4A5
NHS
GRM7
LAMB3
H4C16
LPAR1
RASGEF1A
FOXD6
S1PR3
PSMB9
GRIN2B

REAC	REAC
REAC:R-HSA-397014	REAC:R-HSA-5173105
Muscle_contraction	O-linked_glycosylation
22	15
CAMK2B	SEMA5A
ATP2B3	GALNT12
CAMK2A	GALNT15
NOS1	ADAMTS8
CACNA1I	MUC4
FGF12	ADAMTS5
KCNA5	GALNT14
SCN2A	ADAMTS9
SCN7A	ADAMTS15
CACNG8	MUC3A
CASQ1	GALNTL6
SCN2B	GALNT9
GUCY1A2	GALNT17
ATP2B2	GCNT1
TNNI1	ADAMTSL2
GUCY1A1	
KCNK9	
KCND3	
NPPA	
KCNK4	
KCNJ12	
RYR2	

TF<br>TF:M12722_1<br>Factor:_ZXDL;_motif:_GSGSCNNGGGMRGCNCCGGGS;_match_class:_1	TF<br>TF:M07289_1<br>Factor:_GKLF;_motif:_NNNRGGNGNGGSN;_match_class:_1	TF<br>TF:M00986_1<br>Factor:_Churchill;_motif:_CGGGNN;_match_class:_1	TF<br>TF:M09984<br>Factor:_MAZ;_motif:_GGGGGAGGGGGNGRGRRRGNRG	TF<br>TF:M01104_1<br>Factor:_MOVO-B;_motif:_GNGGGGG;_match_class:_1
379	526	516	484	324
HS3ST1	PLXND1	PLXND1	HS3ST1	PLXND1
PLXND1	ITGA3	ITGA3	PLXND1	YBX2
YBX2	YBX2	YBX2	ITGA3	CACNG3
GAS7	CACNG3	CACNG3	YBX2	GAS7
MATK	TAC1	ETV1	TAC1	MATK
CYB561	ETV1	GAS7	ETV1	CYB561
SYT7	PNPLA4	MATK	CEACAM21	SLC6A7
SLC7A14	GAS7	CYB561	GAS7	SYT7
MRI1	MATK	SLC6A7	MATK	SLC7A14
JADE2	CYB561	SYT7	CYB561	JADE2
FSTL4	NFIX	SLC7A14	NFIX	CCDC85A
PTPRN	SLC6A7	PLEKHB1	SLC6A7	GYG2
CCDC85A	SYT7	MRI1	SYT7	CAMK2B
GYG2	SLC7A14	CDH10	SLC7A14	PTPRU
RASGRF1	CNTN1	TNC	CNTN1	LZTS1
CAMK2B	PLEKHB1	CAPG	PLEKHB1	SLC9A3R2
SLC9A3R2	CDH10	JADE2	MRI1	TLE2
TLE2	CAPG	FSTL4	CDH10	PDE4A
PDE4A	TG	PTPRN	TG	NGEF
NGEF	JADE2	CCDC85A	JADE2	MPPED2
[illegible]	[illegible]	[illegible]	[illegible]	[illegible]

TF<br>TF:M00695_1<br>Factor:_ETF;_motif:_GVGGMGG;_match_class:_1	TF<br>TF:M09984_1<br>Factor:_MAZ;_motif:_GGGGGAGGGGGNGRGRRRGNRG;_match_class:_1	TF<br>TF:M01072<br>Factor:_HIC1;_motif:_NSNNNNTGCCCSSNN	TF<br>TF:M12636<br>Factor:_MLLT10;_motif:_CYNNCCNNGGNGCTG	TF<br>TF:M07040_1<br>Factor:_GKLF;_motif:_NNRRGRRNGNSNNN;_match_class:_1
379	318	252	463	415
HS3ST1	HS3ST1	HS3ST1	HS3ST1	HS3ST1
PLXND1	PLXND1	PLXND1	ABCB5	ITGA3
YBX2	ITGA3	ITGA3	YBX2	YBX2
GAS7	YBX2	YBX2	CACNG3	CACNG3
MATK	ETV1	TAC1	ETV1	TAC1
CYB561	MATK	SYT7	CEACAM21	ETV1
SYT7	CYB561	SLC7A14	GAS7	MATK
MRI1	NFIX	PLEKHB1	CYB561	CAMK1G
RAB27B	SYT7	TNC	SLC6A7	NFIX
JADE2	PLEKHB1	XK	SYT7	SLC6A7
XK	MRI1	FSTL4	SLC7A14	SYT7
FSTL4	CDH10	CCDC85A	MRI1	SLC7A14
CCDC85A	JADE2	GYG2	RAB27B	PLEKHB1
GYG2	XK	RASGRF1	TNC	MRI1
CAMK2B	LMO3	SLC9A3R2	JADE2	CDH10
SLC9A3R2	PTPRN	TLE2	XK	CAPG
TLE2	GYG2	NGEF	PTPRN	JADE2
NGEF	F7	MPPED2	GYG2	PTPRN
MPPED2	RASGRF1	ATP2B3	RASGRF1	GYG2
KIF26A	CAMK2B	ACSL4	SLC9A3R2	F7
[illegible]	[illegible]	[illegible]	[illegible]	[illegible]

TF<br>TF:M00986<br>Factor:_Churchill;_motif:_CGGGNN	TF<br>TF:M07436<br>Factor:_WT1;_motif:_NNGGGNGGGSGN	TF<br>TF:M10432<br>Factor:_MAZ;_motif:_GGGMGGGGS	TF<br>TF:M01857_1<br>Factor:_AP-2alpha;_motif:_NGCCYSNNGSN;_match_class:_1	TF<br>TF:M01303<br>Factor:_SP1;_motif:_GGGGYGGGGNS
626	348	457	267	403
HS3ST1	YBX2	PLXND1	PLXND1	PLXND1
PLXND1	PNPLA4	ITGA3	ITGA3	ITGA3
ITGA3	MATK	YBX2	YBX2	YBX2
YBX2	CYB561	CACNG3	MATK	CACNG3
CACNG3	NFIX	TAC1	CYB561	TAC1
TAC1	SLC6A7	ETV1	SYT7	ETV1
ETV1	SYT7	GAS7	CAPG	GAS7
GAS7	SLC7A14	MATK	JADE2	MATK
MATK	CAPG	CYB561	FSTL4	PAX6
PAX6	JADE2	NFIX	CCDC85A	CYB561
CYB561	PTPRN	SLC6A7	GYG2	SLC6A7
NFIX	CCDC85A	SYT7	CAMK2B	SLC7A14
SLC6A7	GYG2	SLC7A14	DGKG	CNTN1
SYT7	RASGRF1	PLEKHB1	TLE2	PLEKHB1
SLC7A14	CAMK2B	CAPG	MPPED2	MRI1
PLEKHB1	DGKG	JADE2	KIF26A	CDH10
MRI1	SLC9A3R2	LMO3	ATP2B3	CAPG
CDH10	TLE2	PTPRN	RASGRP2	TG
TNC	PDE4A	GYG2	TRPC5	JADE2
CAPG	MPPED2	RASGRF1	NUAK1	XK
[illegible]	[illegible]	[illegible]	[illegible]	[illegible]

TF<br>TF:M07322_1<br>Factor:_HSF4;_motif:_CTGCMRN;_match_class:_1	TF<br>TF:M01104<br>Factor:_MOVO-B;_motif:_GNGGGGG	TF<br>TF:M12722<br>Factor:_ZXDL;_motif:_GSGSCNNGGGMRGCNCCGGGS	TF<br>TF:M03876_1<br>Factor:_Kaiso;_motif:_GCMGGGRGCRGS;_match_class:_1	TF<br>TF:M00333_1<br>Factor:_ZF5;_motif:_NRNGNGCGCGCWN;_match_class:_1
542	491	501	447	565
HS3ST1	PLXND1	HS3ST1	HS3ST1	HS3ST1
PLXND1	YBX2	PLXND1	PLXND1	PLXND1
ITGA3	CACNG3	YBX2	ITGA3	ITGA3
YBX2	GAS7	TAC1	YBX2	YBX2
CACNG3	MATK	GAS7	CEACAM21	CACNG3
TAC1	CYB561	MATK	MATK	TAC1
GAS7	NFIX	PAX6	CYB561	PNPLA4
PAX6	SLC6A7	CYB561	SLC6A7	CEACAM21
CAMK1G	SYT7	NFIX	SYT7	GAS7
CYB561	SLC7A14	SYT7	SLC7A14	MATK
NFIX	CDH10	SLC7A14	CNTN1	PAX6
TENM1	TNC	CNTN1	JADE2	CYB561
SYT7	CAPG	PLEKHB1	FSTL4	NFIX
SLC7A14	JADE2	MRI1	PTPRN	SLC6A7
CNTN1	FSTL4	CAPG	CCDC85A	SYT7
PLEKHB1	PTPRN	JADE2	GYG2	SLC7A14
MRI1	CCDC85A	FSTL4	RASGRF1	MRI1
TNC	GYG2	PTPRN	CAMK2B	CDH10
JADE2	CAMK2B	CCDC85A	DGKG	RAB27B
FSTL4	DGKG	GYG2	RIMBP2	TNC
[illegible]	[illegible]	[illegible]	[illegible]	[illegible]

CACNA1E
RYR2
LRRTM3
RASGEF1A
STUM
FOXO6
TCEAL5
TCEAL6
MAFB
C2orf72
DPF3
DENND1C
C22orf42
CPLX3
C10orf105
UBE2QL1
HMX1
TSTD1
SP9
PLXNA4
FNDC10
AJM1
SHISA9
PSMB9
SMKR1
CEBPA
TRNP1
SALL3
ZNF578
TMEM179
FRRS1L
GOLGA6L7
ZIM2
SRXN1
DOC2B
GRIN2B

DENND1B
FAM24B
CPLX3
EML6
UBE2QL1
HMX1
TSTD1
SP9
PLXNA4
CENPVL1
CENPVL3
FNDC10
AJM1
TMEM238
CLEC2L
SHISA9
SMKR1
CEBPA
TRNP1
SALL3
TMEM179
FRRS1L
ZIM2
SRXN1
DOC2B
GRIN2B

SSTR2
SHISA2
FAM83H
ZNF572
RPH3AL
CHRM2
PENK
TMEM132C
TMEM132E
DDX60L
GPR88
GPR3
ADGRB1
FAM181B
KCNK4
GALNT9
GPC6
FAM43B
CALN1
CTNNA3
PCBP3
MARCHF11
EFHC2
NOG
OPCML
ADRA2C
ACOT1
PRKD1
MAP7D2
PABPC1L2B
CLDN6
TCEAL2
FMNL1
ROBO2
SLC24A3
INPP5J
GALNT17
SV2B
FAM131C
AHNAK2
C11orf87
KCNQ5
ADAMTSL5
GABRA5
PDE2A
MPPED1
MAPT
VSTM2B
SHTN1
GCNT1
C11orf96
ATP13A5
C17orf97
COL4A5
NHS
SMTNL2
ENO4
SLC38A3
COL25A1
NKAIN2
PAQR9
VWC2
SHISA6
BEND4
FAM78B
INSYN2A
RELN
MAOA
PTPRT
SPOCK3
GRM7
CPNE4
FUT4
SLC30A10
LAMB3
FAM163B
SLC6A17
ADGRA1
DCHS2
OGDHL
PDGFA
COL4A6
DPP4
H4C16
ADAMTSL2
CYRIA
S100A6
MBP
DLGAP2
CHSY3
LPAR1
TMEM229B
CACNA1E
RYR2
RASGEF1A
STUM
RIPPLY2
FOXO6
TCEAL6
MAFB
C2orf72
COL15A1
DPF3
DENND1C
C22orf42
DENND1B
FAM24B
CPLX3
S1PR3
EML6
UBE2QL1
HMX1
TSTD1
SP9
PLXNA4
CENPVL1
CENPVL3
FNDC10
AJM1
TMEM238
CLEC2L
SHISA9
PSMB9
SMKR1
CEBPA
SELENOP
TRNP1
CTSO
SALL3
TMEM179
FRRS1L
GOLGA6L7
ZIM2
SRXN1
DOC2B
GRIN2B

DPP4
S100A10
H4C16
ADAMTSL2
CYRIA
S100A6
MBP
DLGAP2
CHSY3
LPAR1
TMEM229B
CACNA1E
RYR2
BRINP2
RASGEF1A
STUM
RIPPLY2
FOXO6
TCEAL5
TCEAL6
MAFB
C2orf72
COL15A1
DPF3
DENND1C
C22orf42
DENND1B
FAM24B
S1PR3
EML6
C10orf105
HMX1
TSTD1
SP9
RAB6C
CENPVL1
CENPVL3
FNDC10
AJM1
RAB6D
TMEM238
SHISA9
OR2W3
PSMB9
SMKR1
CEBPA
SELENOP
TMEM179
FRRS1L
ZIM2
SRXN1
DOC2B

GRIN2B

PSMB9
SMKR1
CEBPA
TRNP1
SALL3
TMEM179
FRRS1L
ZIM2
SRXN1
DOC2B
GRIN2B

ENO4
SLC38A3
COL25A1
NKAIN2
PAQR9
VWC2
SHISA6
BEND4
FAM78B
INSYN2A
RELN
IL1RAPL2
MAOA
PTPRT
SPOCK3
GRM7
CPNE4
FUT4
SLC30A10
FAM163B
SLC6A17
ADGRA1
DCHS2
OGDHL
PDGFA
DPP4
H4C16
ADAMTSL2
CYRIA
MBP
CHSY3
LPAR1
TMEM229B
CACNA1E
RYR2
BRINP2
RASGEF1A
STUM
RIPPLY2
FOXO6
TCEAL5
TCEAL6
MAFB
C2orf72
COL15A1
DPF3
DENND1B
FAM24B
CPLX3
EML6
UBE2QL1
HMX1
TSTD1
SP9
PLXNA4
RAB6C
CENPVL1
CENPVL3
FNDC10
RAB6D
TMEM238
CLEC2L
SHISA9
PSMB9
SMKR1
CEBPA
TRNP1
CTSO
SALL3
TMEM179
FRRS1L
ZIM2
SRXN1
DOC2B
GRIN2B

TF
TF:M02036_1
Factor:_WT1;_motif:_CGCCCCCNCN;_match_class:_1

TF
TF:M10111
Factor:_ZF5;_motif:_NGAGCGCGC

TF
TF:M08867_1
Factor:_AP2;_motif:_GCCYGSGGSN;_match_class:_1

TF
TF:M12464
Factor:_ZNF148;_motif:_NNCCCCTCCCCC

TF
TF:M06948
Factor:_Sp2;_motif:_TGGGCGCGCCCA

TF
TF:M00982
Factor:_KROX;_motif:_CCCGCCCCCRCCCC

TF
TF:M07354_1
Factor:_Egr-1;_motif:_GCGGGGGCGG;_match_class:_1

TF
TF:M12694_1
Factor:_ZFP14;_motif:_SCNNYCCNGNNSCTSCNC;_match_class:_1

TF
TF:M08878
Factor:_EGR;_motif:_CGCCCCCGCNN

TF
TF:M01857
Factor:_AP-2alpha;_motif:_NGCCYSNNGGN

TF
TF:M12660
Factor:_SATB2;_motif:_GSCGCTGTCCNNGGTGCTGN

TF
TF:M12704
Factor:_ZNF432;_motif:_NCAGNRCCNSRGRCAGC

TF
TF:M10107
Factor:_DB1;_motif:_GGRRRRGRRGGAGGGGGNGRRR

TF
TF:M10432_1
Factor:_MAZ;_motif:_GGGMGGGGS;_match_class:_1

TF
TF:M10026
Factor:_PATZ;_motif:_GGGGNGGGGGMKGGRRNGGNRN

TF
TF:M01873
Factor:_Egr-1;_motif:_GCGGGGGCGG

TF
TF:M02036
Factor:_WT1;_motif:_CGCCCCCNCN

TF
TF:M07486
Factor:_ZNF300;_motif:_SWGGGGGSN

TF
TF:M00982_1
Factor:_KROX;_motif:_CCCGCCCCCRCCCC;_match_class:_1

TF
TF:M01199
Factor:_RNF96;_motif:_BCCCGCRGCC

TF
TF:M00649
Factor:_MAZ;_motif:_GGGGAGGG

TF
TF:M04863_1
Factor:_TF3C-beta;_motif:_CCNGGAGGGCTTCCTGGAGGAG;_match_class:_1

[illegible]

HS3ST1	TAC1	PLXND1	ITGA3	PLXND1	PLXND1	PLXND1	HS3ST1	PLXND1	HS3ST1	HS3ST1	HS3ST1	HS3ST1	YBX2	HS3ST1	PLXND1	HS3ST1	HS3ST1	PLXND1	PLXND1	HS3ST1	PLXND1
PLXND1	GAS7	ITGA3	YBX2	ITGA3	YBX2	YBX2	ITGA3	YBX2	PLXND1	PLXND1	PLXND1	PLXND1	CACNG3	PLXND1	YBX2	PLXND1	PLXND1	YBX2	YBX2	PLXND1	ITGA3
YBX2	CYB561	YBX2	TAC1	YBX2	CACNG3	GAS7	YBX2	TAC1	ITGA3	ABCB5	ITGA3	YBX2	MATK	ITGA3	PNPLA4	YBX2	ITGA3	GAS7	GAS7	ITGA3	YBX2
TAC1	XK	GAS7	CEACAM21	CACNG3	TAC1	CYB561	GAS7	PNPLA4	YBX2	YBX2	YBX2	MATK	CYB561	YBX2	GAS7	CACNG3	YBX2	CYB561	MATK	YBX2	TAC1
GAS7	RASGRF1	MATK	MATK	TAC1	ETV1	SLC6A7	MATK	GAS7	TAC1	CACNG3	TAC1	CYB561	SLC6A7	CACNG3	CYB561	TAC1	CACNG3	SLC6A7	CYB561	CACNG3	PNPLA4
MATK	CAMK2B	CYB561	CAMK1G	GAS7	PNPLA4	JADE2	CYB561	CYB561	CEACAM21	CEACAM21	ETV1	NFIX	SYT7	PNPLA4	NFIX	PNPLA4	TAC1	TNC	SLC6A7	TAC1	GAS7
CYB561	PTPRU	SYT7	NFIX	MATK	GAS7	CAMK2B	NFIX	NFIX	GAS7	CYB561	CEACAM21	CDH10	PLEKHB1	CEACAM21	SLC6A7	GAS7	PNPLA4	JADE2	SYT7	ETV1	MATK
SLC6A7	MPPED2	PLEKHB1	SLC6A7	CAMK1G	MATK	PTPRU	SLC7A14	SLC6A7	MATK	CNTN1	GAS7	CAPG	CAPG	GAS7	TNC	MATK	CEACAM21	CCDC85A	MRI1	CEACAM21	CYB561
SYT7	KIF26A	JADE2	SYT7	CYB561	PAX6	SLC9A3R2	JADE2	TNC	CYB561	RAB27B	CYB561	JADE2	JADE2	MATK	TG	CYB561	GAS7	GYG2	RAB27B	MATK	NFIX
TNC	ATP2B3	FSTL4	SLC7A14	NFIX	CYB561	TLE2	FSTL4	TG	SYT7	XK	SYT7	LMO3	PTPRN	CYB561	JADE2	NFIX	MATK	CAMK2B	JADE2	CYB561	SLC6A7
CAPG	ACSL4	PTPRN	CNTN1	SLC6A7	NFIX	PDE4A	RASGRF1	JADE2	SLC7A14	FSTL4	PLEKHB1	GYG2	GYG2	NFIX	XK	SLC6A7	CYB561	SLC9A3R2	XK	NFIX	SYT7
JADE2	TRPC7	CCDC85A	PLEKHB1	SYT7	SLC6A7	NGEF	CAMK2B	XK	CNTN1	PTPRN	TG	F7	CAMK2B	SLC6A7	CCDC85A	SYT7	NFIX	TLE2	FSTL4	SLC6A7	SLC7A14
CCDC85A	SPTB	GYG2	CDH10	SLC7A14	TNC	MPPED2	LZTS1	CCDC85A	PLEKHB1	CCDC85A	JADE2	CAMK2B	SLC9A3R2	MRI1	GYG2	SLC7A14	SLC6A7	NGEF	CCDC85A	SYT7	MRI1
GYG2	TRHDE	CAMK2B	CAPG	CAPG	CAPG	KIF26A	TLE2	GYG2	CAPG	GYG2	FSTL4	PTPRU	TLE2	CDH10	CAMK2B	TNC	SYT7	KIF26A	GYG2	SLC7A14	RAB27B
CAMK2B	PPP2R2C	DGKG	JADE2	JADE2	TG	ACSL4	NGEF	RASGRF1	JADE2	RASGRF1	PTPRN	RIMBP2	PDE4A	TG	DGKG	CAPG	SLC7A14	ACSL4	RASGRF1	CNTN1	JADE2
PTPRU	SNCB	RIMBP2	PTPRN	FSTL4	JADE2	RASGRP2	ATP2B3	CAMK2B	XK	TLE2	CCDC85A	SLC9A3R2	NGEF	JADE2	PTPRU	TG	PLEKHB1	RASGRP2	CAMK2B	PLEKHB1	FSTL4
SLC9A3R2	WSCD2	LZTS1	GYG2	CCDC85A	XK	PPP2R2C	RASGRP2	DGKG	FSTL4	NGEF	GYG2	PDE4A	MPPED2	PTPRN	SLC9A3R2	JADE2	MRI1	SPTB	DGKG	CDH10	PTPRN
TLE2	SEMA3C	SLC9A3R2	F7	GYG2	CCDC85A	MGLL	TRHDE	PTPRU	CCDC85A	ATP2B3	CAMK2B	NGEF	KIF26A	RASGRF1	TLE2	XK	CDH10	PPP2R2C	PTPRU	TG	CCDC85A
PDE4A	FOSL2	TLE2	RASGRF1	F7	GYG2	NUAK1	MGLL	SLC9A3R2	GYG2	TRPC7	PTPRU	MPPED2	ATP2B3	CAMK2B	PDE4A	CCDC85A	TNC	MGLL	SLC9A3R2	FSTL4	GYG2
NGEF	BRINP1	NGEF	CAMK2B	CAMK2B	RASGRF1	NEBL	NUAK1	TLE2	F7	SPTB	RIMBP2	ATP2B3	RASGRP2	DGKG	NGEF	GYG2	CAPG	NUAK1	TLE2	PTPRN	RASGRF1
MPPED2	CDH7	ATP2B3	DGKG	DGKG	CAMK2B	ADCY2	WSCD2	PDE4A	CAMK2B	CNGB1	TLE2	RASGRP2	TRPC5	RIMBP2	MPPED2	RASGRF1	TG	FOSL2	NGEF	GYG2	CAMK2B
KIF26A	COL19A1	RASGRP2	PTPRU	PTPRU	DGKG	CARMIL1	FOSL2	NGEF	DGKG	TRPC5	PDE4A	CNGB1	TRHDE	SLC9A3R2	KIF26A	CAMK2B	JADE2	NEBL	MPPED2	F7	DGKG
ATP2B3	P2RX5	SPTB	RIMBP2	RIMBP2	SLC9A3R2	MID2	CACNG5	MPPED2	PTPRU	TRHDE	NGEF	CAMK2A	PPP2R2C	TLE2	ACSL4	DGKG	XK	MID2	KIF26A	RASGRF1	SLC9A3R2
ACSL4	ABCB1	CNGB1	SLC9A3R2	SLC9A3R2	TLE2	STK17B	ADCY2	KIF26A	RIMBP2	PPP2R2C	MPPED2	TRPC5	SNCB	NGEF	RASGRP2	PTPRU	FSTL4	STK17B	ATP2B3	CAMK2B	TLE2
RASGRP2	CRTAC1	TRPC5	TLE2	TLE2	PDE4A	TTC39A	STK17B	ATP2B3	LZTS1	SNCB	KIF26A	TRHDE	NUAK1	MPPED2	SPTB	RIMBP2	CCDC85A	TTC39A	RASGRP2	DGKG	NGEF
SPTB	HCN2	PPP2R2C	NGEF	PDE4A	NGEF	ACHE	ABCB1	ACSL4	SLC9A3R2	WSCD2	ATP2B3	ALPK1	FOSL2	KIF26A	TRHDE	SLC9A3R2	GYG2	ACHE	SPTB	PTPRU	MPPED2
PPP2R2C	RASD2	NUAK1	MPPED2	NGEF	MPPED2	PITPNM2	TTC39A	RASGRP2	TLE2	SEMA3C	ACSL4	PPP2R2C	NEBL	ATP2B3	PPP2R2C	TLE2	F7	PITPNM2	TRPC5	RIMBP2	KIF26A
MGLL	PDGFB	SEMA3C	KIF26A	MPPED2	KIF26A	SEL1L3	ACHE	SPTB	PDE4A	FOSL2	RASGRP2	SNCB	ADCY2	RASGRP2	SNCB	PDE4A	RASGRF1	SEL1L3	TRHDE	SLC9A3R2	ATP2B3
NUAK1	ISM2	FOSL2	ATP2B3	KIF26A	ACSL4	COL9A3	PITPNM2	TRHDE	NGEF	CACNG5	TRPC5	NUAK1	BRINP1	SPTB	MGLL	NGEF	CAMK2B	COL9A3	PPP2R2C	TLE2	RASGRP2
SEMA3C	DOK5	NEBL	ACSL4	ATP2B3	RASGRP2	CRTAC1	SEL1L3	PPP2R2C	MPPED2	ADCY2	TRHDE	IPCEF1	CARMIL1	TRPC5	NUAK1	MPPED2	PTPRU	CRTAC1	SNCB	PDE4A	SPTB
CACNG5	NTSR1	ADCY2	RASGRP2	RASGRP2	SPTB	HCN2	COL9A3	SNCB	KIF26A	RAPGEF3	PPP2R2C	FOSL2	STK17B	TRHDE	SEMA3A	KIF26A	RIMBP2	HCN2	MGLL	NGEF	CAMK2A
NEBL	RSPO4	BRINP1	CNGB1	SPTB	CAMK2A	SEZ6L	HCN2	MGLL	ATP2B3	P2RX5	SNCB	NEBL	ACHE	ALPK1	SEMA3C	ATP2B3	LZTS1	PDGFB	NUAK1	MPPED2	TRHDE
CARMIL1	SAMHD1	STK17B	TRPC5	TRHDE	TRPC5	PDGFB	SUSD2	NUAK1	RASGRP2	ABCB1	MGLL	ADCY2	RPH3A	PPP2R2C	FOSL2	ACSL4	SLC9A3R2	CHGA	WSCD2	KIF26A	PPP2R2C
MID2	CELF4	TTC39A	TRHDE	PPP2R2C	TRHDE	ISM2	SEZ6L	SEMA3A	SPTB	TTC39A	NUAK1	CARMIL1	SEL1L3	SNCB	NEBL	RASGRP2	TLE2	RSPO4	FOSL2	ATP2B3	SNCB
STK17B	STS	NOS1	PPP2R2C	SNCB	PPP2R2C	CHGA	PDGFB	SEMA3C	CAMK2A	ACHE	WSCD2	CDH7	COL9A3	MGLL	ADCY2	SPTB	PDE4A	PPP1R16B	CACNG5	RASGRP2	MGLL
TTC39A	NALCN	PITPNM2	SNCB	MGLL	MGLL	RSPO4	CHGA	FOSL2	TRPC5	RPH3A	SEMA3C	STK17B	HCN2	NUAK1	CARMIL1	TRHDE	MPPED2	ST8SIA5	NEBL	CNGB1	NUAK1
ACHE	SYT17	SEL1L3	MGLL	NUAK1	NUAK1	PPP1R16B	MMP9	CACNG5	PPP2R2C	PITPNM2	FOSL2	P2RX5	SUSD2	WSCD2	MID2	PPP2R2C	KIF26A	MXRA5	ADCY2	TRPC5	FOSL2
PITPNM2	JPH1	CRTAC1	NUAK1	WSCD2	SEMA3C	ST8SIA5	SLCO4A1	NEBL	MGLL	MYH7	CACNG5	COL16A1	SEZ6L	FOSL2	CDH7	SNCB	ACSL4	STS	BRINP1	TRHDE	CACNG5
SEL1L3	SH3GL2	HCN2	FOSL2	FOSL2	FOSL2	MXRA5	RSPO4	ADCY2	NUAK1	COL9A3	ADCY2	WNT11	RASD2	CACNG5	STK17B	MGLL	RASGRP2	TUBB4A	CARMIL1	ALPK1	NEBL
COL9A3	SPOCK2	SEZ6L	CACNG5	CACNG5	CACNG5	STS	ST8SIA5	CARMIL1	SEMA3C	HCN2	BRINP1	ACHE	CHGA	NEBL	COL19A1	NUAK1	SPTB	SLC17A7	MID2	PPP2R2C	ADCY2
CRTAC1	NEURL1	RASD2	DCX	NEBL	NEBL	SYT17	CHRDL1	MID2	FOSL2	MMP11	DCT	PITPNM2	MMP9	ADCY2	P2RX5	SEMA3A	CNGB1	AMH	CDH7	SNCB	CARMIL1
HCN2	SORCS1	PDGFB	NEBL	ADCY2	ADCY2	SFRP1	SYT17	CDH7	CACNG5	RASD2	MID2	SEL1L3	RSPO4	BRINP1	COL16A1	SEMA3C	TRPC5	SHB	P2RX5	MGLL	MID2
MMP11	RASD1	NTSR1	ADCY2	BRINP1	BRINP1	SLC17A7	SFRP1	STK17B	NEBL	PDGFB	COL19A1	COL9A3	SAMHD1	CARMIL1	WNT11	FOSL2	TRHDE	VSIR	COL16A1	NUAK1	CDH7
SEZ6L	PTPN5	PPP1R16B	BRINP1	CDH7	CARMIL1	AMH	TUBB4A	COL19A1	ADCY2	CHGA	P2RX5	CRTAC1	PPP1R16B	MID2	TTC39A	CACNG5	SNCB	NEURL1	ABCB1	FOSL2	STK17B
PDGFB	SYT10	MAP1LC3A	RAPGEF3	STK17B	MID2	SH3GL2	SLC17A7	P2RX5	BRINP1	MMP9	COL16A1	HCN2	MAP1LC3A	STK17B	ACHE	NEBL	MGLL	SORCS1	TTC39A	DCX	COL19A1
[illegible]	[illegible]	[illegible]	[illegible]	[illegible]	[illegible]	[illegible]	[illegible]	[illegible]	[illegible]	[illegible]	[illegible]	[illegible]	[illegible]	[illegible]	[illegible]	[illegible]	[illegible]	[illegible]	[illegible]	[illegible]	[illegible]

[illegible]	[illegible]	[illegible]	[illegible]	[illegible]	[illegible]	[illegible]	[illegible]	[illegible]	[illegible]	[illegible]	[illegible]	[illegible]	[illegible]	[illegible]	[illegible]	[illegible]	[illegible]	[illegible]	[illegible]	[illegible]	[illegible]
CHGA	SEMA5A	ST8SIA5	STK17B	WNT11	STK17B	NEURL1	DNAH11	TTC39A	DCT	NTSR1	TTC39A	SEZ6L	NALCN	COL16A1	SEL1L3	BRINP1	CACNG5	EYA4	NOS1	ADCY2	COL16A1
SLCO4A1	UNC5A	STS	P2RX5	TTC39A	P2RX5	SORCS1	CRHR2	ACHE	CDH7	PPP1R16B	RPH3A	PDGFB	SALL1	WNT11	COL9A3	CARMIL1	DCX	SEMA5A	PITPNM2	BRINP1	WNT11
RSPO4	EFEMP1	SYT17	COL16A1	ACHE	COL16A1	FAM149A	LHX2	PITPNM2	STK17B	MAP1LC3A	NOS1	CACNA1I	SYT17	TTC39A	CRTAC1	MID2	NEBL	EFHD1	SEL1L3	RAPGEF3	ACHE
PPP1R16B	EFHD1	TUBB4A	WNT11	RPH3A	WNT11	CCKBR	CORO2A	SEL1L3	COL16A1	CELF4	PITPNM2	CHGA	TUBB4A	ACHE	HCN2	CDH7	ADCY2	SLC9A2	COL9A3	DCT	RPH3A
ST8SIA5	DLGAP3	SLC17A7	TTC39A	NOS1	TTC39A	PTPN5	CDH23	COL9A3	WNT11	STS	MYH7	MMP9	SLC17A7	NOS1	SEZ6L	STK17B	RAPGEF3	CHD5	CRTAC1	CDH7	NOS1
MXRAS	HPCAL4	AMH	ACHE	PITPNM2	ACHE	EYA4	SPOCK2	CRTAC1	TTC39A	KLF8	COL9A3	DOK5	AMH	PITPNM2	PDGFB	P2RX5	CARMIL1	DLGAP3	HCN2	STK17B	PITPNM2
STS	ACOT2	CRHR2	RPH3A	SEL1L3	PITPNM2	SEMA5A	SFXN3	HCN2	ACHE	NALCN	CRTAC1	RSPO4	LHX2	SEL1L3	ISM2	COL16A1	MID2	IGSF21	MMP11	P2RX5	SEL1L3
SYT17	DUSP4	LHX2	MYH7	MYH7	SEL1L3	HBEGF	NEURL1	MMP11	NOS1	SALL1	PALMD	MAP1LC3A	CORO2A	COL9A3	CHGA	WNT11	STK17B	CDK18	SUSD2	COL16A1	COL9A3
RHOV	FLT3	CORO2A	COL9A3	COL9A3	COL9A3	CDH6	SORCS1	SEZ6L	PITPNM2	JPH1	HCN2	CELF4	CDH23	CRTAC1	SLCO4A1	TTC39A	COL19A1	PLPPR5	SEZ6L	TTC39A	HCN2
JPH1	HS3ST2	SHB	CRTAC1	CRTAC1	CRTAC1	EFHD1	RASD1	PDGFB	SEL1L3	TUBB4A	MMP11	ST8SIA5	SPOCK2	PALMD	RSPO4	ACHE	P2RX5	PPL	RASD2	ACHE	MMP11
TUBB4A	GRM4	CDH23	HCN2	HCN2	HCN2	SLC9A2	FAM149A	ISM2	COL9A3	SLC17A7	SUSD2	MXRAS	SFXN3	HCN2	PPP1R16B	PITPNM2	COL16A1	NEK6	PDGFB	RPH3A	SUSD2
SLC17A7	PACSIN1	SPOCK2	SUSD2	MMP11	MMP11	CHD5	CCKBR	CHGA	CRTAC1	AMH	SEZ6L	KLF8	NEURL1	MMP11	MAP1LC3A	SEL1L3	WNT11	GALNT12	CACNA1I	NOS1	ISM2
AMH	EFNB2	SFXN3	SEZ6L	SUSD2	SEZ6L	DLGAP3	IL10RA	SLCO4A1	HCN2	GCK	RASD2	SALL1	CCKBR	SUSD2	ST8SIA5	MYH7	TTC39A	KLHL29	DOK5	SEL1L3	CHGA
LHX2	BMP2	FBXW4	RASD2	RASD2	RASD2	IGSF21	PTPN5	NTSR1	MMP11	LHX2	PDGFB	SYT17	PTPN5	SEZ6L	MXRAS	COL9A3	ACHE	EGR1	NTSR1	MYH7	MMP9
CORO2A	SYNGR3	NEURL1	PDGFB	PDGFB	PDGFB	NRP2	TPD52L1	RSPO4	SUSD2	CORO2A	CACNA1I	JPH1	EYA4	RASD2	STS	CRTAC1	RPH3A	DUSP4	RSPO4	COL9A3	DOK5
SH3GL2	VGF	SORCS1	CHGA	ISM2	ISM2	PPL	EYA4	PPP1R16B	SEZ6L	SHB	ISM2	TUBB4A	TFEB	PDGFB	CHRDL1	HCN2	NOS1	HPCA	PPP1R16B	CRTAC1	SLCO4A1
SHB	PUDP	RASD1	MMP9	CHGA	CHGA	NEK6	ARFGEF3	MAP1LC3A	RASD2	CDH23	CHGA	LHX2	SEMA5A	ISM2	SALL1	MMP11	SEL1L3	PHF24	MAP1LC3A	HCN2	NTSR1
VSIR	SHFL	FAM149A	NTSR1	NTSR1	DOK5	GALNT12	UNC5A	ST8SIA5	PDGFB	SPOCK2	MMP9	SH3GL2	LOX	CHGA	SYT17	SEZ6L	MYH7	SLC12A5	CELF4	MMP11	RSPO4
NEURL1	RAMP2	CCKBR	RSPO4	RSPO4	SLCO4A1	KLHL29	OTOF	MXRAS	ISM2	FBXW4	DOK5	CDH23	CDH6	MMP9	RHOV	RASD2	COL9A3	TOX2	ST8SIA5	SUSD2	SAMHD1
SORCS1	GCH1	TPD52L1	SAMHD1	SAMHD1	RSPO4	EGR1	EFHD1	STS	CHGA	NEURL1	SLCO4A1	SFXN3	UNC5A	DOK5	SFRP1	PDGFB	CRTAC1	CPNE5	MXRAS	SEZ6L	PPP1R16B
CCKBR	PPARG	ARFGEF3	PPP1R16B	PPP1R16B	PPP1R16B	DUSP4	CHD5	CHRDL1	SLCO4A1	SORCS1	NTSR1	FBXW4	HES1	SLCO4A1	TUBB4A	ISM2	PALMD	PCSK2	STS	RASD2	MXRAS
PTPN5	BCAN	TFEB	MAP1LC3A	MAP1LC3A	MAP1LC3A	HPCA	DLGAP3	SALL1	NTSR1	CCKBR	RSPO4	FAM149A	OTOF	NTSR1	SLC17A7	CHGA	HCN2	TMEM74B	NALCN	PDGFB	CHRDL1
EYA4	MEIS2	SEMA5A	ST8SIA5	CELF4	ST8SIA5	HS3ST2	HPCAL4	SYT17	RSPO4	MGP	SAMHD1	SYT10	EFHD1	TLDC2	AMH	MMP9	MMP11	MCF2L	SALL1	ISM2	NALCN
SEMA5A	RERG	LOX	MXRAS	ST8SIA5	MXRAS	WIPF3	IGSF21	RHOV	TLDC2	ARFGEF3	PPP1R16B	SEMA5A	SLC9A2	PPP1R16B	CORO2A	SLCO4A1	SUSD2	PLAAT1	RHOV	CHGA	SALL1
HBEGF	LMO2	NPR3	CHRDL1	MXRAS	STS	TWIST1	PLPPR5	SFRP1	SAMHD1	SEMA5A	MAP1LC3A	HBEGF	DLGAP3	MAP1LC3A	SH3GL2	NTSR1	SEZ6L	IDUA	SFRP1	MMP9	SYT17
EFEMP1	GABBR2	UNC5A	NALCN	STS	CHRDL1	PHF24	PPL	JPH1	PPP1R16B	HBEGF	CELF4	LOX	HPCAL4	CELF4	SHB	RSPO4	RASD2	RASL11B	JPH1	DOK5	JPH1
EFHD1	CCN3	OTOF	SALL1	SYT17	SALL1	TOX2	NEK6	TUBB4A	MAP1LC3A	LOX	ST8SIA5	UNC5A	RCAN3	MXRAS	CDH23	SAMHD1	PDGFB	RFPL2	TUBB4A	NTSR1	TUBB4A
SLC9A2	FHDC1	CHD5	SYT17	RHOV	SYT17	CPNE5	CNNM1	SLC17A7	CELF4	GABRG2	KLF8	FGF12	NEK6	CHRDL1	VSIR	PPP1R16B	CACNA1I	PUDP	AMH	RSPO4	SLC17A7
CHD5	LRIG3	DLGAP3	RHOV	SFRP1	RHOV	PCSK2	MOB3B	AMH	ST8SIA5	RBP1	SALL1	HES1	KLHL29	NALCN	FBXW4	MAP1LC3A	ISM2	CALY	CRHR2	SAMHD1	AMH
DLGAP3	DUSP6	PLPPR5	JPH1	JPH1	SFRP1	BCL11B	TGFBI	LHX2	STS	HES1	SYT17	OTOF	EGR1	SALL1	NEURL1	ST8SIA5	CHGA	ASS1	LHX2	PPP1R16B	DNAH11
IGSF21	MDGA2	NRP2	TUBB4A	AMH	TUBB4A	PLAAT1	EGR1	CORO2A	CHRDL1	OTOF	RHOV	EFEMP1	DUSP4	SYT17	SORCS1	MXRAS	MMP9	SHFL	SH3GL2	MAP1LC3A	CRHR2
PLPPR5	MAP3K21	GALNT12	SLC17A7	CRHR2	SLC17A7	IDUA	DUSP4	SH3GL2	KLF8	IGFBP5	SFRP1	IGFBP5	HPCA	RHOV	RASD1	STS	DOK5	RAMP2	SHB	ST8SIA5	PPP1R17
NRP2	TMEFF2	KLHL29	AMH	GCK	AMH	RASL11B	FLT3	SHB	SALL1	PRRX1	PLAT	EFHD1	TWIST1	JPH1	FAM149A	CHRDL1	SLCO4A1	PPP1R1B	SPOCK2	MXRAS	LHX2
PPL	IGSF11	CNNM1	LHX2	LHX2	LHX2	RFPL2	HS3ST2	CDH23	SYT17	TNR	JPH1	SLC9A2	PHF24	TUBB4A	CCKBR	NALCN	NTSR1	KHDRBS3	SFXN3	CHRDL1	SHB
NEK6	CPLX2	TGFBI	CDH23	CORO2A	CORO2A	PUDP	NXPH1	VSIR	SFRP1	DLGAP3	SLC17A7	PRRX1	SLC12A5	SLC17A7	PTPN5	SALL1	RSPO4	GCH1	FBXW4	KLF8	CDH23
GALNT12	HMGA2	DUSP4	SPOCK2	SH3GL2	SH3GL2	CALY	TWIST1	NEURL1	JPH1	HPCAL4	AMH	DLGAP3	TOX2	LHX2	SYT10	SYT17	SAMHD1	SLC6A11	NEURL1	NALCN	VSIR
KLHL29	TMEM132D	ECHDC2	SFXN3	SHB	SHB	ASS1	PHF24	SORCS1	TUBB4A	GALNT12	CRHR2	HPCAL4	IQSEC2	CORO2A	EYA4	RHOV	PPP1R16B	PPARG	SORCS1	SALL1	SFXN3
CNNM1	SPOCK1	HS3ST2	PTPN5	CDH23	VSIR	SHFL	SLC12A5	FAM149A	SLC17A7	ACOT2	LHX2	IGSF21	PACSIN1	SHB	SEMA5A	SFRP1	MAP1LC3A	RAP1GAP2	FAM149A	SYT17	FBXW4
EGR1	CCSAP	WIPF3	SYT10	VSIR	SPOCK2	RAMP2	GRM4	CCKBR	AMH	KLHL29	SH3GL2	CDK18	PCSK2	CDH23	HBEGF	JPH1	CELF4	ATP8A2	CCKBR	RHOV	NEURL1
DUSP4	NCAM2	NXPH1	ARFGEF3	FBXW4	FBXW4	PPP1R1B	CPNE5	PTPN5	DNAH11	MOB3B	SHB	RCAN3	MCF2L	VSIR	CDH6	TUBB4A	CDH20	GPR12	IL10RA	PLAT	RASD1
HPCA	PDE1C	TOX2	TFEB	NEURL1	NEURL1	KHDRBS3	BMP2	SYT10	CRHR2	TGFBI	CDH23	NEK6	FGD3	SFXN3	RBP1	SLC17A7	ST8SIA5	TMTC1	SYT10	JPH1	CCKBR
HS3ST2	MARVELD1	IQSEC2	SEMA5A	RASD1	SORCS1	GCH1	TMEM74B	EYA4	LHX2	ECHDC2	VSIR	KLHL29	BCL11B	FBXW4	EFEMP1	AMH	MXRAS	MEIS2	TPD52L1	TUBB4A	PTPN5
WIPF3	PPARGC1B	PACSIN1	HBEGF	FAM149A	FAM149A	SLC6A11	MCF2L	SEMA5A	CORO2A	HPCA	SPOCK2	TGFBI	RFPL2	NEURL1	EFHD1	DNAH11	STS	TSPAN2	ARFGEF3	SLC17A7	TPD52L1
[illegible]	[illegible]	[illegible]	[illegible]	[illegible]	[illegible]	[illegible]	[illegible]	[illegible]	[illegible]	[illegible]	[illegible]	[illegible]	[illegible]	[illegible]	[illegible]	[illegible]	[illegible]	[illegible]	[illegible]	[illegible]	[illegible]

[illegible]	[illegible]	[illegible]	[illegible]	[illegible]	[illegible]	[illegible]	[illegible]	[illegible]	[illegible]	[illegible]	[illegible]	[illegible]	[illegible]	[illegible]	[illegible]	[illegible]	[illegible]	[illegible]	[illegible]	[illegible]	[illegible]
SLC12A5	HTR5A	EFNB2	GABRG2	IL10RA	IL10RA	ATP8A2	FGD3	CDH6	SHB	HS3ST2	FBXW4	DUSP4	IRF5	RASD1	CHD5	LHX2	KLF8	ADAMTS8	SEMA5A	GCK	SEMA5A
TOX2	GALNT14	PCSK2	CDH6	PTPN5	PTPN5	GPR12	LRRC61	HES1	CDH23	WIPF3	LGI1	HPCA	CALY	FAM149A	DLGAP3	CORO2A	NALCN	EPHA7	LOX	LHX2	LOX
CPNE5	GJD2	TMEM74B	NPR3	TPD52L1	EYA4	BEX2	IDUA	EFEMP1	VSIR	NXPH1	RASD1	NXPH1	ASS1	CCKBR	HPCAL4	SH3GL2	SALL1	MMD2	GABRG2	CORO2A	NPR3
EFNB2	SLC37A1	FGD3	FGF12	EYA4	SEMA5A	TMTC1	SYNGR3	EFHD1	SPOCK2	CHST8	CCKBR	TWIST1	SYNE1	PTPN5	IGSF21	SHB	SYT17	FHDC1	UNC5A	CDH23	UNC5A
PCSK2	LY6E	BCL11B	HES1	ARFGEF3	HBEGF	TSPAN2	RASL11B	SLC9A2	SFXN3	EFNB2	IL10RA	SLC12A5	RAMP2	EYA4	PLPPR5	CDH23	RHOV	ARHGAP20	EFEMP1	SPOCK2	OTOF
TMEM74B	KCNF1	PLAAT1	EFHD1	TFEB	LOX	SYT6	VGF	TNR	FBXW4	BMP2	PTPN5	IQSEC2	SLC6A11	ARFGEF3	PLPPR4	VSIR	SFRP1	DUSP5	EFHD1	SFXN3	IGFBP5
MCF2L	GABRB1	SYNGR3	CHD5	SEMA5A	CDH6	ADAMTS8	IRF5	CHD5	NEURL1	PLEKHG3	SYT10	PACSIN1	PPARG	TFEB	RCAN3	SPOCK2	PLAT	KCNH5	PRRX1	NEURL1	EFHD1
BCL11B	ADAMTS9	RASL11B	DLGAP3	LOX	EFEMP1	EPHA7	CALY	DLGAP3	SORCS1	FGD3	TPD52L1	CPNE5	BCAN	SEMA5A	NRP2	NEURL1	JPH1	TMC6	CHD5	PTPN5	SLC9A2
PLAAT1	EXOS	RFPL2	HPCAL4	NPR3	EFHD1	LMO2	SHFL	HPCAL4	RASD1	PLAAT1	SEMA5A	PSD4	GPR12	HBEGF	PPL	SORCS1	TUBB4A	CACNG8	DLGAP3	SYT10	TNR
IDUA	CAMKV	PUDP	IGSF21	UNC5A	SLC9A2	MMD2	RAMP2	IGSF21	FAM149A	IDUA	HBEGF	F10	TSPAN2	CDH6	NEK6	FAM149A	SLC17A7	MAP3K21	HPCAL4	TPD52L1	CHD5
RASL11B	CDCA7L	CALY	CDK18	RBP1	CHD5	GABBR2	KHDRBS3	CDK18	CCKBR	RASL11B	LOX	PLEKHG3	SYT6	NPR3	GALNT12	CCKBR	AMH	MARCHF4	IGSF21	EYA4	DLGAP3
RFPL2	DYNLT3	ASS1	PLPPR5	EFEMP1	DLGAP3	FHDC1	SLC6A11	PLPPR5	IL10RA	VGF	GABRG2	BCL11B	MMD2	UNC5A	KLHL29	IL10RA	DNAH11	GRIP2	PLPPR5	ARFGEF3	HPCAL4
IRF5	PRKCB	RAMP2	RCAN3	SLC9A2	HPCAL4	ARHGAP20	MATN2	PLPPR4	PTPN5	IRF5	CDH6	PLAAT1	FHDC1	RBP1	CNNM1	PTPN5	CRHR2	VWA5B2	PLPPR4	TFEB	IGSF21
PUDP	RAB26	KHDRBS3	NEK6	CHD5	IGSF21	CGREF1	BCAN	RCAN3	TPD52L1	PUDP	NPR3	RFPL2	SHISAL1	OTOF	EGR1	SYT10	GCK	OCIAD2	RCAN3	SEMA5A	PLPPR4
CALY	RAB3IL1	SLC6A11	EGR1	DLGAP3	CDK18	DUSP5	TMTC1	NRP2	EYA4	CALY	UNC5A	KCNA5	KCNH5	IGFBP5	DUSP4	EYA4	LHX2	GABRB2	NEK6	GABRG2	RCAN3
ASS1	RAB31	PPARG	DUSP4	HPCAL4	PLPPR5	KCNH5	TSPAN2	PPL	ARFGEF3	GCH1	RBP1	ASS1	TMC6	EFHD1	HPCA	TFEB	CORO2A	ZC3HAV1L	GALNT12	CDH6	NEK6
SHFL	PHYHIP	BCAN	ECHDC2	IGSF21	RCAN3	TMC6	CMPK2	NEK6	TFEB	BCAN	OTOF	ANGPTL6	CACNG8	SLC9A2	HS3ST2	SEMA5A	SHB	LANCL3	KLHL29	NPR3	GALNT12
RAMP2	NPNT	ATP8A2	HPCA	RCAN3	PPL	CACNG8	ADAMTS8	GALNT12	SEMA5A	BEX2	IGFBP5	RAMP2	CASQ1	CHD5	WIPF3	HBEGF	CDH23	KIAA1755	CNNM1	FGF12	KLHL29
PPP1R1B	CPLX1	GPR12	FLT3	NRP2	NEK6	MAP3K21	LMO2	KLHL29	LOX	TMTC1	SLC9A2	PPP1R1B	DYRK3	DLGAP3	TWIST1	CDH6	VSIR	AP1S3	EGR1	HES1	CNNM1
KHDRBS3	IRS1	PDZD2	WIPF3	PPL	GALNT12	MARCHF4	GPR45	EGR1	NPR3	TSPAN2	PRRX1	SLC6A11	ZNF385B	HPCAL4	PHF24	UNC5A	SPOCK2	JAKMIP1	DUSP4	EFEMP1	EGR1
GCH1	ROR2	CMPK2	NXPH1	NEK6	KLHL29	GRIP2	RNASEH2B	DUSP4	UNC5A	ADAMTS8	TNR	RAP1GAP2	MARCHF4	IGSF21	RAB9B	HES1	SFXN3	AK5	ECHDC2	IGFBP5	DUSP4
SLC6A11	TANC2	ADAMTS8	TWIST1	GALNT12	EGR1	VWA5B2	MMD2	HPCA	FGF12	ENTREP1	DLGAP3	BCAN	OCIAD2	CDK18	TOX2	EFEMP1	FBXW4	CCSAP	FLT3	EFHD1	HPCA
PPARG	ALK	ENTREP1	PHF24	ACOT2	DUSP4	OCIAD2	GABBR2	HS3ST2	HES1	AGAP2	IGSF21	ATP8A2	ADAMTS19	PLPPR5	CPNE5	EFHD1	NEURL1	NCAM2	HS3ST2	SLC9A2	FLT3
RAP1GAP2	NPTX1	PKIB	RAB9B	KLHL29	ECHDC2	GABRB2	FHDC1	WIPF3	OTOF	GPR45	CDK18	TMTC1	GABRB2	RCAN3	EFNB2	SLC9A2	SORCS1	MARVELD1	TWIST1	DLGAP3	HS3ST2
BCAN	NEUROD2	RNASEH2B	SLC12A5	CNNM1	HPCA	ZC3HAV1L	SPTBN5	TWIST1	EFHD1	RNASEH2B	PLPPR5	MEIS2	ZC3HAV1L	PPL	BMP2	CHD5	RASD1	GOLGA7B	PHF24	HPCAL4	WIPF3
ATP8A2	FUT9	MMD2	TOX2	EGR1	HS3ST2	LANCL3	CGREF1	PHF24	CHD5	SCN2A	NRP2	TSPAN2	SNTG1	NEK6	PCSK2	DLGAP3	FAM149A	ADCY8	RAB9B	IGSF21	NXPH1
GPR12	METAP1D	KIF12	PACSIN1	DUSP4	WIPF3	ARHGAP36	SHISAL1	RAB9B	DLGAP3	CCN3	PPL	CD101	CDHR1	GALNT12	BCL11B	HPCAL4	CCKBR	SORCS3	SLC12A5	CDK18	TWIST1
BEX2	VEGFB	GABBR2	CPNE5	ECHDC2	PHF24	MAL2	DUSP6	TOX2	HPCAL4	ARHGAP20	NEK6	EPHA7	CDH22	KLHL29	PLAAT1	IGSF21	IL10RA	FBXO32	TOX2	PLPPR5	PHF24
TMTC1	CNTNAP2	FHDC1	MCF2L	HPCA	RAB9B	FAM135B	KCNH5	CPNE5	IGSF21	CGREF1	GALNT12	MMD2	HMGA2	EGR1	IDUA	CDK18	PTPN5	ATP2B2	IQSEC2	PLPPR4	SLC12A5
TSPAN2	FUT1	ARHGAP20	PLEKHG3	FLT3	SLC12A5	KIAA1755	LYSMD2	BMP2	CDK18	CUZD1	ACOT2	SCN7A	ADRA2A	DUSP4	RASL11B	PLPPR5	SYT10	MX1	GRM4	RCAN3	TOX2
SYT6	LY6H	SPTBN5	FGD3	HS3ST2	TOX2	AP1S3	TMC6	PCSK2	PLPPR5	DUSP5	KLHL29	KIF12	KCNA6	ECHDC2	RFPL1	PLPPR4	MGP	NRG2	PACSIN1	NEK6	IQSEC2
CMPK2	DSCAML1	CGREF1	BCL11B	WIPF3	GRM4	JAKMIP1	CACNG8	MCF2L	NRP2	PARP9	CNNM1	GABBR2	JAKMIP1	HPCA	RFPL2	RCAN3	ADGRD1	SCUBE1	CPNE5	TGFBI	GRM4
ADAMTS8	RIMKLA	DUSP5	PLAAT1	TWIST1	CPNE5	AK5	DYRK3	BCL11B	PPL	LRIG3	KCNJ5	CCN3	THY1	WIPF3	IRF5	NRP2	TPD52L1	ACE	EFNB2	EGR1	PACSIN1
EPHA7	FZD8	PARP9	SYNGR3	PHF24	BMP2	CCSAP	MAP3K21	PLAAT1	NEK6	SLC13A5	TGFBI	FHDC1	CCSAP	NXPH1	PUDP	PPL	EYA4	SLC37A1	PSD4	DUSP4	EFNB2
LMO2	ST8SIA3	MDGA2	RFPL2	RAB9B	PCSK2	NCAM2	TMEFF2	IDUA	GALNT12	CACNG8	EGR1	PLCB2	NCAM2	TWIST1	CALY	NEK6	TFEB	SPTBN4	BMP2	ECHDC2	PSD4
PKIB	KCTD12	LYSMD2	VGF	SLC12A5	TMEM74B	CA10	OCIAD2	SYNGR3	ACOT2	PRKACB	DUSP4	DUSP6	ADAMTS5	PHF24	ASS1	GALNT12	SEMA5A	LY6E	MCF2L	HPCA	TMEM74B
MMD2	CPNE7	SLC13A5	ASS1	TOX2	MCF2L	MARVELD1	TIFA	RASL11B	KLHL29	DYRK3	ECHDC2	MDGA2	SORCS3	RAB9B	SHFL	ACOT2	HBEGF	NTN3	F10	FLT3	PLEKHG3
GABBR2	AKAP5	TMC6	SYNE1	CHST8	FGD3	ADCY8	GABRB2	RFPL1	CNNM1	MAP3K21	FLT3	KCNH5	ESYT3	SLC12A5	RAMP2	KLHL29	LOX	CAMK2N1	BCL11B	HS3ST2	FGD3
FHDC1	TSHZ1	CACNG8	RAMP2	PACSIN1	BCL11B	SORCS3	RASGEF1C	RFPL2	TEK	ZNF385B	HS3ST2	LYSMD2	NRG2	TOX2	PPP1R1B	CNNM1	GABRG2	WNT4	PLAAT1	WIPF3	BCL11B
ARHGAP20	SSTR2	PRKACB	PPP1R1B	CPNE5	PLAAT1	FBXO32	ZC3HAV1L	VGF	MOB3B	PTH2R	WIPF3	SLC13A5	DUSP23	IQSEC2	KHDRBS3	MOB3B	CDH6	KCNJ3	LRRC61	TWIST1	LRRC61
DUSP5	CHRM2	MAP3K21	KHDRBS3	EFNB2	LRRC61	MX1	MAL2	IRF5	TGFBI	MARCHF4	NXPH1	TMC6	CSRP1	GRM4	GCH1	TGFBI	UNC5A	FBXO41	IDUA	PHF24	IDUA
MDGA2	TMEM132E	ZNF385B	SLC6A11	PSD4	IDUA	NRG2	FAM135B	PUDP	EGR1	STAC	TWIST1	DYRK3	ACE	PACSIN1	SLC6A11	EGR1	RBP1	DCLK3	SYNGR3	RAB9B	SYNGR3
KCNH5	FAM181B	PTH2R	ATP8A2	BMP2	RASL11B	GJD2	CDHR1	CALY	DUSP4	VWA5B2	PHF24	NYAP2	SLC37A1	CPNE5	PPARG	DUSP4	HES1	CAMKV	RASL11B	SLC12A5	RFPL1
SLC13A5	RGS6	VWA5B2	CHRM3	PCSK2	RFPL2	SCUBE1	CDH22	ASS1	ECHDC2	TIFA	SLC12A5	MARCHF4	SPTBN4	EFNB2	RAP1GAP2	ECHDC2	OTOF	SAMD3	RFPL2	TOX2	RFPL2
[illegible]	[illegible]	[illegible]	[illegible]	[illegible]	[illegible]	[illegible]	[illegible]	[illegible]	[illegible]	[illegible]	[illegible]	[illegible]	[illegible]	[illegible]	[illegible]	[illegible]	[illegible]	[illegible]	[illegible]	[illegible]	[illegible]

[illegible]	[illegible]	[illegible]	[illegible]	[illegible]	[illegible]	[illegible]	[illegible]	[illegible]	[illegible]	[illegible]	[illegible]	[illegible]	[illegible]	[illegible]	[illegible]	[illegible]	[illegible]	[illegible]	[illegible]	[illegible]	[illegible]
CACNG8	GPC6	CPLX2	MEIS2	MCF2L	IRF5	SLC37A1	PTPRO	RAMP2	FLT3	IGFBP3	CHST8	STAC	FBXO27	PCSK2	ATP8A2	HS3ST2	IGFBP5	PTCHD1	PUDP	GRM4	IRF5
PRKACB	CTNNA3	IGFBP3	TSPAN2	F10	PUDP	SPTBN4	SLC25A4	PPP1R1B	HS3ST2	ZC3HAV1L	GRM4	TIFA	NTN3	TMEM74B	GPR12	WIPF3	EFHD1	SPINT1	KCNA5	PACSIN1	KCNA5
DYRK3	MARCHF11	ZC3HAV1L	ADAMTS8	FGD3	CALY	LY6E	TMEM132D	KHDRBS3	WIPF3	MAL2	PACSIN1	ADAMTS19	ELAVL4	MCF2L	TMTC1	NXPH1	SLC9A2	PRKCB	ASS1	CPNE5	CALY
MAP3K21	ADRA2C	HTR2C	EPHA7	BCL11B	ASS1	NTN3	STK32B	GCH1	NXPH1	FAM135B	CPNE5	GABRB2	FBLIM1	BCL11B	MEIS2	TWIST1	PRRX1	TVP23A	SHFL	EFNB2	SYNE1
PTH2R	ACOT1	ARHGAP36	LMO2	LRRC61	SHFL	CAMK2N1	SPHKAP	SLC6A11	TWIST1	NFIB	EFNB2	IGFBP3	CAMK2N1	PLAAT1	TSPAN2	PHF24	TNR	ZCCHC18	SYNE1	TMEM74B	F12
MARCHF4	PRKD1	MAL2	RNASEL	IDUA	SYNE1	WNT4	AK5	PPARG	SLC12A5	CDHR1	BMP2	LANCL3	WNT4	SYNGR3	SYT6	RAB9B	PAPPA2	RAB26	PPP1R1B	MCF2L	KHDRBS3
GRIP2	MAP7D2	NFIB	MMD2	SYNGR3	GALNT15	KCNJ3	THY1	RAP1GAP2	TOX2	PLCH2	PCSK2	SNTG1	LRRTM1	RASL11B	ADAMTS8	SLC12A5	ANGPTL1	CPLX1	KHDRBS3	BCL11B	GCH1
VWA5B2	PABPC1L2B	CDHR1	GABBR2	RASL11B	RAMP2	FBXO41	CCSAP	BCAN	GRM4	CDH22	F10	MAL2	KCNJ3	RFPL2	EPHA7	TOX2	CHD5	IRS1	GCH1	PLAAT1	PPARG
OCIAD2	FMNL1	PLCH2	CCN3	VGF	PPP1R1B	DCLK3	NCAM2	ATP8A2	PACSIN1	KCNA6	RHOJ	PLPPR1	FBXO41	VGF	LMO2	GRM4	DLGAP3	RAB3B	SLC6A11	IDUA	MATN2
GABRB2	GALNT17	KIAA1755	FHDC1	IRF5	KHDRBS3	CAMKV	ADAMTS5	GPR12	CPNE5	ME3	PLEKHG3	ST8SIA6	SYNPR	KCNA5	MMD2	CPNE5	HPCAL4	KCNK9	RAP1GAP2	SYNGR3	BCAN
CPLX2	AHNAK2	CDH22	DDX60	PUDP	GCH1	SAMD3	SLC24A2	BEX2	EFNB2	AP1S3	FGD3	PLCH2	CAMKV	CALY	SCN7A	EFNB2	CDK18	PLEKHA2	MATN2	RFPL2	TMTC1
LANCL3	KCNQ5	KCNA6	CGREF1	CALY	SLC6A11	HEY1	SORCS3	TMTC1	PCSK2	RIT2	BCL11B	KIRREL3	GRIK2	ASS1	KIF12	BMP2	PLPPR5	REPS2	BCAN	VGF	TSPAN2
HTR2C	GABRA5	SLC25A4	CUZD1	ASS1	PPARG	BAALC	FBXO32	MEIS2	TMEM74B	GUCY1A2	PLAAT1	KIAA1755	SAMD3	SHFL	GABBR2	PCSK2	RCAN3	TANC2	ATP8A2	IRF5	SYT6
ARHGAP36	MPPED1	TMEM132D	SHISAL1	ANGPTL6	RAP1GAP2	PTCHD1	CABP1	TSPAN2	MCF2L	SPHKAP	LRRC61	CDH22	CDCA7L	KHDRBS3	FHDC1	TMEM74B	NRP2	ALK	GPR12	CALY	CMPK2
MAL2	SHTN1	THY1	LRIG3	SHFL	BCAN	SPINT1	ESYT3	SYT6	PLEKHG3	THY1	IDUA	HMGA2	HEY1	SLC6A11	ARHGAP20	MCF2L	PPL	NPTX1	PDZD2	ASS1	ADAMTS8
FAM135B	C11orf96	CCSAP	DUSP6	SYNE1	ATP8A2	LGI3	CLSTN2	ADAMTS8	BCL11B	CCSAP	SYNGR3	KCNA6	NIPSNAP3B	PPARG	PLCB2	RHOJ	NEK6	KNDC1	TMTC1	SHFL	ENTREP1
ST8SIA6	SMTNL2	ADAMTS5	KCNH5	RAMP2	GPR12	CPLX1	RNF207	EPHA7	PLAAT1	PDE1C	RFPL2	GABRA2	TMEM130	BCAN	CGREF1	BCL11B	GALNT12	PRNP	MEIS2	SYNE1	EPHA7
KIAA1755	ENO4	CA10	LYSMD2	PPP1R1B	BEX2	IRS1	NRG2	LMO2	IDUA	MARVELD1	VGF	BEND6	TVP23A	ATP8A2	DUSP5	PLAAT1	KLHL29	CALB2	TSPAN2	GALNT15	LMO2
CDH22	SLC38A3	MARVELD1	SLC13A5	KHDRBS3	PDZD2	ROR2	GJD2	MMD2	SYNGR3	GOLGA7B	IRF5	TMEM132D	ZCCHC18	GPR12	LRIG3	IDUA	CNNM1	ABCG4	CMPK2	RAMP2	PKIB
KCNA6	COL25A1	GOLGA7B	TMC6	GCH1	TMTC1	KCNK9	SCUBE1	SCN7A	RASL11B	PPARGC1B	KCNA5	AP1S3	ISLR2	PDZD2	MDGA2	SYNGR3	TEK	EGFL7	PIWIL4	PPP1R1B	RNASEH2B
SLC25A4	NKAIN2	PPARGC1B	CACNG8	SLC6A11	MEIS2	PLEKHA2	ACE	KIF12	RFPL2	SORCS3	CALY	SPOCK1	RBFOX3	TMTC1	KCNH5	RASL11B	MOB3B	SH3RF3	ADAMTS8	SLC6A11	MMD2
BEND6	PAQR9	ADCY8	CASQ1	PPARG	TSPAN2	REPS2	SLC37A1	GABBR2	VGF	SST	ASS1	STK32B	GNG8	MEIS2	LYSMD2	RFPL1	TGFBI	VEGFB	ENTREP1	MATN2	KIF12
AP1S3	VWC2	SORCS3	DYRK3	ATP8A2	SYT6	TANC2	MPZL3	FHDC1	IRF5	ATP2B2	ANGPTL6	THY1	RAB3IL1	TSPAN2	SLC13A5	RFPL2	DUSP4	ZCCHC12	LMO2	BCAN	GABBR2
GUCY1A2	SHISA6	FBXO32	MAP3K21	GPR12	ADAMTS8	ALK	LY6E	ARHGAP20	PUDP	SYN2	SHFL	CCSAP	NPNT	ADAMTS8	TMC6	IRF5	ECHDC2	TTC9B	PKIB	ATP8A2	KIAA0319
JAKMIP1	INSYN2A	NRG2	MARCHF4	BEX2	EPHA7	NPTX1	FBXO27	PLCB2	CALY	HTR5A	SYNE1	PDE1C	CPLX1	EPHA7	CACNG8	PUDP	HPCA	CHST2	RNASEH2B	CHRM3	FHDC1
AK5	PTPRT	SCUBE1	OCIAD2	PDZD2	LMO2	KNDC1	NTN3	CGREF1	ASS1	TSC22D3	GALNT15	CA10	IRS1	LMO2	C1orf216	CALY	FLT3	LY6H	MMD2	BEX2	ARHGAP20
CCSAP	SPOCK3	ACE	TIFA	TSPAN2	RNASEH2B	PRNP	FBLIM1	DUSP5	SHFL	MX1	RAMP2	GOLGA7B	RAB3B	AGAP2	DYRK3	ASS1	HS3ST2	KCNA3	GABBR2	PDZD2	UNC13C
NCAM2	FAM163B	SLC37A1	ADAMTS19	RERG	MMD2	EGFL7	CAMK2N1	MDGA2	SYNE1	CABP1	GCH1	PPARGC1B	KCNK9	DGKB	MAP3K21	SHFL	WIPF3	FZD8	FHDC1	MEIS2	PLCB2
CA10	SLC6A17	SPATC1L	GABRB2	ENTREP1	SCN7A	SH3RF3	WNT4	KCNH5	GALNT15	GALNT14	RAP1GAP2	ADCY8	PCDH7	MMD2	TMEFF2	SYNE1	TWIST1	KCNJ10	ARHGAP20	TSPAN2	SPTBN5
GOLGA7B	PDGFA	SPTBN4	ZC3HAV1L	LMO2	KIF12	VEGFB	KCNF1	LYSMD2	PPP1R1B	CLSTN2	BCAN	SORCS3	REPS2	GABBR2	MARCHF4	RAMP2	PHF24	ALS2CL	CGREF1	SYT6	CGREF1
PPARGC1B	STUM	LY6E	HTR2C	PKIB	GABBR2	NWD2	KCNJ3	SLC13A5	KHDRBS3	DYNC1I1	ATP8A2	FBXO32	MUC3A	CCN3	GRIP2	PPP1R1B	RAB9B	AMER3	DUSP5	CD101	CUZD1
ADCY8	MAFB	COL26A1	SNTG1	GPR45	FHDC1	ZCCHC12	FBXO41	TMC6	GCH1	DUSP23	BEX2	ATP2B2	GPR37	FHDC1	IGSF11	KHDRBS3	SLC12A5	KCTD12	PARP9	CMPK2	DUSP5
SORCS3	EML6	NTN3	ST8SIA6	RNASEH2B	ARHGAP20	TTC9B	COL6A3	CACNG8	SLC6A11	NBL1	PDZD2	SYN2	TANC2	PLCB2	VWA5B2	GCH1	TOX2	CPNE7	LRIG3	ADAMTS8	PARP9
FBXO32	UBE2QL1	CAMK2N1	CDHR1	KIF12	PLCB2	CHST2	SYNPR	C1orf216	PPARG	GJD2	TMTC1	ESYT3	ALK	DUSP5	OCIAD2	SLC6A11	CHST8	LYNX1	DUSP6	ENTREP1	SHISAL1
SYN2	FNDC10	WNT4	PLCH2	GABBR2	CGREF1	DSCAML1	DCLK3	DYRK3	RAP1GAP2	SCUBE1	MEIS2	NRG2	NPTX1	SHISAL1	SNCA	PPARG	IQSEC2	PLD5	MDGA2	EPHA7	DUSP6
MX1	TMEM238	KCNF1	KIRREL3	MYC	DUSP5	KCNJ10	GUCY1A1	MAP3K21	BCAN	ACE	TSPAN2	VWA5B1	NEUROD2	DUSP6	GABRB2	RAP1GAP2	PACSIN1	SSTR2	LYSMD2	LMO2	MDGA2
CABP1	SHISA9	CAMKV	CDH22	FHDC1	PARP9	AMER3	GRIK2	TMEFF2	ATP8A2	SLC37A1	CMPK2	TNNI1	KNDC1	MDGA2	CPLX2	BCAN	CPNE5	FAM83H	SLC13A5	MMD2	LYSMD2
NRG2	CEBPA	HEY1	HMGA2	ARHGAP20	MDGA2	KCTD12	HEY1	MARCHF4	GPR12	LY6E	ADAMTS8	CSRP1	PRNP	KCNH5	IGFBP3	ATP8A2	EFNB2	TMEM132C	TMC6	SCN7A	SLC13A5
GJD2	CTSO	PTCHD1	GABRA2	UNC13C	KCNH5	TMEM151A	FZD6	GRIP2	BEX2	COL26A1	ENTREP1	SCUBE1	ABCG4	LYSMD2	ZC3HAV1L	GPR12	PSD4	GALNT9	CACNG8	KIF12	TMC6
SCUBE1	SALL3	RET	BEND6	SPTBN5	LYSMD2	SLITRK4	RET	VWA5B2	TMTC1	NTN3	LMO2	ACE	EGFL7	TMC6	LANCL3	BEX2	PCSK2	PCBP3	PRKACB	GABBR2	CACNG8
ACE	DOC2B	PTER	AP1S3	CGREF1	SLC13A5	PLD5	ADAMTS15	OCIAD2	TSPAN2	FBLIM1	AGAP2	SLC37A1	VEGFB	CACNG8	ARHGAP36	PDZD2	TMEM74B	MARCHF11	MAP3K21	MYC	C1orf216
SLC37A1		TMEM130	JAKMIP1	DUSP5	TMC6	SSTR2	SPINT1	SNCA	SYT6	WNT4	GPR45	MPZL3	CNTNAP2	C1orf216	MAL2	TMTC1	MCF2L	NOG	ZNF385B	CCN3	PRKACB
SPTBN4		PRKCB	SPHKAP	SHISAL1	CACNG8	FAM83H	CLMP	GABRB2	CMPK2	BRINP3	RNASEH2B	COL26A1	TTC9B	PRKACB	PLPPR1	MEIS2	RHOJ	PRKD1	TMEFF2	FHDC1	MAP3K21
LY6E		TVP23A	THY1	LRIG3	CASQ1	TMEM132C	PRKCB	CPLX2	ADAMTS8	LRATD1	DGKB	NTN3	SLCO2A1	DYRK3	PLCH2	TSPAN2	PLEKHG3	MAP7D2	PTH2R	DDX60	TMEFF2
COL26A1		RAB26	CCSAP	DUSP6	DYRK3	GPR3	RBFOX3	ZC3HAV1L	ENTREP1	KCNJ3	MMD2	CAMK2N1	LY6H	MAP3K21	KIAA1755	SYT6	FGD3	TCEAL2	MARCHF4	UNC13C	GRIP2
NTN3		RAB3IL1	NCAM2	MDGA2	MAP3K21	GALNT9	TAP1	LANCL3	EPHA7	FBXO41	SCN2A	BRINP3	ST8SIA3	ZNF385B	ADRA2A	CMPK2	BCL11B	FMNL1	STAC	SPTBN5	STAC
CAMK2N1		RAB31	PDE1C	KCNH5	ZNF385B	PCBP3	RAB31	ARHGAP36	LMO2	COL6A3	KIF12	LRRTM1	KCNJ10	NYAP2	KCNA6	ADAMTS8	PLAAT1	SLC24A3	MUC4	CGREF1	MUC4
WNT4		LGI3	ADAMTS5	LYSMD2	PTH2R	MARCHF11	NPNT	MAL2	MMD2	ADAMTS9	GABBR2	KCNJ3	AMER3	MARCHF4	BEND6	EPHA7	LRRC61	SV2B	VWA5B2	CUZD1	VWA5B2
[illegible]		[illegible]	[illegible]	[illegible]	[illegible]	[illegible]	[illegible]	[illegible]	[illegible]	[illegible]	[illegible]	[illegible]	[illegible]	[illegible]	[illegible]	[illegible]	[illegible]	[illegible]	[illegible]	[illegible]	[illegible]

[illegible]	[illegible]	[illegible]	[illegible]	[illegible]	[illegible]	[illegible]	[illegible]	[illegible]	[illegible]	[illegible]	[illegible]	[illegible]	[illegible]	[illegible]	[illegible]	[illegible]	[illegible]	[illegible]	[illegible]	[illegible]
KCNJ3	GRM5	GRIA1	TMC6	GRIP2	ADRA2C	KCNK9	PLPPR1	GABBR2	GUCY1A1	CCN3	VSNL1	CPNE7	OCIAD2	GUCY1A2	PKIB	SYNGR3	PDE2A	ADAMTS19	LRIG3	SNCA
FBXO41	CPLX1	SLC24A2	CACNG8	IGSF11	MAP7D2	PLEKHA2	ST8SIA6	CCN3	ITGA2	KIAA0319	COL6A3	FAM133A	SNCA	JAKMIP1	MMD2	RASL11B	MPPED1	GABRA6	DUSP6	TIFA
SYNPR	ROR2	SORCS3	C1orf216	VWA5B2	TCEAL2	ADRA1B	PLCH2	FHDC1	HEY1	FHDC1	SYNPR	FJX1	ADAMTS19	AK5	KIF12	RFPL1	VSTM2B	GABRB2	KCNH5	CPLX2
DCLK3	PARM1	ATP2B2	PRKACB	OCIAD2	FMNL1	GPR37	SCN2B	SPTBN5	BAALC	ARHGAP20	ADAMTS9	AKAP5	GABRA6	CCSAP	GABBR2	RFPL2	SLC38A3	CPLX2	LYSMD2	RASGEF1C
CAMKV	B3GALNT1	HTR5A	CASQ1	ADAMTS19	SLC24A3	ALK	KIAA1755	CGREF1	KCNC2	SPTBN5	DCLK3	PLD5	GABRB2	NCAM2	CCN3	VGF	BEND4	IGFBP3	SLC13A5	ZC3HAV1L
SAMD3	KCNK9	TSC22D3	MAP3K21	GABRB2	SV2B	NPTX1	CDH22	CUZD1	CLMP	CGREF1	GRIK2	GSX2	CPLX2	PDE1C	FHDC1	IRF5	INSYN2A	ZC3HAV1L	TMC6	LANCL3
HEY1	REPS2	GALNT14	ZNF385B	IGFBP3	FAM131C	KCND3	ADRA2A	DUSP5	PRKCB	SHISAL1	GPR85	FAM83H	IGFBP3	ADAMTS5	ARHGAP20	PUDP	RELN	LANCL3	CACNG8	ARHGAP36
PTCHD1	FRMPD4	ESYT3	PTH2R	ZC3HAV1L	MPPED1	APLN	KCNA6	PARP9	RAB31	LRIG3	NIPSNAP3B	CHRM2	ZC3HAV1L	CA10	PLCB2	CALY	PTPRT	ARHGAP36	C1orf216	MAL2
TMEM130	ADRA1B	NRG2	NYAP2	LANCL3	VSTM2B	CDK5R2	SLC25A4	LRIG3	LGI3	SYT16	DYNLT3	TMEM132C	LANCL3	MARVELD1	CGREF1	ASS1	PDGFA	SNTG1	CASQ1	FAM135B
TVP23A	EMX2	DYNC1I1	GRIP2	ARHGAP36	SMTNL2	KNDC1	BEND6	MDGA2	NPNT	LYSMD2	PTCHD1	KCNK4	HTR2C	PPARGC1B	CUZD1	ANGPTL6	CYRIA	MAL2	DYRK3	NFIB
ZCCHC18	GPR37	SLAMF8	IGSF11	MAL2	SLC38A3	PRNP	AP1S3	LYSMD2	IRS1	PRKACB	RET	GALNT9	SNTG1	ADCY8	DUSP5	SHFL	CHSY3	FAM135B	MAP3K21	PLPPR1
RAB31	TANC2	DUSP23	VWA5B2	FAM135B	NKAIN2	SYNPO	GUCY1A2	SLC13A5	ROR2	CASQ1	KCNC2	PCBP3	PLPPR1	SORCS3	MDGA2	SYNE1	LPAR1	NFIB	NYAP2	ST8SIA6
CPLX1	NPTX1	GJD2	OCIAD2	NFIB	PAQR9	EGFL7	JAKMIP1	TMC6	PARM1	MAP3K21	CLMP	EFHC2	ST8SIA6	FBXO32	KCNH5	F12	RASGEF1A	PLPPR1	MARCHF4	CDHR1
IRS1	KCND3	SCUBE1	GABRB2	PLPPR1	BEND4	MYO5B	AK5	CACNG8	RAB3B	PTH2R	TMEM130	NOG	KIAA1755	SYN2	LYSMD2	GALNT15	FOXO6	ST8SIA6	GRIP2	PLCH2
ROR2	APLN	ACE	CPLX2	KIAA1755	FAM78B	SH3RF3	THY1	C1orf216	PCDH7	MARCHF4	PRKCB	PRKD1	HMGA2	MX1	SLC13A5	RAMP2	C2orf72	CDH22	STAC	CDH22
KCNK9	CDK5R2	SLC37A1	RASGEF1C	CDH22	INSYN2A	VEGFB	CCSAP	PRKACB	REPS2	GRIP2	TVP23A	CLDN6	ADRA2A	CABP1	TMC6	PPP1R1B	DENND1B	HMGA2	MUC4	ADRA2A
PLEKHA2	DSCAM	SPTBN4	IGFBP3	ADRA2A	RELN	NWD2	NCAM2	DYRK3	FRMPD4	STAC	ZCCHC18	TCEAL2	KCNA6	CLSTN2	CACNG8	KHDRBS3	CPLX3	ADRA2A	VWA5B2	CACNA2D4
REPS2	KNDC1	LY6E	LANCL3	CACNA2D4	PTPRT	CNTNAP2	ADAMTS5	MAP3K21	GPR37	MUC4	RBFOX3	FMNL1	BEND6	DUSP23	C1orf216	GCH1	EML6	PTPRO	SLC26A1	KCNA6
MUC3A	PRNP	FBXO27	ARHGAP36	KCNA6	PDGFA	GALNTL6	CA10	ZNF385B	NEUROD2	VWA5B2	GNG8	ROBO2	AP1S3	GJD2	PRKACB	SLC6A11	SP9	FBXO4	OCIAD2	ME3
ADRA1B	ABCG4	NTN3	MAL2	SLC25A4	ADAMTSL2	SLCO2A1	MARVELD1	TMEFF2	DSCAM	SLC26A1	TMC8	GALNT17	GUCY1A2	SCUBE1	CASQ1	PPARG	FNDC10	BEND6	TIFA	SLC25A4
TANC2	EGFL7	CAMK2N1	NFIB	BEND6	LPAR1	FUT1	PPARGC1B	PTH2R	SYCE1	ADAMTS19	LGI3	FAM131C	STK32B	ACE	DYRK3	RAP1GAP2	CLEC2L	TMEM132D	ADAMTS19	FBXO4
ALK	SH3RF3	KCNJ9	PLPPR1	AP1S3	RYR2	CHST2	ADCY8	STAC	KNDC1	CPLX2	PHYHIP	AHNAK2	JAKMIP1	SLC37A1	MAP3K21	BCAN	SHISA9	AP1S3	GABRB2	BEND6
NPTX1	VEGFB	LRRTM1	ST8SIA6	GUCY1A2	RASGEF1A	CREG2	SORCS3	IGSF11	RASGRP1	IGFBP3	CPLX1	ADAMTSL5	SPHKAP	SPTBN4	ZNF385B	ATP8A2	SMKR1	SPOCK1	CPLX2	TMEM132D
KCND3	TTC9B	LRATD1	PLCH2	JAKMIP1	FOXO6	LY6H	FBXO32	VWA5B2	EGFL7	ZC3HAV1L	IRS1	NAP1L3	AK5	LY6E	TMEFF2	GPR12	CEBPA	GUCY1A2	CREB5	AP1S3
DSCAM	FUT1	KCNJ3	KIAA1755	AK5	C2orf72	FZD8	ATP2B2	TIFA	SH3RF3	NLGN4X	PARM1	CYP4X1	THY1	NTN3	PTH2R	CHRM3	TRNP1	JAKMIP1	IGFBP3	SPOCK1
KNDC1	CREG2	FBXO41	CDH22	THY1	CPLX3	ST8SIA3	SYN2	GABRB2	VEGFB	LANCL3	RAB3B	MPPED1	CCSAP	CAMK2N1	MARCHF4	POSTN	SALL3	AK5	HTR2C	GUCY1A2
PRNP	RIMKLA	VSNL1	ADRA2A	CCSAP	EML6	KCNJ10	MX1	CPLX2	NWD2	ARHGAP36	KCNK9	VSTM2B	NCAM2	WNT4	GRIP2	BEX2	FRRS1L	CCSAP	MAL2	STK32B
CALB2	KCNA3	SYNPR	ME3	NCAM2	SP9	KCTD12	CABP1	IGFBP3	VWC2L	MAL2	PCDH7	C11orf96	CA10	DIRAS3	VWA5B2	PDZD2	SRXN1	NCAM2	FAM135B	JAKMIP1
ABCG4	ST8SIA3	ADAMTS9	PTPRO	ADAMTS5	FNDC10	CPNE7	GALNT14	ZC3HAV1L	CNTNAP2	FAM135B	REPS2	NHS	MARVELD1	KCNJ9	OCIAD2	TMTC1	DOC2B	ADAMTS5	PLPPR1	AK5
EGFL7	KCNJ10	ABLIM2	SLC25A4	CA10	TMEM238	TMEM151A	CLSTN2	HTR2C	GALNTL6	NFIB	MUC3A	SMTNL2	GOLGA7B	KCNF1	SNCA	MEIS2		MARVELD1	ST8SIA6	CCSAP
SH3RF3	ZNF354C	CAMKV	BEND6	MARVELD1	SHISA9	FJX1	RNF207	ARHGAP36	TTC9B	ST8SIA6	ADRA1B	SLC38A3	SLC24A2	KCNJ3	ADAMTS19	TSPAN2		GOLGA7B	CDHR1	PDE1C
VEGFB	ALS2CL	GRIK2	TMEM132D	GOLGA7B	SMKR1	TSHZ1	NRG2	MAL2	CHST2	CDHR1	EMX2	CCDC9B	ADCY8	FBXO41	GABRB2	SYT6		PPARGC1B	PLCH2	ADAMTS5
ZCCHC12	CPNE7	TXLNB	SPOCK1	PPARGC1B	CEBPA	PLD5	DYNC1I1	FAM135B	NPPA	PLCH2	ALK	NKAIN2	SORCS3	SERPINI1	CPLX2	RERG		ADCY8	KIRREL3	CA10
TTC9B	TMEM151A	GPR85	GUCY1A2	ADCY8	TRNP1	GSX2	GJD2	NFIB	LY6H	KIAA1755	NPTX1	PAQR9	FBXO32	ADAMTS9	IGFBP3	ADAMTS8		FBXO32	KIAA1755	MARVELD1
CHST2	FJX1	CDCA7L	STK32B	SORCS3	SALL3	SHISA2	SCUBE1	ST8SIA6	RIMKLA	CDH22	KCND3	VWC2	ATP2B2	DCLK3	ZC3HAV1L	EPHA7		ATP2B2	CDH22	PPARGC1B
CREG2	SLITRK4	HEY1	JAKMIP1	FBXO32	FRRS1L	FAM83H	ACE	PLCH2	FZD8	HMGA2	CDK5R2	SHISA6	SYN2	CAMKV	LANCL3	LMO2		SYN2	HMGA2	ADCY8
LY6H	TSHZ1	BAALC	THY1	ATP2B2	SRXN1	ZNF572	SLC37A1	SCN2B	KCNJ10	ADRA2A	NEUROD2	INSYN2A	HTR5A	ITGA2	HTR2C	AGAP2		TSC22D3	ADRA2A	SORCS3
KCNA3	LYNX1	NIPSNAP3B	CCSAP	SYN2	DOC2B	TMEM132C	SPTBN4	KIAA1755	ALS2CL	CACNA2D4	SYCE1	RELN	TSC22D3	SAMD3	ARHGAP36	PKIB		CABP1	CACNA2D4	FBXO32
ST8SIA3	PLD5	PTCHD1	NCAM2	MX1		TMEM132E	LY6E	CDH22	AMER3	KCNA6	FUT9	PTPRT	MX1	GPR85	MAL2	MMD2		GALNT14	GABRA2	ATP2B2
KCNJ10	SSTR2	ADAMTS15	PDE1C	CLSTN2		ADGRB1	COL26A1	HMGA2	TMEM151A	PTPRO	VEGFB	SPOCK3	GALNT14	HEY1	FAM135B	KIF12		CLSTN2	BEND6	SYN2
ALS2CL	FAM83H	TMEM130	CA10	NRG2		RGS6	NTN3	ADRA2A	FJX1	SLC25A4	ZCCHC12	GRM7	ESYT3	BAALC	NFIB	GABBR2		RNF207	AP1S3	HTR5A
AMER3	PENK	PRKCB	MARVELD1	DYNC1I1		GALNT9	ELAVL4	KCNA6	AKAP5	FBXO4	CNTNAP2	LAMB3	DYNC1I1	NIPSNAP3B	PLPPR1	MYC		NRG2	SPOCK1	MX1
KCTD12	TMEM132C	TVP23A	GOLGA7B	DUSP23		FAM43B	CAMK2N1	SLC25A4	TSHZ1	BEND6	GALNTL6	OGDHL	VWA5B1	DYNLT3	ST8SIA6	HCRTR2		NBL1	GUCY1A2	CABP1
TMEM151A	GPR3	RBFOX3	PPARGC1B	GJD2		PCBP3	WNT4	FBXO4	PLD5	TMEM132D	SLCO2A1	PDGFA	CSRP1	PTCHD1	CDHR1	KIAA0319		VWA5B1	JAKMIP1	GALNT14
[illegible]	[illegible]	[illegible]	[illegible]	[illegible]		[illegible]	[illegible]	[illegible]	[illegible]	[illegible]	[illegible]	[illegible]	[illegible]	[illegible]	[illegible]	[illegible]		[illegible]	[illegible]	[illegible]

[illegible]	[illegible]	[illegible]	[illegible]	[illegible]	[illegible]	[illegible]	[illegible]	[illegible]	[illegible]	[illegible]	[illegible]	[illegible]	[illegible]	[illegible]	[illegible]	[illegible]	[illegible]	[illegible]
SLITRK4	FAM181B	RAB3IL1	ADCY8	ACE	ADRA2C	BRINP3	TMEM132D	SSTR2	RIT2	CHST2	CYRIA	SCUBE1	SPINT1	SCN2B	ARHGAP20	ACE	THY1	RNF207
AKAP5	KCNK4	LGI3	SORCS3	SLC37A1	ACOT1	KCNJ9	AP1S3	FAM83H	SPOCK1	PCSK1	LPAR1	ACE	TMEM130	KIAA1755	PLCB2	SLC37A1	CCSAP	DYNC1I1
PLD5	GALNT9	PHYHIP	FBXO32	SPTBN4	PRKD1	KCNF1	SPOCK1	RPH3AL	GUCY1A2	LY6H	CACNA1E	SLC37A1	PRKCB	CDH22	SPTBN5	SPTBN4	NCAM2	DUSP23
SSTR2	FAM43B	GFRA2	SST	LY6E	MAP7D2	LRATD1	GUCY1A2	TMEM132C	STK32B	DSCAML1	STUM	SPTBN4	TVP23A	ADRA2A	CUZD1	LY6E	PDE1C	NBL1
FAM83H	PCBP3	NPNT	ATP2B2	NTN3	FMNL1	KCNJ3	STK32B	GPR88	JAKMIP1	RIMKLA	FOXD6	LY6E	ZCCHC18	KCNA6	DUSP5	COL26A1	ADAMTS5	TNNI1
TMEM132C	MARCHF11	GRM5	SYN2	ELAVL4	ROBO2	FBXO41	JAKMIP1	GPR3	SPHKAP	ST8SIA3	TCEAL5	NTN3	GNG8	ME3	PARP9	FBXO27	GOLGA7B	SCUBE1
GPR3	NOG	CPLX1	HTR5A	CAMK2N1	SLC24A3	SERPINI1	AK5	ADGRB1	THY1	KCNJ10	TCEAL6	CAMK2N1	RAB26	SLC25A4	SHISAL1	CAMK2N1	GRIA1	ACE
FAM181B	ADRA2C	IRS1	CABP1	WNT4	INPP5J	ADAMTS9	THY1	FAM181B	CCSAP	AMER3	MAFB	WNT4	RAB3IL1	BEND6	MDGA2	WNT4	PPARGC1B	SLC37A1
GALNT9	CLDN6	PARM1	GALNT14	DIRAS3	GALNT17	DCLK3	CCSAP	RGS6	NCAM2	KCTD12	DPF3	DIRAS3	RAB31	AP1S3	KCNH5	DIRAS3	SORCS3	SPATC1L
PCBP3	FMNL1	RAB3B	ESYT3	BRINP3	AHNAK2	CAMKV	PDE1C	GALNT9	PDE1C	FJX1	DENND1B	BRINP3	LGI3	SPOCK1	LYSMD2	LRRTM1	FBXO32	SPTBN4
MARCHF11	SLC24A3	KCNK9	CLSTN2	KCNJ9	KCNQ5	GUCY1A1	CA10	FAM43B	ADAMTS5	LACC1	CPLX3	KCNJ9	CPLX1	GUCY1A2	SLC13A5	KCNF1	ATP2B2	LY6E
NOG	GALNT17	PLEKHA2	RNF207	KCNJ3	ADAMTSL5	ITGA2	MARVELD1	PCBP3	CA10	AKAP5	HMX1	KCNF1	IRS1	STK32B	TMC6	ADAMTS9	SYN2	NTN3
ADRA2C	FAM131C	PCDH7	NRG2	FBXO41	GABRA5	SAMD3	GOLGA7B	MARCHF11	MARVELD1	PLD5	TSTD1	KCNJ3	ROR2	JAKMIP1	CACNG8	DCLK3	HTR5A	CAMK2N1
PRKD1	AHNAK2	REPS2	DYNC1I1	SYNPR	PDE2A	GPR85	PPARGC1B	NOG	GOLGA7B	CHRM2	AJM1	FBXO41	KCNK9	AK5	C1orf216	EXO5	TSC22D3	WNT4
MAP7D2	KCNQ5	MUC3A	TNNI1	ADAMTS9	MPPED1	HEY1	SLC24A2	ADRA2C	GRIA1	PENK	SHISA9	VSNL1	PLEKHA2	THY1	PRKACB	HPGD	GALNT14	LRRTM1
TCEAL2	ADAMTSL5	FRMPD4	CSRP1	DCLK3	VSTM2B	BAALC	ADCY8	ACOT1	PPARGC1B	GPR88	CEBPA	COL6A3	REPS2	CCSAP	CASQ1	ITGA2	ESYT3	KCNF1
FMNL1	GABRA5	GPR37	GJD2	CAMKV	SHTN1	NIPSNAP3B	SORCS3	PRKD1	SLC24A2	GPR3	TRNP1	SERPINI1	MUC3A	NCAM2	DYRK3	GRIK2	CLSTN2	KCNJ3
SLC24A3	PDE2A	TANC2	SCUBE1	ITGA2	SLC38A3	PTCHD1	FBXO32	SLC24A3	ADCY8	ADGRB1	SALL3	ADAMTS9	FRMPD4	PDE1C	MAP3K21	CDCA7L	NRG2	FBXO41
GALNT17	MPPED1	NPTX1	ACE	SAMD3	COL25A1	RET	ATP2B2	GALNT17	SORCS3	FAM181B	TMEM179	DCLK3	ADRA1B	ADAMTS5	SLC4A10	HEY1	DYNC1I1	SYNPR
SV2B	VSTM2B	KCND3	SLC37A1	GPR85	CCDC9B	SPINT1	SYN2	SV2B	FBXO32	KCNK4	FRRS1L	CAMKV	GPR37	CA10	ZNF385B	BAALC	SLAMF8	DCLK3
FAM131C	GCNT1	NEUROD2	SPATC1L	HEY1	NKAIN2	PRKCB	TSC22D3	AHNAK2	SST	GALNT9	SRXN1	GRIK2	TANC2	MARVELD1	TMEFF2	FZD6	DUSP23	EXO5
AHNAK2	C11orf96	PRNP	SPTBN4	BAALC	SHISA6	TVP23A	MX1	KCNQ5	ATP2B2	BEGAIN	DOC2B	FABP7	ALK	GOLGA7B	PTH2R	NIPSNAP3B	VWA5B1	CAMKV
KCNQ5	NKAIN2	ABCG4	LY6E	NIPSNAP3B	INSYN2A	ZCCHC18	CABP1	ADAMTSL5	SYN2	FAM43B	GRIN2B	SAMD3	NPTX1	PPARGC1B	NYAP2	PTCHD1	GJD2	GUCY1A1
ADAMTSL5	PAQR9	FUT9	COL26A1	PTCHD1	RELN	GNG8	GALNT14	GABRA5	HTR5A	PCBP3		HEY1	KCND3	SLC24A2	MARCHF4	RET	SCUBE1	HPGD
PDE2A	SHISA6	EGFL7	NTN3	RET	PTPRT	RAB26	ESYT3	CYP4X1	TSC22D3	MARCHF11		BAALC	DSCAM	ADCY8	GRIP2	KCNC2	ACE	GRIK2
MPPED1	BEND4	VEGFB	CAMK2N1	KCNC2	SLC6A17	RAB3IL1	CLSTN2	GCNT1	MX1	EFHC2		FZD6	KNDC1	SORCS3	STAC	ADAMTS15	SLC37A1	SAMD3
VSTM2B	FAM78B	VWC2L	WNT4	SPINT1	ADGRA1	RAB31	NRG2	SLC38A3	GALNT14	NOG		NIPSNAP3B	PRNP	FBXO32	MUC4	SPINT1	SPTBN4	CDCA7L
SHTN1	INSYN2A	CNTNAP2	KCNF1	PRKCB	OGDHL	LGI3	DYNC1I1	CCDC9B	ESYT3	OPCML		DYNLT3	CALB2	SST	VWA5B2	TMEM130	LY6E	HEY1
C11orf96	RELN	TTC9B	LRATD1	TVP23A	PDGFA	CPLX1	TNNI1	NKAIN2	CLSTN2	ADRA2C		PTCHD1	ABCG4	ATP2B2	SLC26A1	PRKCB	COL26A1	NIPSNAP3B
SMTNL2	MAOA	SLCO2A1	KCNJ3	ZCCHC18	ADAMTSL2	IRS1	SCUBE1	PAQR9	RNF207	KCNJ12		RET	FUT9	SYN2	OCIAD2	RBFOX3	FBXO27	DYNLT3
SLC38A3	PTPRT	CHST2	GABRB1	RBFOX3	CYRIA	ROR2	ACE	VWC2	NRG2	PABPC1L2B		KCNC2	EGFL7	MX1	SNCA	GNG8	NTN3	PTCHD1
NKAIN2	FUT4	GAL3ST3	COL6A3	GNG8	MBP	RAB3B	SLC37A1	SHISA6	DYNC1I1	CLDN6		SPINT1	SH3RF3	CABP1	TIFA	TMC8	ELAVL4	RET
PAQR9	SLC30A10	PCSK1	SYNPR	TMC8	CHSY3	KCNK9	SPATC1L	FAM78B	VWA5B1	TCEAL2		PRKCB	VEGFB	GALNT14	CDH18	RAB26	CAMK2N1	BTNL9
BEND4	SLC6A17	ARL4D	DCLK3	RAB26	LPAR1	PLEKHA2	LY6E	INSYN2A	CSRP1	FMNL1		RBFOX3	NWD2	ESYT3	LIX1	RAB3IL1	KCNJ9	SPINT1
FAM78B	ADGRA1	LY6H	EXO5	RAB31	TMEM229B	REPS2	COL26A1	RELN	GJD2	ROBO2		GNG8	ZCCHC12	CLSTN2	ADAMTS19	LGI3	FCRLB	TMEM130
INSYN2A	PDGFA	DSCAML1	CAMKV	LGI3	CACNA1E	MUC3A	NTN3	PTPRT	SCUBE1	SLC24A3		RAB26	CNTNAP2	NRG2	GABRB2	PHYHIP	LRRTM1	PRKCB
RELN	DPP4	KCNA3	GUCY1A1	CPLX1	RYR2	FRMPD4	CAMK2N1	LAMB3	ACE	INPP5J		RAB3IL1	TTC9B	DYNC1I1	CPLX2	GRM5	LRATD1	ZCCHC18
PTPRT	ADAMTSL2	ST8SIA3	HPGD	IRS1	RIPPLY2	ADRA1B	WNT4	FAM163B	SLC37A1	KCNQ5		RAB31	SLCO2A1	GJD2	NLGN4X	CPLX1	KCNJ3	TMC8
FUT4	MBP	KCNJ10	ITGA2	ROR2	FOXD6	EMX2	LRRTM1	ADGRA1	SPATC1L	ADAMTSL5		LGI3	CHST2	SCUBE1	LANCL3	ROR2	FBXO41	RAB26
OGDHL	CHSY3	AMER3	GRIK2	RAB3B	TCEAL6	GPR37	KCNF1	OGDHL	LY6E	GABRA5		CPLX1	CREG2	ACE	HTR2C	B3GALNT1	VSNL1	RAB3IL1
PDGFA	LPAR1	KCTD12	SAMD3	B3GALNT1	MAFB	TANC2	LRATD1	PDGFA	COL26A1	CYP4X1		IRS1	LY6H	SLC37A1	ARHGAP36	KCNK9	GABRB1	FAM107A
CYRIA	TMEM229B	RPP25	CDCA7L	KCNK9	C2orf72	ALK	SYNPR	DPP4	FBXO27	PDE2A		PARM1	DSCAML1	SPATC1L	GNRH1	PLEKHA2	SYNPR	LGI3
CHSY3	RYR2	CPNE7	HEY1	PLEKHA2	HMX1	NPTX1	ADAMTS9	ADAMTSL2	NTN3	VSTM2B		RAB3B	KCNA3	SPTBN4	SNTG1	PCDH7	ADAMTS9	PHYHIP
LPAR1	C2orf72	FAM133A	BAALC	REPS2	PLXNA4	KCND3	DCLK3	S100A6	FBLIM1	C11orf96		KCNK9	ST8SIA3	LY6E	MAL2	ADRA1B	ABLIM2	GFRA2
RYR2	COL15A1	FJX1	NIPSNAP3B	MUC3A	FNDC10	DSCAM	CAMKV	MBP	CAMK2N1	C17orf97		PLEKHA2	KCNJ10	COL26A1	FAM135B	EMX2	CAMKV	NPNT
RASGEF1A	DPF3	SLITRK4	DYNLT3	FRMPD4	TMEM238	KNDC1	GUCY1A1	CHSY3	WNT4	COL4A5		PCDH7	AMER3	NTN3	PLPPR1	GPR37	GUCY1A1	GRM5
STUM	DENND1B	AKAP5	PTCHD1	ADRA1B	CLEC2L	PRNP	GRIK2	TMEM229B	BRINP3	ENO4		REPS2	KCTD12	CAMK2N1	ST8SIA6	TANC2	GRIK2	CPLX1
FOXD6	FAM24B	PLD5	RET	TANC2	OR2W3	CALB2	CDCA7L	RYR2	LRRTM1	COL25A1		FRMPD4	TMEM151A	WNT4	CDHR1	ALK	TXLNB	IRS1
C2orf72	UBE2QL1	TSPYL5	ADAMTS15	ALK	PSMB9	ABCG4	FZD6	STUM	LRATD1	PAQR9		ADRA1B	SLITRK4	DIRAS3	PLCH2	NPTX1	SAMD3	ROR2
[illegible]	[illegible]	[illegible]	[illegible]	[illegible]	[illegible]	[illegible]	[illegible]	[illegible]	[illegible]	[illegible]		[illegible]	[illegible]	[illegible]	[illegible]	[illegible]	[illegible]	[illegible]

DENND1B
CPLX3
EML6
UBE2QL1
HMX1
SP9
PLXNA4
FNDC10
CLEC2L
SHISA9
SMKR1
CEBPA
TRNP1
SALL3
FRRS1L
SRXN1
DOC2B

PLXNA4
TMEM238
CLEC2L
PSMB9
CEBPA
TRNP1
SALL3
FRRS1L
ZIM2
DOC2B

SSTR2
FAM83H
CHRM2
PENK
TMEM132E
GPR88
FAM181B
KCNK4
GALNT9
BEGAIN
FAM43B
CALN1
PCBP3
MARCHF11
NOG
ADRA2C
PRKD1
CLDN6
TCEAL2
FMNL1
ROBO2
SLC24A3
INPP5J
FAM131C
AHNAK2
KCNQ5
ADAMTSL5
NAP1L3
CYP4X1
PDE2A
MPPED1
VSTM2B
SHTN1
C11orf96
COL4A5
SMTNL2
COL25A1
CCDC9B
PAQR9
VWC2
SHISA6
BEND4
INSYN2A
IL1RAPL2
SPOCK3
GRM7
CPNE4
SLC30A10
LAMB3

CLMP
TMEM130
PRKCB
TVP23A
ZCCHC18
GNG8
TMC8
RAB3IL1
RAB31
LGI3
NPNT
GRM5
CPLX1
IRS1
ROR2
RAB3B
B3GALNT1
KCNK9
PLEKHA2
PCDH7
REPS2
ADRA1B
GPR37
TANC2
NPTX1
APLN
CDK5R2
SYCE1
KNDC1
CALB2
ABCG4
FUT9
METAP1D
EGFL7
VEGFB
GALNTL6
TTC9B
SLCO2A1
FUT1
CHST2
PCSK1
LY6H
DSCAML1
RIMKLA
KCNA3
FZD8
ST8SIA3
ZNF354C
ALS2CL

KCND3
CDK5R2
KNDC1
PRNP
CALB2
ABCG4
FUT9
EGFL7
SH3RF3
VEGFB
ZCCHC12
CNTNAP2
TTC9B
CHST2
GAL3ST3
CREG2
LY6H
DSCAML1
KCNA3
FZD8
ST8SIA3
KCNJ10
ALS2CL
AMER3
KCTD12
CPNE7
ALOXE3
TMEM151A
FJX1
SLITRK4
LACC1
AKAP5
LYNX1
PLD5
GSX2
SSTR2
FAM83H
PENK
TMEM132C
TMEM132E
GPR3
KCNK4
GALNT9
CTNNA3
PCBP3
MARCHF11
NOG
ADRA2C
PRKD1

CEBPA
TMEM179
GOLGA8L7
DOC2B
GRIN2B

EGFL7
SH3RF3
VEGFB
NWD2
ZCCHC12
CNTNAP2
GALNTL6
TTC9B
SLCO2A1
CHST2
LY6H
DSCAML1
KCNA3
ST8SIA3
KCNJ10
ALS2CL
AMER3
KCTD12
CPNE7
ALOXE3
TMEM151A
FJX1
SLITRK4
LACC1
AKAP5
PLD5
GSX2
SSTR2
FAM83H
PENK
TMEM132C
TMEM132E
GPR3
FAM181B
KCNK4
GALNT9
BEGAIN
CTNNA3
PCBP3
MARCHF11
NOG
ADRA2C
PRKD1
MAP7D2
TCEAL2
FMNL1
ROBO2
SLC24A3
GALNT17

PTCHD1
RET
PTER
ADAMTS15
SPINT1
CLMP
TMEM130
PRKCB
TVP23A
ZCCHC18
ISLR2
GNG8
TMC8
RAB26
RAB3IL1
TAP1
RAB31
LGI3
NPNT
CPLX1
IRS1
ROR2
PARM1
RAB3B
B3GALNT1
KCNK9
PLEKHA2
REPS2
MUC3A
FRMPD4
ADRA1B
EMX2
GPR37
TANC2
ALK
NPTX1
KCND3
APLN
CDK5R2
DSCAM
SYCE1
KNDC1
PRNP
SYNPO
CALB2
ABCG4
EGFL7
VEGFB
TTC9B

C2orf72
COL15A1
S1PR3
HMX1
TSTD1
PLXNA4
CENPVL1
AJM1
TMEM238
CLEC2L
TRNP1
SALL3
TMEM179
FRRS1L
ZIM2
SRXN1
DOC2B

FBXO41
SERPINI1
ADAMTS9
DCLK3
ABLIM2
EXO5
CAMKV
ITGA2
GRIK2
SAMD3
CDCA7L
HEY1
BAALC
DYNLT3
PTCHD1
ADAMTS15
SPINT1
PRKCB
ZCCHC18
GNG8
TBX10
TMC8
RAB26
FAM107A
TAP1
LGI3
PHYHIP
NPNT
IRS1
PARM1
KCNK9
PLEKHA2
PCDH7
REPS2
MUC3A
FRMPD4
ADRA1B
EMX2
SEMA3E
GPR37
TANC2
ALK
NPTX1
KCND3
APLN
CDK5R2
NEUROD2
SYCE1
KNDC1

SHISA6
INSYN2A
PTPRT
SPOCK3
GRM7
DCHS2
OGDHL
PDGFA
H4C16
ADAMTSL2
CYRIA
S100A6
CHSY3
CACNA1E
LRRTM3
BRINP2
STUM
FOXO6
TCEAL5
TCEAL6
C2orf72
COL15A1
DENND1B
CPLX3
HMX1
PLXNA4
FNDC10
AJM1
CLEC2L
SHISA9
TRNP1
SALL3
TMEM179
FRRS1L
GRIN2B

GPR37
TANC2
HS6ST2
ALK
NPTX1
KCND3
CDK5R2
NEUROD2
SYCE1
KNDC1
PRNP
SYNPO
CALB2
ABCG4
FUT9
EGFL7
VEGFB
ZCCHC12
CNTNAP2
GALNTL6
TTC9B
SLCO2A1
CHST2
CREG2
ARL4D
LY6H
DSCAML1
KCNA3
FZD8
ST8SIA3
KCNJ10
ALS2CL
KCTD12
RPP25
CPNE7
FJX1
SLITRK4
LACC1
AKAP5
LYNX1
PLD5
GSX2
FAM83H
CHRM2
PENK
TMEM132C
TMEM132E
GPR3
KCNK4

PLD5
GSX2
SSTR2
FAM83H
TMEM132C
TMEM132E
GPR3
ADGRB1
FAM181B
GALNT9
BEGAIN
CTNNA3
PCBP3
MARCHF11
NOG
ADRA2C
PRKD1
MAP7D2
TCEAL2
FMNL1
ROBO2
SLC24A3
SV2B
FAM131C
ADAMTSL5
MPPED1
VSTM2B
SHTN1
NHS
SMTNL2
SLC38A3
NKAIN2
PAQR9
SHISA6
BEND4
FAM78B
INSYN2A
RELN
PTPRT
OGDHL
PDGFA
ADAMTSL2
CYRIA
S100A6
RYR2
LRRTM3
RASGEF1A
STUM
FOXO6

KCNJ9
LRRTM1
KCNF1
LRATD1
KCNJ3
FBXO41
SERPINI1
SYNPR
ADAMTS9
DCLK3
CAMKV
ITGA2
SAMD3
GPR85
HEY1
BAALC
FZD6
NIPSNAP3B
PTCHD1
RET
PTER
SPINT1
CLMP
TMEM130
PRKCB
TVP23A
ZCCHC18
GNG8
TMC8
RAB26
RAB3IL1
RAB31
LGI3
GRM5
CPLX1
IRS1
ROR2
RAB3B
KCNK9
PLEKHA2
PCDH7
REPS2
MUC3A
FRMPD4
ADRA1B
GPR37
TANC2
ALK
NPTX1

SCN2B
KIAA1755
CDH22
ADRA2A
CACNA2D4
KCNA6
ME3
SLC25A4
FBXO4
BEND6
TMEM132D
AP1S3
SPOCK1
GUCY1A2
JAKMIP1
SPHKAP
AK5
THY1
CCSAP
PDE1C
ADAMTS5
CA10
MARVELD1
GOLGA7B
GRIA1
PPARGC1B
SLC24A2
ADCY8
SLA
SORCS3
FBXO32
SST
ATP2B2
SYN2
TSC22D3
MX1
CABP1
GALNT14
ESYT3
CLSTN2
RNF207
NRG2
DYNC1I1
NBL1
VWA5B1
TNNI1
GJD2
SCUBE1
SLC37A1

APLN
NEUROD2
DSCAM
SYCE1
KNDC1
PRNP
METAP1D
EGFL7
MYD88
SH3RF3
VEGFB
CNTNAP2
GALNTL6
SLCO2A1
CHST2
PCSK1
CREG2
ARL4D
LY6H
DSCAML1
RIMKLA
KCNA3
FZD8
KCNJ10
ALS2CL
AMER3
KCTD12
RPP25
ALOXE3
TMEM151A
FJX1
LACC1
AKAP5
ZNF154
TSHZ1
LYNX1
PLD5
TSPYL5
GSX2
SSTR2
FAM83H
RPH3AL
CHRM2
PENK
TMEM132C
TMEM132E
GPR3
ADGRB1
FAM181B

HEY1
BAALC
FZD6
NIPSNAP3B
PTCHD1
ADAMTS15
CLMP
TMEM130
PRKCB
TVP23A
ISLR2
RBFOX3
GNG8
RAB3IL1
TAP1
LGI3
PHYHIP
NPNT
GRM5
CPLX1
IRS1
PARM1
RAB3B
KCNK9
PLEKHA2
PCDH7
REPS2
MUC3A
FRMPD4
EMX2
GPR37
TANC2
NPTX1
KCND3
NEUROD2
DSCAM
SYCE1
KNDC1
PRNP
ABCG4
FUT9
EGFL7
VEGFB
VWC2L
CNTNAP2
GALNTL6
TTC9B
SLCO2A1
FUT1

RAB3B
B3GALNT1
KCNK9
PLEKHA2
PCDH7
REPS2
ADRA1B
GPR37
TANC2
NPTX1
KCND3
APLN
SYCE1
KNDC1
PRNP
SYNPO
ABCG4
FUT9
EGFL7
SH3RF3
VEGFB
NWD2
ZCCHC12
CNTNAP2
GALNTL6
TTC9B
SLCO2A1
FUT1
CHST2
NPPA
CREG2
LY6H
DSCAML1
RIMKLA
KCNA3
FZD8
ST8SIA3
ALS2CL
AMER3
KCTD12
RPP25
CPNE7
ALOXE3
TMEM151A
FJX1
SLITRK4
AKAP5
TSHZ1
LYNX1

OGDHL
PDGFA
DPP4
S100A10
ADAMTSL2
CYRIA
S100A6
MBP
DLGAP2
CACNA1E
LRRTM3
STUM
FOXO6
TCEAL5
TCEAL6
CPLX3
EML6
C10orf105
HMX1
SP9
FNDC10
SHISA9
CEBPA
SALL3
TMEM179
FRRS1L
ZIM2
SRXN1
DOC2B
GRIN2B

KCTD12
RPP25
CPNE7
ALOXE3
TMEM151A
FJX1
LACC1
AKAP5
ZNF154
TSHZ1
LYNX1
PLD5
TSPYL5
SSTR2
SHISA2
FAM83H
PENK
TMEM132C
TMEM132E
DDX60L
GPR88
GPR3
FAM181B
KCNK4
GALNT9
BEGAIN
FAM43B
PCBP3
MARCHF11
NOG
OPCML
ADRA2C
ACOT1
PRKD1
MAP7D2
CLDN6
FMNL1
ROBO2
SLC24A3
GALNT17
AHNAK2
KCNQ5
ADAMTSL5
GABRA5
CYP4X1
MPPED1
VSTM2B
SHTN1
GCNT1
C11orf96
C17orf97

PABPC1L2B
TCEAL2
FMNL1
ROBO2
SLC24A3
SV2B
AHNAK2
KCNQ5
ADAMTSL5
PDE2A
MPPED1
VSTM2B
SHTN1
NHS
SMTNL2
SLC38A3
NKAIN2
BEND4
FAM78B
INSYN2A
RELN
PTPRT
SLC30A10
DCHS2
OGDHL
PDGFA
S100A10
H4C16
ADAMTSL2
CYRIA
S100A6
MBP
CHSY3
LPAR1
TMEM229B
RYR2
LRRTM3
RASGEF1A
STUM
FOXO6
TCEAL5
TCEAL6
C2orf72
DPF3
C22orf42
DENND1B
CPLX3
EML6
UBE2QL1
HMX1
TSTD1

FAM131C
ADAMTSL5
MPPED1
VSTM2B
SHTN1
NHS
SMTNL2
SLC38A3
NKAIN2
VWC2
BEND4
FAM78B
INSYN2A
RELN
PTPRT
SLC30A10
OGDHL
PDGFA
H4C16
ADAMTSL2
CYRIA
S100A6
MBP
CHSY3
LPAR1
TMEM229B
RYR2
LRRTM3
RASGEF1A
STUM
FOXO6
TCEAL6
C2orf72
C22orf42
DENND1B
CPLX3
EML6
UBE2QL1
HMX1
TSTD1
SP9
FNDC10
AJM1
TMEM238
CLEC2L
SHISA9
SMKR1
CEBPA
TRNP1
SALL3
FRRS1L

CHST2
PCSK1
CREG2
LY6H
RIMKLA
KCNA3
FZD8
ST8SIA3
KCNJ10
ZNF354C
ALS2CL
AMER3
KCTD12
CPNE7
TMEM151A
FJX1
AKAP5
TSHZ1
LYNX1
PLD5
TSPYL5
GSX2
SSTR2
SHISA2
FAM83H
CHRM2
PENK
TMEM132C
GPR3
ADGRB1
RGS6
GALNT9
BEGAIN
FAM43B
CALN1
PCBP3
MARCHF11
NOG
OPCML
ADRA2C
KCNJ12
PRKD1
PABPC1L2B
CLDN6
FMNL1
SLC24A3
INPP5J
GALNT17
FAM131C
AHNAK2
C11orf87

ABCG4
EGFL7
SH3RF3
ARPP21
VEGFB
VWC2L
ZCCHC12
CNTNAP2
GALNTL6
TTC9B
SLCO2A1
FUT1
CHST2
NPPA
CREG2
LY6H
DSCAML1
RIMKLA
KCNA3
FZD8
ST8SIA3
KCNJ10
ZNF354C
ALS2CL
AMER3
KCTD12
RPP25
CPNE7
FAM133A
TMEM151A
FJX1
LACC1
AKAP5
ZNF154
TSHZ1
LYNX1
PLD5
TSPYL5
GSX2
SSTR2
SHISA2
ZNF572
RPH3AL
PENK
TMEM132C
TMEM132E
GPR3
ADGRB1
MGAT4C
KCNK4
RGS6

GALNT9
FAM43B
CALN1
CTNNA3
PCBP3
MARCHF11
NOG
ADRA2C
PRKD1
MAP7D2
PABPC1L2B
CLDN6
TCEAL2
FMNL1
ROBO2
SLC24A3
SV2B
FAM131C
AHNAK2
C11orf87
ADAMTSL5
GABRA5
CYP4X1
PDE2A
MPPED1
VSTM2B
C11orf96
NHS
SMTNL2
SLC38A3
CCDC9B
NKAIN2
PAQR9
VWC2
SHISA6
BEND4
FAM78B
INSYN2A
RELN
PTPRT
GRM7
LAMB3
DCHS2
PDGFA
COL4A6
DPP4
H4C16
ADAMTSL2
CYRIA
S100A6
MBP

C2orf72
C22orf42
DENND1B
CPLX3
EML6
HMX1
TSTD1
SP9
FNDC10
TMEM238
CLEC2L
SHISA9
SMKR1
CEBPA
TRNP1
CTSO
SALL3
FRRS1L
SRXN1
DOC2B

DSCAM
KNDC1
PRNP
CALB2
ABCG4
FUT9
EGFL7
SH3RF3
VEGFB
NWD2
ZCCHC12
CNTNAP2
TTC9B
SLCO2A1
FUT1
CHST2
GAL3ST3
PCSK1
CREG2
LY6H
DSCAML1
KCNA3
FZD8
ST8SIA3
KCNJ10
ALS2CL
AMER3
KCTD12
CPNE7
TMEM151A
FJX1
SLITRK4
LACC1
AKAP5
LYNX1
PLD5
GSX2
SSTR2
SHISA2
FAM83H
PENK
TMEM132C
TMEM132E
GPR3
FAM181B
GALNT9
BEGAIN
FAM43B
CTNNA3
PCBP3
MARCHF11

LY6E
COL26A1
FBXO27
NTN3
CAMK2N1
WNT4
DIRAS3
BRINP3
VCAM1
KCNJ9
FCRLB
LRRTM1
KCNF1
LRATD1
KCNJ3
FBXO41
VSNL1
GABRB1
SERPINI1
SYNPR
ADAMTS9
DCLK3
ABLIM2
EXO5
CAMKV
GUCY1A1
HPGD
GRIK2
SAMD3
GPR85
CDCA7L
HEY1
BAALC
FZD6
PTCHD1
RET
BTNL9
KCNC2
SPINT1
CLMP
TMEM130
TVP23A
ZCCHC18
RBFOX3
GNG8
TBX10
TMC8
RAB26
RAB3IL1
FAM107A
TAP1

RGS6
GALNT9
CTNNA3
PCBP3
MARCHF11
NOG
ADRA2C
PRKD1
MAP7D2
PABPC1L2B
CLDN6
TCEAL2
FMNL1
ROBO2
SLC24A3
GALNT17
SV2B
FAM131C
AHNAK2
KCNQ5
GABRA5
CYP4X1
PDE2A
MPPED1
VSTM2B
SHTN1
GCNT1
C11orf96
NHS
SMTNL2
SLC38A3
NKAIN2
PAQR9
SHISA6
BEND4
FAM78B
INSYN2A
RELN
IL1RAPL2
PTPRT
SPOCK3
FUT4
SLC30A10
FAM163B
SLC6A17
ADGRA1
DCHS2
OGDHL
PDGFA
COL4A6
ADAMTSL2

NPPA
GAL3ST3
PCSK1
CREG2
LY6H
DSCAML1
RIMKLA
KCNA3
ST8SIA3
KCNJ10
AMER3
KCTD12
RPP25
CPNE7
FAM133A
FJX1
SLITRK4
AKAP5
LYNX1
PLD5
TSPYL5
GSX2
FAM83H
PENK
TMEM132C
GPR88
ADGRB1
FAM181B
RGS6
GALNT9
BEGAIN
GPC6
FAM43B
CALN1
PCBP3
MARCHF11
NOG
OPCML
ADRA2C
KCNJ12
PRKD1
PABPC1L2B
CLDN6
TCEAL2
ROBO2
SLC24A3
INPP5J
GPRIN3
FAM131C
AHNAK2
C11orf87

TSPYL5
PENK
TMEM132E
ADGRB1
FAM181B
KCNK4
GALNT9
BEGAIN
FAM43B
CALN1
PCBP3
MARCHF11
EFHC2
NOG
OPCML
ADRA2C
KCNJ12
PRKD1
MAP7D2
PABPC1L2B
CLDN6
TCEAL2
FMNL1
SLC24A3
GALNT17
FAM131C
AHNAK2
C11orf87
ADAMTSL5
GABRA5
MPPED1
VSTM2B
GCNT1
C11orf96
C17orf97
NHS
SMTNL2
ENO4
SLC38A3
CCDC9B
NKAIN2
PAQR9
VWC2
SHISA6
BEND4
FAM78B
INSYN2A
RELN
PTPRT
GRM7
LAMB3

SMTNL2
ENO4
COL25A1
NKAIN2
PAQR9
VWC2
SHISA6
FAM78B
INSYN2A
RELN
IL1RAPL2
MAOA
GRM7
FUT4
SLC30A10
FAM163B
SLC6A17
OGDHL
PDGFA
S100A6
MBP
CHSY3
LPAR1
CACNA1E
CCDC152
RASGEF1A
STUM
FOXO6
TCEAL6
MAFB
C2orf72
DPF3
DENND1C
DENND1B
CPLX3
EML6
HMX1
TSTD1
PLXNA4
CENPVL1
CENPVL3
FNDC10
RAB6D
TMEM238
CLEC2L

PLXNA4
FNDC10
AJM1
TMEM238
CLEC2L
SHISA9
PSMB9
SMKR1
CEBPA
TRNP1
SALL3
TMEM179
FRRS1L
SRXN1
DOC2B

DOC2B

ADAMTSL5
GABRA5
PDE2A
MPPED1
VSTM2B
SHTN1
GCNT1
C11orf96
C17orf97
NHS
SMTNL2
SLC38A3
CCDC9B
PAQR9
VWC2
SHISA6
BEND4
FAM78B
INSYN2A
RELN
MAOA
PTPRT
FUT4
SLC30A10
LAMB3
FAM163B
SLC6A17
ADGRA1
OGDHL
PDGFA
DPP4
CYRIA
S100A6
MBP
CHSY3
LPAR1
TMEM229B
RYR2
BRINP2
STUM
RIPPLY2
FOXO6
MAFB
C2orf72
COL15A1

GPC6
FAM43B
CALN1
CTNNA3
PCBP3
MARCHF11
NOG
OPCML
ADRA2C
ACOT1
PRKD1
MAP7D2
PABPC1L2B
TCEAL2
FMNL1
ROBO2
SLC24A3
INPP5J
GALNT17
SV2B
FAM131C
AHNAK2
C11orf87
KCNQ5
ADAMTSL5
GABRA5
NAP1L3
CYP4X1
PDE2A
VSTM2B
SHTN1
C11orf96
NHS
SMTNL2
ENO4
SLC38A3
COL25A1
CCDC9B
NKAIN2
PAQR9
VWC2
BEND4
FAM78B
INSYN2A
IL1RAPL2

LPAR1
TMEM229B
CACNA1E
RYR2
LRRTM3
BRINP2
STUM
RIPPLY2
FOXO6
TCEAL5
TCEAL6
C2orf72
DPF3
DENND1C
C22orf42
CPLX3
UBE2QL1
HMX1
TSTD1
PLXNA4
FNDC10
AJM1
TMEM238
CLEC2L
SHISA9
SMKR1
CEBPA
TRNP1
SALL3
TMEM179
FRRS1L
GOLGA8L7
SRXN1
DOC2B
GRIN2B

OPCML
ADRA2C
PRKD1
MAP7D2
TCEAL2
FMNL1
ROBO2
SLC24A3
GALNT17
SV2B
FAM131C
AHNAK2
KCNQ5
ADAMTSL5
GABRA5
PDE2A
MPPED1
VSTM2B
SHTN1
GCNT1
C11orf96
C17orf97
NHS
SMTNL2
SLC38A3
NKAIN2
PAQR9
VWC2
SHISA6
BEND4
FAM78B
INSYN2A
RELN
MAOA
PTPRT
SPOCK3
FUT4
SLC30A10
DCHS2
OGDHL
PDGFA
COL4A6
S100A10
H4C16
ADAMTSL2

LGI3
PHYHIP
GFRA2
GRM5
CPLX1
IRS1
ROR2
RAB3B
B3GALNT1
KCNK9
PLEKHA2
REPS2
MUC3A
FRMPD4
ADRA1B
EMX2
GPR37
TANC2
ALK
SOSTDC1
NPTX1
KCND3
APLN
CDK5R2
NEUROD2
SYCE1
KNDC1
PRNP
SYNPO
CALB2
ABCG4
FUT9
EGFL7
MYD88
VEGFB
NWD2
VWC2L
ZCCHC12
GALNTL6
TTC9B
SLCO2A1
CHST2
NPPA
GAL3ST3
PCSK1

MBP
DLGAP2
CHSY3
LPAR1
TMEM229B
RYR2
RASGEF1A
STUM
FOXO6
TCEAL5
TCEAL6
MAFB
C2orf72
COL15A1
DPF3
DENND1B
CPLX3
EML6
UBE2QL1
HMX1
SP9
PLXNA4
CENPVL1
CENPVL3
FNDC10
TMEM238
CLEC2L
SMKR1
CEBPA
TRNP1
CTSO
SALL3
TMEM179
FRRS1L
ZIM2
SRXN1
DOC2B

ADAMTSL5
GABRA5
NAP1L3
CYP4X1
PDE2A
MPPED1
VSTM2B
C11orf96
C17orf97
COL4A5
NHS
SMTNL2
ENO4
SLC38A3
COL25A1
CCDC9B
PAQR9
VWC2
SHISA6
BEND4
FAM78B
RELN
IL1RAPL2
SPOCK3
GRM7
CPNE4
SLC30A10
LAMB3
FAM163B
ADGRA1
OGDHL
PDGFA
DPP4
S100A10
ADAMTSL2
CYRIA
S100A6
MBP
DLGAP2
LPAR1
CACNA1E
LRRTM3
RASGEF1A
STUM
FOXO6

ADGRA1
DCHS2
PDGFA
DPP4
ADAMTSL2
CYRIA
MBP
CHSY3
LPAR1
TMEM229B
CACNA1E
RYR2
RASGEF1A
STUM
FOXO6
C2orf72
COL15A1
DPF3
DENND1C
FAM24B
CPLX3
EML6
HMX1
PLXNA4
RAB6C
CENPVL1
CENPVL3
FNDC10
AJM1
RAB6D
CLEC2L
SHISA9
CEBPA
SALL3
TMEM179
FRRS1L
SRXN1
DOC2B

SMKR1
CEBPA
TRNP1
SALL3
ZNF578
TMEM179
GOLGA8L7
SRXN1
DOC2B

DENND1B
CPLX3
UBE2QL1
HMX1
PLXNA4
RAB6D
TMEM238
CLEC2L
SHISA9
PSMB9
SMKR1
CEBPA
TRNP1
SALL3
TMEM179
GOLGA8L7
ZIM2
DOC2B
GRIN2B

GRM7
FUT4
SLC30A10
LAMB3
FAM163B
SLC6A17
ADGRA1
DCHS2
OGDHL
COL4A6
DPP4
ADAMTSL2
CYRIA
MBP
CHSY3
LPAR1
TMEM229B
CACNA1E
RYR2
BRINP2
CCDC152
RASGEF1A
FOXD6
TCEAL6
C2orf72
COL15A1
C22orf42
DENND1B
CPLX3
S1PR3
EML6
HMX1
PLXNA4
CENPVL1
FNDC10
AJM1
TMEM238
CLEC2L
SHISA9
OR2W3
PSMB9
SMKR1
TRNP1
CTSO
SALL3
TMEM179
FRRS1L
ZIM2
DOC2B
GRIN2B

MBP
CHSY3
LPAR1
TMEM229B
RYR2
LRRTM3
BRINP2
RASGEF1A
STUM
FOXD6
TCEAL6
MAFB
C2orf72
DPF3
C22orf42
DENND1B
CPLX3
EML6
UBE2QL1
HMX1
TSTD1
SP9
PLXNA4
FNDC10
AJM1
TMEM238
CLEC2L
SHISA9
SMKR1
CEBPA
SELENOP
TRNP1
SALL3
TMEM179
FRRS1L
ZIM2
SRXN1
DOC2B
GRIN2B

LYGH
DSCAML1
RIMKLA
KCNA3
FZD8
ST8SIA3
KCNJ10
ZNF354C
ALS2CL
AMER3
KCTD12
RPP25
CPNE7
TMEM151A
FIX1
SLITRK4
LACC1
AKAP5
TSHZ1
PLD5
TSPYL5
SSTR2
SHISA2
FAM83H
ZNF572
RPH3AL
CHRM2
PENK
TMEM132C
TMEM132E
GPR3
FAM181B
GALNT9
BEGAIN
GPC6
FAM43B
CALN1
CTNNA3
PCBP3
MARCHF11
EFHC2
NOG
ADRA2C
KCNJ12
PRKD1
MAP7D2
PABPC1L2B
CLDN6
TCEAL2
FMNL1
SLC24A3

TCEAL6
MAFB
DPF3
CPLX3
EML6
C10orf105
HMX1
SP9
FNDC10
AJM1
CLEC2L
SHISA9
PSMB9
SMKR1
CEBPA
TRNP1
SALL3
TMEM179
FRRS1L
GOLGA8L7
DOC2B
GRIN2B

[illegible]
GALNT17
SV2B
FAM131C
AHNAK2
C11orf87
KCNQ5
GABRA5
PDE2A
MPPED1
MAPT
VSTM2B

SHTN1
GCNT1
C11orf96
C17orf97
NHS
SMTNL2
ENO4
SLC38A3
COL25A1
CCDC9B
NKAIN2
PAQR9
VWC2
BEND4
FAM78B
INSYN2A
RELN
IL1RAPL2
MAOA
PTPRT
SPOCK3
FUT4
SLC30A10
LAMB3
FAM163B
SLC6A17
ADGRA1
DCHS2
OGDHL
PDGFA
DPP4
S100A10
H4C16
ADAMTSL2
CYRIA
S100A6
MBP
DLGAP2
CHSY3
LPAR1
TMEM229B
RYR2
LRRTM3
BRINP2
RASGEF1A
STUM
FOXO6
TCEAL6
C2orf72
COL15A1
DPF3
DENND1C
C22orf42
DENND1B
CPLX3
EML6
C10orf105
UBE2QL1
HMX1
TSTD1
SP9
PLXNA4
FNDC10
AIM1
TMEM238
CLEC2L
SHISA9
PSMB9
SMKR1
CEBPA
TRNP1
CTSO
SALL3
TMEM179
FRRS1L
GOLGA6L7
ZIM2
DOC2B
GRIN2B

TF	TF	TF	TF	TF	TF	TF	TF	TF	TF
TF:M12694	TF:M07261	TF:M04950	TF:M09636_1	TF:M02089_1	TF:M10529	TF:M03896	TF:M00189_1	TF:M00800_1	TF:M10426_1
Factor:_ZFP14;_motif:_SCNNYCCNGNNSCTSCNC	Factor:_LKLF;_motif:_GGGGTGGKSN	Factor:_Egr-1;_motif:_NGCGTGCGY	Factor:_MAZ;_motif:_GGGMGGGGSSGGGGGGGGGGG;_match_class:_1	Factor:_E2F-3;_motif:_GGCGGGN;_match_class:_1	Factor:_Sp1;_motif:_RGGGMGGRGSNGGGG	Factor:_EGR1;_motif:_NACGCCCACGCANW	Factor:_AP-2;_motif:_MKCCCSCNGGCG;_match_class:_1	Factor:_AP-2;_motif:_GSCCSCRGGCNRNRNN;_match_class:_1	Factor:_ctcf;_motif:_CCRSCAGGGGGCGCN;_match_class:_1
497	465	507	615	451	354	322	320	243	248
[illegible]	[illegible]	[illegible]	[illegible]	[illegible]	[illegible]	[illegible]	[illegible]	[illegible]	[illegible]

TF	TF	TF	TF	TF	TF	TF	TF	TF	TF
TF:M01219	TF:M09826_1	TF:M12704_1	TF:M07461_1	TF:M09973	TF:M07289	TF:M07354	TF:M12648	TF:M09826	TF:M07039_1
Factor:_SP1:SP3;_motif:_CCSCCCCCYCC	Factor:_BTEB3;_motif:_CCNNSCCNSCCCCKCCCCCC;_match_class:_1	Factor:_ZNF432;_motif:_NCAGNRCCNSRGRCAGC;_match_class:_1	Factor:_KLF;_motif:_GGGNGGGG;_match_class:_1	Factor:_CPBP;_motif:_GNNRGGGHGGGGNNGGGRN	Factor:_GKLF;_motif:_NNNRGGNGNGGSN	Factor:_Egr-1;_motif:_GCGGGGGCGG	Factor:_PATZ1;_motif:_SCNCCNCNSCCCCCASCCN	Factor:_BTEB3;_motif:_CCNNSCCNSCCCCKCCCCCC	Factor:_ETF;_motif:_CCCCGCCCCYN;_match_class:_1
349	382	262	204	501	628	390	235	527	595
[illegible]	[illegible]	[illegible]	[illegible]	[illegible]	[illegible]	[illegible]	[illegible]	[illegible]	[illegible]

SALL3
TMEM179
FRRS1L
GOLGA6L7
ZIM2
DOC2B
GRIN2B

SP9
PLXNA4
CENPVL1
CENPVL3
FNDC10
TMEM238
SHISA9
PSMB9
SMKR1
CEBPA
CTSO
SALL3
TMEM179
FRRS1L
ZIM2
DOC2B
GRIN2B

RGS6
GALNT9
BEGAIN
FAM43B
CALN1
CTNNA3
PCBP3
MARCHF11
EFHC2
NOG
OPCML
ADRA2C
KCNJ12
ACOT1
PRKD1
MAP7D2
PABPC1L2B
CLDN6
TCEAL2
FMNL1
ROBO2
SLC24A3
INPP5J
GALNT17
SV2B
FAM131C
AHNAK2
C11orf87
KCNQ5
ADAMTSL5
GABRA5
NAP1L3
CYP4X1
PDE2A
MPPED1
VSTM2B
SHTN1
GCNT1
C11orf96
C17orf97
COL4A5
NHS
SMTNL2
ENO4
SLC38A3
COL25A1
CCDC9B
NKAIN2
PAQR9
VWC2
SHISA6
BEND4
FAM78B
INSYN2A
RELN
PTPRT
SPOCK3
GRM7
CPNE4
FUT4
SLC30A10
LAMB3
SLC6A17
ADGRA1
DCHS2
OGDHL
PDGFA
COL4A6
DPP4
S100A10
H4C16
ADAMTSL2
CYRIA
S100A6
MBP
CHSY3
LPAR1
TMEM229B
CACNA1E
RYR2
LRRTM3
BRINP2
CCDC152
RASGEF1A
STUM
RIPPLY2
FOXD6
TCEAL5
TCEAL6
MAFB
C2orf72
COL15A1
DPF3
DENND1C
DENND1B
FAM24B
CPLX3
S1PR3
EML6
C10orf105
UBE2QL1
HMX1
TSTD1
SP9
PLXNA4
CENPVL1
CENPVL3
FNDC10
AJM1
TMEM238
CLEC2L
SHISA9
PSMB9
SMKR1
CEBPA
TRNP1
SALL3
ZNF578
TMEM179
FRRS1L
GOLGA6L7
ZIM2
SRXN1
DOC2B
GRIN2B

CEBPA
TRNP1
SALL3
ZNF578
TMEM179
FRRS1L
GOLGA6L7
ZIM2
SRXN1
DOC2B
GRIN2B

TSHZ1
PLD5
TSPYL5
GSX2
SSTR2
SHISA2
FAM83H
ZNF572
RPH3AL
CHRM2
PENK
TMEM132C
TMEM132E
DDX60L
GPR88
GPR3
ADGRB1
FAM181B
KCNK4
RGS6
GALNT9
BEGAIN
FAM43B
CALN1
CTNNA3
PCBP3
MARCHF11
EFHC2
NOG
OPCML
ADRA2C
KCNJ12
PRKD1
PABPC1L2B
CLDN6
TCEAL2
FMNL1
ROBO2
SLC24A3
INPP5J
GALNT17
SV2B
FAM131C
AHNAK2
C11orf87
KCNQ5
ADAMTSL5
GABRA5
NAP1L3
CYP4X1
PDE2A
MPPED1
VSTM2B
SHTN1
C11orf96
C17orf97
COL4A5
NHS
SMTNL2
ENO4
SLC38A3
COL25A1
CCDC9B
NKAIN2
PAQR9
VWC2
SHISA6
FAM78B
INSYN2A
RELN
IL1RAPL2
MAOA
PTPRT
SPOCK3
GRM7
CPNE4
FUT4
SLC30A10
LAMB3
FAM163B
SLC6A17
ADGRA1
DCHS2
OGDHL
PDGFA
DPP4
S100A10
ADAMTSL2
CYRIA
S100A6
MBP
CHSY3
LPAR1
TMEM229B
CACNA1E
RYR2
LRRTM3
CCDC152
RASGEF1A
STUM
RIPPLY2
FOXD6
TCEAL5
TCEAL6
MAFB
C2orf72
COL15A1
DPF3
DENND1C
C22orf42
DENND1B
CPLX3
S1PR3
EML6
C10orf105
UBE2QL1
HMX1
TSTD1
SP9
PLXNA4
CENPVL1
FNDC10
AJM1
CLEC2L
SHISA9
PSMB9
SMKR1
CEBPA
TRNP1
SALL3
ZNF578
TMEM179
FRRS1L
GOLGA6L7
ZIM2
SRXN1
DOC2B
GRIN2B

FOXD6
TCEAL5
TCEAL6
MAFB
C2orf72
DPF3
DENND1C
C22orf42
DENND1B
FAM24B
CPLX3
EML6
C10orf105
UBE2QL1
HMX1
TSTD1
SP9
PLXNA4
CENPVL1
CENPVL3
FNDC10
AJM1
TMEM238
CLEC2L
SHISA9
SMKR1
CEBPA
TRNP1
SALL3
ZNF578
TMEM179
FRRS1L
GOLGA6L7
ZIM2
SRXN1
DOC2B
GRIN2B

CLDN6
TCEAL2
FMNL1
ROBO2
SLC24A3
INPP5J
GALNT17
SV2B
FAM131C
AHNAK2
C11orf87
KCNQ5
ADAMTSL5
GABRA5
NAP1L3
CYP4X1
PDE2A
MPPED1
VSTM2B
SHTN1
GCNT1
C11orf96
C17orf97
COL4A5
NHS
SMTNL2
ENO4
SLC38A3
COL25A1
CCDC9B
NKAIN2
PAQR9
VWC2
SHISA6
BEND4
FAM78B
INSYN2A
RELN
IL1RAPL2
MAOA
PTPRT
SPOCK3
GRM7
CPNE4
FUT4
LAMB3
FAM163B
SLC6A17
ADGRA1
DCHS2
OGDHL
PDGFA
DPP4
S100A10
H4C16
ADAMTSL2
CYRIA
S100A6
MBP
CHSY3
LPAR1
TMEM229B
CACNA1E
RYR2
LRRTM3
RASGEF1A
STUM
RIPPLY2
FOXD6
TCEAL5
TCEAL6
MAFB
C2orf72
DPF3
DENND1C
C22orf42
DENND1B
FAM24B
CPLX3
EML6
C10orf105
HMX1
TSTD1
SP9
PLXNA4
CENPVL1
FNDC10
AJM1
TMEM238
CLEC2L
SHISA9
PSMB9
SMKR1
CEBPA
TRNP1
CTSO
SALL3
ZNF578
TMEM179
FRRS1L
GOLGA6L7
ZIM2
SRXN1
DOC2B
GRIN2B

TF<br>TF:M00143<br>Factor:_Pax-5;_motif:_BCNNNRNGCANBGNTGNRTAGCSGCHNB<br>516	TF<br>TF:M09898<br>Factor:_Egr-2;_motif:_GNGRRNGWGKGGGNGGRG<br>333	TF<br>TF:M00695<br>Factor:_ETF;_motif:_GVGGMGG<br>484	TF<br>TF:M12351_1<br>Factor:_TIEG1;_motif:_NCCCNSNCCCCGCCCCC;_match_class:_1<br>401	TF<br>TF:M12160<br>Factor:_KLF15;_motif:_RCCMCRCCCMCN<br>564	TF<br>TF:M08878_1<br>Factor:_EGR;_motif:_CGCCCCCGCNN;_match_cl[illegible]
HS3ST1	HS3ST1	HS3ST1	HS3ST1	HS3ST1	PLXND1
CACNG3	PLXND1	PLXND1	PLXND1	PLXND1	YBX2
TAC1	YBX2	ITGA3	ITGA3	ITGA3	GAS7
ETV1	CEACAM21	YBX2	YBX2	YBX2	CYB561
CEACAM21	CYB561	CACNG3	CACNG3	ETV1	SLC6A7
GAS7	NFIX	PNPLA4	GAS7	CEACAM21	JADE2
MATK	SLC6A7	GAS7	MATK	GAS7	XK
CYB561	PLEKHB1	MATK	CYB561	MATK	GYG2
NFIX	CDH10	PAX6	NFIX	CYB561	CAMK2B
SLC6A7	TG	CYB561	SYT7	NFIX	SLC9A3R2
SYT7	JADE2	SYT7	SLC7A14	SLC6A7	TLE2
SLC7A14	CCDC85A	CNTN1	PLEKHB1	SYT7	PDE4A
CNTN1	F7	MRI1	MRI1	SLC7A14	NGEF
MYO16	CAMK2B	RAB27B	TNC	CNTN1	MPPED2
TNC	PTPRU	TNC	JADE2	PLEKHB1	KIF26A
CAPG	RIMBP2	JADE2	PTPRN	CDH10	ATP2B3
FSTL4	SLC9A3R2	XK	CCDC85A	TNC	ACSL4
PTPRN	TLE2	FSTL4	GYG2	CAPG	RASGRP2
CCDC85A	MPPED2	PTPRN	F7	JADE2	PPP2R2C
GYG2	KIF26A	CCDC85A	RASGRF1	XK	MGLL
RASGRF1	ATP2B3	GYG2	CAMK2B	PTPRN	NUAK1
CAMK2B	ACSL4	RASGRF1	DGKG	CCDC85A	NEBL
DGKG	RASGRP2	CAMK2B	RIMBP2	GYG2	CARMIL1
PTPRU	TRPC7	DGKG	SLC9A3R2	F7	MID2
RIMBP2	SPTB	PTPRU	TLE2	RASGRF1	STK17B
LZTS1	CAMK2A	SLC9A3R2	PDE4A	CAMK2B	TTC39A
SLC9A3R2	TRPC5	TLE2	NGEF	DGKG	ACHE
NGEF	TRHDE	PDE4A	MPPED2	PTPRU	PITPNM2
MPPED2	PPP2R2C	NGEF	KIF26A	RIMBP2	SEL1L3
KIF26A	NUAK1	MPPED2	ATP2B3	LZTS1	COL9A3
ATP2B3	NEBL	KIF26A	ACSL4	SLC9A3R2	CRTAC1
RASGRP2	ADCY2	ATP2B3	RASGRP2	TLE2	HCN2
TRPC7	CARMIL1	ACSL4	SPTB	PDE4A	SEZ6L
SPTB	STK17B	RASGRP2	TRPC5	NGEF	PDGFB
CNGB1	P2RX5	SPTB	TRHDE	MPPED2	CHGA
CAMK2A	COL16A1	TRPC5	PPP2R2C	KIF26A	NTSR1
TRPC5	WNT11	TRHDE	MGLL	ATP2B3	RSPO4
PPP2R2C	TTC39A	PPP2R2C	NUAK1	ACSL4	PPP1R16B
MGLL	ACHE	SNCB	FOSL2	RASGRP2	ST8SIA5
NUAK1	NOS1	MGLL	NEBL	TRPC7	MXRA5
WSCD2	SEL1L3	NUAK1	ADCY2	SPTB	STS
SEMA3A	COL9A3	WSCD2	BRINP1	CNGB1	SYT17
SEMA3C	HCN2	SEMA3C	CARMIL1	CAMK2A	SLC17A7
FOSL2	SEZ6L	FOSL2	MID2	TRPC5	AMH
CACNG5	RASD2	CACNG5	STK17B	TRHDE	NEURL1
NEBL	CACNA1I	NEBL	P2RX5	PPP2R2C	SORCS1
ADCY2	CHGA	ADCY2	COL16A1	SNCB	FAM149A
CARMIL1	DOK5	BRINP1	TTC39A	MGLL	CCKBR
DCT	PPP1R16B	CARMIL1	ACHE	NUAK1	PTPN5
MID2	ST8SIA5	MID2	PITPNM2	CACNG5	EYA4
CDH7	KLF8	CDH7	SEL1L3	DCX	EFHD1
STK17B	NALCN	STK17B	COL9A3	NEBL	SLC9A2
[illegible]	[illegible]	[illegible]	[illegible]	[illegible]	[illegible]

[illegible]

S1PR3
HMX1
TSTD1
PLXNA4
CENPVL1
CENPVL3
FNDC10
AJM1
TMEM238
CLEC2L
SHISA9
OR2W3
PSMB9
SMKR1
CEBPA
SELENOP
TRNP1
CTSO
SALL3
ZNF578
TMEM179
GOLGA6L7
ZIM2
SRXN1
DOC2B
GRIN2B

COL4A5
NHS
ENO4
SLC38A3
COL25A1
CCDC9B
NKAIN2
PAQR9
VWC2
BEND4
FAM78B
INSYN2A
RELN
IL1RAPL2
MAOA
PTPRT
SPOCK3
GRM7
CPNE4
SLC30A10
SLC6A17
ADGRA1
DCHS2
OGDHL
PDGFA
DPP4
S100A10
H4C16
CYRIA
S100A6
MBP
CHSY3
LPAR1
TMEM229B
RYR2
LRRTM3
RASGEF1A
STUM
FOXO6
TCEAL5
TCEAL6
MAFB
C2orf72
COL15A1
DPF3
DENND1C
DENND1B
CPLX3
S1PR3
EML6
C10orf105
UBE2QL1
HMX1
TSTD1
SP9
PLXNA4
CENPVL1
FNDC10
AJM1
TMEM238
SHISA9
OR2W3
PSMB9
SMKR1
TRNP1
CTSO
SALL3
TMEM179
FRRS1L
GOLGA6L7
ZIM2
SRXN1
DOC2B
GRIN2B

IL1RAPL2
PTPRT
SPOCK3
GRM7
FUT4
SLC30A10
FAM163B
SLC6A17
ADGRA1
DCHS2
OGDHL
PDGFA
DPP4
ADAMTSL2
CYRIA
S100A6
MBP
CHSY3
LPAR1
TMEM229B
CACNA1E
RYR2
BRINP2
RASGEF1A
STUM
RIPPLY2
FOXO6
TCEAL6
MAFB
C2orf72
COL15A1
DPF3
DENND1B
FAM24B
CPLX3
EML6
UBE2QL1
HMX1
SP9
PLXNA4
RAB6C
CENPVL1
CENPVL3
FNDC10
RAB6D
TMEM238
CLEC2L
SHISA9
PSMB9
SMKR1
CEBPA
TRNP1
CTSO
SALL3
ZNF578
TMEM179
FRRS1L
ZIM2
SRXN1
DOC2B
GRIN2B

SLC24A3
INPP5J
GALNT17
SV2B
FAM131C
AHNAK2
C11orf87
KCNQ5
ADAMTSL5
GABRA5
NAP1L3
CYP4X1
PDE2A
MPPED1
VSTM2B
SHTN1
GCNT1
C11orf96
NHS
SMTNL2
SLC38A3
COL25A1
CCDC9B
NKAIN2
PAQR9
VWC2
SHISA6
BEND4
FAM78B
INSYN2A
RELN
IL1RAPL2
PTPRT
SPOCK3
GRM7
FUT4
LAMB3
FAM163B
SLC6A17
ADGRA1
DCHS2
OGDHL
PDGFA
DPP4
S100A10
H4C16
ADAMTSL2
CYRIA
S100A6
MBP
DLGAP2
CHSY3
LPAR1
TMEM229B
CACNA1E
RYR2
BRINP2
CCDC152
RASGEF1A
STUM
RIPPLY2
FOXO6
TCEAL5
TCEAL6
MAFB
C2orf72
COL15A1
DPF3
DENND1C
DENND1B
FAM24B
CPLX3
EML6
C10orf105
UBE2QL1
HMX1
SP9
PLXNA4
RAB6C
CENPVL1
CENPVL3
FNDC10
AJM1
TMEM238
CLEC2L
SHISA9
OR2W3
PSMB9
SMKR1
CEBPA
SELENOP
TRNP1
CTSO
SALL3
ZNF578
TMEM179
FRRS1L
GOLGA6L7
ZIM2
SRXN1
DOC2B
GRIN2B

TMEM132E
DDX60L
GPR88
GPR3
ADGRB1
FAM181B
KCNK4
RGS6
GALNT9
BEGAIN
GPC6
FAM43B
CALN1
CTNNA3
PCBP3
MARCHF11
EFHC2
NOG
OPCML
ADRA2C
KCNJ12
ACOT1
PRKD1
MAP7D2
PABPC1L2B
CLDN6
TCEAL2
FMNL1
ROBO2
SLC24A3
GALNT17
SV2B
FAM131C
AHNAK2
C11orf87
KCNQ5
ADAMTSL5
GABRA5
NAP1L3
CYP4X1
PDE2A
MPPED1
VSTM2B
SHTN1
GCNT1
C11orf96
C17orf97
COL4A5
NHS
SMTNL2
ENO4
SLC38A3
COL25A1
NKAIN2
PAQR9
VWC2
SHISA6
BEND4
FAM78B
INSYN2A
RELN
IL1RAPL2
MAOA
PTPRT
SPOCK3
GRM7
FUT4
SLC30A10
LAMB3
FAM163B
SLC6A17
ADGRA1
DCHS2
OGDHL
PDGFA
DPP4
H4C16
ADAMTSL2
CYRIA
S100A6
MBP
CHSY3
LPAR1
TMEM229B
CACNA1E
RYR2
LRRTM3
BRINP2
RASGEF1A
STUM
RIPPLY2
FOXO6
TCEAL6
MAFB
C2orf72
COL15A1
DPF3
DENND1C
C22orf42
DENND1B
FAM24B
CPLX3
EML6
UBE2QL1
HMX1
TSTD1
SP9
PLXNA4
RAB6C
CENPVL1
CENPVL3
FNDC10
AJM1
RAB6D
TMEM238
CLEC2L
SHISA9
PSMB9
SMKR1
CEBPA
SELENOP
TRNP1
CTSO
SALL3
TMEM179
FRRS1L
GOLGA6L7
ZIM2
SRXN1
DOC2B
GRIN2B

TF	TF	TF	TF	TF	TF	TF	TF	TF	TF
TF:M10026_1	TF:M12227	TF:M09734_1	TF:M01858	TF:M02089	TF:M12508	TF:M08819_1	TF:M06948_1	TF:M07461	TF:M00189
Factor:_PAT2;_motif:_GGGGNGGGGGMKGGRRNGGNRN;_match_class:_1	Factor:_ZIC4;_motif:_NNCCNCCCRYNGYGN	Factor:_ZNF692;_motif:_SYNGGSCCCASCCNC;_match_class:_1	Factor:_AP-2beta;_motif:_GCNNNGGSCNGVGGGN	Factor:_E2F-3;_motif:_GGCGGGN	Factor:_EGR1;_motif:_NNMCGCCCACGCNN	Factor:_LKLF;_motif:_CNCCACCCS;_match_class:_1	Factor:_Sp2;_motif:_TGGGCGCGCCCA;_match_class:_1	Factor:_KLF;_motif:_GGGNGGGG	Factor:_AP-2;_motif:_MKCCCSCNGGCG
265	503	321	366	577	326	111	316	417	486
PLXND1	HS3ST1	PLXND1	PLXND1	HS3ST1	HS3ST1	PLXND1	PLXND1	PLXND1	HS3ST1
YBX2	PLXND1	ITGA3	ITGA3	PLXND1	PLXND1	MATK	ITGA3	ETV1	PLXND1
GAS7	TAC1	CEACAM21	YBX2	ITGA3	YBX2	SYT7	YBX2	PNPLA4	ITGA3
MATK	PNPLA4	GAS7	GAS7	YBX2	CYB561	CNTN1	CACNG3	CEACAM21	YBX2
CYB561	GAS7	MATK	CYB561	CACNG3	NFIX	JADE2	GAS7	MATK	GAS7
NFIX	MATK	CYB561	SYT7	TAC1	SLC6A7	XK	MATK	PAX6	MATK
SLC6A7	PAX6	SYT7	CNTN1	PNPLA4	TNC	CCDC85A	NFIX	NFIX	PAX6
JADE2	CAMK1G	SLC7A14	PLEKHB1	CEACAM21	JADE2	F7	SLC6A7	SLC6A7	CYB561
PTPRN	CYB561	PLEKHB1	JADE2	GAS7	XK	RIMBP2	SYT7	SYT7	SLC6A7
CAMK2B	NFIX	CAPG	XK	MATK	CCDC85A	TRPC5	CAPG	SLC7A14	SYT7
RIMBP2	SLC6A7	TG	FSTL4	PAX6	GYG2	PPP2R2C	JADE2	CNTN1	PLEKHB1
SLC9A3R2	SYT7	JADE2	PTPRN	CYB561	CAMK2B	TTC39A	CCDC85A	PLEKHB1	CAPG
TLE2	PLEKHB1	XK	GYG2	NFIX	DGKG	CRTAC1	F7	CDH10	JADE2
NGEF	RAB27B	PTPRN	RASGRF1	SLC6A7	LZTS1	MMP11	PTPRU	CAPG	FSTL4
MPPED2	TNC	CCDC85A	CAMK2B	SYT7	SLC9A3R2	MMP9	RIMBP2	TG	PTPRN
KIF26A	JADE2	GYG2	PTPRU	SLC7A14	TLE2	CELF4	SLC9A3R2	JADE2	CCDC85A
ATP2B3	XK	F7	RIMBP2	CNTN1	PDE4A	MXRA5	TLE2	XK	GYG2
RASGRP2	FSTL4	CAMK2B	SLC9A3R2	PLEKHB1	NGEF	KLF8	PDE4A	PTPRN	RASGRF1
TRPC5	PTPRN	RIMBP2	TLE2	MRI1	MPPED2	NALCN	NGEF	CCDC85A	CAMK2B
TRHDE	CCDC85A	SLC9A3R2	NGEF	CDH10	KIF26A	SYT17	KIF26A	F7	DGKG
ALPK1	GYG2	TLE2	MPPED2	RAB27B	ATP2B3	TUBB4A	ATP2B3	CAMK2B	PTPRU
PPP2R2C	F7	NGEF	KIF26A	TNC	ACSL4	SLC17A7	RASGRP2	DGKG	RIMBP2
SNCB	RASGRF1	MPPED2	RASGRP2	CAPG	RASGRP2	AMH	TRHDE	RIMBP2	LZTS1
NUAK1	CAMK2B	ATP2B3	CNGB1	TG	SPTB	CDH23	SNCB	SLC9A3R2	SLC9A3R2
FOSL2	RIMBP2	ACSL4	CAMK2A	JADE2	TRPC5	SFXN3	MGLL	TLE2	TLE2
CACNG5	SLC9A3R2	RASGRP2	TRPC5	XK	PPP2R2C	CCKBR	NUAK1	PDE4A	NGEF
NEBL	PDE4A	SPTB	TRHDE	FSTL4	MGLL	IGFBP5	WSCD2	MPPED2	MPPED2
ADCY2	NGEF	CAMK2A	PPP2R2C	PTPRN	NUAK1	PPL	FOSL2	ATP2B3	KIF26A
CARMIL1	MPPED2	TRPC5	SNCB	CCDC85A	SEMA3C	HPCA	NEBL	ACSL4	ATP2B3
[illegible]	[illegible]	[illegible]	[illegible]	[illegible]	[illegible]	[illegible]	[illegible]	[illegible]	[illegible]

TF	TF	TF	TF	TF	TF	TF	TF	TF	TF
TF:M07436_1	TF:M01073	TF:M03896_1	TF:M10108	TF:M09973_1	TF:M07261_1	TF:M12725	TF:M00873_1	TF:M12593	TF:M07039
Factor:_WT1;_motif:_NNGGGNGGGSGN;_match_class:_1	Factor:_HIC1;_motif:_NNNGGKTGCCCSNNNNNN	Factor:_EGR1;_motif:_NACGCCCACGCANW;_match_class:_1	Factor:_WT1;_motif:_RGGNGGGGGAGGRGGNGGRG	Factor:_CPBP;_motif:_GNNRGGGHGGGGNNGGGRN;_match_class:_1	Factor:_LKLF;_motif:_GGGGTGGKSN;_match_class:_1	Factor:_PHB;_motif:_NCCCAGCCCCY	Factor:_Egr-1;_motif:_GCGGGGGCGG;_match_class:_1	Factor:_CTCF;_motif:_NNRSYGCCMCCTGSTGGCCN	Factor:_ETF;_motif:_CCCCGCCCCYN
163	228	138	329	333	227	451	163	409	674
MATK	PLXND1	PLXND1	HS3ST1	PLXND1	PLXND1	HS3ST1	PLXND1	HS3ST1	HS3ST1
NFIX	ITGA3	CYB561	PLXND1	ITGA3	PNPLA4	PLXND1	YBX2	PLXND1	PLXND1
SLC7A14	TAC1	SLC6A7	YBX2	YBX2	MATK	ITGA3	GAS7	ITGA3	ITGA3
JADE2	GAS7	JADE2	ETV1	CACNG3	SLC6A7	YBX2	CYB561	YBX2	YBX2
RASGRF1	MATK	CCDC85A	MATK	ETV1	SYT7	TAC1	SLC6A7	GAS7	CACNG3
SLC9A3R2	PAX6	GYG2	CYB561	CEACAM21	CNTN1	ETV1	JADE2	MATK	TAC1
TLE2	SLC6A7	RASGRF1	NFIX	GAS7	PLEKHB1	CEACAM21	CAMK2B	NFIX	ETV1
MPPED2	SLC7A14	CAMK2B	PLEKHB1	MATK	JADE2	GAS7	SLC9A3R2	SLC6A7	PNPLA4
ATP2B3	JADE2	SLC9A3R2	MRI1	CYB561	XK	MATK	TLE2	SYT7	CEACAM21
RASGRP2	CCDC85A	TLE2	CDH10	NFIX	PTPRN	CYB561	NGEF	CAPG	GAS7
SNCB	GYG2	NGEF	JADE2	SLC6A7	CCDC85A	NFIX	MPPED2	JADE2	MATK
NEBL	RASGRF1	MPPED2	PTPRN	SYT7	GYG2	SLC6A7	KIF26A	XK	PAX6
ADCY2	CAMK2B	ATP2B3	GYG2	SLC7A14	F7	SYT7	ACSL4	FSTL4	CAMK1G
COL16A1	DGKG	ACSL4	CAMK2B	CNTN1	RASGRF1	SLC7A14	RASGRP2	PTPRN	CYB561
TTC39A	TLE2	RASGRP2	RIMBP2	PLEKHB1	CAMK2B	CNTN1	PPP2R2C	CCDC85A	NFIX
ACHE	NGEF	SPTB	SLC9A3R2	CAPG	RIMBP2	RAB27B	MGLL	GYG2	SLC6A7
SEL1L3	MPPED2	PPP2R2C	NGEF	TG	LZTS1	CAPG	NUAK1	RASGRF1	SYT7
HCN2	ATP2B3	MGLL	MPPED2	JADE2	SLC9A3R2	TG	FOSL2	CAMK2B	SLC7A14
SEZ6L	ACSL4	NUAK1	KIF26A	XK	TLE2	JADE2	ADCY2	DGKG	CNTN1
CHGA	RASGRP2	FOSL2	ATP2B3	CCDC85A	MPPED2	XK	MID2	PTPRU	PLEKHB1
MMP9	PPP2R2C	ADCY2	RASGRP2	GYG2	ACSL4	PTPRN	TTC39A	SLC9A3R2	MRI1
MAP1LC3A	WSCD2	MID2	SPTB	F7	SPTB	CCDC85A	ACHE	TLE2	CDH10
SYT17	SEMA3C	TTC39A	TRPC5	CAMK2B	TRPC5	GYG2	PITPNM2	PDE4A	TNC
TUBB4A	FOSL2	ACHE	TRHDE	RIMBP2	PPP2R2C	F7	SEL1L3	NGEF	CAPG
SLC17A7	CACNG5	PITPNM2	PPP2R2C	SLC9A3R2	SNCB	RASGRF1	COL9A3	MPPED2	TG
AMH	NEBL	SEL1L3	NUAK1	TLE2	MGLL	CAMK2B	CRTAC1	KIF26A	JADE2
LHX2	ADCY2	COL9A3	FOSL2	NGEF	ADCY2	RIMBP2	HCN2	ATP2B3	XK
NEURL1	CDH7	HCN2	CACNG5	MPPED2	COL16A1	SLC9A3R2	SEZ6L	RASGRP2	LMO3
EYA4	ACHE	ISM2	NEBL	KIF26A	TTC39A	TLE2	PDGFB	TRPC7	FSTL4
[illegible]	[illegible]	[illegible]	[illegible]	[illegible]	[illegible]	[illegible]	[illegible]	[illegible]	[illegible]

PSMB9
SMKR1
CEBPA
TRNP1
SALL3
ZNF578
TMEM179
FRRS1L
GOLGA6L7
ZIM2
SRXN1
DOC2B
GRIN2B

AHNAK2
C11orf87
KCNQ5
ADAMTSL5
GABRA5
CYP4X1
PDE2A
MPPED1
MAPT
VSTM2B
SHTN1
GCNT1
C11orf96
C17orf97
NHS
SMTNL2
ENO4
SLC38A3
COL25A1
NKAIN2
PAQR9
VWC2
SHISA6
BEND4
FAM78B
INSYN2A
RELN
IL1RAPL2
MAOA
PTPRT
SPOCK3
FUT4
SLC30A10
LAMB3
FAM163B
SLC6A17
ADGRA1
DCHS2
OGDHL
PDGFA
COL4A6
H4C16
ADAMTSL2
CYRIA
S100A6
MBP
DLGAP2
CHSY3
LPAR1
TMEM229B
CACNA1E
RYR2
RASGEF1A
STUM
FOXO6
TCEAL5
TCEAL6
MAFB
C2orf72
DPF3
DENND1C
DENND1B
FAM24B
CPLX3
S1PR3
EML6
UBE2QL1
HMX1
TSTD1
FNDC10
AJM1
TMEM238
CLEC2L
SHISA9
PSMB9
SMKR1
CEBPA
TRNP1
SALL3
ZNF578
TMEM179
FRRS1L
GOLGA6L7
ZIM2
SRXN1
DOC2B
GRIN2B

VWC2L
ZCCHC12
CNTNAP2
GALNTL6
TTC9B
SLCO2A1
FUT1
CHST2
NPPA
GAL3ST3
PCSK1
CREG2
ARL4D
LY6H
DSCAML1
RIMKLA
KCNA3
FZD8
ST8SIA3
KCNJ10
ZNF354C
ALS2CL
AMER3
KCTD12
RPP25
CPNE7
FAM133A
ALOXE3
TMEM151A
FIS1
SLITRK4
LACC1
AKAP5
ZNF154
TSHZ1
LYNX1
PLD5
TSPYL5
GSX2
SSTR2
SHISA2
FAM83H
ZNF572
RPH3AL
CHRM2
TMEM132C
TMEM132E
DDX60L
GPR88
GPR3
ADGRB1
FAM181B
KCNK4
RGS6
GALNT9
BEGAIN
FAM43B
CALN1
CTNNA3
PCBP3
MARCHF11
EFHC2
NOG
OPCML
ADRA2C
KCNJ12
ACOT1
PRKD1
MAP7D2
PABPC1L2B
CLDN6
TCEAL2
FMNL1
ROBO2
SLC24A3
INPP5J
GALNT17
SV2B
FAM131C
AHNAK2
C11orf87
KCNQ5
ADAMTSL5
GABRA5
NAP1L3
CYP4X1
PDE2A
MPPED1
MAPT
VSTM2B
SHTN1
GCNT1
C11orf96
C17orf97
COL4A5
NHS
SMTNL2
ENO4
SLC38A3
COL25A1
CCDC9B
NKAIN2
PAQR9
VWC2
SHISA6
BEND4
FAM78B
INSYN2A
RELN
IL1RAPL2
MAOA
PTPRT
SPOCK3
GRM7
CPNE4
FUT4
LAMB3
FAM163B
SLC6A17
ADGRA1
DCHS2
OGDHL
PDGFA
COL4A6
DPP4
S100A10
H4C16
ADAMTSL2
CYRIA
S100A6
MBP
DLGAP2
CHSY3
LPAR1
TMEM229B
CACNA1E
RYR2
LRRTM3
BRINP2
RASGEF1A
STUM
RIPPLY2
FOXO6
TCEAL5
TCEAL6
MAFB
C2orf72
DPF3
DENND1C
C22orf42
DENND1B
FAM24B
CPLX3
S1PR3
EML6
C10orf105
UBE2QL1
HMX1
TSTD1
SP9
PLXNA4
CENPVL1
CENPVL3
FNDC10
AJM1
RAB6D
TMEM238
CLEC2L
SHISA9
PSMB9
SMKR1
CEBPA
SELENOP
TRNP1
CTSO
SALL3
ZNF578
TMEM179
FRRS1L
GOLGA6L7
ZIM2
SRXN1
DOC2B
GRIN2B

TF	TF	TF	TF	TF	TF	TF	TF	TF	TF
TF:M07040	TF:M05327	TF:M07486_1	TF:M07206_1	TF:M12489	TF:M12351	TF:M00143_1	TF:M00469	TF:M12636_1	TF:M01240_1
Factor:_GKLF;_motif:_NNRRGRRNGNSNNN	Factor:_WT1;_motif:_NGCGGGGGGGTSMMCYN	Factor:_ZNF300;_motif:_SWGGGGGSN;_match_class:_1	Factor:_E2F-1;_motif:_NGGGCGGGARV;_match_class:_1	Factor:_KLF4;_motif:_NNCCCCACCCNN	Factor:_TIEG1;_motif:_NCCCNSNCCCCGCCCCC	Factor:_Pax-5;_motif:_BCNNNRNGCANBGNTGNRTAGCSGCHNR;_match_class:_1	Factor:_AP-2alpha;_motif:_GCCNNNRGS	Factor:_MLLT10;_motif:_CYNNCCNNGGNGCTG;_match_class:_1	Factor:_BEN;_motif:_CAGCGRNV;_match_class:_1
555	286	359	491	306	543	263	268	197	572
HS3ST1	PLXND1	HS3ST1	HS3ST1	PLXND1	HS3ST1	HS3ST1	HS3ST1	YBX2	HS3ST1
ITGA3	YBX2	PLXND1	PLXND1	ETV1	PLXND1	CACNG3	PLXND1	CACNG3	PLXND1
YBX2	GAS7	ITGA3	ITGA3	CEACAM21	ITGA3	CYB561	ITGA3	SYT7	ITGA3
CACNG3	MATK	YBX2	YBX2	MATK	YBX2	SYT7	YBX2	MRI1	YBX2
TAC1	CYB561	TAC1	PNPLA4	SLC6A7	CACNG3	CCDC85A	GAS7	RAB27B	CACNG3
ETV1	SLC6A7	CEACAM21	GAS7	SYT7	TAC1	GYG2	MATK	TNC	TAC1
PNPLA4	SYT7	GAS7	MATK	SLC7A14	ETV1	RASGRF1	PAX6	PTPRN	ETV1
CEACAM21	SLC7A14	MATK	PAX6	CNTN1	GAS7	CAMK2B	CYB561	KIF26A	GAS7
MATK	MRI1	CYB561	CYB561	PLEKHB1	MATK	DGKG	SYT7	ATP2B3	MATK
PAX6	CDH10	SLC6A7	NFIX	CDH10	PAX6	PTPRU	CNTN1	SPTB	PAX6
CAMK1G	JADE2	SYT7	SLC6A7	TG	CYB561	LZTS1	PLEKHB1	TRPC5	CAMK1G
CYB561	FSTL4	SLC7A14	SYT7	JADE2	NFIX	SLC9A3R2	JADE2	SNCB	SLC6A7
NFIX	GYG2	PLEKHB1	SLC7A14	XK	SLC6A7	NGEF	PTPRN	NUAK1	SYT7
SLC6A7	RASGRF1	JADE2	CNTN1	PTPRN	SYT7	MPPED2	CCDC85A	WSCD2	SLC7A14
SYT7	CAMK2B	XK	PLEKHB1	CCDC85A	SLC7A14	KIF26A	CAMK2B	CACNG5	PLEKHB1
SLC7A14	DGKG	GYG2	TNC	F7	PLEKHB1	RASGRP2	DGKG	NEBL	MRI1
CNTN1	PTPRU	F7	JADE2	CAMK2B	MRI1	CNGB1	RIMBP2	ADCY2	RAB27B
PLEKHB1	SLC9A3R2	RASGRF1	XK	SLC9A3R2	CDH10	CAMK2A	LZTS1	P2RX5	TNC
MRI1	TLE2	RIMBP2	FSTL4	TLE2	RAB27B	PPP2R2C	TLE2	COL16A1	CAPG
CDH10	NGEF	LZTS1	PTPRN	MPPED2	TNC	MGLL	NGEF	TTC39A	JADE2
CAPG	KIF26A	SLC9A3R2	CCDC85A	ATP2B3	CAPG	SEMA3C	MPPED2	ACHE	LMO3
JADE2	ATP2B3	TLE2	GYG2	ACSL4	TG	ADCY2	KIF26A	RPH3A	FSTL4
LMO3	ACSL4	PDE4A	CAMK2B	RASGRP2	JADE2	CARMIL1	ATP2B3	NOS1	PTPRN
FSTL4	RASGRP2	MPPED2	DGKG	TRPC7	PTPRN	DCT	SPTB	PITPNM2	CCDC85A
PTPRN	TRHDE	KIF26A	PTPRU	SPTB	CCDC85A	CDH7	CAMK2A	CRTAC1	GYG2
[illegible]	[illegible]	[illegible]	[illegible]	[illegible]	[illegible]	[illegible]	[illegible]	[illegible]	[illegible]

TF	TF	TF	TF	TF	TF	TF	TF	TF	TF
TF:M10108_1	TF:M09734	TF:M12313	TF:M00649_1	TF:M12461_1	TF:M07249	TF:M12354	TF:M12461	TF:M10008	TF:M12227_1
Factor:_WT1;_motif:_RGGNGGGGGAGGRGGNGGRG;_match_class:_1	Factor:_ZNF692;_motif:_SYNGGSCCCASCCNC	Factor:_ZNF460;_motif:_NNACNCCCCCNN	Factor:_MAZ;_motif:_GGGGAGGG;_match_class:_1	Factor:_ZBTB14;_motif:_SSCCGCGCACNS;_match_class:_1	Factor:_ctcf;_motif:_CCNCNAGRKGGCRSTN	Factor:_ZNF37A;_motif:_CCYYGGCTCCNTSCCMN	Factor:_ZBTB14;_motif:_SSCCGCGCACNS	Factor:_CSX;_motif:_NKRAGWGS	Factor:_ZIC4;_motif:_NNCCNCCCRYNGYGN;_match_class:_1
168	519	296	225	49	330	490	168	461	298
HS3ST1	PLXND1	HS3ST1	YBX2	MATK	HS3ST1	PLXND1	PLXND1	HS3ST1	HS3ST1
PLXND1	ITGA3	PLXND1	CEACAM21	FSTL4	PLXND1	ABCB5	ITGA3	PLXND1	PLXND1
YBX2	YBX2	ITGA3	NFIX	TLE2	YBX2	ITGA3	GAS7	ITGA3	GAS7
MATK	ETV1	YBX2	SLC6A7	KIF26A	PNPLA4	YBX2	MATK	YBX2	MATK
CYB561	PNPLA4	GAS7	SYT7	ATP2B3	GAS7	CACNG3	CYB561	ETV1	CYB561
NFIX	CEACAM21	NFIX	PLEKHB1	MGLL	MATK	PNPLA4	SLC6A7	PNPLA4	SYT7
JADE2	GAS7	SLC7A14	CDH10	FOSL2	CYB561	GAS7	SYT7	CEACAM21	PLEKHB1
RIMBP2	MATK	CAPG	PTPRN	CDH7	SLC6A7	MATK	SLC7A14	GAS7	JADE2
SLC9A3R2	PAX6	JADE2	GYG2	TTC39A	SYT7	PAX6	CAPG	PAX6	PTPRN
NGEF	CYB561	LMO3	RASGRF1	ISM2	SLC7A14	CYB561	JADE2	CYB561	GYG2
MPPED2	NFIX	FSTL4	SLC9A3R2	STS	CAPG	NFIX	FSTL4	NFIX	RASGRF1
ATP2B3	SYT7	PTPRN	TLE2	SYT17	TG	SLC6A7	GYG2	SYT7	PDE4A
RASGRP2	SLC7A14	CCDC85A	PDE4A	NEURL1	JADE2	SYT7	RIMBP2	SLC7A14	KIF26A
TRPC5	CNTN1	GYG2	MPPED2	FAM149A	XK	GABRA3	SLC9A3R2	CNTN1	ATP2B3
TRHDE	PLEKHB1	RASGRF1	ATP2B3	PTPN5	FSTL4	SLC7A14	TLE2	PLEKHB1	RASGRP2
PPP2R2C	MRI1	LZTS1	RASGRP2	EFEMP1	CCDC85A	CNTN1	KIF26A	MRI1	SPTB
NUAK1	CAPG	TLE2	TRPC5	IGSF21	F7	TNC	ATP2B3	CDH10	TRPC5
FOSL2	TG	KIF26A	PPP2R2C	EFNB2	RASGRF1	TG	ACSL4	MYO16	TRHDE
NEBL	JADE2	ATP2B3	SNCB	BCL11B	CAMK2B	JADE2	MGLL	TNC	PPP2R2C
BRINP1	XK	RASGRP2	NUAK1	PUDP	DGKG	LMO3	NUAK1	CAPG	MGLL
STK17B	LMO3	CAMK2A	FOSL2	MEIS2	PTPRU	FSTL4	FOSL2	JADE2	WSCD2
P2RX5	FSTL4	NUAK1	NEBL	FHDC1	SLC9A3R2	PTPRN	CDH7	LMO3	CACNG5
COL16A1	PTPRN	SEMA3A	ADCY2	LRIG3	PDE4A	GYG2	P2RX5	GYG2	NEBL
SEL1L3	CCDC85A	FOSL2	BRINP1	MDGA2	KIF26A	F7	ABCB1	DGKG	ADCY2
CRTAC1	GYG2	NEBL	COL16A1	GABRB2	ATP2B3	RASGRF1	TTC39A	RIMBP2	BRINP1
[illegible]	[illegible]	[illegible]	[illegible]	[illegible]	[illegible]	[illegible]	[illegible]	[illegible]	[illegible]

VWC2
SHISA6
BEND4
FAM78B
INSYN2A
RELN
IL1RAPL2
PTPRT
SPOCK3
GRM7
CPNE4
SLC30A10
LAMB3
FAM163B
SLC6A17
ADGRA1
DCHS2
OGDHL
PDGFA
COL4A6
S100A10
H4C16
ADAMTSL2
CYRIA
S100A6
MBP
CHSY3
LPAR1
TMEM229B
CACNA1E
RYR2
LRRTM3
RASGEF1A
STUM
RIPPLY2
FOXD6
TCEAL5
TCEAL6
MAFB
COL15A1
DPF3
C22orf42
CPLX3
EML6
HMX1
TSTD1
SP9
PLXNA4
FNDC10
AJM1
TMEM238
CLEC2L
SHISA9
OR2W3
CEBPA
TRNP1
CTSO
SALL3
TMEM179
FRRS1L
GOLGA6L7
ZIM2
SRXN1
DOC2B
GRIN2B

GRIN2B

ADGRA1
DCHS2
OGDHL
PDGFA
DPP4
S100A10
ADAMTSL2
CYRIA
S100A6
MBP
CHSY3
LPAR1
TMEM229B
CACNA1E
RYR2
LRRTM3
BRINP2
RASGEF1A
STUM
FOXD6
TCEAL5
TCEAL6
MAFB
C2orf72
DPF3
DENND1C
C22orf42
DENND1B
CPLX3
EML6
HMX1
TSTD1
SP9
PLXNA4
FNDC10
AJM1
TMEM238
CLEC2L
SHISA9
PSMB9
SMKR1
CEBPA
SELENOP
TRNP1
SALL3
ZNF578
TMEM179
FRRS1L
GOLGA6L7
ZIM2
SRXN1
DOC2B
GRIN2B

MPPED1
VSTM2B
SHTN1
C11orf96
C17orf97
NHS
SMTNL2
ENO4
SLC38A3
COL25A1
CCDC9B
NKAIN2
PAQR9
VWC2
SHISA6
BEND4
FAM78B
INSYN2A
RELN
IL1RAPL2
PTPRT
SPOCK3
GRM7
CPNE4
FUT4
SLC30A10
FAM163B
SLC6A17
ADGRA1
DCHS2
OGDHL
PDGFA
COL4A6
DPP4
H4C16
ADAMTSL2
S100A6
MBP
CHSY3
LPAR1
TMEM229B
CACNA1E
RYR2
BRINP2
CCDC152
RASGEF1A
STUM
RIPPLY2
FOXD6
TCEAL5
TCEAL6
MAFB
C2orf72
COL15A1
DPF3
DENND1B
FAM24B
EML6
C10orf105
UBE2QL1
HMX1
TSTD1
SP9
PLXNA4
CENPVL1
CENPVL3
FNDC10
TMEM238
CLEC2L
SHISA9
PSMB9
SMKR1
CEBPA
TRNP1
SALL3
ZNF578
TMEM179
FRRS1L
ZIM2
SRXN1
DOC2B
GRIN2B

DPF3
DENND1C
C22orf42
DENND1B
CPLX3
EML6
C10orf105
UBE2QL1
HMX1
TSTD1
SP9
PLXNA4
CENPVL1
FNDC10
AJM1
CLEC2L
SHISA9
PSMB9
SMKR1
CEBPA
TRNP1
SALL3
ZNF578
FRRS1L
GOLGA6L7
ZIM2
SRXN1
DOC2B
GRIN2B

TF	TF	TF	TF	TF	TF	TF	TF	TF	TF	TF	TF	TF	TF	TF	TF	TF	TF	TF	TF	TF
TF:M01118	TF:M12732_1	TF:M12590	TF:M03893	TF:M03895	TF:M09723	TF:M11482	TF:M12593_1	TF:M12686	TF:M01100	TF:M11531	TF:M00325	TF:M00051_1	TF:M01587	TF:M07208_1	TF:M01865_1	TF:M12158	TF:M01865	TF:M12354_1	TF:M00256	TF:M04152_1
Factor:_WT1;_motif:_SMCNCCNSC	Factor:_ZNF532;_motif:_NGGRGCTGGG;_match_class:_1	Factor:_CP2;_motif:_NRRRCCGGCYNRARCCGGNN	Factor:_WT1;_motif:_GNGGGGGCGGGG	Factor:_CTCF;_motif:_WGCGCCMYCTAGYGGYN	Factor:_BTEB1;_motif:_GGGGGCGGGGCNGSGGGNGS	Factor:_AP-2gamma;_motif:_NSCCYNNRGSN	Factor:_CTCF;_motif:_NNRSYGCCMCCTGSTGGCCN;_match_class:_1	Factor:_ZBTB10;_motif:_TGGGGTGCNS	Factor:_LRF;_motif:_GGGGKYNNB	Factor:_E2F-2;_motif:_GCGCGCGCGYW	Factor:_NRSE;_motif:_TTYAGCWCCDCGGASAGYRCC	Factor:_NF-kappaB;_motif:_GGGGATYCCC;_match_class:_1	Factor:_FPM315;_motif:_SRGGGAGGAGGN	Factor:_EGR1;_motif:_NCNCCGCCCCCGCN;_match_class:_1	Factor:_BTEB3;_motif:_BNRNGGGAGGNGT;_match_class:_1	Factor:_KLF15;_motif:_NCCMCGCCCMCN	Factor:_BTEB3;_motif:_BNRNGGGAGGNGT	Factor:_ZNF37A;_motif:_CCYYGGCTCCNTSCCMN;_match_class:_1	Factor:_NRSF;_motif:_TTCAGCACCACGGACAGMGCC	Factor:_TFAP2C;_motif:_NGCCCNNRGGCA;_match_class:_1
335	195	493	392	427	462	328	188	190	362	572	106	162	183	135	225	493	449	283	33	279
YBX2	ITGA3	PLXND1	PLXND1	HS3ST1	PLXND1	PLXND1	PLXND1	YBX2	PLXND1	HS3ST1	ITGA3	MATK	HS3ST1	YBX2	HS3ST1	HS3ST1	HS3ST1	PLXND1	RASGRF1	PLXND1
CACNG3	TAC1	ITGA3	YBX2	PLXND1	ITGA3	YBX2	YBX2	TAC1	CACNG3	PLXND1	CACNG3	NFIX	ETV1	CYB561	CEACAM21	PLXND1	PLXND1	ITGA3	TRPC7	YBX2
PNPLA4	MATK	YBX2	PNPLA4	YBX2	YBX2	GAS7	CAPG	GAS7	TAC1	ITGA3	ETV1	CAPG	PNPLA4	JADE2	MATK	ITGA3	ITGA3	YBX2	RPH3A	PNPLA4
CYB561	SYT7	ETV1	GAS7	CACNG3	CEACAM21	MATK	JADE2	SYT7	CEACAM21	YBX2	SLC6A7	F7	CEACAM21	CAMK2B	NFIX	YBX2	YBX2	PNPLA4	CHGA	GAS7
NFIX	CNTN1	PNPLA4	MATK	ETV1	GAS7	CYB561	PTPRN	SLC7A14	MATK	CACNG3	SYT7	DGKG	NFIX	PTPRU	PLEKHB1	GAS7	PNPLA4	GAS7	STS	PAX6
SYT7	CAPG	GAS7	CYB561	PNPLA4	MATK	SLC6A7	CAMK2B	CNTN1	PAX6	TAC1	RASGRF1	PDE4A	FSTL4	SLC9A3R2	CDH10	MATK	CEACAM21	PAX6	HBEGF	CYB561
PLEKHB1	CCDC85A	MATK	SLC6A7	GAS7	PAX6	SYT7	PTPRU	FSTL4	CYB561	PNPLA4	TRPC7	NGEF	GYG2	TLE2	JADE2	PAX6	GAS7	SYT7	GABRG2	SYT7
CDH10	GYG2	PAX6	SYT7	MATK	CYB561	PLEKHB1	SLC9A3R2	F7	SLC6A7	GAS7	SNCB	MPPED2	RASGRF1	NGEF	GYG2	CYB561	MATK	SLC7A14	TNR	PLEKHB1
JADE2	F7	CYB561	CDH10	PAX6	NFIX	TNC	TLE2	CAMK2B	CAPG	MATK	SEMA3C	CNGB1	RIMBP2	MPPED2	RASGRF1	NFIX	CAMK1G	CNTN1	NXPH1	JADE2
FSTL4	SLC9A3R2	SLC6A7	CAPG	SYT7	SLC6A7	JADE2	PDE4A	RIMBP2	JADE2	PAX6	FOSL2	PPP2R2C	TLE2	RASGRP2	RIMBP2	SLC6A7	CYB561	TG	CHST8	CCDC85A
GYG2	MPPED2	SYT7	TG	CNTN1	SYT7	XK	KIF26A	TLE2	GYG2	CAMK1G	NEBL	SNCB	NGEF	TRHDE	MPPED2	SYT7	NFIX	JADE2	VGF	GYG2
F7	ATP2B3	SLC7A14	JADE2	MRI1	SLC7A14	FSTL4	SPTB	NGEF	CAMK2B	CYB561	ADCY2	MGLL	MPPED2	PPP2R2C	KIF26A	SLC7A14	SLC6A7	FSTL4	PUDP	F7
RASGRF1	RASGRP2	CNTN1	XK	CAPG	CNTN1	PTPRN	PPP2R2C	PPP2R2C	DGKG	NFIX	ACHE	NUAK1	KIF26A	NEBL	ATP2B3	PLEKHB1	SYT7	GYG2	BEX2	CAMK2B
DGKG	SPTB	PLEKHB1	PTPRN	JADE2	PLEKHB1	CCDC85A	FOSL2	NUAK1	RIMBP2	SLC6A7	RPH3A	FOSL2	ATP2B3	ADCY2	RASGRP2	TNC	CNTN1	F7	TMTC1	DGKG
PTPRU	PPP2R2C	MRI1	CCDC85A	XK	MRI1	GYG2	NEBL	CARMIL1	SLC9A3R2	SYT7	PITPNM2	COL19A1	SPTB	MID2	SPTB	CAPG	PLEKHB1	CAMK2B	SCN2A	PTPRU
SLC9A3R2	SNCB	TNC	CAMK2B	FSTL4	CAPG	F7	ADCY2	MID2	TLE2	SLC7A14	COL9A3	COL16A1	TRHDE	P2RX5	TRPC5	TG	CDH10	SLC9A3R2	LRIG3	RIMBP2
TLE2	NUAK1	CAPG	DGKG	PTPRN	JADE2	RASGRF1	STK17B	WNT11	PDE4A	MRI1	SEZ6L	WNT11	PPP2R2C	ACHE	TRHDE	JADE2	CAPG	TLE2	PLCH2	LZTS1
PDE4A	FOSL2	JADE2	PTPRU	CCDC85A	XK	PTPRU	COL16A1	PITPNM2	NGEF	TNC	CHGA	ACHE	IPCEF1	SEL1L3	PPP2R2C	XK	JADE2	NGEF	GOLGA7B	SLC9A3R2
NGEF	CACNG5	FSTL4	RIMBP2	GYG2	FSTL4	RIMBP2	PITPNM2	SEL1L3	KIF26A	CAPG	DOK5	NOS1	FOSL2	COL9A3	SNCB	LMO3	XK	MPPED2	SST	TLE2
MPPED2	NEBL	PTPRN	SLC9A3R2	RASGRF1	PTPRN	SLC9A3R2	SEL1L3	MYH7	RASGRP2	JADE2	NTSR1	PITPNM2	BRINP1	HCN2	NUAK1	PTPRN	FSTL4	KIF26A	HTR5A	PDE4A
KIF26A	RAPGEF3	CCDC85A	TLE2	CAMK2B	CCDC85A	TLE2	CRTAC1	HCN2	SPTB	XK	CELF4	MYH7	CARMIL1	RSPO4	FOSL2	CCDC85A	CCDC85A	ATP2B3	ADAMTS9	NGEF
ATP2B3	MID2	GYG2	PDE4A	PTPRU	GYG2	NGEF	HCN2	PDGFB	TRHDE	FSTL4	STS	COL9A3	P2RX5	ST8SIA5	NEBL	GYG2	GYG2	RASGRP2	HEY1	KIF26A
ACSL4	P2RX5	F7	NGEF	SLC9A3R2	F7	KIF26A	RASD2	CACNA1I	SNCB	PTPRN	SFRP1	HCN2	COL16A1	SYT17	BRINP1	F7	RASGRF1	CNGB1	VWC2L	RASGRP2
RASGRP2	WNT11	CAMK2B	MPPED2	TLE2	CAMK2B	ATP2B3	PDGFB	NTSR1	MGLL	CCDC85A	TUBB4A	RASD2	NOS1	RHOV	CARMIL1	RASGRF1	CAMK2B	TRPC5	CNTNAP2	SPTB
CNGB1	CRTAC1	DGKG	KIF26A	PDE4A	PTPRU	RASGRP2	CACNA1I	SAMHD1	NUAK1	GYG2	FBXW4	MMP9	PITPNM2	SLC17A7	WNT11	CAMK2B	DGKG	PPP2R2C	TTC9B	TRPC5
TRHDE	SEZ6L	PTPRU	ATP2B3	NGEF	RIMBP2	SPTB	DOK5	MAP1LC3A	NEBL	RASGRF1	CCKBR	SLCO4A1	SUSD2	NEURL1	ACHE	RIMBP2	PTPRU	SNCB	AMER3	SNCB
PPP2R2C	PDGFB	RIMBP2	ACSL4	MPPED2	SLC9A3R2	CNGB1	NTSR1	SYT17	ADCY2	CAMK2B	SYT10	PPP1R16B	SEZ6L	SORCS1	NOS1	LZTS1	RIMBP2	NUAK1	MARCHF11	MGLL
SNCB	SLCO4A1	LZTS1	RASGRP2	KIF26A	TLE2	TRHDE	PPP1R16B	TUBB4A	RAPGEF3	DGKG	HBEGF	ST8SIA5	CHRDL1	EYA4	SEL1L3	SLC9A3R2	SLC9A3R2	SEMA3C	PAQR9	NUAK1
NUAK1	NTSR1	SLC9A3R2	SPTB	ATP2B3	PDE4A	PPP2R2C	MAP1LC3A	SLC17A7	CARMIL1	PTPRU	EFEMP1	SALL1	KLF8	SEMA5A	COL9A3	TLE2	TLE2	FOSL2	FAM163B	WSCD2
WSCD2	CDH20	TLE2	CAMK2A	ACSL4	NGEF	MGLL	ST8SIA5	AMH	MID2	RIMBP2	IGFBP5	SYT17	SFRP1	SLC9A2	HCN2	PDE4A	NGEF	NEBL	OGDHL	DCX
[illegible]	[illegible]	[illegible]	[illegible]	[illegible]	[illegible]	[illegible]	[illegible]	[illegible]	[illegible]	[illegible]	[illegible]	[illegible]	[illegible]	[illegible]	[illegible]	[illegible]	[illegible]	[illegible]	[illegible]	[illegible]

SRXN1	SHTN1	SRXN1
DOC2B	GCNT1	DOC2B
GRIN2B	C11orf96	GRIN2B
	C17orf97	
	COL4A5	
	NHS	
	SMTNL2	
	ENO4	
	SLC38A3	
	COL25A1	
	NKAIN2	
	PAQR9	
	VWC2	
	SHISA6	
	BEND4	
	FAM78B	
	INSYN2A	
	RELN	
	IL1RAPL2	
	PTPRT	
	SPOCK3	
	GRM7	
	FUT4	
	SLC30A10	
	FAM163B	
	SLC6A17	
	ADGRA1	
	DCHS2	
	OGDHL	
	PDGFA	
	DPP4	
	ADAMTSL2	
	CYRIA	
	S100A6	
	MBP	
	CHSY3	
	LPAR1	
	TMEM229B	
	CACNA1E	
	RYR2	
	LRRTM3	
	BRINP2	
	RASGEF1A	
	STUM	
	RIPPLY2	
	FOXD6	
	TCEAL6	
	MAFB	
	C2orf72	
	COL15A1	
	DPF3	
	DENND1B	
	FAM24B	
	CPLX3	
	EML6	
	UBE2QL1	
	HMX1	
	TSTD1	
	SP9	
	PLXNA4	
	RAB6C	
	CENPVL1	
	CENPVL3	
	FNDC10	
	RAB6D	
	TMEM238	
	CLEC2L	
	SHISA9	
	PSMB9	
	SMKR1	
	CEBPA	
	SELENOP	
	TRNP1	
	CTSO	
	SALL3	
	ZNF578	
	TMEM179	
	FRRS1L	
	ZIM2	
	SRXN1	
	DOC2B	
	GRIN2B	

TF<br>TF:M01855_1<br>Factor:_AHR;_motif:_CACGCN;_match_class:_1<br>160	TF<br>TF:M11595_1<br>Factor:_GCMa;_motif:_RTGCGGGTN;_match_class:_1<br>289	TF<br>TF:M12703<br>Factor:_ZNF383;_motif:_SSNGGGMGGNGSNGGS<br>418	TF<br>TF:M08867<br>Factor:_AP2;_motif:_GCCYGSGGSN<br>454	TF<br>TF:M04869_1<br>Factor:_Egr-1;_motif:_GCGCATGCG;_match_class:_1<br>462	TF<br>TF:M08487_1<br>Factor:_GCMa:Erg;_motif:_ATGCGGGCGGAARKG;_match_class:_1<br>407	TF<br>TF:M09636<br>Factor:_MAZ;_motif:_GGGMGGGGSSGGGGGGGGGGG<br>665
CACNG3	ITGA3	PLXND1	HS3ST1	HS3ST1	HS3ST1	HS3ST1
TAC1	YBX2	ITGA3	PLXND1	PLXND1	PLXND1	PLXND1
MATK	TAC1	YBX2	ITGA3	YBX2	YBX2	ITGA3
SLC6A7	PNPLA4	CACNG3	YBX2	TAC1	CACNG3	YBX2
MRI1	CYB561	ETV1	PNPLA4	PNPLA4	PNPLA4	CACNG3
FSTL4	NFIX	GAS7	GAS7	GAS7	GAS7	TAC1
CCDC85A	SLC6A7	MATK	MATK	MATK	MATK	ETV1
GYG2	SYT7	PAX6	CYB561	PAX6	CYB561	CEACAM21
RASGRF1	SLC7A14	CYB561	SYT7	NFIX	NFIX	GAS7
PTPRU	CNTN1	NFIX	CNTN1	SLC6A7	SLC6A7	MATK
RIMBP2	JADE2	SYT7	PLEKHB1	SYT7	SYT7	PAX6
SLC9A3R2	PTPRN	SLC7A14	TNC	SLC7A14	SLC7A14	CAMK1G
[illegible]	[illegible]	[illegible]	[illegible]	[illegible]	[illegible]	[illegible]

TF<br>TF:M00444_1<br>Factor:_VDR;_motif:_GGGKNARNRRGGWSA;_match_class:_1<br>238	TF<br>TF:M04953<br>Factor:_Sp1;_motif:_GGNDGGRGGCGGGG<br>402	TF<br>TF:M01733<br>Factor:_MZF-1;_motif:_TGGGGAR<br>499	TF<br>TF:M00444<br>Factor:_VDR;_motif:_GGGKNARNRRGGWSA<br>462	TF<br>TF:M03811<br>Factor:_AP-2gamma;_motif:_GCCYNCRGSN<br>407	TF<br>TF:M04869<br>Factor:_Egr-1;_motif:_GCGCATGCG<br>497	TF<br>TF:M10072<br>Factor:_sp4;_motif:_NNGNARGRGGCGGRGCNNRR<br>471
CACNG3	PLXND1	ITGA3	PLXND1	PLXND1	HS3ST1	HS3ST1
MATK	ITGA3	YBX2	YBX2	YBX2	PLXND1	PLXND1
CAMK1G	YBX2	TAC1	CACNG3	CACNG3	YBX2	ITGA3
CYB561	TAC1	ETV1	TAC1	GAS7	CACNG3	YBX2
NFIX	GAS7	PNPLA4	ETV1	PAX6	TAC1	CACNG3
SYT7	MATK	CEACAM21	PNPLA4	CYB561	PNPLA4	CEACAM21
PLEKHB1	CYB561	GAS7	CEACAM21	NFIX	GAS7	GAS7
TNC	NFIX	PAX6	MATK	SLC6A7	MATK	MATK
PTPRN	SLC6A7	CYB561	CAMK1G	SYT7	PAX6	PAX6
CCDC85A	CNTN1	NFIX	CYB561	CNTN1	NFIX	CYB561
F7	PLEKHB1	SLC6A7	NFIX	PLEKHB1	SLC6A7	SLC6A7
RASGRF1	CAPG	SYT7	TENM1	JADE2	SYT7	SYT7
[illegible]	[illegible]	[illegible]	[illegible]	[illegible]	[illegible]	[illegible]

TF<br>TF:M09603<br>Factor:_Egr-1;_motif:_NNNNNGYGKGGGNGGGNN<br>240	TF<br>TF:M04863<br>Factor:_TF3C-beta;_motif:_CCNGGAGGGCTTCCTGGAGGAG<br>578	TF<br>TF:M11478<br>Factor:_AP-2beta;_motif:_NSCCNNNGGSN<br>448	TF<br>TF:M00333<br>Factor:_ZF5;_motif:_NRNGNGCGCGCWN<br>627	TF<br>TF:M02023<br>Factor:_MAZ;_motif:_NKGGGAGGGGGRGGR<br>340	TF<br>TF:M12725_1<br>Factor:_PHR;_motif:_NCCCAGCCCCY;_match_class:_1<br>228	TF<br>TF:M09834<br>Factor:_ZNF148;_motif:_NNNNNNCCNNCCCCTCCCCCACCCN<br>333
YBX2	HS3ST1	HS3ST1	HS3ST1	PLXND1	PLXND1	PLXND1
CYB561	PLXND1	PLXND1	PLXND1	ITGA3	ITGA3	YBX2
TG	ITGA3	ITGA3	ITGA3	YBX2	YBX2	CACNG3
JADE2	YBX2	YBX2	YBX2	CACNG3	TAC1	ETV1
CCDC85A	CACNG3	PNPLA4	CACNG3	ETV1	MATK	GAS7
CAMK2B	TAC1	GAS7	TAC1	PNPLA4	CYB561	CYB561
PTPRU	ETV1	MATK	PNPLA4	MATK	SYT7	NFIX
LZTS1	PNPLA4	CYB561	CEACAM21	NFIX	CNTN1	SLC6A7
SLC9A3R2	CEACAM21	TENM1	GAS7	SLC6A7	CAPG	CNTN1
TLE2	GAS7	SLC6A7	MATK	CNTN1	JADE2	PLEKHB1
NGEF	MATK	SYT7	PAX6	PLEKHB1	GYG2	CDH10
MPPED2	PAX6	SLC7A14	CAMK1G	CDH10	RIMBP2	JADE2
[illegible]	[illegible]	[illegible]	[illegible]	[illegible]	[illegible]	[illegible]

PCSK1
CREG2
ARL4D
LYGH
DSCAML1
RIMKLA
KCNA3
FZD8
ST8SIA3
KCNJ10
ZNF354C
ALS2CL
AMER3
KCTD12
RPP25
CPNE7
FAM133A
ALOXE3
TMEM151A
FJX1
SLITRK4
LACC1
AKAP5
ZNF154
TSHZ1
LYNX1
PLD5
TSPYL5
GSX2
SSTR2
SHISA2
FAM83H
CHRM2
PENK
TMEM132C
TMEM132E
DDX60L
GPR88
GPR3
ADGRB1
FAM181B
KCNK4
RGS6
GALNT9
BEGAIN
FAM43B
CALN1
CTNNA3
PCBP3
MARCHF11
EFHC2
NOG
OPCML
ADRA2C
KCNJ12
ACOT1
PRKD1
MAP7D2
PABPC1L2B
CLDN6
TCEAL2
FMNL1
ROBO2
SLC24A3
INPP5J
GALNT17
SV2B
FAM131C
AHNAK2
C11orf87
KCNQ5
ADAMTSL5
GABRA5
NAP1L3
CYP4X1
PDE2A
MPPED1
VSTM2B
SHTN1
GCNT1
C11orf96
C17orf97
COL4A5
NHS
SMTNL2
ENO4
SLC38A3
COL25A1
CCDC9B
NKAIN2
PAQR9
VWC2
SHISA6
BEND4
FAM78B
INSYN2A
RELN
IL1RAPL2
MAOA
PTPRT
SPOCK3
GRM7
CPNE4
FUT4
SLC30A10
LAMB3
FAM163B
SLC6A17
ADGRA1
DCHS2
OGDHL
PDGFA
COL4A6
DPP4
S100A10
H4C16
ADAMTSL2
CYRIA
S100A6
MBP
DLGAP2
CHSY3
LPAR1
TMEM229B
CACNA1E
RYR2
LRRTM3
BRINP2
CCDC152
RASGEF1A
STUM
RIPPLY2
FOXO6
TCEAL5
TCEAL6
MAFB
C2orf72
COL15A1
DPF3
DENND1C
C22orf42
DENND1B
FAM24B
CPLX3
S1PR3
EML6
C10orf105
UBE2QL1
HMX1
TSTD1
SP9
PLXNA4
RAB6C
CENPVL1
CENPVL3
FNDC10
AJM1
RAB6D
TMEM238
CLEC2L
SHISA9
PSMB9
SMKR1
CEBPA
TRNP1
CTSO
SALL3
ZNF578
TMEM179
FRRS1L
GOLGA6L7
ZIM2
SRXN1
DOC2B
GRIN2B

PSMB9
SMKR1
CEBPA
TRNP1
ZNF578
TMEM179
FRRS1L
GOLGA6L7
GRIN2B

SALL3
ZNF578
TMEM179
FRRS1L
ZIM2
DOC2B
GRIN2B

ADAMTSL5
GABRA5
NAP1L3
CYP4X1
PDE2A
MPPED1
MAPT
VSTM2B
SHTN1
GCNT1
C11orf96
C17orf97
COL4A5
NHS
SMTNL2
ENO4
SLC38A3
COL25A1
CCDC9B
NKAIN2
PAQR9
VWC2
SHISA6
BEND4
FAM78B
INSYN2A
RELN
IL1RAPL2
MAOA
PTPRT
GRM7
FUT4
SLC30A10
LAMB3
FAM163B
SLC6A17
ADGRA1
DCHS2
OGDHL
PDGFA
DPP4
S100A10
H4C16
ADAMTSL2
CYRIA
S100A6
MBP
CHSY3
LPAR1
TMEM229B
CACNA1E
RYR2
BRINP2
RASGEF1A
STUM
RIPPLY2
FOXO6
MAFB
C2orf72
COL15A1
DPF3
DENND1C
FAM24B
CPLX3
EML6
HMX1
TSTD1
SP9
PLXNA4
RAB6C
CENPVL1
CENPVL3
FNDC10
AJM1
RAB6D
TMEM238
CLEC2L
SHISA9
PSMB9
SMKR1
CEBPA
TRNP1
SALL3
TMEM179
FRRS1L
GOLGA6L7
SRXN1
DOC2B

SHISA2
FAM83H
ZNF572
RPH3AL
CHRM2
PENK
TMEM132C
TMEM132E
DDX60L
GPR88
GPR3
ADGRB1
FAM181B
KCNK4
RGS6
GALNT9
BEGAIN
GPC6
FAM43B
CALN1
CTNNA3
PCBP3
MARCHF11
EFHC2
NOG
OPCML
ADRA2C
KCNJ12
ACOT1
PRKD1
MAP7D2
PABPC1L2B
CLDN6
TCEAL2
FMNL1
ROBO2
SLC24A3
INPP5J
GALNT17
SV2B
FAM131C
AHNAK2
C11orf87
KCNQ5
ADAMTSL5
GABRA5
CYP4X1
PDE2A
MPPED1
VSTM2B
SHTN1
GCNT1
C11orf96
C17orf97
COL4A5
NHS
SMTNL2
ENO4
SLC38A3
COL25A1
NKAIN2
PAQR9
VWC2
SHISA6
BEND4
FAM78B
INSYN2A
RELN
IL1RAPL2
MAOA
PTPRT
SPOCK3
GRM7
CPNE4
FUT4
SLC30A10
FAM163B
SLC6A17
ADGRA1
DCHS2
OGDHL
PDGFA
DPP4
S100A10
H4C16
ADAMTSL2
CYRIA
MBP
CHSY3
LPAR1
TMEM229B
CACNA1E
RYR2
BRINP2
CCDC152
RASGEF1A
STUM
RIPPLY2
FOXO6
TCEAL5
TCEAL6
MAFB
C2orf72
COL15A1
DPF3
DENND1B
FAM24B
CPLX3
EML6
UBE2QL1
HMX1
TSTD1
SP9
PLXNA4
RAB6C
CENPVL1
CENPVL3
FNDC10
AJM1
RAB6D
TMEM238
CLEC2L
SHISA9
PSMB9
SMKR1
CEBPA
SELENOP
TRNP1
CTSO
SALL3
ZNF578
TMEM179
FRRS1L
ZIM2
SRXN1
DOC2B
GRIN2B

TF	TF	TF	TF	TF	TF	TF
TF:M12590_1	TF:M11058	TF:M00807	TF:M03807	TF:M10107_1	TF:M04152	TF:M07397
Factor:_CP2;_motif:_NRRRCCGGCYNRARCCGGNN;_match_class:_1	Factor:_Hey1;_motif:_NGCRCGYGYN	Factor:_Egr;_motif:_GTGGGSGCRRS	Factor:_SP2;_motif:_GNNGGGGGCGGGGN	Factor:_DB1;_motif:_GGRRRRGRRGGAGGGGGNGRRR;_match_class:_1	Factor:_TFAP2C;_motif:_NGCCCNNRGGCA	Factor:_ZBP89;_motif:_CCCCKCCCCCNN
324	561	228	390	153	361	340
PLXND1	HS3ST1	PLXND1	PLXND1	HS3ST1	PLXND1	PLXND1
ITGA3	PLXND1	ITGA3	ITGA3	PLXND1	YBX2	YBX2
YBX2	ITGA3	PNPLA4	YBX2	YBX2	ETV1	CACNG3
PNPLA4	YBX2	GAS7	CACNG3	CYB561	PNPLA4	GAS7
GAS7	CACNG3	CYB561	CEACAM21	NFIX	CEACAM21	MATK
PAX6	TAC1	SLC6A7	GAS7	LMO3	GAS7	CYB561
CYB561	PNPLA4	SYT7	MATK	RIMBP2	MATK	NFIX
SYT7	GAS7	SLC7A14	CYB561	NGEF	PAX6	SLC6A7
SLC7A14	MATK	TG	SYT7	MPPED2	CYB561	PLEKHB1
CNTN1	PAX6	JADE2	SLC7A14	ATP2B3	SYT7	CDH10
[illegible]	[illegible]	[illegible]	[illegible]	[illegible]	[illegible]	[illegible]

TF	TF	TF	TF	TF	TF	TF
TF:M11069_1	TF:M07249_1	TF:M10426	TF:M00470	TF:M01100_1	TF:M12598_1	TF:M12160_1
Factor:_HES-5;_motif:_NCACACKY;_match_class:_1	Factor:_ctcf;_motif:_CCNCNAGRKGGCRSTN;_match_class:_1	Factor:_ctcf;_motif:_CCRSCAGGGGGCGCN	Factor:_AP-2gamma;_motif:_GCCYNNGGS	Factor:_LRF;_motif:_GGGGKYNNR;_match_class:_1	Factor:_E2F3;_motif:_NNRGMKGGAR;_match_class:_1	Factor:_KLF15;_motif:_RCCMCRCCCMCN;_match_class:_1
219	126	423	426	156	331	374
CACNG3	PLXND1	HS3ST1	HS3ST1	TAC1	HS3ST1	PLXND1
ETV1	YBX2	PLXND1	PLXND1	MATK	PLXND1	ITGA3
MATK	GAS7	YBX2	ITGA3	JADE2	ITGA3	YBX2
PAX6	JADE2	GAS7	YBX2	GYG2	YBX2	GAS7
NFIX	FSTL4	MATK	PNPLA4	CAMK2B	ETV1	MATK
SYT7	CCDC85A	SLC6A7	GAS7	RIMBP2	PNPLA4	CYB561
PLEKHB1	KIF26A	SYT7	MATK	SLC9A3R2	GAS7	NFIX
CDH10	PPP2R2C	CAPG	PAX6	TLE2	MATK	SLC6A7
RAB27B	WSCD2	JADE2	CYB561	NGEF	PAX6	SYT7
MYO16	FOSL2	XK	SLC6A7	KIF26A	SLC7A14	SLC7A14
[illegible]	[illegible]	[illegible]	[illegible]	[illegible]	[illegible]	[illegible]

TF	TF	TF	TF	TF	TF	TF
TF:M09890	TF:M10151	TF:M09739	TF:M03932	TF:M04797	TF:M11058_1	TF:M01219_1
Factor:_ctcf;_motif:_YGGCCACCAGRKGGCRSYN	Factor:_EZI;_motif:_GGGGAGGGGRRGGRGRRGGRRR	Factor:_ZGPAT;_motif:_GRGGCWGNGGNG	Factor:_ZIC4;_motif:_GRCCCCCCGCNGNGN	Factor:_Egr-1;_motif:_CCGCCCMCG	Factor:_Hey1;_motif:_NGCRCGYGYN;_match_class:_1	Factor:_SP1:SP3;_motif:_CCSCCCCCYCC;_match_class:_1
389	127	380	153	256	479	164
HS3ST1	HS3ST1	HS3ST1	HS3ST1	PLXND1	HS3ST1	PLXND1
PLXND1	YBX2	PLXND1	PLXND1	YBX2	PLXND1	JADE2
ITGA3	MATK	ITGA3	GAS7	GAS7	ITGA3	PTPRN
YBX2	NFIX	YBX2	MATK	MATK	YBX2	RASGRF1
MATK	SLC7A14	CACNG3	NFIX	CYB561	CACNG3	CAMK2B
CYB561	PTPRN	TAC1	SYT7	NFIX	TAC1	SLC9A3R2
NFIX	GYG2	CEACAM21	JADE2	SYT7	PNPLA4	TLE2
SLC6A7	RIMBP2	GAS7	GYG2	MRI1	GAS7	RASGRP2
SYT7	SLC9A3R2	MATK	KIF26A	CDH10	MATK	SPTB
CAPG	TLE2	CYB561	ATP2B3	TNC	CYB561	TRHDE
[illegible]	[illegible]	[illegible]	[illegible]	[illegible]	[illegible]	[illegible]

SHISA6
BEND4
FAM78B
INSYN2A
RELN
PTPRT
SPOCK3
GRM7
FUT4
SLC30A10
FAM163B
SLC6A17
ADGRA1
DCHS2
OGDHL
PDGFA
DPP4
S100A10
H4C16
CYRIA
S100A6
MBP
DLGAP2
CHSY3
LPAR1
TMEM229B
RYR2
BRINP2
RASGEF1A
STUM
RIPPLY2
FOXO6
TCEAL5
TCEAL6
MAFB
C2orf72
COL15A1
DPF3
DENND1C
DENND1B
FAM24B
CPLX3
S1PR3
EML6
UBE2QL1
HMX1
SP9
PLXNA4
RAB6C
CENPVL1
CENPVL3
FNDC10
AIM1
RAB6D
TMEM238
CLEC2L
SHISA9
PSMB9
SMKR1
CEBPA
TRNP1
CTSO
SALL3
ZNF578
TMEM179
FRRS1L
GOLGA6L7
ZIM2
SRXN1
DOC2B
GRIN2B

TF	TF	TF	TF	TF
TF:M11478_1	TF:M07250_1	TF:M09738_1	TF:M04516_1	TF:M09738
Factor:_AP-2beta;_motif:_NSCCNNNGGSN;_match_class:_1	Factor:_E2F-1;_motif:_NNNSSCGCSAANN;_match_class:_1	Factor:_ZNF511;_motif:_GGRRGRGGCWGNG;_match_class:_1	Factor:_E2F-1;_motif:_TTTGGCGCCAAA;_match_class:_1	Factor:_ZNF511;_motif:_GGRRGRGGCWGNG
372	273	240	456	476
HS3ST1	HS3ST1	PLXND1	HS3ST1	HS3ST1
PLXND1	PLXND1	YBX2	PLXND1	PLXND1
ITGA3	ITGA3	MATK	YBX2	ITGA3
YBX2	CACNG3	JADE2	CACNG3	YBX2
GAS7	GAS7	PTPRN	ETV1	TAC1
MATK	PAX6	CCDC85A	GAS7	ETV1
CYB561	JADE2	GYG2	MATK	GAS7
TENM1	XK	RASGRF1	PAX6	MATK
SLC6A7	GYG2	CAMK2B	CYB561	NFIX
SYT7	RASGRF1	RIMBP2	SYT7	SYT7
PLEKHB1	CAMK2B	SLC9A3R2	SLC7A14	SLC7A14
TG	DGKG	TLE2	CNTN1	CNTN1
JADE2	PTPRU	SPTB	MRI1	MRI1
XK	TLE2	MGLL	CAPG	CAPG
FSTL4	NGEF	NUAK1	JADE2	TG
PTPRN	ATP2B3	WSCD2	XK	JADE2
CCDC85A	TRHDE	CACNG5	FSTL4	PTPRN
GYG2	PPP2R2C	ADCY2	PTPRN	CCDC85A
F7	SNCB	BRINP1	GYG2	GYG2
RASGRF1	MGLL	CARMIL1	F7	F7
DGKG	NUAK1	P2RX5	RASGRF1	RASGRF1
PTPRU	FOSL2	WNT11	CAMK2B	CAMK2B
RIMBP2	CACNG5	TTC39A	DGKG	RIMBP2
SLC9A3R2	NEBL	ACHE	PTPRU	SLC9A3R2
PDE4A	BRINP1	PITPNM2	RIMBP2	TLE2
NGEF	CARMIL1	SEL1L3	SLC9A3R2	NGEF
MPPED2	MID2	COL9A3	TLE2	MPPED2
KIF26A	STK17B	CRTAC1	NGEF	RASGRP2
ATP2B3	WNT11	SUSD2	MPPED2	SPTB
[illegible]	[illegible]	[illegible]	[illegible]	[illegible]

TF	TF	TF	TF	TF
TF:M12703_1	TF:M09761	TF:M09897	TF:M09727	TF:M00450
Factor:_ZNF383;_motif:_SSNGGGMGGNGSNGGS;_match_class:_1	Factor:_EGR1;_motif:_NGNGKGGGYGGNGS	Factor:_Egr-1;_motif:_NNNNGCGKGGGYGGNRN	Factor:_GEMIN3;_motif:_NCWGGRARRGRGNGNG	Factor:_Zic3;_motif:_NGGGKGGTC
224	258	219	391	586
PLXND1	PLXND1	YBX2	PLXND1	HS3ST1
YBX2	YBX2	CEACAM21	YBX2	PLXND1
MATK	CYB561	CYB561	CACNG3	YBX2
CYB561	TG	JADE2	TAC1	TAC1
NFIX	JADE2	CCDC85A	PNPLA4	ETV1
SYT7	XK	CAMK2B	CEACAM21	PNPLA4
JADE2	CCDC85A	PTPRU	CAMK1G	CEACAM21
PTPRN	CAMK2B	LZTS1	CYB561	GAS7
CAMK2B	DGKG	SLC9A3R2	NFIX	MATK
RIMBP2	PTPRU	NGEF	TENM1	PAX6
SLC9A3R2	SLC9A3R2	MPPED2	SLC6A7	CAMK1G
NGEF	TLE2	KIF26A	CNTN1	CYB561
MPPED2	PDE4A	ATP2B3	PLEKHB1	NFIX
KIF26A	NGEF	ACSL4	JADE2	TENM1
RASGRP2	MPPED2	RASGRP2	LMO3	SLC6A7
TRPC5	KIF26A	SPTB	GYG2	SYT7
MGLL	ACSL4	TRPC5	RASGRF1	SLC7A14
NUAK1	RASGRP2	TRHDE	RIMBP2	PLEKHB1
FOSL2	SPTB	PPP2R2C	TLE2	MRI1
CACNG5	TRPC5	NUAK1	PDE4A	RAB27B
NEBL	TRHDE	FOSL2	NGEF	JADE2
STK17B	PPP2R2C	NEBL	MPPED2	XK
P2RX5	NUAK1	ADCY2	KIF26A	LMO3
COL16A1	FOSL2	MID2	ATP2B3	FSTL4
WNT11	DCX	P2RX5	ACSL4	PTPRN
TTC39A	NEBL	TTC39A	RASGRP2	CCDC85A
ACHE	MID2	ACHE	TRPC7	GYG2
PITPNM2	STK17B	SEL1L3	SPTB	F7
SEL1L3	P2RX5	COL9A3	TRPC5	RASGRF1
[illegible]	[illegible]	[illegible]	[illegible]	[illegible]

TF	TF	TF	TF	TF
TF:M07348_1	TF:M00931	TF:M09727_1	TF:M12140	TF:M00933
Factor:_AP-2alpha;_motif:_NSCCNCRGGSN;_match_class:_1	Factor:_Sp1;_motif:_GGGGCGGGGC	Factor:_GEMIN3;_motif:_NCWGGRARRGRGNGNG;_match_class:_1	Factor:_Egr-1;_motif:_NNCRCCCMCGCNN	Factor:_Sp1;_motif:_CCCCGCCCCN
163	457	174	266	435
PLXND1	HS3ST1	YBX2	HS3ST1	PLXND1
GAS7	PLXND1	CEACAM21	PLXND1	ITGA3
MATK	ITGA3	CYB561	YBX2	YBX2
SLC6A7	YBX2	SLC6A7	CYB561	CACNG3
SYT7	CACNG3	JADE2	NFIX	TAC1
FSTL4	TAC1	LMO3	SLC6A7	GAS7
CCDC85A	CEACAM21	RIMBP2	TNC	MATK
PTPRU	GAS7	TLE2	JADE2	PAX6
LZTS1	MATK	MPPED2	XK	CYB561
SLC9A3R2	PAX6	KIF26A	PTPRN	NFIX
TLE2	CYB561	ATP2B3	CCDC85A	SLC6A7
MPPED2	SLC6A7	ACSL4	F7	SYT7
KIF26A	SYT7	TRPC7	CAMK2B	SLC7A14
ATP2B3	SLC7A14	TRPC5	DGKG	CNTN1
RASGRP2	CNTN1	NUAK1	LZTS1	PLEKHB1
TRHDE	PLEKHB1	FOSL2	SLC9A3R2	MRI1
NUAK1	MRI1	CARMIL1	TLE2	CDH10
FOSL2	CAPG	COL16A1	PDE4A	CAPG
ADCY2	TG	ACHE	NGEF	JADE2
BRINP1	JADE2	RPH3A	MPPED2	FSTL4
STK17B	XK	SEL1L3	KIF26A	PTPRN
TTC39A	FSTL4	COL9A3	ATP2B3	GYG2
NOS1	PTPRN	SUSD2	ACSL4	F7
PITPNM2	CCDC85A	SEZ6L	RASGRP2	RASGRF1
SEL1L3	GYG2	CACNA1I	SPTB	DGKG
COL9A3	F7	MMP9	TRPC5	PTPRU
HCN2	RASGRF1	DOK5	PPP2R2C	RIMBP2
SEZ6L	CAMK2B	CELF4	MGLL	SLC9A3R2
RASD2	RIMBP2	PLAT	NUAK1	TLE2
[illegible]	[illegible]	[illegible]	[illegible]	[illegible]

TF	TF	TF	TF	TF
TF:M03895_1	TF:M07277	TF:M04595	TF:M09972_1	TF:M12464_1
Factor:_CTCF;_motif:_WGCGCCMYCTAGYGGYN;_match_class:_1	Factor:_BTEB2;_motif:_RGGGNGKGGN	Factor:_SALL2;_motif:_GGGTGGG	Factor:_BTEB2;_motif:_WGGGTGKGGCNGGN;_match_class:_1	Factor:_ZNF148;_motif:_NNCCCCTCCCCC;_match_class:_1
187	389	553	205	144
YBX2	PLXND1	HS3ST1	PLXND1	ITGA3
PNPLA4	ITGA3	PLXND1	ITGA3	YBX2
GAS7	YBX2	ITGA3	YBX2	PLEKHB1
MATK	CACNG3	CACNG3	MATK	PTPRN
MRI1	CEACAM21	ETV1	SYT7	GYG2
CAPG	GAS7	PNPLA4	CNTN1	RASGRF1
JADE2	MATK	CEACAM21	PLEKHB1	SLC9A3R2
XK	CYB561	MATK	TG	TLE2
PTPRN	SLC6A7	PAX6	JADE2	NGEF
GYG2	SLC7A14	CYB561	XK	MPPED2
RASGRF1	CNTN1	NFIX	CCDC85A	KIF26A
CAMK2B	PLEKHB1	SLC6A7	F7	ATP2B3
PTPRU	MRI1	SYT7	CAMK2B	RASGRP2
SLC9A3R2	CAPG	SLC7A14	SLC9A3R2	TRPC5
TLE2	JADE2	CNTN1	TLE2	PPP2R2C
MPPED2	PTPRN	PLEKHB1	KIF26A	SNCB
KIF26A	GYG2	CDH10	ATP2B3	NUAK1
WSCD2	RASGRF1	RAB27B	RASGRP2	ACHE
FOSL2	PTPRU	CAPG	SPTB	COL9A3
CARMIL1	SLC9A3R2	TG	TRPC5	CRTAC1
CDH7	TLE2	JADE2	PPP2R2C	HCN2
PITPNM2	PDE4A	XK	SNCB	SUSD2
SEL1L3	NGEF	PTPRN	CACNG5	SEZ6L
CRTAC1	KIF26A	CCDC85A	RAPGEF3	MMP9
PALMD	RASGRP2	GYG2	MID2	NTSR1
HCN2	SPTB	F7	STK17B	MAP1LC3A
MMP11	CAMK2A	CAMK2B	P2RX5	ST8SIA5
SEZ6L	TRPC5	DGKG	COL16A1	NALCN
CACNA1I	TRHDE	RIMBP2	ACHE	SYT17
[illegible]	[illegible]	[illegible]	[illegible]	[illegible]

INPP5J
GALNT17
GPRIN3
SV2B
FAM131C
AHNAK2
C11orf87
ADAMTSL5
GABRA5
NAP1L3
CYP4X1
PDE2A
MPPED1
VSTM2B
SHTN1
C11orf96
ATP13A5
C17orf97
COL4A5
SMTNL2
ENO4
SLC38A3
COL25A1
CCDC9B
NKAIN2
PAQR9
VWC2
SHISA6
BEND4
FAM78B
INSYN2A
RELN
PTPRT
SPOCK3
CPNE4
FUT4
LAMB3
SLC6A17
ADGRA1
DCHS2
OGDHL
PDGFA
DPP4
S100A10
H4C16
ADAMTSL2
CYRIA
S100A6
MBP
DLGAP2
CHSY3
LPAR1
TMEM229B
CACNA1E
RYR2
LRRTM3
BRINP2
CCDC152
RASGEF1A
STUM
RIPPLY2
FOXD6
TCEAL6
MAFB
COL15A1
DENND1C
C22orf42
CPLX3
S1PR3
C10orf105
UBE2QL1
HMX1
TSTD1
SP9
PLXNA4
RAB6C
FNDC10
AIM1
RAB6D
TMEM238
CLEC2L
SHISA9
OR2W3
PSMB9
SMKR1
CEBPA
TRNP1
CTSO
SALL3
TMEM179
FRRS1L
GOLGA6L7
ZIM2
SRXN1
DOC2B
GRIN2B

FAM78B
INSYN2A
RELN
IL1RAPL2
MAOA
PTPRT
SPOCK3
GRM7
SLC30A10
LAMB3
FAM163B
SLC6A17
ADGRA1
DCHS2
PDGFA
DPP4
S100A10
CYRIA
S100A6
MBP
LPAR1
CACNA1E
RYR2
LRRTM3
BRINP2
RASGEF1A
STUM
FOXD6
TCEAL5
TCEAL6
MAFB
C2orf72
COL15A1
DENND1C
C22orf42
DENND1B
FAM24B
CPLX3
S1PR3
C10orf105
UBE2QL1
HMX1
TSTD1
SP9
PLXNA4
CENPVL1
FNDC10
AIM1
CLEC2L
SHISA9
PSMB9
SMKR1
CEBPA
TRNP1
SALL3
ZNF578
TMEM179
FRRS1L
GOLGA6L7
ZIM2
SRXN1
DOC2B
GRIN2B

TF	TF	TF	TF	TF	TF	TF	TF	TF	TF	TF	TF	TF	TF	TF	TF	TF	TF	TF	TF	TF	TF
TF:M04516	TF:M07329_1	TF:M09892_1	TF:M02105	TF:M10435	TF:M09602	TF:M04146	TF:M00243	TF:M07361	TF:M11066	TF:M00706	TF:M08662	TF:M08911_1	TF:M01858_1	TF:M11155	TF:M03565	TF:M00915	TF:M08440	TF:M01073_1	TF:M12732	TF:M12692	TF:M00196
Factor:_E2F-1;_motif:_TTTGGCGCCAAA	Factor:_Osx;_motif:_CCNCCCCCNNN;_match_class:_1	Factor:_E2F-1;_motif:_NNNNGGCGGGAARN;_match_class:_1	Factor:_NF-E4;_motif:_CHCCCTCKCCWG	Factor:_Sp2;_motif:_GGGGCGGGG	Factor:_ctcf;_motif:_NNNCCASYAGRKGGCRSYNN	Factor:_TFAP2A;_motif:_YGCCCNNRGGCN	Factor:_Egr-1;_motif:_WTGCGTGGGCGK	Factor:_MZF-1;_motif:_GKGGGGARNR	Factor:_HES-7;_motif:_GNCACGYGNN	Factor:_TFII-I;_motif:_RGAGGKAGG	Factor:_AP-2gamma:HES-7;_motif:_NNCACGYGNNNNNNSCCNNNGGS	Factor:_CTCF;_motif:_NCCRSTAGGGGGCGC;_match_class:_1	Factor:_AP-2beta;_motif:_GCNNNGGSCNGVGGGN;_match_class:_1	Factor:_ITF-2;_motif:_NCACCTGN	Factor:_slug;_motif:_KCACCTGN	Factor:_AP-2;_motif:_SNNNCCNCAGGCN	Factor:_AP-2gamma:E2F-8;_motif:_NTTCCCGCNNSCCCSMGGC	Factor:_HIC1;_motif:_NNNGGKTGCCCSNNNNNN;_match_class:_1	Factor:_ZNF532;_motif:_NGGRGCTGGG	Factor:_ZBTB49;_motif:_NGSCGCTGTCCNNGGTGCTGAN	Factor:_Sp1;_motif:_NGGGGGCGGGGYN
496	143	414	148	357	345	309	263	82	496	275	116	204	169	161	161	428	80	57	411	123	452
HS3ST1	HS3ST1	HS3ST1	GAS7	PLXND1	HS3ST1	PLXND1	PLXND1	CEACAM21	HS3ST1	PLXND1	MATK	PLXND1	ITGA3	ABCB5	ABCB5	HS3ST1	HS3ST1	PLXND1	HS3ST1	PLXND1	HS3ST1
PLXND1	PLXND1	PLXND1	CAPG	ITGA3	PLXND1	YBX2	ITGA3	SLC6A7	PLXND1	ITGA3	CYB561	YBX2	YBX2	YBX2	YBX2	PLXND1	PLXND1	ITGA3	ABCB5	ABCB5	PLXND1
YBX2	YBX2	ITGA3	JADE2	YBX2	ITGA3	PNPLA4	TAC1	PLEKHB1	ITGA3	CEACAM21	SLC6A7	SYT7	SYT7	CACNG3	CACNG3	ITGA3	GAS7	GAS7	ITGA3	CACNG3	ITGA3
CACNG3	GAS7	YBX2	RASGRF1	CACNG3	YBX2	GAS7	PNPLA4	TG	YBX2	CAMK1G	FSTL4	CAPG	JADE2	CYB561	CYB561	YBX2	MATK	JADE2	YBX2	CEACAM21	YBX2
TAC1	SYT7	PNPLA4	SLC9A3R2	TAC1	CYB561	PAX6	GAS7	PTPRN	CACNG3	CYB561	CAMK2B	JADE2	FSTL4	CNTN1	CNTN1	GAS7	SYT7	CCDC85A	TAC1	CYB561	CACNG3
[illegible]	[illegible]	[illegible]	[illegible]	[illegible]	[illegible]	[illegible]	[illegible]	[illegible]	[illegible]	[illegible]	[illegible]	[illegible]	[illegible]	[illegible]	[illegible]	[illegible]	[illegible]	[illegible]	[illegible]	[illegible]	[illegible]

FRRS1L
GOLGA6L7
ZIM2
SRXN1
DOC2B
GRIN2B

FRRS1L
GOLGA6L7
ZIM2
SRXN1
DOC2B
GRIN2B

TF	TF	TF	TF	TF	TF	TF	TF	TF	TF
TF:M11533_1	TF:M07297	TF:M05327_1	TF:M01733_1	TF:M08441_1	TF:M09972	TF:M00693	TF:M01837	TF:M10477_1	TF:M03893_1
Factor:_E2F-1;_motif:_NTTTTGGCGCCAAWWWN;_match_class:_1	Factor:_MAZ;_motif:_CCCTCCCYCYN	Factor:_WT1;_motif:_NGCGGGGGGGTSMMCYN;_match_class:_1	Factor:_MZF-1;_motif:_TGGGGAR;_match_class:_1	Factor:_AP-2gamma:Elk-1;_motif:_NGCCKNRGGSGRCGGAAGTG;_match_class:_1	Factor:_BTEB2;_motif:_WGGGTGKGGCNGGN	Factor:_E12;_motif:_RRCAGGTGNCV	Factor:_FKLF;_motif:_BGGGNGGVMD	Factor:_ZNF436;_motif:_TCCTCCAGGAAGCCY;_match_class:_1	Factor:_WT1;_motif:_GNGGGGGCGGGG;_match_class:_1
394	160	107	242	540	411	141	264	399	204
[illegible]	[illegible]	[illegible]	[illegible]	[illegible]	[illegible]	[illegible]	[illegible]	[illegible]	[illegible]

TF	TF	TF	TF	TF	TF	TF	TF	TF	TF
TF:M12691	TF:M04146_1	TF:M12535	TF:M12345	TF:M09894_1	TF:M07322	TF:M01837_1	TF:M11529	TF:M07041_1	TF:M09729
Factor:_ZBTB44;_motif:_NTCAGCACCNNGGACAGCGSCN	Factor:_TFAP2A;_motif:_YGCCCNNRGGCN;_match_class:_1	Factor:_ZNF263;_motif:_NNGGGGAGGANN	Factor:_Zbtb37;_motif:_NYACCGCRNTCACCGCR	Factor:_E2F-4;_motif:_SNGGGCGGGAANN;_match_class:_1	Factor:_HSF4;_motif:_CTGCMRN	Factor:_FKLF;_motif:_BGGGNGGVMD;_match_class:_1	Factor:_E2F-2;_motif:_GCGCGCGCNCS	Factor:_HDAC1;_motif:_KGCARGGTC;_match_class:_1	Factor:_LUMAN;_motif:_CYCAGCYYCY
89	194	151	273	583	665	90	658	296	463
[illegible]	[illegible]	[illegible]	[illegible]	[illegible]	[illegible]	[illegible]	[illegible]	[illegible]	[illegible]

DPP4
S100A10
ADAMTSL2
CYRIA
S100A6
MBP
CHSY3
LPAR1
TMEM229B
RYR2
CCDC152
RASGEF1A
STUM
RIPPLY2
FOXO6
TCEAL5
TCEAL6
MAFB
C2orf72
COL15A1
DENND1B
FAM24B
EML6
UBE2QL1
HMX1
TSTD1
SP9
PLXNA4
RAB6C
CENPVL1
CENPVL3
FNDC10
AIM1
RAB6D
TMEM238
SHISA9
OR2W3
PSMB9
SMKR1
CEBPA
SELENOP
TRNP1
CTSO
SALL3
TMEM179
FRRS1L
GOLGA6L7
SRXN1
DOC2B
GRIN2B

C11orf87
KCNQ5
ADAMTSL5
GABRA5
CYP4X1
PDE2A
MPPED1
MAPT
VSTM2B
SHTN1
GCNT1
C11orf96
C17orf97
NHS
SMTNL2
ENO4
SLC38A3
COL25A1
NKAIN2
PAQR9
VWC2
SHISA6
BEND4
FAM78B
INSYN2A
RELN
IL1RAPL2
MAOA
PTPRT
GRM7
FUT4
SLC30A10
LAMB3
FAM163B
SLC6A17
ADGRA1
DCHS2
OGDHL
PDGFA
S100A10
H4C16
ADAMTSL2
CYRIA
S100A6
MBP
CHSY3
LPAR1
TMEM229B
CACNA1E
RYR2
BRINP2
CCDC152
RASGEF1A
STUM
RIPPLY2
FOXO6
TCEAL5
TCEAL6
MAFB
C2orf72
COL15A1
DPF3
DENND1C
DENND1B
FAM24B
CPLX3
S1PR3
EML6
UBE2QL1
HMX1
TSTD1
SP9
PLXNA4
CENPVL1
CENPVL3
FNDC10
AIM1
TMEM238
CLEC2L
SHISA9
PSMB9
SMKR1
CEBPA
TRNP1
SALL3
ZNF578
TMEM179
FRRS1L
GOLGA6L7
ZIM2
SRXN1
DOC2B
GRIN2B

NPPA
GAL3ST3
PCSK1
CREG2
ARL4D
LY6H
RIMKLA
KCNA3
FZD8
ST8SIA3
KCNJ10
ZNF354C
ALS2CL
AMER3
KCTD12
RPP25
CPNE7
FAM133A
ALOXE3
TMEM151A
FJX1
SLITRK4
LACC1
AKAP5
ZNF154
TSHZ1
LYNX1
PLD5
TSPYL5
GSX2
SSTR2
SHISA2
FAM83H
ZNF572
RPH3AL
CHRM2
TMEM132C
TMEM132E
GPR88
GPR3
ADGRB1
MGAT4C
FAM181B
KCNK4
RGS6
GALNT9
BEGAIN
GPC6
FAM43B
CALN1
CTNNA3
PCBP3
MARCHF11
EFHC2
NOG
OPCML
ADRA2C
KCNJ12
ACOT1
PRKD1
PABPC1L2B
SLITRK6
CLDN6
TCEAL2
FMNL1
ROBO2
SLC24A3
INPP5J
GALNT17
SV2B
FAM131C
AHNAK2
C11orf87
KCNQ5
ADAMTSL5
GABRA5
NAP1L3
CYP4X1
PDE2A
MPPED1
MAPT
SHTN1
GCNT1
C11orf96
ATP13A5
C17orf97
COL4A5
NHS
SMTNL2
ENO4
SLC38A3
CCDC9B
NKAIN2
PAQR9
VWC2
SHISA6
BEND4
FAM78B
INSYN2A
RELN
IL1RAPL2
MAOA
PTPRT
SPOCK3
GRM7
CPNE4
FUT4
SLC30A10
LAMB3
FAM163B
SLC6A17
ADGRA1
DCHS2
OGDHL
PDGFA
COL4A6
DPP4
S100A10
H4C16
ADAMTSL2
CYRIA
S100A6
MBP
DLGAP2
CHSY3
LPAR1
TMEM229B
CACNA1E
RYR2
BRINP2
CCDC152
RASGEF1A
STUM
RIPPLY2
FOXO6
TCEAL5
TCEAL6
MAFB
C2orf72
COL15A1
DPF3
DENND1C
C22orf42
DENND1B
FAM24B
S1PR3
EML6
C10orf105
UBE2QL1
HMX1
TSTD1
SP9
PLXNA4
RAB6C
CENPVL1
CENPVL3
FNDC10
AIM1
RAB6D
TMEM238
CLEC2L
SHISA9
OR2W3
PSMB9
SMKR1
CEBPA
SELENOP
TRNP1
CTSO
TMEM179
FRRS1L
GOLGA6L7
ZIM2
SRXN1
DOC2B

DSCAML1
RIMKLA
KCNA3
FZD8
ST8SIA3
KCNJ10
ZNF354C
ALS2CL
AMER3
KCTD12
RPP25
CPNE7
FAM133A
ALOXE3
TMEM151A
FJX1
SLITRK4
LACC1
AKAP5
ZNF154
TSHZ1
LYNX1
PLD5
TSPYL5
GSX2
SSTR2
SHISA2
FAM83H
ZNF572
RPH3AL
CHRM2
PENK
TMEM132C
TMEM132E
DDX60L
GPR88
GPR3
ADGRB1
FAM181B
KCNK4
RGS6
GALNT9
BEGAIN
GPC6
FAM43B
CALN1
CTNNA3
PCBP3
MARCHF11
EFHC2
NOG
OPCML
ADRA2C
KCNJ12
ACOT1
PRKD1
MAP7D2
PABPC1L2B
CLDN6
TCEAL2
FMNL1
ROBO2
SLC24A3
GALNT17
SV2B
FAM131C
AHNAK2
C11orf87
KCNQ5
ADAMTSL5
GABRA5
NAP1L3
CYP4X1
PDE2A
MPPED1
VSTM2B
SHTN1
GCNT1
C11orf96
C17orf97
COL4A5
NHS
SMTNL2
ENO4
SLC38A3
COL25A1
NKAIN2
PAQR9
VWC2
SHISA6
BEND4
FAM78B
INSYN2A
RELN
IL1RAPL2
MAOA
PTPRT
SPOCK3
GRM7
FUT4
SLC30A10
LAMB3
FAM163B
SLC6A17
ADGRA1
DCHS2
OGDHL
PDGFA
DPP4
H4C16
ADAMTSL2
CYRIA
S100A6
MBP
CHSY3
LPAR1
TMEM229B
CACNA1E
RYR2
LRRTM3
BRINP2
CCDC152
RASGEF1A
STUM
RIPPLY2
FOXO6
TCEAL5
TCEAL6
MAFB
C2orf72
COL15A1
DPF3
DENND1C
C22orf42
DENND1B
FAM24B
CPLX3
S1PR3
EML6
UBE2QL1
HMX1
TSTD1
SP9
PLXNA4
RAB6C
CENPVL1
CENPVL3
FNDC10
AIM1
RAB6D
TMEM238
CLEC2L
SHISA9
OR2W3
PSMB9
SMKR1
CEBPA
SELENOP
TRNP1
CTSO
SALL3
TMEM179
FRRS1L
GOLGA6L7
ZIM2
SRXN1
DOC2B
GRIN2B

TF	TF	TF	TF	TF	WP	WP	WP
TF:M04797_1	TF:M04934_1	TF:M12057	TF:M00915_1	TF:M11595	WP:WP536	WP:WP4222	WP:WP4159
Factor:_Egr-1;_motif:_CCGCCCMCG;_match_class:_1	Factor:_TR4;_motif:_ACCCCGS;_match_class:_1	Factor:_ZBP99;_motif:_GCCCMTCCCCCR	Factor:_AP-2;_motif:_SNNNCCNCAGGCN;_match_class:_1	Factor:_GCMa;_motif:_RTGCGGGTN	Calcium_regulation_in_cardiac_cells	Phosphodiesterases_in_neuronal_function	GABA_receptor_signaling
96	630	253	226	514	22	12	9
PLXND1	HS3ST1	ITGA3	HS3ST1	HS3ST1	CAMK2B	PDE4A	GABRA3
YBX2	PLXND1	YBX2	PLXND1	PLXND1	ATP2B3	CAMK2A	GABRG2
CYB561	ITGA3	CACNG3	YBX2	ITGA3	CAMK2A	ADCY2	SLC6A11
TNC	YBX2	CYB561	MATK	YBX2	ADCY2	NOS1	GABBR2
CAMK2B	CACNG3	NFIX	CYB561	TAC1	KCNJ5	PPP1R1B	GABRA6
SLC9A3R2	TAC1	SYT7	SLC6A7	ETV1	CHRM3	GUCY1A2	GABRB2
PDE4A	ETV1	PLEKHB1	JADE2	PNPLA4	PKIB	PDE1C	GABRA2
NGEF	PNPLA4	CDH10	XK	CEACAM21	PRKACB	GRIA1	GABBR1
MPPED2	CEACAM21	CAPG	CCDC85A	GAS7	CASQ1	ADCY8	GABRA5
RASGRP2	GAS7	JADE2	GYG2	MATK	ADCY8	GUCY1A1	
PPP2R2C	MATK	PTPRN	CAMK2B	CAMK1G	ATP2B2	PDE2A	
NUAK1	PAX6	GYG2	RIMBP2	CYB561	CACNA1D	GRIN2B	
SEMA3C	CAMK1G	F7	SLC9A3R2	NFIX	GJD2		
MID2	CYB561	RASGRF1	TLE2	SLC6A7	KCNJ3		
ACHE	NFIX	DGKG	NGEF	SYT7	PRKCB		
PITPNM2	SLC6A7	PTPRU	MPPED2	SLC7A14	GNG8		
SEL1L3	SYT7	NGEF	ATP2B3	CNTN1	ADRA1B		
HCN2	SLC7A14	MPPED2	RASGRP2	MRI1	CHRM2		
PDGFB	CNTN1	KIF26A	SPTB	TNC	RGS6		
RSPO4	PLEKHB1	ATP2B3	CNGB1	JADE2	PRKD1		
ST8SIA5	MRI1	ACSL4	TRHDE	LMO3	CACNA1E		
JPH1	TNC	RASGRP2	PPP2R2C	FSTL4	RYR2		
SH3GL2	CAPG	CNGB1	SNCB	PTPRN			
NEURL1	TG	PPP2R2C	NUAK1	CCDC85A			
PTPN5	JADE2	SNCB	SEMA3C	GYG2			
SEMA5A	XK	NUAK1	FOSL2	F7			
SLC9A2	LMO3	CACNG5	NEBL	RASGRF1			
CHD5	FSTL4	DCX	BRINP1	CAMK2B			
DLGAP3	PTPRN	NEBL	CDH7	DGKG			
KLHL29	CCDC85A	BRINP1	STK17B	PTPRU			
EGR1	GYG2	STK17B	TTC39A	RIMBP2			
PHF24	F7	P2RX5	NOS1	LZTS1			
TOX2	RASGRF1	COL9A3	PITPNM2	SLC9A3R2			
RASL11B	CAMK2B	CRTAC1	SEL1L3	PDE4A			
RFPL2	DGKG	HCN2	CRTAC1	NGEF			
GCH1	PTPRU	SUSD2	HCN2	MPPED2			
PPARG	RIMBP2	CHGA	SEZ6L	KIF26A			
ATP8A2	LZTS1	MAP1LC3A	PDGFB	ATP2B3			
GPR12	SLC9A3R2	ST8SIA5	ISM2	RASGRP2			
CMPK2	TLE2	CHRDL1	RSPO4	SPTB			
ADAMTS8	PDE4A	NALCN	SAMHD1	CAMK2A			
TMC6	NGEF	SYT17	PPP1R16B	TRPC5			
CACNG8	MPPED2	RHOV	NALCN	TRHDE			
LANCL3	KIF26A	TUBB4A	SALL1	PPP2R2C			
ARHGAP36	ATP2B3	AMH	SYT17	SNCB			
MAL2	ACSL4	CORO2A	TUBB4A	MGLL			
SLC2A4	RASGRP2	CDH23	SLC17A7	NUAK1			
JAKMIP1	SPTB	VSIR	CRHR2	WSCD2			
CCSAP	CNGB1	SFXN3	LHX2	FOSL2			
ADCY8	CAMK2A	NEURL1	SH3GL2	CACNG5			
SORCS3	TRPC5	PTPN5	SHB	NEBL			
ACE	TRHDE	TFEB	CDH23	ADCY2			
[illegible]	[illegible]	[illegible]	[illegible]	[illegible]			

LYNX1
PLD5
TSPYL5
GSX2
SSTR2
SHISA2
FAM83H
ZNF572
RPH3AL
CHRM2
PENK
TMEM132C
TMEM132E
DDX60L
GPR3
ADGRB1
FAM181B
KCNK4
GALNT9
BEGAIN
FAM43B
CALN1
CTNNA3
PCBP3
MARCHF11
EFHC2
NOG
OPCML
ADRA2C
KCNJ12
ACOT1
PRKD1
MAP7D2
PABPC1L2B
CLDN6
TCEAL2
FMNL1
ROBO2
SLC24A3
INPP5J
GALNT17
SV2B
FAM131C
AHNAK2
C11orf87
KCNQ5
ADAMTSL5
GABRA5
PDE2A
MPPED1
MAPT
VSTM2B
GCNT1
C11orf96
C17orf97
COL4A5
NHS
SMTNL2
ENO4
SLC38A3
COL25A1
CCDC9B
NKAIN2
PAQR9
SHISA6
BEND4
FAM78B
INSYN2A
MAOA
PTPRT
SPOCK3
GRM7
CPNE4
FUT4
SLC30A10
LAMB3
FAM163B
SLC6A17
ADGRA1
OGDHL
PDGFA
DPP4
S100A10
HIC1G
ADAMTSL2
CYRIA
S100A6
MBP
DLGAP2
CHSY3
LPAR1
TMEM229B
CACNA1E
RYR2
LRRTM3
BRINP2
CCDC152
RASGEF1A
STUM
RIPPLY2
FOXD6
TCEAL5
TCEAL6
MAFB
C2orf72
COL15A1
DENND1C
C22orf42
FAM24B
CPLX3
S1PR3
EML6
C10orf105
UBE2QL1
HMX1
TSTD1
SP9
PLXNA4
CENPVL1
CENPVL3
FNDC10
AJM1
TMEM238
CLEC2L
SHISA9
OR2W3
PSMB9
SMKR1
CEBPA
TRNP1
CTSO
SALL3
ZNF578
TMEM179
FRRS1L
GOLGA6L7
ZIM2
SRXN1
DOC2B
GRIN2B

EML6
C10orf105
UBE2QL1
HMX1
TSTD1
SP9
CENPVL1
CENPVL3
FNDC10
AJM1
TMEM238
CLEC2L
SHISA9
OR2W3
PSMB9
CEBPA
CTSO
SALL3
ZNF578
TMEM179
FRRS1L
GOLGA6L7
ZIM2
DOC2B

Fig4D GO_upregulated_gene

source	CORUM
term_id	CORUM:7564
term_name	LGR5-RNF43-RSPO1_complex
intersection_size	3
intersection	RNF43
	LGR5
	RSPO1

source	GO:BP
term_id	GO:0048731
term_name	system_development
intersection_size	150
intersection	CYP26B1
	ABCC8
	PROM1
	TEAD3
	TFAP2B
	CD9
	WWTR1
	ATP1A2
	LAMC3
	FGFR3
	TGFBR3
	WNT8B
	PLXNA2
	THOC1
	CEACAM1
	LRP2
	NOX4
	LHX5
	DLL3
	ANGPT2
	TMEM38B
	CYP26A1
	PCSK5
	HMOX1
	SALL4
	JAG1
	SYNDIG1
	RGCC
	CDK6
	ITGB8
	LFNG
	TSPAN12
	WNT3
	CPT1A
	RFX4
	PRDM13
	SMOC2
	MAN2A1
	WNT5A
	CBLB
	WLS
	ID3
	PROX1
	CCN2
	TNN
	ADRA1A
	KCNJ8
	CXCR4
	NEUROD4
	USP45
	CDKN1A
	PODXL
	FOXJ1
	CASZ1
	NES
	CNTN6
	PDGFRA
	CEMIP2
	PAX3
	EDNRB
	ENPP2
	TFAP2A
	THBS1
	SMAD6
	BMPR1B
	FBN2
	LGR5
	ASCL1
	HAPLN3
	MFGE8
	GREB1L
	EPHA2
	SYPL2
	SOX13
	SYT2
	MEIS1
	ACKR3
	HES6
	BOC
	EPHA5
	TPBG
	NTRK2
	CRB2
	KLHL1
	THRB
	POU4F2
	POU4F1
	SEMA3D
	ROBO3
	ANGPT1
	PDLIM3
	ZIC3
	SHROOM4
	SLC45A3
	ADAMTS4
	WNT9B
	NPHS1
	ITGA5
	PDPN
	DHRS3
	LMX1A
	EN1
	MSX1
	ADGRV1
	TLX3
	EN2
	UNCX
	PHOX2A
	RBPMS2
	TMIGD2
	GBX2
	THBS3
	GSX1
	P2RY1
	IRX1
	PKHD1
	FOXB1
	MBOAT1
	NDNF
	FOSL1
	NR2F1
	IRX5
	OLIG3
	IRX3
	NHLH2
	ADAMTSL1
	ZHX2
	GPR157
	UNC5C
	COL18A1
	POU3F2
	ROR1
	NR2F2
	POU3F1
	SLITRK2
	RTN4RL2
	COL4A1
	WNT7B
	POTEE
	HES4
	ZP3
	PCDH18
	HES5
	NTRK1
	NRARP
	DLL1
	RBM20
	LGR4
	SKOR2
	MUSTN1

source	GO:BP
term_id	GO:0007275
term_name	multicellular_organism_development
intersection_size	158
intersection	CYP26B1
	ABCC8
	PROM1
	TEAD3
	TFAP2B
	PGLYRP1
	CD9
	WWTR1
	ATP1A2
	LAMC3
	FGFR3
	TGFBR3
	WNT8B
	PLXNA2
	THOC1
	CEACAM1
	LRP2
	NOX4
	TESC
	LHX5
	DLL3
	ANGPT2
	TMEM38B
	TLL2
	CYP26A1
	PCSK5
	HMOX1
	SALL4
	JAG1
	SYNDIG1
	RGCC
	CDK6
	ITGB8
	LFNG
	TSPAN12
	WNT3
	CPT1A
	RFX4
	PRDM13
	SMOC2
	MAN2A1
	WNT5A
	CBLB
	WLS
	ID3
	PROX1
	CCN2
	TNN
	ADRA1A
	KCNJ8
	CXCR4
	NEUROD4
	USP45
	CDKN1A
	PODXL
	FOXJ1
	CASZ1
	NES
	CNTN6
	PDGFRA
	CEMIP2
	PAX3
	PCDH8
	EDNRB
	ENPP2
	TFAP2A
	THBS1
	SMAD6
	BMPR1B
	FBN2
	LGR5
	ASCL1
	LPAR6
	HAPLN3
	MFGE8
	GREB1L
	EPHA2
	SYPL2
	SOX13
	SYT2
	MEIS1
	ACKR3
	HES6
	BOC
	EPHA5
	TPBG
	NTRK2
	CRB2
	KLHL1
	THRB
	POU4F2
	POU4F1
	SEMA3D
	ROBO3
	ANGPT1
	PDLIM3
	ZIC3
	SHROOM4
	SLC45A3
	ADAMTS4
	WNT9B
	NPHS1
	ITGA5
	PDPN
	DHRS3
	LMX1A
	EN1
	MSX1
	ADGRV1
	TLX3
	EN2
	UNCX
	PHOX2A
	RBPMS2
	TMIGD2
	GBX2
	THBS3
	GSX1
	P2RY1
	IRX1
	PKHD1
	FOXB1
	MBOAT1
	NDNF
	FOSL1
	NR2F1
	IRX5
	OLIG3
	IRX3
	NHLH2
	ADAMTSL1
	ZHX2
	GPR157
	UNC5C
	COL18A1
	POU3F2
	ROR1
	NR2F2
	POU3F1
	SLITRK2
	RTN4RL2
	COL4A1
	ISG15
	WNT7B
	POTEE
	HES4
	ZP3
	PCDH18
	H4C3
	ENPP1
	HES5
	NTRK1
	NRARP
	DLL1
	RBM20
	LGR4
	SKOR2
	MUSTN1

source	GO:BP
term_id	GO:0048513
term_name	animal_organ_development
intersection_size	125
intersection	CYP26B1
	PROM1
	TEAD3
	TFAP2B
	PAX7
	WWTR1
	ATP1A2
	LAMC3
	FGFR3
	TGFBR3
	PLXNA2
	CEACAM1
	LRP2
	NOX4
	LHX5
	DLL3
	ANGPT2
	TMEM38B
	TLL2
	CYP26A1
	PCSK5
	HMOX1
	SALL4
	JAG1
	RGCC
	CDK6
	ITGB8
	LFNG
	TSPAN12
	WNT3
	CPT1A
	RFX4
	PRDM13
	MAN2A1
	WNT5A
	WLS
	ID3
	PROX1
	CCN2
	ESRRB
	TNN
	ADRA1A
	KCNJ8
	CXCR4
	NEUROD4
	USP45
	CDKN1A
	PODXL
	FOXJ1
	NES
	PDGFRA
	PAX3
	EDNRB
	TFAP2A
	SMAD6
	BMPR1B
	FBN2
	LGR5
	ASCL1
	GREB1L
	EPHA2
	SYPL2
	ILDR2
	SOX13
	MEIS1
	EPHA5
	MEGF10
	NTRK2
	CRB2
	KLHL1
	THRB
	POU4F2
	POU4F1
	SEMA3D
	ANGPT1
	PDLIM3
	ZIC3
	SHROOM4
	WNT9B
	NPHS1
	PDPN
	DHRS3
	LMX1A
	EN1
	MSX1
	ADGRV1
	TLX3
	EN2
	UNCX
	PHOX2A
	RBPMS2
	GBX2
	THBS3
	GSX1
	MGMT
	IRX1
	PKHD1
	FOXB1
	NDNF
	FOSL1
	IRX5
	OLIG3
	IRX3
	NHLH2
	ZHX2
	UNC5C
	COL18A1
	POU3F2
	ROR1
	NR2F2
	POU3F1
	COL4A1
	ISG15
	WNT7B
	POTEE
	ZP3
	PCDH18
	ENPP1
	HES5
	NTRK1
	DLL1
	RBM20
	LGR4
	SKOR2
	MUSTN1

source	GO:BP
term_id	GO:0009653
term_name	anatomical_structure_morphogenesis
intersection_size	115
intersection	CYP26B1
	ABCC8
	PROM1
	TFAP2B
	PAX7
	CD9
	WWTR1
	LAMC3
	FGFR3
	TGFBR3
	WNT8B
	PLXNA2
	CEACAM1
	LRP2
	NOX4
	LHX5
	DLL3
	ANGPT2
	PCSK5
	HMOX1
	SALL4
	JAG1
	RGCC
	ITGB8
	LFNG
	TSPAN12
	WNT3
	SMOC2
	MAN2A1
	WNT5A
	WLS
	ID3
	PROX1
	CCN2
	TNN
	CRISPLD1
	CXCR4
	PODXL
	AJUBA
	FOXJ1
	GDF15
	NES
	CNTN6
	PDGFRA
	CEMIP2
	PAX3
	PCDH8
	ENPP2
	TFAP2A
	THBS1
	SMAD6
	BMPR1B
	FBN2
	LGR5
	MFGE8
	GREB1L
	EPHA2
	MAEL
	SOX13
	SYT2
	MEIS1
	ACKR3
	BOC
	EPHA5
	TPBG
	NTRK2
	CRB2
	THRB
	POU4F2
	POU4F1
	SEMA3D
	ROBO3
	ANGPT1
	PGM5
	ZIC3
	WNT9B
	NPHS1
	ITGA5
	PDPN
	DHRS3
	OMA1
	LMX1A
	EN1
	MSX1
	UNCX
	PHOX2A
	RBPMS2
	TMIGD2
	GBX2
	THBS3
	P2RY1
	IRX1
	PKHD1
	FOXB1
	NDNF
	IRX5
	IRX3
	ADAMTSL1
	ZHX2
	UNC5C
	COL18A1
	POU3F2
	ROR1
	NR2F2
	SLITRK2
	COL4A1
	WNT7B
	ZP3
	HES5
	NTRK1
	NRARP
	DLL1
	RBM20
	LGR4
	SKOR2

source	GO:BP
term_id	GO:0032501
term_name	multicellular_organismal_process
intersection_size	206
intersection	CYP26B1
	ABCC8
	PROM1
	TEAD3
	TFAP2B
	PGLYRP1
	CD9
	WWTR1
	ATP1A2
	ABCC2
	FAS
	LTBP1
	LAMC3
	PTGER3
	FGFR3
	ABCC9
	TGFBR3
	LMCD1
	WNT8B
	PLXNA2
	THOC1
	CDC14A
	CEACAM1
	LRP2
	OPRK1
	CHMP2B
	NOX4
	TESC
	LHX5
	DLL3
	ANGPT2
	TMEM38B
	TLL2
	CYP26A1
	PCSK5
	HMOX1
	KCNK10
	SALL4
	JAG1
	SYNDIG1
	RGCC
	CDK6
	ITGB8
	LFNG
	TSPAN12
	WNT3
	P2RX3
	CPT1A
	RFX4
	PRDM13
	SMOC2
	MAN2A1
	WNT5A
	CBLB
	PLA2G4A
	WLS
	ID3
	PROX1
	CCN2
	ESRRB
	TNN
	ADRA1A
	CRISPLD1
	SCPEP1
	KCNJ8
	CXCR4
	NEUROD4
	PDE1B
	USP45
	KCNJ2
	CDKN1A
	CD70
	HSPA2
	TSPAN8
	PODXL
	FOXJ1
	GDF15
	HRC
	SLC6A8
	CASZ1
	NES
	PRAM1
	CNTN6
	PDGFRA
	CEMIP2
	PAX3
	PCDH8
	EDNRB
	ENPP2
	TFAP2A
	THBS1
	SMAD6
	BMPR1B
	FBN2
	LGR5
	ASCL1
	LPAR6
	HAPLN3
	MFGE8
	TAF4B
	GREB1L
	EPHA2
	SYPL2
	MAEL
	ILDR2
	RGS16
	HMCN1
	CREB3L4
	SOX13
	SYT2
	MEIS1
	ACKR3
	HES6
	BOC
	EPHA5
	MEGF10
	GLRA1
	TPBG
	NTRK2
	CRB2
	KLHL1
	THRB
	POU4F2
	POU4F1
	GRM1
	SEMA3D
	ROBO3
	ANGPT1
	PDLIM3
	PIEZO2
	ZIC3
	LDLRAP1
	SHROOM4
	SLC45A3
	ADAMTS4
	WNT9B
	NPHS1
	ITGA5
	PDPN
	DHRS3
	OMA1
	LMX1A
	EN1
	MSX1
	DAPL1
	INAVA
	ADGRV1
	TLX3
	EN2
	UNCX
	PHOX2A
	RBPMS2
	TAC3
	TMIGD2
	GBX2
	THBS3
	GSX1
	P2RY1
	IRX1
	PKHD1
	NETO2
	FOXB1
	SLC9C1
	MBOAT1
	NDNF
	IGDCC3
	FGFBP3
	FOSL1
	NR2F1
	IRX5
	OLIG3
	IRX3
	NHLH2
	IL17RA
	ADAMTSL1
	ZHX2
	GPR157
	UNC5C
	COL18A1
	POU3F2
	ROR1
	NR2F2
	POU3F1
	SLITRK2
	RTN4RL2
	COL4A1
	ISG15
	WNT7B
	POTEE
	HES4
	ZP3
	LITAF
	PCDH18
	POTEI
	H4C3
	ENPP1
	HES5
	NTRK1
	NRARP
	DLL1
	FCGR3A
	RBM20
	MICB
	LGR4
	SKOR2
	MUSTN1

source	GO:BP
term_id	GO:0048856
term_name	anatomical_structure_development
intersection_size	175
intersection	CYP26B1
	ABCC8
	PROM1
	TEAD3
	TFAP2B
	PGLYRP1
	PAX7
	CD9
	WWTR1
	ATP1A2
	LAMC3
	CNN2
	FGFR3
	TGFBR3
	WNT8B
	PLXNA2
	THOC1
	CEACAM1
	LRP2
	NOX4
	TESC
	LHX5
	DLL3
	ANGPT2
	TMEM38B
	TLL2
	CYP26A1
	PCSK5
	HMOX1
	SALL4
	JAG1
	SYNDIG1
	RGCC
	CDK6
	ITGB8
	LFNG
	TSPAN12
	WNT3
	CPT1A
	RFX4
	PRDM13
	SMOC2
	MAN2A1
	WNT5A
	CBLB
	WLS
	ID3
	PROX1
	CCN2
	ESRRB
	TNN
	ADRA1A
	CRISPLD1
	KCNJ8
	CXCR4
	NEUROD4
	PDE1B
	USP45
	CDKN1A
	HSPA2
	PODXL
	AJUBA
	FOXJ1
	ANGPTL8
	GDF15
	CASZ1
	NES
	CNTN6
	PDGFRA
	CEMIP2
	PAX3
	PCDH8
	EDNRB
	ENPP2
	TFAP2A
	THBS1
	SMAD6
	BMPR1B
	FBN2
	LGR5
	ASCL1
	LPAR6
	HAPLN3
	RHCG
	MFGE8
	TAF4B
	GREB1L
	EPHA2
	SYPL2
	MAEL
	ILDR2
	SOX13
	SYT2
	MEIS1
	ACKR3
	HES6
	BOC
	EPHA5
	MEGF10
	TPBG
	NTRK2
	CRB2
	KLHL1
	THRB
	POU4F2
	POU4F1
	SEMA3D
	ROBO3
	ANGPT1
	PGM5
	PDLIM3
	ZIC3
	SHROOM4
	SLC45A3
	ADAMTS4
	WNT9B
	NPHS1
	ITGA5
	PDPN
	DHRS3
	OMA1
	LMX1A
	EN1
	MSX1
	ADGRV1
	TLX3
	EN2
	UNCX
	PHOX2A
	RBPMS2
	TMIGD2
	GBX2
	THBS3
	GSX1
	P2RY1
	MGMT
	IRX1
	PKHD1
	FOXB1
	MBOAT1
	NDNF
	FOSL1
	NR2F1
	IRX5
	OLIG3
	IRX3
	NHLH2
	ADAMTSL1
	ZHX2
	GPR157
	UNC5C
	COL18A1
	POU3F2
	ROR1
	NR2F2
	POU3F1
	SLITRK2
	RTN4RL2
	COL4A1
	ISG15
	WNT7B
	POTEE
	HES4
	ZP3
	PCDH18
	H4C3
	ENPP1
	HES5
	NTRK1
	NRARP
	DLL1
	RBM20
	LGR4
	SKOR2
	MUSTN1

source	GO:BP
term_id	GO:0032502
term_name	developmental_process
intersection_size	182
intersection	CYP26B1
	ABCC8
	PROM1
	TEAD3
	TFAP2B
	PGLYRP1
	PAX7
	CD9
	WWTR1
	ATP1A2
	LAMC3
	CNN2
	FGFR3
	TGFBR3
	WNT8B
	PLXNA2
	THOC1
	CEACAM1
	LRP2
	NOX4
	TESC
	LHX5
	DLL3
	ANGPT2
	TMEM38B
	TLL2
	CYP26A1
	PCSK5
	HMOX1
	SALL4
	JAG1
	SYNDIG1
	RGCC
	CDK6
	ITGB8
	LFNG
	TSPAN12
	WNT3
	CPT1A
	RFX4
	PRDM13
	SMOC2
	MAN2A1
	WNT5A
	CBLB
	WLS
	ID3
	PROX1
	CCN2
	ESRRB
	TNN
	ADRA1A
	CRISPLD1
	KCNJ8
	CXCR4
	ARL4A
	NEUROD4
	PDE1B
	USP45
	CDKN1A
	HSPA2
	TSPAN8
	PODXL
	AJUBA
	FOXJ1
	ANGPTL8
	GDF15
	CASZ1
	NES
	CNTN6
	PDGFRA
	CEMIP2
	PAX3
	PCDH8
	EDNRB
	ENPP2
	TFAP2A
	THBS1
	SMAD6
	BMPR1B
	FBN2
	LGR5
	ASCL1
	LPAR6
	HAPLN3
	RHCG
	MFGE8
	TAF4B
	GREB1L
	EPHA2
	SYPL2
	MAEL
	ILDR2
	CREB3L4
	SOX13
	SYT2
	MEIS1
	ACKR3
	HES6
	BOC
	EPHA5
	MEGF10
	TPBG
	NTRK2
	CRB2
	KLHL1
	THRB
	CCDC3
	POU4F2
	POU4F1
	SEMA3D
	ROBO3
	ANGPT1
	PGM5
	PDLIM3
	ZIC3
	SHROOM4
	SLC45A3
	ADAMTS4
	WNT9B
	NPHS1
	ITGA5
	PDPN
	DHRS3
	OMA1
	LMX1A
	EN1
	MSX1
	DAPL1
	ADGRV1
	TLX3
	EN2
	UNCX
	PHOX2A
	RBPMS2
	TMIGD2
	GBX2
	THBS3
	GSX1
	P2RY1
	MGMT
	IRX1
	PKHD1
	FOXB1
	SLC9C1
	MBOAT1
	NDNF
	FOSL1
	NR2F1
	IRX5
	OLIG3
	IRX3
	NHLH2
	ADAMTSL1
	ZHX2
	GPR157
	UNC5C
	COL18A1
	POU3F2
	ROR1
	NR2F2
	POU3F1
	SLITRK2
	RTN4RL2
	COL4A1
	ISG15
	WNT7B
	POTEE
	HES4
	ZP3
	PCDH18
	H4C3
	ENPP1
	HES5
	NTRK1
	NRARP
	DLL1
	RBM20
	LGR4
	SKOR2
	MIAT
	MUSTN1

source	GO:BP
term_id	GO:0048869
term_name	cellular_developmental_process
intersection_size	143
intersection	CYP26B1
	ABCC8
	PROM1
	TEAD3
	TFAP2B
	PGLYRP1
	CD9
	WWTR1
	LAMC3
	CNN2
	FGFR3
	TGFBR3
	WNT8B
	PLXNA2
	CEACAM1
	LRP2
	NOX4
	TESC
	LHX5
	DLL3
	ANGPT2
	TMEM38B
	TLL2
	JAG1
	RGCC
	CDK6
	ITGB8
	LFNG
	WNT3
	CPT1A
	PRDM13
	MAN2A1
	WNT5A
	ID3
	PROX1
	CCN2
	ESRRB
	TNN
	ADRA1A
	CXCR4
	ARL4A
	NEUROD4
	PDE1B
	HSPA2
	PODXL
	FOXJ1
	ANGPTL8
	GDF15
	CASZ1
	NES
	CNTN6
	PDGFRA
	EDNRB
	ENPP2
	TFAP2A
	SMAD6
	BMPR1B
	FBN2
	LGR5
	ASCL1
	RHCG
	TAF4B
	EPHA2
	SYPL2
	MAEL
	ILDR2
	SOX13
	SYT2
	MEIS1
	ACKR3
	HES6
	BOC
	EPHA5
	MEGF10
	TPBG
	NTRK2
	CRB2
	KLHL1
	THRB
	CCDC3
	POU4F2
	POU4F1
	SEMA3D
	ROBO3
	ANGPT1
	PGM5
	ZIC3
	SLC45A3
	WNT9B
	NPHS1
	ITGA5
	PDPN
	LMX1A
	EN1
	MSX1
	DAPL1
	ADGRV1
	TLX3
	EN2
	UNCX
	PHOX2A
	RBPMS2
	GBX2
	GSX1
	P2RY1
	MGMT
	IRX1
	PKHD1
	FOXB1
	SLC9C1
	MBOAT1
	NDNF
	NR2F1
	IRX5
	OLIG3
	IRX3
	NHLH2
	ADAMTSL1
	ZHX2
	GPR157
	UNC5C
	COL18A1
	POU3F2
	ROR1
	NR2F2
	POU3F1
	SLITRK2
	RTN4RL2
	COL4A1
	ISG15
	WNT7B
	HES4
	ZP3
	H4C3
	ENPP1
	HES5
	NTRK1
	NRARP
	DLL1
	LGR4
	SKOR2
	MIAT
	MUSTN1

source	GO:BP
term_id	GO:0030154
term_name	cell_differentiation
intersection_size	141
intersection	CYP26B1
	ABCC8
	PROM1
	TEAD3
	TFAP2B
	PGLYRP1
	CD9
	WWTR1
	LAMC3
	CNN2
	FGFR3
	TGFBR3
	WNT8B
	PLXNA2
	CEACAM1
	LRP2
	NOX4
	TESC
	LHX5
	DLL3
	ANGPT2
	TMEM38B
	TLL2
	JAG1
	RGCC
	CDK6
	ITGB8
	LFNG
	WNT3
	CPT1A
	PRDM13
	MAN2A1
	WNT5A
	ID3
	PROX1
	CCN2
	ESRRB
	TNN
	ADRA1A
	CXCR4
	ARL4A
	NEUROD4
	PDE1B
	HSPA2
	PODXL
	FOXJ1
	ANGPTL8
	GDF15
	CASZ1
	CNTN6
	PDGFRA
	EDNRB
	TFAP2A
	SMAD6
	BMPR1B
	FBN2
	LGR5
	ASCL1
	RHCG
	TAF4B
	EPHA2
	SYPL2
	MAEL
	ILDR2
	SOX13
	SYT2
	MEIS1
	ACKR3
	HES6
	BOC
	EPHA5
	MEGF10
	TPBG
	NTRK2
	CRB2
	KLHL1
	THRB
	CCDC3
	POU4F2
	POU4F1
	SEMA3D
	ROBO3
	ANGPT1
	PGM5
	ZIC3
	SLC45A3
	WNT9B
	NPHS1
	ITGA5
	PDPN
	LMX1A
	EN1
	MSX1
	DAPL1
	ADGRV1
	TLX3
	EN2
	UNCX
	PHOX2A
	RBPMS2
	GBX2
	GSX1
	P2RY1
	MGMT
	IRX1
	PKHD1
	FOXB1
	SLC9C1
	MBOAT1
	NDNF
	NR2F1
	IRX5
	OLIG3
	IRX3
	NHLH2
	ADAMTSL1
	ZHX2
	GPR157
	UNC5C
	COL18A1
	POU3F2
	ROR1
	NR2F2
	POU3F1
	SLITRK2
	RTN4RL2
	COL4A1
	ISG15
	WNT7B
	HES4
	ZP3
	H4C3
	ENPP1
	HES5
	NTRK1
	NRARP
	DLL1
	LGR4
	SKOR2
	MIAT
	MUSTN1

source	GO:BP
term_id	GO:0035295
term_name	tube_development
intersection_size	63
intersection	ABCC8
	PROM1
	TFAP2B
	WWTR1
	TGFBR3
	PLXNA2
	CEACAM1
	LRP2
	ANGPT2
	TMEM38B
	PCSK5
	HMOX1
	SALL4
	JAG1
	RGCC
	ITGB8
	TSPAN12
	SMOC2
	MAN2A1
	WNT5A
	PROX1
	CCN2
	TNN
	CXCR4
	PODXL
	FOXJ1
	PDGFRA
	CEMIP2
	EDNRB
	ENPP2
	THBS1
	SMAD6
	LGR5
	ASCL1
	MFGE8
	GREB1L
	EPHA2
	MEIS1
	ACKR3
	NTRK2
	THRB
	ANGPT1
	ZIC3
	WNT9B
	ITGA5
	PDPN
	EN1
	RBPMS2
	TMIGD2
	GBX2
	IRX1
	PKHD1
	FOXB1
	NDNF
	IRX3
	COL18A1
	NR2F2
	COL4A1
	WNT7B
	HES5
	NRARP
	DLL1
	LGR4

source	GO:BP
term_id	GO:0048646
term_name	anatomical_structure_formation_involved_in_morphogenesis
intersection_size	65
intersection	ABCC8
	PROM1
	TFAP2B
	CD9
	TGFBR3
	PLXNA2
	CEACAM1
	LRP2
	LHX5
	DLL3
	ANGPT2
	HMOX1
	SALL4
	JAG1
	RGCC
	ITGB8
	LFNG
	TSPAN12
	WNT3
	SMOC2
	WNT5A
	WLS
	PROX1
	CCN2
	TNN
	CXCR4
	PODXL
	FOXJ1
	GDF15
	PDGFRA
	CEMIP2
	PCDH8
	ENPP2
	TFAP2A
	THBS1
	FBN2
	MFGE8
	EPHA2
	MEIS1
	ACKR3
	CRB2
	ANGPT1
	PGM5
	ZIC3
	WNT9B
	NPHS1
	ITGA5
	PDPN
	MSX1
	PHOX2A
	TMIGD2
	GBX2
	THBS3
	IRX1
	FOXB1
	NDNF
	IRX3
	COL18A1
	COL4A1
	ZP3
	HES5
	NRARP
	DLL1
	RBM20
	SKOR2

source	GO:BP
term_id	GO:0022008
term_name	neurogenesis
intersection_size	79
intersection	ABCC8
	PROM1
	TEAD3
	CD9
	LAMC3
	WNT8B
	PLXNA2
	LRP2
	LHX5
	DLL3
	JAG1
	CDK6
	WNT3
	PRDM13
	MAN2A1
	WNT5A
	ID3
	PROX1
	TNN
	CXCR4
	NEUROD4
	CASZ1
	CNTN6
	EDNRB
	BMPR1B
	ASCL1
	EPHA2
	SOX13
	SYT2
	MEIS1
	HES6
	BOC
	EPHA5
	TPBG
	NTRK2
	CRB2
	KLHL1
	THRB
	POU4F2
	POU4F1
	SEMA3D
	ROBO3
	ZIC3
	SLC45A3
	WNT9B
	LMX1A
	EN1
	ADGRV1
	TLX3
	EN2
	UNCX
	PHOX2A
	GBX2
	GSX1
	P2RY1
	IRX1
	FOXB1
	MBOAT1
	NDNF
	NR2F1
	IRX5
	OLIG3
	IRX3
	NHLH2
	ADAMTSL1
	ZHX2
	GPR157
	UNC5C
	POU3F2
	ROR1
	NR2F2
	POU3F1
	SLITRK2
	RTN4RL2
	WNT7B
	HES5
	NTRK1
	DLL1
	SKOR2

source	GO:BP
term_id	GO:0050793
term_name	regulation_of_developmental_process
intersection_size	97
intersection	CYP26B1
	ABCC8
	PROM1
	TFAP2B
	PGLYRP1
	WWTR1
	FGFR3
	TGFBR3
	PLXNA2
	THOC1
	CEACAM1
	LRP2
	TESC
	DLL3
	ANGPT2
	TLL2
	HMOX1
	JAG1
	SYNDIG1
	RGCC
	CDK6
	ITGB8
	LFNG
	TSPAN12
	WNT3
	SMOC2
	MAN2A1
	WNT5A
	ID3
	PROX1
	CCN2
	ESRRB
	TNN
	CXCR4
	NEUROD4
	CDKN1A
	FOXJ1
	GDF15
	CASZ1
	PDGFRA
	CEMIP2
	EDNRB
	ENPP2
	TFAP2A
	THBS1
	SMAD6
	BMPR1B
	FBN2
	ASCL1
	EPHA2
	MAEL
	SOX13
	SYT2
	MEIS1
	HES6
	BOC
	MEGF10
	TPBG
	NTRK2
	CRB2
	CCDC3
	POU4F2
	POU4F1
	SEMA3D
	SLC45A3
	WNT9B
	ITGA5
	PDPN
	OMA1
	LMX1A
	MSX1
	ADGRV1
	TLX3
	UNCX
	RBPMS2
	TMIGD2
	GBX2
	P2RY1
	PKHD1
	IRX3
	ZHX2
	UNC5C
	POU3F2
	ROR1
	SLITRK2
	ISG15
	WNT7B
	ZP3
	H4C3
	ENPP1
	HES5
	NTRK1
	NRARP
	DLL1
	LGR4
	SKOR2
	MUSTN1

source	GO:BP
term_id	GO:0007399
term_name	nervous_system_development
intersection_size	98
intersection	ABCC8
	PROM1
	TEAD3
	TFAP2B
	CD9
	ATP1A2
	LAMC3
	WNT8B
	PLXNA2
	LRP2
	LHX5
	DLL3
	SALL4
	JAG1
	SYNDIG1
	CDK6
	WNT3
	RFX4
	PRDM13
	MAN2A1
	WNT5A
	WLS
	ID3
	PROX1
	TNN
	CXCR4
	NEUROD4
	FOXJ1
	CASZ1
	NES
	CNTN6
	PAX3
	EDNRB
	TFAP2A
	BMPR1B
	ASCL1
	HAPLN3
	EPHA2
	SYPL2
	SOX13
	SYT2
	MEIS1
	ACKR3
	HES6
	BOC
	EPHA5
	TPBG
	NTRK2
	CRB2
	KLHL1
	THRB
	POU4F2
	POU4F1
	SEMA3D
	ROBO3
	ZIC3
	SHROOM4
	SLC45A3
	WNT9B
	LMX1A
	EN1
	MSX1
	ADGRV1
	TLX3
	EN2
	UNCX
	PHOX2A
	GBX2
	GSX1
	P2RY1
	IRX1
	FOXB1
	MBOAT1
	NDNF
	NR2F1
	IRX5
	OLIG3
	IRX3
	NHLH2
	ADAMTSL1
	ZHX2
	GPR157
	UNC5C
	POU3F2
	ROR1
	NR2F2
	POU3F1
	SLITRK2
	RTN4RL2
	COL4A1
	WNT7B
	POTEE
	HES4
	PCDH18
	HES5
	NTRK1
	DLL1
	SKOR2

source	GO:BP
term_id	GO:0051239
term_name	regulation_of_multicellular_organismal_process
intersection_size	107
intersection	CYP26B1
	ABCC8
	PROM1
	PGLYRP1
	CD9
	WWTR1
	ATP1A2
	LTBP1
	PTGER3
	ABCC9
	TGFBR3
	LMCD1
	PLXNA2
	THOC1
	CEACAM1
	LRP2
	OPRK1
	TESC
	DLL3
	ANGPT2
	TMEM38B
	HMOX1
	JAG1
	SYNDIG1
	RGCC
	CDK6
	ITGB8
	LFNG
	TSPAN12
	WNT3
	P2RX3
	SMOC2
	MAN2A1
	WNT5A
	CBLB
	PLA2G4A
	PROX1
	CCN2
	ESRRB
	TNN
	ADRA1A
	CXCR4
	KCNJ2
	CDKN1A
	CD70
	TSPAN8
	FOXJ1
	GDF15
	HRC
	PRAM1
	PDGFRA
	CEMIP2
	EDNRB
	ENPP2
	TFAP2A
	THBS1
	SMAD6
	BMPR1B
	FBN2
	ASCL1
	EPHA2
	MAEL
	ILDR2
	SOX13
	MEIS1
	HES6
	GLRA1
	TPBG
	NTRK2
	CRB2
	THRB
	POU4F2
	POU4F1
	GRM1
	SEMA3D
	ANGPT1
	LDLRAP1
	WNT9B
	ITGA5
	PDPN
	DHRS3
	OMA1
	INAVA
	ADGRV1
	TLX3
	PHOX2A
	TMIGD2
	GBX2
	P2RY1
	NETO2
	IRX3
	IL17RA
	POU3F2
	NR2F2
	SLITRK2
	ISG15
	ZP3
	LITAF
	H4C3
	ENPP1
	HES5
	NTRK1
	NRARP
	DLL1
	FCGR3A
	LGR4
	MUSTN1

GO:BP
GO:0009888
tissue_development
84

CYP26B1
PROM1
TFAP2B
WWTR1
LAMC3
FGFR3
TGFBR3
PLXNA2
CEACAM1
LRP2
NOX4
DLL3
TMEM38B
TLL2
SALL4
JAG1
RGCC
CDK6
ITGB8
LFNG
WNT3
CPT1A
WNT5A
WLS
ID3
PROX1
CCN2
TNN
ADRA1A
CXCR4
PODXL
AJUBA
FOXJ1
PDGFRA
PCDH8
EDNRB
TFAP2A
SMAD6
BMPR1B
FBN2
LGR5
ASCL1
RHCG
GREB1L
EPHA2
MEGF10
CRB2
THRB
POU4F2
POU4F1
SEMA3D
PGM5
ZIC3
WNT9B
NPHS1
ITGA5
PDPN
EN1
MSX1
ADGRV1
UNCX
GBX2
THBS3
MGMT
IRX1
PKHD1
FOXB1
IRX3
UNC5C
COL18A1
POU3F2
ROR1
NR2F2
POU3F1
COL4A1
ISG15
WNT7B
ENPP1
HES5
NTRK1
NRARP
DLL1
LGR4
MUSTN1

GO:BP
GO:0072359
circulatory_system_development
60

ABCC8
TFAP2B
TGFBR3
CEACAM1
LRP2
NOX4
ANGPT2
PCSK5
HMOX1
SALL4
JAG1
RGCC
ITGB8
TSPAN12
SMOC2
WNT5A
ID3
PROX1
CCN2
TNN
ADRA1A
KCNJ8
CXCR4
CDKN1A
FOXJ1
PDGFRA
CEMIP2
ENPP2
THBS1
SMAD6
ASCL1
MFGE8
GREB1L
EPHA2
SYPL2
MEIS1
ACKR3
NTRK2
CRB2
POU4F2
POU4F1
ANGPT1
PDLIM3
ZIC3
ITGA5
PDPN
DHRS3
MSX1
TMIGD2
GBX2
NDNF
FOSL1
IRX3
COL18A1
NR2F2
COL4A1
WNT7B
NRARP
DLL1
RBM20

GO:BP
GO:0007417
central_nervous_system_development
59

ATP1A2
LAMC3
PLXNA2
LRP2
LHX5
CDK6
WNT3
RFX4
PRDM13
WNT5A
WLS
ID3
PROX1
CXCR4
NEUROD4
FOXJ1
NES
CNTN6
TFAP2A
BMPR1B
ASCL1
HAPLN3
SYPL2
SOX13
MEIS1
ACKR3
EPHA5
NTRK2
KLHL1
POU4F1
ZIC3
SHROOM4
SLC45A3
WNT9B
LMX1A
EN1
MSX1
TLX3
EN2
UNCX
PHOX2A
GBX2
GSX1
FOXB1
NDNF
OLIG3
NHLH2
UNC5C
POU3F2
ROR1
NR2F2
POU3F1
COL4A1
WNT7B
POTEE
PCDH18
HES5
DLL1
SKOR2

GO:BP
GO:0072001
renal_system_development
32

CYP26B1
PROM1
TFAP2B
WWTR1
LRP2
ANGPT2
CYP26A1
PCSK5
JAG1
WNT5A
ID3
PROX1
KCNJ8
PODXL
FOXJ1
PDGFRA
EDNRB
TFAP2A
SMAD6
LGR5
GREB1L
ANGPT1
WNT9B
NPHS1
IRX1
PKHD1
IRX3
COL4A1
WNT7B
HES5
DLL1
LGR4

GO:BP
GO:0048699
generation_of_neurons
68

PROM1
TEAD3
WNT8B
PLXNA2
LRP2
LHX5
JAG1
CDK6
WNT3
PRDM13
WNT5A
ID3
PROX1
TNN
CXCR4
NEUROD4
CASZ1
CNTN6
EDNRB
BMPR1B
ASCL1
EPHA2
SYT2
MEIS1
BOC
EPHA5
TPBG
NTRK2
CRB2
KLHL1
THRB
POU4F2
POU4F1
SEMA3D
ROBO3
ZIC3
WNT9B
LMX1A
EN1
ADGRV1
TLX3
EN2
UNCX
PHOX2A
GBX2
GSX1
IRX1
FOXB1
MBOAT1
NDNF
NR2F1
IRX5
OLIG3
IRX3
NHLH2
ADAMTSL1
ZHX2
UNC5C
POU3F2
ROR1
NR2F2
SLITRK2
RTN4RL2
WNT7B
HES5
NTRK1
DLL1
SKOR2

GO:BP
GO:0030182
neuron_differentiation
65

PROM1
WNT8B
PLXNA2
LRP2
LHX5
JAG1
WNT3
PRDM13
WNT5A
ID3
PROX1
TNN
CXCR4
NEUROD4
CASZ1
CNTN6
EDNRB
BMPR1B
ASCL1
EPHA2
SYT2
MEIS1
BOC
EPHA5
TPBG
NTRK2
CRB2
KLHL1
THRB
POU4F2
POU4F1
SEMA3D
ROBO3
ZIC3
WNT9B
LMX1A
EN1
ADGRV1
TLX3
EN2
UNCX
PHOX2A
GBX2
GSX1
IRX1
FOXB1
MBOAT1
NDNF
NR2F1
IRX5
OLIG3
IRX3
NHLH2
ADAMTSL1
ZHX2
UNC5C
POU3F2
ROR1
SLITRK2
RTN4RL2
WNT7B
HES5
NTRK1
DLL1
SKOR2

GO:BP
GO:0048518
positive_regulation_of_biological_process
163

CYP26B1
ABCC8
PROM1
TEAD3
TFAP2B
DDX11
WWTR1
ATP1A2
FAS
PTGER3
TRAF1
CNN2
FGFR3
TGFBR3
LMCD1
PLXNA2
THOC1
CDC14A
CEACAM1
LRP2
OPRK1
NOX4
TESC
LHX5
ANGPT2
CCDC80
PRODH
HMOX1
SALL4
JAG1
SYNDIG1
RGCC
TOX3
CDK6
ITGB8
LFNG
EBF3
WNT3
P2RX3
CPT1A
RFX4
SMOC2
MAN2A1
WNT5A
CBLB
PLA2G4A
WLS
ID3
PROX1
CCN2
ELL2
ESRRB
TNN
ADRA1A
CXCR4
NEUROD4
KCNJ2
CDKN1A
AHNAK
CLYBL
CD70
HSPA2
PODXL
AJUBA
FOXJ1
SH3BP4
ANGPTL8
GDF15
HRC
CASZ1
NES
PRAM1
CNTN6
PDGFRA
PAX3
EDNRB
ENPP2
TFAP2A
THBS1
SMAD6
BMPR1B
PRDM5
FBN2
LGR5
ASCL1
LPAR6
HAPLN3
ABCA8
TAF4B
EPHA2
CREB3L4
SOX13
SYT2
MEIS1
ACKR3
BOC
EPHA5
MEGF10
GLRA1
TPBG
NTRK2
CRB2
THRB
CCDC3
POU4F2
POU4F1
GRM1
SEMA3D
ANGPT1
ZIC3
LDLRAP1
SLC45A3
NPHS1
ITGA5
PDPN
OMA1
LMX1A
EN1
MSX1
INAVA
ADGRV1
EN2
PHOX2A
RBPMS2
TMIGD2
GBX2
RSPO1
GSX1
P2RY1
MGMT
PKHD1
NDNF
TNFRSF10C
SNX33
FGFBP3
FOSL1
NR2F1
IRX3
NHLH2
IL17RA
UNC5C
MAML2
POU3F2
ROR1
MUC1
NR2F2
POU3F1
SLITRK2
ISG15
WNT7B
ZP3
LITAF
HES5
ZXDA
NTRK1
NRARP
DLL1
FCGR3A
RBM20
MICB
LGR4
SKOR2
MUSTN1

GO:BP
GO:0048522
positive_regulation_of_cellular_process
150

CYP26B1
ABCC8
PROM1
TEAD3
TFAP2B
DDX11
WWTR1
FAS
TRAF1
FGFR3
TGFBR3
LMCD1
PLXNA2
THOC1
CDC14A
CEACAM1
LRP2
OPRK1
NOX4
TESC
LHX5
CCDC80
PRODH
HMOX1
SALL4
JAG1
SYNDIG1
RGCC
TOX3
CDK6
LFNG
EBF3
WNT3
P2RX3
CPT1A
RFX4
SMOC2
MAN2A1
WNT5A
PLA2G4A
WLS
ID3
PROX1
CCN2
ELL2
ESRRB
TNN
ADRA1A
CXCR4
NEUROD4
KCNJ2
CDKN1A
AHNAK
CLYBL
CD70
HSPA2
PODXL
AJUBA
FOXJ1
SH3BP4
GDF15
CASZ1
NES
CNTN6
PDGFRA
PAX3
EDNRB
ENPP2
TFAP2A
THBS1
SMAD6
BMPR1B
PRDM5
FBN2
LGR5
ASCL1
LPAR6
HAPLN3
TAF4B
EPHA2
CREB3L4
SOX13
SYT2
MEIS1
ACKR3
BOC
EPHA5
MEGF10
TPBG
NTRK2
CRB2
THRB
CCDC3
POU4F2
POU4F1
GRM1
SEMA3D
ANGPT1
ZIC3
LDLRAP1
SLC45A3
NPHS1
ITGA5
PDPN
OMA1
LMX1A
EN1
MSX1
INAVA
ADGRV1
EN2
PHOX2A
RBPMS2
TMIGD2
GBX2
RSPO1
GSX1
P2RY1
MGMT
PKHD1
NDNF
TNFRSF10C
SNX33
FGFBP3
FOSL1
NR2F1
IRX3
NHLH2
UNC5C
MAML2
POU3F2
ROR1
MUC1
NR2F2
POU3F1
SLITRK2
ISG15
WNT7B
ZP3
LITAF
HES5
ZXDA
NTRK1
NRARP
DLL1
FCGR3A
RBM20
LGR4
SKOR2
MUSTN1

GO:BP
GO:0035239
tube_morphogenesis
49

ABCC8
TFAP2B
TGFBR3
CEACAM1
LRP2
ANGPT2
HMOX1
SALL4
JAG1
RGCC
ITGB8
TSPAN12
SMOC2
WNT5A
PROX1
CCN2
TNN
CXCR4
PODXL
PDGFRA
CEMIP2
ENPP2
THBS1
LGR5
MFGE8
GREB1L
EPHA2
MEIS1
ACKR3
NTRK2
ANGPT1
ZIC3
WNT9B
ITGA5
RBPMS2
TMIGD2
GBX2
IRX1
PKHD1
NDNF
IRX3
COL18A1
NR2F2
COL4A1
WNT7B
HES5
NRARP
DLL1
LGR4

GO:BP
GO:0001822
kidney_development
30

CYP26B1
PROM1
TFAP2B
WWTR1
LRP2
ANGPT2
CYP26A1
PCSK5
JAG1
WNT5A
ID3
PROX1
KCNJ8
PODXL
FOXJ1
PDGFRA
EDNRB
TFAP2A
SMAD6
GREB1L
ANGPT1
WNT9B
NPHS1
IRX1
PKHD1
IRX3
WNT7B
HES5
DLL1
LGR4

GO:BP
GO:0060322
head_development
48

ATP1A2
PLXNA2
LRP2
LHX5
CDK6
WNT3
RFX4
PRDM13
WNT5A
WLS
PROX1
CRISPLD1
CXCR4
FOXJ1
NES
PDGFRA
ASCL1
SYPL2
MEIS1
EPHA5
NTRK2
KLHL1
POU4F1
ZIC3
SHROOM4
WNT9B
LMX1A
EN1
MSX1
EN2
UNCX
PHOX2A
GBX2
GSX1
FOXB1
NDNF
NHLH2
UNC5C
POU3F2
NR2F2
POU3F1
COL4A1
WNT7B
POTEE
PCDH18
HES5
DLL1
SKOR2

GO:BP
GO:0007420
brain_development
46

ATP1A2
PLXNA2
LRP2
LHX5
CDK6
WNT3
RFX4
PRDM13
WNT5A
WLS
PROX1
CXCR4
FOXJ1
NES
ASCL1
SYPL2
MEIS1
EPHA5
NTRK2
KLHL1
POU4F1
ZIC3
SHROOM4
WNT9B
LMX1A
EN1
MSX1
EN2
UNCX
PHOX2A
GBX2
GSX1
FOXB1
NDNF
NHLH2
UNC5C
POU3F2
NR2F2
POU3F1
COL4A1
WNT7B
POTEE
PCDH18
HES5
DLL1
SKOR2

GO:BP
GO:0060429
epithelium_development
58

CYP26B1
PROM1
TFAP2B
WWTR1
PLXNA2
CEACAM1
LRP2
DLL3
TMEM38B
SALL4
JAG1
CDK6
LFNG
WNT3
CPT1A
WNT5A
ID3
PROX1
CXCR4
PODXL
AJUBA
FOXJ1
PCDH8
EDNRB
TFAP2A
SMAD6
LGR5
ASCL1
RHCG
GREB1L
EPHA2
CRB2
THRB
ZIC3
WNT9B
NPHS1
ITGA5
PDPN
EN1
MSX1
GBX2
MGMT
IRX1
PKHD1
FOXB1
IRX3
COL18A1
POU3F2
ROR1
NR2F2
POU3F1
COL4A1
WNT7B
HES5
NTRK1
NRARP
DLL1
LGR4

GO:BP
GO:0065007
biological_regulation
252

CYP26B1
ABCC8
PROM1
RHBDF1
TEAD3
TFAP2B
PGLYRP1
PAX7
CD9
DDX11
WWTR1
ATP1A2
ABCC2
FAS
LTBP1
PTGER3
TRAF1
CNN2
FGFR3
ABCC9
TGFBR3
LMCD1
WNT8B
PLXNA2
THOC1
CDC14A
CEACAM1
LRP2
OPRK1
CHMP2B
NOX4
TESC
LHX5
DLL3
ANGPT2
CCDC80
ADA2
TMEM38B
TLL2
CYP26A1
PCSK5
PRODH
HMOX1
KCNK10
SALL4
NKAIN4
JAG1
SYNDIG1
RGCC
TOX3
CDK6
ITGB8
LFNG
TSPAN12
EBF3
RNF43
WNT3
P2RX3
CPT1A
PRMT8
RFX4
PRDM13
SMOC2
MAN2A1
SERPINI2
WNT5A
CBLB
PLA2G4A
WLS
ID3
PROX1
CCN2
ELL2
ESRRB
TNN
ADRA1A
SCPEP1
KCNJ8
CXCR4
NEUROD4
PDE1B
KCNJ2
CDKN1A
AHNAK
CLYBL
CD70
KIRREL2
HSPA2
TSPAN8
PODXL
USP6
AJUBA
FOXJ1
SH3BP4
DOCK6
ANGPTL8
GDF15
HRC
CASZ1
NES
PRAM1
CNTN6
PDGFRA
CEMIP2
PAX3
PCDH8
EDNRB
ENPP2
TFAP2A
FGFBP2
THBS1
SMAD6
BMPR1B
PRDM5
FBN2
LGR5
ASCL1
LPAR6
GRTP1
HAPLN3
RHCG
ABCA8
TAF4B
EPHA2
MAEL
ILDR2
RGS16
CREB3L4
SOX13
SYT2
MEIS1
THNSL2
ACKR3
HES6
BOC
EPHA5
MEGF10
GLRA1
TPBG
RGS20
NTRK2
CRB2
THRB
ABTB3
CCDC3
POU4F2
POU4F1
GRM1
SEMA3D
ANGPT1
PIEZO2
ZIC3
LDLRAP1
SLC45A3
WNT9B
NPHS1
ITGA5
PDPN
DHRS3
OMA1
OLFML2B
LMX1A
EN1
MSX1
ARHGAP25
DAPL1
INAVA
NUAK2
ADGRV1
EBF1
TLX3
EN2
UNCX
PHOX2A
SLC6A5
CRABP1
RBPMS2
TAC3
TMIGD2
GBX2
RSPO1
GSX1
P2RY1
MGMT
IRX1
PKHD1
NETO2
PAH
FOXB1
SLC9C1
MBOAT1
NDNF
TNFRSF10C
SNX33
GNG5
FGFBP3
FOSL1
NR2F1
PRIMA1
IRX5
OLIG3
IRX3
NHLH2
IL17RA
CA8
ZHX2
GPR157
UNC5C
ASB18
COL18A1
ZNF662
KCNH8
MAML2
POU3F2
ROR1
MUC1
NR2F2
POU3F1
SLITRK2
RTN4RL2
COL4A1
ISG15
WNT7B
HES4
ZP3
SKOR1
LITAF
H4C3
H3C12
ENPP1
HES5
HSD17B11
ZXDA
NTRK1
NRARP
DLL1
PPP1R14C
FCGR3A
RBM20
MICB
LGR4
VGLL3
MIR219A2HG
MIR125B1
TBC1D26
SKOR2
MIR34AHG
XIST
LINC00472
STON1
TSIX
MUSTN1

GO:BP
GO:0009887
animal_organ_morphogenesis
52

CYP26B1
PROM1
TFAP2B
WWTR1
LAMC3
FGFR3
TGFBR3
CEACAM1
LRP2
JAG1
LFNG
TSPAN12
MAN2A1
WNT5A
WLS
ID3
PROX1
CCN2
FOXJ1
PDGFRA
PAX3
TFAP2A
SMAD6
BMPR1B
FBN2
GREB1L
EPHA2
MEIS1
NTRK2
CRB2
THRB
POU4F1
ZIC3
WNT9B
DHRS3
MSX1
UNCX
RBPMS2
GBX2
THBS3
IRX1
PKHD1
IRX5
IRX3
ZHX2
COL18A1
ROR1
WNT7B
HES5
DLL1
RBM20
LGR4

GO:BP
GO:0007389
pattern_specification_process
34

CYP26B1
WNT8B
PLXNA2
LRP2
DLL3
TLL2
PCSK5
LFNG
WNT3
RFX4
WNT5A
WLS
FOXJ1
PCDH8
SMAD6
BMPR1B
ASCL1
MEIS1
HES6
CRB2
ZIC3
EN1
MSX1
UNCX
GBX2
IRX1
FOXB1
IRX3
NR2F2
WNT7B
HES4
HES5
NRARP
DLL1

GO:BP
GO:0045595
regulation_of_cell_differentiation
64

CYP26B1
ABCC8
PROM1
TFAP2B
PGLYRP1
WWTR1
PLXNA2
CEACAM1
LRP2
TESC
DLL3
JAG1
RGCC
CDK6
WNT3
MAN2A1
WNT5A
ID3
PROX1
CCN2
ESRRB
TNN
CXCR4
NEUROD4
FOXJ1
CASZ1
PDGFRA
EDNRB
TFAP2A
SMAD6
BMPR1B
FBN2
ASCL1
SOX13
MEIS1
HES6
BOC
MEGF10
NTRK2
CRB2
CCDC3
POU4F2
POU4F1
SEMA3D
SLC45A3
WNT9B
PDPN
LMX1A
MSX1
ADGRV1
TLX3
UNCX
RBPMS2
IRX3
ZHX2
POU3F2
ISG15
WNT7B
H4C3
ENPP1
HES5
NRARP
DLL1
MUSTN1

GO:BP
GO:0003002
regionalization
32

CYP26B1
WNT8B
PLXNA2
LRP2
DLL3
TLL2
PCSK5
LFNG
WNT3
RFX4
WNT5A
WLS
FOXJ1
PCDH8
SMAD6
BMPR1B
ASCL1
HES6
CRB2
ZIC3
EN1
MSX1
GBX2
IRX1
FOXB1
IRX3
NR2F2
WNT7B
HES4
HES5
NRARP
DLL1

GO:BP
GO:0008283
cell_population_proliferation
73

ABCC8
RHBDF1
TEAD3
TFAP2B
CD9
WWTR1
CNN2
FGFR3
TGFBR3
CEACAM1
LRP2
NOX4
TESC
LHX5
HMOX1
SALL4
JAG1
RGCC
CDK6
RNF43
WNT3
WNT5A
CBLB
PLA2G4A
PROX1
CCN2
ESRRB
TNN
ADRA1A
NEUROD4
CDKN1A
CD70
FOXJ1
SH3BP4
NES
PDGFRA
EDNRB
TFAP2A
THBS1
SMAD6
BMPR1B
LGR5
ASCL1
HAPLN3
EPHA2
MEIS1
ACKR3
MEGF10
TPBG
NTRK2
ANGPT1
LDLRAP1
PDPN
MSX1
UNCX
RBPMS2
TMIGD2
GBX2
PKHD1
FOSL1
COL18A1
POU3F2
NR2F2
WNT7B
ZP3
HES5
NTRK1
NRARP
DLL1
FCGR3A
LGR4
SKOR2
MUSTN1

GO:BP
GO:0007155
cell_adhesion
62

CD9
LAMC3
PLXNA2
CEACAM1
TESC
ANGPT2
CCDC80
JAG1
RGCC
CDK6
ITGB8
SERPINI2
WNT5A
CBLB
CCN2
TNN
CXCR4
CD70
KIRREL2
PODXL
AJUBA
FOXJ1
CNTN6
PDGFRA
PCDH8
THBS1
SMAD6
HAPLN3
MFGE8
EPHA2
ILDR2
HMCN1
SOX13
ACKR3
BOC
MEGF10
TPBG
CRB2
ROBO3
ANGPT1
PGM5
NPHS1
ITGA5
PDPN
ADGRV1
TMIGD2
THBS3
PKHD1
NDNF
IGDCC3
ADAMTSL1
UNC5C
COL18A1
MUC1
SLITRK2
WNT7B
ZP3
PCDH18
HES5
NRARP
DLL1
STON1

GO:BP
GO:2000026
regulation_of_multicellular_organismal_development
59

CYP26B1
ABCC8
PROM1
PGLYRP1
WWTR1
TGFBR3
PLXNA2
CEACAM1
LRP2
TESC
DLL3
ANGPT2
HMOX1
JAG1
SYNDIG1
RGCC
CDK6
ITGB8
TSPAN12
WNT3
SMOC2
MAN2A1
WNT5A
PROX1
CCN2
TNN
CXCR4
FOXJ1
CEMIP2
ENPP2
TFAP2A
THBS1
BMPR1B
FBN2
ASCL1
EPHA2
SOX13
MEIS1
HES6
TPBG
NTRK2
CRB2
POU4F2
POU4F1
SEMA3D
WNT9B
ITGA5
ADGRV1
TMIGD2
GBX2
SLITRK2
ISG15
H4C3
ENPP1
HES5
NTRK1
NRARP
DLL1
MUSTN1

GO:BP
GO:0050794
regulation_of_cellular_process
226

CYP26B1
ABCC8
PROM1
RHBDF1
TEAD3
TFAP2B
PGLYRP1
PAX7
CD9
DDX11
WWTR1
ATP1A2
FAS
LTBP1
PTGER3
TRAF1
CNN2
FGFR3
ABCC9
TGFBR3
LMCD1
WNT8B
PLXNA2
THOC1
CDC14A
CEACAM1
LRP2
OPRK1
CHMP2B
NOX4
TESC
LHX5
DLL3
ANGPT2
CCDC80
ADA2
TMEM38B
CYP26A1
PRODH
HMOX1
KCNK10
SALL4
JAG1
SYNDIG1
RGCC
TOX3
CDK6
ITGB8
LFNG
TSPAN12
EBF3
RNF43
WNT3
P2RX3
CPT1A
RFX4
PRDM13
SMOC2
MAN2A1
SERPINI2
WNT5A
CBLB
PLA2G4A
WLS
ID3
PROX1
CCN2
ELL2
ESRRB
TNN
ADRA1A
KCNJ8
CXCR4
NEUROD4
PDE1B
KCNJ2
CDKN1A
AHNAK
CLYBL
CD70
KIRREL2
HSPA2
PODXL
USP6
AJUBA
FOXJ1
SH3BP4
DOCK6
ANGPTL8
GDF15
HRC
CASZ1
NES
PRAM1
CNTN6
PDGFRA
PAX3
PCDH8
EDNRB
ENPP2
TFAP2A
THBS1
SMAD6
BMPR1B
PRDM5
FBN2
LGR5
ASCL1
LPAR6
HAPLN3
TAF4B
EPHA2
MAEL
ILDR2
RGS16
CREB3L4
SOX13
SYT2
MEIS1
THNSL2
ACKR3
HES6
BOC
EPHA5
MEGF10
GLRA1
TPBG
RGS20
NTRK2
CRB2
THRB
ABTB3
CCDC3
POU4F2
POU4F1
GRM1
SEMA3D
ANGPT1
ZIC3
LDLRAP1
SLC45A3
WNT9B
NPHS1
ITGA5
PDPN
DHRS3
OMA1
OLFML2B
LMX1A
EN1
MSX1
ARHGAP25
DAPL1
INAVA
NUAK2
ADGRV1
EBF1
TLX3
EN2
UNCX
PHOX2A
CRABP1
RBPMS2
TAC3
TMIGD2
GBX2
RSPO1
GSX1
P2RY1
MGMT
IRX1
PKHD1
NETO2
FOXB1
MBOAT1
NDNF
TNFRSF10C
SNX33
GNG5
FGFBP3
FOSL1
NR2F1
IRX5
OLIG3
IRX3
NHLH2
IL17RA
CA8
ZHX2
GPR157
UNC5C
ASB18
COL18A1
ZNF662
KCNH8
MAML2
POU3F2
ROR1
MUC1
NR2F2
POU3F1
SLITRK2
RTN4RL2
COL4A1
ISG15
WNT7B
HES4
ZP3
SKOR1
LITAF
H4C3
ENPP1
HES5
ZXDA
NTRK1
NRARP
DLL1
PPP1R14C
FCGR3A
RBM20
MICB
LGR4
VGLL3
SKOR2
STON1
MUSTN1

GO:BP
GO:0001944
vasculature_development
41

ABCC8
TFAP2B
TGFBR3
CEACAM1
LRP2
ANGPT2
PCSK5
HMOX1
JAG1
RGCC
ITGB8
TSPAN12
SMOC2
WNT5A
PROX1
CCN2
TNN
CXCR4
PDGFRA
CEMIP2
ENPP2
THBS1
SMAD6
MFGE8
EPHA2
MEIS1
ACKR3
NTRK2
ANGPT1
ITGA5
PDPN
TMIGD2
GBX2
NDNF
FOSL1
COL18A1
NR2F2
COL4A1
WNT7B
NRARP
DLL1

GO:BP
GO:0048468
cell_development
89

CYP26B1
ABCC8
PGLYRP1
LAMC3
CNN2
TGFBR3
PLXNA2
CEACAM1
LRP2
TESC
DLL3
ANGPT2
JAG1
CDK6
ITGB8
LFNG
WNT3
MAN2A1
WNT5A
PROX1
TNN
ADRA1A
CXCR4
NEUROD4
PDE1B
HSPA2
PODXL
FOXJ1
ANGPTL8
CNTN6
PDGFRA
EDNRB
BMPR1B
LGR5
ASCL1
TAF4B
EPHA2
SYPL2
MAEL
SOX13
SYT2
MEIS1
HES6
BOC
EPHA5
MEGF10
TPBG
NTRK2
CRB2
KLHL1
THRB
POU4F2
POU4F1
SEMA3D
ROBO3
ANGPT1
PGM5
NPHS1
LMX1A
EN1
ADGRV1
EN2
GBX2
IRX1
PKHD1
FOXB1
MBOAT1
NDNF
NR2F1
IRX5
IRX3
NHLH2
ADAMTSL1
UNC5C
COL18A1
POU3F2
ROR1
POU3F1
SLITRK2
RTN4RL2
ISG15
WNT7B
ZP3
H4C3
HES5
NTRK1
NRARP
DLL1
SKOR2

GO:BP
GO:0001568
blood_vessel_development
40

ABCC8
TFAP2B
TGFBR3
CEACAM1
LRP2
ANGPT2
PCSK5
HMOX1
JAG1
RGCC
ITGB8
TSPAN12
SMOC2
WNT5A
PROX1
CCN2
TNN
CXCR4
PDGFRA
CEMIP2
ENPP2
THBS1
SMAD6
MFGE8
EPHA2
MEIS1
ACKR3
NTRK2
ANGPT1
ITGA5
TMIGD2
GBX2
NDNF
FOSL1
COL18A1
NR2F2
COL4A1
WNT7B
NRARP
DLL1

GO:BP
GO:0051094
positive_regulation_of_developmental_process
56

CYP26B1
PROM1
WWTR1
TGFBR3
PLXNA2
CEACAM1
LRP2
TESC
ANGPT2
HMOX1
JAG1
SYNDIG1
RGCC
ITGB8
WNT3
SMOC2
MAN2A1
WNT5A
PROX1
CCN2
ESRRB
TNN
CXCR4
NEUROD4
FOXJ1
GDF15
ENPP2
TFAP2A
THBS1
BMPR1B
FBN2
ASCL1
SOX13
SYT2
BOC
TPBG
NTRK2
CRB2
CCDC3
POU4F2
POU4F1
ITGA5
PDPN
ADGRV1
TMIGD2
IRX3
UNC5C
POU3F2
SLITRK2
ISG15
WNT7B
ZP3
NTRK1
DLL1
LGR4
MUSTN1

GO:BP
GO:0048514
blood_vessel_morphogenesis
37

ABCC8
TFAP2B
TGFBR3
CEACAM1
LRP2
ANGPT2
HMOX1
JAG1
RGCC
ITGB8
TSPAN12
SMOC2
WNT5A
PROX1
CCN2
TNN
CXCR4
PDGFRA
CEMIP2
ENPP2
THBS1
MFGE8
EPHA2
MEIS1
ACKR3
NTRK2
ANGPT1
ITGA5
TMIGD2
GBX2
NDNF
COL18A1
NR2F2
COL4A1
WNT7B
NRARP
DLL1

GO:BP
GO:0007423
sensory_organ_development
36

CYP26B1
PROM1
TFAP2B
LAMC3
JAG1
TSPAN12
MAN2A1
WNT5A
PROX1
ESRRB
NEUROD4
USP45
NES
PDGFRA
TFAP2A
BMPR1B
FBN2
LGR5
ASCL1
EPHA2
MEIS1
NTRK2
CRB2
THRB
POU4F2
ZIC3
WNT9B
MSX1
ADGRV1
GBX2
ZHX2
ROR1
COL4A1
WNT7B
HES5
DLL1

GO:BP
GO:0072006
nephron_development
19

PROM1
TFAP2B
WWTR1
ANGPT2
JAG1
PODXL
FOXJ1
PDGFRA
EDNRB
GREB1L
ANGPT1
WNT9B
NPHS1
IRX1
IRX3
WNT7B
HES5
DLL1
LGR4

GO:BP
GO:0048523
negative_regulation_of_cellular_process
125

CYP26B1
ABCC8
RHBDF1
TFAP2B
PGLYRP1
PAX7
CD9
WWTR1
ATP1A2
FAS
LTBP1
CNN2
TGFBR3
LMCD1
PLXNA2
THOC1
CEACAM1
LRP2
OPRK1
CHMP2B
NOX4
TESC
DLL3
ANGPT2
TMEM38B
CYP26A1
HMOX1
SALL4
JAG1
RGCC
TOX3
CDK6
LFNG
RNF43
WNT3
RFX4
PRDM13
WNT5A
CBLB
ID3
PROX1
CCN2
ESRRB
TNN
ADRA1A
CDKN1A
KIRREL2
HSPA2
PODXL
AJUBA
FOXJ1
SH3BP4
GDF15
HRC
NES
PDGFRA
EDNRB
TFAP2A
THBS1
SMAD6
BMPR1B
PRDM5
FBN2
ASCL1
EPHA2
MAEL
ILDR2
RGS16
SOX13
MEIS1
ACKR3
HES6
GLRA1
TPBG
RGS20
NTRK2
THRB
CCDC3
POU4F2
POU4F1
SEMA3D
ANGPT1
ITGA5
PDPN
DHRS3
OMA1
LMX1A
EN1
MSX1
ARHGAP25
DAPL1
NUAK2
ADGRV1
TLX3
EN2
RBPMS2
P2RY1
MGMT
IRX1
PKHD1
FOXB1
NDNF
SNX33
FOSL1
NR2F1
OLIG3
IRX3
ZHX2
COL18A1
MUC1
NR2F2
RTN4RL2
ISG15
ZP3
SKOR1
LITAF
H4C3
ENPP1
HES5
NTRK1
NRARP
DLL1
RBM20
LGR4
SKOR2

GO:BP
GO:0050789
regulation_of_biological_process
242

CYP26B1
ABCC8
PROM1
RHBDF1
TEAD3
TFAP2B
PGLYRP1
PAX7
CD9
DDX11
WWTR1
ATP1A2
ABCC2
FAS
LTBP1
PTGER3
TRAF1
CNN2
FGFR3
ABCC9
TGFBR3
LMCD1
WNT8B
PLXNA2
THOC1
CDC14A
CEACAM1
LRP2
OPRK1
CHMP2B
NOX4
TESC
LHX5
DLL3
ANGPT2
CCDC80
ADA2
TMEM38B
TLL2
CYP26A1
PCSK5
PRODH
HMOX1
KCNK10
SALL4
NKAIN4
JAG1
SYNDIG1
RGCC
TOX3
CDK6
ITGB8
LFNG
TSPAN12
EBF3
RNF43
WNT3
P2RX3
CPT1A
RFX4
PRDM13
SMOC2
MAN2A1
SERPINI2
WNT5A
CBLB
PLA2G4A
WLS
ID3
PROX1
CCN2
ELL2
ESRRB
TNN
ADRA1A
KCNJ8
CXCR4
NEUROD4
PDE1B
KCNJ2
CDKN1A
AHNAK
CLYBL
CD70
KIRREL2
HSPA2
TSPAN8
PODXL
USP6
AJUBA
FOXJ1
SH3BP4
DOCK6
ANGPTL8
GDF15
HRC
CASZ1
NES
PRAM1
CNTN6
PDGFRA
CEMIP2
PAX3
PCDH8
EDNRB
ENPP2
TFAP2A
FGFBP2
THBS1
SMAD6
BMPR1B
PRDM5
FBN2
LGR5
ASCL1
LPAR6
HAPLN3
ABCA8
TAF4B
EPHA2
MAEL
ILDR2
RGS16
CREB3L4
SOX13
SYT2
MEIS1
THNSL2
ACKR3
HES6
BOC
EPHA5
MEGF10
GLRA1
TPBG
RGS20
NTRK2
CRB2
THRB
ABTB3
CCDC3
POU4F2
POU4F1
GRM1
SEMA3D
ANGPT1
ZIC3
LDLRAP1
SLC45A3
WNT9B
NPHS1
ITGA5
PDPN
DHRS3
OMA1
OLFML2B
LMX1A
EN1
MSX1
ARHGAP25
DAPL1
INAVA
NUAK2
ADGRV1
EBF1
TLX3
EN2
UNCX
PHOX2A
SLC6A5
CRABP1
RBPMS2
TAC3
TMIGD2
GBX2
RSPO1
GSX1
P2RY1
MGMT
IRX1
PKHD1
NETO2
FOXB1
MBOAT1
NDNF
TNFRSF10C
SNX33
GNG5
FGFBP3
FOSL1
NR2F1
IRX5
OLIG3
IRX3
NHLH2
IL17RA
CA8
ZHX2
GPR157
UNC5C
ASB18
COL18A1
ZNF662
KCNH8
MAML2
POU3F2
ROR1
MUC1
NR2F2
POU3F1
SLITRK2
RTN4RL2
COL4A1
ISG15
WNT7B
HES4
ZP3
SKOR1
LITAF
H4C3
H3C12
ENPP1
HES5
ZXDA
NTRK1
NRARP
DLL1
PPP1R14C
FCGR3A
RBM20
MICB
LGR4
VGLL3
MIR219A2HG
MIR125B1
SKOR2
MIR34AHG
XIST
LINC00472
STON1
TSIX
MUSTN1

GO:BP
GO:0043010
camera-type_eye_development
27

PROM1
TFAP2B
LAMC3
JAG1
TSPAN12
MAN2A1
WNT5A
PROX1
NEUROD4
USP45
NES
PDGFRA
TFAP2A
BMPR1B
FBN2
EPHA2
MEIS1
NTRK2
CRB2
THRB
POU4F2
WNT9B
ZHX2
COL4A1
WNT7B
HES5
DLL1

GO:BP
GO:0007166
cell_surface_receptor_signaling_pathway
89

RHBDF1
TFAP2B
WWTR1
FAS
LTBP1
TRAF1
FGFR3
TGFBR3
WNT8B
PLXNA2
CEACAM1
LRP2
DLL3
ANGPT2
HMOX1
JAG1
CDK6
ITGB8
LFNG
TSPAN12
RNF43
WNT3
P2RX3
RFX4
SMOC2
WNT5A
CBLB
WLS
CCN2
TNN
CXCR4
NEUROD4
CD70
GDF15
PRAM1
CNTN6
PDGFRA
EDNRB
THBS1
SMAD6
BMPR1B
FBN2
LGR5
ASCL1
EPHA2
SOX13
ACKR3
BOC
EPHA5
GLRA1
TPBG
NTRK2
CRB2
ABTB3
CCDC3
GRM1
SEMA3D
ANGPT1
ZIC3
WNT9B
ITGA5
MSX1
ADGRV1
RBPMS2
TMIGD2
RSPO1
P2RY1
TNFRSF10C
FGFBP3
IL17RA
GPR157
UNC5C
MAML2
ROR1
RTN4RL2
COL4A1
ISG15
WNT7B
SKOR1
ENPP1
HES5
NTRK1
NRARP
DLL1
FCGR3A
MICB
LGR4
SKOR2
STON1

GO:BP
GO:0007154
cell_communication
155

CYP26B1
ABCC8
RHBDF1
TEAD3
TFAP2B
WWTR1
ATP1A2
FAS
LTBP1
PTGER3
TRAF1
FGFR3
TGFBR3
LMCD1
WNT8B
PLXNA2
THOC1
CEACAM1
LRP2
OPRK1
NOX4
LHX5
DLL3
ANGPT2
ADA2
TMEM38B
CYP26A1
PCSK5
PRODH
HMOX1
KCNK10
JAG1
CDK6
ITGB8
LFNG
TSPAN12
RNF43
WNT3
P2RX3
CPT1A
RFX4
SMOC2
WNT5A
CBLB
PLA2G4A
WLS
PROX1
CCN2
ESRRB
TNN
ADRA1A
CXCR4
NEUROD4
PDE1B
CDKN1A
CD70
AJUBA
SH3BP4
DOCK6
ANGPTL8
GDF15
HRC
PRAM1
CNTN6
PDGFRA
PCDH8
EDNRB
FGFBP2
THBS1
SMAD6
BMPR1B
FBN2
LGR5
ASCL1
LPAR6
EPHA2
MAEL
ILDR2
RGS16
CREB3L4
SOX13
SYT2
THNSL2
ACKR3
BOC
EPHA5
GLRA1
TPBG
RGS20
NTRK2
CRB2
THRB
ABTB3
CCDC3
POU4F2
POU4F1
GRM1
SEMA3D
ANGPT1
ZIC3
WNT9B
NPHS1
ITGA5
PDPN
DHRS3
OLFML2B
MSX1
ARHGAP25
DAPL1
INAVA
NUAK2
ADGRV1
SLC6A5
CRABP1
RBPMS2
TAC3
TMIGD2
RSPO1
P2RY1
PKHD1
NETO2
FOXB1
NDNF
TNFRSF10C
GNG5
FGFBP3
FOSL1
NR2F1
IRX3
IL17RA
CA8
GPR157
UNC5C
ASB18
MAML2
ROR1
MUC1
NR2F2
RTN4RL2
COL4A1
ISG15
WNT7B
ZP3
SKOR1
LITAF
ENPP1
HES5
NTRK1
NRARP
DLL1
FCGR3A
MICB
LGR4
SKOR2
STON1

GO:BP
GO:0051093
negative_regulation_of_developmental_process
44

ABCC8
PGLYRP1
WWTR1
FGFR3
THOC1
CEACAM1
DLL3
ANGPT2
TLL2
JAG1
RGCC
CDK6
WNT3
WNT5A
ID3
PROX1
ESRRB
TNN
CDKN1A
FOXJ1
GDF15
EDNRB
THBS1
SMAD6
ASCL1
EPHA2
MAEL
MEIS1
POU4F2
SEMA3D
WNT9B
OMA1
LMX1A
MSX1
ADGRV1
TLX3
RBPMS2
IRX3
ZHX2
H4C3
ENPP1
HES5
NRARP
DLL1

GO:BP
GO:0042127
regulation_of_cell_population_proliferation
62

ABCC8
TFAP2B
CD9
WWTR1
CNN2
FGFR3
TGFBR3
CEACAM1
LRP2
NOX4
TESC
LHX5
HMOX1
JAG1
RGCC
CDK6
WNT3
WNT5A
CBLB
PLA2G4A
PROX1
CCN2
TNN
ADRA1A
CDKN1A
CD70
FOXJ1
SH3BP4
NES
PDGFRA
EDNRB
TFAP2A
THBS1
SMAD6
BMPR1B
LGR5
ASCL1
HAPLN3
MEIS1
ACKR3
MEGF10
TPBG
NTRK2
ANGPT1
LDLRAP1
PDPN
MSX1
RBPMS2
TMIGD2
PKHD1
FOSL1
COL18A1
POU3F2
NR2F2
ZP3
HES5
NTRK1
NRARP
DLL1
FCGR3A
SKOR2
MUSTN1

GO:BP
GO:0023052
signaling
152

CYP26B1
ABCC8
RHBDF1
TEAD3
TFAP2B
WWTR1
ATP1A2
FAS
LTBP1
PTGER3
TRAF1
FGFR3
TGFBR3
LMCD1
WNT8B
PLXNA2
THOC1
CEACAM1
LRP2
OPRK1
NOX4
LHX5
DLL3
ANGPT2
ADA2
TMEM38B
CYP26A1
PCSK5
PRODH
HMOX1
KCNK10
JAG1
CDK6
ITGB8
LFNG
TSPAN12
RNF43
WNT3
P2RX3
CPT1A
RFX4
SMOC2
WNT5A
CBLB
PLA2G4A
WLS
PROX1
CCN2
ESRRB
TNN
ADRA1A
CXCR4
NEUROD4
PDE1B
CDKN1A
CD70
AJUBA
SH3BP4
DOCK6
ANGPTL8
GDF15
HRC
PRAM1
CNTN6
PDGFRA
PCDH8
EDNRB
FGFBP2
THBS1
SMAD6
BMPR1B
FBN2
LGR5
ASCL1
LPAR6
EPHA2
MAEL
ILDR2
RGS16
CREB3L4
SOX13
SYT2
THNSL2
ACKR3
BOC
EPHA5
GLRA1
TPBG
RGS20
NTRK2
CRB2
THRB
ABTB3
CCDC3
POU4F2
POU4F1
GRM1
SEMA3D
ANGPT1
ZIC3
WNT9B
NPHS1
ITGA5
PDPN
DHRS3
OLFML2B
MSX1
ARHGAP25
DAPL1
INAVA
NUAK2
ADGRV1
SLC6A5
CRABP1
RBPMS2
TAC3
TMIGD2
RSPO1
P2RY1
PKHD1
NETO2
NDNF
TNFRSF10C
GNG5
FGFBP3
NR2F1
IL17RA
CA8
GPR157
UNC5C
ASB18
MAML2
ROR1
MUC1
NR2F2
RTN4RL2
COL4A1
ISG15
WNT7B
ZP3
SKOR1
LITAF
ENPP1
HES5
NTRK1
NRARP
DLL1
FCGR3A
MICB
LGR4
SKOR2
STON1

GO:BP
GO:0051240
positive_regulation_of_multicellular_organismal_process
62

ABCC8
WWTR1
ATP1A2
PTGER3
TGFBR3
PLXNA2
LRP2
OPRK1
TESC
ANGPT2
HMOX1
JAG1
SYNDIG1
RGCC
ITGB8
WNT3
P2RX3
SMOC2
MAN2A1
WNT5A
CBLB
PLA2G4A
PROX1
CCN2
ESRRB
TNN
ADRA1A
CXCR4
CDKN1A
CD70
FOXJ1
HRC
EDNRB
TFAP2A
THBS1
BMPR1B
FBN2
ASCL1
SOX13
TPBG
NTRK2
CRB2
POU4F2
POU4F1
LDLRAP1
ITGA5
PDPN
OMA1
INAVA
ADGRV1
TMIGD2
P2RY1
IL17RA
POU3F2
SLITRK2
ISG15
ZP3
NTRK1
DLL1
FCGR3A
LGR4
MUSTN1

GO:BP
GO:0048729
tissue_morphogenesis
35

TGFBR3
CEACAM1
LRP2
SALL4
JAG1
WNT3
WNT5A
WLS
PROX1
CXCR4
PODXL
AJUBA
PCDH8
TFAP2A
LGR5
GREB1L
EPHA2
CRB2
POU4F1
ZIC3
WNT9B
ITGA5
PDPN
MSX1
GBX2
IRX1
PKHD1
IRX3
ROR1
COL4A1
WNT7B
HES5
NRARP
DLL1
LGR4

GO:BP
GO:0072009
nephron_epithelium_development
16

PROM1
TFAP2B
WWTR1
JAG1
PODXL
FOXJ1
EDNRB
GREB1L
WNT9B
NPHS1
IRX1
IRX3
WNT7B
HES5
DLL1
LGR4

GO:BP
GO:0048880
sensory_system_development
28

PROM1
TFAP2B
LAMC3
JAG1
TSPAN12
MAN2A1
WNT5A
PROX1
NEUROD4
USP45
NES
PDGFRA
TFAP2A
BMPR1B
FBN2
EPHA2
MEIS1
NTRK2
CRB2
THRB
POU4F2
POU4F1
WNT9B
ZHX2
COL4A1
WNT7B
HES5
DLL1

GO:BP
GO:0009893
positive_regulation_of_metabolic_process
105

CYP26B1
ABCC8
TEAD3
TFAP2B
DDX11
WWTR1
FAS
CNN2
FGFR3
THOC1
LRP2
NOX4
TESC
LHX5
HMOX1
SALL4
JAG1
RGCC
TOX3
CDK6
ITGB8
EBF3
WNT3
CPT1A
RFX4
SMOC2
WNT5A
CBLB
ID3
PROX1
CCN2
ELL2
ESRRB
ADRA1A
CXCR4
CDKN1A
CLYBL
HSPA2
AJUBA
FOXJ1
SH3BP4
ANGPTL8
GDF15
CASZ1
PDGFRA
PAX3
EDNRB
ENPP2
TFAP2A
THBS1
SMAD6
BMPR1B
PRDM5
ASCL1
TAF4B
EPHA2
CREB3L4
MEIS1
EPHA5
NTRK2
THRB
CCDC3
POU4F2
POU4F1
ANGPT1
ZIC3
LDLRAP1
SLC45A3
ITGA5
OMA1
LMX1A
EN1
MSX1
INAVA
EN2
PHOX2A
TMIGD2
GBX2
RSPO1
GSX1
P2RY1
MGMT
SNX33
FOSL1
NR2F1
IRX3
NHLH2
IL17RA
MAML2
POU3F2
ROR1
MUC1
NR2F2
POU3F1
ISG15
ZP3
LITAF
HES5
ZXDA
NTRK1
DLL1
FCGR3A
RBM20
LGR4
MUSTN1

GO:BP
GO:0060041
retina_development_in_camera-type_eye
18

PROM1
TFAP2B
LAMC3
TSPAN12
MAN2A1
PROX1
NEUROD4
USP45
PDGFRA
TFAP2A
BMPR1B
NTRK2
CRB2
THRB
POU4F2
ZHX2
COL4A1
DLL1

GO:BP
GO:0048519
negative_regulation_of_biological_process
141

CYP26B1
ABCC8
RHBDF1
TFAP2B
PGLYRP1
PAX7
CD9
WWTR1
ATP1A2
ABCC2
FAS
LTBP1
PTGER3
CNN2
FGFR3
TGFBR3
LMCD1
PLXNA2
THOC1
CEACAM1
LRP2
OPRK1
CHMP2B
NOX4
TESC
DLL3
ANGPT2
TMEM38B
TLL2
CYP26A1
HMOX1
SALL4
JAG1
RGCC
TOX3
CDK6
ITGB8
LFNG
RNF43
WNT3
RFX4
PRDM13
SERPINI2
WNT5A
CBLB
ID3
PROX1
CCN2
ESRRB
TNN
ADRA1A
CDKN1A
KIRREL2
HSPA2
TSPAN8
PODXL
AJUBA
FOXJ1
SH3BP4
GDF15
HRC
NES
PDGFRA
EDNRB
TFAP2A
THBS1
SMAD6
BMPR1B
PRDM5
FBN2
ASCL1
EPHA2
MAEL
ILDR2
RGS16
SOX13
MEIS1
ACKR3
HES6
GLRA1
TPBG
RGS20
NTRK2
CRB2
THRB
CCDC3
POU4F2
POU4F1
SEMA3D
ANGPT1
WNT9B
ITGA5
PDPN
DHRS3
OMA1
LMX1A
EN1
MSX1
ARHGAP25
DAPL1
NUAK2
ADGRV1
TLX3
EN2
RBPMS2
P2RY1
MGMT
IRX1
PKHD1
FOXB1
NDNF
SNX33
FOSL1
NR2F1
OLIG3
IRX3
ZHX2
COL18A1
POU3F2
MUC1
NR2F2
RTN4RL2
ISG15
ZP3
SKOR1
LITAF
H4C3
ENPP1
HES5
NTRK1
NRARP
DLL1
RBM20
MICB
LGR4
MIR219A2HG
MIR125B1
SKOR2
MIR34AHG
LINC00472
TSIX

GO:BP
GO:0001654
eye_development
27

PROM1
TFAP2B
LAMC3
JAG1
TSPAN12
MAN2A1
WNT5A
PROX1
NEUROD4
USP45
NES
PDGFRA
TFAP2A
BMPR1B
FBN2
EPHA2
MEIS1
NTRK2
CRB2
THRB
POU4F2
WNT9B
ZHX2
COL4A1
WNT7B
HES5
DLL1

GO:BP
GO:0030900
forebrain_development
27

ATP1A2
LRP2
LHX5
CDK6
RFX4
PRDM13
WNT5A
PROX1
CXCR4
ASCL1
EPHA5
NTRK2
POU4F1
ZIC3
LMX1A
MSX1
UNCX
GBX2
GSX1
FOXB1
NDNF
NHLH2
POU3F2
NR2F2
POU3F1
WNT7B
HES5

GO:BP
GO:0072073
kidney_epithelium_development
17

PROM1
TFAP2B
WWTR1
JAG1
PODXL
FOXJ1
EDNRB
SMAD6
GREB1L
WNT9B
NPHS1
IRX1
IRX3
WNT7B
HES5
DLL1
LGR4

GO:BP
GO:0150063
visual_system_development
27

PROM1
TFAP2B
LAMC3
JAG1
TSPAN12
MAN2A1
WNT5A
PROX1
NEUROD4
USP45
NES
PDGFRA
TFAP2A
BMPR1B
FBN2
EPHA2
MEIS1
NTRK2
CRB2
THRB
POU4F2
WNT9B
ZHX2
COL4A1
WNT7B
HES5
DLL1

GO:BP
GO:0007165
signal_transduction
142

CYP26B1
RHBDF1
TEAD3
TFAP2B
WWTR1
ATP1A2
FAS
LTBP1
PTGER3
TRAF1
FGFR3
TGFBR3
LMCD1
WNT8B
PLXNA2
THOC1
CEACAM1
LRP2
OPRK1
NOX4
DLL3
ANGPT2
ADA2
TMEM38B
CYP26A1
PRODH
HMOX1
KCNK10
JAG1
CDK6
ITGB8
LFNG
TSPAN12
RNF43
WNT3
P2RX3
RFX4
SMOC2
WNT5A
CBLB
WLS
PROX1
CCN2
ESRRB
TNN
ADRA1A
CXCR4
NEUROD4
PDE1B
CDKN1A
CD70
AJUBA
SH3BP4
DOCK6
ANGPTL8
GDF15
HRC
PRAM1
CNTN6
PDGFRA
EDNRB
THBS1
SMAD6
BMPR1B
FBN2
LGR5
ASCL1
LPAR6
EPHA2
MAEL
RGS16
CREB3L4
SOX13
THNSL2
ACKR3
BOC
EPHA5
GLRA1
TPBG
RGS20
NTRK2
CRB2
THRB
ABTB3
CCDC3
POU4F2
POU4F1
GRM1
SEMA3D
ANGPT1
ZIC3
WNT9B
NPHS1
ITGA5
PDPN
DHRS3
OLFML2B
MSX1
ARHGAP25
DAPL1
INAVA
NUAK2
ADGRV1
CRABP1
RBPMS2
TAC3
TMIGD2
RSPO1
P2RY1
PKHD1
NETO2
NDNF
TNFRSF10C
GNG5
FGFBP3
NR2F1
IL17RA
CA8
GPR157
UNC5C
ASB18
MAML2
ROR1
MUC1
NR2F2
RTN4RL2
COL4A1
ISG15
WNT7B
ZP3
SKOR1
LITAF
ENPP1
HES5
NTRK1
NRARP
DLL1
FCGR3A
MICB
LGR4
SKOR2
STON1

GO:BP
GO:0051716
cellular_response_to_stimulus
166

CYP26B1
ABCC8
RHBDF1
TEAD3
TFAP2B
CD9
DDX11
WWTR1
ATP1A2
ABCC2
FAS
LTBP1
PTGER3
TRAF1
CNN2
FGFR3
TGFBR3
LMCD1
WNT8B
PLXNA2
THOC1
CEACAM1
LRP2
OPRK1
NOX4
TESC
DLL3
ANGPT2
ADA2
TMEM38B
CYP26A1
PRODH
HMOX1
KCNK10
JAG1
RGCC
CDK6
ITGB8
LFNG
TSPAN12
RNF43
WNT3
P2RX3
CPT1A
RFX4
SMOC2
WNT5A
CBLB
PLA2G4A
WLS
TTF2
ID3
PROX1
CCN2
ESRRB
TNN
ADRA1A
CXCR4
NEUROD4
PDE1B
USP45
KCNJ2
CDKN1A
CD70
HSPA2
AJUBA
SH3BP4
DOCK6
ANGPTL8
GDF15
HRC
PRAM1
CNTN6
PDGFRA
EDNRB
TFAP2A
THBS1
SMAD6
BMPR1B
PRDM5
FBN2
LGR5
ASCL1
LPAR6
EPHA2
MAEL
RGS16
CREB3L4
SOX13
SYT2
THNSL2
ACKR3
BOC
EPHA5
GLRA1
TPBG
RGS20
NTRK2
CRB2
THRB
ABTB3
CCDC3
POU4F2
POU4F1
GRM1
SEMA3D
ANGPT1
PIEZO2
ZIC3
LDLRAP1
WNT9B
NPHS1
ITGA5
PDPN
DHRS3
OMA1
OLFML2B
MSX1
ARHGAP25
DAPL1
INAVA
NUAK2
ADGRV1
CRABP1
RBPMS2
TAC3
TMIGD2
RSPO1
P2RY1
MGMT
PKHD1
NETO2
HSPA6
NDNF
TNFRSF10C
GNG5
FGFBP3
FOSL1
NR2F1
IL17RA
CA8
GPR157
UNC5C
ASB18
MAML2
POU3F2
ROR1
MUC1
NR2F2
RTN4RL2
COL4A1
ISG15
WNT7B
ZP3
SKOR1
LITAF
ENPP1
HES5
NTRK1
NRARP
DLL1
FCGR3A
MICB
LGR4
SKOR2
STON1

GO:BP
GO:0009952
anterior/posterior_pattern_specification
20

PLXNA2
DLL3
PCSK5
LFNG
WNT3
WNT5A
WLS
PCDH8
HES6
CRB2
ZIC3
EN1
MSX1
GBX2
FOXB1
NR2F2
HES4
HES5
NRARP
DLL1

GO:BP
GO:0045165
cell_fate_commitment
22

CYP26B1
TEAD3
WNT8B
JAG1
WNT3
WNT5A
PROX1
NEUROD4
ASCL1
SOX13
POU4F1
WNT9B
PDPN
TLX3
GSX1
OLIG3
POU3F2
NR2F2
WNT7B
HES5
DLL1
MIAT

GO:BP
GO:0001525
angiogenesis
30

ABCC8
CEACAM1
ANGPT2
HMOX1
JAG1
RGCC
ITGB8
TSPAN12
SMOC2
WNT5A
CCN2
TNN
CXCR4
PDGFRA
CEMIP2
ENPP2
THBS1
MFGE8
EPHA2
MEIS1
ACKR3
ANGPT1
ITGA5
TMIGD2
GBX2
NDNF
COL18A1
COL4A1
NRARP
DLL1

GO:BP
GO:0061326
renal_tubule_development
14

PROM1
TFAP2B
WWTR1
JAG1
LGR5
GREB1L
WNT9B
IRX1
IRX3
COL4A1
WNT7B
HES5
DLL1
LGR4

GO:BP
GO:0009790
embryo_development
46

CYP26B1
TEAD3
WNT8B
PLXNA2
LRP2
DLL3
PCSK5
SALL4
JAG1
LFNG
WNT3
MAN2A1
WNT5A
WLS
ID3
PROX1
NES
PDGFRA
PCDH8
TFAP2A
SMAD6
FBN2
LPAR6
EPHA2
MEIS1
CRB2
ANGPT1
ZIC3
WNT9B
ITGA5
EN1
MSX1
EN2
RBPMS2
GBX2
IRX1
FOXB1
FOSL1
IRX5
IRX3
NR2F2
WNT7B
ZP3
HES5
NRARP
DLL1

GO:BP
GO:0031325
positive_regulation_of_cellular_metabolic_process
85

TEAD3
TFAP2B
DDX11
WWTR1
FAS
FGFR3
THOC1
NOX4
TESC
LHX5
HMOX1
SALL4
JAG1
RGCC
TOX3
EBF3
CPT1A
RFX4
SMOC2
WNT5A
PROX1
CCN2
ELL2
ESRRB
ADRA1A
CDKN1A
CLYBL
HSPA2
AJUBA
FOXJ1
SH3BP4
GDF15
CASZ1
PDGFRA
PAX3
EDNRB
ENPP2
TFAP2A
THBS1
SMAD6
BMPR1B
PRDM5
ASCL1
TAF4B
EPHA2
CREB3L4
MEIS1
EPHA5
NTRK2
THRB
POU4F2
POU4F1
ANGPT1
ZIC3
SLC45A3
ITGA5
LMX1A
EN1
MSX1
EN2
PHOX2A
GBX2
RSPO1
GSX1
P2RY1
MGMT
SNX33
FOSL1
NR2F1
IRX3
NHLH2
MAML2
POU3F2
ROR1
MUC1
NR2F2
POU3F1
ZP3
LITAF
HES5
ZXDA
NTRK1
DLL1
RBM20
LGR4

GO:BP
GO:0050896
response_to_stimulus
187

CYP26B1
ABCC8
RHBDF1
TEAD3
TFAP2B
PGLYRP1
CD9
DDX11
WWTR1
ATP1A2
ABCC2
FAS
LTBP1
PTGER3
TRAF1
CNN2
FGFR3
ABCC9
TGFBR3
LMCD1
WNT8B
PLXNA2
THOC1
CEACAM1
LRP2
OPRK1
CHMP2B
NOX4
TESC
DLL3
ANGPT2
CCDC80
ADA2
TMEM38B
CYP26A1
PRODH
HMOX1
KCNK10
JAG1
RGCC
CDK6
ITGB8
LFNG
TSPAN12
RNF43
WNT3
P2RX3
CPT1A
RFX4
SMOC2
WNT5A
CBLB
PLA2G4A
WLS
TTF2
ID3
PROX1
CCN2
ESRRB
TNN
ADRA1A
KCNJ8
CXCR4
NEUROD4
PDE1B
USP45
KCNJ2
CDKN1A
AHNAK
CD70
HSPA2
TSPAN8
AJUBA
FOXJ1
SH3BP4
DOCK6
ANGPTL8
GDF15
HRC
PRAM1
CNTN6
PDGFRA
EDNRB
ENPP2
TFAP2A
THBS1
SMAD6
BMPR1B
PRDM5
FBN2
LGR5
ASCL1
LPAR6
HAPLN3
EPHA2
MAEL
ILDR2
RGS16
HMCN1
CREB3L4
SOX13
SYT2
THNSL2
ACKR3
BOC
EPHA5
GLRA1
TPBG
RGS20
NTRK2
CRB2
THRB
ABTB3
CCDC3
POU4F2
POU4F1
GRM1
SEMA3D
ROBO3
ANGPT1
PIEZO2
ZIC3
LDLRAP1
WNT9B
NPHS1
ITGA5
PDPN
DHRS3
OMA1
OLFML2B
LMX1A
RFTN2
EN1
MSX1
ARHGAP25
DAPL1
INAVA
NUAK2
ADGRV1
CRABP1
RBPMS2
TAC3
TMIGD2
GBX2
RSPO1
P2RY1
MGMT
PKHD1
NETO2
FOXB1
HSPA6
NDNF
TNFRSF10C
GNG5
FGFBP3
CHST1
FOSL1
NR2F1
IRX5
IL17RA
ADAMTSL1
CA8
GPR157
UNC5C
ASB18
MAML2
POU3F2
ROR1
MUC1
NR2F2
RTN4RL2
COL4A1
ISG15
WNT7B
ZP3
SKOR1
LITAF
ENPP1
HES5
NTRK1
NRARP
DLL1
FCGR3A
MICB
LGR4
SKOR2
STON1

GO:BP
GO:0048593
camera-type_eye_morphogenesis
15

PROM1
TFAP2B
JAG1
TSPAN12
MAN2A1
WNT5A
PROX1
TFAP2A
EPHA2
MEIS1
NTRK2
CRB2
THRB
ZHX2
DLL1

GO:BP
GO:0002009
morphogenesis_of_an_epithelium
29

CEACAM1
LRP2
SALL4
JAG1
WNT5A
PROX1
CXCR4
PODXL
AJUBA
PCDH8
TFAP2A
LGR5
GREB1L
EPHA2
ZIC3
WNT9B
ITGA5
PDPN
GBX2
IRX1
PKHD1
IRX3
ROR1
COL4A1
WNT7B
HES5
NRARP
DLL1
LGR4

GO:BP
GO:0048583
regulation_of_response_to_stimulus
103

CYP26B1
ABCC8
RHBDF1
TFAP2B
PGLYRP1
CD9
DDX11
WWTR1
FAS
LTBP1
PTGER3
TRAF1
FGFR3
TGFBR3
LMCD1
THOC1
CEACAM1
LRP2
OPRK1
NOX4
ANGPT2
CYP26A1
HMOX1
JAG1
RGCC
LFNG
TSPAN12
RNF43
WNT3
P2RX3
RFX4
SMOC2
WNT5A
CBLB
PLA2G4A
WLS
PROX1
CCN2
TNN
ADRA1A
CXCR4
TSPAN8
AJUBA
FOXJ1
SH3BP4
GDF15
PRAM1
CNTN6
PDGFRA
EDNRB
THBS1
SMAD6
BMPR1B
FBN2
LGR5
ASCL1
LPAR6
EPHA2
RGS16
SOX13
ACKR3
TPBG
RGS20
NTRK2
CRB2
THRB
CCDC3
POU4F2
GRM1
SEMA3D
ANGPT1
ITGA5
DHRS3
MSX1
ARHGAP25
INAVA
NUAK2
ADGRV1
RBPMS2
TMIGD2
RSPO1
P2RY1
MGMT
PKHD1
NETO2
FGFBP3
IL17RA
ROR1
MUC1
ISG15
WNT7B
ZP3
SKOR1
LITAF
ENPP1
HES5
NTRK1
NRARP
DLL1
FCGR3A
MICB
LGR4
SKOR2

GO:BP
GO:0010646
regulation_of_cell_communication
92

CYP26B1
ABCC8
RHBDF1
TFAP2B
WWTR1
ATP1A2
FAS
LTBP1
TRAF1
FGFR3
TGFBR3
LMCD1
THOC1
CEACAM1
LRP2
OPRK1
NOX4
CYP26A1
HMOX1
JAG1
ITGB8
LFNG
TSPAN12
RNF43
WNT3
P2RX3
CPT1A
RFX4
SMOC2
WNT5A
CBLB
WLS
PROX1
CCN2
TNN
ADRA1A
CXCR4
AJUBA
SH3BP4
GDF15
HRC
CNTN6
PDGFRA
PCDH8
THBS1
SMAD6
BMPR1B
FBN2
LGR5
ASCL1
LPAR6
EPHA2
RGS16
SOX13
ACKR3
EPHA5
GLRA1
TPBG
RGS20
NTRK2
CRB2
THRB
CCDC3
GRM1
ANGPT1
ITGA5
DHRS3
MSX1
ARHGAP25
INAVA
NUAK2
ADGRV1
RBPMS2
RSPO1
P2RY1
PKHD1
NETO2
FGFBP3
IRX3
ROR1
MUC1
ISG15
WNT7B
SKOR1
LITAF
ENPP1
HES5
NTRK1
NRARP
DLL1
LGR4
SKOR2

GO:BP
GO:0030855
epithelial_cell_differentiation
34

CYP26B1
PROM1
WWTR1
CEACAM1
TMEM38B
JAG1
CDK6
CPT1A
WNT5A
ID3
PROX1
CXCR4
PODXL
FOXJ1
EDNRB
ASCL1
RHCG
EPHA2
THRB
WNT9B
NPHS1
PDPN
MGMT
PKHD1
FOXB1
COL18A1
POU3F2
NR2F2
POU3F1
COL4A1
WNT7B
HES5
NTRK1
DLL1

GO:BP
GO:0016477
cell_migration
53

ABCC8
RHBDF1
CD9
LAMC3
CNN2
TGFBR3
PLXNA2
CEACAM1
ANGPT2
HMOX1
JAG1
RGCC
ITGB8
SMOC2
WNT5A
PROX1
CCN2
TNN
CXCR4
NEUROD4
USP45
PODXL
AJUBA
FOXJ1
PDGFRA
EDNRB
ENPP2
THBS1
ASCL1
EPHA2
ACKR3
MEGF10
TPBG
NTRK2
CRB2
POU4F1
SEMA3D
ANGPT1
ITGA5
PDPN
TLX3
GBX2
P2RY1
FOXB1
NDNF
OLIG3
NHLH2
IL17RA
UNC5C
POU3F2
NR2F2
ZP3
STON1

GO:BP
GO:0060042
retina_morphogenesis_in_camera-type_eye
11

PROM1
TFAP2B
TSPAN12
MAN2A1
PROX1
TFAP2A
NTRK2
CRB2
THRB
ZHX2
DLL1

GO:BP
GO:0022603
regulation_of_anatomical_structure_morphogenesis
38

ABCC8
PLXNA2
CEACAM1
ANGPT2
HMOX1
JAG1
RGCC
ITGB8
LFNG
TSPAN12
WNT3
SMOC2
WNT5A
TNN
CXCR4
FOXJ1
CEMIP2
ENPP2
THBS1
EPHA2
SYT2
NTRK2
CRB2
POU4F2
SEMA3D
WNT9B
ITGA5
PDPN
OMA1
MSX1
TMIGD2
P2RY1
PKHD1
POU3F2
ROR1
DLL1
LGR4
SKOR2

GO:BP
GO:0006357
regulation_of_transcription_by_RNA_polymerase_II
75

TEAD3
TFAP2B
PAX7
WWTR1
LMCD1
LHX5
HMOX1
SALL4
JAG1
RGCC
TOX3
CDK6
EBF3
RFX4
PRDM13
WNT5A
ID3
PROX1
ELL2
ESRRB
NEUROD4
AJUBA
FOXJ1
CASZ1
PAX3
EDNRB
TFAP2A
BMPR1B
PRDM5
ASCL1
TAF4B
MAEL
CREB3L4
SOX13
MEIS1
HES6
THRB
POU4F2
POU4F1
ZIC3
LMX1A
EN1
MSX1
EBF1
TLX3
EN2
UNCX
PHOX2A
GBX2
GSX1
P2RY1
IRX1
FOXB1
FOSL1
NR2F1
IRX5
OLIG3
IRX3
NHLH2
ZHX2
ZNF662
MAML2
POU3F2
MUC1
NR2F2
POU3F1
HES4
SKOR1
LITAF
HES5
ZXDA
NRARP
DLL1
VGLL3
SKOR2

GO:BP
GO:0048592
eye_morphogenesis
16

PROM1
TFAP2B
JAG1
TSPAN12
MAN2A1
WNT5A
PROX1
TFAP2A
FBN2
EPHA2
MEIS1
NTRK2
CRB2
THRB
ZHX2
DLL1

GO:BP
GO:0032835
glomerulus_development
11

PROM1
WWTR1
ANGPT2
JAG1
PODXL
FOXJ1
PDGFRA
EDNRB
ANGPT1
NPHS1
LGR4

GO:BP
GO:0009966
regulation_of_signal_transduction
83

CYP26B1
RHBDF1
TFAP2B
WWTR1
FAS
LTBP1
TRAF1
FGFR3
TGFBR3
LMCD1
THOC1
CEACAM1
LRP2
OPRK1
NOX4
CYP26A1
HMOX1
JAG1
LFNG
TSPAN12
RNF43
WNT3
P2RX3
RFX4
SMOC2
WNT5A
CBLB
WLS
PROX1
CCN2
TNN
ADRA1A
CXCR4
AJUBA
SH3BP4
GDF15
CNTN6
PDGFRA
THBS1
SMAD6
BMPR1B
FBN2
LGR5
ASCL1
LPAR6
EPHA2
RGS16
SOX13
ACKR3
TPBG
RGS20
NTRK2
CRB2
THRB
CCDC3
GRM1
ANGPT1
ITGA5
DHRS3
MSX1
ARHGAP25
INAVA
NUAK2
ADGRV1
RBPMS2
RSPO1
P2RY1
PKHD1
NETO2
FGFBP3
ROR1
MUC1
ISG15
WNT7B
SKOR1
LITAF
ENPP1
HES5
NTRK1
NRARP
DLL1
LGR4
SKOR2

GO:BP
GO:0010604
positive_regulation_of_macromolecule_metabolic_process
93

CYP26B1
ABCC8
TEAD3
TFAP2B
DDX11
WWTR1
FAS
CNN2
FGFR3
THOC1
LRP2
NOX4
TESC
LHX5
HMOX1
SALL4
JAG1
RGCC
TOX3
CDK6
ITGB8
EBF3
WNT3
RFX4
SMOC2
WNT5A
CBLB
ID3
PROX1
CCN2
ELL2
ESRRB
ADRA1A
CDKN1A
HSPA2
AJUBA
FOXJ1
ANGPTL8
GDF15
CASZ1
PAX3
EDNRB
ENPP2
TFAP2A
THBS1
SMAD6
BMPR1B
PRDM5
ASCL1
TAF4B
CREB3L4
MEIS1
NTRK2
THRB
POU4F2
POU4F1
ANGPT1
ZIC3
ITGA5
LMX1A
EN1
MSX1
INAVA
EN2
PHOX2A
TMIGD2
GBX2
RSPO1
GSX1
P2RY1
MGMT
SNX33
FOSL1
NR2F1
IRX3
NHLH2
IL17RA
MAML2
POU3F2
MUC1
NR2F2
POU3F1
ISG15
ZP3
LITAF
HES5
ZXDA
NTRK1
DLL1
FCGR3A
RBM20
LGR4
MUSTN1

GO:BP
GO:0048666
neuron_development
44

PLXNA2
LRP2
WNT3
WNT5A
TNN
CXCR4
NEUROD4
CNTN6
EDNRB
BMPR1B
ASCL1
EPHA2
SYT2
BOC
EPHA5
TPBG
NTRK2
CRB2
KLHL1
THRB
POU4F2
POU4F1
SEMA3D
ROBO3
LMX1A
EN1
ADGRV1
EN2
GBX2
FOXB1
MBOAT1
NDNF
NR2F1
NHLH2
ADAMTSL1
UNC5C
POU3F2
ROR1
SLITRK2
RTN4RL2
WNT7B
HES5
NTRK1
SKOR2

GO:BP
GO:0007167
enzyme-linked_receptor_protein_signaling_pathway
40

RHBDF1
TFAP2B
WWTR1
LTBP1
FGFR3
TGFBR3
CEACAM1
LRP2
ANGPT2
ITGB8
TSPAN12
SMOC2
WNT5A
CBLB
CCN2
GDF15
PDGFRA
THBS1
SMAD6
BMPR1B
FBN2
EPHA2
EPHA5
NTRK2
CRB2
ABTB3
ANGPT1
ITGA5
MSX1
RBPMS2
FGFBP3
ROR1
COL4A1
SKOR1
ENPP1
HES5
NTRK1
DLL1
SKOR2
STON1

GO:BP
GO:0045596
negative_regulation_of_cell_differentiation
32

ABCC8
PGLYRP1
WWTR1
CEACAM1
DLL3
JAG1
CDK6
WNT3
WNT5A
ID3
PROX1
ESRRB
TNN
FOXJ1
EDNRB
SMAD6
ASCL1
MEIS1
POU4F2
SEMA3D
LMX1A
MSX1
ADGRV1
TLX3
RBPMS2
IRX3
ZHX2
H4C3
ENPP1
HES5
NRARP
DLL1

GO:BP
GO:0072070
loop_of_Henle_development
6

JAG1
IRX1
IRX3
WNT7B
HES5
DLL1

GO:BP
GO:0032989
cellular_component_morphogenesis
35

CD9
PLXNA2
LRP2
WNT3
WNT5A
PROX1
TNN
CXCR4
NES
CNTN6
PDGFRA
ENPP2
BMPR1B
EPHA2
SYT2
BOC
EPHA5
TPBG
NTRK2
POU4F2
POU4F1
SEMA3D
ROBO3
PGM5
PDPN
LMX1A
GBX2
FOXB1
ADAMTSL1
UNC5C
POU3F2
SLITRK2
WNT7B
NTRK1
SKOR2

GO:BP
GO:0006366
transcription_by_RNA_polymerase_II
76

TEAD3
TFAP2B
PAX7
WWTR1
LMCD1
LHX5
HMOX1
SALL4
JAG1
RGCC
TOX3
CDK6
EBF3
RFX4
PRDM13
WNT5A
TTF2
ID3
PROX1
ELL2
ESRRB
NEUROD4
AJUBA
FOXJ1
CASZ1
PAX3
EDNRB
TFAP2A
BMPR1B
PRDM5
ASCL1
TAF4B
MAEL
CREB3L4
SOX13
MEIS1
HES6
THRB
POU4F2
POU4F1
ZIC3
LMX1A
EN1
MSX1
EBF1
TLX3
EN2
UNCX
PHOX2A
GBX2
GSX1
P2RY1
IRX1
FOXB1
FOSL1
NR2F1
IRX5
OLIG3
IRX3
NHLH2
ZHX2
ZNF662
MAML2
POU3F2
MUC1
NR2F2
POU3F1
HES4
SKOR1
LITAF
HES5
ZXDA
NRARP
DLL1
VGLL3
SKOR2

GO:BP
GO:0090596
sensory_organ_morphogenesis
20

CYP26B1
PROM1
TFAP2B
JAG1
TSPAN12
MAN2A1
WNT5A
PROX1
TFAP2A
FBN2
EPHA2
MEIS1
NTRK2
CRB2
THRB
ZIC3
MSX1
GBX2
ZHX2
DLL1

GO:BP
GO:0045944
positive_regulation_of_transcription_by_RNA_polymerase_II
46

TEAD3
TFAP2B
WWTR1
SALL4
JAG1
RGCC
TOX3
EBF3
RFX4
WNT5A
PROX1
ELL2
ESRRB
FOXJ1
CASZ1
PAX3
TFAP2A
BMPR1B
PRDM5
ASCL1
TAF4B
CREB3L4
MEIS1
THRB
POU4F2
POU4F1
ZIC3
LMX1A
EN1
MSX1
EN2
PHOX2A
GBX2
GSX1
P2RY1
FOSL1
NR2F1
IRX3
NHLH2
MAML2
POU3F2
MUC1
NR2F2
LITAF
HES5
DLL1

GO:BP
GO:0035282
segmentation
13

PLXNA2
DLL3
LFNG
WNT5A
PCDH8
CRB2
ZIC3
IRX1
FOXB1
IRX3
HES5
NRARP
DLL1

GO:BP
GO:0001656
metanephros_development
12

TFAP2B
WWTR1
ID3
FOXJ1
PDGFRA
GREB1L
WNT9B
IRX1
IRX3
WNT7B
HES5
LGR4

GO:BP
GO:0030901
midbrain_development
12

WNT3
WNT5A
WLS
SYPL2
WNT9B
LMX1A
EN1
MSX1
EN2
PHOX2A
FOXB1
POTEE

GO:BP
GO:0001501
skeletal_system_development
28

CYP26B1
FGFR3
DLL3
TMEM38B
PCSK5
ITGB8
WNT5A
CCN2
TNN
PDGFRA
TFAP2A
BMPR1B
FBN2
HAPLN3
EPHA2
MEIS1
ZIC3
ADAMTS4
WNT9B
DHRS3
EN1
MSX1
UNCX
THBS3
IRX5
WNT7B
HES5
MUSTN1

GO:BP
GO:0009967
positive_regulation_of_signal_transduction
52

FAS
TRAF1
FGFR3
TGFBR3
LMCD1
LRP2
OPRK1
NOX4
HMOX1
JAG1
LFNG
WNT3
P2RX3
SMOC2
WNT5A
WLS
PROX1
CCN2
ADRA1A
CXCR4
AJUBA
GDF15
CNTN6
PDGFRA
THBS1
BMPR1B
LGR5
ASCL1
LPAR6
ACKR3
TPBG
NTRK2
CRB2
THRB
GRM1
ANGPT1
ITGA5
MSX1
INAVA
ADGRV1
RSPO1
P2RY1
FGFBP3
ROR1
WNT7B
LITAF
HES5
NTRK1
NRARP
DLL1
LGR4
SKOR2

GO:BP
GO:0000902
cell_morphogenesis
39

LAMC3
PLXNA2
LRP2
NOX4
WNT3
WNT5A
PROX1
TNN
CXCR4
NES
CNTN6
ENPP2
BMPR1B
EPHA2
MAEL
SYT2
BOC
EPHA5
TPBG
NTRK2
POU4F2
POU4F1
SEMA3D
ROBO3
PDPN
LMX1A
MSX1
GBX2
P2RY1
PKHD1
FOXB1
ADAMTSL1
UNC5C
COL18A1
POU3F2
SLITRK2
WNT7B
NTRK1
SKOR2

GO:BP
GO:0021761
limbic_system_development
13

ATP1A2
LHX5
CDK6
PRDM13
PROX1
EPHA5
ZIC3
LMX1A
GSX1
FOXB1
NDNF
NHLH2
POU3F2

GO:BP
GO:0030902
hindbrain_development
15

PLXNA2
LHX5
WLS
PROX1
ASCL1
KLHL1
POU4F1
LMX1A
EN1
EN2
PHOX2A
GBX2
NHLH2
DLL1
SKOR2

GO:BP
GO:0023051
regulation_of_signaling
89

CYP26B1
ABCC8
RHBDF1
TFAP2B
WWTR1
ATP1A2
FAS
LTBP1
TRAF1
FGFR3
TGFBR3
LMCD1
THOC1
CEACAM1
LRP2
OPRK1
NOX4
CYP26A1
HMOX1
JAG1
ITGB8
LFNG
TSPAN12
RNF43
WNT3
P2RX3
CPT1A
RFX4
SMOC2
WNT5A
CBLB
WLS
PROX1
CCN2
TNN
ADRA1A
CXCR4
AJUBA
SH3BP4
GDF15
CNTN6
PDGFRA
PCDH8
THBS1
SMAD6
BMPR1B
FBN2
LGR5
ASCL1
LPAR6
EPHA2
RGS16
SOX13
ACKR3
EPHA5
TPBG
RGS20
NTRK2
CRB2
THRB
CCDC3
GRM1
ANGPT1
ITGA5
DHRS3
MSX1
ARHGAP25
INAVA
NUAK2
ADGRV1
RBPMS2
RSPO1
P2RY1
PKHD1
NETO2
FGFBP3
ROR1
MUC1
ISG15
WNT7B
SKOR1
LITAF
ENPP1
HES5
NTRK1
NRARP
DLL1
LGR4
SKOR2

GO:BP
GO:0048858
cell_projection_morphogenesis
31

PLXNA2
LRP2
WNT3
WNT5A
TNN
CXCR4
NES
CNTN6
ENPP2
BMPR1B
EPHA2
SYT2
BOC
EPHA5
TPBG
NTRK2
POU4F2
POU4F1
SEMA3D
ROBO3
PDPN
LMX1A
GBX2
FOXB1
ADAMTSL1
UNC5C
POU3F2
SLITRK2
WNT7B
NTRK1
SKOR2

GO:BP
GO:0070848
response_to_growth_factor
32

TFAP2B
LTBP1
FGFR3
TGFBR3
LRP2
ANGPT2
ITGB8
TSPAN12
SMOC2
WNT5A
CCN2
GDF15
PDGFRA
THBS1
SMAD6
BMPR1B
FBN2
ASCL1
EPHA2
NTRK2
CRB2
ITGA5
MSX1
RBPMS2
P2RY1
NDNF
FGFBP3
SKOR1
HES5
NTRK1
DLL1
SKOR2

GO:BP
GO:0009891
positive_regulation_of_biosynthetic_process
63

TEAD3
TFAP2B
DDX11
WWTR1
THOC1
NOX4
TESC
LHX5
SALL4
JAG1
RGCC
TOX3
EBF3
RFX4
SMOC2
WNT5A
PROX1
CCN2
ELL2
ESRRB
AJUBA
FOXJ1
CASZ1
PAX3
TFAP2A
THBS1
SMAD6
BMPR1B
PRDM5
ASCL1
TAF4B
CREB3L4
MEIS1
THRB
CCDC3
POU4F2
POU4F1
ZIC3
SLC45A3
LMX1A
EN1
MSX1
EN2
PHOX2A
GBX2
GSX1
P2RY1
FOSL1
NR2F1
IRX3
NHLH2
MAML2
POU3F2
MUC1
NR2F2
POU3F1
ZP3
LITAF
HES5
ZXDA
DLL1
LGR4
MUSTN1

GO:BP
GO:0003008
system_process
67

ABCC8
WWTR1
ATP1A2
ABCC2
LAMC3
PTGER3
ABCC9
LMCD1
CDC14A
CEACAM1
LRP2
OPRK1
CHMP2B
NOX4
TMEM38B
PCSK5
HMOX1
KCNK10
P2RX3
CCN2
ADRA1A
SCPEP1
KCNJ8
CXCR4
PDE1B
KCNJ2
HRC
SLC6A8
PAX3
EDNRB
TFAP2A
ASCL1
RGS16
HMCN1
GLRA1
TPBG
NTRK2
CRB2
THRB
POU4F2
POU4F1
GRM1
ANGPT1
PIEZO2
SHROOM4
ITGA5
DHRS3
LMX1A
EN1
DAPL1
INAVA
ADGRV1
TLX3
TAC3
P2RY1
NETO2
FOXB1
IGDCC3
FGFBP3
FOSL1
IRX5
IRX3
COL18A1
ROR1
NR2F2
NTRK1
DLL1

GO:BP
GO:0072080
nephron_tubule_development
12

PROM1
TFAP2B
WWTR1
JAG1
GREB1L
WNT9B
IRX1
IRX3
WNT7B
HES5
DLL1
LGR4

GO:BP
GO:0048870
cell_motility
55

ABCC8
RHBDF1
CD9
LAMC3
CNN2
TGFBR3
PLXNA2
CEACAM1
ANGPT2
HMOX1
JAG1
RGCC
CDK6
ITGB8
SMOC2
WNT5A
PROX1
CCN2
TNN
CXCR4
NEUROD4
USP45
PODXL
AJUBA
FOXJ1
PDGFRA
EDNRB
ENPP2
THBS1
ASCL1
EPHA2
ACKR3
MEGF10
TPBG
NTRK2
CRB2
POU4F1
SEMA3D
ANGPT1
ITGA5
PDPN
TLX3
GBX2
P2RY1
FOXB1
SLC9C1
NDNF
OLIG3
NHLH2
IL17RA
UNC5C
POU3F2
NR2F2
ZP3
STON1

GO:BP
GO:0003407
neural_retina_development
11

PROM1
TFAP2B
TSPAN12
NEUROD4
USP45
TFAP2A
NTRK2
CRB2
THRB
POU4F2
ZHX2

GO:BP
GO:0045893
positive_regulation_of_DNA-templated_transcription
55

TEAD3
TFAP2B
DDX11
WWTR1
THOC1
TESC
LHX5
SALL4
JAG1
RGCC
TOX3
EBF3
RFX4
WNT5A
PROX1
ELL2
ESRRB
FOXJ1
CASZ1
PAX3
TFAP2A
SMAD6
BMPR1B
PRDM5
ASCL1
TAF4B
CREB3L4
MEIS1
THRB
POU4F2
POU4F1
ZIC3
LMX1A
EN1
MSX1
EN2
PHOX2A
GBX2
GSX1
P2RY1
FOSL1
NR2F1
IRX3
NHLH2
MAML2
POU3F2
MUC1
NR2F2
POU3F1
ZP3
LITAF
HES5
ZXDA
DLL1
LGR4

GO:BP
GO:1903508
positive_regulation_of_nucleic_acid-templated_transcription
55

TEAD3
TFAP2B
DDX11
WWTR1
THOC1
TESC
LHX5
SALL4
JAG1
RGCC
TOX3
EBF3
RFX4
WNT5A
PROX1
ELL2
ESRRB
FOXJ1
CASZ1
PAX3
TFAP2A
SMAD6
BMPR1B
PRDM5
ASCL1
TAF4B
CREB3L4
MEIS1
THRB
POU4F2
POU4F1
ZIC3
LMX1A
EN1
MSX1
EN2
PHOX2A
GBX2
GSX1
P2RY1
FOSL1
NR2F1
IRX3
NHLH2
MAML2
POU3F2
MUC1
NR2F2
POU3F1
ZP3
LITAF
HES5
ZXDA
DLL1
LGR4

GO:BP
GO:1902680
positive_regulation_of_RNA_biosynthetic_process
55

TEAD3
TFAP2B
DDX11
WWTR1
THOC1
TESC
LHX5
SALL4
JAG1
RGCC
TOX3
EBF3
RFX4
WNT5A
PROX1
ELL2
ESRRB
FOXJ1
CASZ1
PAX3
TFAP2A
SMAD6
BMPR1B
PRDM5
ASCL1
TAF4B
CREB3L4
MEIS1
THRB
POU4F2
POU4F1
ZIC3
LMX1A
EN1
MSX1
EN2
PHOX2A
GBX2
GSX1
P2RY1
FOSL1
NR2F1
IRX3
NHLH2
MAML2
POU3F2
MUC1
NR2F2
POU3F1
ZP3
LITAF
HES5
ZXDA
DLL1
LGR4

GO:BP
GO:0032990
cell_part_morphogenesis
31

PLXNA2
LRP2
WNT3
WNT5A
TNN
CXCR4
NES
CNTN6
ENPP2
BMPR1B
EPHA2
SYT2
BOC
EPHA5
TPBG
NTRK2
POU4F2
POU4F1
SEMA3D
ROBO3
PDPN
LMX1A
GBX2
FOXB1
ADAMTSL1
UNC5C
POU3F2
SLITRK2
WNT7B
NTRK1
SKOR2

GO:BP
GO:0030155
regulation_of_cell_adhesion
33
CD9
PLXNA2
CEACAM1
TESC
ANGPT2
CCDC80
JAG1
RGCC
CDK6
SERPINI2
WNT5A
CBLB
TNN
CXCR4
CD70
PODXL
FOXJ1
PCDH8
THBS1
EPHA2
ILDR2
SOX13
MEGF10
ANGPT1
ITGA5
PDPN
TMIGD2
PKHD1
NDNF
MUC1
ZP3
NRARP
DLL1

GO:BP
GO:0010557
positive_regulation_of_macromolecule_biosynthetic_process
59
TEAD3
TFAP2B
DDX11
WWTR1
THOC1
NOX4
TESC
LHX5
SALL4
JAG1
RGCC
TOX3
EBF3
RFX4
SMOC2
WNT5A
PROX1
ELL2
ESRRB
FOXJ1
CASZ1
PAX3
TFAP2A
THBS1
SMAD6
BMPR1B
PRDM5
ASCL1
TAF4B
CREB3L4
MEIS1
THRB
POU4F2
POU4F1
ZIC3
LMX1A
EN1
MSX1
EN2
PHOX2A
GBX2
GSX1
P2RY1
FOSL1
NR2F1
IRX3
NHLH2
MAML2
POU3F2
MUC1
NR2F2
POU3F1
ZP3
LITAF
HES5
ZXDA
DLL1
LGR4
MUSTN1

GO:BP
GO:0120039
plasma_membrane_bounded_cell_projection_morphogenesis
30
PLXNA2
LRP2
WNT3
WNT5A
TNN
CXCR4
CNTN6
ENPP2
BMPR1B
EPHA2
SYT2
BOC
EPHA5
TPBG
NTRK2
POU4F2
POU4F1
SEMA3D
ROBO3
PDPN
LMX1A
GBX2
FOXB1
ADAMTSL1
UNC5C
POU3F2
SLITRK2
WNT7B
NTRK1
SKOR2

GO:BP
GO:0021953
central_nervous_system_neuron_differentiation
15
LHX5
PROX1
BMPR1B
ASCL1
NTRK2
POU4F1
LMX1A
PHOX2A
GBX2
GSX1
NDNF
OLIG3
NHLH2
HES5
SKOR2

GO:BP
GO:0048585
negative_regulation_of_response_to_stimulus
52
CYP26B1
ABCC8
PGLYRP1
CD9
WWTR1
LTBP1
TGFBR3
THOC1
CEACAM1
LRP2
ANGPT2
CYP26A1
HMOX1
LFNG
RNF43
WNT3
RFX4
WNT5A
CBLB
TNN
ADRA1A
TSPAN8
AJUBA
FOXJ1
SH3BP4
GDF15
PDGFRA
THBS1
SMAD6
FBN2
EPHA2
RGS16
SOX13
ACKR3
TPBG
RGS20
CCDC3
SEMA3D
DHRS3
ARHGAP25
RBPMS2
PKHD1
MUC1
ISG15
SKOR1
LITAF
ENPP1
NRARP
DLL1
MICB
LGR4
SKOR2

GO:BP
GO:0008285
negative_regulation_of_cell_population_proliferation
31
ABCC8
TFAP2B
CD9
TGFBR3
CEACAM1
NOX4
TESC
HMOX1
RGCC
CDK6
WNT5A
CBLB
PROX1
TNN
ADRA1A
CDKN1A
FOXJ1
SH3BP4
TFAP2A
THBS1
SMAD6
BMPR1B
ACKR3
TPBG
PDPN
MSX1
FOSL1
COL18A1
NR2F2
NTRK1
DLL1

GO:BP
GO:0051173
positive_regulation_of_nitrogen_compound_metabolic_process
82
TEAD3
TFAP2B
DDX11
WWTR1
FAS
FGFR3
THOC1
LRP2
NOX4
TESC
LHX5
SALL4
JAG1
RGCC
TOX3
EBF3
RFX4
SMOC2
WNT5A
CBLB
PROX1
CCN2
ELL2
ESRRB
ADRA1A
CDKN1A
CLYBL
HSPA2
AJUBA
FOXJ1
ANGPTL8
GDF15
CASZ1
PAX3
EDNRB
ENPP2
TFAP2A
THBS1
SMAD6
BMPR1B
PRDM5
ASCL1
TAF4B
CREB3L4
MEIS1
NTRK2
THRB
POU4F2
POU4F1
ANGPT1
ZIC3
ITGA5
LMX1A
EN1
MSX1
INAVA
EN2
PHOX2A
GBX2
RSPO1
GSX1
P2RY1
MGMT
SNX33
FOSL1
NR2F1
IRX3
NHLH2
MAML2
POU3F2
MUC1
NR2F2
POU3F1
ZP3
LITAF
HES5
ZXDA
NTRK1
DLL1
RBM20
LGR4
MUSTN1

GO:BP
GO:0043009
chordate_embryonic_development
30
PLXNA2
LRP2
DLL3
PCSK5
SALL4
LFNG
MAN2A1
WNT5A
PROX1
PDGFRA
PCDH8
TFAP2A
LPAR6
EPHA2
CRB2
ANGPT1
ZIC3
WNT9B
EN1
MSX1
GBX2
FOXB1
FOSL1
IRX5
NR2F2
WNT7B
ZP3
HES5
NRARP
DLL1

GO:BP
GO:0009719
response_to_endogenous_stimulus
54
ABCC8
TFAP2B
CD9
DDX11
ATP1A2
LTBP1
FGFR3
TGFBR3
CEACAM1
LRP2
OPRK1
ANGPT2
TMEM38B
HMOX1
ITGB8
TSPAN12
P2RX3
SMOC2
WNT5A
CCN2
ESRRB
ADRA1A
CXCR4
SH3BP4
GDF15
PDGFRA
EDNRB
THBS1
SMAD6
BMPR1B
FBN2
ASCL1
HAPLN3
EPHA2
GLRA1
NTRK2
CRB2
THRB
POU4F2
ITGA5
MSX1
RBPMS2
P2RY1
NDNF
FGFBP3
FOSL1
COL4A1
WNT7B
SKOR1
ENPP1
HES5
NTRK1
DLL1
SKOR2

GO:BP
GO:0031328
positive_regulation_of_cellular_biosynthetic_process
60
TEAD3
TFAP2B
DDX11
WWTR1
THOC1
NOX4
TESC
LHX5
SALL4
JAG1
RGCC
TOX3
EBF3
RFX4
SMOC2
WNT5A
PROX1
ELL2
ESRRB
AJUBA
FOXJ1
CASZ1
PAX3
TFAP2A
THBS1
SMAD6
BMPR1B
PRDM5
ASCL1
TAF4B
CREB3L4
MEIS1
THRB
POU4F2
POU4F1
ZIC3
SLC45A3
LMX1A
EN1
MSX1
EN2
PHOX2A
GBX2
GSX1
P2RY1
FOSL1
NR2F1
IRX3
NHLH2
MAML2
POU3F2
MUC1
NR2F2
POU3F1
ZP3
LITAF
HES5
ZXDA
DLL1
LGR4

GO:BP
GO:0040011
locomotion
46
ABCC8
CD9
ATP1A2
CNN2
PLXNA2
CEACAM1
OPRK1
ANGPT2
HMOX1
JAG1
RGCC
CDK6
WNT3
SMOC2
WNT5A
PROX1
TNN
CXCR4
PODXL
AJUBA
CNTN6
PDGFRA
EDNRB
ENPP2
THBS1
BMPR1B
EPHA2
ACKR3
BOC
EPHA5
TPBG
POU4F2
SEMA3D
ROBO3
ANGPT1
ITGA5
PDPN
LMX1A
GBX2
FOSL1
IL17RA
ADAMTSL1
UNC5C
NR2F2
ZP3
NTRK1

GO:BP
GO:0045665
negative_regulation_of_neuron_differentiation
10

JAG1
ASCL1
MEIS1
POU4F2
LMX1A
TLX3
IRX3
ZHX2
HES5
DLL1

GO:BP
GO:0006355
regulation_of_DNA-templated_transcription
87

TEAD3
TFAP2B
PAX7
DDX11
WWTR1
TRAF1
LMCD1
THOC1
TESC
LHX5
HMOX1
SALL4
JAG1
RGCC
TOX3
CDK6
EBF3
RFX4
PRDM13
WNT5A
ID3
PROX1
ELL2
ESRRB
NEUROD4
AJUBA
FOXJ1
CASZ1
PAX3
EDNRB
TFAP2A
SMAD6
BMPR1B
PRDM5
ASCL1
TAF4B
MAEL
CREB3L4
SOX13
MEIS1
HES6
EPHA5
THRB
POU4F2
POU4F1
ZIC3
LMX1A
EN1
MSX1
EBF1
TLX3
EN2
UNCX
PHOX2A
GBX2
GSX1
P2RY1
IRX1
PKHD1
FOXB1
FOSL1
NR2F1
IRX5
OLIG3
IRX3
NHLH2
ZHX2
ZNF662
MAML2
POU3F2
ROR1
MUC1
NR2F2
POU3F1
HES4
ZP3
SKOR1
LITAF
ENPP1
HES5
ZXDA
NTRK1
NRARP
DLL1
LGR4
VGLL3
SKOR2

GO:BP
GO:1903506
regulation_of_nucleic_acid-templated_transcription
87

TEAD3
TFAP2B
PAX7
DDX11
WWTR1
TRAF1
LMCD1
THOC1
TESC
LHX5
HMOX1
SALL4
JAG1
RGCC
TOX3
CDK6
EBF3
RFX4
PRDM13
WNT5A
ID3
PROX1
ELL2
ESRRB
NEUROD4
AJUBA
FOXJ1
CASZ1
PAX3
EDNRB
TFAP2A
SMAD6
BMPR1B
PRDM5
ASCL1
TAF4B
MAEL
CREB3L4
SOX13
MEIS1
HES6
EPHA5
THRB
POU4F2
POU4F1
ZIC3
LMX1A
EN1
MSX1
EBF1
TLX3
EN2
UNCX
PHOX2A
GBX2
GSX1
P2RY1
IRX1
PKHD1
FOXB1
FOSL1
NR2F1
IRX5
OLIG3
IRX3
NHLH2
ZHX2
ZNF662
MAML2
POU3F2
ROR1
MUC1
NR2F2
POU3F1
HES4
ZP3
SKOR1
LITAF
ENPP1
HES5
ZXDA
NTRK1
NRARP
DLL1
LGR4
VGLL3
SKOR2

GO:BP
GO:0009792
embryo_development_ending_in_birth_or_egg_hatching
30

PLXNA2
LRP2
DLL3
PCSK5
SALL4
LFNG
MAN2A1
WNT5A
PROX1
PDGFRA
PCDH8
TFAP2A
LPAR6
EPHA2
CRB2
ANGPT1
ZIC3
WNT9B
EN1
MSX1
GBX2
FOXB1
FOSL1
IRX5
NR2F2
WNT7B
ZP3
HES5
NRARP
DLL1

GO:BP
GO:0060562
epithelial_tube_morphogenesis
20

LRP2
SALL4
WNT5A
PROX1
CXCR4
PODXL
LGR5
GREB1L
EPHA2
ZIC3
WNT9B
GBX2
IRX1
PKHD1
IRX3
COL4A1
HES5
NRARP
DLL1
LGR4

GO:BP
GO:0051254
positive_regulation_of_RNA_metabolic_process
56

TEAD3
TFAP2B
DDX11
WWTR1
THOC1
TESC
LHX5
SALL4
JAG1
RGCC
TOX3
EBF3
RFX4
WNT5A
PROX1
ELL2
ESRRB
FOXJ1
CASZ1
PAX3
TFAP2A
SMAD6
BMPR1B
PRDM5
ASCL1
TAF4B
CREB3L4
MEIS1
THRB
POU4F2
POU4F1
ZIC3
LMX1A
EN1
MSX1
EN2
PHOX2A
GBX2
GSX1
P2RY1
FOSL1
NR2F1
IRX3
NHLH2
MAML2
POU3F2
MUC1
NR2F2
POU3F1
ZP3
LITAF
HES5
ZXDA
DLL1
RBM20
LGR4

GO:BP
GO:0019222
regulation_of_metabolic_process
152

CYP26B1
ABCC8
RHBDF1
TEAD3
TFAP2B
PGLYRP1
PAX7
DDX11
WWTR1
ABCC2
FAS
LTBP1
TRAF1
CNN2
FGFR3
LMCD1
THOC1
CEACAM1
LRP2
NOX4
TESC
LHX5
HMOX1
SALL4
JAG1
RGCC
TOX3
CDK6
ITGB8
EBF3
WNT3
CPT1A
RFX4
PRDM13
SMOC2
SERPINI2
WNT5A
CBLB
ID3
PROX1
CCN2
ELL2
ESRRB
ADRA1A
CXCR4
NEUROD4
PDE1B
CDKN1A
AHNAK
CLYBL
KIRREL2
HSPA2
TSPAN8
AJUBA
FOXJ1
SH3BP4
ANGPTL8
GDF15
HRC
CASZ1
PDGFRA
PAX3
EDNRB
ENPP2
TFAP2A
THBS1
SMAD6
BMPR1B
PRDM5
ASCL1
TAF4B
EPHA2
MAEL
ILDR2
CREB3L4
SOX13
MEIS1
HES6
EPHA5
NTRK2
CRB2
THRB
CCDC3
POU4F2
POU4F1
ANGPT1
ZIC3
LDLRAP1
SLC45A3
WNT9B
ITGA5
OMA1
LMX1A
EN1
MSX1
DAPL1
INAVA
EBF1
TLX3
EN2
UNCX
PHOX2A
TMIGD2
GBX2
RSPO1
GSX1
P2RY1
MGMT
IRX1
PKHD1
FOXB1
SNX33
FOSL1
NR2F1
IRX5
OLIG3
IRX3
NHLH2
IL17RA
ZHX2
ZNF662
MAML2
POU3F2
ROR1
MUC1
NR2F2
POU3F1
ISG15
HES4
ZP3
SKOR1
LITAF
H3C12
ENPP1
HES5
ZXDA
NTRK1
NRARP
DLL1
PPP1R14C
FCGR3A
RBM20
LGR4
VGLL3
MIR219A2HG
MIR125B1
SKOR2
MIR34AHG
XIST
LINC00472
TSIX
MUSTN1

GO:BP
GO:0061005
cell_differentiation_involved_in_kidney_development
9

PROM1
WWTR1
JAG1
PODXL
FOXJ1
EDNRB
WNT9B
NPHS1
LGR4

GO:BP
GO:0010468
regulation_of_gene_expression
122

CYP26B1
ABCC8
TEAD3
TFAP2B
PGLYRP1
PAX7
DDX11
WWTR1
ABCC2
LTBP1
TRAF1
CNN2
LMCD1
THOC1
CEACAM1
TESC
LHX5
HMOX1
SALL4
JAG1
RGCC
TOX3
CDK6
ITGB8
EBF3
WNT3
RFX4
PRDM13
WNT5A
ID3
PROX1
CCN2
ELL2
ESRRB
NEUROD4
CDKN1A
AHNAK
TSPAN8
AJUBA
FOXJ1
ANGPTL8
CASZ1
PDGFRA
PAX3
EDNRB
TFAP2A
THBS1
SMAD6
BMPR1B
PRDM5
ASCL1
TAF4B
EPHA2
MAEL
ILDR2
CREB3L4
SOX13
MEIS1
HES6
EPHA5
NTRK2
THRB
CCDC3
POU4F2
POU4F1
ANGPT1
ZIC3
LMX1A
EN1
MSX1
INAVA
EBF1
TLX3
EN2
UNCX
PHOX2A
TMIGD2
GBX2
GSX1
P2RY1
IRX1
PKHD1
FOXB1
FOSL1
NR2F1
IRX5
OLIG3
IRX3
NHLH2
IL17RA
ZHX2
ZNF662
MAML2
POU3F2
ROR1
MUC1
NR2F2
POU3F1
ISG15
HES4
ZP3
SKOR1
LITAF
H3C12
ENPP1
HES5
ZXDA
NTRK1
NRARP
DLL1
FCGR3A
RBM20
LGR4
VGLL3
MIR219A2HG
MIR125B1
SKOR2
MIR34AHG
XIST
LINC00472
TSIX
MUSTN1

GO:BP
GO:0031323
regulation_of_cellular_metabolic_process
122

TEAD3
TFAP2B
PAX7
DDX11
WWTR1
FAS
TRAF1
FGFR3
LMCD1
THOC1
CEACAM1
NOX4
TESC
LHX5
HMOX1
SALL4
JAG1
RGCC
TOX3
CDK6
EBF3
CPT1A
RFX4
PRDM13
SMOC2
WNT5A
CBLB
ID3
PROX1
CCN2
ELL2
ESRRB
ADRA1A
NEUROD4
PDE1B
CDKN1A
AHNAK
CLYBL
KIRREL2
HSPA2
AJUBA
FOXJ1
SH3BP4
ANGPTL8
GDF15
HRC
CASZ1
PDGFRA
PAX3
EDNRB
ENPP2
TFAP2A
THBS1
SMAD6
BMPR1B
PRDM5
ASCL1
TAF4B
EPHA2
MAEL
ILDR2
CREB3L4
SOX13
MEIS1
HES6
EPHA5
NTRK2
THRB
POU4F2
POU4F1
ANGPT1
ZIC3
SLC45A3
WNT9B
ITGA5
LMX1A
EN1
MSX1
DAPL1
EBF1
TLX3
EN2
UNCX
PHOX2A
GBX2
RSPO1
GSX1
P2RY1
MGMT
IRX1
PKHD1
FOXB1
SNX33
FOSL1
NR2F1
IRX5
OLIG3
IRX3
NHLH2
ZHX2
ZNF662
MAML2
POU3F2
ROR1
MUC1
NR2F2
POU3F1
HES4
ZP3
SKOR1
LITAF
ENPP1
HES5
ZXDA
NTRK1
NRARP
DLL1
PPP1R14C
RBM20
LGR4
VGLL3
SKOR2

GO:BP
GO:2001141
regulation_of_RNA_biosynthetic_process
87

TEAD3
TFAP2B
PAX7
DDX11
WWTR1
TRAF1
LMCD1
THOC1
TESC
LHX5
HMOX1
SALL4
JAG1
RGCC
TOX3
CDK6
EBF3
RFX4
PRDM13
WNT5A
ID3
PROX1
ELL2
ESRRB
NEUROD4
AJUBA
FOXJ1
CASZ1
PAX3
EDNRB
TFAP2A
SMAD6
BMPR1B
PRDM5
ASCL1
TAF4B
MAEL
CREB3L4
SOX13
MEIS1
HES6
EPHA5
THRB
POU4F2
POU4F1
ZIC3
LMX1A
EN1
MSX1
EBF1
TLX3
EN2
UNCX
PHOX2A
GBX2
GSX1
P2RY1
IRX1
PKHD1
FOXB1
FOSL1
NR2F1
IRX5
OLIG3
IRX3
NHLH2
ZHX2
ZNF662
MAML2
POU3F2
ROR1
MUC1
NR2F2
POU3F1
HES4
ZP3
SKOR1
LITAF
ENPP1
HES5
ZXDA
NTRK1
NRARP
DLL1
LGR4
VGLL3
SKOR2

GO:BP
GO:0006935
chemotaxis
28

PLXNA2
ANGPT2
WNT3
SMOC2
WNT5A
CXCR4
CNTN6
PDGFRA
EDNRB
ENPP2
THBS1
BMPR1B
EPHA2
ACKR3
BOC
EPHA5
TPBG
POU4F2
SEMA3D
ROBO3
ANGPT1
LMX1A
GBX2
FOSL1
IL17RA
ADAMTSL1
UNC5C
NTRK1

GO:BP
GO:0007507
heart_development
27

TGFBR3
LRP2
NOX4
PCSK5
SALL4
JAG1
WNT5A
ID3
PROX1
ADRA1A
KCNJ8
CDKN1A
FOXJ1
PDGFRA
SMAD6
ASCL1
GREB1L
SYPL2
POU4F2
POU4F1
PDLIM3
ZIC3
DHRS3
MSX1
IRX3
DLL1
RBM20

GO:BP
GO:0042330
taxis
28

PLXNA2
ANGPT2
WNT3
SMOC2
WNT5A
CXCR4
CNTN6
PDGFRA
EDNRB
ENPP2
THBS1
BMPR1B
EPHA2
ACKR3
BOC
EPHA5
TPBG
POU4F2
SEMA3D
ROBO3
ANGPT1
LMX1A
GBX2
FOSL1
IL17RA
ADAMTSL1
UNC5C
NTRK1

GO:BP
GO:0090287
regulation_of_cellular_response_to_growth_factor_stimulus
19

TFAP2B
LTBP1
TGFBR3
LRP2
TSPAN12
SMOC2
WNT5A
THBS1
SMAD6
FBN2
CRB2
ITGA5
MSX1
RBPMS2
FGFBP3
SKOR1
HES5
DLL1
SKOR2

GO:BP
GO:0001503
ossification
22

WWTR1
FGFR3
TMEM38B
JAG1
CDK6
WNT3
WNT5A
ID3
CCN2
TNN
TFAP2A
SMAD6
BMPR1B
FBN2
EPHA2
DHRS3
ADGRV1
THBS3
ISG15
WNT7B
ENPP1
LGR4

GO:BP
GO:0021510
spinal_cord_development
11

LHX5
RFX4
PROX1
ASCL1
SOX13
UNCX
PHOX2A
GSX1
FOXB1
OLIG3
DLL1

GO:BP
GO:0010648
negative_regulation_of_cell_communication
45

CYP26B1
ABCC8
WWTR1
LTBP1
TGFBR3
THOC1
CEACAM1
LRP2
OPRK1
CYP26A1
HMOX1
LFNG
RNF43
RFX4
WNT5A
CBLB
TNN
ADRA1A
AJUBA
SH3BP4
GDF15
THBS1
SMAD6
FBN2
EPHA2
RGS16
SOX13
ACKR3
GLRA1
TPBG
RGS20
CCDC3
DHRS3
ARHGAP25
RBPMS2
PKHD1
MUC1
ISG15
SKOR1
LITAF
ENPP1
NRARP
DLL1
LGR4
SKOR2

GO:BP
GO:0045765
regulation_of_angiogenesis
18

ABCC8
CEACAM1
ANGPT2
HMOX1
RGCC
ITGB8
TSPAN12
SMOC2
WNT5A
TNN
CXCR4
CEMIP2
ENPP2
THBS1
EPHA2
ITGA5
TMIGD2
DLL1

GO:BP
GO:0048584
positive_regulation_of_response_to_stimulus
64

DDX11
FAS
PTGER3
TRAF1
FGFR3
TGFBR3
LMCD1
CEACAM1
LRP2
OPRK1
NOX4
HMOX1
JAG1
RGCC
LFNG
WNT3
P2RX3
SMOC2
WNT5A
CBLB
PLA2G4A
WLS
PROX1
CCN2
ADRA1A
CXCR4
AJUBA
GDF15
PRAM1
CNTN6
PDGFRA
THBS1
BMPR1B
LGR5
ASCL1
LPAR6
ACKR3
TPBG
NTRK2
CRB2
THRB
GRM1
ANGPT1
ITGA5
MSX1
INAVA
ADGRV1
TMIGD2
RSPO1
P2RY1
MGMT
FGFBP3
IL17RA
ROR1
WNT7B
ZP3
LITAF
HES5
NTRK1
NRARP
DLL1
MICB
LGR4
SKOR2

GO:BP
GO:0003013
circulatory_system_process
27

ABCC8
WWTR1
ATP1A2
ABCC2
ABCC9
CEACAM1
LRP2
NOX4
TMEM38B
PCSK5
HMOX1
CCN2
ADRA1A
SCPEP1
KCNJ8
CXCR4
KCNJ2
HRC
EDNRB
THRB
ANGPT1
TAC3
P2RY1
FGFBP3
IRX3
NR2F2
DLL1

GO:BP
GO:0010033
response_to_organic_substance
74

CYP26B1
ABCC8
CD9
DDX11
ATP1A2
FAS
TRAF1
ABCC9
TGFBR3
WNT8B
CEACAM1
LRP2
OPRK1
NOX4
TESC
ANGPT2
TMEM38B
CYP26A1
HMOX1
JAG1
WNT3
P2RX3
CPT1A
WNT5A
ID3
CCN2
ESRRB
ADRA1A
KCNJ8
CXCR4
PDE1B
CD70
HSPA2
SH3BP4
GDF15
PDGFRA
EDNRB
THBS1
SMAD6
PRDM5
ASCL1
HAPLN3
ILDR2
CREB3L4
SYT2
ACKR3
EPHA5
GLRA1
NTRK2
THRB
CCDC3
POU4F2
POU4F1
ANGPT1
LDLRAP1
WNT9B
RFTN2
EN1
INAVA
ADGRV1
P2RY1
MGMT
HSPA6
FOSL1
IL17RA
POU3F2
NR2F2
COL4A1
ISG15
WNT7B
LITAF
ENPP1
NTRK1
MICB

GO:BP
GO:0023056
positive_regulation_of_signaling
53

ABCC8
FAS
TRAF1
FGFR3
TGFBR3
LMCD1
LRP2
OPRK1
NOX4
HMOX1
JAG1
LFNG
WNT3
P2RX3
SMOC2
WNT5A
WLS
PROX1
CCN2
ADRA1A
CXCR4
AJUBA
GDF15
CNTN6
PDGFRA
THBS1
BMPR1B
LGR5
ASCL1
LPAR6
ACKR3
TPBG
NTRK2
CRB2
THRB
GRM1
ANGPT1
ITGA5
MSX1
INAVA
ADGRV1
RSPO1
P2RY1
FGFBP3
ROR1
WNT7B
LITAF
HES5
NTRK1
NRARP
DLL1
LGR4
SKOR2

GO:BP
GO:0010647
positive_regulation_of_cell_communication
53

ABCC8
FAS
TRAF1
FGFR3
TGFBR3
LMCD1
LRP2
OPRK1
NOX4
HMOX1
JAG1
LFNG
WNT3
P2RX3
SMOC2
WNT5A
WLS
PROX1
CCN2
ADRA1A
CXCR4
AJUBA
GDF15
CNTN6
PDGFRA
THBS1
BMPR1B
LGR5
ASCL1
LPAR6
ACKR3
TPBG
NTRK2
CRB2
THRB
GRM1
ANGPT1
ITGA5
MSX1
INAVA
ADGRV1
RSPO1
P2RY1
FGFBP3
ROR1
WNT7B
LITAF
HES5
NTRK1
NRARP
DLL1
LGR4
SKOR2

GO:BP
GO:0071363
cellular_response_to_growth_factor_stimulus
29

TFAP2B
LTBP1
FGFR3
TGFBR3
LRP2
ANGPT2
ITGB8
TSPAN12
SMOC2
WNT5A
CCN2
GDF15
PDGFRA
THBS1
SMAD6
BMPR1B
FBN2
NTRK2
CRB2
ITGA5
MSX1
RBPMS2
NDNF
FGFBP3
SKOR1
HES5
NTRK1
DLL1
SKOR2

GO:BP
GO:1901342
regulation_of_vasculature_development
18

ABCC8
CEACAM1
ANGPT2
HMOX1
RGCC
ITGB8
TSPAN12
SMOC2
WNT5A
TNN
CXCR4
CEMIP2
ENPP2
THBS1
EPHA2
ITGA5
TMIGD2
DLL1

GO:BP
GO:0006351
DNA-templated_transcription
88

TEAD3
TFAP2B
PAX7
DDX11
WWTR1
TRAF1
LMCD1
THOC1
TESC
LHX5
HMOX1
SALL4
JAG1
RGCC
TOX3
CDK6
EBF3
RFX4
PRDM13
WNT5A
TTF2
ID3
PROX1
ELL2
ESRRB
NEUROD4
AJUBA
FOXJ1
CASZ1
PAX3
EDNRB
TFAP2A
SMAD6
BMPR1B
PRDM5
ASCL1
TAF4B
MAEL
CREB3L4
SOX13
MEIS1
HES6
EPHA5
THRB
POU4F2
POU4F1
ZIC3
LMX1A
EN1
MSX1
EBF1
TLX3
EN2
UNCX
PHOX2A
GBX2
GSX1
P2RY1
IRX1
PKHD1
FOXB1
FOSL1
NR2F1
IRX5
OLIG3
IRX3
NHLH2
ZHX2
ZNF662
MAML2
POU3F2
ROR1
MUC1
NR2F2
POU3F1
HES4
ZP3
SKOR1
LITAF
ENPP1
HES5
ZXDA
NTRK1
NRARP
DLL1
LGR4
VGLL3
SKOR2

GO:BP
GO:0097659
nucleic_acid-templated_transcription
88

TEAD3
TFAP2B
PAX7
DDX11
WWTR1
TRAF1
LMCD1
THOC1
TESC
LHX5
HMOX1
SALL4
JAG1
RGCC
TOX3
CDK6
EBF3
RFX4
PRDM13
WNT5A
TTF2
ID3
PROX1
ELL2
ESRRB
NEUROD4
AJUBA
FOXJ1
CASZ1
PAX3
EDNRB
TFAP2A
SMAD6
BMPR1B
PRDM5
ASCL1
TAF4B
MAEL
CREB3L4
SOX13
MEIS1
HES6
EPHA5
THRB
POU4F2
POU4F1
ZIC3
LMX1A
EN1
MSX1
EBF1
TLX3
EN2
UNCX
PHOX2A
GBX2
GSX1
P2RY1
IRX1
PKHD1
FOXB1
FOSL1
NR2F1
IRX5
OLIG3
IRX3
NHLH2
ZHX2
ZNF662
MAML2
POU3F2
ROR1
MUC1
NR2F2
POU3F1
HES4
ZP3
SKOR1
LITAF
ENPP1
HES5
ZXDA
NTRK1
NRARP
DLL1
LGR4
VGLL3
SKOR2

GO:BP
GO:0045935
positive_regulation_of_nucleobase-containing_compound_metabolic_proces:
59

TEAD3
TFAP2B
DDX11
WWTR1
THOC1
NOX4
TESC
LHX5
SALL4
JAG1
RGCC
TOX3
EBF3
RFX4
SMOC2
WNT5A
PROX1
ELL2
ESRRB
FOXJ1
CASZ1
PAX3
TFAP2A
SMAD6
BMPR1B
PRDM5
ASCL1
TAF4B
CREB3L4
MEIS1
THRB
POU4F2
POU4F1
ZIC3
LMX1A
EN1
MSX1
EN2
PHOX2A
GBX2
GSX1
P2RY1
MGMT
FOSL1
NR2F1
IRX3
NHLH2
MAML2
POU3F2
MUC1
NR2F2
POU3F1
ZP3
LITAF
HES5
ZXDA
DLL1
RBM20
LGR4

GO:BP
GO:0048812
neuron_projection_morphogenesis
28

PLXNA2
LRP2
WNT3
WNT5A
TNN
CXCR4
CNTN6
BMPR1B
EPHA2
SYT2
BOC
EPHA5
TPBG
NTRK2
POU4F2
POU4F1
SEMA3D
ROBO3
LMX1A
GBX2
FOXB1
ADAMTSL1
UNC5C
POU3F2
SLITRK2
WNT7B
NTRK1
SKOR2

GO:BP
GO:0048483
autonomic_nervous_system_development
8

TFAP2B
EDNRB
TFAP2A
ASCL1
ACKR3
PHOX2A
GBX2
NTRK1

GO:BP
GO:0060255
regulation_of_macromolecule_metabolic_process
142

CYP26B1
ABCC8
RHBDF1
TEAD3
TFAP2B
PGLYRP1
PAX7
DDX11
WWTR1
ABCC2
FAS
LTBP1
TRAF1
CNN2
FGFR3
LMCD1
THOC1
CEACAM1
LRP2
NOX4
TESC
LHX5
HMOX1
SALL4
JAG1
RGCC
TOX3
CDK6
ITGB8
EBF3
WNT3
RFX4
PRDM13
SMOC2
SERPINI2
WNT5A
CBLB
ID3
PROX1
CCN2
ELL2
ESRRB
ADRA1A
NEUROD4
CDKN1A
AHNAK
KIRREL2
HSPA2
TSPAN8
AJUBA
FOXJ1
ANGPTL8
GDF15
HRC
CASZ1
PDGFRA
PAX3
EDNRB
ENPP2
TFAP2A
THBS1
SMAD6
BMPR1B
PRDM5
ASCL1
TAF4B
EPHA2
MAEL
ILDR2
CREB3L4
SOX13
MEIS1
HES6
EPHA5
NTRK2
CRB2
THRB
CCDC3
POU4F2
POU4F1
ANGPT1
ZIC3
WNT9B
ITGA5
LMX1A
EN1
MSX1
INAVA
EBF1
TLX3
EN2
UNCX
PHOX2A
TMIGD2
GBX2
RSPO1
GSX1
P2RY1
MGMT
IRX1
PKHD1
FOXB1
SNX33
FOSL1
NR2F1
IRX5
OLIG3
IRX3
NHLH2
IL17RA
ZHX2
ZNF662
MAML2
POU3F2
ROR1
MUC1
NR2F2
POU3F1
ISG15
HES4
ZP3
SKOR1
LITAF
H3C12
ENPP1
HES5
ZXDA
NTRK1
NRARP
DLL1
FCGR3A
RBM20
LGR4
VGLL3
MIR219A2HG
MIR125B1
SKOR2
MIR34AHG
XIST
LINC00472
TSIX
MUSTN1

GO:BP
GO:0009968
negative_regulation_of_signal_transduction
42

CYP26B1
WWTR1
LTBP1
TGFBR3
THOC1
CEACAM1
LRP2
CYP26A1
HMOX1
LFNG
RNF43
RFX4
WNT5A
CBLB
TNN
ADRA1A
AJUBA
SH3BP4
GDF15
THBS1
SMAD6
FBN2
EPHA2
RGS16
SOX13
ACKR3
TPBG
RGS20
CCDC3
DHRS3
ARHGAP25
RBPMS2
PKHD1
MUC1
ISG15
SKOR1
LITAF
ENPP1
NRARP
DLL1
LGR4
SKOR2

GO:BP
GO:0035850
epithelial_cell_differentiation_involved_in_kidney_development
8

PROM1
WWTR1
JAG1
PODXL
FOXJ1
EDNRB
WNT9B
NPHS1

GO:BP
GO:0048598
embryonic_morphogenesis
27

CYP26B1
WNT8B
LRP2
SALL4
WNT3
WNT5A
WLS
PROX1
PDGFRA
PCDH8
TFAP2A
FBN2
EPHA2
CRB2
ZIC3
WNT9B
ITGA5
EN1
MSX1
RBPMS2
GBX2
IRX1
IRX5
IRX3
WNT7B
HES5
DLL1

GO:BP
GO:0080090
regulation_of_primary_metabolic_process
126

RHBDF1
TEAD3
TFAP2B
PAX7
DDX11
WWTR1
FAS
TRAF1
FGFR3
LMCD1
THOC1
CEACAM1
LRP2
NOX4
TESC
LHX5
HMOX1
SALL4
JAG1
RGCC
TOX3
CDK6
EBF3
CPT1A
RFX4
PRDM13
SMOC2
SERPINI2
WNT5A
CBLB
ID3
PROX1
CCN2
ELL2
ESRRB
ADRA1A
NEUROD4
PDE1B
CDKN1A
AHNAK
KIRREL2
HSPA2
AJUBA
FOXJ1
ANGPTL8
GDF15
HRC
CASZ1
PDGFRA
PAX3
EDNRB
ENPP2
TFAP2A
THBS1
SMAD6
BMPR1B
PRDM5
ASCL1
TAF4B
MAEL
ILDR2
CREB3L4
SOX13
MEIS1
HES6
EPHA5
NTRK2
CRB2
THRB
CCDC3
POU4F2
POU4F1
ANGPT1
ZIC3
LDLRAP1
SLC45A3
WNT9B
ITGA5
LMX1A
EN1
MSX1
INAVA
EBF1
TLX3
EN2
UNCX
PHOX2A
GBX2
RSPO1
GSX1
P2RY1
MGMT
IRX1
PKHD1
FOXB1
SNX33
FOSL1
NR2F1
IRX5
OLIG3
IRX3
NHLH2
ZHX2
ZNF662
MAML2
POU3F2
ROR1
MUC1
NR2F2
POU3F1
ISG15
HES4
ZP3
SKOR1
LITAF
ENPP1
HES5
ZXDA
NTRK1
NRARP
DLL1
RBM20
LGR4
VGLL3
SKOR2
MUSTN1

GO:BP
GO:0042221
response_to_chemical
97

CYP26B1
ABCC8
TFAP2B
CD9
DDX11
ATP1A2
ABCC2
FAS
TRAF1
ABCC9
TGFBR3
WNT8B
PLXNA2
CEACAM1
LRP2
OPRK1
NOX4
TESC
ANGPT2
TMEM38B
CYP26A1
PRODH
HMOX1
JAG1
RGCC
WNT3
P2RX3
CPT1A
SMOC2
WNT5A
PLA2G4A
ID3
CCN2
ESRRB
ADRA1A
KCNJ8
CXCR4
PDE1B
CD70
HSPA2
AJUBA
SH3BP4
GDF15
CNTN6
PDGFRA
EDNRB
ENPP2
TFAP2A
THBS1
SMAD6
BMPR1B
PRDM5
ASCL1
HAPLN3
EPHA2
ILDR2
CREB3L4
SYT2
ACKR3
BOC
EPHA5
GLRA1
TPBG
NTRK2
THRB
CCDC3
POU4F2
POU4F1
SEMA3D
ROBO3
ANGPT1
LDLRAP1
WNT9B
LMX1A
RFTN2
EN1
DAPL1
INAVA
ADGRV1
GBX2
P2RY1
MGMT
HSPA6
NDNF
FOSL1
IL17RA
ADAMTSL1
UNC5C
POU3F2
NR2F2
COL4A1
ISG15
WNT7B
LITAF
ENPP1
NTRK1
MICB

GO:BP
GO:0031175
neuron_projection_development
36

PLXNA2
LRP2
WNT3
WNT5A
TNN
CXCR4
CNTN6
BMPR1B
EPHA2
SYT2
BOC
EPHA5
TPBG
NTRK2
KLHL1
POU4F2
POU4F1
SEMA3D
ROBO3
LMX1A
ADGRV1
GBX2
FOXB1
MBOAT1
NDNF
NR2F1
ADAMTSL1
UNC5C
POU3F2
ROR1
SLITRK2
RTN4RL2
WNT7B
HES5
NTRK1
SKOR2

GO:BP
GO:0032774
RNA_biosynthetic_process
88

TEAD3
TFAP2B
PAX7
DDX11
WWTR1
TRAF1
LMCD1
THOC1
TESC
LHX5
HMOX1
SALL4
JAG1
RGCC
TOX3
CDK6
EBF3
RFX4
PRDM13
WNT5A
TTF2
ID3
PROX1
ELL2
ESRRB
NEUROD4
AJUBA
FOXJ1
CASZ1
PAX3
EDNRB
TFAP2A
SMAD6
BMPR1B
PRDM5
ASCL1
TAF4B
MAEL
CREB3L4
SOX13
MEIS1
HES6
EPHA5
THRB
POU4F2
POU4F1
ZIC3
LMX1A
EN1
MSX1
EBF1
TLX3
EN2
UNCX
PHOX2A
GBX2
GSX1
P2RY1
IRX1
PKHD1
FOXB1
FOSL1
NR2F1
IRX5
OLIG3
IRX3
NHLH2
ZHX2
ZNF662
MAML2
POU3F2
ROR1
MUC1
NR2F2
POU3F1
HES4
ZP3
SKOR1
LITAF
ENPP1
HES5
ZXDA
NTRK1
NRARP
DLL1
LGR4
VGLL3
SKOR2

GO:BP
GO:0000904
cell_morphogenesis_involved_in_differentiation
28

LAMC3
PLXNA2
WNT3
WNT5A
PROX1
TNN
CNTN6
BMPR1B
EPHA2
BOC
EPHA5
TPBG
NTRK2
POU4F2
POU4F1
SEMA3D
ROBO3
LMX1A
GBX2
PKHD1
FOXB1
ADAMTSL1
UNC5C
COL18A1
POU3F2
SLITRK2
NTRK1
SKOR2

GO:BP
GO:0061061
muscle_structure_development
28

CYP26B1
PAX7
CD9
TGFBR3
LRP2
NOX4
TLL2
JAG1
WNT5A
ID3
PROX1
ADRA1A
GDF15
PDGFRA
PAX3
EDNRB
SYPL2
BOC
MEGF10
POU4F1
PGM5
PDLIM3
NPHS1
MSX1
RBPMS2
IRX3
NR2F2
DLL1

GO:BP
GO:0023057
negative_regulation_of_signaling
44

CYP26B1
ABCC8
WWTR1
LTBP1
TGFBR3
THOC1
CEACAM1
LRP2
OPRK1
CYP26A1
HMOX1
LFNG
RNF43
RFX4
WNT5A
CBLB
TNN
ADRA1A
AJUBA
SH3BP4
GDF15
THBS1
SMAD6
FBN2
EPHA2
RGS16
SOX13
ACKR3
TPBG
RGS20
CCDC3
DHRS3
ARHGAP25
RBPMS2
PKHD1
MUC1
ISG15
SKOR1
LITAF
ENPP1
NRARP
DLL1
LGR4
SKOR2

GO:BP
GO:0030509
BMP_signaling_pathway
13

TFAP2B
TGFBR3
LRP2
WNT5A
GDF15
SMAD6
BMPR1B
CRB2
MSX1
RBPMS2
SKOR1
HES5
SKOR2

GO:BP
GO:0001756
somitogenesis
9

PLXNA2
DLL3
LFNG
WNT5A
PCDH8
CRB2
FOXB1
NRARP
DLL1

GO:BP
GO:0072311
glomerular_epithelial_cell_differentiation
6

PROM1
JAG1
PODXL
FOXJ1
EDNRB
NPHS1

GO:BP
GO:0002064
epithelial_cell_development
15

JAG1
CDK6
WNT5A
PROX1
CXCR4
PODXL
FOXJ1
EDNRB
EPHA2
NPHS1
PKHD1
COL18A1
WNT7B
NTRK1
DLL1

GO:BP
GO:0007411
axon_guidance
16

PLXNA2
WNT3
WNT5A
CNTN6
BMPR1B
EPHA2
BOC
EPHA5
POU4F2
SEMA3D
ROBO3
LMX1A
GBX2
ADAMTSL1
UNC5C
NTRK1

GO:BP
GO:0051252
regulation_of_RNA_metabolic_process
90

TEAD3
TFAP2B
PAX7
DDX11
WWTR1
TRAF1
LMCD1
THOC1
TESC
LHX5
HMOX1
SALL4
JAG1
RGCC
TOX3
CDK6
EBF3
RFX4
PRDM13
WNT5A
ID3
PROX1
ELL2
ESRRB
NEUROD4
AHNAK
AJUBA
FOXJ1
CASZ1
PAX3
EDNRB
TFAP2A
SMAD6
BMPR1B
PRDM5
ASCL1
TAF4B
MAEL
ILDR2
CREB3L4
SOX13
MEIS1
HES6
EPHA5
THRB
POU4F2
POU4F1
ZIC3
LMX1A
EN1
MSX1
EBF1
TLX3
EN2
UNCX
PHOX2A
GBX2
GSX1
P2RY1
IRX1
PKHD1
FOXB1
FOSL1
NR2F1
IRX5
OLIG3
IRX3
NHLH2
ZHX2
ZNF662
MAML2
POU3F2
ROR1
MUC1
NR2F2
POU3F1
HES4
ZP3
SKOR1
LITAF
ENPP1
HES5
ZXDA
NTRK1
NRARP
DLL1
RBM20
LGR4
VGLL3
SKOR2

GO:BP
GO:0097485
neuron_projection_guidance
16

PLXNA2
WNT3
WNT5A
CNTN6
BMPR1B
EPHA2
BOC
EPHA5
POU4F2
SEMA3D
ROBO3
LMX1A
GBX2
ADAMTSL1
UNC5C
NTRK1

GO:BP
GO:0051171
regulation_of_nitrogen_compound_metabolic_process
122

RHBDF1
TEAD3
TFAP2B
PAX7
DDX11
WWTR1
FAS
TRAF1
FGFR3
LMCD1
THOC1
CEACAM1
LRP2
NOX4
TESC
LHX5
HMOX1
SALL4
JAG1
RGCC
TOX3
CDK6
EBF3
RFX4
PRDM13
SMOC2
SERPINI2
WNT5A
CBLB
ID3
PROX1
CCN2
ELL2
ESRRB
ADRA1A
NEUROD4
PDE1B
CDKN1A
AHNAK
CLYBL
KIRREL2
HSPA2
AJUBA
FOXJ1
ANGPTL8
GDF15
HRC
CASZ1
PAX3
EDNRB
ENPP2
TFAP2A
THBS1
SMAD6
BMPR1B
PRDM5
ASCL1
TAF4B
MAEL
ILDR2
CREB3L4
SOX13
MEIS1
HES6
EPHA5
NTRK2
CRB2
THRB
POU4F2
POU4F1
ANGPT1
ZIC3
WNT9B
ITGA5
LMX1A
EN1
MSX1
INAVA
EBF1
TLX3
EN2
UNCX
PHOX2A
GBX2
RSPO1
GSX1
P2RY1
MGMT
IRX1
PKHD1
FOXB1
SNX33
FOSL1
NR2F1
IRX5
OLIG3
IRX3
NHLH2
ZHX2
ZNF662
MAML2
POU3F2
ROR1
MUC1
NR2F2
POU3F1
ISG15
HES4
ZP3
SKOR1
LITAF
ENPP1
HES5
ZXDA
NTRK1
NRARP
DLL1
RBM20
LGR4
VGLL3
SKOR2
MUSTN1

GO:BP
GO:0007610
behavior
27

ABCC8
ATP1A2
OPRK1
KCNK10
P2RX3
CPT1A
PDE1B
GDF15
EDNRB
THBS1
MEIS1
GLRA1
TPBG
NTRK2
KLHL1
THRB
POU4F1
GRM1
ITGA5
LMX1A
EN1
P2RY1
FOXB1
FOSL1
NHLH2
GPR157
NTRK1

GO:BP
GO:0051402
neuron_apoptotic_process
16

TFAP2B
FAS
HMOX1
TOX3
NES
TFAP2A
ASCL1
NTRK2
THRB
POU4F1
ANGPT1
EN1
EN2
FOXB1
NDNF
NTRK1

GO:BP
GO:0042063
gliogenesis
18

ABCC8
CD9
LAMC3
LRP2
CDK6
CXCR4
NEUROD4
ASCL1
SOX13
NTRK2
SLC45A3
P2RY1
GPR157
POU3F2
ROR1
POU3F1
HES5
DLL1

GO:BP
GO:0007409
axonogenesis
22

PLXNA2
WNT3
WNT5A
TNN
CNTN6
BMPR1B
EPHA2
BOC
EPHA5
NTRK2
POU4F2
POU4F1
SEMA3D
ROBO3
LMX1A
GBX2
FOXB1
ADAMTSL1
UNC5C
POU3F2
SLITRK2
NTRK1

GO:BP
GO:0051960
regulation_of_nervous_system_development
22

ABCC8
PLXNA2
LRP2
DLL3
SYNDIG1
WNT3
MAN2A1
WNT5A
PROX1
CXCR4
ASCL1
HES6
TPBG
NTRK2
POU4F2
POU4F1
SEMA3D
GBX2
SLITRK2
HES5
NTRK1
DLL1

GO:BP
GO:0048568
embryonic_organ_development
22

TEAD3
PCSK5
WNT5A
ID3
PROX1
NES
PDGFRA
TFAP2A
FBN2
EPHA2
CRB2
ZIC3
WNT9B
EN1
MSX1
EN2
RBPMS2
GBX2
IRX5
NR2F2
WNT7B
DLL1

GO:BP
GO:0071772
response_to_BMP
13

TFAP2B
TGFBR3
LRP2
WNT5A
GDF15
SMAD6
BMPR1B
CRB2
MSX1
RBPMS2
SKOR1
HES5
SKOR2

GO:BP
GO:0071773
cellular_response_to_BMP_stimulus
13

TFAP2B
TGFBR3
LRP2
WNT5A
GDF15
SMAD6
BMPR1B
CRB2
MSX1
RBPMS2
SKOR1
HES5
SKOR2

GO:BP
GO:0048589
developmental_growth
27

CD9
WWTR1
FGFR3
TGFBR3
TMEM38B
TLL2
SALL4
WNT3
WNT5A
PROX1
TNN
ADRA1A
CXCR4
CDKN1A
GDF15
BMPR1B
MAEL
SYT2
POU4F2
SEMA3D
EN1
THBS3
POU3F2
WNT7B
ZP3
DLL1
MUSTN1

GO:BP
GO:0021854
hypothalamus_development
6

PRDM13
GSX1
FOXB1
NDNF
NHLH2
POU3F2

GO:BP
GO:0072010
glomerular_epithelium_development
6

PROM1
JAG1
PODXL
FOXJ1
EDNRB
NPHS1

GO:BP
GO:0019219
regulation_of_nucleobase-containing_compound_metabolic_proces
94

TEAD3
TFAP2B
PAX7
DDX11
WWTR1
TRAF1
LMCD1
THOC1
NOX4
TESC
LHX5
HMOX1
SALL4
JAG1
RGCC
TOX3
CDK6
EBF3
RFX4
PRDM13
SMOC2
WNT5A
ID3
PROX1
ELL2
ESRRB
NEUROD4
CDKN1A
AHNAK
AJUBA
FOXJ1
CASZ1
PAX3
EDNRB
TFAP2A
SMAD6
BMPR1B
PRDM5
ASCL1
TAF4B
MAEL
ILDR2
CREB3L4
SOX13
MEIS1
HES6
EPHA5
THRB
POU4F2
POU4F1
ZIC3
LMX1A
EN1
MSX1
EBF1
TLX3
EN2
UNCX
PHOX2A
GBX2
GSX1
P2RY1
MGMT
IRX1
PKHD1
FOXB1
FOSL1
NR2F1
IRX5
OLIG3
IRX3
NHLH2
ZHX2
ZNF662
MAML2
POU3F2
ROR1
MUC1
NR2F2
POU3F1
HES4
ZP3
SKOR1
LITAF
ENPP1
HES5
ZXDA
NTRK1
NRARP
DLL1
RBM20
LGR4
VGLL3
SKOR2

GO:BP
GO:0009889
regulation_of_biosynthetic_process
96

TEAD3
TFAP2B
PAX7
DDX11
WWTR1
TRAF1
LMCD1
THOC1
CEACAM1
NOX4
TESC
LHX5
HMOX1
SALL4
JAG1
RGCC
TOX3
CDK6
EBF3
RFX4
PRDM13
SMOC2
WNT5A
ID3
PROX1
CCN2
ELL2
ESRRB
NEUROD4
CDKN1A
AJUBA
FOXJ1
CASZ1
PAX3
EDNRB
TFAP2A
THBS1
SMAD6
BMPR1B
PRDM5
ASCL1
TAF4B
MAEL
CREB3L4
SOX13
MEIS1
HES6
EPHA5
THRB
CCDC3
POU4F2
POU4F1
ZIC3
SLC45A3
LMX1A
EN1
MSX1
EBF1
TLX3
EN2
UNCX
PHOX2A
GBX2
GSX1
P2RY1
IRX1
PKHD1
FOXB1
FOSL1
NR2F1
IRX5
OLIG3
IRX3
NHLH2
ZHX2
ZNF662
MAML2
POU3F2
ROR1
MUC1
NR2F2
POU3F1
HES4
ZP3
SKOR1
LITAF
ENPP1
HES5
ZXDA
NTRK1
NRARP
DLL1
LGR4
VGLL3
SKOR2
MUSTN1

GO:BP
GO:0008284
positive_regulation_of_cell_population_proliferation
34

TFAP2B
WWTR1
FGFR3
TGFBR3
LRP2
LHX5
HMOX1
CDK6
WNT5A
PROX1
CCN2
CDKN1A
CD70
NES
PDGFRA
EDNRB
THBS1
ASCL1
HAPLN3
MEIS1
MEGF10
NTRK2
LDLRAP1
RBPMS2
TMIGD2
PKHD1
FOSL1
POU3F2
ZP3
HES5
NRARP
DLL1
FCGR3A
MUSTN1

GO:BP
GO:0010556
regulation_of_macromolecule_biosynthetic_process
92

TEAD3
TFAP2B
PAX7
DDX11
WWTR1
TRAF1
LMCD1
THOC1
NOX4
TESC
LHX5
HMOX1
SALL4
JAG1
RGCC
TOX3
CDK6
EBF3
RFX4
PRDM13
SMOC2
WNT5A
ID3
PROX1
ELL2
ESRRB
NEUROD4
CDKN1A
AJUBA
FOXJ1
CASZ1
PAX3
EDNRB
TFAP2A
THBS1
SMAD6
BMPR1B
PRDM5
ASCL1
TAF4B
MAEL
CREB3L4
SOX13
MEIS1
HES6
EPHA5
THRB
POU4F2
POU4F1
ZIC3
LMX1A
EN1
MSX1
EBF1
TLX3
EN2
UNCX
PHOX2A
GBX2
GSX1
P2RY1
IRX1
PKHD1
FOXB1
FOSL1
NR2F1
IRX5
OLIG3
IRX3
NHLH2
ZHX2
ZNF662
MAML2
POU3F2
ROR1
MUC1
NR2F2
POU3F1
HES4
ZP3
SKOR1
LITAF
ENPP1
HES5
ZXDA
NTRK1
NRARP
DLL1
LGR4
VGLL3
SKOR2
MUSTN1

GO:BP
GO:0021536
diencephalon_development
9

PRDM13
WNT5A
POU4F1
GBX2
GSX1
FOXB1
NDNF
NHLH2
POU3F2

GO:BP
GO:0061564
axon_development
23

PLXNA2
WNT3
WNT5A
TNN
CNTN6
BMPR1B
EPHA2
BOC
EPHA5
NTRK2
POU4F2
POU4F1
SEMA3D
ROBO3
LMX1A
GBX2
FOXB1
ADAMTSL1
UNC5C
POU3F2
SLITRK2
RTN4RL2
NTRK1

GO:BP
GO:0008015
blood_circulation
23

ABCC8
ATP1A2
ABCC9
CEACAM1
TMEM38B
PCSK5
HMOX1
CCN2
ADRA1A
SCPEP1
KCNJ8
CXCR4
KCNJ2
HRC
EDNRB
THRB
ANGPT1
TAC3
P2RY1
FGFBP3
IRX3
NR2F2
DLL1

GO:BP
GO:0035108
limb_morphogenesis
12

CYP26B1
TFAP2B
PLXNA2
PCSK5
SALL4
WNT3
WNT5A
TFAP2A
FBN2
ZIC3
EN1
MSX1

GO:BP
GO:0035107
appendage_morphogenesis
12

CYP26B1
TFAP2B
PLXNA2
PCSK5
SALL4
WNT3
WNT5A
TFAP2A
FBN2
ZIC3
EN1
MSX1

GO:BP
GO:0030030
cell_projection_organization
47

WWTR1
PLXNA2
CDC14A
LRP2
WNT3
RFX4
WNT5A
TNN
CXCR4
PODXL
AJUBA
FOXJ1
NES
CNTN6
ENPP2
BMPR1B
EPHA2
SYT2
BOC
EPHA5
TPBG
NTRK2
KLHL1
POU4F2
POU4F1
SEMA3D
ROBO3
PDPN
LMX1A
ADGRV1
GBX2
PKHD1
FOXB1
MBOAT1
NDNF
NR2F1
ADAMTSL1
UNC5C
POU3F2
ROR1
SLITRK2
RTN4RL2
WNT7B
HES5
NTRK1
SKOR2
STON1

GO:BP
GO:0071542
dopaminergic_neuron_differentiation
7

WNT3
WNT5A
WNT9B
LMX1A
EN1
EN2
PHOX2A

GO:BP
GO:0060839
endothelial_cell_fate_commitment
4

PROX1
PDPN
NR2F2
DLL1

GO:BP
GO:0051241
negative_regulation_of_multicellular_organismal_process
37

ABCC8
PGLYRP1
CD9
ATP1A2
PTGER3
THOC1
CEACAM1
OPRK1
DLL3
ANGPT2
HMOX1
RGCC
CDK6
WNT3
WNT5A
CBLB
PROX1
ADRA1A
TSPAN8
FOXJ1
GDF15
PDGFRA
THBS1
SMAD6
EPHA2
ILDR2
GLRA1
POU4F2
SEMA3D
ANGPT1
WNT9B
NR2F2
ENPP1
HES5
NRARP
DLL1
LGR4

GO:BP
GO:0043524
negative_regulation_of_neuron_apoptotic_process
12

TFAP2B
HMOX1
TOX3
NES
NTRK2
POU4F1
ANGPT1
EN1
EN2
FOXB1
NDNF
NTRK1

GO:BP
GO:0060838
lymphatic_endothelial_cell_fate_commitment
3

PROX1
PDPN
NR2F2

GO:BP
GO:0120036
plasma_membrane_bounded_cell_projection_organization
46

WWTR1
PLXNA2
CDC14A
LRP2
WNT3
RFX4
WNT5A
TNN
CXCR4
PODXL
AJUBA
FOXJ1
CNTN6
ENPP2
BMPR1B
EPHA2
SYT2
BOC
EPHA5
TPBG
NTRK2
KLHL1
POU4F2
POU4F1
SEMA3D
ROBO3
PDPN
LMX1A
ADGRV1
GBX2
PKHD1
FOXB1
MBOAT1
NDNF
NR2F1
ADAMTSL1
UNC5C
POU3F2
ROR1
SLITRK2
RTN4RL2
WNT7B
HES5
NTRK1
SKOR2
STON1

GO:BP
GO:0060537
muscle_tissue_development
20

CYP26B1
TFAP2B
TGFBR3
LRP2
NOX4
TLL2
WNT5A
PROX1
TNN
ADRA1A
PDGFRA
MEGF10
POU4F1
PGM5
ZIC3
NPHS1
MSX1
IRX3
NR2F2
DLL1

GO:BP
GO:0034654
nucleobase-containing_compound_biosynthetic_process
93

TEAD3
TFAP2B
PAX7
DDX11
WWTR1
TRAF1
LMCD1
THOC1
NOX4
TESC
LHX5
ADA2
HMOX1
SALL4
JAG1
RGCC
TOX3
CDK6
EBF3
RFX4
PRDM13
SMOC2
WNT5A
TTF2
ID3
PROX1
CCN2
ELL2
ESRRB
NEUROD4
CDKN1A
AJUBA
FOXJ1
CASZ1
PAX3
EDNRB
TFAP2A
SMAD6
BMPR1B
PRDM5
ASCL1
TAF4B
MAEL
CREB3L4
SOX13
MEIS1
HES6
EPHA5
THRB
POU4F2
POU4F1
ZIC3
LMX1A
EN1
MSX1
EBF1
TLX3
EN2
UNCX
PHOX2A
GBX2
GSX1
P2RY1
IRX1
PKHD1
FOXB1
FOSL1
NR2F1
IRX5
OLIG3
IRX3
NHLH2
ZHX2
ZNF662
MAML2
POU3F2
ROR1
MUC1
NR2F2
POU3F1
HES4
ZP3
SKOR1
LITAF
ENPP1
HES5
ZXDA
NTRK1
NRARP
DLL1
LGR4
VGLL3
SKOR2

GO:BP
GO:1901362
organic_cyclic_compound_biosynthetic_process
97

TEAD3
TFAP2B
PAX7
DDX11
WWTR1
TRAF1
OSBPL3
LMCD1
THOC1
NOX4
TESC
LHX5
ADA2
HMOX1
SALL4
JAG1
RGCC
TOX3
CDK6
EBF3
RFX4
PRDM13
SMOC2
WNT5A
TTF2
ID3
PROX1
CCN2
ELL2
ESRRB
NEUROD4
CDKN1A
AJUBA
FOXJ1
CASZ1
PAX3
EDNRB
TFAP2A
SMAD6
BMPR1B
PRDM5
ASCL1
TAF4B
MAEL
CREB3L4
SOX13
MEIS1
HES6
EPHA5
THRB
POU4F2
POU4F1
ZIC3
FDXR
LMX1A
EN1
MSX1
EBF1
TLX3
EN2
UNCX
PHOX2A
GBX2
GSX1
P2RY1
IRX1
PKHD1
PAH
FOXB1
FOSL1
NR2F1
IRX5
OLIG3
IRX3
NHLH2
ZHX2
ZNF662
MAML2
POU3F2
ROR1
MUC1
NR2F2
POU3F1
HES4
ZP3
SKOR1
LITAF
ENPP1
HES5
HSD17B11
ZXDA
NTRK1
NRARP
DLL1
LGR4
VGLL3
SKOR2

GO:BP
GO:0021537
telencephalon_development
16

ATP1A2
LHX5
CDK6
RFX4
WNT5A
PROX1
CXCR4
ASCL1
EPHA5
NTRK2
ZIC3
LMX1A
UNCX
FOXB1
POU3F2
HES5

GO:BP
GO:0045597
positive_regulation_of_cell_differentiation
31

CYP26B1
PROM1
WWTR1
PLXNA2
CEACAM1
LRP2
TESC
JAG1
RGCC
WNT3
MAN2A1
PROX1
CCN2
CXCR4
NEUROD4
FOXJ1
BMPR1B
FBN2
ASCL1
SOX13
BOC
NTRK2
CRB2
CCDC3
POU4F2
POU4F1
PDPN
IRX3
ISG15
WNT7B
MUSTN1

GO:BP
GO:0030510
regulation_of_BMP_signaling_pathway
10

TFAP2B
LRP2
WNT5A
SMAD6
CRB2
MSX1
RBPMS2
SKOR1
HES5
SKOR2

GO:BP
GO:0031326
regulation_of_cellular_biosynthetic_process
93

TEAD3
TFAP2B
PAX7
DDX11
WWTR1
TRAF1
LMCD1
THOC1
CEACAM1
NOX4
TESC
LHX5
HMOX1
SALL4
JAG1
RGCC
TOX3
CDK6
EBF3
RFX4
PRDM13
SMOC2
WNT5A
ID3
PROX1
ELL2
ESRRB
NEUROD4
CDKN1A
AJUBA
FOXJ1
CASZ1
PAX3
EDNRB
TFAP2A
THBS1
SMAD6
BMPR1B
PRDM5
ASCL1
TAF4B
MAEL
CREB3L4
SOX13
MEIS1
HES6
EPHA5
THRB
POU4F2
POU4F1
ZIC3
SLC45A3
LMX1A
EN1
MSX1
EBF1
TLX3
EN2
UNCX
PHOX2A
GBX2
GSX1
P2RY1
IRX1
PKHD1
FOXB1
FOSL1
NR2F1
IRX5
OLIG3
IRX3
NHLH2
ZHX2
ZNF662
MAML2
POU3F2
ROR1
MUC1
NR2F2
POU3F1
HES4
ZP3
SKOR1
LITAF
ENPP1
HES5
ZXDA
NTRK1
NRARP
DLL1
LGR4
VGLL3
SKOR2

GO:BP
GO:0007178
transmembrane_receptor_protein_serine/threonine_kinase_signaling_pathway
19

TFAP2B
WWTR1
LTBP1
TGFBR3
LRP2
ITGB8
WNT5A
GDF15
THBS1
SMAD6
BMPR1B
FBN2
CRB2
ABTB3
MSX1
RBPMS2
SKOR1
HES5
SKOR2

GO:BP
GO:0021879
forebrain_neuron_differentiation
7

LHX5
PROX1
ASCL1
GBX2
NDNF
NHLH2
HES5

GO:BP
GO:1901700
response_to_oxygen-containing_compounc
51

CYP26B1
ABCC8
DDX11
ATP1A2
ABCC9
TGFBR3
WNT8B
CEACAM1
OPRK1
NOX4
TESC
ANGPT2
CYP26A1
HMOX1
JAG1
WNT3
P2RX3
CPT1A
WNT5A
ID3
CCN2
KCNJ8
CXCR4
SH3BP4
GDF15
PDGFRA
EDNRB
THBS1
SMAD6
ASCL1
ILDR2
SYT2
EPHA5
GLRA1
NTRK2
POU4F2
POU4F1
WNT9B
EN1
INAVA
ADGRV1
P2RY1
MGMT
FOSL1
NR2F2
COL4A1
WNT7B
LITAF
ENPP1
NTRK1
MICB

GO:BP
GO:0009605
response_to_external_stimulus
72

CYP26B1
ABCC8
PGLYRP1
CD9
ATP1A2
FAS
PTGER3
CNN2
ABCC9
PLXNA2
CEACAM1
OPRK1
ANGPT2
CCDC80
CYP26A1
HMOX1
CDK6
ITGB8
WNT3
P2RX3
CPT1A
SMOC2
WNT5A
PROX1
ADRA1A
KCNJ8
CXCR4
KCNJ2
CDKN1A
TSPAN8
GDF15
CNTN6
PDGFRA
EDNRB
ENPP2
THBS1
SMAD6
BMPR1B
ASCL1
EPHA2
HMCN1
ACKR3
BOC
EPHA5
GLRA1
TPBG
POU4F2
SEMA3D
ROBO3
ANGPT1
PIEZO2
WNT9B
OMA1
LMX1A
DAPL1
INAVA
NUAK2
ADGRV1
GBX2
P2RY1
MGMT
FOSL1
IL17RA
ADAMTSL1
UNC5C
ISG15
ZP3
LITAF
NTRK1
FCGR3A
MICB
LGR4

GO:BP
GO:0043523
regulation_of_neuron_apoptotic_process
14

TFAP2B
HMOX1
TOX3
NES
TFAP2A
ASCL1
NTRK2
POU4F1
ANGPT1
EN1
EN2
FOXB1
NDNF
NTRK1

GO:BP
GO:0019438
aromatic_compound_biosynthetic_process
94

TEAD3
TFAP2B
PAX7
DDX11
WWTR1
TRAF1
LMCD1
THOC1
NOX4
TESC
LHX5
ADA2
HMOX1
SALL4
JAG1
RGCC
TOX3
CDK6
EBF3
RFX4
PRDM13
SMOC2
WNT5A
TTF2
ID3
PROX1
CCN2
ELL2
ESRRB
NEUROD4
CDKN1A
AJUBA
FOXJ1
CASZ1
PAX3
EDNRB
TFAP2A
SMAD6
BMPR1B
PRDM5
ASCL1
TAF4B
MAEL
CREB3L4
SOX13
MEIS1
HES6
EPHA5
THRB
POU4F2
POU4F1
ZIC3
LMX1A
EN1
MSX1
EBF1
TLX3
EN2
UNCX
PHOX2A
GBX2
GSX1
P2RY1
IRX1
PKHD1
PAH
FOXB1
FOSL1
NR2F1
IRX5
OLIG3
IRX3
NHLH2
ZHX2
ZNF662
MAML2
POU3F2
ROR1
MUC1
NR2F2
POU3F1
HES4
ZP3
SKOR1
LITAF
ENPP1
HES5
ZXDA
NTRK1
NRARP
DLL1
LGR4
VGLL3
SKOR2

GO:CC
GO:0071944
cell_periphery
159

ABCC8
PROM1
CD9
WWTR1
ATP1A2
ABCC2
FAS
LTBP1
LAMC3
PTGER3
TRAF1
SLC6A16
FGFR3
ABCC9
TGFBR3
OSBPL3
HMMR
PLXNA2
CEACAM1
LRP2
OPRK1
CHMP2B
SLCO1A2
FOLH1
NOX4
TESC
ANGPT2
CCDC80
HMOX1
KCNK10
NKAIN4
JAG1
SYNDIG1
CD37
ITGB8
TSPAN12
RNF43
WNT3
P2RX3
PRMT8
SMOC2
SLC27A6
WNT5A
CBLB
WLS
CA14
CCN2
TNN
ADRA1A
KCNJ8
CXCR4
ARL4A
KCNJ2
AHNAK
CD70
KIRREL2
HSPA2
TSPAN8
PODXL
USP6
AJUBA
SH3BP4
GDF15
SLC6A8
PRAM1
CNTN6
PDGFRA
CEMIP2
PCDH8
EDNRB
GYPC
ENPP2
THBS1
BMPR1B
FBN2
LGR5
LPAR6
HAPLN3
RHCG
MFGE8
ABCA8
TTYH2
EPHA2
RGS16
HMCN1
SYT2
ACKR3
BOC
EPHA5
MEGF10
GLRA1
TPBG
RGS20
NTRK2
CRB2
SLC5A12
GRM1
SEMA3D
ROBO3
ANGPT1
PGM5
PIEZO2
LDLRAP1
SHROOM4
SLC45A3
ADAMTS4
NPHS1
ITGA5
PDPN
DHRS3
OLFML2B
RFTN2
ARHGAP25
ADGRV1
SLC6A5
TMIGD2
THBS3
P2RY1
PKHD1
NETO2
SLC9C1
SUCLG2
HSPA6
NDNF
TNFRSF10C
SNX33
GNG5
CTSF
IGDCC3
FGFBP3
FOSL1
PRIMA1
TMPRSS7
IL17RA
ADAMTSL1
GPR157
UNC5C
COL18A1
KCNH8
ROR1
MUC1
SLITRK2
RTN4RL2
RHD
TMPRSS11A
COL4A1
WNT7B
ZP3
LITAF
PCDH18
TMEM63A
ENPP1
NTRK1
DLL1
FCGR3A
MICB
LGR4
TSPAN19
STON1

GO:CC
GO:0005886
plasma_membrane
144

ABCC8
PROM1
CD9
WWTR1
ATP1A2
ABCC2
FAS
PTGER3
TRAF1
SLC6A16
FGFR3
ABCC9
TGFBR3
OSBPL3
HMMR
PLXNA2
CEACAM1
LRP2
OPRK1
CHMP2B
SLCO1A2
FOLH1
NOX4
TESC
ANGPT2
HMOX1
KCNK10
NKAIN4
JAG1
SYNDIG1
CD37
ITGB8
TSPAN12
RNF43
WNT3
P2RX3
PRMT8
SLC27A6
WNT5A
CBLB
WLS
CA14
CCN2
ADRA1A
KCNJ8
CXCR4
ARL4A
KCNJ2
AHNAK
CD70
KIRREL2
HSPA2
TSPAN8
PODXL
USP6
AJUBA
SH3BP4
SLC6A8
PRAM1
CNTN6
PDGFRA
CEMIP2
PCDH8
EDNRB
GYPC
ENPP2
THBS1
BMPR1B
LGR5
LPAR6
RHCG
MFGE8
ABCA8
TTYH2
EPHA2
RGS16
HMCN1
SYT2
ACKR3
BOC
EPHA5
MEGF10
GLRA1
TPBG
RGS20
NTRK2
CRB2
SLC5A12
GRM1
SEMA3D
ROBO3
ANGPT1
PGM5
PIEZO2
LDLRAP1
SHROOM4
SLC45A3
NPHS1
ITGA5
PDPN
DHRS3
RFTN2
ARHGAP25
ADGRV1
SLC6A5
TMIGD2
P2RY1
PKHD1
NETO2
SLC9C1
SUCLG2
HSPA6
TNFRSF10C
SNX33
GNG5
CTSF
IGDCC3
FOSL1
PRIMA1
TMPRSS7
IL17RA
ADAMTSL1
GPR157
UNC5C
KCNH8
ROR1
MUC1
SLITRK2
RTN4RL2
RHD
TMPRSS11A
WNT7B
ZP3
LITAF
PCDH18
TMEM63A
ENPP1
NTRK1
DLL1
FCGR3A
MICB
LGR4
TSPAN19
STON1

GO:CC
GO:0000785
chromatin
51

TEAD3
TFAP2B
PAX7
DDX11
LHX5
SALL4
EBF3
RFX4
PROX1
ESRRB
NEUROD4
FOXJ1
CASZ1
PAX3
TFAP2A
SMAD6
ASCL1
MAEL
CREB3L4
SOX13
MEIS1
HES6
THRB
POU4F2
POU4F1
LMX1A
EN1
MSX1
EBF1
TLX3
EN2
UNCX
PHOX2A
GBX2
GSX1
IRX1
FOXB1
FOSL1
IRX5
OLIG3
IRX3
NHLH2
ZHX2
POU3F2
MUC1
POU3F1
HES4
H4C3
H3C12
HES5
TSIX

GO:CC
GO:0031012
extracellular_matrix
28

LTBP1
LAMC3
TGFBR3
ANGPT2
CCDC80
WNT3
SMOC2
WNT5A
CCN2
TNN
CXCR4
GDF15
THBS1
FBN2
HAPLN3
MFGE8
HMCN1
ANGPT1
ADAMTS4
OLFML2B
THBS3
NDNF
CTSF
FGFBP3
COL18A1
RTN4RL2
COL4A1
ZP3

GO:CC
GO:0030312
external_encapsulating_structure
28

LTBP1
LAMC3
TGFBR3
ANGPT2
CCDC80
WNT3
SMOC2
WNT5A
CCN2
TNN
CXCR4
GDF15
THBS1
FBN2
HAPLN3
MFGE8
HMCN1
ANGPT1
ADAMTS4
OLFML2B
THBS3
NDNF
CTSF
FGFBP3
COL18A1
RTN4RL2
COL4A1
ZP3

GO:CC
GO:0016020
membrane
191

CYP26B1
ABCC8
PROM1
RHBDF1
CD9
WWTR1
ATP1A2
ABCC2
FAS
LAMC3
PTGER3
TRAF1
SLC6A16
CNN2
FGFR3
ABCC9
TGFBR3
OSBPL3
HMMR
PLXNA2
CEACAM1
LRP2
OPRK1
CHMP2B
SLCO1A2
FOLH1
NOX4
TESC
DLL3
ANGPT2
CCDC80
TMEM38B
CYP26A1
PCSK5
PRODH
HMOX1
FAM118A
KCNK10
NKAIN4
JAG1
SYNDIG1
CD37
ITGB8
LFNG
TSPAN12
RNF43
WNT3
P2RX3
CPT1A
PRMT8
MAN2A1
SLC27A6
WNT5A
CBLB
PLA2G4A
WLS
CA14
CCN2
ADRA1A
KCNJ8
CXCR4
ARL4A
KCNJ2
AHNAK
CLYBL
CD70
KIRREL2
HSPA2
TSPAN8
PODXL
USP6
AJUBA
SH3BP4
HRC
SLC6A8
PRAM1
CNTN6
PDGFRA
CEMIP2
PCDH8
EDNRB
GYPC
ENPP2
THBS1
BMPR1B
LGR5
LPAR6
RHCG
MFGE8
RHBDL3
ABCA8
GREB1L
TTYH2
EPHA2
SYPL2
ILDR2
RGS16
HMCN1
CREB3L4
SYT2
ACKR3
BOC
EPHA5
MEGF10
GLRA1
TPBG
RGS20
NTRK2
CRB2
SLC5A12
ABTB3
GRM1
SEMA3D
ANKRD29
ROBO3
ANGPT1
PGM5
PIEZO2
FRRS1
LDLRAP1
SHROOM4
SLC45A3
ADAMTS4
NPHS1
FDXR
ITGA5
PDPN
DHRS3
OMA1
RFTN2
EN1
ARHGAP25
DAPL1
ADGRV1
EN2
SLC6A5
TMIGD2
P2RY1
MGMT
PKHD1
NETO2
SLC9C1
MBOAT1
SUCLG2
HSPA6
TNFRSF10C
SNX33
GNG5
CTSF
IGDCC3
CHST1
FOSL1
PRIMA1
TMPRSS7
IL17RA
ADAMTSL1
TMTC2
RPRML
GPR157
UNC5C
ASB18
KCNH8
TMEM255B
ROR1
MUC1
SRL
SLITRK2
RTN4RL2
RHD
TMPRSS11A
COL4A1
DIPK1C
WNT7B
ZP3
LITAF
PCDH18
TMEM63A
TMEM26
H4C3
H3C12
ENPP1
NTRK1
DLL1
PPP1R14C
FCGR3A
MICB
LGR4
TSPAN19
STON1
PTCHD4
LINC00672

GO:CC
GO:0098590
plasma_membrane_region
45

PROM1
ATP1A2
ABCC2
CEACAM1
LRP2
OPRK1
SLCO1A2
TESC
HMOX1
JAG1
SYNDIG1
P2RX3
ADRA1A
KCNJ8
KCNJ2
PODXL
SH3BP4
SLC6A8
CNTN6
PCDH8
RHCG
ABCA8
EPHA2
HMCN1
ACKR3
GLRA1
CRB2
SLC5A12
GRM1
LDLRAP1
SHROOM4
ITGA5
PDPN
DHRS3
ADGRV1
SLC6A5
P2RY1
PKHD1
NETO2
FOSL1
GPR157
MUC1
ENPP1
DLL1
STON1

GO:CC
GO:0009986
cell_surface
36

PROM1
CD9
ABCC2
FAS
FGFR3
TGFBR3
HMMR
CEACAM1
LRP2
FOLH1
ITGB8
WNT5A
TNN
CXCR4
HSPA2
TSPAN8
PDGFRA
THBS1
MFGE8
EPHA2
ACKR3
EPHA5
GLRA1
TPBG
ITGA5
ADGRV1
P2RY1
PKHD1
TNFRSF10C
UNC5C
ROR1
RTN4RL2
ENPP1
NTRK1
FCGR3A
MICB

GO:CC
GO:0005576
extracellular_region
98

PROM1
PGLYRP1
CD9
DDX11
ATP1A2
FAS
LTBP1
LAMC3
CNN2
FGFR3
TGFBR3
WNT8B
CEACAM1
LRP2
CHMP2B
FOLH1
ANGPT2
CCDC80
ADA2
TLL2
PCSK5
HMOX1
JAG1
CD37
ITGB8
LFNG
WNT3
SMOC2
MAN2A1
SERPINI2
WNT5A
WLS
CCN2
TNN
CRISPLD1
SCPEP1
CXCR4
AHNAK
CD70
HSPA2
TSPAN8
CPA4
PODXL
SH3BP4
ANGPTL8
GDF15
CEMIP2
ENPP2
FGFBP2
THBS1
FBN2
HAPLN3
RHCG
MFGE8
HMCN1
THNSL2
CRB2
SLC5A12
CCDC3
SEMA3D
ANGPT1
CLEC18A
ADAMTS4
WNT9B
NPHS1
OLFML2B
ADGRV1
FNDC1
ARMC3
TAC3
TMIGD2
RSPO1
THBS3
PKHD1
HSPA6
NDNF
GNG5
CTSF
FGFBP3
IL17RA
ADAMTSL1
COL18A1
MUC1
RTN4RL2
TMPRSS11A
COL4A1
ISG15
WNT7B
POTEE
ZP3
TMEM63A
POTEI
H4C3
H3C12
ENPP1
DLL1
FCGR3A
MICB

GO:CC
GO:0005694
chromosome
55

TEAD3
TFAP2B
PAX7
DDX11
THOC1
CHMP2B
LHX5
SALL4
EBF3
RFX4
PROX1
ESRRB
NEUROD4
HSPA2
FOXJ1
CASZ1
PAX3
TFAP2A
SMAD6
ASCL1
MAEL
CREB3L4
SOX13
MEIS1
HES6
THRB
POU4F2
POU4F1
LMX1A
EN1
MSX1
EBF1
TLX3
EN2
UNCX
PHOX2A
GBX2
GSX1
IRX1
PKHD1
FOXB1
FOSL1
IRX5
OLIG3
IRX3
NHLH2
ZHX2
POU3F2
MUC1
POU3F1
HES4
H4C3
H3C12
HES5
TSIX

GO:CC
GO:0062023
collagen-containing_extracellular_matrix
21

LTBP1
LAMC3
ANGPT2
CCDC80
SMOC2
WNT5A
CCN2
TNN
GDF15
THBS1
FBN2
MFGE8
HMCN1
ANGPT1
ADAMTS4
THBS3
CTSF
FGFBP3
COL18A1
COL4A1
ZP3

GO:CC	GO:CC	GO:CC	GO:CC	GO:CC	GO:CC	GO:MF	GO:MF	GO:MF	GO:MF	GO:MF
GO:0031004	GO:0005615	GO:0008282	GO:1903561	GO:0065010	GO:0043230	GO:0000981	GO:0003700	GO:0000987	GO:1990837	GO:0000977
potassium_ion-transporting_ATPase_complex	extracellular_space	inward_rectifying_potassium_channel	extracellular_vesicle	extracellular_membrane-bounded_organelle	extracellular_organelle	DNA-binding_transcription_factor_activity__RNA_polymerase_II-specific	DNA-binding_transcription_factor_activity	cis-regulatory_region_sequence-specific_DNA_binding	sequence-specific_double-stranded_DNA_binding	RNA_polymerase_II_transcription_regulatory_region_sequence-specific_DNA_binding
3	78	3	54	54	54	51	52	47	55	51
ABCC8	PROM1	ABCC8	PROM1	PROM1	PROM1	TEAD3	TEAD3	TEAD3	TEAD3	TEAD3
ABCC9	PGLYRP1	ABCC9	PGLYRP1	PGLYRP1	PGLYRP1	TFAP2B	TFAP2B	TFAP2B	TFAP2B	TFAP2B
KCNJ8	CD9	KCNJ8	CD9	CD9	CD9	PAX7	PAX7	PAX7	PAX7	PAX7
	DDX11		DDX11	DDX11	DDX11	LHX5	LHX5	SALL4	DDX11	LHX5
	FAS		ATP1A2	ATP1A2	ATP1A2	SALL4	SALL4	EBF3	LHX5	SALL4
	TGFBR3		FAS	FAS	FAS	EBF3	EBF3	RFX4	SALL4	EBF3
	WNT8B		TGFBR3	TGFBR3	TGFBR3	RFX4	RFX4	PROX1	EBF3	RFX4
	CEACAM1		CEACAM1	CEACAM1	CEACAM1	PROX1	PROX1	ELL2	RFX4	PROX1
	LRP2		LRP2	LRP2	LRP2	ESRRB	ESRRB	ESRRB	PROX1	ESRRB
	CHMP2B		CHMP2B	CHMP2B	CHMP2B	NEUROD4	NEUROD4	NEUROD4	ELL2	NEUROD4
	FOLH1		FOLH1	FOLH1	FOLH1	FOXJ1	FOXJ1	FOXJ1	ESRRB	FOXJ1
	ANGPT2		CD37	CD37	CD37	CASZ1	CASZ1	PAX3	NEUROD4	CASZ1
	ADA2		ITGB8	ITGB8	ITGB8	PAX3	PAX3	TFAP2A	FOXJ1	PAX3
	TLL2		LFNG	LFNG	LFNG	TFAP2A	TFAP2A	PRDM5	CASZ1	TFAP2A
	PCSK5		WNT3	WNT3	WNT3	PRDM5	PRDM5	ASCL1	PAX3	PRDM5
	HMOX1		MAN2A1	MAN2A1	MAN2A1	ASCL1	ASCL1	CREB3L4	TFAP2A	ASCL1
	CD37		SERPINI2	SERPINI2	SERPINI2	CREB3L4	CREB3L4	SOX13	SMAD6	CREB3L4
	ITGB8		WNT5A	WNT5A	WNT5A	SOX13	SOX13	MEIS1	PRDM5	SOX13
	WNT3		WLS	WLS	WLS	MEIS1	MEIS1	HES6	ASCL1	MEIS1
	SMOC2		CRISPLD1	CRISPLD1	CRISPLD1	HES6	HES6	THRB	CREB3L4	HES6
	MAN2A1		SCPEP1	SCPEP1	SCPEP1	THRB	THRB	POU4F2	SOX13	THRB
	SERPINI2		CXCR4	CXCR4	CXCR4	POU4F2	POU4F2	POU4F1	MEIS1	POU4F2
	WNT5A		AHNAK	AHNAK	AHNAK	POU4F1	POU4F1	ZIC3	HES6	POU4F1
	WLS		CD70	CD70	CD70	ZIC3	ZIC3	EN1	THRB	ZIC3
	CCN2		HSPA2	HSPA2	HSPA2	LMX1A	LMX1A	MSX1	POU4F2	LMX1A
	TNN		TSPAN8	TSPAN8	TSPAN8	EN1	EN1	EBF1	POU4F1	EN1
	CRISPLD1		PODXL	PODXL	PODXL	MSX1	MSX1	TLX3	ZIC3	MSX1
	SCPEP1		SH3BP4	SH3BP4	SH3BP4	EBF1	EBF1	EN2	LMX1A	EBF1
	CXCR4		GDF15	GDF15	GDF15	TLX3	TLX3	GSX1	EN1	TLX3
	AHNAK		CEMIP2	CEMIP2	CEMIP2	EN2	EN2	IRX1	MSX1	EN2
	CD70		THBS1	THBS1	THBS1	UNCX	UNCX	FOXB1	EBF1	PHOX2A
	HSPA2		RHCG	RHCG	RHCG	PHOX2A	PHOX2A	FOSL1	TLX3	GBX2
	TSPAN8		MFGE8	MFGE8	MFGE8	GBX2	GBX2	NR2F1	EN2	GSX1
	CPA4		HMCN1	HMCN1	HMCN1	GSX1	GSX1	IRX5	UNCX	IRX1
	PODXL		CRB2	CRB2	CRB2	IRX1	IRX1	OLIG3	PHOX2A	FOXB1
	SH3BP4		SLC5A12	SLC5A12	SLC5A12	FOXB1	FOXB1	IRX3	GBX2	FOSL1
	GDF15		ANGPT1	ANGPT1	ANGPT1	FOSL1	FOSL1	NHLH2	GSX1	NR2F1
	CEMIP2		NPHS1	NPHS1	NPHS1	NR2F1	NR2F1	ZNF662	IRX1	IRX5
	ENPP2		ADGRV1	ADGRV1	ADGRV1	IRX5	IRX5	POU3F2	FOXB1	OLIG3
	FGFBP2		ARMC3	ARMC3	ARMC3	OLIG3	OLIG3	MUC1	FOSL1	IRX3
	THBS1		PKHD1	PKHD1	PKHD1	IRX3	IRX3	NR2F2	NR2F1	NHLH2
	HAPLN3		HSPA6	HSPA6	HSPA6	NHLH2	NHLH2	POU3F1	IRX5	ZNF662
	RHCG		GNG5	GNG5	GNG5	ZHX2	ZHX2	HES4	OLIG3	POU3F2
	MFGE8		CTSF	CTSF	CTSF	POU3F2	ZNF662	SKOR1	IRX3	MUC1
	HMCN1		COL18A1	COL18A1	COL18A1	NR2F2	POU3F2	LITAF	NHLH2	NR2F2
	THNSL2		MUC1	MUC1	MUC1	POU3F1	NR2F2	HES5	ZNF662	POU3F1
	CRB2		RTN4RL2	RTN4RL2	RTN4RL2	HES4	POU3F1	SKOR2	POU3F2	HES4
	SLC5A12		WNT7B	WNT7B	WNT7B	SKOR1	HES4		MUC1	SKOR1
	SEMA3D		POTEE	POTEE	POTEE	LITAF	SKOR1		NR2F2	LITAF
	ANGPT1		TMEM63A	TMEM63A	TMEM63A	HES5	LITAF		POU3F1	HES5
	CLEC18A		POTEI	POTEI	POTEI	SKOR2	HES5		HES4	SKOR2
	ADAMTS4		H4C3	H4C3	H4C3		SKOR2		SKOR1	
	WNT9B		H3C12	H3C12	H3C12				LITAF	
	NPHS1		FCGR3A	FCGR3A	FCGR3A				HES5	
	OLFML2B								SKOR2	
	ADGRV1									
	ARMC3									
	TAC3									
	TMIGD2									
	PKHD1									
	HSPA6									
	NDNF									
	GNG5									
	CTSF									
	COL18A1									
	MUC1									
	RTN4RL2									
	COL4A1									
	WNT7B									
	POTEE									
	ZP3									
	TMEM63A									
	POTEI									
	H4C3									
	H3C12									
	ENPP1									
	FCGR3A									
	MICB									

GO:MF
GO:0000976
transcription_cis-regulatory_region_binding
53

TEAD3
TFAP2B
PAX7
LHX5
SALL4
EBF3
RFX4
PROX1
ELL2
ESRRB
NEUROD4
FOXJ1
CASZ1
PAX3
TFAP2A
SMAD6
PRDM5
ASCL1
CREB3L4
SOX13
MEIS1
HES6
THRB
POU4F2
POU4F1
ZIC3
LMX1A
EN1
MSX1
EBF1
TLX3
EN2
PHOX2A
GBX2
GSX1
IRX1
FOXB1
FOSL1
NR2F1
IRX5
OLIG3
IRX3
NHLH2
ZNF662
POU3F2
MUC1
NR2F2
POU3F1
HES4
SKOR1
LITAF
HES5
SKOR2

GO:MF
GO:0001067
transcription_regulatory_region_nucleic_acid_binding
53

TEAD3
TFAP2B
PAX7
LHX5
SALL4
EBF3
RFX4
PROX1
ELL2
ESRRB
NEUROD4
FOXJ1
CASZ1
PAX3
TFAP2A
SMAD6
PRDM5
ASCL1
CREB3L4
SOX13
MEIS1
HES6
THRB
POU4F2
POU4F1
ZIC3
LMX1A
EN1
MSX1
EBF1
TLX3
EN2
PHOX2A
GBX2
GSX1
IRX1
FOXB1
FOSL1
NR2F1
IRX5
OLIG3
IRX3
NHLH2
ZNF662
POU3F2
MUC1
NR2F2
POU3F1
HES4
SKOR1
LITAF
HES5
SKOR2

GO:MF
GO:0019838
growth_factor_binding
14

RHBDF1
LTBP1
FGFR3
TGFBR3
LRP2
CCN2
PDGFRA
FGFBP2
THBS1
EPHA2
NTRK2
FGFBP3
COL4A1
NTRK1

GO:MF
GO:0043565
sequence-specific_DNA_binding
56

TEAD3
TFAP2B
PAX7
DDX11
LHX5
SALL4
EBF3
RFX4
PROX1
ELL2
ESRRB
NEUROD4
FOXJ1
CASZ1
PAX3
TFAP2A
SMAD6
PRDM5
ASCL1
MAEL
CREB3L4
SOX13
MEIS1
HES6
THRB
POU4F2
POU4F1
ZIC3
LMX1A
EN1
MSX1
EBF1
TLX3
EN2
UNCX
PHOX2A
GBX2
GSX1
IRX1
FOXB1
FOSL1
NR2F1
IRX5
OLIG3
IRX3
NHLH2
ZNF662
POU3F2
MUC1
NR2F2
POU3F1
HES4
SKOR1
LITAF
HES5
SKOR2

GO:MF
GO:0000978
RNA_polymerase_II_cis-regulatory_region_sequence-specific_DNA_binding
45

TEAD3
TFAP2B
PAX7
SALL4
EBF3
RFX4
PROX1
ESRRB
NEUROD4
FOXJ1
PAX3
TFAP2A
PRDM5
ASCL1
CREB3L4
SOX13
MEIS1
HES6
THRB
POU4F2
POU4F1
ZIC3
EN1
EBF1
TLX3
EN2
GSX1
IRX1
FOXB1
FOSL1
NR2F1
IRX5
OLIG3
IRX3
NHLH2
ZNF662
POU3F2
MUC1
NR2F2
POU3F1
HES4
SKOR1
LITAF
HES5
SKOR2

GO:MF
GO:0003690
double-stranded_DNA_binding
55

TEAD3
TFAP2B
PAX7
DDX11
LHX5
SALL4
EBF3
RFX4
PROX1
ELL2
ESRRB
NEUROD4
FOXJ1
CASZ1
PAX3
TFAP2A
SMAD6
PRDM5
ASCL1
CREB3L4
SOX13
MEIS1
HES6
THRB
POU4F2
POU4F1
ZIC3
LMX1A
EN1
MSX1
EBF1
TLX3
EN2
UNCX
PHOX2A
GBX2
GSX1
IRX1
FOXB1
FOSL1
NR2F1
IRX5
OLIG3
IRX3
NHLH2
ZNF662
POU3F2
MUC1
NR2F2
POU3F1
HES4
SKOR1
LITAF
HES5
SKOR2

GO:MF
GO:0140110
transcription_regulator_activity
59

TEAD3
TFAP2B
PAX7
WWTR1
LMCD1
LHX5
SALL4
TOX3
EBF3
RFX4
PROX1
ESRRB
NEUROD4
AJUBA
FOXJ1
CASZ1
PAX3
TFAP2A
PRDM5
ASCL1
CREB3L4
SOX13
MEIS1
HES6
THRB
POU4F2
POU4F1
ZIC3
LMX1A
EN1
MSX1
EBF1
TLX3
EN2
UNCX
PHOX2A
GBX2
GSX1
IRX1
FOXB1
FOSL1
NR2F1
IRX5
OLIG3
IRX3
NHLH2
ZHX2
ZNF662
MAML2
POU3F2
MUC1
NR2F2
POU3F1
HES4
SKOR1
LITAF
HES5
ZXDA
SKOR2

GO:MF
GO:0019199
transmembrane_receptor_protein_kinase_activity
10

LTBP1
FGFR3
TGFBR3
PDGFRA
BMPR1B
EPHA2
EPHA5
NTRK2
ROR1
NTRK1

GO:MF
GO:0005102
signaling_receptor_binding
48

CD9
TRAF1
TGFBR3
WNT8B
OPRK1
DLL3
ANGPT2
ADA2
JAG1
SYNDIG1
ITGB8
RNF43
WNT3
WNT5A
CBLB
CCN2
TNN
KCNJ8
CD70
TSPAN8
ANGPTL8
GDF15
NES
PDGFRA
EDNRB
THBS1
SMAD6
MFGE8
THNSL2
MEGF10
SEMA3D
ANGPT1
LDLRAP1
WNT9B
ITGA5
PDPN
TAC3
RSPO1
P2RY1
NETO2
IL17RA
ISG15
WNT7B
ZP3
ENPP1
NTRK1
DLL1
MICB

GO:MF
GO:0001228
DNA-binding_transcription_activator_activity__RNA_polymerase_II-specific
22

TFAP2B
EBF3
RFX4
ESRRB
FOXJ1
CASZ1
TFAP2A
CREB3L4
MEIS1
POU4F2
POU4F1
ZIC3
LMX1A
MSX1
PHOX2A
GBX2
GSX1
FOSL1
IRX3
NHLH2
POU3F2
LITAF

GO:MF
GO:0001216
DNA-binding_transcription_activator_activity
22

TFAP2B
EBF3
RFX4
ESRRB
FOXJ1
CASZ1
TFAP2A
CREB3L4
MEIS1
POU4F2
POU4F1
ZIC3
LMX1A
MSX1
PHOX2A
GBX2
GSX1
FOSL1
IRX3
NHLH2
POU3F2
LITAF

GO:MF
GO:0003677
DNA_binding
65

TEAD3
TFAP2B
PAX7
DDX11
THOC1
LHX5
SALL4
TOX3
EBF3
RFX4
PRDM13
TTF2
PROX1
ELL2
ESRRB
NEUROD4
FOXJ1
CASZ1
PAX3
TFAP2A
SMAD6
PRDM5
ASCL1
TAF4B
MAEL
CREB3L4
SOX13
MEIS1
HES6
THRB
POU4F2
POU4F1
ZIC3
LMX1A
EN1
MSX1
EBF1
TLX3
EN2
UNCX
PHOX2A
GBX2
GSX1
MGMT
IRX1
FOXB1
FOSL1
NR2F1
IRX5
OLIG3
IRX3
NHLH2
ZHX2
ZNF662
POU3F2
MUC1
NR2F2
POU3F1
HES4
SKOR1
LITAF
H4C3
H3C12
HES5
SKOR2

GO:MF
GO:0060089
molecular_transducer_activity
44

ABCC8
PGLYRP1
FAS
LTBP1
PTGER3
FGFR3
ABCC9
TGFBR3
PLXNA2
OPRK1
ITGB8
P2RX3
ESRRB
ADRA1A
CXCR4
PDGFRA
EDNRB
BMPR1B
LGR5
LPAR6
EPHA2
ACKR3
EPHA5
GLRA1
NTRK2
THRB
GRM1
DAPL1
ADGRV1
TMIGD2
P2RY1
PKHD1
TNFRSF10C
NR2F1
IL17RA
GPR157
UNC5C
ROR1
NR2F2
RTN4RL2
TMEM63A
NTRK1
FCGR3A
LGR4

GO:MF
GO:0038023
signaling_receptor_activity
44

ABCC8
PGLYRP1
FAS
LTBP1
PTGER3
FGFR3
ABCC9
TGFBR3
PLXNA2
OPRK1
ITGB8
P2RX3
ESRRB
ADRA1A
CXCR4
PDGFRA
EDNRB
BMPR1B
LGR5
LPAR6
EPHA2
ACKR3
EPHA5
GLRA1
NTRK2
THRB
GRM1
DAPL1
ADGRV1
TMIGD2
P2RY1
PKHD1
TNFRSF10C
NR2F1
IL17RA
GPR157
UNC5C
ROR1
NR2F2
RTN4RL2
TMEM63A
NTRK1
FCGR3A
LGR4

GO:MF
GO:0005109
frizzled_binding
6

WNT8B
RNF43
WNT3
WNT5A
WNT9B
WNT7B

KEGG
KEGG:05165
Human_papillomavirus_infection
23

FAS
LAMC3
WNT8B
JAG1
CDK6
ITGB8
LFNG
WNT3
WNT5A
TNN
CDKN1A
THBS1
CREB3L4
HES6
WNT9B
ITGA5
THBS3
MAML2
COL4A1
ISG15
WNT7B
HES4
HES5

KEGG
KEGG:04550
Signaling_pathways_regulating_pluripotency_of_stem_cells
12

FGFR3
WNT8B
LHX5
WNT3
WNT5A
ID3
ESRRB
BMPR1B
MEIS1
ZIC3
WNT9B
WNT7B

KEGG
KEGG:04151
PI3K-Akt_signaling_pathway
19

LAMC3
FGFR3
ANGPT2
CDK6
ITGB8
TNN
CDKN1A
PDGFRA
THBS1
LPAR6
EPHA2
CREB3L4
NTRK2
ANGPT1
ITGA5
THBS3
GNG5
COL4A1
NTRK1

KEGG
KEGG:05200
Pathways_in_cancer
24

FAS
LAMC3
PTGER3
TRAF1
FGFR3
WNT8B
DLL3
HMOX1
JAG1
CDK6
WNT3
WNT5A
CXCR4
CDKN1A
PDGFRA
EDNRB
LPAR6
WNT9B
GNG5
COL4A1
WNT7B
HES5
NTRK1
DLL1

KEGG
KEGG:05224
Breast_cancer
11

WNT8B
DLL3
JAG1
CDK6
WNT3
WNT5A
CDKN1A
WNT9B
WNT7B
HES5
DLL1

REAC
REAC:R-HSA-3238698
WNT_ligand_biogenesis_and_trafficking
6

WNT8B
WNT3
WNT5A
WLS
WNT9B
WNT7B

REAC
REAC:R-HSA-162582
Signal_Transduction
69

CYP26B1
WWTR1
FAS
LTBP1
LAMC3
PTGER3
TRAF1
WNT8B
CDC14A
OPRK1
CYP26A1
PCSK5
SALL4
JAG1
ITGB8
LFNG
RNF43
WNT3
CPT1A
WNT5A
PLA2G4A
WLS
ID3
ADRA1A
CXCR4
PDE1B
CDKN1A
DOCK6
PDGFRA
EDNRB
FGFBP2
THBS1
SMAD6
BMPR1B
LGR5
ASCL1
LPAR6
EPHA2
RGS16
SOX13
ACKR3
BOC
RGS20
NTRK2
GRM1
ANGPT1
WNT9B
ITGA5
DHRS3
ARHGAP25
CRABP1
TAC3
RSPO1
THBS3
P2RY1
GNG5
FGFBP3
FOSL1
MAML2
ROR1
COL4A1
WNT7B
POTEE
H4C3
H3C12
HES5
NTRK1
DLL1
LGR4

REAC
REAC:R-HSA-1296025
ATP_sensitive_Potassium_channels
3

ABCC8
ABCC9
KCNJ8

TF
TF:M01865
Factor:_BTEB3;_motif:_BNRNGGGAGGNGT
211

CYP26B1
ABCC8
RHBDF1
TEAD3
TFAP2B
CD9
DDX11
WWTR1
ATP1A2
FAS
LTBP1
LAMC3
PTGER3
FGFR3
TGFBR3
LMCD1
PLXNA2
CDC14A
CEACAM1
LRP2
CHMP2B
SLCO1A2
FOLH1
NOX4
DLL3
CCDC80
ADA2
TMEM38B
CYP26A1
PCSK5
PRODH
KCNK10
SALL4
NKAIN4
JAG1
SYNDIG1
TOX3
ITGB8
LFNG
TSPAN12
EBF3
RNF43
WNT3
PRMT8
RFX4
SMOC2
MAN2A1
WNT5A
CBLB
EFCC1
CFAP92
WLS
ID3
PROX1
CA14
CCN2
ELL2
ESRRB
TNN
SCPEP1
KCNJ8
CXCR4
NEUROD4
PDE1B
CDKN1A
CLYBL
CD70
PODXL
AJUBA
FOXJ1
SH3BP4
DOCK6
ANGPTL8
HRC
SLC6A8
CASZ1
IQCA1
NES
PDGFRA
CEMIP2
PCDH8
EDNRB
GYPC
FGFBP2
THBS1
SMAD6
PRDM5
FBN2
LGR5
ASCL1
GRTP1
HAPLN3
RHCG
MFGE8
GREB1L
TTYH2
EPHA2
SYPL2
ILDR2
CREB3L4
SYT2
MEIS1
THNSL2
HES6
BOC
EPHA5
GLRA1
RGS20
NTRK2
CRB2
KLHL35
ABTB3
CCDC3
POU4F2
GRM1
SEMA3D
ANKRD29
ROBO3
PDLIM3
ZIC3
LDLRAP1
WNT9B
NPHS1
ITGA5
OMA1
LMX1A
EN1
MSX1
INAVA
NUAK2
ADGRV1
EBF1
EN2
UNCX
ARMC3
HECTD2
PHOX2A
SLC6A5
CRABP1
RBPMS2
TAC3
TMIGD2
GBX2
THBS3
GSX1
IRX1
NETO2
FOXB1
MBOAT1
SUCLG2
STARD5
NDNF
TNFRSF10C
SNX33
GNG5
FAM53A
IGDCC3
CHST1
FOSL1
NR2F1
PRIMA1
IRX5
IRX3
IL17RA
CA8
ZHX2
TMTC2
RPRML
GPR157
FAM89A
UNC5C
COL18A1
ZNF662
KCNH8
POU3F2
TMEM255B
ROR1
MUC1
POU3F1
SLITRK2
RTN4RL2
COL4A1
DIPK1C
WNT7B
POTEE
HES4
ZP3
DYNLT4
SKOR1
PCDH18
TMEM63A
POTEI
TMEM26
ENPP1
HES5
ZXDA
NTRK1
DLL1
PPP1R14C
FCGR3A
RBM20
MICB
LGR4
VGLL3
TBC1D26
SKOR2
TSPAN19
STON1
FIGNL2
LINC00672
MUSTN1

TF
TF:M01587
Factor:_FPM315;_motif:_SRGGGAGGAGGN
88

CYP26B1
ABCC8
RHBDF1
TEAD3
DDX11
WWTR1
LTBP1
PTGER3
FGFR3
TGFBR3
LMCD1
CDC14A
CEACAM1
ADA2
CYP26A1
KCNK10
SALL4
NKAIN4
SYNDIG1
TOX3
ITGB8
RFX4
SLC27A6
CBLB
EFCC1
CCN2
ESRRB
CXCR4
PDE1B
KCNJ2
CDKN1A
USP6
FOXJ1
DOCK6
SLC6A8
NES
TFAP2A
ASCL1
MFGE8
RHBDL3
TTYH2
SYPL2
SYT2
HES6
RGS20
NTRK2
CRB2
KLHL1
ABTB3
POU4F2
GRM1
ANKRD29
ZIC3
LDLRAP1
WNT9B
NUAK2
ADGRV1
EN2
SLC6A5
TAC3
GBX2
MGMT
SUCLG2
TNFRSF10C
SNX33
IGDCC3
CHST1
NR2F1
TMTC2
GPR157
FAM89A
UNC5C
POU3F2
MUC1
SLITRK2
WNT7B
POTEE
POTEI
TMEM26
ENPP1
HES5
ZXDA
NTRK1
DLL1
PPP1R14C
RBM20
STON1
FIGNL2

TF
TF:M10107
Factor:_DB1;_motif:_GGRRRRGRRGGAGGGGGNGRRR
136

CYP26B1
ABCC8
TEAD3
PAX7
WWTR1
FAS
LTBP1
LAMC3
FGFR3
TGFBR3
LMCD1
PLXNA2
CDC14A
FOLH1
NOX4
LHX5
PCSK5
KCNK10
SALL4
NKAIN4
JAG1
TOX3
ITGB8
EBF3
RNF43
P2RX3
PRMT8
RFX4
SMOC2
MAN2A1
SLC27A6
EFCC1
WLS
PROX1
ESRRB
SCPEP1
KCNJ8
PDE1B
PODXL
FOXJ1
DOCK6
GDF15
HRC
SLC6A8
PRAM1
PDGFRA
CEMIP2
ENPP2
TFAP2A
FGFBP2
SMAD6
FBN2
ASCL1
GRTP1
GREB1L
SYT2
THNSL2
HES6
BOC
EPHA5
MEGF10
GLRA1
TPBG
RGS20
CRB2
KLHL35
KLHL1
CCDC3
POU4F2
SEMA3D
ANKRD29
ROBO3
PDLIM3
PIEZO2
ZIC3
CLEC18A
SHROOM4
WNT9B
ITGA5
PDPN
DHRS3
OMA1
EN1
MSX1
NUAK2
ADGRV1
EBF1
EN2
UNCX
SLC6A5
RBPMS2
GBX2
RSPO1
IRX1
PAH
FOXB1
SUCLG2
NDNF
TNFRSF10C
SNX33
GNG5
FAM53A
IGDCC3
CHST1
NR2F1
IRX5
OLIG3
IRX3
ZHX2
TMTC2
UNC5C
COL18A1
KCNH8
MAML2
POU3F2
CA13
ROR1
MUC1
NR2F2
SLITRK2
RTN4RL2
COL4A1
HES4
SKOR1
PCDH18
TMEM26
HES5
ZXDA
DLL1
PPP1R14C
RBM20
VGLL3
STON1
PTCHD4
FIGNL2
LINC00672

WP
WP:WP2064
Neural_crest_differentiation
13

TFAP2B
PAX7
FGFR3
LHX5
DLL3
PAX3
TFAP2A
ASCL1
MSX1
GBX2
OLIG3
HES5
DLL1

WP
WP:WP5052
Nephrogenesis
5

JAG1
GREB1L
MEIS1
WNT9B
RSPO1

WP
WP:WP2855
Dopaminergic_neurogenesis
6

ASCL1
LMX1A
EN1
MSX1
EN2
GBX2

Fig4G Down reg genes_Trevino

Intersection of Downregulated gene with cell type specific clusters in Trevino et. al. 2021

**ENSEMBL_gene_ID**	**Gene_symbol**	**cluster**	**cluser_name**
ENSG00000002587	HS3ST1	c0	Glutamatergic Neuron 2
ENSG00000008441	NFIX	c0	Glutamatergic Neuron 2
ENSG00000041515	MYO16	c0	Glutamatergic Neuron 2
ENSG00000075223	SEMA3C	c0	Glutamatergic Neuron 2
ENSG00000099260	PALMD	c0	Glutamatergic Neuron 2
ENSG00000101938	CHRDL1	c0	Glutamatergic Neuron 2
ENSG00000104888	SLC17A7	c0	Glutamatergic Neuron 2
ENSG00000106341	PPP1R17	c0	Glutamatergic Neuron 2
ENSG00000106689	LHX2	c0	Glutamatergic Neuron 2
ENSG00000109794	FAM149A	c0	Glutamatergic Neuron 2
ENSG00000120875	DUSP4	c0	Glutamatergic Neuron 2
ENSG00000134138	MEIS2	c0	Glutamatergic Neuron 2
ENSG00000147862	NFIB	c0	Glutamatergic Neuron 2
ENSG00000154678	PDE1C	c0	Glutamatergic Neuron 2
ENSG00000155886	SLC24A2	c0	Glutamatergic Neuron 2
ENSG00000155926	SLA	c0	Glutamatergic Neuron 2
ENSG00000166250	CLMP	c0	Glutamatergic Neuron 2
ENSG00000170370	EMX2	c0	Glutamatergic Neuron 2
ENSG00000171532	NEUROD2	c0	Glutamatergic Neuron 2
ENSG00000172461	FUT9	c0	Glutamatergic Neuron 2
ENSG00000172575	RASGRP1	c0	Glutamatergic Neuron 2
ENSG00000172985	SH3RF3	c0	Glutamatergic Neuron 2
ENSG00000180616	SSTR2	c0	Glutamatergic Neuron 2
ENSG00000181195	PENK	c0	Glutamatergic Neuron 2
ENSG00000185008	ROBO2	c0	Glutamatergic Neuron 2
ENSG00000006468	ETV1	c10	Late Radial Glia
ENSG00000007372	PAX6	c10	Late Radial Glia
ENSG00000021300	PLEKHB1	c10	Late Radial Glia
ENSG00000041982	TNC	c10	Late Radial Glia
ENSG00000042493	CAPG	c10	Late Radial Glia
ENSG00000066382	MPPED2	c10	Late Radial Glia
ENSG00000074416	MGLL	c10	Late Radial Glia
ENSG00000078295	ADCY2	c10	Late Radial Glia
ENSG00000079691	CARMIL1	c10	Late Radial Glia
ENSG00000080166	DCT	c10	Late Radial Glia
ENSG00000091490	SEL1L3	c10	Late Radial Glia
ENSG00000101134	DOK5	c10	Late Radial Glia
ENSG00000101938	CHRDL1	c10	Late Radial Glia
ENSG00000104332	SFRP1	c10	Late Radial Glia
ENSG00000106689	LHX2	c10	Late Radial Glia
ENSG00000113083	LOX	c10	Late Radial Glia
ENSG00000114315	HES1	c10	Late Radial Glia
ENSG00000115461	IGFBP5	c10	Late Radial Glia
ENSG00000115468	EFHD1	c10	Late Radial Glia
ENSG00000116194	ANGPTL1	c10	Late Radial Glia
ENSG00000119408	NEK6	c10	Late Radial Glia
ENSG00000120162	MOB3B	c10	Late Radial Glia
ENSG00000120738	EGR1	c10	Late Radial Glia
ENSG00000121310	ECHDC2	c10	Late Radial Glia
ENSG00000125266	EFNB2	c10	Late Radial Glia
ENSG00000126785	RHOJ	c10	Late Radial Glia
ENSG00000131018	SYNE1	c10	Late Radial Glia
ENSG00000132692	BCAN	c10	Late Radial Glia
ENSG00000135063	FAM189A2	c10	Late Radial Glia
ENSG00000137959	IFI44L	c10	Late Radial Glia
ENSG00000143318	CASQ1	c10	Late Radial Glia
ENSG00000144407	PTH2R	c10	Late Radial Glia
ENSG00000146592	CREB5	c10	Late Radial Glia
ENSG00000151834	GABRA2	c10	Late Radial Glia
ENSG00000154654	NCAM2	c10	Late Radial Glia
ENSG00000158234	FAIM	c10	Late Radial Glia
ENSG00000159176	CSRP1	c10	Late Radial Glia
ENSG00000162692	VCAM1	c10	Late Radial Glia
ENSG00000162981	FAM84A	c10	Late Radial Glia
ENSG00000163288	GABRB1	c10	Late Radial Glia
ENSG00000164171	ITGA2	c10	Late Radial Glia
ENSG00000164434	FABP7	c10	Late Radial Glia
ENSG00000164649	CDCA7L	c10	Late Radial Glia
ENSG00000164683	HEY1	c10	Late Radial Glia
ENSG00000168309	FAM107A	c10	Late Radial Glia
ENSG00000168461	RAB31	c10	Late Radial Glia
ENSG00000172461	FUT9	c10	Late Radial Glia
ENSG00000173511	VEGFB	c10	Late Radial Glia
ENSG00000177133	LINC00982	c10	Late Radial Glia
ENSG00000177283	FZD8	c10	Late Radial Glia

ENSG00000177807	KCNJ10	c10	Late Radial Glia
ENSG00000182050	MGAT4C	c10	Late Radial Glia
ENSG00000182103	FAM181B	c10	Late Radial Glia
ENSG00000183098	GPC6	c10	Late Radial Glia
ENSG00000188153	COL4A5	c10	Late Radial Glia
ENSG00000189221	MAOA	c10	Late Radial Glia
ENSG00000197565	COL4A6	c10	Late Radial Glia
ENSG00000197956	S100A6	c10	Late Radial Glia
ENSG00000198121	LPAR1	c10	Late Radial Glia
ENSG00000198739	LRRTM3	c10	Late Radial Glia
ENSG00000204128	C2orf72	c10	Late Radial Glia
ENSG00000213694	S1PR3	c10	Late Radial Glia
ENSG00000250722	SELENOP	c10	Late Radial Glia
ENSG00000256463	SALL3	c10	Late Radial Glia
ENSG00000263146	LINC01896	c10	Late Radial Glia
ENSG00000006468	ETV1	c11	Oligodendrocyte intermediate progenitor cell
ENSG00000021300	PLEKHB1	c11	Oligodendrocyte intermediate progenitor cell
ENSG00000041982	TNC	c11	Oligodendrocyte intermediate progenitor cell
ENSG00000079691	CARMIL1	c11	Oligodendrocyte intermediate progenitor cell
ENSG00000100095	SEZ6L	c11	Oligodendrocyte intermediate progenitor cell
ENSG00000103449	SALL1	c11	Oligodendrocyte intermediate progenitor cell
ENSG00000112902	SEMA5A	c11	Oligodendrocyte intermediate progenitor cell
ENSG00000114315	HES1	c11	Oligodendrocyte intermediate progenitor cell
ENSG00000115461	IGFBP5	c11	Oligodendrocyte intermediate progenitor cell
ENSG00000120738	EGR1	c11	Oligodendrocyte intermediate progenitor cell
ENSG00000126785	RHOJ	c11	Oligodendrocyte intermediate progenitor cell
ENSG00000132164	SLC6A11	c11	Oligodendrocyte intermediate progenitor cell
ENSG00000132692	BCAN	c11	Oligodendrocyte intermediate progenitor cell
ENSG00000139318	DUSP6	c11	Oligodendrocyte intermediate progenitor cell
ENSG00000146592	CREB5	c11	Oligodendrocyte intermediate progenitor cell
ENSG00000146938	NLGN4X	c11	Oligodendrocyte intermediate progenitor cell
ENSG00000152214	RIT2	c11	Oligodendrocyte intermediate progenitor cell
ENSG00000162670	BRINP3	c11	Oligodendrocyte intermediate progenitor cell
ENSG00000164434	FABP7	c11	Oligodendrocyte intermediate progenitor cell
ENSG00000164649	CDCA7L	c11	Oligodendrocyte intermediate progenitor cell
ENSG00000164683	HEY1	c11	Oligodendrocyte intermediate progenitor cell
ENSG00000168309	FAM107A	c11	Oligodendrocyte intermediate progenitor cell
ENSG00000168461	RAB31	c11	Oligodendrocyte intermediate progenitor cell
ENSG00000170370	EMX2	c11	Oligodendrocyte intermediate progenitor cell
ENSG00000171385	KCND3	c11	Oligodendrocyte intermediate progenitor cell
ENSG00000171867	PRNP	c11	Oligodendrocyte intermediate progenitor cell
ENSG00000173511	VEGFB	c11	Oligodendrocyte intermediate progenitor cell
ENSG00000182103	FAM181B	c11	Oligodendrocyte intermediate progenitor cell
ENSG00000187479	C11orf96	c11	Oligodendrocyte intermediate progenitor cell
ENSG00000197956	S100A6	c11	Oligodendrocyte intermediate progenitor cell
ENSG00000198739	LRRTM3	c11	Oligodendrocyte intermediate progenitor cell
ENSG00000204128	C2orf72	c11	Oligodendrocyte intermediate progenitor cell
ENSG00000205683	DPF3	c11	Oligodendrocyte intermediate progenitor cell
ENSG00000250722	SELENOP	c11	Oligodendrocyte intermediate progenitor cell
ENSG00000256463	SALL3	c11	Oligodendrocyte intermediate progenitor cell
ENSG00000260807	AC009041.2	c11	Oligodendrocyte intermediate progenitor cell
ENSG00000263146	LINC01896	c11	Oligodendrocyte intermediate progenitor cell
ENSG00000008441	NFIX	c12	Glutamatergic Neuron 6
ENSG00000048540	LMO3	c12	Glutamatergic Neuron 6
ENSG00000058404	CAMK2B	c12	Glutamatergic Neuron 6
ENSG00000068366	ACSL4	c12	Glutamatergic Neuron 6
ENSG00000075223	SEMA3C	c12	Glutamatergic Neuron 6
ENSG00000099260	PALMD	c12	Glutamatergic Neuron 6
ENSG00000101460	MAP1LC3A	c12	Glutamatergic Neuron 6
ENSG00000114279	FGF12	c12	Glutamatergic Neuron 6
ENSG00000117598	PLPPR5	c12	Glutamatergic Neuron 6
ENSG00000119771	KLHL29	c12	Glutamatergic Neuron 6
ENSG00000120875	DUSP4	c12	Glutamatergic Neuron 6
ENSG00000132932	ATP8A2	c12	Glutamatergic Neuron 6
ENSG00000132975	GPR12	c12	Glutamatergic Neuron 6
ENSG00000133134	BEX2	c12	Glutamatergic Neuron 6
ENSG00000134138	MEIS2	c12	Glutamatergic Neuron 6
ENSG00000136531	SCN2A	c12	Glutamatergic Neuron 6
ENSG00000145335	SNCA	c12	Glutamatergic Neuron 6
ENSG00000150594	ADRA2A	c12	Glutamatergic Neuron 6
ENSG00000151490	PTPRO	c12	Glutamatergic Neuron 6
ENSG00000152377	SPOCK1	c12	Glutamatergic Neuron 6
ENSG00000152953	STK32B	c12	Glutamatergic Neuron 6
ENSG00000155926	SLA	c12	Glutamatergic Neuron 6
ENSG00000159200	RCAN1	c12	Glutamatergic Neuron 6
ENSG00000164604	GPR85	c12	Glutamatergic Neuron 6
ENSG00000166250	CLMP	c12	Glutamatergic Neuron 6

ENSG00000171532	NEUROD2	c12	Glutamatergic Neuron 6
ENSG00000171951	SCG2	c12	Glutamatergic Neuron 6
ENSG00000172995	ARPP21	c12	Glutamatergic Neuron 6
ENSG00000176956	LY6H	c12	Glutamatergic Neuron 6
ENSG00000184905	TCEAL2	c12	Glutamatergic Neuron 6
ENSG00000186732	MPPED1	c12	Glutamatergic Neuron 6
ENSG00000186868	MAPT	c12	Glutamatergic Neuron 6
ENSG00000187164	SHTN1	c12	Glutamatergic Neuron 6
ENSG00000188580	NKAIN2	c12	Glutamatergic Neuron 6
ENSG00000196277	GRM7	c12	Glutamatergic Neuron 6
ENSG00000221866	PLXNA4	c12	Glutamatergic Neuron 6
ENSG00000260664	AC004158.1	c12	Glutamatergic Neuron 6
ENSG00000005884	ITGA3	c13	Subplate
ENSG00000006116	CACNG3	c13	Subplate
ENSG00000007237	GAS7	c13	Subplate
ENSG00000009694	TENM1	c13	Subplate
ENSG00000011347	SYT7	c13	Subplate
ENSG00000011677	GABRA3	c13	Subplate
ENSG00000018236	CNTN1	c13	Subplate
ENSG00000040731	CDH10	c13	Subplate
ENSG00000041515	MYO16	c13	Subplate
ENSG00000048540	LMO3	c13	Subplate
ENSG00000058335	RASGRF1	c13	Subplate
ENSG00000058404	CAMK2B	c13	Subplate
ENSG00000060656	PTPRU	c13	Subplate
ENSG00000066248	NGEF	c13	Subplate
ENSG00000068366	ACSL4	c13	Subplate
ENSG00000068831	RASGRP2	c13	Subplate
ENSG00000074317	SNCB	c13	Subplate
ENSG00000074416	MGLL	c13	Subplate
ENSG00000074590	NUAK1	c13	Subplate
ENSG00000075213	SEMA3A	c13	Subplate
ENSG00000078295	ADCY2	c13	Subplate
ENSG00000078725	BRINP1	c13	Subplate
ENSG00000079691	CARMIL1	c13	Subplate
ENSG00000091490	SEL1L3	c13	Subplate
ENSG00000100604	CHGA	c13	Subplate
ENSG00000101489	CELF4	c13	Subplate
ENSG00000102290	PCDH11X	c13	Subplate
ENSG00000104888	SLC17A7	c13	Subplate
ENSG00000107295	SH3GL2	c13	Subplate
ENSG00000108018	SORCS1	c13	Subplate
ENSG00000108231	LGI1	c13	Subplate
ENSG00000110786	PTPN5	c13	Subplate
ENSG00000112379	ARFGEF3	c13	Subplate
ENSG00000113327	GABRG2	c13	Subplate
ENSG00000113361	CDH6	c13	Subplate
ENSG00000113389	NPR3	c13	Subplate
ENSG00000114279	FGF12	c13	Subplate
ENSG00000115461	IGFBP5	c13	Subplate
ENSG00000116147	TNR	c13	Subplate
ENSG00000116183	PAPPA2	c13	Subplate
ENSG00000116254	CHD5	c13	Subplate
ENSG00000116983	HPCAL4	c13	Subplate
ENSG00000117154	IGSF21	c13	Subplate
ENSG00000117598	PLPPR5	c13	Subplate
ENSG00000117600	PLPPR4	c13	Subplate
ENSG00000121905	HPCA	c13	Subplate
ENSG00000124140	SLC12A5	c13	Subplate
ENSG00000124772	CPNE5	c13	Subplate
ENSG00000125851	PCSK2	c13	Subplate
ENSG00000127152	BCL11B	c13	Subplate
ENSG00000127561	SYNGR3	c13	Subplate
ENSG00000130643	CALY	c13	Subplate
ENSG00000131771	PPP1R1B	c13	Subplate
ENSG00000131773	KHDRBS3	c13	Subplate
ENSG00000132932	ATP8A2	c13	Subplate
ENSG00000132975	GPR12	c13	Subplate
ENSG00000133019	CHRM3	c13	Subplate
ENSG00000133134	BEX2	c13	Subplate
ENSG00000133401	PDZD2	c13	Subplate
ENSG00000133816	MICAL2	c13	Subplate
ENSG00000134207	SYT6	c13	Subplate
ENSG00000135333	EPHA7	c13	Subplate
ENSG00000136531	SCN2A	c13	Subplate
ENSG00000136928	GABBR2	c13	Subplate
ENSG00000136999	NOV	c13	Subplate

ENSG00000137261	KIAA0319	c13	Subplate
ENSG00000137727	ARHGAP20	c13	Subplate
ENSG00000138944	KIAA1644	c13	Subplate
ENSG00000139915	MDGA2	c13	Subplate
ENSG00000139973	SYT16	c13	Subplate
ENSG00000142408	CACNG8	c13	Subplate
ENSG00000142875	PRKACB	c13	Subplate
ENSG00000144290	SLC4A10	c13	Subplate
ENSG00000144331	ZNF385B	c13	Subplate
ENSG00000144460	NYAP2	c13	Subplate
ENSG00000145335	SNCA	c13	Subplate
ENSG00000145526	CDH18	c13	Subplate
ENSG00000145864	GABRB2	c13	Subplate
ENSG00000147676	MAL2	c13	Subplate
ENSG00000148123	PLPPR1	c13	Subplate
ENSG00000150594	ADRA2A	c13	Subplate
ENSG00000151917	BEND6	c13	Subplate
ENSG00000152377	SPOCK1	c13	Subplate
ENSG00000152402	GUCY1A2	c13	Subplate
ENSG00000153820	SPHKAP	c13	Subplate
ENSG00000154096	THY1	c13	Subplate
ENSG00000154429	CCSAP	c13	Subplate
ENSG00000154654	NCAM2	c13	Subplate
ENSG00000155265	GOLGA7B	c13	Subplate
ENSG00000155511	GRIA1	c13	Subplate
ENSG00000155886	SLC24A2	c13	Subplate
ENSG00000157087	ATP2B2	c13	Subplate
ENSG00000157152	SYN2	c13	Subplate
ENSG00000157388	CACNA1D	c13	Subplate
ENSG00000158258	CLSTN2	c13	Subplate
ENSG00000158560	DYNC1I1	c13	Subplate
ENSG00000158747	NBL1	c13	Subplate
ENSG00000159200	RCAN1	c13	Subplate
ENSG00000159307	SCUBE1	c13	Subplate
ENSG00000160460	SPTBN4	c13	Subplate
ENSG00000162552	WNT4	c13	Subplate
ENSG00000162975	KCNF1	c13	Subplate
ENSG00000163032	VSNL1	c13	Subplate
ENSG00000163536	SERPINI1	c13	Subplate
ENSG00000164076	CAMKV	c13	Subplate
ENSG00000164418	GRIK2	c13	Subplate
ENSG00000166501	PRKCB	c13	Subplate
ENSG00000167178	ISLR2	c13	Subplate
ENSG00000167414	GNG8	c13	Subplate
ENSG00000168959	GRM5	c13	Subplate
ENSG00000169213	RAB3B	c13	Subplate
ENSG00000169255	B3GALNT1	c13	Subplate
ENSG00000169851	PCDH7	c13	Subplate
ENSG00000169933	FRMPD4	c13	Subplate
ENSG00000170381	SEMA3E	c13	Subplate
ENSG00000170775	GPR37	c13	Subplate
ENSG00000170921	TANC2	c13	Subplate
ENSG00000171243	SOSTDC1	c13	Subplate
ENSG00000171246	NPTX1	c13	Subplate
ENSG00000171587	DSCAM	c13	Subplate
ENSG00000171867	PRNP	c13	Subplate
ENSG00000172995	ARPP21	c13	Subplate
ENSG00000174521	TTC9B	c13	Subplate
ENSG00000176956	LY6H	c13	Subplate
ENSG00000178695	KCTD12	c13	Subplate
ENSG00000180616	SSTR2	c13	Subplate
ENSG00000182050	MGAT4C	c13	Subplate
ENSG00000182732	RGS6	c13	Subplate
ENSG00000183166	CALN1	c13	Subplate
ENSG00000183715	OPCML	c13	Subplate
ENSG00000184905	TCEAL2	c13	Subplate
ENSG00000185008	ROBO2	c13	Subplate
ENSG00000185477	GPRIN3	c13	Subplate
ENSG00000186297	GABRA5	c13	Subplate
ENSG00000186310	NAP1L3	c13	Subplate
ENSG00000186642	PDE2A	c13	Subplate
ENSG00000186732	MPPED1	c13	Subplate
ENSG00000186868	MAPT	c13	Subplate
ENSG00000187135	VSTM2B	c13	Subplate
ENSG00000187164	SHTN1	c13	Subplate
ENSG00000197872	FAM49A	c13	Subplate
ENSG00000198216	CACNA1E	c13	Subplate

ENSG00000198626	RYR2	c13	Subplate
ENSG00000203685	STUM	c13	Subplate
ENSG00000215218	UBE2QL1	c13	Subplate
ENSG00000225206	MIR137HG	c13	Subplate
ENSG00000237515	SHISA9	c13	Subplate
ENSG00000255082	GRM5-AS1	c13	Subplate
ENSG00000260230	FRRS1L	c13	Subplate
ENSG00000260664	AC004158.1	c13	Subplate
ENSG00000272636	DOC2B	c13	Subplate
ENSG00000273079	GRIN2B	c13	Subplate
ENSG00000007372	PAX6	c14	neuronal intermedate progenitor cell
ENSG00000061337	LZTS1	c14	neuronal intermedate progenitor cell
ENSG00000091490	SEL1L3	c14	neuronal intermedate progenitor cell
ENSG00000100095	SEZ6L	c14	neuronal intermedate progenitor cell
ENSG00000106341	PPP1R17	c14	neuronal intermedate progenitor cell
ENSG00000106689	LHX2	c14	neuronal intermedate progenitor cell
ENSG00000120738	EGR1	c14	neuronal intermedate progenitor cell
ENSG00000122574	WIPF3	c14	neuronal intermedate progenitor cell
ENSG00000136104	RNASEH2B	c14	neuronal intermedate progenitor cell
ENSG00000145920	CPLX2	c14	neuronal intermedate progenitor cell
ENSG00000162374	ELAVL4	c14	neuronal intermedate progenitor cell
ENSG00000172575	RASGRP1	c14	neuronal intermedate progenitor cell
ENSG00000179431	FJX1	c14	neuronal intermedate progenitor cell
ENSG00000180616	SSTR2	c14	neuronal intermedate progenitor cell
ENSG00000181195	PENK	c14	neuronal intermedate progenitor cell
ENSG00000183098	GPC6	c14	neuronal intermedate progenitor cell
ENSG00000215218	UBE2QL1	c14	neuronal intermedate progenitor cell
ENSG00000256463	SALL3	c14	neuronal intermedate progenitor cell
ENSG00000007237	GAS7	c15	Glutamatergic Neuron 8
ENSG00000009694	TENM1	c15	Glutamatergic Neuron 8
ENSG00000018236	CNTN1	c15	Glutamatergic Neuron 8
ENSG00000040731	CDH10	c15	Glutamatergic Neuron 8
ENSG00000041515	MYO16	c15	Glutamatergic Neuron 8
ENSG00000048540	LMO3	c15	Glutamatergic Neuron 8
ENSG00000058404	CAMK2B	c15	Glutamatergic Neuron 8
ENSG00000060709	RIMBP2	c15	Glutamatergic Neuron 8
ENSG00000068366	ACSL4	c15	Glutamatergic Neuron 8
ENSG00000074211	PPP2R2C	c15	Glutamatergic Neuron 8
ENSG00000074317	SNCB	c15	Glutamatergic Neuron 8
ENSG00000078114	NEBL	c15	Glutamatergic Neuron 8
ENSG00000101134	DOK5	c15	Glutamatergic Neuron 8
ENSG00000101460	MAP1LC3A	c15	Glutamatergic Neuron 8
ENSG00000101489	CELF4	c15	Glutamatergic Neuron 8
ENSG00000102290	PCDH11X	c15	Glutamatergic Neuron 8
ENSG00000104833	TUBB4A	c15	Glutamatergic Neuron 8
ENSG00000107295	SH3GL2	c15	Glutamatergic Neuron 8
ENSG00000112379	ARFGEF3	c15	Glutamatergic Neuron 8
ENSG00000113327	GABRG2	c15	Glutamatergic Neuron 8
ENSG00000114279	FGF12	c15	Glutamatergic Neuron 8
ENSG00000116147	TNR	c15	Glutamatergic Neuron 8
ENSG00000116254	CHD5	c15	Glutamatergic Neuron 8
ENSG00000116983	HPCAL4	c15	Glutamatergic Neuron 8
ENSG00000117154	IGSF21	c15	Glutamatergic Neuron 8
ENSG00000117600	PLPPR4	c15	Glutamatergic Neuron 8
ENSG00000121905	HPCA	c15	Glutamatergic Neuron 8
ENSG00000127561	SYNGR3	c15	Glutamatergic Neuron 8
ENSG00000130643	CALY	c15	Glutamatergic Neuron 8
ENSG00000131018	SYNE1	c15	Glutamatergic Neuron 8
ENSG00000131773	KHDRBS3	c15	Glutamatergic Neuron 8
ENSG00000132932	ATP8A2	c15	Glutamatergic Neuron 8
ENSG00000132975	GPR12	c15	Glutamatergic Neuron 8
ENSG00000133134	BEX2	c15	Glutamatergic Neuron 8
ENSG00000135439	AGAP2	c15	Glutamatergic Neuron 8
ENSG00000136531	SCN2A	c15	Glutamatergic Neuron 8
ENSG00000136928	GABBR2	c15	Glutamatergic Neuron 8
ENSG00000137261	KIAA0319	c15	Glutamatergic Neuron 8
ENSG00000137727	ARHGAP20	c15	Glutamatergic Neuron 8
ENSG00000139973	SYT16	c15	Glutamatergic Neuron 8
ENSG00000140015	KCNH5	c15	Glutamatergic Neuron 8
ENSG00000142408	CACNG8	c15	Glutamatergic Neuron 8
ENSG00000142875	PRKACB	c15	Glutamatergic Neuron 8
ENSG00000144339	TMEFF2	c15	Glutamatergic Neuron 8
ENSG00000145335	SNCA	c15	Glutamatergic Neuron 8
ENSG00000145864	GABRB2	c15	Glutamatergic Neuron 8
ENSG00000146090	RASGEF1C	c15	Glutamatergic Neuron 8
ENSG00000148123	PLPPR1	c15	Glutamatergic Neuron 8
ENSG00000151490	PTPRO	c15	Glutamatergic Neuron 8

ENSG00000151834	GABRA2	c15	Glutamatergic Neuron 8
ENSG00000152214	RIT2	c15	Glutamatergic Neuron 8
ENSG00000152377	SPOCK1	c15	Glutamatergic Neuron 8
ENSG00000152402	GUCY1A2	c15	Glutamatergic Neuron 8
ENSG00000152953	STK32B	c15	Glutamatergic Neuron 8
ENSG00000152969	JAKMIP1	c15	Glutamatergic Neuron 8
ENSG00000153820	SPHKAP	c15	Glutamatergic Neuron 8
ENSG00000154096	THY1	c15	Glutamatergic Neuron 8
ENSG00000154975	CA10	c15	Glutamatergic Neuron 8
ENSG00000155265	GOLGA7B	c15	Glutamatergic Neuron 8
ENSG00000155511	GRIA1	c15	Glutamatergic Neuron 8
ENSG00000157087	ATP2B2	c15	Glutamatergic Neuron 8
ENSG00000158560	DYNC1I1	c15	Glutamatergic Neuron 8
ENSG00000158716	DUSP23	c15	Glutamatergic Neuron 8
ENSG00000159200	RCAN1	c15	Glutamatergic Neuron 8
ENSG00000159307	SCUBE1	c15	Glutamatergic Neuron 8
ENSG00000162951	LRRTM1	c15	Glutamatergic Neuron 8
ENSG00000163288	GABRB1	c15	Glutamatergic Neuron 8
ENSG00000163536	SERPINI1	c15	Glutamatergic Neuron 8
ENSG00000164076	CAMKV	c15	Glutamatergic Neuron 8
ENSG00000164116	GUCY1A3	c15	Glutamatergic Neuron 8
ENSG00000164604	GPR85	c15	Glutamatergic Neuron 8
ENSG00000165186	PTCHD1	c15	Glutamatergic Neuron 8
ENSG00000166006	KCNC2	c15	Glutamatergic Neuron 8
ENSG00000166501	PRKCB	c15	Glutamatergic Neuron 8
ENSG00000167414	GNG8	c15	Glutamatergic Neuron 8
ENSG00000168546	GFRA2	c15	Glutamatergic Neuron 8
ENSG00000168743	NPNT	c15	Glutamatergic Neuron 8
ENSG00000168959	GRM5	c15	Glutamatergic Neuron 8
ENSG00000169255	B3GALNT1	c15	Glutamatergic Neuron 8
ENSG00000169933	FRMPD4	c15	Glutamatergic Neuron 8
ENSG00000170381	SEMA3E	c15	Glutamatergic Neuron 8
ENSG00000171450	CDK5R2	c15	Glutamatergic Neuron 8
ENSG00000171867	PRNP	c15	Glutamatergic Neuron 8
ENSG00000171951	SCG2	c15	Glutamatergic Neuron 8
ENSG00000172995	ARPP21	c15	Glutamatergic Neuron 8
ENSG00000173511	VEGFB	c15	Glutamatergic Neuron 8
ENSG00000174469	CNTNAP2	c15	Glutamatergic Neuron 8
ENSG00000174521	TTC9B	c15	Glutamatergic Neuron 8
ENSG00000176956	LY6H	c15	Glutamatergic Neuron 8
ENSG00000182050	MGAT4C	c15	Glutamatergic Neuron 8
ENSG00000183166	CALN1	c15	Glutamatergic Neuron 8
ENSG00000184905	TCEAL2	c15	Glutamatergic Neuron 8
ENSG00000185477	GPRIN3	c15	Glutamatergic Neuron 8
ENSG00000185742	C11orf87	c15	Glutamatergic Neuron 8
ENSG00000186310	NAP1L3	c15	Glutamatergic Neuron 8
ENSG00000186732	MPPED1	c15	Glutamatergic Neuron 8
ENSG00000186868	MAPT	c15	Glutamatergic Neuron 8
ENSG00000187135	VSTM2B	c15	Glutamatergic Neuron 8
ENSG00000187164	SHTN1	c15	Glutamatergic Neuron 8
ENSG00000188580	NKAIN2	c15	Glutamatergic Neuron 8
ENSG00000196090	PTPRT	c15	Glutamatergic Neuron 8
ENSG00000196277	GRM7	c15	Glutamatergic Neuron 8
ENSG00000197872	FAM49A	c15	Glutamatergic Neuron 8
ENSG00000198216	CACNA1E	c15	Glutamatergic Neuron 8
ENSG00000215218	UBE2QL1	c15	Glutamatergic Neuron 8
ENSG00000237515	SHISA9	c15	Glutamatergic Neuron 8
ENSG00000253368	TRNP1	c15	Glutamatergic Neuron 8
ENSG00000260230	FRRS1L	c15	Glutamatergic Neuron 8
ENSG00000272636	DOC2B	c15	Glutamatergic Neuron 8
ENSG00000273079	GRIN2B	c15	Glutamatergic Neuron 8
ENSG00000004399	PLXND1	c16	Microglia
ENSG00000007264	MATK	c16	Microglia
ENSG00000042493	CAPG	c16	Microglia
ENSG00000074706	IPCEF1	c16	Microglia
ENSG00000100311	PDGFB	c16	Microglia
ENSG00000101347	SAMHD1	c16	Microglia
ENSG00000103449	SALL1	c16	Microglia
ENSG00000107738	VSIR	c16	Microglia
ENSG00000110324	IL10RA	c16	Microglia
ENSG00000118257	NRP2	c16	Microglia
ENSG00000118785	SPP1	c16	Microglia
ENSG00000119408	NEK6	c16	Microglia
ENSG00000120738	EGR1	c16	Microglia
ENSG00000134207	SYT6	c16	Microglia
ENSG00000135363	LMO2	c16	Microglia
ENSG00000139318	DUSP6	c16	Microglia

ENSG00000140280	LYSMD2	c16	Microglia
ENSG00000141524	TMC6	c16	Microglia
ENSG00000157514	TSC22D3	c16	Microglia
ENSG00000158716	DUSP23	c16	Microglia
ENSG00000158747	NBL1	c16	Microglia
ENSG00000166501	PRKCB	c16	Microglia
ENSG00000167895	TMC8	c16	Microglia
ENSG00000167994	RAB3IL1	c16	Microglia
ENSG00000168461	RAB31	c16	Microglia
ENSG00000172889	EGFL7	c16	Microglia
ENSG00000173511	VEGFB	c16	Microglia
ENSG00000178695	KCTD12	c16	Microglia
ENSG00000181381	DDX60L	c16	Microglia
ENSG00000184922	FMNL1	c16	Microglia
ENSG00000197971	MBP	c16	Microglia
ENSG00000204103	MAFB	c16	Microglia
ENSG00000245848	CEBPA	c16	Microglia
ENSG00000006468	ETV1	c17	Oligodendrocyte Progenitor Cell/Oligodendrocyte
ENSG00000018236	CNTN1	c17	Oligodendrocyte Progenitor Cell/Oligodendrocyte
ENSG00000021300	PLEKHB1	c17	Oligodendrocyte Progenitor Cell/Oligodendrocyte
ENSG00000078725	BRINP1	c17	Oligodendrocyte Progenitor Cell/Oligodendrocyte
ENSG00000092758	COL9A3	c17	Oligodendrocyte Progenitor Cell/Oligodendrocyte
ENSG00000100095	SEZ6L	c17	Oligodendrocyte Progenitor Cell/Oligodendrocyte
ENSG00000101445	PPP1R16B	c17	Oligodendrocyte Progenitor Cell/Oligodendrocyte
ENSG00000101542	CDH20	c17	Oligodendrocyte Progenitor Cell/Oligodendrocyte
ENSG00000102290	PCDH11X	c17	Oligodendrocyte Progenitor Cell/Oligodendrocyte
ENSG00000103528	SYT17	c17	Oligodendrocyte Progenitor Cell/Oligodendrocyte
ENSG00000104368	PLAT	c17	Oligodendrocyte Progenitor Cell/Oligodendrocyte
ENSG00000104833	TUBB4A	c17	Oligodendrocyte Progenitor Cell/Oligodendrocyte
ENSG00000112379	ARFGEF3	c17	Oligodendrocyte Progenitor Cell/Oligodendrocyte
ENSG00000112902	SEMA5A	c17	Oligodendrocyte Progenitor Cell/Oligodendrocyte
ENSG00000114279	FGF12	c17	Oligodendrocyte Progenitor Cell/Oligodendrocyte
ENSG00000116132	PRRX1	c17	Oligodendrocyte Progenitor Cell/Oligodendrocyte
ENSG00000116147	TNR	c17	Oligodendrocyte Progenitor Cell/Oligodendrocyte
ENSG00000117154	IGSF21	c17	Oligodendrocyte Progenitor Cell/Oligodendrocyte
ENSG00000120738	EGR1	c17	Oligodendrocyte Progenitor Cell/Oligodendrocyte
ENSG00000122584	NXPH1	c17	Oligodendrocyte Progenitor Cell/Oligodendrocyte
ENSG00000125845	BMP2	c17	Oligodendrocyte Progenitor Cell/Oligodendrocyte
ENSG00000127252	HRASLS	c17	Oligodendrocyte Progenitor Cell/Oligodendrocyte
ENSG00000131018	SYNE1	c17	Oligodendrocyte Progenitor Cell/Oligodendrocyte
ENSG00000131773	KHDRBS3	c17	Oligodendrocyte Progenitor Cell/Oligodendrocyte
ENSG00000132692	BCAN	c17	Oligodendrocyte Progenitor Cell/Oligodendrocyte
ENSG00000133019	CHRM3	c17	Oligodendrocyte Progenitor Cell/Oligodendrocyte
ENSG00000136997	MYC	c17	Oligodendrocyte Progenitor Cell/Oligodendrocyte
ENSG00000139318	DUSP6	c17	Oligodendrocyte Progenitor Cell/Oligodendrocyte
ENSG00000139915	MDGA2	c17	Oligodendrocyte Progenitor Cell/Oligodendrocyte
ENSG00000144339	TMEFF2	c17	Oligodendrocyte Progenitor Cell/Oligodendrocyte
ENSG00000146592	CREB5	c17	Oligodendrocyte Progenitor Cell/Oligodendrocyte
ENSG00000146938	NLGN4X	c17	Oligodendrocyte Progenitor Cell/Oligodendrocyte
ENSG00000147481	SNTG1	c17	Oligodendrocyte Progenitor Cell/Oligodendrocyte
ENSG00000148123	PLPPR1	c17	Oligodendrocyte Progenitor Cell/Oligodendrocyte
ENSG00000151729	SLC25A4	c17	Oligodendrocyte Progenitor Cell/Oligodendrocyte
ENSG00000152214	RIT2	c17	Oligodendrocyte Progenitor Cell/Oligodendrocyte
ENSG00000154096	THY1	c17	Oligodendrocyte Progenitor Cell/Oligodendrocyte
ENSG00000154654	NCAM2	c17	Oligodendrocyte Progenitor Cell/Oligodendrocyte
ENSG00000154975	CA10	c17	Oligodendrocyte Progenitor Cell/Oligodendrocyte
ENSG00000159200	RCAN1	c17	Oligodendrocyte Progenitor Cell/Oligodendrocyte
ENSG00000162670	BRINP3	c17	Oligodendrocyte Progenitor Cell/Oligodendrocyte
ENSG00000164434	FABP7	c17	Oligodendrocyte Progenitor Cell/Oligodendrocyte
ENSG00000164649	CDCA7L	c17	Oligodendrocyte Progenitor Cell/Oligodendrocyte
ENSG00000164929	BAALC	c17	Oligodendrocyte Progenitor Cell/Oligodendrocyte
ENSG00000168461	RAB31	c17	Oligodendrocyte Progenitor Cell/Oligodendrocyte
ENSG00000168959	GRM5	c17	Oligodendrocyte Progenitor Cell/Oligodendrocyte
ENSG00000169851	PCDH7	c17	Oligodendrocyte Progenitor Cell/Oligodendrocyte
ENSG00000171385	KCND3	c17	Oligodendrocyte Progenitor Cell/Oligodendrocyte
ENSG00000171587	DSCAM	c17	Oligodendrocyte Progenitor Cell/Oligodendrocyte
ENSG00000173511	VEGFB	c17	Oligodendrocyte Progenitor Cell/Oligodendrocyte
ENSG00000181790	ADGRB1	c17	Oligodendrocyte Progenitor Cell/Oligodendrocyte
ENSG00000182103	FAM181B	c17	Oligodendrocyte Progenitor Cell/Oligodendrocyte
ENSG00000183715	OPCML	c17	Oligodendrocyte Progenitor Cell/Oligodendrocyte
ENSG00000187135	VSTM2B	c17	Oligodendrocyte Progenitor Cell/Oligodendrocyte
ENSG00000187479	C11orf96	c17	Oligodendrocyte Progenitor Cell/Oligodendrocyte
ENSG00000197177	ADGRA1	c17	Oligodendrocyte Progenitor Cell/Oligodendrocyte
ENSG00000197461	PDGFA	c17	Oligodendrocyte Progenitor Cell/Oligodendrocyte
ENSG00000197956	S100A6	c17	Oligodendrocyte Progenitor Cell/Oligodendrocyte
ENSG00000197971	MBP	c17	Oligodendrocyte Progenitor Cell/Oligodendrocyte
ENSG00000198739	LRRTM3	c17	Oligodendrocyte Progenitor Cell/Oligodendrocyte

ENSG00000204128	C2orf72	c17	Oligodendrocyte Progenitor Cell/Oligodendrocyte
ENSG00000215612	HMX1	c17	Oligodendrocyte Progenitor Cell/Oligodendrocyte
ENSG00000237515	SHISA9	c17	Oligodendrocyte Progenitor Cell/Oligodendrocyte
ENSG00000256463	SALL3	c17	Oligodendrocyte Progenitor Cell/Oligodendrocyte
ENSG00000260807	AC009041.2	c17	Oligodendrocyte Progenitor Cell/Oligodendrocyte
ENSG00000263146	LINC01896	c17	Oligodendrocyte Progenitor Cell/Oligodendrocyte
ENSG00000007372	PAX6	c18	Truncated Radial Glia
ENSG00000075426	FOSL2	c18	Truncated Radial Glia
ENSG00000078114	NEBL	c18	Truncated Radial Glia
ENSG00000101134	DOK5	c18	Truncated Radial Glia
ENSG00000105877	DNAH11	c18	Truncated Radial Glia
ENSG00000108551	RASD1	c18	Truncated Radial Glia
ENSG00000114315	HES1	c18	Truncated Radial Glia
ENSG00000115461	IGFBP5	c18	Truncated Radial Glia
ENSG00000115468	EFHD1	c18	Truncated Radial Glia
ENSG00000116194	ANGPTL1	c18	Truncated Radial Glia
ENSG00000119408	NEK6	c18	Truncated Radial Glia
ENSG00000120738	EGR1	c18	Truncated Radial Glia
ENSG00000130643	CALY	c18	Truncated Radial Glia
ENSG00000131018	SYNE1	c18	Truncated Radial Glia
ENSG00000132692	BCAN	c18	Truncated Radial Glia
ENSG00000135363	LMO2	c18	Truncated Radial Glia
ENSG00000139263	LRIG3	c18	Truncated Radial Glia
ENSG00000146592	CREB5	c18	Truncated Radial Glia
ENSG00000156804	FBXO32	c18	Truncated Radial Glia
ENSG00000159176	CSRP1	c18	Truncated Radial Glia
ENSG00000162692	VCAM1	c18	Truncated Radial Glia
ENSG00000164171	ITGA2	c18	Truncated Radial Glia
ENSG00000177283	FZD8	c18	Truncated Radial Glia
ENSG00000177807	KCNJ10	c18	Truncated Radial Glia
ENSG00000183690	EFHC2	c18	Truncated Radial Glia
ENSG00000188153	COL4A5	c18	Truncated Radial Glia
ENSG00000197747	S100A10	c18	Truncated Radial Glia
ENSG00000204128	C2orf72	c18	Truncated Radial Glia
ENSG00000213694	S1PR3	c18	Truncated Radial Glia
ENSG00000215845	TSTD1	c18	Truncated Radial Glia
ENSG00000250722	SELENOP	c18	Truncated Radial Glia
ENSG00000256463	SALL3	c18	Truncated Radial Glia
ENSG00000263146	LINC01896	c18	Truncated Radial Glia
ENSG00000004399	PLXND1	c19	Pericyte
ENSG00000061337	LZTS1	c19	Pericyte
ENSG00000065054	SLC9A3R2	c19	Pericyte
ENSG00000068831	RASGRP2	c19	Pericyte
ENSG00000074416	MGLL	c19	Pericyte
ENSG00000075426	FOSL2	c19	Pericyte
ENSG00000101460	MAP1LC3A	c19	Pericyte
ENSG00000101825	MXRA5	c19	Pericyte
ENSG00000104368	PLAT	c19	Pericyte
ENSG00000111341	MGP	c19	Pericyte
ENSG00000111452	ADGRD1	c19	Pericyte
ENSG00000112902	SEMA5A	c19	Pericyte
ENSG00000113070	HBEGF	c19	Pericyte
ENSG00000113361	CDH6	c19	Pericyte
ENSG00000114115	RBP1	c19	Pericyte
ENSG00000114315	HES1	c19	Pericyte
ENSG00000116132	PRRX1	c19	Pericyte
ENSG00000120708	TGFBI	c19	Pericyte
ENSG00000122691	TWIST1	c19	Pericyte
ENSG00000125895	TMEM74B	c19	Pericyte
ENSG00000126785	RHOJ	c19	Pericyte
ENSG00000133401	PDZD2	c19	Pericyte
ENSG00000135363	LMO2	c19	Pericyte
ENSG00000136997	MYC	c19	Pericyte
ENSG00000138166	DUSP5	c19	Pericyte
ENSG00000152402	GUCY1A2	c19	Pericyte
ENSG00000154096	THY1	c19	Pericyte
ENSG00000155846	PPARGC1B	c19	Pericyte
ENSG00000157514	TSC22D3	c19	Pericyte
ENSG00000158747	NBL1	c19	Pericyte
ENSG00000159176	CSRP1	c19	Pericyte
ENSG00000163638	ADAMTS9	c19	Pericyte
ENSG00000164116	GUCY1A3	c19	Pericyte
ENSG00000165169	DYNLT3	c19	Pericyte
ENSG00000166501	PRKCB	c19	Pericyte
ENSG00000168461	RAB31	c19	Pericyte
ENSG00000172889	EGFL7	c19	Pericyte
ENSG00000175040	CHST2	c19	Pericyte

ENSG00000179431	FJX1	c19	Pericyte
ENSG00000180287	PLD5	c19	Pericyte
ENSG00000183570	PCBP3	c19	Pericyte
ENSG00000187479	C11orf96	c19	Pericyte
ENSG00000196660	SLC30A10	c19	Pericyte
ENSG00000197747	S100A10	c19	Pericyte
ENSG00000213694	S1PR3	c19	Pericyte
ENSG00000250722	SELENOP	c19	Pericyte
ENSG00000267280	TBX2-AS1	c19	Pericyte
ENSG00000065717	TLE2	c1	CGE Interneuron
ENSG00000077279	DCX	c1	CGE Interneuron
ENSG00000078725	BRINP1	c1	CGE Interneuron
ENSG00000101489	CELF4	c1	CGE Interneuron
ENSG00000101638	ST8SIA5	c1	CGE Interneuron
ENSG00000103449	SALL1	c1	CGE Interneuron
ENSG00000103528	SYT17	c1	CGE Interneuron
ENSG00000114115	RBP1	c1	CGE Interneuron
ENSG00000114279	FGF12	c1	CGE Interneuron
ENSG00000120162	MOB3B	c1	CGE Interneuron
ENSG00000127084	FGD3	c1	CGE Interneuron
ENSG00000127152	BCL11B	c1	CGE Interneuron
ENSG00000144339	TMEFF2	c1	CGE Interneuron
ENSG00000144596	GRIP2	c1	CGE Interneuron
ENSG00000145920	CPLX2	c1	CGE Interneuron
ENSG00000152377	SPOCK1	c1	CGE Interneuron
ENSG00000156395	SORCS3	c1	CGE Interneuron
ENSG00000157514	TSC22D3	c1	CGE Interneuron
ENSG00000162374	ELAVL4	c1	CGE Interneuron
ENSG00000162545	CAMK2N1	c1	CGE Interneuron
ENSG00000162989	KCNJ3	c1	CGE Interneuron
ENSG00000163630	SYNPR	c1	CGE Interneuron
ENSG00000172137	CALB2	c1	CGE Interneuron
ENSG00000174460	ZCCHC12	c1	CGE Interneuron
ENSG00000175906	ARL4D	c1	CGE Interneuron
ENSG00000177103	DSCAML1	c1	CGE Interneuron
ENSG00000177181	RIMKLA	c1	CGE Interneuron
ENSG00000179981	TSHZ1	c1	CGE Interneuron
ENSG00000186310	NAP1L3	c1	CGE Interneuron
ENSG00000187164	SHTN1	c1	CGE Interneuron
ENSG00000187479	C11orf96	c1	CGE Interneuron
ENSG00000204103	MAFB	c1	CGE Interneuron
ENSG00000217236	SP9	c1	CGE Interneuron
ENSG00000260664	AC004158.1	c1	CGE Interneuron
ENSG00000004399	PLXND1	c20	Endothelial Cell
ENSG00000065054	SLC9A3R2	c20	Endothelial Cell
ENSG00000074416	MGLL	c20	Endothelial Cell
ENSG00000075426	FOSL2	c20	Endothelial Cell
ENSG00000085563	ABCB1	c20	Endothelial Cell
ENSG00000092758	COL9A3	c20	Endothelial Cell
ENSG00000099260	PALMD	c20	Endothelial Cell
ENSG00000100311	PDGFB	c20	Endothelial Cell
ENSG00000101347	SAMHD1	c20	Endothelial Cell
ENSG00000104368	PLAT	c20	Endothelial Cell
ENSG00000107738	VSIR	c20	Endothelial Cell
ENSG00000107742	SPOCK2	c20	Endothelial Cell
ENSG00000112561	TFEB	c20	Endothelial Cell
ENSG00000112902	SEMA5A	c20	Endothelial Cell
ENSG00000113070	HBEGF	c20	Endothelial Cell
ENSG00000113361	CDH6	c20	Endothelial Cell
ENSG00000114115	RBP1	c20	Endothelial Cell
ENSG00000114315	HES1	c20	Endothelial Cell
ENSG00000118257	NRP2	c20	Endothelial Cell
ENSG00000120156	TEK	c20	Endothelial Cell
ENSG00000122691	TWIST1	c20	Endothelial Cell
ENSG00000124772	CPNE5	c20	Endothelial Cell
ENSG00000126217	MCF2L	c20	Endothelial Cell
ENSG00000126785	RHOJ	c20	Endothelial Cell
ENSG00000130813	C19orf66	c20	Endothelial Cell
ENSG00000131477	RAMP2	c20	Endothelial Cell
ENSG00000131979	GCH1	c20	Endothelial Cell
ENSG00000132561	MATN2	c20	Endothelial Cell
ENSG00000133401	PDZD2	c20	Endothelial Cell
ENSG00000135063	FAM189A2	c20	Endothelial Cell
ENSG00000135363	LMO2	c20	Endothelial Cell
ENSG00000137959	IFI44L	c20	Endothelial Cell
ENSG00000138166	DUSP5	c20	Endothelial Cell
ENSG00000138496	PARP9	c20	Endothelial Cell

ENSG00000139318	DUSP6	c20	Endothelial Cell
ENSG00000140280	LYSMD2	c20	Endothelial Cell
ENSG00000141524	TMC6	c20	Endothelial Cell
ENSG00000146674	IGFBP3	c20	Endothelial Cell
ENSG00000148488	ST8SIA6	c20	Endothelial Cell
ENSG00000155846	PPARGC1B	c20	Endothelial Cell
ENSG00000157514	TSC22D3	c20	Endothelial Cell
ENSG00000157601	MX1	c20	Endothelial Cell
ENSG00000158747	NBL1	c20	Endothelial Cell
ENSG00000159176	CSRP1	c20	Endothelial Cell
ENSG00000159200	RCAN1	c20	Endothelial Cell
ENSG00000159640	ACE	c20	Endothelial Cell
ENSG00000160932	LY6E	c20	Endothelial Cell
ENSG00000162981	FAM84A	c20	Endothelial Cell
ENSG00000163638	ADAMTS9	c20	Endothelial Cell
ENSG00000164120	HPGD	c20	Endothelial Cell
ENSG00000164683	HEY1	c20	Endothelial Cell
ENSG00000164930	FZD6	c20	Endothelial Cell
ENSG00000165810	BTNL9	c20	Endothelial Cell
ENSG00000171388	APLN	c20	Endothelial Cell
ENSG00000172889	EGFL7	c20	Endothelial Cell
ENSG00000174640	SLCO2A1	c20	Endothelial Cell
ENSG00000175040	CHST2	c20	Endothelial Cell
ENSG00000178695	KCTD12	c20	Endothelial Cell
ENSG00000188338	SLC38A3	c20	Endothelial Cell
ENSG00000197747	S100A10	c20	Endothelial Cell
ENSG00000197859	ADAMTSL2	c20	Endothelial Cell
ENSG00000250722	SELENOP	c20	Endothelial Cell
ENSG00000157005	SST	c21	Red Blood Cell
ENSG00000163032	VSNL1	c21	Red Blood Cell
ENSG00000065054	SLC9A3R2	c22	Vascular and Leptomeningeal Cell
ENSG00000075426	FOSL2	c22	Vascular and Leptomeningeal Cell
ENSG00000084636	COL16A1	c22	Vascular and Leptomeningeal Cell
ENSG00000104368	PLAT	c22	Vascular and Leptomeningeal Cell
ENSG00000111341	MGP	c22	Vascular and Leptomeningeal Cell
ENSG00000112902	SEMA5A	c22	Vascular and Leptomeningeal Cell
ENSG00000114115	RBP1	c22	Vascular and Leptomeningeal Cell
ENSG00000114315	HES1	c22	Vascular and Leptomeningeal Cell
ENSG00000115380	EFEMP1	c22	Vascular and Leptomeningeal Cell
ENSG00000115461	IGFBP5	c22	Vascular and Leptomeningeal Cell
ENSG00000116132	PRRX1	c22	Vascular and Leptomeningeal Cell
ENSG00000120738	EGR1	c22	Vascular and Leptomeningeal Cell
ENSG00000121310	ECHDC2	c22	Vascular and Leptomeningeal Cell
ENSG00000122691	TWIST1	c22	Vascular and Leptomeningeal Cell
ENSG00000125845	BMP2	c22	Vascular and Leptomeningeal Cell
ENSG00000126218	F10	c22	Vascular and Leptomeningeal Cell
ENSG00000126785	RHOJ	c22	Vascular and Leptomeningeal Cell
ENSG00000130707	ASS1	c22	Vascular and Leptomeningeal Cell
ENSG00000131477	RAMP2	c22	Vascular and Leptomeningeal Cell
ENSG00000131771	PPP1R1B	c22	Vascular and Leptomeningeal Cell
ENSG00000133110	POSTN	c22	Vascular and Leptomeningeal Cell
ENSG00000135333	EPHA7	c22	Vascular and Leptomeningeal Cell
ENSG00000136997	MYC	c22	Vascular and Leptomeningeal Cell
ENSG00000136999	NOV	c22	Vascular and Leptomeningeal Cell
ENSG00000137727	ARHGAP20	c22	Vascular and Leptomeningeal Cell
ENSG00000138166	DUSP5	c22	Vascular and Leptomeningeal Cell
ENSG00000149633	KIAA1755	c22	Vascular and Leptomeningeal Cell
ENSG00000155254	MARVELD1	c22	Vascular and Leptomeningeal Cell
ENSG00000157514	TSC22D3	c22	Vascular and Leptomeningeal Cell
ENSG00000158747	NBL1	c22	Vascular and Leptomeningeal Cell
ENSG00000160932	LY6E	c22	Vascular and Leptomeningeal Cell
ENSG00000162552	WNT4	c22	Vascular and Leptomeningeal Cell
ENSG00000162692	VCAM1	c22	Vascular and Leptomeningeal Cell
ENSG00000163359	COL6A3	c22	Vascular and Leptomeningeal Cell
ENSG00000163638	ADAMTS9	c22	Vascular and Leptomeningeal Cell
ENSG00000164683	HEY1	c22	Vascular and Leptomeningeal Cell
ENSG00000165169	DYNLT3	c22	Vascular and Leptomeningeal Cell
ENSG00000166106	ADAMTS15	c22	Vascular and Leptomeningeal Cell
ENSG00000169891	REPS2	c22	Vascular and Leptomeningeal Cell
ENSG00000172889	EGFL7	c22	Vascular and Leptomeningeal Cell
ENSG00000178695	KCTD12	c22	Vascular and Leptomeningeal Cell
ENSG00000181234	TMEM132C	c22	Vascular and Leptomeningeal Cell
ENSG00000183098	GPC6	c22	Vascular and Leptomeningeal Cell
ENSG00000188153	COL4A5	c22	Vascular and Leptomeningeal Cell
ENSG00000188176	SMTNL2	c22	Vascular and Leptomeningeal Cell
ENSG00000188338	SLC38A3	c22	Vascular and Leptomeningeal Cell
ENSG00000197565	COL4A6	c22	Vascular and Leptomeningeal Cell

ENSG00000197747	S100A10	c22	Vascular and Leptomeningeal Cell
ENSG00000197956	S100A6	c22	Vascular and Leptomeningeal Cell
ENSG00000198121	LPAR1	c22	Vascular and Leptomeningeal Cell
ENSG00000204103	MAFB	c22	Vascular and Leptomeningeal Cell
ENSG00000204291	COL15A1	c22	Vascular and Leptomeningeal Cell
ENSG00000250722	SELENOP	c22	Vascular and Leptomeningeal Cell
ENSG00000002587	HS3ST1	c2	Glutamatergic Neuron 1
ENSG00000074317	SNCB	c2	Glutamatergic Neuron 1
ENSG00000106341	PPP1R17	c2	Glutamatergic Neuron 1
ENSG00000107338	SHB	c2	Glutamatergic Neuron 1
ENSG00000108551	RASD1	c2	Glutamatergic Neuron 1
ENSG00000113763	UNC5A	c2	Glutamatergic Neuron 1
ENSG00000125266	EFNB2	c2	Glutamatergic Neuron 1
ENSG00000127152	BCL11B	c2	Glutamatergic Neuron 1
ENSG00000135333	EPHA7	c2	Glutamatergic Neuron 1
ENSG00000145247	OCIAD2	c2	Glutamatergic Neuron 1
ENSG00000147862	NFIB	c2	Glutamatergic Neuron 1
ENSG00000150594	ADRA2A	c2	Glutamatergic Neuron 1
ENSG00000154429	CCSAP	c2	Glutamatergic Neuron 1
ENSG00000155926	SLA	c2	Glutamatergic Neuron 1
ENSG00000163673	DCLK3	c2	Glutamatergic Neuron 1
ENSG00000167281	RBFOX3	c2	Glutamatergic Neuron 1
ENSG00000171246	NPTX1	c2	Glutamatergic Neuron 1
ENSG00000171532	NEUROD2	c2	Glutamatergic Neuron 1
ENSG00000174469	CNTNAP2	c2	Glutamatergic Neuron 1
ENSG00000180616	SSTR2	c2	Glutamatergic Neuron 1
ENSG00000181195	PENK	c2	Glutamatergic Neuron 1
ENSG00000185008	ROBO2	c2	Glutamatergic Neuron 1
ENSG00000214595	EML6	c2	Glutamatergic Neuron 1
ENSG00000006128	TAC1	c3	MGE Interneuron
ENSG00000009694	TENM1	c3	MGE Interneuron
ENSG00000066382	MPPED2	c3	MGE Interneuron
ENSG00000077279	DCX	c3	MGE Interneuron
ENSG00000089169	RPH3A	c3	MGE Interneuron
ENSG00000101489	CELF4	c3	MGE Interneuron
ENSG00000101638	ST8SIA5	c3	MGE Interneuron
ENSG00000102290	PCDH11X	c3	MGE Interneuron
ENSG00000114115	RBP1	c3	MGE Interneuron
ENSG00000120162	MOB3B	c3	MGE Interneuron
ENSG00000120875	DUSP4	c3	MGE Interneuron
ENSG00000122584	NXPH1	c3	MGE Interneuron
ENSG00000124191	TOX2	c3	MGE Interneuron
ENSG00000127084	FGD3	c3	MGE Interneuron
ENSG00000127152	BCL11B	c3	MGE Interneuron
ENSG00000134207	SYT6	c3	MGE Interneuron
ENSG00000137965	IFI44	c3	MGE Interneuron
ENSG00000144460	NYAP2	c3	MGE Interneuron
ENSG00000144596	GRIP2	c3	MGE Interneuron
ENSG00000147481	SNTG1	c3	MGE Interneuron
ENSG00000151834	GABRA2	c3	MGE Interneuron
ENSG00000154096	THY1	c3	MGE Interneuron
ENSG00000154736	ADAMTS5	c3	MGE Interneuron
ENSG00000155511	GRIA1	c3	MGE Interneuron
ENSG00000157005	SST	c3	MGE Interneuron
ENSG00000157514	TSC22D3	c3	MGE Interneuron
ENSG00000162975	KCNF1	c3	MGE Interneuron
ENSG00000166006	KCNC2	c3	MGE Interneuron
ENSG00000174469	CNTNAP2	c3	MGE Interneuron
ENSG00000175906	ARL4D	c3	MGE Interneuron
ENSG00000177103	DSCAML1	c3	MGE Interneuron
ENSG00000178695	KCTD12	c3	MGE Interneuron
ENSG00000178718	RPP25	c3	MGE Interneuron
ENSG00000179431	FJX1	c3	MGE Interneuron
ENSG00000179542	SLITRK4	c3	MGE Interneuron
ENSG00000179841	AKAP5	c3	MGE Interneuron
ENSG00000187164	SHTN1	c3	MGE Interneuron
ENSG00000187479	C11orf96	c3	MGE Interneuron
ENSG00000188848	BEND4	c3	MGE Interneuron
ENSG00000196090	PTPRT	c3	MGE Interneuron
ENSG00000197971	MBP	c3	MGE Interneuron
ENSG00000198797	BRINP2	c3	MGE Interneuron
ENSG00000204103	MAFB	c3	MGE Interneuron
ENSG00000217236	SP9	c3	MGE Interneuron
ENSG00000260664	AC004158.1	c3	MGE Interneuron
ENSG00000002587	HS3ST1	c4	Glutamatergic Neuron 5
ENSG00000008441	NFIX	c4	Glutamatergic Neuron 5
ENSG00000075223	SEMA3C	c4	Glutamatergic Neuron 5

ENSG00000092758	COL9A3	c4	Glutamatergic Neuron 5
ENSG00000099260	PALMD	c4	Glutamatergic Neuron 5
ENSG00000101460	MAP1LC3A	c4	Glutamatergic Neuron 5
ENSG00000104888	SLC17A7	c4	Glutamatergic Neuron 5
ENSG00000106689	LHX2	c4	Glutamatergic Neuron 5
ENSG00000108551	RASD1	c4	Glutamatergic Neuron 5
ENSG00000134138	MEIS2	c4	Glutamatergic Neuron 5
ENSG00000145335	SNCA	c4	Glutamatergic Neuron 5
ENSG00000147862	NFIB	c4	Glutamatergic Neuron 5
ENSG00000150594	ADRA2A	c4	Glutamatergic Neuron 5
ENSG00000155926	SLA	c4	Glutamatergic Neuron 5
ENSG00000162545	CAMK2N1	c4	Glutamatergic Neuron 5
ENSG00000166250	CLMP	c4	Glutamatergic Neuron 5
ENSG00000171532	NEUROD2	c4	Glutamatergic Neuron 5
ENSG00000221866	PLXNA4	c4	Glutamatergic Neuron 5
ENSG00000058404	CAMK2B	c5	Glutamatergic Neuron 3
ENSG00000074317	SNCB	c5	Glutamatergic Neuron 3
ENSG00000075213	SEMA3A	c5	Glutamatergic Neuron 3
ENSG00000101134	DOK5	c5	Glutamatergic Neuron 3
ENSG00000104833	TUBB4A	c5	Glutamatergic Neuron 3
ENSG00000107338	SHB	c5	Glutamatergic Neuron 3
ENSG00000112379	ARFGEF3	c5	Glutamatergic Neuron 3
ENSG00000145247	OCIAD2	c5	Glutamatergic Neuron 3
ENSG00000145335	SNCA	c5	Glutamatergic Neuron 3
ENSG00000147862	NFIB	c5	Glutamatergic Neuron 3
ENSG00000150594	ADRA2A	c5	Glutamatergic Neuron 3
ENSG00000152402	GUCY1A2	c5	Glutamatergic Neuron 3
ENSG00000155926	SLA	c5	Glutamatergic Neuron 3
ENSG00000160932	LY6E	c5	Glutamatergic Neuron 3
ENSG00000163673	DCLK3	c5	Glutamatergic Neuron 3
ENSG00000164604	GPR85	c5	Glutamatergic Neuron 3
ENSG00000171532	NEUROD2	c5	Glutamatergic Neuron 3
ENSG00000172995	ARPP21	c5	Glutamatergic Neuron 3
ENSG00000176956	LY6H	c5	Glutamatergic Neuron 3
ENSG00000186868	MAPT	c5	Glutamatergic Neuron 3
ENSG00000187164	SHTN1	c5	Glutamatergic Neuron 3
ENSG00000188580	NKAIN2	c5	Glutamatergic Neuron 3
ENSG00000221866	PLXNA4	c5	Glutamatergic Neuron 3
ENSG00000006468	ETV1	c6	Early Radial Glia
ENSG00000007372	PAX6	c6	Early Radial Glia
ENSG00000041982	TNC	c6	Early Radial Glia
ENSG00000042493	CAPG	c6	Early Radial Glia
ENSG00000091490	SEL1L3	c6	Early Radial Glia
ENSG00000101134	DOK5	c6	Early Radial Glia
ENSG00000103449	SALL1	c6	Early Radial Glia
ENSG00000104332	SFRP1	c6	Early Radial Glia
ENSG00000104369	JPH1	c6	Early Radial Glia
ENSG00000106689	LHX2	c6	Early Radial Glia
ENSG00000112902	SEMA5A	c6	Early Radial Glia
ENSG00000114315	HES1	c6	Early Radial Glia
ENSG00000120162	MOB3B	c6	Early Radial Glia
ENSG00000120738	EGR1	c6	Early Radial Glia
ENSG00000121310	ECHDC2	c6	Early Radial Glia
ENSG00000125266	EFNB2	c6	Early Radial Glia
ENSG00000126785	RHOJ	c6	Early Radial Glia
ENSG00000132692	BCAN	c6	Early Radial Glia
ENSG00000137959	IFI44L	c6	Early Radial Glia
ENSG00000146592	CREB5	c6	Early Radial Glia
ENSG00000149948	HMGA2	c6	Early Radial Glia
ENSG00000156804	FBXO32	c6	Early Radial Glia
ENSG00000158234	FAIM	c6	Early Radial Glia
ENSG00000164171	ITGA2	c6	Early Radial Glia
ENSG00000164434	FABP7	c6	Early Radial Glia
ENSG00000164649	CDCA7L	c6	Early Radial Glia
ENSG00000164683	HEY1	c6	Early Radial Glia
ENSG00000168309	FAM107A	c6	Early Radial Glia
ENSG00000168461	RAB31	c6	Early Radial Glia
ENSG00000170370	EMX2	c6	Early Radial Glia
ENSG00000173511	VEGFB	c6	Early Radial Glia
ENSG00000177133	LINC00982	c6	Early Radial Glia
ENSG00000177283	FZD8	c6	Early Radial Glia
ENSG00000182103	FAM181B	c6	Early Radial Glia
ENSG00000188153	COL4A5	c6	Early Radial Glia
ENSG00000197565	COL4A6	c6	Early Radial Glia
ENSG00000197956	S100A6	c6	Early Radial Glia
ENSG00000198739	LRRTM3	c6	Early Radial Glia
ENSG00000204128	C2orf72	c6	Early Radial Glia

ENSG00000229847	EMX2OS	c6	Early Radial Glia
ENSG00000250722	SELENOP	c6	Early Radial Glia
ENSG00000256463	SALL3	c6	Early Radial Glia
ENSG00000263146	LINC01896	c6	Early Radial Glia
ENSG00000007237	GAS7	c7	Glutamatergic Neuron 7
ENSG00000018236	CNTN1	c7	Glutamatergic Neuron 7
ENSG00000040731	CDH10	c7	Glutamatergic Neuron 7
ENSG00000058404	CAMK2B	c7	Glutamatergic Neuron 7
ENSG00000060709	RIMBP2	c7	Glutamatergic Neuron 7
ENSG00000075213	SEMA3A	c7	Glutamatergic Neuron 7
ENSG00000075223	SEMA3C	c7	Glutamatergic Neuron 7
ENSG00000101134	DOK5	c7	Glutamatergic Neuron 7
ENSG00000101489	CELF4	c7	Glutamatergic Neuron 7
ENSG00000102290	PCDH11X	c7	Glutamatergic Neuron 7
ENSG00000106689	LHX2	c7	Glutamatergic Neuron 7
ENSG00000110786	PTPN5	c7	Glutamatergic Neuron 7
ENSG00000112379	ARFGEF3	c7	Glutamatergic Neuron 7
ENSG00000114279	FGF12	c7	Glutamatergic Neuron 7
ENSG00000116254	CHD5	c7	Glutamatergic Neuron 7
ENSG00000117598	PLPPR5	c7	Glutamatergic Neuron 7
ENSG00000117600	PLPPR4	c7	Glutamatergic Neuron 7
ENSG00000119771	KLHL29	c7	Glutamatergic Neuron 7
ENSG00000125266	EFNB2	c7	Glutamatergic Neuron 7
ENSG00000125851	PCSK2	c7	Glutamatergic Neuron 7
ENSG00000128045	RASL11B	c7	Glutamatergic Neuron 7
ENSG00000130643	CALY	c7	Glutamatergic Neuron 7
ENSG00000132932	ATP8A2	c7	Glutamatergic Neuron 7
ENSG00000135333	EPHA7	c7	Glutamatergic Neuron 7
ENSG00000136531	SCN2A	c7	Glutamatergic Neuron 7
ENSG00000136928	GABBR2	c7	Glutamatergic Neuron 7
ENSG00000137261	KIAA0319	c7	Glutamatergic Neuron 7
ENSG00000137727	ARHGAP20	c7	Glutamatergic Neuron 7
ENSG00000142408	CACNG8	c7	Glutamatergic Neuron 7
ENSG00000142875	PRKACB	c7	Glutamatergic Neuron 7
ENSG00000144339	TMEFF2	c7	Glutamatergic Neuron 7
ENSG00000144460	NYAP2	c7	Glutamatergic Neuron 7
ENSG00000147481	SNTG1	c7	Glutamatergic Neuron 7
ENSG00000151490	PTPRO	c7	Glutamatergic Neuron 7
ENSG00000151834	GABRA2	c7	Glutamatergic Neuron 7
ENSG00000152377	SPOCK1	c7	Glutamatergic Neuron 7
ENSG00000152402	GUCY1A2	c7	Glutamatergic Neuron 7
ENSG00000155511	GRIA1	c7	Glutamatergic Neuron 7
ENSG00000157087	ATP2B2	c7	Glutamatergic Neuron 7
ENSG00000158560	DYNC1I1	c7	Glutamatergic Neuron 7
ENSG00000159307	SCUBE1	c7	Glutamatergic Neuron 7
ENSG00000163536	SERPINI1	c7	Glutamatergic Neuron 7
ENSG00000164076	CAMKV	c7	Glutamatergic Neuron 7
ENSG00000164418	GRIK2	c7	Glutamatergic Neuron 7
ENSG00000164604	GPR85	c7	Glutamatergic Neuron 7
ENSG00000167178	ISLR2	c7	Glutamatergic Neuron 7
ENSG00000169933	FRMPD4	c7	Glutamatergic Neuron 7
ENSG00000170921	TANC2	c7	Glutamatergic Neuron 7
ENSG00000172995	ARPP21	c7	Glutamatergic Neuron 7
ENSG00000174521	TTC9B	c7	Glutamatergic Neuron 7
ENSG00000176956	LY6H	c7	Glutamatergic Neuron 7
ENSG00000179431	FJX1	c7	Glutamatergic Neuron 7
ENSG00000186732	MPPED1	c7	Glutamatergic Neuron 7
ENSG00000186868	MAPT	c7	Glutamatergic Neuron 7
ENSG00000187135	VSTM2B	c7	Glutamatergic Neuron 7
ENSG00000187164	SHTN1	c7	Glutamatergic Neuron 7
ENSG00000188580	NKAIN2	c7	Glutamatergic Neuron 7
ENSG00000196090	PTPRT	c7	Glutamatergic Neuron 7
ENSG00000196277	GRM7	c7	Glutamatergic Neuron 7
ENSG00000197872	FAM49A	c7	Glutamatergic Neuron 7
ENSG00000198010	DLGAP2	c7	Glutamatergic Neuron 7
ENSG00000198216	CACNA1E	c7	Glutamatergic Neuron 7
ENSG00000198626	RYR2	c7	Glutamatergic Neuron 7
ENSG00000221866	PLXNA4	c7	Glutamatergic Neuron 7
ENSG00000225206	MIR137HG	c7	Glutamatergic Neuron 7
ENSG00000237515	SHISA9	c7	Glutamatergic Neuron 7
ENSG00000260230	FRRS1L	c7	Glutamatergic Neuron 7
ENSG00000273079	GRIN2B	c7	Glutamatergic Neuron 7
ENSG00000007372	PAX6	c8	Cycling Progenitor
ENSG00000042493	CAPG	c8	Cycling Progenitor
ENSG00000066382	MPPED2	c8	Cycling Progenitor
ENSG00000103449	SALL1	c8	Cycling Progenitor
ENSG00000104332	SFRP1	c8	Cycling Progenitor

ENSG00000106341	PPP1R17	c8	Cycling Progenitor
ENSG00000106689	LHX2	c8	Cycling Progenitor
ENSG00000114315	HES1	c8	Cycling Progenitor
ENSG00000120738	EGR1	c8	Cycling Progenitor
ENSG00000122574	WIPF3	c8	Cycling Progenitor
ENSG00000136104	RNASEH2B	c8	Cycling Progenitor
ENSG00000146592	CREB5	c8	Cycling Progenitor
ENSG00000164171	ITGA2	c8	Cycling Progenitor
ENSG00000164649	CDCA7L	c8	Cycling Progenitor
ENSG00000168309	FAM107A	c8	Cycling Progenitor
ENSG00000170370	EMX2	c8	Cycling Progenitor
ENSG00000177283	FZD8	c8	Cycling Progenitor
ENSG00000181195	PENK	c8	Cycling Progenitor
ENSG00000182103	FAM181B	c8	Cycling Progenitor
ENSG00000183098	GPC6	c8	Cycling Progenitor
ENSG00000256463	SALL3	c8	Cycling Progenitor
ENSG00000263146	LINC01896	c8	Cycling Progenitor
ENSG00000155926	SLA	c9	Glutamatergic Neuron 4
ENSG00000171532	NEUROD2	c9	Glutamatergic Neuron 4
c0_GluN5	25		
c10_Late_RG	65		
c11_mGPC	37		
c12_GluN6	37		
c13_SP	152		
c14_nIPC	18		
c15_GluN8	110		
c16_MG	33		
c17_OPC_Oligo	66		
c18_tRG	33		
c19_Peric	47		
c1_CGE_IN	34		
c20_EC	62		
c21_RBC	2		
c22_VLMC	53		
c2_GluN1	23		
c3_MGE_IN	45		
c4_GluN4	18		
c5_GluN2	23		
c6_Early_RG	43		
c7_GluN7	68		
c8_Cyc_Prog	22		
c9_GluN3	2		

Fig4G Up reg genes_Trevino

Intersection of Upregulated gene with cell type specific clusters in Trevino et. al. 2021

ENSEMBL_gene_ID	Gene_symbol	cluster	cluser_name
ENSG00000141540	TTYH2	c0	Glutamatergic Neuron 2
ENSG00000171208	NETO2	c0	Glutamatergic Neuron 2
ENSG00000174498	IGDCC3	c0	Glutamatergic Neuron 2
ENSG00000184486	POU3F2	c0	Glutamatergic Neuron 2
ENSG00000272168	CASC15	c0	Glutamatergic Neuron 2
ENSG00000010278	CD9	c10	Late Radial Glia
ENSG00000018625	ATP1A2	c10	Late Radial Glia
ENSG00000049323	LTBP1	c10	Late Radial Glia
ENSG00000083937	CHMP2B	c10	Late Radial Glia
ENSG00000100433	KCNK10	c10	Late Radial Glia
ENSG00000101198	NKAIN4	c10	Late Radial Glia
ENSG00000102760	RGCC	c10	Late Radial Glia
ENSG00000105855	ITGB8	c10	Late Radial Glia
ENSG00000106003	LFNG	c10	Late Radial Glia
ENSG00000110090	CPT1A	c10	Late Radial Glia
ENSG00000111783	RFX4	c10	Late Radial Glia
ENSG00000124942	AHNAK	c10	Late Radial Glia
ENSG00000132688	NES	c10	Late Radial Glia
ENSG00000136160	EDNRB	c10	Late Radial Glia
ENSG00000138696	BMPR1B	c10	Late Radial Glia
ENSG00000140511	HAPLN3	c10	Late Radial Glia
ENSG00000140545	MFGE8	c10	Late Radial Glia
ENSG00000143333	RGS16	c10	Late Radial Glia
ENSG00000144857	BOC	c10	Late Radial Glia
ENSG00000145794	MEGF10	c10	Late Radial Glia
ENSG00000147509	RGS20	c10	Late Radial Glia
ENSG00000148204	CRB2	c10	Late Radial Glia
ENSG00000154553	PDLIM3	c10	Late Radial Glia
ENSG00000162493	PDPN	c10	Late Radial Glia
ENSG00000162944	RFTN2	c10	Late Radial Glia
ENSG00000164199	ADGRV1	c10	Late Radial Glia
ENSG00000172340	SUCLG2	c10	Late Radial Glia
ENSG00000174021	GNG5	c10	Late Radial Glia
ENSG00000174721	FGFBP3	c10	Late Radial Glia
ENSG00000179104	TMTC2	c10	Late Radial Glia
ENSG00000185985	SLITRK2	c10	Late Radial Glia
ENSG00000187773	FAM69C	c10	Late Radial Glia
ENSG00000188290	HES4	c10	Late Radial Glia
ENSG00000189067	LITAF	c10	Late Radial Glia
ENSG00000197921	HES5	c10	Late Radial Glia
ENSG00000198435	NRARP	c10	Late Radial Glia
ENSG00000231064	AC234582.1	c10	Late Radial Glia
ENSG00000010278	CD9	c11	Oligodendrocyte intermediate progenitor cell
ENSG00000018408	WWTR1	c11	Oligodendrocyte intermediate progenitor cell
ENSG00000018625	ATP1A2	c11	Oligodendrocyte intermediate progenitor cell
ENSG00000068078	FGFR3	c11	Oligodendrocyte intermediate progenitor cell
ENSG00000090932	DLL3	c11	Oligodendrocyte intermediate progenitor cell
ENSG00000091986	CCDC80	c11	Oligodendrocyte intermediate progenitor cell
ENSG00000101198	NKAIN4	c11	Oligodendrocyte intermediate progenitor cell
ENSG00000101384	JAG1	c11	Oligodendrocyte intermediate progenitor cell
ENSG00000102760	RGCC	c11	Oligodendrocyte intermediate progenitor cell
ENSG00000105810	CDK6	c11	Oligodendrocyte intermediate progenitor cell
ENSG00000105855	ITGB8	c11	Oligodendrocyte intermediate progenitor cell
ENSG00000106003	LFNG	c11	Oligodendrocyte intermediate progenitor cell
ENSG00000106025	TSPAN12	c11	Oligodendrocyte intermediate progenitor cell
ENSG00000111783	RFX4	c11	Oligodendrocyte intermediate progenitor cell
ENSG00000117318	ID3	c11	Oligodendrocyte intermediate progenitor cell
ENSG00000122644	ARL4A	c11	Oligodendrocyte intermediate progenitor cell
ENSG00000126803	HSPA2	c11	Oligodendrocyte intermediate progenitor cell
ENSG00000130821	SLC6A8	c11	Oligodendrocyte intermediate progenitor cell
ENSG00000132688	NES	c11	Oligodendrocyte intermediate progenitor cell
ENSG00000134853	PDGFRA	c11	Oligodendrocyte intermediate progenitor cell
ENSG00000136160	EDNRB	c11	Oligodendrocyte intermediate progenitor cell
ENSG00000138696	BMPR1B	c11	Oligodendrocyte intermediate progenitor cell
ENSG00000139352	ASCL1	c11	Oligodendrocyte intermediate progenitor cell
ENSG00000140545	MFGE8	c11	Oligodendrocyte intermediate progenitor cell
ENSG00000143195	ILDR2	c11	Oligodendrocyte intermediate progenitor cell
ENSG00000143333	RGS16	c11	Oligodendrocyte intermediate progenitor cell
ENSG00000144857	BOC	c11	Oligodendrocyte intermediate progenitor cell
ENSG00000147509	RGS20	c11	Oligodendrocyte intermediate progenitor cell
ENSG00000148053	NTRK2	c11	Oligodendrocyte intermediate progenitor cell
ENSG00000154553	PDLIM3	c11	Oligodendrocyte intermediate progenitor cell
ENSG00000162493	PDPN	c11	Oligodendrocyte intermediate progenitor cell
ENSG00000162944	RFTN2	c11	Oligodendrocyte intermediate progenitor cell
ENSG00000172340	SUCLG2	c11	Oligodendrocyte intermediate progenitor cell

ENSG00000174021	GNG5	c11	Oligodendrocyte intermediate progenitor cell
ENSG00000176771	NCKAP5	c11	Oligodendrocyte intermediate progenitor cell
ENSG00000184384	MAML2	c11	Oligodendrocyte intermediate progenitor cell
ENSG00000185985	SLITRK2	c11	Oligodendrocyte intermediate progenitor cell
ENSG00000187773	FAM69C	c11	Oligodendrocyte intermediate progenitor cell
ENSG00000189067	LITAF	c11	Oligodendrocyte intermediate progenitor cell
ENSG00000197921	HES5	c11	Oligodendrocyte intermediate progenitor cell
ENSG00000198435	NRARP	c11	Oligodendrocyte intermediate progenitor cell
ENSG00000198719	DLL1	c11	Oligodendrocyte intermediate progenitor cell
ENSG00000171208	NETO2	c12	Glutamatergic Neuron 6
ENSG00000184486	POU3F2	c12	Glutamatergic Neuron 6
ENSG00000186907	RTN4RL2	c12	Glutamatergic Neuron 6
ENSG00000198729	PPP1R14C	c12	Glutamatergic Neuron 6
ENSG00000095596	CYP26A1	c13	Subplate
ENSG00000099139	PCSK5	c13	Subplate
ENSG00000148053	NTRK2	c13	Subplate
ENSG00000150361	KLHL1	c13	Subplate
ENSG00000175264	CHST1	c13	Subplate
ENSG00000176771	NCKAP5	c13	Subplate
ENSG00000179673	RPRML	c13	Subplate
ENSG00000188064	WNT7B	c13	Subplate
ENSG00000198221	AFDN-AS1	c13	Subplate
ENSG00000225783	MIAT	c13	Subplate
ENSG00000049323	LTBP1	c14	neuronal intermedate progenitor cell
ENSG00000090932	DLL3	c14	neuronal intermedate progenitor cell
ENSG00000101384	JAG1	c14	neuronal intermedate progenitor cell
ENSG00000105810	CDK6	c14	neuronal intermedate progenitor cell
ENSG00000123307	NEUROD4	c14	neuronal intermedate progenitor cell
ENSG00000139352	ASCL1	c14	neuronal intermedate progenitor cell
ENSG00000143333	RGS16	c14	neuronal intermedate progenitor cell
ENSG00000144485	HES6	c14	neuronal intermedate progenitor cell
ENSG00000144857	BOC	c14	neuronal intermedate progenitor cell
ENSG00000162944	RFTN2	c14	neuronal intermedate progenitor cell
ENSG00000164199	ADGRV1	c14	neuronal intermedate progenitor cell
ENSG00000174021	GNG5	c14	neuronal intermedate progenitor cell
ENSG00000197921	HES5	c14	neuronal intermedate progenitor cell
ENSG00000198719	DLL1	c14	neuronal intermedate progenitor cell
ENSG00000007062	PROM1	c15	Glutamatergic Neuron 8
ENSG00000136099	PCDH8	c15	Glutamatergic Neuron 8
ENSG00000148053	NTRK2	c15	Glutamatergic Neuron 8
ENSG00000151090	THRB	c15	Glutamatergic Neuron 8
ENSG00000198729	PPP1R14C	c15	Glutamatergic Neuron 8
ENSG00000088992	TESC	c16	Microglia
ENSG00000093072	ADA2	c16	Microglia
ENSG00000100292	HMOX1	c16	Microglia
ENSG00000101463	SYNDIG1	c16	Microglia
ENSG00000102760	RGCC	c16	Microglia
ENSG00000104894	CD37	c16	Microglia
ENSG00000118985	ELL2	c16	Microglia
ENSG00000121064	SCPEP1	c16	Microglia
ENSG00000124762	CDKN1A	c16	Microglia
ENSG00000136732	GYPC	c16	Microglia
ENSG00000136960	ENPP2	c16	Microglia
ENSG00000139679	LPAR6	c16	Microglia
ENSG00000162493	PDPN	c16	Microglia
ENSG00000162496	DHRS3	c16	Microglia
ENSG00000163219	ARHGAP25	c16	Microglia
ENSG00000167664	TMIGD2	c16	Microglia
ENSG00000170430	MGMT	c16	Microglia
ENSG00000172340	SUCLG2	c16	Microglia
ENSG00000174021	GNG5	c16	Microglia
ENSG00000177663	IL17RA	c16	Microglia
ENSG00000182118	FAM89A	c16	Microglia
ENSG00000189067	LITAF	c16	Microglia
ENSG00000198189	HSD17B11	c16	Microglia
ENSG00000203747	FCGR3A	c16	Microglia
ENSG00000007062	PROM1	c17	Oligodendrocyte Progenitor Cell/Oligodendrocyte
ENSG00000010278	CD9	c17	Oligodendrocyte Progenitor Cell/Oligodendrocyte
ENSG00000090932	DLL3	c17	Oligodendrocyte Progenitor Cell/Oligodendrocyte
ENSG00000101198	NKAIN4	c17	Oligodendrocyte Progenitor Cell/Oligodendrocyte
ENSG00000102760	RGCC	c17	Oligodendrocyte Progenitor Cell/Oligodendrocyte
ENSG00000105855	ITGB8	c17	Oligodendrocyte Progenitor Cell/Oligodendrocyte
ENSG00000122644	ARL4A	c17	Oligodendrocyte Progenitor Cell/Oligodendrocyte
ENSG00000132688	NES	c17	Oligodendrocyte Progenitor Cell/Oligodendrocyte
ENSG00000134853	PDGFRA	c17	Oligodendrocyte Progenitor Cell/Oligodendrocyte
ENSG00000136160	EDNRB	c17	Oligodendrocyte Progenitor Cell/Oligodendrocyte
ENSG00000139352	ASCL1	c17	Oligodendrocyte Progenitor Cell/Oligodendrocyte

ENSG00000143195	ILDR2	c17	Oligodendrocyte Progenitor Cell/Oligodendrocyte
ENSG00000162944	RFTN2	c17	Oligodendrocyte Progenitor Cell/Oligodendrocyte
ENSG00000163362	INAVA	c17	Oligodendrocyte Progenitor Cell/Oligodendrocyte
ENSG00000174721	FGFBP3	c17	Oligodendrocyte Progenitor Cell/Oligodendrocyte
ENSG00000182118	FAM89A	c17	Oligodendrocyte Progenitor Cell/Oligodendrocyte
ENSG00000184384	MAML2	c17	Oligodendrocyte Progenitor Cell/Oligodendrocyte
ENSG00000185985	SLITRK2	c17	Oligodendrocyte Progenitor Cell/Oligodendrocyte
ENSG00000198719	DLL1	c17	Oligodendrocyte Progenitor Cell/Oligodendrocyte
ENSG00000207955	MIR219A2	c17	Oligodendrocyte Progenitor Cell/Oligodendrocyte
ENSG00000263958	AC091138.1	c17	Oligodendrocyte Progenitor Cell/Oligodendrocyte
ENSG00000018408	WWTR1	c18	Truncated Radial Glia
ENSG00000018625	ATP1A2	c18	Truncated Radial Glia
ENSG00000026103	FAS	c18	Truncated Radial Glia
ENSG00000049323	LTBP1	c18	Truncated Radial Glia
ENSG00000068078	FGFR3	c18	Truncated Radial Glia
ENSG00000083937	CHMP2B	c18	Truncated Radial Glia
ENSG00000105855	ITGB8	c18	Truncated Radial Glia
ENSG00000106025	TSPAN12	c18	Truncated Radial Glia
ENSG00000111783	RFX4	c18	Truncated Radial Glia
ENSG00000116729	WLS	c18	Truncated Radial Glia
ENSG00000118523	CTGF	c18	Truncated Radial Glia
ENSG00000121005	CRISPLD1	c18	Truncated Radial Glia
ENSG00000122644	ARL4A	c18	Truncated Radial Glia
ENSG00000124942	AHNAK	c18	Truncated Radial Glia
ENSG00000129474	AJUBA	c18	Truncated Radial Glia
ENSG00000129654	FOXJ1	c18	Truncated Radial Glia
ENSG00000132688	NES	c18	Truncated Radial Glia
ENSG00000138696	BMPR1B	c18	Truncated Radial Glia
ENSG00000139352	ASCL1	c18	Truncated Radial Glia
ENSG00000144857	BOC	c18	Truncated Radial Glia
ENSG00000148053	NTRK2	c18	Truncated Radial Glia
ENSG00000148204	CRB2	c18	Truncated Radial Glia
ENSG00000154188	ANGPT1	c18	Truncated Radial Glia
ENSG00000154553	PDLIM3	c18	Truncated Radial Glia
ENSG00000162493	PDPN	c18	Truncated Radial Glia
ENSG00000162944	RFTN2	c18	Truncated Radial Glia
ENSG00000164199	ADGRV1	c18	Truncated Radial Glia
ENSG00000164694	FNDC1	c18	Truncated Radial Glia
ENSG00000165309	ARMC3	c18	Truncated Radial Glia
ENSG00000169860	P2RY1	c18	Truncated Radial Glia
ENSG00000170430	MGMT	c18	Truncated Radial Glia
ENSG00000174021	GNG5	c18	Truncated Radial Glia
ENSG00000174721	FGFBP3	c18	Truncated Radial Glia
ENSG00000176771	NCKAP5	c18	Truncated Radial Glia
ENSG00000187773	FAM69C	c18	Truncated Radial Glia
ENSG00000188290	HES4	c18	Truncated Radial Glia
ENSG00000189067	LITAF	c18	Truncated Radial Glia
ENSG00000197921	HES5	c18	Truncated Radial Glia
ENSG00000198435	NRARP	c18	Truncated Radial Glia
ENSG00000198719	DLL1	c18	Truncated Radial Glia
ENSG00000231064	AC234582.1	c18	Truncated Radial Glia
ENSG00000010278	CD9	c19	Pericyte
ENSG00000018408	WWTR1	c19	Pericyte
ENSG00000018625	ATP1A2	c19	Pericyte
ENSG00000050555	LAMC3	c19	Pericyte
ENSG00000069431	ABCC9	c19	Pericyte
ENSG00000088992	TESC	c19	Pericyte
ENSG00000091879	ANGPT2	c19	Pericyte
ENSG00000091986	CCDC80	c19	Pericyte
ENSG00000100376	FAM118A	c19	Pericyte
ENSG00000101384	JAG1	c19	Pericyte
ENSG00000106025	TSPAN12	c19	Pericyte
ENSG00000110090	CPT1A	c19	Pericyte
ENSG00000112562	SMOC2	c19	Pericyte
ENSG00000116729	WLS	c19	Pericyte
ENSG00000117318	ID3	c19	Pericyte
ENSG00000118523	CTGF	c19	Pericyte
ENSG00000121064	SCPEP1	c19	Pericyte
ENSG00000121361	KCNJ8	c19	Pericyte
ENSG00000122644	ARL4A	c19	Pericyte
ENSG00000124762	CDKN1A	c19	Pericyte
ENSG00000124942	AHNAK	c19	Pericyte
ENSG00000126803	HSPA2	c19	Pericyte
ENSG00000130158	DOCK6	c19	Pericyte
ENSG00000136160	EDNRB	c19	Pericyte
ENSG00000136732	GYPC	c19	Pericyte
ENSG00000139679	LPAR6	c19	Pericyte

ENSG00000140545	MFGE8	c19	Pericyte
ENSG00000141338	ABCA8	c19	Pericyte
ENSG00000141540	TTYH2	c19	Pericyte
ENSG00000143333	RGS16	c19	Pericyte
ENSG00000150636	CCDC102B	c19	Pericyte
ENSG00000151468	CCDC3	c19	Pericyte
ENSG00000158859	ADAMTS4	c19	Pericyte
ENSG00000162745	OLFML2B	c19	Pericyte
ENSG00000163132	MSX1	c19	Pericyte
ENSG00000164330	EBF1	c19	Pericyte
ENSG00000166831	RBPMS2	c19	Pericyte
ENSG00000170430	MGMT	c19	Pericyte
ENSG00000172340	SUCLG2	c19	Pericyte
ENSG00000174021	GNG5	c19	Pericyte
ENSG00000182871	COL18A1	c19	Pericyte
ENSG00000184384	MAML2	c19	Pericyte
ENSG00000185551	NR2F2	c19	Pericyte
ENSG00000186907	RTN4RL2	c19	Pericyte
ENSG00000187498	COL4A1	c19	Pericyte
ENSG00000187608	ISG15	c19	Pericyte
ENSG00000188290	HES4	c19	Pericyte
ENSG00000189184	PCDH18	c19	Pericyte
ENSG00000198435	NRARP	c19	Pericyte
ENSG00000247809	NR2F2-AS1	c19	Pericyte
ENSG00000248441	LINC01197	c19	Pericyte
ENSG00000103460	TOX3	c1	CGE Interneuron
ENSG00000114251	WNT5A	c1	CGE Interneuron
ENSG00000116729	WLS	c1	CGE Interneuron
ENSG00000117707	PROX1	c1	CGE Interneuron
ENSG00000121966	CXCR4	c1	CGE Interneuron
ENSG00000145242	EPHA5	c1	CGE Interneuron
ENSG00000149243	KLHL35	c1	CGE Interneuron
ENSG00000151090	THRB	c1	CGE Interneuron
ENSG00000171208	NETO2	c1	CGE Interneuron
ENSG00000175745	NR2F1	c1	CGE Interneuron
ENSG00000185551	NR2F2	c1	CGE Interneuron
ENSG00000237187	NR2F1-AS1	c1	CGE Interneuron
ENSG00000010278	CD9	c20	Endothelial Cell
ENSG00000018408	WWTR1	c20	Endothelial Cell
ENSG00000018625	ATP1A2	c20	Endothelial Cell
ENSG00000064666	CNN2	c20	Endothelial Cell
ENSG00000069431	ABCC9	c20	Endothelial Cell
ENSG00000071282	LMCD1	c20	Endothelial Cell
ENSG00000084453	SLCO1A2	c20	Endothelial Cell
ENSG00000088992	TESC	c20	Endothelial Cell
ENSG00000091879	ANGPT2	c20	Endothelial Cell
ENSG00000100376	FAM118A	c20	Endothelial Cell
ENSG00000101384	JAG1	c20	Endothelial Cell
ENSG00000102760	RGCC	c20	Endothelial Cell
ENSG00000110090	CPT1A	c20	Endothelial Cell
ENSG00000116729	WLS	c20	Endothelial Cell
ENSG00000117318	ID3	c20	Endothelial Cell
ENSG00000118523	CTGF	c20	Endothelial Cell
ENSG00000121966	CXCR4	c20	Endothelial Cell
ENSG00000122644	ARL4A	c20	Endothelial Cell
ENSG00000123700	KCNJ2	c20	Endothelial Cell
ENSG00000124762	CDKN1A	c20	Endothelial Cell
ENSG00000124942	AHNAK	c20	Endothelial Cell
ENSG00000128567	PODXL	c20	Endothelial Cell
ENSG00000130147	SH3BP4	c20	Endothelial Cell
ENSG00000130158	DOCK6	c20	Endothelial Cell
ENSG00000130513	GDF15	c20	Endothelial Cell
ENSG00000132688	NES	c20	Endothelial Cell
ENSG00000135048	TMEM2	c20	Endothelial Cell
ENSG00000137834	SMAD6	c20	Endothelial Cell
ENSG00000139679	LPAR6	c20	Endothelial Cell
ENSG00000140511	HAPLN3	c20	Endothelial Cell
ENSG00000141540	TTYH2	c20	Endothelial Cell
ENSG00000142627	EPHA2	c20	Endothelial Cell
ENSG00000143341	HMCN1	c20	Endothelial Cell
ENSG00000156925	ZIC3	c20	Endothelial Cell
ENSG00000157978	LDLRAP1	c20	Endothelial Cell
ENSG00000158352	SHROOM4	c20	Endothelial Cell
ENSG00000161638	ITGA5	c20	Endothelial Cell
ENSG00000162496	DHRS3	c20	Endothelial Cell
ENSG00000163132	MSX1	c20	Endothelial Cell
ENSG00000164330	EBF1	c20	Endothelial Cell

ENSG00000172340	SUCLG2	c20	Endothelial Cell
ENSG00000174021	GNG5	c20	Endothelial Cell
ENSG00000182118	FAM89A	c20	Endothelial Cell
ENSG00000182871	COL18A1	c20	Endothelial Cell
ENSG00000184497	TMEM255B	c20	Endothelial Cell
ENSG00000185483	ROR1	c20	Endothelial Cell
ENSG00000187498	COL4A1	c20	Endothelial Cell
ENSG00000187608	ISG15	c20	Endothelial Cell
ENSG00000188290	HES4	c20	Endothelial Cell
ENSG00000198435	NRARP	c20	Endothelial Cell
ENSG00000198719	DLL1	c20	Endothelial Cell
ENSG00000088992	TESC	c21	Red Blood Cell
ENSG00000136732	GYPC	c21	Red Blood Cell
ENSG00000010278	CD9	c22	Vascular and Leptomeningeal Cell
ENSG00000018408	WWTR1	c22	Vascular and Leptomeningeal Cell
ENSG00000018625	ATP1A2	c22	Vascular and Leptomeningeal Cell
ENSG00000069702	TGFBR3	c22	Vascular and Leptomeningeal Cell
ENSG00000091879	ANGPT2	c22	Vascular and Leptomeningeal Cell
ENSG00000091986	CCDC80	c22	Vascular and Leptomeningeal Cell
ENSG00000102760	RGCC	c22	Vascular and Leptomeningeal Cell
ENSG00000112893	MAN2A1	c22	Vascular and Leptomeningeal Cell
ENSG00000114251	WNT5A	c22	Vascular and Leptomeningeal Cell
ENSG00000116729	WLS	c22	Vascular and Leptomeningeal Cell
ENSG00000117318	ID3	c22	Vascular and Leptomeningeal Cell
ENSG00000121064	SCPEP1	c22	Vascular and Leptomeningeal Cell
ENSG00000122644	ARL4A	c22	Vascular and Leptomeningeal Cell
ENSG00000124942	AHNAK	c22	Vascular and Leptomeningeal Cell
ENSG00000126803	HSPA2	c22	Vascular and Leptomeningeal Cell
ENSG00000130513	GDF15	c22	Vascular and Leptomeningeal Cell
ENSG00000134853	PDGFRA	c22	Vascular and Leptomeningeal Cell
ENSG00000136732	GYPC	c22	Vascular and Leptomeningeal Cell
ENSG00000137801	THBS1	c22	Vascular and Leptomeningeal Cell
ENSG00000138829	FBN2	c22	Vascular and Leptomeningeal Cell
ENSG00000141338	ABCA8	c22	Vascular and Leptomeningeal Cell
ENSG00000143333	RGS16	c22	Vascular and Leptomeningeal Cell
ENSG00000144476	ACKR3	c22	Vascular and Leptomeningeal Cell
ENSG00000145794	MEGF10	c22	Vascular and Leptomeningeal Cell
ENSG00000146242	TPBG	c22	Vascular and Leptomeningeal Cell
ENSG00000148053	NTRK2	c22	Vascular and Leptomeningeal Cell
ENSG00000158859	ADAMTS4	c22	Vascular and Leptomeningeal Cell
ENSG00000162493	PDPN	c22	Vascular and Leptomeningeal Cell
ENSG00000162496	DHRS3	c22	Vascular and Leptomeningeal Cell
ENSG00000162745	OLFML2B	c22	Vascular and Leptomeningeal Cell
ENSG00000163132	MSX1	c22	Vascular and Leptomeningeal Cell
ENSG00000166426	CRABP1	c22	Vascular and Leptomeningeal Cell
ENSG00000173376	NDNF	c22	Vascular and Leptomeningeal Cell
ENSG00000175745	NR2F1	c22	Vascular and Leptomeningeal Cell
ENSG00000182871	COL18A1	c22	Vascular and Leptomeningeal Cell
ENSG00000185483	ROR1	c22	Vascular and Leptomeningeal Cell
ENSG00000185551	NR2F2	c22	Vascular and Leptomeningeal Cell
ENSG00000187498	COL4A1	c22	Vascular and Leptomeningeal Cell
ENSG00000187608	ISG15	c22	Vascular and Leptomeningeal Cell
ENSG00000189184	PCDH18	c22	Vascular and Leptomeningeal Cell
ENSG00000076356	PLXNA2	c2	Glutamatergic Neuron 1
ENSG00000137801	THBS1	c2	Glutamatergic Neuron 1
ENSG00000144485	HES6	c2	Glutamatergic Neuron 1
ENSG00000149243	KLHL35	c2	Glutamatergic Neuron 1
ENSG00000174498	IGDCC3	c2	Glutamatergic Neuron 1
ENSG00000175745	NR2F1	c2	Glutamatergic Neuron 1
ENSG00000185668	POU3F1	c2	Glutamatergic Neuron 1
ENSG00000225783	MIAT	c2	Glutamatergic Neuron 1
ENSG00000272168	CASC15	c2	Glutamatergic Neuron 1
ENSG00000116729	WLS	c3	MGE Interneuron
ENSG00000117318	ID3	c3	MGE Interneuron
ENSG00000121966	CXCR4	c3	MGE Interneuron
ENSG00000130147	SH3BP4	c3	MGE Interneuron
ENSG00000135048	TMEM2	c3	MGE Interneuron
ENSG00000136099	PCDH8	c3	MGE Interneuron
ENSG00000144476	ACKR3	c3	MGE Interneuron
ENSG00000171208	NETO2	c3	MGE Interneuron
ENSG00000175264	CHST1	c3	MGE Interneuron
ENSG00000076356	PLXNA2	c4	Glutamatergic Neuron 5
ENSG00000136099	PCDH8	c4	Glutamatergic Neuron 5
ENSG00000149243	KLHL35	c4	Glutamatergic Neuron 5
ENSG00000174498	IGDCC3	c4	Glutamatergic Neuron 5
ENSG00000184486	POU3F2	c4	Glutamatergic Neuron 5
ENSG00000229807	XIST	c4	Glutamatergic Neuron 5

ENSG00000272168	CASC15	c4	Glutamatergic Neuron 5
ENSG00000076356	PLXNA2	c5	Glutamatergic Neuron 3
ENSG00000175745	NR2F1	c5	Glutamatergic Neuron 3
ENSG00000185668	POU3F1	c5	Glutamatergic Neuron 3
ENSG00000018625	ATP1A2	c6	Early Radial Glia
ENSG00000049323	LTBP1	c6	Early Radial Glia
ENSG00000068078	FGFR3	c6	Early Radial Glia
ENSG00000101198	NKAIN4	c6	Early Radial Glia
ENSG00000105810	CDK6	c6	Early Radial Glia
ENSG00000105855	ITGB8	c6	Early Radial Glia
ENSG00000111783	RFX4	c6	Early Radial Glia
ENSG00000118523	CTGF	c6	Early Radial Glia
ENSG00000132688	NES	c6	Early Radial Glia
ENSG00000136732	GYPC	c6	Early Radial Glia
ENSG00000138829	FBN2	c6	Early Radial Glia
ENSG00000140545	MFGE8	c6	Early Radial Glia
ENSG00000144857	BOC	c6	Early Radial Glia
ENSG00000145794	MEGF10	c6	Early Radial Glia
ENSG00000147509	RGS20	c6	Early Radial Glia
ENSG00000148204	CRB2	c6	Early Radial Glia
ENSG00000154553	PDLIM3	c6	Early Radial Glia
ENSG00000162493	PDPN	c6	Early Radial Glia
ENSG00000164199	ADGRV1	c6	Early Radial Glia
ENSG00000170430	MGMT	c6	Early Radial Glia
ENSG00000174021	GNG5	c6	Early Radial Glia
ENSG00000174721	FGFBP3	c6	Early Radial Glia
ENSG00000175745	NR2F1	c6	Early Radial Glia
ENSG00000176771	NCKAP5	c6	Early Radial Glia
ENSG00000185985	SLITRK2	c6	Early Radial Glia
ENSG00000187773	FAM69C	c6	Early Radial Glia
ENSG00000188290	HES4	c6	Early Radial Glia
ENSG00000189067	LITAF	c6	Early Radial Glia
ENSG00000197921	HES5	c6	Early Radial Glia
ENSG00000114423	CBLB	c7	Glutamatergic Neuron 7
ENSG00000148053	NTRK2	c7	Glutamatergic Neuron 7
ENSG00000225783	MIAT	c7	Glutamatergic Neuron 7
ENSG00000049323	LTBP1	c8	Cycling Progenitor
ENSG00000072571	HMMR	c8	Cycling Progenitor
ENSG00000105810	CDK6	c8	Cycling Progenitor
ENSG00000105855	ITGB8	c8	Cycling Progenitor
ENSG00000116830	TTF2	c8	Cycling Progenitor
ENSG00000123307	NEUROD4	c8	Cycling Progenitor
ENSG00000132688	NES	c8	Cycling Progenitor
ENSG00000139352	ASCL1	c8	Cycling Progenitor
ENSG00000140545	MFGE8	c8	Cycling Progenitor
ENSG00000144485	HES6	c8	Cycling Progenitor
ENSG00000144857	BOC	c8	Cycling Progenitor
ENSG00000154553	PDLIM3	c8	Cycling Progenitor
ENSG00000162493	PDPN	c8	Cycling Progenitor
ENSG00000164199	ADGRV1	c8	Cycling Progenitor
ENSG00000174021	GNG5	c8	Cycling Progenitor
ENSG00000175745	NR2F1	c8	Cycling Progenitor
ENSG00000189067	LITAF	c8	Cycling Progenitor
ENSG00000197061	HIST1H4C	c8	Cycling Progenitor
ENSG00000197921	HES5	c8	Cycling Progenitor
ENSG00000245694	CRNDE	c8	Cycling Progenitor

Fig4G and SuppFig 2 Abbreviations

Cluster ID	Abbr.	Name
c0	GluN5	Glutamatergic Neuron 5
c1	CGE IN	CGE Interneuron
c2	GluN1	Glutamatergic Neuron 1
c3	MGE IN	MGE Interneuron
c4	GluN4	Glutamatergic Neuron 4
c5	GluN2	Glutamatergic Neuron 2
c6	Early RG	Early Radial Glia
c7	GluN7	Glutamatergic Neuron 7
c8	Cyc. Prog.	Cycling Progenitor
c9	GluN3	Glutamatergic Neuron 3
c10	Late RG	Late Radial Glia
c11	mGPC	Multipotent glial progenitor cell
c12	GluN6	Glutamatergic Neuron 6
c13	SP	Subplate
c14	nIPC	neuronal intermedate progenitor cell
c15	GluN8	Glutamatergic Neuron 8
c16	MG	Microglia
c17	OPC/Oligo	Oligodendrocyte Progenitor Cell/Oligodendrocyte
c18	tRG	Truncated Radial Glia
c19	Peric	Pericyte
c20		Endothelial Cell
c21		RBC
c22		Vascular and Leptomeningeal Cell

Fig4H 4I Cell fraction values

	Averaged N (Fig 4)		
	HC1	A1	A2
c0	0	0.165	0
c1	1.231	0.849	1.014
c2	1.363	1.315	1.388
c3	0.661	0.777	0.394
c4	3.274	3.001	2.943
c5	0.238	0.578	0.036
c6	0.371	0.788	0.76
c7	0.717	0.824	0.835
c8	0.893	1.069	1.327
c10	0.958	0.754	0
c12	0.142	0	0.076
c13	0.614	0.261	0.14
c14	1.298	0.796	1.524
c17	0.158	0.11	0.134
c18	0.025	0	0.257
c9	0	0	0
c20	0	0	0
c19	0	0	0
c16	0	0	0
c11	0	0	0
P-value	0.000	0.000	0.000
Correlation	0.873	0.813	0.736
RMSE	0.61	0.654	0.723

Fig 4J c10 Downreg

Genes	logFC	logCPM	PValue	FDR
ARX	7.651266291	9.213176982	5.64E-36	4.89E-33
LHX2	6.014925851	8.948118135	1.06E-22	4.58E-20
FZD8	4.610628486	9.050918743	1.41E-17	4.08E-15
NR2E1	7.582399041	7.942100314	4.19E-17	9.08E-15
TFAP2C	6.526819295	6.518316073	8.57E-14	1.49E-11
LINC01551	7.486923348	9.488010113	2.95E-13	4.26E-11
EMX2OS	5.286193164	8.545527388	3.92E-13	4.85E-11
TNC	3.474654565	9.711183402	8.96E-13	9.71E-11
NPY	6.436382279	5.08308365	2.26E-12	2.17E-10
COL4A6	3.548149411	10.47356843	3.56E-12	3.09E-10
MPPED2	3.490644634	10.19923001	6.76E-11	5.33E-09
EMX2	5.461574141	8.276389691	1.53E-10	1.10E-08
SALL3	3.439065225	8.933396692	3.36E-10	2.24E-08
DMRTA2	6.040746112	7.239423054	5.06E-10	3.13E-08
HOPX	5.170294459	5.746149386	5.72E-10	3.31E-08
FBXO32	4.725488976	7.661038184	8.75E-10	4.46E-08
CHRDL1	2.740989012	9.554902119	7.56E-09	3.64E-07
MEIS2	3.463639311	10.7259814	2.04E-08	9.33E-07
SFRP1	4.091986518	11.0907591	4.78E-08	2.07E-06
FEZF2	4.879633657	7.026289873	6.64E-08	2.74E-06
CREB5	2.672810011	9.765301031	9.41E-08	3.55E-06
CRH	4.998847081	4.444810736	1.38E-07	4.90E-06
PMP2	4.319899659	5.01610409	1.41E-07	4.90E-06
IL33	5.037658797	6.12988917	2.06E-06	6.86E-05
LINC01965	4.624345542	6.266172024	2.27E-06	7.30E-05
TCIM	4.476210695	6.04221848	5.83E-06	0.000180655
TSTD1	3.64579281	4.79842617	6.65E-06	0.000198801
ETV1	2.454144902	9.751497304	7.31E-06	0.000211255
FUT9	2.069387911	10.35617641	1.11E-05	0.000310112
LRRTM3	2.623124785	8.257152033	1.80E-05	0.000473746
FAM181B	2.893150801	8.096019048	2.40E-05	0.000612241
CDCA7L	2.236449075	8.804685883	3.08E-05	0.000762826
SNTG1	2.279022676	8.833807078	4.06E-05	0.000977453
PAX6	2.014910319	11.2693284	5.33E-05	0.001247855
SNCA	2.101072384	9.068989415	7.49E-05	0.001665065
FAM84A	1.977099828	9.24150978	0.000105852	0.002185079
C1QL2	3.35101538	3.151814773	0.000125227	0.00252492
HTR2A	3.595163313	4.161454343	0.00023627	0.004655599
AC009041.2	2.200766776	8.415468331	0.000255546	0.004923518
WIPF3	2.736130154	7.421917356	0.000312966	0.00577322
NCAM2	1.933804999	8.680041719	0.000485908	0.008101579
C2orf72	2.153023653	8.131545964	0.000509662	0.008337295

Fig 4J c10 Upregulated

**Genes**	**logFC**	**logCPM**	**P Value**	**FDR**
SLITRK2	-5.145962875	8.604066593	7.92E-10	4.29E-08
PDPN	-3.253565672	10.07789938	8.55E-08	3.37E-06
EDNRB	-2.600674454	11.04094093	1.15E-05	0.00031085
PCDH8	-2.31228097	10.45237921	5.75E-05	0.001311909
RFTN2	-2.262875981	9.617872294	9.80E-05	0.002124081
CXCR4	-2.386834104	10.77715662	0.000105522	0.002185079
NR2F1	-2.241465686	11.64787763	0.000274361	0.005171102
FGFR3	-2.730161018	11.16238133	0.000323903	0.005850493
RFX4	-2.283958926	10.95849407	0.000409955	0.007253687
NKAIN4	-2.003610102	10.19883299	0.000448873	0.007783455
ADGRV1	-2.079324148	11.20983189	0.000476163	0.008094767

Supp Fig 2 - Signature Matrix

NAME	c16	c11	c17	c0	c4	c10	c9	c19	c13	c14	c1	c8	c18	c3	c5	c6	c7	c20	c2	c12
A2M	712.0761	1	1	1	1	1	1	295.9129	1	1	1	1	1	1	1	1	1	908.0203	1	1
A4GALT	1	1	1	1	1	1	1	1	1	1	1	1	1	1	1	1	1	51.86744	1	1
AAAS	1	1	31.62408	1	1	32.38318	1	1	1	52.9653	1	43.39219	32.30922	1	58.73609	1	1	1	1	70.02264
AACS	1	1	1	1	41.24182	1	1	1	49.93302	36.58306	1	35.58428	1	1	69.76785	1	1	1	1	70.02264
AADAT	1	1	51.14729	1	1	1	1	1	1	1	1	1	1	1	1	1	1	1	1	1
AAED1	98.07128	1	1	1	1	34.38414	1	33.66055	1	1	1	1	43.0043	1	1	1	1	1	1	1
AAMP	85.37603	60.62942	59.01116	46.75495	47.59384	72.05308	1	68.81261	1	58.9841	66.10307	54.04475	53.73341	1	1	1	1	40.20016	112.2106	1
AASS	1	55.456	45.4718	1	1	39.6761	1	39.10886	1	1	1	32.50573	44.03583	1	1	1	1	1	1	1
AATF	1	40.84373	47.8583	51.07496	45.57247	1	1	25.03394	1	54.19839	1	35.09408	35.6649	1	1	1	1	1	1	103.6483
ABAT	1	72.02013	149.0165	1	1	139.3254	1	1	130.5365	48.47499	1	70.25269	109.6967	1	1	93.33331	1	1	1	139.0453
ABCA1	70.01483	1	1	59.73284	1	1	1	42.7577	1	1	1	1	45.84827	1	1	1	1	122.5686	1	1
ABCA10	1	1	1	1	1	1	1	48.97558	1	1	1	1	1	1	1	1	1	1	1	1
ABCA8	1	1	1	1	1	1	1	81.60561	1	1	1	1	1	1	1	1	1	1	1	1
ABCA9	1	1	1	1	1	1	1	82.51317	1	1	1	1	1	1	1	1	1	1	1	1
ABCB1	1	1	1	1	1	1	1	1	1	1	1	1	1	1	1	1	1	204.1499	1	1
ABCC5	1	1	76.3641	57.78872	53.36313	1	1	31.38526	1	1	74.20087	1	1	72.32041	60.88709	1	116.4449	15.26364	1	1
ABCC9	1	1	1	1	1	1	1	732.1897	1	1	1	1	1	1	1	1	1	1	1	1
ABCG1	48.64527	1	1	1	1	1	1	1	1	1	1	1	1	1	1	1	1	71.8404	1	1
ABCG2	1	1	1	1	1	1	1	1	1	1	1	1	1	1	1	1	1	546.6366	1	1
ABHD10	1	36.93059	1	1	1	47.79008	1	1	1	1	1	30.34442	39.92793	1	1	1	1	1	1	139.0453
ABHD14A	1	1	50.96505	1	1	22.94043	1	19.2134	157.5179	1	1	1	37.42393	1	1	31.27809	1	26.58682	1	1
ABHD17B	1	40.2997	56.0572	47.15789	67.99096	32.35587	1	28.41564	1	54.6017	79.81123	44.2304	46.49874	87.58458	1	1	1	67.89047	1	1
ABHD3	1	51.60152	1	1	1	106.6153	1	1	1	28.75838	1	42.87383	38.81664	1	1	1	1	22.56221	1	1
ABHD6	1	1	1	1	1	1	1	1	1	1	1	1	1	1	1	1	84.23204	1	1	1
ABL1	1	43.3154	42.14219	47.24017	1	1	1	32.74818	1	46.83377	1	43.7866	38.62287	1	65.92312	1	1	41.21574	1	103.6483
ABLIM1	1	43.98534	1	1	1	1	1	1	78.90943	1	1	1	33.79779	1	1	1	122.6939	459.1226	1	1
ABLIM3	1	1	1	1	1	1	1	1	1	1	1	1	1	1	1	1	1	167.3448	1	1
ABR	44.54362	38.30017	1	50.45084	57.48051	30.06398	1	1	161.8584	35.49615	1	27.59957	45.3437	1	58.23387	1	236.7652	1	55.94744	1
ABRACL	137.9712	1	1	132.2512	160.4134	28.80212	147.524	1	95.57453	1	112.9441	25.71158	1	85.89014	182.5144	1	1	38.18246	209.1531	172.671
ABRAXAS1	1	1	1	1	1	1	1	1	1	1	1	1	26.02575	1	1	1	1	29.76842	88.8115	1
ABTB1	1	1	1	1	1	1	1	63.69141	1	1	1	1	1	1	1	1	1	1	1	70.02264
AC003681.	1	1	1	57.85809	52.13032	1	1	1	1	36.82514	87.50548	1	25.16236	1	1	1	58.48795	1	80.74097	190.2284
AC004158.	1	114.1517	295.4554	78.61512	92.33366	1	1	1	427.3839	1	200.6891	1	1	337.0374	119.334	1	421.4001	1	1	103.6483
AC004540.	1	1	98.59322	1	1	1	1	1	1	1	1	1	1	1	1	1	1	1	1	103.6483
AC004951.	1	1	1	1	1	1	1	1	1	1	1	23.68312	1	1	1	1	1	12.43253	1	103.6483
AC005332.	1	1	1	1	1	36.99489	1	1	1	1	1	1	34.3	1	1	1	1	1	1	1
AC005332.	1	1	1	1	1	39.0236	1	1	1	1	1	21.75424	1	1	1	1	1	1	1	1
AC006450.	1	1	1	1	1	1	1	1	1	34.76101	1	32.22272	1	1	1	1	1	1	1	1
AC007240.	1	1	1	1	1	1	1	1	1	1	1	32.76522	1	1	1	1	1	1	1	1
AC007952.	52.45429	1	1	1	1	36.93938	1	1	1	1	1	1	1	1	1	1	1	24.02513	1	70.02264
AC008124.	1	40.98577	100.8017	1	1	1	1	1	1	1	1	28.63489	34.85733	1	1	1	1	1	1	87.58009
AC009041.	1	140.6918	427.2773	55.40898	53.73757	63.43892	1	1	1	1	92.98666	1	1	1	1	49.84382	114.9757	1	1	1
AC010931.	1	1	1	1	41.27715	1	1	1	1	1	1	1	1	1	1	1	1	1	63.64884	1
AC011447.	1	1	1	1	1	1	1	1	1	41.76227	1	32.70987	1	1	1	1	1	1	1	1
AC012306.	1	1	85.78043	1	1	31.58104	1	21.6818	1	1	1	27.13708	1	1	1	1	1	1	1	103.6483
AC016746.	1	1	1	1	1	1	1	1	1	1	1	1	1	1	1	1	245.9023	1	1	103.6483
AC020916.	111.1259	1	1	1	1	85.69228	1	47.78562	1	1	1	27.22091	77.44079	1	1	1	1	49.23025	1	1
AC022613.	1	1	1	1	1	1	1	1	1	1	1	1	1	1	1	1	1	64.05924	1	1
AC026401.	1	1	1	1	1	1	1	1	1	1	1	30.50454	1	1	1	1	1	1	1	1
AC027644.	1	1	1	1	1	27.96084	1	1	1	1	1	1	1	1	1	1	1	1	1	1
AC044849.	1	1	1	1	1	1	1	1	1	1	1	1	53.42847	1	1	1	1	1	1	1
AC060780.	1	1	1	1	1	29.79754	1	1	1	1	1	1	50.92934	1	1	113.0126	1	1	1	1
AC068491.	1	1	1	1	1	1	1	67.38029	1	1	1	1	1	1	1	1	1	1	1	1
AC084033.	1	1	1	1	1	44.38091	1	1	1	27.54438	1	52.40688	124.4639	63.73078	1	1	1	1	1	1
AC087241.	1	1	1	1	1	39.09179	1	1	1	1	1	1	1	1	1	1	1	1	1	1
AC090114.	1	1	1	1	1	1	1	1	1	1	1	28.45542	1	1	1	1	1	1	1	1
AC090152.	1	1	1	1	1	42.22387	1	1	1	1	1	23.34663	1	1	1	1	1	1	1	1
AC090692.	1	35.26177	1	1	1	1	1	1	1	1	1	1	1	1	1	1	1	1	1	1
AC091138.	1	1	35.70378	1	1	1	1	1	1	1	1	1	1	1	1	1	1	1	1	1
AC092376.	1	1	1	1	1	1	1	1	1	1	1	1	1	60.23635	1	1	1	1	1	1
AC093323.	1	1	1	1	1	1	1	1	1	29.08628	1	28.54304	1	1	1	1	1	1	1	1
AC097376.	1	1	1	49.43431	72.01485	27.94738	1	1	133.7706	1	1	1	1	1	107.5903	1	102.0386	1	105.0018	308.945
AC097534.	1	1	1	1	1	1	1	1	1	1	1	38.11243	1	1	1	1	1	1	1	1
AC098850.	1	1	1	1	1	1	1	1	158.5757	1	1	1	1	1	1	1	91.98093	1	1	70.02264
AC099792.	1	1	1	1	1	27.54472	1	1	1	1	1	1	1	1	1	1	1	1	1	1
AC108463.	1	1	1	1	1	1	1	49.55459	1	1	1	1	1	1	1	1	1	1	1	1
AC116565.	1	1	1	1	1	1	1	1	174.2447	1	1	1	1	1	1	1	1	1	1	70.02264
AC120042.	1	90.72747	32.78993	144.9966	68.44407	200.0589	1	29.48349	1	308.7344	1	239.0724	242.7181	1	64.06772	171.7615	1	1	92.07109	1
AC131571.	1	1	1	1	1	59.91337	1	1	1	1	1	1	28.09394	1	1	44.9885	1	1	1	1
AC234582.	1	1	1	1	1	43.10838	1	1	1	1	1	1	48.35794	1	1	1	1	1	1	1
AC245014.	76.74539	1	1	1	1	1	1	1	1	1	1	1	1	1	1	1	1	1	1	1
AC245060.	1	1	1	1	47.67436	1	1	1	57.821	1	1	1	1	1	1	1	219.2993	1	1	1
AC245297.	1	43.68394	1	1	1	66.49152	1	61.13813	1	1	1	1	69.50372	1	1	1	1	1	1	1
AC245595.	1	1	1	1	1	1	1	1	1	1	1	1	1	1	1	1	58.90086	38.95455	1	139.0453
ACAA2	84.63719	102.8733	67.63432	1	1	114.5158	1	50.0543	1	98.01503	1	86.17367	66.0116	1	1	123.733	1	23.90477	69.98894	1
ACAD8	1	1	88.28897	1	1	48.96043	1	1	1	1	1	23.53938	30.39462	1	1	1	1	19.46931	1	1
ACAP3	1	67.6268	171.1617	77.83538	73.99258	1	1	21.0285	121.7158	57.30863	1	35.06092	1	68.7539	99.23077	1	147.1605	1	84.60074	103.6483
ACE	1	1	1	1	1	1	1	1	1	1	1	1	1	1	1	1	1	92.9574	1	1
ACKR3	1	1	1	1	1	1	1	1	1	1	1	1	1	199.4472	1	1	1	1	1	1
ACOT13	80.18038	29.68902	1	1	1	47.15963	1	43.20087	1	1	1	1	38.95515	1	1	51.02939	1	17.29617	1	70.02264
ACOT7	1	1	1	1	60.95994	1	1	1	67.17232	41.6631	1	39.57152	1	1	103.7399	1	139.1215	1	1	87.58009
ACOX1	1	48.24039	37.23223	49.64755	54.20381	88.73025	1	1	1	45.30112	1	35.95862	46.095	1	1	1	96.79147	43.89817	1	1
ACP2	1	1	1	1	1	35.15964	1	1	1	1	1	1	1	1	1	1	1	31.28522	1	1
ACSBG1	1	1	1	1	1	44.99891	1	1	1	1	1	1	1	1	1	1	1	1	1	1
ACSL4	1	1	1	1	46.14595	1	1	25.99623	91.60618	1	1	1	1	1	69.517	1	50.11835	1	1	1
ACSL6	1	1	1	1	1	35.86324	1	1	105.7861	1	1	1	1	1	1	1	184.1863	1	1	1
ACSS1	1	44.51861	26.39862	1	1	29.313	1	1	1	1	1	1	1	1	1	1	1	29.24488	1	1
ACTL6B	1	1	1	61.92428	84.37092	1	1	1	50.73104	1	97.17035	1	1	100.7697	91.54178	1	94.80643	1	101.0741	70.02264
ACTN1	1	62.87087	1	1	1	134.5323	1	189.8129	1	45.23187	1	64.38862	161.3313	1	1	115.8441	1	76.09507	1	1
ACTN2	1	1	1	1	1	1	1	1	1	1	1	1	1	1	1	1	142.8803	1	1	1
ACTR1B	1	1	1	1	1	34.99556	1	1	1	33.16906	1	32.41114	30.46043	1	1	1	30.7295	38.86405	38.04797	1
ACTR3B	1	1	1	1	1	59.16559	1	1	52.51037	46.2372	1	34.17002	27.83009	1	1	43.07499	1	1	1	70.02264
ACVR1	1	31.03256	1	1	1	30.76655	1	1	1	1	154.5165	32.30254	1	1	1	1	1	65.46119	1	1
ACVR1B	1	1	53.44296	47.09633	54.38072	1	1	1	133.8749	1	1	24.99879	1	1	54.34739	1	1	1	51.28247	1
ACVR2B	1	65.519	54.67889	123.4303	90.25061	59.69224	1	1	1	79.72421	122.3742	79.62242	43.48742	152.8848	73.28588	74.5155	1	1	109.6553	1
ACVRL1	1	1	1	1	1	1	1	1	1	1	1	1	1	1	1	1	1	150.401	1	1
ADA	1	1	1	1	1	1	1	125.5899	1	1	1	1	1	1	1	1	1	18.54863	1	1
ADAM12	1	1	1	1	1	1	1	192.1569	1	1	1	1	1	1	1	1	1	1	1	1
ADAM22	1	1	43.70522	1	1	1	1	1	55.47914	1	1	1	1	1	55.42147	1	1	1	1	1
ADAM23	1	1	78.82044	1	1	1	1	1	93.15727	1	1	1	1	1	1	1	1	1	1	1

ADAM28	135.8998	1	1	1	1	1	1	1	1	1	1	1	1	1	1	1	1	1	1	1
ADAM33	1	1	1	1	1	1	1	31.83158	1	1	1	1	33.80222	1	1	1	1	1	1	1
ADAMTS1	1	1	1	1	1	1	1	576.0968	1	1	1	1	34.31704	1	1	1	1	170.3577	1	1
ADAMTS4	1	1	1	1	1	1	1	224.9289	1	1	1	1	1	1	1	1	1	1	1	1
ADAMTS6	1	57.8177	1	1	1	62.76127	1	1	1	1	1	29.96017	42.31252	1	1	1	1	1	1	1
ADAMTS9	1	1	1	1	1	1	1	93.80307	1	1	1	1	1	1	1	1	1	32.04028	1	1
ADAP2	243.3187	1	1	1	1	1	1	110.6157	1	1	1	1	1	1	1	1	1	1	1	1
ADARB2	1	1	1	1	1	1	1	1	1	1	81.20438	1	1	1	1	1	1	1	1	1
ADAT1	1	1	1	1	1	1	1	1	1	33.28046	1	29.33386	1	1	1	1	1	39.22026	1	1
ADCK2	62.15168	1	1	1	1	1	1	1	1	1	1	1	1	1	1	1	1	1	1	1
ADCY1	1	1	1	1	122.2537	1	237.7414	1	697.5635	1	1	1	1	1	243.5169	1	467.2887	24.89423	96.01706	1
ADCY2	1	1	1	1	1	45.49494	1	1	108.2225	1	1	1	1	1	1	1	1	1	1	1
ADCY6	1	1	39.16301	1	1	1	1	31.80585	1	1	1	1	1	1	1	1	1	1	1	1
ADCY9	1	1	1	1	1	1	1	52.50577	1	1	1	1	1	1	1	1	1	1	1	1
ADD2	1	41.45078	30.3388	87.35433	110.336	58.51728	165.9806	1	155.4765	48.68605	84.2561	67.36893	28.92309	102.4445	166.1605	69.76803	127.1432	1	204.9639	446.9903
ADD3	1	104.7639	100.5996	47.85922	60.74499	117.3552	1	180.457	139.0742	1	1	102.0921	124.7278	1	1	1	1	152.3855	178.0089	206.2967
ADGRA1	1	1	48.38271	1	1	1	1	1	1	1	1	1	1	1	1	1	1	1	1	1
ADGRA2	1	1	1	1	1	1	1	167.8112	1	1	1	1	1	1	1	1	1	71.14368	1	1
ADGRA3	1	45.11947	1	59.8974	43.92214	44.90712	1	1	1	54.4286	1	41.51666	51.09736	1	1	81.97701	1	1	1	70.02264
ADGRB1	1	40.00961	105.6042	1	1	26.69801	1	1	102.6233	1	1	1	1	1	1	1	1	1	1	103.6483
ADGRB3	1	73.89756	64.69075	105.4232	83.20147	139.8146	1	1	206.1735	71.25071	67.26343	69.58181	69.5806	1	1	90.54103	125.425	1	47.42976	103.6483
ADGRD1	1	1	1	1	1	1	1	113.7706	1	1	1	1	1	1	1	1	1	1	1	1
ADGRF5	1	1	1	1	1	1	1	222.3654	1	1	1	1	1	1	1	1	1	330.5476	1	1
ADGRG2	1	1	1	1	1	1	1	1	120.8406	1	1	1	1	1	1	1	1	1	1	1
ADGRG6	1	1	1	1	1	70.49135	1	1	1	1	1	1	1	1	1	1	1	29.16956	1	1
ADGRL2	1	1	1	1	1	28.34209	1	26.36547	1	1	1	1	1	1	1	1	1	94.69287	1	103.6483
ADGRL3	1	75.80783	267.0818	1	117.6674	1	1	39.22581	84.57697	1	149.7499	1	1	214.6759	239.8675	1	545.847	78.46726	1	208.0679
ADGRL4	1	1	1	1	1	1	1	1	1	1	1	1	1	1	1	1	1	280.1322	1	1
ADGRV1	1	172.8184	1	280.5299	201.9416	801.1939	156.1242	1	1	544.1354	1	305.9803	429.2276	1	87.1186	694.618	110.8252	1	282.2815	103.6483
ADK	1	51.89377	1	1	1	94.96285	1	74.1023	1	63.09046	1	67.34301	62.54876	1	1	130.5608	1	1	1	1
ADM	1	124.7659	1	1	1	1	1	73.32589	1	1	1	1	1	1	1	1	1	375.8157	1	1
ADO	1	34.82625	60.25984	1	1	1	1	1	1	1	1	1	34.33397	1	1	1	1	1	1	1
ADORA2B	1	1	1	1	1	1	1	170.7713	1	1	1	1	1	1	1	1	1	1	1	1
ADORA3	51.85719	1	1	1	1	1	1	1	1	1	1	1	1	1	1	1	1	1	1	1
ADRA2A	1	1	1	92.3261	235.5759	1	394.9026	1	291.0033	1	1	1	1	1	449.7629	1	1	1	190.4336	275.3193
ADSL	1	1	1	1	1	1	1	1	1	34.43987	1	1	1	1	1	1	1	1	1	103.6483
AEBP1	1	45.98838	1	1	1	1	1	88.11603	1	1	1	1	40.50893	1	1	1	1	17.69428	1	1
AFAP1	1	1	1	54.41678	71.52528	1	1	32.55437	1	1	125.7414	1	1	110.6147	91.65025	1	114.509	28.78612	1	172.671
AFAP1L1	1	1	1	1	1	1	1	1	1	1	1	1	1	1	1	1	1	208.0862	1	1
AFAP1L2	1	1	82.59167	1	1	1	1	73.20166	1	1	1	1	1	1	1	1	1	46.90137	1	1
AFDN.DT	1	1	1	53.01025	1	1	1	1	48.41824	45.65197	1	1	1	1	1	1	1	1	1	1
AFF2	1	1	1	49.26701	1	1	1	1	1	1	1	1	1	1	1	1	1	1	1	1
AFF3	1	1	1	101.4412	172.4595	30.01545	228.0869	1	365.9081	148.3216	106.4295	93.70915	78.78447	75.55384	276.5118	83.35932	482.7838	1	53.63248	275.3193
AFMID	1	1	42.48404	1	1	1	1	1	1	1	1	1	1	1	1	1	1	19.4397	1	1
AGAP1	1	74.78049	161.8995	1	58.1701	1	1	104.8706	251.8865	30.77624	94.69776	41.52523	25.83359	90.50676	1	1	1	34.83507	1	103.6483
AGAP2	1	1	1	1	1	1	1	1	64.2196	1	1	1	1	1	1	1	1	1	1	1
AGO4	1	31.28867	65.61986	45.94468	47.99614	31.33595	1	1	1	31.45246	1	31.91643	34.02746	69.83057	62.98696	1	1	24.3854	1	1
AGPAT1	1	29.90261	38.79812	52.89185	41.36795	42.59517	1	47.37743	109.1417	52.25094	1	40.1316	34.10668	1	1	1	1	38.38909	1	70.02264
AGPAT2	1	1	1	1	1	1	1	80.31156	1	1	1	1	1	1	1	1	1	1	1	1
AGPAT4	1	1	46.91063	59.7216	51.81024	32.3015	1	1	119.009	29.83109	1	36.63204	23.13423	78.59767	51.57975	1	1	35.4559	103.2596	1
AGPS	1	1	71.74327	1	1	47.38531	1	1	1	32.30156	1	34.63447	28.20962	1	1	1	1	14.40585	1	1
AGRN	1	1	32.33786	1	1	20.69285	1	770.377	1	1	1	1	1	1	1	1	85.09554	107.675	1	103.6483
AGT	1	1	1	1	1	49.40242	1	1	1	1	1	1	84.62812	1	1	1	1	1	1	1
AGTPBP1	1	1	1	1	48.69522	1	1	1	178.0486	1	1	1	1	78.56071	76.37501	1	100.5014	36.19836	1	1
AGTRAP	1	1	1	1	1	1	1	25.82708	1	1	1	1	33.52855	1	1	54.15327	1	21.51238	1	1
AHDC1	1	1	1	106.0377	96.02351	37.23013	1	23.75018	1	46.46821	1	46.14306	67.30007	1	65.71602	1	111.9778	29.41316	72.05567	1
AHI1	1	79.33508	60.67111	1	1	76.69576	1	32.20408	231.6178	69.23606	1	123.4384	112.5922	1	1	155.9808	1	36.29433	1	70.02264
AHNAK	1	1	1	1	1	41.72381	1	69.72666	1	1	1	1	62.73542	1	1	1	1	125.3949	1	1
AHNAK2	1	1	1	1	1	1	1	1	1	1	1	1	1	1	1	1	81.24089	1	1	1
AIF1	2362.712	1	1	1	1	1	1	1	1	1	1	1	1	1	1	1	1	1	1	1
AIF1L	1	94.20524	57.83753	1	1	142.6335	1	1	1	57.7597	1	72.47686	47.45106	1	1	58.21238	1	31.38341	1	1
AIFM1	1	1	44.56408	1	1	1	1	1	1	1	1	1	1	1	1	1	1	1	1	1
AJUBA	1	1	1	1	1	1	1	1	1	1	1	1	39.36097	1	1	1	1	1	1	1
AK1	1	1	1	1	1	1	1	37.32491	1	1	1	1	81.92419	1	1	1	1	1	1	87.58009
AK4	1	54.31511	1	1	1	40.6393	1	1	1	31.7004	1	32.23216	1	1	1	64.39779	1	1	1	1
AK5	1	1	1	1	1	1	1	1	1	33.67346	1	1	1	1	1	1	1	1	1	1
AK6	61.86628	69.59393	49.45573	1	1	77.18138	1	65.44224	1	56.60121	1	65.90122	68.17168	1	1	105.8618	1	42.54806	1	1
AK9	1	1	1	1	1	1	1	1	1	1	1	30.31043	37.64786	1	1	1	1	1	1	1
AKAP1	1	1	30.55414	1	1	1	1	21.92701	75.71587	1	1	1	1	1	1	1	1	27.73582	1	1
AKAP5	1	1	1	1	1	1	1	1	1	1	1	1	1	222.3155	1	1	1	1	1	1
AKAP6	1	1	43.09375	75.35418	77.56531	72.92839	1	1	117.454	1	138.7919	30.87094	53.74415	126.7147	47.6669	89.00404	123.9713	1	79.9292	1
AKAP7	1	58.38093	66.35514	1	1	43.04288	1	1	329.4496	1	1	34.03501	1	179.244	69.33811	1	86.98704	1	1	70.02264
AKR1C1	1	1	1	1	1	1	1	1	1	1	1	1	1	97.56434	1	1	1	1	1	70.02264
AKR1C3	1	1	1	1	1	48.21264	1	1	1	1	1	1	1	1	1	1	1	1	1	1
AKT1S1	1	1	33.38479	1	1	27.02645	1	1	1	1	1	1	1	1	1	1	1	1	1	1
AL021368.	1	1	1	59.47931	44.92193	26.48975	1	1	1	44.98809	1	23.65134	1	1	1	1	1	1	1	1
AL031777.	1	1	1	1	1	1	1	1	1	1	1	43.72813	1	1	1	1	1	1	1	1
AL096711.	1	1	1	44.83139	51.72996	1	1	1	138.733	1	1	1	1	1	68.99346	1	1	1	1	1
AL117339.	1	1	1	1	1	27.94707	1	1	1	1	1	1	1	1	1	1	1	1	1	1
AL118516.	1	1	1	1	1	1	1	71.75799	1	1	1	1	1	1	1	1	1	51.98399	1	1
AL121603.	1	39.38316	1	1	1	1	1	1	1	1	1	1	1	1	1	1	1	1	1	1
AL139393.	1	1	1	1	1	31.94323	1	1	1	1	1	34.99413	1	1	1	1	1	1	1	1
AL161772.	1	43.03124	1	1	1	62.54909	1	1	1	1	1	1	1	1	1	1	1	1	1	1
AL162231.	1	1	1	1	1	26.56118	1	1	1	29.31897	1	28.64969	1	1	1	1	1	1	1	1
AL355338.	1	1	1	1	1	1	1	80.15211	1	1	1	1	1	1	1	1	1	16.69977	1	1
AL365361.	1	1	1	1	1	1	1	1	1	1	1	1	1	88.71241	1	1	1	1	1	1
AL391807.	1	48.85533	78.22686	177.288	98.42052	101.1458	1	1	1	121.9205	97.73606	65.61644	79.35033	1	61.44549	148.5619	137.2598	1	136.5756	1
AL450998.	1	1	1	1	1	1	1	1	1	1	1	31.07803	1	1	55.12994	1	1	22.02827	1	103.6483
AL589740.	1	56.35526	1	198.8886	132.711	98.48988	152.8329	1	1	269.0557	1	127.0362	38.68422	1	58.11736	100.3666	102.3157	1	289.0242	70.02264
AL683813.	1	1	1	1	44.35068	33.64692	1	1	1	1	1	1	30.28837	1	1	1	1	1	1	1
AL691432.	1	38.68979	1	1	1	35.24313	1	1	1	29.46381	1	1	30.36718	1	1	1	1	1	1	103.6483
ALAS1	93.07284	1	1	1	1	25.50614	1	41.88924	1	31.19721	1	25.89243	1	1	51.99199	1	1	25.43907	1	1
ALCAM	1	1	133.1823	1	1	35.06308	1	1	1	1	1	1	1	1	1	1	1	1	1	1
ALDH18A1	1	39.81347	44.50697	1	1	1	1	1	1	34.75463	1	26.63612	29.00274	1	1	41.13817	1	1	1	1
ALDH2	293.5203	72.36011	61.28183	85.67344	84.26091	125.2123	173.8759	126.6239	1	43.88624	86.06268	46.54659	84.29983	68.4904	60.14442	121.505	1	99.43253	36.83479	1
ALDH5A1	1	1	1	1	1	27.62001	1	1	1	1	1	1	1	1	1	1	1	1	1	1
ALDH6A1	1	98.13172	54.45608	1	1	128.8585	1	1	1	48.02598	1	58.5231	74.01951	1	1	65.00762	1	1	1	1
ALDOC	1	1	1	1	1	153.736	1	1	1	1	1	1	39.09523	1	1	1	1	1	1	1
ALG8	1	1	1	1	1	31.31451	1	1	1	50.88962	1	39.88272	25.86742	1	1	1	1	1	1	1
ALG9	1	1	1	1	1	1	1	52.82634	1	1	1	1	1	1	1	1	1	1	1	1
ALKBH4	1	35.52864	1	1	1	26.1085	1	1	1	30.32492	1	1	1	1	1	1	1	1	1	1

ALMS1	1	1	1	1	1	31.93624	1	1	1	48.85341	1	51.52975	47.79853	1	1	1	1	1	1	1
ALOX5AP	308.5031	1	1	1	1	1	1	1	1	1	1	1	1	1	1	1	1	1	1	1
ALPK3	1	1	1	1	1	1	1	1	1	1	1	1	1	1	1	1	1	54.68128	1	1
ALPL	1	1	1	1	1	1	1	1	1	1	1	1	1	1	1	1	1	74.34322	1	1
AMDHD2	63.79826	1	1	1	1	1	1	1	1	1	1	1	1	1	1	1	1	1	1	1
AMOTL1	1	1	1	65.20475	54.84638	1	1	226.3214	1	52.45186	1	42.96252	32.4698	1	1	1	1	67.83355	58.13973	1
ANAPC15	1	31.18482	55.92015	1	1	22.61546	1	1	71.60545	47.26289	1	47.79167	1	77.4805	62.85844	1	1	1	59.68702	1
ANAPC4	1	1	1	1	1	24.59921	1	1	1	1	1	1	1	1	1	1	1	111.7717	1	1
ANAPC7	1	34.61869	43.73129	1	41.0747	38.75647	1	1	1	44.44591	1	52.27002	42.71065	1	1	1	1	1	161.1678	1
ANGEL2	1	33.06615	25.24082	1	41.67423	40.39677	1	1	1	53.61973	1	44.32891	31.79155	1	1	80.27033	30.95524	25.80146	1	103.6483
ANGPT1	1	1	1	1	1	1	1	1	1	1	1	1	32.34033	1	1	1	1	1	1	1
ANGPT2	1	1	1	1	1	1	1	174.811	1	1	1	1	1	1	1	1	1	138.9854	1	70.02264
ANGPTL1	1	34.22415	1	1	1	89.92199	1	1	1	44.7022	1	43.07389	52.9081	1	1	71.54449	1	1	1	1
ANGPTL2	1	1	134.8303	1	1	1	1	1	1	1	1	1	1	1	1	1	1	1	1	1
ANGPTL4	1	1	1	1	1	1	1	305.246	1	1	1	1	1	1	1	1	1	29.20301	1	1
ANK2	1	175.3804	1	221.6278	402.3096	205.2729	439.6832	1	441.4692	190.4254	371.0374	144.8877	117.8867	225.9847	615.8558	161.2309	830.8199	1	260.1626	308.945
ANK3	1	1	172.8869	204.7753	157.7021	26.61522	1	1	869.0242	35.11111	238.2495	39.82964	1	175.2687	1	1	408.1992	1	63.27439	277.0906
ANKH	203.1612	1	47.40687	78.97507	61.87757	1	1	36.66475	129.1587	27.42354	79.37296	1	1	1	66.1921	1	373.527	75.79114	1	70.02264
ANKHD1	1	1	1	1	1	1	1	1	1	32.90996	1	24.05677	1	1	1	1	1	21.44912	1	1
ANKLE2	1	58.72038	51.68466	82.51162	75.06487	59.69058	1	76.11702	157.2775	97.46533	97.57391	74.97935	66.32824	94.85528	54.15078	67.92835	1	75.87565	84.58561	208.0679
ANKMY2	1	1	1	1	45.9844	28.33432	1	1	165.3598	1	1	1	1	1	1	1	1	1	1	1
ANKRD13B	1	1	37.24087	1	47.13482	1	1	1	1	1	1	1	1	1	42.50876	1	71.82142	1	1	1
ANKRD13C	1	32.71137	1	1	1	37.52695	1	1	1	1	1	29.07553	23.63673	1	1	1	1	1	1	1
ANKRD28	1	47.6088	78.63281	1	45.72113	33.59318	1	49.85433	71.56111	33.42456	69.32899	39.01132	26.12192	70.34016	1	1	1	35.27222	1	1
ANKRD29	1	1	1	1	1	28.44243	1	1	1	1	1	1	1	1	1	1	1	1	1	1
ANKRD33B	1	1	1	1	1	1	1	1	185.6929	1	1	1	1	1	1	1	1	1	1	1
ANKRD35	1	35.35956	1	1	1	47.70599	1	1	1	1	1	29.2067	26.55945	1	1	1	1	1	1	1
ANKRD36B	1	1	1	63.24281	70.08181	31.62292	1	1	99.31413	31.0126	105.6765	30.61147	35.66945	65.11852	47.34977	1	108.7124	1	71.00875	1
ANKRD39	1	1	32.44392	1	41.85977	1	1	1	1	1	1	22.35924	1	1	1	1	1	44.92338	1	1
ANKRD40	1	49.34525	1	1	1	35.93027	1	43.15593	1	32.71609	1	36.82506	41.14822	1	1	1	108.1851	59.91125	1	1
ANKRD42	1	1	1	1	1	1	1	1	1	1	1	1	40.94741	1	1	1	1	1	1	1
ANKRD44	57.22642	1	89.96918	69.38123	55.64943	28.44463	1	68.71068	79.27651	50.37529	1	1	1	1	62.35808	1	159.42	20.81596	41.6468	70.02264
ANKRD54	1	1	33.15443	1	1	1	1	1	1	1	1	25.62436	51.7947	1	1	1	1	1	1	1
ANKRD9	1	33.76379	1	1	1	101.7421	1	54.36693	1	29.16248	1	41.93084	33.77378	1	1	45.69978	1	1	1	1
ANKS1B	1	1	162.6717	119.7407	167.5831	65.05838	1	1	213.501	41.91966	290.1051	39.2906	46.74467	73.02313	186.6135	104.5192	360.5066	1	81.59878	103.6483
ANKS3	1	36.16119	1	50.74086	46.16974	28.17756	1	1	69.98172	33.32361	1	1	24.05261	1	1	108.7962	205.3337	1	133.4259	70.02264
ANKZF1	1	1	1	50.23352	45.28955	23.54924	1	1	1	35.87594	1	1	35.45391	1	62.64167	1	1	1	53.01477	139.0453
ANLN	1	1	1	1	1	29.75014	1	1	1	1	1	78.67711	26.21626	1	1	1	1	1	1	1
ANOS1	1	1	1	1	1	66.81235	1	1	1	1	1	1	88.1316	1	1	1	1	1	1	1
ANP32E	97.71369	174.3503	142.7356	53.64329	1	158.4456	1	83.22464	1	168.2799	1	494.9675	134.7842	1	1	123.5182	1	53.98871	197.3845	103.6483
ANPEP	1	1	1	1	1	1	1	52.22409	1	1	1	1	1	1	1	1	1	1	1	1
ANTXR1	1	71.27238	71.88101	48.72403	1	29.1062	1	51.53458	1	58.37825	1	32.06016	46.96001	1	1	1	1	1	1	103.6483
ANXA1	1	1	1	1	1	38.48081	1	317.8461	1	1	1	1	407.1035	1	1	1	1	465.7356	1	1
ANXA11	90.76917	1	1	1	1	31.31326	1	46.09584	1	1	1	1	1	1	1	1	1	38.18547	1	1
ANXA2	1	1	1	1	1	110.0303	1	185.1795	1	1	1	48.27213	186.3837	1	1	135.7915	1	20.44228	1	70.02264
ANXA2R	1	1	1	1	1	1	1	1	1	1	1	1	1	1	1	1	1	50.10692	1	1
ANXA4	159.5872	1	1	1	1	1	1	1	1	1	1	1	1	1	1	1	1	1	1	1
ANXA5	639.429	365.9161	107.0124	1	1	322.1996	1	250.2625	1	130.5771	1	226.0502	277.0636	1	1	324.9024	1	397.7886	1	103.6483
ANXA6	1	66.2218	133.339	1	1	145.3969	1	141.3603	1	54.21617	1	58.4556	73.2696	1	1	84.95805	1	1	1	1
AP000459.	1	1	1	1	1	1	1	1	1	1	1	1	1	1	1	1	1	109.1462	1	1
AP002884.	1	1	1	1	1	1	1	1	73.46287	1	60.82255	1	1	78.78113	1	1	39.66949	1	1	87.58009
AP003481.	209.2047	1	1	1	1	1	1	1	1	1	1	1	1	1	1	1	1	1	1	1
AP1AR	1	38.78919	1	69.27246	85.7317	33.64807	150.729	25.73782	46.4122	69.11936	1	50.8185	32.13069	74.78392	83.59655	1	1	1	71.87739	172.671
AP1B1	161.9214	1	1	1	1	1	1	1	1	1	1	1	1	1	1	1	1	25.99715	1	1
AP3B2	1	1	1	1	37.84991	1	1	1	1	1	1	1	1	1	1	1	1	1	1	275.3193
AP3M1	1	52.87064	41.43971	50.68193	44.75071	43.37672	1	21.07923	1	65.65513	1	51.66581	48.12724	1	1	1	1	1	1	1
AP3M2	1	1	38.09885	1	1	1	1	57.98827	1	1	1	28.65167	32.39368	1	1	1	1	36.71429	69.25791	1
AP4M1	1	1	1	1	1	26.16316	1	1	1	1	1	34.93771	26.17863	1	62.17926	1	1	1	1	87.58009
AP5M1	1	1	1	1	1	33.99962	1	1	1	36.63543	1	38.56089	32.83934	1	1	68.25588	1	23.2082	1	1
APBA1	1	1	1	1	1	1	1	1	91.31945	1	1	1	1	1	1	1	1	1	1	1
APBB1IP	413.4927	1	1	1	1	1	1	1	1	1	1	1	1	1	1	1	1	1	1	1
APBB2	1	86.14834	124.6688	1	1	121.7752	1	83.05043	1	1	1	44.11533	55.385	138.025	1	98.25934	1	47.41061	1	1
APCDD1	1	39.43876	84.28695	1	1	33.21069	1	1	1	1	1	1	26.40533	1	1	1	1	1148.723	1	1
APEH	1	1	1	1	1	38.40561	1	27.56586	1	1	1	30.52082	29.97674	1	1	1	39.96312	1	1	1
APLN	1	1	1	1	1	1	1	1	1	1	1	1	1	1	1	1	1	206.3845	1	1
APLNR	1	1	1	1	1	1	1	59.33745	1	1	1	1	1	1	1	1	1	170.8812	1	1
APLP1	1	1	147.9249	170.1593	220.0218	44.70135	227.0737	1	275.9967	56.4657	174.2371	82.5664	51.49518	165.6462	227.2261	62.90104	163.5612	1	175.9924	323.568
APOC1	2205.302	1	1	1	1	1	1	1	1	1	1	1	1	1	1	1	1	1	1	1
APOD	1	1	463.0884	1	1	1	1	1	1	1	1	1	1	1	1	1	1	1	1	1
APOE	6680.035	1	1	1	1	426.8024	1	2405.378	1	1	1	1	1	1	1	1	1	28.63361	1	1
APOL4	1	1	1	1	1	1	1	1	1	1	82.49315	1	1	1	1	1	1	52.71507	1	1
APOLD1	1	1	1	1	1	1	1	229.0362	1	1	1	51.1327	1	1	1	1	1	555.8085	1	1
APOO	1	35.88988	56.02912	1	43.04504	42.34611	1	1	1	38.54008	1	42.45433	42.80415	1	65.88316	68.9873	1	1	1	103.6483
APRT	187.9901	116.0396	154.2361	74.03946	87.09632	111.8868	140.3331	130.0385	1	103.8546	1	56.46094	119.7711	1	100.2467	129.1574	1	141.0563	1	1
AQP4	1	195.3832	1	1	1	216.3196	1	1	1	1	1	1	315.7271	1	1	136.4298	1	1	1	1
ARAP2	1	47.19593	1	1	1	53.27597	1	1	1	1	1	34.23633	34.42623	1	1	50.16086	1	32.83726	1	1
ARAP3	1	1	1	1	1	1	1	1	1	1	1	1	1	1	1	1	1	47.42377	1	1
ARC	1	205.787	181.0991	1	65.94441	126.2767	1	1	1	53.83399	1	37.83464	157.8655	1	1	115.7086	1	40.77604	1	1
ARFGEF3	1	1	85.76585	1	1	1	1	1	180.5551	1	1	1	1	1	82.97021	1	173.6613	29.91925	1	208.0679
ARG2	1	1	37.63112	1	40.51512	1	1	48.83545	1	1	1	1	1	1	73.12076	1	1	1	1	103.6483
ARHGAP10	1	1	1	1	1	1	1	63.59973	1	1	1	1	1	1	1	1	1	1	1	1
ARHGAP11	1	1	1	1	1	1	1	1	1	1	1	173.4133	1	1	1	1	1	1	1	1
ARHGAP12	131.1204	87.35264	59.10986	1	1	84.1798	1	35.06083	1	45.07737	63.80565	35.88921	58.02617	1	1	1	1	31.46687	1	1
ARHGAP15	1	1	1	1	1	1	1	48.12677	1	1	1	1	1	1	1	1	1	1	1	1
ARHGAP18	1	1	1	1	1	37.26562	1	1	1	1	1	1	1	1	1	1	1	422.2718	1	1
ARHGAP19	1	1	1	1	1	1	1	1	1	1	1	26.27476	1	1	1	1	1	1	1	1
ARHGAP20	1	1	1	1	1	1	1	1	99.93116	1	1	1	1	1	1	1	1	1	1	1
ARHGAP23	1	1	1	1	1	1	1	1	101.766	1	1	1	1	64.25867	1	1	133.5289	25.46887	1	1
ARHGAP25	48.23546	1	1	1	1	1	1	1	1	1	1	1	1	1	1	1	1	1	1	1
ARHGAP26	57.46948	1	1	1	1	1	1	108.7355	1	1	1	1	1	1	1	1	1	41.66356	1	1
ARHGAP29	1	1	1	1	1	1	1	700.395	1	1	1	1	1	1	1	1	1	520.0088	1	1
ARHGAP32	1	53.81147	38.78278	53.79778	53.03953	44.72338	1	29.04285	93.37256	31.79055	82.85509	31.30293	41.31193	82.5115	1	1	1	1	34.62764	1
ARHGAP33	1	1	46.52996	58.0443	72.05332	1	1	1	119.2567	48.63162	71.2354	104.8042	28.37094	89.29809	104.0296	1	118.6976	1	1	1
ARHGAP42	1	1	36.01394	1	1	1	1	327.7468	1	29.54972	1	1	1	1	1	1	1	1	1	1
ARHGAP44	1	1	1	1	1	1	1	31.35962	109.5452	1	1	1	1	1	1	1	1	1	1	206.2967
ARHGDIB	659.3999	1	1	1	1	1	1	261.6587	1	1	1	1	1	1	1	1	1	150.2185	1	1
ARHGDIG	1	1	1	1	1	1	1	1	122.836	1	1	1	1	1	1	1	1	1	1	1
ARHGEF15	1	1	1	1	1	1	1	1	1	1	1	1	1	1	1	1	1	51.91929	1	1
ARHGEF17	1	1	1	1	1	1	1	194.386	1	1	1	1	21.92026	1	1	1	1	1	1	1
ARHGEF2	1	46.3093	49.4433	90.49394	84.31743	1	1	1	1	86.1353	112.2111	54.12489	29.77837	109.9585	71.27745	1	1	45.66958	93.3031	103.6483
ARHGEF25	1	1	1	1	1	1	1	1	1	37.88323	1	1	1	1	1	1	1	1	1	1

ARHGEF26	1	98.17039	52.21467	1	1	37.77421	1	1	1	1	1	38.60459	33.74546	1	1	1	1	1	1	1
ARHGEF39	1	1	1	1	1	1	1	1	1	1	1	38.62793	1	1	1	1	1	1	1	1
ARHGEF4	1	1	76.55798	1	1	1	1	1	1	1	1	1	1	1	1	1	1	1	1	1
ARHGEF40	1	54.81848	1	1	1	50.83678	1	135.3658	1	1	1	43.11755	47.80413	1	1	60.24803	1	1	1	1
ARHGEF6	1	66.04218	1	1	1	70.26666	1	88.67159	1	1	1	37.05095	37.37976	1	1	45.71304	1	1	1	1
ARID3A	53.06812	1	1	1	1	1	1	31.6967	1	1	1	1	1	1	1	1	1	63.99006	1	1
ARID5A	88.72945	1	1	1	1	40.71632	1	75.67607	1	1	1	1	68.94661	1	1	1	1	92.58252	1	1
ARID5B	1	94.76513	134.418	1	1	45.21358	1	249.7221	1	1	1	1	50.74489	92.81004	1	1	1	1	1	1
ARL13B	1	41.83918	1	1	1	56.94975	1	69.49049	1	33.71309	1	56.73362	72.41324	68.30235	1	93.56933	1	58.06161	1	1
ARL15	1	1	1	1	1	1	1	1	1	1	1	1	1	1	1	1	1	63.50438	1	1
ARL17A	1	1	1	1	1	1	1	1	76.15131	1	1	1	1	1	1	1	1	1	80.65116	103.6483
ARL17B	1	1	1	1	1	1	1	1	83.45303	1	1	1	1	1	1	1	1	1	1	1
ARL4A	54.62765	132.452	508.389	1	1	45.72699	1	146.8106	1	1	93.74231	65.35598	82.49953	1	1	1	67.15728	273.9454	1	70.02264
ARL4C	223.3715	332.2365	99.613	793.4961	803.3426	166.969	324.1462	1	206.2245	399.0824	1497.904	272.0475	448.0353	1568.101	246.195	205.9268	355.4772	167.7679	484.5561	103.6483
ARL4D	1	1	1	74.90274	116.1136	36.14906	1	83.91533	77.55411	46.49617	142.2951	48.40971	1	640.4621	70.66824	1	1	30.27463	88.39048	139.0453
ARMC3	1	1	1	1	1	1	1	1	1	1	1	1	84.74629	1	1	1	1	1	1	1
ARMCX5	1	1	1	1	1	1	1	1	1	1	1	1	1	1	1	1	50.01609	1	1	70.02264
ARNT2	1	39.19241	52.97541	43.74134	1	49.6581	1	1	1	42.66751	98.78088	39.18771	42.92564	60.41628	1	1	1	20.81037	43.9366	1
ARPC1B	274.6492	1	1	1	1	1	1	1	1	1	1	1	1	1	1	1	1	59.12532	1	1
ARPIN	1	1	1	1	1	1	1	1	1	30.97931	1	24.11226	29.02808	1	1	1	1	1	1	1
ARPP21	1	1	129.2329	64.26472	167.055	1	188.6919	1	1335.133	1	1	1	1	1	299.606	1	1088.085	1	95.86552	599.6586
ARRB2	287.8138	32.58512	40.98876	1	44.23581	1	1	1	1	66.64654	1	42.43703	30.30105	1	1	1	1	1	1	103.6483
ARRDC4	1	1	1	1	1	35.19735	1	46.36402	1	1	76.47531	1	46.51573	1	1	1	1	1	1	1
ARSD	1	1	1	1	1	48.03657	1	1	1	1	1	1	30.7525	1	1	1	1	17.59719	1	1
ARV1	1	1	1	1	1	31.51866	1	1	1	1	1	1	1	1	1	1	1	1	1	1
ARX	1	156.2828	1	1	1	249.6561	1	1	1	92.31157	682.5054	127.6583	193.4978	1093.871	1	167.7843	1	1	1	1
ASB1	1	1	1	1	1	1	1	1	1	25.24302	1	1	1	1	1	1	1	1	1	1
ASB3	1	1	87.26029	1	1	30.03949	1	1	1	32.48103	1	28.11539	25.20675	1	1	1	1	1	1	1
ASCC2	1	1	1	1	1	1	1	1	1	29.24303	1	1	1	1	1	1	160.3666	14.39737	73.15477	1
ASCL1	1	485.9509	234.7939	108.4509	1	103.4425	1	1	1	385.9087	177.9419	322.9805	268.3816	179.6394	1	174.2476	1	1	1	1
ASF1B	1	1	1	1	1	1	1	1	1	31.11292	1	53.17853	1	1	1	1	1	1	1	1
ASGR1	1	1	42.59237	1	51.53305	26.2481	1	23.43134	1	1	65.46382	1	23.51519	89.45371	72.41233	1	1	28.04877	1	103.6483
ASH2L	1	37.60497	30.77197	1	1	41.50642	1	34.50592	1	55.16975	1	45.97985	32.10643	1	77.4154	53.29996	1	22.20189	1	1
ASIC1	1	72.04359	110.4102	68.74659	47.75531	25.25261	1	1	100.9458	47.73813	65.02722	1	1	1	69.96093	1	86.08842	1	1	1
ASIC4	1	1	82.39823	1	1	1	1	1	1	1	1	1	1	1	1	1	1	1	1	1
ASMTL	1	1	1	1	1	28.04775	1	35.15501	1	1	1	1	1	1	1	1	68.69449	24.65827	1	1
ASNS	1	1	1	69.89214	101.8439	1	1	1	87.7476	39.05559	1	26.58979	1	64.43777	86.77299	1	1	1	82.92124	116.5001
ASPHD1	1	1	1	1	50.66734	1	1	1	1	1	1	1	1	1	1	1	1	1	1	70.02264
ASPHD2	1	1	1	1	1	1	1	1	155.8056	1	1	1	1	1	1	1	1	1	1	1
ASPM	1	1	1	1	1	1	1	1	1	44.47015	1	609.5981	1	1	1	73.06364	1	1	1	1
ASPN	1	1	1	1	1	1	1	158.8793	1	1	1	1	1	1	1	1	1	1	1	1
ASTN1	1	1	65.03019	1	1	47.73985	1	1	79.17615	1	1	1	1	68.84241	1	1	1	1	1	1
ASXL3	1	38.08564	1	125.8916	146.803	1	148.9642	18.56726	93.05007	45.00644	172.5171	30.94688	1	197.3229	167.3486	1	156.0549	1	86.1144	70.02264
ATAD2	1	58.94652	52.42237	1	1	59.27087	1	42.61796	1	186.6315	1	230.9605	90.77048	1	1	74.66186	1	1	1	1
ATAD5	1	38.00979	1	1	1	1	1	1	1	114.2632	1	131.1629	36.10277	77.99764	1	76.52236	1	1	1	1
ATCAY	1	1	484.9997	231.8405	247.109	1	246.3872	1	159.8757	39.24832	305.992	45.19192	1	296.8332	178.6194	1	325.9045	1	297.4643	149.721
ATE1	1	46.74852	52.65214	45.39945	1	34.75448	1	44.98682	1	56.70708	1	42.66084	33.30981	1	1	1	1	1	1	1
ATF3	406.5044	180.4702	1	1	1	254.2725	1	704.412	1	50.34186	1	86.68247	442.3891	1	1	104.7279	1	90.54318	1	1
ATF6	1	34.14065	1	45.89265	1	35.11189	1	38.10843	1	31.25528	1	37.33483	33.8569	1	68.32032	1	1	64.17226	1	1
ATF7	1	45.39633	1	58.27389	57.68227	55.0116	1	43.89404	1	50.24457	1	35.88017	34.47847	1	1	1	1	114.9798	1	1
ATG10	1	1	1	1	1	30.83466	1	23.436	1	47.76496	1	35.29031	1	1	1	1	1	11.49109	1	1
ATG14	1	32.72592	1	1	1	29.38128	1	1	1	1	1	1	23.7283	1	1	1	1	1	1	1
ATG2B	1	1	67.45037	1	1	1	1	1	1	1	1	1	1	1	1	1	1	1	1	1
ATG5	1	44.48508	40.39573	1	43.43641	37.26595	1	38.84381	1	45.55473	1	39.02737	34.66994	70.14709	1	1	87.65146	34.76245	99.52943	1
ATIC	1	45.19412	38.55335	1	1	32.89361	1	1	1	46.12552	1	34.15757	34.5416	1	57.26939	1	1	1	51.75377	1
ATP10D	1	1	1	1	1	39.15841	1	54.8539	1	1	1	1	1	1	1	1	1	1	1	1
ATP11C	1	1	1	1	47.96154	1	1	1	1	1	1	34.65274	1	1	66.03244	1	1	1	1	103.6483
ATP13A2	1	1	49.38552	1	48.0387	30.0813	1	1	94.69543	1	1	1	1	1	1	1	162.2347	24.16047	1	206.2967
ATP1A2	1	250	63.42418	1	1	479.3461	1	1383.863	1	1	1	57.19158	162.3035	1	1	223.0146	1	1	1	1
ATP1A3	1	1	1	1	45.59606	1	1	1	256.6035	1	1	1	1	1	68.22081	1	130.0208	1	1	1
ATP1B2	1	326.0667	72.33651	47.69329	42.43548	472.6496	1	211.6679	71.56077	1	1	85.20419	181.0486	1	1	282.6938	107.7816	1	1	1
ATP2B1.AS	51.74056	1	1	1	1	1	1	1	1	1	1	1	1	1	1	1	1	1	1	1
ATP2B2	1	1	1	1	1	1	1	1	403.767	1	1	1	26.22556	1	1	1	208.0263	1	1	1
ATP6V0A2	1	1	43.24901	1	1	1	1	1	61.20562	43.49801	1	24.70346	33.04116	1	60.00966	1	57.5548	15.90075	1	1
ATP6V1E1	82.51189	1	47.28561	57.89741	50.04594	35.92595	1	62.86962	69.53513	53.43082	1	52.7833	43.18967	1	1	1	1	115.3587	35.03598	70.02264
ATP6V1G2	1	1	46.76828	87.00619	108.9334	1	145.6391	1	94.88925	1	1	1	1	1	98.76834	1	67.86691	1	200.671	139.0453
ATP8A1	1	1	53.26646	1	1	1	1	1	188.2498	1	105.6074	1	1	62.19089	1	1	103.1347	1	1	1
ATP8A2	1	1	1	1	1	1	1	1	140.9419	1	1	1	1	1	1	1	220.9906	1	60.95044	1
ATP8B1	1	1	1	1	1	1	1	51.83469	1	1	1	1	1	1	1	1	1	1	1	1
ATP8B2	1	1	1	1	1	1	1	1	1	1	1	1	27.94947	1	1	1	1	1	1	1
ATP9B	1	1	1	1	1	1	1	1	1	37.89571	1	1	1	1	1	1	1	1	1	1
ATRAID	119.8822	119.9381	108.9947	54.89206	50.83692	140.4474	1	140.9262	1	96.70229	1	106.6215	134.8331	1	55.13084	97.12063	1	106.6895	1	116.5001
ATRN	1	1	1	1	1	35.51601	1	30.04997	63.80184	1	1	1	25.7617	1	71.29615	1	1	1	1	1
ATRNL1	1	1	1	1	1	1	1	1	63.63952	1	1	1	1	180.7738	1	1	1	1	1	1
ATXN7L1	1	1	1	1	1	1	1	1	1	1	1	1	1	1	1	1	85.48973	1	1	1
ATXN7L3	1	1	1	1	1	1	1	1	1	1	1	1	1	1	59.98713	1	1	23.52804	1	1
AUP1	77.36498	62.40821	71.13847	44.15586	56.90888	61.28307	1	107.8192	73.32835	70.54216	56.81159	59.52715	49.83269	1	56.65837	85.05505	1	75.12373	1	103.6483
AURKA	1	1	1	1	1	1	1	1	1	1	1	132.2498	1	1	1	1	1	1	1	1
AURKB	1	1	1	1	1	1	1	1	1	1	1	154.8684	1	1	1	1	1	1	1	1
AVL9	1	1	1	46.06047	49.12368	30.28052	1	1	1	27.83473	1	25.93575	26.78982	1	41.9669	1	1	28.37735	56.91457	1
AVPR1A	1	1	1	1	1	1	1	174.3778	1	1	1	1	1	1	1	1	1	1	1	1
AXL	90.85705	1	1	1	1	53.02465	1	129.7913	1	1	1	1	1	1	1	1	1	1	1	1
B2M	2754.637	797.5194	251.2001	174.6874	71.9302	627.1807	1	2602.933	1	269.2246	68.67705	437.9919	630.2876	82.67564	1	436.2258	1	2057.751	1	1
B3GALNT2	1	38.59819	1	50.98463	1	57.56254	1	27.74594	1	72.26323	1	49.86196	71.01088	1	1	105.828	1	1	1	1
B3GALT1	1	1	1	1	60.77299	1	1	1	1	1	1	1	1	1	76.70904	1	1	1	57.11219	241.6936
B3GALT2	1	1	1	1	40.06447	42.37465	1	1	264.0722	31.37269	1	1	1	1	70.36875	1	1	1	1	1
B3GALT6	1	36.2164	1	1	1	32.15353	1	44.13569	1	30.78105	1	29.87884	35.04675	1	60.15975	1	98.31935	118.73	1	1
B3GAT1	1	56.46169	99.17783	1	1	52.69386	1	1	174.6965	1	1	30.7465	26.58264	1	1	1	1	1	1	1
B3GAT2	1	91.04595	165.0099	1	1	281.3125	1	1	1	1	1	77.26044	42.382	1	1	210.4411	1	1	1	1
B3GLCT	1	37.22427	1	1	1	1	1	1	1	1	1	1	1	1	1	1	1	1	1	1
B3GNT2	1	1	1	1	1	1	1	90.02594	1	1	1	1	1	1	1	1	1	44.71876	1	1
B3GNT5	121.7705	34.59777	1	1	1	1	1	1	1	36.98193	1	1	48.2127	1	1	1	1	1	1	1
B4GALNT1	1	1	1	1	57.07088	1	1	1	1	32.6276	74.98648	1	1	71.78692	68.25628	1	1	1	1	70.02264
B4GALT1	120.4085	1	1	1	1	1	1	1	1	1	1	1	1	1	1	1	1	21.81076	1	1
B4GALT2	1	38.89338	41.22253	1	46.28398	42.25324	1	61.41833	79.82167	37.66613	1	36.28305	39.05801	1	92.5035	51.5817	1	1	1	1
B4GALT3	1	1	1	1	43.11542	1	1	45.64181	1	1	1	1	1	1	1	1	1	1	1	1
B4GALT6	1	1	38.3262	1	1	1	1	1	1	1	1	1	1	1	1	1	1	1	1	1
B9D1	1	1	1	1	1	1	1	1	1	1	1	1	43.96731	1	1	1	1	1	1	1
B9D2	1	1	1	1	1	1	1	1	1	1	1	1	30.96878	1	1	1	1	1	1	1
BAALC	1	44.8736	391.7146	49.74641	1	58.94617	1	1	74.01093	86.06224	1	57.03065	29.00773	75.66282	1	1	1	36.39289	1	103.6483
BABAM2	1	1	1	1	1	32.22281	1	1	1	1	1	1	27.9367	1	1	1	1	1	1	1

BACE1	1	1	39.0293	1	44.895	34.79138	1	24.99056	63.38594	48.41185	1	31.9086	33.86033	1	60.32792	1	131.8145	1	92.39968	1
BACH2	1	1	1	1	66.83719	40.82815	1	1	306.8474	1	103.439	23.06657	1	113.9266	84.65868	1	57.86844	1	1	1
BAG4	1	1	1	1	47.30424	28.59518	1	25.50959	1	44.35567	1	31.17844	25.2678	57.66601	1	1	1	21.08744	1	70.02264
BAIAP2	1	1	1	1	1	1	1	1	70.90333	1	1	1	47.13635	1	1	1	1	28.87757	1	1
BAIAP2.AS	1	1	1	54.49148	69.4147	1	1	1	1	1	1	1	24.44524	1	64.82329	1	89.35021	1	1	70.02264
BAIAP3	1	1	1	1	1	1	1	1	1	1	1	1	45.93713	1	1	1	1	1	1	1
BAMBI	1	1	188.8376	1	1	1	1	127.7789	1	1	1	1	1	1	1	1	1	63.49594	1	1
BARD1	1	115.8543	77.21591	1	1	73.43765	1	22.07067	1	139.5027	1	163.8086	78.51791	1	1	95.7485	1	24.28249	1	1
BBC3	1	1	1	1	48.20827	1	1	29.05978	1	1	1	1	1	1	1	1	1	79.02378	1	70.02264
BBOX1	1	69.93436	1	1	1	34.41178	1	1	1	1	1	1	44.74278	1	1	1	1	1	1	1
BBS2	1	45.21666	1	1	1	61.67215	1	1	1	29.98562	1	25.65079	29.3866	1	1	1	1	1	1	1
BBS9	1	1	1	1	1	1	1	1	1	1	1	1	1	1	1	1	1	49.30521	32.51665	1
BCAM	1	1	1	1	1	1	1	35.45377	1	1	1	1	1	1	1	1	1	62.199	1	1
BCAN	1	1275.814	1785.321	1	1	729.1504	1	1	1	1	1	107.9793	458.0983	1	1	290.977	1	1	1	1
BCAS1	1	1	702.4759	1	1	1	1	1	1	1	1	1	1	1	1	1	1	1	1	1
BCAS3	1	1	1	1	1	1	1	76.25676	1	1	1	1	1	1	1	1	1	1	1	1
BCAS4	1	1	1	1	1	1	1	1	1	1	1	32.65382	1	1	1	1	1	1	1	1
BCAT1	1	1	95.24453	1	1	1	1	1	1	1	1	1	1	1	1	1	1	73.89333	1	1
BCHE	1	110.5124	304.9984	1	1	1	1	129.9456	1	1	1	1	1	1	1	1	1	1	1	1
BCKDHB	1	1	1	1	1	37.30633	1	1	1	1	1	1	43.32129	1	1	1	1	20.03443	1	1
BCL10	1	39.85457	30.76051	60.93407	60.4354	44.10842	1	1	1	36.12653	96.31413	42.84423	36.61276	104.6436	64.59542	1	1	43.0295	1	1
BCL11B	1	1	1	117.7607	88.89938	1	1	1	327.3975	1	386.3849	1	1	583.9471	1	1	1	1	123.8512	1
BCL2	1	1	1	1	1	31.73596	1	1	96.95877	1	1	22.7429	36.10389	1	60.96389	1	147.0887	108.9575	1	1
BCL2A1	502.2837	1	1	1	1	1	1	1	1	1	1	1	1	1	1	1	1	1	1	1
BCL2L1	84.19376	1	1	47.53055	1	1	1	1	1	33.92067	1	1	1	1	1	1	1	116.2645	1	1
BCL2L11	1	1	1	1	1	1	1	1	1	1	1	1	1	1	1	1	1	57.97764	1	1
BCL6	1	1	1	1	1	1	1	42.61089	1	1	1	1	58.3211	1	1	1	146.4164	1	1	70.02264
BCL6B	1	1	1	1	1	1	1	1	1	1	1	1	1	1	1	1	1	74.46352	1	1
BCL7B	1	56.3039	33.97843	1	43.61202	32.65454	1	59.64926	1	57.49111	1	43.54362	45.04882	65.77311	1	1	1	40.0473	1	1
BCL9	1	1	1	53.43838	55.76218	37.98322	1	1	1	1	1	34.51864	1	89.40285	41.14194	1	65.28482	1	70.44156	206.2967
BCL9L	1	1	1	58.40966	58.74539	25.43176	1	32.80534	71.82467	38.81809	1	37.65435	39.86108	1	1	1	1	29.88613	1	1
BCLAF3	1	32.62698	44.64607	62.32266	59.45263	45.25902	1	1	65.06167	34.25939	57.89677	23.14352	33.38785	75.20585	1	1	1	1	1	70.02264
BCOR	1	1	1	59.09491	57.81056	1	1	37.90663	1	72.43709	105.0719	47.51331	1	97.43706	1	1	1	1	158.4598	70.02264
BDH2	1	56.99761	1	1	1	41.49977	1	1	1	36.73764	1	21.29174	28.67315	1	1	1	1	21.62442	1	1
BEND3	1	1	1	1	1	1	1	1	1	41.88092	1	1	1	1	1	1	1	1	1	1
BEND4	1	1	1	1	1	1	1	1	1	1	1	1	1	142.8486	1	1	1	1	1	70.02264
BEND5	1	1	45.73705	91.82356	90.27976	1	1	1	1	57.35672	93.46191	33.78314	1	125.6077	71.43653	1	1	1	1	70.02264
BEND7	1	70.56659	36.21613	1	1	35.08229	1	1	1	29.77864	1	57.96417	40.94253	1	1	1	1	23.22426	1	1
BEX5	1	1	78.48839	61.11374	127.9888	31.47924	1	1	135.974	1	135.2597	35.52318	38.97279	62.95878	182.7024	1	53.31239	48.0949	91.50017	208.0679
BGN	1	1	1	1	1	1	1	1944.983	1	1	1	1	1	1	1	1	1	180.1506	1	1
BHLHB9	1	1	1	49.97847	54.06925	35.55243	1	1	1	1	1	34.07667	28.46996	1	54.70962	1	1	1	1	139.0453
BHLHE22	1	1	1	231.6342	287.8906	1	537.5797	1	1	52.01089	1	1	1	1	390.8097	1	409.9187	1	154.803	265.2211
BHLHE40	1	1	1	1	1	29.56753	1	69.60352	1	1	1	1	40.76046	1	1	1	1	374.8293	1	1
BHLHE41	404.3134	1	1	1	1	1	1	1	1	1	1	1	39.98538	1	1	1	1	1	1	1
BICC1	1	1	1	1	1	1	1	1	1	1	1	33.81617	38.47299	1	1	1	1	1	1	1
BICDL1	1	1	1	1	1	1	1	1	90.30722	1	1	1	1	1	1	1	1	1	1	1
BICRAL	1	1	1	1	1	1	1	1	1	1	1	1	1	1	1	1	74.56457	1	1	1
BIN1	1336.457	1	83.5095	1	1	1	1	28.40502	35.60582	1	1	1	1	1	1	1	1	79.5932	1	1
BIN2	127.6802	1	1	1	1	1	1	1	1	1	1	1	1	1	1	1	1	1	1	1
BIRC5	1	1	1	1	1	1	1	1	1	32.20151	1	370.459	1	1	1	1	1	1	1	1
BIVM	1	31.97938	61.01663	1	41.09055	27.14308	1	1	1	34.49585	1	27.29796	24.63148	1	65.38008	1	1	11.4509	1	1
BLM	1	1	49.91325	1	1	1	1	1	1	36.00231	1	51.84214	1	1	1	1	1	1	1	1
BLVRB	253.6571	1	1	1	1	54.55683	1	41.91389	1	1	1	1	25.43741	1	1	58.40797	1	19.66973	1	103.6483
BLZF1	1	33.15799	1	1	1	27.67304	1	1	1	31.06734	1	25.65273	1	1	1	1	1	1	1	1
BMP2	1	1	44.40243	1	1	1	1	1	1	1	1	1	1	1	1	1	1	22.35296	1	1
BMP2K	188.4271	1	1	1	1	51.93413	1	70.08735	1	1	1	33.03766	29.40356	1	1	1	1	59.14906	1	139.0453
BMP3	1	1	1	1	1	1	1	1	1	1	1	1	1	63.58742	1	1	1	1	1	1
BMP7	1	119.5377	66.82887	1	1	165.8879	1	1	1	49.05263	1	70.7675	47.65292	1	1	109.7808	1	1	1	1
BMPR1A	1	45.73275	1	57.47584	46.60396	44.29555	1	42.62609	1	48.12956	1	41.94654	46.97246	1	1	1	150.4777	1	1	1
BMPR1B	1	62.67415	1	1	1	41.2882	1	1	1	1	1	1	43.79654	1	1	1	1	1	1	1
BMT2	1	1	1	1	42.24327	1	1	23.2583	1	1	1	23.06876	1	1	1	1	1	1	1	1
BNIP1	1	1	79.2751	1	1	21.01531	1	1	1	1	1	24.28198	1	1	1	1	1	1	1	1
BOC	1	72.07388	1	1	1	150.2758	1	1	1	62.9435	1	54.60475	193.367	1	1	136.6253	1	1	1	1
BOLA1	1	1	1	1	1	22.43564	1	1	1	29.91675	1	1	1	1	1	1	1	20.02927	1	1
BOLA3	101.8146	1	30.57783	1	1	33.9113	1	53.39622	1	34.84636	1	33.69116	28.85507	1	1	82.16579	1	116.9962	1	1
BOP1	1	1	64.29014	1	1	1	1	1	1	34.00159	1	1	1	1	1	1	1	19.47629	1	1
BORA	1	1	1	1	1	1	1	1	1	1	1	35.1117	1	1	1	1	1	1	1	1
BPNT1	1	1	1	1	1	35.02979	1	24.05763	1	1	1	1	1	1	1	1	1	1	1	1
BRAF	1	87.62344	73.90178	144.4334	136.8378	58.73372	1	76.02461	1	93.79919	156.7312	78.65098	64.62883	145.5148	83.77153	1	1	62.43143	64.96111	1
BRCA1	1	1	1	1	1	1	1	1	1	109.2023	1	131.028	38.51641	1	1	53.69977	1	1	1	1
BRCA2	1	1	1	1	1	1	1	1	1	82.45986	1	119.1447	1	1	1	51.65546	1	94.5395	1	1
BRD3OS	1	1	1	1	1	39.35159	1	1	1	1	1	1	42.3243	1	1	1	1	1	1	1
BRINP1	1	1	55.80029	1	1	1	1	1	1	1	67.06275	1	1	1	1	1	1	1	1	1
BRINP2	1	1	1	1	1	1	1	1	1	1	1	1	1	119.1515	1	1	1	1	1	1
BRINP3	1	1	124.7313	1	1	1	1	1	1	1	1	1	1	1	1	1	1	1	1	1
BRIP1	1	1	1	1	1	1	1	1	1	56.44598	1	62.4577	1	1	1	53.79591	1	1	1	1
BRSK1	1	1	34.3918	62.6945	57.65026	1	1	1	164.2766	32.26837	1	26.12725	1	1	47.85289	1	104.4651	1	57.91256	70.02264
BRSK2	1	37.31234	44.08918	130.0952	108.4066	1	1	1	105.5301	70.38677	1	44.21332	29.8188	69.66519	119.847	1	82.05673	1	48.24263	1
BRWD3	1	1	1	59.42822	52.44834	26.59598	1	1	1	37.37341	1	28.12346	1	61.34095	1	1	1	19.62448	1	1
BST2	81.76699	1	1	1	1	1	1	1	1	1	1	1	1	1	1	1	1	118.3859	1	1
BTBD17	1	39.98323	1	1	1	53.25271	1	1	1	1	1	1	1	1	1	1	1	1	1	1
BTBD8	1	1	1	80.72042	249.4382	1	1	1	90.60677	1	81.44357	1	1	102.0695	142.4963	1	131.7721	34.81134	137.004	370.0455
BTN2A1	1	1	1	53.4654	43.65667	1	1	27.53113	74.81481	42.77719	1	46.90093	29.66502	1	1	1	1	11.97852	1	103.6483
BTN2A2	1	1	1	1	1	1	1	1	1	1	1	1	29.72579	1	1	1	1	1	1	1
BTRC	1	1	61.69765	1	1	37.09064	1	1	1	1	1	1	1	1	1	1	1	22.50919	1	1
BUB1	1	1	1	1	1	1	1	1	1	1	1	122.3079	1	1	1	1	1	1	1	1
BUB1B	1	1	1	1	1	1	1	1	1	1	1	127.5229	1	1	1	1	1	1	1	1
BUD13	1	1	1	1	1	1	1	1	1	1	1	28.1163	1	1	1	1	1	1	1	1
BX890604.	1	1	1	1	1	1	1	1	1	1	1	1	1	63.54737	1	1	1	1	1	1
BYSL	1	1	1	1	1	1	1	1	1	33.99605	1	1	24.60072	1	1	1	1	1	1	1
BZW2	1	40.41452	204.7791	128.0357	186.7559	1	248.9416	52.89431	93.95813	43.51059	171.6617	31.42037	1	135.4052	262.1823	47.49132	1	14.88346	164.1806	87.58009
C11orf54	1	83.81748	1	62.90903	44.6208	75.09228	1	40.68639	1	60.6138	61.47523	55.73566	49.63147	1	1	63.57029	1	32.18007	1	1
C11orf68	1	1	1	1	1	30.30155	1	1	1	1	1	1	1	1	53.05907	1	1	1	68.84102	1
C11orf70	1	1	1	1	1	1	1	1	1	1	1	1	61.53346	1	1	1	1	1	1	1
C11orf95	1	1	1	102.2196	87.91273	25.16684	1	20.42858	1	59.25915	83.17557	34.15675	29.74091	1	49.71736	1	1	1	46.86629	103.6483
C11orf96	1	282.1224	291.9098	1	1	1	1	2759.203	1	66.48799	609.0459	63.07007	1	862.247	1	1	1	201.8768	1	1
C12orf73	1	1	1	1	1	34.66368	1	1	1	39.20007	1	27.43317	30.31267	1	1	1	48.70842	1	1	103.6483
C12orf75	320.6102	1	1	1	1	74.03098	1	1	1	1	1	70.37035	40.73164	1	1	146.6462	1	82.40866	1	1
C14orf119	84.34243	53.83145	40.8003	1	1	66.37777	1	62.1986	1	55.6454	1	42.06612	49.07563	1	1	62.19147	1	114.396	102.6256	103.6483
C14orf132	1	65.21937	39.97238	220.322	198.3199	64.26331	191.078	1	107.2895	135.9312	184.518	98.00754	54.05684	150.5402	1	58.7903	1	65.59818	249.1238	1
C15orf40	1	1	1	1	1	45.97008	1	1	1	40.99114	1	31.42293	36.24822	1	1	1	1	1	1	1

C15orf41	1	1	1	48.87962	1	52.67427	1	1	1	48.80885	1	34.57067	29.72897	1	1	86.41316	1	1	1	1
C15orf59	1	1	1	1	1	26.2192	1	1	1	1	1	1	1	1	1	1	1	1	1	1
C16orf45	1	94.75724	126.8137	101.572	105.1418	71.32618	203.5663	1	193.7901	56.98572	135.9363	56.6987	49.3654	159.8764	96.407	69.04375	70.75404	1	69.69504	310.7162
C16orf74	1	1	1	1	1	34.00667	1	1	1	1	1	1	1	1	1	1	1	1	1	1
C17orf51	1	1	53.3335	1	41.16616	1	1	1	1	1	1	1	1	1	1	1	1	1	1	1
C17orf62	58.1565	1	54.76821	1	40.12871	44.10311	1	1	1	37.66649	1	32.60794	31.52491	1	1	1	1	30.75695	56.2826	1
C17orf67	1	1	1	1	1	1	1	1	1	1	1	1	1	1	1	1	1	111.3571	1	1
C17orf75	1	1	1	45.64651	1	27.36052	1	1	1	1	1	36.95682	30.74411	1	55.48903	1	1	57.66897	1	1
C17orf97	1	1	1	1	1	1	1	1	1	1	1	1	33.18358	1	1	1	1	1	1	1
C18orf32	1	1	1	1	1	33.94557	1	1	80.69476	1	1	1	1	1	59.0737	1	1	26.6535	1	1
C18orf54	1	1	1	1	1	1	1	1	1	31.10352	1	45.13403	1	1	1	1	1	1	1	1
C19orf12	1	31.23855	54.09448	1	39.85223	39.48755	1	28.98207	1	33.52421	1	37.44162	36.73656	58.66561	1	1	1	1	1	1
C19orf48	1	56.12793	41.79474	1	1	39.65241	1	1	1	109.1271	1	97.52389	52.40385	1	1	65.586	1	24.58604	1	1
C1GALT1C1	1	1	74.35084	1	1	26.98779	1	1	1	1	1	1	1	1	1	1	1	18.36132	1	1
C1QA	426.3613	1	1	1	1	1	1	1	1	1	1	1	1	1	1	1	1	1	1	1
C1QB	531.8014	1	1	1	1	1	1	1	1	1	1	1	1	1	1	1	1	1	1	1
C1QC	685.9102	1	1	1	1	1	1	1	1	1	1	1	1	1	1	1	1	1	1	1
C1QL1	1	191.3096	520.6159	1	1	109.8876	1	1	1	47.92464	1	58.21693	115.4671	1	1	94.66181	1	1	1	1
C1QL2	1	1	1	1	1	108.2316	1	1	1	1	1	1	1	1	1	1	1	1	1	1
C1QTNF4	1	67.34616	60.93928	111.66	137.5954	29.59547	210.1147	27.31262	580.2889	61.84692	150.1187	35.26926	64.73992	104.7419	185.6512	1	69.87818	14.34985	182.0118	103.6483
C1orf112	1	1	1	1	1	1	1	1	1	1	1	29.18741	1	1	1	1	1	1	1	1
C1orf115	1	1	1	1	1	1	1	1	1	1	1	1	1	1	105.358	1	1	231.5258	1	1
C1orf194	1	1	1	1	1	1	1	1	1	1	1	1	136.0533	1	1	1	1	1	1	1
C1orf198	1	1	1	1	1	58.46241	1	1	1	1	1	28.2957	44.72248	1	1	43.42212	1	1	1	1
C1orf216	1	1	1	1	44.08779	1	1	1	98.52333	1	65.48827	1	1	68.79072	34.07956	1	32.47948	1	1	1
C1orf50	1	1	1	1	1	1	1	1	1	1	1	1	1	1	1	1	1	1	92.07128	1
C1orf54	1	1	1	1	1	1	1	64.78037	1	1	1	1	1	1	1	1	1	282.1539	1	1
C2	104.283	1	1	1	1	1	1	1	1	1	1	1	1	1	1	1	1	1	1	1
C20orf194	1	1	36.53543	77.29997	75.11814	44.255	1	32.68763	191.9752	1	89.93429	29.94591	30.87562	100.9047	75.27126	1	1	1	76.7093	70.02264
C20orf27	136.1863	1	1	1	1	39.81065	1	53.60944	1	1	1	35.22105	45.71134	1	1	85.86948	1	27.46093	1	70.02264
C20orf96	1	1	1	1	1	1	1	1	1	1	1	1	36.88661	1	1	1	1	1	1	70.02264
C21orf58	1	1	1	1	1	1	1	1	1	48.89418	1	122.952	1	1	1	1	1	1	1	1
C21orf91	1	1	1	1	1	1	1	57.9863	1	1	1	1	1	1	1	1	1	1	1	1
C2CD4B	1	1	1	1	1	1	1	1	1	1	1	1	1	1	1	1	1	1527.357	1	1
C2CD5	1	1	1	61.69898	54.13407	1	1	1	1	32.06981	1	38.43272	1	1	1	1	1	18.18365	1	70.02264
C2orf27B	1	1	44.92639	1	1	1	1	1	1	1	1	1	1	1	1	1	1	1	1	1
C2orf40	1	1	1	1	1	1	1	1	1	1	1	1	97.71758	1	1	1	1	1	1	1
C2orf48	1	1	1	1	1	1	1	1	1	1	1	32.93668	1	1	1	1	1	1	1	1
C2orf49	1	42.21102	1	1	43.29592	43.55243	1	1	1	1	1	38.24942	46.47507	1	80.39336	1	1	1	1	1
C2orf68	1	1	40.23835	1	43.83315	28.20844	1	23.07028	1	40.04378	1	35.52644	34.17937	1	1	1	1	1	1	1
C2orf72	1	41.05628	63.02843	1	1	120.121	1	1	1	1	1	1	67.03308	1	1	66.05221	1	1	1	1
C2orf80	1	1	198.5528	1	1	1	1	1	1	1	1	1	1	1	1	1	1	1	1	1
C3	342.0948	1	1	1	1	1	1	1	1	1	1	1	1	1	1	1	1	1	1	1
C3AR1	62.463	1	1	1	1	1	1	1	1	1	1	1	1	1	1	1	1	1	1	1
C3orf70	1	141.182	208.6595	1	1	65.03338	1	21.25135	1	92.09489	83.2377	66.86364	34.47415	72.6781	1	1	1	1	1	1
C4orf46	1	1	1	1	1	1	1	1	1	56.27922	1	50.43755	1	1	1	1	1	1	1	1
C4orf47	1	1	1	1	1	1	1	1	1	1	1	1	31.25841	1	1	1	1	1	1	1
C5orf15	58.51716	35.41159	72.32753	1	1	54.69755	1	49.74905	1	27.0331	1	39.62029	40.77533	1	1	1	1	1	1	206.2967
C5orf30	1	67.58022	1	1	1	62.19222	1	1	1	64.77161	1	61.15723	37.81614	101.6831	1	1	1	1	1	70.02264
C5orf34	1	1	1	1	1	1	1	1	1	1	1	39.26686	1	1	1	1	1	1	1	1
C5orf49	1	1	1	1	1	33.29869	1	1	1	1	1	1	144.0108	1	1	1	1	1	1	1
C6orf118	1	1	1	1	1	1	1	1	1	1	1	1	94.73398	1	1	1	1	1	1	1
C6orf120	1	1	1	1	1	45.81846	1	32.68093	1	1	1	1	27.25139	1	1	1	1	1	1	1
C6orf203	1	51.28193	1	47.59491	43.35221	45.25381	1	1	1	47.2975	1	36.4562	29.06491	1	74.58333	45.61322	1	1	1	103.6483
C7orf57	1	1	1	1	1	1	1	1	1	1	1	1	40.6851	1	1	1	1	1	1	1
C8orf34	1	1	1	1	1	1	1	1	176.1672	1	1	1	32.69394	1	1	1	1	1	1	1
C8orf46	1	37.65295	89.87358	1	1	1	1	1	1	1	1	1	1	1	1	1	1	1	1	1
C8orf76	1	1	1	58.3923	1	49.61163	1	1	1	81.00026	1	44.47683	79.3234	1	1	44.2585	1	45.15995	1	1
C9orf116	1	1	1	1	1	1	1	1	1	1	1	1	91.10613	1	1	1	1	1	1	1
C9orf24	1	1	1	1	1	1	1	1	1	1	1	1	215.5996	1	1	1	1	1	1	1
C9orf3	1	1	1	1	1	1	1	1	1	1	1	1	29.08711	1	1	1	1	1	1	1
C9orf72	1	1	1	48.61046	53.13768	53.95676	1	1	1	50.26771	1	48.72201	85.45842	1	1	79.46096	1	59.4408	61.00684	70.02264
CA10	1	1	190.0069	1	1	1	1	1	1	1	1	1	1	1	1	1	1	1	1	1
CA12	1	75.53736	1	117.4342	1	74.69137	1	1	1	208.2863	1	95.78942	55.70719	1	1	75.32496	1	1	1	1
CA2	1	55.75546	1	1	1	29.52906	1	253.4652	1	1	1	1	1	1	1	1	1	489.6411	1	1
CA3	1	1	1	1	1	1	1	1	1	1	1	1	44.97723	1	1	1	1	1	1	1
CA4	1	1	1	1	1	1	1	162.6992	1	1	1	1	1	1	1	1	1	39.58694	1	1
CACHD1	1	1	1	1	1	40.21331	1	1	1	1	1	1	35.22027	1	1	1	1	1	1	1
CACNA1A	1	1	52.42942	1	1	1	1	1	502.092	1	1	1	1	1	1	1	75.7348	1	1	1
CACNA1D	1	1	1	1	1	1	1	1	93.82242	1	1	1	1	1	1	1	1	1	1	1
CACNA1E	1	1	1	1	1	1	1	1	195.5402	1	1	1	1	1	1	1	121.9285	1	1	1
CACNA1G	1	1	1	1	1	1	1	1	1	1	1	1	1	1	1	1	126.3901	1	1	70.02264
CACNA2D1	1	38.00983	1	117.5645	133.6829	31.64328	214.2052	45.45372	140.7534	72.72221	1	55.616	30.70042	1	217.7316	106.3489	501.6827	1	118.9231	172.671
CACNB2	1	1	50.39873	1	1	1	1	1	1	1	1	1	1	1	1	1	1	1	1	1
CACNB3	1	1	1	1	46.5558	1	1	1	1	1	1	1	1	1	101.0032	1	57.86955	1	1	1
CACNB4	1	57.92375	84.98522	112.4663	117.5164	1	1	127.433	162.2692	1	159.137	1	44.14426	103.8452	106.7995	1	316.6113	1	95.90241	206.2967
CACNG4	1	68.22665	120.9548	1	1	37.43768	1	1	57.11449	1	102.5596	33.75881	1	100.8429	1	1	1	1	1	70.02264
CACNG8	1	1	1	1	1	1	1	1	180.0117	1	1	1	1	1	1	1	209.3945	1	1	139.0453
CAD	1	1	1	1	1	1	1	1	1	32.02504	1	1	1	1	1	1	1	1	1	1
CADM1	1	145.2449	202.6198	181.0078	176.4155	81.12164	177.5306	1	299.1903	93.80654	661.9711	110.1434	66.63071	433.0647	180.3826	119.7381	244.1295	84.75783	250.2883	139.0453
CADM3	1	1	1	76.8847	92.16668	1	1	1	181.4839	1	95.84189	1	55.33419	98.97269	66.16817	1	150.5483	1	88.3263	103.6483
CADPS	1	1	1	1	1	1	1	1	252.3231	1	123.6829	1	1	327.044	1	1	43.85451	1	1	208.0679
CALB2	1	1	1	1	1	1	1	1	1	1	270.4732	1	1	1	1	1	1	1	1	1
CALCRL	1	1	61.51819	1	1	1	1	29.00121	1	1	1	1	1	1	1	1	1	162.654	1	1
CALD1	1	313.3863	170.2879	292.7739	152.8028	376.4832	1	4109.762	1	197.3575	183.9438	254.983	316.1542	95.70697	52.83596	322.2166	1	437.8012	220.7067	103.6483
CALN1	1	1	1	1	1	1	1	1	125.6237	1	1	1	1	1	1	1	1	1	1	70.02264
CALY	1	1	1	1	1	1	1	1	357.421	1	1	1	1	1	1	1	1	1	1	1
CAMK1	1	1	1	1	1	42.2054	1	76.54693	1	1	1	1	1	1	1	1	1	1	1	103.6483
CAMK2B	1	1	1	1	89.65023	1	163.7254	1	426.611	1	1	1	1	1	269.2107	1	405.6432	1	1	87.58009
CAMK2D	60.09979	160.3508	85.09189	1	1	76.5655	1	141.3782	178.3776	40.70914	86.51777	51.55358	58.34841	1	1	33.17719	1	20.12628	1	1
CAMK2N2	1	1	93.50107	1	1	1	1	1	1	1	1	1	1	1	55.74181	1	1	1	1	139.0453
CAMK4	1	32.519	1	121.997	171.1474	1	130.0912	1	215.0542	36.32944	218.9877	44.37421	1	79.09925	229.1952	74.85926	213.069	1	177.2913	174.1602
CAMKK2	1	1	1	1	52.69015	1	1	31.54073	125.6901	1	1	1	1	63.45488	85.50167	1	1	69.11535	61.93884	1
CAMKV	1	1	1	1	83.66014	1	1	1	271.497	1	1	1	1	1	77.94936	1	165.5142	1	1	139.0453
CAMSAP1	1	51.73338	36.53676	111.3537	120.2024	37.2829	183.3581	1	88.32187	85.43307	143.0876	81.05862	47.17437	170.5797	84.87345	51.83274	103.382	1	70.27422	70.02264
CAP2	1	1	1	46.93915	75.93362	58.49991	1	1	1	1	1	26.15817	59.86552	1	140.7267	1	72.84512	1	105.6723	116.5001
CAPG	316.5031	1	1	1	1	37.38106	1	1	1	1	1	48.50728	44.62901	1	1	1	1	1	1	1
CAPN10	1	1	1	1	1	1	1	1	1	1	1	1	1	1	1	1	40.16675	23.011	1	1
CAPN7	1	49.74406	58.99195	1	46.6241	38.74837	1	1	56.88033	37.74952	1	35.92525	41.35671	1	1	49.65466	89.80712	1	1	1
CAPRIN2	1	1	1	1	46.52523	1	1	1	1	32.50996	1	24.50633	1	1	68.82583	1	88.58826	1	49.72395	1
CAPS	1	1	1	1	1	1	1	1	1	1	1	1	179.4114	1	1	1	1	1	1	1

CARD8	1	1	1	1	1	1	1	1	1	1	1	50.36972	1	1	1	1	1	40.6091	1	1
CARM1	1	33.32613	1	46.30545	40.63477	31.72424	1	20.43843	1	47.51993	1	50.3039	37.99556	92.97059	1	1	139.954	1	1	1
CARMIL1	1	53.03416	1	1	1	62.94815	1	1	218.4116	1	1	1	30.35541	1	1	62.4296	1	1	1	1
CARMN	1	1	1	1	1	1	1	113.36	1	1	1	1	1	1	1	1	1	1	1	1
CARS	1	1	55.84539	1	1	32.13303	1	1	1	1	1	30.27202	28.41581	1	1	1	1	1	1	1
CARS2	1	1	34.216	1	39.72764	26.84517	1	1	1	34.69965	1	29.81877	29.3961	1	60.49826	1	1	1	1	87.58009
CASP1	110.8983	1	1	1	1	1	1	1	1	1	1	1	1	1	1	1	1	1	1	1
CASP10	1	1	1	1	1	1	1	1	1	1	1	1	1	1	1	1	1	48.20658	1	1
CASP3	1	80.97842	99.68075	1	1	276.7531	1	94.63296	1	54.97758	1	81.5908	166.2319	1	1	98.49816	1	154.1215	1	103.6483
CASP4	1	1	1	1	1	1	1	1	1	1	1	1	1	1	1	1	1	63.37278	1	1
CASP6	1	1	1	1	1	43.72006	1	28.91192	1	55.49529	1	63.51899	37.95944	1	1	48.70298	1	1	1	1
CAST	248.9776	79.80684	1	1	1	95.8576	1	223.6874	1	1	1	32.77951	110.4726	1	1	77.35881	1	146.0932	1	1
CAV1	1	1	1	1	1	1	1	35.49133	1	1	1	1	1	1	1	1	1	479.0573	1	1
CAV2	1	1	1	1	1	1	1	106.5745	1	1	1	1	1	1	1	1	1	120.4066	1	1
CAVIN1	1	1	1	1	1	1	1	1	1	1	1	1	29.65321	1	1	1	1	240.5302	1	1
CAVIN2	1	1	1	1	1	1	1	1	1	1	1	1	1	1	1	1	1	215.696	1	1
CAVIN3	1	1	1	1	1	1	1	330.0952	1	1	1	1	1	1	1	1	1	531.4942	1	1
CBFA2T2	1	1	1	144.2645	140.2494	35.20752	1	31.89115	65.60273	192.447	80.82878	63.93742	34.07122	61.19044	106.5168	1	1	1	122.8457	308.945
CBFA2T3	1	1	1	1	1	1	1	1	1	1	1	1	1	114.6207	1	1	1	1	1	1
CBFB	1	54.79611	41.48098	1	1	23.54084	1	1	1	36.57063	1	39.11532	35.35391	1	1	1	1	21.6519	1	1
CBLB	1	37.05394	1	64.88329	83.50369	1	1	41.46948	114.2592	1	77.38483	1	1	56.8694	80.27469	1	1	1	85.90154	275.3193
CBR1	1	102.8126	52.91484	1	1	94.93268	1	44.88764	1	39.36771	1	39.1905	76.577	1	1	103.9975	1	1	1	1
CBR3	1	1	1	1	1	1	1	1	1	1	1	27.00533	1	1	1	1	1	1	1	1
CBR4	1	42.70119	89.72586	51.47476	44.34299	65.52692	1	1	1	54.19109	1	52.01332	40.1142	82.36043	69.72657	1	1	27.92331	1	1
CBX2	1	1	1	1	1	1	1	1	1	34.79254	1	29.46846	1	1	1	1	1	111.7561	1	1
CBX4	45.25605	94.33853	141.7908	57.79049	101.7718	80.51424	1	93.63828	1	39.68229	169.5747	39.77789	65.34337	169.9587	60.92789	1	1	180.8298	1	1
CC2D1A	1	1	1	1	1	24.09749	1	1	50.37569	38.96637	1	1	26.12428	1	1	1	85.12422	21.2232	1	1
CC2D2A	1	52.29554	1	1	1	43.65819	1	47.44502	1	42.71175	1	32.1629	60.93299	1	1	50.03488	1	1	1	1
CCBE1	1	1	1	1	1	1	1	1	1	1	1	1	1	1	1	1	174.512	1	1	103.6483
CCDC102B	1	1	1	1	1	1	1	95.44645	1	1	1	1	1	1	1	1	1	1	1	1
CCDC106	1	1	1	1	1	23.99165	1	1	1	1	1	1	24.97655	1	1	1	1	115.2613	1	241.6936
CCDC113	1	1	1	1	1	21.70751	1	1	1	1	1	1	92.93216	1	1	1	1	1	1	1
CCDC136	1	1	1	48.15345	58.74679	1	1	1	143.5612	31.44482	1	1	24.985	1	47.42617	1	164.8589	1	1	1
CCDC138	1	1	1	1	1	1	1	1	1	35.0485	1	34.44179	37.90433	1	1	1	1	1	1	1
CCDC141	1	1	1	1	1	1	1	1	1	1	1	1	1	1	1	1	1	68.30909	1	1
CCDC144A	1	44.95314	1	1	1	31.78806	1	53.08436	264.0464	37.00734	1	25.51197	1	1	1	1	94.05959	1	1	139.0453
CCDC146	1	1	1	1	1	1	1	1	1	1	1	1	103.5128	1	1	1	1	1	1	1
CCDC15	1	1	1	1	1	1	1	1	1	25.16713	1	21.634	1	1	1	1	1	1	1	1
CCDC150	1	1	1	1	1	1	1	1	1	1	1	42.84619	1	1	1	1	1	1	1	1
CCDC152	1	34.76699	1	1	1	29.80757	1	1	1	51.5412	1	40.27125	27.56706	1	1	1	1	1	1	1
CCDC170	1	1	1	1	1	1	1	49.65319	1	1	1	1	102.6187	1	1	1	1	1	1	1
CCDC171	1	1	1	1	1	29.53273	1	1	94.22102	40.79043	1	27.9879	36.97591	1	1	1	1	1	1	1
CCDC173	1	1	1	1	1	1	1	29.10766	1	1	1	1	74.0897	1	1	1	1	1	1	1
CCDC174	1	39.73204	46.75241	64.1678	57.88557	56.60728	1	1	1	39.87895	85.26223	39.87614	48.16644	112.1676	1	53.07356	1	27.3913	52.97421	1
CCDC175	1	1	1	1	1	36.11388	1	1	1	1	1	1	1	1	1	1	1	1	1	1
CCDC18	1	1	1	1	1	1	1	1	1	1	1	54.28159	1	1	1	1	1	1	1	1
CCDC181	1	49.48481	1	1	1	31.88728	1	1	1	30.33984	1	66.72221	101.2541	1	1	1	1	1	1	1
CCDC184	1	1	1	1	1	1	1	1	96.75245	1	1	1	27.48623	59.12256	1	1	1	1	1	1
CCDC191	1	1	1	1	1	23.12015	1	1	1	1	1	1	62.31335	1	1	1	1	1	1	1
CCDC3	1	1	1	1	1	1	1	969.5174	1	1	1	1	1	1	1	1	1	1	1	1
CCDC30	1	1	1	1	1	1	1	1	1	1	1	1	52.57553	1	1	1	1	1	1	1
CCDC32	1	1	37.83355	1	1	34.26016	1	47.27096	1	1	1	1	30.9637	1	1	1	45.28881	44.45016	1	1
CCDC34	1	40.32678	1	1	1	1	1	1	1	37.17367	1	109.0958	52.0178	1	1	1	1	1	1	70.02264
CCDC40	1	1	1	1	1	1	1	1	1	1	1	1	57.45524	1	1	1	1	1	1	1
CCDC43	1	37.94166	38.83132	1	1	37.14725	1	1	106.7003	51.99954	59.49183	43.3637	34.90473	1	1	37.14424	1	123.5545	65.33955	1
CCDC65	1	1	1	1	1	1	1	1	1	1	1	1	35.60177	1	1	1	1	1	1	1
CCDC74A	1	1	1	1	1	1	1	1	1	1	1	27.30811	70.27788	1	1	1	1	1	1	70.02264
CCDC74B	1	1	1	1	1	1	1	1	1	1	1	1	49.31574	1	1	1	1	1	1	1
CCDC77	1	1	1	1	1	1	1	1	1	1	1	43.79942	1	1	1	1	1	1	1	103.6483
CCDC8	1	1	1	1	1	1	1	1	1	1	1	1	29.8117	1	1	1	1	1	1	1
CCDC80	1	184.3317	1	1	1	1	1	122.6338	1	1	1	1	1	1	1	1	1	1	1	1
CCDC84	1	1	23.33012	1	1	33.28328	1	1	1	38.68996	1	35.10387	1	1	1	1	1	1	1	1
CCDC85C	1	1	1	1	1	1	1	1	1	1	1	25.41936	1	1	1	1	1	1	1	1
CCDC88C	1	1	1	111.5878	68.64718	1	136.2963	1	1	49.27493	1	43.82587	33.32005	1	66.24283	1	1	25.73737	1	1
CCDC96	1	1	1	1	1	1	1	1	1	1	1	1	54.67348	1	1	1	1	1	1	1
CCHCR1	1	1	1	1	1	1	1	1	1	1	1	29.46006	1	1	1	1	1	1	1	1
CCL2	1	1	1	1	1	188.041	1	304.2991	1	1	1	1	153.4905	1	1	1	1	1	1	1
CCL3	11630.58	1	1	1	1	1	1	60.97172	1	1	1	1	1	1	1	1	1	1	1	1
CCL3L1	5844.101	1	1	1	1	1	1	1	1	1	1	1	1	1	1	1	1	1	1	1
CCL4	10671.82	1	1	1	1	1	1	1	1	1	1	1	1	1	1	1	1	1	1	1
CCL4L2	5192.304	1	1	1	1	1	1	1	1	1	1	1	1	1	1	1	1	1	1	1
CCL5	299.9149	1	1	1	1	1	1	1	1	1	1	1	1	1	1	1	1	1	1	1
CCM2	1	1	1	1	1	36.13177	1	44.40893	1	54.06538	1	52.85329	1	1	55.56422	1	1	35.42913	1	1
CCM2L	1	1	1	1	1	1	1	124.3108	1	1	1	1	1	1	1	1	1	51.29012	1	1
CCNA1	1	1	1	1	1	1	1	1	1	1	1	61.78317	66.88642	1	1	1	1	1	1	1
CCNA2	1	1	1	1	1	1	1	1	1	1	1	254.9232	1	1	1	1	1	1	1	1
CCNB1	1	1	1	1	1	1	1	1	1	1	1	388.4148	62.8919	1	1	1	1	1	1	1
CCNB2	1	1	1	1	1	1	1	1	1	34.39442	1	409.1177	1	1	1	1	1	1	1	1
CCND1	204.7347	157.4447	623.2342	1	1	1	1	196.8806	1	1	1	1	1	1	1	1	1	78.64454	1	1
CCND3	1	1	30.28515	1	1	1	1	1	1	1	1	1	1	1	1	1	1	25.49175	1	1
CCNDBP1	1	1	1	1	1	34.7209	1	28.82959	1	28.99355	1	23.77076	38.12117	1	1	1	1	33.29747	1	70.02264
CCNE2	1	1	26.88923	1	1	1	1	1	1	77.03661	1	53.35296	1	1	1	1	1	1	1	1
CCNF	1	1	1	1	1	1	1	1	1	1	1	52.00589	1	1	1	1	1	1	1	1
CCNQ	1	1	1	1	1	1	1	1	1	1	1	1	1	1	38.39449	1	1	29.45855	1	1
CCNT1	1	51.24475	1	50.12657	54.63128	42.00396	1	76.26971	1	52.99515	1	43.45597	52.73428	67.86608	1	1	1	83.70485	1	1
CCR1	74.80192	1	1	1	1	1	1	1	1	1	1	1	1	1	1	1	1	1	1	1
CCSAP	1	1	102.7585	156.4488	87.77268	26.18466	1	1	227.8492	76.55277	127.1139	87.90643	29.849	101.2314	66.28057	1	143.4317	1	131.7874	1
CCSER1	1	1	1	1	82.38657	1	1	1	187.5613	1	155.5899	1	1	153.7075	89.25846	1	398.0962	1	52.02016	208.0679
CD109	1	1	1	1	1	1	1	1	1	1	1	1	1	1	1	1	1	52.38864	1	1
CD14	443.5243	1	1	1	1	1	1	1	1	1	1	1	1	1	1	1	1	1	1	1
CD200	1	1	41.72199	105.0402	113.5351	1	1	1	176.8978	43.28214	118.9719	31.28679	38.27551	119.3488	117.5352	1	1	91.40023	152.6026	1
CD248	1	1	1	1	1	1	1	365.2914	1	1	1	1	1	1	1	1	1	1	1	1
CD276	63.02569	31.81772	1	51.12832	45.9725	33.6814	1	40.35304	1	40.93778	1	31.17101	22.70135	1	1	1	1	1	93.66024	103.6483
CD300A	143.1868	1	1	1	1	1	1	186.6868	1	1	1	1	1	1	1	1	1	1	1	1
CD302	1	1	1	1	1	1	1	54.59688	1	1	1	1	1	1	1	1	1	1	1	1
CD320	1	37.01824	48.41391	1	40.89873	38.01754	1	1	1	44.62675	1	35.64857	37.40474	1	62.38462	72.44373	1	592.7084	1	1
CD34	1	1	1	1	1	1	1	1	1	1	1	1	1	1	1	1	1	293.5176	1	1
CD36	224.9403	1	1	1	1	1	1	1	1	1	1	1	1	1	1	1	1	1	1	1
CD37	347.8041	1	1	1	1	1	1	1	1	1	1	1	1	1	1	1	1	1	1	1
CD38	1	1	1	1	1	78.22335	1	1	1	1	1	1	86.03936	1	1	1	1	1	1	1
CD4	70.55112	1	1	1	1	1	1	67.96527	1	1	1	1	1	1	1	1	1	1	1	1

CD40	1	1	1	1	1	1	1	96.72579	1	1	1	1	1	1	1	1	1	23.158	1	1
CD44	1	1	1	1	1	47.10262	1	42.65603	1	1	1	1	84.47996	1	1	1	1	1	1	1
CD53	218.8091	1	1	1	1	1	1	1	1	1	1	1	1	1	1	1	1	1	1	1
CD55	1	1	1	1	1	1	1	1	1	1	1	1	1	1	1	1	1	68.73627	1	1
CD5L	75.43677	1	1	1	1	1	1	1	1	1	1	1	1	1	1	1	1	1	1	1
CD68	685.531	1	1	1	1	1	1	1	1	1	1	1	1	1	1	1	1	1	1	1
CD74	577.1719	1	1	1	1	1	1	1	1	1	1	1	1	1	1	1	1	1	1	1
CD82	1	99.59093	146.978	1	1	28.14131	1	1	1	1	1	1	1	1	1	1	1	239.446	1	1
CD83	612.4193	1	1	1	1	1	1	1	1	1	1	1	1	1	1	1	1	1	1	1
CD84	217.3923	1	1	1	1	1	1	1	1	1	1	1	1	1	1	1	1	1	1	1
CD86	69.97023	1	1	1	1	1	1	1	1	1	1	1	1	1	1	1	1	1	1	1
CD9	1	218.0182	144.2343	1	1	167.8477	1	279.5666	1	1	1	51.18619	53.96278	1	1	78.59684	1	66.02912	1	1
CD93	1	1	1	1	1	1	1	1	1	1	1	1	1	1	1	1	1	262.8598	1	1
CD99	146.5387	111.2271	1	1	1	234.8166	1	273	1	1	1	71.26924	227.9453	1	1	211.2625	1	200.1465	1	1
CD99L2	1	1	1	1	1	1	1	1	1	1	1	1	1	1	1	1	1	102.7349	1	1
CDC14B	1	40.02203	1	1	1	59.70351	1	1	1	1	1	1	27.04295	1	1	1	1	1	1	1
CDC20	1	1	1	1	1	1	1	1	1	1	1	212.4641	1	1	1	1	1	1	1	1
CDC25A	1	1	1	1	1	1	1	1	1	31.45946	1	37.83761	1	1	1	1	1	1	1	1
CDC25B	1	1	1	1	1	1	1	1	1	1	1	93.7772	1	1	1	1	1	1	1	1
CDC25C	1	1	1	1	1	1	1	1	1	1	1	50.51557	1	1	1	1	1	1	1	1
CDC42EP1	1	37.89916	1	1	1	70.3217	1	80.63084	1	36.62619	1	1	101.8503	1	1	1	1	23.92363	1	1
CDC42EP3	1	1	1	114.5702	97.00614	27.40077	1	1	63.63894	113.7811	110.6477	88.72898	1	118.5747	96.34338	1	1	51.6502	44.60282	139.0453
CDC42EP4	1	40.19221	1	1	1	75.51862	1	73.53736	1	93.57656	110.8223	60.91831	63.37861	93.90145	1	63.32856	42.22042	67.37569	1	1
CDC42EP5	1	1	1	1	1	1	1	88.63466	1	1	1	1	1	1	1	1	1	27.90898	1	1
CDC45	1	1	1	1	1	1	1	1	1	64.29805	1	58.7651	1	1	1	1	1	1	1	1
CDC6	1	1	1	1	1	1	1	1	1	34.15588	1	31.08784	1	1	1	1	1	1	1	1
CDC7	1	1	1	1	1	1	1	1	1	32.82916	1	35.03075	1	1	1	1	1	1	1	1
CDCA2	1	1	1	1	1	1	1	1	1	1	1	111.5502	1	1	1	1	1	1	1	1
CDCA3	1	1	1	1	1	1	1	1	1	1	1	174.5551	1	1	1	1	1	1	1	1
CDCA4	1	1	1	1	1	1	1	1	1	38.43832	1	124.1337	1	1	1	1	1	1	1	1
CDCA5	1	1	1	1	1	1	1	1	1	43.29991	1	120.6472	1	1	1	1	1	1	1	1
CDCA7	1	77.3652	1	1	1	104.2518	1	1	1	259.2105	81.9665	134.8428	64.58683	100.4468	1	148.3625	1	1	1	1
CDCA7L	1	142.4504	163.4453	1	1	87.12491	1	1	1	61.3603	1	75.04037	38.96051	1	1	56.98088	1	31.44246	1	1
CDCA8	1	1	1	1	1	1	1	1	1	1	1	110.1059	1	1	1	1	1	1	1	1
CDH10	1	1	47.5716	1	1	1	1	1	209.7557	1	1	1	1	1	1	1	1	1	1	1
CDH11	1	1	42.38862	135.6079	147.7355	1	1	489.439	291.5816	1	1	1	25.32166	67.29022	152.3693	1	188.233	142.1986	1	139.0453
CDH13	1	1	1	1	1	1	1	1	1	1	1	1	1	1	1	1	160.9562	1	1	70.02264
CDH18	1	1	1	1	1	1	1	1	82.09517	1	1	1	1	1	1	1	1	1	1	1
CDH24	1	1	1	1	1	1	1	1	1	40.68868	1	23.83088	1	1	1	1	1	1	1	1
CDH4	1	32.03756	1	98.32514	223.1538	71.62769	216.756	1	1	43.68006	1	39.25821	50.28876	1	314.0792	73.94745	131.9	1	269.4494	276.8085
CDH5	1	1	1	1	1	1	1	1	1	1	1	1	1	1	1	1	1	294.9704	1	1
CDH6	1	1	1	1	1	1	1	393.3533	79.15151	1	1	1	1	1	1	1	1	109.1661	1	1
CDIP1	1	1	1	1	1	41.5221	1	1	1	44.62303	1	27.31412	28.6773	1	1	1	1	47.84162	1	1
CDK1	1	1	1	1	1	1	1	1	1	77.87445	1	459.6961	78.84682	1	1	114.3865	1	1	1	1
CDK14	1	38.60282	33.96386	1	1	28.01721	1	42.60645	1	1	77.93345	24.32734	29.09153	124.9938	1	1	1	1	1	1
CDK17	1	34.15838	1	1	1	37.27369	1	34.28693	90.0873	1	68.85008	35.5385	50.20658	56.15163	1	1	1	52.12827	1	1
CDK2	1	1	1	1	1	1	1	28.56676	1	51.13108	1	38.48085	1	1	1	1	1	98.97736	1	1
CDK5	1	1	1	1	1	1	1	1	104.2006	1	1	1	1	1	92.8034	1	58.94906	1	1	139.0453
CDK5RAP1	1	1	1	1	1	1	1	1	1	1	1	1	1	1	1	1	1	28.96892	58.40669	1
CDK6	1	185.9342	92.37335	1	1	25.6066	1	51.1855	1	224.9138	1	97.40843	38.71157	1	1	56.87763	1	32.51012	1	1
CDK8	1	1	1	1	1	1	1	1	1	35.32842	1	22.23859	1	1	1	1	1	1	1	1
CDKAL1	1	34.82592	1	1	1	39.89277	1	1	1	51.20039	1	34.873	1	1	1	1	1	33.53896	1	1
CDKL2	1	1	1	1	1	25.74713	1	1	151.1773	1	1	1	29.23837	1	1	1	1	1	1	1
CDKL5	1	1	1	53.93908	79.12964	31.73827	1	49.45112	222.8154	1	1	1	36.8389	111.1533	89.62208	1	125.1134	37.16975	1	1
CDKN1A	103.1229	1	1	1	1	1	1	115.9264	1	1	1	1	1	1	1	1	1	116.5581	1	1
CDKN1C	1	86.50847	81.02047	1	62.33811	1	1	95.7366	1	1	82.74299	1	1	121.9714	79.7874	1	89.87287	35.95251	1	103.6483
CDKN2AIP	76.67399	87.82823	47.83334	70.60066	78.5428	56.25986	1	27.95516	1	100.3523	127.0221	69.53398	62.57912	95.71094	69.46241	1	1	49.53515	1	70.02264
CDKN2C	1	1	1	1	1	1	1	1	1	1	1	139.8833	1	1	1	1	1	1	1	1
CDKN2D	1	1	54.2491	77.6527	152.5311	26.19294	183.1842	59.45721	308.9149	1	136.8652	110.3064	34.59354	119.2737	164.1671	1	304.2053	62.93182	107.4422	208.0679
CDKN3	1	1	1	1	1	1	1	1	1	1	1	263.3358	1	1	1	1	1	1	1	1
CDO1	1	67.29685	1	63.86484	51.5896	525.5246	1	1	1	103.6605	93.42752	134.6941	292.0061	289.9479	51.09069	299.3168	1	1	1	70.02264
CDON	1	53.65507	1	51.72433	1	108.5721	1	1	1	104.019	1	84.22969	195.4184	1	1	188.2674	1	1	1	1
CDR2L	1	1	1	1	1	1	1	35.78997	1	1	1	1	1	1	1	1	1	66.22585	1	1
CDT1	1	1	1	1	1	1	1	1	1	85.30085	1	83.69567	32.50539	1	1	1	1	1	1	1
CDYL	1	82.50629	66.21373	1	1	39.01503	1	27.29941	1	1	1	48.05999	52.98328	1	1	67.30537	1	27.66168	1	1
CEBPA	186.018	1	1	1	1	1	1	1	1	1	1	1	1	1	1	1	1	1	1	1
CEBPB	416.0877	1	1	1	1	1	1	652.2666	1	1	1	1	48.49141	1	1	1	1	71.23528	1	1
CEBPD	273.2718	121.0831	1	1	1	174.11	1	1174.195	1	1	1	1	480.3809	1	1	1	1	49.78245	1	1
CELF3	1	1	1	87.58632	90.44374	1	1	1	1	1	79.09136	1	1	91.13051	70.77701	1	118.4691	1	53.29788	103.6483
CELF4	1	1	1	1	1	1	1	1	405.4011	1	421.6947	1	1	261.6334	1	1	36.10813	1	1	1
CELF5	1	1	93.64586	175.5233	215.4079	1	297.0845	1	421.4237	51.58678	179.1443	46.28141	1	193.8339	279.1686	92.47621	240.5124	1	183.0557	275.3193
CELSR2	1	50.66501	60.83345	153.1242	153.6449	70.30358	169.7896	1	136.8659	84.85619	64.76023	58.42246	59.89372	86.73461	155.1042	67.02007	86.16674	1	198.2121	1
CELSR3	1	1	1	48.36372	45.78066	1	1	1	68.72945	1	1	1	1	1	57.68417	1	113.7766	1	1	70.02264
CEND1	1	1	1	1	1	48.51344	1	1	78.82355	1	1	1	28.53137	1	1	1	1	1	1	103.6483
CENPA	1	1	1	1	1	1	1	1	1	1	1	140.2761	1	1	1	1	1	1	1	1
CENPE	1	1	1	1	1	1	1	1	1	1	1	412.8547	1	1	1	1	1	1	1	1
CENPF	1	108.0124	117.0242	67.24173	48.33672	39.61256	1	54.43237	1	274.2292	1	2025.934	338.3586	1	68.97439	251.0695	1	1	1	1
CENPH	1	31.90741	1	1	1	1	1	1	1	122.5561	1	148.5648	47.38825	1	1	88.2686	1	1	53.85712	70.02264
CENPJ	1	31.06115	84.82559	1	48.60262	1	1	1	119.806	94.46151	68.04676	101.2334	1	73.95664	1	1	214.3626	1	1	1
CENPK	1	50.6813	82.49297	1	1	1	1	1	1	124.6981	1	210.3663	78.70775	1	1	106.3179	1	1	1	1
CENPL	1	1	1	1	1	1	1	1	1	1	1	97.94206	1	1	1	1	1	1	1	1
CENPM	1	1	1	1	1	1	1	1	1	56.4625	1	116.7377	1	1	1	1	1	1	1	1
CENPN	1	1	1	1	1	1	1	1	1	51.07641	1	146.1644	33.29072	1	1	52.49983	1	1	1	1
CENPO	1	1	1	1	1	1	1	1	1	27.66777	1	66.3873	1	1	1	1	1	1	1	1
CENPQ	1	1	1	1	1	1	1	1	1	35.11458	1	44.42069	1	1	1	1	1	1	1	1
CENPT	1	1	1	1	40.5638	22.29221	1	1	1	1	1	43.61588	21.80793	1	52.70997	1	1	26.51381	1	87.58009
CENPU	1	1	1	1	1	1	1	1	1	132.0368	1	160.5267	38.31744	1	1	79.93212	1	1	1	1
CENPW	1	1	1	1	1	1	1	1	1	39.1861	1	135.5299	1	1	1	1	1	1	1	206.2967
CEP104	1	1	1	1	1	1	1	1	1	1	1	1	1	1	1	1	1	116.249	65.29314	70.02264
CEP120	1	1	1	1	1	1	1	1	1	1	1	28.20155	1	1	1	1	1	1	1	1
CEP126	1	1	1	68.39419	122.6297	30.88827	1	1	195.3991	26.02773	1	1	95.2011	1	261.2186	1	189.2882	1	83.92264	103.6483
CEP128	1	1	1	1	1	43.59336	1	1	1	43.2987	1	63.95243	1	1	1	1	1	1	1	1
CEP135	1	32.25401	1	1	1	1	1	40.31186	1	79.20192	1	137.5149	64.22792	1	1	55.20048	1	12.27254	1	1
CEP152	1	1	1	1	1	1	1	1	1	47.38124	1	86.03306	1	1	1	1	1	1	1	1
CEP164	1	1	1	1	1	38.41996	1	35.38157	87.70925	29.82706	1	32.47902	34.5615	1	1	1	87.09886	1	1	1
CEP192	1	1	1	1	1	28.04234	1	1	1	48.07235	1	70.46785	29.4183	1	1	1	1	1	1	1
CEP250	1	1	1	1	44.28848	1	1	1	1	1	1	30.33955	1	69.19028	1	1	144.5679	1	70.36176	103.6483
CEP295	1	1	25.7331	1	49.58465	28.86396	1	1	1	55.56306	1	75.01298	34.03285	1	1	41.17805	54.74461	29.78161	45.99864	70.02264
CEP41	1	1	1	1	42.39617	1	1	1	1	46.30376	1	48.49565	48.39011	1	1	1	42.00817	18.75502	79.06677	1
CEP44	1	1	35.82377	1	1	1	1	1	1	37.45097	1	47.00787	1	1	1	1	1	1	1	103.6483
CEP55	1	1	1	1	1	1	1	1	1	1	1	71.52286	1	1	1	1	1	1	1	1

CEP57L1	1	1	54.09315	1	38.93659	1	1	33.56188	1	41.19788	1	78.77895	28.63875	1	52.79209	1	1	1	75.88247	1
CEP70	1	1	70.48932	45.20117	42.82596	41.98375	1	1	1	35.38045	62.11861	100.936	47.67147	1	1	63.50768	1	1	1	103.6483
CEP76	1	1	1	1	1	1	1	1	1	1	1	26.85196	1	1	1	1	1	1	1	1
CEP78	1	62.77961	90.95382	73.57436	96.75439	69.94494	1	1	152.3372	71.41872	112.8021	135.0576	67.73922	95.13547	66.015	120.2322	102.6669	30.25073	132.436	310.7162
CEP83	1	112.7389	62.76947	1	1	36.45839	1	1	1	42.09481	1	63.10109	60.62424	1	1	117.9212	1	1	1	1
CEP85	1	1	1	1	1	1	1	1	1	1	1	29.14559	1	1	1	1	1	1	1	1
CEP89	1	36.00863	1	49.64469	49.55004	55.62481	1	1	52.85257	35.57226	86.81323	61.3137	44.76633	1	1	100.1485	120.7029	1	1	1
CERK	1	1	1	78.23675	103.2048	1	113.9284	1	1	43.56018	117.9193	39.5234	26.2752	150.0099	127.892	1	78.40148	32.85042	99.89687	70.02264
CERS1	1	30.5975	47.33859	1	1	33.72354	1	1	1	1	1	1	38.86265	1	1	1	1	1	1	1
CERS4	1	1	68.72446	1	1	34.05855	1	1	1	1	1	1	1	1	1	1	1	1	1	103.6483
CERS5	1	45.02506	42.5256	51.74471	42.47217	48.17094	1	38.55837	1	37.47544	73.5135	33.14194	39.25199	65.19088	1	1	1	20.3405	1	1
CES4A	1	1	1	1	1	1	1	1	97.64669	1	1	1	1	1	1	1	1	1	1	1
CETN2	1	67.28413	35.7384	1	1	87.7431	1	40.01056	1	41.07098	1	56.44596	175.3478	1	49.88829	102.9696	1	16.48362	1	1
CFAP126	1	1	1	1	1	1	1	1	1	1	1	1	125.3763	1	1	1	1	1	1	1
CFAP43	1	1	1	1	1	1	1	1	1	1	1	1	47.19979	1	1	1	1	1	1	1
CFAP44	1	1	1	1	1	1	1	1	1	1	1	1	70.87441	1	1	1	1	1	1	1
CFAP45	1	1	1	1	1	1	1	1	1	1	1	1	46.37633	1	1	1	1	1	1	1
CFAP46	1	1	1	1	1	1	1	1	1	1	1	1	74.14819	1	1	1	1	1	1	1
CFAP52	1	1	1	1	1	1	1	1	1	1	1	1	50.32983	1	1	1	1	1	1	1
CFAP54	1	1	1	1	1	1	1	1	1	1	1	1	62.34773	1	1	1	1	1	1	1
CFAP73	1	1	1	1	1	1	1	1	1	1	1	1	32.78595	1	1	1	1	1	1	1
CFD	51.02083	1	1	1	1	1	1	1	1	1	1	1	1	1	1	1	1	1	1	1
CFH	1	1	1	1	1	1	1	189.5486	1	1	1	1	1	1	1	1	1	43.64561	1	1
CGNL1	1	1	1	1	1	1	1	1	1	1	1	1	1	1	1	1	1	221.7396	1	1
CGRRF1	1	65.53177	45.87062	1	1	37.73305	1	42.23017	1	1	1	29.13798	36.25807	1	1	1	1	1	1	1
CH25H	319.9415	1	1	1	1	1	1	1	1	1	1	1	1	1	1	1	1	1	1	1
CHADL	1	1	51.09225	1	1	1	1	1	1	1	1	1	1	1	1	1	1	1	1	1
CHAF1A	1	1	1	1	1	1	1	1	1	112.4155	1	70.3019	1	1	1	1	1	1	1	1
CHAF1B	1	1	1	1	1	1	1	1	1	41.3579	1	26.05465	1	1	1	1	1	1	1	1
CHAMP1	1	35.74183	102.3222	1	1	30.57669	1	1	1	40.16629	1	41.44667	32.61072	1	1	1	1	1	1	1
CHCHD10	492.8801	62.51459	1	1	1	47.51336	1	188.2559	1	1	1	1	29.71069	1	1	1	1	32.46284	1	70.02264
CHCHD7	114.8271	1	1	46.50639	1	51.72655	1	31.45763	1	57.83958	1	44.45889	1	1	1	1	1	42.32455	40.40896	1
CHD5	1	1	1	1	1	1	1	1	216.5247	1	1	1	1	1	1	1	157.0216	1	1	1
CHD7	69.71152	375.4636	373.7206	132.2846	49.65001	102.4541	1	77.96148	1	344.2315	508.0079	195.2822	148.9491	140.3065	1	135.4907	1	63.29853	51.826	1
CHDH	1	41.84942	1	1	1	1	1	1	1	1	1	1	1	1	1	1	1	1	1	1
CHEK1	1	38.51158	1	1	1	1	1	1	1	137.2007	1	146.6263	36.49488	1	1	71.75218	1	1	1	1
CHEK2	1	1	1	1	1	1	1	1	1	1	1	29.58942	1	1	1	1	1	1	1	1
CHERP	1	37.54869	1	1	47.15289	38.07316	1	25.73969	1	50.65495	64.87115	45.04799	41.96648	66.0048	34.26784	1	1	29.4638	52.81487	1
CHFR	1	1	1	1	1	26.24077	1	23.61868	1	1	1	1	1	1	1	1	120.4078	27.46059	1	1
CHGA	1	1	1	80.72652	61.86254	1	1	1	449.8122	1	1	1	1	1	1	1	1	1	1	1
CHGB	1	1	1	51.33275	67.80542	1	1	1	290.8453	1	84.96974	1	1	77.05721	62.04724	1	65.41087	1	96.33414	103.6483
CHI3L1	63.34045	1	1	1	1	1	1	1	1	1	1	1	1	1	1	1	1	1	1	1
CHIC2	1	73.35086	72.04982	1	1	1	1	1	1	1	1	37.25563	39.87847	1	1	1	1	42.19849	1	1
CHL1	1	136.8146	31.62113	68.3488	176.2614	87.15574	325.6821	1	2564.088	1	512.9549	1	54.97938	551.3937	543.8565	85.79166	901.6464	1	120.8505	276.8085
CHM	1	42.68498	1	49.56286	1	39.05099	1	74.86441	105.5125	39.48237	1	31.97815	48.24984	1	1	1	1	26.24398	1	1
CHMP1A	1	22.47224	39.327	44.92136	44.98036	43.35565	1	30.88742	1	48.65377	1	35.50183	32.92278	1	1	1	1	21.74159	76.33698	1
CHMP7	1	1	36.94015	48.88156	44.58192	31.13949	1	1	1	37.12603	1	28.67633	22.70159	1	1	1	1	1	1	70.02264
CHN2	1	1	1	1	1	1	1	1	1	1	1	1	1	1	1	1	151.2946	1	1	1
CHPF	1	1	1	1	1	41.53484	1	28.39232	1	1	1	1	29.47972	1	1	1	36.24776	1	1	1
CHRDL1	1	1	1	119.071	1	66.53384	1	1	1	58.33423	1	59.36634	1	1	1	1	1	1	64.28649	1
CHRM3	1	1	82.76052	1	1	1	1	1	143.8951	1	1	1	1	1	1	1	1	1	1	1
CHRNA4	1	1	1	1	1	1	1	1	85.94469	1	1	1	1	1	1	1	1	1	1	1
CHRNB1	1	1	1	1	1	26.73378	1	1	1	1	1	1	1	1	1	1	1	1	1	1
CHST1	1	1	1	1	1	39.08593	1	1	95.98306	1	1	1	1	1	1	1	1	1	1	1
CHST10	1	69.79926	1	50.61232	58.17354	128.5344	1	1	1	80.04114	1	77.60787	134.7111	1	1	77.62796	231.0481	1	1	103.6483
CHST11	1	69.65533	153.3446	1	1	32.52579	1	1	1	1	1	1	1	1	1	1	1	1	1	1
CHST12	1	32.18776	1	1	1	1	1	23.79231	1	32.11352	1	1	1	1	1	1	1	76.90065	1	1
CHST15	1	1	1	1	1	1	1	1	60.17662	1	1	1	1	1	1	1	1	80.78311	1	1
CHST2	1	1	1	1	1	1	1	264.2824	1	1	1	1	1	1	1	1	1	72.29337	1	1
CHST3	1	1	41.93601	1	1	1	1	1	1	1	1	1	1	1	1	1	1	1	1	1
CHST7	1	64.12971	1	1	1	1	1	1	1	1	1	1	1	1	1	1	1	53.80959	1	1
CHST9	1	1	1	1	1	49.62407	1	1	1	1	1	1	70.45882	1	1	1	1	1	1	1
CHSY1	1	26.01576	1	1	1	34.72283	1	53.34834	1	28.13543	1	28.71858	36.3436	1	56.44	1	1	23.65591	1	103.6483
CHTF18	1	1	1	1	1	1	1	1	1	1	1	36.24407	1	1	1	1	1	1	1	103.6483
CHTF8	1	1	1	1	1	1	1	1	1	1	1	29.44644	1	1	1	1	84.62955	18.18584	1	1
CIB1	71.39708	48.91836	1	1	1	61.59527	1	71.98685	1	1	1	30.56722	92.55389	1	1	92.40987	1	40.93826	1	1
CIB2	1	54.26538	1	1	1	45.54998	1	1	1	58.51638	1	65.80296	29.03721	1	1	54.76038	1	1	1	1
CIC	1	46.86088	1	1	49.23424	40.17958	1	39.70647	1	38.58892	1	41.50036	42.84854	1	1	1	79.31532	31.50591	1	103.6483
CIDEA	1	1	1	1	1	1	1	1	73.49965	1	1	1	1	1	1	1	1	1	1	1
CINP	1	46.54963	61.0567	1	1	46.50216	1	34.9364	1	1	1	28.68513	33.94078	1	1	1	1	27.85449	43.31938	1
CIP2A	1	1	1	1	1	1	1	1	1	40.91643	1	115.7294	1	1	1	1	1	1	1	1
CIPC	1	1	1	1	1	33.95625	1	1	1	38.46087	1	1	46.63514	1	1	1	1	1	1	1
CISD3	53.40073	1	1	1	1	1	1	43.07257	1	1	1	1	1	1	1	1	1	20.66246	1	1
CIT	1	1	1	1	1	1	1	1	1	1	1	68.60514	1	1	1	1	1	1	1	1
CITED1	1	80.37817	1	1	1	52.93191	1	1	1	1	1	1	48.74504	1	1	1	1	1	1	1
CITED2	235.7609	53.67665	1	71.99823	60.92336	42.15829	1	113.5368	1	52.34484	476.3015	53.20338	78.68773	332.9006	48.70248	1	1	25.67116	103.7276	1
CITED4	97.56012	1	1	1	1	1	1	1	1	1	1	1	28.42974	1	1	1	1	34.12234	1	1
CKAP2	1	61.32518	85.21708	48.13561	56.79729	55.73005	1	1	1	77.90534	1	475.2951	98.94858	1	1	70.52838	1	48.24422	1	1
CKAP2L	1	1	1	1	1	1	1	1	1	1	1	155.7584	1	1	1	1	1	1	1	1
CKS1B	72.14724	47.31871	47.61115	1	1	71.97864	1	89.01314	1	118.6326	1	324.0797	67.17628	1	1	112.3443	1	16.39973	1	1
CKS2	141.9824	97.2268	41.23821	50.32094	64.08494	73.24394	1	172.0904	1	158.2773	1	558.4729	182.289	1	1	100.8395	1	214.4188	1	1
CLASP1	1	37.93962	45.94727	64.9294	73.54098	37.74645	1	47.62084	66.54105	45.77986	230.7992	47.94803	32.92396	120.9615	87.7665	51.83743	89.49128	26.7767	1	139.0453
CLCN4	1	1	1	1	1	1	1	1	301.029	1	1	1	1	1	1	1	1	1	1	1
CLDN11	1	1	277.0637	1	1	1	1	1	1	1	1	1	1	1	1	1	1	1	1	1
CLDN5	1	1	1	1	1	1	1	1	1	1	1	1	1	1	1	1	1	5343.474	1	1
CLEC11A	1	1	55.67487	1	1	1	1	323.4683	1	1	1	1	27.72629	1	1	1	1	1	1	70.02264
CLEC14A	1	1	1	1	1	1	1	1	1	1	1	1	1	1	1	1	1	273.0561	1	1
CLEC1A	1	1	1	1	1	1	1	1	1	1	1	1	1	1	1	1	1	137.2003	1	1
CLEC2B	1	1	1	1	1	1	1	1	1	1	1	1	1	1	1	1	1	156.7585	1	1
CLEC3B	1	1	1	1	1	1	1	1	1	1	1	1	1	1	1	1	1	61.96963	1	1
CLEC7A	169.7912	1	1	1	1	1	1	1	1	1	1	1	1	1	1	1	1	1	1	1
CLGN	1	1	44.46365	1	1	1	1	1	1	27.27314	69.57892	41.5019	1	1	1	1	1	1	1	1
CLHC1	1	1	1	1	1	1	1	1	1	1	1	1	39.32913	1	1	1	1	1	1	1
CLIC1	407.5301	63.38592	1	48.32697	1	130.9508	1	462.7874	1	100.0064	1	184.3421	100.9913	1	1	99.07045	1	444.7671	1	1
CLK3	1	36.68743	35.89505	43.9162	43.85734	30.59399	1	1	1	35.17352	1	32.85106	30.48372	1	1	1	1	36.78552	1	1
CLMP	1	1	1	477.7593	379.1807	1	448.0786	1	111.5513	134.3124	1	148.2561	1	1	420.9252	1	233.2541	1	417.1185	308.945
CLOCK	1	1	1	1	42.72229	34.06297	1	69.00206	101.0239	35.75809	1	46.49451	45.47051	1	63.75819	1	1	25.77294	1	1
CLPB	1	39.80754	1	1	1	1	1	1	1	31.15148	1	21.82482	1	1	1	1	1	1	1	1
CLSPN	1	1	1	1	1	1	1	1	1	136.5176	1	122.632	1	1	1	70.83762	1	1	1	1
CLSTN2	1	1	36.78097	1	1	1	1	1	1	1	1	1	1	1	1	1	1	1	1	1
CLU	1	404.9044	1	47.33595	1	1434.227	1	1	1	109.3922	1	205.3561	1162.414	1	1	633.001	1	1	1	1

CLVS1	1	1	1	75.71519	73.91192	1	1	1	1	1	1	1	1	1	1	1	30.95524	1	87.38625	70.02264
CLVS2	1	1	1	87.09536	73.25649	37.75711	1	1	1	45.40898	117.2866	52.03258	1	60.69047	54.08078	1	52.12955	1	91.84805	1
CMBL	1	35.71231	39.14713	1	1	53.66268	1	1	1	1	1	1	45.29521	1	1	37.08002	1	1	1	1
CMKLR1	1	1	1	1	1	1	1	58.82606	1	1	1	1	1	1	1	1	1	1	1	1
CMTM5	1	1	61.56381	1	1	1	1	1	1	1	1	1	1	1	1	1	1	1	1	1
CMTM8	1	1	1	1	1	1	1	1	1	1	1	1	1	1	1	1	1	57.91496	1	1
CMTR2	1	33.92536	35.97809	1	1	32.85654	1	1	1	1	1	1	1	1	1	1	1	1	1	70.02264
CNIH2	1	46.86958	54.43119	169.898	155.2968	53.21286	199.4253	1	220.6092	134.9861	1	94.75769	112.1027	66.48633	182.312	132.973	189.9465	1	143.7752	139.0453
CNKSR2	1	38.65845	1	146.0129	190.7329	113.1869	1	1	160.3882	71.56758	70.47879	60.73077	51.137	1	96.59008	80.9681	1	1	179.9175	87.58009
CNKSR3	1	63.73399	35.7484	1	1	78.69623	1	1	1	1	1	38.1226	69.29901	1	1	1	1	136.3457	1	1
CNMD	1	1	52.27564	1	1	89.02331	1	1	1	1	1	1	42.81672	1	1	51.28805	1	1	1	1
CNN2	1	1	1	1	1	1	1	1	1	1	1	1	34.18891	1	1	1	1	48.24269	1	1
CNOT10	1	30.9826	1	1	1	1	1	1	1	32.14035	1	39.6675	28.41916	1	1	1	143.2837	20.56823	1	1
CNOT6L	1	57.49841	65.67112	64.31764	72.71594	48.61628	1	46.08751	1	65.36064	122.228	43.64378	52.71801	90.90422	1	1	1	100.2117	56.44068	172.671
CNP	1	98.41412	658.0535	1	1	95.10147	1	32.40815	1	53.86037	1	54.33838	50.72969	1	55.0325	48.79047	1	44.26708	1	70.02264
CNPPD1	1	1	38.79917	1	1	28.18569	1	1	1	1	1	1	1	1	1	1	1	21.13527	1	1
CNPY2	54.90094	119.9697	197.3767	93.82894	82.44754	85.95182	1	73.02644	181.0065	108.2841	93.20394	71.88863	93.08234	86.88189	97.45708	149.6004	1	64.77046	115.0343	275.3193
CNPY3	309.079	33.34714	30.82998	1	1	32.66595	1	68.16875	1	45.62794	1	36.94332	33.24128	1	1	1	1	61.75496	1	1
CNR1	1	1	1	486.8628	328.3929	70.64632	245.764	1	1	149.2746	318.9949	140.8818	98.45139	1	213.4541	94.41193	228.9467	51.32513	410.5278	103.6483
CNST	1	1	1	1	1	1	1	27.24272	1	1	1	1	1	1	1	1	1	56.75649	1	1
CNTFR	1	90.67789	1	54.74503	43.80133	161.233	1	1	72.58983	76.38598	97.68577	86.03939	77.01375	63.73899	1	110.4223	1	1	38.08606	1
CNTLN	1	97.31367	67.05103	1	1	77.4797	1	1	1	163.7638	1	149.4663	72.10007	1	1	107.3239	1	31.26916	1	1
CNTN1	1	1	363.5955	1	1	1	1	1	401.8982	1	1	1	1	1	1	1	99.90188	1	1	70.02264
CNTN2	1	1	1	57.58253	55.20876	1	1	1	147.3798	1	1	1	1	1	52.2485	1	1	1	145.1433	1
CNTN3	1	1	26.94213	1	1	34.9225	1	1	1	1	1	1	30.63819	1	1	106.7454	123.8176	24.82339	1	1
CNTN4	1	1	1	1	1	1	1	1	73.15682	1	1	1	1	1	1	1	1	1	1	1
CNTNAP2	1	1	74.20516	57.05359	1	1	1	1	1	1	1	1	1	124.225	1	1	1	1	1	1
CNTNAP4	1	1	1	1	1	1	1	1	1	1	1	1	1	96.40856	1	1	1	1	1	1
CNTRL	1	68.72502	32.88565	56.00848	54.41443	41.03664	1	1	1	57.11099	1	141.4228	96.58172	1	1	152.3364	1	34.71513	1	1
CNTROB	1	1	1	1	1	1	1	1	1	1	1	34.02793	1	1	1	1	1	1	1	1
COA7	1	1	1	1	43.84569	1	1	1	1	1	1	23.70083	24.28827	1	1	1	1	1	1	103.6483
COBL	1	1	47.4396	1	1	1	1	1	1	1	1	1	37.20045	1	1	1	1	1	1	1
COBLL1	1	1	1	1	1	1	1	367.2838	1	1	1	1	1	1	1	1	1	459.1768	1	1
COG4	1	1	1	1	1	41.24089	1	51.50592	1	39.89524	1	33.98462	30.38693	1	1	74.58577	1	13.73865	1	1
COG5	1	1	1	1	1	1	1	1	1	31.06368	1	1	24.31596	1	1	1	109.5225	103.8173	1	103.6483
COG6	1	37.90048	1	1	1	24.97909	1	1	1	32.06848	1	1	1	1	35.64434	1	1	1	1	1
COG7	1	1	42.33318	1	1	1	1	1	1	1	1	1	25.22954	1	1	1	1	1	1	1
COL11A1	1	61.84311	180.1502	1	1	209.5344	1	1	1	140.6571	1	107.5417	232.7089	1	1	169.949	1	1	1	1
COL12A1	1	1	1	1	1	1	1	38.09793	171.2439	1	1	1	1	1	1	1	1	1	1	1
COL18A1	1	1	1	1	1	1	1	392.1848	1	1	1	1	1	1	1	1	1	1	1	1
COL1A1	1	1	1	1	1	1	1	111.821	1	1	1	1	1	1	1	1	1	1	1	1
COL1A2	1	1	1	1	1	1	1	1315.239	1	1	1	55.28655	1	1	1	1	1	1	1	1
COL20A1	1	1	277.802	1	1	1	1	1	1	1	1	1	1	1	1	1	1	1	1	1
COL3A1	1	1	1	1	1	1	1	865.1061	1	1	1	1	1	1	1	1	1	1	1	1
COL4A1	1	1	1	1	1	1	1	5083.742	1	1	1	1	1	1	1	1	1	558.2133	1	1
COL4A2	1	36.78003	1	1	1	1	1	2974.783	1	38.25659	1	1	1	1	1	1	1	315.4047	1	1
COL4A5	1	36.68855	1	1	1	93.78415	1	63.84213	1	40.8627	1	37.49168	59.66354	1	1	48.35114	1	1	1	1
COL4A6	1	1	1	1	1	57.1901	1	1	1	1	1	1	38.18588	1	1	1	1	1	1	1
COL5A2	1	1	1	1	1	57.10379	1	373.0543	1	1	1	25.79417	29.92392	1	1	1	1	43.63383	1	1
COL5A3	1	1	1	1	1	1	1	145.02	1	1	1	1	1	1	1	1	1	1	1	1
COL6A1	1	36.58529	33.68419	46.16471	50.72659	79.48746	1	561.9156	1	1	1	25.36734	61.36811	1	1	1	127.365	1	49.70743	1
COL6A2	1	1	1	1	1	39.27957	1	295.9492	1	1	1	1	24.66402	1	1	1	1	80.21245	1	1
COL9A1	1	40.1631	315.1238	1	1	27.2352	1	608.174	1	34.37286	1	51.62196	26.70323	1	1	1	1	1	1	1
COL9A2	1	1	100.8394	109.5676	116.7524	46.41145	1	1	1	1	1	1	30.09885	1	97.22397	1	180.714	1	164.4939	206.2967
COL9A3	1	1	174.8396	59.2145	97.28583	38.51754	1	1	1	1	1	1	1	1	89.49227	1	120.0323	71.90699	1	1
COLEC12	1	1	1	1	1	1	1	787.2213	1	1	1	1	1	1	1	1	1	1	1	1
COLGALT1	1	1	1	1	1	1	1	30.72051	1	1	1	1	1	1	1	1	96.33619	30.20025	1	1
COMMD1	57.88856	45.47791	110.5902	64.57982	55.96625	65.29973	1	64.57905	1	53.27423	1	55.0066	54.85761	61.17806	47.30582	72.63432	41.62871	67.5624	1	206.2967
COMMD4	114.27	63.19561	53.68258	1	1	57.14622	1	38.65304	1	98.79791	1	117.5815	48.57063	1	1	78.856	1	102.9656	1	103.6483
COMMD9	1	1	43.20226	51.15134	43.97981	49.11867	1	1	1	47.50065	1	35.65504	32.91546	1	68.34315	1	1	20.59314	1	70.02264
COMTD1	1	1	1	1	1	1	1	1	59.01758	1	1	1	1	1	1	1	1	1	1	1
COP1	1	1	41.36296	1	38.21319	27.66312	1	31.26097	112.691	33.81318	1	37.06925	25.99095	65.26254	64.79531	1	1	15.456	1	103.6483
COPG1	1	1	41.76243	1	1	31.11987	1	28.52892	1	1	1	1	1	1	1	1	1	1	56.88161	70.02264
COPS7B	1	1	39.64326	1	1	1	1	1	1	1	1	29.24543	1	1	1	1	1	1	1	1
COQ2	49.633	1	1	1	1	1	1	1	1	27.40063	1	41.25509	1	1	1	1	1	1	1	1
COQ6	1	1	20.79448	1	1	1	1	1	1	1	1	1	1	1	1	1	1	1	1	1
COQ7	1	53.4068	40.06917	63.69658	47.69037	47.88404	1	1	1	49.57903	63.73958	53.46839	48.09988	74.19623	1	33.37292	1	1	1	1
CORO1A	315.2438	1	1	1	1	1	1	1	1	1	1	1	1	1	1	1	1	1	1	1
CORO1B	105.5661	1	1	1	1	31.64728	1	39.21401	1	1	1	1	1	1	1	1	1	132.0094	1	1
CORO2B	1	59.86611	1	141.3509	112.4548	57.47107	1	66.70912	1	75.76755	67.17374	38.05495	28.23901	151.0733	107.4319	1	1	1	70.28868	116.5001
CORO7	57.00721	1	61.4183	1	1	1	1	23.31052	1	1	1	1	1	1	1	1	1	1	1	1
COX15	1	1	1	1	1	30.86011	1	1	1	44.93242	1	1	28.66032	1	1	1	1	1	1	1
COX19	1	39.67944	33.59952	46.65037	46.34027	32.61538	1	26.42145	1	41.81417	75.19639	24.62736	35.54527	1	1	1	1	48.04958	67.22381	1
COX7A1	1	1	1	1	1	1	1	208.8052	1	1	1	1	1	1	1	1	1	72.04546	1	1
CPD	1	1	32.01608	1	1	1	1	34.63095	1	1	1	1	29.78907	1	1	1	1	296.0706	1	1
CPED1	1	1	1	1	1	1	1	107.6186	1	1	1	1	1	1	1	1	1	1	1	1
CPLX2	1	1	1	1	1	1	1	1	74.82358	64.42003	210.5883	42.24969	1	1	1	1	1	1	1	1
CPPED1	1	1	1	1	1	1	1	90.8877	1	1	1	1	1	1	1	1	1	1	1	1
CPSF1	1	1	1	1	1	1	1	1	1	27.59738	1	22.24651	1	1	1	1	1	1	1	1
CPSF4	1	1	1	1	1	1	1	1	1	34.4759	1	30.99148	1	1	1	1	1	1	1	1
CPSF7	1	70.93956	29.0947	55.45868	50.73274	47.1315	1	1	1	69.39148	63.65838	63.68405	58.23237	1	1	1	1	1	61.20043	1
CPT1A	1	41.54887	1	1	1	49.61657	1	60.30331	1	1	1	1	1	1	1	1	1	52.92388	1	1
CPT1C	1	1	31.51448	1	47.67612	1	1	1	1	1	1	1	1	1	1	1	99.2592	1	45.36495	206.2967
CPVL	220.4873	1	1	1	1	1	1	1	1	1	1	1	1	1	1	1	1	1	1	1
CPXM1	1	1	1	1	1	1	1	1	1	28.63269	1	1	1	1	1	1	1	1	1	1
CRAT	1	1	1	1	1	1	1	1	1	1	1	31.38562	1	1	1	1	1	1	1	70.02264
CRB1	1	56.79272	95.93662	1	1	1	1	1	1	66.69609	1	42.21923	57.50657	72.64844	1	1	1	1	1	1
CRB2	1	1	1	1	1	63.23037	1	1	1	1	1	1	64.56663	1	1	1	1	1	1	1
CREB5	1	377.7945	195.2741	55.89881	1	372.2306	1	26.48467	1	146.5579	1	236.4862	339.2084	1	1	206.0376	1	77.14836	1	1
CREBL2	54.47058	1	1	1	1	1	1	1	1	30.22427	1	1	1	1	1	1	1	1	1	70.02264
CREG1	495.4776	1	52.0586	1	1	1	1	24.10925	1	1	1	1	1	1	1	1	1	45.62919	1	87.58009
CREM	96.80136	56.1104	44.74526	1	1	51.58912	1	227.0048	1	1	1	30.18799	34.92252	1	1	1	1	119.7078	1	1
CRH	1	1	1	1	1	99.10284	1	1	1	1	1	1	1	1	1	1	1	1	1	1
CRHR2	1	1	1	1	1	1	1	1	1	43.2775	1	1	1	1	1	1	1	1	1	1
CRIM1	1	58.03526	1	1	1	97.349	1	35.84063	1	1	84.4415	34.88393	45.88911	1	1	69.69111	1	68.76932	65.64071	1
CRISPLD1	1	37.48122	1	1	1	28.52235	1	1	1	1	1	29.57669	95.43099	1	1	52.10847	1	1	1	1
CRISPLD2	1	1	1	1	1	1	1	89.07776	1	1	1	1	1	1	1	1	1	1	1	1
CRLS1	1	77.30577	119.1496	1	1	51.84142	1	28.90626	1	41.86591	1	40.03043	48.4993	1	1	50.53189	1	30.71526	85.88071	1
CRNDE	1	1	1	1	1	1	1	1	1	1	1	76.94981	1	1	1	1	1	1	1	1
CRNKL1	1	41.92607	59.35989	62.90953	65.126	35.63435	1	1	1	66.31043	92.81304	56.72468	38.35285	63.50842	1	42.03712	1	34.67521	1	1
CROCC	1	1	1	41.49863	40.78494	34.77495	1	1	1	1	1	29.89536	30.22462	1	1	35.61019	1	1	1	1

CROT	1	1	1	1	1	38.594	1	1	1	1	1	1	1	1	1	1	1	1	1	1
CRTC1	1	1	1	1	1	1	1	1	154.9182	1	1	1	1	1	1	1	1	1	1	1
CRY1	1	65.82889	1	1	1	34.65525	1	50.84149	1	1	1	33.11949	47.37659	1	1	1	155.7742	25.85761	1	1
CRYAB	1	66.33411	1	1	1	441.1349	1	54.41011	1	1	1	1	4494.947	1	1	936.4628	1	1	1	1
CRYBB1	160.4979	1	1	1	1	1	1	1	1	1	1	1	1	1	1	1	1	1	1	1
CRYBG3	1	1	1	1	1	1	1	37.68124	1	1	1	1	1	1	1	1	1	101.1506	1	1
CRYL1	1	60.99902	90.92892	1	1	104.0932	1	1	1	1	1	35.51633	35.41332	1	1	54.68771	1	1	1	1
CRYM	1	1	1	1	1	1	1	1	1504.127	1	1	1	1	1	1	1	1	1	1	1
CRYZ	1	1	1	1	1	42.37898	1	1	1	1	1	27.11212	26.22246	1	1	1	1	1	1	1
CRYZL1	1	38.41092	1	86.97639	89.69576	65.17826	1	1	1	50.03835	83.32769	43.79665	51.07748	76.16306	77.99189	54.77518	72.13764	1	68.52259	70.02264
CSAD	1	33.57074	51.11487	83.47471	73.67227	32.45283	1	44.90021	143.7321	33.46148	140.4605	41.17245	32.2592	70.56698	59.47234	1	296.0176	38.6387	68.11591	116.5001
CSF1	329.833	1	1	1	1	84.9329	1	1	1	1	1	1	106.9336	1	1	1	1	1	1	1
CSF1R	259.8616	1	1	1	1	1	1	1	1	1	1	1	1	1	1	1	1	1	1	1
CSF2RA	208.4769	1	1	1	1	1	1	1	1	1	1	1	1	1	1	1	1	1	1	1
CSF3R	79.53752	1	1	1	1	1	1	1	1	1	1	1	1	1	1	1	1	1	1	1
CSGALNACT	40.25506	1	45.34714	1	1	1	1	1	1	1	1	1	1	1	1	1	1	30.31197	1	1
CSK	65.27618	1	1	1	1	1	1	30.7818	1	1	1	1	1	1	1	1	1	1	1	1
CSMD1	1	1	45.84258	1	1	1	1	1	1	1	1	1	1	1	1	1	1	1	1	1
CSMD2	1	1	1	1	1	33.29962	1	1	159.0645	1	1	1	1	1	1	1	94.38453	1	1	1
CSMD3	1	1	82.7266	1	1	1	1	1	1	1	86.86111	1	1	1	1	1	1	1	1	1
CSNK1G1	1	28.08098	1	50.61825	41.2475	28.13808	1	1	1	40.07325	1	22.23267	30.14109	1	38.27635	1	1	1	1	1
CSNK1G2	1	32.61449	50.18088	45.19855	51.65122	50.75014	1	59.34916	75.69992	62.59526	1	59.11839	57.11114	1	1	1	144.7599	124.6984	46.37016	70.02264
CSPG4	1	1	82.46962	1	1	1	1	289.6499	1	1	1	1	1	1	1	1	1	1	1	1
CSPG5	1	415.6262	546.5858	1	1	362.2307	1	1	168.4303	43.05242	1	56.0305	149.7223	1	1	209.751	1	1	1	1
CSRNP1	140.5983	65.48593	1	1	1	62.46322	1	305.9676	1	1	1	29.36806	142.7067	1	1	1	1	248.3983	1	1
CSRNP2	1	1	1	1	53.9696	1	1	41.0672	48.05852	1	1	24.16611	24.23801	1	58.09659	1	1	1	1	1
CSRNP3	1	1	75.13191	104.5962	179.5371	1	235.1796	57.22074	355.3439	1	202.5129	1	27.49174	389.358	253.9879	1	254.2693	1	100.8699	1
CSRP1	1	1	1	1	1	36.59041	1	1	1	1	1	1	57.73895	1	1	1	1	90.52744	1	1
CSRP2	81.84184	108.2959	1	524.9767	700.7879	69.93815	1147.251	133.0978	234.1791	530.514	1	617.4304	227.9654	1	1276.602	178.1369	1204.453	968.3889	449.2584	1852.212
CST3	463.3079	3400.793	457.7911	93.89137	89.61612	2502.243	1	370.8549	1	78.6563	80.35554	560.1725	385.8604	79.46689	111.354	1480.11	1	207.1386	85.32749	308.945
CSTF1	1	1	1	1	1	1	1	1	1	37.53215	1	35.29968	1	1	1	1	1	1	1	1
CSTF2T	1	40.00677	37.48937	49.01761	47.0253	33.21037	1	1	1	44.71811	1	50.6353	31.43706	67.24295	1	1	1	36.64889	1	1
CTBS	57.94862	1	1	1	1	1	1	17.86228	1	1	1	1	1	1	1	1	1	23.79432	1	1
CTDSPL	1	1	1	1	1	27.74645	1	169.2116	1	1	1	1	29.56901	1	1	1	1	20.71177	1	1
CTGF	1	1	1	1	1	1	1	139.5343	1	1	1	1	308.2952	1	1	1	1	201.6248	1	1
CTH	1	1	1	1	1	1	1	1	1	1	1	1	29.50199	1	1	1	1	1	1	1
CTHRC1	1	1	85.6472	1	1	1	1	1	1	1	1	1	1	1	1	1	1	162.5892	1	1
CTIF	1	1	37.4368	53.66268	52.51939	1	1	1	1	1	1	1	1	1	1	1	120.1635	27.92224	71.22096	103.6483
CTNNA2	1	43.19981	28.45445	1	50.19627	1	1	1	1	1	84.91332	1	125.4367	83.25942	85.00863	1	1	1	1	103.6483
CTNNAL1	1	46.28096	64.5138	1	1	51.4088	1	56.53122	1	78.42623	1	77.85456	54.21523	1	1	90.42351	1	13.18994	1	1
CTNNBIP1	1	43.99295	43.22835	1	1	51.82022	1	1	1	63.19475	1	64.99678	29.68934	87.69689	1	60.06631	1	146.7235	1	1
CTPS1	1	1	1	1	1	1	1	1	1	46.603	1	39.86854	24.54278	1	1	1	1	22.98325	1	1
CTSA	104.238	1	1	1	1	1	1	61.99455	1	1	1	1	1	1	1	1	1	52.94279	1	1
CTSB	866.7122	62.40854	57.62292	44.03194	52.72337	75.85457	1	128.2063	61.1432	51.33779	1	53.26373	64.92177	1	1	62.7466	72.78664	128.3532	1	70.02264
CTSC	565.9895	1	1	1	1	1	1	1	1	1	1	1	1	1	1	1	1	1	1	1
CTSD	974.4807	62.39553	103.7347	1	1	48.67454	1	64.37277	1	38.0901	1	33.59656	50.1392	1	1	41.55293	1	114.6276	1	1
CTSF	1	60.69075	60.87331	47.36596	49.22389	62.99816	1	35.79174	1	1	1	24.37868	60.39439	1	1	1	1	26.00563	1	1
CTSH	112.4195	1	1	1	1	53.58286	1	1	1	1	1	1	122.5777	1	1	1	1	31.33083	1	1
CTSK	1	1	1	1	1	1	1	134.5261	1	1	1	1	1	1	1	1	1	1	1	1
CTSL	248.466	35.86801	1	1	1	64.5136	1	96.36799	1	1	1	1	50.87797	1	1	1	1	28.08053	1	70.02264
CTSS	213.0825	1	1	1	1	1	1	1	1	1	1	1	1	1	1	1	1	1	1	1
CTSZ	551.1815	1	1	1	1	42.18606	1	106.6389	1	1	1	1	36.31151	1	1	1	1	159.9197	1	1
CUEDC1	1	38.30222	54.92476	1	1	30.13358	1	1	1	1	1	1	27.13371	1	1	1	1	1	1	1
CUX2	1	1	1	88.34871	168.6419	1	195.1949	1	1	1	1	1	1	142.8411	258.9223	1	138.2229	1	202.5028	172.671
CX3CR1	471.378	1	1	1	1	1	1	1	1	1	1	1	1	1	1	1	1	1	1	1
CXADR	1	51.78475	336.9851	153.7989	323.6858	1	297.8943	1	767.2231	1	212.6476	28.86629	34.54625	212.769	344.7341	1	522.7917	1	285.0925	583.2643
CXCL12	1	1	1	1	1	55.97903	1	1	1	1	1	38.98986	395.8431	1	1	1	1	162.3315	1	1
CXCL14	1	65.32055	1	1	1	48.54469	1	1	1	1	1	1	1	1	1	1	1	1	1	1
CXCL16	137.7811	1	1	1	1	1	1	1	1	1	1	1	1	1	1	1	1	1	1	1
CXCL2	1	1	1	1	1	1	1	128.2544	1	1	1	1	1	1	1	1	1	28.64009	1	1
CXCL8	1768.428	1	1	1	1	1	1	1	1	1	1	1	1	1	1	1	1	34.05102	1	1
CXCR4	68.39591	1	1	1	1	64.51225	1	48.96664	1	1	306.5322	45.40125	71.37233	668.7409	1	1	1	76.16252	1	1
CXXC4	1	38.42329	42.52633	42.5263	1	56.69728	1	1	1	1	1	34.11626	35.46413	113.1836	1	1	1	1	1	1
CXorf21	75.70674	1	1	1	1	1	1	1	1	1	1	1	1	1	1	1	1	1	1	1
CXorf36	1	1	1	1	1	1	1	1	1	1	1	1	1	1	1	1	1	117.6002	1	1
CYB5R4	1	1	1	1	1	1	1	1	1	1	1	22.83833	1	1	1	1	1	1	38.4348	70.02264
CYBA	900.8101	98.70567	32.37432	1	1	89.8232	1	656.9919	1	48.9137	1	49.71227	115.8194	1	1	104.0774	1	222.4777	1	1
CYBB	147.022	1	1	1	1	1	1	1	1	1	1	1	1	1	1	1	1	1	1	1
CYLD	1	1	27.96532	44.99774	58.69456	29.99008	1	47.82705	86.04077	1	1	1	31.28425	1	1	1	113.4705	56.42388	1	70.02264
CYP2R1	1	1	1	1	1	1	1	1	1	1	1	1	1	1	1	73.93531	1	1	1	1
CYP7B1	1	1	1	1	1	42.47784	1	1	1	1	1	1	1	1	1	1	1	1	1	1
CYR61	1	425.5471	1	1	1	685.1092	1	447.5422	1	129.9767	1	311.107	1376.317	1	1	315.3777	1	156.9738	1	1
CYSLTR2	1	1	1	1	1	1	1	183.5028	1	1	1	1	1	1	1	1	1	1	1	1
CYSTM1	100.7523	1	1	1	1	71.70836	1	100.9642	1	1	1	29.81465	70.68953	1	1	1	1	34.50033	1	1
CYTH1	34.94092	1	37.94073	61.04909	49.76881	1	1	1	172.4389	1	81.45437	1	1	84.23316	56.89759	1	1	33.94991	56.58597	1
CYTH3	93.20862	1	1	1	1	1	1	538.2674	1	1	1	1	1	1	1	1	1	43.01825	1	1
CYTOR	1	1	1	1	1	1	1	938.1145	1	1	1	1	1	1	1	1	1	102.2271	1	1
CYYR1	1	1	1	1	1	1	1	1	1	47.45654	1	1	1	1	1	1	1	160.8427	1	1
D2HGDH	1	1	40.47154	1	1	27.92312	1	1	1	1	1	1	1	1	1	1	1	1	1	1
DAAM2	1	1	1	1	1	83.3428	1	142.0031	1	1	1	1	24.78175	1	1	1	1	1	1	1
DAB1	1	1	48.15351	1	86.65461	61.69166	1	1	78.0803	1	1	1	1	1	212.3878	78.28837	324.9315	1	1	139.0453
DAB2	189.1002	1	1	1	1	1	1	61.58364	1	1	1	1	1	1	1	1	1	328.6496	1	1
DAB2IP	1	1	1	1	1	1	1	1	1	1	1	1	1	1	1	1	1	40.37969	1	1
DACH1	1	54.72291	1	1	1	127.7035	1	36.031	1	150.3212	1	76.56274	94.27104	1	1	75.59942	1	114.4145	1	1
DACT1	1	29.49533	1	50.14973	105.1957	44.63401	155.0775	55.10882	95.22969	1	160.5785	1	1	353.086	194.6719	1	275.1171	1	58.92638	70.02264
DACT3	1	1	50.89663	1	63.41052	1	1	69.26222	54.15747	1	1	1	1	64.42037	1	1	1	47.50991	1	1
DALRD3	1	31.57305	1	1	1	41.53185	1	23.38914	91.44261	1	1	26.30209	80.4215	1	1	1	1	86.60619	1	1
DAP	1	1	53.13906	62.38264	1	37.82625	1	33.99923	150.2063	45.37807	1	48.85837	37.42157	1	1	1	1	62.11401	1	1
DAPK1	1	46.58511	1	1	42.36692	1	1	1	1	33.90758	144.8497	23.1834	1	99.6736	35.49963	1	1	1	45.58068	103.6483
DAPK2	1	1	1	1	1	1	1	1	1	76.05558	1	36.18567	49.59043	1	1	1	1	1	1	1
DAW1	1	1	1	1	1	1	1	1	1	1	1	1	29.74738	1	1	1	1	1	1	1
DBF4	1	44.18699	40.27465	1	1	31.73401	1	1	1	60.30376	1	190.433	61.08216	1	1	68.31619	1	21.16193	1	1
DBR1	1	1	1	1	1	31.88539	1	1	1	31.70661	1	30.05608	1	1	42.97138	1	1	1	1	1
DCAF1	1	1	1	1	1	1	1	1	1	22.53092	1	29.31784	1	1	71.205	1	1	31.23254	1	1
DCAF11	1	1	54.15642	1	1	25.08829	1	29.77319	1	35.63486	1	34.4411	1	1	1	1	95.21359	22.68799	1	172.671
DCAF15	1	1	1	1	1	1	1	1	1	1	1	46.23008	1	1	42.94406	1	1	26.50013	1	1
DCAF17	1	1	31.00664	1	1	1	1	1	1	1	1	1	1	1	1	1	1	1	1	1
DCAF6	1	38.8024	1	1	1	40.68028	1	33.41085	130.3467	1	1	24.25075	26.4671	1	1	1	1	22.50459	1	1
DCBLD2	1	33.84475	28.36364	1	1	27.08098	1	61.37731	1	49.02982	1	45.13843	1	61.94932	1	1	1	1	1	1
DCC	1	1	37.85678	1	77.8	1	1	1	1	1	1	1	1	1	1	1	1	1	1	1
DCDC2	1	1	1	1	1	1	1	1	1	1	1	1	55.62017	1	1	1	1	1	1	1

DCHS1	1	53.78014	1	91.24018	76.46723	32.70903	1	1	1	48.35082	67.92139	39.4347	1	73.77965	1	59.68605	1	44.73175	76.50424	1
DCLK2	1	152.5475	96.387	46.38817	49.57165	54.2511	1	1	1	33.10328	289.9016	36.52677	29.34511	210.8765	1	1	120.4008	60.87702	68.06204	1
DCLK3	1	1	1	1	1	1	1	32.93448	1	1	1	1	1	1	86.05951	1	1	1	1	1
DCLRE1C	1	39.94747	32.04744	1	51.04578	36.1201	1	1	1	35.26197	1	56.0224	44.44344	62.35558	77.51423	1	36.10813	1	123.3759	103.6483
DCN	1	1	1	1	1	1	1	971.36	1	1	1	1	1	1	1	1	1	1	1	70.02264
DCPS	1	1	1	1	1	19.62483	1	1	1	34.37712	1	37.42222	1	1	1	1	1	1	1	1
DCTN4	1	54.32726	48.90493	78.47176	69.15345	57.10014	1	30.75877	1	63.24547	73.94015	43.30304	58.88556	66.95576	1	43.12941	1	54.53657	1	87.58009
DCUN1D4	1	1	53.90462	49.86075	47.73448	28.65775	1	1	1	34.35693	1	32.25941	30.74379	57.93885	1	1	1	22.63721	1	1
DCX	210.7132	161.3457	246.0919	1158.356	1066.126	210.258	994.1082	1	516.3424	492.2431	2005.854	282.4573	107.1641	2804.838	951.3504	174.6923	481.1344	69.56133	1258.432	232.0002
DCXR	1	65.99951	78.38848	1	1	102.586	1	63.86289	1	66.72221	1	156.2109	71.21018	1	1	112.2186	1	38.34818	1	1
DDAH1	1	89.15935	192.0588	1	1	237.4578	1	1	1	39.5851	1	90.84539	194.2064	1	1	120.2795	1	1	1	1
DDHD2	1	1	32.70558	49.66919	44.54699	30.00659	1	1	1	1	61.96966	41.74896	30.70823	70.6078	44.11816	1	1	22.86812	1	1
DDIT3	156.507	111.8452	1	1	1	91.01842	1	93.14365	1	69.03839	1	65.92717	250.6203	1	1	1	1	199.5953	1	1
DDR2	1	1	1	1	1	32.46904	1	167.7882	1	1	1	25.48499	34.38983	1	1	1	1	1	1	1
DDX10	1	38.4393	63.46414	62.0641	58.73208	30.55922	1	31.61362	1	47.10639	1	51.88191	32.2596	1	78.70106	1	1	40.43117	45.14634	103.6483
DDX11	1	1	1	1	1	1	1	1	1	39.37228	1	39.76361	1	1	1	1	1	1	1	1
DDX19A	1	1	29.37377	1	1	32.75099	1	20.46237	1	33.76376	1	37.55164	46.05074	1	1	73.03842	1	1	1	1
DDX49	1	36.10826	61.11095	52.71313	53.41424	39.90081	1	33.8595	1	51.70289	69.22526	38.6085	36.38809	70.6827	68.5893	54.99701	247.0803	141.4015	1	1
DDX55	1	43.33469	51.93743	99.12261	66.15382	37.91536	1	27.32308	1	95.77211	1	84.8889	41.8962	1	53.75894	38.76649	1	24.65942	1	87.58009
DDX59	1	37.34951	1	1	1	24.8079	1	1	1	1	1	25.87057	28.98511	1	1	1	1	1	1	1
DEDD2	1	1	1	1	38.85371	1	1	1	1	1	1	1	1	1	49.29429	1	1	159.9694	1	1
DEGS2	1	1	1	1	1	1	1	1	1	1	1	1	1	1	1	1	1	49.56908	1	1
DENND1B	1	1	1	1	1	37.56416	1	1	1	42.2159	1	25.94611	1	1	1	1	1	1	1	1
DENND2A	1	1	1	1	1	1	1	80.43466	1	1	1	1	1	1	1	1	1	32.11913	1	1
DENND2C	1	1	1	1	1	1	1	1	1	1	1	1	1	1	1	1	1	63.77143	1	1
DENND3	1	1	1	1	1	1	1	1	1	1	1	1	1	1	1	1	1	45.55114	1	1
DENND4A	1	1	57.70308	1	1	1	1	44.05848	1	1	1	1	1	1	1	1	1	17.40658	1	70.02264
DENND4C	1	1	1	47.30407	1	37.01524	1	1	1	29.94349	1	42.11113	33.10718	1	1	1	1	47.41188	1	70.02264
DEPDC1	1	1	1	1	1	1	1	1	1	1	1	122.3527	1	1	1	1	1	1	1	1
DEPDC1B	1	1	1	1	1	1	1	1	1	1	1	96.3049	1	1	1	1	1	1	1	70.02264
DEPP1	1	1	1	1	1	1	1	1	1	1	1	1	1	1	1	1	1	454.8881	1	1
DEXI	1	1	34.85258	1	1	28.48698	1	30.22987	1	1	1	1	25.70125	1	1	1	1	30.48318	46.35526	1
DGCR2	1	32.90536	33.82482	1	1	32.82175	1	1	1	1	1	1	1	1	43.89432	1	1	1	1	1
DGCR8	1	53.80611	1	52.61007	52.57464	1	1	1	1	51.17316	69.1673	51.42939	34.1495	1	1	96.39917	1	1	72.55769	103.6483
DGKD	1	1	1	1	1	1	1	1	193.1316	1	1	1	1	1	1	1	1	1	1	1
DGKE	1	1	1	1	1	1	1	1	1	1	1	1	1	1	1	1	1	97.38844	1	103.6483
DGKH	1	1	1	1	1	1	1	89.9549	1	1	1	1	1	1	1	1	1	89.44635	1	1
DGKI	1	1	94.6224	1	1	1	1	1	121.6776	1	1	1	1	1	1	1	1	1	1	1
DGKZ	1	1	1	1	1	1	1	37.82699	1	1	1	29.72415	30.64113	1	71.05381	1	1	52.38326	75.31347	70.02264
DHCR7	1	1	109.0659	1	1	70.60666	1	1	1	31.60956	1	27.18909	42.18885	1	1	101.765	148.586	44.192	1	1
DHDDS	1	1	1	1	1	28.84723	1	1	1	1	1	1	1	1	1	1	1	1	1	1
DHFR	1	78.00229	59.54898	1	1	64.76428	1	1	1	258.3617	1	221.7552	107.6453	1	1	171.1139	1	1	1	1
DHRS13	1	1	35.13886	1	1	1	1	1	1	1	1	1	1	1	1	1	1	1	1	1
DHRS3	57.2824	1	1	1	1	1	1	1	1	1	1	1	1	1	1	1	1	67.42227	1	1
DHRS4	1	1	1	1	1	35.41581	1	29.75961	1	1	1	1	1	1	1	1	1	1	1	1
DHRS9	122.6831	1	1	1	1	1	1	1	1	1	1	1	1	1	1	1	1	1	1	1
DHRSX	1	54.32162	1	1	1	25.28191	1	1	1	1	1	29.86011	28.35291	1	1	1	1	1	1	1
DHTKD1	1	63.05549	1	1	1	46.37301	1	1	1	106.2621	1	89.64152	40.17904	1	1	61.05998	1	1	1	206.2967
DHX57	1	43.65333	1	60.19123	60.342	37.73104	1	1	57.78325	41.3406	69.39359	30.6523	42.88882	1	1	58.95831	91.26455	1	75.71498	1
DHX8	1	1	1	1	1	1	1	1	1	34.47587	1	41.65732	19.54113	1	1	1	1	1	112.3516	1
DIAPH1	1	1	46.10558	1	49.64234	1	1	1	1	37.6344	70.47053	64.02381	37.44148	65.87191	48.06359	1	1	13.79104	1	1
DIAPH2	1	1	46.74787	1	1	34.53826	1	70.01974	1	1	1	1	1	1	1	1	1	1	1	1
DIAPH3	1	1	1	1	1	1	1	1	1	46.39839	1	144.097	48.10128	1	1	1	1	1	1	1
DIO2	1	53.51543	1	1	1	226.5389	1	1	1	1	1	44.81473	1	1	1	73.31299	1	1	1	1
DIRAS1	1	1	1	1	1	1	1	1	1	1	1	1	1	1	53.7649	1	1	1	1	1
DIRC2	1	1	1	1	1	30.95823	1	1	1	1	1	1	1	1	1	1	1	1	1	1
DISP2	1	1	1	85.32811	112.0019	1	1	1	159.7676	1	1	1	1	56.63608	143.6907	1	167.9352	1	36.31472	103.6483
DISP3	1	1	1	164.1219	186.8979	1	1	1	1	39.34192	1	1	1	1	90.63352	1	136.8274	1	130.1412	116.5001
DKC1	1	60.31005	39.15313	1	44.76415	49.93098	1	1	1	67.62215	1	70.37471	59.42526	1	56.7116	51.40603	1	52.94244	94.78183	70.02264
DKK2	73.6887	1	1	1	1	1	1	1	1	1	1	1	1	1	1	1	1	1	1	1
DKK3	1	34.1187	1	1	45.20211	239.7812	1	158.5168	215.977	48.46249	1	62.74284	122.3194	1	1	132.439	1	1	1	1
DLC1	1	1	1	1	1	1	1	614.4823	148.7592	1	1	1	53.71337	1	1	1	1	346.6167	1	1
DLD	1	39.15049	58.69227	42.68807	59.65478	50.06125	1	51.28622	1	38.23291	1	37.73298	42.99045	62.61356	59.71595	1	1	29.20245	112.1418	1
DLEU2	1	1	1	43.70233	1	1	1	45.76528	1	75.38943	1	92.74934	1	1	1	1	1	1	1	70.02264
DLEU7	61.55095	1	1	1	1	1	1	1	1	1	1	1	1	1	1	1	1	1	1	1
DLG2	1	1	1	68.2717	107.9211	1	1	25.54947	288.3399	1	90.7537	1	1	1	164.0616	1	569.103	1	1	208.0679
DLG3	1	1	1	54.16171	57.88536	1	1	1	99.00645	32.20689	64.55268	30.4686	21.10337	68.25214	51.37059	1	1	1	95.40595	174.1602
DLG4	1	1	1	1	39.69598	1	1	1	1	1	1	1	23.08786	1	48.944	1	1	1	63.95512	1
DLG5	1	58.06777	1	61.78365	50.41382	67.79657	1	1	109.3108	63.74386	1	52.23297	45.00873	57.82813	1	1	1	24.58847	81.98293	70.02264
DLGAP1	1	62.5774	62.34263	129.2559	130.0049	36.68954	1	1	93.85859	77.24695	194.4392	40.91118	1	175.9731	69.79865	68.83887	1	1	148.4838	70.02264
DLGAP5	1	1	1	1	1	1	1	1	1	1	1	229.4831	1	1	1	1	1	1	1	1
DLL1	1	210.7278	151.2308	1	1	1	1	1	1	83.07585	1	67.40185	69.548	1	1	1	1	57.18577	1	1
DLL3	1	285.5543	497.6107	61.42714	51.21563	1	1	1	99.95362	147.8746	67.52052	1	1	63.26308	1	1	1	1	63.89676	1
DLL4	1	1	1	1	1	1	1	1	1	1	1	1	1	1	1	1	1	103.4714	1	1
DLX1	1	1	1	1	1	1	1	1	1	1	751.3707	1	1	357.5701	1	1	1	1	1	1
DLX2	1	1	1	1	1	1	1	1	1	1	729.9938	1	1	784.3352	1	1	1	1	1	1
DLX5	1	1	1	1	1	1	1	1	1	1	811.4701	1	1	798.5099	1	1	1	1	1	1
DLX6	1	1	1	1	1	1	1	1	1	1	144.9252	1	1	181.1241	1	1	1	1	1	1
DLX6.AS1	136.3784	1	1	1	1	1	1	1	1	1	5123.453	1	1	2762.351	1	1	1	1	1	1
DMAC2	1	36.76453	33.34612	55.48299	1	29.31898	1	1	1	55.75388	1	42.26235	31.03067	1	1	1	1	40.35257	1	1
DMAP1	1	55.16034	46.49611	57.8395	64.07724	54.54105	1	27.87982	1	54.94841	53.75225	42.4724	38.6818	1	73.80258	1	1	23.06627	73.30323	116.5001
DMD	1	43.41651	1	1	1	54.13074	1	99.12998	302.1598	1	1	37.04001	42.84413	1	1	1	1	1	1	206.2967
DMRTA2	1	45.49992	1	54.22316	1	101.7858	1	1	1	115.1262	1	117.9948	85.22715	1	1	83.28408	1	1	1	1
DNA2	1	1	1	1	1	1	1	1	1	52.67373	1	74.26517	1	1	1	1	1	1	1	1
DNAAF1	1	1	1	1	1	1	1	1	1	1	1	1	98.55432	1	1	1	1	1	1	1
DNAAF2	1	45.15995	37.55411	51.36712	58.8775	37.72502	1	31.79815	1	54.64243	1	44.66243	46.01068	1	1	1	1	121.7463	98.95228	70.02264
DNAAF4	1	46.05804	1	1	1	42.64777	1	1	1	1	1	1	103.8879	1	1	1	1	1	1	1
DNAAF5	1	1	1	1	1	1	1	1	1	1	1	1	28.1087	1	1	1	1	1	1	1
DNAH10	1	1	1	1	41.88013	1	1	1	1	1	1	1	1	1	1	1	1	1	1	103.6483
DNAH11	1	1	1	1	1	1	1	1	1	1	1	1	38.21309	1	1	1	1	1	1	1
DNAH6	1	1	1	1	1	1	1	1	1	1	1	1	32.84804	1	1	1	1	1	1	1
DNAH7	1	1	1	1	1	1	1	1	1	1	1	1	68.50213	1	1	1	1	1	1	1
DNAH9	1	1	1	1	1	1	1	1	1	1	1	1	51.31855	1	1	1	1	1	1	1
DNAJA3	1	1	50.02202	1	1	1	1	1	1	1	1	27.5652	25.89912	1	1	1	1	1	1	1
DNAJA4	102.3018	1	1	1	1	1	1	84.29299	1	1	1	1	1	1	1	1	1	41.96212	1	1
DNAJB12	1	38.97904	33.00201	1	1	41.00139	1	33.34835	1	48.34121	1	39.89788	32.25163	1	1	1	55.83311	16.4448	1	70.02264
DNAJB2	1	63.71018	97.09151	42.36566	44.13097	77.15342	1	41.14742	1	30.43428	71.47847	35.91587	56.70748	1	1	41.10549	1	39.18206	1	1
DNAJB4	95.16606	49.66774	1	1	1	41.46552	1	623.2008	89.91799	1	1	35.38111	47.84773	1	1	1	1	169.9099	1	1
DNAJB5	1	1	29.83013	1	47.22488	32.04442	1	1	1	29.4868	1	1	24.37359	1	1	1	1	1	1	70.02264
DNAJC10	93.1475	41.40117	35.56561	1	1	64.34454	1	77.18954	1	50.08537	1	45.22464	75.33266	1	1	41.63265	151.6322	50.7234	1	70.02264
DNAJC11	1	1	1	1	1	1	1	1	1	1	1	1	1	1	1	1	1	1	61.58997	1

DNAJC12	1	1	1	1	1	1	1	66.65814	1	1	1	1	1	1	1	1	1	1	1	1
DNAJC17	1	1	72.25515	1	1	1	1	1	1	1	1	1	1	1	1	1	1	15.1098	1	103.6483
DNAJC24	1	36.59151	1	1	1	38.63584	1	1	1	1	1	29.34032	23.52141	1	1	59.36787	1	24.19583	1	1
DNAJC25	1	1	1	1	1	1	1	1	1	1	1	1	49.28034	1	1	1	1	23.84289	1	1
DNAJC27	1	44.26337	1	1	1	1	1	1	1	1	1	43.66822	34.13423	1	1	1	1	56.82009	1	1
DNAJC30	1	1	1	1	1	1	1	1	1	1	1	1	25.27235	1	1	1	1	1	1	103.6483
DNAJC4	68.84015	32.42266	1	53.45569	1	34.84468	1	63.34167	1	1	1	1	29.32446	1	1	1	1	39.21352	1	70.02264
DNAJC6	1	1	1	1	1	1	1	1	117.9223	1	1	1	1	1	1	1	1	1	1	172.671
DNAJC9	1	43.99028	1	1	44.94183	45.18584	1	1	1	138.6007	1	149.4582	47.3572	1	46.12172	89.69906	1	17.88022	1	1
DNAL1	1	1	1	1	1	43.1326	1	1	1	1	1	27.102	61.28968	1	1	73.20657	1	25.25354	1	1
DNALI1	1	34.477	1	1	1	29.34276	1	1	1	1	1	30.66471	154.4057	1	1	1	1	1	1	1
DNASE1	1	46.10864	1	1	1	32.79058	1	1	1	1	1	31.01595	1	1	1	1	1	1	1	1
DNASE2	208.2324	1	1	1	1	23.89631	1	1	1	1	1	1	25.76548	1	1	1	1	1	1	1
DNM1	1	1	32.58509	1	70.0873	1	1	1	222.36	1	1	1	1	1	82.8365	1	261.7231	1	95.8892	103.6483
DNM3	1	1	247.5691	1	1	1	1	1	315.2809	1	1	1	1	101.6421	72.37082	1	1	1	1	1
DNMT3A	1	42.85813	50.88851	1	40.47636	37.81532	1	33.08893	1	36.55633	96.84227	34.13309	27.15095	105.0904	71.31641	1	1	31.32098	85.06514	1
DNPEP	1	43.99436	1	1	1	35.71829	1	32.87531	1	1	1	1	38.58505	1	1	1	1	42.15132	1	1
DNPH1	170.9604	132.5385	71.7306	1	1	107.7025	1	48.54441	55.98274	41.40795	1	50.55175	137.3962	1	1	123.5306	1	1	1	1
DNTTIP1	1	44.59688	1	1	1	34.22542	1	35.665	1	47.49309	1	44.06287	48.01692	1	1	1	1	1	1	1
DOC2B	1	1	1	1	1	1	1	1	230.0692	1	1	1	1	1	1	1	1	20.77781	1	1
DOCK10	47.75702	1	91.23956	1	1	1	1	48.48427	1	1	1	1	1	1	1	1	1	1	1	1
DOCK2	66.63601	1	1	1	1	1	1	1	1	1	1	1	1	1	1	1	1	1	1	1
DOCK3	1	1	1	1	1	1	1	1	85.68531	1	1	1	1	1	1	1	1	1	1	1
DOCK4	150.0274	52.92121	59.63652	93.71257	92.08822	1	1	1	276.9031	1	1	1	1	1	102.5544	1	1	43.89161	95.69356	1
DOCK6	1	1	1	1	1	1	1	91.65546	1	1	1	1	1	1	1	1	1	31.76601	1	1
DOCK8	160.1344	1	1	1	1	1	1	1	1	1	1	1	1	1	1	1	1	1	1	1
DOCK9	1	1	1	1	1	1	1	1	332.9539	1	1	1	1	1	1	1	348.7852	174.2626	1	1
DOK4	1	1	1	1	1	1	1	1	1	1	1	1	1	1	1	1	1	81.89928	1	1
DOK5	1	81.40673	1	1	175.9472	630.5835	410.2059	1	1	58.54981	1	159.2048	317.2159	1	675.1233	486.6802	656.3782	99.56925	1	415.1358
DOK6	1	1	1	367.0273	299.7978	1	233.8587	54.76638	276.5107	43.73314	1	56.81338	1	1	130.0973	68.08148	1	107.3081	357.5587	241.6936
DONSON	1	1	1	1	1	1	1	1	1	1	1	27.34986	1	1	1	1	1	1	1	1
DOPEY1	1	1	49.49612	1	1	1	1	1	1	1	1	1	1	1	38.86156	1	1	23.65094	1	1
DOPEY2	1	1	1	72.13094	84.56226	1	1	1	130.231	1	1	1	1	1	127.8995	1	116.1722	59.61651	122.7224	139.0453
DOT1L	1	1	1	1	1	1	1	1	1	42.61165	1	53.82394	25.98685	1	1	1	1	28.56372	1	1
DPCD	1	1	1	1	1	26.71703	1	25.41527	1	1	1	1	60.73512	1	1	1	1	1	1	1
DPEP1	1	1	1	1	1	1	1	1	1	1	1	1	1	1	1	1	1	404.6826	1	1
DPF1	1	1	80.71999	1	1	1	1	27.70801	238.7093	40.86414	91.45409	44.4446	1	169.4535	64.13123	1	86.24629	1	1	87.58009
DPF3	1	94.01738	1	1	1	1	1	1	1	1	1	1	1	1	1	1	1	1	1	1
DPH7	1	32.02281	53.83701	1	1	29.01898	1	1	1	29.05261	1	29.01261	40.6219	1	41.82124	1	1	28.4534	1	1
DPP10	1	1	99.79789	1	1	1	1	1	167.7499	1	1	1	1	1	1	1	1	1	1	1
DPP6	1	74.79204	197.4598	72.42873	74.08779	1	1	1	1	1	98.19195	1	40.21829	1	47.36415	1	59.05088	1	73.36881	1
DPP7	215.7636	67.74115	65.93029	1	1	64.82881	1	24.67712	1	1	1	25.87335	86.08943	1	1	50.70631	1	50.20157	1	1
DPY19L1	1	32.89661	1	145.9325	198.4931	39.52692	137.3712	36.35003	1	1	1	37.27125	47.54227	1	185.5753	1	125.425	45.67778	272.472	206.2967
DPY19L2	1	1	1	1	1	1	1	23.27298	101.3703	1	1	1	1	1	1	1	1	1	1	1
DPY19L4	1	41.47556	1	1	1	51.86195	1	37.97624	1	27.39723	1	45.40398	33.51246	1	1	53.3756	1	16.91212	1	1
DRAXIN	1	1	1	101.3978	156.8222	1	161.6044	1	1	1	270.6895	1	1	185.6247	178.0258	1	221.0385	1	113.2305	232.0002
DRC1	1	1	1	1	1	1	1	1	1	1	1	1	73.8077	1	1	1	1	1	1	1
DRC3	1	1	1	1	1	1	1	1	1	1	1	1	42.4727	1	1	1	1	1	1	1
DROSHA	1	1	1	1	1	1	1	1	1	1	1	1	1	1	1	1	1	1	32.48191	1
DSCAM	1	1	52.70608	1	54.21479	1	1	1	91.47112	1	1	1	1	1	71.63903	1	1	1	1	1
DSCAML1	1	1	49.84367	1	1	1	1	1	121.8697	1	89.25861	1	1	133.4875	1	1	1	1	1	103.6483
DSE	1	1	1	1	1	1	1	70.98655	1	1	1	1	28.70563	1	1	1	1	1	1	70.02264
DSG2	1	1	1	1	1	26.81892	1	1	1	1	1	1	1	1	1	1	1	1	1	1
DSN1	1	1	1	1	1	1	1	1	1	56.74749	1	61.48799	1	1	1	1	1	1	1	1
DSP	1	1	1	1	1	1	1	1	1	1	1	1	1	1	1	1	1	96.44795	1	1
DTD2	1	1	1	1	1	37.486	1	1	1	1	1	1	1	1	1	1	1	1	1	1
DTL	1	1	1	1	1	1	1	1	1	94.76253	1	80.06535	1	1	1	1	1	1	1	1
DTNA	1	134.632	53.65939	1	1	129.3728	1	1	1	1	1	29.1134	117.9915	1	1	114.5826	1	1	1	1
DTX1	1	1	1	1	1	1	1	1	1	1	1	1	1	1	50.46126	1	1	1	1	70.02264
DTX4	1	1	1	1	1	38.15089	1	1	1	1	1	1	26.61368	1	1	1	1	1	1	1
DTYMK	1	44.63408	54.22412	1	1	53.0462	1	26.62653	1	89.86576	1	209.7204	55.40827	1	55.21418	91.67045	1	40.6999	1	1
DUOX1	1	1	1	1	1	1	1	1	1	1	1	1	1	1	1	1	1	1	53.20259	1
DUSP10	1	105.6312	42.74821	63.03875	1	49.24981	1	1	1	110.6383	1	97.37449	116.2566	1	1	1	1	38.18215	1	1
DUSP14	1	1	1	65.32577	69.95094	36.36446	1	141.1642	1	1	1	1	33.45014	1	1	1	1	36.89403	60.68379	103.6483
DUSP16	1	1	1	56.12485	52.92467	32.36927	1	1	1	31.60953	1	49.89434	54.02135	1	1	1	1	1	1	1
DUSP18	1	1	1	1	1	1	1	1	1	1	1	1	35.8365	1	1	1	49.9934	1	1	1
DUSP2	608.4855	1	1	1	1	1	1	1	1	1	1	1	1	1	1	1	1	1	1	1
DUSP23	50.74141	1	1	1	1	1	1	1	1	1	1	1	1	1	1	1	1	18.87733	1	1
DUSP26	1	1	1	1	48.09392	1	1	1	1	1	65.98582	1	1	64.78107	1	1	1	1	1	1
DUSP4	1	1	1	56.96172	83.47191	1	1	1	1	1	77.82048	1	1	193.4784	1	1	1	29.78425	1	70.02264
DUSP5	1	1	1	1	1	1	1	249.3504	1	1	1	1	1	1	1	1	1	240.5265	1	1
DUSP6	63.95025	190.2681	146.2641	1	1	1	1	60.60739	1	1	1	1	1	1	1	1	1	698.4095	1	1
DVL2	1	39.41786	1	1	1	32.19607	1	1	1	41.11249	1	37.32142	28.13996	1	1	42.50545	1	1	1	1
DVL3	1	60.39297	50.27012	83.25898	65.25308	48.54108	1	60.1437	58.95906	57.31264	1	61.21412	55.07386	1	84.95186	40.71641	1	43.1065	180.2294	1
DYDC2	1	1	1	1	1	1	1	1	1	1	1	1	46.39973	1	1	1	1	1	1	1
DYM	1	1	1	1	44.7976	22.1154	1	1	1	45.91759	1	41.37851	30.39002	1	1	1	1	27.55665	56.50752	103.6483
DYNC1I1	1	1	1	1	1	1	1	1	215.3796	1	1	1	1	1	83.24458	1	100.1111	1	1	190.2284
DYNC2H1	1	43.06787	1	1	1	51.63541	1	35.15927	83.76027	1	1	42.88873	111.5536	1	1	1	1	1	1	1
DYNC2LI1	1	49.90102	1	1	1	28.93315	1	1	1	1	1	1	56.07701	1	1	1	1	1	1	1
DYNLRB2	1	1	1	1	1	1	1	1	1	1	1	1	66.18637	1	1	1	1	1	1	1
DYNLT3	78.30571	1	1	1	1	1	1	154.4338	1	1	1	1	1	1	1	1	1	42.10499	1	103.6483
DYRK1B	1	1	1	1	1	1	1	1	1	1	1	1	32.23903	1	1	1	1	1	1	1
E2F1	1	1	1	1	1	1	1	1	1	114.4067	1	46.8558	1	1	1	29.75349	1	1	1	1
E2F2	1	1	1	1	1	1	1	1	1	1	1	36.56218	1	1	1	1	1	1	1	1
E2F3	1	33.61929	90.53058	1	1	41.57035	1	1	1	94.74858	1	87.02666	1	1	1	74.66781	1	30.20147	1	1
E2F5	1	61.00605	39.20738	1	1	104.4693	1	1	1	48.27735	1	60.60111	126.4324	1	1	136.7817	1	1	1	1
E2F7	1	1	1	1	1	1	1	1	1	1	1	58.4564	1	1	1	1	1	1	1	1
E2F8	1	1	1	1	1	1	1	1	1	1	1	38.03034	1	1	1	1	1	1	1	1
EBF1	1	1	1	1	1	1	1	279.0241	1	1	1	1	1	1	1	1	1	91.87324	1	1
EBF4	1	1	1	1	1	1	1	26.06469	1	33.21255	1	26.04697	1	1	1	1	1	1	1	1
EBNA1BP2	1	66.06367	59.63696	63.17635	65.4833	59.61653	123.2906	59.64081	71.45168	62.88594	1	63.45157	82.54219	1	91.02143	64.38844	1	27.29328	99.17545	1
EBP	1	1	75.54328	1	56.96337	38.74821	1	1	51.78815	57.31019	1	51.55601	24.14088	1	95.29446	68.07879	1	1	22.07964	70.02264
ECD	1	38.79422	1	1	42.5878	1	1	32.16176	1	34.95681	1	32.2696	24.14464	64.4102	1	1	1	53.80677	1	1
ECE1	1	1	1	1	1	1	1	219.723	1	1	1	1	1	1	1	1	1	191.3553	1	1
ECHDC2	1	1	1	1	1	52.41595	1	1	1	1	1	33.40549	47.19888	1	1	1	1	1	1	1
ECI1	80.34155	125.2438	65.92347	46.74161	1	111.2267	1	55.51152	1	140.3137	1	141.8926	100.2746	1	1	164.1426	1	35.95554	1	1
ECM1	1	1	1	1	1	1	1	70.1126	1	1	1	1	1	1	1	1	1	110.4381	1	1
ECM2	1	1	1	1	1	1	1	58.86974	1	1	1	1	1	1	1	1	1	1	1	1
ECPAS	1	1	1	1	1	27.30415	1	1	65.64831	1	1	20.35428	22.16699	1	1	1	1	99.18031	1	1
ECSCR	1	1	1	1	1	1	1	45.06094	1	1	1	1	1	1	1	1	1	693.8972	1	1
ECSIT	1	41.3561	100.2638	57.50426	43.79561	33.17092	1	1	1	40.74759	1	34.18997	31.98804	1	1	1	1	1	39.02128	1

ECT2	1	1	1	1	1	1	1	1	1	27.73126	1	246.3202	37.10527	1	1	1	1	1	1	1
EDEM3	1	1	1	1	1	26.51967	1	38.38941	51.53247	1	1	23.63997	24.13705	1	1	1	107.4827	36.09496	1	70.02264
EDIL3	1	1	85.15519	1	48.72605	1	1	174.5013	1	1	1	1	1	1	86.35717	1	1	1	1	1
EDN1	1	1	1	1	1	1	1	1	1	1	1	1	1	1	1	1	1	223.1013	1	1
EDN3	1	1	1	1	1	1	1	1	1	1	1	1	1	1	1	1	1	59.79242	1	1
EDNRA	1	1	1	1	1	1	1	878.0441	1	1	1	1	1	1	1	1	1	1	1	1
EDNRB	1	94.64757	177.0841	1	1	93.34105	1	57.38725	1	1	1	1	32.145	1	1	1	1	1	1	1
EDRF1	1	34.1103	1	48.17198	49.9807	25.24535	1	1	1	75.01374	1	44.40123	27.17294	1	1	1	101.123	1	78.48264	70.02264
EEF1A2	1	1	147.2458	88.05762	182.9485	1	290.745	1	286.4669	1	1	1	1	1	282.2109	1	384.2848	1	290.4576	172.671
EEF2K	1	1	28.00472	1	1	1	1	1	1	1	1	1	1	1	1	1	1	1	1	1
EEPD1	1	1	1	1	1	126.058	1	1	1	1	1	1	31.75717	1	1	47.51941	1	1	1	1
EFCAB1	1	1	1	1	1	1	1	1	1	1	1	1	66.65539	1	1	1	1	1	1	1
EFCAB2	1	1	1	1	1	1	1	1	1	1	1	1	41.48926	1	1	1	1	16.32658	1	1
EFCAB7	1	1	1	1	1	25.45313	1	1	91.0387	36.4497	1	28.25746	39.39748	1	1	1	26.88605	1	1	1
EFEMP2	1	50.42525	27.27859	1	1	74.05396	1	244.1952	1	1	1	36.40813	70.45184	1	1	41.48162	1	47.11715	1	1
EFHC1	1	33.77426	1	1	1	30.61683	1	29.74403	85.17355	1	1	27.70165	147.8363	1	1	1	1	31.12193	1	1
EFHC2	1	1	1	1	1	1	1	1	1	1	1	1	31.47507	1	1	1	1	1	1	1
EFHD1	1	1	1	1	1	51.40753	1	1	1	1	1	1	76.35727	1	1	1	1	1	1	1
EFHD2	274.0227	103.8986	1	1	1	60.12807	1	216.3315	1	42.66819	1	35.37469	53.26485	1	1	84.18579	1	1	1	1
EFNA1	1	1	1	1	1	1	1	31.54305	1	1	1	1	1	1	1	1	1	236.9083	1	1
EFNA5	1	1	1	1	1	1	1	1	153.7886	1	1	1	1	1	1	1	1	1	1	1
EFNB1	1	34.46322	1	1	51.15652	75.61568	1	1	1	44.15424	1	42.52474	36.81834	83.2294	1	1	1	1	1	1
EFNB3	1	1	1	55.69384	61.19904	30.30374	1	1	1	1	66.97882	1	36.31052	1	51.25789	1	1	1	1	1
EFR3A	1	1	1	1	1	27.0605	1	52.32265	1	1	1	1	1	1	1	1	59.67434	46.38581	1	1
EFR3B	1	1	1	1	1	44.5192	1	1	1	1	1	27.52872	31.04141	1	65.70752	1	1	75.72002	1	1
EFS	1	39.00337	59.31569	44.12634	1	29.49342	1	1	1	114.7127	1	61.10385	24.68215	1	1	1	1	1	53.36619	1
EGFL7	66.37235	1	1	1	1	1	1	180.8686	1	1	1	1	1	1	1	1	1	681.1517	1	1
EGFR	1	680.2992	1	1	1	70.85707	1	33.79629	1	108.5913	1	123.2996	171.8249	1	1	54.25538	1	1	1	1
EGLN3	1	1	48.66586	1	1	100.1673	1	1	1	1	1	38.06841	95.16836	1	1	1	1	1	1	1
EGR2	120.47	56.65918	1	1	1	1	1	53.17712	1	1	1	1	61.70626	1	1	1	1	23.564	1	1
EGR3	136.5434	61.90294	1	1	61.1435	1	1	53.66247	1	1	73.33659	1	69.99943	1	1	1	1	30.07397	1	1
EHD1	1	35.07114	61.94435	1	1	32.09122	1	32.97851	1	1	1	26.33659	1	1	1	1	1	15.76919	1	1
EHD2	1	1	1	1	1	1	1	96.45624	1	1	1	1	1	1	1	1	1	1	1	1
EHD3	1	1	34.43718	1	1	1	1	1	1	1	1	1	1	1	1	1	1	1	1	1
EHD4	88.30987	1	1	1	1	1	1	1	1	1	1	1	1	1	1	1	1	149.2126	1	1
EID2B	1	1	38.67032	1	1	1	1	1	1	38.61204	1	24.15948	42.91536	1	1	1	1	1	1	1
EIF2AK4	1	44.22247	55.97568	47.4919	53.69615	55.98064	1	40.2552	1	45.2312	1	45.46095	44.06078	1	61.17668	1	36.90931	20.59277	1	70.02264
EIF2B2	1	35.76372	1	1	1	37.49675	1	65.25863	1	27.63516	1	30.02562	36.73036	1	1	1	1	44.92784	1	87.58009
EIF2B3	1	1	42.64824	1	44.16348	1	1	33.88646	1	29.30775	1	25.21179	1	1	1	1	1	27.36044	1	70.02264
EIF2B4	1	1	44.57452	1	1	34.56839	1	1	1	55.34357	65.30133	35.78733	39.00115	1	1	1	1	1	1	1
EIF3J.AS1	1	51.13127	65.09035	1	1	71.65312	1	1	1	1	1	38.94031	50.66726	1	1	53.60301	85.62927	1	1	70.02264
EIF4ENIF1	1	1	1	1	1	27.38806	1	29.96548	1	34.3408	1	23.18845	28.36539	1	47.56305	1	1	27.97279	1	1
EIF5A2	1	1	1	1	1	1	1	1	1	1	1	1	28.34201	1	1	1	1	1	1	1
EIPR1	1	1	39.95067	1	1	28.40417	1	1	1	35.5356	1	22.9717	29.97822	66.72291	1	1	1	98.65164	1	1
ELAVL2	1	36.49599	1	426.3245	308.8519	1	277.4651	1	137.3852	365.3371	210.7689	138.8914	1	324.2223	121.0466	39.61345	83.8833	1	454.1956	1
ELF1	248.8581	44.23318	1	1	1	26.02878	1	51.65469	1	1	1	1	37.32557	1	1	1	1	85.97677	1	1
ELFN1	1	1	1	1	1	1	1	1	1	1	1	1	1	155.173	1	1	1	15.67209	1	1
ELFN2	1	1	60.58315	1	1	1	1	1	1	1	1	1	1	1	1	1	1	1	1	103.6483
ELK1	1	1	1	1	1	1	1	30.38656	1	1	1	1	1	1	1	1	1	36.48739	121.2214	1
ELK3	88.47773	1	1	1	1	1	1	34.38749	1	1	1	1	1	1	1	1	1	97.41315	1	1
ELL2	1	48.50662	36.57059	1	1	1	1	70.36996	1	1	1	1	29.45575	1	1	1	1	30.1023	1	1
ELMO1	1	1	92.29504	66.96868	1	1	1	1	1	54.80663	128.0465	38.83753	1	147.512	1	1	1	16.53062	1	1
ELMOD1	1	1	1	1	1	1	1	1	117.3098	1	1	1	1	1	1	1	150.2168	1	1	70.02264
ELN	1	1	1	1	1	1	1	1	1	1	1	1	1	1	1	1	1	111.788	1	1
ELOVL2	1	82.13094	1	1	1	136.7891	1	1	1	77.94782	1	79.81065	42.12279	1	1	59.71668	1	1	1	70.02264
ELOVL4	1	1	38.6192	1	49.18388	1	1	1	1	1	1	1	1	61.72757	46.81306	1	1	1	1	1
ELOVL5	1	89.99242	76.6053	1	1	85.93361	1	74.86633	1	54.3116	1	57.11471	80.27639	1	1	121.0099	1	147.9666	1	1
ELOVL6	1	1	40.61749	1	41.7612	45.37768	1	1	1	40.07794	145.2681	38.33812	1	1	71.66657	54.53012	99.60647	1	1	1
ELP1	1	1	1	1	1	1	1	1	1	1	1	28.73241	1	1	1	1	1	1	1	1
ELP4	1	56.77169	55.33862	1	1	54.88127	1	27.80205	1	64.11458	1	61.0493	51.48296	1	1	126.8219	1	1	1	1
EMC2	1	76.84997	1	1	1	66.83715	1	54.37599	1	28.39639	1	38.05935	41.30736	1	1	1	1	36.82608	1	70.02264
EMC8	1	52.58226	63.53809	1	1	62.61417	1	53.61905	1	44.58469	1	46.99675	47.34026	1	1	105.8307	1	65.24858	1	1
EMC9	1	1	1	1	1	1	1	1	1	31.30437	1	100.417	30.4544	1	1	1	1	33.97202	1	1
EMCN	1	1	1	1	1	1	1	52.97474	1	1	1	1	1	1	1	1	1	101.4977	1	1
EMID1	1	1	1	1	1	97.04375	1	1	1	45.50689	1	32.7337	64.05872	1	44.02481	114.6454	1	1	1	1
EMILIN1	1	1	1	1	1	1	1	120.026	1	1	1	1	1	1	1	1	1	1	1	1
EML1	1	1	1	110.6412	113.9118	31.02584	1	1	100.0679	113.5176	1	78.08815	29.92513	1	175.0529	48.32987	97.38396	33.97203	125.241	87.58009
EML6	1	1	1	1	1	1	1	1	1	1	1	1	1	1	1	1	1	1	66.45837	1
EMP1	1	1	1	1	1	1	1	1	1	1	1	1	1	1	1	1	1	260.5826	1	1
EMP2	1	45.49551	1	1	1	103.218	1	157.3494	1	64.30345	1	88.03644	67.31719	1	1	87.866	1	95.1215	1	1
EMP3	1	42.59079	1	1	1	59.44949	1	91.3949	1	1	1	48.69432	93.03953	1	1	1	1	1	1	1
EMSY	1	1	35.46059	57.98554	60.58627	37.49891	1	1	1	57.97747	68.70144	56.276	37.09297	67.82351	1	40.46337	1	1	68.65544	1
EMX1	1	1	1	98.64476	85.12017	43.00821	1	1	1	80.77305	1	44.80015	31.30158	1	87.61746	1	1	1	173.9243	103.6483
EMX2	1	146.7475	1	136.1927	119.935	129.3473	1	1	1	128.9527	1	137.4216	120.6543	1	1	87.36982	1	1	52.82672	70.02264
EMX2OS	1	35.81466	1	1	1	55.98369	1	1	1	1	1	1	35.93108	1	1	69.26142	1	1	1	1
ENC1	163.5821	175.7536	82.7131	1646.561	1499.714	231.7346	1747.467	48.95471	275.9218	874.8361	241.6023	539.6796	214.0048	305.1657	979.085	349.3741	473.722	371.7405	2049.195	379.7389
ENDOD1	1	1	1	1	1	1	1	54.4617	1	1	1	1	1	1	1	1	1	1	1	1
ENDOG	1	42.86053	65.62165	1	49.7481	41.41448	1	1	1	31.6304	1	31.08257	61.51595	1	87.12346	28.38794	1	23.13088	1	103.6483
ENG	1	1	1	1	1	1	1	153.8059	1	1	1	1	1	1	1	1	1	474.2917	1	1
ENKD1	1	1	1	1	1	1	1	1	1	1	1	1	33.15951	1	1	1	1	1	1	1
ENKUR	1	72.50097	1	1	1	50.97638	1	1	1	1	1	1	74.86302	1	1	1	1	1	1	1
ENO2	1	41.25022	72.20826	134.2394	149.216	57.46117	167.4892	1	215.4243	57.96534	142.8049	91.2314	47.37053	137.0328	164.3933	1	132.3043	1	119.3326	103.6483
ENOX1	1	1	1	1	1	1	1	1	1	48.79212	1	1	1	1	1	1	59.67434	1	1	1
ENPEP	1	1	1	1	1	1	1	87.59894	1	1	1	1	1	1	1	1	1	1	1	1
ENTPD1	110.1899	1	1	1	1	1	1	53.04076	1	1	1	1	1	1	1	1	1	94.97173	1	1
ENTPD4	1	32.20274	28.92937	1	39.82593	1	1	1	1	41.66666	1	28.90275	1	1	1	1	25.11449	27.89863	1	1
EOGT	1	1	1	1	1	1	1	1	1	1	1	1	1	1	1	1	1	65.97369	1	1
EOMES	1	1	1	343.8415	1	1	1	1	1	1109.831	1	587.8631	142.0424	1	1	1	1	1	102.4457	1
EPAS1	1	1	1	1	1	1	1	330.9038	1	1	1	1	1	1	1	1	1	426.3883	1	1
EPB41	1	1	46.22478	62.90944	75.46371	27.57132	1	1	1	61.62296	1	39.27392	32.27291	1	93.26815	1	107.4909	33.75303	127.2584	139.0453
EPB41L1	1	1	36.50214	1	1	1	1	211.6412	1	1	1	1	1	1	1	1	79.62927	1	1	1
EPB41L2	132.6603	50.89115	373.1405	1	1	31.32467	1	298.2666	1	1	1	41.96397	28.61634	1	1	1	1	31.01534	1	1
EPB41L3	83.94712	67.98684	1	106.0419	92.76393	123.5861	1	1	103.8993	1	1	42.99978	111.5776	1	132.9586	102.051	1	1	147.7476	206.2967
EPB41L4A	1	1	1	1	1	52.12895	1	29.80251	96.09659	25.72483	1	20.90468	33.62366	1	1	1	1	18.32858	1	1
EPB41L5	1	42.41871	1	52.06105	44.98165	39.76811	1	1	1	43.49107	1	39.30584	31.2068	1	51.92437	1	1	26.97535	41.42356	1
EPDR1	1	1	1	1	1	30.98291	1	1	1	1	1	1	28.1416	1	1	1	1	1	1	1
EPHA3	1	1	1	227.9582	255.6027	1	207.0968	1	1	93.77214	1	47.63302	1	143.8619	218.2418	1	462.8068	1	264.9658	1
EPHA4	1	46.81337	1	151.532	224.1364	38.27376	200.4903	1	106.6692	1	106.6979	25.29177	1	54.55613	184.012	1	117.1184	60.1139	198.2149	116.5001
EPHA5	1	1	1	198.0415	210.329	71.56906	171.7782	1	225.5852	41.09129	322.6954	37.19552	28.37944	184.8571	215.5796	61.94413	162.0491	1	228.4843	87.58009
EPHA7	1	1	1	1	1	1	1	1	69.50695	1	1	1	1	1	1	1	1	1	85.74163	1
EPHB1	1	1	126.1038	139.8663	135.5424	57.98478	1	1	109.4161	70.83964	1	51.07735	58.67996	1	139.8096	84.24856	223.7104	1	56.63825	1

EPHB2	1	39.26712	1	88.10901	65.95263	65.24444	1	1	1	69.89455	1	48.44286	28.22919	1	56.21777	1	1	1	119.0813	103.6483
EPHB6	1	1	1	150.9763	124.5209	1	1	1	1	32.2983	1	43.89421	1	1	70.73666	1	100.6652	1	161.1431	1
EPS15	1	35.56617	1	1	1	80.34431	1	36.72989	155.2698	33.45999	1	33.97133	60.18047	1	1	98.31151	1	59.354	1	1
EPS15L1	1	33.18812	36.56557	62.60949	56.99333	1	1	31.0981	61.76407	36.10097	1	30.83715	31.18342	58.75669	1	50.73068	1	1	1	1
EPSTI1	1	1	1	1	1	1	1	47.2193	1	1	1	1	1	1	1	1	1	1	1	1
ERBB2	1	1	1	1	1	37.00007	1	1	1	1	1	1	33.70445	1	1	1	1	1	1	1
ERBB3	1	1	98.26053	1	1	1	1	1	1	1	1	1	1	1	1	1	1	1	1	1
ERBB4	1	1	30.03592	1	1	1	1	1	1	1	1163.155	1	1	1300.929	1	1	1	1	1	1
ERC2	1	1	1	1	1	1	1	1	69.24657	1	92.55638	1	1	1	1	1	89.0427	1	1	1
ERCC3	1	1	1	1	1	1	1	28.57556	1	40.49183	1	1	24.67913	1	1	1	1	1	1	1
ERCC6L	1	1	1	1	1	1	1	1	1	1	1	28.50287	1	1	1	1	1	1	1	1
ERG	1	1	1	1	1	1	1	1	1	1	1	1	1	1	1	1	1	108.2769	1	1
ERI1	1	1	1	1	1	1	1	1	1	37.69433	1	31.88082	20.67358	1	1	1	1	1	1	1
ERI2	1	1	1	1	1	1	1	1	1	1	1	27.40594	1	1	1	1	1	1	1	1
ERICH3	1	1	1	1	1	1	1	1	112.2578	1	1	1	66.83067	1	1	1	1	1	1	1
ERLIN2	1	1	1	1	1	40.0972	1	1	1	1	1	1	35.52979	1	1	1	1	11.56798	1	1
ERO1A	1	1	29.13987	1	44.98522	1	1	88.94704	97.60517	41.13754	1	27.09949	1	1	71.07598	1	1	72.28588	1	206.2967
ERO1B	1	33.38087	1	1	1	1	1	87.52415	1	1	86.03489	1	1	97.92048	1	1	1	24.96584	1	103.6483
ERV3.1	1	32.7168	56.61494	69.37974	60.3186	1	1	1	1	45.07569	89.88125	27.58405	1	86.49596	76.97999	1	50.8802	1	53.84722	172.671
ERVK3.1	1	1	47.80729	1	1	1	1	1	1	40.26503	1	26.16083	1	1	1	1	1	25.72403	1	1
ESAM	1	1	1	1	1	1	1	174.5681	1	1	1	1	1	1	1	1	1	743.1395	1	1
ESCO2	1	1	1	1	1	1	1	1	1	62.18849	1	175.728	1	1	1	1	1	1	1	1
ESPL1	1	1	1	1	1	1	1	1	1	1	1	36.64071	1	1	1	1	1	1	1	1
ESYT2	1	1	1	1	42.0406	1	1	41.4632	1	1	1	1	1	1	83.89697	1	83.41069	93.25575	1	139.0453
ETAA1	1	1	1	1	1	26.04361	1	1	1	1	1	1	1	1	1	1	1	1	1	1
ETFDH	1	42.81958	34.85938	49.47013	55.95544	29.74263	1	1	1	34.20187	1	29.9522	24.43899	1	1	1	1	115.8311	1	1
ETS1	1	1	1	1	1	1	1	344.5386	1	1	1	1	1	1	1	1	1	179.6103	1	1
ETS2	60.68747	1	1	1	1	1	1	57.45743	1	1	1	1	1	1	1	1	1	420.2539	1	1
ETV1	1	294.0229	198.9288	1	1	163.6045	1	79.43067	1	35.74398	1	69.44591	1	1	1	77.94124	1	11.99867	1	1
ETV5	71.47294	158.9429	173.5129	1	1	52.09464	1	26.57527	110.2666	1	1	30.44298	1	1	1	1	119.1209	1	1	139.0453
ETV6	1	38.67307	1	1	1	1	1	50.42774	1	1	1	1	26.86374	1	1	1	1	66.17773	1	1
EVA1B	48.5106	1	1	1	1	1	1	463.2724	1	1	1	1	1	1	1	1	1	267.8256	1	1
EVA1C	1	1	1	1	1	27.53144	1	1	1	1	1	1	1	1	1	1	1	1	1	1
EVI2A	91.08283	1	82.77036	1	1	1	1	1	1	1	1	1	1	1	1	1	1	1	1	1
EVI2B	64.45505	1	1	1	1	1	1	1	1	1	1	1	1	1	1	1	1	1	1	1
EVI5	1	75.04213	96.86204	1	1	99.51529	1	75.00775	1	1	1	31.832	139.6281	1	1	1	1	35.40849	1	1
EXD2	1	29.95685	35.98337	1	1	34.64153	1	1	1	1	1	1	29.97988	1	1	1	1	1	1	1
EXO1	1	1	1	1	1	1	1	1	1	40.11487	1	27.58808	1	1	1	1	1	1	1	1
EXOG	1	1	1	1	1	1	1	1	1	39.81294	1	38.53416	1	1	1	1	137.0769	1	1	1
EXOSC2	1	1	1	1	1	1	1	1	1	47.31622	1	35.45625	24.74323	1	1	1	1	1	1	70.02264
EXOSC3	1	1	38.91884	1	1	1	1	1	1	37.41845	1	41.85797	28.75607	1	1	1	1	1	1	1
EXOSC5	1	34.41348	50.26344	57.95848	47.86334	1	1	1	1	53.83959	1	1	1	1	1	1	67.0686	35.07405	65.21108	70.02264
EXOSC7	1	41.35495	37.82555	1	39.72016	43.72498	1	1	1	52.82083	1	44.9113	36.47838	1	1	1	1	27.96653	1	103.6483
EXTL3	1	41.15235	1	1	1	24.99666	1	36.91172	1	1	1	23.77688	1	1	1	1	1	1	1	70.02264
EZH1	1	1	36.6148	56.13423	49.82339	37.25311	1	23.27137	1	39.13222	73.86901	27.39369	26.82412	60.03782	1	1	16.97423	29.22183	1	1
F11R	1	1	1	1	1	1	1	1	1	1	1	1	1	1	1	1	1	138.7396	1	1
F3	1	201.2843	1	1	1	263.2779	1	155.1957	1	52.50473	1	82.29826	179.6023	1	1	149.6621	186.337	1	1	1
FABP3	1	1	1	1	1	1	1	1	95.35707	1	1	1	1	1	1	1	1	1	1	139.0453
FADD	1	1	1	1	1	25.12549	1	1	1	1	1	1	40.93059	1	1	1	1	1	1	1
FADS3	1	1	1	1	1	1	1	23.11523	171.5081	1	1	1	1	1	1	1	1	1	1	1
FAF1	1	1	65.45582	1	42.1132	32.16531	1	1	1	41.26604	1	48.33627	35.83629	1	69.59988	1	74.48068	25.61511	1	1
FAH	78.76196	1	1	1	1	1	1	1	1	1	1	1	1	1	1	1	1	1	1	1
FAHD2A	1	1	1	1	1	32.79981	1	1	1	40.65316	1	30.91804	29.05643	1	1	76.1021	1	15.0974	1	1
FAIM	1	57.69013	1	1	1	73.24594	1	34.06025	1	37.77976	1	41.18377	53.2323	1	1	68.04004	1	1	1	1
FAIM2	1	1	40.77037	1	1	30.19149	1	1	1	1	1	1	44.85657	1	1	1	1	1	1	1
FAM102A	1	1	54.47289	1	1	1	1	1	1	1	1	1	1	1	1	1	1	1	1	1
FAM105A	156.5271	1	1	1	1	1	1	1	1	1	1	1	1	1	1	1	1	64.34787	1	1
FAM107A	1	63.37702	1	1	1	381.325	1	1	1	1	1	90.07183	40.07622	1	1	202.438	1	1	1	1
FAM110D	1	1	1	1	1	1	1	1	1	1	1	1	1	1	1	1	1	85.31534	1	70.02264
FAM111A	1	35.31157	1	1	1	36.93039	1	75.35295	1	97.04631	1	163.9718	63.14636	1	1	1	1	42.50069	1	1
FAM111B	1	1	1	1	1	1	1	1	1	103.9306	1	104.8698	1	1	1	1	1	1	1	1
FAM114A1	1	1	1	1	1	80.21895	1	79.79912	1	1	1	51.04829	42.57629	1	1	62.31933	1	37.30117	1	1
FAM117A	1	1	1	1	1	26.80874	1	1	1	1	1	1	43.06616	1	1	1	1	1	1	1
FAM118A	1	1	1	1	49.76289	32.30759	1	30.9574	1	1	1	28.05509	45.53819	1	1	1	1	40.42411	1	103.6483
FAM118B	1	1	1	1	1	36.99229	1	23.51198	1	1	1	24.41155	1	1	1	1	1	1	1	1
FAM120C	1	1	1	1	1	28.55159	1	1	1	1	1	1	1	1	1	1	1	1	1	1
FAM122A	1	1	37.40802	1	1	39.75657	1	27.21869	1	33.05481	63.84794	35.29289	30.16597	1	1	1	1	23.66326	1	70.02264
FAM122B	1	1	1	1	1	1	1	1	1	1	1	41.4999	1	1	1	1	1	1	1	1
FAM124A	1	1	1	1	1	1	1	1	1	1	1	1	1	1	60.89614	1	1	1	1	1
FAM124B	1	1	1	1	1	1	1	1	1	1	1	1	1	1	1	1	1	221.9215	1	1
FAM126A	1	50.20341	82.37248	194.196	193.7767	34.94473	1	50.1318	1	68.5526	1	46.5541	22.37956	1	87.01427	1	89.05268	77.74759	246.8613	116.5001
FAM126B	1	1	47.18032	55.53135	47.86581	1	1	1	112.8405	30.0601	1	31.55536	1	57.49533	41.89792	1	174.8758	1	1	1
FAM129B	1	1	1	1	1	1	1	84.1228	1	1	1	1	1	1	1	1	1	52.43245	1	1
FAM13C	1	1	49.69627	1	1	1	1	31.61252	1	1	1	1	1	1	1	1	1	1	1	1
FAM149A	1	1	1	53.95682	58.07897	1	1	1	1	1	1	1	1	1	1	1	1	1	1	1
FAM155A	1	1	43.08839	63.5046	77.25247	1	1	1	201.9276	1	1	1	1	1	1	1	1	1	1	1
FAM161A	1	81.48414	1	70.72923	61.77912	82.17651	1	1	79.32248	76.05259	71.48943	83.93287	97.86929	1	1	84.60744	1	1	68.45645	1
FAM162B	1	1	1	1	1	1	1	140.0511	1	1	1	1	1	1	1	1	1	1	1	1
FAM168A	1	56.9936	1	59.31938	71.03395	52.54608	1	44.7625	52.12843	65.37645	123.8858	62.22232	35.07498	70.95095	49.44442	77.02046	1	1	63.73223	1
FAM172A	1	63.70458	73.11425	87.15071	88.75729	79.49953	1	26.00604	167.1821	70.09191	123.9553	59.89063	50.47253	89.225	89.20543	109.4723	51.05561	64.35763	85.05773	310.7162
FAM181A	1	59.2735	1	1	1	1	1	1	1	1	1	1	43.64825	1	1	1	1	1	1	1
FAM181B	1	374.4609	179.5952	1	1	174.1301	1	1	1	1	1	70.95841	29.90809	1	1	78.95456	1	1	1	1
FAM183A	1	1	1	1	1	1	1	1	1	1	1	1	73.06783	1	1	1	1	1	1	1
FAM189A2	1	1	1	1	1	39.11	1	1	1	1	1	1	37.3517	1	1	1	1	20.28887	1	1
FAM193B	1	1	1	1	39.11641	1	1	1	1	1	1	24.46971	1	1	1	1	1	1	68.47062	1
FAM19A1	1	1	1	1	1	29.76166	1	1	1	1	1	1	1	1	1	1	247.4718	1	1	70.02264
FAM206A	1	1	1	1	1	1	1	1	1	1	1	1	31.474	1	1	1	1	1	1	1
FAM207A	1	45.20512	44.56161	1	1	1	1	1	1	38.77201	1	22.92907	28.10448	1	1	1	1	1	1	1
FAM20A	1	1	1	1	1	1	1	63.39574	1	1	1	1	1	1	1	1	1	1	1	1
FAM20B	1	1	1	1	1	1	1	1	1	37.41132	1	36.75149	1	1	1	1	1	1	1	70.02264
FAM20C	1	1	1	1	1	1	1	56.31636	1	1	1	1	1	1	1	1	1	1	1	1
FAM210A	1	33.18274	38.03401	1	1	39.35229	1	36.95453	1	1	1	38.80286	38.52619	108.9806	1	1	1	33.8495	1	1
FAM212A	64.96507	1	1	1	1	1	1	1	1	1	1	1	1	1	1	1	1	1	1	1
FAM214A	1	41.26556	36.50887	73.62335	53.56252	44.54931	1	26.45496	1	68.97794	142.2107	43.12119	52.5179	1	1	1	1	19.51445	1	70.02264
FAM216A	1	1	34.78927	1	1	1	1	1	1	1	1	1	24.50223	1	1	1	1	1	1	87.58009
FAM219A	1	32.41459	1	1	1	96.4894	1	1	1	40.04242	1	32.76037	36.90981	1	1	48.89727	1	1	1	1
FAM219B	1	28.7373	52.40785	67.0808	59.31245	38.29385	1	1	66.69845	52.31571	74.98937	26.79781	35.55282	70.37238	61.92938	44.95152	193.3988	1	1	70.02264
FAM221A	1	79.67604	61.41417	1	1	28.22739	1	1	1	41.16616	1	27.4719	39.88508	1	1	1	1	19.66187	1	1
FAM222A	1	1	30.51037	1	1	1	1	1	1	1	1	1	1	104.6115	1	1	1	1	1	1
FAM222B	1	38.49363	39.38528	56.92424	51.49714	1	1	24.0561	1	1	54.29216	29.1926	25.57182	1	1	1	1	26.00122	90.19149	1
FAM227A	1	1	1	1	1	30.95074	1	1	1	1	1	1	80.68617	1	1	1	1	1	1	1

FAM228B	1	31.96097	1	45.47877	54.97596	34.82162	1	1	1	1	1	1	31.97027	1	59.3802	1	1	1	70.84042	103.6483
FAM229B	1	52.13678	55.83238	1	1	67.17706	1	1	1	36.07068	88.52025	42.59255	130.7206	98.79555	72.05465	82.90217	36.14671	26.95737	1	70.02264
FAM241A	1	1	1	1	1	1	1	91.94447	1	1	1	1	1	1	1	1	1	41.54205	1	1
FAM241B	1	1	1	46.44322	60.49476	1	1	1	1	42.26605	1	1	1	59.89232	70.75041	1	222.8825	1	46.31392	70.02264
FAM43A	1	1	1	1	1	1	1	34.21046	1	1	1	1	1	1	1	1	1	185.7733	1	1
FAM46A	1	1	1	1	1	1	1	184.9048	1	1	1	1	1	1	1	1	1	1	1	1
FAM49A	1	1	1	1	1	46.27319	1	1	360.3991	1	1	1	1	1	97.18059	1	278.0397	1	1	70.02264
FAM53B	1	1	1	46.77056	42.42404	1	1	1	1	71.46041	1	53.09977	28.40967	1	1	1	1	1	60.60804	1
FAM57A	1	1	40.77269	1	1	1	1	1	1	1	1	1	1	1	1	1	1	1	1	1
FAM57B	1	1	1	106.4735	115.8568	1	172.6874	1	1	38.52035	1	1	1	76.00943	116.4502	1	67.0686	1	208.4548	103.6483
FAM66C	1	1	39.65289	1	1	1	1	1	1	1	1	1	1	1	1	1	1	1	1	1
FAM69B	1	1	1	1	1	35.25116	1	1	1	1	1	1	32.30547	1	1	1	1	151.4635	1	1
FAM69C	1	99.90643	41.98834	1	1	223.3665	1	1	1	1	1	30.18185	102.323	1	1	66.24744	1	1	1	1
FAM76A	1	1	1	1	1	37.81301	1	45.97637	1	1	1	30.01663	54.64102	1	61.38934	1	36.10813	1	1	1
FAM81A	1	1	1	1	1	1	1	1	112.3366	1	1	1	1	1	1	1	1	1	1	1
FAM83D	1	1	1	1	1	1	1	1	1	1	1	101.6191	1	1	1	1	1	1	1	1
FAM84A	1	69.53753	28.68187	1	1	166.1056	1	38.52045	1	1	1	1	31.29867	1	1	36.24162	1	80.72435	1	1
FAM84B	1	82.77223	1	1	1	115.2427	1	1	1	1	1	53.89903	226.1099	1	1	1	1	1	1	1
FAM89A	187.5574	1	1	1	1	1	1	32.29564	1	1	1	1	1	1	1	1	1	321.775	1	1
FAM8A1	1	30.18003	33.59736	50.08047	65.61772	34.28723	1	22.51095	1	1	61.68588	29.84783	43.22734	65.85134	1	1	1	32.09803	59.49446	1
FAM96A	51.88825	62.52169	101.6365	1	1	53.59142	1	1	1	54.72978	1	53.63617	38.77881	57.69537	1	44.06691	1	50.02503	1	1
FAN1	1	1	1	1	1	28.90731	1	1	1	1	1	1	1	1	1	1	1	1	1	1
FANCA	1	1	1	1	1	1	1	1	1	74.75393	1	53.76735	1	1	1	68.56865	1	1	1	1
FANCB	1	1	1	1	1	1	1	1	1	1	1	38.77514	1	1	1	1	1	1	1	1
FANCD2	1	1	1	1	1	1	1	1	1	32.22755	1	64.32988	1	1	1	65.55454	1	1	1	1
FANCF	1	1	1	66.77386	51.65631	31.57777	1	1	1	58.02515	1	38.4803	54.43306	1	43.98806	1	70.5831	106.0449	45.85936	103.6483
FANCG	1	1	1	1	1	1	1	1	1	40.94523	1	52.32462	1	1	1	1	1	1	1	1
FANCI	1	1	1	1	1	1	1	1	1	51.05281	1	94.74297	1	1	1	1	1	1	1	1
FANCL	1	38.61873	78.70899	1	51.05111	29.23782	1	1	1	67.59788	1	57.78251	1	1	65.80489	66.56628	1	1	1	103.6483
FANCM	1	1	1	1	1	1	1	1	1	33.71928	1	36.09546	1	1	1	1	1	1	1	1
FARP2	1	1	45.63606	1	1	1	1	48.65682	1	1	1	26.78776	1	1	1	1	1	1	1	103.6483
FARSA	1	48.15138	50.64093	1	39.36864	29.67074	1	1	1	49.44414	1	48.229	31.10307	1	57.15692	1	1	109.2028	77.08912	1
FARSB	1	1	34.5042	1	1	1	1	1	1	40.27143	1	25.84811	1	1	55.68959	1	1	1	1	1
FAS	1	1	1	1	1	31.64662	1	1	1	1	1	1	39.86146	1	1	1	1	1	1	1
FASTKD1	1	1	1	1	1	1	1	1	1	1	1	1	1	1	1	1	111.2901	1	1	1
FAT1	1	58.11202	1	1	1	65.0391	1	106.2197	1	1	1	48.87953	56.11762	1	1	46.18861	1	1	1	1
FAT3	1	49.24184	1	1	96.41082	55.04286	1	1	389.2381	51.41578	1	58.226	37.36361	1	279.6116	1	291.0437	1	1	208.0679
FAT4	1	1	1	1	1	25.97234	1	1	107.5212	1	1	1	1	1	56.90923	1	67.39414	1	1	1
FAXC	1	1	1	57.47685	71.53482	1	1	1	65.33299	30.3554	91.10597	1	26.23269	1	78.51141	1	1	1	75.41008	103.6483
FAXDC2	1	1	1	1	1	35.27455	1	1	1	1	1	1	1	1	1	1	1	1	1	1
FBLN1	1	1	1	175.7165	114.1893	63.91056	1	132.4156	95.81242	268.329	69.13109	244.015	42.37998	66.59524	90.36709	78.70475	1	1	220.5255	206.2967
FBLN7	1	1	1	1	1	1	1	1	1	1	1	30.34731	1	1	1	1	1	1	1	1
FBN1	1	1	1	1	1	1	1	84.39586	1	1	1	1	1	1	1	1	1	1	1	1
FBN2	1	1	1	1	1	34.05264	1	1	1	1	1	1	1	1	1	1	1	1	1	1
FBRSL1	1	33.93372	42.53562	64.40974	54.807	27.23513	1	1	80.54127	46.32602	1	26.0621	1	59.31202	68.0535	1	1	19.98915	95.07618	1
FBXL15	1	1	39.77175	1	37.71112	1	1	1	1	1	1	1	1	1	54.29932	1	115.0709	33.74157	1	1
FBXL16	1	1	1	1	1	1	1	1	90.34515	1	1	1	1	1	40.78322	1	1	1	1	70.02264
FBXL17	1	32.61344	1	1	1	32.96943	1	1	135.3957	1	1	1	32.05594	1	1	1	1	22.96838	1	1
FBXL2	1	1	1	1	1	1	1	1	1	1	1	1	1	1	65.05002	1	1	1	1	1
FBXL7	1	1	1	1	1	59.99381	1	1	1	1	1	23.718	50.32921	1	1	1	1	1	1	1
FBXO16	1	1	1	1	1	1	1	1	1	1	1	1	1	1	1	1	1	1	1	206.2967
FBXO17	1	39.74856	1	1	1	37.72343	1	41.96397	1	1	1	34.73241	28.3156	1	1	42.24876	1	1	1	1
FBXO28	1	37.15426	1	47.74835	48.52695	33.71145	1	1	1	41.83256	1	28.75465	37.03669	1	1	1	1	34.26032	1	1
FBXO32	1	1	1	1	1	87.86856	1	1	1	1	1	44.08005	229.2836	1	1	74.12419	1	1	1	1
FBXO33	1	49.88048	1	45.77695	64.29856	28.77446	1	43.02749	1	27.99643	77.97671	37.14941	36.6867	71.40854	1	34.38242	1	51.93862	1	1
FBXO34	1	1	1	1	1	32.63536	1	1	1	1	1	50.26	30.10835	1	1	58.38652	111.1878	36.42291	1	1
FBXO42	1	1	1	60.32283	53.99913	34.00293	1	1	1	50.82015	1	43.88961	39.92614	64.78851	69.25925	1	1	1	53.53966	1
FBXO45	1	36.80428	1	1	52.5069	33.65019	1	1	1	44.05458	1	42.2148	26.9794	1	1	1	1	1	63.29371	1
FBXO5	1	48.06593	1	1	1	31.49187	1	1	1	73.77469	1	251.3018	53.41181	1	1	1	1	1	1	1
FBXO8	1	1	61.33577	1	1	46.99617	1	1	1	1	1	1	26.88819	1	1	1	1	18.16759	1	1
FBXW9	1	1	1	1	1	1	1	1	1	1	1	1	36.48973	1	1	1	1	1	1	1
FCER1G	619.9877	1	1	1	1	1	1	1	1	1	1	1	1	1	1	1	1	1	1	1
FCGR1A	372.8424	1	1	1	1	1	1	1	1	1	1	1	1	1	1	1	1	1	1	1
FCGR1B	197.0919	1	1	1	1	1	1	1	1	1	1	1	1	1	1	1	1	1	1	1
FCGR2A	249.2486	1	1	1	1	1	1	1	1	1	1	1	1	1	1	1	1	1	1	1
FCGR3A	256.9423	1	1	1	1	1	1	1	1	1	1	1	1	1	1	1	1	1	1	1
FCGRT	598.9459	1	1	1	1	1	1	283.3633	1	1	1	1	1	1	1	1	1	338.1908	1	1
FCHSD2	1	54.98012	117.3881	1	1	38.82191	1	46.64435	92.44532	25.07398	1	39.97148	38.4971	64.58679	1	1	1	49.66742	1	1
FEM1A	1	39.17887	42.10046	49.90597	58.0235	23.35461	1	26.08949	1	35.58393	79.01683	37.48455	27.30763	67.62004	50.62369	1	1	21.85518	1	1
FEM1C	1	46.20488	32.14342	70.42277	51.17566	42.14722	1	39.27514	1	74.81789	1	48.4511	47.16454	1	1	1	1	75.533	1	70.02264
FEN1	1	1	52.20877	1	1	1	1	37.48585	1	105.1284	1	103.6335	34.47125	1	1	39.34484	1	1	1	1
FERMT1	1	1	85.26936	1	1	1	1	1	1	1	1	1	1	1	1	1	1	1	1	1
FERMT3	65.11194	1	1	1	1	1	1	1	1	1	1	1	1	1	1	1	1	1	1	1
FEZF2	1	1	1	1	1	124.2146	1	1	199.4226	37.15576	1	39.63051	76.5236	1	1	48.8002	1	1	1	1
FGD2	54.9966	1	1	1	1	1	1	1	1	1	1	1	1	1	1	1	1	1	1	1
FGD3	1	1	1	1	1	1	1	1	1	1	70.28867	1	1	90.76249	1	1	1	1	1	1
FGD4	1	44.46251	1	88.19186	86.16617	39.60211	1	36.5857	115.5348	121.2781	60.5069	60.72791	28.45693	68.0726	73.33816	1	268.5986	33.70439	94.50982	206.2967
FGD6	1	1	1	1	1	1	1	1	1	1	1	1	1	1	1	1	1	93.60618	1	1
FGF12	1	1	226.5866	1	1	1	1	1	247.621	1	202.5049	1	1	1	63.57426	1	260.5545	1	1	70.02264
FGF13	1	1	1	1	58.79511	1	1	1	99.17121	1	67.6487	35.51159	1	1	74.59514	1	69.16341	1	1	1
FGF14	1	1	53.37327	1	1	1	1	1	1	1	1	1	1	1	1	1	1	1	1	70.02264
FGF2	1	1	1	1	1	41.85477	1	1	1	1	1	1	73.9157	1	1	1	1	1	1	70.02264
FGF9	1	1	1	1	1	1	1	1	1	1	77.3307	1	1	98.18915	1	1	1	1	1	208.0679
FGFBP3	1	89.32909	108.3564	1	1	256.8584	1	1	1	46.99012	1	56.01988	173.1814	1	56.36841	189.1718	1	1	1	87.58009
FGFR2	1	47.2205	1	130.8617	156.8861	110.395	1	1	1	75.22195	1	72.29664	59.44049	1	88.26862	98.69976	1	1	124.0713	232.0002
FGFR3	1	63.91038	1	1	1	87.01989	1	1	1	1	1	1	69.17026	1	1	39.87437	1	1	1	1
FGFRL1	1	42.25082	1	1	1	44.85363	1	1	1	1	1	1	37.21135	1	1	1	1	1	1	1
FGGY	1	1	1	1	1	68.29759	1	1	1	1	1	1	1	1	1	1	1	1	1	1
FHAD1	1	1	1	1	1	1	1	1	1	1	1	1	31.38812	1	1	1	1	1	1	1
FHDC1	1	1	1	66.74749	1	1	1	1	1	1	1	1	1	1	1	1	1	1	48.94179	1
FHIT	1	1	46.24113	1	1	1	1	1	1	1	1	1	1	1	1	1	1	1	1	1
FHL3	1	1	1	1	1	1	1	1	1	1	1	1	1	1	1	1	1	106.4574	1	1
FHOD3	1	1	1	48.33424	49.97696	111.8206	1	1	1	55.19193	1	55.75814	57.49974	1	55.6049	61.60177	1	1	1	103.6483
FIBIN	1	81.8582	134.4298	1	1	1	1	1	1	1	1	1	1	1	1	1	1	1	1	1
FIGN	1	1	63.2017	1	1	29.80607	1	1	1	48.89985	1	69.34354	1	120.9873	1	1	1	1	1	1
FIGNL1	1	1	1	1	1	1	1	1	1	37.7255	1	42.96934	1	1	1	1	1	1	1	1
FILIP1L	1	1	1	1	1	1	1	179.2258	1	1	1	1	1	1	1	1	1	12.27685	1	1
FIZ1	1	1	1	1	1	1	1	1	1	31.15979	1	23.92975	1	1	1	1	1	1	1	1
FKBP15	68.48297	1	1	1	1	1	1	24.38939	1	1	1	1	1	1	1	1	1	1	1	1
FKBP1B	1	1	1	1	63.38879	32.83784	1	1	153.6041	1	1	1	1	1	127.3717	1	138.6195	1	101.043	172.671
FKBP5	1	1	1	1	1	1	1	80.00072	1	1	1	1	1	1	1	1	1	23.78511	1	1

FKBP7	1	1	1	1	1	1	1	53.89373	1	1	1	1	1	1	1	1	1	66.19918	70.46364	1
FKBP9	1	1	1	1	1	54.66907	1	47.20186	1	1	1	1	40.01278	1	1	1	1	85.74696	1	1
FLAD1	1	1	1	1	45.03235	28.87838	1	1	1	43.99706	1	24.90911	1	1	1	1	1	25.97871	1	70.02264
FLI1	1	1	1	1	1	1	1	1	1	1	1	1	1	1	1	1	1	118.0254	1	1
FLNA	1	61.85167	1	1	1	65.51513	1	218.3687	1	60.29139	1	54.22488	86.48663	1	1	83.27058	1	68.91412	1	1
FLNB	1	1	1	1	1	1	1	1	1	36.21733	1	1	32.07337	1	1	1	1	95.96551	1	1
FLOT2	1	46.49594	1	1	1	44.36134	1	1	1	1	1	33.03597	31.0909	1	1	1	1	41.77174	1	1
FLRT1	1	1	35.90579	1	1	1	1	1	1	1	1	1	1	1	1	1	1	1	1	1
FLRT2	1	1	1	1	51.75509	1	1	1	181.5813	1	1	1	1	1	84.26745	1	68.26239	1	1	139.0453
FLRT3	1	56.07327	97.29736	1	1	1	1	1	1	1	1	37.27952	35.94529	1	1	1	1	1	1	1
FLT1	1	1	1	1	1	1	1	48.32165	1	1	1	1	1	1	1	1	1	644.5087	1	1
FLT4	1	1	1	1	1	1	1	1	1	1	1	1	1	1	1	1	1	45.88872	1	1
FLVCR1	1	60.38635	76.95055	57.19606	60.67915	49.85683	1	1	1	71.6294	1	48.7049	59.18955	1	1	70.60804	1	1	1	1
FLYWCH2	1	45.47018	42.3093	1	1	71.66658	1	53.16072	1	44.43904	1	37.96308	43.63968	1	1	123.8978	1	26.16317	1	1
FMNL2	59.80766	99.86009	62.78871	225.5648	213.5354	56.31259	197.1473	1	150.9793	97.84232	1	85.33297	73.92133	1	132.2539	65.70556	1	68.15365	239.8465	1
FN1	1	1	1	1	1	1	1	5585.038	1	1	1	1	1	1	1	1	1	1349.819	1	1
FNDC1	1	1	1	1	1	1	1	1	1	1	1	1	77.58211	1	1	1	1	1	1	1
FNDC4	1	48.54965	42.68703	1	54.65988	59.76687	1	1	88.08908	1	1	30.84736	50.2503	1	1	50.93246	1	106.3278	67.64248	70.02264
FNDC5	1	1	1	1	1	1	1	1	1	1	1	1	1	84.82671	1	1	1	1	1	139.0453
FOLH1	1	1	1	1	1	33.97754	1	1	1	1	1	1	1	1	1	1	1	1	1	1
FOLR2	523.9236	1	1	1	1	1	1	1	1	1	1	1	1	1	1	1	1	1	1	1
FOSL2	1	1	1	1	1	1	1	69.01236	1	1	1	1	49.85272	1	1	1	1	87.03217	1	1
FOXC1	1	1	1	1	1	1	1	482.9476	1	1	1	1	1	1	1	1	1	393.4166	1	1
FOXC2	1	1	1	1	1	1	1	113.1487	1	1	1	1	1	1	1	1	1	1	1	1
FOXD1	1	1	1	1	1	1	1	473.0266	1	1	1	1	1	1	1	1	1	1	1	1
FOXF1	1	1	1	1	1	1	1	1	1	1	1	1	1	1	1	1	1	95.99655	1	1
FOXF2	1	1	1	1	1	1	1	410.5967	1	1	1	1	1	1	1	1	1	120.4819	1	1
FOXJ1	1	1	1	1	1	1	1	1	1	1	1	1	137.78	1	1	1	1	1	1	1
FOXK2	1	32.59227	51.27242	1	1	39.96084	1	31.95675	1	42.58802	1	47.97519	44.92677	1	85.51235	47.94874	1	39.3249	1	103.6483
FOXL2	1	1	1	1	1	1	1	1	1	1	1	1	1	1	1	1	1	151.1414	1	1
FOXM1	1	1	1	1	1	1	1	1	1	1	1	119.5376	1	1	1	1	1	1	1	1
FOXN2	1	71.57901	87.37226	85.22154	70.64982	45.16644	1	1	1	115.4955	1	74.6973	36.3289	1	1	65.87053	1	16.25274	1	1
FOXN4	1	1	1	1	1	1	1	1	1	1	1	30.69774	1	1	1	1	1	1	1	1
FOXO1	1	42.6576	1	1	1	1	1	26.82968	1	1	1	1	30.24395	1	1	1	1	70.43835	1	1
FOXP1	1	132.7019	1	1	1	64.14867	1	263.46	1	60.0592	1	58.03611	64.95023	1	1	58.65631	108.1851	281.0012	1	1
FOXP2	1	1	1	1	1	1	1	1	95.26784	50.20496	1	32.06732	1	1	1	1	1	1	1	1
FOXP4	1	1	1	67.87772	1	1	1	1	1	64.7947	1	33.35598	1	1	1	1	1	1	24.80211	1
FOXQ1	1	1	1	1	1	1	1	149.5407	1	1	1	1	1	1	1	1	1	198.026	1	1
FOXRED1	1	1	1	1	1	1	1	21.64547	1	51.75097	1	33.73969	1	1	1	1	1	1	67.1029	1
FOXRED2	1	1	1	1	1	1	1	1	1	33.18891	1	30.90139	1	1	1	1	1	1	1	1
FOXS1	1	1	1	1	1	1	1	302.9289	1	1	1	1	1	1	1	1	1	1	1	1
FPR1	111.359	1	1	1	1	1	1	1	1	1	1	1	1	1	1	1	1	1	1	1
FRAT1	1	1	1	1	1	1	1	1	1	1	1	1	1	63.16533	1	1	1	1	1	1
FRAT2	1	1	1	1	1	1	1	1	1	1	1	1	1	118.188	1	1	1	30.10375	1	70.02264
FRMD3	1	1	1	1	1	1	1	1	70.15756	1	1	1	1	1	1	1	61.79633	1	1	1
FRMD4B	85.74637	1	107.9187	271.135	356.505	33.72117	350.7774	137.2085	1	1	76.03617	73.4988	31.87791	1	391.7591	1	655.4421	137.16	248.2941	206.2967
FRMD6	1	1	1	1	1	1	1	170.5155	1	1	1	1	1	1	1	1	1	26.46484	1	1
FRRS1L	1	1	87.96494	1	1	1	1	1	604.1084	1	1	1	1	1	93.96866	1	121.256	1	1	149.721
FRY	1	1	1	1	1	1	1	30.60588	131.3008	1	1	1	1	1	1	1	1	37.09345	1	1
FRZB	1	1	1	1	1	1	1	901.8843	1	1	1	1	1	1	1	1	1	1	1	1
FSD1	1	58.63308	61.06697	54.8623	76.76439	40.77948	1	1	53.36276	60.1055	1	41.40888	28.80803	1	101.1859	1	165.2513	1	106.8198	1
FSD1L	1	1	26.78226	86.43103	106.6636	60.32026	1	1	174.0326	54.74315	80.71653	59.25109	79.7524	59.65869	96.32496	70.64132	208.8312	1	107.7134	275.3193
FST	1	1	1	1	1	1	1	1	1	1	1	36.39342	1	1	1	1	1	1	1	1
FSTL1	1	36.67071	1	1	1	223.3463	1	696.5923	1	29.37791	1	88.6645	237.1596	1	1	144.1465	1	200.5331	1	1
FSTL5	1	1	1	1	1	73.76256	1	1	124.528	1	1	30.15532	61.79106	1	1	1	1	1	1	1
FTO	1	1	40.39429	1	45.62075	26.60941	1	64.72945	67.93814	1	1	29.09376	30.64306	89.78152	63.23	1	1	26.97699	1	1
FUBP3	1	75.21576	50.38811	1	1	75.17231	1	40.61779	1	47.1881	1	41.82272	62.30462	1	45.05067	108.1363	1	41.3489	1	1
FUOM	152.3792	1	1	1	1	24.83389	1	29.56421	1	1	1	1	1	1	1	1	1	1	1	1
FUT9	1	115.898	1	147.5736	117.2861	254.4002	1	1	394.2688	108.7836	1	139.3972	93.40184	66.29013	1	145.6991	1	1	145.4935	70.02264
FUZ	1	1	1	1	1	26.38144	1	1	1	1	1	61.07402	31.0542	1	1	1	1	1	1	1
FXR2	1	1	40.46056	52.22598	51.07724	1	1	1	1	37.51028	1	35.3648	23.33738	1	59.57134	1	1	1	1	1
FXYD5	1	1	1	1	1	1	1	1	1	1	1	1	1	1	1	1	1	78.34728	1	1
FYB1	276.2999	1	1	1	1	1	1	1	1	1	1	1	1	1	1	1	1	1	1	1
FZD10	1	1	1	1	1	1	1	67.04792	1	1	1	1	1	1	1	1	1	1	1	1
FZD2	1	1	1	1	1	173.0624	1	1	1	52.58835	1	68.28109	96.53899	1	1	94.24374	1	1	1	1
FZD6	1	1	1	1	1	1	1	1	1	1	1	1	1	1	1	1	1	53.33226	1	1
FZD8	1	1	1	1	1	194.096	1	1	1	36.09438	1	61.40372	91.74526	1	1	81.26616	1	1	1	1
FZR1	1	1	1	1	1	1	1	1	1	1	1	54.40931	30.4893	1	1	1	139.2602	105.3814	1	1
G0S2	1	43.79388	1	1	1	1	1	1	1	1	1	1	1	1	1	1	1	1	1	1
G2E3	1	54.81689	78.92338	81.95282	89.96468	43.08072	1	1	84.95643	55.49805	104.9263	168.6735	44.13916	94.9458	104.3755	1	1	1	76.47012	87.58009
GAA	80.73365	1	1	1	1	1	1	1	1	1	1	1	1	1	1	1	1	21.55242	1	1
GAB1	1	114.443	89.44918	1	1	129.0359	1	94.70925	1	57.69317	1	79.01683	189.7389	1	1	86.30137	1	69.56798	1	1
GABBR2	1	1	1	1	1	1	1	1	311.924	1	1	1	1	1	1	1	152.0692	1	1	1
GABPB1	1	1	1	1	1	1	1	1	1	1	1	64.57702	29.70604	1	1	1	1	101.0878	1	1
GABRA1	1	1	1	1	1	1	1	1	104.0777	1	1	1	1	1	1	1	1	1	1	1
GABRA2	1	1	1	1	1	91.56167	1	1	1	1	1	1	54.66366	1	1	1	1	1	1	1
GABRA3	1	1	1	1	1	1	1	1	144.3026	1	1	1	1	1	1	1	1	1	1	1
GABRB1	1	1	1	1	1	111.7148	1	1	1	1	1	1	1	1	1	1	1	1	1	1
GABRB2	1	1	1	1	49.01235	1	1	1	330.1097	1	1	1	1	1	88.64079	1	59.67434	1	1	1
GABRB3	1	1	56.03769	82.14038	106.9261	1	1	1	188.564	1	79.08873	1	1	121.4886	165.534	1	250.7769	1	118.7413	1
GABRG1	1	1	1	1	1	1	1	1	231.6911	1	1	1	41.14	1	1	1	1	1	1	1
GABRG2	1	1	1	1	1	1	1	1	47.6419	1	1	1	1	1	1	1	1	1	1	1
GABRG3	1	1	1	1	1	1	1	1	346.8151	1	1	1	1	1	1	1	1	1	1	1
GAD1	1	1	1	1	1	1	1	1	1	1	151.1703	1	1	401.0219	1	1	1	1	1	1
GAD2	1	1	1	1	1	1	1	1	1	1	315.4482	1	1	306.8591	1	1	1	1	1	1
GADD45A	1	158.4525	56.68492	1	1	340.289	1	415.4221	1	98.40926	1	109.2414	181.505	97.0409	1	179.0736	1	686.2333	1	1
GADD45B	1287.688	139.0741	1	1	1	205.0091	1	530.5499	1	1	1	1	632.3607	1	1	168.5477	1	581.0745	1	1
GAL3ST1	1	1	124.2081	1	1	1	1	1	1	1	1	1	1	1	1	1	1	1	1	1
GAL3ST4	99.82327	1	1	1	1	1	1	1	1	1	1	1	1	1	1	1	1	1	1	1
GALK1	1	28.71516	1	1	1	27.19589	1	37.68825	1	1	1	1	1	1	1	1	1	1	1	1
GALK2	1	1	49.10927	1	1	26.77134	1	1	1	1	1	1	29.57905	1	1	1	1	43.05498	1	1
GALNT10	1	1	54.48149	1	1	1	1	1	51.60031	1	1	1	1	1	1	1	203.3943	54.82154	1	1
GALNT13	1	1	138.7806	1	1	1	1	1	106.0074	1	1	1	1	1	1	1	1	1	1	1
GALNT16	1	1	1	1	1	88.85094	1	1	1	1	1	22.47553	44.74737	1	1	1	1	1	1	1
GALNT18	1	1	1	1	1	1	1	97.65484	1	1	1	1	1	1	1	1	1	85.97233	1	1
GAMT	62.9416	80.68759	60.11765	1	60.24838	67.84651	1	33.94993	102.111	41.92301	1	44.5035	70.21738	1	61.6552	75.16676	1	1	98.16621	172.671
GAN	1	75.92854	1	46.73053	44.67897	44.4031	1	1	1	1	80.40644	33.90873	56.01747	78.69874	1	1	1	22.58845	45.33333	1
GAP43	1	68.27054	160.5531	283.2191	450.7477	1	607.594	1	660.6517	93.12725	190.8524	68.39705	42.77474	281.4358	641.0589	1	827.728	1	293.9122	784.1813
GAREM1	1	1	1	1	1	31.84415	1	1	118.3762	1	1	1	1	1	1	1	1	1	1	103.6483
GAREM2	1	37.4602	1	1	1	59.31269	1	1	1	46.21703	1	33.70436	36.78223	1	1	1	1	1	1	1
GARNL3	1	1	1	1	1	32.64769	1	1	77.61078	1	1	1	1	1	1	1	1	1	1	1

GAS1	1	735.3119	63.02277	1	1	101.7886	1	1	1	138.1295	1	179.7619	74.73008	1	1	62.21262	1	1	1	1
GAS2L3	1	1	1	1	1	1	1	38.53442	1	1	202.5346	159.7115	1	138.113	1	1	1	28.73863	1	1
GAS6	185.8017	1	1	1	1	26.30958	1	1	1	1	1	1	1	1	1	1	57.01662	74.00089	1	70.02264
GAS7	1	1	1	1	1	1	1	1	466.3046	1	1	1	1	1	1	1	127.4167	1	1	1
GATA2	1	1	1	1	1	1	1	1	1	1	1	1	1	1	1	1	1	94.18863	1	1
GATC	1	44.47782	1	45.86617	48.72101	37.41502	1	1	1	39.27701	1	38.22647	26.05645	1	1	1	1	36.56017	1	70.02264
GATD1	1	1	1	1	1	38.14617	1	1	1	31.68591	1	28.27644	32.35167	1	53.19178	1	1	1	74.39618	1
GATM	224.4434	306.7088	139.0223	1	1	488.2944	1	1	1	51.55069	1	99.93704	239.2153	1	1	257.172	1	1	1	70.02264
GBF1	1	1	39.08177	1	1	1	1	37.0844	1	1	1	24.77685	1	1	1	1	1	23.10059	53.28627	1
GBP4	1	1	1	1	1	1	1	1	1	1	1	1	1	1	1	1	1	69.34652	1	1
GCA	60.38179	53.49082	1	1	1	138.8458	1	1	1	78.07193	1	124.9627	70.16728	1	1	100.2291	1	20.16895	1	1
GCFC2	1	52.82948	1	1	1	50.27012	1	1	1	55.08323	1	39.39228	39.47426	1	1	1	1	18.42183	1	1
GCH1	1	1	1	1	1	1	1	1	1	1	1	1	1	1	1	1	1	88.51759	1	1
GDA	1	1	1	1	1	1	1	1	82.00099	1	1	1	1	1	1	1	1	1	1	1
GDAP1L1	1	1	99.0228	198.476	187.8994	1	259.8908	1	96.5788	56.66333	217.1935	37.89943	1	229.3368	259.4506	1	155.661	1	303.4222	206.2967
GDAP2	1	1	35.57855	1	1	24.68225	1	1	1	1	1	1	1	1	1	1	1	1	1	1
GDPD1	1	1	34.30683	1	1	1	1	1	157.682	1	1	1	1	1	1	1	1	1	83.65888	1
GEM	1	1	1	1	1	71.06647	1	332.2027	1	1	1	38.86561	96.33954	1	1	1	1	1	1	1
GEN1	1	1	1	1	1	1	1	1	1	32.10721	1	45.02881	1	1	1	1	1	1	1	1
GFAP	1	432.479	1	1	1	651.6547	1	1	1	1	1	101.6812	1281.237	1	1	849.0386	1	1	1	1
GFOD1	1	1	1	63.83692	63.21499	1	1	1	1	1	1	1	1	1	48.21908	1	1	44.72487	1	1
GGA2	90.08399	1	1	1	1	1	1	33.82411	1	1	1	25.57733	26.28025	1	1	1	1	35.22299	1	1
GGT5	1	1	1	1	1	1	1	254.5129	1	1	1	1	1	1	1	1	1	68.78916	1	1
GGT7	1	1	37.20045	1	1	1	1	1	1	1	1	1	1	1	1	1	1	1	1	1
GHR	1	1	1	1	1	1	1	51.21131	1	1	1	1	1	1	1	1	1	1	1	1
GIMAP1	1	1	1	1	1	1	1	1	1	1	1	1	1	1	1	1	1	154.4096	1	1
GIMAP4	128.3527	1	1	1	1	1	1	1	1	1	1	1	1	1	1	1	1	145.4518	1	1
GIMAP6	1	1	1	1	1	1	1	1	1	1	1	1	1	1	1	1	1	92.25001	1	1
GIMAP7	1	1	1	1	1	1	1	1	1	1	1	1	1	1	1	1	1	170.9617	1	1
GINS1	1	1	1	1	1	1	1	1	1	29.47304	1	38.00586	1	1	1	1	1	1	1	1
GINS2	1	78.03636	60.30523	1	1	45.78643	1	1	1	219.1679	1	116.7909	43.99212	1	1	108.2724	1	1	1	1
GINS3	1	1	1	1	1	1	1	1	1	1	1	26.15131	1	1	1	1	1	1	1	1
GINS4	1	1	1	1	1	1	1	1	1	48.44391	1	58.56944	1	1	1	1	1	1	1	1
GIPC1	69.85516	53.33466	156.3523	1	1	63.79965	1	57.41241	1	1	1	29.0817	32.41705	1	1	1	1	64.28358	1	1
GIT1	1	44.345	49.93299	1	51.56385	25.58932	1	1	1	44.31712	1	28.00915	31.78944	1	60.80619	1	1	41.96822	1	1
GIT2	1	1	1	1	1	1	1	26.39854	1	1	1	1	1	1	1	1	1	50.50721	70.1413	1
GJA1	1	1	1	1	1	1	1	1	1	1	1	1	1	1	1	1	1	237.5176	1	1
GJA4	1	1	1	1	1	1	1	64.12768	1	1	1	1	1	1	1	1	1	138.9158	1	1
GKAP1	1	66.80507	36.49761	1	1	52.78932	1	1	1	79.27256	1	87.37107	77.66644	1	1	36.80038	1	1	1	1
GLA	1	1	1	1	1	1	1	45.64122	1	1	1	24.55213	1	1	1	1	1	53.99053	97.11575	1
GLCE	1	1	1	111.6549	76.43026	1	1	1	1	58.89505	104.1967	1	1	64.90101	1	1	1	37.2904	114.1307	70.02264
GLDC	1	32.1479	1	1	1	1	1	1	1	1	1	1	1	1	1	1	1	1	1	1
GLDN	112.6289	1	1	1	1	1	1	1	1	1	1	1	1	1	1	1	1	1	1	1
GLI2	1	38.15577	1	1	1	1	1	1	1	51.02766	1	40.13164	1	1	1	1	1	1	1	1
GLI3	1	97.03161	1	76.62432	1	371.028	1	1	1	197.9418	1	193.0649	183.5948	1	1	180.7564	1	1	1	1
GLI4	1	1	1	1	1	26.58079	1	1	1	1	1	1	1	1	1	1	1	1	1	1
GLIPR1	126.6057	1	1	1	1	1	1	1	1	1	1	1	1	1	1	1	1	1	1	1
GLIS3	1	1	1	1	1	27.95437	1	1	1	1	1	1	71.56762	1	1	1	1	1	1	1
GLMN	1	1	1	1	1	1	1	1	1	31.92305	1	1	1	1	1	1	1	1	1	1
GLRA2	1	1	1	1	1	1	1	1	1	1	87.40801	1	1	1	69.01771	1	1	1	1	70.02264
GLRA3	1	1	70.18462	1	1	1	1	1	1	1	1	1	1	1	1	1	1	1	1	70.02264
GLRB	1	1	42.47366	1	1	1	1	1	1	1	1	1	1	1	1	1	1	1	1	1
GLRX	221.6349	1	1	61.12917	60.74764	1	1	67.96488	1	1	58.42665	1	1	1	101.6517	1	97.64506	156.2011	1	70.02264
GLT8D2	1	1	1	1	1	73.67293	1	32.3394	1	1	1	1	79.97587	1	1	1	1	1	1	1
GLTP	1	72.33521	127.6666	1	1	75.25397	1	61.48201	1	49.37444	94.35299	54.03197	55.25896	1	1	64.85795	1	85.0465	1	1
GLUD1	1	147.3981	97.31048	1	1	167.5947	1	139.121	1	49.3093	1	75.82076	100.6716	1	1	99.99196	1	124.453	1	1
GM2A	70.85634	1	1	1	1	1	1	1	1	1	1	1	1	1	1	1	1	1	1	1
GMDS	1	1	1	1	1	1	1	272.9493	1	1	1	1	1	1	1	1	1	67.71597	1	1
GMEB1	1	1	36.71524	63.62472	75.84139	1	1	35.41164	1	66.90363	103.7659	48.43277	24.24776	72.14556	1	1	94.84994	20.61594	1	206.2967
GMFG	163.0252	1	1	1	1	1	1	99.54953	1	1	1	1	1	1	1	1	1	186.5133	1	1
GMNN	1	39.13231	1	1	1	41.3514	1	1	1	123.4702	1	162.34	43.61955	1	1	94.66364	1	23.22999	1	1
GMPR2	1	36.73359	1	1	1	44.94627	1	23.94719	1	1	1	34.52223	46.14367	1	49.74038	1	1	40.75537	1	1
GNA13	52.46161	40.88557	1	46.78046	45.24888	57.4308	1	48.1388	1	1	58.809	43.91406	47.12282	1	1	1	1	27.51514	1	1
GNAI1	1	1	100.0595	116.2174	152.275	1	163.566	92.27058	83.02722	68.42209	83.69348	51.40957	1	93.32893	196.162	1	190.4543	57.33666	182.397	208.0679
GNAL	1	1	1	1	1	1	1	1	435.4908	1	1	1	1	1	1	1	1	1	1	1
GNAO1	1	65.21154	86.12468	129.4466	149.4917	167.6336	1	1	550.9825	40.88675	274.063	66.31113	53.162	191.8863	211.2173	110.0883	254.5858	1	130.4763	87.58009
GNAZ	1	1	1	1	1	1	1	1	105.9738	28.82534	1	30.46743	32.08658	1	1	1	34.17467	1	1	1
GNB5	1	1	35.06069	54.12766	75.022	34.37637	1	1	93.42436	32.31635	106.485	51.85164	45.99401	77.00944	93.12529	55.80239	1	1	82.49588	103.6483
GNG11	1	43.6305	1	1	1	49.27905	1	700.9887	1	1	1	41.02586	1	1	1	53.16469	1	879.0451	1	1
GNG12	1	121.8047	88.44698	1	1	151.6153	1	116.9227	1	1	1	53.17842	99.79066	1	1	95.61236	1	1	1	1
GNG2	1	34.06188	183.1689	158.565	152.4462	1	189.8314	1	230.0029	56.21419	260.8271	37.84901	1	239.3089	174.6539	1	69.39889	1	209.9614	275.3193
GNG3	1	1	96.51079	239.9312	259.9198	59.21197	307.7819	1	127.537	1	1	41.15269	59.16179	1	142.7685	102.4568	49.13445	23.22612	229.6476	298.442
GNG4	1	62.5086	125.82	135.9684	86.59809	1	1	35.56205	1	144.8477	88.32546	132.9827	47.83599	1	1	1	1	1	129.0976	1
GNG7	196.3682	56.62742	88.40475	1	1	1	1	1	1	1	1	1	1	1	1	1	1	1	1	1
GNL2	1	33.74396	67.19863	1	1	25.54249	1	52.81389	1	58.6321	1	40.47816	42.25722	1	1	1	1	18.06135	63.08625	1
GNPDA1	137.0635	56.16926	37.0792	1	1	86.84615	1	1	1	81.62929	1	60.49354	64.40758	1	1	86.22557	1	1	1	1
GNPDA2	1	45.39831	57.32085	58.84044	60.88602	51.32123	1	29.40634	1	73.58077	1	49.42874	40.10528	69.60005	1	44.29964	1	44.10637	131.7852	1
GNPNAT1	1	1	1	1	1	1	1	34.47055	1	1	1	26.5529	23.95617	1	1	1	1	28.09158	1	103.6483
GNPTG	53.26709	44.83346	39.0088	1	1	60.23093	1	22.61581	1	33.20686	1	29.11447	39.7599	1	1	81.31732	1	24.18954	1	1
GOLGA7B	1	1	1	1	1	1	1	1	42.75942	1	1	1	1	1	1	1	1	1	1	1
GOLGA8A	1	44.95042	75.33549	120.6945	80.83191	36.9628	1	1	193.9602	30.37168	1	39.28655	35.45654	1	50.35751	196.2184	1	1	109.6081	103.6483
GOLGA8B	1	47.94312	125.5979	156.1743	117.9506	56.1708	1	1	430.9279	38.29428	1	36.54992	39.79431	1	68.09734	148.0669	103.1347	1	114.4407	116.5001
GOLPH3L	1	1	1	1	1	1	1	1	1	1	1	1	26.29191	1	1	1	1	1	1	1
GON7	1	1	1	1	1	1	1	1	1	1	1	25.05141	40.74119	1	1	1	1	1	1	1
GORASP2	1	57.78104	38.62258	1	1	42.31263	1	41.98897	1	53.58067	1	35.55584	38.24746	61.90447	1	45.99538	1	29.41977	1	103.6483
GOT1	1	1	1	1	1	1	1	1	1	1	1	38.26976	1	1	1	1	1	1	1	103.6483
GOT2	1	1	55.96438	1	42.9068	34.47468	1	37.91133	1	33.54064	1	34.94788	39.89671	67.13294	1	1	1	41.70657	76.2962	103.6483
GPANK1	1	50.80967	1	1	1	44.04099	1	1	1	65.92273	1	63.73447	40.59433	1	1	1	1	1	54.85441	103.6483
GPATCH4	1	37.38471	81.32671	1	1	25.78971	1	1	128.2253	34.60616	1	1	34.08357	1	1	1	134.4752	1	1	1
GPC2	1	1	81.59561	203.5667	187.4577	1	132.2757	1	1	108.8636	169.435	74.48062	1	199.9107	132.7618	1	106.8614	1	261.5362	116.5001
GPC4	1	1	1	1	1	80.64776	1	1	1	1	1	36.87948	81.21738	1	1	1	1	1	1	1
GPC6	1	1	46.33509	48.59108	1	68.53514	1	1	1	93.5407	1	76.30356	41.26067	1	1	1	1	1	1	1
GPD1	1	1	1	1	1	1	1	1	1	1	138.8834	1	1	1	1	1	1	1	1	1
GPD2	1	1	1	1	1	26.41852	1	1	107.7085	31.26598	1	38.46166	1	1	1	1	1	1	1	70.02264
GPER1	1	1	1	1	1	1	1	221.9235	1	1	1	1	1	1	1	1	1	1	1	1
GPHN	1	1	35.8179	1	46.39844	24.32514	1	1	1	1	1	1	1	1	44.38141	1	1	1	1	1
GPN3	257.2734	44.0854	39.54214	1	1	24.02461	1	1	1	47.79403	1	44.14614	32.79813	1	1	1	1	30.59581	1	70.02264
GPNMB	320.9216	1	1	1	1	1	1	1	1	1	1	1	1	1	1	1	1	1	1	1
GPR12	1	1	1	1	48.65334	1	1	1	84.25041	1	1	1	1	1	1	1	57.36032	1	80.72312	70.02264
GPR137B	1	42.94934	1	1	1	104.2462	1	1	1	1	1	1	42.32827	1	1	1	1	1	1	1
GPR137C	1	1	29.349	76.08196	74.45889	1	1	1	1	38.64781	1	46.23158	1	67.63711	1	1	1	1	1	1

GPR146	1	1	1	1	1	1	1	1	1	1	1	1	1	1	1	1	1	54.78741	1	1
GPR155	1	1	213.7507	1	1	30.06271	1	1	70.30598	1	1	1	1	1	1	1	1	46.29471	1	1
GPR161	1	49.18099	32.31256	139.6125	143.8426	64.26508	147.1812	27.35632	118.2171	88.70238	159.0781	89.08175	50.90162	179.4557	148.3343	1	72.54682	1	124.7113	1
GPR162	1	1	1	1	1	1	1	1	71.92653	1	1	1	32.54149	1	1	1	1	1	1	1
GPR17	1	1	147.3399	1	1	1	1	1	1	1	1	1	1	1	1	1	1	1	1	1
GPR173	1	1	1	1	1	1	1	1	1	1	1	1	1	1	1	1	1	53.82796	1	1
GPR180	1	1	1	1	1	32.63375	1	1	1	40.65593	1	41.4595	29.51207	1	1	1	1	1	1	1
GPR183	375.3781	1	1	1	1	1	1	32.48341	1	1	1	1	1	1	1	1	1	1	1	1
GPR22	1	1	1	1	1	1	1	1	214.1562	1	1	1	1	1	1	1	263.944	1	1	103.6483
GPR27	1	1	1	1	1	26.38791	1	1	151.7585	1	1	1	1	1	1	1	132.7428	1	1	103.6483
GPR34	457.9892	1	1	1	1	1	1	1	1	1	1	1	1	1	1	1	1	1	1	1
GPR37	1	1	1	1	1	1	1	1	121.3282	1	1	1	1	1	1	1	1	1	1	1
GPR82	1	1	56.2732	1	1	1	1	1	1	1	1	1	1	1	1	1	1	1	1	1
GPR84	57.08859	1	1	1	1	1	1	1	1	1	1	1	1	1	1	1	1	1	1	1
GPR85	1	1	1	1	61.56166	1	1	1	64.03543	1	1	1	1	1	150.2261	1	65.39263	1	1	1
GPRASP1	1	1	1	1	1	1	1	1	1	1	1	1	1	1	1	1	250.3045	1	1	1
GPRASP2	1	1	1	1	54.55549	30.18273	1	1	1	1	1	28.38671	27.13714	1	1	1	1	1	1	70.02264
GPRC5B	1	131.9472	87.42791	57.13496	1	1	1	1	1	101.6147	1	57.21197	108.8019	1	1	59.43092	1	1	1	103.6483
GPRC5C	1	1	1	1	1	1	1	107.6953	1	1	1	1	1	1	1	1	1	1	1	1
GPRIN1	1	1	1	1	1	1	1	1	80.5904	1	1	1	1	1	1	1	112.9362	1	1	103.6483
GPRIN3	1	1	1	1	1	1	1	1	131.8229	1	1	1	1	1	1	1	1	1	1	1
GPS2	1	1	1	1	37.98527	29.0353	1	32.74678	1	1	69.25578	35.24761	23.77198	64.15649	41.15976	1	1	15.62882	1	103.6483
GPSM3	106.7253	1	1	1	1	1	1	49.06341	1	1	1	1	1	1	1	1	1	1	1	1
GPT2	1	61.07756	63.643	1	1	60.43845	1	1	1	49.62099	75.35503	55.31015	52.78718	1	1	1	140.3389	1	1	1
GPX3	1	1	1	1	1	481.9284	1	1	1	1	1	53.03115	85.52025	1	1	116.6244	1	1	1	1
GRAMD1A	1	47.42809	69.58825	71.90864	58.58005	27.97219	1	67.05053	1	43.43086	1	34.80839	46.67062	1	1	1	1	57.7449	34.9243	1
GRAMD1B	1	1	1	134.0506	67.06215	1	1	1	1	122.0364	1	72.75029	35.17535	1	1	1	1	1	53.10048	103.6483
GRAMD1C	1	1	1	1	1	40.75132	1	1	1	1	1	1	1	1	1	1	1	1	1	1
GRAMD2B	1	1	69.42653	1	1	1	1	57.13222	1	1	1	1	50.47055	1	1	1	1	1	1	1
GRAP	1	1	1	1	1	1	1	1	1	1	1	1	1	1	1	1	1	78.02145	1	1
GRASP	106.8523	1	1	1	1	1	1	1	1	1	1	1	1	1	1	1	1	111.538	1	1
GRB14	1	1	98.20414	1	1	51.67689	1	1	1	1	1	1	37.81667	1	1	1	1	1	1	70.02264
GRIA1	1	1	1	1	47.25185	1	1	1	168.0093	1	97.43762	1	24.30183	195.5614	104.2324	1	194.6739	1	54.24806	103.6483
GRIA3	1	79.46116	150.9255	163.1953	113.0278	31.25145	1	1	147.9796	41.34269	1	32.2769	1	1	86.93224	1	123.6505	1	83.50627	206.2967
GRIA4	1	69.69537	139.9764	1	1	1	1	1	1	1	1	1	1	169.3067	1	1	1	1	1	1
GRID2	1	114.3467	363.9101	1	1	91.78928	1	1	564.0529	76.00217	1	87.2758	30.80858	1	1	134.7479	150.9646	1	1	1
GRIK2	1	1	50.62546	1	1	1	1	1	69.14066	1	1	1	1	1	1	1	1	1	1	1
GRIK3	1	1	42.74149	1	1	1	1	1	384.3675	1	1	1	1	1	1	1	1	1	1	1
GRIK5	1	1	1	1	40.65595	31.62673	1	1	103.2445	1	1	1	1	1	62.48348	1	1	1	1	1
GRIN1	1	1	1	1	1	1	1	1	106.4096	1	1	1	1	1	1	1	1	1	1	70.02264
GRIN2A	1	1	1	1	1	62.29275	1	1	1	1	1	1	93.15623	1	1	1	1	1	1	1
GRIN2B	1	1	1	1	1	1	1	1	678.6514	1	94.12102	1	1	69.90354	1	1	364.0801	1	1	139.0453
GRIP1	1	1	1	1	1	1	1	1	1	1	1	1	1	102.3924	1	1	1	1	1	1
GRIP2	1	1	1	1	1	1	1	1	1	1	1	1	1	71.60593	1	1	1	1	1	1
GRK2	1	1	1	1	47.48567	1	1	39.00975	50.80723	29.49	1	31.86657	1	1	1	1	1	35.37808	61.78101	103.6483
GRK3	1	1	1	1	1	1	1	39.73198	209.7836	1	1	1	1	1	1	1	1	1	1	1
GRK4	1	1	1	1	41.3297	34.62695	1	1	97.37272	28.09562	1	24.57053	1	1	57.52495	1	1	1	1	1
GRM3	1	1	1	1	1	175.0861	1	133.1697	316.1528	1	1	42.64748	1	1	1	103.0542	97.19939	1	1	1
GRM5	1	1	63.2991	1	1	1	1	1	313.3481	1	1	1	1	1	1	1	1	1	1	1
GRN	294.2764	30.74286	1	1	1	41.45107	1	111.4066	1	1	1	1	1	1	1	1	1	123.9107	1	103.6483
GSDMD	1	1	1	1	1	1	1	1	1	1	1	1	1	1	1	1	1	41.77665	1	1
GSDME	1	1	1	1	1	26.81349	1	1	1	1	1	1	1	1	1	1	1	1	1	1
GSE1	1	1	1	153.9032	140.6801	1	1	1	119.9802	147.9748	240.0879	102.0297	44.57203	112.7932	142.562	50.29793	115.7813	35.9262	202.5397	103.6483
GSK3A	1	54.33091	71.06927	72.74347	84.21861	52.46484	1	64.6519	71.35673	55.98493	80.15335	51.53454	52.17269	90.39768	71.35659	1	130.6803	26.30554	73.35837	265.2211
GSN	396.34	1	1	1	1	30.53551	1	232.6862	1	35.72064	1	1	1	1	1	1	1	56.76583	1	1
GSPT2	1	35.89324	56.67393	55.0392	50.35496	49.39442	1	1	48.46658	29.31908	69.8926	39.36165	34.61297	1	1	1	1	1	1	70.02264
GSTK1	109.5852	50.39546	1	1	1	67.8716	1	80.57057	1	1	1	1	43.07861	1	1	57.02316	112.7576	40.98646	1	1
GSTM4	1	1	1	1	1	1	1	1	1	1	1	1	1	1	1	112.829	1	1	1	1
GSTM5	1	1	1	1	1	57.58089	1	1	1	1	1	1	1	1	1	72.64769	1	1	1	1
GTDC1	1	1	1	56.796	70.67747	1	1	57.2982	1	1	88.57907	33.37552	1	75.42819	99.8722	1	178.9373	1	1	1
GTF2H1	1	41.85558	44.62059	1	1	28.49608	1	35.89647	1	30.61824	1	29.60661	37.97972	1	1	41.86371	118.2607	26.99428	1	70.02264
GTF2H2	1	60.34378	107.287	65.4252	61.84055	50.7343	1	26.4695	169.2039	65.09069	77.28817	56.49875	38.8461	65.99064	61.8138	55.05666	94.09951	19.12946	1	103.6483
GTF2IRD2	1	1	1	60.5728	47.61301	31.4686	1	1	1	36.40215	1	24.48518	31.82925	1	55.2696	1	1	1	114.4428	116.5001
GTF3C1	1	49.26972	66.94699	48.82333	1	38.68092	1	29.89423	1	55.26891	65.2743	56.25439	43.19834	1	1	1	1	35.46601	1	70.02264
GTF3C2	1	1	1	1	1	1	1	1	1	34.18718	1	31.5765	27.7264	1	1	1	1	26.152	1	1
GTF3C3	1	31.2348	1	1	1	1	1	1	1	31.18071	1	1	23.15933	1	1	1	1	1	1	1
GTF3C4	1	1	1	1	1	30.14089	1	23.15136	1	48.81147	1	51.74941	47.2504	1	37.34904	69.98208	1	14.46619	1	1
GTF3C5	1	1	1	1	1	24.06416	1	1	1	29.71735	1	41.25448	1	1	1	1	1	1	1	1
GTPBP1	1	1	1	1	46.01787	24.63291	1	21.79293	1	38.01271	1	49.90887	30.32304	1	1	88.18949	1	21.364	1	1
GTPBP4	1	84.40271	90.2999	64.61303	63.60271	60.63366	1	65.75916	1	102.3455	1	71.93346	73.38256	70.00864	52.83682	107.5045	1	33.21149	56.36527	241.6936
GTPBP6	1	49.07685	44.24351	1	1	41.22681	1	29.59466	1	1	1	28.74699	37.01319	1	1	54.34443	1	34.89874	1	1
GTPBP8	1	42.51664	1	1	1	32.16629	1	1	1	41.60867	1	31.7798	30.07616	1	1	1	1	1	1	1
GTSE1	1	1	1	1	1	1	1	1	1	1	1	322.816	1	1	1	1	1	1	1	1
GUCY1A1	1	1	1	1	1	1	1	281.4681	1	1	1	1	1	1	1	1	1	1	1	1
GUCY1A2	1	1	47.93603	1	71.71505	1	1	547.0441	152.61	1	114.4774	1	1	1	179.9108	1	212.5516	1	1	70.02264
GUCY1B1	1	1	1	1	1	1	1	232.7318	1	1	1	1	1	1	1	1	1	1	1	1
GUF1	1	46.02525	30.96851	1	1	57.07271	1	1	1	41.51951	1	39.39576	38.03175	1	1	1	1	18.36366	57.2609	1
GULP1	1	119.598	35.55727	60.03773	1	239.5501	1	1	1	94.57054	1	103.6367	107.9934	1	1	99.95249	1	44.97542	1	1
GUSB	77.20541	1	1	1	1	28.20017	1	23.05829	1	40.11701	1	48.20658	26.32705	1	1	71.70959	1	38.19546	1	1
GYPC	721.6116	1	1	1	1	63.50467	1	116.3739	1	1	1	39.48634	56.98114	1	1	77.75363	1	1	1	1
GZF1	1	1	1	1	1	27.64108	1	1	1	32.51916	1	1	26.33799	1	1	1	1	1	1	1
H2AFJ	111.8082	1	1	1	1	1	1	118.5774	1	1	1	1	1	1	1	1	1	92.33142	1	1
H2AFX	1	101.7771	55.20234	1	1	89.93104	1	153.2283	1	212.5583	1	651.8144	161.8253	1	1	239.7	1	50.42632	1	70.02264
H2AFY2	1	53.85396	100.0213	1	1	42.13532	1	1	1	81.08067	119.4148	63.03355	38.929	107.9646	1	80.69435	1	1	1	1
HABP4	1	37.99241	1	1	1	64.82859	1	1	1	1	1	33.50834	40.29308	1	1	81.17481	120.2151	1	1	1
HACD2	1	1	1	1	48.2663	25.29902	1	1	179.8112	35.36663	1	33.82641	1	1	1	1	1	35.33212	1	1
HACL1	1	1	1	1	1	1	1	1	1	1	1	25.66124	27.68028	1	1	1	68.16057	30.41038	1	103.6483
HAGHL	1	1	1	1	1	1	1	1	117.2172	1	1	1	1	1	1	1	1	1	1	1
HAPLN1	1	1	1	1	1	53.55959	1	1	1	55.05049	1	1	1	1	1	1	1	1	1	1
HAPLN3	1	1	1	1	1	1	1	1	1	1	1	1	1	1	1	1	1	156.5607	1	1
HARS	1	37.72329	44.23249	59.37258	61.4839	35.95903	1	38.96498	104.7999	58.72225	1	45.53171	30.90098	1	69.1881	113.4408	1	36.49119	104.2029	103.6483
HAS2	1	1	45.11496	1	1	1	1	1	1	1	1	1	1	1	1	1	1	1	1	1
HAS3	1	1	1	1	1	1	1	1	116.4544	1	1	1	1	1	1	1	1	1	1	1
HAT1	1	80.16061	41.56649	1	1	89.99517	1	48.64943	1	122.81	1	122.5245	90.92138	1	1	131.9183	1	48.39507	1	1
HAUS2	1	51.49714	1	1	1	40.81416	1	1	1	1	1	48.07652	37.14317	1	1	1	1	1	1	1
HAUS3	1	1	37.5423	1	1	25.59599	1	1	1	41.24536	1	49.78029	1	1	1	1	1	1	1	1
HAUS5	1	1	1	1	1	1	1	1	1	1	1	35.24451	1	1	1	1	1	1	1	1
HAUS6	1	59.83243	48.07289	1	1	51.21462	1	1	1	103.2732	1	80.02994	58.72748	1	46.50622	95.58719	1	1	1	70.02264
HAUS8	1	1	1	1	1	1	1	1	1	1	1	48.43064	1	1	1	1	1	1	1	1
HAVCR2	176.0141	1	1	1	1	1	1	1	1	1	1	1	1	1	1	1	1	1	1	1
HBA1	1	1	1	1	1	1	1	1	1	1	1	1	1	1	1	1	1	70.27915	1	1

HBA2	1	1	1	1	1	1	1	1	1	1	1	1	1	1	1	1	1	304.9857	1	1
HBB	1	1	1	1	1	1	1	1	1	1	1	1	1	1	1	1	1	242.1177	1	1
HBEGF	1	1	1	1	1	43.37898	1	260.1827	1	1	1	1	46.47651	1	1	1	1	243.7376	1	70.02264
HBG2	1	1	1	1	1	1	1	1	1	1	1	1	1	1	1	1	1	224.6131	1	1
HCFC1R1	1	1	1	62.54283	81.33671	42.91004	1	98.50704	1	32.72131	79.84736	57.00959	41.16444	1	104.0834	52.59884	1	21.79343	57.59357	275.3193
HCFC2	1	28.61534	1	46.24585	43.4255	53.22813	1	33.05848	1	26.46727	1	35.95596	44.20076	65.60295	1	1	1	1	1	1
HCG11	1	1	1	1	1	40.8444	1	32.16938	1	1	1	1	1	1	1	1	1	105.9796	1	1
HCG23	1	49.7935	62.71327	51.70492	59.94626	52.09382	1	1	53.35868	48.43979	1	25.06905	26.59164	62.20601	53.38458	1	1	1	1	1
HCK	143.6052	1	1	1	1	1	1	1	1	1	1	1	1	1	1	1	1	1	1	1
HCLS1	91.45871	1	1	1	1	1	1	1	1	1	1	1	1	1	1	1	1	1	1	1
HCN3	1	1	1	1	1	1	1	1	1	1	1	1	1	1	1	1	81.34271	1	1	1
HCST	76.23602	1	1	1	1	1	1	121.1918	1	1	1	1	1	1	1	1	1	1	1	1
HDAC4	1	37.4973	1	1	1	1	1	1	1	1	1	1	39.16568	1	1	1	1	1	1	1
HDAC5	1	35.97049	39.15551	77.14736	94.36362	32.43054	1	81.13375	67.15572	40.38009	114.1251	34.5805	36.46045	113.9208	1	1	1	67.53441	1	308.945
HDAC7	1	1	1	1	1	1	1	28.57627	1	1	1	1	1	1	1	1	131.1356	76.20945	1	1
HDAC8	1	1	52.34652	1	1	1	1	1	1	24.84514	1	28.61799	1	1	1	1	33.72883	1	40.79837	1
HDAC9	1	1	1	64.82506	49.58786	55.16237	1	1	1	118.0023	1	39.59538	1	1	48.60792	1	123.0854	1	1	103.6483
HDDC2	93.45698	89.35897	116.1275	49.40979	56.2372	82.32097	1	88.80759	1	96.3198	1	58.30246	68.51897	1	73.39861	72.20147	152.2393	19.4363	1	139.0453
HDHD2	1	52.87816	67.00205	1	1	60.63011	1	29.00838	1	42.34882	1	35.80511	44.23134	1	1	97.79905	1	1	1	103.6483
HDX	1	1	1	1	41.64227	1	1	1	1	30.80896	1	1	1	1	1	1	117.7568	31.96523	1	1
HEATR1	1	29.83411	1	1	1	24.73706	1	1	1	1	1	1	24.78505	1	1	1	1	1	1	1
HEATR5A	1	1	1	1	1	73.52916	1	1	1	1	1	26.82411	60.91932	1	1	64.83112	1	1	1	1
HEATR6	1	30.06135	1	39.49762	1	35.8129	1	1	1	36.21022	1	25.97519	29.62814	1	1	1	1	1	1	70.02264
HEBP1	52.33521	1	1	1	40.88526	79.037	1	1	1	1	1	29.64029	45.56169	1	1	72.46277	1	129.4065	1	1
HEBP2	81.14149	57.81393	1	94.63087	121.05	77.71829	228.0345	75.92101	1	61.84469	56.22742	71.3157	66.47888	1	259.7612	92.17058	65.77305	63.45774	116.0474	1
HECA	1	35.84391	1	47.52029	55.30948	28.26904	1	51.35461	1	39.16488	1	34.56596	25.54878	1	1	1	1	21.48344	1	1
HECTD2	1	1	1	49.4826	47.33107	31.9848	1	1	98.98058	36.30571	1	34.40866	25.1677	1	49.47929	1	1	1	1	70.02264
HECW1	1	1	1	86.38244	51.08004	1	1	1	1	38.42374	1	1	1	61.62872	1	1	1	1	1	1
HECW2	1	1	1	1	1	1	1	1	67.71558	1	1	1	1	1	1	1	1	1	1	1
HEG1	1	47.5505	1	55.39546	1	104.8274	1	61.37936	1	164.8562	1	108.8433	123.9655	1	1	94.52335	1	171.0313	1	1
HELB	1	42.29819	1	1	1	39.27777	1	25.76663	1	1	1	1	52.40283	1	1	1	1	1	107.5691	1
HELLS	1	101.3845	52.42258	53.03755	1	66.36481	1	1	1	319.1799	1	247.1283	98.84	1	1	143.168	1	1	1	1
HEPACAM	1	107.7789	1	1	1	109.5484	1	1	1	1	1	1	53.63939	1	1	64.93935	1	1	1	1
HEPN1	1	214.3578	60.37126	1	1	221.8581	1	1	1	1	1	44.50035	96.45424	1	1	93.66102	1	1	1	1
HERC3	1	1	37.38757	1	39.85232	1	1	1	90.85131	1	1	1	1	1	60.61719	1	73.95346	1	1	1
HERC4	1	61.67118	31.21524	75.98952	60.52877	57.80315	1	57.75967	1	40.80518	70.49483	31.25135	31.05596	1	86.20947	1	1	127.0289	1	103.6483
HERPUD2	1	56.04015	52.31319	40.814	42.18753	49.33505	1	66.7783	1	1	1	1	27.07514	94.2686	1	1	1	113.7149	1	1
HES1	129.5548	345.1672	1	1	1	884.6367	1	313.104	1	79.65053	1	190.9474	966.9257	1	1	349.0871	1	1436.732	1	1
HES5	1	649.182	72.61464	1	1	600.3046	1	1	1	276.6834	1	236.5503	276.8685	1	1	306.4763	1	1	1	1
HEXB	91.5377	1	1	1	1	1	1	29.39459	1	1	1	1	1	1	1	1	1	37.22223	1	1
HEXDC	1	1	35.21977	57.21166	61.41094	35.08586	1	1	1	41.27769	1	1	32.49694	73.21702	49.12398	1	1	1	1	139.0453
HEY1	1	121.4005	79.75175	1	1	112.341	1	29.88105	1	1	1	39.31933	50.99935	1	1	1	1	69.38115	1	70.02264
HEY2	1	1	1	1	1	1	1	143.4454	1	1	1	1	1	1	1	1	1	21.63677	1	1
HEYL	1	1	1	1	1	1	1	83.60199	1	1	1	1	1	1	1	1	1	1	1	1
HGF	1	1	1	1	1	64.49298	1	1	1	1	1	1	26.88829	1	1	1	1	1	1	1
HGS	1	31.42969	1	1	39.22785	31.48427	1	21.74887	1	1	1	31.84852	35.47907	1	1	1	175.1452	40.15305	1	1
HHEX	102.4375	1	1	1	1	1	1	1	1	1	1	1	1	1	1	1	1	133.6566	1	1
HIC1	1	1	1	1	1	1	1	199.7118	1	1	1	1	1	1	1	1	1	36.76947	1	1
HIC2	1	1	1	48.32016	50.54581	1	1	1	1	35.69892	1	27.50699	1	77.87665	1	1	1	1	1	1
HIGD1B	1	1	1	1	1	1	1	1033.969	1	1	1	1	1	1	1	1	1	1	1	1
HINFP	1	1	1	1	1	1	1	1	1	1	1	1	1	1	1	1	1	1	64.93015	1
HIP1R	1	36.47882	114.3318	63.80336	49.7475	1	1	1	189.585	1	73.71284	1	1	103.7561	87.71931	1	126.9176	65.70545	92.96704	103.6483
HIPK3	44.00032	1	34.24879	1	1	34.13717	1	62.07551	1	1	69.40388	28.74276	51.03166	1	1	1	1	56.24011	1	1
HIRIP3	1	38.63225	47.98167	1	1	25.72682	1	1	1	93.15156	1	119.1198	26.44022	1	1	54.45184	1	1	1	70.02264
HIST1H1A	1	1	1	1	1	1	1	1	1	45.76563	1	350.8218	1	1	1	1	1	1	1	70.02264
HIST1H1B	1	1	1	1	1	1	1	1	1	1	1	340.8768	1	1	1	1	1	1	1	1
HIST1H1C	172.1443	101.0204	67.69247	44.62297	1	181.512	1	39.30976	1	99.80181	90.0985	583.3781	180.0103	1	1	112.1546	1	89.03625	1	1
HIST1H1D	1	71.14707	37.06156	71.21957	67.92898	48.51297	1	1	1	165.8706	81.00154	886.5969	229.2707	1	1	132.0193	1	32.83431	63.99638	1
HIST1H1E	53.20112	65.94366	1	87.83113	57.33816	73.77888	1	1	1	103.7172	137.5503	298.5081	118.5697	83.22367	1	91.46911	1	29.35969	52.34311	70.02264
HIST1H2AC	85.10224	1	1	63.66787	66.65609	125.83	1	37.67172	1	1	1	54.12654	1	1	1	1	1	71.26596	1	1
HIST1H2AE	1	1	1	1	1	1	1	1	1	1	1	35.90764	1	1	1	1	1	1	1	1
HIST1H2AG	1	1	1	1	1	1	1	1	1	1	1	70.46752	1	1	1	1	1	1	1	1
HIST1H2AH	1	1	1	1	1	1	1	1	1	1	1	82.41851	1	1	1	1	1	1	1	1
HIST1H2AK	1	1	1	1	1	1	1	1	1	1	1	33.83487	1	1	1	1	1	1	1	70.02264
HIST1H2AL	1	1	1	1	1	1	1	1	1	1	1	91.38808	1	1	1	1	1	1	1	1
HIST1H2BC	1	1	1	1	1	1	1	1	1	1	1	26.66522	1	1	1	1	1	1	1	1
HIST1H2BD	1	1	1	1	1	29.77997	1	1	1	1	1	1	1	1	1	1	1	1	1	1
HIST1H2BF	1	1	1	1	1	1	1	1	1	1	1	38.29878	1	1	1	1	1	1	1	1
HIST1H2BG	1	1	1	1	1	46.04311	1	1	1	1	1	35.84806	1	1	1	1	1	1	1	70.02264
HIST1H2BH	1	1	1	1	1	1	1	1	1	1	1	51.82659	1	1	1	1	1	1	1	1
HIST1H2BN	1	1	1	1	1	30.06141	1	1	62.7417	1	1	39.25107	1	1	1	1	1	1	1	139.0453
HIST1H3B	1	1	1	1	1	1	1	1	1	1	1	68.49169	1	1	1	1	1	1	1	1
HIST1H3F	1	1	1	1	1	1	1	1	1	1	1	29.41488	1	1	1	1	1	1	1	1
HIST1H3G	1	1	1	1	1	1	1	1	1	1	1	78.75875	1	1	1	1	1	1	1	1
HIST1H4E	1	1	1	1	1	1	1	1	1	1	1	27.43097	1	1	1	1	1	1	1	1
HIST2H2AC	1	1	1	1	1	1	1	1	1	1	1	172.2721	44.05511	1	1	1	1	1	1	1
HIST2H2BE	1	1	1	1	1	66.41131	1	1	1	1	1	1	28.82606	1	1	1	1	1	1	1
HIVEP1	1	42.74117	1	1	43.66802	28.69733	1	1	63.39014	1	87.59687	32.67937	36.47404	1	1	1	1	1	1	70.02264
HIVEP2	1	1	1	57.27696	87.28526	1	1	70.80894	305.4481	34.29422	1	24.6832	1	1	100.4867	1	132.819	1	170.8838	103.6483
HIVEP3	1	1	1	1	58.79432	1	1	36.05376	87.93112	1	80.00032	1	1	117.6858	41.38233	1	92.05504	1	1	1
HJURP	1	1	1	1	1	1	1	1	1	1	1	125.0015	1	1	1	1	1	1	1	1
HK1	1	1	1	1	52.31811	50.75046	1	60.89984	1	1	1	21.45078	39.01405	1	49.35983	1	143.7699	45.10876	1	70.02264
HK2	59.13991	1	1	1	1	1	1	1	1	1	1	1	1	1	1	1	1	1	1	1
HLA.B	342.667	110.7777	1	53.79458	1	69.67366	1	362.8315	1	1	1	49.98629	82.69863	1	1	1	1	429.5701	1	1
HLA.C	291.6528	65.85468	1	46.68397	1	78.48976	1	184.393	1	28.3766	1	49.79996	64.60587	1	1	1	1	234.5754	1	1
HLA.DMA	89.30733	1	1	1	1	1	1	1	1	1	1	1	1	1	1	1	1	1	1	1
HLA.DMB	98.78988	1	1	1	1	1	1	1	1	1	1	1	1	1	1	1	1	1	1	1
HLA.DPA1	169.4456	1	1	1	1	1	1	1	1	1	1	1	1	1	1	1	1	1	1	1
HLA.DPB1	62.76412	1	1	1	1	1	1	28.10933	1	1	1	1	1	1	1	1	1	1	1	1
HLA.DRA	509.8256	1	1	1	1	1	1	1	1	1	1	1	1	1	1	1	1	1	1	1
HLA.DRB1	96.39385	1	1	1	1	1	1	1	1	1	1	1	1	1	1	1	1	1	1	1
HLA.E	465.1222	99.53563	1	1	1	122.0794	1	403.0585	1	1	1	1	270.7052	1	1	237.6522	1	944.4762	1	1
HMCES	1	33.73084	1	1	47.0391	37.33991	1	1	1	44.14744	1	46.14559	29.92774	1	1	1	1	111.6976	1	103.6483
HMG20A	1	1	1	43.24942	45.00614	29.61734	1	34.74729	1	33.77439	1	21.92396	1	1	1	1	1	63.30128	1	70.02264
HMGB2	136.6684	201.2304	95.02175	56.09706	47.91696	121.3916	1	128.8054	1	590.8552	1	2352.811	364.1428	1	1	425.6114	1	73.89619	1	1
HMGCR	1	139.7143	164.9458	62.56011	105.1746	168.6609	1	33.73377	180.462	145.9319	57.63655	115.9547	121.4466	1	150.8927	161.6552	232.7527	21.59108	32.76375	185.5228
HMGN5	1	60.6259	1	1	1	57.37232	1	1	1	98.67354	1	120.9939	35.81158	1	1	68.79228	1	1	1	1
HMGXB3	1	45.26849	30.54688	48.93115	46.58371	35.85632	1	36.62572	1	31.01754	1	34.66198	46.75181	1	1	1	106.4405	66.24696	1	172.671
HMMR	1	1	1	1	1	1	1	1	1	1	1	96.54196	1	1	1	1	1	1	1	1
HMOX1	314.3186	1	1	1	1	1	1	1	1	1	1	1	1	1	1	1	1	1	1	1
HMOX2	1	1	35.05987	1	1	39.35153	1	40.38354	1	1	1	1	24.64325	1	43.25492	1	1	22.60349	1	1

HNMT	117.7063	88.50562	1	1	1	144.8725	1	36.94827	1	48.36437	1	57.37531	75.95367	1	1	118.3857	1	1	1	1
HNRNPA1P	1	1	1	1	1	1	1	1	1	32.39792	1	27.56208	1	1	1	1	1	1	1	1
HOMER1	1	1	45.07447	1	1	1	1	1	97.15723	1	1	1	1	1	48.50171	1	141.3005	17.45583	1	1
HOOK2	1	1	35.16305	54.15742	57.77484	1	1	30.30042	48.16606	1	1	1	30.63261	1	57.16323	1	160.8304	22.65195	1	70.02264
HOPX	1	706.8969	1	1	1	612.0049	1	64.73667	1	1	1	175.4643	1	1	1	658.7301	1	768.7592	1	1
HPCA	1	1	1	1	1	1	1	1	95.11685	1	1	1	1	1	1	1	1	1	1	1
HPCAL1	1	1	1	118.6194	88.84814	1	1	1	1	68.9343	68.18607	40.71463	30.55112	1	1	1	1	55.62935	68.79986	1
HPGD	1	1	1	1	1	1	1	1	1	1	1	1	1	1	1	1	1	142.6449	1	1
HPGDS	357.903	1	1	1	1	1	1	1	1	1	1	1	1	1	1	1	1	1	1	1
HPRT1	63.87298	1	1	1	1	1	1	1	1	48.46988	1	32.10574	1	1	1	1	1	1	1	70.02264
HPS4	1	37.01749	65.24658	58.52211	53.41862	47.50218	1	1	61.82287	33.87444	1	57.37789	35.47096	1	74.38585	1	1	1	1	1
HPS5	1	1	1	1	1	24.72812	1	1	1	32.27711	1	35.23134	1	1	1	1	1	1	1	1
HPSE	116.1668	1	1	1	1	1	1	1	1	1	1	1	1	1	1	1	1	1	1	1
HRASLS	1	1	72.88814	1	1	1	1	1	1	1	1	1	1	1	1	1	1	1	1	1
HRH2	1	1	1	1	1	1	1	1	106.1495	1	1	1	1	1	1	1	1	1	1	1
HRK	1	1	1	1	1	1	1	1	1	1	1	1	46.05931	1	1	1	1	1	1	1
HS2ST1	1	62.69117	1	1	1	38.24822	1	1	1	1	1	38.02483	27.57542	1	1	1	1	18.01751	1	103.6483
HS3ST1	1	1	1	155.2345	164.7672	1	1	1	1	1	1	1	1	1	90.71461	1	97.85096	55.22157	187.1715	1
HS3ST4	1	1	1	1	1	1	1	1	348.1996	1	1	1	1	1	1	1	1	1	1	1
HS6ST1	1	1	1	46.6875	47.0885	44.68237	1	1	86.93605	30.06313	1	27.99277	29.54307	1	80.32911	1	1	34.14221	1	1
HS6ST3	1	1	1	1	60.23275	1	1	1	1	1	1	1	1	1	60.54918	1	1	1	1	1
HSD11B1L	1	1	1	1	1	37.11815	1	1	1	1	1	1	34.7216	1	1	1	1	1	1	1
HSD17B11	65.20449	52.19341	1	48.89606	42.50678	1	1	1	1	34.32408	1	45.04299	1	1	1	44.02797	42.97965	39.16811	1	1
HSD17B14	153.5336	1	1	1	1	1	1	1	1	1	1	1	1	1	1	1	1	1	1	1
HSD17B7	1	1	1	1	1	53.46766	1	1	1	1	1	25.30959	1	1	1	1	1	1	1	1
HSDL2	1	90.28649	1	1	1	138.4058	1	1	1	44.38871	1	72.95581	65.84322	1	1	88.23066	1	1	1	1
HSF1	1	35.41218	1	1	1	23.57499	1	1	1	41.61518	1	32.84035	32.58227	1	1	1	1	36.02315	50.38031	1
HSPA12A	1	1	1	1	1	1	1	53.06613	138.9167	1	1	1	1	1	1	1	1	1	1	1
HSPA12B	1	1	1	1	1	1	1	1	1	1	1	1	1	1	1	1	1	141.5807	1	1
HSPA2	1	45.71095	1	1	1	1	1	216.9259	1	1	1	43.91542	1	1	1	1	1	1	1	1
HSPA4L	1	1	1	1	1	53.11013	1	1	1	1	1	39.65825	48.59242	1	1	67.79801	1	1	1	1
HSPB1	624.9058	264.6339	1	99.61775	1	462.5274	1	322.4705	1	176.1546	1	313.3113	259.073	1	1	284.4605	1	351.0242	1	1
HSPB8	1	1	1	1	1	1	1	1	1	1	1	1	64.37948	1	1	1	1	1	1	1
HSPG2	1	1	1	1	1	1	1	1	1	1	1	1	1	1	1	1	1	173.6369	1	1
HTR2A	1	1	1	1	1	75.20515	1	1	1	1	1	1	39.93141	1	1	1	1	1	1	1
HTRA1	654.6739	194.653	93.15385	55.59305	52.58471	276.8775	1	1	1	134.621	1	124.1186	146.0613	1	47.55255	138.7755	1	202.4963	1	139.0453
HTRA2	1	37.1557	42.98446	1	1	34.47358	1	29.18212	98.57134	41.07633	1	40.62867	36.22932	1	67.38569	1	1	1	1	1
HTRA3	1	1	1	1	1	1	1	208.0454	1	1	1	1	1	1	1	1	1	1	1	1
HUNK	1	1	1	94.17191	104.0233	1	1	1	1	41.02552	1	36.53216	1	1	72.90099	1	1	1	175.982	1
HUS1	1	1	1	1	1	1	1	1	1	1	1	31.95513	1	1	1	1	1	96.43155	1	1
HVCN1	82.19295	1	1	1	1	1	1	1	1	1	1	1	1	1	1	1	1	22.78387	1	1
HYDIN	1	1	1	1	1	1	1	1	1	1	1	1	81.18063	1	1	1	1	1	1	1
HYLS1	1	1	1	1	1	1	1	1	1	1	1	54.89782	1	1	1	1	1	1	1	1
IARS	1	76.80503	61.71342	1	48.77924	58.12452	1	21.7178	1	72.70061	1	67.43544	59.88531	1	57.55802	62.15406	1	29.57541	77.26994	190.2284
ICA1	1	1	1	1	1	1	1	1	96.37973	1	1	1	1	1	1	1	1	41.29061	1	1
ICA1L	1	46.15222	32.67033	81.19199	135.9905	72.03699	1	1	239.7555	66.95965	70.55335	83.89598	97.88918	1	178.7466	69.95117	385.5848	22.07691	100.5703	185.5228
ICAM1	145.4801	1	1	1	1	1	1	30.1945	1	1	1	1	1	1	1	1	1	49.53517	1	1
ICAM2	1	1	1	1	1	1	1	1	1	1	1	1	1	1	1	1	1	277.1386	1	1
ICK	1	52.95351	92.08754	95.34291	73.01618	24.1591	1	1	125.1426	49.24607	102.8647	62.5237	32.10427	90.74559	1	1	1	27.96358	73.87427	1
ID1	1	295.1197	1	1	1	114.8133	1	46.77847	1	75.80385	1	192.9119	339.5691	1	1	110.0628	1	476.8016	1	1
ID3	1	93.61211	1	1	1	1	1	774.258	1	1	1	1	44.6887	1	1	1	1	1113.551	1	1
ID4	1	416.1912	1	55.24059	49.94022	2052.059	1	59.18789	1	181.3384	410.9292	425.3106	1854.854	1	1	1042.764	1	1	1	1
IDH3B	1	49.33554	62.04684	69.55269	65.57217	52.98784	1	47.6688	1	63.05586	76.43555	47.13303	35.64139	78.08592	1	1	1	92.66831	119.5565	1
IER3	1363.483	1	1	1	1	1	1	642.2674	1	1	1	1	68.79295	1	1	1	1	188.2491	1	1
IFI16	297.589	90.78956	31.16353	1	1	1	1	168.5806	1	1	1	1	1	1	1	1	1	249.6029	1	1
IFI27	1	1	1	1	1	1	1	1	1	1	1	1	1	1	1	1	1	623.8026	1	1
IFI27L1	1	32.91712	44.98064	1	1	47.52121	1	45.80745	1	1	1	43.96665	37.50359	1	1	59.55661	1	24.33178	1	70.02264
IFI30	107.5377	1	1	1	1	1	1	1	1	1	1	1	1	1	1	1	1	1	1	1
IFI44	1	1	1	87.08208	75.22744	1	1	32.5666	1	54.91358	84.04966	34.57364	1	214.331	89.00265	1	1	153.2856	107.314	1
IFI44L	1	1	1	1	1	50.43361	1	1	1	1	1	1	1	1	1	1	1	125.4042	1	1
IFI6	1	1	1	1	1	52.28214	1	55.92524	1	1	1	1	51.99899	1	1	37.21378	1	270.7053	1	1
IFIT2	1	1	1	1	1	1	1	50.80116	1	1	1	1	1	1	1	1	1	1	1	1
IFIT3	1	1	1	1	1	1	1	78.67703	1	1	1	1	1	1	1	1	1	47.56205	1	1
IFIT5	1	48.86221	46.77141	40.18535	47.10436	50.53587	1	27.80626	1	41.9354	78.77846	33.40395	58.29081	76.13535	1	1	1	24.39965	1	1
IFITM2	58.68	1	1	1	1	1	1	199.323	1	1	1	1	1	1	1	1	1	260.2785	1	1
IFITM3	1	93.88046	1	1	1	118.5766	1	898.4842	1	1	1	42.03002	129.8604	1	1	73.68297	1	755.5663	1	1
IFNAR2	1	1	1	1	1	1	1	1	1	1	1	1	1	1	1	1	1	168.2569	1	1
IFNGR1	411.7831	48.70255	1	1	1	40.13644	1	53.75797	93.28001	34.55262	1	34.67336	48.78467	1	1	1	1	80.26547	1	1
IFRD2	1	1	1	1	1	1	1	1	1	30.92261	1	1	1	1	1	1	1	24.38382	1	1
IFT122	1	1	1	1	1	1	1	1	1	1	1	34.4739	32.17795	1	1	1	1	1	1	1
IFT46	1	1	1	1	1	27.5732	1	1	1	1	1	24.92686	45.43896	1	1	1	1	1	1	1
IFT57	1	68.91407	23.65357	1	1	96.06077	1	66.21063	1	50.51042	1	60.40066	163.1567	1	1	109.1358	111.9047	1	1	1
IFT74	64.44639	32.73674	1	1	1	32.51909	1	1	1	1	1	44.12225	54.59947	1	1	63.81027	1	1	1	1
IFT80	1	1	1	1	1	38.46838	1	1	1	1	1	33.2663	41.38968	1	1	1	104.4651	1	1	1
IFT88	1	1	1	1	1	28.61811	1	1	1	40.92797	1	37.66425	53.50899	1	66.05742	1	1	1	1	1
IGDCC3	1	40.3357	1	172.82	153.8918	1	1	1	1	108.0091	106.0398	40.25879	1	93.22506	101.2303	1	103.2416	37.787	145.0091	1
IGDCC4	1	47.25928	1	1	1	33.23526	1	1	1	1	1	1	1	1	1	1	1	1	1	1
IGF1	226.8573	1	1	1	1	1	1	1	1	1	1	1	1	124.651	1	1	1	1	1	1
IGFBP4	1	37.58759	1	1	1	80.43916	1	142.5281	1	1	1	27.86061	1	1	1	1	1	224.2803	1	1
IGFBP5	1	57.22877	1	1	1	160.5206	1	1	1	1	1	1	413.5093	1	1	228.9619	1	1	1	1
IGFBP7	1	1	1	1	1	1	1	5474.919	1	1	1	1	1	1	1	1	1	5587.152	1	1
IGFBPL1	1	1	1	61.02642	1	1	1	1	1	88.90748	1	40.82295	1	1	1	1	1	1	1	70.02264
IGLON5	1	1	73.27591	54.10054	63.08555	1	1	1	1	1	1	37.13243	1	79.62513	81.85802	1	1	37.12692	1	70.02264
IGSF11	1	1	39.16012	1	1	32.02803	1	1	1	1	1	1	31.63919	1	1	1	1	1	1	1
IGSF21	1	1	71.84463	1	1	1	1	1	458.2327	1	1	1	1	1	1	1	1	1	1	1
IGSF3	1	1	1	67.30772	95.77766	1	1	1	165.7714	1	1	1	1	1	97.67969	1	206.7394	1	118.0115	1
IGSF6	310.2966	1	1	1	1	1	1	1	1	1	1	1	1	1	1	1	1	1	1	1
IGSF8	1	1	1	59.76255	61.32229	54.00927	1	1	1	1	1	1	1	1	58.93799	1	116.2904	30.69712	52.90666	1
IGSF9	1	1	1	1	1	1	1	1	1	1	1	33.713	1	1	1	1	1	1	1	1
IKBIP	61.13942	1	1	1	1	1	1	70.24665	1	1	1	55.02011	26.23983	1	1	1	1	33.17456	1	1
IKZF1	55.6168	1	1	1	1	1	1	1	1	1	1	1	1	1	1	1	1	1	1	1
IKZF4	1	31.58281	1	1	1	1	1	23.29158	1	1	1	23.93311	31.45759	59.26133	1	1	1	1	1	1
IL10RA	50.31639	1	1	1	1	1	1	1	1	1	1	1	1	1	1	1	1	1	1	1
IL13RA1	116.5447	1	1	1	1	1	1	1	1	1	1	1	1	1	1	1	1	34.91036	1	1
IL13RA2	1	1	1	1	1	32.52511	1	1	1	1	1	1	117.8436	1	1	1	1	1	1	1
IL17D	1	1	1	1	1	40.64554	1	1	1	1	1	1	1	1	1	1	1	1	1	1
IL17RA	42.04189	1	1	1	1	1	1	1	1	1	1	1	1	1	1	1	1	1	1	1
IL17RB	1	1	37.72244	1	1	1	1	1	1	1	1	1	1	1	1	1	1	1	1	1
IL17RD	1	40.78041	1	65.32536	70.7733	60.52426	1	1	1	1	71.46891	27.9702	28.6472	60.85931	61.63327	1	128.1499	55.73658	89.57852	1
IL18	164.6774	1	1	1	1	1	1	1	1	1	1	1	1	1	1	1	1	1	1	1
IL1A	166.0281	1	1	1	1	1	1	1	1	1	1	1	1	1	1	1	1	1	1	1

IL1B	1670.753	1	1	1	1	1	1	1	1	1	1	1	1	1	1	1	1	1	1	1
IL1RAPL1	1	1	1	1	1	1	1	1	74.54726	1	1	1	1	1	1	1	1	1	1	1
IL32	1	1	1	1	1	1	1	1	1	1	1	1	1	1	1	1	1	133.9089	1	1
IL33	1	1	1	1	1	110.5402	1	1	1	1	1	30.01738	72.03828	1	1	1	1	1	1	1
IL34	1	1	1	1	1	1	1	50.97472	1	1	1	1	1	1	1	1	1	1	1	1
IL3RA	1	1	1	1	1	1	1	1	1	1	1	1	1	1	1	1	1	127.1414	1	1
IL6R	86.48827	1	1	1	1	1	1	1	1	1	1	1	1	1	1	1	1	1	1	1
ILDR2	1	88.20312	75.89056	1	1	1	1	1	1	1	1	1	1	1	1	1	1	1	1	1
IMMP1L	1	32.03818	60.76148	1	1	37.79441	1	1	1	45.87788	70.25565	58.27051	31.49154	86.14964	1	31.59136	1	17.0416	1	103.6483
IMP4	36.92738	56.63718	57.10103	1	1	44.27138	1	36.06986	1	39.07181	1	37.37681	36.81564	1	1	42.47891	1	47.39209	1	103.6483
IMPA1	1	1	1	1	1	50.14125	1	1	1	1	1	29.37251	43.86958	1	1	1	1	1	1	70.02264
IMPACT	1	35.07348	74.60832	1	1	45.68236	1	1	1	1	1	23.45441	32.74326	1	1	1	1	27.70111	1	1
INA	1	1	59.89694	1	73.0113	1	1	1	141.4619	1	403.7794	1	1	584.7099	215.8026	1	198.7013	1	1	70.02264
INAFM1	1	1	1	1	1	1	1	89.81021	1	1	1	1	1	1	1	79.52804	1	1	1	70.02264
INAFM2	1	1	1	1	1	1	1	65.44636	1	1	1	1	1	1	1	1	1	1	1	1
INAVA	1	1	45.76244	1	1	1	1	1	1	1	1	1	1	1	1	1	1	1	1	1
INCENP	1	1	1	1	1	1	1	1	1	1	1	49.40818	1	1	1	1	1	1	1	70.02264
ING2	1	53.90673	52.95825	1	61.08206	41.36963	1	56.07169	1	51.83785	96.59381	63.89406	49.00901	74.5573	1	1	1	67.50227	1	70.02264
ING5	1	54.33041	38.07618	1	1	37.0158	1	1	1	35.32128	1	40.57515	36.05389	1	1	56.20932	1	1	73.9861	1
INHBA	1	1	1	1	1	1	1	1	1	1	1	1	1	1	118.0521	1	231.6209	1	1	1
INHBB	1	77.53138	44.37529	122.0765	60.81103	88.3024	1	1	1	81.48914	1	68.62352	132.2883	1	1	41.94577	1	1	1	1
INIP	1	1	43.43994	53.77921	47.93504	38.24046	1	1	1	67.78752	61.25698	54.49163	39.88396	1	38.24662	1	1	33.84826	64.42281	87.58009
INO80	1	50.82575	1	55.79609	53.6052	32.87291	1	31.57538	1	53.51762	69.59281	41.44473	47.30009	68.63426	1	1	51.05561	123.9769	1	1
INO80E	1	1	1	1	1	1	1	1	1	35.76153	1	28.6097	1	1	1	1	1	1	42.10528	1
INPP1	1	1	1	1	1	80.44629	1	1	1	1	1	1	40.44636	1	1	1	1	1	1	1
INPP4A	1	1	1	1	42.47166	1	1	28.63832	83.15125	1	1	1	1	1	1	1	1	1	79.63683	103.6483
INPP4B	1	1	1	1	1	1	1	113.211	1	1	1	1	1	1	1	1	1	1	1	1
INPP5D	68.69099	1	1	1	1	1	1	1	1	1	1	1	1	1	1	1	1	82.6673	1	1
INPP5F	1	50.23419	102.6687	71.5415	102.5877	36.41718	1	1	174.6817	104.2609	93.62741	82.74129	32.40553	127.298	116.028	1	221.1983	30.59531	75.80142	139.0453
INPPL1	1	33.17279	1	1	1	33.14749	1	1	1	1	1	1	1	1	1	1	1	23.48693	1	1
INSIG1	1	123.1899	68.69981	1	80.13876	216.0537	1	45.39011	1	139.7516	1	117.3499	142.313	1	1	149.888	169.3238	164.0889	1	1
INSIG2	1	42.09736	33.25736	52.09491	1	34.83883	1	1	1	43.30699	1	45.33402	29.5391	1	1	1	1	1	1	1
INSM1	1	192.103	58.03095	428.248	456.4672	40.74729	436.5109	1	1	626.974	397.4159	460.5798	151.7426	157.4689	306.8146	79.53029	1	91.38798	391.3264	232.0002
INTS13	1	37.14819	1	1	1	27.02682	1	1	1	1	1	1	1	1	1	1	1	1	1	70.02264
INTS7	1	1	1	1	1	1	1	1	1	30.26649	1	24.38457	1	1	1	1	1	1	1	1
INTU	1	85.2425	1	74.26009	54.07692	335.7016	1	1	1	117.1081	1	125.657	166.0621	1	1	180.3608	206.2368	1	1	1
IP6K1	1	58.69859	66.99274	70.28864	71.40363	37.453	1	38.92856	61.52788	48.64094	119.7807	47.75284	36.60142	89.39087	56.4858	1	1	33.94864	1	87.58009
IPCEF1	87.03258	1	1	1	1	24.32243	1	1	1	1	1	1	1	1	1	1	1	1	1	1
IPO11	1	1	1	1	1	1	1	1	1	1	1	1	1	1	1	1	1	66.35028	1	1
IPO8	1	1	49.17698	1	1	1	1	1	1	1	1	29.1168	1	1	1	1	1	21.7211	1	1
IQCE	1	1	1	1	1	1	1	1	72.75617	1	1	1	44.47886	1	1	1	1	1	1	1
IQCG	1	1	1	1	1	33.17501	1	1	1	1	1	1	88.95354	1	1	1	1	1	1	103.6483
IQCK	1	50.67691	1	1	1	1	1	23.29389	1	1	1	1	62.01168	1	1	1	1	1	1	70.02264
IQGAP2	1	71.4794	1	157.5067	1	456.7141	1	1	1	168.1446	1	180.3021	258.2421	1	1	259.1825	1	163.1823	1	1
IQGAP3	1	1	1	1	1	1	1	1	1	1	1	43.38395	1	1	1	1	1	1	1	1
IQSEC1	1	1	34.83717	1	1	50.42883	1	103.0908	1	1	1	1	1	66.99094	1	1	1	34.80062	1	1
IRAK1	51.9191	1	1	1	1	1	1	1	1	1	1	1	1	1	1	1	1	109.935	1	1
IRAK1BP1	1	1	1	1	1	34.80798	1	1	1	54.23747	1	38.09953	53.91839	1	1	1	1	1	1	1
IRF1	88.34536	109.2141	1	1	1	75.83464	1	502.4873	1	1	1	49.02584	288.5942	1	1	1	1	301.0412	1	1
IRF2BP1	1	1	1	1	1	1	1	1	1	1	1	29.08629	1	1	1	1	1	1	1	1
IRF3	1	39.64602	1	1	1	29.3135	1	26.81875	1	1	1	1	40.95695	1	1	1	1	27.5696	1	1
IRF8	209.5202	1	1	1	1	1	1	1	1	1	1	1	1	1	1	1	1	1	1	1
IRF9	1	1	1	1	1	1	1	1	1	1	1	1	34.54218	1	1	1	1	23.90814	1	1
IRS1	1	1	1	1	44.80783	1	1	1	1	1	1	1	49.39229	1	1	1	1	1	1	1
ISG15	1	1	1	1	1	1	1	209.5162	1	1	1	1	1	1	1	1	1	194.5033	1	1
ISG20L2	1	48.73904	38.3228	1	56.3926	39.15797	1	36.42373	1	38.81049	72.044	36.89947	47.0074	1	1	1	1	29.99451	1	172.671
ISLR2	1	1	1	91.13457	96.01824	1	1	1	284.6493	1	1	1	1	1	106.6498	1	124.916	1	204.3358	87.58009
ISY1	1	31.43826	55.45731	1	1	31.29661	1	22.71687	1	40.87717	1	34.87678	25.92382	1	1	1	1	101.4004	1	103.6483
ISYNA1	1	1	1	50.45644	76.29906	56.93986	1	198.3531	95.29409	44.74467	1	50.30724	51.78094	1	59.92266	48.74867	1	29.33207	1	172.671
ITFG2	1	1	1	1	1	1	1	1	1	1	1	1	1	1	1	1	1	1	72.54736	1
ITGA1	1	1	1	1	1	1	1	702.3982	1	1	1	1	1	1	1	1	1	134.885	1	1
ITGA10	1	1	1	1	1	1	1	55.56769	1	1	1	1	1	1	1	1	1	1	1	1
ITGA11	1	1	1	1	1	1	1	50.75814	1	1	1	1	1	1	1	1	1	1	1	1
ITGA2	1	61.16401	1	1	1	122.2241	1	1	1	49.71178	1	74.50267	193.4605	1	1	57.59773	1	1	1	1
ITGA3	1	1	1	1	1	1	1	1	77.01353	1	1	1	1	1	1	1	1	1	1	1
ITGA4	1	1	1	1	1	1	1	140.3825	1	1	1	1	1	1	1	1	1	1	1	1
ITGA5	1	1	1	1	1	1	1	1	1	1	1	1	1	1	1	1	1	62.68697	1	1
ITGA6	1	59.99272	1	1	1	87.54282	1	1	1	1	1	1	40.68235	1	1	1	1	134.8249	1	1
ITGA7	1	38.23994	1	1	1	1	1	1	1	1	1	1	1	1	1	1	1	1	1	1
ITGA8	1	1	1	1	1	1	1	52.62794	1	1	1	1	1	1	1	1	1	1	1	1
ITGAX	113.2088	1	1	1	1	1	1	1	1	1	1	1	1	1	1	1	1	1	1	1
ITGB2	239.0753	1	1	1	1	1	1	1	1	1	1	1	1	1	1	1	1	1	1	1
ITGB3BP	1	1	1	1	1	1	1	64.98927	1	81.87345	1	96.99228	33.31256	1	1	1	1	1	1	1
ITGB5	1	37.5538	1	1	1	98.55598	1	185.7628	1	1	1	1	52.70213	1	1	108.0183	1	1	1	1
ITGB8	1	316.3966	344.392	104.9546	79.86095	556.4537	1	1	1	157.9859	1	227.0023	483.4164	1	1	439.4683	1	1	1	1
ITIH5	1	1	1	1	1	1	1	1613.783	1	1	1	1	1	1	1	1	1	39.52607	1	1
ITM2A	1	1	93.02694	1	1	1	1	1	1	1	1	1	1	1	1	1	1	3764.028	1	1
ITM2C	288.0953	291.8951	421.0652	1	1	383.9287	1	1125.783	266.1503	1	1	103.0042	172.7	1	1	195.8836	1	1	1	70.02264
ITPR1	1	1	1	1	1	1	1	78.70607	1	1	1	1	1	1	1	1	106.0842	1	1	70.02264
ITPR2	108.7727	64.82948	198.5163	1	1	55.44658	1	185.5999	1	1	1	1	29.87897	1	41.55226	1	113.7766	45.41228	1	1
ITPR3	1	1	1	1	1	1	1	1	1	1	1	1	1	1	1	1	1	43.83922	1	1
ITPRIP	1	1	1	1	1	1	1	55.88819	1	41.44067	1	1	44.28066	1	1	1	1	81.74975	1	1
ITPRIPL2	1	1	1	1	1	1	1	149.2088	1	1	1	1	1	1	1	1	1	34.88206	1	1
JAG1	1	121.2676	1	1	1	44.98588	1	224.08	1	42.35191	1	32.74968	28.8067	1	1	58.48598	1	169.1673	1	1
JAG2	1	1	1	1	1	1	1	1	1	1	1	1	1	1	1	1	1	96.38714	1	1
JAKMIP1	1	1	1	1	1	1	1	1	89.92445	1	1	1	1	1	59.99368	1	170.1335	1	1	70.02264
JAKMIP2	1	61.05204	77.25401	89.48889	80.54507	94.96496	1	1	131.9778	64.22482	230.5759	60.34946	69.62429	283.3565	89.38722	113.6059	234.609	1	1	1
JAM2	1	135.1577	65.78563	84.05032	1	113.5579	1	1	1	101.9199	1	105.0251	115.3523	1	1	80.52371	1	136.3588	1	1
JAM3	1	105.4038	67.18995	1	1	104.6588	1	86.33508	1	36.90796	1	54.5872	111.0051	1	1	116.9822	1	1	1	1
JARID2	1	65.45998	52.99964	54.00866	49.09741	1	1	29.98406	1	31.4371	104.5979	25.29858	1	173.5622	64.16879	1	222.4744	30.46291	1	1
JAZF1	73.86523	1	1	1	1	1	1	1	66.67155	1	1	1	1	64.52162	50.38189	1	36.24776	1	1	1
JCAD	1	1	1	1	1	1	1	37.7316	1	1	1	1	1	1	1	1	1	227.1381	1	1
JDP2	1	37.07966	1	1	1	53.47384	1	1	1	1	1	1	106.1869	1	1	1	1	1	1	1
JHY	1	1	1	1	1	1	1	1	1	1	1	1	35.41514	1	1	1	1	1	1	1
JPH1	1	1	1	1	1	47.78137	1	1	1	1	1	1	1	1	1	1	1	1	1	1
JPH3	1	1	1	1	1	1	1	1	94.21557	1	1	1	1	1	1	1	1	1	1	1
JPH4	1	1	1	1	98.02684	1	1	1	231.8871	1	72.17531	1	1	62.19204	133.4522	1	91.85892	1	101.5015	208.0679
JPT2	1	1	1	1	1	42.64223	1	1	1	52.23978	1	53.58077	31.16562	1	1	39.79359	1	1	1	70.02264
JUNB	1006.286	623.589	187.2513	1	98.39642	431.3998	1	2984.291	1	82.5241	113.9591	169.9714	741.0789	118.4493	1	238.0199	1	2866.01	1	1
JUP	1	1	1	64.57395	55.03914	1	1	1	1	1	1	1	1	1	53.00157	1	1	143.844	1	70.02264
KALRN	1	1	1	157.2265	160.5089	30.53841	1	53.94282	172.4454	83.81785	240.2057	46.52049	31.45133	200.9561	149.1368	118.8078	183.2849	1	124.3415	87.58009

KANK1	1	1	171.3338	1	1	1	1	1	1	1	1	1	1	1	1	1	1	1	1	1
KANK2	1	1	1	1	1	1	1	238.0049	1	1	1	1	1	1	1	1	1	58.01106	1	1
KANK3	1	1	1	1	1	1	1	64.41281	1	1	1	1	1	1	1	1	1	182.921	1	1
KANSL1L	1	41.45856	1	65.95764	58.49678	42.62718	1	40.82849	1	1	1	46.54085	44.50755	1	1	1	1	26.23467	1	139.0453
KANSL2	1	48.91906	1	50.07683	51.79979	31.46674	1	27.09683	1	49.24012	60.24586	50.38857	35.06262	88.13435	1	1	1	20.89123	1	1
KANSL3	1	1	1	1	1	1	1	1	1	29.18993	1	22.84395	1	1	1	1	1	16.11524	1	1
KANTR	1	1	1	52.81893	43.4609	1	1	1	1	1	1	28.06123	1	1	50.05328	1	1	32.76741	1	1
KAT2B	1	92.68508	75.82153	1	1	30.95863	1	1	1	1	1	1	1	1	1	1	1	1	1	1
KAT7	1	115.1088	79.28432	57.74909	47.03326	46.19301	1	48.98984	1	57.69873	60.11371	50.56199	63.00054	64.7064	1	1	1	20.66146	1	139.0453
KAZN	1	37.58663	59.96674	1	1	99.66001	1	86.42723	181.6753	1	1	1	41.57833	1	1	51.44588	1	1	1	1
KBTBD11	1	1	1	1	41.47199	30.53872	1	1	100.3269	1	1	1	1	1	1	1	1	1	1	1
KBTBD3	1	1	1	1	1	1	1	1	1	1	1	1	1	1	1	1	1	112.5059	1	103.6483
KBTBD7	1	1	62.14797	63.2765	64.35994	43.33315	1	1	71.32554	51.88891	82.72037	35.84999	33.62851	90.9929	70.76115	42.31209	1	1	1	1
KCNAB3	1	1	1	1	1	1	1	1	1	43.47366	1	37.57	1	1	1	1	1	1	1	1
KCNB2	1	1	1	1	1	1	1	1	92.22157	1	1	1	1	1	1	1	87.56328	1	1	1
KCNC1	1	1	1	1	41.5544	1	1	1	1	1	1	1	1	1	1	1	1	1	75.82844	103.6483
KCND2	1	45.36966	266.7536	58.38678	66.28544	1	1	1	1	1	1	1	1	1	1	1	1	1	103.9816	1
KCND3	1	73.33719	99.04114	1	1	1	1	1	1	1	1	1	1	1	1	1	1	1	1	1
KCNE4	1	1	1	1	1	1	1	389.9634	1	1	1	1	1	1	1	1	1	1	1	1
KCNE5	1	143.8392	1	1	1	1	1	1	1	1	1	1	1	1	1	1	1	1	1	1
KCNF1	1	37.57465	1	1	1	1	1	1	116.9959	1	1	1	1	73.89236	1	1	1	1	1	1
KCNG1	1	1	1	1	1	49.94147	1	1	205.1112	59.25786	1	80.09256	60.41538	1	1	1	1	1	1	1
KCNH2	1	1	1	1	1	1	1	1	1	1	1	1	1	1	42.55606	1	94.39557	1	1	1
KCNH8	1	1	1	1	1	1	1	1	1	1	65.57101	1	1	1	1	1	1	1	1	1
KCNIP1	1	58.12488	42.68951	1	1	1	1	1	1	1	1	1	1	1	1	1	1	1	1	1
KCNIP3	1	1	57.00242	1	1	1	1	1	1	1	1	1	1	1	1	1	1	1	1	1
KCNIP4.IT1	1	1	38.81288	1	1	1	1	1	1	1	1	1	1	1	1	1	1	1	1	1
KCNJ10	1	38.11735	62.7577	1	1	54.38455	1	1	1	1	1	1	42.33465	1	1	1	1	1	1	1
KCNJ2	1	1	1	1	1	1	1	1	1	1	1	1	1	1	1	1	1	213.9398	1	1
KCNJ3	1	1	1	1	1	1	1	1	1	1	105.2974	1	1	1	1	1	1	1	1	1
KCNJ6	1	1	1	1	1	1	1	1	86.64184	1	1	1	1	1	1	1	1	1	201.9436	1
KCNJ8	1	1	1	1	1	1	1	648.2264	1	1	1	1	1	1	1	1	1	1	1	1
KCNK1	1	1	1	1	1	1	1	1	115.0968	1	1	1	1	1	1	1	1	1	1	1
KCNK10	1	1	1	1	1	68.7057	1	1	1	1	1	1	1	1	1	1	1	1	1	1
KCNK12	1	1	1	1	1	1	1	1	1	1	1	1	1	1	62.83721	1	1	1	1	1
KCNK2	1	1	1	1	62.51547	1	1	1	1	1	1	1	1	1	1	1	48.79715	1	1	70.02264
KCNMA1	1	1	1	1	1	31.4839	1	1	391.5546	1	1	1	43.24144	1	1	1	1	1	1	139.0453
KCNMB4	1	1	1	88.14636	94.50284	1	1	1	197.2711	44.8043	71.51703	1	1	60.74964	47.40125	1	1	23.263	120.9993	1
KCNN3	1	1	1	156.7337	78.09731	96.68651	1	1	1	49.00291	1	37.50943	143.1558	1	1	87.60909	1	1	173.0486	103.6483
KCNQ1OT1	59.48715	426.3039	737.7989	200.4133	289.3344	1	1	396.1888	773.5506	1	229.682	38.64539	1	332.7975	356.4072	1	587.7648	81.51951	1	377.9676
KCNQ2	1	161.4632	228.506	141.3542	114.2549	64.18572	155.6851	1	102.2985	217.8013	115.3662	105.5337	38.83864	122.3662	88.84273	115.3573	1	1	134.3951	1
KCNQ3	1	1	1	368.8954	262.2991	1	230.9131	1	116.5012	139.5966	1	87.9712	29.00231	1	172.2878	1	123.035	1	277.8721	87.58009
KCTD1	1	1	1	1	1	26.03053	1	1	1	1	1	1	37.83525	1	1	1	1	1	1	1
KCTD12	286.5647	1	1	1	1	1	1	1	483.7929	1	1	1	1	112.2663	1	1	1	50.85771	1	1
KCTD13	1	1	1	1	44.54823	1	1	1	1	1	1	26.86517	1	1	1	1	1	1	1	103.6483
KCTD15	1	34.55435	1	50.80026	1	43.97501	1	44.97992	1	122.9465	1	82.66456	68.19358	1	1	76.98357	1	14.41135	1	1
KCTD16	1	1	1	114.3793	70.18147	1	1	1	1	1	1	1	44.04756	1	1	1	1	1	1	70.02264
KCTD2	1	1	40.14251	1	1	1	1	1	1	1	1	1	24.67572	1	1	1	1	1	1	1
KCTD6	1	68.9775	66.33435	121.007	103.3983	123.5854	1	1	1	36.58522	1	64.79769	61.68856	1	1	56.84659	1	27.42872	73.32967	1
KCTD7	1	1	1	1	40.45578	1	1	1	1	1	1	1	1	1	1	1	1	1	1	172.671
KDM4A	1	39.66241	1	46.52835	1	1	1	1	1	31.85824	1	40.16095	23.52627	1	50.00329	1	1	1	1	1
KDM4B	1	61.6618	53.40385	97.569	104.8361	34.02705	182.0594	52.51782	70.39861	34.06065	80.87728	45.95309	35.88313	94.42368	174.9831	1	92.31643	52.89801	100.1087	103.6483
KDM5C	1	1	1	46.37672	1	1	1	23.64874	1	1	1	32.27645	31.93328	1	55.54986	1	142.781	48.67337	1	103.6483
KDM6A	1	36.11531	1	1	1	30.36917	1	34.03593	1	43.22133	1	37.66116	33.39466	1	1	81.7343	1	48.07713	1	1
KDM7A	1	1	1	1	1	1	1	1	1	1	1	1	1	1	1	1	1	59.32139	1	1
KDR	1	1	1	1	1	1	1	1	1	1	1	1	1	1	1	1	1	275.9318	1	1
KHDRBS2	1	1	1	1	1	1	1	1	1	1	1	1	1	1	1	1	173.4646	1	1	70.02264
KHDRBS3	1	35.41939	442.3329	70.06051	84.0622	1	1	31.67035	606.3879	61.88716	1	43.98758	1	1	1	1	93.14186	82.31529	1	103.6483
KIAA0586	1	1	36.24427	50.07042	48.43925	1	1	1	1	35.91775	1	63.78242	27.36839	82.18386	1	1	92.05504	1	1	1
KIAA0895	1	1	1	1	1	1	1	1	1	1	1	1	32.49981	1	1	1	1	1	1	1
KIAA0895L	1	1	37.58842	70.17852	69.99236	1	1	1	174.0277	36.26807	67.93845	32.25972	1	67.06249	92.11984	1	143.8153	1	72.62351	70.02264
KIAA0930	39.13529	1	1	1	43.42466	1	1	1	1	1	1	1	1	1	57.08424	1	1	1	1	1
KIAA1147	1	48.15857	1	1	1	85.38521	1	1	1	31.5651	1	29.68381	34.67042	1	1	39.52917	1	16.46637	1	1
KIAA1211	1	1	1	1	1	1	1	1	1	29.03776	116.1341	1	1	83.89251	1	1	45.94706	1	1	70.02264
KIAA1324	1	1	1	67.0822	86.03531	1	1	1	1	1	1	1	1	1	74.62358	1	1	1	93.37276	70.02264
KIAA1328	1	40.62279	1	1	1	24.33862	1	1	1	1	72.24289	33.46237	1	94.6592	1	1	1	1	1	1
KIAA1468	1	1	1	43.66111	44.06259	1	1	1	53.76305	1	1	28.81635	1	1	1	1	1	1	31.98838	1
KIAA1549L	1	1	1	1	52.2788	1	1	1	145.444	1	1	1	1	1	50.55248	1	1	1	1	1
KIAA1671	1	1	1	1	1	1	1	1	1	1	1	1	1	1	1	1	1	55.96634	1	1
KIAA1958	1	69.28882	48.16792	1	1	48.21685	1	1	1	55.58767	75.97222	49.6383	45.37247	96.93425	1	1	1	1	1	1
KIAA2013	1	1	1	1	1	1	1	49.25942	1	38.69552	1	1	1	1	1	1	1	30.13141	1	103.6483
KIF11	1	1	1	1	1	1	1	1	1	48.06965	1	314.2701	36.26112	1	1	1	1	1	1	1
KIF13A	1	1	90.90665	1	1	1	1	21.35301	1	1	1	1	1	1	1	1	1	21.97938	1	1
KIF14	1	1	1	1	1	1	1	1	1	1	1	128.5997	1	1	1	1	1	1	1	1
KIF15	1	1	1	1	1	1	1	1	1	64.61302	1	257.1828	1	1	1	1	1	1	1	1
KIF18A	1	1	1	1	1	1	1	1	1	1	1	106.9405	1	1	1	1	1	1	1	1
KIF18B	1	1	1	1	1	1	1	1	1	1	1	65.00716	1	1	1	1	1	1	1	1
KIF1A	1	77.8734	110.8409	115.8199	126.0702	202.2117	298.178	1	159.2148	82.47422	150.783	95.0521	150.6134	95.36695	179.2379	216.4048	359.9309	1	176.2751	139.0453
KIF20A	1	1	1	1	1	1	1	1	1	1	1	85.90363	1	1	1	1	1	1	1	1
KIF21B	1	1	40.5885	124.31	101.4707	1	1	1	196.3795	1	123.7241	1	1	177.9661	103.4248	1	1	1	89.23386	139.0453
KIF22	1	1	1	1	1	22.85234	1	28.70354	1	44.71271	1	190.5381	37.29674	1	1	1	1	1	1	1
KIF23	1	1	1	1	1	1	1	1	1	1	1	208.8415	1	1	1	1	1	1	1	1
KIF26B	1	70.48427	1	63.77872	84.2142	71.94737	1	1	1	200.7552	1	76.4556	53.26227	1	186.3615	43.57236	207.5016	1	1	70.02264
KIF27	1	1	1	1	1	1	1	1	1	1	1	1	33.86279	1	1	1	1	1	1	1
KIF2C	1	1	1	1	1	1	1	1	1	1	1	128.5032	1	1	1	1	1	1	1	1
KIF3C	1	1	32.30288	94.96637	113.1078	30.3344	1	1	152.0235	40.13586	132.0789	41.60107	1	139.6636	79.96651	1	1	29.31702	102.8334	1
KIF4A	1	1	1	1	1	1	1	1	1	1	1	150.3847	1	1	1	1	1	1	1	1
KIF5A	1	1	65.6904	151.0683	125.4354	52.10594	186.3119	1	581.7264	46.2344	171.9733	35.2371	36.19317	204.8724	158.2149	41.86662	227.5497	1	142.2617	174.1602
KIF5C	1	132.9553	150.9665	410.825	435.2879	288.3934	658.0752	1	832.5642	285.8454	394.0268	229.4825	132.039	258.3908	486.049	240.5077	501.8266	70.98904	475.1372	449.9687
KIF9	1	1	1	1	1	1	1	1	1	1	1	1	172.8267	1	1	1	1	31.16365	1	1
KIFC1	1	1	1	1	1	1	1	1	1	1	1	89.15782	1	1	1	1	1	1	1	1
KIFC2	1	1	1	1	1	1	1	1	137.0992	1	1	1	1	1	1	1	1	1	1	70.02264
KIRREL1	1	1	1	1	1	1	1	106.2722	1	1	1	1	1	1	1	1	1	1	1	1
KIT	1	1	1	1	1	1	1	1	136.9297	1	1	1	1	1	1	1	1	28.44367	1	1
KITLG	1	1	1	1	1	1	1	84.67764	1	1	166.6348	1	1	1	52.41485	1	115.5502	142.5855	1	1
KIZ	1	1	94.38876	1	1	1	1	1	1	1	1	1	1	64.78085	1	1	1	1	1	1
KLF10	64.02646	50.18467	1	1	69.06072	1	1	174.0382	1	1	1	51.2494	121.2099	1	1	1	1	215.1954	1	1
KLF11	1	1	1	1	49.96581	1	1	47.54166	1	1	1	1	26.67007	1	1	1	1	66.86174	94.69181	1
KLF12	1	85.3722	110.4176	74.25253	44.90859	40.4434	1	53.79502	1	80.0608	164.132	69.61451	52.13006	149.5014	1	40.85718	1	34.13021	82.89544	103.6483
KLF2	1012.966	1	1	1	43.65844	1	1	508.0419	1	1	1	1	51.01219	1	1	1	1	1775.2	1	1
KLF4	112.9666	77.18212	1	1	1	45.07184	1	380.9105	1	1	1	31.45688	36.65483	1	1	1	1	640.2737	1	1

KLF9	1	1	1	1	1	33.00805	1	342.7225	1	1	1	1	35.10896	1	1	1	1	24.19713	1	1
KLHDC8A	1	91.02293	1	1	1	1	1	1	1	1	1	1	1	1	1	1	1	1	1	1
KLHDC8B	1	1	1	1	1	1	1	105.5589	1	1	1	1	1	1	1	1	1	1	1	1
KLHL15	1	1	1	1	1	1	1	87.61285	1	1	1	1	1	1	1	1	1	27.62794	1	1
KLHL2	1	1	1	1	1	1	1	1	73.37792	1	1	1	1	1	36.4812	1	1	19.82187	1	1
KLHL20	1	1	51.6699	1	1	34.82455	1	19.73704	1	39.4901	1	32.82254	37.10112	1	1	1	1	31.28579	76.98039	1
KLHL21	1	1	1	1	1	1	1	1	1	1	1	1	31.54386	1	1	1	1	1	1	1
KLHL25	1	1	1	1	1	34.21084	1	1	1	1	1	22.76611	35.47784	1	41.50679	1	1	1	1	1
KLHL32	1	1	1	1	1	1	1	1	124.8587	1	1	1	1	1	1	1	1	1	1	1
KLHL35	1	1	1	178.4272	194.7109	1	232.0009	1	87.42836	1	326.1707	1	1	1	84.55859	1	1	1	311.5853	1
KLHL36	1	1	1	1	1	1	1	52.71496	1	1	1	1	1	1	1	1	1	1	52.64782	1
KLHL4	1	54.75555	1	1	1	1	1	1	1	1	1	1	1	1	1	1	1	22.00195	1	70.02264
KLHL5	1	1	71.38607	1	1	38.44664	1	35.21437	188.3885	1	1	25.88052	32.5041	1	1	1	1	151.886	1	70.02264
KLHL6	35.84971	1	1	1	1	1	1	1	1	1	1	1	1	1	1	1	1	1	1	1
KLHL8	1	1	45.08509	66.17908	72.37252	1	1	31.34099	1	63.05028	1	53.07526	1	68.33008	108.2344	1	133.7138	1	65.58503	1
KNDC1	1	1	1	1	1	1	1	1	1	1	1	1	27.1478	1	1	1	1	1	1	1
KNL1	1	1	1	1	1	1	1	1	1	35.57311	1	258.2227	1	1	1	1	1	1	1	1
KNOP1	1	94.55478	62.13477	56.27802	49.16316	56.89815	1	81.21236	123.4151	102.1253	1	65.08624	56.04975	1	49.20898	89.28024	140.8035	36.10114	98.18705	116.5001
KNSTRN	1	1	1	1	1	1	1	1	1	1	1	188.6071	1	1	1	1	1	1	1	1
KNTC1	1	1	1	1	1	1	1	1	1	48.42699	1	47.42934	1	1	1	1	1	1	1	1
KRBOX1	1	1	1	1	1	31.1324	1	1	1	40.64105	1	52.68586	1	1	1	1	1	1	1	1
KRCC1	1	51.30236	39.99227	1	1	60.8529	1	28.05124	1	51.46474	1	39.27891	57.75327	1	1	86.60808	1	36.99446	1	1
KREMEN1	1	1	1	1	1	1	1	1	1	1	1	1	1	1	54.3161	1	1	1	1	1
KRI1	1	1	53.19467	1	1	1	1	1	1	1	1	42.12994	34.92529	1	1	1	1	1	1	1
KTI12	1	1	1	1	1	1	1	1	1	1	1	1	27.62921	1	1	1	1	113.9598	1	1
KXD1	65.23645	57.20207	47.6479	47.59043	42.1505	52.97386	1	37.14515	1	64.33764	1	48.60169	53.10524	1	1	51.82459	1	51.56816	1	103.6483
L1CAM	1	1	1	1	66.94119	1	1	1	177.8258	1	1	1	1	66.95344	63.35133	1	276.5929	1	55.93428	70.02264
L3HYPDH	1	1	1	1	1	31.00585	1	1	1	1	1	1	22.36829	1	1	1	1	1	1	1
L3MBTL1	1	1	1	1	1	1	1	1	68.94225	1	1	1	1	1	1	1	1	1	1	1
L3MBTL3	1	1	1	1	1	1	1	1	1	1	1	1	1	1	1	1	70.43027	23.73457	1	1
LACTB	70.27197	1	1	1	1	27.52082	1	43.84453	1	1	1	1	1	1	1	1	1	29.82549	1	1
LAIR1	57.10506	1	1	1	1	1	1	1	1	1	1	1	1	1	1	1	1	1	1	1
LAMA1	1	1	1	1	1	50.17077	1	1	1	1	1	23.53396	30.51705	1	1	1	1	24.53955	1	1
LAMA2	1	1	1	1	1	1	1	155.2708	1	1	1	1	1	1	1	1	1	24.58357	1	1
LAMA4	1	1	1	1	1	1	1	521.4713	1	1	1	1	1	1	1	1	1	314.0101	1	1
LAMB1	1	1	1	1	1	1	1	292.5733	1	1	1	1	1	1	1	1	1	144.8395	1	1
LAMB2	1	1	1	1	1	36.51995	1	104.416	1	1	1	1	27.34883	1	1	1	1	53.07525	1	1
LAMC1	1	1	1	1	1	1	1	259.7245	1	1	1	1	1	1	1	1	1	145.1819	1	1
LAMC3	1	1	1	1	1	1	1	201.162	1	1	1	1	1	1	1	1	1	1	1	1
LAMP5	1	38.05048	1	1	1	45.89264	1	1	1	1	1	1	46.14584	1	1	1	1	1	1	1
LAPTM5	548.3261	1	1	1	1	1	1	1	1	1	1	1	1	1	1	1	1	19.55325	1	1
LARP1B	1	51.89723	27.30449	1	42.55118	35.45848	1	1	1	45.69591	1	61.33119	47.93721	1	1	1	171.222	26.59313	30.12242	1
LARP6	1	1	1	1	1	1	1	1	123.9079	1	1	1	1	1	1	1	1	26.9603	1	1
LAS1L	1	1	33.37945	47.33511	40.20396	1	1	28.13632	1	42.54475	1	35.30214	27.43999	1	1	1	34.40398	27.27953	1	1
LAT2	58.96408	1	1	1	1	1	1	1	1	1	1	1	1	1	1	1	1	1	1	1
LATS2	1	1	1	1	1	1	1	40.48031	1	1	1	1	45.02464	1	1	1	1	72.40472	1	1
LBH	1	1	71.7517	71.95978	106.9193	1	1	166.3549	1	1	179.9975	1	1	150.0977	74.18835	1	1	104.5422	1	70.02264
LCA5	1	1	28.18997	56.19007	49.10493	40.42747	1	41.5422	1	1	1	32.46905	72.02855	1	1	1	1	1	42.71822	1
LCP1	284.5532	1	1	1	1	1	1	1	1	1	1	1	1	1	1	1	1	1	1	1
LCP2	60.96977	1	1	1	1	1	1	42.89982	1	1	1	1	1	1	1	1	1	1	1	1
LDAH	1	1	1	1	1	1	1	1	1	27.79107	1	1	1	1	1	1	1	1	1	1
LDB1	1	1	1	82.83446	75.59206	1	1	21.83642	1	37.61907	1	1	74.37132	73.77411	1	1	1	29.05585	107.6615	103.6483
LDB2	1	1	1	1	1	47.25704	1	33.57092	214.6604	30.06621	1	1	30.52509	1	1	40.74091	1	33.01396	1	1
LDLR	1	167.736	54.65345	1	46.04708	219.489	1	91.23657	1	140.68	1	143.286	327.6578	1	1	204.5477	233.9435	170.5736	1	1
LDLRAD3	1	144.5526	52.95476	1	1	27.20822	1	46.5072	1	67.4224	1	57.65085	22.38434	1	1	1	1	39.73837	1	1
LDLRAD4	34.31784	62.24406	32.88335	96.03942	91.78475	29.22074	1	1	1	73.15562	1	42.88364	70.98431	139.6725	76.80258	1	155.0984	1	1	139.0453
LDOC1	1	1	41.11574	88.1567	122.1188	43.28976	1	1	1	1	1	1	24.453	118.3691	89.24968	1	58.0172	1	110.6836	206.2967
LEF1	1	1	1	1	1	1	1	57.56392	1	1	1	1	1	1	1	1	1	380.3942	1	1
LEMD1	1	1	1	46.08963	41.39369	1	1	1	1	1	1	1	1	1	56.44173	1	1	1	1	1
LEMD2	1	39.43674	1	1	1	1	1	1	1	1	1	1	1	1	1	1	1	40.48037	1	70.02264
LETM1	1	1	30.24889	1	41.21498	26.36584	1	1	96.3182	24.51101	1	1	33.57217	1	1	1	1	25.99905	117.2312	70.02264
LETMD1	1	1	1	1	1	25.14609	1	16.99592	90.59451	1	1	1	32.07322	1	1	49.26192	1	1	1	1
LFNG	1	63.72415	1	1	1	58.42249	1	1	1	1	1	1	28.50838	1	1	1	1	25.77927	1	1
LGALS1	1258.825	331.8118	1	1	1	435.6945	1	1474.686	1	263.3738	1	443.1138	590.5885	1	1	492.0341	1	136.1621	1	1
LGALS3	817.0067	412.8077	1	1	1	362.1889	1	1	1	39.4635	1	92.53101	61.65936	1	1	287.4943	1	1	1	1
LGALS3BP	1	37.88445	1	1	1	75.82457	1	77.42216	1	35.84848	1	1	51.88402	1	1	57.89024	1	1	1	1
LGALS8	54.68316	1	1	1	1	1	1	45.46241	1	1	1	1	1	1	1	1	1	21.84012	1	1
LGALS9	72.43885	1	1	1	1	1	1	1	1	1	1	1	1	1	1	1	1	32.47665	1	1
LGALSL	1	1	1	1	1	49.15596	1	29.81983	1	1	1	1	43.26494	1	1	1	1	1	1	103.6483
LGMN	462.2886	1	39.70281	1	1	26.71689	1	67.48486	1	1	1	1	1	1	1	1	1	24.97799	1	70.02264
LHFPL2	97.17012	1	1	1	1	1	1	1	1	1	1	1	1	1	1	1	1	66.41447	1	1
LHFPL3	1	1	110.6402	1	1	1	1	1	1	1	1	1	1	1	1	1	1	1	1	1
LHFPL6	1	35.02645	70.54917	1	1	1	1	1063.071	1	1	1	1	34.76179	1	62.48937	1	1	97.30964	1	1
LHX2	1	449.1055	107.2188	515.2164	484.5342	772.5528	440.4309	1	1	628.5177	1	570.7241	458.0134	1	324.6733	478.3777	245.3735	1	303.356	103.6483
LHX6	1	1	1	1	1	1	1	1	1	1	1	1	1	390.6116	1	1	1	1	1	1
LIAS	1	38.24777	1	51.68936	1	1	1	1	1	47.94353	1	39.66582	30.3547	1	1	1	1	31.76169	50.93224	1
LIFR	1	116.5637	1	1	1	211.1569	1	30.6017	1	56.04713	1	119.0408	68.50952	1	1	146.1873	1	60.20248	1	1
LIG1	1	1	1	1	1	1	1	1	1	82.53191	1	83.20754	28.50345	1	1	1	1	1	1	1
LIG3	1	34.13909	1	1	1	32.31777	1	1	1	45.10189	1	38.5053	24.80674	1	1	70.8037	1	1	1	70.02264
LILRA2	62.06266	1	1	1	1	1	1	1	1	1	1	1	1	1	1	1	1	1	1	1
LILRB4	178.3817	1	1	1	1	1	1	1	1	1	1	1	1	1	1	1	1	1	1	1
LIMA1	1	330.2191	620.6767	1	1	101.7709	1	172.8444	1	110.9297	109.1936	77.88234	120.3561	1	1	107.2072	1	56.98157	1	1
LIMCH1	1	41.37367	1	1	106.949	146.0219	138.4462	1	484.729	1	1	47.30744	293.7822	1	462.2054	75.48721	460.5759	80.52603	85.52129	411.5933
LIMD1	1	1	1	1	1	1	1	1	1	36.21103	1	40.2748	35.53027	1	1	1	1	1	1	1
LIMK1	1	1	1	1	1	1	1	1	1	1	1	1	1	1	1	1	1	32.41539	56.65986	1
LIMK2	1	1	1	1	1	1	1	1	1	1	1	1	1	1	1	1	1	67.79571	1	1
LIMS2	1	1	1	1	1	1	1	1	1	1	1	1	1	1	1	1	1	72.3148	1	1
LIN52	1	1	1	1	1	1	1	1	1	25.82259	1	37.02855	1	1	1	1	1	1	1	1
LIN54	1	1	1	1	1	1	1	1	1	1	1	36.26443	1	1	1	1	1	21.51697	1	1
LIN7A	1	1	1	1	1	1	1	37.7453	1	1	1	1	1	1	72.84753	1	1	1	1	1
LIN9	1	1	1	1	1	1	1	1	1	1	1	35.72717	1	1	1	1	1	1	1	1
LINC00511	1	66.4924	127.9554	1	1	26.21342	1	1	1	1	1	1	1	1	1	1	1	1	1	1
LINC00595	1	1	1	1	1	26.78596	1	1	1	1	1	1	1	1	1	1	1	1	1	1
LINC00599	1	1	1	73.7626	115.7854	1	1	1	233.231	1	64.63522	1	1	132.8946	140.5804	1	491.499	1	68.84536	103.6483
LINC00623	1	1	1	1	1	41.43101	1	1	1	1	1	1	31.29026	1	1	1	1	1	1	1
LINC00632	1	90.90053	116.8903	127.564	152.0606	126.1761	1	1	263.9729	119.2304	245.2683	126.2798	119.268	127.3152	83.76654	80.96279	1	1	76.58268	70.02264
LINC00667	1	45.79796	46.53557	62.214	51.77689	36.42457	1	66.04566	1	42.40458	1	33.23149	35.37656	66.33959	59.78734	1	1	1	49.95177	232.0002
LINC00672	1	1	39.78645	1	1	1	1	1	1	1	1	1	1	1	1	1	1	1	1	70.02264
LINC00685	1	1	1	1	1	34.76821	1	1	1	1	1	1	1	1	1	1	120.8287	1	1	1
LINC00909	1	34.06911	52.95269	45.42034	1	49.66435	1	28.02578	1	54.14673	68.89142	43.42578	42.71036	1	1	56.16688	1	1	1	103.6483
LINC00943	1	66.27303	1	1	1	131.527	1	1	1	1	1	34.95956	1	1	1	67.09678	1	1	1	1

LINC00982	1	1	1	1	1	103.0322	1	1	1	27.38375	1	43.38017	40.74957	1	1	50.73747	1	1	1	1
LINC01003	1	73.2368	99.46706	1	42.94636	48.26251	1	1	1	35.71279	1	1	34.24076	1	51.58001	1	1	1	1	70.02264
LINC01089	1	1	1	47.31962	40.81941	1	1	1	1	34.70682	1	1	1	1	53.03437	1	1	1	1	1
LINC01094	128.1456	1	1	1	1	1	1	1	1	1	1	1	1	1	1	1	1	1	1	1
LINC01102	1	49.73348	57.56783	167.338	242.3897	41.17706	258.7883	1	1	46.98376	1	43.31154	28.47148	1	384.2663	81.39245	204.4485	1	121.105	232.0002
LINC01122	1	1	1	125.8815	144.7844	1	1	1	298.8245	1	70.78289	1	1	136.8113	132.7852	1	281.3262	1	178.1164	87.58009
LINC01197	1	1	1	1	1	1	1	56.40443	1	1	1	1	1	1	1	1	1	1	1	1
LINC01224	1	1	1	1	1	1	1	1	1	1	1	32.89124	1	1	1	1	1	1	1	1
LINC01235	121.5352	1	1	1	1	1	1	1	1	1	1	1	1	1	1	1	1	1	1	1
LINC01315	1	1	1	1	1	1	1	1	1	1	1	1	1	1	48.70219	1	1	1	1	1
LINC01351	1	1	61.73494	1	1	36.69605	1	1	1	1	1	1	1	1	1	1	1	1	1	1
LINC01551	1	77.85435	1	250.0508	271.9521	103.294	343.266	1	1	344.3705	77.32302	186.9278	74.33101	86.52561	300.5967	194.6838	123.6802	1	394.2911	206.2967
LINC01736	140.0455	1	1	1	1	1	1	1	1	1	1	1	1	1	1	1	1	1	1	1
LINC01896	1	175.4763	249.7676	1	1	180.4315	1	1	1	57.28371	1	84.23286	78.83666	1	1	85.65988	1	1	1	1
LINC01965	1	69.4961	1	1	1	127.7725	1	1	1	155.1072	1	93.59296	98.90291	1	1	195.1042	1	1	1	1
LINC02001	1	1	43.72833	1	49.95272	1	1	1	1	43.87969	1	39.9103	33.44392	59.82553	1	1	1	1	69.23604	1
LINC02147	1	1	1	1	1	1	1	61.12494	1	1	1	1	1	1	1	1	1	1	1	1
LINC02172	1	1	1	1	1	1	1	49.02827	1	1	1	1	1	1	1	1	1	1	1	1
LINGO1	1	1	87.08535	1	84.89662	1	1	187.5539	104.0047	1	1	1	1	1	149.8176	1	126.5777	1	96.30435	174.1602
LINS1	1	1	1	1	1	1	1	1	1	1	1	1	1	1	1	1	1	1	52.0934	1
LIPA	121.9182	1	1	1	1	25.80515	1	38.18284	1	1	1	1	1	1	1	1	1	40.52493	1	1
LIPG	1	1	1	1	1	152.5472	1	1	1	36.0635	1	28.17095	94.61194	1	1	63.18381	1	1	1	1
LITAF	180.167	112.4357	53.64296	47.50179	1	244.4297	1	1	1	93.9738	1	104.6414	130.8272	1	1	148.2505	1	14.76588	1	1
LMBRD1	1	47.03194	52.74352	51.16856	56.43096	72.70089	1	79.15002	154.0998	30.44094	1	36.82926	48.43309	75.47014	1	80.53761	39.77054	46.37164	63.8412	103.6483
LMBRD2	1	1	43.74647	1	1	23.48512	1	1	111.0019	1	1	1	1	1	1	1	1	1	1	1
LMCD1	1	1	1	1	1	1	1	1	1	1	1	1	1	1	1	1	1	158.1397	1	1
LMNA	1	120.6763	1	59.63584	44.1082	147.1433	1	788.2052	1	52.7836	1	50.59281	481.7417	1	1	142.8414	1	133.2519	1	1
LMNB2	1	1	23.77571	1	1	1	1	59.74448	1	46.41744	1	133.3738	41.60984	1	1	1	1	17.53973	1	1
LMO1	1	1	29.62907	1	1	1	1	1	1	1	1	1	1	1	1	1	1	1	1	1
LMO2	86.54546	81.18553	1	1	1	92.84794	1	91.21834	1	60.37275	1	40.95686	87.2364	1	1	1	1	1316.637	1	1
LMO3	1	108.6387	1	74.12655	98.5821	128.9262	1	1	1777.861	1	126.3518	50.2568	67.86193	101.6056	1	207.9575	84.32185	1	53.29864	1
LMO7	1	1	1	1	1	1	1	33.66394	613.5012	1	1	41.08163	1	1	1	1	1	1	1	70.02264
LMTK2	1	1	1	1	1	1	1	1	96.70705	1	1	1	1	1	1	1	1	1	1	1
LNPEP	1	57.17153	1	1	1	50.51877	1	27.03062	1	53.10968	1	59.46237	48.74708	65.56622	1	57.59224	1	29.09114	1	1
LNX1	1	1	1	1	67.08732	1	1	1	1	1	1	1	1	1	77.44099	1	1	1	94.51928	116.5001
LONP1	1	36.12407	1	1	1	29.41029	1	26.86575	89.59094	1	1	28.25367	27.91071	1	1	1	1	1	1	1
LONRF2	1	37.42173	42.90645	50.9657	63.76379	55.64662	1	1	242.1283	28.54529	83.62638	1	46.6345	1	108.1896	1	40.85678	1	82.80224	1
LOXL1	1	1	1	1	1	49.78922	1	1	1	1	1	1	107.2177	1	1	84.36201	1	1	1	1
LPAR1	1	1	1	1	1	40.43104	1	1	1	1	1	1	1	1	1	1	1	1	1	1
LPAR2	1	1	1	63.00834	61.32707	41.36431	1	1	1	40.82434	1	1	20.79157	1	36.55975	44.82175	1	1	1	1
LPAR4	1	1	1	1	1	34.71929	1	1	1	1	1	1	1	1	1	1	1	1	1	1
LPAR5	66.1466	1	1	1	1	1	1	1	1	1	1	1	1	1	1	1	1	1	1	1
LPAR6	88.09271	1	1	1	1	1	1	75.20334	1	1	1	1	1	1	1	1	1	241.1321	1	1
LPCAT1	1	67.5556	33.30986	1	1	153.3413	1	1	1	118.4897	1	101.6312	71.0948	1	1	139.1435	1	1	40.0119	70.02264
LPCAT2	56.22991	1	1	1	1	1	1	1	1	1	1	1	1	1	1	1	1	1	1	1
LPIN1	1	40.22418	45.74499	61.05223	63.89637	1	1	27.10476	1	46.10447	94.69396	29.80969	34.82083	73.56039	62.63727	1	128.7925	1	83.82119	1
LPL	152.7653	1	1	1	1	1	1	165.1258	274.4888	1	1	29.12748	1	1	1	1	210.233	1	1	70.02264
LPP	45.53776	72.55582	108.9793	1	1	60.84593	1	256.3665	1	1	1	33.85086	62.2843	1	1	1	1	17.86533	1	1
LRCH2	1	1	1	49.33576	46.96232	23.78725	1	36.02347	1	40.33489	123.1748	1	1	95.46115	52.07971	1	1	1	1	103.6483
LRFN4	1	1	1	84.86483	55.41047	29.29144	1	1	1	57.41357	1	38.98233	29.41658	1	61.91493	1	1	1	93.27702	1
LRIG1	1	215.9191	1	1	1	236.2376	1	77.01128	1	58.47667	1	65.73119	110.4485	1	1	124.9047	1	1	1	1
LRIG2	1	1	45.73876	1	1	37.86662	1	1	1	1	1	1	1	1	1	1	1	1	1	1
LRIG3	1	1	1	1	1	44.44214	1	1	1	49.57426	1	37.85407	69.54142	1	1	1	1	1	1	1
LRP10	1	46.2664	1	1	1	86.05814	1	33.058	1	1	1	34.10431	48.49935	1	1	1	1	82.87456	1	1
LRP12	1	36.46461	48.31634	94.1716	88.13528	1	1	1	1	85.24314	90.33606	47.3676	32.31588	70.36106	85.01735	1	1	28.79059	41.15317	1
LRP1B	1	1	173.2711	1	1	1	1	1	260.307	1	1	1	1	1	1	1	1	1	1	1
LRP2BP	1	1	1	1	1	1	1	1	1	1	1	1	41.09618	1	1	1	1	1	1	1
LRP3	1	57.12341	70.65582	51.18901	65.2554	49.47325	1	1	161.0941	53.57649	1	42.62408	31.26824	71.23766	71.3936	84.59455	89.10047	1	72.47333	103.6483
LRP4	1	49.24437	1	1	1	58.6273	1	1	1	1	1	1	30.35378	1	1	1	1	1	1	1
LRP8	1	53.44551	1	638.8297	401.6111	37.49957	206.1789	1	213.7695	367.5862	81.86573	131.8217	84.2995	56.22448	127.9685	83.22107	178.5438	203.2784	373.9142	1
LRR1	1	1	1	1	1	1	1	1	1	39.22268	1	64.73987	1	1	1	1	1	1	1	1
LRRC1	1	1	32.20936	1	1	41.98051	1	1	1	1	99.59323	1	30.69896	1	1	1	1	1	1	1
LRRC10B	1	1	1	1	1	73.70497	1	1	1	1	1	1	33.36533	1	1	1	1	1	1	1
LRRC17	1	75.90805	1	1	1	127.7694	1	41.11586	1	32.23292	1	1	1	1	1	1	1	71.47602	1	1
LRRC23	1	1	1	1	1	1	1	33.98324	1	1	1	1	61.56817	1	1	1	1	1	1	1
LRRC24	1	1	1	1	1	1	1	1	135.72	1	1	1	1	1	1	1	1	1	1	103.6483
LRRC27	1	1	1	1	1	28.66997	1	1	1	1	1	1	50.58958	1	1	1	1	1	1	116.5001
LRRC32	1	1	1	1	1	1	1	128.3816	1	1	1	1	1	1	1	1	1	1	1	1
LRRC34	1	1	1	1	1	1	1	1	1	1	1	1	61.59805	1	1	1	1	1	1	1
LRRC36	1	1	1	1	1	1	1	1	1	1	1	1	1	1	1	1	1	59.00201	1	1
LRRC37B	1	39.00745	1	1	1	42.69402	1	1	1	51.94051	1	40.35767	30.34329	1	1	1	1	1	1	1
LRRC39	159.5892	1	1	1	1	1	1	1	1	1	1	1	1	1	1	1	1	1	1	1
LRRC3B	1	80.85059	1	1	1	550.6504	1	1	1	63.47907	1	122.6741	176.6306	1	1	270.3315	1	1	1	1
LRRC4	60.06311	1	76.59132	1	1	42.30581	1	1	79.88195	1	1	27.2268	41.12293	1	1	1	1	1	1	1
LRRC42	1	28.50274	1	1	1	58.50814	1	1	1	53.80821	1	45.9019	55.12271	1	1	82.01715	1	1	1	1
LRRC49	1	43.35756	32.61872	66.95572	54.98769	41.5883	1	1	96.67847	56.0972	102.8235	47.29619	57.17009	74.91929	39.32089	1	1	1	46.12273	1
LRRC4C	1	59.7072	178.8416	1	1	45.46043	1	1	120.2837	1	1	1	29.34612	1	1	1	113.5854	1	1	1
LRRC55	1	1	1	1	1	1	1	1	1	1	1	1	1	1	1	1	1	73.50732	1	1
LRRC57	1	42.98616	1	1	1	38.61302	1	1	1	29.86623	1	46.15103	27.70143	1	1	103.0751	1	18.89896	1	1
LRRC58	1	126.1681	115.6633	93.91778	99.45944	152.2665	1	75.41415	1	130.556	97.64263	143.8769	157.15	99.79377	62.93604	127.7809	1	1	1	103.6483
LRRC6	1	1	1	1	1	1	1	1	1	1	1	1	37.74256	1	1	1	1	1	1	1
LRRC61	1	1	49.18044	1	1	1	1	1	1	1	1	1	26.47144	1	70.89296	1	1	1	1	103.6483
LRRC7	1	1	76.55535	284.0407	409.876	1	329.4605	1	283.7531	1	224.6054	1	1	166.3948	481.709	1	276.5548	1	322.1115	344.3419
LRRC75B	1	1	1	1	1	38.87527	1	1	1	1	1	1	31.99533	1	1	1	1	1	1	1
LRRC8A	1	53.3056	39.58705	56.17828	56.11567	28.21666	1	14.26712	1	51.71201	80.18521	58.68182	41.95031	67.89847	1	1	1	46.29897	1	103.6483
LRRC8B	1	1	47.15791	1	1	1	1	37.50679	1	1	1	25.38519	1	89.72454	1	1	1	1	1	1
LRRC8D	1	1	38.22797	1	1	1	1	1	1	26.11006	1	1	1	1	1	1	1	1	1	1
LRRC9	1	1	1	1	1	1	1	1	1	1	1	1	1	42.17746	1	1	1	1	1	1
LRRCC1	1	51.74666	1	1	1	69.29849	1	1	1	53.14309	1	77.52151	139.7105	1	1	73.8232	1	1	1	1
LRRIQ1	1	1	1	1	1	32.2882	1	1	1	1	1	27.94506	185.4641	1	1	177.4023	1	1	1	1
LRRK1	37.34844	1	1	1	1	1	1	1	1	1	1	1	1	1	1	1	1	1	1	1
LRRK2	1	1	56.09273	1	1	1	1	1	1	1	1	1	1	1	1	1	1	1	1	1
LRRN1	1	129.3707	131.5556	1	1	28.26491	1	1	1	1	66.70889	38.69614	38.78307	98.15694	1	1	75.67985	1	1	70.02264
LRRN3	1	89.5139	103.8666	1	1	59.24826	1	34.33427	1	1	149.7958	1	75.45231	1	1	1	1	1	1	70.02264
LRRTM2	1	75.27969	100.8492	147.2457	198.1706	48.02777	1	1	194.7996	141.7359	133.6708	99.5074	1	125.0581	117.2386	1	316.3967	30.05704	107.989	103.6483
LRRTM3	1	132.9738	164.3	1	1	97.75079	1	1	117.2558	1	1	37.51405	1	1	1	1	1	1	1	1
LRRTM4	1	1	62.53107	1	1	1	1	1	79.88485	1	1	1	1	1	1	1	1	1	1	1
LRTOMT	1	1	1	1	1	1	1	1	1	1	1	1	39.34882	1	1	1	1	1	1	1
LRWD1	1	1	1	1	1	1	1	1	1	1	1	1	32.6081	1	1	1	1	1	1	1
LSAMP	1	139.8333	554.0692	83.30527	1	218.882	1	104.3541	131.5978	89.24615	1	84.5734	101.2667	104.4269	1	124.3187	105.8336	1	1	1
LSM11	1	1	1	1	1	1	1	1	136.2408	1	1	1	1	1	1	1	1	1	1	70.02264

LSR	1	1	1	1	1	1	1	1	1	1	1	1	1	1	1	1	1	134.438	1	1
LSS	1	1	1	1	1	73.36765	1	1	1	41.82803	1	30.01924	24.43292	1	1	76.16909	1	1	1	1
LST1	256.5409	1	1	1	1	1	1	1	1	1	1	1	1	1	1	1	1	1	1	1
LTA4H	1	1	52.1652	54.69162	1	60.40085	1	1	1	53.19385	1	36.84932	65.59926	1	1	75.74742	1	64.6485	1	70.02264
LTBP1	1	66.09509	1	1	1	225.3427	1	1	1	95.52787	1	94.04242	254.9224	1	1	261.2711	1	63.58301	1	1
LTBP4	1	1	1	1	1	31.79353	1	177.1237	58.81206	40.39245	1	39.5319	43.90649	1	1	1	1	1	1	1
LTBR	51.86223	1	1	1	1	1	1	67.56494	1	1	1	1	1	1	1	1	1	31.5263	1	1
LTC4S	231.7446	1	1	1	1	1	1	1	1	1	1	1	1	1	1	1	1	1	1	1
LUM	1	1	1	1	1	1	1	217.945	1	1	1	1	1	1	1	1	1	1	1	1
LURAP1L	1	1	1	1	1	1	1	99.26779	1	1	1	1	1	1	1	1	1	46.00285	1	1
LUZP2	1	326.1617	415.2121	1	1	1	1	1	188.3088	1	1	1	1	1	1	1	1	1	1	1
LXN	1	1	1	1	1	1	1	1	1	1	1	1	1	1	1	1	1	259.0872	1	1
LY6E	1	1	53.44498	1	63.30551	1	1	1	1	1	1	1	26.57397	1	103.5997	42.68703	78.6104	160.9152	1	1
LY6H	1	65.03222	156.4843	110.9535	212.633	249.5492	231.5304	1	552.3652	34.18894	180.59	44.75473	173.332	150.2137	265.9756	193.0271	366.5243	1	157.2939	241.6936
LY86	418.9363	1	1	1	1	1	1	1	1	1	1	1	1	1	1	1	1	1	1	1
LY96	214.0709	1	1	1	1	1	1	1	1	1	1	1	1	1	1	1	1	1	1	1
LYAR	1	37.36435	64.10006	1	1	1	1	1	1	39.80858	1	36.9582	1	1	1	1	1	1	1	87.58009
LYL1	162.374	1	1	1	1	1	1	1	1	1	1	1	1	1	1	1	1	95.70757	1	1
LYN	173.3729	1	1	1	1	86.1757	1	35.24689	1	1	1	1	1	1	1	1	1	140.9208	1	1
LYPD1	1	1	124.7884	1	1	47.19328	1	39.24359	1	56.23035	1	31.36406	28.25131	1	1	1	1	1	1	1
LYPLA2	1	1	38.56166	1	1	1	1	1	1	1	1	1	47.91905	1	1	1	1	111.6126	1	1
LYPLAL1	1	31.29856	1	1	1	61.38877	1	1	1	1	1	26.32306	32.31658	1	1	1	1	1	1	1
LYSMD2	72.48403	1	25.12022	1	1	1	1	1	1	1	1	1	1	1	1	1	1	35.54234	1	1
LYST	69.66246	1	1	1	1	1	1	27.2251	1	1	1	1	1	1	1	1	116.4449	15.98418	1	1
LZTFL1	1	1	1	1	1	22.85571	1	1	1	1	1	1	60.91301	1	1	1	1	1	1	1
LZTR1	1	1	1	1	1	1	1	1	1	1	1	1	1	1	1	1	81.65652	1	1	1
LZTS1	1	1	1	1	1	1	1	171.7381	1	45.38893	1	1	1	1	1	1	1	1	1	1
MAATS1	1	1	1	1	1	1	1	1	1	1	1	1	53.80696	1	1	1	1	1	1	1
MACO1	1	1	85.00774	182.0354	196.6064	30.35048	221.5081	77.42912	124.6623	88.18352	194.6542	67.13629	29.09499	223.446	209.9797	1	119.3519	45.73894	230.5284	208.0679
MACROD1	1	1	1	1	1	1	1	1	1	1	1	32.51477	1	1	1	1	1	1	1	1
MAD1L1	1	1	1	1	1	1	1	1	1	1	1	1	1	61.52802	1	1	92.67176	1	1	1
MAD2L1	1	34.22774	92.47227	1	1	1	1	1	1	119.5439	1	338.6895	66.16612	1	1	72.89493	1	1	1	1
MAD2L1BP	1	1	1	1	1	24.0701	1	1	1	31.63128	1	27.55723	1	1	1	1	1	1	1	1
MADD	1	1	1	1	1	1	1	1	122.2372	1	1	25.63897	1	1	1	1	1	1	1	70.02264
MAF	461.7454	1	1	1	1	1	1	65.38932	1	1	1	1	1	1011.638	1	1	1	1	1	1
MAFB	412.413	51.24115	1	1	1	1	1	107.7714	1	1	129.6638	1	1	287.2675	1	1	1	1	1	1
MAFF	252.5642	112.4231	43.52187	1	43.47506	101.9815	1	328.9554	1	34.51314	1	52.80847	248.4733	1	1	195.2115	1	266.401	1	1
MAFK	1	1	1	1	1	1	1	1	1	1	1	1	1	1	1	1	1	56.62245	1	1
MAGI1	1	186.9099	182.7686	1	1	101.8676	1	1	53.75881	209.0228	270.3827	165.7458	108.5638	188.2585	1	146.8932	1	53.28846	1	1
MAGI2	1	51.37403	142.0436	99.42067	83.56092	78.44225	1	1	438.947	60.69224	113.8593	42.70015	62.10934	329.8858	70.67519	80.82825	1	27.27333	43.33508	139.0453
MAGI3	1	37.66775	1	62.93395	64.05886	52.94478	1	52.52279	1	57.46372	1	61.95278	36.5817	1	46.85194	1	119.4247	22.00434	1	1
MAK	1	1	1	1	1	1	1	1	1	1	1	1	29.97415	1	1	1	1	1	1	1
MAL2	1	1	1	1	1	1	1	1	141.5035	1	1	1	21.20779	1	1	1	1	1	1	1
MAML1	1	43.95229	1	56.81184	54.98995	34.66657	1	25.73561	1	48.88525	60.32517	53.59902	28.61946	75.95609	1	1	1	21.76074	1	103.6483
MAML2	74.01822	105.4486	155.0316	1	1	58.95139	1	83.79271	1	33.3701	1	32.5273	43.58399	1	1	1	1	16.08478	1	70.02264
MAML3	1	1	1	1	1	1	1	1	77.50662	1	1	1	1	1	1	1	1	26.30195	1	1
MAN1C1	1	1	1	1	1	1	1	26.05625	1	1	1	1	1	1	1	1	111.2652	1	1	1
MANEAL	1	1	1	1	1	1	1	1	1	1	1	1	1	1	1	1	1	1	49.95262	1
MAOA	1	1	1	1	1	39.15926	1	1	1	1	1	1	1	1	1	1	1	36.66338	1	1
MAP2K3	58.11343	1	1	1	1	1	1	39.72929	1	1	1	1	1	1	1	1	1	62.748	1	1
MAP2K4	1	1	60.17801	1	1	1	1	28.32399	107.6345	39.00539	1	27.68688	38.96107	1	1	1	1	19.241	66.47245	1
MAP2K6	1	1	1	1	1	29.25862	1	1	1	1	1	1	26.19002	1	47.25039	1	1	98.30187	1	103.6483
MAP3K1	1	170.2726	145.4955	1	1	1	1	30.60673	1	1	1	1	1	1	1	1	1	37.62803	1	1
MAP3K11	1	1	1	1	1	1	1	37.75545	1	1	1	1	1	1	1	1	1	152.5235	1	103.6483
MAP3K12	1	32.62637	1	1	40.78202	28.28458	1	38.37568	1	40.22466	1	24.28752	26.19554	1	1	1	1	18.57891	1	1
MAP3K14	1	1	1	1	1	1	1	1	1	1	1	1	31.99559	1	1	1	1	1	1	1
MAP3K19	1	1	1	1	1	1	1	1	1	1	1	1	38.53062	1	1	1	1	1	1	1
MAP3K20	1	49.52112	1	61.56108	1	52.03035	1	153.0372	1	99.67487	1	82.71626	56.17984	111.5407	1	42.44824	1	31.87602	1	1
MAP3K3	1	1	1	1	1	1	1	31.08071	1	1	1	1	1	1	1	1	1	68.70277	1	1
MAP3K4	1	45.79692	52.63547	1	1	25.8436	1	1	1	35.9835	1	40.91009	28.56608	1	1	1	97.64516	27.65944	1	1
MAP3K5	1	1	1	1	1	34.52997	1	1	1	1	1	1	29.60677	1	1	1	1	1	1	1
MAP3K7	1	1	83.21678	46.5946	45.39715	38.017	1	1	1	40.08242	1	46.01436	39.14822	1	1	1	1	20.75725	89.54971	1
MAP4K2	1	1	1	1	1	1	1	1	1	1	1	1	1	1	1	1	140.5658	112.913	1	1
MAP4K3	1	1	1	50.95464	41.74798	30.59264	1	80.72078	1	44.04185	1	27.85261	39.20564	1	1	1	1	88.05561	1	1
MAP6	1	36.71005	1	186.2784	190.0521	59.74136	168.1272	1	424.6676	164.9959	1	75.7568	100.4581	1	202.1202	81.58597	323.1152	1	172.8011	346.1132
MAP7D2	1	1	1	1	1	1	1	1	45.02688	1	1	1	1	1	1	1	1	1	1	1
MAP7D3	1	1	1	1	1	1	1	1	1	1	1	1	31.15412	1	1	1	1	1	1	1
MAPK11	1	1	1	1	1	1	1	1	1	1	1	1	1	1	34.66148	1	1	38.99013	1	103.6483
MAPK4	1	1	1	1	1	30.83714	1	1	1	1	1	1	1	1	1	1	1	1	1	1
MAPK7	1	37.35877	1	51.77633	44.69219	29.3791	1	1	1	34.53898	1	42.24347	34.32499	1	31.44893	1	1	20.37201	1	1
MAPK8IP1	1	73.98767	46.00457	43.44429	55.38151	171.5332	1	1	1	38.934	1	52.95551	112.603	1	1	143.0259	1	1	1	1
MAPK8IP2	1	1	42.60892	51.14306	72.74259	1	1	1	155.4101	1	68.06612	1	1	59.85966	115.9358	1	70.84331	1	97.5062	1
MAPKBP1	1	1	1	1	1	1	1	1	56.55484	1	1	1	1	1	1	1	1	41.86444	1	1
MAPRE3	1	1	70.3216	1	42.71633	1	1	1	180.4922	1	1	1	29.64612	1	46.49329	1	1	1	179.4756	308.945
Mar-01	1	1	1	1	1	35.36428	1	1	1	1	1	1	22.28578	1	1	1	1	1	1	1
Mar-02	1	1	1	1	1	52.55039	1	1	1	1	1	1	1	1	1	1	1	49.55125	1	1
Mar-01	1	1	87.91858	65.92349	1	1	1	1	1	61.56981	1	43.19265	1	1	1	1	1	1	1	1
Mar-02	60.94299	1	1	1	1	29.45583	1	23.35883	1	1	1	1	1	1	1	1	1	45.24859	1	1
Mar-03	99.94191	1	1	1	1	1	1	1	1	1	1	1	1	1	1	1	1	53.19684	1	1
Mar-04	1	1	1	1	1	1	1	1	84.13823	1	1	1	1	1	1	1	1	1	1	70.02264
MARF1	1	1	1	61.95708	50.41241	1	1	1	1	33.78837	1	46.34168	26.85907	67.56129	64.78725	37.22086	1	19.70789	1	1
MARK2	1	1	1	1	49.67628	38.38671	1	36.34309	1	40.10212	1	42.12171	33.32728	1	40.88495	1	1	23.80661	1	1
MASP1	1	1	54.81635	1	1	77.13883	1	1	1	1	1	1	98.1926	1	137.8541	1	75.96227	1	1	103.6483
MAST1	1	1	1	43.55008	62.10323	1	1	1	63.49705	1	1	1	1	1	66.07171	1	103.5139	1	1	1
MAST2	1	1	1	1	41.47188	1	1	1	1	1	60.54687	25.1935	1	51.28277	53.78021	1	1	1	68.46541	174.1602
MAST4	1	72.49363	1	1	1	128.192	1	114.4438	1	31.17496	1	40.15464	116.7801	1	1	115.6757	1	376.9233	1	1
MASTL	1	1	1	1	1	1	1	1	1	60.15272	1	61.00214	1	1	1	1	1	1	1	1
MATR3.1	1	34.42723	1	53.9116	49.34405	1	1	1	1	45.97508	1	36.93481	26.84145	1	1	1	82.4329	23.19244	1	1
MB21D2	1	1	26.36686	1	55.3883	25.84596	1	1	1	1	1	1	1	1	108.701	1	1	1	1	1
MBD6	1	1	54.56993	1	1	28.93041	1	1	1	1	1	29.97827	28.55937	1	1	1	1	13.00306	1	1
MBP	85.17376	1	440.3155	1	1	1	1	1	1	1	1	1	1	1	1	1	1	1	1	1
MBTD1	1	59.61707	36.434	70.69736	64.54729	39.68092	1	1	1	50.91228	1	49.40588	35.95584	73.72209	1	1	118.4691	40.98786	1	1
MCAM	1	81.69014	186.4083	1	1	26.6427	1	640.1754	1	1	1	53.99217	88.41841	1	1	1	1	158.555	1	1
MCC	1	60.19689	1	1	1	1	1	1	1	1	1	1	1	1	1	1	1	25.17848	1	1
MCCC1	1	1	43.03568	1	1	25.73724	1	1	1	1	1	1	23.4724	1	1	1	1	1	1	206.2967
MCCC2	1	1	1	1	1	41.47557	1	1	1	1	1	1	31.07036	1	1	1	1	102.2608	1	1
MCEE	1	1	31.86701	1	1	1	1	1	1	1	1	1	1	1	1	1	1	1	1	1
MCF2L	1	1	1	69.45535	64.39014	1	1	1	67.11552	1	67.15494	1	1	67.64477	56.62053	1	83.30366	67.11457	54.34164	1
MCM10	1	1	1	1	1	1	1	1	1	91.56032	1	78.04288	1	1	1	1	1	1	1	1
MCM2	1	1	1	1	1	1	1	1	1	131.3591	1	69.38002	35.30318	1	1	72.99166	1	1	1	1
MCM3	1	73.2999	36.23151	1	1	41.44491	1	30.16874	1	232.3651	1	94.87329	62.18187	1	1	117.2202	1	34.9973	1	1

MCM3AP.A	1	1	1	1	1	1	1	1	1	1	1	1	1	1	94.63234	1	1	1	1	103.6483
MCM4	1	82.48815	77.83667	1	1	43.76088	1	1	1	186.4872	1	124.0652	66.50944	1	1	111.3343	1	1	1	1
MCM5	1	48.49893	63.06355	1	1	1	1	1	1	144.5649	1	55.55059	24.25356	1	1	63.8678	1	1	1	1
MCM6	1	65.87845	1	1	1	40.50465	1	1	1	139.6266	1	61.85325	42.0069	1	1	57.93088	1	27.20553	1	70.02264
MCM8	1	1	1	1	1	1	1	1	1	47.29801	1	48.72404	1	1	1	1	1	1	1	1
MCOLN1	80.09978	1	1	1	1	1	1	1	1	1	1	1	1	1	1	1	1	1	1	103.6483
MCRIP2	86.03017	1	1	1	1	1	1	1	1	1	1	1	1	1	1	1	1	1	1	1
MCTP1	1	1	1	1	1	1	1	1	149.0265	1	1	1	1	1	69.77202	1	1	1	1	70.02264
MCUB	1	1	1	1	1	55.63834	1	1	1	28.80811	144.0827	66.71478	51.74182	1	1	36.78441	1	1	1	1
MCUR1	53.33565	45.88084	22.3643	57.23563	183.6403	58.35088	297.0665	32.33664	1	46.48129	1	58.85202	54.35681	1	428.1723	68.51892	284.4869	46.12902	73.974	275.3193
MDC1	1	1	1	1	1	1	1	27.53061	1	40.00396	1	49.80967	1	1	1	1	1	1	1	1
MDFIC	1	1	1	1	1	61.87435	1	61.1005	1	35.83231	1	52.10124	40.23321	1	1	84.27608	1	61.98403	1	1
MDGA2	1	1	58.10567	1	1	1	1	1	130.3277	1	1	1	1	1	1	1	1	1	1	1
MDK	1	321.9203	129.4927	335.8655	244.5208	606.8507	340.6031	292.7432	1	791.7858	1	493.143	355.6218	94.90672	191.827	663.6771	1	91.02274	308.833	103.6483
MDM1	1	1	1	1	1	41.23593	1	1	1	36.68202	1	39.32487	54.60496	1	1	76.93341	1	1	1	70.02264
MDN1	1	41.7508	48.02167	48.03554	45.08459	1	1	1	57.86284	42.45817	1	1	32.38017	1	1	37.98255	1	68.28805	138.2381	1
ME3	1	1	1	1	1	1	1	1	1	36.88026	1	1	1	1	1	1	1	1	1	1
MEA1	65.84566	57.16618	51.66797	48.30638	50.29461	70.84289	1	50.78773	117.9536	69.60849	1	78.70911	55.06049	1	44.97765	89.3807	1	29.35629	1	172.671
MECOM	1	1	1	1	1	1	1	1	1	1	1	1	1	1	1	1	1	194.2825	1	1
MECR	1	1	47.93402	1	1	1	1	1	1	1	1	1	1	1	1	1	1	1	1	139.0453
MED12L	1	1	1	44.57238	1	1	1	1	1	88.98275	1	58.83868	55.17929	1	1	1	1	1	1	1
MED17	1	1	1	47.5388	1	1	1	25.55063	1	35.78985	1	30.46622	26.95718	1	1	1	83.17096	1	1	87.58009
MED27	1	36.54912	23.30068	1	1	30.71871	1	42.34949	1	31.90917	1	34.68102	28.19882	1	58.59481	1	1	1	1	1
MED7	1	1	1	1	1	33.26338	1	1	1	1	1	42.64185	26.48148	1	1	1	1	1	1	1
MEF2C	340.1027	1	1	1	160.4613	1	249.4248	432.0427	1372.144	1	268.1449	1	31.3467	556.323	571.2204	104.371	3165.996	138.8584	62.41796	1864.611
MEF2D	1	1	1	1	44.38617	1	1	103.0898	1	1	1	1	31.5954	1	1	1	1	72.3128	1	70.02264
MEG3	1	1	382.1386	187.5939	274.8737	1	1	2120.929	2386.205	54.89364	1609.875	1	1	1005.495	1	1	466.7772	288.1386	174.6486	622.2038
MEG8	1	1	1	1	1	1	1	193.5112	1	1	1	1	1	1	1	1	1	1	1	1
MEGF10	1	34.27652	1	1	1	94.94208	1	1	1	44.36811	1	34.66217	41.20385	1	1	54.13026	1	1	1	1
MEIS2	1	374.0314	112.4732	1082.591	1040.918	722.8114	656.699	1	193.0527	783.4819	181.97	614.0362	486.0268	1	547.0785	658.1371	607.9988	180.2227	926.4376	347.5003
MEIS3	1	1	57.04532	101.8629	103.0798	47.98873	130.1344	23.79764	96.11002	41.55883	99.80917	1	41.07382	126.1866	87.01656	1	46.80094	1	79.39076	1
MELK	1	1	1	1	1	1	1	1	1	39.43323	1	101.669	1	1	1	1	1	1	1	1
MEMO1	1	1	35.63659	49.64217	46.69148	1	1	1	1	1	1	1	1	1	80.86018	1	70.43027	1	1	1
MERTK	58.11559	1	1	1	1	1	1	1	1	1	1	1	1	1	1	1	1	14.77485	1	1
MESP1	1	1	1	1	1	1	1	1	93.32743	1	1	1	1	1	1	1	1	1	1	1
MEST	1	244.0078	108.6246	1	1	111.7148	1	85.29007	1	92.4431	1	65.81048	57.77407	1	1	100.5964	1	1	1	1
METRN	1	640.6656	961.5011	1	1	388.282	1	62.24283	1	80.91584	1	160.9006	435.6156	1	1	479.3387	1	1	1	1
METRNL	55.77914	49.74877	1	1	1	60.68061	1	33.71242	1	1	1	1	56.03774	1	1	1	1	1	1	1
METTL14	1	1	1	49.03794	1	27.64968	1	1	1	57.33461	1	36.62617	38.03502	1	1	1	1	1	1	1
METTL21A	1	1	1	1	1	30.96942	1	32.04052	1	28.2494	1	27.4371	33.35669	1	1	49.85248	1	34.79637	1	1
METTL23	77.31016	40.9642	25.6714	1	1	51.96212	1	51.92038	1	47.9745	1	32.51804	51.03307	1	1	1	1	41.82112	57.40958	1
METTL2A	1	40.53872	29.62401	1	39.43133	34.79152	1	1	1	43.483	1	42.7437	28.64963	1	1	63.51401	1	1	54.10225	172.671
METTL2B	1	35.0653	1	51.30221	46.52648	39.82474	1	1	1	37.26054	1	44.18671	35.70108	1	1	1	1	20.71254	1	103.6483
METTL4	1	1	1	1	1	1	1	1	1	1	1	38.63293	1	1	1	1	1	39.38816	1	1
METTL6	1	1	1	1	1	36.41622	1	1	1	1	1	1	24.52407	1	43.38593	1	103.1347	1	1	70.02264
METTL7A	1	44.78416	1	1	1	90.64005	1	1	1	1	1	41.02637	38.01775	1	1	1	1	1	1	1
METTL8	1	1	1	1	1	37.27462	1	1	1	1	1	1	1	1	1	1	1	1	1	1
MEX3B	1	1	92.60565	205.9278	231.2563	1	123.3365	59.37998	185.6259	126.5211	198.5477	68.34489	25.48799	305.4927	149.5197	1	111.8326	41.08736	120.8913	103.6483
MFAP2	1	1	1	69.84409	1	50.31946	1	55.73542	1	87.23879	1	49.09382	70.23285	1	1	50.11909	1	53.65766	1	70.02264
MFAP4	1	1	1	1	1	1	1	1	1	36.25586	1	1	75.21858	1	1	1	1	1	1	1
MFHAS1	1	34.57102	1	1	51.11808	54.99923	1	1	1	25.33554	1	29.91147	68.11157	57.39454	35.30108	1	1	88.56373	1	1
MFNG	1	1	1	1	1	1	1	1	1	46.4233	1	1	1	1	1	1	1	49.11608	1	1
MFSD1	144.7067	1	43.56313	1	1	1	1	1	1	1	1	1	1	1	1	1	1	1	1	1
MFSD12	51.87465	1	1	1	1	1	1	1	1	1	1	1	1	1	1	1	1	24.54835	1	103.6483
MFSD14B	1	1	1	1	1	41.24496	1	38.30735	1	28.07473	1	25.6731	26.92901	1	1	78.11152	1	25.92244	1	1
MFSD2A	1	1	1	1	1	1	1	1	1	1	1	1	1	1	1	1	1	618.7126	1	1
MGAT1	50.97723	1	1	1	1	1	1	110.0114	1	1	1	1	1	1	1	1	1	163.5293	1	1
MGAT4A	78.62745	1	1	1	1	1	1	1	1	1	1	1	1	1	1	1	1	31.91832	1	1
MGAT4C	1	1	1	1	1	129.3494	1	1	197.7372	1	1	40.83928	48.40017	1	1	79.77531	1	1	1	139.0453
MGAT5	1	1	1	1	1	1	1	74.21697	1	1	1	1	1	1	1	1	1	25.77823	1	103.6483
MGLL	103.4983	1	49.8741	1	1	148.9325	1	591.8024	177.8778	1	1	1	1	1	1	51.95674	118.1877	123.8908	1	1
MGME1	1	36.28796	36.93742	1	1	42.41586	1	1	1	40.35056	1	78.01176	30.43196	1	1	42.84411	1	1	1	1
MGMT	83.78849	34.16741	49.14709	1	1	56.49734	1	93.04826	1	1	1	39.09164	53.05514	1	1	78.62256	1	30.92763	1	1
MGP	1	1	1	1	1	1	1	339.648	1	1	1	1	1	1	1	1	1	1	1	1
MGRN1	1	53.94686	63.91789	62.99725	64.08934	38.46474	1	1	1	51.17538	96.27835	31.52497	45.92984	65.5255	1	1	1	1	55.94499	70.02264
MGST2	200.4679	1	1	1	1	1	1	32.81612	1	1	1	1	1	1	1	1	1	305.2719	1	1
MIAT	1	1	1	324.2234	368.6707	1	1	1	655.7276	35.51462	483.6526	1	1	403.0477	377.7305	1	834.9592	25.96729	309.8316	232.0002
MICAL1	48.48673	1	1	50.50252	50.74586	1	1	1	1	1	1	27.04039	1	1	49.21656	1	1	1	1	103.6483
MICAL2	1	1	1	1	1	1	1	1	70.40082	1	1	1	1	1	1	1	1	1	1	1
MICAL3	1	1	44.41045	1	69.41769	33.76144	1	1	130.1141	1	83.00783	28.71819	37.23012	62.37382	95.28835	1	93.30639	1	1	139.0453
MID1	1	48.06412	1	69.99183	55.54202	141.1828	1	1	1	64.29277	1	100.2691	222.0934	1	64.55186	124.4158	1	1	1	1
MID1IP1	1	76.11257	1	1	1	70.96984	1	81.03244	1	81.23994	1	53.45195	64.69019	1	1	1	1	1	1	139.0453
MIF4GD	1	1	46.98189	1	1	1	1	1	1	1	1	1	1	1	1	1	1	1	1	1
MINDY3	1	1	1	1	1	25.33946	1	1	1	1	1	1	1	1	1	1	1	25.67483	79.88175	103.6483
MINPP1	1	1	1	1	1	25.10668	1	1	1	49.36329	1	36.55434	30.69504	1	1	1	1	1	1	103.6483
MIR124.2H	1	1	1	81.23644	81.83362	1	1	1	189.4878	38.81459	174.8564	27.14717	1	316.1151	119.6395	1	110.1565	1	79.99364	149.721
MIR137HG	1	1	1	1	1	1	1	1	203.1333	1	1	1	1	1	1	1	1	1	1	70.02264
MIR181A1I	1	1	32.45931	60.65395	66.24732	1	1	1	1	41.66236	1	30.01739	1	187.408	56.19837	1	98.10287	37.9222	67.85421	103.6483
MIR222HG	1	1	1	1	1	34.32026	1	1	1	1	1	1	1	1	1	1	1	16.59761	1	1
MIR22HG	1	1	1	1	1	1	1	237.219	1	1	1	1	1	1	1	1	1	43.15442	1	1
MIR4435.2	1	1	1	1	1	1	1	947.4909	1	1	1	1	1	1	1	1	1	84.56595	1	1
MIR9.3HG	1	50.82843	51.07814	60.77218	46.82219	52.11177	1	1	1	74.55846	1	47.50979	59.54222	1	1	82.70009	126.7763	1	1	1
MIS12	1	1	1	1	1	20.56427	1	1	1	40.7443	1	38.20352	32.80462	1	1	1	1	1	1	1
MIS18A	1	45.25185	1	1	1	1	1	42.14455	1	71.78827	1	83.69145	30.35636	1	1	1	1	1	1	1
MIS18BP1	118.3032	53.71264	51.93011	50.38973	1	36.34142	1	76.40738	1	113.1226	1	371.2534	60.91842	1	1	153.3191	1	40.28084	1	70.02264
MITD1	1	35.34839	1	1	1	34.98302	1	32.95766	1	42.19623	1	31.27563	37.49	1	1	1	1	26.6918	67.50178	1
MITF	67.74934	1	1	1	1	1	1	1	1	1	1	1	1	1	1	1	1	1	1	1
MKI67	1	1	1	1	1	1	1	1	1	102.0966	1	986.6226	80.17417	1	1	90.67292	1	1	1	1
MKNK1	176.8328	1	1	1	1	1	1	1	1	1	1	1	1	1	1	1	1	17.46237	1	1
MKRN2	1	1	30.65009	1	37.5991	35.18973	1	1	1	29.99498	1	32.87356	26.61345	1	1	1	107.4909	39.36627	1	1
MLC1	1	140.9355	1	1	1	182.4307	1	1	1	106.3563	1	88.28659	150.3087	1	1	188.9431	1	1	1	1
MLLT1	1	30.13084	1	45.84333	44.34532	54.80056	1	49.71592	1	1	1	47.97375	110.5168	58.86295	1	65.93874	1	19.68659	1	1
MLLT10	1	88.56851	83.85859	74.97797	58.1891	71.44012	1	55.03987	1	109.9138	76.46057	95.90647	71.03256	1	1	81.78606	1	53.23595	80.80387	149.721
MLYCD	1	1	1	1	1	1	1	1	1	1	1	1	1	1	1	1	121.1562	1	1	1
MMAB	1	1	34.64765	1	1	56.3557	1	1	1	42.15346	1	31.50439	32.14847	1	1	68.15622	1	1	1	1
MMD	62.50987	1	86.8085	1	45.57468	27.11159	1	30.30419	98.37306	50.48309	1	44.45752	23.8191	1	91.21925	1	67.09577	174.8874	1	277.0906
MMP14	1	1	1	1	1	1	1	47.63824	1	1	1	1	1	1	1	1	1	1	1	1
MMP15	1	1	1	1	1	1	1	54.65767	1	1	1	1	1	1	1	1	1	1	1	1
MMP16	1	56.58446	413.569	91.94166	55.66037	25.45239	1	44.34202	180.4413	72.80969	1	49.77974	38.71113	58.33916	1	1	1	1	1	1
MMP2	56.3776	90.70123	49.93892	1	1	1	1	1	1	1	1	1	1	1	1	1	1	31.40577	1	1
MMP25	1	1	1	1	1	1	1	49.04781	1	1	1	1	1	1	1	1	1	64.21986	1	1

MMP28	1	1	1	1	1	1	1	1	1	1	1	1	1	1	1	1	1	173.1619	1	1
MMRN2	1	1	1	1	1	1	1	1	1	1	1	1	1	1	1	1	1	88.7722	1	1
MMS22L	1	51.09747	1	52.8392	52.60808	57.0931	1	1	1	150.7155	1	182.1241	38.90871	1	43.64296	82.00864	1	21.14556	1	1
MN1	1	50.7974	47.46968	269.7768	391.1579	57.39518	423.3349	1	96.99398	70.80211	147.3731	88.41689	31.88849	213.0925	422.8225	128.9197	263.367	1	275.2	139.0453
MND1	1	1	1	1	1	1	1	1	1	34.65024	1	82.80894	1	1	1	1	1	1	1	1
MNDA	318.2276	1	1	1	1	1	1	1	1	1	1	1	1	1	1	1	1	1	1	1
MNS1	1	1	1	1	1	1	1	1	1	65.86602	1	106.4706	129.4136	1	1	1	1	1	1	1
MNT	1	1	1	1	44.73882	1	1	23.13373	1	1	1	1	36.66315	1	1	1	1	19.59044	1	139.0453
MOB1A	189.2405	95.23121	37.5968	69.94393	54.00517	90.7485	1	123.2402	1	110.3353	1	91.81628	104.7746	56.5677	1	58.48146	1	44.68791	117.0456	116.5001
MOB2	1	1	1	1	1	28.08661	1	30.12573	1	31.66433	1	25.60463	31.79247	1	1	1	1	93.91053	107.9051	103.6483
MOB3B	1	90.0801	94.71342	1	1	258.891	1	17.88306	1	73.62241	198.0412	85.51033	31.968	293.0527	1	109.7682	1	25.29533	1	1
MOCS2	1	70.44432	1	1	1	98.6639	1	39.40624	1	31.90273	1	46.65379	50.14277	1	1	75.96901	1	29.05997	1	1
MOK	1	1	1	1	47.34637	27.30456	1	1	1	1	1	1	98.54536	1	39.19708	1	112.0057	1	1	277.0906
MON2	1	38.67051	1	55.36204	52.35217	39.01567	1	33.70578	1	39.93461	1	26.93181	35.09715	65.16103	1	1	122.2647	16.75064	1	1
MORC2	1	1	1	1	44.3962	1	1	1	1	1	1	1	1	57.53186	1	1	1	1	1	139.0453
MORC2.AS	1	1	1	1	40.28402	1	1	1	1	1	1	1	1	74.79263	1	1	1	1	1	1
MORC4	1	34.85641	1	1	1	23.28183	1	1	1	1	1	36.87297	1	1	1	1	1	1	1	1
MORN2	1	54.9556	73.49723	1	1	65.38131	1	1	1	37.05061	1	41.01497	164.7005	1	1	79.96331	1	25.76798	1	70.02264
MORN3	1	1	1	1	1	1	1	1	1	1	1	1	35.53913	1	1	1	1	1	1	1
MORN5	1	1	1	1	1	1	1	1	1	1	1	1	46.70734	1	1	1	1	1	1	1
MOSPD3	1	40.97283	1	42.93712	47.25142	30.82026	1	1	1	1	1	28.90679	1	1	47.37192	1	1	19.74903	1	103.6483
MOXD1	1	105.1903	1	1	1	370.8684	1	1	1	52.27368	1	120.0945	40.74698	1	1	203.2462	1	1	1	1
MPDU1	1	50.80868	31.02627	1	1	32.60909	1	43.93997	1	62.68519	1	47.33729	24.42199	1	1	1	1	34.93731	49.23901	1
MPEG1	46.22644	1	1	1	1	1	1	1	1	1	1	1	1	1	1	1	1	1	1	1
MPG	41.98954	37.87135	22.02835	1	1	49.20103	1	74.20434	1	30.53068	1	27.77528	50.23814	1	1	1	1	21.95488	67.10145	1
MPHOSPH(	1	44.63233	90.87087	1	39.71958	40.93711	1	28.21016	1	38.09075	1	34.72007	33.17117	79.97155	1	1	1	14.23285	1	103.6483
MPHOSPH!	1	51.28513	35.11355	69.87513	50.31547	32.86514	1	1	1	71.50953	1	117.1393	37.55729	1	45.70382	1	1	1	110.0455	1
MPP1	115.6545	1	1	1	1	1	1	1	1	1	1	1	1	1	1	1	1	193.4818	1	1
MPP3	1	1	1	1	1	1	1	1	1	1	1	1	1	1	65.11675	1	1	1	1	70.02264
MPP5	1	54.96664	1	1	1	94.53646	1	1	1	35.13631	1	49.60615	59.07022	1	1	49.37891	1	1	1	1
MPP6	1	50.61575	37.2633	1	83.63821	34.38901	1	1	245.0469	50.81136	1	64.30685	37.58657	1	216.2188	80.57356	152.3203	1	1	208.0679
MPPE1	1	1	48.8714	1	1	1	1	1	1	1	1	1	1	1	1	1	1	28.5793	1	1
MPPED1	1	1	1	1	72.58748	1	1	1	205.5181	1	1	1	1	84.74865	118.3969	1	81.35015	1	98.03391	103.6483
MPPED2	1	137.8205	67.21401	182.128	132.2234	226.1805	1	1	102.1368	217.3185	1	217.8986	141.242	311.698	67.63818	165.1405	1	45.37956	130.5353	1
MRAS	1	37.53453	42.29311	1	43.27431	38.98966	1	1	64.76832	45.83458	1	33.23145	1	1	1	1	1	25.50418	62.99643	103.6483
MRC2	1	53.75292	1	1	1	77.10193	1	98.3595	1	1	1	1	47.99648	1	1	49.15085	1	1	1	1
MRGBP	1	30.41985	1	44.90276	48.1967	31.39109	1	1	1	44.65651	1	41.85838	36.68493	68.69464	1	1	1	60.32267	40.37434	1
MRM2	1	1	1	1	1	22.55763	1	1	1	41.56882	1	33.76174	23.89079	1	1	1	1	23.06345	1	1
MRM3	1	1	1	1	1	1	1	1	1	36.31957	1	1	1	1	1	1	1	21.98072	1	1
MROH1	1	1	1	1	1	1	1	1	88.22437	1	1	1	1	1	1	1	49.07455	1	1	1
MRPL13	1	59.93144	83.36214	53.96243	60.0084	90.74258	1	85.47486	1	72.88539	73.39604	64.75155	64.17128	78.14847	61.02083	83.26056	1	142.2381	1	116.5001
MRPL2	1	1	1	1	1	29.79775	1	35.6786	1	33.96295	1	23.33827	24.3959	1	1	35.32782	1	1	1	103.6483
MRPL23	1	33.29474	35.521	1	1	31.44392	1	1	1	47.15641	1	41.13426	31.0579	1	51.82413	1	1	1	1	1
MRPL34	82.56891	42.49129	55.34251	1	1	40.25051	1	95.52076	1	33.30384	1	32.61547	35.18174	1	1	89.74868	1	115.492	1	103.6483
MRPL44	1	45.98363	31.88574	77.0603	73.35973	42.20806	1	1	1	62.56008	107.2717	51.86446	41.14713	107.3612	84.34739	1	1	43.78531	82.9902	87.58009
MRPL58	1	43.5768	1	1	1	48.19409	1	1	1	32.54515	1	38.21742	40.66962	1	1	49.30067	1	1	1	103.6483
MRPS18A	1	1	84.95775	1	1	23.79897	1	1	1	34.02416	1	24.54986	1	1	1	1	1	1	1	1
MRPS22	1	70.49442	44.53455	1	1	54.76547	1	50.21444	1	35.78662	67.10623	35.54872	37.3835	63.19607	1	61.59092	1	16.07932	1	1
MRPS27	1	1	65.47398	1	1	37.20235	1	1	1	42.3966	1	30.90774	37.10862	1	39.40355	43.38518	1	1	41.32273	1
MRPS28	1	1	63.5873	1	1	38.19976	1	1	1	1	1	1	20.98025	1	1	1	1	17.4204	1	1
MRS2	1	1	1	1	1	38.35852	1	1	1	1	1	1	30.0985	1	1	1	1	1	1	103.6483
MRVI1	1	1	1	1	1	1	1	81.79762	1	1	1	1	1	1	1	1	1	1	1	1
MS4A7	219.5274	1	1	1	1	1	1	1	1	1	1	1	1	1	1	1	1	1	1	1
MSANTD2	1	1	1	1	1	1	1	24.47821	1	38.12266	1	1	1	1	1	1	164.6652	1	1	1
MSH3	1	1	1	1	1	27.39668	1	32.90663	1	40.86515	1	39.57429	26.79214	1	1	1	1	18.96271	68.75259	1
MSI1	1	46.42475	1	48.83818	1	78.8198	1	1	1	83.11394	1	83.97007	84.15209	1	40.11509	73.10001	1	1	1	1
MSL2	1	1	1	1	1	28.77627	1	1	1	1	1	36.87832	28.99097	1	1	1	1	28.13257	75.26677	1
MSRA	36.55964	40.1524	48.18952	82.75265	172.5828	1	151.8666	1	137.5435	1	171.8067	1	45.83316	286.0845	222.0567	1	567.4211	41.8455	1	277.0906
MSRB2	67.50548	52.53196	48.74873	1	44.78368	104.6517	1	84.70517	103.4941	58.51934	1	44.7324	53.92942	1	1	61.71409	1	34.09773	1	103.6483
MSRB3	1	1	1	1	1	1	1	47.0433	1	1	1	1	1	1	1	1	1	115.8306	1	1
MSTN	1	1	1	1	1	1	1	1	1	1	1	1	48.21935	1	1	1	1	1	1	1
MSX1	1	1	1	1	1	1	1	243.8423	1	1	1	1	1	1	1	1	1	75.77028	1	1
MT.ATP8	1	55.7502	69.85608	1	45.38461	1	155.5068	31.74924	61.70958	33.40413	1	29.54942	31.95082	1	81.22732	1	1	51.01418	1	1
MT1E	1	1	1	1	1	215.3699	1	1	1	1	1	1	1	1	1	1	1	109.2922	1	1
MT1M	1	1	1	1	1	43.97742	1	1	1	1	1	1	1	1	1	1	1	1	1	1
MT1X	1	1	1	1	1	109.4049	1	1	1	1	1	1	32.37174	1	1	1	1	69.73817	1	1
MT2A	1	224.2801	85.57715	1	1	650.6111	1	166.5948	1	35.92592	1	226.408	104.9154	1	1	251.4	1	345.409	1	1
MT3	1	583.3501	158.672	1	1	387.0902	1	1	1	1	1	124.0976	1	1	1	578.4903	1	1	1	1
MTA3	1	46.35698	1	1	1	40.97106	1	59.35254	1	1	1	33.15887	30.11385	1	1	1	1	1	1	1
MTAP	1	65.6121	62.11856	1	1	48.817	1	1	1	70.08894	1	47.82135	32.50249	1	1	63.87149	1	1	1	1
MTCL1	1	1	1	1	1	1	1	1	1	1	1	1	1	73.09708	1	1	1	1	1	1
MTERF2	1	45.84877	1	1	1	28.96117	1	30.8182	1	1	1	27.59007	1	1	1	1	1	19.83409	1	1
MTERF3	1	1	1	1	1	24.40301	1	1	1	31.6934	1	1	1	1	1	1	1	1	1	1
MTF1	1	1	1	1	1	1	1	1	1	1	1	26.70048	43.87807	1	1	1	1	1	1	1
MTFR1L	1	53.95101	1	1	39.33205	44.8642	1	55.66696	1	1	1	34.47009	39.44655	1	53.43909	31.33464	1	1	1	206.2967
MTHFD1	1	63.90647	37.13264	1	1	38.75837	1	1	1	103.3401	1	49.9335	38.44464	1	1	51.99194	1	1	1	103.6483
MTHFD2	1	1	1	1	1	1	1	1	1	31.88205	1	48.20447	1	1	1	1	1	1	1	103.6483
MTHFR	35.94481	1	40.31852	1	1	26.04526	1	61.29321	1	1	1	1	25.3037	1	1	1	131.8145	31.34718	1	1
MTMR10	1	1	1	1	1	31.15845	1	1	1	1	1	1	1	1	1	1	1	1	1	1
MTMR12	1	1	1	1	1	1	1	1	1	1	1	1	1	1	1	1	1	27.49118	45.58591	139.0453
MTMR14	71.86848	1	1	1	1	1	1	1	1	1	1	1	1	1	1	1	1	1	1	1
MTMR2	1	1	1	1	1	1	1	29.05986	1	36.15839	1	26.41959	1	1	1	1	1	33.70251	1	1
MTMR3	1	39.90687	1	45.85693	41.8614	33.6553	1	1	1	31.23783	1	30.22599	25.80126	1	1	1	1	15.29917	1	103.6483
MTRNR2L8	1	31.30269	1	1	1	27.8164	1	87.45132	85.28288	1	1	1	36.54582	1	1	1	1	36.35112	1	1
MTSS1L	1	36.40876	80.9478	1	1	43.27778	1	1	1	1	1	1	22.36233	1	1	1	1	1	1	70.02264
MTTP	1	1	1	1	1	1	1	1	1	1	1	1	44.58967	1	1	1	1	1	1	1
MTUS1	119.5779	1	1	1	1	32.90505	1	636.9926	1	38.21939	161.3787	29.54836	35.8206	98.38007	1	1	1	317.1568	1	1
MVB12B	1	1	40.51871	1	1	27.03025	1	1	1	1	1	1	1	1	1	1	1	16.70068	1	1
MVD	1	50.08393	46.90793	1	48.89804	100.8609	1	35.80869	96.26517	45.30056	1	52.02184	51.27426	1	57.99319	107.0861	458.6678	29.97397	1	139.0453
MVK	1	1	1	1	1	30.33274	1	1	1	1	1	1	1	1	1	1	1	1	1	1
MVP	1	1	1	1	1	1	1	1	1	1	1	1	1	1	1	1	1	100.9227	1	1
MX1	1	1	1	1	1	1	1	1	1	1	1	1	1	1	1	1	1	57.59504	1	1
MXD1	1	55.33604	1	47.77894	64.53969	37.9078	1	41.85116	1	52.96488	1	28.51462	42.13813	1	1	1	1	20.9796	65.56309	1
MXD3	1	1	1	1	1	1	1	1	1	1	1	170.6842	1	1	1	1	1	1	1	1
MXRA5	1	1	1	1	1	1	1	132.6458	1	1	1	1	1	1	1	1	1	1	1	1
MXRA8	1	1	1	1	1	50.04806	1	1	1	1	1	1	27.64598	1	1	1	1	1	1	1
MYBL1	1	1	1	1	1	1	1	1	1	37.7658	1	83.01657	1	1	1	1	1	1	1	1
MYBL2	1	1	1	1	1	1	1	1	1	51.59778	1	74.25114	1	1	1	69.77679	1	1	1	1
MYC	1	62.92091	88.50581	1	1	1	1	184.9276	1	1	1	1	46.77934	1	1	1	1	11.0499	77.24761	1
MYCL	1	1	1	1	1	1	1	1	1	51.52294	1	1	1	1	1	1	1	1	1	1
MYCN	1	62.27246	1	75.39836	135.1516	62.43918	1	1	1	44.84799	1	43.48971	81.3054	1	131.2926	86.11756	46.80094	1	106.1569	1

MYCT1	1	1	1	1	1	1	1	1	1	1	1	1	1	1	1	1	1	244.1803	1	1
MYD88	1	1	1	1	1	1	1	1	1	1	1	1	1	1	1	1	1	52.49106	1	1
MYH9	56.98986	1	1	1	1	1	1	364.1097	1	1	1	1	1	1	1	1	1	189.9084	1	1
MYL12A	568.8156	89.67577	124.1988	1	1	88.11196	1	584.6086	1	39.74887	1	58.58087	113.5448	1	1	53.11133	1	522.5488	52.02371	1
MYL9	1	1	1	1	1	1	1	197.3045	1	1	1	1	1	1	1	1	1	1	1	1
MYLIP	65.60097	1	1	1	1	1	1	181.256	1	1	1	1	1	1	1	1	1	21.40477	1	1
MYLK	1	1	1	1	1	1	1	105.2642	1	1	1	1	1	1	1	1	1	22.3865	1	1
MYLK2	1	1	1	1	1	1	1	1	1	1	1	1	1	1	1	1	1	65.64209	1	1
MYNN	1	41.44601	1	44.88268	41.70863	27.60597	1	1	1	42.19796	1	34.74601	38.07121	1	1	1	1	49.33725	1	1
MYO1B	1	1	1	1	1	1	1	887.2497	1	1	1	1	1	97.61385	1	1	1	399.8056	1	1
MYO1C	1	1	1	1	1	1	1	121.3912	1	1	1	1	27.40153	1	1	1	1	22.01333	1	103.6483
MYO1E	80.95686	1	1	1	1	28.86342	1	1	1	1	1	1	36.93545	1	1	1	1	73.41804	1	1
MYO1F	65.74647	1	1	1	1	1	1	1	1	1	1	1	1	1	1	1	1	1	1	1
MYO3A	1	1	1	1	1	1	1	1	1	1	1	1	27.26902	1	1	1	1	1	1	1
MYOF	1	1	1	1	1	1	1	194.525	1	1	1	1	1	1	1	1	1	1	1	1
MYORG	1	1	1	1	1	36.05308	1	1	1	1	1	1	1	1	1	1	1	35.67975	1	1
MYRIP	1	1	1	1	1	1	1	1	1	1	1	1	1	1	1	1	1	71.2713	1	1
MYT1	1	1	174.9324	86.50723	1	1	1	1	1	68.74757	77.25994	31.12932	1	70.6607	1	1	1	1	45.19849	1
MYT1L	1	1	1	279.1093	357.4102	1	354.8732	1	899.7075	1	408.0151	1	1	265.7825	435.7245	1	498.7094	110.7297	493.1527	346.1132
MZT1	1	44.94852	47.08948	1	51.6573	53.45469	1	29.53404	1	63.38508	1	148.4332	45.62188	1	85.92075	38.54904	45.94706	35.74319	132.6273	241.6936
N4BP2L1	1	1	1	1	1	90.31627	1	1	1	1	1	1	34.90142	1	1	1	1	1	1	1
NAA16	1	80.81892	67.40765	46.96509	53.13216	38.00082	1	1	1	76.66597	71.87838	71.73265	53.45373	70.56171	1	79.09929	1	22.17139	1	1
NAA25	1	61.09451	50.05683	1	40.3798	33.14096	1	1	1	40.67013	1	32.32256	41.24356	1	1	1	1	1	1	1
NAA30	1	1	55.98701	1	42.54004	28.4679	1	1	75.85596	32.5297	1	20.15018	1	1	1	1	1	38.26861	1	70.02264
NAA35	1	37.81354	37.42592	65.82182	66.14068	45.17054	1	1	97.48678	36.97313	74.83033	47.06732	41.25509	73.22351	103.0271	1	1	1	1	1
NAB1	1	42.25837	1	59.30957	1	29.05159	1	75.29807	1	59.95584	1	70.8132	28.86777	1	1	1	1	23.7479	1	1
NABP1	46.21032	1	1	1	1	1	1	74.22128	1	1	1	1	1	1	1	1	1	1	1	1
NABP2	1	37.30844	71.29746	1	1	31.14192	1	24.46319	1	74.04015	1	45.87604	31.83614	1	45.86839	51.70628	1	1	1	116.5001
NACAD	1	1	1	1	1	33.60629	1	1	1	1	1	1	1	1	1	1	1	1	1	1
NACC2	1	1	1	1	1	38.66011	1	1	1	1	1	1	38.11399	1	1	1	1	19.98125	1	1
NADK	1	1	33.73794	1	1	1	1	1	1	1	1	1	1	1	1	1	1	1	1	1
NADK2	1	65.64433	1	1	1	146.7512	1	1	1	30.13302	1	54.14848	125.7931	1	1	72.23696	1	1	1	1
NADSYN1	1	1	1	1	1	1	1	1	1	1	1	1	1	1	1	1	1	110.1726	1	1
NAGA	44.38874	1	1	1	1	1	1	33.89108	1	1	1	1	1	1	1	1	1	1	1	1
NAIP	111.25	1	1	50.54604	51.51291	1	1	1	1	33.96971	1	30.912	1	1	68.55346	1	1	1	1	1
NAMPT	101.3797	93.26945	39.11435	1	63.02935	67.69787	1	89.90693	1	42.69645	1	47.82377	152.1832	1	1	195.3474	1	544.3355	1	1
NAP1L3	1	65.00516	112.1714	1	65.26732	42.93848	1	1	284.4829	1	128.7494	1	46.84477	104.4164	82.07418	60.10499	64.84518	1	1	1
NAPB	1	1	1	1	1	1	1	1	62.8527	1	1	1	1	1	1	1	1	1	1	139.0453
NAPEPLD	1	37.42896	54.40095	1	1	117.8633	1	1	1	39.39675	1	68.07924	69.84558	1	1	79.71094	1	1	1	1
NARS2	1	1	1	1	40.08048	29.06842	1	1	1	30.56958	1	25.80994	1	1	1	1	1	1	49.44329	1
NAT8L	1	1	1	1	1	37.5032	1	1	1	1	1	1	1	1	1	1	108.5358	1	1	1
NAV2	1	1	1	66.08696	61.25453	1	1	1	133.3213	1	1	1	1	1	68.64292	1	118.4691	1	1	1
NAV3	1	1	1	1	52.78271	49.48471	1	1	420.9652	1	194.8205	1	27.69763	143.5399	91.62374	1	261.1755	1	1	70.02264
NAXD	1	1	34.3454	1	1	33.14818	1	29.30932	1	30.7682	1	1	24.06896	1	59.71	1	1	1	50.68246	1
NAXE	1	61.94118	207.1888	1	1	80.00122	1	33.26164	1	60.1214	1	52.99135	84.70657	1	1	75.33225	1	26.39595	1	1
NBL1	181.4962	1	1	1	1	1	1	156.6184	196.7794	1	1	1	1	1	1	1	1	58.1275	1	1
NBPF14	1	40.06817	1	1	1	73.64043	1	49.81001	1	30.73169	1	41.41591	35.73696	1	1	1	1	1	1	1
NBPF19	1	32.71681	1	1	1	55.65512	1	20.78595	1	1	1	28.22445	1	1	1	1	1	1	1	1
NCALD	1	49.93357	235.7113	1	54.73146	47.10502	1	31.88119	56.12642	1	1	1	29.62212	1	103.6725	1	70.49152	13.22339	1	172.671
NCAM2	1	67.24615	180.9647	1	1	70.12242	1	1	1	1	1	26.16363	52.48854	1	1	1	1	1	1	1
NCAPD2	1	1	1	1	1	1	1	1	1	20.66897	1	116.254	1	1	1	1	1	1	1	1
NCAPD3	1	1	33.85066	1	1	1	1	1	1	45.93532	1	94.09878	32.63037	1	1	1	204.7942	1	1	1
NCAPG	1	1	1	1	1	1	1	1	1	43.31245	1	219.6902	1	1	1	1	1	1	1	1
NCAPG2	1	1	1	1	1	1	1	1	1	29.04606	1	56.14543	1	1	1	1	1	1	1	1
NCAPH	1	1	1	1	1	1	1	1	1	1	1	109.712	1	1	1	1	1	1	1	1
NCAPH2	1	1	1	1	1	1	1	1	1	35.55452	1	56.5886	1	1	1	1	1	1	1	1
NCBP1	1	1	59.48321	1	1	25.2835	1	1	1	1	1	34.51064	28.10373	1	1	1	1	1	1	1
NCF2	71.75709	1	1	1	1	1	1	1	1	1	1	1	1	1	1	1	1	1	1	1
NCF4	135.48	1	1	1	1	1	1	1	1	1	1	1	1	1	1	1	1	1	1	1
NCKAP1L	96.75802	1	1	1	1	1	1	1	1	1	1	1	1	1	1	1	1	1	1	1
NCKAP5	48.13702	109.1538	74.22246	1	1	92.51191	1	1	106.8761	1	1	30.40508	123.771	1	1	117.2909	51.24693	1	1	70.02264
NCKAP5L	1	1	44.42669	1	1	31.01872	1	95.87137	1	1	1	1	1	1	1	1	158.5524	52.4953	1	1
NCOA2	1	31.60403	26.16972	55.50073	60.59448	48.73898	1	54.07233	70.07403	42.94807	1	56.00411	45.11064	157.6356	1	1	1	27.04422	62.88691	1
NCOA5	1	1	28.91596	1	1	1	1	1	1	36.28153	1	33.78762	26.63486	1	1	1	1	1	1	1
NCOA7	87.13567	1	1	1	1	1	1	49.59348	85.05646	1	1	1	1	1	1	1	1	124.9162	1	70.02264
NCS1	1	40.91802	38.3972	103.9268	137.2678	49.222	254.5432	1	131.4672	75.46533	87.02797	65.65092	45.72792	148.1374	167.1991	46.45847	213.2972	1	110.5043	275.3193
NDC1	1	1	1	1	1	1	1	1	1	1	1	30.55023	1	1	1	1	1	1	1	1
NDC80	1	1	1	1	1	1	1	1	1	1	1	227.7514	1	1	1	1	1	1	1	1
NDE1	1	1	1	1	1	26.52354	1	1	1	1	1	80.15783	36.18745	1	1	1	1	25.20078	1	1
NDFIP2	1	37.66455	75.67657	1	1	33.51066	1	31.24514	1	1	1	1	1	1	1	1	1	23.68502	1	1
NDP	1	40.36702	1	1	1	43.82948	1	1	1	1	1	1	1	1	1	1	1	1	1	1
NDRG1	1	1	1	1	60.3767	1	1	1	183.5536	1	1	1	1	1	67.14578	1	70.75404	14.82823	65.93593	1
NDRG2	1	225.8175	88.14116	1	43.80176	263.6254	1	90.77795	1	40.74694	59.60047	54.92979	118.1119	1	36.96835	187.3856	1	1	1	1
NDRG3	1	1	44.39802	1	1	26.69451	1	1	96.63218	1	1	1	1	1	1	1	1	1	1	1
NDST3	1	1	1	1	1	1	1	1	1	1	1	1	1	1	42.07754	1	120.8287	1	1	1
NDUFA4L2	1	1	1	1	1	1	1	2031.257	1	1	1	1	1	1	1	1	1	1	1	70.02264
NDUFAF2	1	44.45107	96.00787	54.9147	124.195	49.66606	191.7801	31.85193	125.3064	43.1394	61.07572	40.68034	39.28602	92.78306	184.6749	74.09668	92.60533	1	107.0881	172.671
NDUFAF4	1	48.59058	59.90147	52.30859	54.69741	37.06313	1	47.28352	1	46.10357	1	35.10112	38.47773	1	41.46753	1	1	40.54812	91.3603	87.58009
NDUFAF5	1	1	1	1	38.39385	1	1	1	1	1	1	26.24879	1	1	51.66417	1	1	1	1	1
NDUFAF6	1	1	66.10793	1	1	1	1	1	1	47.78278	1	35.32191	26.97177	1	1	1	1	1	1	1
NDUFAF7	1	1	42.32301	1	1	1	1	1	1	1	1	1	25.44965	1	1	1	1	1	1	1
NEAT1	1521.725	178.3966	65.36059	1	1	457.8308	1	1198.787	1	168.0679	1	114.7253	442.4091	1	1	349.9381	1	1602.993	1	1
NEBL	1	1	1	1	1	24.47637	1	1	1	1	1	1	61.7556	1	1	1	1	50.26368	1	1
NECAB3	1	1	1	1	1	44.58295	1	1	1	1	1	1	1	1	1	1	1	1	1	1
NECTIN1	1	1	1	1	1	1	1	1	1	1	70.15951	1	1	1	1	1	1	1	1	1
NECTIN2	1	1	1	1	1	31.78522	1	134.6626	1	1	1	1	43.72485	1	1	1	1	255.0364	1	103.6483
NECTIN3	1	1	49.0938	1	1	1	1	23.69188	66.99782	1	1	1	1	87.72509	1	1	1	1	1	1
NEDD1	1	37.56715	1	1	1	43.93465	1	1	1	42.89631	1	77.71132	36.97028	1	1	65.19895	1	1	1	1
NEDD9	1	89.87836	1	54.18437	74.77333	134.4715	1	28.68232	1	78.45073	1	70.79653	208.4808	1	1	131.9655	1	254.8347	136.8365	1
NEFL	1	1	1	1	1	1	1	1	1	1	1	1	1	1	1	1	278.6383	1	1	1
NEFM	1	1	1	1	118.0107	1	164.1648	1	91.14156	1	1	1	1	1	358.1592	1	686.3863	1	1	1
NEGR1	1	43.09248	38.82059	1	1	1	1	1	100.0236	1	1	29.13834	1	1	1	1	1	1	1	1
NEIL1	1	1	1	1	1	1	1	1	55.08141	1	1	1	1	1	1	1	76.12877	38.67254	1	70.02264
NEIL2	1	34.72175	35.68437	1	1	38.09042	1	1	1	1	1	1	1	1	1	47.11478	1	1	1	1
NEIL3	1	1	1	1	1	1	1	1	1	1	1	40.23449	1	1	1	1	1	1	1	1
NEK11	1	1	1	1	1	42.1035	1	1	1	1	1	1	86.42032	1	1	1	1	1	1	103.6483
NEK2	1	1	1	1	1	1	1	1	1	1	1	113.8662	1	1	1	1	1	1	1	1
NEK5	1	1	1	1	1	1	1	1	1	1	1	1	85.96665	1	1	1	1	1	1	1
NEK6	189.6563	54.91048	1	1	1	58.21504	1	1	1	34.7453	1	26.06947	65.90997	1	1	1	1	1	1	1
NELFA	1	45.79772	43.75515	55.55414	52.54426	53.20575	1	1	1	55.79809	67.18045	50.00065	41.93399	58.51536	1	1	1	1	68.80542	172.671
NELFB	1	62.54546	46.9603	1	49.36071	41.40158	1	60.87749	1	62.63734	1	48.07196	35.86517	1	1	1	1	48.63085	92.53809	1

NELFCD	1	111.147	52.63465	101.3926	99.39189	85.46116	1	66.36026	75.88249	112.1768	127.8776	106.2561	92.39066	122.0128	92.24991	105.8918	154.6304	42.27729	84.70182	308.945
NELL2	1	1	1	97.15611	183.9856	1	177.2476	1	672.6316	56.96995	1	1	1	79.50954	385.2172	1	736.2824	1	180.4628	344.3419
NEMP1	1	1	1	1	1	1	1	1	1	34.52485	1	50.34277	1	1	1	1	1	1	1	1
NEO1	1	50.29227	37.64365	49.2927	56.23736	53.94936	1	1	113.0349	81.52388	1	35.71963	65.78226	1	62.67529	1	242.8442	30.93114	36.04484	1
NEPRO	1	66.87736	49.22923	1	1	42.0989	1	39.30169	1	53.49694	65.30465	54.75463	38.12693	67.84494	1	1	1	96.37692	1	1
NES	1	258.9334	147.6499	53.91586	1	110.0079	1	36.79614	1	129.4261	76.71072	117.1888	139.8907	85.53622	1	133.5919	1	87.64308	1	1
NETO1	1	1	133.2737	1	1	1	1	1	1	1	1	1	1	1	1	1	1	1	1	1
NEU1	149.3203	1	1	1	1	29.3734	1	99.2406	1	1	1	1	1	1	1	1	1	84.01122	1	1
NEU4	1	1	78.24035	1	1	1	1	1	1	1	1	1	1	1	1	1	1	1	1	1
NEURL1B	1	32.38684	1	1	1	1	1	1	1	1	1	34.16401	1	1	1	1	1	1	1	1
NEUROD1	1	1	1	127.9511	93.66227	1	1	1	1	74.73253	1	69.92076	1	1	77.02491	1	1	1	57.74978	1
NEUROD2	1	1	1	664.4097	962.6555	1	946.2902	1	524.9948	115.4802	1	63.62363	1	1	975.752	1	569.1284	24.84917	868.6196	687.6839
NEUROD4	1	1	1	69.70583	1	1	1	1	1	202.3207	1	57.26009	1	1	1	1	1	1	1	1
NEUROD6	1	1	1	333.5782	531.7317	1	687.8332	1	588.4596	1	1	1	1	1	805.7403	1	694.5645	1	561.2001	823.9579
NEUROG1	1	1	1	1	1	1	1	1	1	238.1941	1	49.7247	1	1	1	1	1	1	1	1
NEUROG2	1	1	1	60.52317	72.6551	1	1	1	1	328.7601	1	95.801	1	1	1	1	1	1	52.73124	206.2967
NFASC	1	1	155.8241	126.6479	157.3393	1	1	1	173.0083	1	1	1	1	1	121.7044	1	184.2955	1	84.97257	1
NFATC2	39.84905	1	1	1	1	1	1	1	1	1	1	1	90.83312	1	1	1	1	17.63065	1	1
NFATC3	1	1	1	1	1	1	1	35.40872	1	70.24058	1	74.25777	38.43395	1	1	1	1	58.60815	1	70.02264
NFIL3	1	77.65579	1	61.8226	105.5826	83.24358	1	208.2372	1	48.83016	133.1902	50.30746	143.2701	138.303	78.16716	51.37957	1	79.82752	1	103.6483
NFIX	1	310.0976	110.3116	676.2247	642.6836	272.6375	894.7251	1	555.7401	502.4881	217.3245	359.5211	400.1589	1	606.3329	370.0417	376.4038	122.703	507.4024	208.0679
NFKB1	67.34362	1	1	1	1	1	1	96.23723	1	1	1	1	27.10902	1	1	1	1	51.49273	1	1
NFKB2	1	1	1	1	1	1	1	69.73439	1	1	1	1	31.63289	1	1	1	1	1	1	1
NFKBIA	2286.024	80.3896	74.07235	70.68702	76.21202	46.53606	1	755.708	1	69.8893	78.67553	64.79851	125.4432	58.01165	1	56.74539	1	275.1728	1	116.5001
NFKBID	128.2415	1	1	1	1	1	1	1	1	1	1	1	1	1	1	1	1	1	1	1
NFKBIE	84.45837	1	1	1	1	1	1	1	1	1	1	1	1	1	1	1	1	1	1	1
NFKBIL1	1	30.88735	1	42.96139	39.92986	33.77044	1	28.55882	1	44.04265	1	40.62098	40.32229	1	1	1	1	20.43206	87.85045	1
NFKBIZ	215.3461	68.62649	1	1	1	39.91357	1	492.4093	1	1	1	1	84.22581	1	1	1	1	63.91432	1	1
NFYA	1	1	1	1	1	1	1	1	1	1	1	1	1	1	1	1	73.37273	1	1	70.02264
NGEF	1	1	1	1	1	1	1	1	167.8316	1	1	1	1	1	1	1	1	1	1	1
NGLY1	1	37.47247	78.13408	1	1	58.35203	1	1	1	1	1	51.90753	38.47265	1	1	71.84786	1	36.00916	1	1
NGRN	1	1	1	1	1	29.83522	1	1	1	41.48042	1	30.88029	25.22373	1	1	1	74.48068	1	1	1
NHLH1	1	1	1	198.1162	52.10391	1	1	1	1	100.3759	1	1	1	1	1	1	1	1	50.66127	1
NHSL1	1	65.0555	52.79752	151.4679	199.1537	44.94177	310.237	40.99102	1	55.79282	96.28618	41.45717	73.76388	129.1871	316.2371	1	390.3432	80.2299	150.1064	254.5454
NID1	1	1	1	1	1	66.30356	1	854.6234	1	1	1	1	43.69199	1	1	1	1	141.434	1	1
NID2	1	1	1	1	1	1	1	108.4878	1	1	1	1	1	1	1	1	1	107.5723	1	1
NIF3L1	1	1	1	1	1	1	1	1	1	1	1	32.93073	1	1	1	1	1	1	1	87.58009
NINJ1	488.0149	49.75244	40.64742	1	1	35.84186	1	47.32654	1	1	1	29.87044	46.49435	1	1	1	1	24.01246	1	1
NINL	1	1	1	1	1	29.97493	1	1	1	44.55706	1	23.22837	25.41106	1	1	1	105.6459	1	1	70.02264
NIPA1	1	1	1	1	1	1	1	1	1	1	1	1	1	1	1	1	97.44436	1	1	1
NIPSNAP3A	1	1	1	1	42.14837	45.96359	1	1	1	1	1	22.76087	28.84184	1	39.39068	1	1	1	1	1
NIT1	1	1	1	1	1	41.71724	1	1	1	1	1	34.30735	27.32452	1	1	1	1	1	1	1
NIT2	1	48.23546	47.39358	1	1	47.68612	1	48.02144	1	50.14292	1	37.05493	46.24183	1	1	128.8831	1	18.43998	1	103.6483
NKAIN1	1	1	1	66.27229	61.73572	1	1	1	90.39508	36.59727	1	1	1	1	60.46659	1	1	1	48.13721	103.6483
NKAIN2	1	1	1	1	61.39528	1	1	1	1	1	72.54942	1	1	1	159.8254	1	310.5733	1	1	208.0679
NKAIN4	1	340.8806	435.4673	1	1	63.94378	1	1	1	1	1	1	1	1	1	113.5347	1	1	1	1
NKD1	1	46.32194	30.00771	1	1	1	1	48.04499	1	1	1	1	1	1	1	1	1	65.60248	1	1
NKD2	1	1	1	1	1	1	1	29.74168	1	1	1	1	1	1	1	1	1	71.41805	1	1
NKIRAS1	1	1	1	1	1	25.07557	1	1	1	1	1	27.50542	28.18923	51.70307	1	1	1	1	1	1
NKRF	1	1	73.0099	1	52.51583	1	1	51.12351	1	1	60.1036	1	25.35101	67.39961	1	1	1	27.85392	1	1
NKX2.2	1	1	261.3685	1	1	1	1	1	1	1	1	1	1	1	1	1	1	1	1	1
NLGN1	1	98.61884	156.4559	69.03655	60.1341	81.36011	1	1	299.3496	85.03783	1	95.20507	72.2498	1	1	52.65842	1	1	1	1
NLGN2	1	35.77224	127.6692	1	1	40.47744	1	25.89338	1	1	1	28.84906	30.28988	1	46.46543	1	1	1	1	1
NLGN3	1	54.51549	97.96774	1	1	38.45808	1	1	1	1	1	1	28.97405	1	1	1	1	1	1	103.6483
NLGN4X	1	180.797	153.5268	53.88735	54.29174	77.10088	1	1	1	96.36863	1	88.66609	53.5371	1	1	1	81.5297	1	1	308.945
NLK	1	1	1	1	1	1	1	1	1	1	1	1	1	1	1	1	1	26.82447	92.40528	70.02264
NLN	1	1	29.74758	55.44246	78.97156	33.37184	1	1	72.09797	1	110.0596	1	28.81509	87.37369	109.7524	1	222.8562	1	135.5199	139.0453
NMB	1	1	1	1	1	1	1	1	1	1	1	1	36.63153	1	1	1	1	1	1	1
NMD3	1	41.18021	57.42305	1	1	54.5987	1	36.83887	1	37.15775	1	41.55174	51.64249	1	1	77.06786	1	41.89758	1	1
NME5	1	1	1	1	1	1	1	1	1	1	1	1	64.80803	1	1	1	1	1	1	1
NMNAT2	1	1	1	1	47.74477	1	1	1	129.7212	1	78.66804	1	1	160.3102	68.93964	1	1	1	61.31178	206.2967
NMRAL1	1	34.9886	1	1	1	50.13305	1	1	1	52.28242	1	47.49822	39.11397	1	1	61.15757	1	1	1	206.2967
NMRK1	63.95328	1	1	1	1	1	1	1	1	1	1	1	1	1	1	1	1	25.807	1	1
NMT2	1	1	1	51.91564	49.52923	29.43915	1	25.91827	133.9895	41.45287	1	49.74002	34.83568	90.29701	1	1	1	18.24008	107.2656	1
NMU	1	1	1	1	1	1	1	1	1	1	1	39.20036	1	1	1	1	1	1	1	1
NOA1	1	1	1	44.93965	39.90629	22.71319	1	1	1	29.48938	1	24.69125	26.13439	1	91.77849	1	1	23.81581	1	1
NOB1	1	43.96915	43.83968	1	1	27.89478	1	1	1	34.91939	1	26.06798	45.07788	1	1	85.41726	1	23.33203	1	1
NODAL	1	1	1	1	1	1	1	314.5202	1	1	1	1	1	1	1	1	1	1	1	1
NOL10	1	51.32009	68.44403	1	1	31.73364	1	42.05029	1	41.24947	1	37.1005	26.37559	1	1	1	83.18734	25.85231	1	1
NOL4	1	1	70.39901	77.76369	112.0408	1	1	1	1	53.45479	103.0857	33.29268	25.58109	112.0285	116.5803	1	126.5315	1	77.11495	1
NOL9	1	36.87589	61.90143	1	1	29.39427	1	1	1	34.6469	1	35.00144	28.77054	1	1	51.21202	1	1	1	1
NOP14	1	1	1	1	1	1	1	1	1	33.30605	1	1	29.16294	1	1	1	1	1	1	139.0453
NOP16	1	1	1	1	1	1	1	1	1	25.60687	1	1	1	1	1	1	1	1	1	70.02264
NOS1AP	1	30.95597	1	72.16322	82.55454	50.10096	1	1	1	37.86071	97.63264	38.179	44.15236	69.30122	1	1	1	15.23972	44.74951	1
NOS3	1	1	1	1	1	1	1	1	1	1	1	1	1	1	1	1	1	46.21814	1	1
NOSTRIN	1	1	1	1	1	1	1	108.2513	1	1	1	1	1	1	1	1	1	130.3918	1	1
NOTCH2	1	103.0214	1	1	1	181.8216	1	118.0878	1	42.80569	1	86.36246	66.34674	1	1	38.26105	1	1	1	1
NOTCH2NL	1	1	1	1	1	35.51011	1	1	1	1	1	1	1	1	1	1	1	1	1	1
NOTCH3	1	36.41457	1	1	1	70.42955	1	542.1573	1	1	1	24.90501	46.49508	1	1	32.13209	1	1	1	1
NOTCH4	1	1	1	1	1	1	1	1	1	1	1	1	1	1	1	1	1	189.2006	1	1
NPAS1	1	1	71.31946	1	1	1	1	1	1	1	298.7626	1	1	221.1969	1	1	1	1	1	1
NPAS3	1	324.1823	238.4742	1	1	234.5071	1	1	1	41.13922	254.8601	77.09827	220.356	250.5293	1	198.4417	1	1	1	1
NPAS4	1	1	1	1	65.30884	1	1	1	1	1	1	1	1	1	1	1	1	1	1	1
NPAT	1	33.07387	1	41.9799	48.85724	26.21562	1	1	1	56.71989	65.45341	48.36898	42.61683	1	1	1	1	1	1	1
NPC2	786.2659	1	1	1	1	89.1774	1	32.76902	1	24.78837	1	32.90663	33.85459	1	1	70.97125	1	93.73031	1	1
NPHP1	1	1	1	1	1	1	1	1	1	1	1	1	58.91976	1	1	1	1	1	1	1
NPIPB4	1	1	1	1	1	1	1	1	1	1	1	1	1	1	1	1	1	63.3076	1	1
NPIPB5	1	32.65062	49.91002	1	1	1	1	22.92555	1	1	1	1	1	1	1	1	146.0378	287.0407	1	70.02264
NPL	368.3213	1	1	1	1	1	1	1	1	1	1	1	1	1	1	1	1	1	1	1
NPM2	1	1	1	1	1	1	1	1	72.40211	1	1	1	1	1	1	1	1	1	1	1
NPR3	1	1	1	1	1	1	1	1	145.061	1	1	1	1	1	1	1	1	1	1	1
NPRL2	1	1	32.54604	1	1	1	1	1	1	1	1	1	1	1	1	1	1	1	1	1
NPTX1	1	1	1	1	1	1	1	1	87.15711	1	1	1	1	1	1	1	1	1	1	1
NPTX2	1	1	1	75.37619	1	1	1	1	1	1	1	1	1	1	1	1	1	1	1	1
NPY	1	273.129	1	62.631	1	63.31002	1	1	1	309.2194	1	192.9496	1	1	1	259.6373	1	1	1	1
NQO1	1	1	1	1	1	1	1	1	1	1	1	1	1	1	1	1	1	113.2662	1	1
NR0B1	1	1	31.79834	1	1	1	1	1	1	1	1	1	1	1	1	1	1	1	1	1
NR2C2	1	1	1	48.18256	56.42837	31.95125	1	39.17995	1	50.21427	1	41.20938	30.73755	1	1	1	118.5013	16.25236	1	1
NR2E1	1	41.55343	1	1	1	74.09336	1	1	1	32.99792	1	52.35254	51.2859	1	1	1	1	1	1	1
NR2F1	1	228.3846	86.17854	429.2585	306.6998	371.3886	296.4364	371.7483	196.3008	383.8092	888.8606	410.6074	217.1907	155.0536	313.7873	330.6037	178.0325	1	331.5186	206.2967
NR2F1.AS1	1	32.73017	1	69.40525	49.43134	36.21384	1	31.56644	1	41.36838	150.1188	46.193	27.24193	1	1	1	1	1	79.3947	1

NR2F2	1	1	1	1	1	1	1	913.8248	1	1	898.5731	1	1	1	1	1	1	54.1196	1	1
NR3C1	79.08834	1	47.56825	1	1	1	1	193.1117	1	1	1	1	1	1	1	1	1	190.9331	1	1
NR4A1	248.5637	94.56571	27.37394	1	93.6161	121.6224	1	351.6246	1	1	1	48.97858	350.3104	1	1	1	1	348.2948	1	1
NR4A2	152.1983	1	1	1	112.0417	1	1	259.281	130.3361	1	1	28.73118	34.6477	1	1	1	1	138.5943	1	70.02264
NR4A3	1	1	1	1	1	1	1	121.4232	1	1	1	1	48.74101	1	1	1	1	1	1	1
NRARP	1	38.19552	1	1	1	80.58056	1	80.29135	1	1	1	32.52189	82.90366	1	1	1	1	49.73522	1	1
NRBP2	1	1	52.07647	49.26217	51.62427	90.31186	1	1	153.4119	35.08006	1	29.29851	48.78683	1	1	110.1726	157.6506	1	1	70.02264
NRCAM	1	85.36917	47.27995	1	1	58.12779	1	1	361.9116	48.19758	1	50.78157	1	105.1998	1	1	58.08036	1	1	103.6483
NRG1	1	1	1	76.81207	42.85822	393.5327	1	1	1	90.88451	1	154.6015	169.1131	1	1	206.1582	1	1	77.51812	1
NRIP3	1	1	109.1061	1	1	1	1	1	1	1	113.1589	1	1	1	1	1	1	1	1	1
NRM	1	1	1	1	1	1	1	1	1	1	1	34.11008	1	1	1	1	1	1	1	1
NRN1	1	1	1	404.1829	192.8121	1	1	1	1	89.89087	1	70.67225	1	1	1	1	60.00075	106.1285	102.0995	1
NRP1	1	1	1	234.5873	325.7528	1	243.0067	1	1	1	1	1	1	1	137.2216	1	70.38595	149.7128	353.4382	103.6483
NRP2	95.33457	43.82489	1	1	1	1	1	54.47567	1	1	1	1	1	1	1	1	66.13048	125.7241	1	1
NRSN1	1	1	1	1	1	1	1	1	108.6586	1	1	1	1	1	1	1	1	1	1	1
NRXN2	1	81.66402	122.5397	60.8278	82.83947	56.40862	1	1	356.7276	1	142.1455	31.22025	31.19469	167.0826	122.4779	1	241.1095	1	25.64395	1
NRXN3	1	1	1	1	1	1	1	1	1	1	335.0338	1	1	385.9983	1	1	1	1	1	1
NSDHL	1	1	1	1	1	27.90084	1	1	1	1	1	1	1	1	1	1	1	1	1	1
NSF	1	1	45.31176	1	1	24.82319	1	33.28619	137.394	26.43859	1	26.99376	1	1	1	1	1	46.00386	1	1
NSG1	1	1	1	173.936	345.5362	1	319.0037	1	355.101	1	354.0232	1	1	354.9757	447.1528	1	284.7609	1	267.4058	446.9903
NSG2	1	1	90.49935	171.0108	188.9176	1	1	1	879.415	53.40024	399.0319	1	1	482.0927	259.5219	1	438.0556	1	326.6341	379.4568
NSMCE4A	1	43.20323	41.1615	1	42.17993	53.29598	1	1	1	84.37279	1	86.36	42.88807	1	53.58898	88.00179	1	43.18582	148.7192	70.02264
NSMF	1	43.51481	1	1	69.04653	44.1183	1	1	124.745	1	1	1	49.99914	1	1	1	68.93133	1	47.2634	1
NSUN5	1	1	1	1	1	1	1	1	1	29.28084	1	1	1	1	1	1	1	1	1	1
NSUN6	1	39.62835	48.77133	59.91222	59.79656	30.73015	1	1	1	42.85169	61.66053	37.47745	25.31168	1	45.05179	77.29782	126.7763	38.15213	57.29025	70.02264
NT5E	1	1	66.21373	1	1	1	1	1	1	1	1	1	1	1	1	1	1	1	1	1
NT5M	1	1	1	1	1	1	1	1	1	38.44065	1	1	1	1	1	1	1	1	1	87.58009
NTF3	1	1	1	1	1	1	1	1	1	1	1	1	1	1	1	1	70.84331	1	1	208.0679
NTHL1	1	47.653	1	1	1	35.74379	1	27.44375	1	32.47361	1	25.99255	29.65722	1	1	28.24407	51.46861	58.3247	104.0645	70.02264
NTM	1	107.4585	366.5485	369.7305	348.0694	78.37914	346.0576	629.569	998.2538	1	97.78008	1	57.06084	1	279.5883	61.74488	350.3563	108.4359	277.2786	174.1602
NTN1	1	1	52.79551	1	1	1	1	1	1	1	1	1	1	1	1	1	1	1	1	1
NTN4	1	1	1	1	1	28.35651	1	1	1	1	94.22135	1	1	120.2725	1	1	1	1	78.64451	1
NTNG1	1	1	83.14922	1	1	28.30772	1	1	1	1	1	1	1	1	1	1	1	1	1	1
NTRK2	1	118.2715	63.48013	1	1	1	1	1	255.9222	1	1	1	150.271	1	1	1	81.73785	1	1	1
NTRK3	1	99.59384	213.9541	76.03873	136.9316	96.65062	1	1	339.7603	1	1	1	32.03974	1	160.9003	1	131.2519	1	167.9182	1
NUAK1	1	1	1	52.8361	53.55038	28.42112	1	45.56065	426.385	1	1	1	38.48368	55.72008	60.19878	1	207.7134	28.22148	59.97135	1
NUB1	1	42.82288	50.59705	49.302	46.82136	41.62957	1	25.88642	1	42.036	57.84831	33.6204	40.08547	1	1	1	1	41.15878	1	1
NUDCD1	1	37.79599	1	1	44.27131	1	1	38.10075	117.441	48.60718	1	34.94732	23.52446	1	70.27557	1	1	42.52842	1	1
NUDCD3	1	1	1	49.3446	46.23387	32.41243	1	1	1	1	1	25.39187	29.91113	1	45.88752	1	48.80569	15.15809	1	70.02264
NUDT10	1	1	1	1	1	1	1	1	1	1	1	28.61864	1	1	1	1	1	1	1	1
NUDT11	1	1	62.21173	55.2693	71.65581	1	1	1	1	1	90.58064	37.80028	29.5305	73.40247	83.10954	1	1	1	50.39449	70.02264
NUDT12	1	1	1	1	1	33.75076	1	1	1	1	1	1	1	1	1	1	1	1	1	1
NUDT14	128.4051	1	1	1	56.47282	1	1	1	1	1	1	1	1	1	102.2972	1	1	115.551	1	103.6483
NUDT15	1	1	1	1	1	1	1	23.26057	1	40.78805	1	44.36826	1	1	1	1	1	37.4517	1	1
NUDT19	1	1	1	1	1	1	1	1	1	1	1	1	1	1	1	1	1	102.0089	1	1
NUDT7	1	1	1	1	1	28.70172	1	1	1	1	1	1	1	1	1	1	1	1	1	1
NUF2	1	1	1	1	1	1	1	1	1	31.76527	1	328.9269	1	1	1	1	1	1	1	1
NUMBL	1	1	1	60.28172	56.94883	29.90607	1	1	1	37.87885	1	37.12467	49.05131	1	1	1	85.24881	1	129.2517	103.6483
NUP155	1	1	53.20385	1	1	1	1	1	1	39.72111	1	40.23081	27.20248	1	1	1	1	1	1	70.02264
NUP188	1	1	1	1	1	1	1	1	1	36.80904	1	33.26569	1	1	1	1	1	13.41263	1	1
NUP205	1	1	1	1	1	1	1	1	1	40.1122	1	31.44341	1	1	1	1	1	1	1	1
NUP214	74.97618	50.08297	1	1	1	29.00249	1	1	1	40.95089	1	41.68425	27.7472	1	1	90.08388	1	30.43815	1	1
NUP35	1	33.56673	65.53518	1	1	1	1	1	1	30.64434	1	42.50279	1	1	1	1	1	1	1	1
NUP37	1	1	1	1	1	1	1	1	1	28.87378	1	45.7257	27.33515	1	1	1	1	1	1	1
NUP50	1	49.21151	50.45137	1	47.61861	49.54665	1	40.5192	1	44.1405	85.40762	95.75703	38.54756	69.44792	85.17114	1	1	48.12253	1	1
NUP85	1	1	1	1	1	1	1	1	1	31.26542	1	25.96586	1	1	1	1	1	1	1	1
NUP88	1	1	43.38299	1	1	1	1	1	1	42.14781	1	56.96815	33.08715	1	1	1	1	1	1	1
NUP93	1	37.95453	1	69.42586	66.49598	74.92523	1	24.21805	1	55.20629	1	65.86742	38.17751	1	76.75156	65.01938	71.21626	25.53056	34.19618	103.6483
NUPL2	49.59416	61.61923	108.5931	49.61985	55.99102	38.9516	1	30.25996	1	39.70199	1	45.93425	55.36131	60.21197	53.77023	1	1	42.37415	68.42779	70.02264
NUPR1	185.8123	1	1	1	1	1	1	98.07769	1	1	1	1	1	1	1	1	1	1	1	1
NUSAP1	1	36.36321	1	1	1	28.96402	1	1	1	87.28433	1	948.7292	142.5722	1	1	86.68357	1	1	1	1
NXN	1	1	1	64.5849	57.22342	40.58288	1	64.6544	1	43.11	74.04134	35.0674	1	1	1	1	1	92.6864	61.39235	1
NXPE3	1	1	65.431	1	1	1	1	1	1	1	1	1	1	1	51.46876	1	1	1	1	1
NXPH1	1	61.93795	186.9615	1	1	1	1	1	1	1	1	1	1	281.0455	1	1	1	1	1	1
NXPH3	1	1	1	1	1	1	1	1	296.5137	1	1	1	1	1	1	1	1	1	1	1
NXT1	64.84808	71.88832	40.46466	59.4552	86.23983	41.04027	1	70.16777	1	63.8384	112.5454	60.04555	66.12792	93.51022	1	41.76975	1	114.9865	1	116.5001
NXT2	1	45.8427	1	1	1	1	1	1	1	1	1	1	1	1	1	1	1	1	1	1
NYAP1	1	1	1	1	1	1	1	1	1	1	1	1	1	1	1	1	131.6899	1	1	1
NYAP2	1	1	1	1	1	1	1	1	164.4339	1	1	1	1	91.50537	1	1	1	1	1	70.02264
OAF	1	1	1	1	1	38.77181	1	36.76342	1	1	1	1	1	1	1	1	1	1	1	1
OAT	81.50405	124.7576	129.4965	49.98342	49.36395	384.9731	1	96.79338	1	92.02777	1	98.31746	240.6142	1	1	181.3005	1	40.43738	1	1
OBSL1	1	44.19668	57.37948	1	1	66.71697	1	1	1	1	1	24.62263	48.70993	1	1	60.16627	1	1	1	1
OCIAD2	1	37.49257	71.42105	251.0438	314.9907	46.81991	450.9014	1	200.6293	64.82654	192.6131	55.09384	55.83531	226.8562	370.7963	60.48497	109.3083	122.0946	407.0149	411.5933
OCLN	1	1	1	1	1	1	1	1	1	1	1	1	1	1	1	1	1	139.9562	1	70.02264
ODF2	1	1	1	1	1	1	1	1	1	1	1	43.9239	38.88802	1	1	1	1	20.77969	1	1
OFD1	71.45798	63.46865	77.05087	91.35625	77.18952	58.75652	1	32.1462	60.53989	84.61	1	52.93081	70.87064	80.58518	63.3636	1	74.90723	45.26771	94.40079	172.671
OGFRL1	136.967	66.90955	1	1	1	138.4965	1	1	1	1	1	36.28902	84.45873	1	1	103.2938	1	1	1	1
OGG1	1	1	1	43.03794	1	41.32921	1	23.4665	1	38.88146	63.86555	35.68389	1	91.15822	51.7054	59.82047	111.2134	1	1	172.671
OIP5	1	1	1	1	1	1	1	1	1	1	1	52.2265	1	1	1	1	1	1	1	1
OLFM1	1	59.17063	93.73498	1	59.71246	1	1	1	436.5964	80.26199	131.466	99.58976	1	135.2138	150.8977	31.2093	67.78039	1	149.4792	206.2967
OLFM3	1	1	1	1	1	1	1	1	86.56013	1	1	1	1	1	1	1	1	1	1	1
OLFML1	1	1	1	1	1	1	1	69.98738	1	1	1	1	1	1	1	1	1	1	1	1
OLFML2A	1	1	1	1	1	1	1	87.95501	1	1	1	1	1	1	1	1	1	1	1	1
OLFML3	353.2714	1	1	1	1	1	1	509.9335	1	1	1	1	1	1	1	1	1	1	1	1
OLIG1	1	977.1527	2294.864	1	1	1	1	1	1	1	1	1	1	1	1	1	1	1	1	1
OLIG2	1	546.062	1035.393	1	1	1	1	1	1	1	1	1	1	1	1	1	1	1	1	1
OLR1	459.4657	1	1	1	1	1	1	1	1	1	1	1	1	1	1	1	1	1	1	1
OMG	1	1	230.3774	1	1	1	1	1	1	1	1	1	1	1	1	1	1	1	1	1
OPA3	1	1	1	1	1	1	1	63.22252	1	1	1	1	1	1	1	1	1	31.85942	1	1
OPCML	1	1	96.55832	1	1	1	1	1	133.5582	1	1	1	1	1	1	1	1	1	1	1
OPHN1	1	1	1	46.67948	1	76.18433	1	1	1	1	1	1	29.24248	1	1	1	1	1	1	103.6483
OPTN	1	1	1	62.45683	96.49747	59.34869	173.6307	116.4226	173.0675	1	134.703	27.84979	40.14992	106.3776	107.3402	1	116.362	25.79173	140.2456	185.5228
ORAI1	1	1	1	1	1	1	1	30.55039	1	1	1	1	1	1	1	1	1	57.79141	1	1
ORAI2	1	75.45984	56.16428	1	1	64.64563	1	42.25338	143.3083	1	1	36.23746	81.29409	1	1	52.21167	120.6369	24.26736	1	1
ORAOV1	1	1	1	1	1	1	1	1	1	28.16557	1	1	1	1	1	1	1	1	1	70.02264
ORC2	1	1	1	1	1	1	1	1	1	35.87484	1	21.99038	1	1	1	1	1	14.49099	1	1
ORC3	1	33.76971	1	1	1	40.14229	1	1	1	52.16853	1	41.86775	37.87899	1	1	1	1	53.12318	77.38788	70.02264
ORC6	1	33.82165	50.73929	1	1	38.68772	1	1	1	181.9078	1	216.8079	57.7378	1	1	86.39296	1	1	1	1
OSBP	1	60.04953	76.32305	58.75293	59.03708	38.93449	1	60.76853	1	45.35744	64.63208	57.46618	47.70151	1	1	1	1	50.45425	1	1
OSBPL10	1	1	1	1	1	1	1	1	176.2748	1	1	1	1	1	1	1	1	22.66616	1	1
OSBPL11	1	1	1	1	1	1	1	1	1	1	1	30.35985	27.81662	1	1	1	1	1	42.55163	103.6483

OSBPL2	1	1	1	1	1	48.25459	1	1	1	44.70515	1	20.21311	34.79877	1	1	1	1	34.67607	24.84846	103.6483
OSBPL6	1	43.2764	35.86765	1	1	39.29744	1	1	101.0234	32.04886	1	32.63308	32.57944	1	65.14926	1	1	1	1	1
OSCP1	1	1	1	1	1	1	1	1	1	1	1	1	33.88459	1	1	1	1	1	1	1
OSER1.AS1	1	1	1	1	1	1	1	1	1	1	1	29.02612	1	1	1	1	1	1	1	1
OSM	77.13718	1	1	1	1	1	1	1	1	1	1	1	1	1	1	1	1	1	1	1
OSMR	1	1	1	1	1	1	1	1	1	1	1	1	1	1	1	1	1	204.9299	1	1
OSTF1	265.1794	1	1	1	1	1	1	63.2958	1	1	1	1	1	1	1	1	1	57.75611	1	1
OTUD1	718.9493	1	1	1	1	1	1	27.8118	1	1	1	1	33.46366	1	1	1	1	72.86786	1	103.6483
OTUD4	1	41.61441	1	44.89505	42.70854	28.41653	1	36.05064	1	42.26224	1	42.10484	50.52864	1	1	1	1	35.28886	68.31443	1
OTUD7B	1	1	1	1	1	1	1	1	1	1	1	1	30.948	1	1	1	1	10.57459	1	1
OTX1	1	45.1982	1	1	1	45.85727	1	1	1	41.33896	1	41.39994	40.59246	1	1	1	1	1	1	1
OXCT1	1	33.69349	78.06663	1	1	1	1	1	80.32076	37.99582	1	43.04094	1	1	1	58.24425	1	1	1	70.02264
OXLD1	1	1	1	1	1	34.8688	1	1	1	1	1	1	25.83185	1	1	1	1	1	1	1
OXSR1	1	41.45481	37.13564	75.7078	75.42538	50.13421	1	31.89861	1	53.60801	1	73.454	40.39174	1	1	39.11895	1	49.66059	92.17034	1
P2RX4	88.29475	1	1	1	1	1	1	1	1	1	1	1	1	1	1	1	1	24.55448	1	139.0453
P2RX7	1	1	65.30888	1	1	1	1	1	1	1	1	1	1	1	1	1	1	1	1	1
P2RY1	1	33.24261	1	1	1	37.62827	1	1	1	32.71321	1	1	47.28945	1	1	1	1	1	1	1
P2RY12	157.0292	1	1	1	1	1	1	1	1	1	1	1	1	1	1	1	1	1	1	1
P2RY13	295.3905	1	1	1	1	1	1	1	1	1	1	1	1	1	1	1	1	1	1	1
P2RY14	1	1	1	1	1	1	1	147.5269	1	1	1	1	1	1	1	1	1	1	1	1
P3H2	1	1	1	1	1	1	1	1	1	1	1	1	45.96669	1	1	1	1	1	1	1
P3H4	1	53.02811	1	1	1	44.1762	1	1	1	1	1	1	40.66084	1	1	56.91809	1	46.45484	1	70.02264
P4HB	134.2083	111.0819	172.9912	61.03147	53.85391	95.58491	1	297.6901	1	101.3709	1	102.755	81.82799	1	50.87705	67.17874	131.9746	253.0433	1	172.671
PAAF1	1	28.38153	62.76529	1	1	60.62534	1	1	1	28.13968	1	34.09588	44.67979	1	1	1	1	1	77.06562	1
PACRG	1	1	1	1	1	1	1	1	1	1	1	1	57.57283	1	1	1	1	1	1	70.02264
PACRGL	1	1	1	1	1	28.13709	1	1	1	1	1	1	34.52685	1	1	1	1	1	1	1
PACS1	1	32.72177	30.69027	1	53.73145	28.39631	1	42.59059	1	1	1	1	26.27959	51.95467	1	1	1	21.89355	1	1
PACSIN3	1	33.82717	1	1	1	64.65382	1	1	1	1	1	22.94675	36.94546	1	1	56.4994	1	1	1	1
PAICS	1	167.8493	98.90691	42.29029	1	105.2641	1	1	1	147.9636	1	133.7524	95.72557	1	1	181.7798	1	15.42181	1	1
PAK1	1	1	1	60.70989	52.6952	28.40891	1	1	73.23713	51.69719	1	38.73652	1	1	1	1	1	1	1	1
PAK5	1	1	1	139.9936	152.7504	1	142.1534	1	118.7552	1	133.8483	1	1	143.0154	241.1291	1	172.0917	1	144.5725	1
PALD1	1	1	1	1	1	1	1	130.1142	1	1	1	1	1	1	1	1	1	111.415	1	1
PALLD	1	150.5732	134.7889	76.73607	51.11979	303.4282	1	1	1	98.7934	1	129.7967	343.2396	67.46759	1	193.8002	1	1	1	70.02264
PALM2	1	1	1	1	1	1	1	1	250.6953	1	1	1	1	1	1	1	1	1	1	1
PALMD	1	1	1	339.1684	491.4129	1	292.5743	1	206.065	1	1	1	1	1	187.8366	1	206.6958	447.3156	225.3313	480.6159
PAM	1	37.9304	1	1	1	61.31796	1	69.56016	1	48.27191	1	51.54199	36.09399	146.9624	1	46.61211	1	87.95217	94.09173	1
PANK1	1	1	1	1	1	28.31046	1	1	1	1	1	1	1	1	1	1	1	1	1	1
PAPD4	1	40.07539	27.06019	1	1	38.46403	1	1	1	38.23542	1	37.68928	26.74497	1	51.2367	1	1	35.1144	1	1
PAPD5	1	33.63101	25.51499	1	48.14311	32.87026	1	39.69235	1	1	1	24.62549	36.67576	1	1	1	1	25.11759	1	1
PAPD7	1	1	36.94929	1	1	1	1	28.34014	1	1	1	49.8718	27.35683	1	52.03274	1	1	12.72186	1	70.02264
PAPOLG	1	1	1	1	51.12673	1	1	1	100.795	1	1	35.19579	1	1	1	1	215.4456	18.67091	1	1
PAPPA2	1	1	1	1	1	1	1	1	342.9364	1	1	1	1	1	1	1	1	1	1	1
PAPSS2	1	1	1	1	1	1	1	167.8527	1	1	1	1	1	1	1	1	1	20.70964	1	1
PAQR3	1	1	1	1	1	35.65863	1	1	1	43.6319	1	37.68946	29.05033	1	1	1	68.04639	1	86.88947	1
PAQR4	1	35.10615	49.99899	1	1	29.14622	1	1	1	57.02869	1	46.00042	49.30416	1	1	47.69827	1	1	1	1
PAQR8	1	1	1	1	1	89.34382	1	1	1	1	1	1	1	1	1	1	1	48.82616	1	1
PARD3B	1	1	1	1	1	49.32508	1	1	1	1	1	1	39.44673	1	1	1	1	1	1	1
PARD6A	1	1	1	1	41.27754	1	1	1	1	1	1	29.35394	27.75497	1	1	1	1	1	1	1
PARD6B	1	1	1	1	1	1	1	1	1	1	1	1	95.74745	1	1	1	1	1	1	1
PARD6G.AS	1	1	1	1	1	1	1	1	1	1	1	27.30254	1	1	1	1	1	1	1	1
PARG	1	29.33514	57.94125	1	1	24.30375	1	1	1	1	1	31.20344	31.81004	77.19269	1	1	139.363	26.64745	1	70.02264
PARP11	1	1	1	1	1	1	1	1	1	35.98146	1	29.41273	1	1	1	1	1	1	1	103.6483
PARP14	1	56.12747	1	1	1	56.9848	1	74.23042	1	1	1	1	51.19025	1	1	1	1	60.73591	1	1
PARP6	1	1	1	50.10703	51.09399	1	1	1	1	1	1	1	1	75.84906	67.64896	1	98.06675	1	1	103.6483
PARP8	1	1	1	1	1	1	1	21.63928	1	1	1	1	1	84.55216	1	1	1	32.72363	1	1
PARPBP	1	1	1	1	1	1	1	1	1	1	1	64.44362	1	1	1	1	1	1	1	1
PARVA	1	1	1	1	1	41.7933	1	106.5968	1	1	1	1	1	1	1	1	1	20.61367	1	1
PARVB	1	1	1	1	1	1	1	62.03992	1	1	1	1	1	1	1	1	1	121.6601	1	1
PARVG	45.72436	1	1	1	1	1	1	1	1	1	1	1	1	1	1	1	1	1	1	1
PATJ	1	1	1	1	1	1	1	1	1	1	1	1	1	1	1	1	1	1	41.39504	1
PAWR	1	57.98838	1	1	1	79.4869	1	107.0463	1	44.70039	1	41.89837	159.0735	1	1	101.1333	1	126.7205	1	70.02264
PAX6	1	217.3998	1	182.6777	69.00221	515.5397	1	1	1	412.9122	95.66347	481.8288	359.8433	1	1	355.6479	1	1	1	1
PAXIP1	1	1	1	1	1	32.38614	1	1	1	39.05536	1	35.08326	47.86491	1	1	1	1	1	1	1
PAXIP1.AS1	1	1	1	1	1	45.71717	1	1	1	1	1	26.40207	50.86262	1	1	1	1	1	1	1
PAXX	54.91451	81.57773	64.20891	1	59.65152	50.81267	1	50.72744	1	82.46139	1	92.63688	51.39492	1	64.90901	107.8469	112.0057	37.97072	1	103.6483
PBK	1	1	1	1	1	1	1	1	1	46.40151	1	286.8015	1	1	1	57.41664	1	1	1	103.6483
PBX3	1	1	55.02201	1	1	1	1	1	1	34.88461	1	35.73996	1	1	1	1	1	1	1	1
PBXIP1	1	53.3685	1	1	1	91.70334	1	1	1	1	1	22.10055	81.9437	1	1	1	1	1	1	1
PCAT19	1	1	1	1	1	1	1	1	1	1	1	1	1	1	1	1	1	98.40872	1	1
PCBD2	1	1	1	1	1	1	1	1	1	1	1	39.64061	1	1	1	1	1	1	1	1
PCBP4	1	1	169.9903	70.35035	50.71073	32.71652	1	1	59.18445	88.97162	1	36.27272	28.09995	1	1	1	115.7496	16.44873	71.46699	103.6483
PCCB	1	42.78797	31.9156	1	1	41.0345	1	1	1	1	1	27.29786	35.49768	1	1	1	1	1	1	1
PCDH10	1	83.84622	126.8939	1	1	1	1	1	287.3081	1	1	1	1	1	1	1	1	1	1	1
PCDH11X	1	1	80.79019	1	1	1	1	1	212.474	1	1	1	1	86.55026	1	1	1	1	1	1
PCDH12	1	1	1	1	1	1	1	1	1	1	1	1	1	1	1	1	1	125.8675	1	1
PCDH15	1	1	214.3917	1	1	1	1	1	1	1	1	1	1	1	1	1	1	1	1	1
PCDH17	1	171.2649	399.4096	1	1	30.42772	1	54.02645	113.3621	1	1	1	31.92118	1	1	1	1	24.04526	1	1
PCDH18	1	1	1	1	1	1	1	610.4751	1	1	1	1	1	1	1	1	1	1	1	1
PCDH19	1	1	47.90248	1	1	1	1	1	1	1	1	1	1	85.71465	1	1	1	25.81149	1	1
PCDH7	1	1	150.5119	54.52842	57.95031	30.05162	1	1	366.646	47.91004	1	42.28936	64.42917	1	52.44346	1	207.162	1	1	70.02264
PCDH8	1	52.94041	1	52.83299	119.2609	102.4063	1	1	1	30.5861	87.66318	45.69795	1	118.5906	57.76754	1	1	1	1	1
PCDH9	1	160.0248	624.6979	62.56409	1	107.075	1	38.62515	794.4349	83.87972	774.7258	87.41206	103.7288	1	1	1	340.0731	1	1	139.0453
PCDHA10	1	1	1	1	1	1	1	1	1	1	1	1	1	60.64059	49.34553	1	60.45966	1	1	1
PCDHGA10	1	56.91589	1	62.73345	59.11758	1	1	27.42606	133.1498	1	79.56556	1	1	87.64958	65.49641	1	240.2217	1	1	172.671
PCDHGB6	1	1	1	1	1	1	1	1	1	1	1	1	1	1	1	1	1	1	48.65011	70.02264
PCDHGC3	1	52.56019	138.1355	1	1	1	1	23.00755	1	1	1	1	1	1	1	1	1	1	1	1
PCGF5	1	42.64471	1	1	1	121.9634	1	1	1	65.78968	1	62.94764	154.6336	1	1	132.102	1	1	1	1
PCGF6	1	1	1	1	1	1	1	1	1	38.14311	1	29.81336	26.60464	1	1	60.47902	1	1	1	1
PCID2	1	41.47491	73.53358	51.68649	52.98193	33.71176	1	1	1	49.94782	1	41.83997	46.15042	67.32619	1	1	1	58.06715	1	1
PCIF1	1	39.29179	37.48337	1	45.37136	1	1	1	1	1	1	32.37878	33.14641	64.55877	1	1	1	23.50455	1	1
PCLAF	1	68.72863	70.83116	1	1	1	1	1	1	487.4037	1	547.3206	106.6347	1	1	217.6546	1	1	1	1
PCLO	1	1	1	127.2955	205.3391	1	168.0792	1	466.1269	1	90.28012	1	1	113.7846	334.3992	1	672.5649	1	269.3155	618.6612
PCNA	1	132.0429	66.09732	1	50.13684	72.53105	1	74.17147	1	522.3541	1	381.3131	108.0701	1	1	230.0606	1	117.3285	1	1
PCNT	1	1	1	1	1	1	1	29.2872	1	50.81763	1	71.68961	1	1	1	1	1	1	1	1
PCNX2	1	1	1	1	1	1	1	1	119.4556	1	1	1	1	1	1	1	1	1	1	1
PCOLCE	1	1	1	1	1	1	1	298.1446	1	1	1	1	1	1	1	1	1	1	1	1
PCP4	1	1	1	1	1	1	1	1	884.6803	1	1	1	1	1	1	1	1	1	1	1
PCSK1N	1	95.49337	342.8991	278.0691	381.7973	137.644	543.3348	1	953.3078	86.27951	285.3157	64.45412	138.7192	337.2659	653.4313	134.5857	591.1443	1	540.0327	618.6612
PCSK2	1	1	1	1	1	1	1	1	76.75962	1	1	1	1	1	1	1	1	1	1	1
PCSK6	1	1	1	1	1	37.90909	1	1	1	1	1	1	42.72577	1	1	1	1	1	1	1
PCYT2	1	1	1	1	1	36.05594	1	1	1	1	1	1	23.21141	1	1	1	1	1	1	1

PDCD2	58.476	58.38538	59.93061	56.7631	63.14975	57.94408	119.156	39.56079	1	66.55564	61.94759	45.14751	62.45063	1	85.99126	72.42264	1	130.0346	1	70.02264
PDE10A	1	1	1	1	43.59913	1	1	1	1	1	1	1	1	1	50.89142	1	1	22.88391	83.15837	1
PDE1A	1	1	1	1	1	26.2056	1	1	490.4385	31.45685	1	30.14315	60.03477	1	1	1	1	1	66.74201	1
PDE1C	1	1	1	97.68249	65.34114	1	1	1	1	44.7349	1	34.05158	1	1	1	1	1	1	1	1
PDE2A	1	1	1	1	1	1	1	71.33358	101.5912	1	1	1	1	1	1	1	1	1	1	1
PDE3B	1	1	73.8589	1	1	1	1	1	1	1	1	1	1	1	1	1	1	1	1	1
PDE4B	1	68.46584	243.6176	1	1	40.594	1	67.56327	1	1	1	32.08845	35.86394	1	1	1	1	77.59901	1	1
PDE4D	1	1	43.00541	50.6977	85.53335	1	1	65.17108	80.24535	1	106.4467	1	1	228.0794	111.4298	1	198.7915	59.43132	87.94547	1
PDE4DIP	90.70633	66.36682	79.36786	147.9958	149.5122	119.6713	1	33.99282	195.9727	93.42249	422.932	95.25191	88.11614	1210.882	144.87	117.0589	315.2422	1	45.85936	208.0679
PDE5A	1	1	1	1	1	1	1	232.6928	1	1	1	1	1	1	1	1	1	1	1	1
PDE6D	1	30.1377	66.78457	1	1	50.03867	1	33.33574	1	1	1	30.24664	26.76677	1	55.62456	1	1	1	1	1
PDE7A	1	34.24788	1	95.63523	94.41815	28.41105	1	1	1	85.07484	105.4466	83.41041	32.38281	69.85613	85.13636	1	150.3042	39.56676	90.51802	1
PDE7B	1	1	1	1	1	1	1	107.9135	1	1	1	1	1	1	1	1	1	1	1	1
PDE8A	1	1	1	1	1	1	1	126.2332	1	1	1	1	1	1	1	1	1	30.24374	1	1
PDE8B	1	1	1	1	1	29.61105	1	281.1326	1	1	1	1	1	1	1	1	1	1	1	1
PDE9A	1	1	1	56.41338	52.36087	54.82309	1	1	1	55.59814	1	39.29126	50.97352	1	1	1	1	1	1	1
PDGFA	1	1	89.74211	1	1	1	1	1	1	1	1	1	1	1	1	1	1	1	1	1
PDGFB	1	1	1	1	1	1	1	1	1	1	1	1	1	1	1	1	1	142.188	1	1
PDGFD	1	1	1	1	1	73.73466	1	1	1	1	1	29.42273	46.68388	1	1	72.18648	1	1	1	1
PDGFRA	1	376.5935	2292.08	1	1	1	1	1	1	1	1	1	1	1	1	1	1	1	1	1
PDGFRB	1	1	1	1	1	73.45662	1	1032.003	1	1	1	1	36.38771	1	1	1	1	1	1	1
PDHA1	1	58.176	56.30276	89.23654	123.4794	73.00624	173.7491	42.91591	129.6695	91.55848	63.96124	72.16641	62.47006	72.32591	161.5445	73.23799	1	33.63554	151.4162	1
PDHX	1	1	1	1	1	29.99958	1	1	1	32.5839	1	1	28.32279	1	1	1	1	1	1	1
PDK4	194.5673	1	1	1	1	1	1	1	1	1	1	1	1	1	1	1	1	46.2663	1	1
PDLIM1	1	79.52328	1	1	1	1	1	169.9807	1	1	1	1	63.12799	1	1	1	1	1	1	1
PDLIM2	66.19431	47.99032	64.8954	1	1	64.4767	1	296.7317	1	1	1	32.74224	50.59766	1	1	1	1	224.4417	1	1
PDLIM3	1	172.5642	30.106	1	1	225.8701	1	1	1	85.41596	1	109.3578	300.7405	1	1	187.1799	1	1	1	1
PDLIM4	1	41.6454	1	1	1	45.15083	1	1	1	37.16303	1	29.0661	51.91629	1	1	55.47756	1	1	1	1
PDLIM5	1	1	91.65811	1	1	108.1387	1	55.9279	1	1	1	32.18169	82.54113	1	1	61.93172	1	48.74857	1	1
PDLIM7	1	1	1	1	1	45.25069	1	86.60618	1	25.49057	1	38.86708	45.58266	1	1	65.28859	1	29.19423	1	1
PDP1	1	1	1	64.27412	78.44084	1	1	25.06875	54.45469	46.26304	111.321	26.41432	36.61035	158.3125	57.31574	1	1	49.01849	1	1
PDPN	193.7984	120.4196	1	1	1	142.9592	1	1	1	61.47219	1	76.84866	75.32162	1	1	117.7946	1	1	1	1
PDSS1	1	30.24176	1	1	1	1	1	1	1	1	1	1	1	1	1	1	1	1	1	1
PDXDC1	1	1	1	1	38.21965	1	1	1	1	24.7489	1	25.10763	25.9433	1	1	1	1	22.05679	1	1
PDXK	213.705	36.49345	1	1	1	57.2705	1	53.2346	1	42.82559	1	31.26639	89.48271	1	1	1	1	552.0679	1	1
PDZD2	1	1	71.17523	1	1	1	1	248.3513	412.2286	1	1	1	1	1	1	1	1	43.70937	1	1
PDZD4	1	1	39.00827	43.23488	44.01932	1	1	1	90.59725	1	81.2037	1	23.41443	84.6166	59.69189	1	85.11225	1	1	1
PDZRN3	1	1	1	1	1	1	1	47.29132	1	1	541.0113	1	1	123.1902	1	1	1	1	1	1
PDZRN4	1	1	38.34026	1	1	35.49598	1	1	1	1	138.0462	1	1	465.5118	1	1	1	1	1	1
PEAK1	1	44.72308	125.469	1	1	1	1	182.3553	1	1	1	1	1	1	1	1	1	20.52232	1	1
PECAM1	1	1	1	1	1	1	1	1	1	1	1	1	1	1	1	1	1	403.2239	1	1
PELI2	1	55.01009	34.57929	1	50.84479	155.2017	1	30.57473	1	76.36824	88.33274	81.73169	147.6041	1	1	70.38542	1	45.16985	1	103.6483
PELO	1	1	69.57233	1	1	1	1	175.2336	1	1	1	1	27.91721	1	1	1	1	350.9245	1	1
PENK	1	1	1	193.9844	1	1	1	1	1	134.3501	1	180.9209	1	1	59.6414	1	1	1	76.60632	1
PER1	1	1	1	1	1	28.12671	1	90.18551	1	1	1	24.55336	59.9871	1	1	1	1	34.45655	1	1
PER2	1	1	1	1	1	36.15264	1	1	1	40.34528	1	32.80455	96.82495	1	1	69.22046	1	19.81875	1	1
PEX10	1	41.95939	49.19934	1	1	56.40825	1	1	1	1	1	1	37.2119	1	1	50.6157	1	1	1	1
PEX16	1	1	1	1	1	27.4999	1	1	1	1	1	1	1	1	1	1	1	1	1	1
PEX19	1	1	1	1	1	37.81603	1	1	1	1	1	25.36089	27.30128	1	1	57.16614	1	107.1906	1	1
PEX3	1	1	1	1	1	27.5982	1	1	1	1	1	1	1	1	1	1	1	1	1	1
PEX5L	1	1	1	1	1	1	1	1	117.3538	1	1	1	1	1	1	1	1	1	1	1
PFDN6	57.35733	59.24087	39.64004	46.17876	53.68525	61.57286	1	33.91865	1	63.6324	1	42.12185	54.04308	1	90.42447	79.0854	1	25.81919	1	87.58009
PFKFB3	1	45.18103	1	1	50.18828	104.2572	1	64.60178	1	1	1	38.86791	130.1345	1	1	1	1	63.18692	1	1
PGBD1	1	1	1	1	1	1	1	1	1	42.67837	1	29.23543	1	1	1	1	1	1	1	1
PGBD5	1	1	1	1	1	1	1	1	1	1	78.80469	1	1	1	1	1	1	1	1	1
PGF	1	1	1	1	1	1	1	264.8466	1	1	1	1	1	1	1	1	1	1	1	1
PGM1	1	64.31991	78.95543	1	1	69.62865	1	1	1	1	1	29.1083	26.18733	1	1	1	1	1	1	1
PGM2	1	1	24.167	1	1	54.00796	1	29.97788	1	30.76763	1	43.24466	56.67537	1	1	57.61826	1	18.02741	1	1
PGM2L1	1	36.71172	1	153.8143	221.3195	1	1	45.5722	542.1587	72.92712	177.3914	65.42808	1	183.066	224.4928	1	354.3246	63.32314	139.3439	139.0453
PGM3	1	82.31882	166.6486	1	1	75.80597	1	31.07629	1	54.29094	68.44414	58.26661	51.98476	1	1	64.53972	1	26.81323	1	1
PGPEP1	1	32.1634	1	1	1	30.79848	1	1	1	34.68045	1	1	1	1	1	1	1	1	1	1
PHACTR3	1	35.52595	103.124	103.7962	97.36779	44.62881	1	1	105.4986	36.84502	89.20206	1	1	111.1794	93.07151	1	225.4535	1	102.0902	139.0453
PHC1	1	35.3436	36.64708	49.55497	47.95607	48.85602	1	23.41998	1	44.19614	71.11622	47.36784	52.0448	1	1	72.17515	1	25.02367	173.7504	70.02264
PHF11	1	37.6787	1	1	1	51.06065	1	41.41989	1	1	1	1	35.96519	1	1	1	1	13.29548	1	1
PHF13	1	33.05785	1	1	1	35.50174	1	1	1	1	1	31.32818	55.93033	1	1	1	1	1	1	1
PHF19	1	1	1	1	1	1	1	1	1	37.23095	1	84.43136	28.63145	1	1	58.34197	1	1	1	1
PHF21B	1	28.4754	1	55.12935	47.18437	1	1	1	1	36.62688	85.78879	30.74618	1	101.7153	1	1	1	1	1	1
PHGDH	1	173.9401	134.781	1	1	311.0825	1	1	1	96.91566	1	245.8225	314.2585	1	1	249.5822	1	1	1	1
PHLDB1	1	69.05874	141.2729	46.35732	1	39.41713	1	83.04432	77.61252	1	89.53883	31.43763	60.01813	67.24398	1	1	1	57.10718	1	1
PHLDB2	1	1	1	1	1	1	1	76.15946	1	1	1	1	1	1	1	1	1	36.73166	1	1
PHLDB3	1	1	1	1	1	1	1	75.1017	1	1	1	1	1	1	1	1	1	1	1	1
PHLPP1	1	96.57108	75.67545	1	1	110.0692	1	39.72615	1	56.54431	1	62.13098	55.09552	1	1	105.1752	1	27.85874	41.93573	1
PHRF1	1	1	1	1	1	1	1	1	1	45.55352	1	29.42225	23.55155	1	1	1	1	23.96679	1	1
PHYKPL	1	40.41521	1	50.45091	1	44.05327	1	1	1	36.2066	1	25.74969	33.33271	1	1	60.79259	1	103.6708	1	1
PI4KA	1	1	1	1	1	1	1	32.36626	126.6084	1	1	29.43288	1	1	1	1	1	1	1	70.02264
PIANP	1	1	41.37156	1	44.78314	26.74216	1	1	100.4356	28.76628	1	18.01464	25.04889	1	1	1	1	1	1	206.2967
PIAS4	1	27.69798	33.91042	1	1	27.73961	1	1	1	42.5558	1	29.51792	29.32636	1	1	1	1	99.6989	95.73244	1
PICK1	1	1	1	1	1	1	1	1	1	1	1	1	1	1	55.95471	1	1	1	1	103.6483
PID1	1	68.98732	150.5187	1	93.40882	1	1	67.93793	1	1	192.079	40.19867	1	1	174.1433	1	221.4467	1	74.74432	103.6483
PIDD1	1	1	1	1	1	1	1	1	1	36.97236	1	1	1	1	1	1	1	1	1	1
PIF1	1	1	1	1	1	1	1	1	1	1	1	61.81109	1	1	1	1	1	1	1	1
PIFO	1	1	1	1	1	1	1	1	1	1	1	1	139.8737	1	1	1	1	1	1	1
PIGBOS1	1	30.71418	30.06917	1	1	32.9528	1	27.76365	1	1	1	23.85693	28.60586	1	1	1	1	1	1	103.6483
PIGH	1	32.41595	81.53777	1	1	32.17005	1	29.32002	1	1	1	27.66137	29.32141	1	1	1	1	25.14622	1	1
PIGM	1	1	48.61615	1	1	34.6147	1	1	1	36.15234	1	26.85856	39.32409	1	1	1	126.332	1	48.92959	1
PIGP	1	1	1	1	1	22.78369	1	30.25779	1	1	1	1	30.81518	1	1	1	108.6686	22.34208	1	1
PIH1D1	505.7113	59.92228	109.3629	60.75026	57.01712	62.52739	1	39.87178	124.2847	66.41479	1	67.2687	66.39871	1	1	61.31626	1	19.8579	90.18104	116.5001
PIK3C2B	1	1	1	1	1	27.01269	1	1	1	1	1	1	1	1	1	1	1	20.81864	1	103.6483
PIK3CB	1	35.5086	1	1	1	33.69863	1	1	1	1	1	28.63784	30.68209	1	1	1	1	1	1	1
PIK3IP1	47.56395	1	1	1	1	1	1	26.57526	1	1	1	1	1	1	1	1	1	19.08024	1	1
PIK3R3	1	1	63.10079	48.90758	54.35101	1	1	40.56593	1	39.68141	1	57.74767	1	80.41549	64.8721	1	1	31.66349	1	70.02264
PIK3R6	1	1	1	1	1	1	1	1	1	1	1	1	1	1	1	1	1	62.28103	1	1
PIM1	1	38.99193	1	1	44.72979	60.88965	1	1	1	33.14755	1	61.12726	145.0995	1	1	1	1	68.67937	1	1
PIMREG	1	1	1	1	1	1	1	1	1	1	1	191.0155	1	1	1	1	1	1	1	1
PIP4P2	1	36.4719	39.54104	1	42.68497	64.46756	1	67.72148	1	38.75839	1	40.43089	39.2672	69.22975	1	1	1	1	1	103.6483
PIP5K1C	1	1	1	1	1	24.6293	1	33.40467	132.5683	33.86868	1	1	1	1	1	1	117.7096	71.59368	1	1
PIR	1	1	1	1	1	27.29097	1	1	1	1	1	1	1	1	1	1	1	1	1	1
PISD	1	1	1	1	51.98854	1	1	1	1	1	1	1	27.14256	1	1	1	1	31.43359	1	1
PITPNA	79.70067	51.53952	1	1	1	64.47429	1	45.44174	1	1	1	40.14182	59.14369	1	1	51.33241	1	113.4526	56.46744	1
PITPNM1	1	1	1	1	1	1	1	32.81065	1	1	1	1	41.67129	1	1	1	1	1	1	1
PKD1	1	42.84193	38.51684	67.03278	65.81107	37.59163	1	20.83181	213.4024	52.37068	1	44.15583	43.5762	53.73185	1	1	228.5765	17.42877	1	70.02264

PKIA	1	76.13371	194.1205	205.3637	276.7859	98.83464	403.6128	1	631.1162	131.08	165.0693	98.08637	76.69481	150.0437	411.5666	92.64995	442.9576	1	149.8513	377.9676
PKMYT1	1	1	1	1	1	1	1	1	1	1	1	34.43038	1	1	1	1	1	1	1	1
PKNOX1	1	28.77071	1	43.43826	51.42309	33.50293	1	1	1	31.14512	1	63.39404	29.76336	1	1	45.33002	1	32.11582	93.78558	1
PKP4	1	1	89.61439	1	1	1	1	1	1	1	1	39.06831	1	1	1	1	1	166.1341	1	1
PLA2G12A	1	1	1	1	42.23203	36.36707	1	35.29202	1	28.49406	1	30.65093	30.70919	1	1	1	1	1	1	139.0453
PLA2G15	84.58978	1	1	1	1	1	1	1	1	1	1	1	1	1	1	1	1	1	1	1
PLA2G16	128.1053	1	1	1	1	1	1	1	1	1	1	1	1	1	1	1	1	291.9271	1	1
PLA2G7	354.5545	1	1	1	1	1	1	1	1	1	1	1	1	1	1	1	1	1	1	1
PLA2R1	1	1	1	1	1	1	1	56.20918	1	1	1	1	1	1	1	1	1	1	1	1
PLAC9	1	1	1	1	1	80.54278	1	530.469	1	1	1	1	1	1	1	113.4971	1	1	1	1
PLAT	1	1	97.30065	1	1	47.73998	1	207.8976	1	1	1	1	1	1	1	1	1	62.8596	1	1
PLAU	90.59344	1	1	1	1	1	1	51.70768	1	1	1	1	1	1	1	1	1	1	1	1
PLAUR	75.83925	1	1	1	1	1	1	45.98107	1	1	1	1	1	1	1	1	1	22.5434	1	1
PLCB1	1	58.714	161.6112	327.3597	245.9361	51.75598	184.1805	1	162.7368	187.9885	1	115.8947	39.88412	1	128.8379	104.9472	132.1691	91.99145	285.9233	1
PLCB4	1	1	1	110.8524	1	1	1	1	1	81.76008	1	56.87102	1	1	1	1	1	46.4636	1	1
PLCE1	1	144.774	43.94032	100.7792	57.1634	139.8839	1	359.0645	1	74.31066	1	94.61564	204.9806	1	1	163.4131	1	1	62.04869	1
PLCG2	1	1	1	1	1	1	1	1	110.9746	1	1	1	1	1	1	1	102.4485	18.7637	1	1
PLCH1	1	1	1	1	1	29.42209	1	1	1	1	1	1	40.41255	1	1	1	1	1	1	1
PLCL1	1	1	1	1	1	1	1	124.1795	43.17127	1	1	1	1	1	1	1	1	1	1	1
PLCL2	1	1	1	48.14757	43.57441	1	1	1	77.88325	1	1	1	1	1	1	1	1	1	1	1
PLCXD1	1	1	1	1	1	1	1	1	1	41.28289	1	27.40067	1	1	1	1	1	1	1	1
PLD4	237.6194	1	1	1	1	1	1	1	1	1	1	1	1	1	1	1	1	1	1	1
PLEK	502.0329	1	1	1	1	1	1	1	1	1	1	1	1	1	1	1	1	1	1	1
PLEKHA7	1	1	1	1	1	1	1	1	1	1	1	1	41.68798	1	1	1	1	1	1	1
PLEKHB1	1	46.84966	91.07372	1	1	52.94003	1	1	1	1	1	1	26.12173	1	1	1	1	1	1	1
PLEKHF2	53.79046	37.59701	1	1	1	1	1	46.28935	1	62.04139	67.69254	36.4428	1	1	1	1	1	12.57997	1	1
PLEKHG1	1	1	1	1	1	57.53709	1	29.52006	1	1	1	28.31823	44.61129	1	1	1	1	114.1256	1	1
PLEKHG2	1	1	1	1	1	1	1	80.99067	1	1	1	1	22.57773	1	1	1	1	31.76097	1	1
PLEKHG4B	1	1	1	1	1	1	1	1	1	1	1	28.68203	45.09852	1	1	1	1	1	1	1
PLEKHH2	1	100.0982	110.1986	1	1	1	1	62.40225	368.2966	1	1	1	1	1	1	1	1	1	1	1
PLEKHH3	1	1	1	1	1	1	1	68.84366	1	1	1	1	1	1	1	1	1	1	1	1
PLEKHO2	49.9761	1	1	1	1	1	1	1	1	1	1	1	1	1	1	1	1	25.55868	1	1
PLIN2	1524.144	55.74619	1	1	1	59.57028	1	47.04389	1	119.1876	1	94.53155	53.88422	1	1	54.98411	1	257.1324	1	1
PLK1	1	1	1	1	1	1	1	1	1	1	1	121.7288	1	1	1	1	1	1	1	1
PLK2	1	1	1	167.3081	225.7023	35.50904	1	246.1451	298.7975	63.34989	490.7962	49.05949	1	226.3846	84.12386	1	183.3758	634.2086	1	1
PLK3	70.25401	1	1	1	1	1	1	50.38521	1	1	1	1	29.27512	1	1	1	1	60.3766	1	1
PLK4	1	1	1	1	1	1	1	1	1	1	1	60.34442	1	1	1	1	1	1	1	1
PLLP	1	1	131.2908	1	1	1	1	1	1	1	1	1	1	1	1	1	1	158.4225	1	1
PLOD1	1	1	1	1	1	1	1	237.7871	1	1	1	1	1	1	1	1	1	36.00921	1	1
PLOD2	1	87.06465	103.7871	1	1	56.39766	1	63.86888	1	1	1	33.69644	76.63808	1	1	1	1	1	1	1
PLP1	1	1	368.6233	1	1	1	1	1	1	1	115.8612	1	57.63549	1	1	1	1	1	1	1
PLP2	1	1	1	1	1	36.04003	1	1	1	27.49213	1	46.46941	57.56523	1	1	1	1	1	1	1
PLPP3	1	182.0089	1	1	1	282.6398	1	1	1	46.21602	1	95.74253	80.57954	1	1	199.5117	1	1	1	1
PLPP4	1	1	33.11248	1	1	1	1	1	1	1	1	1	1	1	1	1	1	1	1	1
PLPPR1	1	1	381.5305	89.42229	55.54714	29.17511	1	1	157.9294	81.39021	1	47.1279	1	1	84.27435	1	1	1	52.28269	116.5001
PLPPR2	1	1	54.73911	1	42.86762	25.90122	1	44.09735	108.7166	1	1	1	1	1	52.84642	1	1	1	1	1
PLPPR3	1	32.96841	42.30114	140.7573	169.4583	57.10982	249.4738	1	291.3308	65.79333	160.3372	45.25472	54.95905	197.9955	191.043	1	389.5186	1	130.943	308.945
PLPPR4	1	1	1	86.03458	79.09843	1	1	1	252.0808	94.95905	1	81.60846	50.10951	1	1	45.4756	120.708	1	184.4959	1
PLPPR5	1	1	39.28549	1	67.91402	1	1	1	115.0842	1	1	1	1	1	102.9324	1	1	1	57.33456	1
PLS3	1	43.13814	1	1	1	1	1	306.886	1	1	604.5185	1	1	600.0499	1	1	1	96.40663	1	1
PLSCR1	121.9537	29.70204	1	1	1	34.37482	1	82.68458	1	1	1	1	57.59756	1	1	1	1	84.73793	1	1
PLSCR4	1	1	1	1	1	1	1	61.42107	1	1	1	1	1	1	1	1	1	24.41939	1	1
PLTP	82.00547	61.56704	1	1	1	58.87586	1	273.5508	1	1	1	1	107.9029	1	1	1	1	19.16512	1	103.6483
PLVAP	64.59452	1	1	1	1	1	1	1	1	1	1	1	1	1	1	1	1	1	1	1
PLXDC1	1	1	1	1	1	1	1	1116.736	1	1	1	1	1	1	1	1	1	1	1	1
PLXDC2	350.3961	1	1	1	1	1	1	31.92504	75.69245	1	1	1	1	1	1	1	1	1	1	1
PLXNA1	1	1	49.60205	1	1	1	1	1	81.08193	1	1	1	1	1	1	1	103.2416	1	1	70.02264
PLXNA2	1	53.59824	51.39573	321.4852	429.1169	1	424.1999	1	757.3308	129.0722	91.50601	65.06211	1	196.9152	616.807	87.57354	425.1729	66.945	464.4811	172.671
PLXNA3	1	1	1	52.00652	59.10038	24.69482	1	1	65.01958	31.68969	1	1	1	62.14513	1	1	1	1	1	1
PLXNA4	1	1	1	117.93	210.7917	1	292.7135	1	1	36.01612	1	35.31312	33.55037	1	455.6672	82.87457	438.6505	1	124.0133	411.5933
PLXNB1	1	1	1	1	1	43.79843	1	1	1	1	1	1	52.42562	1	1	1	1	1	1	1
PLXNB2	1	30.55163	1	58.8878	1	37.30713	1	51.08236	1	39.24917	1	35.26082	37.8106	1	1	1	1	41.44615	1	1
PLXNC1	1	98.76807	1	1	1	54.15824	1	1	96.67575	73.50617	1	68.12042	28.55719	1	1	59.95154	1	1	122.2829	1
PLXND1	1	1	1	1	1	1	1	40.58975	1	1	1	1	1	1	1	1	1	108.813	1	1
PM20D2	1	49.52509	55.63268	1	1	51.66972	1	1	1	60.89992	1	42.26638	61.79512	1	1	49.59011	1	1	1	1
PMAIP1	162.2127	1	1	1	1	1	1	41.93098	1	1	1	1	1	1	1	1	1	110.802	1	1
PMM1	1	1	1	1	39.74073	86.37074	1	1	1	45.95176	1	46.51324	48.67779	1	1	86.69181	1	1	1	1
PMP2	1	285.8408	895.1263	1	1	604.543	1	1	1	57.10524	1	104.4575	216.3577	1	1	181.7821	1	1	1	1
PMS1	1	49.83418	1	49.432	42.90418	38.04846	1	22.28435	1	46.45563	1	44.01771	40.20501	1	1	1	1	1	64.85478	1
PMS2	1	1	1	1	1	1	1	1	1	1	1	1	1	1	1	1	1	1	59.2474	1
PNCK	1	1	1	1	1	1	1	1	106.2696	1	1	1	1	1	1	1	1	1	1	1
PNMA8C	1	1	1	1	1	1	1	1	1	1	1	1	57.70487	1	1	1	1	1	1	1
PNO1	1	59.0182	46.77799	1	1	24.39382	1	1	1	1	1	1	31.80291	1	1	1	1	1	1	1
PNOC	1	1	1	1	1	1	1	1	1	1	88.72141	1	1	1	1	1	1	1	1	1
PNPT1	1	45.12358	55.8235	1	1	31.6361	1	1	1	46.60945	1	37.61703	42.89493	1	49.35046	1	1	34.09701	1	1
POC1A	1	1	1	1	1	1	1	1	1	1	1	58.31105	1	1	1	1	1	1	1	1
PODXL	1	1	1	1	1	1	1	1	1	1	1	1	1	1	1	1	1	383.8169	1	1
POFUT2	1	1	1	1	1	1	1	35.70931	1	1	1	1	38.58482	1	1	1	1	1	1	1
POLA1	1	1	1	1	1	1	1	1	1	37.20237	1	35.73259	1	1	1	1	1	1	1	1
POLA2	1	1	1	1	1	1	1	1	1	34.396	1	26.12843	1	1	1	1	1	1	1	1
POLB	1	1	1	51.52191	53.87122	1	1	1	1	30.04089	1	27.78889	1	77.19227	56.12201	1	71.67022	19.13579	1	1
POLD3	1	44.74742	1	1	1	40.2462	1	1	1	99.71282	1	96.49038	60.64241	1	1	39.9856	1	1	1	1
POLE	1	1	1	1	1	1	1	1	1	40.37596	1	31.76968	1	1	1	1	1	1	1	1
POLE4	1	1	86.81876	1	1	46.13282	1	90.40611	1	26.47402	1	43.28812	32.90081	1	47.21433	1	97.33836	1	1	1
POLG	1	1	1	1	1	1	1	1	1	1	1	24.06761	1	1	1	1	120.1635	22.03248	1	103.6483
POLG2	1	1	1	1	1	1	1	57.84903	1	1	1	1	1	1	1	1	1	1	1	1
POLH	1	1	1	1	1	1	1	1	1	1	1	36.4844	1	1	1	1	1	1	1	1
POLI	1	43.49115	1	1	1	36.54288	1	1	1	1	1	23.38631	29.71059	1	1	1	1	1	1	1
POLQ	1	1	1	1	1	1	1	1	1	1	1	31.40519	1	1	1	1	1	1	1	1
POLR1A	1	1	30.65589	1	1	1	1	1	1	1	1	1	1	1	1	1	1	1	1	1
POLR1C	1	1	1	1	1	1	1	1	1	1	1	1	39.04721	1	1	1	1	1	1	1
POLR1E	1	49.87538	1	1	1	33.81312	1	1	1	32.39796	1	28.20682	79.61149	1	1	1	1	1	1	1
POLR3A	1	1	1	50.65265	44.81066	1	1	26.76057	1	34.40412	1	31.47606	31.24811	67.69107	54.48954	1	1	108.2662	1	70.02264
POLR3H	1	1	1	1	1	51.45285	1	1	1	1	1	1	47.94822	1	1	1	1	1	1	103.6483
POM121C	1	44.20892	34.23697	49.29145	45.26296	30.05326	1	38.85338	1	45.94071	1	42.44455	54.1217	1	1	1	144.8553	48.9712	1	103.6483
POMT1	1	1	1	1	1	30.92891	1	1	1	1	1	1	1	1	1	1	1	1	1	1
POMT2	1	32.83652	74.46055	1	1	1	1	1	1	1	1	1	1	1	1	1	1	1	1	70.02264
PON2	1	411.5905	134.7777	1	1	566.903	1	124.0888	1	88.39431	1	188.776	242.7974	1	1	253.7831	1	588.6708	1	1
POR	1	45.40565	1	1	1	49.54844	1	45.37853	85.76675	1	1	1	34.72724	1	46.87715	1	94.44166	29.12089	1	1
POU2F1	1	80.98352	62.54143	144.6233	109.4043	113.7499	1	47.09118	1	146.3957	249.9234	118.9562	81.55205	174.6693	85.75772	192.7921	1	34.79042	61.49748	1
POU2F2	53.85544	1	1	1	55.69017	1	1	1	121.7145	1	94.68542	1	1	77.3949	64.63368	1	191.9381	1	25.16361	172.671

POU3F1	1	1	1	1	69.36085	1	1	1	1	1	1	1	1	1	102.7127	1	118.2866	1	128.3612	70.02264
POU3F2	1	358.8055	199.823	1283.989	1407.204	343.593	1187.617	34.63966	108.3993	923.842	91.82874	811.768	233.8198	138.0726	1160.073	459.2398	1010.065	139.863	1245.244	997.4001
POU3F3	1	285.4129	129.7104	710.4659	769.4072	388.5305	696.3014	1	112.8308	511.8218	331.7738	449.7567	321.8639	368.1691	615.9102	298.4038	335.734	102.2493	518.6033	892.6985
POU3F4	1	55.75987	1	76.37569	69.24487	72.80095	1	1	1	83.82127	268.0267	90.18114	71.27671	131.2222	1	72.55134	1	1	30.37662	1
POU6F1	1	1	1	1	1	1	1	1	1	1	1	1	1	1	1	1	146.6903	1	1	1
PPARA	1	1	1	1	1	28.56173	1	1	1	1	1	1	1	1	1	1	1	1	1	1
PPARD	1	1	1	1	1	1	1	36.23829	1	1	1	1	1	1	1	1	1	151.496	1	1
PPAT	1	46.72043	61.58862	1	1	49.40239	1	1	1	45.35077	1	65.78339	36.82524	1	1	62.98177	1	14.67742	1	1
PPFIA3	1	1	1	1	1	1	1	1	1	1	1	1	1	1	1	1	57.9244	16.96535	1	70.02264
PPFIBP1	1	1	62.87545	1	1	1	1	171.5181	1	1	1	1	1	1	1	1	81.65152	130.3642	1	1
PPFIBP2	1	1	1	1	1	1	1	52.57624	1	1	1	1	1	1	1	1	1	1	1	1
PPIC	1	1	1	1	1	1	1	54.25351	1	1	1	1	1	1	1	1	1	64.31242	1	1
PPIF	137.5978	44.1962	34.98601	1	1	42.90761	1	73.65329	1	71.41587	1	77.03054	46.63572	1	1	101.4735	1	1	1	70.02264
PPIL2	1	1	35.71308	47.68208	48.21014	1	1	1	1	1	1	34.40861	24.4312	56.33122	49.19444	1	225.7526	1	1	1
PPIL3	1	1	1	40.06404	1	33.28689	1	1	1	31.04863	1	43.16806	31.85461	1	1	1	1	1	1	1
PPIL6	1	1	1	1	1	1	1	1	1	1	1	1	88.44089	1	1	1	1	1	1	103.6483
PPM1D	1	33.10369	1	1	44.26096	1	1	1	1	68.56434	1	48.91608	30.44321	1	1	1	1	30.40717	1	70.02264
PPM1E	1	1	1	1	1	1	1	1	113.8141	1	1	1	1	1	1	1	1	1	1	1
PPM1H	1	1	1	1	1	1	1	1	1	1	1	1	1	1	1	1	1	49.75993	1	1
PPM1L	1	1	28.29103	66.69142	54.35232	35.55326	1	30.86985	336.9909	40.91744	1	32.82234	1	62.89878	1	1	35.69297	1	89.81422	103.6483
PPME1	1	34.78931	71.79307	54.04956	55.01478	26.91716	1	1	57.55081	57.68873	63.31724	59.6728	41.41673	71.03787	1	1	99.11571	1	1	139.0453
PPP1R12B	1	1	108.6389	68.4345	59.49809	27.39869	1	36.94955	72.11066	58.03354	274.6776	47.84463	43.25429	100.1835	65.43748	1	1	1	78.38452	87.58009
PPP1R13B	1	1	1	1	1	1	1	1	63.24129	1	1	1	1	1	1	1	1	98.67519	1	1
PPP1R14A	1	1	1	1	1	1	1	208.5298	1	1	1	1	1	1	1	1	1	1	1	1
PPP1R14C	1	1	47.33767	1	1	1	1	1	1	38.97959	1	1	1	1	1	1	48.45371	1	1	103.6483
PPP1R16A	1	1	1	1	1	98.57602	1	1	1	1	1	1	48.32793	1	1	114.8137	1	1	1	70.02264
PPP1R17	1	1	1	357.9242	1	1	1	1	1	390.4503	1	295.3955	1	1	1	1	1	1	300.6422	1
PPP1R18	154.5626	1	1	58.81375	59.7143	1	1	62.85394	1	1	1	1	1	92.47544	49.88453	1	1	41.76204	89.21528	1
PPP1R1A	1	1	1	1	1	1	1	1	1	1	1	1	34.06926	1	1	1	1	1	1	70.02264
PPP1R1B	1	1	1	1	1	1	1	1	225.81	1	1	1	1	1	1	1	1	1	1	103.6483
PPP1R21	1	1	1	1	1	29.76096	1	1	1	35.92009	1	1	1	1	1	1	1	1	26.91201	1
PPP1R35	1	1	1	1	1	37.04159	1	1	1	1	1	55.09466	32.33367	1	1	1	1	1	1	1
PPP2R1B	1	1	1	49.62704	1	1	1	37.41899	1	29.05242	1	1	24.10886	1	1	1	1	26.52287	1	1
PPP2R2B	1	76.56924	259.1462	500.9416	447.5026	96.05239	440.4008	1	284.9438	133.9564	1	77.04466	1	1	184.247	79.66782	324.4505	1	319.0468	185.5228
PPP2R2D	1	75.39178	34.82742	58.54073	75.39177	44.0883	1	61.11559	1	70.62536	84.88523	68.08618	56.41884	90.65359	1	1	1	72.59132	1	1
PPP2R3A	1	1	33.04185	95.87554	109.1602	50.47134	1	1	1	31.70838	89.39162	36.02876	38.28766	81.82906	135.9322	1	355.7581	1	112.98	1
PPP2R3B	1	1	93.40999	1	1	1	1	1	1	35.1247	1	54.9357	28.04119	1	1	1	1	1	1	1
PPP2R3C	49.77491	85.65192	37.79153	53.2921	57.38156	58.61726	1	34.76118	1	74.61244	77.05554	96.15696	54.0926	55.53292	60.8973	61.64441	1	142.063	1	241.6936
PPP2R5A	1	46.96501	1	1	1	58.33967	1	32.2538	1	1	1	1	27.59213	1	1	1	1	44.02973	1	1
PPP6C	1	44.25264	29.28945	43.97843	40.32149	41.34883	1	43.56169	1	32.16706	1	47.42825	40.25354	88.98036	1	1	1	37.9976	62.89125	70.02264
PPP6R3	1	51.64012	1	55.91863	44.35992	42.01109	1	37.14658	1	40.936	1	61.54468	48.81424	1	39.70763	83.0794	1	25.80025	129.7366	1
PPRC1	1	1	1	1	1	1	1	39.13214	1	35.77964	1	1	1	1	1	1	1	1	1	1
PPWD1	1	1	1	1	1	1	1	1	1	29.86517	1	25.10092	1	1	1	1	1	14.21268	1	1
PRADC1	1	1	1	1	1	1	1	1	1	1	1	34.69028	1	1	1	1	1	1	1	1
PRAG1	1	50.02288	1	120.072	88.59742	57.73731	1	1	1	89.51761	1	52.11213	78.38135	1	1	54.58231	1	1	81.4325	103.6483
PRC1	1	1	1	1	1	1	1	1	1	34.59319	1	141.2136	1	1	1	1	1	1	1	1
PRCP	1	46.1779	1	1	1	42.40019	1	33.73765	1	1	1	1	31.46231	1	1	1	1	93.97293	1	1
PRDM1	115.5734	1	1	1	1	1	1	78.13942	1	1	1	1	1	1	1	1	1	164.3707	1	1
PRDM16	1	1	1	47.46547	1	125.3289	1	1	1	72.39415	1	67.06885	71.87201	1	1	65.41096	1	1	1	1
PRDM4	1	1	1	1	1	1	1	1	1	1	1	29.62902	1	1	1	1	1	1	1	1
PRDM8	1	1	1	107.8285	110.8849	1	147.5911	1	222.4964	1	1	1	1	1	110.1093	1	95.22692	1	99.4461	206.2967
PRDX4	82.57056	155.002	174.9348	69.18228	83.91946	148.228	1	140.9746	1	139.7938	1	107.7311	144.473	1	103.8603	214.3907	1	47.63671	79.19661	172.671
PRELID3A	1	1	1	1	1	1	1	1	1	1	1	1	1	1	50.19632	1	1	1	1	1
PRELP	1	1	1	1	1	1	1	448.9333	1	1	1	1	1	1	1	1	1	1	1	1
PREX1	78.25658	87.52645	49.16115	93.8486	65.06639	58.57506	1	1	1	51.35237	1	40.49252	1	1	67.79858	94.57199	1	367.983	139.3551	103.6483
PREX2	1	30.06261	58.48424	1	1	185.6089	1	54.01673	1	1	1	47.87314	1	1	1	64.31379	1	217.1451	1	1
PRH1	1	1	74.74778	83.72559	72.56126	34.36177	1	1	121.9595	43.85275	85.75434	38.94927	27.77828	79.66796	125.7964	1	58.00843	1	100.7196	103.6483
PRICKLE2	1	1	1	1	1	1	1	1	199.9854	1	1	1	1	1	1	1	1	1	1	1
PRIM1	1	39.91824	65.63561	1	1	1	1	1	1	66.64943	1	70.2732	30.79547	1	45.53655	65.62838	1	1	54.0816	1
PRIM2	1	1	1	1	1	1	1	1	1	53.63196	1	44.56548	1	1	1	1	1	1	1	1
PRKAA2	1	37.39991	1	1	1	30.37132	1	1	1	1	1	1	25.04035	1	1	1	1	1	1	1
PRKAB2	1	42.25875	32.19789	1	39.12436	32.5702	1	1	1	1	1	31.12315	1	1	46.26508	1	127.7044	1	1	172.671
PRKACA	56.67479	1	1	1	1	29.24373	1	50.02412	1	39.46304	1	32.57786	28.2407	1	62.50141	1	1	21.54436	30.13653	103.6483
PRKAG2	1	1	1	53.53051	45.9184	48.82192	1	1	79.00614	1	1	1	30.17484	1	51.17846	1	100.3164	1	63.9261	1
PRKAR1B	1	1	38.21632	1	1	29.38278	1	1	1	1	1	1	28.20593	1	1	1	1	1	1	1
PRKCA	42.09848	74.15086	125.6448	1	1	48.53961	1	53.00977	164.4393	1	498.247	29.58514	25.00149	78.51129	1	48.04273	1	1	1	87.58009
PRKCB	1	1	1	1	1	1	1	108.5486	197.7546	1	1	1	1	1	1	1	1	55.22766	1	1
PRKCE	1	1	1	1	1	1	1	1	212.9246	1	1	1	1	1	1	1	1	103.1697	1	116.5001
PRKCH	1	1	1	1	1	1	1	1	1	1	1	1	1	1	1	1	1	182.0092	1	1
PRKCZ	1	1	38.0699	1	41.27918	1	1	1	1	1	1	1	1	1	39.21424	1	82.42292	1	1	70.02264
PRKG1	1	1	1	1	1	58.56464	1	291.529	104.3145	41.29325	1	28.08721	38.33522	1	1	1	1	1	1	1
PRKRIP1	1	43.35035	1	54.99701	56.87837	50.20294	1	1	1	41.83138	60.35823	42.15916	41.07511	66.38022	1	1	1	27.87011	100.4657	1
PRKX	1	1	1	166.1969	133.9038	1	121.9138	28.20858	1	68.24876	143.9304	41.09463	1	121.1284	112.2333	1	1	211.3366	154.8831	206.2967
PRMT5	1	30.29918	30.52665	1	1	33.52385	1	31.11972	1	35.89155	1	49.03398	31.61068	1	1	44.24037	1	1	73.71086	1
PROCR	1	1	1	1	1	1	1	1	1	1	1	1	1	1	1	1	1	208.8037	1	1
PROS1	1	1	1	1	1	1	1	62.29184	1	1	1	1	1	1	1	1	1	91.53484	1	1
PROSER3	1	1	1	1	1	1	1	1	1	1	1	1	30.03142	1	1	1	1	1	1	1
PROX1	1	79.513	33.07413	1	1	1	1	1	1	1	515.9996	1	1	1	1	1	1	1	1	1
PRPF18	1	1	27.05815	1	1	1	1	1	1	39.41502	1	29.9308	1	1	1	1	1	11.89539	1	1
PRPF19	1	50.92311	41.73937	1	1	24.17266	1	48.55445	1	61.03695	1	53.72668	29.52325	1	69.24189	1	1	20.58701	1	1
PRPF3	1	32.93283	1	46.16321	39.19825	28.66494	1	1	1	39.87619	1	24.64888	30.25273	1	1	1	1	16.92973	1	1
PRPF39	1	48.27664	39.63725	49.3177	51.39971	24.4616	1	1	50.22687	64.45256	73.65738	36.97879	27.93604	1	1	26.2109	1	1	1	103.6483
PRPS2	1	1	1	1	1	1	1	1	1	1	1	29.96246	1	1	1	1	1	1	1	1
PRR11	1	1	1	1	1	1	1	1	1	1	1	131.6181	1	1	1	1	1	1	1	1
PRR13	118.0832	1	1	1	1	32.32836	1	40.65178	1	1	1	31.80451	35.21472	1	1	1	1	12.39039	1	1
PRR14	1	1	34.36018	1	1	29.0379	1	1	1	29.84222	1	25.94599	23.09732	1	48.71092	1	1	1	1	1
PRR14L	1	44.61625	1	84.96494	66.85931	44.27613	1	31.41696	1	77.51938	63.28552	34.82786	46.05567	64.86331	1	1	1	29.0665	115.7592	70.02264
PRR36	1	1	1	65.30486	72.49403	1	1	1	83.91766	33.11338	1	24.10488	1	1	79.39783	1	72.78664	1	77.81085	139.0453
PRR5	1	1	1	1	1	1	1	76.582	1	1	1	1	1	1	1	1	1	1	1	70.02264
PRR7	1	1	1	1	47.0748	30.78784	1	1	140.3572	1	1	1	71.10865	1	1	1	1	15.04052	1	1
PRRX1	1	1	1	1	1	1	1	73.19836	1	1	1	1	1	1	1	1	1	1	1	1
PRSS12	1	1	1	152.545	264.2247	1	249.2425	1	1	1	1	1	1	1	250.0345	1	255.1548	73.35412	192.5528	208.0679
PRSS23	1	1	1	1	1	1	1	105.2983	1	1	1	1	35.09426	1	1	1	1	1	1	103.6483
PRSS35	1	1	1	1	1	53.69965	1	1	1	1	1	1	49.34906	1	1	1	1	1	1	1
PRTFDC1	1	73.52044	69.60451	1	1	80.81037	1	1	1	73.50622	1	87.93024	65.36458	1	1	69.77145	1	1	1	1
PRUNE2	1	1	1	1	1	29.22089	1	1	1	1	1	1	1	1	1	1	1	1	1	1
PSAT1	1	257.8412	162.5367	1	1	612.3978	1	1	1	90.58334	1	185.8096	330.5019	1	1	393.322	1	21.50425	1	70.02264
PSENEN	61.93661	33.45265	1	1	1	46.78117	1	38.01386	1	1	1	40.60456	125.2742	1	1	1	1	22.29013	1	1
PSMC3IP	1	1	1	1	1	1	1	1	1	41.13615	1	48.75148	23.04524	1	1	1	1	1	1	1
PSMD10	1	37.15686	66.51711	1	1	50.65153	1	1	1	34.54609	1	53.71684	47.36966	1	62.07476	43.53853	1	1	1	1
PSMG1	1	1	1	1	1	1	1	1	1	1	1	1	1	1	1	37.26633	1	103.549	1	1

PSMG4	1	1	40.41264	47.63113	48.40779	33.87854	1	1	1	41.70436	1	1	24.94652	1	52.83816	1	242.9664	26.21354	110.4987	103.6483
PSPH	1	1	1	1	1	31.99741	1	1	1	1	1	1	24.78114	1	1	1	1	1	1	1
PSRC1	1	84.23792	1	1	1	143.6458	1	1	1	40.96553	1	189.0957	254.5684	1	1	167.3317	1	1	1	1
PTAFR	114.8801	1	1	1	1	1	1	1	1	1	1	1	1	1	1	1	1	1	1	1
PTAR1	1	139.2106	63.43589	73.65944	58.31131	102.2794	1	1	1	60.40231	1	88.19351	67.7217	1	1	56.42047	1	61.29012	1	1
PTBP3	63.62684	1	1	50.15127	50.34698	1	1	58.4723	1	1	99.73905	1	1	161.1349	61.72985	1	75.57699	51.0426	1	103.6483
PTCD2	1	1	1	1	1	1	1	1	1	1	1	1	1	1	1	1	1	1	1	70.02264
PTCH1	1	41.44883	35.39363	1	1	1	1	1	1	1	71.77698	1	1	1	1	1	1	1	1	70.02264
PTDSS2	1	40.07194	1	1	1	27.80982	1	1	1	1	1	1	23.61034	1	1	1	1	105.5496	1	1
PTGDR2	1	1	1	1	1	1	1	57.71268	1	1	1	1	1	1	1	1	1	1	1	1
PTGER4	71.62534	1	1	1	1	1	1	1	1	1	1	1	1	1	1	1	1	1	1	1
PTGES2	1	33.62639	42.9742	1	1	25.37667	1	44.76873	1	1	1	1	1	1	1	1	108.2791	39.69298	1	1
PTGFRN	1	1	1	1	1	77.19369	1	24.36118	1	33.85538	1	39.08727	41.6762	1	1	64.6812	1	1	1	1
PTGS2	1	1	1	1	1	1	1	113.4306	1	1	1	1	1	1	1	1	1	111.6963	1	1
PTH1R	1	1	1	1	1	1	1	76.7519	1	1	1	1	1	1	1	1	1	1	1	1
PTH2R	1	1	1	1	1	47.8296	1	1	1	1	1	1	1	1	1	1	1	1	1	1
PTK7	1	1	1	59.86087	1	33.02094	1	25.9652	69.37155	50.60989	1	42.21676	34.57076	1	1	1	1	1	99.5608	1
PTN	1	2680.002	1499.942	309.8209	229.0776	6741.946	213.2282	4181.67	268.8402	746.6685	1	2015.901	628.5491	1	291.9064	4211.113	223.0168	60.96853	233.6499	103.6483
PTP4A3	1	1	1	1	1	1	1	41.92849	1	1	1	1	1	1	1	1	1	135.1344	1	1
PTPN18	86.78141	1	1	1	1	1	1	29.97051	1	1	1	1	1	1	1	1	1	1	1	1
PTPN2	1	1	1	1	48.67457	29.25573	1	43.45369	1	33.14226	1	23.13557	34.99967	90.84613	67.91761	1	323.0883	1	1	139.0453
PTPN21	1	1	1	1	1	1	1	1	1	1	1	1	28.35556	1	1	1	1	1	1	1
PTPN23	1	1	1	1	1	1	1	1	1	1	1	1	1	1	49.11025	1	1	1	1	1
PTPN5	1	1	1	1	1	1	1	1	1	1	1	1	1	1	1	1	123.5434	1	1	1
PTPN6	67.05085	1	1	1	1	1	1	1	1	1	1	1	1	1	1	1	1	1	1	1
PTPRB	1	1	1	1	1	1	1	1	1	1	1	1	1	1	1	1	1	278.7248	1	1
PTPRC	167.675	1	1	1	1	1	1	1	1	1	1	1	1	1	1	1	1	1	1	1
PTPRD.AS1	1	1	1	1	1	1	1	1	1	1	1	1	1	1	1	1	1	1	1	172.671
PTPRE	90.60502	42.7777	157.7288	1	1	1	1	1	1	1	1	1	1	1	72.86318	1	91.49307	39.89479	1	139.0453
PTPRF	1	40.75925	40.78174	47.37283	50.96815	83.02596	1	1	94.60238	72.16389	1	62.25659	41.96254	1	48.81972	72.92594	188.882	1	61.28561	1
PTPRJ	50.55309	1	1	1	1	1	1	1	1	1	1	1	1	1	1	1	1	1	1	1
PTPRK	1	1	42.1146	58.19833	1	35.41393	1	202.0398	1	39.09895	1	53.59154	44.73167	1	1	1	1	62.19101	1	1
PTPRM	1	1	1	1	1	56.76775	1	49.60779	1	1	1	1	1	1	1	80.30675	1	73.54098	1	1
PTPRN2	76.96259	1	62.77573	1	1	47.94434	1	42.18619	1	1	80.95993	1	55.04278	71.19119	1	1	1	69.0384	1	1
PTPRO	1	1	106.4912	51.23441	63.92219	1	1	1	1	1	79.40371	1	1	84.03269	75.17038	1	189.1235	1	1	139.0453
PTPRR	1	1	1	1	1	1	1	1	1	1	62.35535	1	1	1	1	1	1	1	1	1
PTPRS	1	1	1	51.1226	54.38116	1	1	1	73.1129	30.56904	1	26.33569	1	1	58.71994	1	88.72705	1	1	70.02264
PTPRT	1	1	1	1	1	1	1	1	1	1	1	1	1	113.8259	1	1	1	1	1	1
PTTG1	1	67.4094	54.8178	1	1	35.17365	1	41.82057	1	61.21377	1	822.0148	93.23803	1	1	87.97792	1	23.90257	1	1
PTX3	1	71.1467	1	1	54.0692	63.02143	1	1	1	45.65434	1	69.81501	104.4027	1	1	61.204	1	1	1	1
PURG	1	1	1	1	1	1	1	1	70.24815	1	68.69805	1	1	87.2892	1	1	1	1	1	103.6483
PUS3	1	1	1	1	1	1	1	1	1	39.56632	1	1	1	1	1	1	1	1	1	1
PUS7	1	1	1	1	1	44.74851	1	1	1	1	1	1	1	1	1	1	1	1	1	1
PWAR6	1	38.57069	66.79291	121.423	126.8602	32.5388	1	1	174.6756	1	100.5773	1	24.93883	129.8073	126.4344	1	148.2157	1	55.46388	1
PXDC1	1	87.10665	139.7683	1	1	1	1	65.03812	1	1	1	1	56.68384	177.8788	1	1	1	66.03155	1	1
PXDN	1	1	1	1	1	61.91484	1	138.4115	1	1	1	26.83291	60.78745	63.97841	1	1	1	104.1959	1	1
PXMP2	1	60.31937	24.04432	1	1	62.21343	1	1	1	70.28737	1	78.24566	43.53874	1	1	92.71708	1	1	1	1
PXN	1	1	1	1	1	1	1	1	1	1	1	1	1	1	1	1	1	63.56574	1	1
PXYLP1	1	1	1	1	1	47.84257	1	30.19937	1	53.58235	1	29.08323	142.1874	73.74181	1	36.34407	1	1	1	1
PYCARD	330.9411	1	1	1	1	1	1	1	1	1	1	1	1	1	1	1	1	41.72126	1	1
PYGL	1	1	1	1	1	39.64042	1	1	1	1	1	1	1	1	1	1	1	1	1	1
QPCT	1	1	1	1	1	1	1	72.87295	1	1	1	1	1	1	1	1	1	105.8655	1	1
QPRT	106.3511	1	1	1	1	42.4835	1	108.9874	1	1	1	1	1	1	1	1	1	1	1	1
QRSL1	1	52.38362	20.84273	1	1	27.14383	1	1	1	37.51893	1	33.50784	30.66697	1	1	1	1	1	1	1
QSOX2	1	1	1	1	1	1	1	1	1	50.69973	1	26.70019	1	1	1	1	1	11.98621	1	103.6483
QTRT2	1	1	1	1	1	1	1	1	1	32.86912	1	27.70004	33.71885	1	1	1	1	1	1	1
R3HDM1	1	41.48921	26.49553	45.2739	69.97705	35.56767	1	1	94.28235	48.95853	1	54.30321	34.11493	1	120.0139	1	399.5957	52.41891	1	139.0453
RAB11FIP4	1	1	1	1	1	1	1	1	58.54185	1	1	1	1	1	1	1	1	1	1	1
RAB12	1	1	1	81.94068	88.59632	29.00812	1	16.76812	1	31.71679	1	24.12929	28.28632	1	71.90368	1	1	27.25843	104.2065	1
RAB13	109.358	143.3432	1	1	1	36.70159	1	119.1956	1	78.67741	1	95.33373	78.07533	1	1	61.05009	1	183.2812	1	1
RAB20	100.667	1	1	1	1	1	1	31.91221	1	1	1	1	1	1	1	1	1	1	1	1
RAB22A	1	63.01566	78.76913	48.58397	45.91977	70.65739	1	52.6699	49.39788	49.25678	1	56.82444	52.31647	53.37002	1	1	1	22.29978	34.83532	103.6483
RAB30.AS1	1	51.05522	41.62674	52.17496	40.60314	51.26363	1	1	1	46.15706	1	60.26533	47.77635	1	1	72.66592	1	15.0231	110.3209	70.02264
RAB31	123.4468	256.2367	380.4246	1	1	132.7436	1	77.67879	1	1	1	46.88813	54.59128	1	1	86.26398	1	1	1	1
RAB32	291.2928	1	1	1	1	39.33314	1	43.32644	1	47.90373	1	38.96317	70.04694	1	1	39.10594	1	69.09152	1	1
RAB33A	1	1	140.5351	45.66076	66.1165	1	1	1	1	1	78.51824	1	1	1	105.3539	1	1	24.73621	111.6645	1
RAB33B	1	1	1	1	45.58972	1	1	1	1	1	1	1	29.60052	1	1	1	1	47.96386	1	116.5001
RAB39B	1	1	33.78845	1	1	1	1	1	115.8956	1	1	1	1	1	1	1	1	1	1	1
RAB3A	1	1	1	109.4514	149.1532	1	175.1386	1	138.4779	1	98.35626	1	1	122.8654	106.5461	1	99.90188	1	101.6322	116.5001
RAB3IL1	52.20131	1	1	1	1	1	1	1	1	1	1	1	1	1	1	1	1	1	1	1
RAB3IP	1	91.04087	156.2865	1	1	1	1	1	55.56803	1	142.4706	1	1	82.75421	1	1	1	1	1	70.02264
RAB40B	1	1	1	1	1	40.39473	1	1	1	1	1	1	29.87232	1	1	1	1	1	1	1
RABEP1	159.9721	61.36298	104.5729	79.47818	86.57413	58.47389	1	72.20893	102.3137	66.62231	119.809	54.37762	40.68874	128.8618	170.2157	66.00881	176.1782	36.28298	70.06205	323.568
RABEPK	1	36.93811	1	1	1	1	1	1	1	1	1	1	1	1	1	1	1	1	1	1
RABGAP1	1	64.8144	68.02927	111.2267	114.2021	88.29535	1	84.43786	114.6235	85.66473	104.0049	73.69025	66.5376	129.1633	122.6017	85.48276	161.8378	58.12215	134.5044	232.0002
RABGAP1L	1	33.61851	111.8655	1	1	34.28505	1	29.29482	191.6169	1	63.4365	1	1	53.46932	1	1	1	21.83836	50.90398	1
RABIF	1	1	1	1	1	1	1	1	1	1	1	1	1	1	56.02846	1	1	12.35707	1	1
RABL2A	1	1	1	1	1	1	1	1	1	1	1	1	32.49646	1	1	1	1	1	1	1
RABL2B	1	1	1	1	1	1	1	1	1	1	1	1	51.53935	1	1	1	113.3313	1	1	1
RABL3	1	1	1	1	1	26.712	1	1	1	1	1	1	1	1	1	1	1	1	1	1
RABL6	35.08928	69.95796	77.91605	48.52773	54.23276	62.41946	1	80.92976	86.37519	95.7726	72.64334	76.18757	51.49841	1	54.65336	46.16701	1	136.3702	1	139.0453
RAC2	64.0246	1	1	1	1	1	1	1	1	1	1	1	1	1	1	1	1	1	1	1
RAC3	1	45.19031	127.8379	57.38854	98.03635	70.60586	159.808	1	186.9634	78.34573	1	89.03843	43.32166	66.10029	155.4269	66.97275	323.2515	1	67.6125	292.8767
RACGAP1	1	1	1	1	1	1	1	1	1	25.86013	1	204.4197	1	1	1	1	1	1	1	1
RAD17	1	47.96593	1	51.50618	1	42.2328	1	26.27607	1	45.16999	1	43.9315	30.86525	1	1	1	1	123.7303	1	1
RAD18	1	1	1	1	1	1	1	1	1	59.64025	1	65.87963	1	1	1	65.09468	1	1	1	1
RAD51AP1	1	1	1	1	1	1	1	1	1	64.95864	1	150.8802	1	1	1	1	1	1	1	70.02264
RAD51C	1	33.92275	30.862	1	1	40.65046	1	1	1	53.65056	1	54.5295	35.38548	1	1	1	56.99662	110.9197	1	1
RAD51D	1	1	1	1	1	1	1	1	1	31.14906	1	1	1	1	1	1	1	1	1	1
RAD54B	1	1	1	1	1	1	1	1	1	35.11637	1	25.61634	1	1	1	1	1	1	1	1
RAE1	1	33.47092	33.61201	50.85098	56.82547	46.62001	1	26.18951	1	41.25674	60.10539	56.56259	48.50685	80.69955	1	1	1	21.87592	1	1
RAI1	1	1	1	43.55397	45.75551	1	1	1	1	1	1	25.95339	1	63.68018	1	1	1	29.99967	1	70.02264
RAI14	1	1	1	54.37778	77.69069	97.08536	1	29.59156	1	68.0502	1	66.15867	54.61611	1	124.967	64.01752	74.62497	129.0818	1	1
RALB	1	1	1	1	1	27.88022	1	62.79561	1	1	1	1	1	1	1	1	1	102.6416	1	70.02264
RALGPS1	1	1	1	46.98455	65.01973	24.61183	1	1	1	1	1	27.46997	1	59.26682	69.84842	1	75.57424	1	1	70.02264
RALYL	1	1	101.8224	1	1	1	1	1	273.0139	1	1	1	24.62537	1	1	1	130.9775	1	1	139.0453
RAMP1	1	123.7517	280.348	1	1	1	1	1	1	1	1	1	31.35031	1	1	1	1	1	1	1
RAMP2	1	1	33.89249	1	1	1	1	28.1435	1	1	1	1	1	1	1	1	1	851.6438	1	1
RANBP17	1	1	49.7005	1	1	1	1	1	60.92126	1	1	1	1	1	1	1	1	18.40479	1	1
RANBP3	1	1	1	1	1	26.91117	1	1	1	26.95913	1	36.43629	28.61664	1	1	1	1	1	79.136	1
RANBP6	1	42.20681	39.95524	61.78952	69.41828	32.23681	1	1	64.7578	57.33372	90.45442	43.75744	37.20827	88.19545	1	1	1	1	71.16318	1

RANBP9	66.22017	65.72415	1	71.22124	75.68334	52.42219	1	74.1589	1	68.24094	1	51.96399	68.26834	58.01088	81.19264	65.19779	1	72.6696	74.92029	185.5228
RANGAP1	1	1	1	1	1	1	1	1	1	1	1	58.53564	1	1	1	1	1	1	1	1
RAP1GAP	1	1	51.67567	1	1	1	1	1	1	1	1	1	1	1	1	1	1	1	1	1
RAPGEF1	66.81905	1	70.52959	1	1	1	1	1	132.2824	1	1	29.29605	1	1	1	1	1	38.96308	1	1
RAPGEF5	1	1	1	1	1	1	1	81.88374	187.1271	1	1	1	1	1	1	1	1	330.4839	1	1
RAPH1	1	1	23.65102	1	1	1	1	124.3503	148.0688	1	1	26.27582	1	74.86314	1	1	1	46.572	1	1
RARA	1	1	30.87167	1	1	1	1	1	1	1	77.81279	1	1	87.89872	1	1	1	1	1	1
RARRES2	1	185.5137	104.9239	1	1	95.0549	1	1	1	38.18234	1	84.73387	258.035	1	1	92.53895	1	1	1	1
RARS2	1	1	1	1	38.846	38.16081	1	28.24491	59.83138	38.59958	56.69682	29.84201	22.12123	1	1	1	1	34.57884	1	1
RASA4	1	1	1	106.9611	57.66438	35.0708	1	1	134.3348	56.05918	1	25.63084	39.94395	1	1	1	1	1	1	1
RASD1	1	76.70805	1	97.19972	105.3574	1	1	51.46763	1	77.58968	1	1	127.3684	1	1	1	1	1	52.89141	70.02264
RASGEF1B	141.5417	1	35.26471	163.9624	118.5309	1	1	1	1	51.65742	1	32.92614	1	1	1	1	1	1	83.65659	1
RASGRP1	1	40.83124	1	114.7596	1	39.90009	1	1	1	168.5052	1	77.0398	1	1	1	1	1	1	1	1
RASGRP2	1	1	1	1	1	1	1	125.8028	1	1	1	1	1	1	1	1	1	1	1	1
RASGRP3	1	1	1	1	1	1	1	1	1	1	1	1	1	1	1	1	1	282.5265	1	1
RASIP1	1	1	1	1	1	1	1	23.50771	1	1	1	1	1	1	1	1	1	166.0451	1	1
RASL10A	1	1	1	1	1	1	1	1	95.55165	1	1	1	1	1	1	1	211.685	1	1	346.1132
RASL12	1	1	1	1	1	1	1	260.9621	1	1	1	1	1	1	1	1	1	1	1	1
RASSF3	1	1	1	1	1	1	1	1	1	1	1	1	1	1	1	1	1	54.55097	1	1
RASSF4	79.27823	1	1	1	1	1	1	1	1	1	1	1	1	1	1	1	1	1	1	1
RASSF8	1	1	1	1	1	24.73776	1	58.37973	1	1	1	37.00292	59.85775	1	1	1	1	153.4834	1	1
RBAK	1	1	1	1	1	1	1	1	1	1	1	1	23.23673	1	1	1	1	32.46356	65.72211	1
RBAK.RBAK	1	1	1	1	1	30.62352	1	1	1	1	1	1	1	1	1	1	1	1	1	1
RBBP5	1	42.44089	1	43.87163	1	1	1	1	1	46.52337	56.37178	39.71926	26.41098	1	1	1	1	1	1	1
RBBP8	1	43.91357	1	1	1	1	1	1	1	62.20449	1	81.18135	33.59944	1	1	1	1	99.07195	1	1
RBBP9	1	1	1	1	1	31.35072	1	1	1	1	1	31.43222	1	1	1	1	1	1	1	1
RBCK1	36.99289	42.06033	1	1	1	41.54409	1	48.14532	1	32.96881	1	35.99879	46.31743	1	1	72.36051	1	38.30663	1	1
RBFOX1	1	1	1	125.9378	248.7722	1	227.1646	1	313.0003	1	1	1	1	1	354.3241	1	445.9429	1	242.9534	70.02264
RBFOX3	1	1	1	102.3862	79.65845	1	1	1	184.9706	1	1	1	1	1	94.24391	1	1	1	44.93409	206.2967
RBL1	1	37.05375	1	1	1	28.2613	1	1	1	75.29405	1	60.55702	25.45476	1	1	1	1	1	1	1
RBM15	1	1	1	1	1	1	1	1	1	1	1	30.9459	1	1	1	1	1	1	1	116.5001
RBM27	1	1	1	1	1	23.50167	1	1	1	40.02244	1	30.01121	38.00989	1	1	1	1	25.95901	58.66739	1
RBM38	1	1	1	1	1	1	1	1	1	35.95089	1	1	45.5134	1	1	1	1	1	1	103.6483
RBM41	1	1	1	1	1	1	1	1	1	48.05316	1	39.99641	25.7071	1	1	1	1	1	1	1
RBM47	63.28061	1	1	1	1	1	1	1	1	1	1	1	1	1	1	1	1	1	1	1
RBM48	1	37.36647	1	1	1	1	1	1	1	1	1	26.08406	1	1	1	1	1	26.1494	1	1
RBMS1	36.57287	1	1	1	1	1	1	308.6708	1	1	1	1	1	1	1	1	1	95.95691	1	1
RBMS2	1	1	1	1	1	1	1	73.95482	1	1	1	1	1	1	1	1	1	144.1583	1	1
RBMS3	1	1	1	1	1	1	1	206.1614	1	1	1	1	1	1	1	1	1	36.45228	1	1
RBMX2	1	54.23652	93.813	70.38027	61.93232	53.76672	1	30.57247	1	52.78252	1	72.50611	57.21651	1	52.88773	66.54817	1	112.6831	33.58645	172.671
RBMXL1	1	64.62745	46.70655	1	40.88699	42.82734	1	33.45082	87.65283	47.42188	69.07967	42.72681	46.54431	1	61.33809	1	1	14.34445	1	1
RBP1	1	149.8662	262.4419	111.9563	51.86752	1	1	517.3844	1	262.9252	397.5298	77.05378	92.71531	980.3229	1	1	1	546.6326	106.0466	1
RBPMS	1	1	1	1	1	1	1	111.8344	1	1	1	1	1	1	1	1	1	31.37612	1	1
RBSN	1	46.7182	56.88151	54.12297	49.92879	39.74333	1	1	1	36.26612	63.39332	40.95058	34.72689	77.54513	35.81439	1	1	1	1	1
RCC1	1	1	1	1	1	1	1	1	1	29.4901	1	50.59123	1	1	1	1	1	1	1	1
RCHY1	1	44.93192	1	43.30662	43.57596	33.39477	1	1	1	1	70.98273	35.99525	35.12899	58.50452	1	1	1	21.71148	1	139.0453
RCN1	1	131.6333	86.90601	1	1	200.8899	1	53.24852	1	105.1452	1	101.1608	145.8094	1	1	201.8734	1	34.95704	1	1
RCOR2	1	1	39.85542	74.28891	47.64971	1	1	1	1	53.84128	1	1	1	75.71447	1	1	1	1	57.11469	1
RCSD1	65.5938	1	1	1	1	1	1	197.9532	1	1	1	1	1	1	1	1	1	79.49172	1	1
RDH10	1	1	1	1	1	1	1	97.48044	1	1	1	1	1	1	1	1	1	1	1	1
RECQL	1	55.39295	1	1	1	46.0799	1	57.00477	1	78.21596	61.92332	51.25836	34.02304	1	1	1	1	52.1897	1	70.02264
RECQL4	1	1	1	1	1	1	1	1	1	1	1	31.4737	1	1	1	1	1	1	1	1
REEP1	1	1	1	63.93059	98.29094	48.9447	170.3716	1	223.4106	54.17529	96.97242	35.54839	36.10147	102.5178	150.3228	118.0401	414.5873	1	71.11707	344.3419
RELA	1	52.98507	1	1	42.1812	46.76648	1	50.51459	1	47.29082	1	39.95063	48.78868	1	1	1	1	44.80003	1	172.671
RELL1	1	1	1	1	1	1	1	1	1	1	1	1	1	1	1	1	1	83.77133	1	1
RELT	52.58286	1	1	1	1	1	1	1	1	1	1	24.67385	1	1	1	1	1	18.16198	1	1
RERGL	1	1	1	1	1	1	1	80.64092	1	1	1	1	1	1	1	1	1	1	1	1
RETREG1	1	1	1	48.05295	64.25616	111.6632	1	1	166.3732	1	1	1	92.13581	1	1	1	91.0889	1	1	103.6483
REXO2	1	83.98747	1	1	1	182.8129	1	69.06639	1	78.82652	1	68.26846	63.42239	1	1	126.2706	1	59.46114	1	1
REXO5	1	1	1	1	1	1	1	1	1	1	1	26.90673	1	1	1	1	1	1	1	1
RFC2	1	61.06551	1	1	1	33.90232	1	37.2812	1	77.87807	1	75.84841	37.54433	1	1	69.92206	1	1	1	1
RFC3	1	1	1	1	1	1	1	1	1	58.49452	1	65.35545	1	1	1	96.99739	1	1	1	1
RFC4	1	1	1	1	1	1	1	1	1	103.1514	1	84.96128	33.2625	1	1	34.6358	1	1	1	1
RFC5	1	32.12734	1	1	1	1	1	1	1	49.83255	1	42.24927	23.65599	1	1	58.41667	1	1	1	1
RFFL	1	44.41289	40.64339	1	1	23.62744	1	1	1	29.99623	1	29.57844	1	1	1	1	1	1	1	1
RFLNB	1	1	1	1	1	1	1	1	86.8532	1	1	1	1	1	1	1	1	124.2971	1	70.02264
RFPL1S	1	1	1	1	42.18894	1	1	1	113.9946	1	1	1	1	1	1	1	1	1	1	1
RFTN1	1	1	1	1	1	1	1	243.8941	1	1	1	1	1	1	1	1	1	1	1	1
RFTN2	1	135.1098	146.8878	1	1	122.1982	1	1	1	69.15492	1	48.07915	93.56537	1	1	56.14198	1	23.40537	1	1
RFWD3	1	1	1	1	1	1	1	1	1	39.63222	1	43.47158	1	1	1	1	1	1	1	1
RFX1	1	1	1	1	1	1	1	54.75224	55.11795	1	1	1	27.47257	1	1	1	1	1	1	1
RFX2	1	1	1	1	1	1	1	1	1	1	1	23.4494	57.98315	1	1	1	1	1	1	1
RFX4	1	194.8315	1	1	1	88.44529	1	1	1	1	1	31.62464	110.2221	1	1	66.21841	1	1	1	1
RFX7	1	51.57309	34.5932	73.60872	75.1947	32.68002	1	1	110.2606	57.94205	71.93744	40.98517	33.517	78.79701	70.49854	1	1	26.18226	1	103.6483
RFXANK	1	65.61825	1	1	1	96.82812	1	164.5567	1	49.24231	1	40.88081	57.73633	1	1	86.52392	1	55.27962	1	1
RFXAP	1	1	32.62331	1	40.35281	1	1	28.26114	1	1	1	1	33.20479	1	1	1	1	1	1	1
RGCC	251.4515	144.0899	121.437	1	1	160.2268	1	1	1	1	1	38.58814	59.12733	1	1	86.2354	1	1445.296	1	1
RGL1	62.67371	33.40499	1	1	50.38027	52.67566	1	75.09714	1	27.29697	1	29.2836	34.31633	1	87.94642	1	1	83.58362	1	149.721
RGMA	1	147.3312	1	1	1	126.1049	1	1	1	32.07309	1	56.51537	86.90887	1	1	74.04535	1	1	1	1
RGMB	1	1	63.21013	1	1	1	1	1	1	1	67.8991	1	33.84724	105.8404	1	1	1	1	1	1
RGN	1	1	1	1	1	36.57237	1	1	1	1	1	1	1	1	1	1	1	1	1	1
RGS1	826.2318	1	1	1	1	1	1	1	1	1	1	1	1	1	1	1	1	1	1	1
RGS10	1538.085	1	1	1	1	1	1	1	1	1	1	1	1	1	1	1	1	1	1	1
RGS16	1	73.93562	133.6606	1	1	249.9449	1	1198.636	1	93.96437	1	69.69228	60.61263	1	1	1	1	1	1	1
RGS17	1	1	66.6245	67.31706	89.63125	28.20079	1	1	418.9199	1	83.57518	1	1	106.743	103.3106	1	93.04602	35.40355	68.68149	70.02264
RGS19	166.9111	1	1	1	1	1	1	1	1	1	1	1	1	1	1	1	1	36.64575	1	1
RGS2	79.48334	1	62.72662	1	54.05719	1	1	53.76773	1	35.74864	1	33.33878	1	65.38787	1	1	1	1	1	1
RGS20	1	57.34569	1	1	1	123.8411	1	1	1	1	1	49.5664	1	1	1	34.98118	1	1	1	1
RGS22	1	1	1	1	1	1	1	1	1	1	1	1	38.58269	1	1	1	1	1	1	1
RGS3	1	53.29121	1	1	1	31.79205	1	32.77739	1	1	1	34.77915	29.32074	1	1	1	1	161.2986	1	1
RGS5	1	1	1	1	1	1	1	2257.592	1	1	1	1	1	1	1	1	1	49.4627	1	1
RGS7	1	1	1	1	1	1	1	1	231.6834	1	1	1	1	1	1	1	1	1	1	1
RHBDL2	1	1	1	1	1	1	1	59.47944	1	1	1	1	1	1	1	1	1	1	1	1
RHNO1	1	1	1	1	1	34.8299	1	1	1	26.58341	1	75.7023	1	1	1	1	1	1	1	1
RHOC	1	84.62136	62.15645	1	1	169.4548	1	249.5846	1	33.25309	1	61.6454	110.1376	1	1	130.8813	1	351.0906	1	1
RHOG	190.3025	1	1	1	1	28.32991	1	37.66952	1	1	1	1	23.83106	1	1	1	1	38.79963	1	1
RHOJ	1	89.66706	1	1	1	55.2277	1	125.4273	1	35.69544	1	1	1	1	1	1	1	184.4208	1	1
RHOU	1	1	1	1	1	1	1	1	1	1	1	1	1	1	1	1	92.37251	1	1	1
RHPN1	1	1	1	1	1	32.79088	1	1	1	1	1	1	45.64259	1	1	1	1	18.65654	1	1
RIC3	1	1	132.3124	1	43.91497	1	1	1	100.821	1	1	1	1	1	70.6804	1	1	1	1	1
RIC8B	1	42.66431	1	1	1	1	1	1	1	1	1	36.06214	30.70128	68.94037	1	1	1	1	69.80038	1

RIDA	1	68.9973	65.60138	1	1	90.18749	1	1	1	49.39935	1	40.72573	61.8493	1	1	106.3298	1	16.37148	1	1
RILPL1	1	43.5432	1	1	1	35.59441	1	38.45419	1	1	1	1	28.93181	1	1	1	1	33.74931	1	1
RILPL2	199.2634	1	1	1	1	1	1	59.20039	1	1	1	1	26.39563	1	1	1	1	122.3321	1	1
RIMBP2	1	1	1	1	1	1	1	1	1	1	1	1	1	1	1	1	143.9938	1	66.54483	1
RIMKLA	1	1	1	1	1	1	1	1	1	1	113.4161	1	1	61.16955	60.32846	1	1	1	1	1
RIMS1	1	1	59.91876	1	1	1	1	1	127.154	1	75.45102	24.84756	1	114.0319	1	1	92.67176	1	1	1
RIMS2	1	1	83.09758	1	1	1	1	1	277.7672	1	1	1	1	65.32452	1	1	1	1	1	1
RIN2	189.2237	43.52328	62.45221	1	1	26.0468	1	1	1	1	1	1	1	1	1	1	1	1	1	1
RING1	1	47.4634	31.23824	1	1	39.91269	1	41.12185	1	27.90472	1	1	31.0499	1	1	1	1	15.75429	41.45079	70.02264
RIOX2	1	1	1	1	1	38.76985	1	1	1	1	1	1	44.8793	1	1	1	1	1	1	1
RIPOR1	1	1	1	1	1	1	1	30.45699	80.44241	1	1	1	1	1	1	1	1	77.26468	1	1
RIPOR2	1	1	1	1	1	1	1	1	1	1	512.7396	1	1	339.6536	1	1	1	1	1	1
RIT2	1	1	104.5788	1	1	1	1	1	1	1	1	1	1	1	1	1	1	1	1	1
RITA1	1	1	1	1	1	1	1	1	1	1	1	1	1	1	1	1	1	115.1522	1	1
RMI1	1	1	1	1	1	32.29319	1	1	1	70.10917	1	65.77428	27.47303	1	1	49.53216	1	1	34.91052	1
RMI2	1	1	1	1	1	1	1	1	1	37.303	1	25.6282	1	1	1	1	1	1	1	1
RMND1	1	1	1	1	1	26.4752	1	1	1	35.73451	1	22.42489	23.14152	1	1	1	161.6425	1	1	1
RNASE1	1	1	1	1	1	1	1	1	1	1	1	1	1	1	1	1	1	279.5478	1	1
RNASET2	544.0729	1	1	1	1	1	1	29.4624	1	1	1	1	1	1	1	1	1	1	1	1
RND1	1	1	1	1	1	43.50521	1	1	1	1	1	1	42.69145	1	1	1	1	1	1	1
RND2	1	33.01361	154.6049	1	1	1	1	1	1	104.0021	1	1	37.2958	1	1	1	1	1	1	1
RNF103	1	30.33258	1	60.75719	58.55801	35.43539	1	40.49639	1	48.75029	1	41.37132	40.8787	1	1	1	1	23.09713	101.3998	1
RNF111	1	1	1	1	40.1132	1	1	1	1	1	1	33.78906	27.21192	1	44.73855	1	1	35.54002	1	103.6483
RNF114	84.05654	54.46954	47.06697	58.34554	60.60814	43.16177	1	52.45379	70.08895	66.86255	76.56293	71.04933	56.02677	1	85.79112	1	1	66.61242	64.17826	241.6936
RNF121	1	1	26.74777	1	1	1	1	1	1	35.26695	1	1	1	1	1	1	1	1	1	70.02264
RNF125	1	1	39.94568	1	1	1	1	1	1	1	1	1	1	1	1	1	1	1	1	1
RNF139	1	75.37807	73.32482	77.4576	87.82923	70.30462	1	65.11616	1	66.41815	88.22119	67.93526	83.75027	97.40599	1	31.42015	1	34.66689	1	103.6483
RNF14	1	1	1	1	1	1	1	24.98511	1	41.23991	1	26.89165	1	1	45.67008	1	1	28.05923	41.95046	70.02264
RNF141	1	46.60317	63.78379	1	1	65.80717	1	1	1	42.84406	1	41.8563	48.63828	1	1	80.6231	1	35.13955	1	1
RNF144A	1	1	116.8042	67.24172	55.62973	1	1	1	1	1	1	1	1	1	58.07634	1	1	38.76656	72.76194	103.6483
RNF149	58.30765	1	1	1	1	27.27819	1	1	1	1	1	1	26.87749	1	1	1	108.6686	1	1	1
RNF150	1	1	1	1	44.01869	1	1	1	1	1	1	1	1	1	62.59465	1	1	1	1	1
RNF152	1	1	1	1	1	1	1	170.1402	1	1	1	1	1	1	1	1	1	1	1	1
RNF157	1	64.04108	84.9705	1	1	1	1	1	1	33.31707	1	34.09088	1	1	65.73565	1	1	1	1	70.02264
RNF165	1	65.87558	72.88022	182.1471	181.9963	1	191.4105	1	229.5306	150.4927	162.8902	65.00757	1	209.2565	223.7093	1	260.2361	1	128.0287	415.1358
RNF166	49.3249	1	1	1	1	1	1	1	1	1	1	1	1	1	1	1	1	33.58104	1	1
RNF170	1	1	1	1	1	32.56769	1	35.45801	1	1	1	1	1	1	1	1	1	1	1	1
RNF175	1	1	34.84899	1	1	32.76968	1	1	1	1	1	1	1	1	1	1	1	1	1	1
RNF180	1	98.52868	33.29644	42.10178	39.14268	125.697	1	26.70134	75.26359	59.30208	170.4619	54.49897	55.02507	1	1	74.00683	1	1	1	1
RNF182	1	1	1	61.08369	160.5314	1	193.7132	1	1	1	1	26.55546	36.4735	1	279.9892	1	76.89319	1	133.9972	275.3193
RNF185	1	1	1	1	1	28.9736	1	1	1	1	1	1	1	1	1	1	1	1	1	1
RNF213	117.1658	38.88329	1	1	1	59.1066	1	88.6945	1	1	1	1	36.18465	1	1	1	1	99.98938	1	1
RNF26	1	1	1	1	1	1	1	1	1	1	1	36.02574	1	1	1	1	1	1	1	103.6483
RNF34	1	1	1	1	1	33.65862	1	1	1	34.07434	1	40.01754	31.09783	1	72.91084	64.40764	1	1	1	1
RNF38	1	29.95145	1	87.9502	73.67483	40.16748	1	1	1	68.39467	70.10671	62.19153	42.77049	70.09112	1	1	1	57.34022	109.1551	70.02264
RNF41	1	35.60424	1	1	45.29636	38.26981	1	38.69319	1	28.1398	1	38.06579	31.54283	1	1	1	1	44.80334	1	1
RNF44	1	1	1	61.82565	71.84741	1	1	28.53644	97.92584	28.57221	70.71204	27.55487	1	66.86914	57.17371	1	125.4256	35.27327	1	103.6483
RNF8	1	37.78099	31.65778	54.13875	56.42469	74.77513	1	1	1	58.874	81.00888	49.90364	36.33315	84.51881	1	73.33238	1	31.70627	1	1
RNFT2	1	1	36.97535	1	1	1	1	1	1	1	1	1	1	1	1	1	1	1	1	1
RNGTT	1	1	1	1	47.66473	1	1	1	1	46.81039	1	34.1872	25.58112	50.16283	54.95907	1	1	19.80494	1	208.0679
RNPC3	1	39.36239	74.03373	50.30714	44.15391	35.41707	1	1	96.05129	38.76866	65.28718	25.32247	1	1	1	1	93.86422	1	72.01623	70.02264
ROBO4	1	1	1	1	1	1	1	1	1	1	1	1	1	1	1	1	1	275.4371	1	1
ROGDI	1	1	33.77749	45.95361	39.54194	27.24141	1	1	108.6489	1	1	1	1	1	64.9905	1	73.17241	33.08564	1	1
RORA	1	69.17	36.27145	1	1	33.52362	1	82.56347	1	1	1	1	26.16212	1	1	1	1	71.35108	1	1
RORB	1	36.29202	1	1	1	38.2801	1	1	1	1	1	1	24.27613	1	60.06822	1	75.36436	1	1	70.02264
RPA1	1	80.71731	40.12582	1	1	49.13508	1	33.43639	1	89.1606	1	80.06675	46.9094	1	1	1	1	27.18639	1	1
RPA3	1	63.009	62.92967	1	1	47.06621	1	1	1	97.09409	1	113.8818	52.45215	1	1	74.47075	1	1	1	1
RPAP3	1	36.22905	1	1	1	31.35634	1	1	1	1	1	33.64328	43.63524	1	1	1	1	1	1	1
RPE	1	39.15603	1	1	1	33.58992	1	1	1	32.47106	1	31.77387	1	1	1	1	1	1	1	1
RPF2	1	52.39107	37.41083	1	1	24.74537	1	1	1	41.06679	1	36.35211	37.54663	1	1	1	1	131.5445	66.58669	1
RPGR	1	1	1	1	1	1	1	1	1	1	1	22.01244	126.1702	1	1	1	1	1	1	1
RPGRIP1L	1	1	1	1	1	1	1	1	1	1	1	25.6642	53.52778	1	1	1	1	1	1	1
RPH3A	1	1	1	1	1	1	1	1	1	1	1	1	1	92.60192	1	1	1	1	1	1
RPL39L	1	1	1	1	1	30.56251	1	1	1	54.12952	1	58.80482	38.88937	1	1	1	1	1	1	1
RPP14	1	30.53867	1	1	1	31.6704	1	1	1	38.38133	1	31.45692	33.84375	1	40.39947	1	1	24.48776	1	1
RPP25	1	1	1	1	1	1	1	1	1	1	1	1	1	88.63993	1	1	1	1	1	70.02264
RPP25L	1	1	41.42146	1	1	1	1	1	1	1	1	1	1	1	1	1	1	1	1	1
RPP38	1	1	1	1	1	1	1	1	1	1	1	23.31275	40.34035	1	1	1	1	1	1	1
RPRD1A	1	67.25947	91.6706	69.25495	71.48888	82.35124	1	22.20029	1	70.70835	84.15601	62.73964	62.7584	101.1683	79.7918	77.48007	62.85372	81.96754	1	344.3419
RPRD1B	1	1	1	1	1	1	1	1	1	42.45476	1	33.72181	26.71626	1	1	1	1	25.49602	1	1
RPRM	1	37.58491	112.7522	1	1	1	1	1	250.0605	1	1	1	1	1	1	1	1	1	1	70.02264
RPRML	1	1	1	1	1	1	1	1	206.9422	1	1	1	1	1	1	1	1	1	1	1
RPS6KA2	1	1	1	1	1	1	1	45.40238	1	1	1	1	1	86.7137	1	1	1	103.4225	1	1
RPS6KA3	1	1	1	1	1	1	1	50.61322	1	1	1	1	1	1	1	1	1	122.5112	1	1
RPS6KL1	1	1	34.75635	1	1	1	1	1	82.40908	1	1	1	1	1	67.04472	1	48.97303	1	1	103.6483
RRAD	1	1	1	1	1	1	1	65.59187	1	1	1	1	1	1	1	1	1	1	1	1
RRAGB	1	1	1	49.75548	46.96226	39.76038	1	1	114.5374	1	1	1	28.07631	1	1	1	1	1	1	1
RRAGD	73.24858	1	49.05082	1	1	1	1	1	65.54123	1	1	26.07274	24.61683	1	1	81.56721	1	1	1	1
RRAS	1	1	1	1	1	1	1	200.2272	1	1	1	1	1	1	1	1	1	62.9744	1	1
RRBP1	67.04108	81.8584	54.43938	1	1	29.91508	1	143.9235	1	1	1	1	1	1	1	1	1	190.4144	1	1
RRM2	1	1	1	1	1	1	1	1	1	95.68755	1	230.3514	1	1	1	75.71571	1	1	1	1
RRM2B	1	1	1	1	1	35.5414	1	1	1	1	1	1	29.26287	1	67.99417	1	1	1	1	1
RRN3	1	1	26.0421	1	44.88501	29.10132	1	26.69711	1	41.56495	1	25.25839	27.4974	1	1	1	1	29.52107	1	1
RRP8	1	34.23231	1	1	1	34.22298	1	1	1	42.43117	1	34.1862	33.31454	1	1	50.52234	1	1	1	1
RRS1	1	40.05763	1	1	1	44.46707	1	1	1	32.82879	1	1	35.92963	1	1	1	1	1	1	1
RSPH1	1	1	1	1	1	1	1	1	1	1	1	1	170.8827	1	1	1	1	1	1	1
RSPH3	1	1	1	1	1	28.72556	1	1	1	1	1	1	57.95933	1	1	1	1	1	1	1
RSPH4A	1	1	1	1	1	1	1	1	1	1	1	1	64.89756	1	1	1	1	1	1	1
RSPH9	1	1	1	1	1	1	1	1	1	1	1	1	60.5455	1	1	1	1	1	1	1
RTCA	91.33849	48.96139	1	1	39.3749	36.98469	1	1	1	1	1	26.90185	28.24371	70.32499	1	1	1	1	1	1
RTKN	1	1	33.97672	1	1	1	1	1	1	1	1	1	1	1	1	1	1	1	1	1
RTKN2	1	1	46.52279	1	1	1	1	1	1	36.55644	1	131.7915	1	1	1	1	1	1	1	1
RTL5	1	1	1	53.08824	47.40128	1	1	67.9666	1	48.25378	1	36.56791	1	1	1	1	1	1	1	1
RTN2	1	1	1	55.99654	62.18592	1	1	1	1	1	1	1	1	1	63.80421	1	186.0982	1	62.79841	1
RTN4R	1	1	1	1	44.71473	1	1	1	1	1	1	1	1	1	1	1	1	1	1	103.6483
RTN4RL2	1	1	1	1	1	1	1	107.444	1	1	1	1	1	1	1	1	1	1	1	1
RTTN	65.65896	1	1	1	1	1	1	1	1	1	1	1	1	1	1	1	1	1	1	1
RUNDC1	1	1	1	1	1	34.42202	1	1	66.85692	32.17442	1	33.34356	56.83673	1	1	1	1	1	1	1
RUNDC3A	1	1	47.47951	1	40.89646	1	1	1	48.1868	1	1	1	1	1	65.5326	1	1	1	1	1
RUNDC3B	1	1	1	1	1	1	1	1	1	1	1	1	1	1	1	1	1	176.3003	1	1
RUNX1	49.08867	1	1	1	1	1	1	61.7828	1	1	1	1	1	1	1	1	1	1	1	1

RUNX1T1	1	1	1	86.43588	153.4001	1	1	134.5128	127.8434	35.59555	266.8625	1	1	1114.347	358.3749	73.14704	619.348	37.07598	106.6267	308.945
RUNX3	1	1	1	1	1	1	1	86.98373	1	1	1	1	1	1	1	1	1	1	1	1
RUSC2	1	1	1	1	49.28931	1	1	1	77.38496	1	71.94789	1	1	65.41707	54.86123	1	88.17949	1	1	70.02264
RUVBL1	1	77.31286	58.41973	1	47.45603	62.90224	1	31.49944	1	77.01389	1	73.97408	120.4848	1	1	56.83795	1	31.74433	81.85016	103.6483
RXRA	1	36.88177	1	1	1	29.02501	1	1	1	1	143.1249	1	1	1	1	1	1	19.90137	1	1
RXRB	1	1	42.34814	1	1	1	1	1	1	1	1	1	1	1	1	1	1	22.35417	1	1
RXYLT1	1	1	55.88253	1	1	35.29527	1	70.22634	1	1	1	1	30.32072	1	1	1	1	1	1	1
RYR2	1	1	1	1	1	1	1	1	121.3447	1	1	1	1	1	1	1	1	1	1	1
RYR3	1	1	1	1	1	1	1	1	126.1688	1	1	1	1	1	1	1	1	1	1	1
S100A1	747.3012	1	1	1	1	1	1	1	1	1	1	1	1	1	1	1	1	1	1	1
S100A10	1	1	1	1	1	1	1	56.33259	1	1	1	1	1	1	1	1	1	335.9092	1	1
S100A11	1717.702	1	1	1	1	1	1	685.1569	1	1	1	1	1	1	1	1	1	273.5439	1	1
S100A13	138.6787	87.60301	1	1	1	53.01387	1	1	1	1	1	1	33.03351	1	1	1	1	148.917	1	1
S100A16	1	104.9479	1	1	1	100.5908	1	1	1	1	1	1	54.91746	1	1	1	1	306.1405	1	1
S100A6	1	97.42842	73.19531	1	1	65.77285	1	1	1	1	1	36.05901	44.39545	1	1	56.12843	1	1	1	1
S100A8	118.0417	1	1	1	1	1	1	1	1	1	1	1	1	1	1	1	1	1	1	1
S100A9	181.2978	1	1	1	1	1	1	1	1	1	1	1	1	1	1	1	1	1	1	1
S100B	1	226.5141	1189.946	1	1	70.65387	1	1	1	1	1	1	43.59407	1	1	1	1	1	1	1
S1PR1	1	1	1	1	1	105.098	1	1	1	1	1	28.05034	42.51233	1	1	1	1	78.95658	1	1
S1PR3	1	1	1	1	1	50.89109	1	307.8396	1	1	1	32.49089	82.21868	1	1	1	1	1	1	1
SAAL1	1	1	1	1	1	1	1	1	1	51.19177	1	42.50435	28.20587	1	1	1	1	1	1	70.02264
SAC3D1	1	1	26.95358	1	44.82181	26.958	1	1	1	58.04887	1	81.85275	26.47613	1	1	1	1	1	1	1
SACM1L	1	40.90628	1	1	1	46.08995	1	1	1	43.28427	1	36.02429	48.48194	1	1	42.14374	1	18.02791	56.90348	70.02264
SALL1	63.33606	93.90007	1	1	1	64.24636	1	1	1	54.4034	114.1385	63.30448	55.56733	1	1	60.3607	1	1	1	1
SALL2	1	39.65671	1	1	1	48.46897	1	1	1	1	1	25.79838	38.60866	1	1	1	1	1	1	1
SALL3	1	166.4639	116.7781	1	1	158.2859	1	1	1	98.45042	1	131.6682	101.4681	1	1	139.1329	1	1	1	1
SAMD15	1	1	1	1	1	1	1	1	1	1	1	1	32.61328	1	1	1	1	1	1	1
SAMD4A	1	61.21564	34.97907	1	1	89.30359	1	1	1	1	1	43.47612	175.3103	1	1	1	1	15.30456	1	1
SAMHD1	65.67023	1	1	1	1	1	1	1	1	1	1	1	1	1	1	1	1	33.24336	1	1
SAMSN1	448.0439	1	1	1	1	1	1	1	1	1	1	1	1	1	1	1	1	1	1	1
SAPCD2	1	1	77.45365	1	1	1	1	1	1	1	1	52.05098	1	1	1	1	1	1	1	1
SART1	1	1	76.19608	1	1	29.30256	1	32.86107	1	36.54813	1	31.38369	32.67633	1	1	1	1	11.79736	1	1
SASH1	1	60.27859	108.3276	1	1	27.32716	1	83.52384	1	1	1	1	36.19558	1	1	1	1	71.30731	1	1
SASS6	1	45.63896	1	1	1	1	1	1	1	42.62049	1	45.04819	1	1	1	1	1	1	1	1
SATB1	1	132.7939	272.1872	121.0144	62.49499	67.22773	1	23.14534	149.5393	59.82	160.3163	44.48225	51.0605	302.7994	1	1	1	1	61.64333	1
SATB2	1	1	1	132.5368	524.0553	1	650.2642	1	269.8422	1	1	1	1	1	1207.125	1	1432.051	1	277.0125	792.1035
SATB2.AS1	1	1	1	1	1	1	1	1	1	1	1	1	1	1	64.57055	1	1	1	1	1
SAV1	1	75.92879	1	1	1	64.39894	1	130.9196	1	37.65835	1	68.93002	81.91218	1	1	1	1	134.1113	1	1
SAYSD1	1	1	52.19834	1	1	30.58174	1	1	1	1	1	32.86555	1	1	1	1	1	1	1	1
SBK1	1	50.82726	56.63245	168.7035	188.0324	1	192.7699	1	99.93153	77.51744	254.3742	67.16855	39.1656	264.2012	198.0255	80.23468	122.6581	1	157.0541	377.9676
SC5D	1	96.61957	91.60635	61.17949	89.25105	156.5928	1	24.45326	55.64948	78.84682	1	80.17099	106.7695	1	147.2872	124.1353	191.3348	25.07626	1	241.6936
SCAMP2	119.9536	55.75668	32.61957	1	1	47.06573	1	43.13031	1	39.595	1	28.74593	32.86324	1	1	1	1	47.11375	62.6946	1
SCAMP4	1	1	52.21544	1	46.59271	34.31328	1	30.60377	1	1	1	35.09915	55.36193	1	1	1	105.1222	42.65515	1	103.6483
SCAMP5	1	1	38.41718	50.88455	48.21606	1	1	1	75.46627	1	1	1	1	69.53713	65.02822	1	1	1	74.24053	1
SCAP	1	1	29.2451	1	1	24.9673	1	1	1	1	1	1	25.40987	1	1	1	52.54484	20.33648	1	1
SCARA3	1	1	1	1	1	39.79751	1	1	1	1	1	36.67503	1	1	1	1	1	1	1	1
SCARB1	1	1	48.7294	1	1	1	1	1	1	1	1	1	1	1	1	1	1	157.5597	1	1
SCARF1	1	1	1	1	1	1	1	1	1	1	1	1	1	1	1	1	1	108.9503	1	1
SCD	30.5645	117.4477	216.9554	1	77.71082	319.8798	1	1	86.94971	143.4082	1	113.0958	98.46981	1	118.5846	219.8565	301.5977	1	1	103.6483
SCFD2	1	25.98306	1	1	1	1	1	1	1	1	1	1	1	1	1	1	1	1	1	1
SCG3	1	170.0008	481.7356	214.9326	248.4016	1	286.0741	1	578.6165	45.4546	412.7029	52.41986	1	318.3971	309.457	1	373.2651	1	345.9282	172.671
SCG5	1	52.88353	202.3739	155.395	200.1266	65.71932	232.9955	26.6122	759.3113	1	91.45039	1	79.96994	72.49057	335.1452	90.38069	224.9877	1	227.8254	529.4422
SCGN	1	1	1	1	1	1	1	1	1	1	456.3791	1	1	1	1	1	1	1	1	1
SCIMP	45.05934	1	1	1	1	1	1	1	1	1	1	1	1	1	1	1	1	1	1	1
SCIN	121.7243	1	1	1	1	1	1	1	1	1	1	1	1	1	1	1	1	1	1	1
SCMH1	1	1	1	1	1	1	1	1	1	1	1	1	1	1	1	1	46.80094	1	1	1
SCML2	1	1	1	1	1	1	1	1	1	1	1	1	1	67.49172	1	1	1	1	1	1
SCN1A	1	49.61378	169.3462	1	1	1	1	1	1	1	1	1	1	1	1	1	1	1	1	1
SCN2A	1	1	1	1	47.32085	1	1	1	494.6397	1	1	1	1	1	1	1	217.4716	1	1	139.0453
SCN3A	1	1	89.57652	109.0478	88.50283	1	1	1	316.1689	44.93365	109.0659	29.0714	1	91.5141	144.4361	1	274.434	1	118.2455	139.0453
SCN3B	1	1	1	68.73561	117.4996	1	1	1	304.6604	1	124.5939	1	1	96.07812	124.9226	1	211.7702	1	1	308.945
SCN8A	1	1	1	1	1	1	1	1	173.6892	1	77.40199	1	1	59.14544	1	1	1	1	43.85953	1
SCNM1	65.72696	1	57.54365	50.20642	44.58188	29.54663	1	1	1	56.70578	1	36.16567	24.38748	1	1	1	1	33.18408	1	1
SCO1	1	42.22256	1	1	1	38.78733	1	1	1	42.79102	1	32.7867	41.47193	1	71.53157	1	1	26.57352	1	1
SCPEP1	64.70192	1	1	1	1	1	1	53.82218	1	1	1	1	1	1	1	1	1	23.31917	1	1
SCRG1	1	216.8868	1537.599	1	1	301.109	1	1	1	35.79828	1	80.64447	148.9808	1	1	142.0552	1	1	1	1
SCRIB	1	1	1	1	1	39.6253	1	1	1	1	1	24.83341	35.99444	1	1	1	1	1	1	1
SCRN3	1	1	44.58341	1	1	31.1997	1	1	1	1	1	1	23.64693	1	1	1	1	1	1	1
SCRT1	1	1	1	1	1	1	1	1	1	1	1	1	1	70.06023	1	1	45.05381	1	1	103.6483
SCRT2	1	1	1	183.6695	102.7231	1	1	1	1	39.60117	68.21891	1	1	95.68664	92.42301	1	1	1	140.2754	103.6483
SCUBE1	1	1	1	1	1	1	1	1	251.7953	1	1	1	1	1	39.32149	1	100.8049	1	1	1
SCX	1	1	1	1	1	1	1	1	1	1	1	1	1	1	1	1	1	62.15165	1	1
SCYL1	1	36.86584	1	1	1	26.93053	1	35.75801	1	1	1	25.71718	23.68593	1	53.24832	1	1	113.3505	1	1
SDC2	1	1	1	1	1	96.43732	1	77.35615	1	108.9162	1	77.13984	128.1625	1	1	42.38585	1	1	1	1
SDC3	1	239.022	140.9415	1	1	68.54076	1	46.21156	125.317	37.80587	1	49.62225	51.02261	1	64.53713	1	1	111.6524	83.99158	1
SDCCAG3	1	36.81369	53.72593	1	1	30.11059	1	36.61797	1	35.57196	1	34.52129	30.27474	1	1	1	231.6209	122.9921	73.13856	1
SDHAF1	1	1	57.31752	1	1	1	1	1	1	1	1	1	1	1	1	1	1	1	1	1
SDHAF2	1	35.74714	59.34569	41.69851	1	40.07351	1	1	1	47.98284	1	40.68467	46.33222	66.84541	59.8604	41.673	1	25.73785	1	1
SEC22C	1	45.40106	38.44118	1	43.1898	50.77626	1	1	1	62.9168	1	62.47824	54.2366	1	63.07776	1	117.0749	48.90542	1	1
SEC24D	1	1	1	1	1	1	1	85.61177	1	1	1	1	34.47072	1	1	1	1	17.8853	1	1
SEC61A2	1	1	41.10333	1	1	37.21975	1	1	58.95603	1	1	27.53538	1	1	1	1	1	1	1	1
SEH1L	1	40.94461	1	1	47.58076	24.79877	1	1	1	1	1	23.25814	1	1	1	1	67.65446	32.11859	1	1
SEL1L3	1	93.13332	1	1	1	114.024	1	1	166.3381	147.8972	1	74.6318	1	1	1	79.40373	89.92952	1	1	1
SELENOI	1	1	68.70567	1	1	44.829	1	1	1	37.89642	1	26.3356	31.95062	1	1	1	1	1	1	139.0453
SELENOM	1	1	1	1	1	37.74199	1	154.1178	1	1	1	1	1	1	1	1	1	58.67418	1	1
SELENON	1	44.55638	31.06095	1	1	55.01552	1	68.46748	1	48.28357	1	48.36502	36.83848	1	1	1	1	46.71919	1	103.6483
SELENOP	1	157.9419	1	1	1	181.3192	1	68.87278	1	61.55927	1	37.90014	95.93991	1	1	88.61192	1	143.561	1	1
SELPLG	42.1757	1	1	1	1	1	1	1	1	1	1	1	1	1	1	1	1	1	1	1
SEMA3A	1	1	1	48.55502	98.99397	28.81545	1	1	133.2631	1	1	1	1	1	196.6218	1	68.30011	1	1	70.02264
SEMA3C	1	1	1	250.5586	384.7438	1	307.4146	1	207.746	1	1	1	1	1	362.609	1	69.39899	50.72467	381.7616	310.7162
SEMA3E	1	1	1	1	1	1	1	1	554.8682	1	1	1	1	1	1	1	1	1	1	1
SEMA3G	1	1	1	1	1	1	1	1	1	1	1	1	1	1	1	1	1	159.6632	1	1
SEMA4A	1	1	1	1	1	1	1	76.4204	1	1	1	1	1	1	1	1	1	1	1	1
SEMA4C	1	1	1	50.8018	51.37121	1	1	58.96211	89.51212	1	1	23.27257	25.13433	1	1	1	82.45844	53.19661	83.34446	103.6483
SEMA4D	1	1	1	1	61.45651	1	1	1	1	1	1	1	1	1	1	1	1	1	65.64405	1
SEMA4G	1	1	1	1	1	1	1	1	1	1	1	1	1	1	1	1	1	1	1	206.2967
SEMA5A	1	79.59089	375.1602	1	1	1	1	853.2846	1	1	1	1	1	1	1	1	1	1	1	1
SEMA5B	1	42.57556	1	50.86404	1	87.44625	1	1	1	69.04181	1	50.33196	71.53315	1	1	1	1	1	1	1
SEMA6A	1	104.0773	79.23175	78.37099	60.13397	134.3562	1	1	1	71.78734	1	69.29839	65.6718	206.5231	56.071	53.93909	1	150.2684	51.53781	1
SEMA6C	1	1	1	61.98944	63.05281	1	1	1	1	1	1	1	1	61.70481	56.82581	1	78.41469	35.41638	82.10692	87.58009
SEMA6D	1	37.38399	123.6056	1	1	1	1	26.27957	1	1	1	1	1	1	1	1	1	1	1	103.6483

SENP2	1	39.53358	40.954	1	1	38.55993	1	1	1	33.74536	1	44.56251	38.29734	1	1	1	1	15.37556	90.78278	1
Sep-04	1	1	1	1	1	1	1	151.5467	1	1	1	1	1	1	1	1	1	1	1	103.6483
Sep-08	1	59.89596	84.99884	1	1	43.10227	1	45.36744	1	1	1	27.23714	45.97114	1	1	1	1	49.19903	1	1
SERAC1	1	1	1	1	1	1	1	1	1	1	1	1	34.57828	1	1	1	1	1	1	1
SERINC5	1	93.54158	168.9981	65.7253	45.55443	1	1	79.40324	246.1133	1	95.55314	1	27.49009	121.361	1	1	122.7969	102.1781	40.6956	1
SERP2	1	45.91369	50.95193	82.92973	97.32465	35.0732	166.9531	35.99902	70.88396	37.27499	75.28395	1	1	94.95558	164.543	1	115.3929	116.2825	81.65871	103.6483
SERPINA1	96.61372	1	1	1	1	1	1	1	1	1	1	1	1	1	1	1	1	1	1	1
SERPINB1	147.0062	1	1	1	1	1	1	55.08219	1	1	1	1	1	1	1	1	1	25.13151	1	1
SERPINB9	115.0738	1	1	1	1	1	1	42.42135	1	1	1	1	1	1	1	1	1	49.45693	1	1
SERPINE2	1	326.7389	548.0323	69.27312	132.4992	140.7525	162.8126	1	1	57.09335	1	82.14881	138.2314	1	360.0817	118.6664	181.7716	1	143.9584	310.7162
SERPINF1	215.0125	1	1	1	1	1	1	1	1	1	1	1	1	1	1	1	1	1	1	1
SERPING1	1	1	1	1	1	28.75775	1	225.7673	1	35.99922	1	28.05627	31.61146	1	1	1	1	22.3484	1	1
SERPINH1	1	49.03922	1	1	1	47.86617	1	531.2031	1	1	1	34.29529	38.36855	1	1	1	1	424.6682	1	1
SERPINI1	1	1	1	1	1	1	1	1	566.1621	1	1	1	1	1	1	1	88.78955	1	1	1
SERTAD1	103.6806	52.06273	1	1	1	47.58939	1	409.9202	1	30.13808	1	31.97446	47.73493	1	1	1	1	337.6764	1	1
SERTAD4	1	1	1	1	1	29.09039	1	1	1	1	1	1	1	1	1	1	1	1	1	1
SESN1	1	1	1	1	54.55349	1	1	26.22414	1	1	1	1	1	1	1	1	40.06493	1	1	1
SETD1A	1	1	1	1	1	1	1	1	1	1	1	1	1	1	1	1	1	1	1	116.5001
SETD1B	1	1	1	1	1	1	1	1	1	1	1	28.48581	1	1	1	1	1	1	1	1
SETD6	1	1	1	1	1	1	1	1	1	1	1	1	28.25534	1	1	1	1	1	1	1
SETD7	1	1	1	252.3143	158.2641	1	1	1	90.12108	129.3279	1	75.66454	44.44243	1	1	1	1	169.2047	163.1146	1
SETDB1	1	1	1	1	1	1	1	1	1	36.76481	1	1	20.6157	1	1	1	1	22.87413	1	1
SEZ6	1	46.96746	1	232.8899	137.2864	1	1	1	248.7951	79.6405	1	52.55711	1	1	67.25353	1	197.0704	1	146.8954	206.2967
SEZ6L	1	325.3514	181.0896	154.7707	85.71809	66.68017	1	1	1	245.2857	1	99.80494	51.4773	1	82.7483	1	1	1	91.13326	103.6483
SEZ6L2	1	1	1	78.65614	103.007	1	1	1	298.1885	1	97.97399	1	1	140.9485	149.7075	1	137.2861	1	35.65614	70.02264
SF3A2	1	1	1	1	1	1	1	1	1	1	1	22.66104	1	1	45.58221	1	1	1	1	1
SFMBT2	114.5755	1	1	1	1	1	1	1	1	1	1	1	1	1	1	1	1	1	1	1
SFR1	1	1	1	1	1	1	1	1	1	1	1	35.73228	1	1	1	1	1	1	1	70.02264
SFRP1	1	297.0584	44.03894	193.4747	164.3314	1941.543	147.6579	1	1	467.7488	82.14514	745.1929	230.8632	1	48.39482	1425.741	1	1	295.6561	1
SFRP2	1	72.6632	1	1	1	101.025	1	1	1	1	1	136.0937	277.59	1	1	91.98566	1	1	1	1
SFRP4	1	1	1	1	1	1	1	1	1	1	1	1	37.28127	1	1	1	1	1	1	1
SFTA1P	1	1	1	1	1	1	1	59.39862	1	1	1	1	1	1	1	1	1	1	1	1
SFXN3	1	1	1	1	53.64707	1	1	1	1	1	1	1	1	1	49.67583	1	173.6621	21.69178	1	1
SFXN4	1	32.39131	42.79958	1	1	1	1	1	1	33.86401	1	1	26.03537	1	1	1	81.66848	1	1	1
SFXN5	1	72.17741	1	48.2179	1	151.6207	1	1	1	86.74482	1	75.74982	54.52325	1	1	188.8429	1	11.07157	1	1
SGCD	1	1	65.79011	1	1	1	1	1	1	1	1	1	1	1	1	1	1	1	1	1
SGIP1	1	1	1	69.84991	58.3758	1	1	69.6451	92.7841	1	91.10817	1	1	66.97929	1	1	1	1	1	1
SGK1	199.9421	1	132.389	1	1	1	1	166.5807	1	1	1	1	1	1	1	1	110.1925	265.7742	1	1
SGK3	1	1	1	1	1	88.469	1	1	1	1	1	1	31.99787	1	1	1	1	116.7157	1	1
SGMS1	1	1	41.29022	1	1	1	1	1	1	1	1	1	1	1	1	1	1	48.77568	1	1
SGO1	1	1	1	1	1	1	1	1	1	33.19296	1	198.334	1	1	1	1	1	1	1	1
SGO2	1	1	1	1	1	1	1	1	1	1	1	258.1815	1	1	1	1	1	1	1	1
SGSM2	1	32.76006	1	1	42.76312	54.21626	1	29.85783	84.7672	1	1	28.46713	55.89807	1	1	119.4721	1	18.41071	60.51438	70.02264
SGTB	1	1	48.67905	51.61109	46.43066	41.18085	1	1	1	1	1	41.78071	35.61084	1	1	1	1	1	1	1
SH2B3	51.57679	1	1	1	1	1	1	65.81076	1	1	1	1	1	1	1	1	1	33.57081	1	1
SH2D3C	1	1	1	1	1	1	1	104.3635	1	1	1	1	1	1	1	1	1	57.04616	1	1
SH3BP2	1	1	1	1	47.05317	33.56525	1	1	1	1	1	1	1	1	41.57019	1	1	33.04948	86.73844	70.02264
SH3BP4	1	35.8204	43.22992	1	1	1	1	1	1	1	1	1	1	78.77373	1	1	1	191.2909	1	1
SH3BP5	1	1	1	161.6381	246.4034	1	334.5878	1	278.48	1	314.296	1	1	277.5748	277.4137	1	271.5716	198.1623	259.0055	275.3193
SH3BP5.AS	1	1	1	1	1	1	1	1	1	1	1	1	1	1	1	1	98.52528	1	1	1
SH3D19	1	36.44429	69.79297	1	1	1	1	32.85239	1	1	1	1	1	1	1	1	1	1	1	1
SH3GL2	1	1	1	1	1	1	1	1	83.30429	1	1	1	1	1	1	1	149.0106	1	1	1
SH3GL3	1	1	1	1	53.74316	1	1	1	219.2696	1	1	1	1	1	102.3441	1	131.3353	1	1	1
SH3GLB2	1	1	80.69671	1	1	49.66728	1	1	1	31.75854	1	33.31861	35.88362	1	1	128.2464	1	20.62109	1	1
SH3KBP1	112.035	1	79.17936	1	1	25.22803	1	197.957	1	1	1	1	1	1	1	1	1	31.89804	1	139.0453
SH3PXD2A	1	1	1	104.4972	93.38319	1	1	92.20439	1	62.07315	1	48.6746	29.66504	1	118.2963	1	119.0647	35.14314	167.7376	1
SH3PXD2B	1	40.45481	1	1	1	36.64719	1	1	1	26.38069	1	1	1	1	1	1	1	1	1	1
SH3RF3	1	1	1	51.08759	62.22723	1	1	1	1	1	1	1	1	1	1	1	1	1	1	103.6483
SHANK2	1	1	34.08876	1	61.76578	1	1	1	151.7515	1	73.58085	1	1	1	51.57794	1	1	1	1	70.02264
SHANK3	1	1	1	1	1	1	1	1	1	1	1	1	1	1	1	1	1	144.6147	1	1
SHB	1	1	1	49.69542	56.87498	1	1	1	1	1	1	1	1	1	73.60584	1	1	19.71711	76.15705	103.6483
SHC1	1	1	1	1	1	1	1	71.05451	1	1	1	1	1	1	1	1	1	82.70289	1	1
SHC2	1	1	56.17911	1	1	23.21067	1	50.34567	1	1	1	1	1	1	1	1	1	61.18834	1	1
SHC3	1	99.19226	166.7825	1	48.22814	65.45706	1	1	1	1	1	1	32.18839	1	43.13228	1	1	1	1	1
SHCBP1	1	1	1	1	1	1	1	1	1	1	1	83.55816	1	1	1	1	1	1	1	1
SHD	1	1	71.82593	1	1	1	1	1	1	1	1	1	1	1	1	1	1	1	1	1
SHE	1	1	1	1	1	1	1	1	1	1	1	1	1	1	1	1	1	50.2675	1	1
SHF	1	1	1	111.2747	94.57068	1	1	1	59.25276	89.1521	61.62044	41.57829	1	62.09654	42.31613	1	51.56966	61.26829	88.4827	206.2967
SHISA2	1	1	1	1	1	45.27786	1	1	1	1	1	28.15314	1	1	1	1	1	1	1	103.6483
SHISA4	1	55.38835	108.0376	1	1	40.04402	1	1	1	1	1	1	1	1	1	1	1	1	1	1
SHISA5	1	1	47.49664	48.46325	53.22221	48.62627	1	51.37342	1	31.56342	1	44.58923	40.17641	1	55.16362	62.63347	1	50.44033	87.33552	1
SHISA9	1	1	79.67775	1	1	1	1	1	65.29609	1	1	1	25.81845	1	1	1	1	1	1	1
SHROOM1	1	1	1	1	1	1	1	1	1	1	1	1	1	1	1	1	1	65.15084	1	1
SHROOM2	1	1	37.94224	1	1	1	1	1	1	1	1	1	1	1	1	1	1	19.99406	1	1
SHROOM3	1	65.74159	1	1	1	107.6027	1	69.67739	1	48.7614	1	52.03627	162.6956	1	1	94.95504	1	1	1	1
SHROOM4	1	1	1	1	1	1	1	1	1	1	1	1	1	1	1	1	1	67.96365	1	1
SHTN1	189.6263	1	58.82232	188.9367	374.1257	36.74363	497.0534	1	904.1301	69.62871	402.1476	50.73159	26.18051	641.4445	447.8004	251.1451	501.1542	1	420.8862	687.6839
SIAH2	1	40.80764	42.59269	57.08209	62.50599	24.07135	1	20.33709	1	30.83644	111.4174	32.31952	25.77697	86.31315	1	1	1	41.04382	1	1
SIAH3	1	1	1	1	1	1	1	1	1	1	1	1	1	128.5198	1	1	1	1	1	1
SIGIRR	1	1	1	1	1	1	1	86.82294	1	1	1	1	1	1	1	1	1	47.15318	1	70.02264
SIGLEC10	93.91598	1	1	1	1	1	1	1	1	1	1	1	1	1	1	1	1	1	1	1
SIK3	1	73.69804	71.99373	1	1	33.93624	1	46.1988	91.96841	46.91857	1	32.17491	67.61169	1	41.42038	1	130.2198	25.75534	1	70.02264
SIMC1	1	1	1	1	1	1	1	1	1	1	1	36.79096	1	1	1	1	1	1	1	1
SIN3B	1	35.30458	25.92415	50.47556	46.76036	31.86898	1	1	113.6174	1	1	28.14511	31.88207	1	1	1	206.6844	50.10781	1	1
SIPA1	86.57892	1	1	1	1	1	1	66.38592	1	1	1	1	1	1	1	1	1	69.74577	1	1
SIPA1L1	1	54.24337	1	1	1	53.065	1	1	95.59666	35.28591	1	38.77808	45.33033	1	1	1	1	1	1	1
SIPA1L2	83.82955	57.15385	1	95.88974	1	29.82938	1	43.97352	1	143.2754	101.5483	73.8081	28.58617	99.78177	1	1	1	101.0053	80.12886	70.02264
SIRPA	92.85993	1	1	1	1	1	1	1	1	1	1	1	1	1	1	1	1	13.49729	1	1
SIRT2	109.626	88.12888	1141.039	61.50993	66.25152	76.60591	1	61.78152	1	47.33875	90.63923	33.64496	57.60523	91.35376	66.7001	116.2475	1	114.189	1	1
SIRT7	1	36.75564	1	1	1	1	1	1	1	1	1	1	23.91295	1	1	1	1	1	1	1
SKA1	1	1	1	1	1	1	1	1	1	1	1	58.46049	1	1	1	1	1	1	1	1
SKA3	1	1	1	1	1	1	1	1	1	1	1	95.83614	1	1	1	1	1	1	1	1
SKAP2	72.39817	1	1	1	1	1	1	1	1	1	1	1	1	1	1	1	1	25.64238	1	1
SKI	37.00457	46.23582	38.26708	1	39.95034	40.52301	1	92.98137	1	1	1	24.18437	41.30284	70.04828	1	1	1	103.4965	1	1
SKIDA1	1	1	1	1	1	1	1	1	1	1	1	32.05158	1	1	1	1	1	1	54.44798	1
SKP2	1	34.69112	1	1	1	33.64418	1	1	1	63.44693	1	45.51999	39.60023	1	1	31.55085	1	1	1	1
SLA	98.22203	1	1	398.8283	781.2836	1	1166.335	35.00089	411.4082	1	1	1	1	1	1148.839	1	718.2436	105.7868	844.2942	292.8767
SLAIN1	1	64.79728	152.1429	63.39744	84.09905	55.21655	1	1	1	57.1989	526.4379	45.49059	41.42933	593.2171	80.4303	107.3103	1	1	1	103.6483
SLC11A1	66.25371	1	1	1	1	1	1	1	1	1	1	1	1	1	1	1	1	1	1	1
SLC11A2	40.77378	1	36.46155	1	43.11348	32.41213	1	1	70.91132	31.86493	1	1	30.05507	1	1	1	96.14752	27.71498	55.63563	1
SLC12A2	1	38.81503	40.15879	1	1	42.81803	1	605.3568	1	47.54755	1	30.44518	27.78236	1	1	1	1	60.99486	1	70.02264

SLC12A5	1	1	1	1	1	1	1	1	69.81958	1	1	1	1	1	1	1	1	1	1	208.0679
SLC12A7	1	1	1	1	1	1	1	251.137	1	1	1	1	1	1	1	1	1	1	1	1
SLC15A2	1	1	1	1	1	41.84545	1	1	1	1	1	1	1	1	1	1	1	1	1	1
SLC15A3	92.26463	1	1	1	1	1	1	1	1	1	1	1	1	1	1	1	1	1	1	1
SLC16A1.A!	1	1	1	1	1	29.18128	1	1	62.92059	30.38945	1	33.10896	24.56587	1	94.79232	77.05771	1	1	1	1
SLC16A12	1	1	1	1	1	1	1	1	1	1	1	1	28.78353	1	1	1	1	1	1	1
SLC16A14	1	1	33.52371	1	1	1	1	1	77.79148	1	1	1	1	1	1	1	1	71.88847	1	1
SLC16A2	1	38.83642	1	1	1	1	1	23.86499	1	36.80048	1	1	1	1	1	1	1	94.89063	1	1
SLC16A3	162.5169	1	1	1	1	1	1	1	1	1	1	1	1	1	1	1	1	43.60178	1	1
SLC16A7	1	1	1	1	1	1	1	1	1	1	1	1	1	1	1	1	111.1961	1	1	1
SLC16A9	1	1	1	1	1	28.40413	1	1	1	34.00186	1	1	26.66179	1	1	1	1	1	1	1
SLC17A5	1	1	1	1	1	1	1	1	1	1	1	1	1	1	32.26685	1	1	22.47928	1	1
SLC17A6	1	1	1	111.7342	93.82379	1	1	1	1	1	1	1	1	1	1	1	1	1	75.49494	1
SLC17A7	1	1	1	181.2602	159.1545	1	1	1	121.7297	1	1	1	1	1	1	1	172.5095	1	109.0227	1
SLC18B1	1	61.19222	1	1	1	35.10637	1	1	1	1	1	1	23.89326	1	1	1	1	1	1	1
SLC19A1	1	1	1	1	1	1	1	116.0472	1	1	1	1	1	1	1	1	1	1	1	1
SLC1A1	1	1	121.362	1	1	1	1	1	1	1	1	1	1	1	1	1	1	1	1	70.02264
SLC1A3	224.0079	480.6165	118.499	84.08466	1	969.3062	1	118.071	1	312.2492	1	368.5819	537.762	1	1	921.3439	1	1	1	1
SLC1A4	1	1	1	1	1	1	1	1	1	1	1	1	1	1	1	1	1	123.3997	1	1
SLC1A6	1	1	1	1	1	1	1	1	50.30502	1	1	1	1	1	1	1	1	1	1	1
SLC20A2	1	1	1	1	1	1	1	248.1451	1	1	1	1	1	1	1	1	1	50.56139	1	1
SLC22A23	1	1	1	188.0287	177.9178	39.0592	189.0798	44.93861	1	62.40132	1	36.48948	1	1	195.6763	1	108.4265	75.47008	91.06313	514.2416
SLC23A2	1	1	26.88885	1	1	1	1	1	93.32077	1	1	1	1	1	50.80895	1	133.7208	9.951081	65.80822	1
SLC24A2	1	1	1	74.89702	56.76954	1	1	1	98.21287	1	1	1	1	1	1	1	1	1	1	1
SLC25A17	1	1	1	1	1	24.48131	1	28.20708	1	33.67278	1	35.49026	27.81006	1	53.65413	1	1	1	1	1
SLC25A18	1	1	1	1	1	46.85985	1	1	1	1	1	1	1	1	1	1	1	1	1	1
SLC25A22	1	1	1	1	1	1	1	1	1	1	1	1	1	1	1	1	73.62874	1	1	1
SLC25A23	1	44.36297	42.12982	1	1	61.57273	1	52.20694	1	1	1	25.46034	36.05397	1	58.42839	43.31687	1	1	1	1
SLC25A24	68.10564	33.19521	1	1	1	36.19798	1	80.38179	1	34.1808	84.04388	53.89145	30.83299	93.8466	1	1	1	63.84661	1	103.6483
SLC25A46	1	35.10519	1	48.82407	40.14115	48.59536	1	28.72911	1	33.09504	1	1	30.77556	1	48.69222	1	1	29.05366	83.02318	1
SLC26A2	1	1	32.57381	1	1	1	1	1	1	1	1	1	1	1	1	1	1	207.9249	1	1
SLC27A1	1	38.18805	35.08265	1	1	35.78228	1	1	1	1	1	1	1	1	1	1	1	16.56395	1	1
SLC29A4	1	1	1	1	1	1	1	1	42.60686	1	1	1	1	1	46.598	1	1	1	1	1
SLC2A1	1	1	1	1	1	1	1	1	1	1	1	1	33.61232	1	1	1	1	771.8797	1	1
SLC2A10	1	1	1	1	1	40.09454	1	1	1	1	1	1	1	1	1	1	1	1	1	1
SLC2A13	1	1	101.7999	1	1	1	1	1	1	1	1	1	1	1	1	1	1	1	1	1
SLC2A3	1	79.39134	1	1	1	183.5427	1	845.4211	1	53.54359	1	47.50574	121.9243	1	1	82.4592	73.54199	720.9491	1	1
SLC2A4RG	1	1	1	1	1	36.83192	1	70.75379	1	1	1	1	29.81126	1	1	68.83128	1	32.66825	1	1
SLC2A6	1	1	76.55481	1	1	1	1	1	1	1	1	1	1	1	1	1	1	1	1	1
SLC30A1	1	1	1	1	1	46.17869	1	1	1	1	1	1	1	1	1	1	1	122.2119	1	1
SLC30A10	1	1	1	1	1	1	1	69.87186	1	1	1	1	1	1	1	1	1	1	1	1
SLC32A1	1	1	1	1	1	1	1	1	1	1	175.9521	1	1	153.4543	1	1	1	1	1	1
SLC35A3	1	34.70315	1	1	1	41.19362	1	1	1	1	67.30878	31.39516	37.70158	1	1	1	68.81081	20.99092	68.3782	1
SLC35B4	1	1	80.11111	1	46.00423	1	1	1	1	36.2681	1	28.53695	23.65094	1	45.33576	1	73.82716	1	1	1
SLC35E1	1	46.39524	29.36871	52.56442	57.13478	31.71739	1	32.23554	1	46.19367	64.22885	40.49671	40.72462	1	58.29035	1	1	42.0612	114.9901	70.02264
SLC35E2	1	1	1	46.37265	41.32223	1	1	1	1	35.15463	1	28.27793	1	1	56.89324	1	1	1	1	1
SLC35E2B	1	1	36.80799	56.4766	45.19591	1	1	1	1	34.16544	1	24.05026	1	1	57.74022	1	90.20319	1	1	87.58009
SLC35F1	1	73.15978	170.5341	1	1	172.7894	1	72.45485	117.0501	38.50878	1	75.98124	84.51945	1	1	75.46081	1	1	1	1
SLC35G1	1	1	1	1	1	1	1	1	105.476	1	1	1	1	1	1	1	1	1	1	1
SLC37A3	1	31.35454	1	1	43.01265	27.7196	1	1	1	27.71962	1	1	24.27173	1	49.63725	1	1	38.48437	58.34274	139.0453
SLC38A1	1	1	122.7804	165.7696	202.3006	1	235.6731	1	277.8517	36.37097	201.4083	31.25596	1	119.0474	268.7678	1	302.5814	111.2501	242.8659	306.3607
SLC38A11	1	1	1	1	1	1	1	163.4816	1	1	1	1	1	1	1	1	1	1	1	1
SLC38A3	1	1	1	1	1	1	1	1	1	1	1	1	1	1	1	1	1	199.3607	1	1
SLC38A5	1	1	1	1	1	1	1	1	1	1	1	1	1	1	1	1	1	816.5019	1	1
SLC39A13	1	34.74201	1	1	1	23.67594	1	30.35891	1	1	1	1	1	1	1	1	1	19.16029	1	1
SLC39A8	1	1	1	1	1	1	1	1	1	1	1	1	1	1	1	1	1	110.0599	1	1
SLC40A1	85.0369	1	1	1	1	1	1	158.0898	1	1	1	1	1	1	1	1	1	116.0066	1	70.02264
SLC41A1	1	1	1	1	1	37.95459	1	35.7138	1	1	1	1	67.26784	1	1	1	1	40.75083	1	1
SLC43A2	69.55432	1	1	1	1	1	1	1	1	1	1	1	1	1	1	1	1	112.7403	1	1
SLC43A3	1	1	1	1	1	1	1	92.55858	1	1	1	1	1	1	1	1	1	26.00721	1	1
SLC44A5	1	35.55605	41.27983	1	92.15338	29.47496	1	1	1	1	1	1	1	1	127.5732	1	284.5625	1	50.35578	208.0679
SLC4A10	1	1	1	1	1	1	1	1	175.4815	1	1	1	1	1	1	1	1	1	1	70.02264
SLC4A1AP	1	52.5696	63.31837	47.90882	42.74918	34.41426	1	1	165.6562	45.07637	1	36.4665	34.8703	66.2647	53.60258	1	1	35.19054	1	116.5001
SLC4A3	1	1	1	1	1	1	1	1	98.5393	1	1	1	1	1	1	1	1	1	1	103.6483
SLC4A4	1	45.49729	1	1	1	58.95675	1	1	1	1	1	1	1	116.8753	1	1	1	1	1	1
SLC4A8	1	1	1	1	42.21449	28.1281	1	1	42.78383	1	1	89.04617	31.98561	1	1	1	1	1	1	1
SLC52A2	1	1	48.24134	1	1	30.45321	1	48.28303	1	1	1	1	1	1	1	1	1	180.4297	1	1
SLC5A6	1	1	1	1	1	1	1	1	1	1	1	1	1	1	1	1	1	49.85119	1	1
SLC6A1	1	88.89357	74.13163	1	1	73.16873	1	445.5717	1	1	163.751	21.82544	27.79521	179.7204	1	70.3966	1	1	1	1
SLC6A12	1	1	1	1	1	1	1	206.4295	1	1	1	1	1	1	1	1	1	1	1	70.02264
SLC6A15	1	1	1	1	1	1	1	1	1	1	1	1	1	1	1	1	87.0555	1	1	1
SLC6A8	1	75.37704	56.0612	1	1	38.76344	1	1	1	1	1	25.5656	50.74657	1	1	1	1	18.3454	1	1
SLC7A1	1	1	1	1	1	1	1	1	1	1	1	1	1	1	1	1	1	372.5704	1	1
SLC7A11	1	1	1	1	1	48.09724	1	1	1	1	1	1	1	1	1	1	1	136.4536	1	1
SLC7A5	1	1	1	1	1	1	1	1	1	1	1	1	1	1	1	1	1	826.2338	1	70.02264
SLC7A7	197.8239	1	1	1	1	1	1	1	1	1	1	1	1	1	1	1	1	1	1	1
SLC7A8	1	1	1	1	1	1	1	1	1	1	1	1	1	1	1	1	1	146.9147	1	70.02264
SLC9A1	1	1	1	1	1	1	1	175.9655	1	1	1	1	1	1	1	1	1	23.47104	1	1
SLC9A3R1	1	55.30929	61.00831	1	1	146.1303	1	229.0231	1	56.88034	1	46.60499	77.53752	1	1	116.224	1	26.97233	1	1
SLC9A3R2	1	1	1	1	1	1	1	88.63791	1	1	1	1	1	1	1	1	1	525.3874	1	1
SLC9A6	1	1	1	1	1	22.66246	1	1	159.8917	1	1	1	1	1	1	1	1	1	1	1
SLC9A9	46.97029	1	1	1	1	1	1	1	1	1	1	1	1	1	1	1	1	1	1	1
SLCO1A2	1	1	1	1	1	1	1	1	1	1	1	1	1	1	1	1	1	187.5035	1	1
SLCO1C1	1	77.51475	1	1	1	187.2865	1	39.93182	1	1	1	34.89104	1	1	1	137.753	1	1	1	1
SLCO2A1	1	1	1	1	1	1	1	1	1	1	1	1	1	1	1	1	1	71.18313	1	1
SLCO2B1	94.62533	1	1	1	1	1	1	1	1	1	1	1	1	1	1	1	1	318.3571	1	1
SLCO3A1	1	1	1	1	1	1	1	26.21399	61.9602	1	1	1	1	1	1	1	211.7193	1	1	1
SLCO5A1	1	1	1	72.24302	58.33757	1	1	1	1	38.78841	136.8545	27.61484	1	169.7766	1	1	156.9407	1	1	1
SLFN5	1	1	1	1	1	1	1	30.30708	1	1	1	1	1	1	1	1	1	104.364	1	1
SLIT1	1	1	35.79878	1	1	1	1	1	375.7944	1	127.7875	1	1	1	1	1	1	1	1	1
SLIT2	1	1	1	1	1	1	1	1	1	1	1	1	43.56225	1	1	1	1	1	1	1
SLITRK1	1	1	40.1127	1	1	1	1	1	114.0639	1	1	1	1	1	1	1	1	1	1	1
SLITRK2	1	90.46577	207.4632	1	1	88.88658	1	1	1	38.32638	1	50.6583	1	1	1	98.20535	1	1	1	1
SLITRK4	1	1	1	1	51.69849	30.11554	1	1	1	1	1	1	1	125.2656	1	1	1	1	1	1
SLITRK5	1	1	48.86412	1	1	1	1	1	296.4666	1	1	1	1	1	1	1	202.2266	1	1	1
SLMAP	1	1	40.77939	1	41.52932	34.14578	1	28.03779	1	34.59032	1	34.07559	28.83622	1	1	1	1	40.63092	66.17703	103.6483
SLX4IP	1	1	1	1	1	1	1	1	1	1	1	1	1	1	1	1	1	19.46557	43.79813	1
SMAD1	1	47.75474	50.30201	1	1	51.0743	1	1	1	57.40823	1	70.85246	53.04062	1	1	1	1	333.0748	86.5339	1
SMAD3	1	1	1	1	1	1	1	1	1	1	1	1	1	1	1	1	1	49.43534	1	1
SMAD9	1	60.6943	63.70079	51.77489	1	56.3823	1	1	1	63.81374	1	87.2144	70.77464	1	1	76.5319	1	1	1	1
SMAP2	240.7231	1	138.3948	1	45.98265	1	1	1	95.51899	1	62.6806	1	1	114.8909	97.80746	1	1	1	1	70.02264

SMC2	1	113.105	118.2871	1	1	64.75102	1	68.80861	1	224.0298	62.31214	423.5199	85.75967	1	1	113.7917	26.90383	18.14501	1	87.58009
SMC4	1	107.7286	78.44875	63.59635	51.38822	87.80151	1	141.9661	1	293.3563	1	947.8904	158.2928	60.89599	66.62445	123.0127	120.8011	44.07452	1	103.6483
SMC6	1	49.95712	49.85849	1	1	42.31441	1	101.8816	1	57.6103	1	56.20673	46.48865	1	1	1	110.0607	31.34091	1	1
SMCO4	1	1	1	1	1	1	1	1	64.74772	1	1	1	1	1	1	1	1	52.81068	1	1
SMG7	1	37.45788	1	46.35972	45.22322	32.42067	1	1	1	43.62169	1	37.89542	32.46977	1	1	1	1	24.42534	1	70.02264
SMG9	1	1	28.847	1	1	1	1	1	1	1	1	34.54649	1	1	1	1	1	1	1	1
SMIM10	1	1	1	1	1	1	1	82.92833	1	1	1	1	1	1	1	1	1	31.5165	1	1
SMIM13	1	31.37971	1	1	46.10178	36.87743	1	27.55574	126.2717	32.03659	1	34.76317	42.27469	1	1	1	1	1	1	1
SMIM15	105.5885	52.98167	56.1782	73.5159	84.80767	70.09044	166.9882	47.04765	1	68.57092	1	56.31828	53.8595	70.67753	78.71281	1	106.2917	53.39936	69.52501	103.6483
SMIM18	1	1	45.8547	1	1	1	1	1	1	1	1	1	1	1	63.1524	1	1	1	42.02827	1
SMIM29	107.789	1	1	1	1	1	1	1	1	1	1	1	1	1	1	1	1	1	1	70.02264
SMIM4	1	1	1	1	1	1	1	1	1	1	1	1	28.80861	1	1	1	1	1	1	1
SMO	1	1	1	1	1	1	1	1	1	28.59984	1	1	1	1	1	1	1	1	1	70.02264
SMOC1	1	239.8114	275.9245	91.4867	1	1	1	1	1	193.5857	1	138.0288	59.42323	1	1	1	1	1	1	1
SMOC2	1	1	1	1	1	1	1	59.2842	1	1	1	1	1	1	1	1	1	1	1	1
SMPD3	1	1	1	52.87199	79.25174	1	1	1	134.2518	1	77.5875	1	1	60.62814	105.9562	1	1	1	1	1
SMPD4	1	1	1	1	1	1	1	1	1	37.09429	1	51.44305	26.50731	1	1	1	1	14.71756	1	1
SMTN	1	1	1	1	1	1	1	78.01441	1	1	1	1	1	1	1	1	1	106.7407	1	1
SMYD3	185.9608	1	32.68115	1	1	1	1	31.09289	1	1	1	1	1	1	1	1	1	30.02779	1	103.6483
SMYD4	1	1	1	43.12895	1	1	1	1	1	41.7894	1	28.07874	1	1	1	1	1	1	1	1
SNAI2	1	1	1	1	1	1	1	196.6246	1	1	1	1	1	1	1	1	1	1	1	1
SNAP25	1	1	52.68473	108.4179	102.3342	38.49482	1	1	128.1475	1	101.6426	1	1	201.0873	150.035	1	60.13169	1	107.2705	208.0679
SNAP47	1	1	1	1	44.27461	29.23164	1	1	1	26.50293	1	39.53994	33.96075	1	1	1	1	1	101.1364	1
SNAP91	1	1	70.97568	1	39.5675	1	1	1	177.0073	1	1	1	1	1	56.61234	1	111.7136	1	1	172.671
SNAPC2	1	1	34.33317	1	1	1	1	39.4376	1	1	1	1	1	1	1	1	1	1	1	103.6483
SNAPIN	1	62.37149	64.70104	1	1	78.87991	1	49.34143	1	64.81311	1	65.53287	54.39564	1	1	96.70831	1	16.27588	1	1
SNCA	96.97554	1	1	1	111.2655	55.72499	155.6122	36.38331	251.5609	1	1	1	79.78608	1	122.5818	1	137.5597	21.70125	1	70.02264
SND1	1	1	62.18152	1	1	31.06651	1	36.01747	1	35.23044	1	31.85004	49.64468	1	1	59.22433	1	70.31293	1	1
SNHG18	1	1	1	1	1	1	1	73.60789	1	1	1	1	1	1	1	1	1	1	1	1
SNHG19	1	1	32.10601	1	1	1	1	1	1	1	1	1	35.79689	1	1	1	1	1	1	1
SNRNP35	1	34.90082	33.27678	54.74932	50.96101	42.34726	1	24.39128	1	64.00989	77.71729	48.59097	34.28585	1	61.85713	1	1	26.9834	1	1
SNRPA	1	43.51885	42.12008	1	1	45.46814	1	30.98331	1	64.97239	1	53.04619	39.71696	1	1	53.27115	1	28.60945	76.28022	1
SNRPA1	62.40303	84.33103	65.40441	67.514	69.43511	66.96749	1	67.69486	86.11626	95.18297	97.89388	98.43304	62.79297	93.52655	79.93835	99.54396	1	57.05285	1	206.2967
SNTA1	1	1	1	1	1	74.74708	1	1	1	1	1	1	31.00358	1	1	1	1	1	1	103.6483
SNTG1	1	60.24384	123.5518	59.0428	60.69355	55.63553	1	1	1	1	92.59345	1	108.4275	104.9986	1	77.68702	128.689	1	1	116.5001
SNUPN	1	1	1	1	1	27.73144	1	25.22906	1	1	1	28.90747	28.39502	1	1	1	1	1	40.47132	1
SNX10	1	1	52.50786	1	1	1	1	1	1	1	1	1	1	1	1	1	1	1	1	1
SNX14	1	41.90991	47.75793	45.65752	52.75822	53.61784	1	42.25181	1	1	1	27.53094	41.505	1	1	1	1	30.26846	1	1
SNX22	1	1	144.6676	1	1	1	1	1	1	1	1	1	1	1	1	1	1	1	1	1
SNX29	106.1546	1	1	1	1	24.69518	1	1	1	43.42859	1	24.28095	23.60703	1	64.50406	1	1	29.72235	1	1
SNX7	1	46.45479	36.23196	78.66623	143.0881	54.73736	170.47	1	1	1	1	45.66347	37.65437	1	90.30171	59.93626	114.5936	31.11273	100.171	206.2967
SOAT1	1	1	1	1	1	158.7637	1	1	1	1	1	38.43274	52.91078	1	1	1	1	1	1	1
SOBP	1	77.27383	126.615	111.4188	221.8215	35.76691	247.4906	42.85043	946.2171	57.4034	151.5987	36.91322	57.51192	99.37014	400.4061	97.49802	250.5374	1	103.6311	415.1358
SOCS1	1	1	1	1	1	70.20987	1	85.62161	1	1	1	1	28.93915	1	1	1	1	52.56211	1	1
SOCS3	1	104.8275	1	1	1	190.1222	1	459.7259	1	1	1	1	212.2382	1	1	137.695	1	1049.159	1	1
SOCS5	1	37.82056	1	1	1	1	1	1	1	1	1	1	1	1	1	1	1	20.50998	1	70.02264
SOCS6	313.7852	33.64253	37.70416	1	1	1	1	1	1	1	1	1	26.49167	1	1	1	1	1	1	1
SOCS7	1	1	28.31843	60.86105	61.01372	28.62618	1	1	122.3237	38.51832	1	41.01666	32.49714	1	63.56795	1	1	1	105.2973	103.6483
SOD2	383.9255	45.07092	1	1	1	56.47515	1	114.9373	1	34.26798	1	31.44457	76.52022	1	1	1	1	479.6463	1	1
SORBS2	1	46.72913	60.74197	559.8355	328.2385	39.54984	193.769	69.09231	302.7073	66.0082	1	73.26543	56.88858	68.13294	209.326	108.9659	153.6841	110.4212	441.9532	1
SORCS1	1	1	1	1	1	1	1	1	432.4501	1	1	1	1	1	1	1	1	1	1	1
SORCS3	1	1	1	1	1	1	1	1	1	1	89.11646	1	1	1	1	1	1	1	1	1
SORD	1	1	1	1	1	40.26968	1	1	1	1	1	28.75394	1	1	1	1	1	1	1	1
SORL1	66.5928	1	1	113.2855	128.1168	1	1	1	1	1	1	1	1	1	175.309	1	329.5554	1	196.2933	206.2967
SOWAHC	1	77.37098	28.88219	47.48614	58.93241	66.05156	1	74.42223	1	1	97.14923	48.58283	71.63088	90.49951	1	1	1	1	1	70.02264
SOX1	1	75.19648	1	1	1	97.7888	1	1	1	117.8277	284.1739	101.8253	70.94971	456.7322	1	90.61169	1	1	1	1
SOX10	1	1	317.75	1	1	1	1	1	1	1	1	1	1	1	1	1	1	1	1	1
SOX12	1	57.16086	64.77436	88.87716	121.985	58.35971	115.6925	26.51397	69.69594	46.94536	102.058	50.85068	48.32704	102.5609	114.0424	1	1	1	133.5286	70.02264
SOX15	1	45.24139	40.77859	1	1	30.61111	1	1	1	1	1	1	35.60783	1	1	1	1	1	81.56624	1
SOX17	1	1	1	1	1	1	1	1	1	1	1	1	1	1	1	1	1	540.1698	1	1
SOX18	1	1	1	1	1	1	1	1	1	1	1	1	1	1	1	1	1	706.6241	1	1
SOX2	1	1168.776	897.3894	105.3172	1	1170.376	1	1	1	786.8247	645.2701	934.5218	1123.34	450.7264	1	736.0708	1	1	1	1
SOX21	1	93.40092	1	1	1	115.0797	1	1	1	50.08227	1	69.10577	76.82582	1	1	58.75116	1	1	1	1
SOX3	1	51.94794	1	1	1	212.7826	1	1	1	32.76175	1	81.87199	166.4502	1	1	129.7318	1	1	1	1
SOX6	1	232.7403	650.6461	1	1	236.8363	1	1	1	173.1921	1	117.1113	236.9692	292.8324	1	287.7397	1	1	1	1
SOX7	1	1	1	1	1	1	1	1	1	1	1	1	1	1	1	1	1	183.0805	1	1
SOX8	1	122.9514	506.8942	1	1	44.2085	1	1	1	39.13656	70.93137	1	1	1	1	1	1	1	1	1
SOX9	1	1116.197	392.866	240.182	180.1437	1245.349	1	1	1	379.5384	283.0442	552.4657	1224.698	133.2228	1	580.599	1	1	61.46049	1
SP100	71.05832	1	1	1	1	1	1	44.86889	1	1	1	1	1	1	1	1	1	61.38599	1	1
SP110	1	33.84187	1	1	1	36.34948	1	1	1	1	1	1	27.09921	1	1	1	1	1	1	1
SP4	1	47.83112	1	66.92463	68.73782	48.61782	1	1	1	54.79132	116.8068	50.99862	54.66855	101.9034	61.59736	53.2898	1	24.01257	58.25674	1
SP5	1	50.38501	1	1	1	1	1	1	1	1	1	1	1	1	1	1	1	1	1	1
SP8	1	1	1	1	1	31.24129	1	1	1	1	193.7034	24.94053	1	1	1	1	1	1	1	1
SP9	1	1	1	1	1	1	1	1	1	1	472.9189	1	1	642.3494	1	1	1	1	1	1
SPA17	1	1	1	1	1	1	1	1	1	1	1	35.75189	81.62128	1	1	1	1	1	1	1
SPACA9	1	1	1	1	1	1	1	1	1	1	1	1	35.70495	1	1	1	1	1	1	70.02264
SPAG1	1	1	1	1	1	1	1	1	1	1	1	1	104.2281	1	1	1	1	1	1	1
SPAG5	1	1	1	1	1	1	1	1	1	1	1	84.30028	1	1	1	1	1	1	1	1
SPARC	1	185.463	101.6432	1	1	191.6322	1	3599.079	1	1	1	70.73162	376.3015	1	33.02125	89.84232	1	1349.071	1	1
SPARCL1	1	431.8076	1	1	1	64.22454	1	984.3927	1	1	1	1	1	1	1	1	1	202.1916	1	1
SPATA13	1	39.77327	1	1	1	90.85296	1	1	1	77.84314	1	66.92402	76.52047	1	1	80.31907	1	1	1	1
SPATA17	1	1	1	1	1	1	1	1	1	1	1	1	42.83661	1	1	1	1	1	1	1
SPATA2	1	1	1	1	1	1	1	1	1	1	1	1	27.01364	1	1	1	1	1	1	1
SPATA2L	1	1	1	1	1	1	1	1	1	1	1	1	41.28586	1	1	1	1	1	1	1
SPATA33	1	1	1	1	1	1	1	1	61.602	32.74558	1	47.41265	80.82652	1	1	1	1	24.90829	1	139.0453
SPATA5	1	1	1	1	1	1	1	1	1	29.02614	1	31.76463	1	1	1	1	1	1	1	1
SPATA6	1	155.1705	161.7496	1	1	63.0814	1	27.35559	1	1	1	44.1074	64.94295	1	1	47.99352	1	1	1	1
SPATA7	1	1	1	1	40.63058	27.82668	1	1	1	1	64.5352	1	51.58515	73.56392	1	1	1	1	1	1
SPATC1L	1	1	62.9765	1	1	1	1	1	1	1	1	1	22.2508	1	1	1	1	21.82758	1	1
SPATS2L	226.9498	41.45005	1	1	1	61.92394	1	459.3398	1	1	1	28.15177	36.76043	108.0003	1	1	1	55.55008	1	1
SPC24	1	1	1	1	1	1	1	1	1	1	1	88.4921	1	1	1	1	1	1	1	1
SPC25	1	1	1	1	1	1	1	1	1	27.5704	1	146.473	1	1	1	1	1	1	1	1
SPDL1	1	1	1	1	1	1	1	1	1	1	1	58.48715	1	1	1	1	1	16.46461	1	1
SPEF1	1	1	1	1	1	1	1	1	1	1	1	1	40.17788	1	1	1	1	1	1	1
SPEF2	1	1	1	1	1	1	1	1	1	1	1	1	68.09702	1	1	1	1	1	1	1
SPG11	1	43.31164	38.25497	48.11691	1	44.8855	1	33.85925	1	51.17826	1	30.37248	35.50969	1	1	1	1	26.48966	65.85771	1
SPHK1	1	1	1	1	1	1	1	1	61.36094	1	1	1	1	1	1	1	1	1	1	1
SPHKAP	1	1	1	1	1	1	1	1	127.9422	1	1	1	1	1	60.26753	1	1	1	1	1
SPI1	201.7005	1	1	1	1	1	1	1	1	1	1	1	1	1	1	1	1	1	1	1
SPICE1	1	1	1	1	1	1	1	1	1	1	1	1	31.47956	1	1	1	1	1	1	1
SPIDR	1	1	1	1	1	1	1	1	1	36.58683	1	42.2609	29.1534	1	1	1	1	1	1	1

SPIN4	1	1	1	1	1	1	1	1	1	1	1	29.36601	1	1	1	1	1	1	1	1
SPINK8	1	1	1	1	1	1	1	1	1	1	1	1	1	1	1	1	1	275.8334	1	1
SPINT2	87.92276	1	1	1	67.27536	1	1	1	260.7188	1	1	1	1	1	108.9053	1	116.0644	1	105.877	70.02264
SPOCK1	1	1	1	1	1	1	1	1	137.0187	1	153.0969	1	1	1	1	1	1	1	1	116.5001
SPOCK2	1	1	1	1	1	25.39163	1	1	1	1	1	1	25.42557	1	1	1	1	142.484	1	1
SPON1	1	44.36196	1	1	1	1	1	1	1	1	1	1	30.0434	1	1	1	1	1	1	1
SPON2	1	1	1	1	1	1	1	791.968	1	1	1	1	1	1	1	1	1	1	1	1
SPP1	18030.69	1	1	1	1	1	1	1	1	1	1	1	1	1	1	1	1	1	1	1
SPRED2	1	58.77792	1	1	1	26.7925	1	54.83166	1	1	1	1	25.02888	1	1	1	1	25.52607	1	1
SPRN	1	1	1	1	1	1	1	1	89.72775	1	1	1	1	1	1	1	1	1	1	1
SPRY1	1	121.156	65.4526	1	1	42.63812	1	393.9686	1	1	1	1	1	1	1	1	1	1	1	1
SPRY2	1	342.7153	147.3049	1	1	228.7459	1	55.27217	1	110.4096	1	136.5775	126.8149	113.3532	1	80.64677	1	22.1778	1	1
SPRY4	1	62.71782	206.1225	1	1	1	1	128.3461	1	1	1	1	1	1	1	1	1	73.42339	1	70.02264
SPSB4	1	1	1	1	1	1	1	1	1	1	1	1	1	78.88566	1	1	1	1	1	1
SPTBN2	1	1	1	1	1	1	1	1	1	36.76778	1	1	1	1	73.86755	1	1	1	1	103.6483
SPTBN4	1	1	1	1	1	1	1	1	153.2652	1	1	1	1	1	1	1	1	1	1	1
SPTLC2	37.67191	1	83.5493	1	1	1	1	1	1	1	1	1	1	1	1	1	1	62.02614	1	1
SRARP	1	1	1	1	1	1	1	1	1	1	1	1	1	1	1	1	1	623.4767	1	1
SRBD1	1	1	1	1	1	27.17035	1	1	1	1	1	1	1	1	1	1	1	1	1	1
SRC	1	1	1	1	40.84697	1	1	35.66409	1	39.38393	1	24.48234	1	1	1	1	1	32.34594	1	1
SRCIN1	1	1	1	54.73554	68.11611	1	1	1	187.6628	26.78761	1	1	1	65.80332	76.46882	1	112.2967	1	88.02439	103.6483
SRD5A1	1	1	1	1	1	1	1	1	79.2741	1	1	1	1	1	41.82189	1	1	1	1	1
SRD5A3	1	1	1	1	1	1	1	39.69588	36.52711	1	1	1	1	1	1	1	1	104.2628	1	1
SREBF2	1	65.78094	56.14335	58.62831	83.57708	84.02855	1	26.74321	98.84245	95.97853	1	57.10589	64.70566	1	135.9707	84.56701	92.90915	44.63643	43.72304	116.5001
SRF	1	1	1	1	1	1	1	1	1	30.08724	1	30.36972	34.26311	1	1	1	1	1	1	1
SRGAP2B	1	65.07796	1	1	1	33.44206	1	1	1	1	1	38.44882	25.43365	1	1	1	1	36.83701	1	1
SRGN	919.8357	1	1	1	1	1	1	1	1	1	1	1	1	1	1	1	1	663.4997	1	1
SRM	116.6451	120.8729	244.6217	132.7475	298.881	97.82472	632.1682	81.79438	105.773	171.4282	104.9476	67.0123	95.13867	104.3321	519.338	169.8905	222.8717	117.9647	197.4793	878.5234
SRR	1	39.26779	1	1	1	50.85543	1	1	1	38.33096	1	48.07942	30.78379	1	49.18024	1	1	1	1	1
SRRM3	1	1	1	59.35789	71.79638	1	1	1	233.3679	1	108.474	1	1	72.34863	78.41684	1	186.9883	1	1	103.6483
SRRM4	1	1	1	192.0822	228.2649	1	273.2947	1	537.4638	1	246.7312	1	1	276.1519	199.1228	1	267.4294	1	221.5928	87.58009
SRSF12	1	1	65.65746	1	1	32.57817	1	1	1	28.88333	1	32.42734	27.58571	1	1	1	1	1	1	1
SS18L1	1	1	1	67.04428	64.72408	28.19374	1	1	1	40.22882	90.14763	38.54862	33.06992	78.12728	47.34592	1	182.6584	19.42819	1	1
SSH2	1	1	51.31897	45.83551	47.67512	1	1	60.51411	1	1	84.99255	29.49355	1	95.03066	53.57366	1	1	42.92526	1	1
SSPN	1	42.58532	1	1	1	47.62816	1	66.19236	1	1	1	1	69.4478	1	1	1	1	1	1	1
SSSCA1	1	32.5001	1	1	1	23.12223	1	1	1	1	1	1	22.25406	1	1	1	1	115.9439	1	1
SST	1	1	1	1	1	1	1	1	1	1	1	1	1	1275.566	1	1	1	1	1	1
SSTR2	1	1	1	803.8502	223.2877	1	1	1	491.9978	505.349	1	252.5908	1	1	1	1	1	1	221.8479	206.2967
SSX2IP	1	1	1	72.22593	85.91377	95.29181	1	1	66.27577	139.0493	1	104.2568	63.30086	1	53.45414	106.4529	131.2928	1	1	1
ST18	1	1	1	1	1	1	1	1	103.789	1	1	1	1	1	1	1	1	1	1	1
ST3GAL1	34.5303	1	1	1	51.98332	1	1	1	1	1	1	1	1	1	1	1	1	170.3846	1	1
ST3GAL3	1	1	1	1	1	1	1	1	1	1	1	30.8936	1	1	1	1	1	1	1	1
ST3GAL5	1	1	85.82732	1	1	28.85891	1	97.47949	1	1	1	1	1	1	1	1	1	1	1	1
ST3GAL6	1	1	48.94956	1	41.06291	1	1	1	1	1	1	1	1	1	1	1	1	1	1	1
ST5	1	88.49317	45.90737	1	1	82.57019	1	90.04588	1	42.22113	1	55.49791	181.9501	1	1	173.1721	1	16.74018	1	1
ST6GAL1	78.64316	1	1	1	41.01934	1	1	1	1	1	1	1	1	1	1	1	1	80.05221	54.73152	1
ST6GAL2	1	54.10355	1	136.7385	128.8755	34.04249	1	1	129.3099	1	1	1	1	1	78.47989	1	1	1	163.2998	70.02264
ST7	1	1	1	1	1	1	1	1	1	1	70.15701	30.3698	30.20613	85.23745	1	1	1	1	1	103.6483
ST7L	1	1	43.40119	1	1	1	1	1	1	30.02991	1	24.20453	1	1	1	1	1	1	1	1
ST8SIA1	1	76.89996	46.65545	207.6617	146.0295	111.5236	1	62.51683	1	35.75238	1	61.46924	63.34978	1	77.33783	93.57487	1	46.53549	196.2106	1
ST8SIA2	1	1	1	106.9937	118.76	1	1	1	62.35588	60.74284	108.1459	37.53942	1	140.4626	147.9293	50.55338	152.465	1	205.6464	292.8767
ST8SIA4	42.19334	1	1	1	1	1	1	27.81005	1	1	1	1	1	101.0853	51.42126	1	1	61.31985	1	1
ST8SIA5	1	1	1	1	1	1	1	1	1	1	217.0461	1	1	131.2899	1	1	1	1	1	1
STAB1	56.18469	1	1	1	1	1	1	1	1	1	1	1	1	1	1	1	1	55.21424	1	1
STAMBP	1	45.29344	44.20529	1	1	70.87453	1	51.07278	1	57.93606	1	50.86619	43.80936	1	45.25579	76.51732	1	23.90281	1	70.02264
STARD13	1	1	1	1	1	1	1	179.5616	1	1	1	1	1	1	1	1	1	27.80709	1	1
STARD3	1	1	1	1	1	1	1	1	1	1	1	1	1	1	1	1	1	56.04371	1	1
STARD4	1	38.29915	72.88129	1	1	59.90499	1	46.82891	1	35.4617	1	26.22846	33.6888	1	1	1	1	221.3998	1	1
STARD4.AS	1	1	1	73.40412	68.40081	1	1	1	237.195	31.85452	77.45057	1	1	83.79777	74.35649	1	88.94203	41.43372	1	1
STIL	1	1	1	1	1	1	1	1	1	1	1	51.33481	1	1	1	1	1	1	1	1
STK17A	1	133.164	1	52.51924	1	100.5895	1	1	1	181.4801	1	163.5964	36.82001	1	1	60.31862	1	94.91473	1	1
STK17B	1	1	1	1	1	1	1	1	1	1	1	36.72211	1	1	1	1	1	1	1	1
STK19	1	1	38.94188	1	1	37.66698	1	29.71186	1	1	1	28.52349	40.60965	1	1	1	1	18.20822	79.18056	1
STK32A	1	1	48.95174	1	1	1	1	1	1	1	1	1	1	1	1	1	1	1	1	1
STK33	1	1	1	1	1	34.24553	1	1	1	1	1	1	64.04055	1	1	59.92316	1	1	1	1
STK39	1	1	49.50218	1	1	85.7775	1	111.0077	1	38.91129	1	54.31505	1	1	1	70.07435	1	25.38443	1	1
STMN2	601.0383	155.0438	146.8174	2716.446	4502.682	100.5362	6128.725	74.57654	3207.363	167.3783	3663.694	193.7297	84.779	4158.152	7545.16	478.6632	5189.338	372.0622	4084.765	7022.11
STMN3	1	73.72474	69.80284	72.51751	77.10819	56.3779	134.8933	1	97.52578	83.65767	95.70595	72.23338	31.90535	114.5335	63.11001	93.98914	125.0494	1	1	206.2967
STOM	53.99754	1	1	1	1	68.75157	1	270.3395	1	1	1	1	1	1	1	50.99346	1	74.44461	1	1
STON1	1	1	1	1	1	42.18045	1	1	1	1	1	1	1	1	1	1	1	1	1	1
STON2	1	49.5356	1	1	1	78.23865	1	1	1	49.25448	1	40.40429	106.4416	1	1	95.52356	1	1	1	1
STOX1	1	1	1	1	1	76.06911	1	1	1	1	1	25.97417	69.65217	1	1	82.52904	1	1	1	1
STOX2	1	63.50879	72.91559	100.8035	62.61013	113.375	1	1	58.06562	77.4306	127.012	79.41192	101.4191	87.22449	1	53.12205	1	16.20392	1	1
STRA6	1	1	1	1	1	1	1	1	1	1	1	1	1	1	1	1	1	207.5035	1	1
STRIP1	1	1	1	1	48.34058	1	1	1	1	1	1	25.30477	24.77546	1	53.57703	1	1	1	1	1
STRN	1	1	123.2513	1	1	32.58181	1	62.12654	1	33.6338	1	35.81938	1	1	1	1	1	37.27292	1	1
STUM	1	1	1	1	1	1	1	1	214.9235	1	1	1	1	1	1	1	1	1	1	1
STX1A	1	1	1	1	1	1	1	1	1	1	1	1	1	1	56.29437	1	1	1	1	1
STX1B	1	1	1	1	1	1	1	1	58.15111	1	1	1	1	1	1	1	1	1	1	103.6483
STX3	1	1	1	1	1	1	1	1	1	1	1	1	1	1	1	1	1	88.85839	1	1
STX4	68.16215	1	105.192	1	1	38.50901	1	47.35494	1	1	1	1	26.9322	1	1	1	1	25.31196	1	1
STX8	1	60.69536	41.11073	1	1	41.85846	1	30.22079	1	32.15023	73.42491	41.63188	33.91511	53.96775	1	37.44536	1	31.19055	1	1
STXBP1	1	32.50423	45.2182	170.2562	198.2677	31.10217	134.1067	45.35561	237.7367	69.45392	105.9305	61.27681	32.56858	123.9598	207.3941	68.07015	206.8561	158.2711	215.872	344.3419
STXBP4	1	35.32561	1	1	1	1	1	1	1	29.54511	1	25.24384	24.45649	1	1	1	1	1	1	1
STXBP5	1	1	1	1	41.37377	1	1	1	1	1	1	1	1	1	86.76292	1	1	27.06635	1	1
STXBP5L	1	1	1	1	1	1	1	1	1	1	1	1	1	74.4753	1	1	1	1	1	1
STXBP6	1	1	1	1	1	99.09401	1	1	1	105.2931	1	79.11539	43.22212	1	1	93.78062	1	55.4585	1	1
SUCLG2	72.01422	71.33116	1	1	1	43.84492	1	55.35325	1	1	1	24.44431	1	1	1	1	1	33.70243	1	1
SULF2	1	1	125.5569	1	1	1	1	1	95.34555	1	1	1	1	1	1	1	1	1	1	1
SULT1A1	1	1	1	1	1	1	1	1	115.7515	1	1	1	1	1	1	1	1	1	1	1
SULT1C4	1	29.63966	1	1	1	41.03381	1	124.3535	1	1	1	1	68.6356	1	1	1	1	247.3759	1	1
SULT1E1	1	1	1	1	1	1	1	214.3713	1	1	1	1	1	1	1	1	1	1	1	1
SULT4A1	1	1	1	1	1	1	1	1	95.20736	1	1	1	1	1	53.08247	1	132.3519	1	1	103.6483
SUN2	1	1	1	69.61828	73.57551	1	1	39.24931	1	32.59187	85.4545	61.68499	1	58.118	33.98953	1	1	31.39926	101.6248	1
SUPT7L	1	1	1	1	1	24.91185	1	1	1	32.03396	1	1	21.39005	1	1	1	1	27.21669	78.5916	70.02264
SURF2	1	26.0875	1	1	1	33.67359	1	1	1	1	1	31.63175	39.08191	1	48.66232	1	1	1	1	70.02264
SUSD3	65.96734	1	1	1	1	1	1	1	1	1	1	1	1	1	1	1	1	1	1	1
SUV39H2	1	1	32.14971	1	1	34.8911	1	1	1	52.48275	1	39.14553	35.72924	1	1	1	1	1	1	1
SVIL	1	1	1	1	1	31.08359	1	221.0335	1	48.44231	1	26.33018	1	1	1	1	1	62.03488	1	1
SWAP70	96.71307	96.11082	84.43355	1	1	36.1447	1	75.63544	1	1	1	30.78333	39.86451	1	1	1	1	96.17016	1	1
SYBU	1	1	1	1	1	32.12921	1	1	1	1	1	1	22.83613	1	1	1	143.9709	1	1	1

SYDE1	1	1	1	1	1	1	1	85.57701	1	1	1	1	1	1	1	1	1	27.30701	1	1
SYK	102.1865	1	1	1	1	1	1	1	1	1	1	1	1	1	1	1	1	1	1	1
SYMPK	1	52.1468	1	1	1	30.68889	1	1	1	43.00332	1	24.98385	33.70123	1	1	1	61.68173	1	1	103.6483
SYN1	1	1	1	1	1	1	1	1	77.3891	1	1	1	1	1	1	1	1	1	1	1
SYN3	1	1	1	1	1	1	1	1	101.0712	1	1	1	1	1	1	1	1	1	1	1
SYNC	1	39.243	70.63304	76.84345	84.85579	29.70304	138.0279	1	1	44.37819	88.68654	38.61536	46.19094	82.32749	65.4589	92.22148	210.7941	1	55.35015	1
SYNGAP1	1	1	1	1	45.75873	31.38518	1	46.43844	1	1	1	1	29.7831	1	1	1	1	1	1	70.02264
SYNGR2	107.5913	1	1	1	1	1	1	1	1	1	1	1	1	1	1	1	1	44.99179	1	1
SYNGR3	1	1	1	1	1	1	1	1	91.57301	1	1	1	1	1	1	1	1	1	1	1
SYNM	1	1	1	1	1	1	1	366.4341	1	1	1	1	125.8722	1	1	1	1	73.75966	1	1
SYNPO2	1	1	1	1	1	1	1	59.40641	1	1	1	1	1	1	1	1	1	1	1	1
SYT1	70.83039	88.27919	153.0117	452.9795	540.1441	36.82121	519.4032	1	1547.05	61.78123	589.4358	58.58704	1	991.391	570.8605	91.45506	1067.277	53.4245	506.0459	620.4325
SYT13	1	1	1	1	1	1	1	1	248.4483	1	1	1	1	1	1	1	1	1	1	70.02264
SYT14	1	1	1	49.92386	63.74075	29.71776	1	1	68.49694	1	133.9256	1	1	107.1451	79.98112	1	112.3672	1	1	1
SYT16	1	1	1	1	1	1	1	1	55.82259	1	1	1	1	1	1	1	1	1	1	70.02264
SYT17	1	1	119.1931	1	1	1	1	1	1	1	1	1	28.1218	1	1	1	1	1	1	1
SYT2	1	1	1	1	1	1	1	1	1	42.07246	1	1	1	1	1	1	1	1	1	1
SYT4	1	1	1	123.1964	334.6978	1	413.9328	1	282.9533	47.76188	1	33.26571	1	65.30418	598.5041	1	458.2217	1	170.9842	277.0906
SYT5	1	1	1	1	1	1	1	1	82.75863	1	1	1	1	1	1	1	92.28928	1	1	70.02264
SYT6	57.76526	1	1	1	1	1	1	1	293.2367	1	1	1	1	1	1	1	1	1	1	1
SYTL2	1	1	1	1	1	1	1	116.2954	1	1	1	1	1	1	1	1	1	1	1	1
TACC2	1	81.44912	75.53746	83.77095	109.1566	47.86251	1	1	1	1	70.61522	34.10942	1	1	71.23733	1	1	1	114.5587	1
TACC3	1	1	1	1	1	1	1	1	1	1	1	164.7062	1	1	1	1	1	1	1	1
TADA1	1	1	1	1	1	1	1	1	1	40.18592	1	22.45273	1	1	1	1	1	1	1	1
TADA2A	1	1	1	1	1	1	1	1	1	36.55797	1	35.23337	1	1	1	1	1	1	1	1
TAF1	1	41.62513	1	43.45364	1	30.56419	1	32.27971	1	39.75067	1	29.69356	33.94198	1	1	1	1	73.73142	1	1
TAF13	1	53.1713	1	1	1	45.467	1	42.73818	1	1	1	31.32212	56.28021	1	1	1	1	61.72913	1	1
TAF1A	1	1	1	1	1	1	1	1	1	32.88394	1	28.36914	1	1	1	1	1	1	1	70.02264
TAF1B	1	50.03349	102.2468	1	1	46.89848	1	1	1	39.11473	1	42.8863	50.71774	1	1	58.70424	1	1	1	1
TAF1C	1	1	1	1	1	1	1	1	1	1	1	1	1	1	1	1	92.05504	19.20881	1	70.02264
TAF2	1	1	1	46.37351	50.97249	38.14623	1	1	1	46.54635	1	35.16324	31.12771	1	1	1	1	30.19293	142.7189	1
TAF5	1	1	1	1	1	1	1	1	1	1	1	26.39242	1	1	1	1	1	1	1	1
TAF5L	1	1	31.95264	1	1	1	1	1	1	1	63.2141	1	1	1	1	1	1	1	1	70.02264
TAF8	1	32.35969	1	1	1	1	1	1	1	1	1	24.26218	1	1	1	1	1	1	1	1
TAGAP	125.224	1	1	1	1	1	1	1	1	1	1	1	1	1	1	1	1	1	1	1
TAGLN	1	1	1	1	1	1	1	333.4983	1	1	1	1	1	1	1	1	1	1	1	1
TAGLN2	1	118.6582	1	67.7339	1	290.3232	1	474.8611	1	98.59091	1	276.3505	449.6042	1	1	243.6302	1	323.9078	1	1
TAGLN3	1	68.43338	147.5784	369.3166	371.3751	48.53845	438.9157	1	189.2114	77.50863	366.1668	99.80044	88.81914	437.3032	374.6497	88.55938	216.7956	40.41371	604.7037	344.3419
TAMM41	1	1	1	1	1	1	1	1	1	1	1	1	1	1	1	1	1	1	48.01606	1
TARBP1	1	1	35.15178	1	1	33.49448	1	1	84.55702	32.50681	1	1	31.32033	1	1	66.76192	1	15.35997	1	1
TARS	61.23276	78.03697	71.93417	1	1	78.97489	1	87.73298	1	77.07326	1	64.87057	84.63179	71.81901	1	93.32919	1	72.09477	1	172.671
TARSL2	1	1	87.22738	1	1	37.28083	1	1	104.4244	38.59914	1	1	1	1	1	1	1	1	1	1
TAX1BP3	1	1	1	1	1	1	1	62.22229	1	28.37849	1	24.93316	1	1	1	1	1	144.0771	1	1
TBC1D12	59.66098	1	1	1	1	28.51261	1	33.60145	1	1	1	26.13708	1	1	1	1	1	1	1	1
TBC1D14	1	53.73322	67.71342	53.767	73.63103	46.57281	125.701	1	96.97745	47.00347	61.59147	38.7148	47.9988	72.13823	73.51882	65.65144	1	1	72.90547	139.0453
TBC1D22A	1	1	1	1	1	1	1	1	1	1	1	1	1	1	1	1	1	100.3837	1	1
TBC1D24	1	1	60.92287	1	39.89508	1	1	1	1	27.96704	1	29.21285	1	1	1	1	1	1	1	1
TBC1D30	1	1	1	1	1	1	1	1	1	1	1	1	1	1	54.64984	1	1	1	1	1
TBC1D31	1	1	1	1	1	1	1	1	1	1	1	42.03762	1	1	1	1	1	1	1	1
TBC1D32	1	1	1	1	1	1	1	1	105.835	1	1	1	1	1	1	1	1	1	1	103.6483
TBC1D4	42.31379	1	1	1	1	1	1	1	1	1	1	1	1	1	1	1	1	62.90659	1	1
TBC1D9	1	1	1	1	1	1	1	1	1	1	1	1	1	1	1	1	1	60.27523	1	103.6483
TBCC	1	27.7067	1	113.0443	138.5862	32.88111	1	36.22535	1	76.3005	165.1334	55.84627	43.06385	168.8106	50.11038	61.57559	1	117.3048	80.2003	103.6483
TBCD	1	36.05927	1	1	1	31.19173	1	1	1	33.70525	1	43.20628	1	1	1	1	94.93687	25.66713	1	1
TBCK	1	37.04094	1	54.3108	45.03495	29.35639	1	1	1	1	1	1	28.4914	1	73.37974	1	1	1	1	1
TBK1	1	1	1	1	1	21.45393	1	1	1	39.06814	1	27.76893	1	1	1	1	1	30.18106	1	1
TBL1X	1	1	1	1	1	46.36012	1	48.80943	1	1	1	30.59963	36.57778	1	1	1	1	1	1	1
TBR1	1	1	1	229.8164	206.69	1	203.1249	1	656.1361	52.28146	1	33.91157	1	1	197.9575	1	123.9077	1	346.4626	206.2967
TBRG4	1	38.38836	42.14257	1	1	1	1	25.2584	1	31.38335	1	1	1	1	1	1	1	1	1	70.02264
TBX18	1	1	1	1	1	1	1	57.98833	1	1	1	1	1	1	1	1	1	1	1	1
TBX2	1	1	1	1	1	1	1	272.0229	1	1	1	1	1	1	1	1	1	1	1	1
TBX3	1	1	1	1	1	1	1	68.20882	1	1	1	1	1	1	1	1	1	108.468	1	1
TBXA2R	1	1	1	1	1	1	1	84.0194	1	1	1	1	1	1	1	1	1	1	1	1
TBXAS1	81.82774	1	1	1	1	1	1	1	1	1	1	1	1	1	1	1	1	1	1	1
TC2N	1	1	1	1	1	32.73836	1	1	1	1	1	1	1	1	1	1	1	1	1	1
TCAIM	1	1	1	1	1	45.37433	1	1	1	39.79462	1	40.95395	43.32403	1	1	37.40494	1	24.00283	1	1
TCEAL1	1	54.86113	51.42234	1	53.06657	47.76299	1	31.15126	149.1124	29.36736	1	27.58713	53.00063	1	57.65084	1	1	22.53354	44.47283	103.6483
TCEAL2	1	110.9154	169.9841	140.5483	196.8307	147.9572	246.2804	1	306.3417	107.7208	211.1364	135.1174	121.6806	181.8873	214.7652	95.77737	216.0732	1	276.4948	379.7389
TCF15	1	1	1	1	1	1	1	1	1	1	1	1	1	1	1	1	1	135.5674	1	1
TCF19	1	1	1	1	1	1	1	1	1	1	1	26.88294	1	1	1	1	1	1	1	1
TCF20	1	1	1	1	49.97231	1	1	1	1	34.37763	74.51124	1	1	1	65.37894	1	1	14.41458	1	1
TCF7L1	1	53.08489	70.31699	1	1	40.87579	1	67.94172	1	1	1	1	44.64827	1	1	1	1	51.72813	1	1
TCF7L2	1	91.98955	348.1519	1	1	59.97565	1	39.30181	1	1	1	34.7974	48.87376	1	1	1	1	32.20674	1	1
TCHP	1	1	1	1	1	23.73426	1	1	1	37.9572	1	28.21244	1	1	1	1	1	1	1	1
TCIM	1	148.5142	1	1	1	471.364	1	177.9598	1	89.55206	1	150.1682	180.0316	1	1	219.4908	1	86.77831	1	1
TCOF1	1	33.10563	1	1	41.61763	1	1	32.5201	1	41.56771	1	42.75537	1	1	1	1	1	23.71754	1	103.6483
TCTEX1D1	1	1	1	1	1	1	1	1	1	1	1	1	101.332	1	1	1	1	16.52188	1	1
TCTN1	1	32.55566	1	1	1	50.17558	1	32.91417	1	1	1	25.82407	112.4084	1	1	89.53781	1	1	1	1
TDP2	61.00103	1	1	46.95634	48.75303	28.11561	1	1	1	40.65559	64.08652	30.38585	1	71.20687	1	1	1	1	52.96525	1
TDRD7	1	1	1	1	1	1	1	1	1	1	1	1	1	1	41.84574	1	1	1	1	70.02264
TDRP	1	1	1	1	1	1	1	1	1	1	1	1	1	1	1	1	1	65.48813	1	1
TECPR1	1	1	1	1	1	28.84087	1	1	1	1	1	1	1	1	1	1	1	27.93537	1	1
TECPR2	1	33.17257	57.37001	1	1	38.25374	1	1	1	1	1	1	1	1	1	1	1	23.38019	1	1
TEDC1	1	1	1	1	1	1	1	1	1	1	1	29.48439	1	1	1	1	1	1	1	1
TEDC2	1	1	1	1	1	1	1	1	1	37.23833	1	1	1	1	1	1	1	1	1	70.02264
TEK	1	1	1	1	1	1	1	1	1	1	1	1	1	1	1	1	1	117.9399	1	1
TEKT1	1	1	1	1	1	1	1	1	1	1	1	1	59.46063	1	1	1	1	1	1	1
TEKT2	1	1	1	1	1	1	1	1	1	1	1	1	26.77085	1	1	1	1	1	1	1
TENM1	1	1	62.34608	1	1	1	1	1	150.829	1	1	1	1	64.11883	1	1	1	1	1	1
TENM2	1	1	1	1	1	1	1	1	109.7072	1	1	1	1	70.16418	1	1	73.62874	1	1	1
TENM3	1	1	1	1	1	1	1	1	150.8988	1	1	1	1	1	1	1	1	1	1	1
TENM4	1	1	1	150.3614	180.6883	33.17746	293.4151	1	1	46.00845	1	30.432	1	1	265.111	1	267.6551	1	122.803	70.02264
TESC	1	1	1	1	1	1	1	290.9557	1	1	1	1	1	1	1	1	1	52.50015	1	1
TESK1	1	1	1	1	1	27.21182	1	1	1	1	1	1	1	1	1	1	1	1	1	1
TET2	1	41.63926	50.34211	48.80169	53.28634	31.37891	1	53.62031	1	35.41046	72.75196	36.29594	27.24388	124.8082	53.42802	1	1	32.06057	67.21724	1
TET3	1	1	1	1	1	1	1	1	1	1	71.25458	1	1	69.98429	1	1	1	1	1	1
TEX2	1	1	1	1	1	1	1	28.06203	82.04278	1	1	1	1	1	1	1	1	1	1	1
TEX26	1	1	1	1	1	25.60803	1	1	1	1	1	1	37.92894	1	1	1	1	1	1	1
TEX30	1	1	1	1	1	1	1	1	1	55.48586	1	70.40276	29.22485	1	1	1	1	1	1	1
TEX9	1	1	1	1	1	1	1	1	1	63.90632	1	46.13728	72.13187	1	1	1	1	1	1	1
TF	1	1	114.0496	1	1	1	1	1	1	1	1	1	1	1	1	1	1	1	1	1

TFAP2C	1	1	1	1	1	175.5173	1	1	1	145.0899	1	97.12777	126.9905	1	1	137.7147	1	1	1	1
TFB2M	1	1	1	1	1	1	1	1	1	1	1	1	1	1	1	1	1	23.18316	106.887	1
TFEC	55.50849	1	1	1	1	1	1	1	1	1	1	1	1	1	1	1	1	1	1	1
TFPI	1	67.38433	1	1	1	239.3049	1	1237.513	1	1	1	57.64707	33.8708	1	1	74.42817	1	113.1168	1	1
TFRC	1	47.19145	1	1	1	44.51296	1	49.02248	1	42.55813	1	31.6445	37.52567	1	1	1	1	799.997	1	1
TGFB1	164.3507	1	1	1	1	1	1	131.461	1	1	1	1	1	1	1	1	1	55.87054	1	1
TGFB1I1	1	1	1	1	1	25.34762	1	241.1714	1	1	1	1	27.10375	1	1	1	1	54.78058	1	1
TGFB2	1	119.7134	1	1	1	45.33712	1	31.94714	1	1	1	1	162.2263	1	1	1	50.84221	1	1	346.1132
TGFBI	1	1	1	1	1	1	1	52.98159	1	1	1	1	1	1	1	1	1	1	1	1
TGFBR2	81.87051	1	1	1	1	1	1	259.7671	1	1	1	1	1	1	1	1	1	111.7868	1	1
TGFBRAP1	1	1	1	1	1	1	1	1	1	25.75137	1	1	1	1	50.03757	1	1	25.63853	1	1
TGIF1	50.99226	135.7821	1	1	1	105.4084	1	37.76656	1	49.06979	1	109.5521	179.9637	1	1	103.8126	1	65.55873	1	206.2967
TGIF2	1	50.49726	1	1	1	46.95972	1	1	1	80.01624	1	65.89991	55.85793	1	1	46.12585	1	23.9161	1	1
TGM2	1	1	1	1	1	1	1	1	1	1	1	1	1	1	1	1	1	334.808	1	1
THAP2	1	1	1	43.05048	45.55042	44.57822	1	1	1	34.28741	1	30.81081	28.84444	1	1	1	1	1	51.19226	1
THBD	1	1	1	1	1	1	1	1	1	1	1	1	1	1	1	1	1	98.16929	1	1
THBS1	1	1	1	68.09576	60.31121	1	1	46.24825	1	1	1	1	1	1	1	1	1	1	1	139.0453
THBS2	1	1	1	1	1	54.99481	1	1	1	1	1	1	1	1	1	1	1	1	1	1
THG1L	1	1	1	1	1	1	1	1	1	1	1	1	27.01625	1	1	1	1	1	1	1
THOC3	1	1	50.14988	1	1	23.87334	1	1	1	73.74683	1	36.95197	24.9399	1	1	1	1	23.31524	1	70.02264
THOC5	1	1	1	1	1	1	1	1	1	32.54549	1	23.91728	1	1	1	1	1	1	1	1
THRB	1	1	1	1	1	1	1	1	1	1	267.4227	1	1	1	1	1	1	1	1	1
THSD1	1	1	1	1	1	39.19503	1	1	1	1	1	1	1	1	1	1	1	119.4679	1	1
THSD7A	1	69.1892	58.01898	152.7722	128.3287	84.83378	1	1	644.9066	32.23182	155.9222	52.46916	1	111.4066	1	101.5502	1	111.5708	239.6793	70.02264
THY1	1	1	235.4244	1	52.76378	22.64022	1	628.3458	260.8449	1	57.59994	1	1	114.6135	69.14309	1	1	1	1	1
TIAM1	1	1	1	1	1	1	1	1	1	1	1	1	1	1	1	1	1	92.19249	1	1
TIAM2	1	111.2001	1	82.76929	146.1497	35.53711	1	1	1	43.12561	186.1086	42.26323	1	103.2824	151.2491	1	263.7651	1	205.7786	87.58009
TICRR	1	1	1	1	1	1	1	1	1	1	1	30.03885	1	1	1	1	1	1	1	1
TIE1	1	1	1	1	1	1	1	1	1	1	1	1	1	1	1	1	1	197.0326	1	1
TIFA	1	1	1	1	1	1	1	1	1	50.16629	1	37.97353	26.60615	1	1	1	1	1	1	70.02264
TIGAR	63.77426	1	79.40898	1	1	1	1	1	1	1	1	1	1	1	1	1	1	1	1	1
TIMELESS	1	1	1	1	1	1	1	1	1	62.89492	1	75.52477	1	1	1	1	1	1	1	1
TIMM10B	1	46.54175	57.72345	1	1	47.36824	1	1	1	58.43725	1	35.7506	50.67939	1	1	1	97.56341	31.84643	1	1
TIMM17A	86.86243	90.12548	106.0892	69.46365	75.75958	52.23643	1	68.84583	101.7097	74.77528	81.45756	75.40155	56.20308	82.81761	106.9717	76.02449	1	47.93568	84.16228	185.5228
TIMM21	1	1	1	1	1	25.79658	1	1	1	40.53466	1	27.9426	1	1	1	1	1	1	1	1
TIMM22	1	1	59.96213	1	1	33.31149	1	26.34816	1	36.94499	1	37.95753	29.18166	1	1	1	84.16934	56.81726	1	1
TIMM29	1	35.3986	1	1	1	31.33015	1	1	1	1	1	40.97927	29.66891	1	1	1	1	27.04735	1	70.02264
TIMM50	1	59.33144	71.73473	1	1	27.28365	1	1	1	48.88459	1	50.32072	29.2119	1	1	1	1	19.49751	1	1
TIMP1	1	117.1925	60.66553	1	1	87.33857	1	306.2589	1	1	1	37.67181	64.09861	1	1	52.7558	1	385.7299	1	1
TIMP3	1	63.899	1	1	1	105.5413	1	154.8012	1	1	1	74.07907	143.0825	1	1	54.3238	1	147.9809	1	1
TIMP4	1	1	83.42088	1	1	1	1	1	1	1	1	1	1	1	1	1	1	1	1	1
TINF2	1	1	1	1	51.64765	43.84453	1	20.25114	1	40.50094	1	45.25106	26.5327	1	44.65403	1	1	29.20158	1	87.58009
TIPARP	69.88307	88.45478	1	1	1	54.98662	1	168.4557	1	50.47822	1	35.15322	67.94334	92.73638	1	1	1	53.71597	1	1
TIPIN	1	1	1	1	1	1	1	36.05187	1	75.32367	1	42.81598	29.52023	1	1	45.17959	1	1	1	1
TJP1	1	176.5294	177.2761	1	1	114.3259	1	543.0361	92.2724	81.53597	1	91.19377	114.4957	1	1	102.1614	1	474.6916	1	1
TK1	1	1	1	1	1	1	1	1	1	66.40223	1	83.63469	1	1	1	1	1	1	1	1
TKTL1	1	1	1	1	1	39.98029	1	1	1	1	1	1	1	1	1	1	1	1	1	1
TLE4	46.97401	114.3257	133.0855	71.31232	98.1767	191.1641	1	179.7383	1304.715	64.51267	1	95.3222	164.6991	1	125.8835	356.7009	147.0317	69.29909	1	1
TLK1	1	224.8554	45.00037	1	1	93.60262	1	1	1	74.78245	1	75.72592	70.19278	1	1	123.4146	1	43.17373	1	1
TLN1	213.388	47.11175	36.79608	1	1	62.94998	1	294.7724	1	47.08293	1	49.77753	52.28562	1	1	1	1	97.16442	1	1
TLN2	1	1	1	1	1	37.091	1	1	111.3859	1	1	1	35.61533	1	1	1	1	1	1	1
TLNRD1	46.87734	1	1	1	1	1	1	39.62048	1	1	1	1	1	1	1	1	1	97.57599	1	1
TLR1	52.49757	1	1	1	1	1	1	1	1	1	1	1	1	1	1	1	1	1	1	1
TLR4	141.059	1	1	1	1	1	1	1	1	1	1	1	1	1	1	1	1	30.1057	1	1
TM4SF1	1	1	1	1	1	1	1	1	1	1	1	1	1	1	1	1	1	590.3219	1	1
TM4SF18	1	1	1	1	1	1	1	1	1	1	1	1	1	1	1	1	1	309.2058	1	1
TM6SF1	67.07116	1	1	1	1	1	1	1	1	1	1	1	1	1	1	1	1	1	1	1
TM7SF2	1	47.55535	102.1646	1	1	88.79496	1	1	71.16037	1	1	1	46.99606	1	1	1	1	17.67087	1	70.02264
TMA16	1	67.12104	1	1	1	45.93983	1	1	1	85.92725	1	44.91915	44.02343	1	55.18974	43.03473	1	107.9867	1	1
TMBIM1	49.45041	1	1	1	1	1	1	46.9376	1	1	1	1	1	1	1	1	1	25.03882	1	1
TMCC1	1	1	123.6411	51.79662	54.52277	29.32245	1	32.50784	83.23263	1	99.38447	32.26695	32.35991	97.16962	48.78356	1	1	22.71115	1	70.02264
TMED3	1	1	63.79612	1	1	38.01002	1	1	1	45.15624	1	34.63755	35.81475	1	1	55.38069	1	29.7329	1	1
TMEFF2	1	1	93.77553	1	1	1	1	1	72.30316	1	83.239	1	1	1	1	1	1	1	1	1
TMEM100	1	1	70.77266	1	1	1	1	1	1	1	1	1	1	1	1	1	1	1	1	1
TMEM101	1	40.14123	1	1	1	1	1	21.09766	1	1	1	1	1	1	1	1	1	31.97922	1	103.6483
TMEM107	64.44432	1	1	1	1	43.422	1	25.58972	1	36.51544	1	60.10961	74.73345	1	1	1	1	1	1	1
TMEM109	1	1	1	1	1	1	1	119.9549	1	1	1	1	1	1	1	1	1	73.29694	1	1
TMEM120/	1	1	1	1	1	40.67454	1	1	1	1	1	1	1	1	1	1	1	101.1521	1	1
TMEM121	1	40.6392	134.5528	1	1	1	1	1	1	1	1	1	21.40857	1	1	1	1	1	1	1
TMEM123	88.45056	199.0888	97.44177	87.55105	1	164.1122	1	63.7696	1	251.1295	581.2938	196.4808	184.8309	502.3026	1	162.9366	1	330.9931	1	1
TMEM129	1	1	1	1	1	30.14047	1	1	1	1	1	1	1	1	1	1	1	1	1	1
TMEM131L	1	1	27.6352	47.74075	1	24.48115	1	1	1	46.58243	83.64296	39.89461	1	119.9301	1	1	1	1	76.38372	1
TMEM132E	1	149.3412	101.0165	75.26168	43.64809	580.4054	1	1	191.2759	120.5439	1	163.9397	142.2735	1	1	412.6092	1	1	61.23928	103.6483
TMEM141	40.40617	1	1	1	1	26.69785	1	70.17733	1	1	1	30.68269	24.08927	1	1	64.62997	1	49.56008	1	70.02264
TMEM144	90.63131	1	1	1	1	1	1	1	1	1	1	1	1	1	1	1	1	1	1	1
TMEM145	1	1	1	1	1	1	1	1	63.28028	1	1	1	1	1	1	1	1	1	1	1
TMEM147	118.3987	114.2631	165.9468	76.42173	64.52702	111.551	1	53.3502	79.25406	96.20369	1	74.70339	102.9379	1	66.20369	122.4889	1	59.72997	54.34125	308.945
TMEM150(	1	1	1	1	46.90958	41.1881	1	1	253.5514	1	1	1	1	1	1	1	234.4691	1	1	103.6483
TMEM151E	1	1	1	1	40.11548	1	1	1	1	1	1	1	1	1	1	1	1	1	1	70.02264
TMEM158	1	148.0754	63.50791	1109.414	293.3184	106.9913	274.3996	1	88.34896	1300.623	71.74894	509.4482	266.3974	1	141.5828	182.4128	192.0289	84.72511	720.952	87.58009
TMEM159	1	1	1	49.26734	1	25.08195	1	1	1	1	1	1	1	1	1	1	1	1	1	1
TMEM161/	1	1	35.84167	1	1	1	1	1	1	1	1	1	1	1	1	1	1	11.46241	1	103.6483
TMEM161E	1	1	1	52.28688	50.52847	31.74237	1	1	1	43.61433	1	34.72326	33.17891	1	1	1	1	1	1	1
TMEM161E	1	140.631	41.3178	326.9465	353.1137	254.1431	484.9926	1	323.7015	194.1443	532.1042	207.2483	263.0463	364.6267	297.2682	242.3684	178.5399	47.23509	240.5381	185.5228
TMEM163	1	1	225.9672	1	1	79.56416	1	1	1	1	1	1	48.77941	1	1	1	1	1	1	1
TMEM170E	1	55.05711	56.6568	1	1	57.72201	1	1	1	1	1	32.01874	46.53495	1	1	1	1	1	1	1
TMEM173	1	1	1	1	1	1	1	137.1226	1	1	1	1	1	1	1	1	1	108.1723	1	1
TMEM178/	1	1	1	1	1	1	1	1	73.76835	1	1	1	1	1	1	1	1	50.39628	1	1
TMEM178E	1	36.62501	45.45263	51.88855	50.86476	39.34371	1	1	1	51.16831	84.01285	34.16636	28.54633	88.48854	1	1	1	1	1	1
TMEM185/	1	1	1	1	47.58188	1	1	1	1	1	1	1	1	1	65.67318	1	59.75064	22.79927	50.97167	308.945
TMEM19	1	1	1	1	1	1	1	1	1	1	1	31.46301	1	1	1	1	1	1	1	1
TMEM192	1	1	1	1	54.73104	30.8677	1	1	1	1	1	1	29.70737	1	1	1	201.202	1	80.96494	70.02264
TMEM196	1	1	57.69631	1	41.50703	1	1	1	1	1	1	1	1	1	1	1	1	1	1	1
TMEM2	1	1	1	1	1	1	1	116.04	1	1	86.76041	1	27.39547	245.2889	1	1	1	197.0085	1	1
TMEM204	1	1	1	1	1	1	1	190.8372	1	1	1	1	1	1	1	1	1	184.6579	1	1
TMEM209	1	1	1	1	1	1	1	1	1	1	1	28.0128	1	1	1	1	1	1	1	1
TMEM216	1	1	1	1	1	37.86604	1	1	1	1	1	1	27.69854	1	1	1	1	1	1	1
TMEM218	1	46.56082	1	1	1	41.56282	1	49.91654	1	34.89894	1	40.57642	43.22452	1	1	66.58311	1	1	1	1
TMEM222	1	1	36.59958	1	1	29.40276	1	35.4489	1	34.17956	1	35.31224	27.49922	1	1	1	1	113.6893	50.35578	1
TMEM231	1	1	1	1	1	1	1	1	1	1	1	1	107.8638	1	1	1	1	1	1	1
TMEM237	1	36.09372	1	1	1	45.70901	1	1	1	78.58863	1	82.27606	60.28441	1	56.33154	1	1	1	1	1

TMEM243	1	35.29867	1	1	1	24.51312	1	1	1	1	1	1	1	1	1	1	1	79.68794	1	1
TMEM246	1	1	36.90245	1	1	1	1	1	1	1	1	1	1	1	1	1	121.7083	1	49.63405	70.02264
TMEM25	1	1	1	43.84792	45.64511	34.57876	1	1	1	1	1	1	1	1	51.20589	1	1	1	62.41742	1
TMEM255B	1	1	1	1	1	1	1	1	1	1	1	1	1	1	1	1	1	57.04323	1	1
TMEM263	1	66.1811	99.09786	1	1	60.10103	1	114.8467	1	40.33025	1	43.88084	41.04462	1	1	80.13441	1	49.88878	1	1
TMEM38B	1	1	1	1	1	1	1	1	1	48.55179	1	29.34091	1	1	1	1	1	1	1	1
TMEM39B	1	1	1	1	1	1	1	1	1	1	1	31.82754	1	76.05332	1	1	1	1	1	1
TMEM43	1	1	81.58393	57.19925	53.04234	40.00901	1	68.9471	1	31.74033	1	28.5705	42.69421	1	1	1	91.71292	55.34133	1	103.6483
TMEM44	1	1	1	1	1	1	1	1	1	1	1	1	1	1	45.99212	1	1	53.61393	1	1
TMEM47	1	36.01913	1	1	1	191.9719	1	135.5099	1	50.69984	1	82.03093	235.8376	1	1	89.76455	1	1	1	1
TMEM50B	1	55.03784	112.8323	1	1	67.9867	1	69.0392	1	26.14525	1	1	57.47254	1	1	1	1	79.53008	1	1
TMEM52B	94.17739	1	1	1	1	1	1	1	1	1	1	1	1	1	1	1	1	1	1	1
TMEM60	1	1	1	1	1	36.97798	1	1	1	1	1	41.07482	1	1	1	1	1	1	1	70.02264
TMEM65	1	57.7297	1	1	1	79.73963	1	1	1	39.22285	1	52.36571	75.78047	1	1	1	1	39.66054	1	1
TMEM67	1	38.64222	1	1	1	30.34427	1	1	1	1	1	34.66561	111.9756	1	1	1	1	1	1	70.02264
TMEM68	1	42.85464	70.1374	45.99207	42.53146	33.97587	1	1	1	55.195	1	43.16153	37.60141	1	54.42862	1	1	1	1	1
TMEM74	1	1	1	1	1	1	1	1	1	71.97739	1	1	1	1	1	1	1	1	1	1
TMEM74B	1	1	1	1	1	1	1	92.41222	1	1	1	1	1	1	1	1	1	1	1	1
TMEM88	1	1	1	1	1	1	1	40.9356	1	1	1	1	1	1	1	1	1	72.84309	1	1
TMEM8A	1	1	1	1	1	1	1	26.03189	1	1	1	1	1	1	1	1	1	45.1651	1	1
TMEM97	1	90.18278	120.8667	59.79679	46.46025	74.17287	1	1	1	151.3317	1	95.89856	43.26194	1	1	67.06629	1	1	1	1
TMIGD3	377.8227	1	1	1	1	1	1	1	1	1	1	1	1	1	1	1	1	1	1	1
TMOD1	1	1	201.069	54.43453	121.2237	1	189.6283	1	1	1	1	1	1	1	271.7321	1	224.7205	1	115.6501	310.7162
TMOD3	67.72057	1	1	1	67.14264	54.22651	1	222.353	1	1	84.88149	33.72909	41.08672	86.51198	78.17734	1	1	206.7726	113.6392	1
TMPO.AS1	1	1	1	1	1	1	1	1	1	1	1	33.96695	1	1	1	1	1	1	1	1
TMSB15B.1	1	1	1	1	48.94288	1	1	1	1	35.84456	1	23.48403	1	1	1	1	1	1	54.19353	1
TMTC2	1	1	1	65.19463	1	64.58648	1	1	1	84.10599	1	57.01632	68.52048	1	1	1	1	1	1	1
TMTC4	1	55.02349	44.73658	1	1	77.38577	1	1	1	85.28046	246.8998	76.54577	38.12953	1	47.80014	75.90005	53.73692	35.63595	1	1
TMUB2	1	1	1	1	45.68756	32.95241	1	1	1	1	1	44.25776	25.43496	1	1	1	1	22.02719	109.9864	1
TNC	1	203.7251	1	1	1	617.2315	1	1	1	40.11288	1	118.5926	73.50821	1	1	208.5216	1	1	1	1
TNF	452.1828	1	1	1	1	1	1	1	1	1	1	1	1	1	1	1	1	1	1	1
TNFAIP3	325.8111	1	1	1	1	1	1	297.9938	1	1	1	1	1	1	1	1	1	23.63161	1	1
TNFAIP8L1	1	1	1	1	1	1	1	88.58801	1	1	1	1	1	1	1	1	1	97.48311	1	1
TNFAIP8L3	70.96136	1	1	1	1	1	1	1	1	1	1	1	1	1	1	1	1	1	1	1
TNFRSF10D	1	1	1	1	1	1	1	67.34978	1	1	1	1	1	1	1	1	1	76.51633	1	1
TNFRSF12A	1	60.56426	1	1	1	1	1	20.77055	1	1	1	1	173.8389	1	1	1	1	21.49781	1	1
TNFRSF14	45.40546	1	1	1	1	1	1	1	1	1	1	1	1	1	1	1	1	38.90318	1	1
TNFRSF19	1	1	1	1	1	43.65709	1	1	1	1	1	1	44.74998	1	1	1	1	19.51515	1	1
TNFRSF1A	56.08217	1	1	1	1	37.74752	1	122.9364	1	1	1	1	24.02993	1	1	1	1	86.76904	1	1
TNFRSF1B	67.66807	1	1	1	1	1	1	28.72439	1	1	1	1	1	1	1	1	1	56.63498	1	1
TNFRSF21	1	1	75.01304	1	1	1	1	161.9716	1	1	1	1	1	1	1	1	1	1	1	1
TNFSF10	1	1	1	1	1	1	1	1	1	1	1	1	1	1	1	1	1	213.0891	1	1
TNFSF12	63.85396	1	1	1	1	26.48159	1	58.56098	1	1	1	1	1	1	1	1	1	38.39477	1	1
TNFSF9	1	1	1	1	1	1	1	85.43235	1	1	1	1	1	1	1	1	1	1	1	1
TNIK	1	57.8346	71.38814	129.8344	169.5104	1	176.0215	1	288.0052	41.54232	1	39.26573	27.69631	76.64563	135.1403	1	169.5523	1	193.8629	208.0679
TNIP1	1	41.84554	1	1	1	26.32387	1	45.80052	1	1	1	23.84332	1	1	1	1	1	140.5905	1	1
TNK2	1	80.3062	183.0607	1	1	1	1	1	1	1	1	1	1	1	1	1	1	1	1	1
TNKS1BP1	1	1	1	1	1	23.06875	1	147.1521	1	1	1	1	1	1	1	1	1	1	1	1
TNR	1	1	452.8521	1	1	1	1	1	111.0471	1	1	1	1	1	1	1	1	1	1	70.02264
TNS1	1	1	1	1	1	1	1	200.1828	1	1	1	1	1	1	1	1	1	69.63579	1	1
TNS2	1	1	55.10537	1	1	1	1	188.0676	1	1	67.02265	1	1	1	1	1	1	67.00849	1	70.02264
TNS3	39.56893	1	107.1821	1	1	1	1	322.3745	1	1	1	1	1	1	1	1	1	1	1	1
TOE1	1	1	1	1	1	1	1	1	1	30.63616	1	23.67156	1	1	1	1	1	1	1	1
TOGARAM1	1	46.19092	65.06112	64.47535	62.98915	34.74188	1	26.56582	132.7503	48.73455	114.348	49.92267	71.19249	83.07223	1	1	228.0977	1	1	103.6483
TOLLIP	1	33.89418	53.63772	1	1	33.32802	1	33.39884	1	1	1	27.8466	23.38773	1	49.88775	1	1	33.69874	1	1
TOM1L2	1	1	1	1	1	50.60202	1	1	1	1	1	1	39.56458	1	1	1	1	40.21554	1	1
TOMM40	53.59477	62.36258	84.40563	1	56.35098	54.17221	1	71.21578	1	84.53535	1	68.45383	59.81543	79.25115	63.44467	54.23586	1	45.57506	1	172.671
TOP2A	1	60.80106	1	1	1	52.3961	1	1	1	150.7621	1	1853.355	226.2508	1	1	196.05	1	1	1	1
TOP3A	1	1	1	1	1	1	1	1	1	46.43446	1	37.51328	1	1	1	1	39.66949	1	1	1
TOR2A	1	1	1	1	1	1	1	1	1	1	1	1	1	1	1	1	1	1	65.4844	1
TOR3A	80.80962	1	34.79345	1	1	1	1	1	1	1	1	30.06486	30.52865	1	1	1	1	50.09202	1	1
TOX	1	1	1	1	1	52.54423	1	1	1	48.86591	1	32.94894	1	68.28574	1	1	1	1	1	1
TOX2	1	1	1	1	1	1	1	1	1	1	1	1	1	70.47426	1	1	1	1	1	1
TOX3	1	142.2882	112.2514	55.82829	80.71821	132.4327	1	1	1	160.8814	206.1389	128.0901	118.2347	140.484	118.5521	91.60929	72.6678	1	92.33066	70.02264
TP53	1	58.87673	1	1	1	28.71675	1	34.02347	1	63.02958	1	36.37504	42.62829	1	1	80.67013	1	34.49775	1	1
TP53BP2	1	77.59216	57.07853	81.81717	71.41868	96.92878	1	75.5102	1	76.3264	62.40081	88.86899	70.73102	52.68793	43.90959	45.1537	1	31.87725	1	87.58009
TP53I11	1	1	1	1	43.96351	1	1	1	513.401	1	1	1	1	1	78.78825	1	34.22783	32.92427	70.15397	1
TP53INP1	1	1	1	50.67478	1	27.98018	1	43.06189	1	45.43945	1	23.33906	23.19054	1	1	1	1	1	1	1
TPD52L2	79.85185	38.81334	1	1	1	36.48372	1	1	1	32.31294	1	37.70072	28.18785	1	1	82.72381	1	28.64105	1	103.6483
TPM2	1	116.8584	104.429	1	1	155.301	1	418.8829	1	92.998	1	125.4061	131.2926	1	1	260.6185	1	1	1	1
TPP1	67.94204	1	1	1	1	41.58769	1	1	1	1	1	1	1	1	1	1	1	45.65813	1	1
TPP2	1	52.47478	76.73646	1	42.2666	36.88937	1	44.72291	1	63.34016	1	42.04752	37.71841	1	1	1	115.6844	29.24087	1	1
TPPP3	1	1	1	1	1	1	1	1	1	1	1	1	126.6795	1	1	1	1	1	1	1
TPST2	49.04923	1	1	1	1	1	1	1	1	1	1	1	1	1	1	1	1	33.74669	1	1
TPX2	1	1	1	1	1	1	1	1	1	40.23378	1	580.8282	69.02598	1	1	73.15312	1	1	1	103.6483
TRABD	1	1	1	1	1	1	1	30.86731	1	32.36863	1	32.52586	1	1	1	1	1	1	1	103.6483
TRAF3	1	1	41.15178	1	1	1	1	1	1	1	1	1	1	1	1	1	1	1	1	1
TRAF3IP1	1	1	1	1	1	24.87907	1	1	1	29.65672	1	42.38728	57.29591	1	1	1	155.8	1	1	1
TRAF3IP2	1	74.18615	44.50157	1	1	1	1	1	1	1	1	1	30.24081	1	1	1	1	16.14649	1	1
TRAF3IP2.A	1	74.28456	55.95271	1	1	38.35595	1	1	1	46.58487	60.48372	42.29241	41.17746	71.12883	1	1	1	21.05207	1	1
TRAFD1	1	1	40.77895	1	1	1	1	1	1	29.26376	1	1	1	1	1	1	1	13.7688	1	1
TRAIP	1	1	1	1	1	1	1	1	1	1	1	27.99928	1	1	1	1	1	1	1	1
TRAK1	1	1	56.9311	77.24368	108.0654	29.80448	1	68.79036	1	34.91836	1	29.4556	24.03746	69.90226	109.5563	1	1	17.51718	145.2203	116.5001
TRAK2	1	1	1	1	1	1	1	63.68581	1	1	1	1	1	1	1	1	1	1	1	1
TRAP1	1	66.25154	107.1897	52.11824	53.93083	43.65894	1	1	1	64.54857	1	37.96119	47.39597	1	77.24348	38.4974	1	49.37791	1	87.58009
TRAPPC1	136.0001	52.65918	71.2363	59.49295	87.87614	119.5653	144.6566	82.75239	1	42.55181	1	60.40102	58.41832	70.00551	73.67517	149.9474	1	61.01848	1	1
TRAPPC10	1	1	1	1	1	1	1	39.78587	1	1	1	29.02872	1	1	53.73909	1	1	1	1	1
TRAPPC11	1	47.05938	31.41028	1	1	31.66529	1	1	1	29.80722	1	33.80601	30.67238	1	71.88234	1	1	25.31867	1	1
TRAPPC2	1	1	1	50.341	52.23491	28.06351	1	1	68.86276	1	1	28.50972	24.98947	1	49.26395	1	1	1	98.77782	172.671
TRAPPC6A	64.81885	1	29.78282	1	1	52.05481	1	1	1	1	1	1	34.97713	1	1	83.62067	1	58.48136	1	1
TRAPPC8	1	1	1	1	1	1	1	1	1	30.08419	1	20.36997	1	1	1	1	1	27.39874	1	1
TRAPPC9	1	1	1	1	1	1	1	1	1	27.5434	1	1	1	1	1	1	1	1	1	1
TREM2	1130.903	1	1	1	1	1	1	1	1	1	1	1	1	1	1	1	1	1	1	1
TRIAP1	1	59.32593	51.75289	1	45.54251	37.42161	1	32.54853	1	40.1913	61.25187	45.183	34.3188	92.66385	1	45.90505	1	123.935	1	206.2967
TRIB1	86.31588	1	1	1	1	36.89725	1	74.49199	1	1	1	1	29.06468	1	1	1	1	43.07289	1	1
TRIB2	1	288.6549	330.952	56.19674	61.1697	25.28607	1	131.546	47.70116	1	1	31.81609	1	1	102.3625	1	74.01583	44.50284	103.9364	139.0453
TRIL	1	48.25251	1	1	1	1	1	1	1	64.59832	1	1	1	1	1	1	1	1	1	1
TRIM16	1	1	1	1	1	1	1	1	1	1	1	1	1	1	1	1	1	49.91869	1	1
TRIM27	1	50.50367	81.47546	50.24624	50.13815	27.36183	1	25.51107	1	66.12043	1	44.27803	32.08832	1	1	77.74045	1	22.49739	108.7759	1
TRIM36	1	1	1	62.85337	76.92197	31.5505	1	1	85.63246	52.64457	125.393	48.7698	42.49017	111.2624	96.02226	1	133.7349	1	72.84332	70.02264
TRIM46	1	1	1	1	1	1	1	1	1	1	1	1	1	1	1	1	1	1	54.98083	1

TRIM47	1	58.17684	1	1	1	40.19818	1	1	1	1	1	1	1	1	1	1	1	1	1	1
TRIM52	1	41.37889	60.20929	46.18443	48.00021	1	1	38.5589	1	1	75.40591	39.90916	30.97139	75.78669	1	1	1	22.09482	1	70.02264
TRIM59	1	1	1	1	1	1	1	1	1	1	1	76.71238	1	1	1	1	1	1	1	1
TRIM9	1	225.5032	105.3034	152.7895	92.68037	401.4092	1	1	1	154.089	1	127.0031	226.4706	1	1	234.6768	1	1	1	1
TRIP13	1	1	1	1	1	1	1	1	1	1	1	45.34136	1	1	1	1	1	1	1	1
TRIP6	1	49.80517	1	1	1	63.14141	1	47.06327	1	1	1	47.93804	76.29729	1	1	1	1	26.09921	1	1
TRMT10B	1	1	1	1	1	24.57742	1	1	1	31.60899	1	1	1	1	1	1	119.1209	1	1	1
TRMT13	1	51.75916	1	57.50257	56.93278	39.82663	1	1	1	53.74169	70.06349	32.81733	42.86762	65.02275	51.29852	1	142.5611	1	1	70.02264
TRMT5	1	43.40521	46.46359	1	1	28.16687	1	1	1	1	1	1	1	1	1	1	1	1	1	1
TRMT6	1	1	1	1	1	1	1	1	1	35.70519	1	1	1	1	1	1	1	1	1	1
TRMT61B	1	30.99602	113.2179	43.63834	42.72069	33.99729	1	1	1	49.84635	1	35.27126	46.07927	64.27183	1	38.32214	1	1	1	70.02264
TRMT9B	1	1	1	82.11013	104.8437	1	1	1	352.9074	84.50106	1	1	1	1	117.4683	1	187.3001	1	1	103.6483
TRMU	1	42.79376	1	1	1	1	1	1	1	36.50231	1	51.64579	1	1	1	1	1	1	1	1
TRNT1	1	38.74767	1	1	1	27.88541	1	1	1	32.22359	1	30.68097	28.82965	1	47.94097	1	1	26.10282	1	70.02264
TROAP	1	1	1	1	1	1	1	1	1	1	1	86.88432	1	1	1	1	1	1	1	1
TRPC6	1	1	1	1	1	1	1	84.3971	1	1	1	1	1	1	1	1	1	1	1	1
TRPM3	1	1	1	1	1	1	1	1	485.888	1	1	1	1	1	1	1	1	1	1	70.02264
TRPM7	1	30.90042	26.66367	40.96062	47.52967	26.36559	1	26.84551	1	1	1	29.98359	33.15552	1	1	1	1	34.39757	1	1
TRPS1	1	75.57181	81.70683	1	1	116.1529	1	52.03529	1	57.66289	1	48.19588	88.32356	1	1	106.8711	1	33.25168	1	1
TRPT1	1	39.69139	1	1	1	32.64783	1	1	1	37.08379	1	24.90961	24.73135	1	1	1	1	1	1	1
TRUB2	1	34.85984	32.93845	1	1	40.12963	1	1	1	37.12659	1	26.26796	29.09076	1	1	78.30573	1	11.84802	1	1
TSC1	1	32.44666	72.20593	66.99343	61.38584	41.721	1	1	1	36.65733	1	33.8456	28.25026	73.24433	1	1	1	13.96008	63.07119	241.6936
TSC22D3	134.2251	1	1	1	1	1	1	193.798	1	1	71.84591	1	1	59.86177	1	1	1	379.2571	1	1
TSC22D4	60.28732	136.4225	259.1386	1	1	94.35113	1	41.65583	1	1	1	38.853	96.62275	1	1	62.42419	1	1	1	1
TSFM	1	33.83619	41.20265	1	40.15547	42.94002	1	1	1	36.65875	1	41.8425	30.99491	1	78.82074	45.05708	1	30.74618	1	1
TSHZ1	1	57.38909	54.08728	1	1	27.73781	1	32.46053	1	1	84.3761	32.83706	1	1	1	1	1	13.98894	1	1
TSHZ3	1	1	1	1	1	1	1	21.655	86.35605	1	1	1	1	1	1	1	1	1	1	1
TSIX	1	51.3409	54.53266	95.28074	103.3225	34.42209	1	31.83991	379.9439	57.60743	181.2733	31.08104	22.47962	141.4966	97.04004	217.8603	623.4171	22.13085	106.8427	174.1602
TSPAN12	1	48.0031	1	1	1	1	1	69.92319	1	1	1	1	93.54128	1	1	1	1	35.73417	1	1
TSPAN13	1	1	93.48133	135.7	204.9162	1	210.0687	1	242.5897	1	202.8454	1	1	149.9582	295.5898	1	238.0687	89.42177	154.3941	344.3419
TSPAN15	1	1	38.24555	1	1	1	1	118.3274	1	1	1	1	1	1	1	1	1	78.18893	1	1
TSPAN18	1	1	1	1	1	83.40825	1	1	1	30.1152	1	1	1	1	1	1	1	57.36488	1	116.5001
TSPAN2	1	1	1	1	47.8168	1	1	1	140.3064	1	1	1	34.11556	1	40.54353	1	1	1	1	1
TSPAN4	1	1	1	1	1	1	1	87.0414	1	1	1	1	1	1	1	1	1	172.8125	1	70.02264
TSPYL2	1	1	52.82994	1	53.41249	34.33321	1	130.7541	1	1	1	1	32.95423	1	1	1	1	90.183	1	1
TSPYL5	1	1	1	1	38.92543	28.67919	1	1	1	29.46656	1	30.54445	1	1	1	1	106.7086	25.0295	1	70.02264
TSR2	1	41.18951	44.95535	1	1	40.14261	1	23.86056	1	45.89222	1	43.03303	34.19867	1	1	1	1	20.76104	125.3833	1
TST	1	1	1	1	1	37.48221	1	1	1	1	1	1	27.72304	1	1	1	1	1	1	1
TSTA3	1	1	33.41461	1	1	1	1	1	1	33.70968	1	30.71813	31.18487	1	1	1	101.7934	49.53738	1	1
TSTD1	1	36.86745	1	48.43778	63.50105	66.88385	1	1	116.1172	1	1	47.29791	97.02856	1	39.75054	44.88289	109.2453	1	1	275.3193
TSTD2	1	1	1	51.14697	51.34332	25.27826	1	1	1	48.70257	1	30.67538	29.10928	1	1	1	107.7816	1	1	1
TTBK2	1	38.51196	48.42681	47.78043	49.51643	39.12652	1	1	1	30.96366	1	37.93677	32.54947	64.83249	1	1	1	1	170.7985	1
TTC14	1	83.26884	91.11843	109.3032	88.57706	77.71332	1	55.81297	110.7913	104.958	108.8417	72.96838	70.1454	90.6711	113.8947	139.3656	344.0939	39.86451	175.5909	308.945
TTC21B	1	1	38.59404	1	1	1	1	30.68366	1	44.54033	1	31.76394	29.05197	72.85363	1	1	1	16.62888	1	70.02264
TTC25	1	1	1	1	1	1	1	1	1	1	1	1	42.91809	1	1	1	1	1	1	1
TTC28	1	65.71444	1	280.1299	398.2809	43.1139	423.7748	60.59239	1	68.63464	138.4053	75.2162	48.85027	114.9661	351.4531	70.94724	227.9441	125.4087	375.4401	172.671
TTC32	1	1	1	1	1	1	1	1	1	43.21133	1	44.20824	28.11034	1	1	1	1	1	1	1
TTC39C	1	1	42.23438	1	1	1	1	1	1	1	1	1	1	1	1	1	1	1	1	1
TTC5	1	31.63513	1	1	36.97555	25.78275	1	1	31.0623	35.12491	1	1	46.09626	1	1	1	1	1	1	1
TTC8	1	1	1	1	1	29.73171	1	1	1	1	1	1	32.9787	1	1	1	1	1	1	1
TTC9	1	1	1	1	1	50.50883	1	1	77.31345	1	1	33.30443	1	1	1	1	109.4861	1	1	103.6483
TTC9B	1	1	191.4744	110.6995	116.7353	1	200.0643	1	790.1753	1	117.0045	1	1	90.93494	203.8171	1	184.6075	1	160.7771	190.2284
TTC9C	1	1	49.36956	1	1	26.11249	1	1	1	1	1	31.88412	1	1	1	1	1	1	1	1
TTF2	1	1	1	1	1	1	1	1	1	42.41547	1	50.06183	1	1	1	1	1	1	1	1
TTI1	1	1	1	1	1	1	1	1	1	1	1	31.07711	1	1	1	1	1	1	1	1
TTK	1	1	1	1	1	1	1	1	1	1	1	151.2307	1	1	1	1	1	1	1	1
TTLL4	1	1	1	1	1	35.11392	1	1	1	1	1	1	23.11453	1	1	1	1	1	1	1
TTLL5	1	1	1	1	51.10017	27.96615	1	1	1	31.2158	1	43.23183	53.73697	1	1	1	1	26.22962	1	1
TTLL7	1	80.83091	77.48124	213.7689	226.1166	76.28523	176.4854	1	382.1549	98.57232	198.2422	156.8787	79.80519	213.4402	219.0997	166.6291	297.2118	1	199.3612	517.7842
TTN	1	1	1	1	1	1	1	1	1	1	1	1	1	1	1	1	1	123.4646	1	1
TTYH1	1	421.9495	361.4875	1	1	1176.359	1	38.40514	168.6656	44.41302	1	195.0213	526.7664	1	48.22639	663.3192	1	1	1	1
TTYH2	1	1	1	106.791	1	1	1	84.02242	1	41.64352	1	1	1	1	1	1	1	108.6258	1	1
TUB	1	1	57.54383	1	1	1	1	1	1	1	1	1	1	1	1	1	1	1	1	1
TUBA1C	122.8312	1	1	57.23214	81.33061	24.74709	1	60.15233	1	1	1	203.5187	43.89937	1	54.27449	1	1	45.54698	67.16846	87.58009
TUBB6	1	1	1	1	1	37.48682	1	89.21232	1	1	1	125.1386	1	1	1	1	1	63.71975	1	1
TUBG1	1	1	1	1	1	1	1	1	1	66.55024	1	75.44715	26.40895	1	1	1	1	14.19298	1	103.6483
TUBGCP3	1	1	1	1	1	1	1	1	1	36.46401	1	52.09385	1	1	1	1	1	99.79266	1	70.02264
TULP3	1	58.45173	1	1	42.64465	81.3299	1	49.28973	1	68.99717	55.88954	95.24445	49.51752	1	55.23534	50.14111	1	34.18988	1	1
TUSC2	1	31.23163	40.77068	1	44.45191	43.78873	1	1	1	36.7617	1	31.31825	37.374	61.341	1	1	1	23.54383	1	1
TWF1	1	63.18595	66.92051	1	42.4454	93.20183	1	72.35237	1	65.39792	1	54.21878	61.6693	1	1	54.05956	1	28.45354	104.2434	1
TWF2	103.6064	1	1	1	1	26.59229	1	49.01611	67.66979	1	1	1	1	1	1	1	1	45.37237	72.30942	103.6483
TWIST1	1	1	1	1	1	1	1	200.125	1	1	1	1	1	1	1	1	1	1	1	1
TXNDC16	1	1	1	51.18935	55.37682	1	1	1	94.82552	29.20443	1	28.34076	1	1	1	1	107.0625	42.77168	1	87.58009
TYMP	93.72853	1	1	1	1	1	1	1	1	1	1	1	1	1	1	1	1	1	1	1
TYMS	1	87.58418	147.9208	1	1	1	1	1	1	325.7122	1	473.0236	114.1292	1	1	144.7063	1	1	1	1
TYRO3	1	55.59082	1	1	1	1	1	1	1	1	1	28.20294	30.1969	1	1	1	1	1	1	1
TYROBP	1493.203	1	1	1	1	1	1	1	1	1	1	1	1	1	1	1	1	1	1	1
TYW1	1	1	1	1	1	1	1	1	1	1	1	1	1	1	1	1	1	1	68.32347	1
UACA	1	64.90404	1	1	1	1	1	873.3499	1	1	1	34.46386	1	1	63.72089	1	118.3565	220.4951	1	1
UBA5	1	60.72793	49.93232	47.43102	42.13023	54.62767	1	46.94237	1	57.02151	1	43.24964	48.94599	56.72723	75.62557	46.88721	1	24.46373	70.93416	116.5001
UBAP1	1	27.55525	44.7329	1	1	37.81627	1	1	1	1	1	25.1191	31.90811	1	1	35.12267	1	21.19461	1	1
UBAP2L	1	52.34507	77.41842	55.05191	50.93741	46.05255	1	44.70586	79.32646	54.56229	1	41.52405	56.09495	1	51.83704	66.71493	1	107.7005	47.65373	87.58009
UBE2C	1	1	1	1	1	1	1	1	1	1	1	744.6627	1	1	1	1	1	1	1	1
UBE2E2	54.85255	1	78.95852	1	1	1	1	36.22662	1	1	1	1	29.61188	1	1	1	1	1	1	1
UBE2F	84.33944	1	1	1	1	30.67506	1	1	1	41.27437	1	26.51939	28.7724	1	1	1	1	29.91084	1	1
UBE2O	1	37.91764	37.64578	62.16683	66.96613	28.15702	1	1	58.51568	43.06665	1	38.88648	34.98022	1	1	1	1	26.77903	1	1
UBE2QL1	1	1	1	56.78673	55.39111	1	1	1	249.5616	122.4716	74.72583	32.0799	1	89.78523	136.0506	1	89.92952	1	1	1
UBE2T	1	1	1	1	1	1	1	1	1	130.4899	1	325.4672	44.49948	1	61.95786	54.50852	1	1	1	1
UBE3C	1	1	1	1	40.12179	34.25736	1	39.69022	1	43.18008	69.93747	40.8467	35.20132	1	1	1	1	65.28608	1	1
UBE4B	1	1	29.36518	49.60947	39.77411	33.68453	1	1	1	37.25632	1	37.31333	1	1	1	1	1	17.47804	1	1
UBIAD1	1	1	43.35552	1	1	1	1	1	1	1	1	20.8111	1	1	1	1	1	1	1	1
UBL4A	1	1	1	1	1	23.95766	1	1	1	32.85254	1	33.48298	31.45506	1	1	41.98934	1	1	1	1
UBL7	1	43.98843	1	1	1	30.61423	1	1	1	60.65368	1	45.7756	21.92341	1	1	1	1	30.74389	1	1
UBL7.AS1	1	1	1	1	1	1	1	1	1	1	1	40.00475	1	1	1	1	1	1	1	1
UBQLN4	1	1	1	1	1	1	1	1	1	34.47041	1	22.4507	1	1	1	1	1	1	1	1
UBR1	1	47.57052	32.32587	51.47222	1	39.00012	1	68.788	1	42.97706	73.15743	44.3882	39.26711	1	1	1	140.5658	1	1	1
UBR2	1	47.59639	64.40178	45.94472	38.93501	31.76144	1	32.46781	1	39.95626	1	26.99048	29.27368	64.67185	1	1	123.7602	19.18889	1	1
UBR7	1	1	1	1	1	1	1	1	1	40.52422	1	43.20047	1	1	1	1	1	1	1	1
UBXN11	1	1	1	1	1	1	1	1	1	1	1	1	55.34893	1	1	1	1	1	1	1
UCHL1	113.7884	119.3289	271.9818	368.1702	653.2982	465.2458	923.2379	26.19693	1015.013	151.528	742.3314	186.7755	243.8999	499.8837	1111.639	480.3077	1399.134	27.46	448.4563	1820.358
UCHL5	1	1	1	1	46.73115	26.85466	1	1	96.78775	41.66987	1	37.75286	37.05988	1	1	1	1	44.63006	1	1

UCK2	1	1	1	1	1	1	1	1	1	48.71344	1	40.50475	1	1	1	1	1	31.61719	1	87.58009
UCP2	341.9048	1	1	1	1	32.03625	1	1	1	62.3193	1	65.68146	34.70027	1	1	1	1	1	1	1
UGDH	1	39.98116	178.1876	1	1	51.9189	1	52.56408	1	35.55999	1	40.05707	39.24871	1	1	54.34748	1	24.84613	1	1
UGGT1	1	1	1	1	1	1	1	1	1	42.7804	1	33.03572	26.97473	1	1	1	1	25.43892	1	1
UGGT2	1	1	63.73477	1	1	27.41646	1	33.436	1	43.024	1	40.2633	32.4502	1	1	80.55019	1	1	1	1
UGT8	1	1	290.1569	1	1	1	1	1	1	1	1	1	1	1	1	1	1	1	1	1
UHRF1	1	72.64033	1	1	1	1	1	1	1	168.223	1	91.18535	43.14745	1	1	69.22365	1	1	1	1
ULK1	1	1	1	58.3634	49.35105	1	1	1	88.49953	47.46402	1	29.07132	1	1	67.8517	1	1	1	1	1
ULK3	1	1	1	1	1	1	1	1	198.8077	29.48984	1	1	1	1	1	1	1	1	1	70.02264
UMPS	1	38.47092	46.00535	1	1	39.45655	1	1	1	68.28977	1	47.33629	40.53056	1	1	1	1	1	1	1
UNC119	1	1	1	1	1	36.40811	1	1	1	1	1	1	25.2886	1	49.94501	1	1	1	1	87.58009
UNC119B	1	1	1	1	1	1	1	1	1	1	1	1	33.46194	1	1	1	1	1	1	1
UNC13A	1	1	1	1	1	1	1	1	85.02908	1	1	1	1	1	1	1	42.47042	1	1	1
UNC45A	1	1	1	1	42.86255	30.39702	1	25.72284	1	1	1	1	25.77459	1	1	1	1	39.70127	1	70.02264
UNC5B	1	1	1	1	1	1	1	263.492	1	1	1	1	1	1	1	1	1	263.4726	1	1
UNC5D	1	1	1	296.3971	244.9112	28.49499	144.4316	1	1	99.52461	1	51.72496	1	95.45289	108.2895	1	301.3334	1	292.5297	1
UNC79	1	1	1	1	1	1	1	1	1	1	1	1	1	1	1	1	67.75551	1	1	70.02264
UNC80	1	1	62.3816	1	1	1	1	1	1	1	1	1	1	1	1	1	1	1	1	1
UNC93B1	75.84545	1	1	1	1	1	1	1	1	1	1	1	1	1	1	1	1	1	1	1
UNG	1	41.79665	1	57.22663	56.81958	52.42103	1	1	1	147.2861	1	50.78417	41.44988	1	62.62777	82.86993	1	38.76672	1	1
UNK	1	33.90647	35.34278	1	42.51981	28.6631	1	1	1	48.73304	1	44.71934	38.77364	70.68404	1	1	1	21.99539	1	103.6483
UQCC3	1	57.01245	71.39092	1	1	47.64455	1	47.00211	1	37.04887	1	48.52918	37.4598	1	1	69.92218	1	38.71125	1	1
USF2	147.1366	70.2043	95.5435	62.21175	71.28163	79.30985	1	39.56703	79.20267	67.50302	71.91776	73.85802	64.38851	81.7343	103.4662	90.09973	1	43.51881	50.3278	241.6936
USF3	1	1	1	52.48586	45.90281	27.54875	1	42.79458	1	31.30521	1	28.5594	28.5401	1	1	1	1	41.71192	94.15741	1
USHBP1	1	1	1	1	1	1	1	1	1	1	1	1	1	1	1	1	1	116.0738	1	1
USP12	1	30.91987	1	1	1	1	1	30.87707	1	1	1	25.92006	25.46713	1	1	1	144.8607	48.40937	1	1
USP13	1	1	1	1	1	1	1	22.97398	1	60.9482	1	63.06172	37.82751	1	1	1	1	1	1	103.6483
USP24	1	39.98371	117.4654	1	1	49.86159	1	1	1	1	1	27.91123	27.72362	1	1	1	1	1	1	139.0453
USP31	1	1	1	45.48453	70.92546	1	1	1	1	1	1	30.75892	33.65935	1	52.64467	1	1	49.70047	1	1
USP37	1	1	1	1	1	1	1	23.52873	1	44.29438	1	37.93299	26.13866	1	1	1	1	15.34988	1	1
USP39	1	1	27.68728	1	1	25.61888	1	28.88395	1	51.30047	1	44.88993	34.43955	1	1	67.3685	1	34.36431	1	1
USP42	1	46.08899	1	64.00295	58.73842	48.74814	1	44.38194	1	52.65248	71.24002	49.54606	65.10551	81.7343	56.50341	1	1	23.5821	1	1
USP49	1	1	37.40255	82.13611	78.90267	1	1	1	1	68.33986	70.1294	46.9403	1	93.97446	47.45957	1	1	1	75.36275	103.6483
USP51	1	1	1	1	1	1	1	1	1	1	1	1	32.61043	1	1	1	1	1	1	1
USP53	1	1	1	1	1	1	1	32.35564	1	1	1	1	1	1	1	1	1	170.5512	1	1
USP54	1	1	1	1	1	1	1	1	1	1	1	1	1	1	1	1	1	48.03868	1	1
USP6NL	1	1	1	1	1	1	1	46.09695	132.4679	34.34876	1	35.52829	28.69175	1	1	1	1	40.37004	1	1
USPL1	1	34.43865	1	1	50.64457	40.30005	1	1	1	35.76702	1	36.71595	26.88339	52.60634	1	1	1	43.4333	1	1
UTP14A	1	1	1	1	1	1	1	1	1	29.64455	1	1	22.91646	1	1	1	1	1	1	1
UTP14C	1	34.72594	1	1	1	32.54424	1	46.17193	1	43.017	1	37.81796	28.07789	1	1	60.77593	1	1	1	1
UTP20	1	1	33.88573	1	1	1	1	1	1	44.05646	1	1	25.83289	1	1	1	1	23.42899	1	1
UTP23	1	39.79931	53.04506	45.70923	40.79509	36.5739	1	30.71074	1	59.64562	70.76936	41.14708	39.89762	56.75421	1	1	1	1	1	70.02264
UTP3	1	47.24564	41.46412	39.963	50.21576	42.19561	1	39.88684	1	44.87792	1	36.22384	49.52413	1	1	1	1	114.8904	97.52856	1
UTRN	1	44.22785	1	1	1	39.47273	1	170.8961	120.935	1	1	42.73426	38.60763	1	1	1	1	327.6234	1	1
UVRAG	1	1	1	47.92494	47.97706	24.66272	1	35.86398	1	31.33122	1	23.61376	1	1	39.60423	1	1	26.56188	1	1
UVSSA	1	1	1	1	1	25.64046	1	28.79449	123.4374	1	63.49883	26.27988	1	1	76.61754	1	87.56392	1	1	103.6483
UXS1	1	1	1	1	1	23.5571	1	39.5476	1	49.06191	1	21.94344	1	1	1	1	1	27.79677	1	1
VAMP5	1	1	1	1	1	1	1	312.2131	1	1	1	1	40.70998	1	1	1	1	292.5776	1	1
VAMP8	403.6622	1	1	1	1	1	1	90.70661	1	1	1	1	1	1	1	1	1	1	1	1
VANGL1	1	1	1	1	1	1	1	1	1	1	1	1	43.40683	1	1	1	1	1	1	1
VANGL2	1	51.41519	41.68876	96.13427	85.60355	30.69635	1	1	1	64.31233	1	51.88187	58.39076	66.10051	62.83585	1	111.6084	57.375	1	149.721
VARS	1	39.18027	48.78626	1	44.72852	1	1	1	1	36.65736	1	1	1	1	61.46553	1	1	20.08645	1	70.02264
VASH1	65.3106	33.46048	37.58634	75.06687	63.4321	1	1	1	101.7031	29.04477	128.0938	26.77138	1	64.79423	48.61761	1	1	103.3898	84.89203	1
VASH2	1	1	1	60.94877	82.09824	1	1	26.7798	1	1	98.5412	1	1	92.77006	59.33192	1	151.8274	1	78.28393	139.0453
VASN	1	1	1	1	1	1	1	55.26441	1	1	1	1	51.25795	1	1	1	1	1	1	1
VASP	93.77913	1	1	1	1	27.26044	1	136.0354	1	1	1	1	24.56744	1	1	1	1	50.01869	1	1
VAT1L	1	1	1	1	1	43.96908	1	1	1	1	1	1	1	1	1	1	1	1	1	1
VAV2	1	1	1	1	1	1	1	1	1	41.51522	1	30.07535	1	1	1	1	1	1	1	1
VCAM1	1	1	1	1	1	126.8089	1	1	1	1	1	1	58.8648	1	1	1	1	1	1	1
VCL	1	1	1	1	1	85.79189	1	465.3318	1	1	1	28.05328	116.3989	1	1	94.36091	1	147.2151	1	1
VCPIP1	1	1	1	50.13151	44.79478	40.01316	1	30.85535	1	1	1	43.70544	33.77942	1	1	1	1	31.81293	1	103.6483
VCPKMT	1	1	1	1	1	1	1	1	1	38.39902	1	38.53388	37.99027	58.16149	1	1	51.07769	41.18996	1	1
VEGFA	1	198.4713	197.1596	1	52.85787	172.078	1	107.6388	1	83.85864	1	65.49212	99.96229	1	1	72.39085	1	1	1	1
VEGFC	1	1	1	1	1	1	1	1	1	1	1	1	1	1	1	1	1	87.2771	1	1
VEPH1	1	1	1	1	1	89.33247	1	1	1	1	1	1	1	1	1	1	1	1	1	1
VIM	154.4066	3953.085	129.9956	119.8624	71.57776	8346.146	199.5348	1572.222	132.742	1296.298	108.0712	3709.734	8988.373	80.5395	86.8447	7354.996	241.7648	3766.084	113.8659	1
VLDLR	1	1	1	1	1	1	1	1	82.03967	1	1	1	1	1	73.56404	1	1	1	1	1
VLDLR.AS1	1	1	1	1	1	1	1	1	112.9756	1	1	1	1	1	34.97556	1	117.7096	1	1	103.6483
VOPP1	1	1	1	1	41.94524	55.63018	1	24.83695	296.5633	38.17368	91.28724	51.69056	39.12985	1	51.47817	103.5167	62.7519	62.63123	1	1
VPS16	1	1	62.52796	1	1	31.0039	1	1	1	1	1	1	1	1	1	1	88.9043	1	59.0043	70.02264
VPS26B	1	34.59182	89.3404	48.85918	49.71455	26.46967	1	46.27782	1	30.04563	70.83218	37.60136	28.95409	68.31103	1	1	1	31.60289	84.04633	190.2284
VPS37D	1	1	27.47819	53.25229	52.13946	1	1	1	1	41.37422	1	1	1	1	1	1	1	1	34.18716	1
VPS45	1	64.74258	30.00279	48.02777	48.02088	40.95755	1	1	1	44.72667	1	41.80307	28.69187	1	1	39.96621	1	1	1	1
VPS53	1	1	31.60158	51.76242	59.20622	30.86879	1	1	1	41.90385	1	25.98649	32.38086	57.22985	1	1	1	34.08788	1	1
VPS8	1	1	1	1	1	1	1	1	1	1	1	1	1	1	1	1	1	1	54.94097	1
VRK1	1	33.31667	21.64062	1	1	1	1	1	1	97.24556	1	126.4772	34.65006	1	1	1	1	101.2086	1	103.6483
VRK3	1	46.02068	1	1	1	36.97528	1	1	1	30.7488	1	52.30914	37.38434	1	39.9542	1	1	30.74467	1	103.6483
VSIG4	592.2685	1	1	1	1	1	1	1	1	1	1	1	1	1	1	1	1	1	1	1
VSIR	94.66515	1	1	1	1	1	1	1	1	1	1	1	1	1	1	1	1	68.7704	1	1
VSNL1	1	1	1	1	1	1	1	1	188.8268	1	1	1	1	1	1	1	1	1	1	1
VSTM2A	1	1	1	1	1	1	1	1	1	1	150.3124	1	1	1	1	1	1	1	1	1
VSTM2B	1	1	97.9665	1	1	1	1	1	150.2869	1	1	1	1	1	1	1	1	1	1	1
VSTM4	1	1	1	1	1	1	1	54.5657	1	1	1	1	1	1	1	1	1	1	1	1
VTA1	56.4136	67.04156	35.67718	128.8063	101.5536	67.19665	1	33.73209	142.0102	82.22682	86.4166	96.24167	57.8367	90.08728	103.9453	59.79237	1	54.33051	147.1785	241.6936
VTI1A	1	1	40.17323	1	1	1	1	1	1	1	1	25.78138	1	1	1	1	1	18.04792	1	1
VWA1	1	1	1	1	1	1	1	1	1	1	1	1	1	1	1	1	1	339.8915	1	1
VWF	1	1	1	1	1	1	1	1	1	1	1	1	1	1	1	1	1	362.3314	1	1
WAC.AS1	1	1	1	1	1	35.17342	1	1	59.0091	1	1	22.5161	25.62589	59.10127	1	1	1	1	1	1
WASF3	1	52.95205	1	1	1	61.17128	1	1	1	1	1	1	56.16515	1	1	1	1	1	1	70.02264
WASHC1	1	39.87779	1	1	45.97165	32.81041	1	40.70711	47.14054	45.42063	1	32.84843	33.19046	73.65455	1	1	1	30.79083	43.43407	103.6483
WBP4	1	101.7941	62.24362	72.05307	59.09059	95.7716	1	38.14716	61.81391	91.45968	78.87365	93.24303	93.70866	72.70592	1	81.68688	1	56.66545	57.16254	206.2967
WDHD1	1	1	1	1	1	1	1	1	1	70.5567	1	39.67367	1	1	1	1	1	1	1	1
WDR17	1	1	1	1	1	30.31144	1	1	104.727	26.31045	1	21.30626	37.73236	1	1	1	1	1	1	103.6483
WDR19	1	1	1	1	1	27.05311	1	1	1	1	1	1	53.34129	1	1	1	1	1	1	1
WDR20	1	40.20238	41.66983	65.47296	88.46154	39.79414	1	1	1	46.5347	1	29.50342	24.19974	1	100.0944	1	1	24.54862	88.30805	1
WDR27	1	1	1	1	1	1	1	1	1	1	1	1	1	1	1	1	74.11079	25.10272	1	1
WDR34	1	32.34808	1	1	1	45.85203	1	1	1	80.9131	1	109.9858	72.19825	1	1	79.52515	1	95.51819	1	1
WDR35	1	1	1	1	1	1	1	1	1	1	1	1	44.10551	1	1	1	1	1	1	1
WDR36	1	50.02962	41.72678	53.21268	42.8877	27.88464	1	1	1	52.2112	1	32.82912	35.751	1	1	1	1	32.84285	1	1
WDR41	1	58.06362	73.6541	1	1	107.1917	1	42.36262	1	35.37741	1	59.95205	41.12171	1	1	74.90714	1	30.33188	1	1
WDR45	1	43.81713	1	1	1	34.62412	1	38.2058	1	1	1	22.70669	37.63125	1	1	1	1	22.96769	1	1

WDR47	1	37.22766	1	150.8894	172.3628	77.69917	199.2805	37.12144	178.6046	91.98438	153.0137	73.38268	52.515	117.6524	134.0394	42.21573	127.2926	36.52088	173.2956	1
WDR49	1	1	1	1	1	1	1	1	1	1	1	1	29.70716	1	1	1	1	1	1	1
WDR5	1	1	1	1	1	1	1	1	1	34.58787	1	25.44632	1	1	1	1	1	1	1	1
WDR59	1	42.40694	1	44.49998	40.44378	49.73435	1	1	61.11819	1	1	1	31.49088	1	1	1	1	1	39.65689	1
WDR62	1	1	1	1	1	1	1	1	1	1	1	35.10122	1	1	1	1	1	1	1	1
WDR70	1	41.92988	1	45.06258	42.04235	28.75399	1	1	1	27.3704	1	24.28924	29.54877	1	65.84951	1	1	117.2034	1	1
WDR76	1	1	1	1	1	1	1	1	1	88.4459	1	66.07772	1	1	1	1	1	1	1	1
WDR77	1	1	32.95318	1	1	1	1	1	1	36.79673	1	32.14547	23.02479	1	54.53589	1	1	49.89297	1	1
WDR78	1	1	1	1	1	1	1	1	1	1	1	1	37.22111	1	1	1	1	1	1	1
WDR90	1	1	1	1	1	1	1	1	1	1	1	1	31.85782	1	1	1	1	1	1	1
WDR92	1	1	1	1	1	1	1	1	1	1	1	1	36.55256	1	1	1	1	1	1	1
WDYHV1	1	1	1	1	1	1	1	1	1	43.13666	1	1	29.79145	1	1	1	1	1	1	1
WEE1	1	130.3738	49.14006	49.76118	46.72191	100.6531	1	1	1	87.18119	1	195.0655	157.0485	1	1	166.235	1	1	1	70.02264
WFDC2	1	1	1	1	1	1	1	1	1	1	1	1	1	1	72.831	1	1	1	1	172.671
WHRN	1	1	1	1	1	1	1	1	1	1	1	1	33.48809	1	1	1	1	1	1	1
WIPF3	1	1	1	65.44355	49.89276	60.47958	1	1	1	120.9872	1	89.90689	29.69362	1	95.25027	49.73179	1	1	79.24013	172.671
WIPI1	1	1	1	1	1	1	1	49.97292	1	1	1	1	1	1	1	1	1	72.77146	1	1
WIZ	1	40.39644	71.02891	1	43.93865	33.60794	1	20.90395	1	64.76097	1	55.53716	32.76602	57.37065	1	1	1	44.68733	1	1
WLS	1	1	1	1	1	1	1	155.7903	1	1	123.7813	1	105.9107	110.6783	1	1	1	73.42093	1	1
WNK2	1	1	1	92.11064	96.65721	26.68106	1	1	69.96011	1	1	1	32.95842	1	72.96428	1	112.1909	1	96.71824	70.02264
WNT5A	1	1	1	1	1	1	1	1	1	1	104.3524	1	1	1	1	1	1	1	1	1
WNT7A	1	1	1	1	1	1	1	1	1	1	1	1	1	60.16725	1	1	1	1	1	1
WNT7B	1	1	1	1	1	1	1	1	77.60648	1	1	1	1	1	1	1	1	1	1	1
WRAP73	1	1	1	1	1	1	1	1	1	25.57301	1	1	1	1	1	1	1	1	1	1
WRN	1	1	1	1	1	1	1	1	1	31.25646	1	23.61076	1	67.41072	1	1	1	1	1	1
WSCD1	1	184.2382	148.5467	1	1	92.5896	1	1	1	72.57953	1	52.12147	36.36633	1	1	72.1588	1	1	1	1
WWC1	1	1	1	1	1	48.62897	1	1	1	1	1	1	111.4063	1	48.52684	59.21308	140.4523	1	1	1
WWC2	1	1	1	1	1	28.44687	1	31.24137	1	1	1	1	55.84847	1	1	1	1	43.7563	1	1
WWP1	1	1	1	1	1	1	1	76.13443	1	1	1	1	1	1	1	1	1	32.01058	1	103.6483
WWTR1	1	59.96174	1	1	1	31.53191	1	100.7373	1	1	1	1	132.7031	1	1	1	1	261.7943	1	1
XAF1	1	1	1	1	1	1	1	1	1	1	1	1	1	1	1	1	1	91.22251	1	1
XKR4	1	1	33.62453	1	1	1	1	1	273.5736	1	1	1	1	76.96071	1	1	195.3841	1	1	1
XPNPEP1	1	1	53.141	1	1	1	1	58.18892	1	1	1	1	30.82139	1	1	1	1	33.66463	1	1
XPO5	1	1	49.9064	1	1	1	1	1	1	1	1	1	1	1	1	1	1	1	1	1
XPO6	1	1	41.63269	1	1	1	1	23.73715	1	37.29555	1	22.71964	23.65253	1	44.1588	1	1	18.95233	1	1
XPO7	1	1	1	48.83967	44.92501	24.36215	1	1	1	40.23703	1	38.02789	29.70917	1	45.333	1	1	31.06875	1	1
XRCC1	1	1	1	1	1	1	1	1	1	1	1	29.54771	1	1	1	1	1	1	1	1
XRCC2	1	1	1	1	1	1	1	1	1	84.09401	1	78.08755	1	1	1	40.79769	1	1	1	1
XXYLT1	1	1	1	1	1	1	1	1	1	37.24127	1	25.5292	25.33733	1	57.24152	1	1	14.35024	1	1
XYLT1	1	44.94842	100.6826	1	1	1	1	1	1	30.68337	1	31.14269	1	1	1	1	1	1	1	1
YAE1D1	1	35.80275	43.80127	1	44.05095	33.70781	1	1	1	44.64798	1	33.60097	35.34592	53.02304	1	1	1	1	1	1
YAP1	1	62.40504	1	1	1	81.1687	1	72.68917	1	30.4933	1	40.04503	67.68557	1	1	82.15834	1	20.65906	1	1
YARS	1	38.86219	63.93798	1	1	38.07053	1	45.20597	1	31.16798	1	28.71492	43.08833	1	1	53.47663	1	19.91446	1	103.6483
YARS2	1	37.04022	1	1	44.53109	24.53341	1	1	1	36.34396	1	30.69841	25.37241	1	1	64.94442	1	20.07992	1	1
YBX3	94.97672	152.0533	1	1	1	153.6726	1	271.561	1	44.24289	1	91.64517	282.8577	1	1	319.2649	1	311.6529	1	1
YES1	1	78.55172	52.24714	1	1	68.52526	1	53.44125	1	36.97837	1	48.77288	55.56088	1	1	1	1	434.3286	1	1
YOD1	1	1	41.26954	1	1	1	1	1	1	40.68237	1	23.86622	27.59703	1	1	1	1	1	1	1
YPEL1	1	29.03766	43.7683	93.21195	92.76166	26.96308	139.455	1	105.9272	95.34279	116.8288	92.63387	27.88223	106.6816	72.132	1	121.8853	1	74.4722	139.0453
YPEL2	1	1	38.30054	1	1	1	1	28.61784	1	1	1	1	1	1	1	1	1	104.2301	1	1
YTHDF1	1	44.63204	45.58463	1	44.69358	39.83509	1	1	1	1	1	44.19922	38.92575	1	1	1	1	47.7581	65.31337	1
ZBED3	1	60.7501	52.42034	1	1	77.1038	1	1	1	1	1	31.19424	61.73362	1	1	76.46599	1	66.49112	1	1
ZBTB1	42.90902	51.37141	54.00681	1	1	41.06947	1	85.52922	1	57.98885	103.1323	54.74112	43.78903	112.3165	1	64.05383	1	67.61325	62.29012	1
ZBTB14	1	1	1	1	1	1	1	1	1	26.76099	1	26.28833	1	1	1	1	1	1	1	1
ZBTB16	1	1	1	1	1	1	1	1	1	1	151.7001	1	1	57.74495	1	1	1	1	1	1
ZBTB18	1	94.80208	25.04496	650.0491	867.154	134.2717	958.8473	1	498.7329	316.4077	164.0719	229.7367	163.1011	1	1253.305	186.1467	1062.367	75.37992	701.1389	1267.34
ZBTB21	1	39.84683	1	39.40362	48.37136	33.77698	1	35.19924	1	30.55146	1	40.0055	58.97438	71.47174	1	1	1	19.79142	1	1
ZBTB24	1	1	1	1	1	1	1	1	1	1	1	1	1	1	1	1	1	1	1	103.6483
ZBTB25	1	1	1	1	1	1	1	1	1	1	1	20.77376	1	1	1	1	1	1	100.4736	1
ZBTB33	1	42.04695	1	52.79393	48.9942	44.12913	1	1	1	52.91201	1	44.56352	44.03153	1	1	1	1	1	50.44191	1
ZBTB34	1	1	1	1	1	1	1	1	1	1	1	1	1	1	1	1	219.5482	103.5744	1	1
ZBTB37	1	55.4502	1	53.69698	56.91788	38.75307	1	28.34528	57.8904	1	76.55172	30.91284	1	68.20172	1	1	1	32.994	1	103.6483
ZBTB38	35.40322	1	1	52.8955	91.02567	38.66967	149.9815	146.6926	297.6432	1	1	1	1	82.4005	123.3667	1	1	183.7339	36.04484	190.2284
ZBTB4	1	49.07001	1	42.73465	1	42.77397	1	24.88368	1	39.50142	1	33.45756	55.23599	1	1	1	1	1	1	1
ZBTB5	1	1	1	1	1	1	1	1	1	1	1	1	30.82995	1	1	1	1	1	1	1
ZC3H12C	1	57.1777	1	1	1	30.67592	1	1	1	59.2884	1	49.08495	32.95331	1	1	1	1	1	1	1
ZC3H4	1	36.50825	72.6404	47.38272	43.23587	28.38819	1	1	1	60.13298	1	41.92182	28.47868	65.40707	1	46.78919	1	1	1	70.02264
ZC3H6	1	49.44175	1	89.49167	102.8399	67.3534	159.8753	1	263.7701	42.17812	135.925	53.20626	44.92514	135.9671	112.9142	1	161.0129	26.39661	87.82623	70.02264
ZC3H8	1	36.55086	70.56612	82.51253	85.96873	39.61436	1	1	86.34314	49.6539	95.31293	33.22835	43.27927	91.82298	105.7127	47.7144	79.89048	36.48781	1	172.671
ZC3HAV1	1	48.77438	1	1	1	32.2139	1	209.8959	1	1	1	24.77418	33.58493	1	1	1	1	96.25711	1	1
ZCCHC12	1	1	1	1	1	1	1	1	1	1	72.25209	1	1	1	1	1	1	1	1	1
ZCCHC24	1	122.5769	348.4808	1	1	40.84104	1	58.90155	1	1	1	1	29.94146	1	1	1	1	30.88907	1	1
ZCCHC6	68.39005	1	1	1	1	26.46404	1	49.01531	1	1	1	21.86134	1	1	1	1	1	1	1	1
ZCCHC7	1	52.23889	1	53.38131	57.04294	31.53865	1	1	1	47.25172	1	31.09763	38.97178	1	43.04946	1	1	1	1	116.5001
ZCCHC8	1	39.66297	31.1781	1	41.63523	33.23359	1	41.55148	1	28.8404	60.66627	58.4083	47.66779	1	1	1	53.77506	52.4598	1	1
ZCCHC9	1	38.18353	1	1	1	24.31108	1	1	1	1	1	26.31306	23.08506	1	1	1	1	1	1	1
ZDBF2	1	1	1	1	54.00635	33.66786	1	74.6601	1	1	1	29.40194	46.79241	1	1	1	1	24.73949	1	1
ZDHHC13	1	1	1	1	1	47.46848	1	1	1	1	1	24.86496	22.44718	1	1	1	1	1	1	70.02264
ZDHHC2	1	1	51.84785	1	1	60.73903	1	1	1	1	1	1	50.96443	1	1	1	1	1	1	70.02264
ZDHHC20	1	47.84762	1	1	1	1	1	25.94596	1	34.96754	1	29.53992	1	1	1	1	1	34.0359	1	1
ZDHHC21	1	81.64076	58.93656	47.57418	50.67625	65.21114	1	1	104.2955	49.03944	84.34608	35.17507	38.65812	1	1	1	1	42.28506	30.17077	1
ZDHHC3	1	1	38.03021	1	1	25.22516	1	26.89346	1	1	1	32.85411	1	1	1	1	34.90597	67.80832	1	1
ZDHHC6	1	35.60533	1	1	1	33.8979	1	1	1	35.99692	1	31.96215	30.49977	1	1	58.78815	1	23.29582	1	70.02264
ZDHHC9	1	1	54.35522	1	1	1	1	1	1	1	1	1	1	47.29398	1	1	143.6332	61.22348	1	1
ZEB1	1	630.701	503.8565	179.3189	84.33186	221.6005	1	853.9876	1	441.4282	86.27628	344.9653	153.6773	1	1	206.1564	1	269.7605	1	1
ZFHX3	178.5508	53.93759	1	1	1	1	1	181.7595	132.5089	39.17945	1	41.2419	1	1	1	1	1	93.13935	1	70.02264
ZFHX4	1	109.9952	75.56303	236.0023	221.6366	245.5002	1	1	1	263.7556	1	199.4381	145.5033	1	57.5124	137.5584	1	1	145.4376	70.02264
ZFHX4.AS1	1	1	1	1	1	30.48994	1	1	1	1	1	1	1	1	1	1	1	1	1	1
ZFP1	1	1	1	49.94362	47.3957	24.07738	1	1	1	1	1	28.0927	25.45075	74.76208	1	1	1	33.66892	1	1
ZFP30	1	1	1	1	1	25.41699	1	1	1	1	1	1	1	1	63.78816	1	127.5858	1	1	139.0453
ZFP36	723.078	233.4404	1	1	1	608.2017	1	724.5816	1	1	1	1	1037.094	1	1	176.928	1	1205.662	1	1
ZFP36L1	766.1857	861.1736	210.6298	1	1	1297.296	1	964.0094	1	199.3833	1	374.2715	1958.496	1	1	653.146	1	1095.164	1	1
ZFP36L2	392.4303	369.4183	295.1385	1	1	334.3427	1	278.9765	1	125.521	1	160.3905	244.4398	1	1	165.3272	1	191.1426	1	1
ZFP37	1	1	1	48.63538	46.36012	1	1	1	1	1	1	30.75617	1	1	1	1	1	1	1	70.02264
ZFP82	1	1	39.26711	1	44.57355	37.36043	1	1	1	41.9547	62.39017	38.10855	33.18071	1	1	1	1	1	1	1
ZFP90	1	54.43694	42.70211	109.4352	102.1016	61.62314	1	32.66251	93.09896	74.93547	79.95132	54.73109	54.93848	83.37188	124.4517	1	1	24.06157	116.6708	172.671
ZFP91	1	68.0988	40.9357	60.45192	57.58642	53.29929	1	49.54235	1	61.05392	1	80.2076	51.4579	1	1	1	1	39.428	97.83045	1
ZFPL1	1	1	1	1	1	28.06619	1	1	1	29.18085	1	29.0323	23.78386	1	1	1	1	112.5135	72.56194	1
ZFPM2	1	1	1	1	1	1	1	1	104.0826	1	1	1	1	1	1	1	1	1	1	1
ZFYVE21	1	43.02854	1	50.95794	1	68.28978	1	37.61606	134.2998	33.94825	1	41.39589	41.07574	1	1	61.30619	1	49.17724	112.7604	1
ZFYVE9	1	1	1	1	1	29.44144	1	28.92607	1	29.93638	1	1	26.13652	1	1	1	1	1	1	70.02264
ZGRF1	1	1	1	1	1	1	1	1	1	72.55888	1	71.65022	1	1	1	1	1	1	1	1

ZHX2	1	1	1	1	1	1	1	1	1	1	1	1	51.8112	1	1	1	1	1	1	1
ZHX3	1	42.82088	58.45085	65.50056	1	80.34624	1	1	1	96.17371	1	75.98559	65.20934	1	1	46.8069	1	1	1	1
ZIC1	1	59.14262	1	1	1	1	1	401.0101	1	1	1	1	1	1	1	1	1	1	1	1
ZIC2	1	152.14	1	1	1	128.9735	1	148.6303	1	69.44669	1	116.7247	121.1031	1	1	99.0751	1	253.8047	1	1
ZIC4	1	1	1	1	1	1	1	61.78524	1	1	1	1	1	1	1	1	1	1	1	1
ZIC5	1	49.60152	1	1	1	52.80393	1	1	1	1	1	39.13796	48.80412	1	1	1	1	55.42447	1	1
ZIK1	1	1	1	1	1	1	1	1	1	1	1	26.8864	1	1	1	1	1	1	1	1
ZMAT1	1	90.42141	79.01534	1	1	139.9143	1	1	1	67.07267	1	52.1663	102.2085	1	1	100.6897	1	13.28091	107.4348	1
ZMAT3	1	83.01794	137.587	1	1	89.08066	1	56.01912	1	38.7865	1	35.95946	37.31209	1	1	1	1	12.91261	1	70.02264
ZMIZ2	1	1	1	1	38.65752	1	1	1	1	36.39882	1	31.20632	1	1	1	1	108.2791	1	1	1
ZMYM1	1	1	1	1	1	1	1	1	1	1	1	40.84203	24.59463	1	1	1	1	1	1	1
ZMYM3	1	1	1	1	1	1	1	1	92.13409	1	1	36.27296	23.27489	1	1	1	1	1	1	1
ZMYND10	1	1	1	1	1	1	1	1	1	1	1	1	84.9229	1	1	1	1	1	1	1
ZMYND19	1	1	1	1	1	1	1	1	1	44.30474	1	24.49173	1	1	46.63247	1	1	1	1	1
ZNF100	1	42.41635	44.84862	1	1	24.08564	1	1	1	36.01123	1	32.41431	30.0165	1	1	1	1	31.88075	54.13059	1
ZNF107	1	1	1	1	1	1	1	1	1	35.98771	1	45.19681	25.67654	1	1	1	1	1	1	1
ZNF124	1	1	1	1	51.29493	1	1	1	1	31.05695	1	1	1	1	1	1	1	1	57.45727	70.02264
ZNF134	1	49.45543	28.74792	47.23778	46.14607	26.00866	1	1	1	42.62671	65.94151	32.27611	42.65562	1	1	1	1	1	1	1
ZNF135	1	1	1	1	1	1	1	1	1	30.42206	1	1	1	1	1	1	1	1	1	1
ZNF136	1	32.09117	1	1	1	32.81975	1	1	1	58.60407	1	46.19937	32.39363	1	1	1	1	16.25124	1	1
ZNF140	1	1	1	1	1	1	1	1	1	29.41587	1	26.9723	1	1	1	1	1	1	1	1
ZNF154	1	1	1	48.71894	45.07543	1	1	1	1	37.20432	1	23.14288	1	1	1	1	1	1	1	1
ZNF160	1	41.48027	1	47.1662	43.406	42.41575	1	22.78794	1	32.89166	1	33.90798	25.87922	1	1	1	1	50.40518	1	1
ZNF184	1	1	1	1	49.08455	27.20773	1	1	1	46.65935	1	53.12766	24.50117	1	1	1	1	15.97538	29.96317	1
ZNF189	1	1	1	1	1	1	1	1	1	45.02435	1	29.52197	29.29697	1	1	1	1	35.67741	40.11348	103.6483
ZNF197	1	1	1	46.71841	42.78036	24.24018	1	1	1	44.04045	1	29.77733	1	1	1	1	1	1	1	1
ZNF219	1	52.62124	57.78406	50.57926	1	36.4318	1	1	1	65.01065	1	58.76427	44.68793	64.56743	1	1	1	1	1	1
ZNF224	1	1	57.00158	1	1	1	1	1	42.85581	1	1	1	1	1	1	1	1	17.23394	1	103.6483
ZNF226	1	37.94728	46.98596	43.20358	40.0967	42.0323	1	1	1	1	1	30.75887	31.96782	1	1	1	1	27.02492	1	70.02264
ZNF229	1	1	1	1	1	27.3671	1	1	1	1	1	1	1	1	1	1	1	1	1	1
ZNF232	1	1	1	1	1	1	1	1	1	37.55748	1	1	1	1	1	1	1	1	1	1
ZNF236	1	1	1	1	1	30.68303	1	1	1	40.29424	1	22.88097	26.10082	1	1	76.56968	1	1	1	103.6483
ZNF25	1	1	1	1	38.86239	25.88849	1	1	1	39.05038	1	26.03855	1	1	1	1	1	27.52216	1	1
ZNF250	1	1	1	51.27583	53.74285	1	1	1	1	29.01852	1	27.73415	25.44371	62.34085	1	1	1	16.13871	77.61332	1
ZNF253	1	35.73315	1	77.69424	66.91639	29.56473	1	1	1	47.58166	1	36.36462	28.3166	1	59.29631	41.38356	1	1	117.2653	1
ZNF254	1	84.6122	96.11332	116.4984	92.23492	63.06857	1	27.23106	1	113.6639	141.3682	63.66676	54.99815	149.4122	90.63292	1	140.4191	40.33891	76.23071	172.671
ZNF26	1	1	45.53961	1	1	1	1	1	1	1	1	1	1	1	1	1	1	31.54522	1	1
ZNF260	1	44.05895	40.42863	46.03074	1	47.35517	1	1	1	70.16563	1	50.36957	32.79781	1	1	1	1	27.32194	1	1
ZNF263	1	1	1	1	1	1	1	1	1	1	1	1	1	1	1	1	1	49.77989	1	1
ZNF264	1	40.87376	1	43.44132	50.07789	24.06029	1	1	1	26.81586	64.25277	26.53786	30.63849	71.82466	1	1	1	26.92849	1	1
ZNF267	69.77394	1	1	1	1	1	1	1	1	1	1	1	1	1	1	1	1	27.94871	1	1
ZNF273	1	41.27888	1	1	1	24.45083	1	1	1	55.60792	1	63.86584	44.16197	1	1	1	1	1	1	1
ZNF274	1	33.63582	37.93827	44.12725	44.46447	49.41741	1	1	1	40.72214	1	53.23893	46.77236	66.34381	1	79.05675	1	1	1	1
ZNF275	1	34.81793	1	1	1	1	1	1	1	1	1	1	31.97212	1	1	1	1	1	1	1
ZNF276	1	1	120.2464	1	1	1	1	1	1	1	1	26.33029	24.97634	1	1	1	1	1	1	87.58009
ZNF3	1	49.93971	39.62263	63.4438	55.69685	45.35431	1	1	1	64.5136	83.0241	48.79931	59.28697	64.68374	1	1	1	67.61346	120.7225	70.02264
ZNF300	1	1	1	70.93042	72.93486	1	1	1	1	57.14912	78.13945	46.8756	26.56205	94.41677	1	1	173.1996	1	73.65089	70.02264
ZNF318	1	1	1	1	1	1	1	1	1	1	1	37.92508	27.88496	1	1	1	1	1	1	70.02264
ZNF32	1	69.58258	92.32843	84.98883	86.34328	52.90662	152.7506	32.59019	1	87.40729	78.15264	58.6853	64.31829	93.31639	79.25617	50.1298	1	42.95349	155.0527	70.02264
ZNF324	1	1	1	1	1	1	1	1	1	1	1	1	1	1	1	1	72.84512	1	1	1
ZNF331	1	44.03629	27.73526	1	62.42131	30.01953	1	79.0107	1	44.99437	66.73431	40.82343	37.19013	1	1	1	1	1	1	1
ZNF334	1	36.22627	1	1	1	34.00937	1	1	1	1	1	21.38691	23.70688	1	1	1	1	1	1	1
ZNF337	1	1	1	1	38.48098	1	1	1	1	1	1	1	23.50553	1	1	1	166.2964	1	1	1
ZNF33B	1	1	1	54.648	66.00996	32.8258	1	1	1	1	1	1	26.16507	1	97.12311	1	184.9945	1	53.78388	172.671
ZNF350	1	1	1	1	1	1	1	1	1	1	1	1	1	1	1	1	1	1	50.69089	1
ZNF354A	1	1	1	1	1	1	1	1	1	28.0568	1	1	26.19447	1	51.9309	1	1	1	50.55048	1
ZNF354B	1	1	1	1	1	1	1	1	1	1	1	1	1	1	1	1	49.07455	1	1	1
ZNF354C	1	1	64.0478	1	1	24.05383	1	1	1	1	1	23.49074	1	1	1	1	1	1	1	1
ZNF362	1	1	1	1	1	1	1	31.34894	1	34.27538	1	39.36675	1	1	56.9197	1	1	63.59912	55.59355	103.6483
ZNF367	1	1	1	1	1	1	1	1	1	51.02101	1	42.43205	1	1	1	1	1	1	1	1
ZNF382	1	1	1	1	1	1	1	1	73.09647	1	1	1	1	1	1	1	89.35021	1	1	103.6483
ZNF385A	1	1	1	1	1	1	1	58.75307	1	1	1	1	1	1	1	1	1	1	1	1
ZNF385D	1	1	50.96653	1	1	1	1	1	452.538	1	1	1	1	1	1	1	1	1	1	1
ZNF407	1	1	50.72892	1	1	1	1	1	1	1	1	1	1	1	1	1	1	21.83511	1	70.02264
ZNF414	1	1	1	1	1	1	1	1	1	1	1	1	1	1	1	1	75.10496	1	1	1
ZNF420	1	1	32.57606	1	1	1	1	1	1	1	1	21.52227	1	1	1	1	1	1	42.10604	1
ZNF429	1	1	37.41225	1	1	1	1	1	1	1	1	1	1	1	1	1	1	1	1	1
ZNF432	1	34.7185	1	1	1	23.95231	1	1	1	1	1	25.16135	28.22989	1	1	1	1	1	1	1
ZNF439	1	40.30705	47.26345	52.51956	58.2427	30.75356	1	1	1	33.33197	1	42.48671	31.17701	86.65544	41.77603	1	1	1	1	1
ZNF454	1	1	1	1	1	1	1	1	86.75925	1	1	1	1	1	1	1	145.7102	1	1	70.02264
ZNF462	1	185.1316	381.6184	411.9376	481.4661	106.6486	604.105	40.97402	214.6422	174.8611	205.8687	131.4173	118.4808	355.5075	622.3394	101.4913	212.161	1	430.0891	190.2284
ZNF471	1	1	1	1	1	1	1	1	1	1	1	1	1	1	1	1	98.22528	1	1	139.0453
ZNF48	1	1	1	1	40.69364	1	1	1	1	33.0534	1	31.19647	1	56.41074	1	1	1	1	1	1
ZNF487	1	1	1	1	1	1	1	1	1	1	1	1	54.53506	1	1	1	1	1	1	1
ZNF492	1	1	1	1	1	1	1	1	1	1	1	30.64617	1	1	1	1	1	1	1	1
ZNF503	1	1	1	1	1	1	1	112.3066	1	1	1	1	1	1	1	1	1	245.8388	1	1
ZNF506	1	1	1	63.48277	64.8628	39.37661	1	1	1	1	1	30.71233	27.06151	56.78934	34.77549	1	1	47.19585	1	1
ZNF507	1	1	1	1	1	22.99945	1	1	1	39.8297	1	1	33.34965	1	1	1	1	1	1	1
ZNF512	1	1	33.45482	60.56349	57.02362	31.70143	1	1	1	42.93041	1	35.71975	31.0617	65.93443	53.50404	1	1	1	91.54545	87.58009
ZNF516	1	1	41.6895	1	1	1	1	32.77382	1	1	1	1	1	1	1	1	1	1	1	1
ZNF518B	1	41.90077	48.19343	48.7667	59.63661	46.57906	1	1	1	57.88683	1	42.89033	40.30477	72.93885	82.19026	1	1	1	57.4027	1
ZNF519	1	32.39121	39.20183	52.48514	62.60052	24.0634	1	1	1	75.80055	81.06496	50.01178	1	72.90949	46.4972	81.48721	1	1	137.0676	103.6483
ZNF521	1	68.94247	67.01124	1	1	32.67939	1	1	1	1	1	1	28.46386	1	1	1	1	231.9785	1	1
ZNF536	1	1	1	1	1	1	1	1	1	1	320.6565	1	1	240.7581	1	1	1	1	1	1
ZNF543	1	1	1	1	1	1	1	1	1	1	1	1	1	1	1	1	153.8378	1	1	1
ZNF544	1	1	1	1	1	22.4073	1	1	1	1	1	29.00405	26.58442	1	1	1	1	31.85159	101.7868	1
ZNF548	1	36.84493	1	1	42.19137	1	1	32.68015	1	1	1	40.81002	35.43498	59.33791	1	1	1	1	1	1
ZNF559	1	1	1	44.15221	1	27.96376	1	1	1	34.818	1	26.17199	26.70731	1	1	1	1	1	1	1
ZNF561	1	1	46.76082	49.48975	41.96001	1	1	1	1	33.32324	1	30.87023	42.99614	1	1	1	1	34.31851	1	1
ZNF562	1	51.23111	35.96801	92.34425	87.05154	38.87952	1	1	1	77.99355	1	53.59711	41.28822	68.60201	83.2399	92.20611	144.1144	1	190.8692	1
ZNF566	1	39.30863	76.15523	58.76213	59.51843	37.02275	1	1	1	55.49992	1	64.68778	24.91007	1	56.54063	1	134.6115	1	1	1
ZNF567	1	34.44728	1	1	1	26.09392	1	1	1	45.176	1	29.87668	25.10056	1	1	1	1	1	1	1
ZNF568	1	1	1	1	44.6943	50.45839	1	1	1	47.94648	1	31.03103	30.60226	1	42.10817	1	1	1	1	1
ZNF570	1	1	1	43.09817	42.05613	1	1	1	1	29.87949	1	24.56475	1	1	1	1	1	1	1	1
ZNF581	1	1	1	1	1	1	1	1	1	1	1	1	29.56088	1	1	1	1	23.18836	55.12168	1
ZNF583	1	1	31.74557	51.38178	56.60355	1	1	1	74.30584	1	72.73682	1	1	81.17404	1	1	86.06436	1	1	1
ZNF587	1	1	1	1	1	1	1	1	1	32.84961	1	27.86848	23.42518	1	1	1	1	1	1	1
ZNF605	1	1	39.53917	65.94875	61.92932	38.49564	1	1	113.3335	33.47149	1	32.3598	30.3887	1	101.1948	42.43147	60.60068	1	37.58658	103.6483
ZNF606	1	1	1	54.29113	1	33.631	1	1	1	44.42926	1	32.9258	29.81238	1	41.30879	1	1	1	1	70.02264
ZNF611	1	1	42.8479	1	1	1	1	1	1	1	1	1	1	1	1	1	1	1	1	103.6483
ZNF618	1	1	26.61502	1	1	1	1	84.0525	1	1	77.08641	1	1	167.0514	1	1	1	36.43046	1	1

ZNF620	1	1	54.99608	1	1	1	1	1	1	46.96179	1	37.37656	28.35983	1	1	1	1	1	59.9078	1
ZNF621	1	53.55865	37.03284	1	1	37.08456	1	1	1	39.54176	1	29.00624	40.68354	1	1	1	1	1	1	1
ZNF622	1	1	25.41146	55.06892	58.89972	28.08445	1	28.81414	1	34.56405	1	32.46292	34.75683	79.34441	67.66165	1	1	19.0266	90.5775	1
ZNF625	1	1	1	1	1	1	1	1	1	29.0728	1	1	1	1	1	1	1	1	1	1
ZNF627	1	1	1	1	42.08558	1	1	1	1	26.88651	1	25.28296	1	1	1	1	1	1	1	1
ZNF649	1	51.53031	68.95357	1	1	1	1	1	1	1	1	27.67379	36.03123	1	1	1	1	1	1	1
ZNF654	1	37.54548	1	1	1	27.80248	1	1	1	1	1	31.3001	31.14396	65.86785	1	1	1	21.73466	1	1
ZNF660	1	34.98965	1	53.71665	46.172	29.47036	1	1	1	50.21584	1	30.38109	1	53.41465	1	1	1	1	1	1
ZNF667	1	37.47928	53.08726	64.23326	61.56578	1	1	1	113.6836	37.43174	69.40247	24.49612	34.4167	1	1	1	156.6563	1	103.9652	1
ZNF672	1	1	1	1	1	1	1	1	1	42.5824	1	24.05459	28.21575	1	1	1	1	1	1	1
ZNF675	1	46.51231	55.97183	51.3674	58.90447	30.79535	1	1	1	54.96456	74.66241	49.23516	1	103.485	59.58761	1	1	22.01281	105.1994	103.6483
ZNF681	1	1	66.12623	1	1	31.12224	1	1	1	33.7146	1	29.66756	1	1	1	43.87897	1	1	1	1
ZNF682	1	1	1	1	50.12686	30.89649	1	1	1	65.16826	1	46.9357	1	1	1	1	1	1	1	1
ZNF692	1	1	1	1	1	1	1	1	1	1	1	1	1	1	47.95709	1	35.45667	1	1	1
ZNF7	1	43.31547	1	1	1	1	1	1	1	34.99643	1	26.28161	28.02933	1	1	1	1	1	61.60056	1
ZNF703	1	1	1	1	1	50.83063	1	314.8411	1	53.01498	1	34.55089	47.53255	1	1	1	1	38.78933	1	1
ZNF71	1	1	1	1	46.70549	1	1	1	1	28.8147	1	1	1	66.33085	1	1	1	1	64.39964	1
ZNF726	1	1	1	1	1	1	1	1	1	28.89837	1	28.51438	1	1	1	1	1	1	1	1
ZNF730	1	1	1	1	1	1	1	1	1	40.55454	1	25.38936	1	1	1	1	1	1	1	1
ZNF736	1	1	1	1	1	1	1	1	1	29.62118	1	29.63534	1	1	1	1	1	1	1	1
ZNF737	1	1	1	1	1	1	1	1	1	1	1	1	1	1	67.26118	1	1	1	57.03033	70.02264
ZNF74	1	1	1	43.93084	42.4143	1	1	1	1	1	1	1	1	1	43.97844	1	1	1	1	103.6483
ZNF766	1	49.39776	1	65.19672	62.65894	27.48046	1	1	1	78.02262	1	36.93632	32.27724	60.31839	91.39035	1	1	1	82.17898	1
ZNF775	1	38.999	1	1	44.03693	37.36061	1	1	1	34.08094	1	50.23489	33.61478	1	64.61154	1	1	99.40489	1	70.02264
ZNF785	1	1	24.87386	46.44288	47.00658	1	1	1	1	38.20728	1	1	1	1	1	1	112.7576	23.15865	1	70.02264
ZNF787	1	47.59625	1	1	1	43.05208	1	1	1	28.07663	1	27.50551	40.40123	1	1	1	1	25.54448	1	1
ZNF790	1	1	44.33331	1	1	1	1	1	1	1	1	1	1	1	1	1	1	1	1	1
ZNF8	1	1	1	1	44.53591	1	1	1	1	1	1	1	1	1	1	1	1	1	39.48227	1
ZNF804A	1	1	1	123.1455	120.5482	1	1	1	1	44.91369	1	1	1	1	1	1	1	1	84.19751	1
ZNF814	1	40.51149	35.29974	52.51006	48.3916	1	1	1	1	41.09929	1	32.60584	25.86862	1	1	1	1	1	1	1
ZNF821	1	45.37292	43.66108	111.5997	122.3058	31.78646	122.1367	1	62.31078	74.39284	135.8543	52.80769	35.15296	141.5265	136.0158	1	1	48.72278	76.47768	70.02264
ZNF83	1	1	1	67.72296	55.21483	49.42211	1	36.07142	1	44.59431	1	77.94645	42.98657	1	1	94.91734	150.4056	35.65741	61.32793	1
ZNF85	1	1	1	1	1	1	1	1	1	41.99553	1	36.93819	1	1	1	1	1	1	1	1
ZNF891	1	1	59.33538	60.87006	75.37644	1	1	1	55.68947	1	1	26.39309	1	1	90.12354	1	128.9455	1	64.60706	1
ZNF93	1	1	1	1	1	1	1	1	1	44.1729	1	33.48441	1	1	1	1	1	1	1	1
ZNHIT2	1	1	1	1	1	1	1	1	1	1	1	1	32.58054	1	1	1	1	1	1	1
ZNHIT6	1	63.35499	74.89665	1	1	48.74892	1	28.9294	1	43.96579	1	38.87579	39.91935	1	1	1	1	1	97.13685	1
ZNRF2	44.51192	1	1	1	1	1	1	1	74.67619	1	1	26.37774	25.67069	1	1	1	59.35056	1	1	1
ZNRF3	1	47.07639	1	1	1	50.43139	1	1	1	1	1	26.27124	27.68829	1	1	1	1	17.46535	1	1
ZPR1	1	39.30747	29.15697	57.06398	54.43293	31.91466	1	1	1	45.88992	61.27617	31.62211	40.31188	68.30541	1	54.86988	1	35.72991	1	1
ZSCAN9	1	34.86983	1	64.69641	52.2483	1	1	1	1	35.15451	1	24.18229	1	1	52.10284	1	1	1	86.06508	1
ZUFSP	1	1	31.78851	1	1	1	1	1	1	29.41247	1	23.09622	1	1	1	1	1	1	1	1
ZWILCH	1	1	1	1	1	1	1	1	1	29.52074	1	41.33384	1	1	1	1	1	1	1	1
ZWINT	1	1	1	1	1	1	1	1	1	98.37903	1	218.9059	1	1	1	56.04881	1	1	1	1
ZXDC	1	42.58932	1	43.63522	1	1	1	1	1	61.62599	1	33.60707	1	1	1	1	1	1	1	1
