# Supplementary material for: Altered neuroepithelial morphogenesis and migration defects in iPSC-derived cerebral organoids and 2D neural stem cells in familial bipolar disorder
Source: Oxf Open Neurosci. 2024 Apr 3;3:kvae007. doi: 10.1093/oons/kvae007 (PMC11024480; doi:10.1093/oons/kvae007)
Supplement: Web_Material_kvae007 [file web_material_kvae007.zip › Phalnikar et al OON 2024 Reviewers Comments.docx]

We thank the reviewers for their valuable insights into our manuscript. Their constructive feedback has greatly enhanced the quality of our work. We have carefully addressed each comment, offering detailed responses as below. We anticipate that the revised version of our manuscript will meet with the approval of the reviewers.

**Reviewer: 1**

Comments to the Author
The authors present a study to elucidate the neurodevelopmental origins of familial bipolar disorder using patient derived 2D and 3D iPSC neural cell models. To this end, the authors differentiate 3/4 iPSC lines of 1/2 healthy donors and 2 BD patients into young 3D cerebral organoids (~40/50 days) and analyze regions resembling neuroepithelial cell clusters (referred to as buds), neural progenitor cell and neuron frequency and positioning, gene expression patterns and NSC migration behavior. They report deficits/differences in all these assayed parameters between BD and HC derived organoids/NSCs.
This study is certainly interesting but requires several additions and refinements that are essential before publication. These encompass 1.) text changes in framing and conclusions 2.) details on experimental methods, 3.) additional analyses 4.) additional experimental data. The authors need to frame their study accordingly.

1.1) The evidence for BD as a neurodevelopmental disorder is limited. Thus, the statement needs to be toned down to e.g. that there is evidence for a neurodevelopmental contribution to BD (in a subset of cases). E.g. statements like this need to be modified: "Bipolar disorder (BD) which is a severe mental illness, has its origins in neurodevelopment"

Response: We thank the reviewer for bringing this to our attention and have revised these statements to reflect the limited evidence on the neurodevelopmental origins of bipolar disorder.

- Abstract (Lines 2-3, 5-7)
- Introduction (Lines 62-65, 92-96)

1.2) Discuss difference between familial and sporadic BD. The experimental design chosen here is certainly justified and interesting to study a particular neurodevelopmental contribution of BD in THIS family. Thus, generalizability of these results to BD in general are unclear at best and likely low as presumably CNVs/rare copy number variants are contributing to the observed phenotype. In any case, the donor number employed here does not justify any generalization beyond this specific family. This should be clearly stated. Thus statements like this need to be toned down:
"Our data reveal that the disorder is initiated during very early stages of neurodevelopment, namely neuroepithelial bud ..."

Response:

- We have now discussed familial and sporadic BD (Introduction, Lines 48-56)
- We have included statements on selecting clinically dense families as an experimental paradigm (Lines 92-96).
- We have clearly stated that the generalizability of the results is limited (Discussion, Lines 429 -434)

1.3) The authors should more clearly define what they consider a neuroepithelial bud.

Response: We have now included a description of the neuroepithelial bud and referenced previous studies that have described it. (Results, Lines 138-143).

1.4) The authors should discuss the state of the art in the BD organoid field in more detail as quite a few papers have been published here. This will help also to frame their work appropriately.

Response: We have cited additional references and compared our study with previously published work (Introduction, Lines- 57-82)

1.5) The evidence for cortical malformations as a general feature of BD is nonexistent. There is evidence that if there are cortical malformations, BD can arise. This something different than:
"Such brain malformations during neurodevelopment are thought to underlie observed clinical manifestations."

Response: We have removed this statement and revised other statements which convey that cortical malformation could be an underlying pathophysiological mechanism in BD.

1.6) Wording in organoid models: It is important not to confuse the model with the real cortex. The wording used seems to frequently suggest that the authors equate the two:
"The newly born neurons migrate and ultimately reside in the cortical plate (CP)"

Response: Thank you for bringing this to our attention. We have now provided a description of the two regions, namely the ventricular/intermediate zone containing the progenitors and the post-mitotic zone comprising the newly migrated neurons. (Lines 143-151)

2.1)   Details on the image analysis statistics need to be provided. The authors should include a supplementary table that contains all relevant processed data values alongside relevant meta data information. For example, how many fields of view per organoid were analyzed, what was the background intensity how many independent batches, were controls and cases matched in each batch? was the analysis carried out in a blinded fashion? In addition, statistical testing should account for covariates if they are relevant (e.g. significant effect of batch), using e.g. linear mixed models if necessary.

Response: Thank you for this valuable suggestion.

We have included a detailed image analysis with statistics in Supplementary Table 1, containing relevant metadata. This includes information on number of fields of view per organoid, number of organoids, number of independent batches etc. Additionally, Supplementary Table for reviewers, provided for reviewers, contains all relevant processed data values.

We tested the "batch effect" in organoid culture using a linear mixed model, as recommended by the reviewer. Detailed descriptions are provided in the Statistical Analysis section (Lines 652-656).

We evaluated the presence of a batch effect from two independent inductions for organoid generation using two-way ANOVA mixed model analysis. Our analysis revealed no significant batch effect (P < 0.05) across most parameters. However, for the total number of NEBs, the batch effect constituted 11.3% of the total variation (Supplementary Table 1). Given the absence of a batch effect for most tested parameters, we proceeded with data analysis using Welch’s T-test, an adaptation of the Student’s T-test that accommodates unequal sample sizes. For graphical representation and statistical analysis, we pooled data from both patients and controls. This approach aligns with methodologies employed in previous studies for investigating neurodevelopmental or psychiatric disorders across multiple iPSC lines. These are now detailed in the Statistical Analysis (lines- 644-657).

Whenever feasible, we ensured experiments were matched by batch. Specifically, HC1 and A1 organoids were matched for 50 days in vitro (DIV). For 40 DIV organoids, HC1, A1, and A2 were batch matched. HC2 was processed independently as it was part of a separate experiment.

The analysis could not be conducted in a blinded fashion because the patient organoids exhibited distinct topological defects.

3.1) I did not see any evidence supporting the claim that the used 3D cultures resemble cortical cell types. If this claim is made, evidence should be provided for that.

Response: We have incorporated a figure (Supplementary Figure 1) demonstrating the presence of key cortical markers in our organoids, which includes the expression of apical radial glia markers such as PAX6, SOX2, NESTIN, as well as pan-neuronal markers like TUJ1. Furthermore, we evaluated the presence of deep-layer cortical neuronal subtype markers such as TBR1 and CTIP2 (Lines 152-155). Additionally, we have included a description of these procedures in the Materials and Methods section under "Culturing hiPSCs and generating cortical organoids."

3.2.) The authors should test whether nor not their organoids are composed of distinct proportions of different cell types, which their RNA-Seq data seems to suggest. This can be done using in silico deconvolution based on their bulk-RNA-Seq data and reference panels of publicly available scRNA-Seq data from organoids/post-mortem brain at different developmental stages. Details can be found in multiple previously published cerebral organoid papers.

Response: Thank you for this valuable suggestion. We have conducted the deconvolution analysis as recommended, utilizing the *Trevino et al., 2021* dataset, which encompasses scRNA-seq data from the human fetal brain at critical developmental time points. Our analysis employed scRNA-seq data from Gestational week (GW) 16-24 from the study. The results of the deconvolution analysis indicate that the cluster representing late radial glia (Radial glia expressing CD9 and GPX3 and expressed during GW20-24) exhibits the most substantial reduction, followed by CGE Interneurons, Glutamatergic neurons type 4, and subplate neurons. This analysis has been instrumental in identifying the distinct cluster most affected in the BD patient organoids. It also underscores that the organoids generated in our study appear to encompass distinct cell types, suggestive of these organoids mimicking relevant physiological development of human fetal neural tissue.

Detailed information regarding this analysis can be found in the Results (Lines 234-254) and Discussion (Lines 403-411) sections, with additional data plots presented in the main figure and supplementary figures (Figure 4H-J) and Supplementary Figure 2, Supplementary Table 2.

3.3) Is there a possibility to connect the observations from the organoid models to clinically relevant data from these patients? Is there evidence for cortical malformations in these patients? How many patients might be affected by this?

Response: Structural variations in BD often manifest subtly and may not align with severe malformations such as polymicrogyria or lissencephaly. Accurately assessing the prevalence of these variations in our cohort necessitates comprehensive neuroimaging analysis, which is an ongoing component of our research efforts. We aim to provide more definitive data regarding the prevalence of such malformations in future updates of our study.

In the current scope of our research, we have provided global volume patterns for the Bipolar disorder group, as well as specific individuals, A1 and A2, from the cohorts in question (Figure 7) and discussed the same in Lines 339- 354. While this may not directly address cortical malformations in the traditional sense, it does offer valuable insights into the structural brain variations associated with BD.

4.1) It is unclear to me why only 1 healthy control line was used in most of the 3D experiments whereas the 2 lines were used in the migration assay. In order to support the robustness of the results, the experiments in Fig. 2 and 3. Need to be repeated with at least one more HC line.

Response: We have included an additional control line to the study (HC2), which is a non-family control. The data from this control line has been incorporated in this study.

4.2.) It is likely that the severe phenotype observed in the 3D cultures is partially driven by high penetrance genetic aberrations in the patients. The authors should evaluate the genetic data of these patients to assess this and connect it to their e.g. differential gene expression data. This can be either done by exome-Seq or alternatively by GWAS microarray to detect e.g. larger CNVs such as the GSA array.

Response: We have incorporated information regarding potential genetic risk variants in patients, as determined through whole exome sequence analyses conducted by the ADBS consortium. These details are elaborated upon in the Results section, Lines 320-338, and are also presented as Table 1.

**Reviewer: 2**

Comments to the Author
The research titled "Altered neuroepithelial morphogenesis and migration defects in iPSC-derived cerebral organoids and 2D neural stem cells in familial bipolar disorder" explores early developmental defects associated with bipolar disorder using a cortical organoid model and 2D culture of neural stem cells derived from iPSCs of two siblings with familial bipolar disorder and related controls from the same family. Immunohistochemistry revealed differences in the size and organization of cortical rosettes. RNAseq of the organoids was conducted to identify differentially expressed genes, and a migration assay showed reduced motility of disease-related neuronal progenitors.

1. The article lacks several references of similar studies (psychiatric disease models) conducted in iPSC-derived neural stem cells and organoids. The authors should add additional references. For example: PMID: 30401811, review, PMID: 35948659 and references within, and others.

Response: Thank you for this valuable suggestion. We have incorporated additional references as recommended into the Introduction (Lines 57-82) and Discussion (Lines 412- 419)

2. The study reveals several differences between bipolar and healthy organoids, prompting questions such as whether the observed differences are neuronal-specific, if there are differences at the iPSC level, and the possible need of additional time-points in the study to understand the temporal nature of observed changes during organoid development. Additionally, is would be interesting to define what are the suggested molecular mechanisms for 2D migration defects. We understand that additional time-points may be difficult to conduct. However, if there are differences in the timing of neuronal differentiation, for example, the conclusions regarding comparisons, should be carefully phrased.

Response: Thank you for this insightful comment.

- To address potential differences at the iPSC level and ascertain whether the observed proliferation deficits in patient-derived organoids stem from the iPSC lines themselves, we conducted a proliferation assay on each iPSC line. Our findings indicate no significant variation in the percentage of cells undergoing cell division between iPSC lines of patients and controls (Lines 275-287, Figure 5D-F). However, similar proliferation experiments using EdU in organoids revealed a significant reduction in cycling progenitors, as evidenced by immunostaining for SOX2, EdU, and Ki67 (Figure 5A-C). This suggests that the observed variations in organoid cultures are attributable to differences in the neural stem cells generated post-differentiation from iPSCs into the neuroectodermal lineage. Interestingly, this implies that altered proliferation deficits manifest upon differentiation into the neural lineage, possibly illuminating why the disease may present as a neurological disorder despite the potential presence of strong genetic risk variants. This area presents an intriguing avenue for future research. (Discussion Lines- 372-380)
- We appreciate the reviewer's understanding regarding the challenges of incorporating additional time points within the limited timeframe of our study. We also believe that further time points may not be essential for the current analysis, as we are already observing neural stem cell (NSC) proliferation deficits, now quantified by EdU staining at the selected time point of 40DIV. The chosen age of the organoid is deemed appropriate for observing both NSC proliferation and neuronal differentiation, processes that occur concurrently at this stage. Moreover, even if additional later time points were to reveal that deficits in patient organoids are eventually rescued or corrected, this would still represent a developmental alteration when compared to controls. Such findings would not alter the observed differences between patient and control groups at the specified stage of development.
- Suggested molecular mechanisms for 2D migration defects-

We have included these underlying molecular mechanisms in the Results (Lines- 313-317)

Additionally, the genetic risk variants identified in our patients could potentially explain the observed migration deficits, as detailed in lines 320-338.

3. Concerning Figure 2, clarification is sought on the selection of organoid slices for analysis and presentation, and whether only one slice per organoid was analyzed is unclear from the legend or methods.

In Figure 2C, there are outliers in the HC1 condition, and it is unclear if the difference is mainly due to these outliers. More measurements and additional controls are required. It is unclear why the second control is only in the supplementary figure.

The differentiation in color within dots of A1 in graph C needs clarification.

Response: Please also see our response to review 1 Point number 2.1 regarding image analysis statistics and metadata.

Response: We have now amended the data analysis part as mentioned in response to reviewer 1, point number 2.1. We have now included a detailed image analysis with statistics in Supplementary Table 1, containing relevant metadata. We have now included another control, HC2, in this study. In the revised manuscript, we have adopted a different methodology, comparing measurements across patients and controls as pooled data - refer to response to reviewer 1-point number 2.1. This method aligns with the previous studies on neurodevelopment or disorders, employing multiple iPSC lines for both patient and control groups. This collective approach aims to assess the variability between the control and patient groups as a whole. Consequently, in our current graphical presentations, we have opted not to color-code the data points.

4. A1a, DCX IHS is different from all the rest and there is no obvious reduction of the signal in the areas of Sox2. Is this slice specific or a general feature of this mutant?

Response: The IHC with SOX2 and DCX are only used as representative images to show how NEB morphology/organoid topology that varies across patients and controls. This difference observed in A1a is slice specific. This is not a general feature. Also, we have not performed any quantification for DCX.

5. For Figure 3: SOX2 staining intensity, was not normalized to the amount of SOX2-positive cells. Given the differences in the number of positive cells, there is a need to clarify what this measurement is describing. From the provided images, the individual intensity of SOX2-positive cells actually looks brighter in A1a compared to HC1.

**Response:** We quantified the percentage of SOX2-positive cells by sampling approximately 200 cells in each neuroepithelial bud. The SOX2 intensity measurement reflects the expression level of SOX2 across the entire area of the NEB that was imaged. This additional measurement was conducted to verify whether the percentage of SOX2 counts is mirrored in the intensity measurements. Given the distinct units represented by these two measurements, normalizing them may not be the most appropriate approach.

The image provided for A1a serves as a representative example. It may appear brighter due to the high intensity of SOX2 in a subset of cells. Nevertheless, the overall proportion of SOX2 positive cells relative to DAPI-stained nuclei is lower in comparison to the control.

6. While counting TUJ1-positive cells may be challenging, using intensity per region does not appear to be the most suitable method for demonstrating the mis-localization of TUJ1-positive cells.

**Response:** The migration defect is strikingly evident in the images presented in the manuscript (Figure 3). We primarily use TUJ1 intensity in the ventricular zone (VZ) region as an indirect measure to highlight that neuronal cells are present in the VZ/intermediate zone (IZ) of patient organoids. This approach serves to underscore the abnormal localization of neuronal cells in these regions, indicative of migration defects in the patient-derived organoids. This approach has been used previously to quantify a cellular marker in mouse neocortical sections (see references 1 and 2 below) and cortical organoids (see reference 3 below). The method involves analyzing the intensity of specific neuronal or proliferation markers, in designated regions to deduce the presence and distribution of neural cells.

7. In Figure 4G, a more detailed explanation about how the comparison of RNAseq to single-cell data was performed is requested. The addition of RT-PCR or protein-level verification for significant RNA-seq results, such as Reelin, Sema3A, etc., is required. The levels of SOX2 and TUJ1 in the RNAseq should be provided.

Response: Thank you for this suggestion.

- We have now added details of RNAseq to single-cell data comparison in the methods section. (Lines 611-617). We have validated the downregulation of key genes from RNA sequencing results using quantitative RT-PCR (Lines 232-233, Supplementary Figure 3A). As requested by Reviewer 1, we have now performed deconvolution using published methods to reveal distinct cell type-specific clusters most affected in the BD patient organoids. Please refer to the response to reviewer 1- point number 3.2.
- We do not find levels of SOX2 and TUJ1 altered in patient organoids from our bulk RNA seq (data in Supplementary Table for reviewers). This could be because %SOX2 positive cells and SOX2/TUJ1 intensities are only quantified in the specified region of the organoid, which is NEB. The organoid is composed of NEBs as well as a population of cells that have not developed into an organized neuroepithelium. Since bulk RNA sequencing includes the whole organoid tissue, gene expression levels across NEBs and the whole organoid may not correlate.

8. For Figure 5, additional information is required regarding the levels of genes that were found differentially expressed in organoids, in the neuronal stem cell culture.

**Response:** For the key genes identified as differentially expressed in organoids, we conducted quantitative RT-PCR on neural stem cell (NSC) cultures. Our findings indicate that, with the exception of SEMA3A, other genes are also downregulated in NSC cultures. This observation is consistent with our quantitative PCR (qPCR) analysis for the organoids, further substantiating the gene expression patterns observed in our study (Supplementary Figure 3B and Lines -309-317).

**References**

1. Sokpor G, Kerimoglu C, Nguyen H, Pham L, Rosenbusch J, Wagener R, Nguyen HP, Fischer A, Staiger JF, Tuoc T. Loss of BAF Complex in Developing Cortex Perturbs Radial Neuronal Migration in a WNT Signaling-Dependent Manner. Front Mol Neurosci. 2021 Jun 16;14:687581. doi: 10.3389/fnmol.2021.687581. PMID: 34220450; PMCID: PMC8243374.
2. Hourigan, B., Balay, S.D., Yee, G. et al. Capicua regulates the development of adult-born neurons in the hippocampus. Sci Rep 11, 11725 (2021). <https://doi.org/10.1038/s41598-021-91168-5>
3. Klaus, J., Kanton, S., Kyrousi, C. *et al.* Altered neuronal migratory trajectories in human cerebral organoids derived from individuals with neuronal heterotopia. *Nat Med* **25**, 561–568 (2019). https://doi.org/10.1038/s41591-019-0371-0
